# Global *in silico* analysis reveals a core gut microbiota in fall armyworm (*Spodoptera frugiperda*) shaped by geography and diet

**DOI:** 10.1093/ismeco/ycag233

**Published:** 2026-08-22

**Authors:** Lakshay Anand, Kendall R Corbin

**Affiliations:** Department of Horticulture, Martin-Gatton College of Agriculture, Food and Environment, University of Kentucky, Lexington, KY 40546, United States; Department of Horticulture, Martin-Gatton College of Agriculture, Food and Environment, University of Kentucky, Lexington, KY 40546, United States

**Keywords:** gut microbiota, meta-analysis, *in silico*, 16S rRNA, fall armyworm, core microbiota, synthetic microbial community (SynCom), *Spodoptera frugiperda*

## Abstract

Fall armyworms (*Spodoptera frugiperda*) are an invasive agricultural pest whose adaptability and resilience are increasingly linked to their gut microbiota. In this study, we performed a global-scale *in-silico* meta-analysis of publicly available 16S rRNA datasets to identify a conserved core gut microbiota and assess how geography and diet shape microbial community structure and function. Using both prevalence-based and unsupervised clustering approaches, we defined a robust global core microbiota composed of 88 high-confidence ASVs, including genera such as *Enterococcus, Pseudomonas*, and *Acinetobacter*, which were consistently present regardless of geographical region or diet. These taxa are important in nutrient metabolism, detoxification, and stress tolerance, contributing to host fitness and ecological success. Geographic origin significantly influenced gut microbiota diversity and composition, with continent- and country-level differences supported by alpha/beta diversity metrics and supervised machine learning classification (AUC = 1.0). Diet also influenced microbial community structure, although its effect was weaker than that of geography, explaining 11.9% of the variation compared to 13.5%. Functional predictions using PICRUSt2 revealed region-specific enrichment of pathways related to xenobiotic degradation, amino acid metabolism, and vitamin biosynthesis, suggesting specific microbial taxa may contribute to host adaptation and resilience through these metabolic functions. This is the first global synthesis of the *S. frugiperda* gut microbiota that integrates geographic and dietary factors. The identification of a global-level conserved core microbiota provides a foundation for developing synthetic microbial communities and microbiome-informed pest management strategies aimed at enhancing control efficacy.

## Introduction

*Spodoptera frugiperda*, commonly known as the fall armyworm (FAW), is one of the most destructive agricultural pests with a documented host range of 353 plant species [1–5]. Over the past decade, FAW has rapidly expanded from the Americas into more than 76 countries outside of its native range (Africa, 47; Asia, 23; Oceania, 6) [5–8], resulting in substantial yield losses in maize and other staple crops [3, 8–10]. Its success as an invasive species is driven by its broad host range, high reproductive capacity, migratory behavior, and resistance to insecticides [11, 12]. Moreover, recent models predict substantial climatic suitability for further FAW expansion, potentially exacerbating food security challenges in vulnerable regions [13]. Despite ongoing efforts to mitigate FAW-induced losses, there remains a critical need for the development and adoption of more effective control strategies.

Beyond conventional chemical and genetic control approaches, manipulation of insect–microbiome interactions have emerged as a novel strategy for pest management. Growing evidence indicates that microbial associations contribute to FAW’s ecological flexibility and invasion success. In insects, the gut microbiota plays a central role in shaping physiology, metabolism, immunity, and ecological adaptation [14–18], functioning as a dynamic symbiotic system that influences development, nutrient acquisition, and resistance to environmental stressors, including chemical control agents. Microbiome-based or microbiome-informed pest management strategies have shown promise in other agricultural pests, including manipulation of symbionts to reduce fitness or enhance susceptibility to pathogens and insecticides [16, 19, 20]. For highly polyphagous pests such as FAW, the gut microbiome has been proposed to facilitate survival across diverse host plants and agroecosystems [21–23]. To-date, most FAW microbiome studies have focused on individual geographic regions or are diet-specific [24–32], limiting our understanding of which microbial taxa are consistently associated with FAW globally versus those shaped by geography or diet.

In this study, we conducted a comprehensive global meta-analysis of publicly available 16S rRNA gene sequencing datasets from FAW gut samples spanning eight countries and four continents. A total of 19 independent studies [22, 24–37] were included, representing diverse geographic regions and insect diets. Our objectives were to (i) define the core gut microbiota of *S. frugiperda*, (ii) evaluate how geographic and dietary factors influence microbial community structure and diversity, and (iii) infer the potential functional roles of FAW gut bacteria across different global populations. By characterizing the FAW “core microbiota”—a set of microbial taxa consistently associated with a host regardless of external variation— microbial partners may be identified that are essential to host fitness and adaptability [38]. Moreover, by delineating the stable and variable components of the FAW gut community, these findings provide the foundation for future development of microbiome-mediated management strategies and/or FAW gut-derived synthetic microbial consortia (SynComs). However the core microbiota identified here represents only a candidate pool for such applications.

## Materials and methods

### Dataset curation and sequence retrieval

An extensive search for publicly available sequencing data was conducted to curate a comprehensive dataset of the *S. frugiperda* gut microbiota, with data curation completed in June 2025. Data was curated from major sequence repositories (NCBI Sequence Read Archive [39], the European Nucleotide Archive [40], and the DNA Data Bank of Japan [41]) using the search terms “fall armyworm,” “*S. frugiperda*,” and “fall-army-gut”. Due to a lack of available metagenomic datasets, the focus was placed on curating data generated using 16S rRNA amplicon sequencing methods. Search results were further filtered to include only studies annotated as “amplicon” and any region of the 16S rRNA gene. In parallel, we conducted a literature-based search to identify relevant studies and attempted to locate the corresponding sequencing data. In several cases, however, data was either not deposited or not publicly available at the time of retrieval and thus excluded from this study. After combining metadata from repository queries and literature mining, we finalized a curated list of 19 datasets that met the inclusion criteria.

These datasets spanned multiple geographic regions with the FAW being reared on a range of host plants (Supplementary Table 1A). Only studies with clear documentation of sequencing type, sample origin (location and the host-plant), and gut tissue specificity were retained to ensure consistency and biological relevance. Due to limited sample sizes and incomplete diet metadata across studies, diet-specific analyses were restricted to maize-fed gut samples, which represented the most consistently annotated FAW diet (Supplementary Table 1B).

### Amplicon processing and bioinformatics analysis

Raw sequencing reads were processed using QIIME2 (v2023.2). Across all samples included in the analysis, the average sequencing depth of merged reads was 111 130 ± 68 010. The dataset consists of studies mainly targeting regions V3-V4, V4, and V4-V5, with the majority of the studies targeting the V3-V4 region (Supplementary Table 1C). Paired-end reads were merged using PEAR [42] and then quality-filtered and denoised using DADA2 [43] generating amplicon sequence variants (ASVs). Taxonomic classification was performed using a Naive Bayes classifier trained on the SILVA 138 database [44]. Downstream analyses were performed on taxonomically assigned ASVs following uniform processing and phylogenetic placement to enable cross-study comparisons across variable 16S rRNA regions. ASVs annotated as chloroplast, mitochondria, and non-bacterial sequences were removed prior to downstream analyses. Alpha diversity metrics, including Shannon index and observed ASV richness, were calculated using the phyloseq [45] and vegan [46] packages in R [47]. An integrative analytical framework previously developed for cross-study comparison of human gut microbiome datasets was applied to harmonize 16S rRNA data across studies [48]. To further mitigate residual batch effects among datasets, fragment insertion was performed using SATé-enabled phylogenetic placement [49].

### Diversity analysis

To account for the compositional nature of microbiome data and differences in sequencing depth across datasets, beta diversity was assessed using Aitchison distance [50]. This metric is derived from the centered log-ratio (CLR) transformation and is robust to sparsity and zero inflation in microbiome datasets [51]. The ASV abundance table was normalized using the CLR transformation implemented in “compositions” [52] package in R, and then multi-dimensional scaling (MDS) using the “divide and conquer” approach [53] using R bigmds package version 3.0.0 was used. Statistical significance was evaluated using Kruskal–Wallis tests for alpha diversity and PERMANOVA (Adonis) for beta diversity. Differentially abundant taxa were identified using the ANCOM-BC approach [54] in R.

### Machine learning classification

Random forest classifiers were trained using CLR-normalized ASV abundance profiles to predict geography (continent or country) or diet. Model performance was evaluated using the area under the receiver operating characteristic curve (AUC-ROC). One-vs-all classification was used for multi-class comparisons. All analysis was performed in R using the randomForest [55] package.

### Functional prediction of microbial communities

Functional profiles were inferred using PICRUSt2 [56]. KEGG Orthology (KO) abundances were predicted from ASV data and used to assess metabolic potential. Principal component analysis (PCA) of KO abundance profiles was performed using the ggpicrust2 [57] R package (pathway_pca function) based on Euclidean distances. Differential pathway enrichment was assessed using ALDEx2 with Benjamini–Hochberg correction for multiple testing. KEGG pathways related to detoxification, nutrient metabolism, and stress tolerance were identified.

### Core microbiota identification

Core taxa were identified using two complementary approaches: (i) An occurrence-based analysis [58] in which taxa present in ≥70% of samples within each continent were considered continent-level core members. Taxa shared across all continents were defined as globally conserved core taxa. (ii) Consensus clustering: Five unsupervised machine learning clustering algorithms [59, 60] (k-means [61], hierarchical clustering, Gaussian mixture models [62], density-based clustering (DBSCAN) [63], and spectral clustering [64]) were applied to ASV abundance data. ASVs identified by ≥3 methods were considered consensus core members. Taxonomic assignments were reported at the finest resolution supported by the SILVA database; however, downstream biological interpretation focused primarily on genus-level patterns due to the heterogeneous amplicon regions represented across studies.

## Results

### The conserved core microbiota in FAW guts

To evaluate the extent to which geography shapes the gut microbiota of FAW, we analyzed 16S rRNA sequencing datasets from Africa, Asia, North America, and South America. We then performed an occurrence-based analysis to compare microbial taxa across samples from these continents. Core taxa were defined as genera detected in ≥70% of samples within each continent, and those shared across all continents were considered globally conserved core members. The analysis revealed a subset of genera present across all regions (Fig. 1A), including *Enterococcus, Acinetobacter, Pseudomonas*, and *Stenotrophomonas*, indicating persistent association with the FAW gut. Additional genera were only found associated with specific continents, suggesting these are region-specific FAW gut taxa. The core taxa exhibited high prevalence and moderate to high abundance (Fig. 1B), supporting their ecological importance. Furthermore, core genera showed significantly lower variability in abundance compared with non-core taxa (Fig. 1C), indicating stable and consistent representation across samples. The complete list of core genera identified across continents is provided in Supplementary Table 2A.

**Figure 1:**
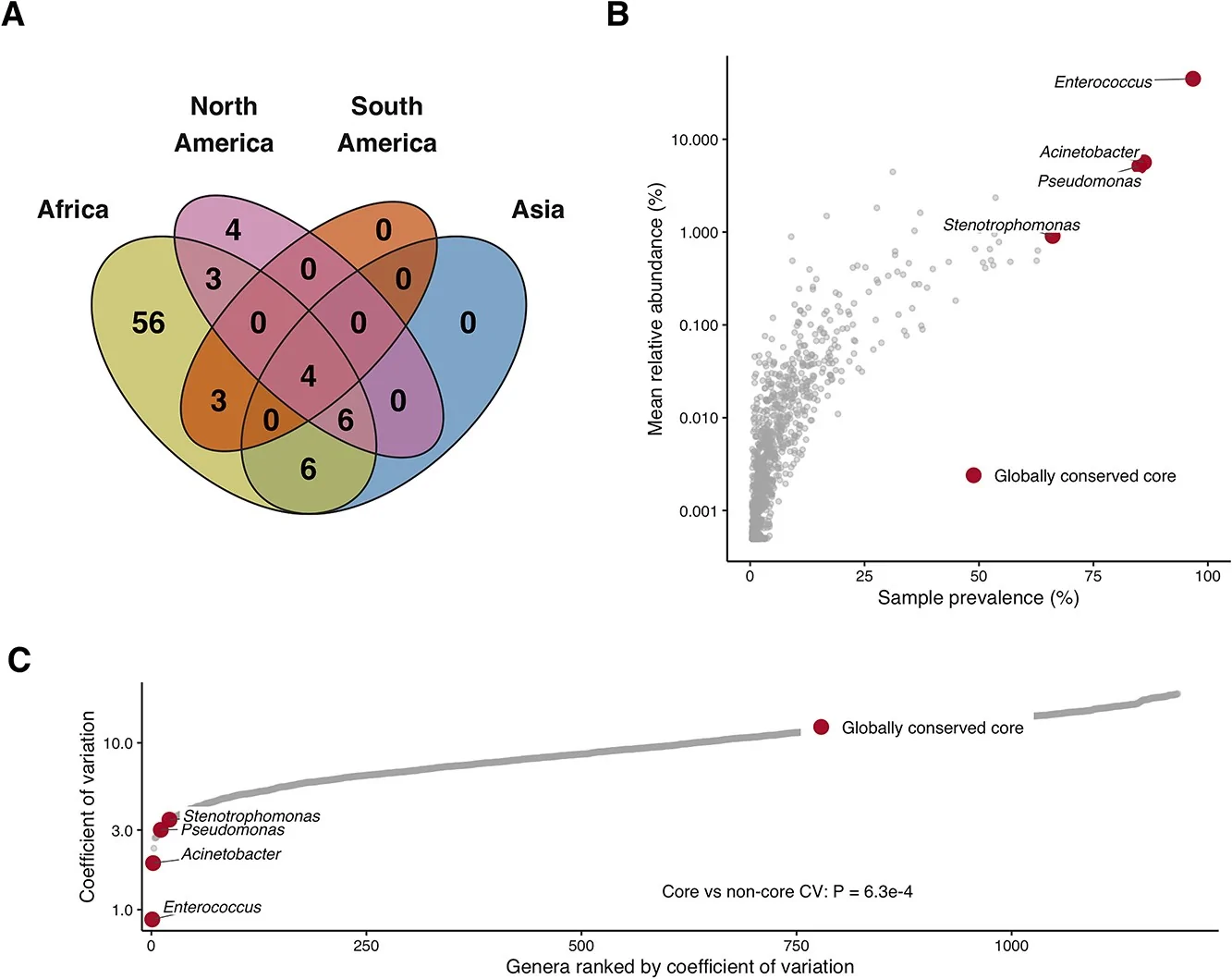
Identification and characterization of a globally conserved core gut microbiota in *S. frugiperda*. (A) Occurrence-based analysis identified bacterial genera consistently present across continents (Africa, Asia, North America, and South America). A genus was considered a continent-level core member if detected in ≥70% of samples within a continent, and those shared across all continents were defined as globally conserved core genera. (B) Prevalence–abundance relationship of bacterial genera. Each point represents a genus, with prevalence (percentage of samples) on the x-axis and mean relative abundance (log10 scale) on the y-axis. Globally conserved core genera are highlighted, indicating both high prevalence and consistent abundance across samples. (C) Variability in abundance of detected genera, measured as coefficient of variation (CV). Genera are ranked from lowest to highest variability. Globally conserved core genera exhibit lower variability compared with non-core taxa, indicating stable representation across diverse samples.

To identify a core microbiota independent of geography or diet, we applied five unsupervised clustering approaches—k-means, hierarchical clustering, Gaussian mixture models, DBSCAN, and spectral clustering—to ASV-level abundance data. ASVs identified by at least three of the five methods were defined as consensus core members. This approach yielded 88 high-confidence ASVs (Supplementary Table 2B), taxonomically classified to the finest possible resolution. These consensus ASVs were further summarized at higher taxonomic levels (Supplementary Fig. 1). The resulting core microbiota included dominant taxa such as *Enterococcus, Pseudomonas*, and *Acinetobacter*, which were also identified in the occurrence-based analysis. The strong overlap between the consensus clustering and occurrence-based approaches supports the robustness of the identified core microbiota and provides consistent evidence for a globally conserved bacterial component in the *S. frugiperda* gut.

Diet was also evaluated as a potential driver of FAW gut community structure. Due to the limited sample size and/or incomplete metadata related to diet for most studies, we focused on *Zea mays* (corn) as the most consistently represented and comparable diet. To examine microbiota composition associated with maize, we conducted an occurrence-based core microbiota analysis restricted to maize-fed *S. frugiperda*. At the genus level (Supplementary Fig. 2A), several bacterial taxa previously identified as globally conserved—*Pseudomonas, Enterococcus*, and *Acinetobacter*—remained part of the core microbiota in maize-fed individuals, reinforcing that these lineages are the backbone of the FAW gut microbiota regardless of diet. Notably, taxa within *Allorhizobium–Neorhizobium–Pararhizobium–Rhizobium* were identified as core members in maize-fed samples but were not part of the global or continent-level core communities, suggesting a diet-specific microbial association.

### FAW gut bacterial communities exhibit strong geographical signatures

To assess geographic variation in FAW gut bacterial communities, we observed significant differences in both alpha diversity and community composition between continents. Shannon diversity and observed ASV richness differed among geographic groups (Kruskal–Wallis test, *p_shannon_ = 0.000408* and *p_observed_ = 1.96 × 10^−9^*; Fig. 2A–B). These patterns were further supported by random forest classification models, which achieved high predictive performance (AUC ≈ 1.0), demonstrating strong and distinct geographic signatures in gut microbial profiles (Fig. 2D). Beta diversity analysis using Aitchison distance revealed clear clustering of samples by continent (Fig. 2E), indicating that microbial community composition differs systematically across geographic regions. PERMANOVA confirmed a significant effect of geography on microbiota structure (*R^2^ =* 0.128*, P = .*001).

**Figure 2:**
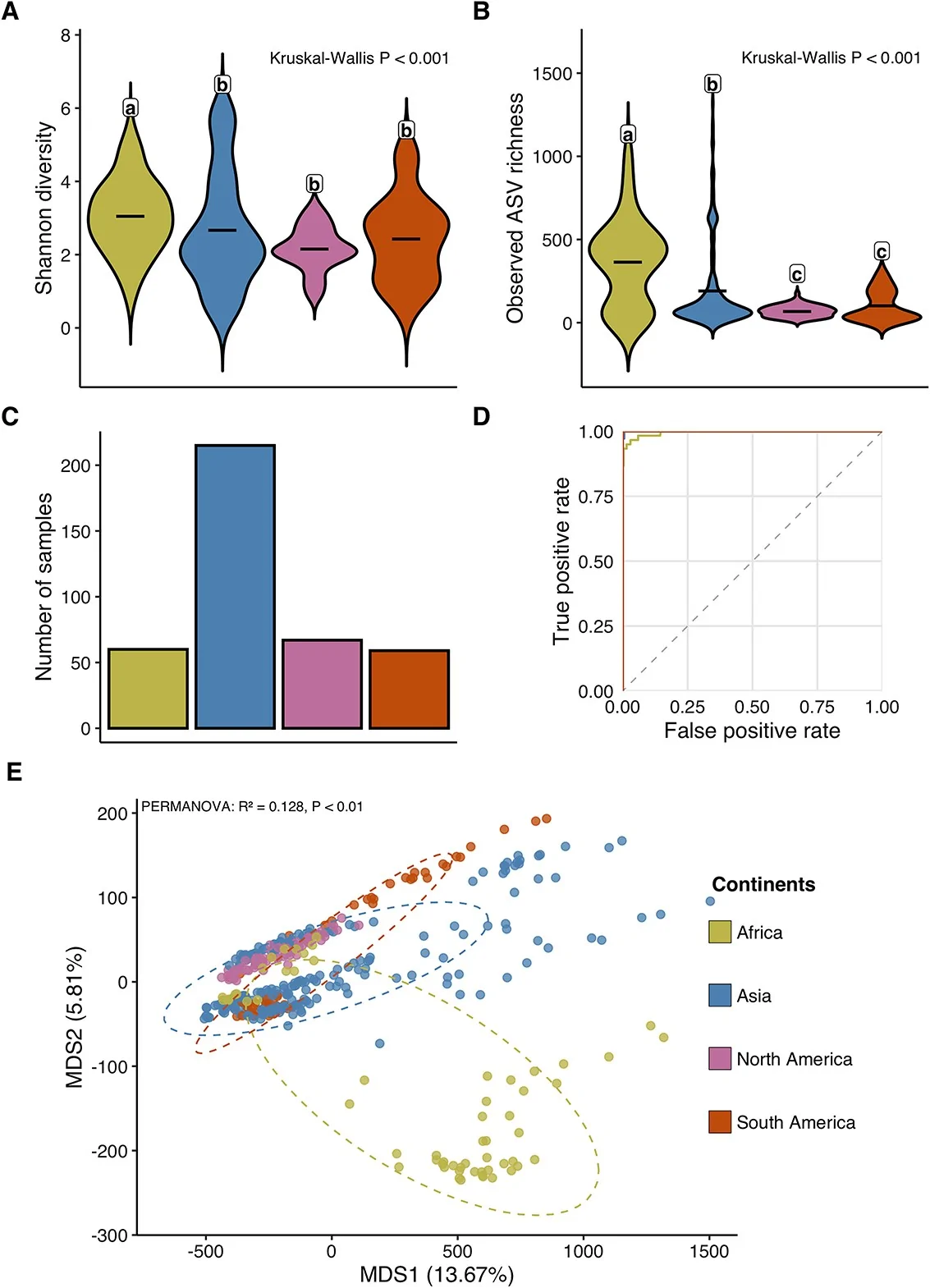
Geographic structuring of gut bacterial diversity and composition in *S. frugiperda.* (A–B) Alpha diversity across continents, shown as Shannon diversity (A) and observed ASV richness (B). Statistical significance was assessed using the Kruskal–Wallis test, with distinct letters indicating significant differences among groups. (C) Distribution of sample counts across continents. (D) Performance of one-vs-all random forest classification models predicting continent of origin from ASV abundance profiles. Model performance is shown as area under the ROC curve (AUC). (E) Aitchison distance-based ordination (MDS) showing clustering of samples by continent. Statistical significance was assessed using PERMANOVA, with R^2^ and p-values indicating the proportion of variance explained by geographic factors.

To further investigate how microbial communities are structured across geographic regions, we constructed bipartite networks based on ASV sharing among samples. Network analysis revealed distinct clustering of samples by continent and country (Fig. 3A–B), indicating that microbial communities are structured according to geographic origin and share region-specific associations. The ASVs included in the network analysis and their corresponding taxonomic annotations are provided in Supplementary Table 3. Consistent with these structural patterns, differential abundance analysis using ANCOM-BC identified several bacterial genera that were significantly enriched across geographic regions (Fig. 3C–D). These results indicate that geographic variation in FAW gut microbiota is driven by shifts in specific bacterial taxa, rather than uniform changes across the community.

**Figure 3:**
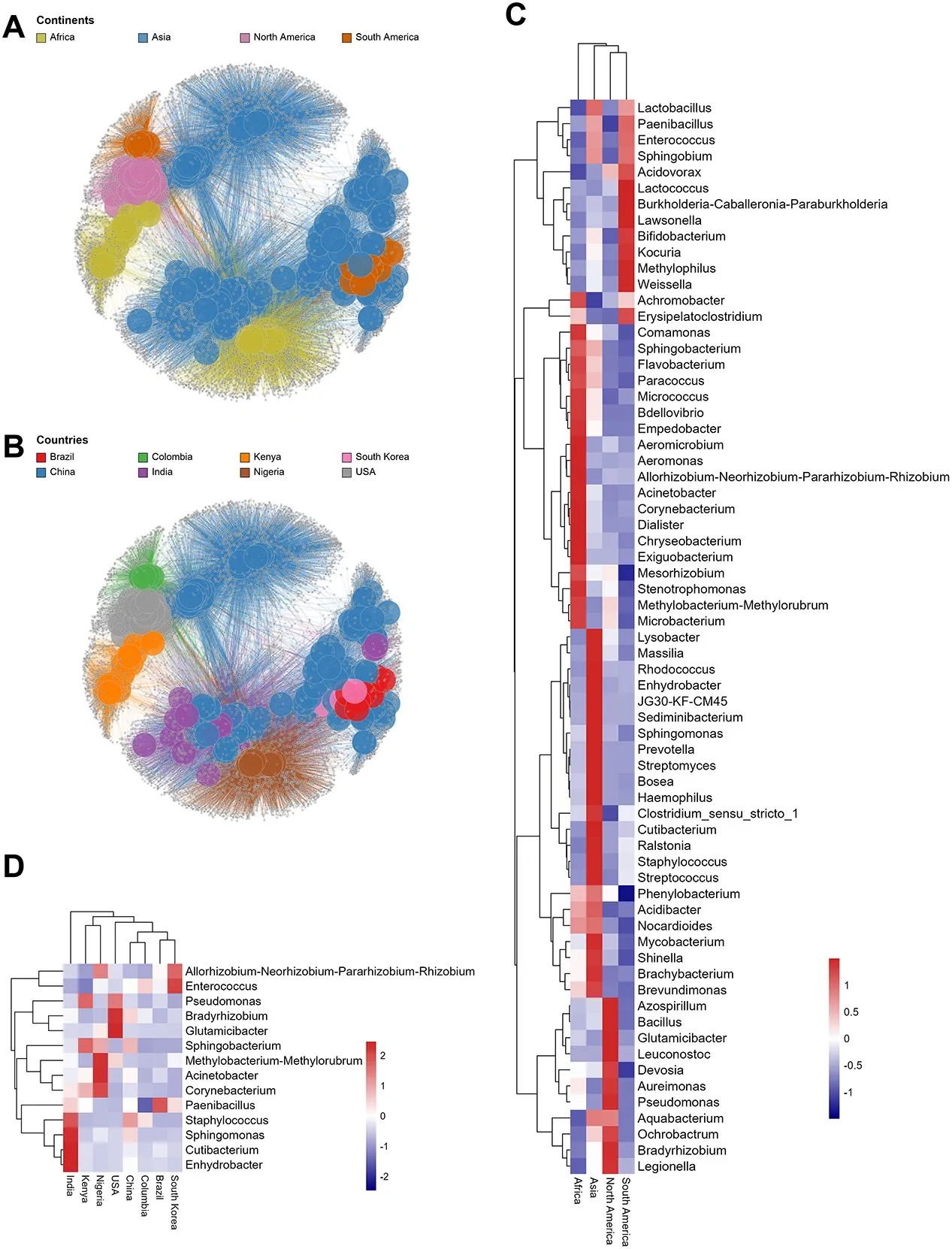
Geographic structuring of *S. frugiperda* gut microbiota revealed by network analysis and differential abundance patterns. (A–B) Bipartite networks representing ASV sharing among samples at the continent level (A) and country level (B). Nodes represent samples and ASVs, and edges indicate presence of ASVs within samples. Samples cluster according to geographic origin, indicating structured microbial communities and region-specific associations. (C–D) Heatmaps of differentially abundant bacterial genera identified using ANCOM-BC at the continent level (C) and country level (D). Color scale represents centered log-fold change values, with positive values indicating enrichment in each region. These results demonstrate that geographic variation in microbiota is driven by shifts in specific bacterial taxa.

Similar patterns were observed at the country level. Alpha and beta diversity analyses showed significant variation among countries (Supplementary Fig. 3A–B, C), and classification models achieved high predictive accuracy (AUC ≈ 1.0; Supplementary Fig. 3D). Together, these findings demonstrate that both community structure and taxon-specific differences contribute to strong geographic signatures in the FAW gut microbiota. These geographically enriched taxa likely reflect differences in environmental microbial reservoirs, agricultural practices, host plant-associated microbiota, or climate-driven selection pressures [22, 27, 29, 65, 66].

### Geographic differences in maize-fed FAW populations

Analysis of maize-fed FAW datasets showed that geographic variation persists even when diet is controlled. Alpha diversity differed significantly among countries (Kruskal–Wallis test*, p_shannon_ = 0.0435* and *p_observed_ = 0.747* for continent and *p_shannon_ = 6.08 × 10^−9^* and *p_observed_ = 3.51 × 10^−11^* for country; Supplementary Figs. 4–5), and beta diversity showed clustering by continent and country (Supplementary Figs. 4D and 5D). PERMANOVA confirmed a significant geographic effect (*R^2^ =* 0.315*, P =* .001).

### Within-continent effects of diet on FAW gut microbiota

Within continents, both diet and country influenced microbial community composition. Aitchison distance-based MDS ordination showed variation in microbial communities within South America, Asia, North America, and Africa (Fig. 4A–D), with samples exhibiting separation associated with both dietary category and country of origin. Alpha diversity also varied significantly by diet within each continent (Supplementary Fig. 6). Random forest models further demonstrated strong diet-associated microbial signatures, with AUC values exceeding 0.95 (Supplementary Fig. 7). PERMANOVA analysis performed on the subset of samples with diet metadata available showed that both geographic region and diet influenced microbial community composition, with geography explaining a slightly greater proportion of the variance (*R^2^ = 0.135* for country) compared with diet (*R^2^ = 0.119*).

**Figure 4:**
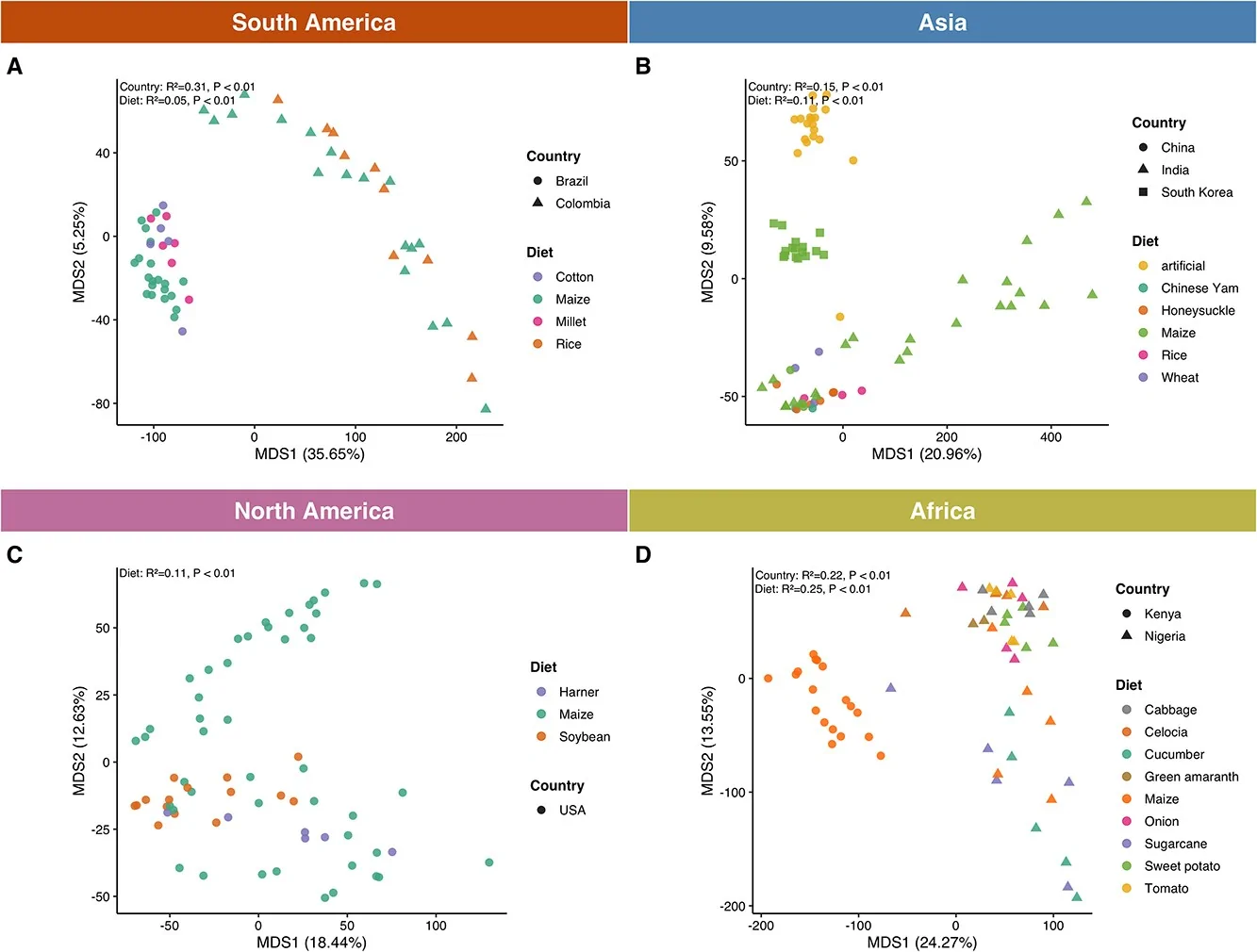
Effects of diet on gut microbiota composition within continents in *S. frugiperda*. (A–D) Aitchison distance-based ordination (MDS) showing microbial community composition within South America (A), Asia (B), North America (C), and Africa (D). Samples are colored by diet category and shaped by country of origin. Within each continent, samples exhibit variation associated with both diet and country, indicating that microbial community structure is influenced by dietary conditions even within a shared geographic background.

### Functional profiles of the FAW gut microbiome vary with geography

To assess the potential functional roles of the gut bacterial communities and their geographic variability, we performed predictive metagenomic profiling using PICRUSt2. Functional predictions were based on KO abundances inferred from 16S rRNA data and analyzed at both continent and country levels. Principal coordinates analysis of KO abundance profiles showed clear clustering of samples by continent (Fig. 5A), indicating that the predicted functional composition of the gut microbiota varied significantly with geographic origin. A similar clustering pattern was observed at the country level (Fig. 5B), supporting the idea that regional microbial assemblages also differ in their metabolic potential.

**Figure 5:**
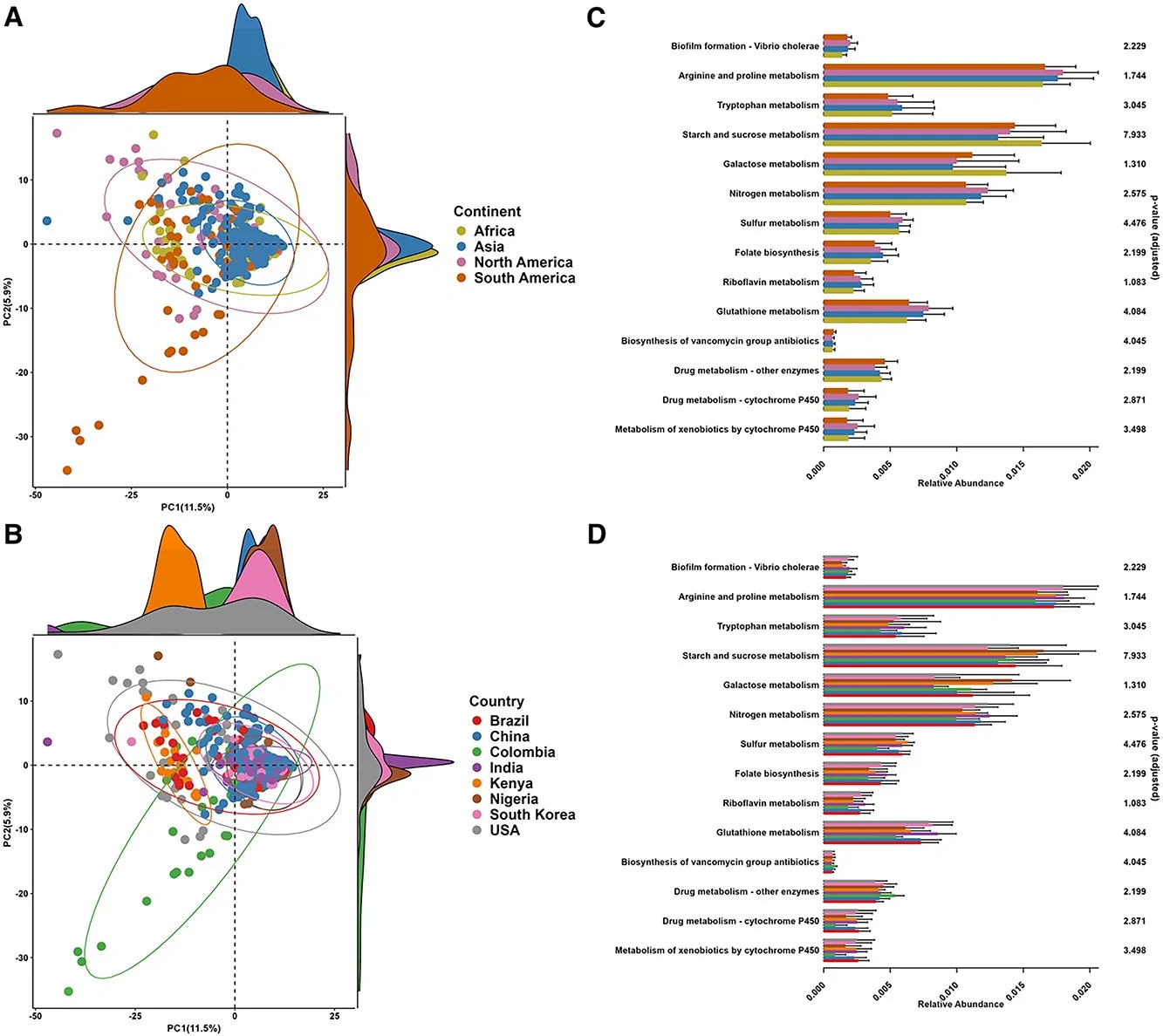
Predicted functional potential of *S. frugiperda* gut microbiota varies with geography. (A–B) PCA of predicted KO profiles based on Euclidean distances, showing clustering of samples by continent (A) and country (B). Data points are enclosed by 95% confidence ellipses, indicating separation of functional profiles across geographic groups. (C–D) Relative abundance of selected differentially enriched KEGG pathways across continents (C) and countries (D). Pathways shown are associated with key microbial functions, including xenobiotic metabolism, amino acid metabolism, and vitamin biosynthesis. Differential enrichment was assessed using ALDEx2 with Benjamini–Hochberg multiple testing correction.

A suite of KEGG pathways that were differentially abundant across continents (Fig. 5C) and countries (Fig. 5D) were identified. Several of the identified pathways are associated with detoxification, nutrient processing, and host–microbe interactions, which play critical roles in host resilience and adaptability. Notably, pathways such as “Drug metabolism – cytochrome P450,” “Metabolism of xenobiotics by cytochrome P450,” and “Glutathione metabolism” are implicated in insecticide resistance and detoxification of plant secondary compounds [20, 67, 68]. “Tryptophan metabolism” and “Arginine and proline metabolism” are linked to host immunity and development [69, 70], while “Biofilm formation – *Vibrio cholerae*” may reflect microbial colonization strategies or resilience under gut stress conditions [71]. Additional pathways such as “Starch and sucrose metabolism,” “Galactose metabolism,” “Folate biosynthesis,” and “Riboflavin metabolism” suggest a role for microbiota in nutrient acquisition and vitamin synthesis, which can be vital for host nutritional adaptation [72]. The presence of “Sulfur metabolism” and “Nitrogen metabolism” pathways suggest microbiota-mediated contributions to nitrogen recycling and elemental balance in nutrient-limited environments [22]. Furthermore, the enrichment of “Biosynthesis of vancomycin group antibiotics” suggests inter-microbial competition or microbial defense strategies in the gut ecosystem. These predicted functional patterns mirror the taxonomic clustering observed in earlier analyses (Fig. 5) and suggest that gut microbiota in FAW may be locally adapted to support host performance in geographically distinct environments. The presence of environmentally responsive functional traits supports the idea that microbial communities contribute to FAW’s ability to thrive across diverse geographies.

## Discussion

Fall armyworm (FAW), *S. frugiperda*, remains one of the most destructive and rapidly spreading agricultural pests worldwide, with continued expansion influenced by global trade, climate suitability, broad host range, and resistance to control strategies [1–4, 6–8]. Although these host-level traits are central to FAW invasion success, increasing evidence indicates that gut-associated microbial communities may also contribute to insect adaptation by influencing nutrition, detoxification, stress tolerance, and interactions with host plants [21–23]. In this study, we provide a global synthesis of the FAW gut microbiome and identify a conserved core microbiota that persists across broad geographic and dietary contexts, and a variable, geographically structured component that reflects local ecological and environmental conditions.

A major finding of this study is the identification of a conserved set of bacterial taxa associated with FAW guts across continents. Using occurrence-based analysis and consensus clustering, we identified core bacterial members including *Enterococcus, Pseudomonas*, and *Acinetobacter*, which were consistently detected in FAW collected from different regions. Previous studies have linked insect gut bacteria to nutrient metabolism, host development, detoxification, and environmental stress responses [23]. Therefore, the persistence of these bacterial genera across geographically diverse FAW populations suggests that they may contribute to basic host-associated functions that are maintained regardless of geographical or environmental differences. However, this study was reliant on published 16S rRNA amplicon data, thus these functional roles should be interpreted as potential functions rather than direct evidence of bacterial activity. Experimental validation using culture-based assays, metagenomics, metabolomics, or gnotobiotic insect systems are needed to determine if/how these core taxa directly contribute to FAW fitness, detoxification, or adaptation.

The presence of a conserved core microbiota does not imply that FAW gut communities are globally uniform. In contrast, we observed strong geographic structuring of gut bacterial communities at both continental and country levels. Alpha diversity, beta diversity, supervised classification, ASV-sharing networks, and differential abundance analyses all supported the presence of region-specific microbial signatures. These findings indicate that the FAW gut maintains a stable set of core bacterial members while also incorporating variable microbial taxa that differ among geographic regions. This distinction helps reconcile the two major patterns observed in this study: the gut microbiota contains conserved members that may support shared host functions, but the broader community is shaped by local geography. Thus, the core and variable components are not contradictory; rather, they suggest a flexible host–microbiome structure in which conserved taxa co-exist with environmentally responsive bacterial assemblages.

Several non-mutually exclusive mechanisms may explain the geographic differences observed in FAW gut microbiota. First, insects are continuously exposed to microorganisms from their surrounding environments, including on the host plant surface, soil, water, and local microbial reservoirs. Because these environmental microbial communities can vary across continents, climates, and agricultural systems, FAW larvae are likely exposed to distinct pools of microorganisms [65, 66, 73]. Second, localized conditions such as temperature, humidity, rainfall patterns, and seasonal climate may influence both the microbial communities available for colonization and the selective pressures acting within the insect gut [74, 75]. Third, agricultural practices, including cropping systems, pesticide exposure, and host plant availability, may further influence gut microbiota composition by altering microbial exposure and selecting for bacteria with particular metabolic traits [22, 27, 29]. Together, these factors provide a plausible ecological explanation for why FAW gut communities differ among continents and countries.

Host-associated factors may also contribute to geographic structuring of the FAW gut microbiome. FAW populations exhibit genetic differentiation across geographic regions and host-associated strains, and host genotype has been shown to influence insect gut microbiota composition [22, 26, 69]. Consequently, part of the geographic signal observed here may reflect differences in host genetic background in addition to variation in environmental microbial exposure. Notably, geographic differences persisted even when analyses were restricted to maize-fed FAW gut samples, suggesting that host plant alone does not fully account for the observed microbiome variation. Future studies integrating microbiome profiling with host genomic data and standardized sampling across regions will be important for disentangling the relative contributions of host genetics and environmental factors.

Within continents, microbial community structure differed among host plants, consistent with previous studies demonstrating that diet shapes insect gut microbiota through variation in nutrient composition, plant defensive chemistry, associated microbial communities, and digestibility [21, 26, 32]. Plant diet can shape gut microbiota through differences in nutrient composition, defensive chemistry, surface-associated microbes, and digestibility. In the maize-fed subset, we identified a core taxon within the *Allorhizobium–Neorhizobium–Pararhizobium–Rhizobium* group. Members of this bacterial complex are commonly associated with nitrogen cycling, and their repeated detection in maize-fed FAW gut samples suggests a potential diet-linked association that may be relevant under nitrogen-limited feeding conditions [22]. However, this interpretation remains speculative because 16S rRNA data cannot determine metabolic function or confirm nitrogen-cycling activity. Accordingly, this taxon should be regarded as a candidate maize-associated symbiont for future functional investigation.

The functional predictions further suggest that FAW gut bacterial communities may differ in metabolic potential across geographic regions. Predicted pathways related to xenobiotic metabolism, amino acid metabolism, glutathione metabolism, vitamin biosynthesis, and nutrient processing varied among continents and countries. These patterns are consistent with the possibility that regionally structured bacterial communities may contribute to local adaptation by influencing detoxification, nutrient acquisition, or stress tolerance. For example, enrichment of pathways associated with xenobiotic degradation may reflect differences in pesticide exposure or plant defensive chemistry across regions, while differences in nutrient metabolism pathways may reflect adaptation to local host plants or environmental conditions [20, 67, 68, 72, 73]. Nevertheless, PICRUSt2-based functional profiles represent predicted metabolic potential rather than directly measured gene expression or metabolite production. Consequently, additional research is still needed to validate the functional role of FAW-associated microbiota.

The identification of a stable core microbiota across geographically diverse FAW populations provides a framework for future microbiome-informed pest management strategies. As these bacterial taxa are consistently associated with FAW across geographic regions and diets, they represent promising candidates for functional investigation. Future work should focus on isolating representative strains, validating their contributions to host physiology and fitness, and evaluating interactions within simplified synthetic microbial communities (SynComs). Such studies may help determine whether manipulation of persistent microbial associates can be leveraged to reduce pest performance or improve biological control strategies. However, because this study is based on 16S rRNA-derived taxonomic profiles, the identified taxa should primarily be viewed as candidates for future experimental investigation rather than confirmed functional symbionts.

Overall findings from this study provide new insights into the global structure and ecological variability of the FAW gut microbiome. Several limitations should be considered when interpreting these results. The analysis integrated datasets generated using different primer sets, sequencing platforms, experimental protocols, and although standardized processing and phylogenetic placement were used to improve comparability, some residual batch effects may remain. Metadata availability also varied among studies, particularly for diet, host plant, developmental stage, and environmental variables. In addition, PICRUSt2-based functional predictions represent inferred metabolic potential rather than direct measurements of microbial activity. Despite these limitations, the consistency of the observed core microbiome and geographic patterns across multiple independent datasets is promising. Future studies incorporating standardized global sampling, host genotyping, shotgun metagenomics, metabolomics, bacterial isolation, and experimental validation will further refine our understanding of the ecological and functional roles of FAW-associated microbiota.

In conclusion, this study demonstrates that the FAW gut microbiome is shaped by both global conservation and local variation. The presence of a conserved core microbiota suggests that FAW maintains a stable bacterial community that may support fundamental host-associated functions. At the same time, strong geographic and diet-associated differences indicate that the broader gut community remains flexible and responsive to ecological influences. This dual structure—a stable core combined with a variable accessory microbiota—may contribute to the ecological success of FAW by preserving essential microbial associations while allowing local adaptation to different environments, host plants, and agricultural landscapes.

## Supplementary Material

Supplementary_Materials_ycag233

## Data Availability

This study utilized publicly available datasets obtained from previously published studies. All datasets analyzed during this study are cited in the manuscript, and the corresponding accession numbers and data sources are provided in Supplementary Table 1A. No new datasets were generated during the current study.
