# Supplementary material for: Global in silico analysis reveals a core gut microbiota in fall armyworm (Spodoptera frugiperda) shaped by geography and diet
Source: ISME Commun. 2026 Aug 22;6(1):ycag233. doi: 10.1093/ismeco/ycag233 (PMC13639100; doi:10.1093/ismeco/ycag233)
Supplement: Supplementary file 1 [file supplementary_materials_ycag233.docx]

**Supplementary Material**

*Global in silico analysis reveals a core gut microbiota in fall armyworm (Spodoptera frugiperda) shaped by geography and diet*

Lakshay Anand^1^ and Kendall R. Corbin^1^*

**Affiliations:**

^1^ Department of Horticulture, Martin-Gatton College of Agriculture, Food and Environment, University of Kentucky, Lexington, Kentucky 40546, USA

# Supplementary Figures

**Supplementary Figure 1. Taxonomic classification of consensus core microbiota identified using unsupervised clustering.** Bar plots showing the taxonomic distribution of the 88 high-confidence ASVs identified as core members using consensus clustering approaches. Taxa are summarized at higher taxonomic levels to illustrate the overall composition of the core microbiota.

**Supplementary Figure 2. Core gut microbiota of maize-fed *S. frugiperda*. Occurrence-based core microbiota identified in maize-fed samples**. Presence–absence heatmaps show core bacterial, where black indicates presence and gray indicates absence across samples. The colors used for continents are same throughout the manuscript. See Figure 2 for legend.

**Supplementary Figure 3. Geographic (country-level) variation in gut bacterial diversity of *S. frugiperda*.** (A–B) Alpha diversity across countries, shown as Shannon diversity (A) and observed ASV richness (B), with statistical significance assessed using the Kruskal–Wallis test. Distinct letters indicate significant differences among groups. (C) Distribution of sample counts across countries. (D) Aitchison distance-based ordination (MDS) showing clustering of samples by country. Statistical significance was assessed using PERMANOVA.

**Supplementary Figure 4. Geographic variation in gut microbiota of maize-fed *S. frugiperda* at the continent level.** (A–B) Alpha diversity across continents, shown as Shannon diversity (A) and observed ASV richness (B), with statistical significance assessed using the Kruskal–Wallis test. (C) Distribution of sample counts across continents. (D) Aitchison distance-based ordination (MDS) showing clustering of samples by continent. Statistical significance was assessed using PERMANOVA.

**Supplementary Figure 5. Geographic variation in gut microbiota of maize-fed *S. frugiperda* at the country level.** (A–B) Alpha diversity across countries, shown as Shannon diversity (A) and observed ASV richness (B), with statistical significance assessed using the Kruskal–Wallis test. (C) Distribution of sample counts across countries. (D) Aitchison distance-based ordination (MDS) showing clustering of samples by country. Statistical significance was assessed using PERMANOVA.

**Supplementary Figure 6. Effects of diet on gut bacterial diversity within continents.** (A–H) Alpha diversity (Shannon diversity and observed ASV richness) across different diet categories within North America (A–B), South America (C–D), Asia (E–F), and Africa (G–H). Statistical significance was assessed using the Kruskal–Wallis test.

**Supplementary Figure 7: Effect of various diets on fall armyworm gut bacterial composition determined using the Random Forest (RF).**

# Supplementary Tables

## Supplementary Table 1A

| **Sample ID** | **SRA Run ID** | **NCBI Project ID** | **Library Layout** | **Country** | **Continent** | **Location** | **Instrument** | **Diet** |
| --- | --- | --- | --- | --- | --- | --- | --- | --- |
| CE1_S383 | none | NA001 | PE250 | Columbia | South America | Columbia | Illumina MiSeq | Maize |
| CE2_S384 | none | NA001 | PE250 | Columbia | South America | Columbia | Illumina MiSeq | Maize |
| CE3_S385 | none | NA001 | PE250 | Columbia | South America | Columbia | Illumina MiSeq | Maize |
| CE4_S386 | none | NA001 | PE250 | Columbia | South America | Columbia | Illumina MiSeq | Maize |
| CFA1_S374 | none | NA001 | PE250 | Columbia | South America | Columbia | Illumina MiSeq | Maize |
| CFA2_S375 | none | NA001 | PE250 | Columbia | South America | Columbia | Illumina MiSeq | Maize |
| CFA3_S376 | none | NA001 | PE250 | Columbia | South America | Columbia | Illumina MiSeq | Maize |
| CLL1_S365 | none | NA001 | PE250 | Columbia | South America | Columbia | Illumina MiSeq | Maize |
| CLL2_S366 | none | NA001 | PE250 | Columbia | South America | Columbia | Illumina MiSeq | Maize |
| CLL3_S367 | none | NA001 | PE250 | Columbia | South America | Columbia | Illumina MiSeq | Maize |
| CMA3_S372 | none | NA001 | PE250 | Columbia | South America | Columbia | Illumina MiSeq | Maize |
| CMA5_S373 | none | NA001 | PE250 | Columbia | South America | Columbia | Illumina MiSeq | Maize |
| CP2_S368 | none | NA001 | PE250 | Columbia | South America | Columbia | Illumina MiSeq | Maize |
| CP3_S369 | none | NA001 | PE250 | Columbia | South America | Columbia | Illumina MiSeq | Maize |
| CP5_S380 | none | NA001 | PE250 | Columbia | South America | Columbia | Illumina MiSeq | Maize |
| CP6_S381 | none | NA001 | PE250 | Columbia | South America | Columbia | Illumina MiSeq | Maize |
| CP7_S382 | none | NA001 | PE250 | Columbia | South America | Columbia | Illumina MiSeq | Maize |
| CYL2_S362 | none | NA001 | PE250 | Columbia | South America | Columbia | Illumina MiSeq | Maize |
| RE1_S387 | none | NA001 | PE250 | Columbia | South America | Columbia | Illumina MiSeq | Rice |
| RE2_S388 | none | NA001 | PE250 | Columbia | South America | Columbia | Illumina MiSeq | Rice |
| RE3_S389 | none | NA001 | PE250 | Columbia | South America | Columbia | Illumina MiSeq | Rice |
| RLL1_S377 | none | NA001 | PE250 | Columbia | South America | Columbia | Illumina MiSeq | Rice |
| RLL2_S378 | none | NA001 | PE250 | Columbia | South America | Columbia | Illumina MiSeq | Rice |
| RLL3_S379 | none | NA001 | PE250 | Columbia | South America | Columbia | Illumina MiSeq | Rice |
| RP1_S370 | none | NA001 | PE250 | Columbia | South America | Columbia | Illumina MiSeq | Rice |
| RP2_S371 | none | NA001 | PE250 | Columbia | South America | Columbia | Illumina MiSeq | Rice |
| RYL1_S363 | none | NA001 | PE250 | Columbia | South America | Columbia | Illumina MiSeq | Rice |
| RYL2_S364 | none | NA001 | PE250 | Columbia | South America | Columbia | Illumina MiSeq | Rice |
| SRR10738524_PRJNA595886 | SRR10738524 | PRJNA595886 | PE250 | Brazil | South America | Brazil: Rio Verde, GO | Illumina MiSeq | Maize |
| SRR10738525_PRJNA595886 | SRR10738525 | PRJNA595886 | PE250 | Brazil | South America | Brazil: Rio Verde, GO | Illumina MiSeq | Maize |
| SRR10738526_PRJNA595886 | SRR10738526 | PRJNA595886 | PE250 | Brazil | South America | Brazil: Rio Verde, GO | Illumina MiSeq | Maize |
| SRR10738527_PRJNA595886 | SRR10738527 | PRJNA595886 | PE250 | Brazil | South America | Brazil: So Miguel do Oeste, SC | Illumina MiSeq | Maize |
| SRR10738528_PRJNA595886 | SRR10738528 | PRJNA595886 | PE250 | Brazil | South America | Brazil: So Miguel do Oeste, SC | Illumina MiSeq | Maize |
| SRR10738529_PRJNA595886 | SRR10738529 | PRJNA595886 | PE250 | Brazil | South America | Brazil: So Miguel do Oeste, SC | Illumina MiSeq | Maize |
| SRR10738531_PRJNA595886 | SRR10738531 | PRJNA595886 | PE250 | Brazil | South America | Brazil: Londrina, PR | Illumina MiSeq | Maize |
| SRR10738532_PRJNA595886 | SRR10738532 | PRJNA595886 | PE250 | Brazil | South America | Brazil: Londrina, PR | Illumina MiSeq | Maize |
| SRR10738533_PRJNA595886 | SRR10738533 | PRJNA595886 | PE250 | Brazil | South America | Brazil: Londrina, PR | Illumina MiSeq | Maize |
| SRR10738534_PRJNA595886 | SRR10738534 | PRJNA595886 | PE250 | Brazil | South America | Brazil: Piracicaba, SP | Illumina MiSeq | Maize |
| SRR10738536_PRJNA595886 | SRR10738536 | PRJNA595886 | PE250 | Brazil | South America | Brazil: Piracicaba, SP | Illumina MiSeq | Maize |
| SRR10738537_PRJNA595886 | SRR10738537 | PRJNA595886 | PE250 | Brazil | South America | Brazil: Viosa, MG | Illumina MiSeq | Maize |
| SRR10738538_PRJNA595886 | SRR10738538 | PRJNA595886 | PE250 | Brazil | South America | Brazil: Viosa, MG | Illumina MiSeq | Maize |
| SRR10738539_PRJNA595886 | SRR10738539 | PRJNA595886 | PE250 | Brazil | South America | Brazil: Viosa, MG | Illumina MiSeq | Maize |
| SRR12391180_PRJNA655399 | SRR12391180 | PRJNA655399 | PE250 | China | Asia | China | Illumina HiSeq 2500 | Unknown |
| SRR12508070_PRJNA658986 | SRR12508070 | PRJNA658986 | PE300 | USA | North America | USA: Pennyslvania State College | Illumina MiSeq | Maize |
| SRR12508071_PRJNA658986 | SRR12508071 | PRJNA658986 | PE300 | USA | North America | USA: Pennyslvania State College | Illumina MiSeq | Maize |
| SRR12508075_PRJNA658986 | SRR12508075 | PRJNA658986 | PE300 | USA | North America | USA: Pennyslvania State College | Illumina MiSeq | Maize |
| SRR12508086_PRJNA658986 | SRR12508086 | PRJNA658986 | PE300 | USA | North America | USA: Pennyslvania State College | Illumina MiSeq | Maize |
| SRR12508097_PRJNA658986 | SRR12508097 | PRJNA658986 | PE300 | USA | North America | USA: Pennyslvania State College | Illumina MiSeq | Maize |
| SRR12508108_PRJNA658986 | SRR12508108 | PRJNA658986 | PE300 | USA | North America | USA: Pennyslvania State College | Illumina MiSeq | Maize |
| SRR12508119_PRJNA658986 | SRR12508119 | PRJNA658986 | PE300 | USA | North America | USA: Pennyslvania State College | Illumina MiSeq | Maize |
| SRR12508130_PRJNA658986 | SRR12508130 | PRJNA658986 | PE300 | USA | North America | USA: Pennyslvania State College | Illumina MiSeq | Maize |
| SRR12508133_PRJNA658986 | SRR12508133 | PRJNA658986 | PE300 | USA | North America | USA: Pennyslvania State College | Illumina MiSeq | Maize |
| SRR12508134_PRJNA658986 | SRR12508134 | PRJNA658986 | PE300 | USA | North America | USA: Pennyslvania State College | Illumina MiSeq | Maize |
| SRR12508135_PRJNA658986 | SRR12508135 | PRJNA658986 | PE300 | USA | North America | USA: Pennyslvania State College | Illumina MiSeq | Maize |
| SRR12508136_PRJNA658986 | SRR12508136 | PRJNA658986 | PE300 | USA | North America | USA: Pennyslvania State College | Illumina MiSeq | Maize |
| SRR12508137_PRJNA658986 | SRR12508137 | PRJNA658986 | PE300 | USA | North America | USA: Pennyslvania State College | Illumina MiSeq | Maize |
| SRR12508138_PRJNA658986 | SRR12508138 | PRJNA658986 | PE300 | USA | North America | USA: Pennyslvania State College | Illumina MiSeq | Maize |
| SRR12508139_PRJNA658986 | SRR12508139 | PRJNA658986 | PE300 | USA | North America | USA: Pennyslvania State College | Illumina MiSeq | Maize |
| SRR12508140_PRJNA658986 | SRR12508140 | PRJNA658986 | PE300 | USA | North America | USA: Pennyslvania State College | Illumina MiSeq | Maize |
| SRR12508141_PRJNA658986 | SRR12508141 | PRJNA658986 | PE300 | USA | North America | USA: Pennyslvania State College | Illumina MiSeq | Maize |
| SRR12508142_PRJNA658986 | SRR12508142 | PRJNA658986 | PE300 | USA | North America | USA: Pennyslvania State College | Illumina MiSeq | Maize |
| SRR13171621_PRJNA681731 | SRR13171621 | PRJNA681731 | PE300 | USA | North America | USA: Pennsylvania | Illumina MiSeq | Maize |
| SRR13171622_PRJNA681731 | SRR13171622 | PRJNA681731 | PE300 | USA | North America | USA: Pennsylvania | Illumina MiSeq | Maize |
| SRR13171625_PRJNA681731 | SRR13171625 | PRJNA681731 | PE300 | USA | North America | USA: Pennsylvania | Illumina MiSeq | Maize |
| SRR13171626_PRJNA681731 | SRR13171626 | PRJNA681731 | PE300 | USA | North America | USA: Pennsylvania | Illumina MiSeq | Maize |
| SRR14730741_PRJNA734744 | SRR14730741 | PRJNA734744 | PE250 | Nigeria | Africa | Nigeria: Ibadan | Illumina NovaSeq 6000 | Cucumber |
| SRR14730742_PRJNA734744 | SRR14730742 | PRJNA734744 | PE250 | Nigeria | Africa | Nigeria: Ibadan | Illumina NovaSeq 6000 | Cucumber |
| SRR14730743_PRJNA734744 | SRR14730743 | PRJNA734744 | PE250 | Nigeria | Africa | Nigeria: Ibadan | Illumina NovaSeq 6000 | Cucumber |
| SRR14730744_PRJNA734744 | SRR14730744 | PRJNA734744 | PE250 | Nigeria | Africa | Nigeria: Ibadan | Illumina NovaSeq 6000 | Cucumber |
| SRR14730745_PRJNA734744 | SRR14730745 | PRJNA734744 | PE250 | Nigeria | Africa | Nigeria: Ibadan | Illumina NovaSeq 6000 | Cucumber |
| SRR14730746_PRJNA734744 | SRR14730746 | PRJNA734744 | PE250 | Nigeria | Africa | Nigeria: Ibadan | Illumina NovaSeq 6000 | Celocia |
| SRR14730747_PRJNA734744 | SRR14730747 | PRJNA734744 | PE250 | Nigeria | Africa | Nigeria: Ibadan | Illumina NovaSeq 6000 | Celocia |
| SRR14730748_PRJNA734744 | SRR14730748 | PRJNA734744 | PE250 | Nigeria | Africa | Nigeria: Ibadan | Illumina NovaSeq 6000 | Celocia |
| SRR14730749_PRJNA734744 | SRR14730749 | PRJNA734744 | PE250 | Nigeria | Africa | Nigeria: Ibadan | Illumina NovaSeq 6000 | Celocia |
| SRR14730750_PRJNA734744 | SRR14730750 | PRJNA734744 | PE250 | Nigeria | Africa | Nigeria: Ibadan | Illumina NovaSeq 6000 | Celocia |
| SRR14730751_PRJNA734744 | SRR14730751 | PRJNA734744 | PE250 | Nigeria | Africa | Nigeria: Ibadan | Illumina NovaSeq 6000 | Sugarcane |
| SRR14730752_PRJNA734744 | SRR14730752 | PRJNA734744 | PE250 | Nigeria | Africa | Nigeria: Ibadan | Illumina NovaSeq 6000 | Onion |
| SRR14730753_PRJNA734744 | SRR14730753 | PRJNA734744 | PE250 | Nigeria | Africa | Nigeria: Ibadan | Illumina NovaSeq 6000 | Onion |
| SRR14730754_PRJNA734744 | SRR14730754 | PRJNA734744 | PE250 | Nigeria | Africa | Nigeria: Ibadan | Illumina NovaSeq 6000 | Onion |
| SRR14730755_PRJNA734744 | SRR14730755 | PRJNA734744 | PE250 | Nigeria | Africa | Nigeria: Ibadan | Illumina NovaSeq 6000 | Onion |
| SRR14730756_PRJNA734744 | SRR14730756 | PRJNA734744 | PE250 | Nigeria | Africa | Nigeria: Ibadan | Illumina NovaSeq 6000 | Onion |
| SRR14730757_PRJNA734744 | SRR14730757 | PRJNA734744 | PE250 | Nigeria | Africa | Nigeria: Ibadan | Illumina NovaSeq 6000 | Sweet potato |
| SRR14730758_PRJNA734744 | SRR14730758 | PRJNA734744 | PE250 | Nigeria | Africa | Nigeria: Ibadan | Illumina NovaSeq 6000 | Sweet potato |
| SRR14730759_PRJNA734744 | SRR14730759 | PRJNA734744 | PE250 | Nigeria | Africa | Nigeria: Ibadan | Illumina NovaSeq 6000 | Sweet potato |
| SRR14730760_PRJNA734744 | SRR14730760 | PRJNA734744 | PE250 | Nigeria | Africa | Nigeria: Ibadan | Illumina NovaSeq 6000 | Sweet potato |
| SRR14730761_PRJNA734744 | SRR14730761 | PRJNA734744 | PE250 | Nigeria | Africa | Nigeria: Ibadan | Illumina NovaSeq 6000 | Sweet potato |
| SRR14730762_PRJNA734744 | SRR14730762 | PRJNA734744 | PE250 | Nigeria | Africa | Nigeria: Ibadan | Illumina NovaSeq 6000 | Sugarcane |
| SRR14730763_PRJNA734744 | SRR14730763 | PRJNA734744 | PE250 | Nigeria | Africa | Nigeria: Ibadan | Illumina NovaSeq 6000 | Tomato |
| SRR14730764_PRJNA734744 | SRR14730764 | PRJNA734744 | PE250 | Nigeria | Africa | Nigeria: Ibadan | Illumina NovaSeq 6000 | Tomato |
| SRR14730765_PRJNA734744 | SRR14730765 | PRJNA734744 | PE250 | Nigeria | Africa | Nigeria: Ibadan | Illumina NovaSeq 6000 | Tomato |
| SRR14730766_PRJNA734744 | SRR14730766 | PRJNA734744 | PE250 | Nigeria | Africa | Nigeria: Ibadan | Illumina NovaSeq 6000 | Tomato |
| SRR14730767_PRJNA734744 | SRR14730767 | PRJNA734744 | PE250 | Nigeria | Africa | Nigeria: Ibadan | Illumina NovaSeq 6000 | Tomato |
| SRR14730769_PRJNA734744 | SRR14730769 | PRJNA734744 | PE250 | Nigeria | Africa | Nigeria: Ibadan | Illumina NovaSeq 6000 | Green amaranth |
| SRR14730772_PRJNA734744 | SRR14730772 | PRJNA734744 | PE250 | Nigeria | Africa | Nigeria: Ibadan | Illumina NovaSeq 6000 | Green amaranth |
| SRR14730773_PRJNA734744 | SRR14730773 | PRJNA734744 | PE250 | Nigeria | Africa | Nigeria: Ibadan | Illumina NovaSeq 6000 | Sugarcane |
| SRR14730774_PRJNA734744 | SRR14730774 | PRJNA734744 | PE250 | Nigeria | Africa | Nigeria: Ibadan | Illumina NovaSeq 6000 | Cabbage |
| SRR14730775_PRJNA734744 | SRR14730775 | PRJNA734744 | PE250 | Nigeria | Africa | Nigeria: Ibadan | Illumina NovaSeq 6000 | Cabbage |
| SRR14730776_PRJNA734744 | SRR14730776 | PRJNA734744 | PE250 | Nigeria | Africa | Nigeria: Ibadan | Illumina NovaSeq 6000 | Cabbage |
| SRR14730777_PRJNA734744 | SRR14730777 | PRJNA734744 | PE250 | Nigeria | Africa | Nigeria: Ibadan | Illumina NovaSeq 6000 | Cabbage |
| SRR14730778_PRJNA734744 | SRR14730778 | PRJNA734744 | PE250 | Nigeria | Africa | Nigeria: Ibadan | Illumina NovaSeq 6000 | Cabbage |
| SRR14730779_PRJNA734744 | SRR14730779 | PRJNA734744 | PE250 | Nigeria | Africa | Nigeria: Ibadan | Illumina NovaSeq 6000 | Maize |
| SRR14730780_PRJNA734744 | SRR14730780 | PRJNA734744 | PE250 | Nigeria | Africa | Nigeria: Ibadan | Illumina NovaSeq 6000 | Maize |
| SRR14730781_PRJNA734744 | SRR14730781 | PRJNA734744 | PE250 | Nigeria | Africa | Nigeria: Ibadan | Illumina NovaSeq 6000 | Maize |
| SRR14730782_PRJNA734744 | SRR14730782 | PRJNA734744 | PE250 | Nigeria | Africa | Nigeria: Ibadan | Illumina NovaSeq 6000 | Maize |
| SRR14730783_PRJNA734744 | SRR14730783 | PRJNA734744 | PE250 | Nigeria | Africa | Nigeria: Ibadan | Illumina NovaSeq 6000 | Maize |
| SRR14730784_PRJNA734744 | SRR14730784 | PRJNA734744 | PE250 | Nigeria | Africa | Nigeria: Ibadan | Illumina NovaSeq 6000 | Sugarcane |
| SRR14730785_PRJNA734744 | SRR14730785 | PRJNA734744 | PE250 | Nigeria | Africa | Nigeria: Ibadan | Illumina NovaSeq 6000 | Sugarcane |
| SRR17286886_PRJNA790707 | SRR17286886 | PRJNA790707 | PE300 | China | Asia | China: Yangling | Illumina MiSeq | Unknown |
| SRR17286887_PRJNA790707 | SRR17286887 | PRJNA790707 | PE300 | China | Asia | China: Yangling | Illumina MiSeq | Unknown |
| SRR17286888_PRJNA790707 | SRR17286888 | PRJNA790707 | PE300 | China | Asia | China: Yangling | Illumina MiSeq | Unknown |
| SRR17286889_PRJNA790707 | SRR17286889 | PRJNA790707 | PE300 | China | Asia | China: Yangling | Illumina MiSeq | Unknown |
| SRR17286890_PRJNA790707 | SRR17286890 | PRJNA790707 | PE300 | China | Asia | China: Yangling | Illumina MiSeq | Unknown |
| SRR17286891_PRJNA790707 | SRR17286891 | PRJNA790707 | PE300 | China | Asia | China: Yangling | Illumina MiSeq | Unknown |
| SRR17286895_PRJNA790707 | SRR17286895 | PRJNA790707 | PE300 | China | Asia | China: Yangling | Illumina MiSeq | Unknown |
| SRR17286896_PRJNA790707 | SRR17286896 | PRJNA790707 | PE300 | China | Asia | China: Yangling | Illumina MiSeq | Unknown |
| SRR17286897_PRJNA790707 | SRR17286897 | PRJNA790707 | PE300 | China | Asia | China: Yangling | Illumina MiSeq | Unknown |
| SRR17286898_PRJNA790707 | SRR17286898 | PRJNA790707 | PE300 | China | Asia | China: Yangling | Illumina MiSeq | Unknown |
| SRR17286900_PRJNA790707 | SRR17286900 | PRJNA790707 | PE300 | China | Asia | China: Yangling | Illumina MiSeq | Unknown |
| SRR17286901_PRJNA790707 | SRR17286901 | PRJNA790707 | PE300 | China | Asia | China: Yangling | Illumina MiSeq | Unknown |
| SRR17286902_PRJNA790707 | SRR17286902 | PRJNA790707 | PE300 | China | Asia | China: Yangling | Illumina MiSeq | Unknown |
| SRR17286903_PRJNA790707 | SRR17286903 | PRJNA790707 | PE300 | China | Asia | China: Yangling | Illumina MiSeq | Unknown |
| SRR17286904_PRJNA790707 | SRR17286904 | PRJNA790707 | PE300 | China | Asia | China: Yangling | Illumina MiSeq | Unknown |
| SRR17286905_PRJNA790707 | SRR17286905 | PRJNA790707 | PE300 | China | Asia | China: Yangling | Illumina MiSeq | Unknown |
| SRR17286907_PRJNA790707 | SRR17286907 | PRJNA790707 | PE300 | China | Asia | China: Yangling | Illumina MiSeq | Unknown |
| SRR17286908_PRJNA790707 | SRR17286908 | PRJNA790707 | PE300 | China | Asia | China: Yangling | Illumina MiSeq | Unknown |
| SRR17286909_PRJNA790707 | SRR17286909 | PRJNA790707 | PE300 | China | Asia | China: Yangling | Illumina MiSeq | Unknown |
| SRR17286910_PRJNA790707 | SRR17286910 | PRJNA790707 | PE300 | China | Asia | China: Yangling | Illumina MiSeq | Unknown |
| SRR17286911_PRJNA790707 | SRR17286911 | PRJNA790707 | PE300 | China | Asia | China: Yangling | Illumina MiSeq | Unknown |
| SRR17286912_PRJNA790707 | SRR17286912 | PRJNA790707 | PE300 | China | Asia | China: Yangling | Illumina MiSeq | Unknown |
| SRR17286913_PRJNA790707 | SRR17286913 | PRJNA790707 | PE300 | China | Asia | China: Yangling | Illumina MiSeq | Unknown |
| SRR17286914_PRJNA790707 | SRR17286914 | PRJNA790707 | PE300 | China | Asia | China: Yangling | Illumina MiSeq | Unknown |
| SRR17286915_PRJNA790707 | SRR17286915 | PRJNA790707 | PE300 | China | Asia | China: Yangling | Illumina MiSeq | Unknown |
| SRR17286916_PRJNA790707 | SRR17286916 | PRJNA790707 | PE300 | China | Asia | China: Yangling | Illumina MiSeq | Unknown |
| SRR17286917_PRJNA790707 | SRR17286917 | PRJNA790707 | PE300 | China | Asia | China: Yangling | Illumina MiSeq | Unknown |
| SRR17286918_PRJNA790707 | SRR17286918 | PRJNA790707 | PE300 | China | Asia | China: Yangling | Illumina MiSeq | Unknown |
| SRR17286919_PRJNA790707 | SRR17286919 | PRJNA790707 | PE300 | China | Asia | China: Yangling | Illumina MiSeq | Unknown |
| SRR17286920_PRJNA790707 | SRR17286920 | PRJNA790707 | PE300 | China | Asia | China: Yangling | Illumina MiSeq | Unknown |
| SRR17286921_PRJNA790707 | SRR17286921 | PRJNA790707 | PE300 | China | Asia | China: Yangling | Illumina MiSeq | Unknown |
| SRR17286922_PRJNA790707 | SRR17286922 | PRJNA790707 | PE300 | China | Asia | China: Yangling | Illumina MiSeq | Unknown |
| SRR17286923_PRJNA790707 | SRR17286923 | PRJNA790707 | PE300 | China | Asia | China: Yangling | Illumina MiSeq | Unknown |
| SRR17286924_PRJNA790707 | SRR17286924 | PRJNA790707 | PE300 | China | Asia | China: Yangling | Illumina MiSeq | Unknown |
| SRR18319644_PRJNA815872 | SRR18319644 | PRJNA815872 | PE300 | China | Asia | China:Jianghuai | Illumina HiSeq 2000 | Unknown |
| SRR18319645_PRJNA815872 | SRR18319645 | PRJNA815872 | PE300 | China | Asia | China:Jianghuai | Illumina HiSeq 2000 | Unknown |
| SRR18319646_PRJNA815872 | SRR18319646 | PRJNA815872 | PE300 | China | Asia | China:Jianghuai | Illumina HiSeq 2000 | Unknown |
| SRR18319647_PRJNA815872 | SRR18319647 | PRJNA815872 | PE300 | China | Asia | China:Hefei | Illumina HiSeq 2000 | Unknown |
| SRR18319648_PRJNA815872 | SRR18319648 | PRJNA815872 | PE300 | China | Asia | China:Hefei | Illumina HiSeq 2000 | Unknown |
| SRR18319649_PRJNA815872 | SRR18319649 | PRJNA815872 | PE300 | China | Asia | China:Hefei | Illumina HiSeq 2000 | Unknown |
| SRR18319650_PRJNA815872 | SRR18319650 | PRJNA815872 | PE300 | China | Asia | China:Hefei | Illumina HiSeq 2000 | Unknown |
| SRR18319651_PRJNA815872 | SRR18319651 | PRJNA815872 | PE300 | China | Asia | China:Hefei | Illumina HiSeq 2000 | Unknown |
| SRR18319652_PRJNA815872 | SRR18319652 | PRJNA815872 | PE300 | China | Asia | China:Hefei | Illumina HiSeq 2000 | Unknown |
| SRR19503647_PRJNA843198 | SRR19503647 | PRJNA843198 | PE300 | China | Asia | China:Guangzhou | Illumina HiSeq 4000 | Unknown |
| SRR19503648_PRJNA843198 | SRR19503648 | PRJNA843198 | PE300 | China | Asia | China:Guangzhou | Illumina HiSeq 4000 | Unknown |
| SRR19503649_PRJNA843198 | SRR19503649 | PRJNA843198 | PE300 | China | Asia | China:Guangzhou | Illumina HiSeq 4000 | Unknown |
| SRR19503650_PRJNA843198 | SRR19503650 | PRJNA843198 | PE300 | China | Asia | China:Guangzhou | Illumina HiSeq 4000 | Unknown |
| SRR19503651_PRJNA843198 | SRR19503651 | PRJNA843198 | PE300 | China | Asia | China:Guangzhou | Illumina HiSeq 4000 | Unknown |
| SRR19503652_PRJNA843198 | SRR19503652 | PRJNA843198 | PE300 | China | Asia | China:Guangzhou | Illumina HiSeq 4000 | Unknown |
| SRR19503653_PRJNA843198 | SRR19503653 | PRJNA843198 | PE300 | China | Asia | China:Guangzhou | Illumina HiSeq 4000 | Unknown |
| SRR19503654_PRJNA843198 | SRR19503654 | PRJNA843198 | PE300 | China | Asia | China:Guangzhou | Illumina HiSeq 4000 | Unknown |
| SRR19503655_PRJNA843198 | SRR19503655 | PRJNA843198 | PE300 | China | Asia | China:Guangzhou | Illumina HiSeq 4000 | Unknown |
| SRR21079098_PRJNA869195 | SRR21079098 | PRJNA869195 | PE300 | China | Asia | China:BaoDing | Illumina MiSeq | Chinese Yam |
| SRR21079099_PRJNA869195 | SRR21079099 | PRJNA869195 | PE300 | China | Asia | China:BaoDing | Illumina MiSeq | Honeysuckle |
| SRR21079100_PRJNA869195 | SRR21079100 | PRJNA869195 | PE300 | China | Asia | China:BaoDing | Illumina MiSeq | Honeysuckle |
| SRR21079101_PRJNA869195 | SRR21079101 | PRJNA869195 | PE300 | China | Asia | China:BaoDing | Illumina MiSeq | Wheat |
| SRR21079102_PRJNA869195 | SRR21079102 | PRJNA869195 | PE300 | China | Asia | China:BaoDing | Illumina MiSeq | Wheat |
| SRR21079103_PRJNA869195 | SRR21079103 | PRJNA869195 | PE300 | China | Asia | China:BaoDing | Illumina MiSeq | Wheat |
| SRR21079104_PRJNA869195 | SRR21079104 | PRJNA869195 | PE300 | China | Asia | China:BaoDing | Illumina MiSeq | Rice |
| SRR21079105_PRJNA869195 | SRR21079105 | PRJNA869195 | PE300 | China | Asia | China:BaoDing | Illumina MiSeq | Rice |
| SRR21079106_PRJNA869195 | SRR21079106 | PRJNA869195 | PE300 | China | Asia | China:BaoDing | Illumina MiSeq | Rice |
| SRR21079107_PRJNA869195 | SRR21079107 | PRJNA869195 | PE300 | China | Asia | China:BaoDing | Illumina MiSeq | Maize |
| SRR21079108_PRJNA869195 | SRR21079108 | PRJNA869195 | PE300 | China | Asia | China:BaoDing | Illumina MiSeq | Maize |
| SRR21079109_PRJNA869195 | SRR21079109 | PRJNA869195 | PE300 | China | Asia | China:BaoDing | Illumina MiSeq | Chinese Yam |
| SRR21079110_PRJNA869195 | SRR21079110 | PRJNA869195 | PE300 | China | Asia | China:BaoDing | Illumina MiSeq | Maize |
| SRR21079111_PRJNA869195 | SRR21079111 | PRJNA869195 | PE300 | China | Asia | China:BaoDing | Illumina MiSeq | Honeysuckle |
| SRR21079112_PRJNA869195 | SRR21079112 | PRJNA869195 | PE300 | China | Asia | China:BaoDing | Illumina MiSeq | Honeysuckle |
| SRR21079113_PRJNA869195 | SRR21079113 | PRJNA869195 | PE300 | China | Asia | China:BaoDing | Illumina MiSeq | Honeysuckle |
| SRR21079114_PRJNA869195 | SRR21079114 | PRJNA869195 | PE300 | China | Asia | China:BaoDing | Illumina MiSeq | Honeysuckle |
| SRR21460370_PRJNA877394 | SRR21460370 | PRJNA877394 | PE250 | China | Asia | China: Yunnan | Illumina MiniSeq | Unknown |
| SRR21460371_PRJNA877394 | SRR21460371 | PRJNA877394 | PE250 | China | Asia | China: Yunnan | Illumina MiniSeq | Unknown |
| SRR21460372_PRJNA877394 | SRR21460372 | PRJNA877394 | PE250 | China | Asia | China: Yunnan | Illumina MiniSeq | Unknown |
| SRR21460373_PRJNA877394 | SRR21460373 | PRJNA877394 | PE250 | China | Asia | China: Hubei | Illumina MiniSeq | Unknown |
| SRR21460374_PRJNA877394 | SRR21460374 | PRJNA877394 | PE250 | China | Asia | China: Hubei | Illumina MiniSeq | Unknown |
| SRR21460375_PRJNA877394 | SRR21460375 | PRJNA877394 | PE250 | China | Asia | China: Hubei | Illumina MiniSeq | Unknown |
| SRR21460376_PRJNA877394 | SRR21460376 | PRJNA877394 | PE250 | China | Asia | China: Hubei | Illumina MiniSeq | Unknown |
| SRR21460377_PRJNA877394 | SRR21460377 | PRJNA877394 | PE250 | China | Asia | China: Yunnan | Illumina MiniSeq | Unknown |
| SRR21460378_PRJNA877394 | SRR21460378 | PRJNA877394 | PE250 | China | Asia | China: Hubei | Illumina MiniSeq | Unknown |
| SRR21460379_PRJNA877394 | SRR21460379 | PRJNA877394 | PE250 | China | Asia | China: Hubei | Illumina MiniSeq | Unknown |
| SRR21460381_PRJNA877394 | SRR21460381 | PRJNA877394 | PE250 | China | Asia | China: Hubei | Illumina MiniSeq | Unknown |
| SRR21460382_PRJNA877394 | SRR21460382 | PRJNA877394 | PE250 | China | Asia | China: Hubei | Illumina MiniSeq | Unknown |
| SRR21460383_PRJNA877394 | SRR21460383 | PRJNA877394 | PE250 | China | Asia | China: Hubei | Illumina MiniSeq | Unknown |
| SRR21460384_PRJNA877394 | SRR21460384 | PRJNA877394 | PE250 | China | Asia | China: Hubei | Illumina MiniSeq | Unknown |
| SRR21460385_PRJNA877394 | SRR21460385 | PRJNA877394 | PE250 | China | Asia | China: Hubei | Illumina MiniSeq | Unknown |
| SRR21460386_PRJNA877394 | SRR21460386 | PRJNA877394 | PE250 | China | Asia | China: Hubei | Illumina MiniSeq | Unknown |
| SRR21460387_PRJNA877394 | SRR21460387 | PRJNA877394 | PE250 | China | Asia | China: Hubei | Illumina MiniSeq | Unknown |
| SRR21460388_PRJNA877394 | SRR21460388 | PRJNA877394 | PE250 | China | Asia | China: Yunnan | Illumina MiniSeq | Unknown |
| SRR21460389_PRJNA877394 | SRR21460389 | PRJNA877394 | PE250 | China | Asia | China: Hubei | Illumina MiniSeq | Unknown |
| SRR21460390_PRJNA877394 | SRR21460390 | PRJNA877394 | PE250 | China | Asia | China: Hubei | Illumina MiniSeq | Unknown |
| SRR21460391_PRJNA877394 | SRR21460391 | PRJNA877394 | PE250 | China | Asia | China: Henan | Illumina MiniSeq | Unknown |
| SRR21460392_PRJNA877394 | SRR21460392 | PRJNA877394 | PE250 | China | Asia | China: Henan | Illumina MiniSeq | Unknown |
| SRR21460393_PRJNA877394 | SRR21460393 | PRJNA877394 | PE250 | China | Asia | China: Henan | Illumina MiniSeq | Unknown |
| SRR21460394_PRJNA877394 | SRR21460394 | PRJNA877394 | PE250 | China | Asia | China: Henan | Illumina MiniSeq | Unknown |
| SRR21460395_PRJNA877394 | SRR21460395 | PRJNA877394 | PE250 | China | Asia | China: Henan | Illumina MiniSeq | Unknown |
| SRR21460396_PRJNA877394 | SRR21460396 | PRJNA877394 | PE250 | China | Asia | China: Henan | Illumina MiniSeq | Unknown |
| SRR21460397_PRJNA877394 | SRR21460397 | PRJNA877394 | PE250 | China | Asia | China: Henan | Illumina MiniSeq | Unknown |
| SRR21460398_PRJNA877394 | SRR21460398 | PRJNA877394 | PE250 | China | Asia | China: Henan | Illumina MiniSeq | Unknown |
| SRR21460399_PRJNA877394 | SRR21460399 | PRJNA877394 | PE250 | China | Asia | China: Yunnan | Illumina MiniSeq | Unknown |
| SRR21460400_PRJNA877394 | SRR21460400 | PRJNA877394 | PE250 | China | Asia | China: Henan | Illumina MiniSeq | Unknown |
| SRR21460401_PRJNA877394 | SRR21460401 | PRJNA877394 | PE250 | China | Asia | China: Henan | Illumina MiniSeq | Unknown |
| SRR21460402_PRJNA877394 | SRR21460402 | PRJNA877394 | PE250 | China | Asia | China: Henan | Illumina MiniSeq | Unknown |
| SRR21460403_PRJNA877394 | SRR21460403 | PRJNA877394 | PE250 | China | Asia | China: Henan | Illumina MiniSeq | Unknown |
| SRR21460404_PRJNA877394 | SRR21460404 | PRJNA877394 | PE250 | China | Asia | China: Henan | Illumina MiniSeq | Unknown |
| SRR21460405_PRJNA877394 | SRR21460405 | PRJNA877394 | PE250 | China | Asia | China: Henan | Illumina MiniSeq | Unknown |
| SRR21460406_PRJNA877394 | SRR21460406 | PRJNA877394 | PE250 | China | Asia | China: Henan | Illumina MiniSeq | Unknown |
| SRR21460407_PRJNA877394 | SRR21460407 | PRJNA877394 | PE250 | China | Asia | China: Henan | Illumina MiniSeq | Unknown |
| SRR21460409_PRJNA877394 | SRR21460409 | PRJNA877394 | PE250 | China | Asia | China: Hainan | Illumina MiniSeq | Unknown |
| SRR21460410_PRJNA877394 | SRR21460410 | PRJNA877394 | PE250 | China | Asia | China: Yunnan | Illumina MiniSeq | Unknown |
| SRR21460411_PRJNA877394 | SRR21460411 | PRJNA877394 | PE250 | China | Asia | China: Hainan | Illumina MiniSeq | Unknown |
| SRR21460412_PRJNA877394 | SRR21460412 | PRJNA877394 | PE250 | China | Asia | China: Hainan | Illumina MiniSeq | Unknown |
| SRR21460413_PRJNA877394 | SRR21460413 | PRJNA877394 | PE250 | China | Asia | China: Hainan | Illumina MiniSeq | Unknown |
| SRR21460414_PRJNA877394 | SRR21460414 | PRJNA877394 | PE250 | China | Asia | China: Hainan | Illumina MiniSeq | Unknown |
| SRR21460415_PRJNA877394 | SRR21460415 | PRJNA877394 | PE250 | China | Asia | China: Hainan | Illumina MiniSeq | Unknown |
| SRR21460416_PRJNA877394 | SRR21460416 | PRJNA877394 | PE250 | China | Asia | China: Hainan | Illumina MiniSeq | Unknown |
| SRR21460417_PRJNA877394 | SRR21460417 | PRJNA877394 | PE250 | China | Asia | China: Hainan | Illumina MiniSeq | Unknown |
| SRR21460418_PRJNA877394 | SRR21460418 | PRJNA877394 | PE250 | China | Asia | China: Hainan | Illumina MiniSeq | Unknown |
| SRR21460419_PRJNA877394 | SRR21460419 | PRJNA877394 | PE250 | China | Asia | China: Hainan | Illumina MiniSeq | Unknown |
| SRR21460420_PRJNA877394 | SRR21460420 | PRJNA877394 | PE250 | China | Asia | China: Hainan | Illumina MiniSeq | Unknown |
| SRR21460421_PRJNA877394 | SRR21460421 | PRJNA877394 | PE250 | China | Asia | China: Yunnan | Illumina MiniSeq | Unknown |
| SRR21460422_PRJNA877394 | SRR21460422 | PRJNA877394 | PE250 | China | Asia | China: Hainan | Illumina MiniSeq | Unknown |
| SRR21460423_PRJNA877394 | SRR21460423 | PRJNA877394 | PE250 | China | Asia | China: Hainan | Illumina MiniSeq | Unknown |
| SRR21460424_PRJNA877394 | SRR21460424 | PRJNA877394 | PE250 | China | Asia | China: Hainan | Illumina MiniSeq | Unknown |
| SRR21460425_PRJNA877394 | SRR21460425 | PRJNA877394 | PE250 | China | Asia | China: Hainan | Illumina MiniSeq | Unknown |
| SRR21460426_PRJNA877394 | SRR21460426 | PRJNA877394 | PE250 | China | Asia | China: Yunnan | Illumina MiniSeq | Unknown |
| SRR21460427_PRJNA877394 | SRR21460427 | PRJNA877394 | PE250 | China | Asia | China: Yunnan | Illumina MiniSeq | Unknown |
| SRR21460428_PRJNA877394 | SRR21460428 | PRJNA877394 | PE250 | China | Asia | China: Yunnan | Illumina MiniSeq | Unknown |
| SRR21460429_PRJNA877394 | SRR21460429 | PRJNA877394 | PE250 | China | Asia | China: Yunnan | Illumina MiniSeq | Unknown |
| SRR21460430_PRJNA877394 | SRR21460430 | PRJNA877394 | PE250 | China | Asia | China: Yunnan | Illumina MiniSeq | Unknown |
| SRR21460431_PRJNA877394 | SRR21460431 | PRJNA877394 | PE250 | China | Asia | China: Yunnan | Illumina MiniSeq | Unknown |
| SRR21460432_PRJNA877394 | SRR21460432 | PRJNA877394 | PE250 | China | Asia | China: Yunnan | Illumina MiniSeq | Unknown |
| SRR21460433_PRJNA877394 | SRR21460433 | PRJNA877394 | PE250 | China | Asia | China: Yunnan | Illumina MiniSeq | Unknown |
| SRR21482814_PRJNA877459 | SRR21482814 | PRJNA877459 | PE250 | Brazil | South America | Brazil: Bahia | Illumina MiSeq | Maize |
| SRR21482815_PRJNA877459 | SRR21482815 | PRJNA877459 | PE250 | Brazil | South America | Brazil: Bahia | Illumina MiSeq | Cotton |
| SRR21482816_PRJNA877459 | SRR21482816 | PRJNA877459 | PE250 | Brazil | South America | Brazil: Bahia | Illumina MiSeq | Cotton |
| SRR21482817_PRJNA877459 | SRR21482817 | PRJNA877459 | PE250 | Brazil | South America | Brazil: Bahia | Illumina MiSeq | Cotton |
| SRR21482818_PRJNA877459 | SRR21482818 | PRJNA877459 | PE250 | Brazil | South America | Brazil: Bahia | Illumina MiSeq | Millet |
| SRR21482819_PRJNA877459 | SRR21482819 | PRJNA877459 | PE250 | Brazil | South America | Brazil: Bahia | Illumina MiSeq | Millet |
| SRR21482820_PRJNA877459 | SRR21482820 | PRJNA877459 | PE250 | Brazil | South America | Brazil: Bahia | Illumina MiSeq | Maize |
| SRR21482821_PRJNA877459 | SRR21482821 | PRJNA877459 | PE250 | Brazil | South America | Brazil: Bahia | Illumina MiSeq | Millet |
| SRR21482822_PRJNA877459 | SRR21482822 | PRJNA877459 | PE250 | Brazil | South America | Brazil: Bahia | Illumina MiSeq | Millet |
| SRR21482824_PRJNA877459 | SRR21482824 | PRJNA877459 | PE250 | Brazil | South America | Brazil: Bahia | Illumina MiSeq | Cotton |
| SRR21482825_PRJNA877459 | SRR21482825 | PRJNA877459 | PE250 | Brazil | South America | Brazil: Bahia | Illumina MiSeq | Maize |
| SRR21482826_PRJNA877459 | SRR21482826 | PRJNA877459 | PE250 | Brazil | South America | Brazil: Bahia | Illumina MiSeq | Maize |
| SRR21482827_PRJNA877459 | SRR21482827 | PRJNA877459 | PE250 | Brazil | South America | Brazil: Bahia | Illumina MiSeq | Millet |
| SRR21482828_PRJNA877459 | SRR21482828 | PRJNA877459 | PE250 | Brazil | South America | Brazil: Bahia | Illumina MiSeq | Maize |
| SRR21482829_PRJNA877459 | SRR21482829 | PRJNA877459 | PE250 | Brazil | South America | Brazil: Bahia | Illumina MiSeq | Maize |
| SRR21482830_PRJNA877459 | SRR21482830 | PRJNA877459 | PE250 | Brazil | South America | Brazil: Bahia | Illumina MiSeq | Millet |
| SRR21482831_PRJNA877459 | SRR21482831 | PRJNA877459 | PE250 | Brazil | South America | Brazil: Bahia | Illumina MiSeq | Cotton |
| SRR22419889_PRJNA905564 | SRR22419889 | PRJNA905564 | PE300 | China | Asia | China: Yangling | Illumina MiSeq | Unknown |
| SRR22419890_PRJNA905564 | SRR22419890 | PRJNA905564 | PE300 | China | Asia | China: Yangling | Illumina MiSeq | Unknown |
| SRR22419891_PRJNA905564 | SRR22419891 | PRJNA905564 | PE300 | China | Asia | China: Yangling | Illumina MiSeq | Unknown |
| SRR22419892_PRJNA905564 | SRR22419892 | PRJNA905564 | PE300 | China | Asia | China: Yangling | Illumina MiSeq | Unknown |
| SRR22419893_PRJNA905564 | SRR22419893 | PRJNA905564 | PE300 | China | Asia | China: Yangling | Illumina MiSeq | Unknown |
| SRR22419894_PRJNA905564 | SRR22419894 | PRJNA905564 | PE300 | China | Asia | China: Yangling | Illumina MiSeq | Unknown |
| SRR22419895_PRJNA905564 | SRR22419895 | PRJNA905564 | PE300 | China | Asia | China: Yangling | Illumina MiSeq | Unknown |
| SRR22419896_PRJNA905564 | SRR22419896 | PRJNA905564 | PE300 | China | Asia | China: Yangling | Illumina MiSeq | Unknown |
| SRR22419897_PRJNA905564 | SRR22419897 | PRJNA905564 | PE300 | China | Asia | China: Yangling | Illumina MiSeq | Unknown |
| SRR22419898_PRJNA905564 | SRR22419898 | PRJNA905564 | PE300 | China | Asia | China: Yangling | Illumina MiSeq | Unknown |
| SRR22419899_PRJNA905564 | SRR22419899 | PRJNA905564 | PE300 | China | Asia | China: Yangling | Illumina MiSeq | Unknown |
| SRR22419900_PRJNA905564 | SRR22419900 | PRJNA905564 | PE300 | China | Asia | China: Yangling | Illumina MiSeq | Unknown |
| SRR22419901_PRJNA905564 | SRR22419901 | PRJNA905564 | PE300 | China | Asia | China: Yangling | Illumina MiSeq | Unknown |
| SRR22419902_PRJNA905564 | SRR22419902 | PRJNA905564 | PE300 | China | Asia | China: Yangling | Illumina MiSeq | Unknown |
| SRR22419903_PRJNA905564 | SRR22419903 | PRJNA905564 | PE300 | China | Asia | China: Yangling | Illumina MiSeq | Unknown |
| SRR22419904_PRJNA905564 | SRR22419904 | PRJNA905564 | PE300 | China | Asia | China: Yangling | Illumina MiSeq | Unknown |
| SRR22419905_PRJNA905564 | SRR22419905 | PRJNA905564 | PE300 | China | Asia | China: Yangling | Illumina MiSeq | Unknown |
| SRR22419906_PRJNA905564 | SRR22419906 | PRJNA905564 | PE300 | China | Asia | China: Yangling | Illumina MiSeq | Unknown |
| SRR22419907_PRJNA905564 | SRR22419907 | PRJNA905564 | PE300 | China | Asia | China: Yangling | Illumina MiSeq | Unknown |
| SRR22419908_PRJNA905564 | SRR22419908 | PRJNA905564 | PE300 | China | Asia | China: Yangling | Illumina MiSeq | Unknown |
| SRR22419909_PRJNA905564 | SRR22419909 | PRJNA905564 | PE300 | China | Asia | China: Yangling | Illumina MiSeq | Unknown |
| SRR23078171_PRJNA922519 | SRR23078171 | PRJNA922519 | PE300 | South Korea | Asia | South Korea: Gangwon-do, Chuncheon | Illumina HiSeq 4000 | Maize |
| SRR23078172_PRJNA922519 | SRR23078172 | PRJNA922519 | PE300 | South Korea | Asia | South Korea: Gangwon-do, Chuncheon | Illumina HiSeq 4000 | Maize |
| SRR23078173_PRJNA922519 | SRR23078173 | PRJNA922519 | PE300 | South Korea | Asia | South Korea: Gangwon-do, Chuncheon | Illumina HiSeq 4000 | Maize |
| SRR23078174_PRJNA922519 | SRR23078174 | PRJNA922519 | PE300 | South Korea | Asia | South Korea: Gangwon-do, Chuncheon | Illumina HiSeq 4000 | Maize |
| SRR23078175_PRJNA922519 | SRR23078175 | PRJNA922519 | PE300 | South Korea | Asia | South Korea: Gangwon-do, Chuncheon | Illumina HiSeq 4000 | Maize |
| SRR23078177_PRJNA922519 | SRR23078177 | PRJNA922519 | PE300 | South Korea | Asia | South Korea: Gangwon-do, Chuncheon | Illumina HiSeq 4000 | Maize |
| SRR23078178_PRJNA922519 | SRR23078178 | PRJNA922519 | PE300 | South Korea | Asia | South Korea: Gangwon-do, Chuncheon | Illumina HiSeq 4000 | Maize |
| SRR23078179_PRJNA922519 | SRR23078179 | PRJNA922519 | PE300 | South Korea | Asia | South Korea: Gangwon-do, Chuncheon | Illumina HiSeq 4000 | Maize |
| SRR23078180_PRJNA922519 | SRR23078180 | PRJNA922519 | PE300 | South Korea | Asia | South Korea: Gangwon-do, Chuncheon | Illumina HiSeq 4000 | Maize |
| SRR23078181_PRJNA922519 | SRR23078181 | PRJNA922519 | PE300 | South Korea | Asia | South Korea: Gangwon-do, Chuncheon | Illumina HiSeq 4000 | Maize |
| SRR23078182_PRJNA922519 | SRR23078182 | PRJNA922519 | PE300 | South Korea | Asia | South Korea: Gangwon-do, Chuncheon | Illumina HiSeq 4000 | Maize |
| SRR23078183_PRJNA922519 | SRR23078183 | PRJNA922519 | PE300 | South Korea | Asia | South Korea: Gangwon-do, Chuncheon | Illumina HiSeq 4000 | Maize |
| SRR23078184_PRJNA922519 | SRR23078184 | PRJNA922519 | PE300 | South Korea | Asia | South Korea: Gangwon-do, Chuncheon | Illumina HiSeq 4000 | Maize |
| SRR23078186_PRJNA922519 | SRR23078186 | PRJNA922519 | PE300 | South Korea | Asia | South Korea: Gangwon-do, Chuncheon | Illumina HiSeq 4000 | Maize |
| SRR23078187_PRJNA922519 | SRR23078187 | PRJNA922519 | PE300 | South Korea | Asia | South Korea: Gangwon-do, Chuncheon | Illumina HiSeq 4000 | Maize |
| SRR23078188_PRJNA922519 | SRR23078188 | PRJNA922519 | PE300 | South Korea | Asia | South Korea: Gangwon-do, Chuncheon | Illumina HiSeq 4000 | Maize |
| SRR24825804_PRJNA979959 | SRR24825804 | PRJNA979959 | PE250 | India | Asia | India | Illumina MiSeq | Maize |
| SRR24825805_PRJNA979959 | SRR24825805 | PRJNA979959 | PE250 | India | Asia | India | Illumina MiSeq | Maize |
| SRR24825806_PRJNA979959 | SRR24825806 | PRJNA979959 | PE250 | India | Asia | India | Illumina MiSeq | Maize |
| SRR24825807_PRJNA979959 | SRR24825807 | PRJNA979959 | PE250 | India | Asia | India | Illumina MiSeq | Maize |
| SRR24825808_PRJNA979959 | SRR24825808 | PRJNA979959 | PE250 | India | Asia | India | Illumina MiSeq | Maize |
| SRR24825810_PRJNA979959 | SRR24825810 | PRJNA979959 | PE250 | India | Asia | India | Illumina MiSeq | Maize |
| SRR24825811_PRJNA979959 | SRR24825811 | PRJNA979959 | PE250 | India | Asia | India | Illumina MiSeq | Maize |
| SRR24825812_PRJNA979959 | SRR24825812 | PRJNA979959 | PE250 | India | Asia | India | Illumina MiSeq | Maize |
| SRR24825813_PRJNA979959 | SRR24825813 | PRJNA979959 | PE250 | India | Asia | India | Illumina MiSeq | Maize |
| SRR24825814_PRJNA979959 | SRR24825814 | PRJNA979959 | PE250 | India | Asia | India | Illumina MiSeq | Maize |
| SRR24825815_PRJNA979959 | SRR24825815 | PRJNA979959 | PE250 | India | Asia | India | Illumina MiSeq | Maize |
| SRR24825816_PRJNA979959 | SRR24825816 | PRJNA979959 | PE250 | India | Asia | India | Illumina MiSeq | Maize |
| SRR24825817_PRJNA979959 | SRR24825817 | PRJNA979959 | PE250 | India | Asia | India | Illumina MiSeq | Maize |
| SRR24825818_PRJNA979959 | SRR24825818 | PRJNA979959 | PE250 | India | Asia | India | Illumina MiSeq | Maize |
| SRR24825819_PRJNA979959 | SRR24825819 | PRJNA979959 | PE250 | India | Asia | India | Illumina MiSeq | Maize |
| SRR24825820_PRJNA979959 | SRR24825820 | PRJNA979959 | PE250 | India | Asia | India | Illumina MiSeq | Maize |
| SRR24825821_PRJNA979959 | SRR24825821 | PRJNA979959 | PE250 | India | Asia | India | Illumina MiSeq | Maize |
| SRR24825822_PRJNA979959 | SRR24825822 | PRJNA979959 | PE250 | India | Asia | India | Illumina MiSeq | Maize |
| SRR24825823_PRJNA979959 | SRR24825823 | PRJNA979959 | PE250 | India | Asia | India | Illumina MiSeq | Maize |
| SRR24825824_PRJNA979959 | SRR24825824 | PRJNA979959 | PE250 | India | Asia | India | Illumina MiSeq | Maize |
| SRR24825825_PRJNA979959 | SRR24825825 | PRJNA979959 | PE250 | India | Asia | India | Illumina MiSeq | Maize |
| SRR24825826_PRJNA979959 | SRR24825826 | PRJNA979959 | PE250 | India | Asia | India | Illumina MiSeq | Maize |
| SRR24825827_PRJNA979959 | SRR24825827 | PRJNA979959 | PE250 | India | Asia | India | Illumina MiSeq | Maize |
| SRR24825828_PRJNA979959 | SRR24825828 | PRJNA979959 | PE250 | India | Asia | India | Illumina MiSeq | Maize |
| SRR24913470_PRJNA983288 | SRR24913470 | PRJNA983288 | PE250 | China | Asia | China:Guangzhou | Illumina NovaSeq 6000 | artificial |
| SRR24913471_PRJNA983288 | SRR24913471 | PRJNA983288 | PE250 | China | Asia | China:Guangzhou | Illumina NovaSeq 6000 | artificial |
| SRR24913472_PRJNA983288 | SRR24913472 | PRJNA983288 | PE250 | China | Asia | China:Guangzhou | Illumina NovaSeq 6000 | artificial |
| SRR24913474_PRJNA983288 | SRR24913474 | PRJNA983288 | PE250 | China | Asia | China:Guangzhou | Illumina NovaSeq 6000 | artificial |
| SRR24913476_PRJNA983288 | SRR24913476 | PRJNA983288 | PE250 | China | Asia | China:Guangzhou | Illumina NovaSeq 6000 | artificial |
| SRR24913477_PRJNA983288 | SRR24913477 | PRJNA983288 | PE250 | China | Asia | China:Guangzhou | Illumina NovaSeq 6000 | artificial |
| SRR24913478_PRJNA983288 | SRR24913478 | PRJNA983288 | PE250 | China | Asia | China:Guangzhou | Illumina NovaSeq 6000 | artificial |
| SRR24913479_PRJNA983288 | SRR24913479 | PRJNA983288 | PE250 | China | Asia | China:Guangzhou | Illumina NovaSeq 6000 | artificial |
| SRR24913480_PRJNA983288 | SRR24913480 | PRJNA983288 | PE250 | China | Asia | China:Guangzhou | Illumina NovaSeq 6000 | artificial |
| SRR24913481_PRJNA983288 | SRR24913481 | PRJNA983288 | PE250 | China | Asia | China:Guangzhou | Illumina NovaSeq 6000 | artificial |
| SRR24913482_PRJNA983288 | SRR24913482 | PRJNA983288 | PE250 | China | Asia | China:Guangzhou | Illumina NovaSeq 6000 | artificial |
| SRR24913483_PRJNA983288 | SRR24913483 | PRJNA983288 | PE250 | China | Asia | China:Guangzhou | Illumina NovaSeq 6000 | artificial |
| SRR24913484_PRJNA983288 | SRR24913484 | PRJNA983288 | PE250 | China | Asia | China:Guangzhou | Illumina NovaSeq 6000 | artificial |
| SRR24913485_PRJNA983288 | SRR24913485 | PRJNA983288 | PE250 | China | Asia | China:Guangzhou | Illumina NovaSeq 6000 | artificial |
| SRR24913486_PRJNA983288 | SRR24913486 | PRJNA983288 | PE250 | China | Asia | China:Guangzhou | Illumina NovaSeq 6000 | artificial |
| SRR24913487_PRJNA983288 | SRR24913487 | PRJNA983288 | PE250 | China | Asia | China:Guangzhou | Illumina NovaSeq 6000 | artificial |
| SRR24913488_PRJNA983288 | SRR24913488 | PRJNA983288 | PE250 | China | Asia | China:Guangzhou | Illumina NovaSeq 6000 | artificial |
| SRR24913489_PRJNA983288 | SRR24913489 | PRJNA983288 | PE250 | China | Asia | China:Guangzhou | Illumina NovaSeq 6000 | artificial |
| SRR24913490_PRJNA983288 | SRR24913490 | PRJNA983288 | PE250 | China | Asia | China:Guangzhou | Illumina NovaSeq 6000 | artificial |
| SRR29001163_PRJNA1110625 | SRR29001163 | PRJNA1110625 | PE250 | China | Asia | not applicable | Illumina NovaSeq 6000 | Unknown |
| SRR29001164_PRJNA1110625 | SRR29001164 | PRJNA1110625 | PE250 | China | Asia | not applicable | Illumina NovaSeq 6000 | Unknown |
| SRR29001165_PRJNA1110625 | SRR29001165 | PRJNA1110625 | PE250 | China | Asia | not applicable | Illumina NovaSeq 6000 | Unknown |
| SRR8268640_PRJNA507591 | SRR8268640 | PRJNA507591 | PE300 | USA | North America | USA: Pennsylvania | Illumina MiSeq | Maize |
| SRR8268641_PRJNA507591 | SRR8268641 | PRJNA507591 | PE300 | USA | North America | USA: Pennsylvania | Illumina MiSeq | Maize |
| SRR8268642_PRJNA507591 | SRR8268642 | PRJNA507591 | PE300 | USA | North America | USA: Pennsylvania | Illumina MiSeq | Maize |
| SRR8268643_PRJNA507591 | SRR8268643 | PRJNA507591 | PE300 | USA | North America | USA: Pennsylvania | Illumina MiSeq | Soybean |
| SRR8268644_PRJNA507591 | SRR8268644 | PRJNA507591 | PE300 | USA | North America | USA: Pennsylvania | Illumina MiSeq | Soybean |
| SRR8268645_PRJNA507591 | SRR8268645 | PRJNA507591 | PE300 | USA | North America | USA: Pennsylvania | Illumina MiSeq | Soybean |
| SRR8268646_PRJNA507591 | SRR8268646 | PRJNA507591 | PE300 | USA | North America | USA: Pennsylvania | Illumina MiSeq | Soybean |
| SRR8268648_PRJNA507591 | SRR8268648 | PRJNA507591 | PE300 | USA | North America | USA: Pennsylvania | Illumina MiSeq | Maize |
| SRR8268649_PRJNA507591 | SRR8268649 | PRJNA507591 | PE300 | USA | North America | USA: Pennsylvania | Illumina MiSeq | Maize |
| SRR8268653_PRJNA507591 | SRR8268653 | PRJNA507591 | PE300 | USA | North America | USA: Pennsylvania | Illumina MiSeq | Harner |
| SRR8268654_PRJNA507591 | SRR8268654 | PRJNA507591 | PE300 | USA | North America | USA: Pennsylvania | Illumina MiSeq | Harner |
| SRR8268655_PRJNA507591 | SRR8268655 | PRJNA507591 | PE300 | USA | North America | USA: Pennsylvania | Illumina MiSeq | Harner |
| SRR8268656_PRJNA507591 | SRR8268656 | PRJNA507591 | PE300 | USA | North America | USA: Pennsylvania | Illumina MiSeq | Harner |
| SRR8268657_PRJNA507591 | SRR8268657 | PRJNA507591 | PE300 | USA | North America | USA: Pennsylvania | Illumina MiSeq | Harner |
| SRR8268660_PRJNA507591 | SRR8268660 | PRJNA507591 | PE300 | USA | North America | USA: Pennsylvania | Illumina MiSeq | Maize |
| SRR8268661_PRJNA507591 | SRR8268661 | PRJNA507591 | PE300 | USA | North America | USA: Pennsylvania | Illumina MiSeq | Maize |
| SRR8268662_PRJNA507591 | SRR8268662 | PRJNA507591 | PE300 | USA | North America | USA: Pennsylvania | Illumina MiSeq | Maize |
| SRR8268663_PRJNA507591 | SRR8268663 | PRJNA507591 | PE300 | USA | North America | USA: Pennsylvania | Illumina MiSeq | Maize |
| SRR8268664_PRJNA507591 | SRR8268664 | PRJNA507591 | PE300 | USA | North America | USA: Pennsylvania | Illumina MiSeq | Maize |
| SRR8268665_PRJNA507591 | SRR8268665 | PRJNA507591 | PE300 | USA | North America | USA: Pennsylvania | Illumina MiSeq | Soybean |
| SRR8268666_PRJNA507591 | SRR8268666 | PRJNA507591 | PE300 | USA | North America | USA: Pennsylvania | Illumina MiSeq | Maize |
| SRR8268667_PRJNA507591 | SRR8268667 | PRJNA507591 | PE300 | USA | North America | USA: Pennsylvania | Illumina MiSeq | Maize |
| SRR8268668_PRJNA507591 | SRR8268668 | PRJNA507591 | PE300 | USA | North America | USA: Pennsylvania | Illumina MiSeq | Soybean |
| SRR8268669_PRJNA507591 | SRR8268669 | PRJNA507591 | PE300 | USA | North America | USA: Pennsylvania | Illumina MiSeq | Soybean |
| SRR8268670_PRJNA507591 | SRR8268670 | PRJNA507591 | PE300 | USA | North America | USA: Pennsylvania | Illumina MiSeq | Harner |
| SRR8268671_PRJNA507591 | SRR8268671 | PRJNA507591 | PE300 | USA | North America | USA: Pennsylvania | Illumina MiSeq | Maize |
| SRR8268672_PRJNA507591 | SRR8268672 | PRJNA507591 | PE300 | USA | North America | USA: Pennsylvania | Illumina MiSeq | Maize |
| SRR8268673_PRJNA507591 | SRR8268673 | PRJNA507591 | PE300 | USA | North America | USA: Pennsylvania | Illumina MiSeq | Maize |
| SRR8268674_PRJNA507591 | SRR8268674 | PRJNA507591 | PE300 | USA | North America | USA: Pennsylvania | Illumina MiSeq | Maize |
| SRR8268675_PRJNA507591 | SRR8268675 | PRJNA507591 | PE300 | USA | North America | USA: Pennsylvania | Illumina MiSeq | Maize |
| SRR8268676_PRJNA507591 | SRR8268676 | PRJNA507591 | PE300 | USA | North America | USA: Pennsylvania | Illumina MiSeq | Maize |
| SRR8268677_PRJNA507591 | SRR8268677 | PRJNA507591 | PE300 | USA | North America | USA: Pennsylvania | Illumina MiSeq | Maize |
| SRR8268678_PRJNA507591 | SRR8268678 | PRJNA507591 | PE300 | USA | North America | USA: Pennsylvania | Illumina MiSeq | Soybean |
| SRR8268679_PRJNA507591 | SRR8268679 | PRJNA507591 | PE300 | USA | North America | USA: Pennsylvania | Illumina MiSeq | Soybean |
| SRR8268680_PRJNA507591 | SRR8268680 | PRJNA507591 | PE300 | USA | North America | USA: Pennsylvania | Illumina MiSeq | Soybean |
| SRR8268681_PRJNA507591 | SRR8268681 | PRJNA507591 | PE300 | USA | North America | USA: Pennsylvania | Illumina MiSeq | Soybean |
| SRR8268682_PRJNA507591 | SRR8268682 | PRJNA507591 | PE300 | USA | North America | USA: Pennsylvania | Illumina MiSeq | Soybean |
| SRR8268683_PRJNA507591 | SRR8268683 | PRJNA507591 | PE300 | USA | North America | USA: Pennsylvania | Illumina MiSeq | Soybean |
| SRR8268684_PRJNA507591 | SRR8268684 | PRJNA507591 | PE300 | USA | North America | USA: Pennsylvania | Illumina MiSeq | Soybean |
| SRR8268685_PRJNA507591 | SRR8268685 | PRJNA507591 | PE300 | USA | North America | USA: Pennsylvania | Illumina MiSeq | Soybean |
| SRR8268686_PRJNA507591 | SRR8268686 | PRJNA507591 | PE300 | USA | North America | USA: Pennsylvania | Illumina MiSeq | Maize |
| SRR8268687_PRJNA507591 | SRR8268687 | PRJNA507591 | PE300 | USA | North America | USA: Pennsylvania | Illumina MiSeq | Maize |
| SRR8268688_PRJNA507591 | SRR8268688 | PRJNA507591 | PE300 | USA | North America | USA: Pennsylvania | Illumina MiSeq | Maize |
| SRR8268690_PRJNA507591 | SRR8268690 | PRJNA507591 | PE300 | USA | North America | USA: Pennsylvania | Illumina MiSeq | Maize |
| SRR8268691_PRJNA507591 | SRR8268691 | PRJNA507591 | PE300 | USA | North America | USA: Pennsylvania | Illumina MiSeq | Maize |
| SRR8561474_PRJNA521837 | SRR8561474 | PRJNA521837 | PE300 | Kenya | Africa | Kenya: Ngeria,Uasin Gishu | Illumina MiSeq | Maize |
| SRR8561475_PRJNA521837 | SRR8561475 | PRJNA521837 | PE300 | Kenya | Africa | Kenya: Chala Irrigation Scheme | Illumina MiSeq | Maize |
| SRR8561476_PRJNA521837 | SRR8561476 | PRJNA521837 | PE300 | Kenya | Africa | Kenya: Chala Irrigation Scheme | Illumina MiSeq | Maize |
| SRR8561477_PRJNA521837 | SRR8561477 | PRJNA521837 | PE300 | Kenya | Africa | Kenya: Chala Irrigation Scheme | Illumina MiSeq | Maize |
| SRR8561478_PRJNA521837 | SRR8561478 | PRJNA521837 | PE300 | Kenya | Africa | Kenya: Msamia, Kitale | Illumina MiSeq | Maize |
| SRR8561479_PRJNA521837 | SRR8561479 | PRJNA521837 | PE300 | Kenya | Africa | Kenya: Chala Irrigation Scheme | Illumina MiSeq | Maize |
| SRR8561480_PRJNA521837 | SRR8561480 | PRJNA521837 | PE300 | Kenya | Africa | Kenya: Shimba Hills, Kwale | Illumina MiSeq | Maize |
| SRR8561481_PRJNA521837 | SRR8561481 | PRJNA521837 | PE300 | Kenya | Africa | Kenya: Shimba Hills, Kwale | Illumina MiSeq | Maize |
| SRR8561482_PRJNA521837 | SRR8561482 | PRJNA521837 | PE300 | Kenya | Africa | Kenya: Shimba Hills, Kwale | Illumina MiSeq | Maize |
| SRR8561483_PRJNA521837 | SRR8561483 | PRJNA521837 | PE300 | Kenya | Africa | Kenya: Shimba Hills, Kwale | Illumina MiSeq | Maize |
| SRR8561484_PRJNA521837 | SRR8561484 | PRJNA521837 | PE300 | Kenya | Africa | Kenya: Chala Irrigation Scheme | Illumina MiSeq | Maize |
| SRR8561485_PRJNA521837 | SRR8561485 | PRJNA521837 | PE300 | Kenya | Africa | Kenya: Chala Irrigation Scheme | Illumina MiSeq | Maize |
| SRR8561486_PRJNA521837 | SRR8561486 | PRJNA521837 | PE300 | Kenya | Africa | Kenya: Ngeria,Uasin Gishu | Illumina MiSeq | Maize |
| SRR8561487_PRJNA521837 | SRR8561487 | PRJNA521837 | PE300 | Kenya | Africa | Kenya: Ngeria,Uasin Gishu | Illumina MiSeq | Maize |
| SRR8561488_PRJNA521837 | SRR8561488 | PRJNA521837 | PE300 | Kenya | Africa | Kenya: Msamia, Kitale | Illumina MiSeq | Maize |
| SRR8561489_PRJNA521837 | SRR8561489 | PRJNA521837 | PE300 | Kenya | Africa | Kenya: Shimba Hills, Kwale | Illumina MiSeq | Maize |
| SRR8561490_PRJNA521837 | SRR8561490 | PRJNA521837 | PE300 | Kenya | Africa | Kenya: Shimba Hills, Kwale | Illumina MiSeq | Maize |
| SRR8561491_PRJNA521837 | SRR8561491 | PRJNA521837 | PE300 | Kenya | Africa | Kenya: Msamia, Kitale | Illumina MiSeq | Maize |

## Supplementary Table 1B

| **Diet** | **Samples** | **Group** | **Label** |
| --- | --- | --- | --- |
| Maize | 150 | Maize | 150 |
| Unknown | 139 | Unknown/not reported | 139 |
| artificial | 19 | Other diets | 19 |
| Soybean | 15 | Other diets | 15 |
| Rice | 13 | Other diets | 13 |
| Harner | 6 | Other diets | 6 |
| Honeysuckle | 6 | Other diets | 6 |
| Millet | 6 | Other diets | 6 |
| Cabbage | 5 | Other diets | 5 |
| Celocia | 5 | Other diets | 5 |
| Cotton | 5 | Other diets | 5 |
| Cucumber | 5 | Other diets | 5 |
| Onion | 5 | Other diets | 5 |
| Sugarcane | 5 | Other diets | 5 |
| Sweet potato | 5 | Other diets | 5 |
| Tomato | 5 | Other diets | 5 |
| Wheat | 3 | Other diets | 3 |
| Chinese Yam | 2 | Other diets | 2 |
| Green amaranth | 2 | Other diets | 2 |

## Supplementary Table 1C

| **Project ID** | **Hypervariable Region** |
| --- | --- |
| PRJNA631705 | v3-v4 |
| PRJNA681731 | v4 |
| PRJNA734744 | v3-v4 |
| PRJNA983288 | v3-v4 |
| PRJNA507591 | v4 |
| PRJNA595886 | v3-v4 |
| PRJNA655399 | v4 |
| PRJNA790707 | v3-v4 |
| PRJNA869195 | v3-v4 |
| PRJNA877394 | v4-v5 |
| PRJNA877459 | v3-v4 |
| PRJNA905564 | NA |
| PRJNA947378 | v3-v4 |
| PRJNA521837 | v4 |
| PRJNA979959 | NA |
| PRJNA954674 | v3-v4 |
| PRJNA1110625 | NA |
| NA001 | v4 |
| PRJNA922519 | v3-v4 |
| PRJNA815872 | NA |
| NA002 | NA |
| PRJNA843198 | v3-v4 |
| PRJNA658986 | v4 |

## Supplementary Table 2A

| **Genus** | **Africa** | **Asia** | **North America** | **South America** | **Core_continent_count** | **Globally_conserved_core** |
| --- | --- | --- | --- | --- | --- | --- |
| Acinetobacter | Present | Present | Present | Present | 4 | Yes |
| Enterococcus | Present | Present | Present | Present | 4 | Yes |
| Pseudomonas | Present | Present | Present | Present | 4 | Yes |
| Stenotrophomonas | Present | Present | Present | Present | 4 | Yes |
| Chryseobacterium | Present | Present | Present | Absent | 3 | No |
| Comamonas | Present | Present | Present | Absent | 3 | No |
| Methylobacterium-Methylorubrum | Present | Present | Present | Absent | 3 | No |
| Microbacterium | Present | Present | Present | Absent | 3 | No |
| Sphingobacterium | Present | Present | Present | Absent | 3 | No |
| Sphingomonas | Present | Present | Present | Absent | 3 | No |
| Allorhizobium-Neorhizobium-Pararhizobium-Rhizobium | Present | Present | Absent | Absent | 2 | No |
| Aureimonas | Present | Absent | Present | Absent | 2 | No |
| Bacillus | Present | Present | Absent | Absent | 2 | No |
| Brevundimonas | Present | Present | Absent | Absent | 2 | No |
| Burkholderia-Caballeronia-Paraburkholderia | Present | Absent | Absent | Present | 2 | No |
| Corynebacterium | Present | Present | Absent | Absent | 2 | No |
| Erysipelatoclostridium | Present | Absent | Absent | Present | 2 | No |
| Escherichia-Shigella | Present | Absent | Present | Absent | 2 | No |
| Flavobacterium | Present | Present | Absent | Absent | 2 | No |
| Rhodococcus | Present | Absent | Present | Absent | 2 | No |
| Turicibacter | Present | Absent | Absent | Present | 2 | No |
| uncultured | Present | Present | Absent | Absent | 2 | No |
| Achromobacter | Present | Absent | Absent | Absent | 1 | No |
| Aeromicrobium | Present | Absent | Absent | Absent | 1 | No |
| Alcaligenes | Present | Absent | Absent | Absent | 1 | No |
| Alistipes | Present | Absent | Absent | Absent | 1 | No |
| Asticcacaulis | Present | Absent | Absent | Absent | 1 | No |
| Bacteriovorax | Present | Absent | Absent | Absent | 1 | No |
| Bacteroides | Present | Absent | Absent | Absent | 1 | No |
| Bdellovibrio | Present | Absent | Absent | Absent | 1 | No |
| Bifidobacterium | Present | Absent | Absent | Absent | 1 | No |
| Blautia | Present | Absent | Absent | Absent | 1 | No |
| Brachybacterium | Present | Absent | Absent | Absent | 1 | No |
| Bradyrhizobium | Absent | Absent | Present | Absent | 1 | No |
| Brevibacterium | Present | Absent | Absent | Absent | 1 | No |
| Cellvibrio | Present | Absent | Absent | Absent | 1 | No |
| Clostridium_sensu_stricto_1 | Present | Absent | Absent | Absent | 1 | No |
| Delftia | Present | Absent | Absent | Absent | 1 | No |
| Devosia | Present | Absent | Absent | Absent | 1 | No |
| Empedobacter | Present | Absent | Absent | Absent | 1 | No |
| Enterobacter | Present | Absent | Absent | Absent | 1 | No |
| Exiguobacterium | Present | Absent | Absent | Absent | 1 | No |
| Falsochrobactrum | Present | Absent | Absent | Absent | 1 | No |
| Gemmobacter | Present | Absent | Absent | Absent | 1 | No |
| Glutamicibacter | Present | Absent | Absent | Absent | 1 | No |
| Gordonia | Present | Absent | Absent | Absent | 1 | No |
| Halomonas | Absent | Absent | Present | Absent | 1 | No |
| Ketogulonicigenium | Present | Absent | Absent | Absent | 1 | No |
| Kineococcus | Present | Absent | Absent | Absent | 1 | No |
| Klebsiella | Present | Absent | Absent | Absent | 1 | No |
| Klenkia | Present | Absent | Absent | Absent | 1 | No |
| Lactococcus | Present | Absent | Absent | Absent | 1 | No |
| Lampropedia | Present | Absent | Absent | Absent | 1 | No |
| Leadbetterella | Present | Absent | Absent | Absent | 1 | No |
| Leucobacter | Present | Absent | Absent | Absent | 1 | No |
| Marmoricola | Present | Absent | Absent | Absent | 1 | No |
| Mesorhizobium | Present | Absent | Absent | Absent | 1 | No |
| Methylobacillus | Present | Absent | Absent | Absent | 1 | No |
| Microbulbifer | Present | Absent | Absent | Absent | 1 | No |
| Micrococcus | Present | Absent | Absent | Absent | 1 | No |
| Mycobacterium | Present | Absent | Absent | Absent | 1 | No |
| Myroides | Present | Absent | Absent | Absent | 1 | No |
| Nocardioides | Present | Absent | Absent | Absent | 1 | No |
| Ochrobactrum | Absent | Absent | Present | Absent | 1 | No |
| Ottowia | Present | Absent | Absent | Absent | 1 | No |
| Oxalicibacterium | Present | Absent | Absent | Absent | 1 | No |
| Paenibacillus | Present | Absent | Absent | Absent | 1 | No |
| Paracoccus | Present | Absent | Absent | Absent | 1 | No |
| Patulibacter | Present | Absent | Absent | Absent | 1 | No |
| Peredibacter | Present | Absent | Absent | Absent | 1 | No |
| Quadrisphaera | Present | Absent | Absent | Absent | 1 | No |
| Rheinheimera | Present | Absent | Absent | Absent | 1 | No |
| Serratia | Present | Absent | Absent | Absent | 1 | No |
| Shewanella | Absent | Absent | Present | Absent | 1 | No |
| Staphylococcus | Present | Absent | Absent | Absent | 1 | No |
| Streptococcus | Present | Absent | Absent | Absent | 1 | No |
| Taibaiella | Present | Absent | Absent | Absent | 1 | No |
| TM7a | Present | Absent | Absent | Absent | 1 | No |
| Tsukamurella | Present | Absent | Absent | Absent | 1 | No |
| Weeksella | Present | Absent | Absent | Absent | 1 | No |
| Weissella | Present | Absent | Absent | Absent | 1 | No |
| ZOR0006 | Present | Absent | Absent | Absent | 1 | No |
| [Aquaspirillum]_arcticum_group | Absent | Absent | Absent | Absent | 0 | No |
| [Clostridium]_innocuum_group | Absent | Absent | Absent | Absent | 0 | No |
| [Eubacterium]_brachy_group | Absent | Absent | Absent | Absent | 0 | No |
| [Eubacterium]_coprostanoligenes_group | Absent | Absent | Absent | Absent | 0 | No |
| [Eubacterium]_eligens_group | Absent | Absent | Absent | Absent | 0 | No |
| [Eubacterium]_hallii_group | Absent | Absent | Absent | Absent | 0 | No |
| [Eubacterium]_nodatum_group | Absent | Absent | Absent | Absent | 0 | No |
| [Eubacterium]_ruminantium_group | Absent | Absent | Absent | Absent | 0 | No |
| [Eubacterium]_siraeum_group | Absent | Absent | Absent | Absent | 0 | No |
| [Eubacterium]_ventriosum_group | Absent | Absent | Absent | Absent | 0 | No |
| [Eubacterium]_xylanophilum_group | Absent | Absent | Absent | Absent | 0 | No |
| [Ruminococcus]_gnavus_group | Absent | Absent | Absent | Absent | 0 | No |
| [Ruminococcus]_torques_group | Absent | Absent | Absent | Absent | 0 | No |
| 0319-6G20 | Absent | Absent | Absent | Absent | 0 | No |
| 0319-7L14 | Absent | Absent | Absent | Absent | 0 | No |
| 1-20 | Absent | Absent | Absent | Absent | 0 | No |
| 11-24 | Absent | Absent | Absent | Absent | 0 | No |
| 1174-901-12 | Absent | Absent | Absent | Absent | 0 | No |
| 1921-2 | Absent | Absent | Absent | Absent | 0 | No |
| 1921-3 | Absent | Absent | Absent | Absent | 0 | No |
| 1959-1 | Absent | Absent | Absent | Absent | 0 | No |
| 37-13 | Absent | Absent | Absent | Absent | 0 | No |
| 67-14 | Absent | Absent | Absent | Absent | 0 | No |
| A0839 | Absent | Absent | Absent | Absent | 0 | No |
| A2 | Absent | Absent | Absent | Absent | 0 | No |
| A21b | Absent | Absent | Absent | Absent | 0 | No |
| A4b | Absent | Absent | Absent | Absent | 0 | No |
| AAP99 | Absent | Absent | Absent | Absent | 0 | No |
| AB1 | Absent | Absent | Absent | Absent | 0 | No |
| Abditibacterium | Absent | Absent | Absent | Absent | 0 | No |
| Abiotrophia | Absent | Absent | Absent | Absent | 0 | No |
| Absconditabacteriales_(SR1) | Absent | Absent | Absent | Absent | 0 | No |
| Acetanaerobacterium | Absent | Absent | Absent | Absent | 0 | No |
| Acetatifactor | Absent | Absent | Absent | Absent | 0 | No |
| Acetitomaculum | Absent | Absent | Absent | Absent | 0 | No |
| Acetoanaerobium | Absent | Absent | Absent | Absent | 0 | No |
| Acetobacter | Absent | Absent | Absent | Absent | 0 | No |
| Acetobacterium | Absent | Absent | Absent | Absent | 0 | No |
| Acetobacteroides | Absent | Absent | Absent | Absent | 0 | No |
| Acholeplasma | Absent | Absent | Absent | Absent | 0 | No |
| Acidaminococcus | Absent | Absent | Absent | Absent | 0 | No |
| Acidibacter | Absent | Absent | Absent | Absent | 0 | No |
| Acidipila | Absent | Absent | Absent | Absent | 0 | No |
| Acidisphaera | Absent | Absent | Absent | Absent | 0 | No |
| Acidobacteriales | Absent | Absent | Absent | Absent | 0 | No |
| Acidocella | Absent | Absent | Absent | Absent | 0 | No |
| Acidothermus | Absent | Absent | Absent | Absent | 0 | No |
| Acidovorax | Absent | Absent | Absent | Absent | 0 | No |
| Actinoallomurus | Absent | Absent | Absent | Absent | 0 | No |
| Actinocatenispora | Absent | Absent | Absent | Absent | 0 | No |
| Actinomadura | Absent | Absent | Absent | Absent | 0 | No |
| Actinomyces | Absent | Absent | Absent | Absent | 0 | No |
| Actinomycetospora | Absent | Absent | Absent | Absent | 0 | No |
| Actinophytocola | Absent | Absent | Absent | Absent | 0 | No |
| Actinoplanes | Absent | Absent | Absent | Absent | 0 | No |
| Actinopolymorpha | Absent | Absent | Absent | Absent | 0 | No |
| Actinospica | Absent | Absent | Absent | Absent | 0 | No |
| Acuticoccus | Absent | Absent | Absent | Absent | 0 | No |
| AD3 | Absent | Absent | Absent | Absent | 0 | No |
| Adhaeribacter | Absent | Absent | Absent | Absent | 0 | No |
| Adlercreutzia | Absent | Absent | Absent | Absent | 0 | No |
| ADurb.Bin063-1 | Absent | Absent | Absent | Absent | 0 | No |
| Aerococcus | Absent | Absent | Absent | Absent | 0 | No |
| Aeromonas | Absent | Absent | Absent | Absent | 0 | No |
| Afipia | Absent | Absent | Absent | Absent | 0 | No |
| Agathobacter | Absent | Absent | Absent | Absent | 0 | No |
| Aggregatibacter | Absent | Absent | Absent | Absent | 0 | No |
| Agrococcus | Absent | Absent | Absent | Absent | 0 | No |
| Agromyces | Absent | Absent | Absent | Absent | 0 | No |
| Ahniella | Absent | Absent | Absent | Absent | 0 | No |
| AKAU4049 | Absent | Absent | Absent | Absent | 0 | No |
| AKIW659 | Absent | Absent | Absent | Absent | 0 | No |
| AKIW781 | Absent | Absent | Absent | Absent | 0 | No |
| Akkermansia | Absent | Absent | Absent | Absent | 0 | No |
| AKYG1722 | Absent | Absent | Absent | Absent | 0 | No |
| AKYG587 | Absent | Absent | Absent | Absent | 0 | No |
| AKYH767 | Absent | Absent | Absent | Absent | 0 | No |
| Alcanivorax | Absent | Absent | Absent | Absent | 0 | No |
| Algoriphagus | Absent | Absent | Absent | Absent | 0 | No |
| Alicycliphilus | Absent | Absent | Absent | Absent | 0 | No |
| Alicyclobacillus | Absent | Absent | Absent | Absent | 0 | No |
| Aliicoccus | Absent | Absent | Absent | Absent | 0 | No |
| Aliihoeflea | Absent | Absent | Absent | Absent | 0 | No |
| Alishewanella | Absent | Absent | Absent | Absent | 0 | No |
| Aliterella | Absent | Absent | Absent | Absent | 0 | No |
| Alkanindiges | Absent | Absent | Absent | Absent | 0 | No |
| Allisonella | Absent | Absent | Absent | Absent | 0 | No |
| Allobaculum | Absent | Absent | Absent | Absent | 0 | No |
| Alloiococcus | Absent | Absent | Absent | Absent | 0 | No |
| Alloprevotella | Absent | Absent | Absent | Absent | 0 | No |
| alphaI_cluster | Absent | Absent | Absent | Absent | 0 | No |
| Alsobacter | Absent | Absent | Absent | Absent | 0 | No |
| Altererythrobacter | Absent | Absent | Absent | Absent | 0 | No |
| Alteribacillus | Absent | Absent | Absent | Absent | 0 | No |
| Alterococcus | Absent | Absent | Absent | Absent | 0 | No |
| Amaricoccus | Absent | Absent | Absent | Absent | 0 | No |
| Amb-16S-1323 | Absent | Absent | Absent | Absent | 0 | No |
| Aminicenantales | Absent | Absent | Absent | Absent | 0 | No |
| Ammoniphilus | Absent | Absent | Absent | Absent | 0 | No |
| Amnipila | Absent | Absent | Absent | Absent | 0 | No |
| Amphiplicatus | Absent | Absent | Absent | Absent | 0 | No |
| Amycolatopsis | Absent | Absent | Absent | Absent | 0 | No |
| Anaerobium | Absent | Absent | Absent | Absent | 0 | No |
| Anaerococcus | Absent | Absent | Absent | Absent | 0 | No |
| Anaerocolumna | Absent | Absent | Absent | Absent | 0 | No |
| Anaeroglobus | Absent | Absent | Absent | Absent | 0 | No |
| Anaerolinea | Absent | Absent | Absent | Absent | 0 | No |
| Anaerolineaceae_UCG-001 | Absent | Absent | Absent | Absent | 0 | No |
| Anaerolineae | Absent | Absent | Absent | Absent | 0 | No |
| Anaeromyxobacter | Absent | Absent | Absent | Absent | 0 | No |
| Anaerophaga | Absent | Absent | Absent | Absent | 0 | No |
| Anaeroplasma | Absent | Absent | Absent | Absent | 0 | No |
| Anaerostipes | Absent | Absent | Absent | Absent | 0 | No |
| Anaerotruncus | Absent | Absent | Absent | Absent | 0 | No |
| Anaerovibrio | Absent | Absent | Absent | Absent | 0 | No |
| Anaerovorax | Absent | Absent | Absent | Absent | 0 | No |
| Ancylobacter | Absent | Absent | Absent | Absent | 0 | No |
| Angustibacter | Absent | Absent | Absent | Absent | 0 | No |
| Anoxybacillus | Absent | Absent | Absent | Absent | 0 | No |
| Antricoccus | Absent | Absent | Absent | Absent | 0 | No |
| Aphanizomenon_MDT14a | Absent | Absent | Absent | Absent | 0 | No |
| Aquabacterium | Absent | Absent | Absent | Absent | 0 | No |
| Aquicella | Absent | Absent | Absent | Absent | 0 | No |
| Aquimonas | Absent | Absent | Absent | Absent | 0 | No |
| Aquisphaera | Absent | Absent | Absent | Absent | 0 | No |
| Arcobacter | Absent | Absent | Absent | Absent | 0 | No |
| Arenimonas | Absent | Absent | Absent | Absent | 0 | No |
| Aridibacter | Absent | Absent | Absent | Absent | 0 | No |
| Armatimonadales | Absent | Absent | Absent | Absent | 0 | No |
| Armatimonas | Absent | Absent | Absent | Absent | 0 | No |
| Arsenicibacter | Absent | Absent | Absent | Absent | 0 | No |
| Arsenophonus | Absent | Absent | Absent | Absent | 0 | No |
| Arthrobacter | Absent | Absent | Absent | Absent | 0 | No |
| Asaia | Absent | Absent | Absent | Absent | 0 | No |
| Atopobium | Absent | Absent | Absent | Absent | 0 | No |
| Atopostipes | Absent | Absent | Absent | Absent | 0 | No |
| Aurantisolimonas | Absent | Absent | Absent | Absent | 0 | No |
| Auricoccus-Abyssicoccus | Absent | Absent | Absent | Absent | 0 | No |
| Azohydromonas | Absent | Absent | Absent | Absent | 0 | No |
| Azorhizobium | Absent | Absent | Absent | Absent | 0 | No |
| Azospira | Absent | Absent | Absent | Absent | 0 | No |
| Azospirillaceae | Absent | Absent | Absent | Absent | 0 | No |
| Azospirillum | Absent | Absent | Absent | Absent | 0 | No |
| Azotobacter | Absent | Absent | Absent | Absent | 0 | No |
| B1-7BS | Absent | Absent | Absent | Absent | 0 | No |
| B10-SB3A | Absent | Absent | Absent | Absent | 0 | No |
| B12-WMSP1 | Absent | Absent | Absent | Absent | 0 | No |
| Babeliales | Absent | Absent | Absent | Absent | 0 | No |
| BacC-u-018 | Absent | Absent | Absent | Absent | 0 | No |
| Bact-08 | Absent | Absent | Absent | Absent | 0 | No |
| bacteriap25 | Absent | Absent | Absent | Absent | 0 | No |
| Bacteroidales_UCG-001 | Absent | Absent | Absent | Absent | 0 | No |
| Bacteroidetes_BD2-2 | Absent | Absent | Absent | Absent | 0 | No |
| Bacteroidetes_vadinHA17 | Absent | Absent | Absent | Absent | 0 | No |
| Bacteroidetes_VC2.1_Bac22 | Absent | Absent | Absent | Absent | 0 | No |
| Baia | Absent | Absent | Absent | Absent | 0 | No |
| Barnesiella | Absent | Absent | Absent | Absent | 0 | No |
| Bauldia | Absent | Absent | Absent | Absent | 0 | No |
| BBMC-4 | Absent | Absent | Absent | Absent | 0 | No |
| BD1-7_clade | Absent | Absent | Absent | Absent | 0 | No |
| BD2-11_terrestrial_group | Absent | Absent | Absent | Absent | 0 | No |
| BD7-11 | Absent | Absent | Absent | Absent | 0 | No |
| BD72BR169 | Absent | Absent | Absent | Absent | 0 | No |
| Berkelbacteria | Absent | Absent | Absent | Absent | 0 | No |
| Bilophila | Absent | Absent | Absent | Absent | 0 | No |
| BIrii41 | Absent | Absent | Absent | Absent | 0 | No |
| Blastocatella | Absent | Absent | Absent | Absent | 0 | No |
| Blastococcus | Absent | Absent | Absent | Absent | 0 | No |
| Blastomonas | Absent | Absent | Absent | Absent | 0 | No |
| Blfdi19 | Absent | Absent | Absent | Absent | 0 | No |
| Bordetella | Absent | Absent | Absent | Absent | 0 | No |
| Bosea | Absent | Absent | Absent | Absent | 0 | No |
| Brachymonas | Absent | Absent | Absent | Absent | 0 | No |
| Bradymonadales | Absent | Absent | Absent | Absent | 0 | No |
| Brevibacillus | Absent | Absent | Absent | Absent | 0 | No |
| Brevifollis | Absent | Absent | Absent | Absent | 0 | No |
| BRH-c20a | Absent | Absent | Absent | Absent | 0 | No |
| Brooklawnia | Absent | Absent | Absent | Absent | 0 | No |
| Bryobacter | Absent | Absent | Absent | Absent | 0 | No |
| Bryocella | Absent | Absent | Absent | Absent | 0 | No |
| BSV13 | Absent | Absent | Absent | Absent | 0 | No |
| BSV26 | Absent | Absent | Absent | Absent | 0 | No |
| Butyricicoccus | Absent | Absent | Absent | Absent | 0 | No |
| Butyricimonas | Absent | Absent | Absent | Absent | 0 | No |
| Butyrivibrio | Absent | Absent | Absent | Absent | 0 | No |
| C0119 | Absent | Absent | Absent | Absent | 0 | No |
| C10-SB1A | Absent | Absent | Absent | Absent | 0 | No |
| Caedibacter | Absent | Absent | Absent | Absent | 0 | No |
| Caenimonas | Absent | Absent | Absent | Absent | 0 | No |
| CAG-352 | Absent | Absent | Absent | Absent | 0 | No |
| Caldalkalibacillus | Absent | Absent | Absent | Absent | 0 | No |
| Caldicoprobacter | Absent | Absent | Absent | Absent | 0 | No |
| Caldilineaceae | Absent | Absent | Absent | Absent | 0 | No |
| Caldisericum | Absent | Absent | Absent | Absent | 0 | No |
| Camelimonas | Absent | Absent | Absent | Absent | 0 | No |
| Campylobacter | Absent | Absent | Absent | Absent | 0 | No |
| Candidatus_Adlerbacteria | Absent | Absent | Absent | Absent | 0 | No |
| Candidatus_Alysiosphaera | Absent | Absent | Absent | Absent | 0 | No |
| Candidatus_Bacilloplasma | Absent | Absent | Absent | Absent | 0 | No |
| Candidatus_Berkiella | Absent | Absent | Absent | Absent | 0 | No |
| Candidatus_Caldatribacterium | Absent | Absent | Absent | Absent | 0 | No |
| Candidatus_Competibacter | Absent | Absent | Absent | Absent | 0 | No |
| Candidatus_Entotheonella | Absent | Absent | Absent | Absent | 0 | No |
| Candidatus_Jidaibacter | Absent | Absent | Absent | Absent | 0 | No |
| Candidatus_Kaiserbacteria | Absent | Absent | Absent | Absent | 0 | No |
| Candidatus_Koribacter | Absent | Absent | Absent | Absent | 0 | No |
| Candidatus_Kuenenia | Absent | Absent | Absent | Absent | 0 | No |
| Candidatus_Methylopumilus | Absent | Absent | Absent | Absent | 0 | No |
| Candidatus_Moranbacteria | Absent | Absent | Absent | Absent | 0 | No |
| Candidatus_Nucleicultrix | Absent | Absent | Absent | Absent | 0 | No |
| Candidatus_Obscuribacter | Absent | Absent | Absent | Absent | 0 | No |
| Candidatus_Ovatusbacter | Absent | Absent | Absent | Absent | 0 | No |
| Candidatus_Paracaedibacter | Absent | Absent | Absent | Absent | 0 | No |
| Candidatus_Peregrinibacteria | Absent | Absent | Absent | Absent | 0 | No |
| Candidatus_Planktophila | Absent | Absent | Absent | Absent | 0 | No |
| Candidatus_Protochlamydia | Absent | Absent | Absent | Absent | 0 | No |
| Candidatus_Saccharimonas | Absent | Absent | Absent | Absent | 0 | No |
| Candidatus_Soleaferrea | Absent | Absent | Absent | Absent | 0 | No |
| Candidatus_Solibacter | Absent | Absent | Absent | Absent | 0 | No |
| Candidatus_Sulcia | Absent | Absent | Absent | Absent | 0 | No |
| Candidatus_Udaeobacter | Absent | Absent | Absent | Absent | 0 | No |
| Candidatus_Vidania | Absent | Absent | Absent | Absent | 0 | No |
| Candidatus_Woesebacteria | Absent | Absent | Absent | Absent | 0 | No |
| Candidatus_Xiphinematobacter | Absent | Absent | Absent | Absent | 0 | No |
| Candidatus_Yanofskybacteria | Absent | Absent | Absent | Absent | 0 | No |
| Candidatus_Zambryskibacteria | Absent | Absent | Absent | Absent | 0 | No |
| Capnocytophaga | Absent | Absent | Absent | Absent | 0 | No |
| Caproiciproducens | Absent | Absent | Absent | Absent | 0 | No |
| Carnobacterium | Absent | Absent | Absent | Absent | 0 | No |
| Castellaniella | Absent | Absent | Absent | Absent | 0 | No |
| Catellatospora | Absent | Absent | Absent | Absent | 0 | No |
| Catenibacterium | Absent | Absent | Absent | Absent | 0 | No |
| Catenulispora | Absent | Absent | Absent | Absent | 0 | No |
| Caulobacter | Absent | Absent | Absent | Absent | 0 | No |
| Cavicella | Absent | Absent | Absent | Absent | 0 | No |
| CCD24 | Absent | Absent | Absent | Absent | 0 | No |
| CCM19a | Absent | Absent | Absent | Absent | 0 | No |
| Cellulomonas | Absent | Absent | Absent | Absent | 0 | No |
| Cellulosimicrobium | Absent | Absent | Absent | Absent | 0 | No |
| CENA518 | Absent | Absent | Absent | Absent | 0 | No |
| Cephalothrix_SAG_75.79 | Absent | Absent | Absent | Absent | 0 | No |
| Cetobacterium | Absent | Absent | Absent | Absent | 0 | No |
| Chelativorans | Absent | Absent | Absent | Absent | 0 | No |
| Chishuiella | Absent | Absent | Absent | Absent | 0 | No |
| Chitinibacter | Absent | Absent | Absent | Absent | 0 | No |
| Chitinimonas | Absent | Absent | Absent | Absent | 0 | No |
| Chitinophaga | Absent | Absent | Absent | Absent | 0 | No |
| Chitinophagaceae | Absent | Absent | Absent | Absent | 0 | No |
| CHKCI001 | Absent | Absent | Absent | Absent | 0 | No |
| Chloronema | Absent | Absent | Absent | Absent | 0 | No |
| Christensenella | Absent | Absent | Absent | Absent | 0 | No |
| Christensenellaceae_R-7_group | Absent | Absent | Absent | Absent | 0 | No |
| Chromobacterium | Absent | Absent | Absent | Absent | 0 | No |
| Chroococcidiopsis_PCC_7203 | Absent | Absent | Absent | Absent | 0 | No |
| Chryseolinea | Absent | Absent | Absent | Absent | 0 | No |
| Chryseomicrobium | Absent | Absent | Absent | Absent | 0 | No |
| Chthoniobacter | Absent | Absent | Absent | Absent | 0 | No |
| Chthonobacter | Absent | Absent | Absent | Absent | 0 | No |
| Chthonomonadales | Absent | Absent | Absent | Absent | 0 | No |
| Chthonomonas | Absent | Absent | Absent | Absent | 0 | No |
| Chujaibacter | Absent | Absent | Absent | Absent | 0 | No |
| Chungangia | Absent | Absent | Absent | Absent | 0 | No |
| CI75cm.2.12 | Absent | Absent | Absent | Absent | 0 | No |
| Ciceribacter | Absent | Absent | Absent | Absent | 0 | No |
| Citrifermentans | Absent | Absent | Absent | Absent | 0 | No |
| CL500-29_marine_group | Absent | Absent | Absent | Absent | 0 | No |
| CL500-3 | Absent | Absent | Absent | Absent | 0 | No |
| Clade_III | Absent | Absent | Absent | Absent | 0 | No |
| Cloacibacillus | Absent | Absent | Absent | Absent | 0 | No |
| Cloacibacterium | Absent | Absent | Absent | Absent | 0 | No |
| Clostridia_UCG-014 | Absent | Absent | Absent | Absent | 0 | No |
| Clostridia_vadinBB60_group | Absent | Absent | Absent | Absent | 0 | No |
| Clostridium_sensu_stricto_10 | Absent | Absent | Absent | Absent | 0 | No |
| Clostridium_sensu_stricto_11 | Absent | Absent | Absent | Absent | 0 | No |
| Clostridium_sensu_stricto_12 | Absent | Absent | Absent | Absent | 0 | No |
| Clostridium_sensu_stricto_13 | Absent | Absent | Absent | Absent | 0 | No |
| Clostridium_sensu_stricto_14 | Absent | Absent | Absent | Absent | 0 | No |
| Clostridium_sensu_stricto_2 | Absent | Absent | Absent | Absent | 0 | No |
| Clostridium_sensu_stricto_3 | Absent | Absent | Absent | Absent | 0 | No |
| Clostridium_sensu_stricto_5 | Absent | Absent | Absent | Absent | 0 | No |
| Clostridium_sensu_stricto_7 | Absent | Absent | Absent | Absent | 0 | No |
| Clostridium_sensu_stricto_8 | Absent | Absent | Absent | Absent | 0 | No |
| Clostridium_sensu_stricto_9 | Absent | Absent | Absent | Absent | 0 | No |
| Cnuella | Absent | Absent | Absent | Absent | 0 | No |
| Cohnella | Absent | Absent | Absent | Absent | 0 | No |
| Colidextribacter | Absent | Absent | Absent | Absent | 0 | No |
| Collimonas | Absent | Absent | Absent | Absent | 0 | No |
| Collinsella | Absent | Absent | Absent | Absent | 0 | No |
| Commensalibacter | Absent | Absent | Absent | Absent | 0 | No |
| Conexibacter | Absent | Absent | Absent | Absent | 0 | No |
| Coprobacillus | Absent | Absent | Absent | Absent | 0 | No |
| Coprococcus | Absent | Absent | Absent | Absent | 0 | No |
| Coralloluteibacterium | Absent | Absent | Absent | Absent | 0 | No |
| Coriobacteriaceae_UCG-002 | Absent | Absent | Absent | Absent | 0 | No |
| Corticicoccus | Absent | Absent | Absent | Absent | 0 | No |
| Corynebacteriaceae | Absent | Absent | Absent | Absent | 0 | No |
| Coxiella | Absent | Absent | Absent | Absent | 0 | No |
| CPla-3_termite_group | Absent | Absent | Absent | Absent | 0 | No |
| Craurococcus-Caldovatus | Absent | Absent | Absent | Absent | 0 | No |
| Crossiella | Absent | Absent | Absent | Absent | 0 | No |
| Cryptanaerobacter | Absent | Absent | Absent | Absent | 0 | No |
| Cryptosporangium | Absent | Absent | Absent | Absent | 0 | No |
| Cupriavidus | Absent | Absent | Absent | Absent | 0 | No |
| Curtobacterium | Absent | Absent | Absent | Absent | 0 | No |
| Curvibacter | Absent | Absent | Absent | Absent | 0 | No |
| Cutibacterium | Absent | Absent | Absent | Absent | 0 | No |
| cvE6 | Absent | Absent | Absent | Absent | 0 | No |
| Cyanobium_PCC-6307 | Absent | Absent | Absent | Absent | 0 | No |
| Cystobacter | Absent | Absent | Absent | Absent | 0 | No |
| Cytophaga | Absent | Absent | Absent | Absent | 0 | No |
| D05-2 | Absent | Absent | Absent | Absent | 0 | No |
| D8A-2 | Absent | Absent | Absent | Absent | 0 | No |
| Dactylosporangium | Absent | Absent | Absent | Absent | 0 | No |
| Dadabacteriales | Absent | Absent | Absent | Absent | 0 | No |
| Dechlorobacter | Absent | Absent | Absent | Absent | 0 | No |
| Dechloromonas | Absent | Absent | Absent | Absent | 0 | No |
| Deferrisoma | Absent | Absent | Absent | Absent | 0 | No |
| Defluviicoccus | Absent | Absent | Absent | Absent | 0 | No |
| Defluviimonas | Absent | Absent | Absent | Absent | 0 | No |
| Defluviitoga | Absent | Absent | Absent | Absent | 0 | No |
| Deinococcus | Absent | Absent | Absent | Absent | 0 | No |
| Denitratisoma | Absent | Absent | Absent | Absent | 0 | No |
| Desemzia | Absent | Absent | Absent | Absent | 0 | No |
| Desertifilum_KR2012-2 | Absent | Absent | Absent | Absent | 0 | No |
| Desulfobulbus | Absent | Absent | Absent | Absent | 0 | No |
| Desulfomicrobium | Absent | Absent | Absent | Absent | 0 | No |
| Desulfomonile | Absent | Absent | Absent | Absent | 0 | No |
| Desulfoprunum | Absent | Absent | Absent | Absent | 0 | No |
| Desulforhabdus | Absent | Absent | Absent | Absent | 0 | No |
| Desulfosporosinus | Absent | Absent | Absent | Absent | 0 | No |
| Desulfovibrio | Absent | Absent | Absent | Absent | 0 | No |
| Desulfuromonas | Absent | Absent | Absent | Absent | 0 | No |
| Dethiobacter | Absent | Absent | Absent | Absent | 0 | No |
| Dethiosulfatibacter | Absent | Absent | Absent | Absent | 0 | No |
| Dethiosulfovibrio | Absent | Absent | Absent | Absent | 0 | No |
| DEV007 | Absent | Absent | Absent | Absent | 0 | No |
| DEV114 | Absent | Absent | Absent | Absent | 0 | No |
| dgA-11_gut_group | Absent | Absent | Absent | Absent | 0 | No |
| Dialister | Absent | Absent | Absent | Absent | 0 | No |
| Diaphorobacter | Absent | Absent | Absent | Absent | 0 | No |
| Dielma | Absent | Absent | Absent | Absent | 0 | No |
| Dietzia | Absent | Absent | Absent | Absent | 0 | No |
| Dinghuibacter | Absent | Absent | Absent | Absent | 0 | No |
| DMER64 | Absent | Absent | Absent | Absent | 0 | No |
| DNF00809 | Absent | Absent | Absent | Absent | 0 | No |
| Dojkabacteria | Absent | Absent | Absent | Absent | 0 | No |
| Dokdonella | Absent | Absent | Absent | Absent | 0 | No |
| Dolosicoccus | Absent | Absent | Absent | Absent | 0 | No |
| Dolosigranulum | Absent | Absent | Absent | Absent | 0 | No |
| Domibacillus | Absent | Absent | Absent | Absent | 0 | No |
| Dongia | Absent | Absent | Absent | Absent | 0 | No |
| Dorea | Absent | Absent | Absent | Absent | 0 | No |
| DS-100 | Absent | Absent | Absent | Absent | 0 | No |
| DSSD61 | Absent | Absent | Absent | Absent | 0 | No |
| DTU014 | Absent | Absent | Absent | Absent | 0 | No |
| DTU089 | Absent | Absent | Absent | Absent | 0 | No |
| Dubosiella | Absent | Absent | Absent | Absent | 0 | No |
| Duganella | Absent | Absent | Absent | Absent | 0 | No |
| Dyadobacter | Absent | Absent | Absent | Absent | 0 | No |
| Dyella | Absent | Absent | Absent | Absent | 0 | No |
| Dysgonomonas | Absent | Absent | Absent | Absent | 0 | No |
| EcFYyy-200 | Absent | Absent | Absent | Absent | 0 | No |
| Edaphobacter | Absent | Absent | Absent | Absent | 0 | No |
| Edaphobaculum | Absent | Absent | Absent | Absent | 0 | No |
| Eggerthella | Absent | Absent | Absent | Absent | 0 | No |
| Eisenbergiella | Absent | Absent | Absent | Absent | 0 | No |
| Elev-1554 | Absent | Absent | Absent | Absent | 0 | No |
| Elev-16S-1166 | Absent | Absent | Absent | Absent | 0 | No |
| Elizabethkingia | Absent | Absent | Absent | Absent | 0 | No |
| Ellin516 | Absent | Absent | Absent | Absent | 0 | No |
| Ellin517 | Absent | Absent | Absent | Absent | 0 | No |
| Ellin6055 | Absent | Absent | Absent | Absent | 0 | No |
| Ellin6067 | Absent | Absent | Absent | Absent | 0 | No |
| Elstera | Absent | Absent | Absent | Absent | 0 | No |
| Emticicia | Absent | Absent | Absent | Absent | 0 | No |
| endosymbionts | Absent | Absent | Absent | Absent | 0 | No |
| Enhydrobacter | Absent | Absent | Absent | Absent | 0 | No |
| Enorma | Absent | Absent | Absent | Absent | 0 | No |
| Ensifer | Absent | Absent | Absent | Absent | 0 | No |
| Enterorhabdus | Absent | Absent | Absent | Absent | 0 | No |
| Entotheonellaceae | Absent | Absent | Absent | Absent | 0 | No |
| env.OPS_17 | Absent | Absent | Absent | Absent | 0 | No |
| Eremococcus | Absent | Absent | Absent | Absent | 0 | No |
| Erysipelothrix | Absent | Absent | Absent | Absent | 0 | No |
| Erysipelotrichaceae_UCG-003 | Absent | Absent | Absent | Absent | 0 | No |
| Estrella | Absent | Absent | Absent | Absent | 0 | No |
| Eubacterium | Absent | Absent | Absent | Absent | 0 | No |
| EV818SWSAP88 | Absent | Absent | Absent | Absent | 0 | No |
| Ezakiella | Absent | Absent | Absent | Absent | 0 | No |
| F0058 | Absent | Absent | Absent | Absent | 0 | No |
| F082 | Absent | Absent | Absent | Absent | 0 | No |
| Facklamia | Absent | Absent | Absent | Absent | 0 | No |
| Faecalibacterium | Absent | Absent | Absent | Absent | 0 | No |
| Faecalibaculum | Absent | Absent | Absent | Absent | 0 | No |
| Faecalitalea | Absent | Absent | Absent | Absent | 0 | No |
| Falsirhodobacter | Absent | Absent | Absent | Absent | 0 | No |
| Family_XIII_AD3011_group | Absent | Absent | Absent | Absent | 0 | No |
| Family_XIII_UCG-001 | Absent | Absent | Absent | Absent | 0 | No |
| Fastidiosipila | Absent | Absent | Absent | Absent | 0 | No |
| FCPS473 | Absent | Absent | Absent | Absent | 0 | No |
| FCPU426 | Absent | Absent | Absent | Absent | 0 | No |
| Fenollaria | Absent | Absent | Absent | Absent | 0 | No |
| Fermentimonas | Absent | Absent | Absent | Absent | 0 | No |
| Ferrovibrio | Absent | Absent | Absent | Absent | 0 | No |
| Ferruginibacter | Absent | Absent | Absent | Absent | 0 | No |
| Fervidobacterium | Absent | Absent | Absent | Absent | 0 | No |
| FFCH16263 | Absent | Absent | Absent | Absent | 0 | No |
| FFCH5858 | Absent | Absent | Absent | Absent | 0 | No |
| FFCH7168 | Absent | Absent | Absent | Absent | 0 | No |
| Fibrobacter | Absent | Absent | Absent | Absent | 0 | No |
| Fictibacillus | Absent | Absent | Absent | Absent | 0 | No |
| Filifactor | Absent | Absent | Absent | Absent | 0 | No |
| Filimonas | Absent | Absent | Absent | Absent | 0 | No |
| Fimbriiglobus | Absent | Absent | Absent | Absent | 0 | No |
| Fimbriimonadaceae | Absent | Absent | Absent | Absent | 0 | No |
| Finegoldia | Absent | Absent | Absent | Absent | 0 | No |
| Flaviaesturariibacter | Absent | Absent | Absent | Absent | 0 | No |
| Flaviflexus | Absent | Absent | Absent | Absent | 0 | No |
| Flavihumibacter | Absent | Absent | Absent | Absent | 0 | No |
| Flavisolibacter | Absent | Absent | Absent | Absent | 0 | No |
| Flavitalea | Absent | Absent | Absent | Absent | 0 | No |
| Flavonifractor | Absent | Absent | Absent | Absent | 0 | No |
| Flectobacillus | Absent | Absent | Absent | Absent | 0 | No |
| Fluviicola | Absent | Absent | Absent | Absent | 0 | No |
| Fodinicurvata | Absent | Absent | Absent | Absent | 0 | No |
| Fontibacter | Absent | Absent | Absent | Absent | 0 | No |
| Fonticella | Absent | Absent | Absent | Absent | 0 | No |
| Fournierella | Absent | Absent | Absent | Absent | 0 | No |
| Frankiales | Absent | Absent | Absent | Absent | 0 | No |
| Frateuria | Absent | Absent | Absent | Absent | 0 | No |
| Fretibacterium | Absent | Absent | Absent | Absent | 0 | No |
| Friedmanniella | Absent | Absent | Absent | Absent | 0 | No |
| Frischella | Absent | Absent | Absent | Absent | 0 | No |
| FTLpost3 | Absent | Absent | Absent | Absent | 0 | No |
| FukuN57 | Absent | Absent | Absent | Absent | 0 | No |
| Fusibacter | Absent | Absent | Absent | Absent | 0 | No |
| Fusicatenibacter | Absent | Absent | Absent | Absent | 0 | No |
| Fusobacterium | Absent | Absent | Absent | Absent | 0 | No |
| G12-WMSP1 | Absent | Absent | Absent | Absent | 0 | No |
| Gaiella | Absent | Absent | Absent | Absent | 0 | No |
| GAL15 | Absent | Absent | Absent | Absent | 0 | No |
| Gallicola | Absent | Absent | Absent | Absent | 0 | No |
| Gammaproteobacteria | Absent | Absent | Absent | Absent | 0 | No |
| Gardnerella | Absent | Absent | Absent | Absent | 0 | No |
| Gastranaerophilales | Absent | Absent | Absent | Absent | 0 | No |
| GCA-900066575 | Absent | Absent | Absent | Absent | 0 | No |
| Gemella | Absent | Absent | Absent | Absent | 0 | No |
| Geminicoccus | Absent | Absent | Absent | Absent | 0 | No |
| Gemmata | Absent | Absent | Absent | Absent | 0 | No |
| Gemmatimonas | Absent | Absent | Absent | Absent | 0 | No |
| Geobacillus | Absent | Absent | Absent | Absent | 0 | No |
| Geobacter | Absent | Absent | Absent | Absent | 0 | No |
| Geodermatophilus | Absent | Absent | Absent | Absent | 0 | No |
| Georgenia | Absent | Absent | Absent | Absent | 0 | No |
| Geothermomicrobium | Absent | Absent | Absent | Absent | 0 | No |
| Geothrix | Absent | Absent | Absent | Absent | 0 | No |
| Gilliamella | Absent | Absent | Absent | Absent | 0 | No |
| Gillisia | Absent | Absent | Absent | Absent | 0 | No |
| Gilvimarinus | Absent | Absent | Absent | Absent | 0 | No |
| Gitt-GS-136 | Absent | Absent | Absent | Absent | 0 | No |
| Gluconacetobacter | Absent | Absent | Absent | Absent | 0 | No |
| Gluconobacter | Absent | Absent | Absent | Absent | 0 | No |
| Glycomyces | Absent | Absent | Absent | Absent | 0 | No |
| Gordonibacter | Absent | Absent | Absent | Absent | 0 | No |
| GOUTA6 | Absent | Absent | Absent | Absent | 0 | No |
| Gracilibacteria | Absent | Absent | Absent | Absent | 0 | No |
| Granulicatella | Absent | Absent | Absent | Absent | 0 | No |
| Granulicella | Absent | Absent | Absent | Absent | 0 | No |
| Guggenheimella | Absent | Absent | Absent | Absent | 0 | No |
| GZKB124 | Absent | Absent | Absent | Absent | 0 | No |
| Haemophilus | Absent | Absent | Absent | Absent | 0 | No |
| Hafnia-Obesumbacterium | Absent | Absent | Absent | Absent | 0 | No |
| Hahella | Absent | Absent | Absent | Absent | 0 | No |
| Halarcobacter | Absent | Absent | Absent | Absent | 0 | No |
| Haliangium | Absent | Absent | Absent | Absent | 0 | No |
| Haliscomenobacter | Absent | Absent | Absent | Absent | 0 | No |
| Halobacillus | Absent | Absent | Absent | Absent | 0 | No |
| Halodesulfovibrio | Absent | Absent | Absent | Absent | 0 | No |
| Hamadaea | Absent | Absent | Absent | Absent | 0 | No |
| Helcobacillus | Absent | Absent | Absent | Absent | 0 | No |
| Helicobacter | Absent | Absent | Absent | Absent | 0 | No |
| Heliimonas | Absent | Absent | Absent | Absent | 0 | No |
| Heliophilum | Absent | Absent | Absent | Absent | 0 | No |
| Herbaspirillum | Absent | Absent | Absent | Absent | 0 | No |
| Herbidospora | Absent | Absent | Absent | Absent | 0 | No |
| Herbinix | Absent | Absent | Absent | Absent | 0 | No |
| Herpetosiphon | Absent | Absent | Absent | Absent | 0 | No |
| hgcI_clade | Absent | Absent | Absent | Absent | 0 | No |
| Hirschia | Absent | Absent | Absent | Absent | 0 | No |
| HN-HF0106 | Absent | Absent | Absent | Absent | 0 | No |
| Holdemanella | Absent | Absent | Absent | Absent | 0 | No |
| Holdemania | Absent | Absent | Absent | Absent | 0 | No |
| HSB_OF53-F07 | Absent | Absent | Absent | Absent | 0 | No |
| Hungatella | Absent | Absent | Absent | Absent | 0 | No |
| Hydrocarboniphaga | Absent | Absent | Absent | Absent | 0 | No |
| Hydrogenedensaceae | Absent | Absent | Absent | Absent | 0 | No |
| Hydrogenispora | Absent | Absent | Absent | Absent | 0 | No |
| Hydrogenoanaerobacterium | Absent | Absent | Absent | Absent | 0 | No |
| Hydrogenophaga | Absent | Absent | Absent | Absent | 0 | No |
| Hydrogenophilus | Absent | Absent | Absent | Absent | 0 | No |
| Hydrotalea | Absent | Absent | Absent | Absent | 0 | No |
| Hymenobacter | Absent | Absent | Absent | Absent | 0 | No |
| Hyphomicrobium | Absent | Absent | Absent | Absent | 0 | No |
| Hyphomonas | Absent | Absent | Absent | Absent | 0 | No |
| Iamia | Absent | Absent | Absent | Absent | 0 | No |
| Ileibacterium | Absent | Absent | Absent | Absent | 0 | No |
| Ilumatobacter | Absent | Absent | Absent | Absent | 0 | No |
| IMCC26134 | Absent | Absent | Absent | Absent | 0 | No |
| IMCC26207 | Absent | Absent | Absent | Absent | 0 | No |
| IMCC26256 | Absent | Absent | Absent | Absent | 0 | No |
| Incertae_Sedis | Absent | Absent | Absent | Absent | 0 | No |
| Inhella | Absent | Absent | Absent | Absent | 0 | No |
| Inquilinus | Absent | Absent | Absent | Absent | 0 | No |
| Intestinibacter | Absent | Absent | Absent | Absent | 0 | No |
| Intestinimonas | Absent | Absent | Absent | Absent | 0 | No |
| IS-44 | Absent | Absent | Absent | Absent | 0 | No |
| Izemoplasmatales | Absent | Absent | Absent | Absent | 0 | No |
| Jatrophihabitans | Absent | Absent | Absent | Absent | 0 | No |
| JCM_18997 | Absent | Absent | Absent | Absent | 0 | No |
| Jeotgalibaca | Absent | Absent | Absent | Absent | 0 | No |
| Jeotgalibacillus | Absent | Absent | Absent | Absent | 0 | No |
| Jeotgalicoccus | Absent | Absent | Absent | Absent | 0 | No |
| JG30-KF-AS9 | Absent | Absent | Absent | Absent | 0 | No |
| JG30-KF-CM45 | Absent | Absent | Absent | Absent | 0 | No |
| JG30-KF-CM66 | Absent | Absent | Absent | Absent | 0 | No |
| JG30a-KF-32 | Absent | Absent | Absent | Absent | 0 | No |
| JG36-GS-52 | Absent | Absent | Absent | Absent | 0 | No |
| JG36-TzT-191 | Absent | Absent | Absent | Absent | 0 | No |
| JGI_0000069-P22 | Absent | Absent | Absent | Absent | 0 | No |
| JGI_0001001-H03 | Absent | Absent | Absent | Absent | 0 | No |
| JGI-0000079-D21 | Absent | Absent | Absent | Absent | 0 | No |
| Jonquetella | Absent | Absent | Absent | Absent | 0 | No |
| JS1 | Absent | Absent | Absent | Absent | 0 | No |
| JTB215 | Absent | Absent | Absent | Absent | 0 | No |
| JTB255_marine_benthic_group | Absent | Absent | Absent | Absent | 0 | No |
| Kaistia | Absent | Absent | Absent | Absent | 0 | No |
| Kapabacteriales | Absent | Absent | Absent | Absent | 0 | No |
| KD3-10 | Absent | Absent | Absent | Absent | 0 | No |
| KD3-93 | Absent | Absent | Absent | Absent | 0 | No |
| KD4-96 | Absent | Absent | Absent | Absent | 0 | No |
| Kerstersia | Absent | Absent | Absent | Absent | 0 | No |
| Ketobacter | Absent | Absent | Absent | Absent | 0 | No |
| KF-JG30-B3 | Absent | Absent | Absent | Absent | 0 | No |
| KI89A_clade | Absent | Absent | Absent | Absent | 0 | No |
| KIST-JJY010 | Absent | Absent | Absent | Absent | 0 | No |
| Kitasatospora | Absent | Absent | Absent | Absent | 0 | No |
| Kocuria | Absent | Absent | Absent | Absent | 0 | No |
| Kosakonia | Absent | Absent | Absent | Absent | 0 | No |
| Kouleothrix | Absent | Absent | Absent | Absent | 0 | No |
| Kribbella | Absent | Absent | Absent | Absent | 0 | No |
| Ktedonobacter | Absent | Absent | Absent | Absent | 0 | No |
| Kurthia | Absent | Absent | Absent | Absent | 0 | No |
| Kytococcus | Absent | Absent | Absent | Absent | 0 | No |
| Labrys | Absent | Absent | Absent | Absent | 0 | No |
| Lachnoanaerobaculum | Absent | Absent | Absent | Absent | 0 | No |
| Lachnoclostridium | Absent | Absent | Absent | Absent | 0 | No |
| Lachnospira | Absent | Absent | Absent | Absent | 0 | No |
| Lachnospiraceae_FCS020_group | Absent | Absent | Absent | Absent | 0 | No |
| Lachnospiraceae_ND3007_group | Absent | Absent | Absent | Absent | 0 | No |
| Lachnospiraceae_NK3A20_group | Absent | Absent | Absent | Absent | 0 | No |
| Lachnospiraceae_NK4A136_group | Absent | Absent | Absent | Absent | 0 | No |
| Lachnospiraceae_UCG-001 | Absent | Absent | Absent | Absent | 0 | No |
| Lachnospiraceae_UCG-003 | Absent | Absent | Absent | Absent | 0 | No |
| Lachnospiraceae_UCG-006 | Absent | Absent | Absent | Absent | 0 | No |
| Lachnospiraceae_UCG-010 | Absent | Absent | Absent | Absent | 0 | No |
| Lachnospiraceae_XPB1014_group | Absent | Absent | Absent | Absent | 0 | No |
| Lacibacter | Absent | Absent | Absent | Absent | 0 | No |
| Lactobacillus | Absent | Absent | Absent | Absent | 0 | No |
| Lacunisphaera | Absent | Absent | Absent | Absent | 0 | No |
| Larkinella | Absent | Absent | Absent | Absent | 0 | No |
| Latescibacteraceae | Absent | Absent | Absent | Absent | 0 | No |
| Latescibacterota | Absent | Absent | Absent | Absent | 0 | No |
| Lautropia | Absent | Absent | Absent | Absent | 0 | No |
| Lawsonella | Absent | Absent | Absent | Absent | 0 | No |
| Lawsonia | Absent | Absent | Absent | Absent | 0 | No |
| LD1-PB3 | Absent | Absent | Absent | Absent | 0 | No |
| LD29 | Absent | Absent | Absent | Absent | 0 | No |
| Lechevalieria | Absent | Absent | Absent | Absent | 0 | No |
| Legionella | Absent | Absent | Absent | Absent | 0 | No |
| Leifsonia | Absent | Absent | Absent | Absent | 0 | No |
| Lentimicrobiaceae | Absent | Absent | Absent | Absent | 0 | No |
| Lentimicrobium | Absent | Absent | Absent | Absent | 0 | No |
| Leptolinea | Absent | Absent | Absent | Absent | 0 | No |
| Leptolyngbya_ANT.L52.2 | Absent | Absent | Absent | Absent | 0 | No |
| Leptolyngbya_EcFYyyy-00 | Absent | Absent | Absent | Absent | 0 | No |
| Leptolyngbya_Es-Yyy1000 | Absent | Absent | Absent | Absent | 0 | No |
| Leptolyngbya_VRUC_135 | Absent | Absent | Absent | Absent | 0 | No |
| Leptotrichia | Absent | Absent | Absent | Absent | 0 | No |
| Leuconostoc | Absent | Absent | Absent | Absent | 0 | No |
| Levilinea | Absent | Absent | Absent | Absent | 0 | No |
| Lewinella | Absent | Absent | Absent | Absent | 0 | No |
| Limnobacter | Absent | Absent | Absent | Absent | 0 | No |
| Limnochordaceae | Absent | Absent | Absent | Absent | 0 | No |
| Limnohabitans | Absent | Absent | Absent | Absent | 0 | No |
| Lineage_IIa | Absent | Absent | Absent | Absent | 0 | No |
| Lineage_IIb | Absent | Absent | Absent | Absent | 0 | No |
| Lineage_IV | Absent | Absent | Absent | Absent | 0 | No |
| Litorilinea | Absent | Absent | Absent | Absent | 0 | No |
| LiUU-11-161 | Absent | Absent | Absent | Absent | 0 | No |
| Longilinea | Absent | Absent | Absent | Absent | 0 | No |
| Longimicrobiaceae | Absent | Absent | Absent | Absent | 0 | No |
| Longimicrobium | Absent | Absent | Absent | Absent | 0 | No |
| Longivirga | Absent | Absent | Absent | Absent | 0 | No |
| Luteibacter | Absent | Absent | Absent | Absent | 0 | No |
| Luteimonas | Absent | Absent | Absent | Absent | 0 | No |
| Luteitalea | Absent | Absent | Absent | Absent | 0 | No |
| Luteolibacter | Absent | Absent | Absent | Absent | 0 | No |
| Lutispora | Absent | Absent | Absent | Absent | 0 | No |
| LWQ8 | Absent | Absent | Absent | Absent | 0 | No |
| Lysinibacillus | Absent | Absent | Absent | Absent | 0 | No |
| Lysobacter | Absent | Absent | Absent | Absent | 0 | No |
| M2PB4-65_termite_group | Absent | Absent | Absent | Absent | 0 | No |
| M55-D21 | Absent | Absent | Absent | Absent | 0 | No |
| Macellibacteroides | Absent | Absent | Absent | Absent | 0 | No |
| Magnetospirillaceae | Absent | Absent | Absent | Absent | 0 | No |
| Magnetospirillum | Absent | Absent | Absent | Absent | 0 | No |
| Marinicella | Absent | Absent | Absent | Absent | 0 | No |
| Marinifilum | Absent | Absent | Absent | Absent | 0 | No |
| Marinilactibacillus | Absent | Absent | Absent | Absent | 0 | No |
| Marinimicrobium | Absent | Absent | Absent | Absent | 0 | No |
| Mariniradius | Absent | Absent | Absent | Absent | 0 | No |
| Marinococcus | Absent | Absent | Absent | Absent | 0 | No |
| Marvinbryantia | Absent | Absent | Absent | Absent | 0 | No |
| Massilia | Absent | Absent | Absent | Absent | 0 | No |
| MB-A2-108 | Absent | Absent | Absent | Absent | 0 | No |
| MBA03 | Absent | Absent | Absent | Absent | 0 | No |
| MBNT15 | Absent | Absent | Absent | Absent | 0 | No |
| Megamonas | Absent | Absent | Absent | Absent | 0 | No |
| Megasphaera | Absent | Absent | Absent | Absent | 0 | No |
| Meiothermus | Absent | Absent | Absent | Absent | 0 | No |
| Melittangium | Absent | Absent | Absent | Absent | 0 | No |
| Mesotoga | Absent | Absent | Absent | Absent | 0 | No |
| Methylobacter | Absent | Absent | Absent | Absent | 0 | No |
| Methylocella | Absent | Absent | Absent | Absent | 0 | No |
| Methyloligellaceae | Absent | Absent | Absent | Absent | 0 | No |
| Methylomicrobium | Absent | Absent | Absent | Absent | 0 | No |
| Methylomonas | Absent | Absent | Absent | Absent | 0 | No |
| Methylophilus | Absent | Absent | Absent | Absent | 0 | No |
| Methylotenera | Absent | Absent | Absent | Absent | 0 | No |
| Methyloversatilis | Absent | Absent | Absent | Absent | 0 | No |
| Microcoleus_Es-Yyy1400 | Absent | Absent | Absent | Absent | 0 | No |
| Microcoleus_PCC-7113 | Absent | Absent | Absent | Absent | 0 | No |
| Microcoleus_SAG_1449-1a | Absent | Absent | Absent | Absent | 0 | No |
| Microcystis_PCC-7914 | Absent | Absent | Absent | Absent | 0 | No |
| Microgenomatia | Absent | Absent | Absent | Absent | 0 | No |
| Microlunatus | Absent | Absent | Absent | Absent | 0 | No |
| Micromonospora | Absent | Absent | Absent | Absent | 0 | No |
| Micropepsaceae | Absent | Absent | Absent | Absent | 0 | No |
| Micropepsis | Absent | Absent | Absent | Absent | 0 | No |
| Micropruina | Absent | Absent | Absent | Absent | 0 | No |
| Microseira_Carmichael-Alabama | Absent | Absent | Absent | Absent | 0 | No |
| Microtrichales | Absent | Absent | Absent | Absent | 0 | No |
| Microvirga | Absent | Absent | Absent | Absent | 0 | No |
| Minicystis | Absent | Absent | Absent | Absent | 0 | No |
| Mitsuaria | Absent | Absent | Absent | Absent | 0 | No |
| Mitsuokella | Absent | Absent | Absent | Absent | 0 | No |
| Mizugakiibacter | Absent | Absent | Absent | Absent | 0 | No |
| mle1-27 | Absent | Absent | Absent | Absent | 0 | No |
| mle1-7 | Absent | Absent | Absent | Absent | 0 | No |
| mle1-8 | Absent | Absent | Absent | Absent | 0 | No |
| MM1 | Absent | Absent | Absent | Absent | 0 | No |
| MM2 | Absent | Absent | Absent | Absent | 0 | No |
| MND1 | Absent | Absent | Absent | Absent | 0 | No |
| Mobiluncus | Absent | Absent | Absent | Absent | 0 | No |
| Modestobacter | Absent | Absent | Absent | Absent | 0 | No |
| Mogibacterium | Absent | Absent | Absent | Absent | 0 | No |
| Moheibacter | Absent | Absent | Absent | Absent | 0 | No |
| Monoglobus | Absent | Absent | Absent | Absent | 0 | No |
| Morganella | Absent | Absent | Absent | Absent | 0 | No |
| Motilibacter | Absent | Absent | Absent | Absent | 0 | No |
| MSB-3C8 | Absent | Absent | Absent | Absent | 0 | No |
| MSB-4B10 | Absent | Absent | Absent | Absent | 0 | No |
| Mucilaginibacter | Absent | Absent | Absent | Absent | 0 | No |
| Mucispirillum | Absent | Absent | Absent | Absent | 0 | No |
| Murdochiella | Absent | Absent | Absent | Absent | 0 | No |
| Muribacter | Absent | Absent | Absent | Absent | 0 | No |
| Muribaculaceae | Absent | Absent | Absent | Absent | 0 | No |
| Muribaculum | Absent | Absent | Absent | Absent | 0 | No |
| MVP-15 | Absent | Absent | Absent | Absent | 0 | No |
| MVP-88 | Absent | Absent | Absent | Absent | 0 | No |
| MWH-UniP1_aquatic_group | Absent | Absent | Absent | Absent | 0 | No |
| Mycoplasma | Absent | Absent | Absent | Absent | 0 | No |
| Myxococcus | Absent | Absent | Absent | Absent | 0 | No |
| Nakamurella | Absent | Absent | Absent | Absent | 0 | No |
| Nannocystis | Absent | Absent | Absent | Absent | 0 | No |
| Natribacillus | Absent | Absent | Absent | Absent | 0 | No |
| Natronincola | Absent | Absent | Absent | Absent | 0 | No |
| Naumannella | Absent | Absent | Absent | Absent | 0 | No |
| NB1-j | Absent | Absent | Absent | Absent | 0 | No |
| Negativibacillus | Absent | Absent | Absent | Absent | 0 | No |
| Neisseria | Absent | Absent | Absent | Absent | 0 | No |
| Neochlamydia | Absent | Absent | Absent | Absent | 0 | No |
| Nesterenkonia | Absent | Absent | Absent | Absent | 0 | No |
| Nevskia | Absent | Absent | Absent | Absent | 0 | No |
| Niabella | Absent | Absent | Absent | Absent | 0 | No |
| Niastella | Absent | Absent | Absent | Absent | 0 | No |
| Nibrella | Absent | Absent | Absent | Absent | 0 | No |
| Nitriliruptoraceae | Absent | Absent | Absent | Absent | 0 | No |
| Nitrincola | Absent | Absent | Absent | Absent | 0 | No |
| Nitrolancea | Absent | Absent | Absent | Absent | 0 | No |
| Nitrosococcus | Absent | Absent | Absent | Absent | 0 | No |
| Nitrosomonas | Absent | Absent | Absent | Absent | 0 | No |
| Nitrosospira | Absent | Absent | Absent | Absent | 0 | No |
| Nitrospira | Absent | Absent | Absent | Absent | 0 | No |
| Niveispirillum | Absent | Absent | Absent | Absent | 0 | No |
| NK4A214_group | Absent | Absent | Absent | Absent | 0 | No |
| Nocardia | Absent | Absent | Absent | Absent | 0 | No |
| Nocardiopsis | Absent | Absent | Absent | Absent | 0 | No |
| Nonomuraea | Absent | Absent | Absent | Absent | 0 | No |
| Nordella | Absent | Absent | Absent | Absent | 0 | No |
| Nostoc_PCC-7524 | Absent | Absent | Absent | Absent | 0 | No |
| Noviherbaspirillum | Absent | Absent | Absent | Absent | 0 | No |
| Novispirillum | Absent | Absent | Absent | Absent | 0 | No |
| Novosphingobium | Absent | Absent | Absent | Absent | 0 | No |
| NS11-12_marine_group | Absent | Absent | Absent | Absent | 0 | No |
| NS3a_marine_group | Absent | Absent | Absent | Absent | 0 | No |
| NS9_marine_group | Absent | Absent | Absent | Absent | 0 | No |
| Nubsella | Absent | Absent | Absent | Absent | 0 | No |
| Obscuribacteraceae | Absent | Absent | Absent | Absent | 0 | No |
| Occallatibacter | Absent | Absent | Absent | Absent | 0 | No |
| Oceanobacillus | Absent | Absent | Absent | Absent | 0 | No |
| Oceanobacter | Absent | Absent | Absent | Absent | 0 | No |
| Odoribacter | Absent | Absent | Absent | Absent | 0 | No |
| Ohtaekwangia | Absent | Absent | Absent | Absent | 0 | No |
| Oikopleura | Absent | Absent | Absent | Absent | 0 | No |
| OLB12 | Absent | Absent | Absent | Absent | 0 | No |
| OLB13 | Absent | Absent | Absent | Absent | 0 | No |
| OLB14 | Absent | Absent | Absent | Absent | 0 | No |
| OLB8 | Absent | Absent | Absent | Absent | 0 | No |
| Oligoflexus | Absent | Absent | Absent | Absent | 0 | No |
| Olivibacter | Absent | Absent | Absent | Absent | 0 | No |
| Olsenella | Absent | Absent | Absent | Absent | 0 | No |
| OM190 | Absent | Absent | Absent | Absent | 0 | No |
| OM27_clade | Absent | Absent | Absent | Absent | 0 | No |
| OM60(NOR5)_clade | Absent | Absent | Absent | Absent | 0 | No |
| Omnitrophales | Absent | Absent | Absent | Absent | 0 | No |
| OPB41 | Absent | Absent | Absent | Absent | 0 | No |
| Opitutus | Absent | Absent | Absent | Absent | 0 | No |
| Oribacterium | Absent | Absent | Absent | Absent | 0 | No |
| Ornithinimicrobium | Absent | Absent | Absent | Absent | 0 | No |
| Oscillibacter | Absent | Absent | Absent | Absent | 0 | No |
| Oxalobacter | Absent | Absent | Absent | Absent | 0 | No |
| P2-11E | Absent | Absent | Absent | Absent | 0 | No |
| P3OB-42 | Absent | Absent | Absent | Absent | 0 | No |
| P3OB-51 | Absent | Absent | Absent | Absent | 0 | No |
| Paenalcaligenes | Absent | Absent | Absent | Absent | 0 | No |
| Paenarthrobacter | Absent | Absent | Absent | Absent | 0 | No |
| Paenochrobactrum | Absent | Absent | Absent | Absent | 0 | No |
| Pajaroellobacter | Absent | Absent | Absent | Absent | 0 | No |
| Paludibacter | Absent | Absent | Absent | Absent | 0 | No |
| Paludibaculum | Absent | Absent | Absent | Absent | 0 | No |
| Pandoraea | Absent | Absent | Absent | Absent | 0 | No |
| Pannonibacter | Absent | Absent | Absent | Absent | 0 | No |
| Pantalinema | Absent | Absent | Absent | Absent | 0 | No |
| Papillibacter | Absent | Absent | Absent | Absent | 0 | No |
| Parabacteroides | Absent | Absent | Absent | Absent | 0 | No |
| Paraclostridium | Absent | Absent | Absent | Absent | 0 | No |
| Parafilimonas | Absent | Absent | Absent | Absent | 0 | No |
| Parapedobacter | Absent | Absent | Absent | Absent | 0 | No |
| Paraprevotella | Absent | Absent | Absent | Absent | 0 | No |
| Parapusillimonas | Absent | Absent | Absent | Absent | 0 | No |
| Parasegetibacter | Absent | Absent | Absent | Absent | 0 | No |
| Parasutterella | Absent | Absent | Absent | Absent | 0 | No |
| Parcubacteria | Absent | Absent | Absent | Absent | 0 | No |
| Parvibacter | Absent | Absent | Absent | Absent | 0 | No |
| Parvibaculum | Absent | Absent | Absent | Absent | 0 | No |
| Parvimonas | Absent | Absent | Absent | Absent | 0 | No |
| Parviterribacter | Absent | Absent | Absent | Absent | 0 | No |
| PAUC26f | Absent | Absent | Absent | Absent | 0 | No |
| PB19 | Absent | Absent | Absent | Absent | 0 | No |
| Pectobacterium | Absent | Absent | Absent | Absent | 0 | No |
| Pediococcus | Absent | Absent | Absent | Absent | 0 | No |
| Pedobacter | Absent | Absent | Absent | Absent | 0 | No |
| Pedomicrobium | Absent | Absent | Absent | Absent | 0 | No |
| Pedosphaeraceae | Absent | Absent | Absent | Absent | 0 | No |
| Pelomonas | Absent | Absent | Absent | Absent | 0 | No |
| Pelospora | Absent | Absent | Absent | Absent | 0 | No |
| Pelotomaculum | Absent | Absent | Absent | Absent | 0 | No |
| PeM15 | Absent | Absent | Absent | Absent | 0 | No |
| Peptoclostridium | Absent | Absent | Absent | Absent | 0 | No |
| Peptococcus | Absent | Absent | Absent | Absent | 0 | No |
| Peptoniphilus | Absent | Absent | Absent | Absent | 0 | No |
| Perlucidibaca | Absent | Absent | Absent | Absent | 0 | No |
| Permianibacter | Absent | Absent | Absent | Absent | 0 | No |
| Persicitalea | Absent | Absent | Absent | Absent | 0 | No |
| Petrimonas | Absent | Absent | Absent | Absent | 0 | No |
| Phascolarctobacterium | Absent | Absent | Absent | Absent | 0 | No |
| Phaselicystis | Absent | Absent | Absent | Absent | 0 | No |
| Phenylobacterium | Absent | Absent | Absent | Absent | 0 | No |
| Phocea | Absent | Absent | Absent | Absent | 0 | No |
| Phormidesmis_ANT.L52.6 | Absent | Absent | Absent | Absent | 0 | No |
| Phormidium_IAM_M-71 | Absent | Absent | Absent | Absent | 0 | No |
| Phormidium_SAG_37.90 | Absent | Absent | Absent | Absent | 0 | No |
| PHOS-HE36 | Absent | Absent | Absent | Absent | 0 | No |
| Photobacterium | Absent | Absent | Absent | Absent | 0 | No |
| Phreatobacter | Absent | Absent | Absent | Absent | 0 | No |
| Phyllobacterium | Absent | Absent | Absent | Absent | 0 | No |
| Pigmentiphaga | Absent | Absent | Absent | Absent | 0 | No |
| Pir4_lineage | Absent | Absent | Absent | Absent | 0 | No |
| Pirellula | Absent | Absent | Absent | Absent | 0 | No |
| Piscinibacter | Absent | Absent | Absent | Absent | 0 | No |
| Pla3_lineage | Absent | Absent | Absent | Absent | 0 | No |
| Pla4_lineage | Absent | Absent | Absent | Absent | 0 | No |
| Planctopirus | Absent | Absent | Absent | Absent | 0 | No |
| Planifilum | Absent | Absent | Absent | Absent | 0 | No |
| Planktothrix_NIVA-CYA_15 | Absent | Absent | Absent | Absent | 0 | No |
| Planococcus | Absent | Absent | Absent | Absent | 0 | No |
| Planosporangium | Absent | Absent | Absent | Absent | 0 | No |
| Pleurocapsa_PCC-7319 | Absent | Absent | Absent | Absent | 0 | No |
| PLTA13 | Absent | Absent | Absent | Absent | 0 | No |
| Polyangium | Absent | Absent | Absent | Absent | 0 | No |
| Polycyclovorans | Absent | Absent | Absent | Absent | 0 | No |
| Pontibacter | Absent | Absent | Absent | Absent | 0 | No |
| Porphyrobacter | Absent | Absent | Absent | Absent | 0 | No |
| Porphyromonas | Absent | Absent | Absent | Absent | 0 | No |
| possible_genus_04 | Absent | Absent | Absent | Absent | 0 | No |
| Prauserella | Absent | Absent | Absent | Absent | 0 | No |
| Prevotella | Absent | Absent | Absent | Absent | 0 | No |
| Prevotellaceae_NK3B31_group | Absent | Absent | Absent | Absent | 0 | No |
| Prevotellaceae_UCG-001 | Absent | Absent | Absent | Absent | 0 | No |
| Prevotellaceae_UCG-003 | Absent | Absent | Absent | Absent | 0 | No |
| Prolixibacteraceae | Absent | Absent | Absent | Absent | 0 | No |
| Promicromonospora | Absent | Absent | Absent | Absent | 0 | No |
| Propionibacterium | Absent | Absent | Absent | Absent | 0 | No |
| Propioniciclava | Absent | Absent | Absent | Absent | 0 | No |
| Prosthecobacter | Absent | Absent | Absent | Absent | 0 | No |
| Prosthecomicrobium | Absent | Absent | Absent | Absent | 0 | No |
| Proteiniclasticum | Absent | Absent | Absent | Absent | 0 | No |
| Proteiniphilum | Absent | Absent | Absent | Absent | 0 | No |
| Proteocatella | Absent | Absent | Absent | Absent | 0 | No |
| Proteus | Absent | Absent | Absent | Absent | 0 | No |
| Providencia | Absent | Absent | Absent | Absent | 0 | No |
| Pseudacidovorax | Absent | Absent | Absent | Absent | 0 | No |
| Pseudanabaena_NgrPSln22 | Absent | Absent | Absent | Absent | 0 | No |
| Pseudanabaena_PCC-7429 | Absent | Absent | Absent | Absent | 0 | No |
| Pseudarthrobacter | Absent | Absent | Absent | Absent | 0 | No |
| Pseudoalteromonas | Absent | Absent | Absent | Absent | 0 | No |
| Pseudochrobactrum | Absent | Absent | Absent | Absent | 0 | No |
| Pseudocitrobacter | Absent | Absent | Absent | Absent | 0 | No |
| Pseudoflavitalea | Absent | Absent | Absent | Absent | 0 | No |
| Pseudofulvimonas | Absent | Absent | Absent | Absent | 0 | No |
| Pseudogracilibacillus | Absent | Absent | Absent | Absent | 0 | No |
| Pseudohongiella | Absent | Absent | Absent | Absent | 0 | No |
| Pseudokineococcus | Absent | Absent | Absent | Absent | 0 | No |
| Pseudolabrys | Absent | Absent | Absent | Absent | 0 | No |
| Pseudonocardia | Absent | Absent | Absent | Absent | 0 | No |
| Pseudonocardiaceae | Absent | Absent | Absent | Absent | 0 | No |
| Pseudopedobacter | Absent | Absent | Absent | Absent | 0 | No |
| Pseudorhodoferax | Absent | Absent | Absent | Absent | 0 | No |
| Pseudorhodoplanes | Absent | Absent | Absent | Absent | 0 | No |
| Pseudoxanthomonas | Absent | Absent | Absent | Absent | 0 | No |
| Psychrobacillus | Absent | Absent | Absent | Absent | 0 | No |
| Psychrobacter | Absent | Absent | Absent | Absent | 0 | No |
| Psychroglaciecola | Absent | Absent | Absent | Absent | 0 | No |
| Puia | Absent | Absent | Absent | Absent | 0 | No |
| Qingshengfania | Absent | Absent | Absent | Absent | 0 | No |
| R7C24 | Absent | Absent | Absent | Absent | 0 | No |
| Ralstonia | Absent | Absent | Absent | Absent | 0 | No |
| Ramlibacter | Absent | Absent | Absent | Absent | 0 | No |
| Raoultibacter | Absent | Absent | Absent | Absent | 0 | No |
| Rathayibacter | Absent | Absent | Absent | Absent | 0 | No |
| RB41 | Absent | Absent | Absent | Absent | 0 | No |
| RBG-13-54-9 | Absent | Absent | Absent | Absent | 0 | No |
| RCP2-54 | Absent | Absent | Absent | Absent | 0 | No |
| Reyranella | Absent | Absent | Absent | Absent | 0 | No |
| RF39 | Absent | Absent | Absent | Absent | 0 | No |
| Rhabdanaerobium | Absent | Absent | Absent | Absent | 0 | No |
| Rhabdobacter | Absent | Absent | Absent | Absent | 0 | No |
| Rhizobacter | Absent | Absent | Absent | Absent | 0 | No |
| Rhizocola | Absent | Absent | Absent | Absent | 0 | No |
| Rhodanobacter | Absent | Absent | Absent | Absent | 0 | No |
| Rhodobacter | Absent | Absent | Absent | Absent | 0 | No |
| Rhodoblastus | Absent | Absent | Absent | Absent | 0 | No |
| Rhodocytophaga | Absent | Absent | Absent | Absent | 0 | No |
| Rhodoferax | Absent | Absent | Absent | Absent | 0 | No |
| Rhodomicrobium | Absent | Absent | Absent | Absent | 0 | No |
| Rhodopirellula | Absent | Absent | Absent | Absent | 0 | No |
| Rhodoplanes | Absent | Absent | Absent | Absent | 0 | No |
| Rhodopseudomonas | Absent | Absent | Absent | Absent | 0 | No |
| Rhodothermaceae | Absent | Absent | Absent | Absent | 0 | No |
| Rhodovarius | Absent | Absent | Absent | Absent | 0 | No |
| Rhodovastum | Absent | Absent | Absent | Absent | 0 | No |
| Rickettsia | Absent | Absent | Absent | Absent | 0 | No |
| Rickettsiella | Absent | Absent | Absent | Absent | 0 | No |
| Rikenella | Absent | Absent | Absent | Absent | 0 | No |
| Rikenellaceae_RC9_gut_group | Absent | Absent | Absent | Absent | 0 | No |
| Robbsia | Absent | Absent | Absent | Absent | 0 | No |
| Rodentibacter | Absent | Absent | Absent | Absent | 0 | No |
| Rokubacteriales | Absent | Absent | Absent | Absent | 0 | No |
| Romboutsia | Absent | Absent | Absent | Absent | 0 | No |
| Roseburia | Absent | Absent | Absent | Absent | 0 | No |
| Roseiarcus | Absent | Absent | Absent | Absent | 0 | No |
| Roseimicrobium | Absent | Absent | Absent | Absent | 0 | No |
| Roseisolibacter | Absent | Absent | Absent | Absent | 0 | No |
| Rosenbergiella | Absent | Absent | Absent | Absent | 0 | No |
| Roseobacter_clade_CHAB-I-5_lineage | Absent | Absent | Absent | Absent | 0 | No |
| Roseococcus | Absent | Absent | Absent | Absent | 0 | No |
| Roseomonas | Absent | Absent | Absent | Absent | 0 | No |
| Rothia | Absent | Absent | Absent | Absent | 0 | No |
| Rubellimicrobium | Absent | Absent | Absent | Absent | 0 | No |
| Rubritaleaceae | Absent | Absent | Absent | Absent | 0 | No |
| Rubrobacter | Absent | Absent | Absent | Absent | 0 | No |
| Ruegeria | Absent | Absent | Absent | Absent | 0 | No |
| Rufibacter | Absent | Absent | Absent | Absent | 0 | No |
| Ruminiclostridium | Absent | Absent | Absent | Absent | 0 | No |
| Ruminococcaceae | Absent | Absent | Absent | Absent | 0 | No |
| Ruminococcus | Absent | Absent | Absent | Absent | 0 | No |
| Ruminofilibacter | Absent | Absent | Absent | Absent | 0 | No |
| Rummeliibacillus | Absent | Absent | Absent | Absent | 0 | No |
| S-BQ2-57_soil_group | Absent | Absent | Absent | Absent | 0 | No |
| S0134_terrestrial_group | Absent | Absent | Absent | Absent | 0 | No |
| S085 | Absent | Absent | Absent | Absent | 0 | No |
| Saccharibacillus | Absent | Absent | Absent | Absent | 0 | No |
| Saccharimonadaceae | Absent | Absent | Absent | Absent | 0 | No |
| Saccharimonadales | Absent | Absent | Absent | Absent | 0 | No |
| Saccharofermentans | Absent | Absent | Absent | Absent | 0 | No |
| Saccharopolyspora | Absent | Absent | Absent | Absent | 0 | No |
| Salinicoccus | Absent | Absent | Absent | Absent | 0 | No |
| Sandaracinobacter | Absent | Absent | Absent | Absent | 0 | No |
| Sandaracinus | Absent | Absent | Absent | Absent | 0 | No |
| Sanguibacter | Absent | Absent | Absent | Absent | 0 | No |
| SAR202_clade | Absent | Absent | Absent | Absent | 0 | No |
| SAR324_clade(Marine_group_B) | Absent | Absent | Absent | Absent | 0 | No |
| SB-5 | Absent | Absent | Absent | Absent | 0 | No |
| SBR1031 | Absent | Absent | Absent | Absent | 0 | No |
| SC-I-84 | Absent | Absent | Absent | Absent | 0 | No |
| Schlegelella | Absent | Absent | Absent | Absent | 0 | No |
| Schlesneria | Absent | Absent | Absent | Absent | 0 | No |
| Schumannella | Absent | Absent | Absent | Absent | 0 | No |
| Sedimentibacter | Absent | Absent | Absent | Absent | 0 | No |
| Sediminibacterium | Absent | Absent | Absent | Absent | 0 | No |
| Segetibacter | Absent | Absent | Absent | Absent | 0 | No |
| Sellimonas | Absent | Absent | Absent | Absent | 0 | No |
| Senegalimassilia | Absent | Absent | Absent | Absent | 0 | No |
| SepB-3 | Absent | Absent | Absent | Absent | 0 | No |
| Sericytochromatia | Absent | Absent | Absent | Absent | 0 | No |
| SH-PL14 | Absent | Absent | Absent | Absent | 0 | No |
| Sh765B-TzT-35 | Absent | Absent | Absent | Absent | 0 | No |
| SHA-26 | Absent | Absent | Absent | Absent | 0 | No |
| Shimazuella | Absent | Absent | Absent | Absent | 0 | No |
| Shinella | Absent | Absent | Absent | Absent | 0 | No |
| Silanimonas | Absent | Absent | Absent | Absent | 0 | No |
| Simplicispira | Absent | Absent | Absent | Absent | 0 | No |
| Singulisphaera | Absent | Absent | Absent | Absent | 0 | No |
| Sinomonas | Absent | Absent | Absent | Absent | 0 | No |
| Siphonobacter | Absent | Absent | Absent | Absent | 0 | No |
| SJA-15 | Absent | Absent | Absent | Absent | 0 | No |
| SJA-28 | Absent | Absent | Absent | Absent | 0 | No |
| Skermanella | Absent | Absent | Absent | Absent | 0 | No |
| Slackia | Absent | Absent | Absent | Absent | 0 | No |
| SM1A02 | Absent | Absent | Absent | Absent | 0 | No |
| SM2D12 | Absent | Absent | Absent | Absent | 0 | No |
| Smaragdicoccus | Absent | Absent | Absent | Absent | 0 | No |
| Snodgrassella | Absent | Absent | Absent | Absent | 0 | No |
| Soehngenia | Absent | Absent | Absent | Absent | 0 | No |
| Solibacillus | Absent | Absent | Absent | Absent | 0 | No |
| Solirubrobacter | Absent | Absent | Absent | Absent | 0 | No |
| Solirubrobacteraceae | Absent | Absent | Absent | Absent | 0 | No |
| Solitalea | Absent | Absent | Absent | Absent | 0 | No |
| Solobacterium | Absent | Absent | Absent | Absent | 0 | No |
| Sorangium | Absent | Absent | Absent | Absent | 0 | No |
| Sphaerisporangium | Absent | Absent | Absent | Absent | 0 | No |
| Sphaerochaeta | Absent | Absent | Absent | Absent | 0 | No |
| Sphingoaurantiacus | Absent | Absent | Absent | Absent | 0 | No |
| Sphingobium | Absent | Absent | Absent | Absent | 0 | No |
| Sphingopyxis | Absent | Absent | Absent | Absent | 0 | No |
| Spirochaeta | Absent | Absent | Absent | Absent | 0 | No |
| Spirochaeta_2 | Absent | Absent | Absent | Absent | 0 | No |
| Spirochaetota | Absent | Absent | Absent | Absent | 0 | No |
| Spiroplasma | Absent | Absent | Absent | Absent | 0 | No |
| Spirosoma | Absent | Absent | Absent | Absent | 0 | No |
| Sporacetigenium | Absent | Absent | Absent | Absent | 0 | No |
| Sporobacter | Absent | Absent | Absent | Absent | 0 | No |
| Sporocytophaga | Absent | Absent | Absent | Absent | 0 | No |
| Sporolactobacillus | Absent | Absent | Absent | Absent | 0 | No |
| Sporomusa | Absent | Absent | Absent | Absent | 0 | No |
| Sporosarcina | Absent | Absent | Absent | Absent | 0 | No |
| ST-12K33 | Absent | Absent | Absent | Absent | 0 | No |
| Stella | Absent | Absent | Absent | Absent | 0 | No |
| Stenotrophobacter | Absent | Absent | Absent | Absent | 0 | No |
| Steroidobacter | Absent | Absent | Absent | Absent | 0 | No |
| Streptomyces | Absent | Absent | Absent | Absent | 0 | No |
| Streptosporangium | Absent | Absent | Absent | Absent | 0 | No |
| Subdoligranulum | Absent | Absent | Absent | Absent | 0 | No |
| Subgroup_10 | Absent | Absent | Absent | Absent | 0 | No |
| Subgroup_11 | Absent | Absent | Absent | Absent | 0 | No |
| Subgroup_12 | Absent | Absent | Absent | Absent | 0 | No |
| Subgroup_13 | Absent | Absent | Absent | Absent | 0 | No |
| Subgroup_17 | Absent | Absent | Absent | Absent | 0 | No |
| Subgroup_18 | Absent | Absent | Absent | Absent | 0 | No |
| Subgroup_2 | Absent | Absent | Absent | Absent | 0 | No |
| Subgroup_20 | Absent | Absent | Absent | Absent | 0 | No |
| Subgroup_21 | Absent | Absent | Absent | Absent | 0 | No |
| Subgroup_22 | Absent | Absent | Absent | Absent | 0 | No |
| Subgroup_25 | Absent | Absent | Absent | Absent | 0 | No |
| Subgroup_5 | Absent | Absent | Absent | Absent | 0 | No |
| Subgroup_7 | Absent | Absent | Absent | Absent | 0 | No |
| Succinivibrio | Absent | Absent | Absent | Absent | 0 | No |
| Sulfuricurvum | Absent | Absent | Absent | Absent | 0 | No |
| Sulfuritalea | Absent | Absent | Absent | Absent | 0 | No |
| Sulfurospirillum | Absent | Absent | Absent | Absent | 0 | No |
| Sulfurovum | Absent | Absent | Absent | Absent | 0 | No |
| Sumerlaea | Absent | Absent | Absent | Absent | 0 | No |
| Sutterella | Absent | Absent | Absent | Absent | 0 | No |
| Sva0485 | Absent | Absent | Absent | Absent | 0 | No |
| SWB02 | Absent | Absent | Absent | Absent | 0 | No |
| Symbiobacterium | Absent | Absent | Absent | Absent | 0 | No |
| Synechococcus_IR11 | Absent | Absent | Absent | Absent | 0 | No |
| Syner-01 | Absent | Absent | Absent | Absent | 0 | No |
| Syntrophobacter | Absent | Absent | Absent | Absent | 0 | No |
| Syntrophomonas | Absent | Absent | Absent | Absent | 0 | No |
| Syntrophorhabdus | Absent | Absent | Absent | Absent | 0 | No |
| Tannerella | Absent | Absent | Absent | Absent | 0 | No |
| TC1 | Absent | Absent | Absent | Absent | 0 | No |
| Tellurimicrobium | Absent | Absent | Absent | Absent | 0 | No |
| Tepidanaerobacter | Absent | Absent | Absent | Absent | 0 | No |
| Tepidicella | Absent | Absent | Absent | Absent | 0 | No |
| Tepidimonas | Absent | Absent | Absent | Absent | 0 | No |
| Tepidiphilus | Absent | Absent | Absent | Absent | 0 | No |
| Tepidisphaerales | Absent | Absent | Absent | Absent | 0 | No |
| Terrabacter | Absent | Absent | Absent | Absent | 0 | No |
| Terribacillus | Absent | Absent | Absent | Absent | 0 | No |
| Terriglobus | Absent | Absent | Absent | Absent | 0 | No |
| Terrimicrobium | Absent | Absent | Absent | Absent | 0 | No |
| Terrimonas | Absent | Absent | Absent | Absent | 0 | No |
| Terrisporobacter | Absent | Absent | Absent | Absent | 0 | No |
| Tessaracoccus | Absent | Absent | Absent | Absent | 0 | No |
| Tetragenococcus | Absent | Absent | Absent | Absent | 0 | No |
| Thauera | Absent | Absent | Absent | Absent | 0 | No |
| Thermincola | Absent | Absent | Absent | Absent | 0 | No |
| Thermoactinomyces | Absent | Absent | Absent | Absent | 0 | No |
| Thermomonas | Absent | Absent | Absent | Absent | 0 | No |
| Thermosporothrix | Absent | Absent | Absent | Absent | 0 | No |
| Thermovirga | Absent | Absent | Absent | Absent | 0 | No |
| Thermus | Absent | Absent | Absent | Absent | 0 | No |
| Thiobacillus | Absent | Absent | Absent | Absent | 0 | No |
| Thiomonas | Absent | Absent | Absent | Absent | 0 | No |
| Thiothrix | Absent | Absent | Absent | Absent | 0 | No |
| Timonella | Absent | Absent | Absent | Absent | 0 | No |
| Tissierella | Absent | Absent | Absent | Absent | 0 | No |
| TK10 | Absent | Absent | Absent | Absent | 0 | No |
| TM7x | Absent | Absent | Absent | Absent | 0 | No |
| TRA3-20 | Absent | Absent | Absent | Absent | 0 | No |
| Treponema | Absent | Absent | Absent | Absent | 0 | No |
| Trichococcus | Absent | Absent | Absent | Absent | 0 | No |
| Truepera | Absent | Absent | Absent | Absent | 0 | No |
| Trueperella | Absent | Absent | Absent | Absent | 0 | No |
| Tumebacillus | Absent | Absent | Absent | Absent | 0 | No |
| Tundrisphaera | Absent | Absent | Absent | Absent | 0 | No |
| Turicella | Absent | Absent | Absent | Absent | 0 | No |
| Turneriella | Absent | Absent | Absent | Absent | 0 | No |
| Tuzzerella | Absent | Absent | Absent | Absent | 0 | No |
| Tychonema_CCAP_1459-11B | Absent | Absent | Absent | Absent | 0 | No |
| type_III | Absent | Absent | Absent | Absent | 0 | No |
| Tyzzerella | Absent | Absent | Absent | Absent | 0 | No |
| UBA12409 | Absent | Absent | Absent | Absent | 0 | No |
| UBA1819 | Absent | Absent | Absent | Absent | 0 | No |
| UCG-002 | Absent | Absent | Absent | Absent | 0 | No |
| UCG-003 | Absent | Absent | Absent | Absent | 0 | No |
| UCG-004 | Absent | Absent | Absent | Absent | 0 | No |
| UCG-005 | Absent | Absent | Absent | Absent | 0 | No |
| UCG-008 | Absent | Absent | Absent | Absent | 0 | No |
| UCG-009 | Absent | Absent | Absent | Absent | 0 | No |
| UCG-010 | Absent | Absent | Absent | Absent | 0 | No |
| UCG-012 | Absent | Absent | Absent | Absent | 0 | No |
| Umezawaea | Absent | Absent | Absent | Absent | 0 | No |
| Undibacterium | Absent | Absent | Absent | Absent | 0 | No |
| URHD0088 | Absent | Absent | Absent | Absent | 0 | No |
| UTCFX1 | Absent | Absent | Absent | Absent | 0 | No |
| vadinHA49 | Absent | Absent | Absent | Absent | 0 | No |
| Vagococcus | Absent | Absent | Absent | Absent | 0 | No |
| Vampirovibrio | Absent | Absent | Absent | Absent | 0 | No |
| Vampirovibrionaceae | Absent | Absent | Absent | Absent | 0 | No |
| Variovorax | Absent | Absent | Absent | Absent | 0 | No |
| Veillonella | Absent | Absent | Absent | Absent | 0 | No |
| Vermiphilaceae | Absent | Absent | Absent | Absent | 0 | No |
| Verrucomicrobium | Absent | Absent | Absent | Absent | 0 | No |
| Verticiella | Absent | Absent | Absent | Absent | 0 | No |
| Vibrio | Absent | Absent | Absent | Absent | 0 | No |
| Vibrionimonas | Absent | Absent | Absent | Absent | 0 | No |
| Vicinamibacter | Absent | Absent | Absent | Absent | 0 | No |
| Vicinamibacteraceae | Absent | Absent | Absent | Absent | 0 | No |
| Vicingus | Absent | Absent | Absent | Absent | 0 | No |
| Virgibacillus | Absent | Absent | Absent | Absent | 0 | No |
| Virgisporangium | Absent | Absent | Absent | Absent | 0 | No |
| Vitellibacter | Absent | Absent | Absent | Absent | 0 | No |
| Vitreoscilla | Absent | Absent | Absent | Absent | 0 | No |
| Vogesella | Absent | Absent | Absent | Absent | 0 | No |
| Vulcaniibacterium | Absent | Absent | Absent | Absent | 0 | No |
| Vulgatibacter | Absent | Absent | Absent | Absent | 0 | No |
| W27 | Absent | Absent | Absent | Absent | 0 | No |
| W5053 | Absent | Absent | Absent | Absent | 0 | No |
| WCHB1-32 | Absent | Absent | Absent | Absent | 0 | No |
| WCHB1-41 | Absent | Absent | Absent | Absent | 0 | No |
| WD2101_soil_group | Absent | Absent | Absent | Absent | 0 | No |
| WD260 | Absent | Absent | Absent | Absent | 0 | No |
| Williamsia | Absent | Absent | Absent | Absent | 0 | No |
| Wolbachia | Absent | Absent | Absent | Absent | 0 | No |
| WPS-2 | Absent | Absent | Absent | Absent | 0 | No |
| WS2 | Absent | Absent | Absent | Absent | 0 | No |
| WS4 | Absent | Absent | Absent | Absent | 0 | No |
| WWE3 | Absent | Absent | Absent | Absent | 0 | No |
| WX65 | Absent | Absent | Absent | Absent | 0 | No |
| Xanthobacter | Absent | Absent | Absent | Absent | 0 | No |
| Xanthomonas | Absent | Absent | Absent | Absent | 0 | No |
| YC-ZSS-LKJ147 | Absent | Absent | Absent | Absent | 0 | No |
| YC-ZSS-LKJ63 | Absent | Absent | Absent | Absent | 0 | No |
| Zavarzinella | Absent | Absent | Absent | Absent | 0 | No |
| Zavarzinia | Absent | Absent | Absent | Absent | 0 | No |
| Zixibacteria | Absent | Absent | Absent | Absent | 0 | No |
| Zoogloea | Absent | Absent | Absent | Absent | 0 | No |

## Supplementary Table 2B

| **ID** | **k-means** | **Hierarchical** | **DBSCAN** | **Gaussian Mixture** | **Spectral** | **Kingdom** | **Phylum** | **Class** | **Order** | **Family** | **Genus** | **Species** |
| --- | --- | --- | --- | --- | --- | --- | --- | --- | --- | --- | --- | --- |
| 139176fcc8a17d683fcd1c3e3f0ba066 | 1 | 0 | 0 | 1 | 1 | Bacteria | Proteobacteria | Alphaproteobacteria | Rhizobiales | Rhizobiaceae | Ochrobactrum |  |
| 19425594 | 1 | 0 | 0 | 1 | 1 | Bacteria | Firmicutes | Bacilli | Erysipelotrichales | Erysipelotrichaceae | ZOR0006 | uncultured_organism |
| 20952725 | 1 | 1 | 0 | 1 | 1 | Bacteria | Firmicutes | Bacilli | Erysipelotrichales | Erysipelotrichaceae | Dubosiella | uncultured_bacterium |
| 4f6f18111fc283699d88b01b81919fe9 | 1 | 1 | 0 | 1 | 1 | Bacteria | Firmicutes | Bacilli | Lactobacillales | Enterococcaceae | Enterococcus |  |
| 57775516161b8b8f28cf889bee97adc9 | 1 | 0 | 0 | 1 | 1 | Bacteria | Chloroflexi | Ktedonobacteria | Ktedonobacterales | Ktedonobacteraceae | Ktedonobacter |  |
| 5d6066c6353ac3de9fbddf986ef0f75b | 1 | 0 | 0 | 1 | 1 | Bacteria | Firmicutes | Clostridia | Lachnospirales | Lachnospiraceae | Lachnospira |  |
| 5e81c85a47970077e5aeba59d40a351b | 1 | 0 | 0 | 1 | 1 | Bacteria | Firmicutes | Bacilli | Lactobacillales | Enterococcaceae | Enterococcus |  |
| 5e8c591558ac6cd7305504a46e29ff83 | 1 | 0 | 0 | 1 | 1 | Bacteria | Firmicutes | Bacilli | Lactobacillales | Enterococcaceae | Enterococcus |  |
| 621f33c02c5ebd06d2dba974d989fedd | 1 | 0 | 0 | 1 | 1 | Bacteria | Bacteroidota | Bacteroidia | Cytophagales | Spirosomaceae | Leadbetterella | Leadbetterella_sp. |
| 77ac8bc3d31c1e89cf7efdf5400c771b | 1 | 0 | 0 | 1 | 1 | Bacteria | Bacteroidota | Bacteroidia | Bacteroidales | Muribaculaceae | Muribaculaceae |  |
| 8bd8f7087d47c8ba0a15e3e88d015b0a | 0 | 1 | 0 | 1 | 1 | Bacteria | Firmicutes | Bacilli | Lactobacillales | Enterococcaceae | Enterococcus |  |
| 9c172d83dbdaa1acf6aeaad7f0ad5792 | 1 | 1 | 0 | 1 | 1 | Bacteria | Proteobacteria | Gammaproteobacteria | Enterobacterales |  |  |  |
| 9c8c1b3b256ce8d328f96428c072c72f | 1 | 1 | 0 | 1 | 1 | Bacteria | Proteobacteria | Alphaproteobacteria | Rhizobiales | Rhizobiaceae |  |  |
| 9e8b3d4bcab81c104b314d6a0b83d2c4 | 0 | 1 | 0 | 1 | 1 | Bacteria | Proteobacteria | Gammaproteobacteria | Pseudomonadales | Moraxellaceae | Acinetobacter |  |
| AB109209 | 1 | 1 | 0 | 1 | 1 | Bacteria | Firmicutes | Bacilli | Bacillales | Bacillaceae | Bacillus |  |
| AF000221 | 0 | 1 | 0 | 1 | 1 | Bacteria | Campilobacterota | Campylobacteria | Campylobacterales | Helicobacteraceae | Helicobacter |  |
| AF480599 | 1 | 1 | 0 | 1 | 1 | Bacteria | Actinobacteriota | Actinobacteria | Corynebacteriales | Mycobacteriaceae | Mycobacterium |  |
| AJ430344 | 1 | 0 | 0 | 1 | 1 | Bacteria | Proteobacteria | Gammaproteobacteria | Burkholderiales | Comamonadaceae | Comamonas | Comamonas_aquatica |
| AJ853909 | 1 | 0 | 0 | 1 | 1 | Bacteria | Actinobacteriota | Actinobacteria | Micrococcales | Microbacteriaceae | Microbacterium |  |
| AM422371 | 1 | 0 | 0 | 1 | 1 | Bacteria | Proteobacteria | Alphaproteobacteria | Rhizobiales | Rhizobiaceae |  |  |
| DQ883810 | 1 | 0 | 0 | 1 | 1 | Bacteria | Proteobacteria | Alphaproteobacteria | Rhizobiales | Rhizobiaceae |  |  |
| EU290155 | 1 | 0 | 0 | 1 | 1 | Bacteria | Proteobacteria | Gammaproteobacteria | Pseudomonadales | Moraxellaceae | Acinetobacter | Acinetobacter_soli |
| EU873314 | 1 | 1 | 0 | 1 | 1 | Bacteria | Actinobacteriota | Actinobacteria | Micrococcales | Microbacteriaceae | Microbacterium |  |
| FN870023 | 1 | 0 | 0 | 1 | 1 | Bacteria | Actinobacteriota | Actinobacteria | Micrococcales | Microbacteriaceae | Microbacterium |  |
| FR775448 | 1 | 0 | 0 | 1 | 1 | Bacteria | Proteobacteria | Alphaproteobacteria | Caulobacterales | Caulobacteraceae | Brevundimonas |  |
| G000488255_5 | 1 | 0 | 0 | 1 | 1 | Bacteria | Proteobacteria | Gammaproteobacteria | Pseudomonadales | Moraxellaceae | Acinetobacter | Acinetobacter_indicus |
| G000759675_1 | 0 | 1 | 0 | 1 | 1 | Bacteria | Firmicutes | Bacilli | Bacillales | Bacillaceae | Bacillus |  |
| G000779535_1 | 1 | 0 | 0 | 1 | 1 | Bacteria | Proteobacteria | Gammaproteobacteria | Enterobacterales | Enterobacteriaceae | Escherichia-Shigella |  |
| G001421785_2 | 1 | 1 | 0 | 1 | 1 | Bacteria | Proteobacteria | Alphaproteobacteria | Sphingomonadales | Sphingomonadaceae | Sphingomonas |  |
| G001945525_1 | 1 | 1 | 0 | 1 | 1 | Bacteria | Firmicutes | Bacilli | Erysipelotrichales | Erysipelotrichaceae | Ileibacterium | Ileibacterium_valens |
| G004127505_2 | 1 | 1 | 0 | 1 | 1 | Bacteria | Firmicutes | Bacilli | Staphylococcales | Staphylococcaceae | Staphylococcus |  |
| G009781045_1 | 0 | 1 | 0 | 1 | 1 | Bacteria | Proteobacteria | Alphaproteobacteria | Rhizobiales | Xanthobacteraceae |  |  |
| GB-GCA-003472845.1-QRMZ01000074.1 | 1 | 1 | 0 | 1 | 1 | Bacteria | Firmicutes | Bacilli | Lactobacillales | Enterococcaceae | Enterococcus |  |
| GB-GCA-004793885.1-SRYG01000051.1 | 1 | 1 | 0 | 1 | 1 | Bacteria | Firmicutes | Bacilli | Erysipelotrichales | Erysipelotrichaceae | Dubosiella | uncultured_bacterium |
| GB-GCA-015554585.1-JADNIT010000076.1 | 1 | 0 | 0 | 1 | 1 | Bacteria | Firmicutes | Clostridia | Lachnospirales | Lachnospiraceae | Agathobacter |  |
| GB-GCA-900762515.1-CAAFHA010000156.1 | 0 | 1 | 0 | 1 | 1 | Bacteria | Firmicutes | Bacilli | Erysipelotrichales | Erysipelatoclostridiaceae | Erysipelotrichaceae_UCG-003 |  |
| GB-GCA-902636635.1-CACPRY010000089.1 | 1 | 0 | 0 | 1 | 1 | Bacteria | Proteobacteria | Gammaproteobacteria | Enterobacterales | Enterobacteriaceae |  |  |
| HF674688 | 1 | 1 | 0 | 1 | 1 | Bacteria | Proteobacteria | Gammaproteobacteria | Burkholderiales | Burkholderiaceae | Burkholderia-Caballeronia-Paraburkholderia |  |
| JN015213 | 1 | 0 | 0 | 1 | 1 | Bacteria | Bacteroidota | Bacteroidia | Sphingobacteriales | Sphingobacteriaceae | Sphingobacterium |  |
| JQ659512 | 1 | 0 | 0 | 1 | 1 | Bacteria | Proteobacteria | Alphaproteobacteria | Sphingomonadales | Sphingomonadaceae | Sphingomonas |  |
| KM407667 | 1 | 0 | 0 | 1 | 1 | Bacteria | Proteobacteria | Alphaproteobacteria | Rhodobacterales | Rhodobacteraceae | Gemmobacter | Gemmobacter_intermedius |
| KT997477 | 1 | 0 | 0 | 1 | 1 | Bacteria | Proteobacteria | Gammaproteobacteria | Pseudomonadales | Moraxellaceae | Acinetobacter |  |
| KU201960 | 1 | 1 | 0 | 1 | 1 | Bacteria | Bacteroidota | Bacteroidia | Sphingobacteriales | Sphingobacteriaceae | Sphingobacterium | Sphingobacterium_zeae |
| LC405931 | 1 | 0 | 0 | 1 | 1 | Bacteria | Actinobacteriota | Actinobacteria | Kineosporiales | Kineosporiaceae | Quadrisphaera | Quadrisphaera_granulorum |
| LT009500 | 1 | 0 | 0 | 1 | 1 | Bacteria | Proteobacteria | Gammaproteobacteria | Burkholderiales | Comamonadaceae | Comamonas | Comamonas_sediminis |
| MJ006-1-barcode39-umi142106bins-ubs-3 | 1 | 0 | 0 | 1 | 1 | Bacteria | Firmicutes | Clostridia | Oscillospirales | Ruminococcaceae | Faecalibacterium |  |
| MJ006-2-barcode71-umi136802bins-ubs-4 | 1 | 0 | 0 | 1 | 1 | Bacteria | Firmicutes | Clostridia | Lachnospirales | Lachnospiraceae | Coprococcus |  |
| MJ007-1-barcode36-umi14966bins-ubs-17 | 0 | 1 | 0 | 1 | 1 | Bacteria | Proteobacteria | Gammaproteobacteria | Pseudomonadales | Moraxellaceae | Acinetobacter |  |
| MJ007-1-barcode36-umi41898bins-ubs-3 | 1 | 1 | 0 | 1 | 1 | Bacteria | Bacteroidota | Bacteroidia | Flavobacteriales | Flavobacteriaceae | Myroides | Myroides_odoratus |
| MJ010-1-barcode25-umi77945bins-ubs-9 | 1 | 0 | 0 | 1 | 1 | Bacteria | Proteobacteria | Gammaproteobacteria | Pseudomonadales | Moraxellaceae | Acinetobacter |  |
| MJ010-1-barcode47-umi10570bins-ubs-50 | 1 | 0 | 0 | 1 | 1 | Bacteria | Bacteroidota | Bacteroidia | Sphingobacteriales | Sphingobacteriaceae | Sphingobacterium |  |
| MJ010-1-barcode47-umi132914bins-ubs-4 | 1 | 0 | 0 | 1 | 1 | Bacteria | Proteobacteria | Gammaproteobacteria | Pseudomonadales | Moraxellaceae | Acinetobacter |  |
| MJ031-1-barcode35-umi10775bins-ubs-53 | 1 | 0 | 0 | 1 | 1 | Bacteria | Proteobacteria | Gammaproteobacteria | Pseudomonadales | Moraxellaceae | Acinetobacter |  |
| MJ031-2-barcode69-umi51122bins-ubs-3 | 1 | 1 | 0 | 1 | 1 | Bacteria | Firmicutes | Bacilli | Lactobacillales | Enterococcaceae | Enterococcus |  |
| MJ032-2-barcode73-umi9961bins-ubs-47 | 1 | 0 | 0 | 1 | 1 | Bacteria | Proteobacteria | Gammaproteobacteria | Enterobacterales |  |  |  |
| MK940276 | 1 | 0 | 0 | 1 | 1 | Bacteria | Proteobacteria | Alphaproteobacteria | Rhizobiales | Rhizobiaceae | Allorhizobium-Neorhizobium-Pararhizobium-Rhizobium |  |
| RS-GCF-000613205.1-NZ-CBXW010000058.1 | 1 | 1 | 0 | 1 | 1 | Bacteria | Proteobacteria | Gammaproteobacteria | Xanthomonadales | Xanthomonadaceae | Stenotrophomonas |  |
| RS-GCF-000632205.1-NZ-JFYW01000015.1 | 1 | 1 | 0 | 1 | 1 | Bacteria | Firmicutes | Bacilli | Exiguobacterales | Exiguobacteraceae | Exiguobacterium |  |
| RS-GCF-001552115.1-NZ-BCQO01000012.1 | 1 | 0 | 0 | 1 | 1 | Bacteria | Actinobacteriota | Actinobacteria | Micrococcales | Micrococcaceae | Glutamicibacter |  |
| RS-GCF-001575215.1-NZ-LSRI01000014.1 | 1 | 1 | 0 | 1 | 1 | Bacteria | Actinobacteriota | Actinobacteria | Corynebacteriales | Tsukamurellaceae | Tsukamurella |  |
| RS-GCF-002144475.1-NZ-MBOU01000032.1 | 1 | 1 | 0 | 1 | 1 | Bacteria | Proteobacteria | Gammaproteobacteria | Enterobacterales | Enterobacteriaceae |  |  |
| RS-GCF-003289665.1-NZ-QMBM01000001.1--2 | 1 | 0 | 0 | 1 | 1 | Bacteria | Proteobacteria | Gammaproteobacteria | Pseudomonadales | Moraxellaceae | Acinetobacter |  |
| RS-GCF-003401015.1-NZ-PXLK01000057.1 | 1 | 0 | 0 | 1 | 1 | Bacteria | Proteobacteria | Gammaproteobacteria | Enterobacterales | Enterobacteriaceae |  |  |
| RS-GCF-003461005.1-NZ-QTWG01000138.1 | 1 | 1 | 0 | 1 | 1 | Bacteria | Firmicutes | Clostridia | Lachnospirales | Lachnospiraceae | [Ruminococcus]_torques_group |  |
| RS-GCF-008041935.1-NZ-VKAC01000026.1 | 1 | 0 | 0 | 1 | 1 | Bacteria | Actinobacteriota | Actinobacteria | Kineosporiales | Kineosporiaceae | Quadrisphaera |  |
| RS-GCF-009013695.1-NZ-BKXR01000133.1 | 0 | 1 | 0 | 1 | 1 | Bacteria | Proteobacteria | Gammaproteobacteria | Pseudomonadales | Moraxellaceae | Acinetobacter |  |
| RS-GCF-009906335.1-NZ-JAAALX010000087.1 | 1 | 0 | 0 | 1 | 1 | Bacteria | Proteobacteria | Gammaproteobacteria | Aeromonadales |  |  |  |
| RS-GCF-013179615.1-NZ-JABMCO010000328.1 | 1 | 1 | 0 | 1 | 1 | Bacteria | Actinobacteriota | Actinobacteria | Micrococcales | Micrococcaceae | Glutamicibacter |  |
| RS-GCF-013180515.1-NZ-PYWU01000001.1--4 | 1 | 1 | 0 | 1 | 1 | Bacteria | Proteobacteria | Gammaproteobacteria | Enterobacterales | Enterobacteriaceae |  |  |
| RS-GCF-014397025.1-NZ-JACJUO010000001.1 | 1 | 1 | 0 | 1 | 1 | Bacteria | Proteobacteria | Alphaproteobacteria | Rhizobiales | Rhizobiaceae | Falsochrobactrum |  |
| RS-GCF-014646955.1-NZ-BMOG01000032.1 | 1 | 1 | 0 | 1 | 1 | Bacteria | Proteobacteria | Gammaproteobacteria | Pseudomonadales | Pseudomonadaceae | Pseudomonas |  |
| RS-GCF-014894875.1-NZ-JACXYT010000012.1 | 1 | 1 | 0 | 1 | 1 | Bacteria | Proteobacteria | Gammaproteobacteria | Enterobacterales | Yersiniaceae | Serratia |  |
| RS-GCF-017776345.1-NZ-JAFIQO010000082.1 | 1 | 0 | 0 | 1 | 1 | Bacteria | Firmicutes | Clostridia | Lachnospirales | Lachnospiraceae | [Eubacterium]_hallii_group |  |
| RS-GCF-018064205.1-NZ-CP072916.1--8 | 1 | 0 | 0 | 1 | 1 | Bacteria | Proteobacteria | Gammaproteobacteria | Burkholderiales | Comamonadaceae | Comamonas | Comamonas_aquatica |
| RS-GCF-018784445.1-NZ-WQOH01000081.1 | 1 | 0 | 0 | 1 | 1 | Bacteria | Firmicutes | Clostridia | Oscillospirales | Ruminococcaceae | Subdoligranulum |  |
| RS-GCF-900102995.1-NZ-FNKT01000009.1 | 1 | 1 | 0 | 1 | 1 | Bacteria | Proteobacteria | Alphaproteobacteria | Caulobacterales | Caulobacteraceae | Brevundimonas |  |
| RS-GCF-900114755.1-NZ-FOUC01000047.1 | 0 | 1 | 0 | 1 | 1 | Bacteria | Firmicutes | Bacilli | Lactobacillales | Enterococcaceae | Enterococcus |  |
| X80739 | 0 | 1 | 0 | 1 | 1 | Bacteria | Actinobacteriota | Actinobacteria | Micrococcales | Micrococcaceae | Glutamicibacter |  |
| a19b13c675ae9d1fcb2e7a17d2eed67a | 1 | 1 | 0 | 1 | 1 | Bacteria | Actinobacteriota | Actinobacteria | Micrococcales | Microbacteriaceae | Microbacterium |  |
| aec9d89f544f86c50ef82d23e685ead1 | 0 | 1 | 0 | 1 | 1 | Bacteria | Firmicutes | Bacilli | Lactobacillales | Enterococcaceae | Enterococcus |  |
| bf8affacb07c895b1fc2b577ecbe47b8 | 1 | 1 | 0 | 1 | 1 | Bacteria | Proteobacteria | Gammaproteobacteria | Cellvibrionales | Cellvibrionaceae | Cellvibrio |  |
| c33a7f5ea399062e47df96fe3787be04 | 1 | 1 | 0 | 1 | 1 | Bacteria | Firmicutes | Bacilli | Erysipelotrichales | Erysipelatoclostridiaceae | Erysipelatoclostridium | uncultured_Clostridium |
| c8fc8663c6b5f491c8f171e61f87cfcb | 0 | 1 | 0 | 1 | 1 | Bacteria | Firmicutes | Bacilli | Lactobacillales | Enterococcaceae | Enterococcus |  |
| d76e561c51d3d36168d0b953bfe79e55 | 1 | 0 | 0 | 1 | 1 | Bacteria | Firmicutes | Clostridia | Lachnospirales | Lachnospiraceae |  |  |
| da06629d3beaa4e2dc2148037b8ebf38 | 1 | 0 | 0 | 1 | 1 | Bacteria | Actinobacteriota | Actinobacteria | Micrococcales | Micrococcaceae | Glutamicibacter |  |
| f9d5311ae5c8aa9f62ae03e42141c661 | 1 | 0 | 0 | 1 | 1 | Bacteria | Proteobacteria | Gammaproteobacteria | Burkholderiales | Burkholderiaceae | Burkholderia-Caballeronia-Paraburkholderia |  |
| fb7d1d7ff193799cc1387d4d71437f9a | 1 | 0 | 0 | 1 | 1 | Bacteria | Firmicutes | Bacilli | Lactobacillales | Enterococcaceae | Enterococcus |  |
| ffa80f6a3a62b0fd2113ee6cd2f6d263 | 1 | 0 | 0 | 1 | 1 | Bacteria | Firmicutes | Bacilli | Lactobacillales | Enterococcaceae | Enterococcus |  |

## Supplementary Table 3

**Supplementary Table S3. ASVs retained in the bipartite network analysis and their corresponding taxonomic annotations.**

| **ASV ID** | **Kingdom** | **Phylum** | **Class** | **Order** | **Family** | **Genus** | **Species** |
| --- | --- | --- | --- | --- | --- | --- | --- |
| a32e8d7e50b6abb310a93314fd19a5e5 | Bacteria | p__Planctomycetota | c__Phycisphaerae | o__Tepidisphaerales | f__WD2101_soil_group | g__WD2101_soil_group | s__uncultured_bacterium |
| RS-GCF-900184035.1-NZ-FXWP01000029.1 | Bacteria | p__Proteobacteria | c__Gammaproteobacteria | o__Enterobacterales | f__Enterobacteriaceae |  |  |
| 6bf3f1a17e2c60fdab2e7863467f306c | Bacteria | p__Firmicutes | c__Bacilli | o__Lactobacillales | f__Lactobacillaceae | g__Lactobacillus |  |
| 04908712 | Bacteria | p__Proteobacteria | c__Alphaproteobacteria | o__Rhizobiales | f__Rhizobiaceae | g__Aureimonas |  |
| c2bd4d8f0a8acb1b5840ba235b1bb470 | Bacteria | p__Latescibacterota | c__Latescibacterota | o__Latescibacterota | f__Latescibacterota | g__Latescibacterota |  |
| 18930876 | Bacteria | p__Proteobacteria | c__Gammaproteobacteria | o__Burkholderiales | f__Comamonadaceae |  |  |
| 00903346 | Bacteria | p__Bacteroidota | c__Bacteroidia | o__Bacteroidales | f__Marinifilaceae | g__Odoribacter | s__uncultured_bacterium |
| AB009828 | Bacteria | p__Proteobacteria | c__Gammaproteobacteria | o__Burkholderiales | f__Hydrogenophilaceae | g__Hydrogenophilus |  |
| dfc565ca1a5d37b28e85f7631a03694d | Bacteria | p__Proteobacteria | c__Gammaproteobacteria | o__Alteromonadales | f__Alteromonadaceae | g__Rheinheimera |  |
| 04928741 | Bacteria | p__Proteobacteria | c__Alphaproteobacteria | o__Rhodobacterales | f__Rhodobacteraceae | g__Rubellimicrobium |  |
| 8a288791eea6ec8aadba3865def75133 | Bacteria | p__Actinobacteriota | c__Thermoleophilia | o__Gaiellales |  |  |  |
| 8867fe2c33e6bc89d1b10fd76696c07b | Bacteria | p__Proteobacteria | c__Gammaproteobacteria | o__Legionellales | f__Legionellaceae | g__Legionella |  |
| 9a2d617244d1c7480665a5c7f7ed3da5 | Bacteria | p__Proteobacteria | c__Alphaproteobacteria | o__Rhizobiales | f__Rhizobiaceae | g__Allorhizobium-Neorhizobium-Pararhizobium-Rhizobium |  |
| 68b17e8374833c3d23054dd61c856e46 | Bacteria | p__Proteobacteria | c__Alphaproteobacteria | o__Rhizobiales | f__Rhizobiales_Incertae_Sedis | g__uncultured |  |
| GB-GCA-016839025.1-JADLKK010000299.1 | Bacteria | p__Firmicutes | c__Bacilli | o__Bacillales | f__Planococcaceae | g__Solibacillus |  |
| 02359367 | Bacteria | p__Actinobacteriota | c__Actinobacteria | o__Frankiales | f__uncultured | g__uncultured |  |
| 17636268 | Bacteria | p__Chloroflexi | c__Ktedonobacteria | o__Ktedonobacterales | f__Ktedonobacteraceae | g__JG30a-KF-32 | s__uncultured_bacterium |
| 04956098 | Bacteria | p__Chloroflexi | c__Chloroflexia | o__Thermomicrobiales | f__JG30-KF-CM45 | g__JG30-KF-CM45 | s__bacterium_QTYC46b |
| 08751168 | Bacteria | p__Proteobacteria | c__Gammaproteobacteria | o__Xanthomonadales | f__Rhodanobacteraceae |  |  |
| 0d7806019e74de8ae26a704f28a0eac3 | Bacteria | p__Gemmatimonadota | c__Gemmatimonadetes | o__Gemmatimonadales | f__Gemmatimonadaceae | g__uncultured |  |
| d87955d115e9450f6b3141b29d6dcdf1 | Bacteria | p__Actinobacteriota | c__Acidimicrobiia | o__Microtrichales | f__uncultured | g__uncultured | s__uncultured_Acidimicrobiia |
| 0c05f6ca5ef881c5fe65d7c49d19f52d | Bacteria | p__Proteobacteria | c__Gammaproteobacteria | o__Burkholderiales | f__Oxalobacteraceae | g__Noviherbaspirillum |  |
| d107084f7ee3fd9d3924b04ae4796047 | Bacteria |  |  |  |  |  |  |
| MG822857 | Bacteria | p__Proteobacteria | c__Alphaproteobacteria | o__Rhizobiales | f__Rhizobiaceae |  |  |
| RS-GCF-003602705.1-NZ-QTLK01000079.1 | Bacteria | p__Bacteroidota | c__Bacteroidia | o__Bacteroidales | f__Bacteroidaceae | g__Bacteroides |  |
| AB727354 | Bacteria | p__Proteobacteria | c__Alphaproteobacteria | o__Rhodobacterales | f__Rhodobacteraceae | g__Paracoccus |  |
| ac23c16714b4922a7ec7c1833807e34e | Bacteria | p__Actinobacteriota | c__Rubrobacteria | o__Rubrobacterales | f__Rubrobacteriaceae | g__Rubrobacter |  |
| 1edd8e2d68a36ede22e5cd1725fa611d | Bacteria | p__Proteobacteria | c__Gammaproteobacteria | o__Enterobacterales |  |  |  |
| ead73e666ad09deb5ed315868e398ab9 | Bacteria | p__Actinobacteriota | c__Actinobacteria | o__Corynebacteriales | f__Corynebacteriaceae | g__Corynebacterium |  |
| 1df654120f8d9fbae242e168c285434b | Bacteria | p__Chloroflexi | c__Chloroflexia | o__Chloroflexales | f__Roseiflexaceae | g__uncultured |  |
| d160b8f315a2a1d70e090e11f225c128 | Bacteria | p__Bacteroidota | c__Bacteroidia | o__Sphingobacteriales | f__Sphingobacteriaceae | g__Sphingobacterium |  |
| 6834bfdeb83380e799f7150de47f57cd | Bacteria | p__Actinobacteriota | c__Rubrobacteria | o__Rubrobacterales | f__Rubrobacteriaceae | g__Rubrobacter |  |
| 78f95a6afc73692ee00a305461352d4e | Bacteria | p__Proteobacteria | c__Gammaproteobacteria | o__Burkholderiales | f__Oxalobacteraceae | g__Massilia |  |
| d3c1c8be2eb139ac2abf97f5de1b1c2c | Bacteria | p__Actinobacteriota | c__Thermoleophilia | o__Solirubrobacterales | f__67-14 | g__67-14 |  |
| 66f416842705d355a3a7e2a577c0377b | Bacteria | p__Actinobacteriota | c__Actinobacteria |  |  |  |  |
| 51fc9de083893ad755d884bea6d69036 | Bacteria | p__Proteobacteria | c__Alphaproteobacteria | o__Sphingomonadales | f__Sphingomonadaceae | g__Altererythrobacter |  |
| RS-GCF-903931235.1-NZ-CAIZBQ010000027.1 | Bacteria | p__Proteobacteria | c__Gammaproteobacteria | o__Enterobacterales | f__Enterobacteriaceae |  |  |
| MJ034-1-barcode30-umi20969bins-ubs-9 | Bacteria | p__Acidobacteriota | c__Acidobacteriae | o__Acidobacteriales | f__uncultured | g__uncultured | s__uncultured_forest |
| a24b426306f76822d68ec4bfc67df200 | Bacteria | p__Bacteroidota | c__Bacteroidia | o__Flavobacteriales | f__Flavobacteriaceae | g__Flavobacterium |  |
| c032349de72c0da26bf36b0d56f88b79 | Bacteria | p__Bacteroidota | c__Bacteroidia | o__Chitinophagales | f__Chitinophagaceae | g__Flavihumibacter |  |
| 8e90d1b3bc9a54cb0b8540c5d9d097bc | Bacteria | p__Proteobacteria | c__Alphaproteobacteria | o__Rhizobiales | f__Rhizobiaceae |  |  |
| EF382666 | Bacteria | p__Proteobacteria | c__Alphaproteobacteria | o__Rhizobiales | f__Labraceae | g__Labrys |  |
| 11312684 | Bacteria | p__Bacteroidota | c__Bacteroidia | o__Bacteroidales | f__Rikenellaceae | g__Alistipes |  |
| 05098472 | Bacteria | p__Proteobacteria | c__Alphaproteobacteria | o__Sphingomonadales | f__Sphingomonadaceae | g__Sphingomonas |  |
| c024d33daac0f36842ef27e482711b96 | Bacteria | p__Actinobacteriota | c__Thermoleophilia | o__Solirubrobacterales | f__67-14 | g__67-14 |  |
| bd23eb4aa40784972457ecd5e74946be | Bacteria | p__Actinobacteriota | c__Thermoleophilia | o__Solirubrobacterales | f__Solirubrobacteraceae | g__Conexibacter | s__uncultured_bacterium |
| d25dd65da12e7b33ba090e34d1cde5a4 | Bacteria | p__Firmicutes | c__Bacilli | o__Lactobacillales | f__Enterococcaceae | g__Enterococcus |  |
| 3e99c1fd5a1d673f44fd7ef0c82d64aa | Bacteria |  |  |  |  |  |  |
| RS-GCF-005233705.1-NZ-QGAD01000046.1 | Bacteria | p__Firmicutes | c__Bacilli | o__Paenibacillales | f__Paenibacillaceae | g__Paenibacillus |  |
| MJ031-2-barcode69-umi38341bins-ubs-7 | Bacteria | p__Acidobacteriota | c__Acidobacteriae | o__Bryobacterales | f__Bryobacteraceae | g__Bryobacter |  |
| 04901404 | Bacteria | p__Proteobacteria | c__Gammaproteobacteria | o__Pseudomonadales | f__Moraxellaceae | g__Acinetobacter |  |
| b4b9738d9ea0bc038431571131b727c0 | Bacteria | p__Proteobacteria | c__Gammaproteobacteria | o__Enterobacterales | f__Enterobacteriaceae |  |  |
| 00924988 | Bacteria | p__Firmicutes | c__Clostridia | o__Peptostreptococcales-Tissierellales | f__Anaerovoracaceae | g__[Eubacterium]_nodatum_group |  |
| MJ034-1-barcode31-umi24596bins-ubs-5 | Bacteria | p__Proteobacteria | c__Gammaproteobacteria | o__Xanthomonadales | f__Rhodanobacteraceae | g__Rhodanobacter | s__uncultured_Xanthomonadaceae |
| 231087cda10fd6b4920e984479313287 | Bacteria | p__Proteobacteria | c__Gammaproteobacteria | o__Burkholderiales | f__Methylophilaceae | g__MM1 | s__uncultured_bacterium |
| 844219d7421a34ad391cc97b4b0f31d3 | Bacteria |  |  |  |  |  |  |
| f4586d611d639ac5a565e370d9ad685f | Bacteria | p__Proteobacteria | c__Alphaproteobacteria | o__Rhizobiales | f__Rhizobiaceae |  |  |
| b06a3a9b8ba50309c96b4674fc86ab1c | Bacteria | p__Nitrospirota | c__Nitrospiria | o__Nitrospirales | f__Nitrospiraceae | g__Nitrospira |  |
| 04960292 | Bacteria | p__Proteobacteria | c__Gammaproteobacteria | o__Burkholderiales | f__Nitrosomonadaceae | g__MND1 |  |
| 31a5d2d251844e637ed55eaf75d5c881 | Bacteria | p__Proteobacteria | c__Gammaproteobacteria | o__Vibrionales | f__Vibrionaceae | g__Vibrio |  |
| 9c8c1b3b256ce8d328f96428c072c72f | Bacteria | p__Proteobacteria | c__Alphaproteobacteria | o__Rhizobiales | f__Rhizobiaceae |  |  |
| bbbddce76592cdbd00546b72aa269f7b | Bacteria | p__Proteobacteria | c__Alphaproteobacteria |  |  |  |  |
| 33952b81456b291c9c3dc15afa46a823 | Bacteria | p__Firmicutes | c__Clostridia | o__Oscillospirales | f__Oscillospiraceae | g__uncultured |  |
| f61cc72a384eb87452717956f7e4dd77 | Bacteria | p__Firmicutes | c__Clostridia | o__Lachnospirales | f__Lachnospiraceae |  |  |
| 1bada7b2bec951d54b92f884d35e0681 | Bacteria | p__Proteobacteria | c__Alphaproteobacteria | o__Tistrellales | f__Geminicoccaceae | g__Candidatus_Alysiosphaera |  |
| fc68afe64fc977ed916b0b553e208a5c | Bacteria | p__Proteobacteria | c__Gammaproteobacteria | o__Competibacterales | f__Competibacteraceae | g__Candidatus_Competibacter |  |
| daeb35f83f3b9c7328667ee3e115277f | Bacteria | p__Proteobacteria | c__Gammaproteobacteria | o__Burkholderiales | f__Comamonadaceae |  |  |
| MJ010-2-barcode64-umi97818bins-ubs-5 | Bacteria | p__Bacteroidota | c__Bacteroidia | o__Bacteroidales | f__Bacteroidaceae | g__Bacteroides |  |
| 04989502 | Bacteria | p__Proteobacteria | c__Gammaproteobacteria | o__Pseudomonadales | f__Moraxellaceae | g__Enhydrobacter |  |
| 7c9452f496d9c43db5d2927c99cb9203 | Bacteria | p__Proteobacteria | c__Gammaproteobacteria | o__Burkholderiales | f__SC-I-84 | g__SC-I-84 |  |
| 04876092 | Bacteria | p__Proteobacteria | c__Alphaproteobacteria | o__Rhizobiales | f__Beijerinckiaceae | g__Methylobacterium-Methylorubrum |  |
| MJ030-2-barcode65-umi111489bins-ubs-3 | Bacteria | p__Proteobacteria | c__Alphaproteobacteria | o__Micropepsales | f__Micropepsaceae | g__uncultured |  |
| 05009768 | Bacteria | p__Proteobacteria | c__Alphaproteobacteria | o__Sphingomonadales | f__Sphingomonadaceae | g__Sphingomonas | s__Sphingomonas_oryziterrae |
| 72681cfbdef36696b2e7d1a4e1d3f8a4 | Bacteria | p__Proteobacteria | c__Gammaproteobacteria | o__Burkholderiales | f__Comamonadaceae | g__Tepidicella | s__uncultured_Tepidicella |
| 78025bb58e7fd0c1e669f2da9ef3bfbd | Bacteria | p__Firmicutes | c__Bacilli | o__Bacillales |  |  |  |
| 09091483 | Bacteria | p__Nitrospirota | c__Nitrospiria | o__Nitrospirales | f__Nitrospiraceae | g__Nitrospira | s__uncultured_Nitrospiraceae |
| 0bf952e4899edb1b8674ebf064f224ef | Bacteria | p__Gemmatimonadota | c__Gemmatimonadetes | o__Gemmatimonadales | f__Gemmatimonadaceae |  |  |
| 36eb9ef3b0153a46774302517467763e | Bacteria | p__Proteobacteria | c__Gammaproteobacteria | o__Xanthomonadales | f__Xanthomonadaceae |  |  |
| 04924221 | Bacteria | p__Bacteroidota | c__Bacteroidia | o__Bacteroidales | f__Porphyromonadaceae | g__Porphyromonas |  |
| 04939007 | Bacteria | p__Gemmatimonadota | c__Gemmatimonadetes | o__Gemmatimonadales | f__Gemmatimonadaceae | g__uncultured | s__uncultured_proteobacterium |
| 2c0b8dd804540be76cb5f3c99b395d81 | Bacteria | p__Proteobacteria | c__Gammaproteobacteria | o__Burkholderiales | f__SC-I-84 | g__SC-I-84 |  |
| RS-GCF-003075295.1-NZ-QDFP01000003.1 | Bacteria | p__Firmicutes | c__Bacilli | o__Bacillales | f__Bacillaceae | g__Bacillus |  |
| 11152517 | Bacteria | p__Bacteroidota | c__Bacteroidia | o__Bacteroidales | f__Rikenellaceae | g__Alistipes |  |
| 04106947 | Bacteria | p__Cyanobacteria | c__Cyanobacteriia |  |  |  |  |
| 49e6fbb818f727a9a33daa812dbcfa5a | Bacteria | p__Proteobacteria | c__Alphaproteobacteria | o__Rhizobiales | f__Xanthobacteraceae | g__Bradyrhizobium |  |
| e62325baa221cc31e5b5137699318829 | Bacteria | p__Acidobacteriota | c__Holophagae | o__Subgroup_7 | f__Subgroup_7 | g__Subgroup_7 |  |
| 11017974 | Bacteria | p__Bacteroidota | c__Bacteroidia | o__Bacteroidales | f__Muribaculaceae | g__Muribaculaceae |  |
| 02383928 | Bacteria | p__Actinobacteriota | c__Actinobacteria | o__Micrococcales | f__Microbacteriaceae | g__Microbacterium |  |
| 04934739 | Bacteria | p__Bacteroidota | c__Bacteroidia | o__Flavobacteriales | f__Weeksellaceae | g__Empedobacter |  |
| MJ030-1-barcode25-umi25731bins-ubs-19 | Bacteria | p__Proteobacteria | c__Gammaproteobacteria | o__Burkholderiales | f__Alcaligenaceae | g__Achromobacter |  |
| c2796ca97ed7536b8092760af246c5b4 | Bacteria | p__Acidobacteriota | c__Vicinamibacteria | o__Vicinamibacterales | f__uncultured | g__uncultured |  |
| 269cdd2063080f4a364d1f7dcec0abed | Bacteria | p__Proteobacteria | c__Gammaproteobacteria | o__Burkholderiales | f__Nitrosomonadaceae | g__IS-44 | s__metagenome |
| 153810ae9c1ef4f702a9d3e43090ba1e | Bacteria | p__Proteobacteria | c__Gammaproteobacteria | o__Pseudomonadales | f__Moraxellaceae | g__Enhydrobacter |  |
| 0a0bacd99565d9b2d7ba557951a8ab6d | Bacteria | p__Proteobacteria | c__Gammaproteobacteria | o__JG36-TzT-191 | f__JG36-TzT-191 | g__JG36-TzT-191 |  |
| 05284158 | Bacteria | p__Firmicutes | c__Clostridia | o__Oscillospirales | f__Oscillospiraceae |  |  |
| AB889541 | Bacteria | p__Actinobacteriota | c__Actinobacteria | o__Micromonosporales | f__Micromonosporaceae | g__Micromonospora |  |
| 099a75373fa0235629120ca69c048875 | Bacteria | p__Chloroflexi | c__Ktedonobacteria | o__Ktedonobacterales | f__Ktedonobacteraceae | g__Ktedonobacter | s__Ktedonobacterales_bacterium |
| 04966207 | Bacteria | p__Firmicutes | c__Bacilli | o__Bacillales | f__Bacillaceae | g__Bacillus |  |
| 7e30884edada600724a13ea44ae9da2b | Bacteria | p__Thermotogota | c__Thermotogae | o__Petrotogales | f__Petrotogaceae | g__Defluviitoga |  |
| c46bf331942ac5e7fdda16b02f92cc68 | Bacteria | p__Proteobacteria | c__Gammaproteobacteria | o__Burkholderiales | f__Burkholderiaceae | g__Limnobacter |  |
| RS-GCF-003752475.1-NZ-RKHP01000004.1 | Bacteria | p__Proteobacteria | c__Gammaproteobacteria | o__Enterobacterales | f__Enterobacteriaceae |  |  |
| d4d2762828a2b91199dd8d92302d84f8 | Bacteria | p__Bacteroidota | c__Bacteroidia | o__Bacteroidales | f__Prevotellaceae | g__Paraprevotella |  |
| GB-GCA-003482275.1-QUMI01000015.1 | Bacteria | p__Firmicutes | c__Clostridia | o__Lachnospirales | f__Lachnospiraceae |  |  |
| 86081b7d70d5b44fcdfbba7b1db68847 | Bacteria | p__Actinobacteriota | c__Actinobacteria | o__Corynebacteriales |  |  |  |
| c383929f25cb42b5bb7995d4dfcfe103 | Bacteria | p__Proteobacteria | c__Gammaproteobacteria | o__Burkholderiales | f__Burkholderiaceae | g__Burkholderia-Caballeronia-Paraburkholderia |  |
| MJ006-1-barcode39-umi116853bins-ubs-4 | Bacteria | p__Firmicutes | c__Clostridia | o__Lachnospirales | f__Lachnospiraceae | g__Coprococcus |  |
| adb496eceb5bf52c1d3bc7fd90b8a5be | Bacteria | p__Proteobacteria | c__Gammaproteobacteria | o__Enterobacterales | f__Morganellaceae | g__Arsenophonus |  |
| 6fd74b9a623f700059b86c81c2803c78 | Bacteria | p__Firmicutes | c__Clostridia | o__Lachnospirales | f__Lachnospiraceae |  |  |
| 34887254ec43c17517ca0e076346b0db | Bacteria | p__Proteobacteria | c__Gammaproteobacteria | o__Pseudomonadales | f__Pseudomonadaceae | g__Pseudomonas |  |
| df22e9bf4b7499e47a8bdc5cb7f0841f | Bacteria | p__Bacteroidota | c__Bacteroidia | o__Bacteroidales | f__Bacteroidaceae | g__Bacteroides |  |
| 17657002 | Bacteria | p__Actinobacteriota | c__Coriobacteriia | o__Coriobacteriales | f__Eggerthellaceae | g__Enterorhabdus | s__Adlercreutzia_mucosicola |
| ebe24d8ed8d74527a0aeefd540764ad7 | Bacteria | p__Proteobacteria | c__Gammaproteobacteria | o__Pseudomonadales | f__Pseudomonadaceae | g__Pseudomonas |  |
| RS-GCF-016405945.1-NZ-JAEFCC010000035.1 | Bacteria | p__Proteobacteria | c__Gammaproteobacteria | o__Burkholderiales | f__Comamonadaceae |  |  |
| 05002703 | Bacteria | p__Bacteroidota | c__Bacteroidia | o__Sphingobacteriales | f__Sphingobacteriaceae | g__Sphingobacterium |  |
| d9e7d937e35ba38edee497b2e2b64ec8 | Bacteria | p__Actinobacteriota | c__Actinobacteria |  |  |  |  |
| ad2c5d2b80e4cb099e7c5e15da87e014 | Bacteria | p__Proteobacteria | c__Gammaproteobacteria | o__Burkholderiales | f__SC-I-84 | g__SC-I-84 |  |
| 27fcb35a0d9f4997d3161ffad2d56d0c | Bacteria | p__Actinobacteriota | c__Thermoleophilia | o__Gaiellales | f__uncultured | g__uncultured |  |
| 1fd23976e3edffaff8910d40e4599c8b | Bacteria | p__Proteobacteria | c__Gammaproteobacteria | o__Legionellales | f__Legionellaceae | g__Legionella |  |
| fb72e7b13fa8ab3b8f362e63a59f16c5 | Bacteria | p__Bacteroidota | c__Bacteroidia | o__Cytophagales | f__Microscillaceae | g__uncultured |  |
| 2888dd073e7c5b66bb807e7ac6a031fb | Bacteria | p__Bacteroidota | c__Bacteroidia | o__Chitinophagales | f__Chitinophagaceae | g__Flavisolibacter | s__uncultured_Flavobacterium |
| 14422586 | Bacteria | p__Acidobacteriota | c__Vicinamibacteria | o__Vicinamibacterales | f__uncultured | g__uncultured |  |
| e0be2a397a874f3167c67939ea2b765f | Bacteria | p__Firmicutes | c__Bacilli | o__Lactobacillales | f__Enterococcaceae | g__Enterococcus |  |
| 8a71c0629408c98b81a8cdf27859aa17 | Bacteria | p__Chloroflexi | c__KD4-96 | o__KD4-96 | f__KD4-96 | g__KD4-96 | s__metagenome |
| ce927c8324dc286f10ec518c7f205a9f | Bacteria | p__Proteobacteria | c__Alphaproteobacteria | o__Acetobacterales | f__Acetobacteraceae | g__Acidisphaera | s__uncultured_Alphaproteobacteria |
| 91e7f9d0f0d298ad9caf97b1cd3a5f9a | Bacteria | p__Proteobacteria | c__Gammaproteobacteria | o__Burkholderiales | f__Comamonadaceae | g__Pelomonas |  |
| 02326654 | Bacteria | p__Actinobacteriota | c__Acidimicrobiia | o__Microtrichales | f__Ilumatobacteraceae | g__Ilumatobacter |  |
| 2b9769e59284fc00cc4693a6e0f6aecd | Bacteria | p__Proteobacteria | c__Alphaproteobacteria | o__Caulobacterales | f__Caulobacteraceae | g__Asticcacaulis | s__uncultured_bacterium |
| 02247858 | Bacteria | p__Proteobacteria | c__Alphaproteobacteria | o__Rhizobiales | f__Rhizobiaceae | g__Allorhizobium-Neorhizobium-Pararhizobium-Rhizobium | s__[Pseudomonas]_geniculata |
| 05283258 | Bacteria | p__Firmicutes | c__Bacilli | o__Erysipelotrichales | f__Erysipelotrichaceae | g__Ileibacterium | s__Ileibacterium_valens |
| 9d01a3823f27deb6dec909aa754b2049 | Bacteria | p__Actinobacteriota | c__Actinobacteria | o__Frankiales | f__uncultured | g__uncultured |  |
| ab7e8007d4097aa7161c1652a73f479f | Bacteria | p__WPS-2 | c__WPS-2 | o__WPS-2 | f__WPS-2 | g__WPS-2 | s__hydrothermal_vent |
| 8cbe8fae121e8db70361f253e14b9a98 | Bacteria | p__Firmicutes | c__Bacilli | o__Erysipelotrichales | f__Erysipelatoclostridiaceae | g__Erysipelatoclostridium | s__uncultured_Clostridium |
| MJ034-1-barcode40-umi23128bins-ubs-7 | Bacteria | p__Verrucomicrobiota | c__Verrucomicrobiae | o__Chthoniobacterales | f__Chthoniobacteraceae | g__Chthoniobacter |  |
| G000803625_1 | Bacteria | p__Patescibacteria | c__Saccharimonadia | o__Saccharimonadales | f__Saccharimonadaceae | g__TM7x |  |
| 8aa9a744e59818aafd408cb7b165247d | Bacteria | p__Actinobacteriota | c__Acidimicrobiia | o__Microtrichales | f__uncultured | g__uncultured |  |
| 3fd821e5d5efbe35c777a9d1843c77c8 | Bacteria | p__Bacteroidota | c__Bacteroidia | o__Sphingobacteriales | f__Sphingobacteriaceae | g__Sphingobacterium |  |
| 62d4cc908136a73a634ac7682376b672 | Bacteria | p__Proteobacteria | c__Gammaproteobacteria | o__Pseudomonadales | f__Moraxellaceae | g__Acinetobacter |  |
| 14295d7f9d75a08cb2d1fee78a5ec5ae | Bacteria | p__Firmicutes | c__Clostridia | o__Lachnospirales | f__Lachnospiraceae | g__Lachnospiraceae_ND3007_group |  |
| 123d52d696dd891eab9cfc4516cf5506 | Bacteria | p__Firmicutes | c__Clostridia | o__Oscillospirales | f__Ruminococcaceae | g__Faecalibacterium |  |
| 08033637 | Bacteria | p__Proteobacteria | c__Alphaproteobacteria | o__Dongiales | f__Dongiaceae | g__Dongia |  |
| 6afb6741733c5ad3c95f9bee655e2338 | Bacteria | p__Actinobacteriota | c__Actinobacteria | o__Micrococcales | f__Brevibacteriaceae | g__Brevibacterium |  |
| 21020019 | Bacteria | p__Firmicutes | c__Bacilli | o__Erysipelotrichales | f__Erysipelotrichaceae |  |  |
| 04310267 | Bacteria | p__WPS-2 | c__WPS-2 | o__WPS-2 | f__WPS-2 | g__WPS-2 | s__uncultured_bacterium |
| e2b56f61acf89838bb4e167b3adb5059 | Bacteria | p__Actinobacteriota | c__Actinobacteria | o__Micrococcales | f__Intrasporangiaceae |  |  |
| 34c5dd23941d455b29ab72a9857216d9 | Bacteria | p__Acidobacteriota | c__Acidobacteriae | o__Acidobacteriales |  |  |  |
| f5b8a633d72811ffe72f6854ba2dc6e1 | Bacteria | p__Actinobacteriota | c__Rubrobacteria | o__Rubrobacterales | f__Rubrobacteriaceae | g__Rubrobacter |  |
| 767f55d11ea41249d2d9dd4da692b098 | Bacteria | p__Proteobacteria | c__Gammaproteobacteria | o__Pseudomonadales | f__Moraxellaceae | g__Acinetobacter | s__Ambystoma_mexicanum |
| b59ee8f427de5f688b0a076687e08eca | Bacteria | p__Planctomycetota | c__Planctomycetes | o__Pirellulales | f__Pirellulaceae | g__Pirellula | s__uncultured_soil |
| GB-GCA-016705825.1-JADJKU010000009.1 | Bacteria | p__Proteobacteria | c__Gammaproteobacteria | o__Burkholderiales | f__SC-I-84 | g__SC-I-84 |  |
| a39e1ce5aa1cb0395378f9464b8131c7 | Bacteria | p__Bacteroidota | c__Bacteroidia | o__Flavobacteriales | f__Flavobacteriaceae | g__Flavobacterium |  |
| 5d65d8719de6c237e735da8b920901f4 | Bacteria | p__Acidobacteriota | c__Acidobacteriae |  |  |  |  |
| FN824365 | Bacteria | p__Actinobacteriota | c__Actinobacteria | o__Kineosporiales | f__Kineosporiaceae | g__Pseudokineococcus | s__Pseudokineococcus_lusitanus |
| 0dfbe4022550fcfc02b6c5ffbf6d9f19 | Bacteria | p__Proteobacteria | c__Gammaproteobacteria | o__Burkholderiales | f__Comamonadaceae |  |  |
| M83810 | Bacteria | p__Proteobacteria | c__Alphaproteobacteria | o__Caulobacterales | f__Caulobacteraceae | g__Brevundimonas |  |
| RS-GCF-000368145.1-NZ-KB849290.1 | Bacteria | p__Proteobacteria | c__Gammaproteobacteria | o__Pseudomonadales | f__Moraxellaceae | g__Acinetobacter |  |
| GB-GCA-016720935.1-JADKJP010000006.1 | Bacteria | p__Bacteroidota | c__Bacteroidia | o__Sphingobacteriales | f__AKYH767 | g__AKYH767 |  |
| 45ec667b1209fdd3613593043b95d564 | Bacteria | p__Actinobacteriota | c__Actinobacteria | o__Micrococcales | f__Microbacteriaceae | g__Microbacterium |  |
| 5dee288b596307314af3ee068b55aecf | Bacteria | p__Proteobacteria | c__Gammaproteobacteria | o__Burkholderiales | f__SC-I-84 | g__SC-I-84 |  |
| 68f319ac71325454ec115282df202a31 | Bacteria | p__Proteobacteria | c__Gammaproteobacteria | o__Burkholderiales | f__Comamonadaceae |  |  |
| 7e1db29ae8ab5d15e5885cebca07b15f | Bacteria | p__Proteobacteria | c__Gammaproteobacteria | o__Pseudomonadales | f__Moraxellaceae | g__Acinetobacter |  |
| 05931014 | Bacteria | p__Chloroflexi | c__Chloroflexia | o__Chloroflexales | f__Roseiflexaceae | g__uncultured | s__uncultured_Chloroflexi |
| RS-GCF-900110655.1-NZ-FOEQ01000033.1 | Bacteria | p__Proteobacteria | c__Gammaproteobacteria | o__Pseudomonadales | f__Pseudomonadaceae | g__Pseudomonas |  |
| GB-GCA-000980225.1-LARG01000024.1 | Bacteria | p__Bacteroidota | c__Bacteroidia | o__Bacteroidales | f__Rikenellaceae | g__Alistipes | s__Alistipes_sp. |
| KC550155 | Bacteria | p__Actinobacteriota | c__Actinobacteria | o__Micrococcales | f__Intrasporangiaceae | g__Ornithinimicrobium |  |
| MJ020-2-barcode52-umi47458bins-ubs-9 | Bacteria | p__Bacteroidota | c__Bacteroidia | o__Chitinophagales | f__Chitinophagaceae | g__Terrimonas |  |
| 85a50317edc4c22e6f5bc6554be3b3bd | Bacteria | p__Actinobacteriota | c__Thermoleophilia | o__Solirubrobacterales | f__67-14 | g__67-14 | s__Solirubrobacterales_bacterium |
| 7c656f18ae8a685d50e09365557adc82 | Bacteria | p__Bacteroidota | c__Bacteroidia | o__Bacteroidales | f__Dysgonomonadaceae | g__Proteiniphilum | s__uncultured_prokaryote |
| ff12fa22cdd7a6241abff7b3431bb8b1 | Bacteria | p__Proteobacteria | c__Gammaproteobacteria | o__Xanthomonadales | f__Xanthomonadaceae | g__Stenotrophomonas |  |
| 05122595 | Bacteria | p__Proteobacteria | c__Alphaproteobacteria | o__Rhizobiales | f__Rhizobiaceae |  |  |
| 57a92db4cd1504da826963757be527e5 | Bacteria | p__Proteobacteria | c__Gammaproteobacteria | o__Burkholderiales | f__Burkholderiaceae | g__Ralstonia |  |
| 04997123 | Bacteria | p__Nitrospirota | c__Nitrospiria | o__Nitrospirales | f__Nitrospiraceae | g__Nitrospira | s__Nitrospira_japonica |
| 1ec165f6bc06cfa38d5931fc0fdcc398 | Bacteria | p__Chloroflexi | c__Anaerolineae | o__Anaerolineales | f__Anaerolineaceae | g__Longilinea |  |
| 04993079 | Bacteria | p__Proteobacteria | c__Alphaproteobacteria | o__Rhizobiales | f__Xanthobacteraceae | g__uncultured |  |
| 11010445 | Bacteria | p__Bacteroidota | c__Bacteroidia | o__Bacteroidales | f__Muribaculaceae | g__Muribaculaceae | s__uncultured_bacterium |
| 6c1bb388075a7ce3d828e6daf9189c55 | Bacteria | p__Proteobacteria | c__Alphaproteobacteria | o__Rhizobiales | f__Hyphomicrobiaceae |  |  |
| 492fd883a6dcd72a4bde4ff36298d2ec | Bacteria | p__Actinobacteriota | c__Thermoleophilia | o__Gaiellales | f__uncultured | g__uncultured |  |
| MJ034-1-barcode41-umi11984bins-ubs-29 | Bacteria | p__Acidobacteriota | c__Blastocatellia | o__Blastocatellales | f__Blastocatellaceae | g__JGI_0001001-H03 |  |
| 6bb884caae54411f8a91c542a1d3cb8f | Bacteria | p__NB1-j | c__NB1-j | o__NB1-j | f__NB1-j | g__NB1-j |  |
| 97c45c0b0715347c2f08f4cbf836411f | Bacteria | p__Bacteroidota | c__Bacteroidia | o__Sphingobacteriales | f__Sphingobacteriaceae | g__Mucilaginibacter |  |
| 0aea7fdd5390022a7adcd1797264eadb | Bacteria | p__Proteobacteria | c__Gammaproteobacteria | o__Burkholderiales | f__Comamonadaceae | g__Ottowia | s__uncultured_bacterium |
| GQ329713 | Bacteria | p__Actinobacteriota | c__Actinobacteria | o__Micrococcales | f__Microbacteriaceae | g__Microbacterium |  |
| RS-GCF-003224295.1-NZ-QKLB01000004.1 | Bacteria | p__Actinobacteriota | c__Actinobacteria | o__Micrococcales | f__Microbacteriaceae |  |  |
| RS-GCF-002940755.1-NZ-POTD01000044.1 | Bacteria | p__Proteobacteria | c__Alphaproteobacteria | o__Azospirillales | f__Azospirillaceae | g__Azospirillum |  |
| 56f44ad6d94231d75ada81945dddc580 | Bacteria | p__Actinobacteriota | c__Acidimicrobiia | o__IMCC26256 | f__IMCC26256 | g__IMCC26256 |  |
| RS-GCF-018066525.1-NZ-JAGKLK010000035.1 | Bacteria | p__Proteobacteria | c__Gammaproteobacteria | o__Burkholderiales | f__Alcaligenaceae | g__Alcaligenes |  |
| 639f9cf31ea3e19140855c04cb067ee1 | Bacteria | p__Chloroflexi | c__Chloroflexia | o__Chloroflexales | f__Chloroflexaceae | g__FFCH7168 | s__uncultured_bacterium |
| cfaf9b5f24c4e93b0d47667d075d8bf7 | Bacteria | p__Proteobacteria | c__Gammaproteobacteria | o__Xanthomonadales | f__Xanthomonadaceae | g__Stenotrophomonas |  |
| RS-GCF-000322425.1-NZ-KB030110.1 | Bacteria | p__Firmicutes | c__Bacilli | o__Lactobacillales | f__Enterococcaceae | g__Enterococcus |  |
| G000425825_1 | Bacteria | p__Firmicutes | c__Bacilli | o__Staphylococcales | f__Staphylococcaceae | g__Jeotgalicoccus |  |
| KM874399 | Bacteria | p__Actinobacteriota | c__Actinobacteria | o__Micrococcales | f__Micrococcaceae | g__Kocuria |  |
| MJ009-1-barcode47-umi78531bins-ubs-4 | Bacteria | p__Proteobacteria | c__Gammaproteobacteria | o__Burkholderiales | f__Comamonadaceae | g__Comamonas |  |
| EF466107 | Bacteria | p__Actinobacteriota | c__Actinobacteria | o__Propionibacteriales | f__Nocardioidaceae | g__Nocardioides |  |
| RS-GCF-001006635.1-NZ-JSWZ01000034.1 | Bacteria | p__Proteobacteria | c__Gammaproteobacteria | o__Enterobacterales | f__Enterobacteriaceae |  |  |
| 04898673 | Bacteria | p__Armatimonadota | c__Fimbriimonadia | o__Fimbriimonadales | f__Fimbriimonadaceae | g__Fimbriimonadaceae | s__metagenome |
| G003481825_1 | Bacteria | p__Firmicutes | c__Clostridia | o__Lachnospirales | f__Lachnospiraceae |  |  |
| abbb3aa878ca82f0dbf284d6cc206da3 | Bacteria | p__Acidobacteriota | c__Acidobacteriae | o__Bryobacterales | f__Bryobacteraceae | g__Bryobacter |  |
| e80f93c0cf8150d2b78e95956549db12 | Bacteria | p__Firmicutes | c__Bacilli | o__Paenibacillales | f__Paenibacillaceae | g__Paenibacillus |  |
| c43a89bc5b3ca02ad146a2469b1c8892 | Bacteria | p__Actinobacteriota | c__Actinobacteria | o__Frankiales | f__Acidothermaceae | g__Acidothermus |  |
| 04918965 | Bacteria | p__Bacteroidota | c__Bacteroidia | o__Sphingobacteriales | f__Sphingobacteriaceae | g__Sphingobacterium | s__Triticum_urartu |
| 265486d83ad2791fbe3da1563fdc1a20 | Bacteria | p__Firmicutes | c__Bacilli | o__Lactobacillales | f__Streptococcaceae | g__Streptococcus |  |
| acae578567244c000c8753a08cda543c | Bacteria | p__Sumerlaeota | c__Sumerlaeia | o__uncultured | f__uncultured | g__uncultured | s__uncultured_organism |
| 05038374 | Bacteria | p__Firmicutes | c__Bacilli | o__Lactobacillales | f__Lactobacillaceae | g__Lactobacillus | s__Lactobacillus_intestinalis |
| c846e9c90d7d736be1a66efb0af4c4de | Bacteria | p__Chloroflexi | c__Ktedonobacteria | o__Ktedonobacterales | f__Ktedonobacteraceae |  |  |
| RS-GCF-014062185.1-NZ-JACGCY010000001.1 | Bacteria | p__Proteobacteria | c__Gammaproteobacteria | o__Pseudomonadales | f__Pseudomonadaceae | g__Pseudomonas |  |
| 04900156 | Bacteria | p__Proteobacteria | c__Gammaproteobacteria | o__Alteromonadales | f__Alteromonadaceae | g__Rheinheimera |  |
| 3ea8c1a73f967b86d332247f9d757f79 | Bacteria | p__Proteobacteria | c__Alphaproteobacteria | o__Caulobacterales | f__Hyphomonadaceae | g__SWB02 |  |
| 02314941 | Bacteria | p__Acidobacteriota | c__Vicinamibacteria | o__Vicinamibacterales | f__Vicinamibacteraceae | g__Vicinamibacteraceae |  |
| G902363025_1 | Bacteria | p__Firmicutes | c__Clostridia | o__Lachnospirales | f__Lachnospiraceae |  |  |
| RS-GCF-001046555.1-NZ-LAMP01000039.1 | Bacteria | p__Firmicutes | c__Bacilli | o__Staphylococcales | f__Staphylococcaceae | g__Staphylococcus |  |
| AB178429 | Bacteria | p__Proteobacteria | c__Alphaproteobacteria | o__Acetobacterales | f__Acetobacteraceae | g__Gluconobacter |  |
| 2cdcf44e65dbe5b96199767bc248bfc1 | Bacteria | p__Firmicutes | c__Bacilli | o__Lactobacillales | f__Enterococcaceae | g__Enterococcus |  |
| 2ad3df2171385edbf27a35102f5b6b30 | Bacteria | p__Proteobacteria | c__Gammaproteobacteria | o__Enterobacterales | f__Enterobacteriaceae |  |  |
| 04933372 | Bacteria | p__Bacteroidota | c__Bacteroidia | o__Chitinophagales | f__Chitinophagaceae | g__uncultured | s__uncultured_microorganism |
| 08f3a3ad13ab0ed847989618ef193c5c | Bacteria | p__Firmicutes | c__Clostridia | o__Clostridiales | f__Clostridiaceae | g__Proteiniclasticum |  |
| MJ006-2-barcode60-umi164883bins-ubs-3 | Bacteria | p__Firmicutes | c__Clostridia | o__Oscillospirales | f__Oscillospiraceae | g__UCG-003 |  |
| 88f833c7e9190e0c9adb9f80b5c55dad | Bacteria | p__Proteobacteria | c__Alphaproteobacteria | o__Sphingomonadales | f__Sphingomonadaceae | g__Sphingomonas |  |
| RS-GCF-009729075.1-NZ-VTGY01000024.1 | Bacteria | p__Actinobacteriota | c__Actinobacteria | o__Corynebacteriales | f__Mycobacteriaceae | g__Mycobacterium |  |
| RS-GCF-017797885.1-NZ-CP072547.1 | Bacteria | p__Proteobacteria | c__Alphaproteobacteria | o__Rhizobiales | f__Rhizobiaceae |  |  |
| 06155109 | Bacteria | p__Proteobacteria | c__Gammaproteobacteria | o__Gammaproteobacteria_Incertae_Sedis | f__Unknown_Family | g__Acidibacter |  |
| 6528ad3d7714f8d9b299d9a96b0e792f | Bacteria | p__Acidobacteriota | c__Acidobacteriae | o__Acidobacteriales | f__uncultured | g__uncultured |  |
| 07ee25423a34359f7e50e21783056012 | Bacteria | p__Cyanobacteria | c__Cyanobacteriia | o__Leptolyngbyales | f__Leptolyngbyaceae | g__Leptolyngbya_Es-Yyy1000 | s__Leptolyngbya_cf. |
| b0c7c04027c8e44ddc5b4f5b34e0f9c5 | Bacteria | p__Thermotogota | c__Thermotogae | o__Thermotogales | f__Fervidobacteriaceae | g__Fervidobacterium | s__Fervidobacterium_nodosum |
| 04480ff8712d13c82603fe78c6a2b825 | Bacteria | p__Gemmatimonadota | c__Gemmatimonadetes | o__Gemmatimonadales | f__Gemmatimonadaceae | g__uncultured | s__uncultured_Pseudomonadales |
| MJ006-1-barcode40-umi104736bins-ubs-4 | Bacteria | p__Firmicutes | c__Clostridia | o__Oscillospirales | f__Oscillospiraceae | g__UCG-005 |  |
| f89317aeb232a0fa1a1d51665c9ef910 | Bacteria | p__Proteobacteria | c__Gammaproteobacteria | o__Burkholderiales | f__Comamonadaceae |  |  |
| 064ec4a719db96b3401e7a8194bd192b | Bacteria | p__Actinobacteriota | c__Actinobacteria | o__Propionibacteriales | f__Nocardioidaceae | g__Nocardioides |  |
| RS-GCF-014851915.1-NZ-JACVAP010000011.1 | Bacteria | p__Proteobacteria | c__Gammaproteobacteria | o__Xanthomonadales | f__Xanthomonadaceae |  |  |
| ab359a39500e866f11a116aa65a4790c | Bacteria | p__Firmicutes | c__Clostridia | o__Lachnospirales | f__Lachnospiraceae |  |  |
| 05038552 | Bacteria | p__Firmicutes | c__Bacilli | o__Lactobacillales | f__Lactobacillaceae | g__Lactobacillus |  |
| RS-GCF-013166595.1-NZ-JABKKG010000004.1 | Bacteria | p__Bacteroidota | c__Bacteroidia | o__Bacteroidales | f__Prevotellaceae |  |  |
| e12b159a16deae4e7f25e8a1e471b60e | Bacteria | p__Actinobacteriota | c__Actinobacteria | o__Micrococcales | f__Microbacteriaceae |  |  |
| RS-GCF-000716785.1-NZ-JNYY01000048.1 | Bacteria | p__Actinobacteriota | c__Actinobacteria | o__Pseudonocardiales | f__Pseudonocardiaceae | g__Amycolatopsis |  |
| 8d1a1287253565eb0f1fc00545133c34 | Bacteria | p__Proteobacteria | c__Gammaproteobacteria | o__Burkholderiales | f__Comamonadaceae |  |  |
| 8bc33d52dcd489ec49b9edcb6c74bcd5 | Bacteria | p__Actinobacteriota | c__Actinobacteria | o__Micrococcales | f__Microbacteriaceae | g__Microbacterium |  |
| 17337586 | Bacteria | p__Firmicutes | c__Bacilli | o__Paenibacillales | f__Paenibacillaceae | g__Paenibacillus |  |
| f935481ecb5431c81fc9e88c53265644 | Bacteria | p__Proteobacteria | c__Gammaproteobacteria | o__Burkholderiales | f__Nitrosomonadaceae | g__Ellin6067 |  |
| 0c45c427f401d1be45ef81012717cca8 | Bacteria | p__Proteobacteria | c__Gammaproteobacteria | o__Cellvibrionales | f__Spongiibacteraceae | g__BD1-7_clade | s__metagenome |
| 8570ca7ae4344aea37157f48976ea6be | Bacteria | p__Firmicutes | c__Bacilli | o__Staphylococcales | f__Staphylococcaceae | g__Staphylococcus |  |
| RS-GCF-000581195.1-NZ-JEWW01000042.1 | Bacteria | p__Proteobacteria | c__Gammaproteobacteria | o__Pseudomonadales | f__Moraxellaceae | g__Acinetobacter |  |
| baf37f26c39edd60b8caf2531f9f651f | Bacteria | p__Latescibacterota | c__Latescibacterota | o__Latescibacterota | f__Latescibacterota | g__Latescibacterota | s__uncultured_Desulfuromonadales |
| 8d37b8e788e94863984052562e901ae4 | Bacteria | p__Actinobacteriota | c__Actinobacteria | o__Propionibacteriales | f__Nocardioidaceae | g__Nocardioides |  |
| 2e0bcb322372ef6f360ddc453da353ce | Bacteria | p__Proteobacteria | c__Gammaproteobacteria | o__Burkholderiales | f__MWH-UniP1_aquatic_group | g__MWH-UniP1_aquatic_group |  |
| 02412064 | Bacteria | p__Proteobacteria | c__Alphaproteobacteria | o__Rhizobiales | f__Beijerinckiaceae | g__Methylobacterium-Methylorubrum |  |
| b26ce5386791c7a7d57b08e53099be33 | Bacteria | p__Actinobacteriota | c__Acidimicrobiia | o__Microtrichales | f__Microtrichaceae | g__IMCC26207 |  |
| 04947928 | Bacteria | p__Firmicutes | c__Clostridia | o__Lachnospirales | f__Lachnospiraceae | g__Lachnospiraceae_NK4A136_group |  |
| e1c4c628e7de3042bd9637d993fea6c9 | Bacteria | p__Gemmatimonadota | c__Gemmatimonadetes | o__Gemmatimonadales | f__Gemmatimonadaceae | g__Gemmatimonas |  |
| AB682297 | Bacteria | p__Proteobacteria | c__Gammaproteobacteria | o__Burkholderiales | f__Burkholderiaceae | g__Burkholderia-Caballeronia-Paraburkholderia |  |
| 7b8cff6a8af88a33fba58ac4a422adfa | Bacteria | p__Proteobacteria | c__Gammaproteobacteria | o__Burkholderiales | f__Comamonadaceae | g__Lampropedia | s__Lampropedia_puyangensis |
| 16ca50de033cc62f98f641721249e0fc | Bacteria | p__Actinobacteriota | c__Thermoleophilia | o__Gaiellales | f__uncultured | g__uncultured |  |
| 46c00c3502071656fcdd5722e4efa4cc | Bacteria | p__Chloroflexi | c__Chloroflexia | o__Thermomicrobiales | f__JG30-KF-CM45 | g__JG30-KF-CM45 | s__uncultured_Chloroflexi |
| ad79efa636fcc1daeb6ddb8fd8e6467b | Bacteria | p__Proteobacteria | c__Gammaproteobacteria | o__Enterobacterales | f__Morganellaceae | g__Providencia |  |
| EF424403 | Bacteria | p__Proteobacteria | c__Alphaproteobacteria | o__Sphingomonadales | f__Sphingomonadaceae | g__Novosphingobium |  |
| AB469788 | Bacteria | p__Proteobacteria | c__Gammaproteobacteria | o__Burkholderiales | f__Oxalobacteraceae |  |  |
| RS-GCF-018069605.1-NZ-JAGPUP010000380.1 | Bacteria | p__Proteobacteria | c__Alphaproteobacteria | o__Rhizobiales | f__Beijerinckiaceae | g__Microvirga |  |
| f7a38935cab8aae32d80e3268b813775 | Bacteria | p__Proteobacteria | c__Alphaproteobacteria | o__Rhizobiales | f__Rhizobiaceae |  |  |
| MJ010-2-barcode64-umi68608bins-ubs-7 | Bacteria | p__Bacteroidota | c__Bacteroidia | o__Flavobacteriales | f__Flavobacteriaceae | g__Myroides | s__Myroides_phaeus |
| d7fee23964a88d566511e568328a67f6 | Bacteria | p__Firmicutes | c__Clostridia | o__Lachnospirales | f__Lachnospiraceae |  |  |
| 5ea1f600288d63256efd6cefe834b62c | Bacteria | p__Bacteroidota | c__Bacteroidia | o__Bacteroidales | f__Rikenellaceae | g__Alistipes | s__uncultured_bacterium |
| JF824799 | Bacteria | p__Bacteroidota | c__Bacteroidia | o__Bacteroidales | f__Rikenellaceae | g__Alistipes |  |
| 75bfa67f0f6129869edea819bdc22814 | Bacteria | p__Proteobacteria | c__Gammaproteobacteria | o__Burkholderiales | f__Comamonadaceae |  |  |
| e103ab97f40b30c7f17f76bfe62edb1c | Bacteria | p__Actinobacteriota | c__Actinobacteria | o__Frankiales | f__Acidothermaceae | g__Acidothermus |  |
| 68ff3a54a6462afe4df8af1ac0640959 | Bacteria | p__Proteobacteria | c__Gammaproteobacteria | o__Enterobacterales |  |  |  |
| 74e64deb016eecfe03496ab43fd2a77a | Bacteria | p__Proteobacteria | c__Gammaproteobacteria | o__Pseudomonadales | f__Moraxellaceae | g__Acinetobacter |  |
| 00003372cffeb51d91565e1ca7c5e048 | Bacteria | p__Firmicutes | c__Bacilli |  |  |  |  |
| 409e5165eceafe3d497d0d79361de5a2 | Bacteria | p__Actinobacteriota | c__Actinobacteria | o__Propionibacteriales | f__Nocardioidaceae | g__Marmoricola |  |
| 04899795 | Bacteria | p__Acidobacteriota | c__Vicinamibacteria | o__Vicinamibacterales | f__Vicinamibacteraceae | g__Vicinamibacteraceae |  |
| ad5bb22951c3ca05e471233698ffa09a | Bacteria | p__Myxococcota | c__Polyangia | o__Polyangiales | f__Polyangiaceae | g__Pajaroellobacter |  |
| 14800514 | Bacteria | p__Chloroflexi | c__Ktedonobacteria | o__Ktedonobacterales | f__JG30-KF-AS9 | g__JG30-KF-AS9 | s__uncultured_bacterium |
| 1400fefd17b79b87953df2174e7fb7d1 | Bacteria | p__Actinobacteriota | c__Thermoleophilia | o__Gaiellales | f__Gaiellaceae | g__Gaiella |  |
| MJ010-1-barcode26-umi85141bins-ubs-8 | Bacteria | p__Acidobacteriota | c__Acidobacteriae | o__Solibacterales | f__Solibacteraceae | g__Candidatus_Solibacter |  |
| 5b7201300c094c9dd3c4fe0d73326d85 | Bacteria | p__Actinobacteriota | c__Actinobacteria | o__Propionibacteriales | f__Propionibacteriaceae | g__Microlunatus |  |
| 7c26ada723e6c3054d5a9a4f6481ef68 | Bacteria | p__Acidobacteriota | c__Blastocatellia | o__Blastocatellales | f__Blastocatellaceae | g__Stenotrophobacter |  |
| d1aaf083fb2343deccd95ae4a1f2fe00 | Bacteria | p__Proteobacteria | c__Gammaproteobacteria | o__Burkholderiales | f__Alcaligenaceae | g__Achromobacter |  |
| 2d007761dd9266367c11c019958c51a5 | Bacteria | p__Proteobacteria | c__Gammaproteobacteria | o__Burkholderiales | f__SC-I-84 | g__SC-I-84 |  |
| MJ034-1-barcode32-umi32070bins-ubs-3 | Bacteria | p__Proteobacteria | c__Alphaproteobacteria | o__Caulobacterales | f__Hyphomonadaceae | g__Hirschia |  |
| MJ034-2-barcode51-umi2438bins-ubs-173 | Bacteria | p__Bacteroidota | c__Bacteroidia | o__Chitinophagales | f__Chitinophagaceae | g__Segetibacter |  |
| 406efa1a304aeb4308011da8fbeb78fa | Bacteria | p__Bacteroidota | c__Bacteroidia | o__Bacteroidales | f__Prevotellaceae | g__Prevotella |  |
| 7efb0dc7ee8f73ba4f194a8f7160ec50 | Bacteria | p__Proteobacteria | c__Gammaproteobacteria | o__Pasteurellales | f__Pasteurellaceae | g__Haemophilus |  |
| 11378058 | Bacteria | p__Armatimonadota | c__Chthonomonadetes | o__Chthonomonadales | f__Chthonomonadales | g__Chthonomonadales | s__uncultured_bacterium |
| c18f3375f8a1a16bd4689f3477a5fa20 | Bacteria | p__Actinobacteriota | c__Actinobacteria | o__Micrococcales | f__Microbacteriaceae | g__Microbacterium |  |
| 34bf6169999da51a11901227bc81b516 | Bacteria | p__Bacteroidota | c__Bacteroidia | o__Sphingobacteriales | f__Sphingobacteriaceae | g__Sphingobacterium |  |
| MJ010-1-barcode29-umi149200bins-ubs-3 | Bacteria | p__Proteobacteria | c__Gammaproteobacteria | o__Burkholderiales | f__Rhodocyclaceae | g__uncultured |  |
| e6fcddb4937c0a6158ca3d524e24ec30 | Bacteria | p__Acidobacteriota | c__Holophagae | o__Subgroup_7 | f__Subgroup_7 | g__Subgroup_7 |  |
| G000187895_3 | Bacteria | p__Bacteroidota | c__Bacteroidia | o__Bacteroidales | f__Bacteroidaceae | g__Bacteroides | s__Bacteroides_plebeius |
| c262df130a049dc34a3d1584a44c4915 | Bacteria | p__Bacteroidota | c__Bacteroidia | o__Sphingobacteriales | f__Sphingobacteriaceae | g__Sphingobacterium |  |
| 04924458 | Bacteria | p__Bacteroidota | c__Bacteroidia | o__Bacteroidales | f__Muribaculaceae | g__Muribaculaceae |  |
| MJ034-1-barcode40-umi19869bins-ubs-10 | Bacteria | p__Acidobacteriota | c__Acidobacteriae | o__Solibacterales | f__Solibacteraceae | g__Candidatus_Solibacter |  |
| f2d7de3425403d59ab6b6bddaf39138d | Bacteria | p__Myxococcota | c__Polyangia | o__Polyangiales | f__Polyangiaceae | g__Pajaroellobacter |  |
| 19126b8d3895505b18011a2bed61afef | Bacteria | p__Proteobacteria | c__Alphaproteobacteria | o__Rhizobiales | f__Xanthobacteraceae | g__uncultured |  |
| e4da8d563e36dfd0e7eb99597ec681b0 | Bacteria | p__Chloroflexi | c__Ktedonobacteria | o__B12-WMSP1 | f__B12-WMSP1 | g__B12-WMSP1 |  |
| 9dcf228bff2aac6eb0eeaefe278104d6 | Bacteria | p__Chloroflexi | c__Ktedonobacteria | o__Ktedonobacterales | f__Ktedonobacteraceae | g__Ktedonobacter | s__Ktedonobacterales_bacterium |
| d559bbc347fe871465c1c9ae8d41c55a | Bacteria | p__Proteobacteria | c__Alphaproteobacteria | o__Acetobacterales | f__Acetobacteraceae | g__uncultured |  |
| GB-GCA-900066055.1-FMEF01000035.1 | Bacteria | p__Firmicutes | c__Clostridia | o__Lachnospirales | f__Lachnospiraceae |  |  |
| MJ010-1-barcode26-umi139173bins-ubs-4 | Bacteria | p__Acidobacteriota | c__Acidobacteriae | o__Acidobacteriales | f__uncultured | g__uncultured |  |
| 5ef5b029ff0ffb4812cd8f24c3195c90 | Bacteria | p__Bacteroidota | c__Bacteroidia | o__Bacteroidales | f__Bacteroidaceae | g__Bacteroides |  |
| b70488f7d7543370eb66f1488f681977 | Bacteria | p__Proteobacteria | c__Gammaproteobacteria | o__Burkholderiales |  |  |  |
| 18446594 | Bacteria | p__Proteobacteria | c__Gammaproteobacteria | o__Burkholderiales | f__Alcaligenaceae |  |  |
| 04919003 | Bacteria | p__Bacteroidota | c__Bacteroidia | o__Sphingobacteriales | f__Sphingobacteriaceae | g__Sphingobacterium |  |
| f05ca9f4d29fc13d6d9a10cc4d578ec8 | Bacteria | p__Bacteroidota | c__Bacteroidia | o__Sphingobacteriales | f__Sphingobacteriaceae | g__Sphingobacterium | s__Sphingobacterium_nematocida |
| 8135efd8ac1737505a5219ca33952376 | Bacteria | p__Proteobacteria | c__Gammaproteobacteria | o__Burkholderiales | f__Comamonadaceae |  |  |
| 765867e97e1472fd0455b8389dafcd6a | Bacteria | p__Proteobacteria | c__Gammaproteobacteria | o__Enterobacterales | f__Enterobacteriaceae | g__Escherichia-Shigella |  |
| 285f1baa2d0da733d25390eb801d9fc5 | Bacteria |  |  |  |  |  |  |
| FJ214364 | Bacteria | p__Actinobacteriota | c__Actinobacteria | o__Pseudonocardiales | f__Pseudonocardiaceae | g__Saccharopolyspora |  |
| 70e10b5fa1b993304eb4b8b8815e24b6 | Bacteria | p__Chloroflexi | c__TK10 | o__TK10 | f__TK10 | g__TK10 |  |
| 25e7ff2084e0ec90d1be1863459c0ff9 | Bacteria | p__Actinobacteriota | c__Actinobacteria | o__Micrococcales | f__Intrasporangiaceae | g__Terrabacter |  |
| 05130584 | Bacteria | p__Actinobacteriota | c__Actinobacteria | o__Micrococcales | f__Microbacteriaceae |  |  |
| a6f5e7c04facdc3cb13139a60c20ea0f | Bacteria | p__Actinobacteriota | c__Acidimicrobiia | o__Microtrichales |  |  |  |
| 04909196 | Bacteria | p__Proteobacteria | c__Alphaproteobacteria | o__Rhizobiales | f__Rhizobiaceae | g__Shinella |  |
| 04924010 | Bacteria | p__Bacteroidota | c__Bacteroidia | o__Bacteroidales | f__Muribaculaceae | g__Muribaculaceae | s__uncultured_bacterium |
| 20208280 | Bacteria | p__Firmicutes | c__Clostridia | o__Oscillospirales | f__Ruminococcaceae | g__Ruminococcaceae | s__uncultured_bacterium |
| 10693065 | Bacteria | p__Cyanobacteria | c__Cyanobacteriia | o__Leptolyngbyales | f__Leptolyngbyaceae | g__Phormidesmis_ANT.L52.6 |  |
| d57e1fc9f986f19836f9497368c078b3 | Bacteria | p__Proteobacteria | c__Alphaproteobacteria | o__Sphingomonadales | f__Sphingomonadaceae | g__Novosphingobium |  |
| G008933705_1 | Bacteria | p__Proteobacteria | c__Alphaproteobacteria | o__Rhizobiales | f__Hyphomicrobiaceae | g__Pedomicrobium |  |
| b37c50d156f28dc758787b1194871ee4 | Bacteria | p__Actinobacteriota | c__Thermoleophilia | o__Gaiellales | f__uncultured | g__uncultured |  |
| MJ034-1-barcode48-umi2660bins-ubs-163 | Bacteria | p__Acidobacteriota | c__Blastocatellia | o__Blastocatellales | f__Blastocatellaceae |  |  |
| AM412238 | Bacteria | p__Proteobacteria | c__Alphaproteobacteria | o__Sphingomonadales | f__Sphingomonadaceae | g__Sphingomonas |  |
| 1d510d7991a7cf0d1c095a7546d4302a | Bacteria | p__Actinobacteriota | c__Acidimicrobiia | o__IMCC26256 | f__IMCC26256 | g__IMCC26256 |  |
| e455bd74a44661935f92dee6f3dcce79 | Bacteria | p__Firmicutes | c__Clostridia | o__Clostridia | f__Hungateiclostridiaceae |  |  |
| 04952704 | Bacteria | p__Actinobacteriota | c__Thermoleophilia | o__Gaiellales |  |  |  |
| MJ031-1-barcode27-umi27930bins-ubs-12 | Bacteria | p__Bacteroidota | c__Bacteroidia | o__Chitinophagales | f__Chitinophagaceae | g__Terrimonas |  |
| G003472385_1 | Bacteria | p__Proteobacteria | c__Gammaproteobacteria | o__Burkholderiales | f__Sutterellaceae | g__Sutterella |  |
| 04958361 | Bacteria | p__Actinobacteriota | c__Actinobacteria | o__Frankiales | f__Frankiaceae | g__Jatrophihabitans |  |
| 19707119 | Bacteria | p__Firmicutes | c__Bacilli | o__Erysipelotrichales | f__Erysipelotrichaceae | g__Ileibacterium | s__Ileibacterium_valens |
| 11151747 | Bacteria | p__Bacteroidota | c__Bacteroidia | o__Bacteroidales | f__Marinifilaceae | g__Odoribacter | s__uncultured_bacterium |
| G000504165_1 | Bacteria | p__Firmicutes | c__Bacilli | o__Bacillales | f__Bacillaceae | g__Bacillus |  |
| 9337cd1f3b9a3cb47581d70ce908f416 | Bacteria | p__Bacteroidota | c__Bacteroidia | o__Chitinophagales | f__Chitinophagaceae | g__Terrimonas |  |
| d2249671a2d627418f2c86b6345c491a | Bacteria | p__Proteobacteria | c__Gammaproteobacteria | o__Burkholderiales | f__Nitrosomonadaceae | g__Ellin6067 |  |
| b2544e656e23d50f949cb791b067e177 | Bacteria | p__Proteobacteria | c__Alphaproteobacteria | o__Rhizobiales | f__Hyphomicrobiaceae |  |  |
| bd456d996b7158def7d3d905dc9989be | Bacteria | p__Firmicutes | c__Clostridia | o__Lachnospirales | f__Lachnospiraceae |  |  |
| e07a739f72b1fb13d3a02e5d207c2179 | Bacteria | p__Firmicutes | c__Bacilli | o__Lactobacillales | f__Enterococcaceae | g__Enterococcus |  |
| 3256f3d493039b2c2613dca264722848 | Bacteria | p__Bacteroidota | c__Bacteroidia | o__Chitinophagales | f__Saprospiraceae | g__uncultured |  |
| 879dc976b29d78979eb0b7c9e2add31d | Bacteria | p__Actinobacteriota | c__Acidimicrobiia | o__Microtrichales | f__Microtrichaceae | g__uncultured | s__metagenome |
| MF980971 | Bacteria | p__Proteobacteria | c__Alphaproteobacteria | o__Rhizobiales | f__Beijerinckiaceae | g__Methylobacterium-Methylorubrum |  |
| AB184219 | Bacteria | p__Actinobacteriota | c__Actinobacteria | o__Streptomycetales | f__Streptomycetaceae | g__Streptomyces |  |
| 3758ce5821c5f4cbe1a0d1a659321854 | Bacteria | p__WPS-2 | c__WPS-2 | o__WPS-2 | f__WPS-2 | g__WPS-2 | s__Burkholderiales_bacterium |
| 11d4e3bf60e1b12e4161a4ebc4ce0536 | Bacteria | p__Entotheonellaeota | c__Entotheonellia | o__Entotheonellales | f__Entotheonellaceae | g__Entotheonellaceae | s__uncultured_bacterium |
| 7f8d2fbb5e57d90b04f00fb2bccd97b6 | Bacteria | p__Firmicutes | c__Clostridia | o__Lachnospirales | f__Lachnospiraceae |  |  |
| 7008e6e549aaa2faa12b0f3f50000d56 | Bacteria | p__Bacteroidota | c__Bacteroidia | o__Sphingobacteriales | f__Sphingobacteriaceae | g__Pedobacter |  |
| 05918371 | Bacteria | p__Acidobacteriota | c__Holophagae | o__Subgroup_7 | f__Subgroup_7 | g__Subgroup_7 |  |
| 18687350 | Bacteria | p__Actinobacteriota | c__Actinobacteria | o__Frankiales | f__Geodermatophilaceae |  |  |
| GB-GCA-016794205.1-JAEUIJ010000002.1 | Bacteria | p__Planctomycetota | c__Planctomycetes | o__Pirellulales | f__Pirellulaceae | g__Pir4_lineage | s__uncultured_Planctomycetaceae |
| 14872294 | Bacteria | p__Chloroflexi | c__Ktedonobacteria | o__Ktedonobacterales | f__JG30-KF-AS9 | g__JG30-KF-AS9 | s__uncultured_bacterium |
| e0a298bf6e4152eb5f06f98dd37a94d6 | Bacteria | p__Firmicutes | c__Clostridia | o__Clostridiales | f__Clostridiaceae | g__Clostridium_sensu_stricto_1 |  |
| MJ030-1-barcode42-umi88462bins-ubs-4 | Bacteria | p__Proteobacteria | c__Gammaproteobacteria | o__Burkholderiales | f__Comamonadaceae |  |  |
| 04909801 | Bacteria | p__Proteobacteria | c__Alphaproteobacteria | o__Rhizobiales | f__Devosiaceae | g__Devosia |  |
| RS-GCF-009906335.1-NZ-JAAALX010000087.1 | Bacteria | p__Proteobacteria | c__Gammaproteobacteria | o__Aeromonadales |  |  |  |
| G006170445_1 | Bacteria | p__Firmicutes | c__Bacilli | o__Brevibacillales | f__Brevibacillaceae | g__Brevibacillus | s__bacterium_YC-LK-LKJ16 |
| 6f798e4e277414aa84b14de4b387b02b | Bacteria | p__Desulfobacterota | c__Desulfovibrionia | o__Desulfovibrionales | f__Desulfovibrionaceae | g__Desulfovibrio | s__metagenome |
| G002159625_1 | Bacteria | p__Actinobacteriota | c__Coriobacteriia | o__Coriobacteriales | f__Atopobiaceae |  |  |
| 69dace152d59db42029b947c3c9c75e0 | Bacteria | p__Bacteroidota | c__Bacteroidia | o__Bacteroidales | f__Bacteroidaceae | g__Bacteroides | s__Bacteroides_graminisolvens |
| 07160096 | Bacteria | p__Planctomycetota | c__Phycisphaerae | o__Tepidisphaerales | f__WD2101_soil_group | g__WD2101_soil_group |  |
| 1aa449cd8715d09ec0e6d8a79c5f21a1 | Bacteria | p__NB1-j | c__NB1-j | o__NB1-j | f__NB1-j | g__NB1-j | s__uncultured_Desulfoglaeba |
| MJ031-1-barcode30-umi33634bins-ubs-8 | Bacteria | p__Proteobacteria | c__Gammaproteobacteria | o__Burkholderiales | f__Comamonadaceae |  |  |
| MJ034-1-barcode41-umi18671bins-ubs-12 | Bacteria | p__Proteobacteria | c__Gammaproteobacteria | o__Gammaproteobacteria_Incertae_Sedis | f__Unknown_Family | g__Acidibacter | s__uncultured_eubacterium |
| e8d2bcbf9d3207c25067946e95a1f889 | Bacteria | p__Actinobacteriota | c__Coriobacteriia | o__Coriobacteriales | f__Coriobacteriales_Incertae_Sedis | g__Raoultibacter |  |
| ccaa1b81600698781d044a13ce85808a | Bacteria | p__Bacteroidota | c__Bacteroidia | o__Bacteroidales | f__Bacteroidaceae | g__Bacteroides | s__Bacteroides_graminisolvens |
| d0e2f778706fa1699656d6335b70b206 | Bacteria | p__Proteobacteria | c__Alphaproteobacteria | o__Rhizobiales | f__Hyphomicrobiaceae | g__Hyphomicrobium |  |
| 40e52f8bf010c6d340f7810d31d1016c | Bacteria | p__Proteobacteria | c__Gammaproteobacteria | o__Competibacterales | f__Competibacteraceae | g__Candidatus_Competibacter |  |
| c5085c44520483bdcf233cd16373a0d1 | Bacteria | p__Chloroflexi | c__Anaerolineae | o__Caldilineales | f__Caldilineaceae | g__uncultured | s__metagenome |
| 983299ad95b74150ef512d24ba4ef770 | Bacteria | p__Proteobacteria | c__Gammaproteobacteria | o__Pseudomonadales | f__Moraxellaceae | g__Acinetobacter |  |
| 0576cce36b6af21a401ee555f50cd86b | Bacteria | p__Actinobacteriota | c__Acidimicrobiia | o__IMCC26256 | f__IMCC26256 | g__IMCC26256 |  |
| MJ031-1-barcode27-umi17615bins-ubs-25 | Bacteria | p__Proteobacteria | c__Gammaproteobacteria | o__Pseudomonadales | f__Moraxellaceae | g__Acinetobacter |  |
| MJ006-1-barcode38-umi146207bins-ubs-3 | Bacteria | p__Firmicutes | c__Clostridia | o__Oscillospirales | f__Ruminococcaceae | g__[Eubacterium]_siraeum_group |  |
| 333a7ba1fe197fafb996007d4340898d | Bacteria | p__Proteobacteria | c__Gammaproteobacteria | o__Burkholderiales | f__Comamonadaceae | g__Aquabacterium |  |
| beea631dbca79bcf3c164c2522322c1c | Bacteria | p__Bacteroidota | c__Bacteroidia | o__Flavobacteriales | f__Weeksellaceae | g__Chryseobacterium |  |
| fd5aa3949ac75ac53e2661d2aa0f6272 | Bacteria | p__Verrucomicrobiota | c__Verrucomicrobiae | o__Verrucomicrobiales | f__Verrucomicrobiaceae | g__Verrucomicrobium |  |
| 04881494 | Bacteria | p__Firmicutes | c__Bacilli | o__Lactobacillales | f__Streptococcaceae | g__Streptococcus |  |
| 0b5a9fd3c34dd857acfea733d69228ad | Bacteria | p__RCP2-54 | c__RCP2-54 | o__RCP2-54 | f__RCP2-54 | g__RCP2-54 |  |
| fb3e366550fdd809996cfad012f9d66a | Bacteria | p__Actinobacteriota | c__Acidimicrobiia | o__Microtrichales |  |  |  |
| 274a306a45d73b28f1d6a5eb2aa69331 | Bacteria | p__Firmicutes | c__Bacilli | o__Exiguobacterales | f__Exiguobacteraceae | g__Exiguobacterium |  |
| 4b6cb86ce6734317285f882ba60170b8 | Bacteria | p__Proteobacteria | c__Alphaproteobacteria | o__Sphingomonadales | f__Sphingomonadaceae |  |  |
| aa5736e14786a586ccabfea7175dbd3e | Bacteria | p__Actinobacteriota | c__Actinobacteria | o__Bifidobacteriales | f__Bifidobacteriaceae | g__Bifidobacterium |  |
| 8c6385d2f56b5984c0d9f2aef89b45a1 | Bacteria | p__Actinobacteriota | c__Actinobacteria | o__Propionibacteriales | f__Nocardioidaceae | g__Nocardioides |  |
| 20809929 | Bacteria | p__Bacteroidota | c__Bacteroidia | o__Cytophagales | f__Microscillaceae | g__uncultured |  |
| 3e3fb34d54f6a137bfff060a9105850d | Bacteria | p__Firmicutes | c__Clostridia | o__Clostridiales | f__Clostridiaceae | g__Clostridium_sensu_stricto_1 |  |
| eb14ba0f7b8368a1ea6d6e758418c33a | Bacteria | p__Bacteroidota | c__Bacteroidia | o__Flavobacteriales | f__Weeksellaceae | g__Chryseobacterium |  |
| d9935e6705c2baec7979d6a985057bea | Bacteria | p__Actinobacteriota | c__Actinobacteria | o__Actinomycetales | f__Actinomycetaceae | g__uncultured | s__metagenome |
| AB178392 | Bacteria | p__Proteobacteria | c__Alphaproteobacteria | o__Acetobacterales | f__Acetobacteraceae | g__Gluconobacter |  |
| RS-GCF-900291475.1-NZ-OLMM01000005.1 | Bacteria | p__Firmicutes | c__Negativicutes | o__Acidaminococcales | f__Acidaminococcaceae | g__Acidaminococcus | s__uncultured_bacterium |
| 26ed00226ad6d9ff38c04b89f8055b84 | Bacteria | p__Firmicutes | c__Bacilli | o__Bacillales | f__Planococcaceae |  |  |
| 08779276 | Bacteria | p__Proteobacteria | c__Gammaproteobacteria | o__Xanthomonadales | f__Xanthomonadaceae | g__Vulcaniibacterium | s__uncultured_bacterium |
| 04919043 | Bacteria | p__Bacteroidota | c__Bacteroidia | o__Sphingobacteriales | f__Sphingobacteriaceae | g__Sphingobacterium |  |
| 81cb3045608893964df8f813a3b43dd7 | Bacteria | p__Proteobacteria | c__Gammaproteobacteria | o__Burkholderiales | f__SC-I-84 | g__SC-I-84 |  |
| 759369db650345081fa1b3a3e0091727 | Bacteria | p__Bacteroidota | c__Bacteroidia | o__Flavobacteriales | f__Flavobacteriaceae |  |  |
| 7fd49b2d9cf7afa8996fefc1c51676e2 | Bacteria | p__Bacteroidota | c__Bacteroidia | o__Flavobacteriales | f__Flavobacteriaceae | g__Myroides | s__Myroides_odoratus |
| AY043359 | Bacteria | p__Actinobacteriota | c__Actinobacteria | o__Micrococcales | f__Micrococcaceae | g__Rothia |  |
| 04891857 | Bacteria | p__Chloroflexi | c__Anaerolineae | o__SBR1031 | f__A4b | g__OLB13 | s__uncultured_bacterium |
| a617ef2c3e93f36f80734e29caf5c9db | Bacteria | p__Acidobacteriota | c__Vicinamibacteria | o__Vicinamibacterales | f__uncultured | g__uncultured |  |
| 6475fc814711e978339588c1fd9150b5 | Bacteria | p__Proteobacteria | c__Alphaproteobacteria | o__Caulobacterales | f__Caulobacteraceae | g__Brevundimonas |  |
| 0ae9eaf09dec87284bcbeddef0426206 | Bacteria | p__Myxococcota | c__Polyangia | o__Haliangiales | f__Haliangiaceae | g__Haliangium |  |
| RS-GCF-002355115.1-NZ-NWSO01000050.1 | Bacteria | p__Proteobacteria | c__Alphaproteobacteria | o__Rhizobiales | f__Rhizobiaceae | g__Allorhizobium-Neorhizobium-Pararhizobium-Rhizobium |  |
| aef78629f94148d098ac23cbba95b693 | Bacteria | p__Firmicutes | c__Negativicutes | o__Veillonellales-Selenomonadales | f__Veillonellaceae | g__Veillonella |  |
| 09604e6bf3f7953d22cd79f96ca1e44a | Bacteria | p__Bacteroidota | c__Bacteroidia | o__Bacteroidales | f__Muribaculaceae | g__Muribaculaceae | s__uncultured_bacterium |
| 39b3f5faee32ecc9299eac6f62768782 | Bacteria | p__Acidobacteriota | c__Acidobacteriae | o__Acidobacteriales | f__uncultured | g__uncultured |  |
| bfb7748def5b81ae4e86a902cff3f610 | Bacteria | p__Proteobacteria | c__Gammaproteobacteria | o__Enterobacterales | f__Enterobacteriaceae |  |  |
| e20976c268ab78e6cd06661368661f3d | Bacteria | p__Actinobacteriota | c__Actinobacteria | o__Propionibacteriales | f__Propionibacteriaceae | g__Microlunatus |  |
| 03847024 | Bacteria | p__Proteobacteria | c__Alphaproteobacteria | o__uncultured | f__uncultured | g__uncultured |  |
| MJ010-2-barcode73-umi121005bins-ubs-4 | Bacteria | p__Proteobacteria | c__Gammaproteobacteria | o__Burkholderiales | f__Burkholderiaceae | g__Lautropia |  |
| 1a44bb6fa9b3fea8c45b1d2c13b92cf1 | Bacteria | p__Proteobacteria | c__Gammaproteobacteria | o__Xanthomonadales | f__Xanthomonadaceae | g__Stenotrophomonas |  |
| 04957715 | Bacteria | p__Proteobacteria | c__Gammaproteobacteria | o__Burkholderiales | f__Oxalobacteraceae | g__Massilia |  |
| 03405320 | Bacteria | p__Planctomycetota | c__Planctomycetes | o__Gemmatales | f__Gemmataceae | g__uncultured |  |
| 04995526 | Bacteria | p__Proteobacteria | c__Alphaproteobacteria | o__Rhizobiales | f__Devosiaceae | g__Devosia |  |
| 13720543 | Bacteria | p__Gemmatimonadota | c__Gemmatimonadetes | o__Gemmatimonadales | f__Gemmatimonadaceae | g__Gemmatimonas |  |
| 36c6e302f47dc390dbc20e8a534e80d7 | Bacteria | p__Proteobacteria | c__Gammaproteobacteria | o__Gammaproteobacteria_Incertae_Sedis | f__Unknown_Family | g__Acidibacter |  |
| RS-GCF-900155535.1-NZ-LT706957.1 | Bacteria | p__Actinobacteriota | c__Actinobacteria | o__Corynebacteriales | f__Corynebacteriaceae | g__Corynebacterium | s__Corynebacterium_appendicis |
| KP657488 | Bacteria | p__Proteobacteria | c__Alphaproteobacteria | o__Sphingomonadales | f__Sphingomonadaceae | g__Novosphingobium |  |
| 8db83fb4c92df55059ab624502e527d0 | Bacteria | p__Firmicutes | c__Symbiobacteriia | o__Symbiobacteriales | f__Symbiobacteraceae | g__Symbiobacterium |  |
| 02360518 | Bacteria | p__Proteobacteria | c__Alphaproteobacteria | o__Rhizobiales | f__Xanthobacteraceae | g__uncultured |  |
| G001571365_1 | Bacteria | p__Proteobacteria | c__Gammaproteobacteria | o__Burkholderiales | f__Alcaligenaceae | g__Achromobacter |  |
| 66ce691afe6555b6782b93b76aa6b5d1 | Bacteria | p__Acidobacteriota | c__Acidobacteriae | o__Acidobacteriales | f__Koribacteraceae | g__Candidatus_Koribacter |  |
| 01254303 | Bacteria | p__Firmicutes | c__Clostridia | o__Oscillospirales | f__Ruminococcaceae | g__uncultured | s__[Clostridium]_leptum |
| 15377018 | Bacteria | p__Firmicutes | c__Clostridia | o__Oscillospirales | f__Ruminococcaceae |  |  |
| MJ010-2-barcode71-umi87595bins-ubs-7 | Bacteria | p__Bacteroidota | c__Bacteroidia | o__Sphingobacteriales | f__NS11-12_marine_group | g__NS11-12_marine_group | s__Bacteroidetes_bacterium |
| RS-GCF-014635225.1-NZ-BMCO01000009.1 | Bacteria | p__Firmicutes | c__Bacilli | o__Staphylococcales | f__Staphylococcaceae | g__Jeotgalicoccus |  |
| 05036921 | Bacteria | p__Firmicutes | c__Bacilli | o__Bacillales | f__Bacillaceae |  |  |
| aceae3b19c65a2fa58563401263558c2 | Bacteria | p__Chloroflexi | c__Anaerolineae | o__C10-SB1A | f__C10-SB1A | g__C10-SB1A | s__metagenome |
| d47b5363ff135712d4fef42bd6cd1761 | Bacteria | p__Firmicutes | c__Clostridia | o__Peptostreptococcales-Tissierellales | f__Peptostreptococcaceae |  |  |
| f0f1aface62f6356efb2d8d9ca92a777 | Bacteria | p__Chloroflexi | c__Anaerolineae | o__SBR1031 | f__A4b | g__A4b |  |
| 629ec0d35f54ff898b1f18b27b605852 | Bacteria | p__Firmicutes | c__Bacilli | o__Bacillales | f__Bacillaceae | g__Bacillus |  |
| 04956040 | Bacteria | p__Actinobacteriota | c__Thermoleophilia | o__Gaiellales | f__uncultured | g__uncultured |  |
| 0a722b6fbcf8af26ad72db75c651e1f0 | Bacteria | p__Bacteroidota | c__Bacteroidia | o__Sphingobacteriales | f__Sphingobacteriaceae | g__Pedobacter |  |
| MJ006-1-barcode26-umi16914bins-ubs-16 | Bacteria | p__Firmicutes | c__Bacilli | o__RF39 | f__RF39 | g__RF39 |  |
| 87a256c2d10fddb593d0a906a4e7f621 | Bacteria | p__Proteobacteria | c__Gammaproteobacteria | o__Burkholderiales | f__Rhodocyclaceae |  |  |
| 906a77e51c956b281c25525bbd5f8e58 | Bacteria |  |  |  |  |  |  |
| a7a6d1148ca2ebe32b773243fe3edd50 | Bacteria |  |  |  |  |  |  |
| 03712448 | Bacteria | p__Planctomycetota | c__BD7-11 | o__BD7-11 | f__BD7-11 | g__BD7-11 | s__uncultured_bacterium |
| 03494570 | Bacteria | p__Planctomycetota | c__Planctomycetes | o__Gemmatales | f__Gemmataceae | g__uncultured | s__uncultured_bacterium |
| 6bcfbe90283b53f43b3a7004536e34d8 | Bacteria | p__Bacteroidota | c__Bacteroidia | o__Sphingobacteriales | f__Sphingobacteriaceae | g__Mucilaginibacter |  |
| 59f7bb34d97cae7a7bbb6babb7bc09fd | Bacteria | p__Proteobacteria | c__Gammaproteobacteria | o__Xanthomonadales | f__Xanthomonadaceae | g__Arenimonas |  |
| 5d17cd6b99986241d20ed3ff27ed5bb0 | Bacteria | p__Myxococcota | c__Myxococcia | o__Myxococcales | f__Myxococcaceae | g__P3OB-42 |  |
| 02858358 | Bacteria | p__Chloroflexi | c__Chloroflexia | o__Chloroflexales | f__Roseiflexaceae | g__uncultured | s__uncultured_bacterium |
| 08238161 | Bacteria | p__Proteobacteria | c__Alphaproteobacteria | o__Rhizobiales | f__Xanthobacteraceae | g__Rhodopseudomonas |  |
| f9829f687f484be51d04f59c04eb1b67 | Bacteria |  |  |  |  |  |  |
| MJ030-1-barcode42-umi103778bins-ubs-3 | Bacteria | p__Proteobacteria | c__Gammaproteobacteria | o__Burkholderiales | f__TRA3-20 | g__TRA3-20 | s__uncultured_Nitrosomonadales |
| 04996392 | Bacteria | p__Proteobacteria | c__Gammaproteobacteria | o__Xanthomonadales | f__Xanthomonadaceae | g__Stenotrophomonas |  |
| f0f1167335c3a789a932675dcbcf5459 | Bacteria | p__Actinobacteriota | c__Thermoleophilia | o__Gaiellales | f__uncultured | g__uncultured |  |
| 04879571 | Bacteria | p__Proteobacteria | c__Gammaproteobacteria | o__Pseudomonadales | f__Pseudomonadaceae | g__Pseudomonas |  |
| cc0f35582550c9809f754849e26feaed | Bacteria | p__Patescibacteria | c__Saccharimonadia | o__Saccharimonadales | f__Saccharimonadales | g__Saccharimonadales |  |
| MJ009-1-barcode25-umi17781bins-ubs-23 | Bacteria | p__Firmicutes | c__Clostridia | o__Oscillospirales | f__Ruminococcaceae | g__Ruminococcus |  |
| AM406674 | Bacteria | p__Actinobacteriota | c__Actinobacteria | o__Corynebacteriales | f__Nocardiaceae | g__Gordonia |  |
| a67d9f3ec7ee8bdaa2cd89bb0f5bf62b | Bacteria | p__Proteobacteria | c__Gammaproteobacteria | o__Burkholderiales | f__Rhodocyclaceae | g__Dechloromonas |  |
| 1caeb87f87e113e48f19076de35b78f1 | Bacteria | p__Firmicutes | c__Clostridia | o__Oscillospirales | f__Ruminococcaceae | g__Ruminococcus |  |
| 3becfd74b266dae8b240a72a7199ee29 | Bacteria | p__Firmicutes | c__Bacilli | o__Bacillales | f__Bacillaceae | g__Bacillus |  |
| 08477450 | Bacteria | p__Proteobacteria | c__Alphaproteobacteria | o__Rhizobiales | f__Rhizobiaceae | g__Aureimonas | s__Aureimonas_frigidaquae |
| 65204e97860f9737462b2a8780e4b6cd | Bacteria | p__Proteobacteria | c__Gammaproteobacteria | o__Pseudomonadales | f__Moraxellaceae | g__Acinetobacter |  |
| 375e779a33a3a9c7ac9608d42c7f673d | Bacteria | p__Proteobacteria | c__Alphaproteobacteria | o__Rhizobiales | f__Xanthobacteraceae | g__uncultured | s__uncultured_Bradyrhizobiaceae |
| AJ853909 | Bacteria | p__Actinobacteriota | c__Actinobacteria | o__Micrococcales | f__Microbacteriaceae | g__Microbacterium |  |
| 04996405 | Bacteria | p__Proteobacteria | c__Gammaproteobacteria | o__Xanthomonadales | f__Xanthomonadaceae | g__Stenotrophomonas |  |
| b575c94bafe9447bbc5fef9db3045fe5 | Bacteria | p__Bdellovibrionota | c__Bdellovibrionia | o__Bdellovibrionales | f__Bdellovibrionaceae | g__Bdellovibrio |  |
| 95d2030f66cd60b7c03e949fff53b448 | Bacteria | p__Actinobacteriota | c__Actinobacteria |  |  |  |  |
| 11355322 | Bacteria | p__Acidobacteriota | c__Subgroup_25 | o__Subgroup_25 | f__Subgroup_25 | g__Subgroup_25 | s__uncultured_Acidobacteria |
| 434a23a5ed5b4790fff997cfb52e3d62 | Bacteria | p__Proteobacteria | c__Alphaproteobacteria | o__Rhizobiales | f__Xanthobacteraceae | g__Bradyrhizobium |  |
| 12a8282b3a6a978f68d9067a205c940e | Bacteria | p__Actinobacteriota | c__Actinobacteria | o__Corynebacteriales | f__Dietziaceae | g__Dietzia |  |
| d6e977c8026db2b9e550aa82f726accf | Bacteria | p__Bacteroidota | c__Bacteroidia | o__Bacteroidales | f__Bacteroidaceae | g__Bacteroides |  |
| 05131795 | Bacteria | p__Actinobacteriota | c__Actinobacteria | o__Micrococcales | f__Microbacteriaceae |  |  |
| 62b2013c6252420081a7e2ec97a6814b | Bacteria | p__Methylomirabilota | c__Methylomirabilia | o__Rokubacteriales | f__Rokubacteriales | g__Rokubacteriales |  |
| d809020286c8e94d9b759221951d7765 | Bacteria | p__Proteobacteria | c__Alphaproteobacteria | o__Rhizobiales | f__Rhizobiaceae | g__Allorhizobium-Neorhizobium-Pararhizobium-Rhizobium |  |
| EU400621 | Bacteria | p__Firmicutes | c__Bacilli | o__Paenibacillales | f__Paenibacillaceae | g__Paenibacillus | s__Paenibacillus_camelliae |
| 9e8551642bb8fe5ae5db4167950da697 | Bacteria | p__Bacteroidota | c__Bacteroidia | o__Bacteroidales | f__Bacteroidaceae | g__Bacteroides |  |
| be20636b54f03b076c58298c5a67a6d9 | Bacteria | p__Actinobacteriota | c__Actinobacteria | o__Streptomycetales | f__Streptomycetaceae | g__Streptomyces |  |
| b8ef2bee9fcc4f88afe8c6f14bd64684 | Bacteria | p__Acidobacteriota | c__Vicinamibacteria | o__Vicinamibacterales | f__uncultured | g__uncultured |  |
| 6eac075f11b22e01d408a7846846d1bf | Bacteria | p__Firmicutes | c__Clostridia | o__Christensenellales | f__Christensenellaceae | g__Christensenellaceae_R-7_group |  |
| 1080ac85878a923a7355f03f61547033 | Bacteria | p__Bacteroidota | c__Bacteroidia | o__Bacteroidales | f__Bacteroidaceae | g__Bacteroides |  |
| 9884868dda85bd2f25a7d127b8db9684 | Bacteria | p__Actinobacteriota | c__Actinobacteria | o__Bifidobacteriales | f__Bifidobacteriaceae | g__Bifidobacterium | s__Bifidobacterium_breve |
| ba399ba0d304778c5d7d3d24118457ad | Bacteria | p__Actinobacteriota | c__MB-A2-108 | o__MB-A2-108 | f__MB-A2-108 | g__MB-A2-108 |  |
| 4f3b060ef772d2c794702eaeb10b51e5 | Bacteria | p__Proteobacteria | c__Alphaproteobacteria | o__Rhizobiales | f__Rhizobiaceae | g__Allorhizobium-Neorhizobium-Pararhizobium-Rhizobium |  |
| 3c30a7b0db7bc9cca75eb9a983859185 | Bacteria | p__Actinobacteriota | c__Acidimicrobiia | o__Microtrichales | f__Ilumatobacteraceae | g__uncultured |  |
| 02237965 | Bacteria | p__Chloroflexi | c__Chloroflexia | o__Thermomicrobiales | f__JG30-KF-CM45 | g__JG30-KF-CM45 |  |
| 04924132 | Bacteria | p__Bacteroidota | c__Bacteroidia | o__Bacteroidales | f__Muribaculaceae | g__Muribaculaceae |  |
| f8f128d1215fc4651738a6af11f9e49f | Bacteria | p__Actinobacteriota | c__Acidimicrobiia | o__Microtrichales | f__Microtrichaceae | g__IMCC26207 |  |
| 7f124d092bd81ef6213a6704a5e952e7 | Bacteria | p__Proteobacteria | c__Gammaproteobacteria | o__Salinisphaerales | f__Solimonadaceae | g__Nevskia |  |
| 64c3e008b1bf65e23831ab59095af548 | Bacteria | p__Actinobacteriota | c__Actinobacteria | o__Corynebacteriales | f__Corynebacteriaceae | g__Corynebacterium |  |
| f5cd92664f02f9e77c46c4fe8a1b6866 | Bacteria | p__Proteobacteria | c__Alphaproteobacteria | o__Sphingomonadales | f__Sphingomonadaceae | g__Sphingomonas |  |
| 39f12576045eaca382c23da785540799 | Bacteria | p__Bacteroidota | c__Bacteroidia | o__Chitinophagales | f__Chitinophagaceae | g__Niastella | s__Niastella_koreensis |
| G000967825_4 | Bacteria | p__Proteobacteria | c__Alphaproteobacteria | o__Rhodobacterales | f__Rhodobacteraceae | g__Paracoccus |  |
| 00169147 | Bacteria | p__Chloroflexi | c__Anaerolineae | o__SBR1031 | f__SBR1031 | g__SBR1031 | s__uncultured_soil |
| b9a632dcac0cf46918f224ecfed899ae | Bacteria | p__Actinobacteriota | c__Thermoleophilia | o__Gaiellales | f__uncultured | g__uncultured |  |
| G000817735_1 | Bacteria | p__Cyanobacteria | c__Cyanobacteriia | o__Cyanobacteriales | f__Chroococcidiopsaceae | g__Chroococcidiopsis_PCC_7203 | s__Gloeocapsa_sp. |
| bead3daeffa975e2e66d80f3cc0f9537 | Bacteria | p__Actinobacteriota | c__Actinobacteria |  |  |  |  |
| c12cad06db4b51bf3a5faaf64d69b4ba | Bacteria | p__Proteobacteria | c__Gammaproteobacteria | o__Thiotrichales | f__Thiotrichaceae | g__Thiothrix |  |
| 4901cc398f48f8a04292ddacbfd081bf | Bacteria | p__Bacteroidota | c__Bacteroidia | o__Bacteroidales | f__Tannerellaceae | g__Parabacteroides |  |
| MJ031-1-barcode30-umi19558bins-ubs-21 | Bacteria | p__Desulfobacterota | c__Desulfovibrionia | o__Desulfovibrionales | f__Desulfomicrobiaceae | g__Desulfomicrobium |  |
| 451414d1a1ca05d5c9580c92e0ee27ff | Bacteria | p__Nitrospirota | c__Nitrospiria | o__Nitrospirales | f__Nitrospiraceae | g__Nitrospira |  |
| 6fe781b14fde054b6ba6a152cec6cfc4 | Bacteria | p__Proteobacteria | c__Alphaproteobacteria | o__Sphingomonadales | f__Sphingomonadaceae | g__Sphingomonas |  |
| 04877832 | Bacteria | p__Actinobacteriota | c__Actinobacteria | o__Micrococcales | f__Micrococcaceae | g__Kocuria |  |
| KT824779 | Bacteria | p__Firmicutes | c__Clostridia | o__Clostridiales | f__Clostridiaceae | g__Clostridium_sensu_stricto_11 |  |
| 41800371074bf122eabebdf3317ba30a | Bacteria | p__Proteobacteria | c__Alphaproteobacteria | o__Acetobacterales | f__Acetobacteraceae | g__uncultured |  |
| 2fcfee290690b6882b6c9377296463d6 | Bacteria | p__Proteobacteria | c__Gammaproteobacteria | o__Pseudomonadales | f__Moraxellaceae | g__Acinetobacter | s__Acinetobacter_ursingii |
| MJ033-1-barcode32-umi9926bins-ubs-34 | Bacteria | p__Acidobacteriota | c__Acidobacteriae | o__Solibacterales | f__Solibacteraceae | g__Candidatus_Solibacter |  |
| 11128156 | Bacteria | p__Bacteroidota | c__Bacteroidia | o__Bacteroidales | f__Muribaculaceae | g__Muribaculaceae |  |
| MJ020-1-barcode44-umi125903bins-ubs-3 | Bacteria | p__Firmicutes | c__Clostridia | o__Clostridiales | f__Clostridiaceae | g__Clostridium_sensu_stricto_10 |  |
| 04958829 | Bacteria | p__Actinobacteriota | c__Actinobacteria | o__Micrococcales | f__Microbacteriaceae |  |  |
| RS-GCF-002074855.1-NZ-JZWJ01000025.1 | Bacteria | p__Proteobacteria | c__Gammaproteobacteria | o__Alteromonadales | f__Shewanellaceae | g__Shewanella |  |
| AB537486 | Bacteria | p__Proteobacteria | c__Gammaproteobacteria | o__Burkholderiales | f__Burkholderiaceae | g__Burkholderia-Caballeronia-Paraburkholderia |  |
| c88df8225981e9c9cf33e84aae97ab29 | Bacteria | p__Firmicutes | c__Bacilli | o__Lactobacillales | f__Enterococcaceae | g__Enterococcus |  |
| 0f27140db3af0d44799fd7106bbe4765 | Bacteria | p__Firmicutes | c__Bacilli | o__Lactobacillales | f__Enterococcaceae | g__Enterococcus |  |
| MJ033-1-barcode32-umi3347bins-ubs-118 | Bacteria | p__Proteobacteria | c__Gammaproteobacteria | o__Burkholderiales | f__Oxalobacteraceae | g__Herbaspirillum |  |
| MJ030-2-barcode65-umi94807bins-ubs-4 | Bacteria | p__Verrucomicrobiota | c__Verrucomicrobiae | o__Pedosphaerales | f__Pedosphaeraceae | g__ADurb.Bin063-1 | s__uncultured_bacterium |
| f98e550e70f2f18e2beda9084d661107 | Bacteria | p__Chloroflexi | c__KD4-96 | o__KD4-96 | f__KD4-96 | g__KD4-96 |  |
| 18bf213235c60abb4707d20e9db88079 | Bacteria | p__Actinobacteriota | c__Actinobacteria | o__Micromonosporales | f__Micromonosporaceae |  |  |
| f1811ab2426c15a992047dde5be19ff1 | Bacteria | p__Proteobacteria | c__Gammaproteobacteria | o__Burkholderiales | f__Nitrosomonadaceae | g__MND1 |  |
| a047ee65b123ddf23c55ef3f8fb2af3e | Bacteria | p__Firmicutes | c__Thermovenabulia | o__Thermovenabulales |  |  |  |
| 31c94402d7bb3cbce2055c1f7ad6aae6 | Bacteria | p__Proteobacteria | c__Gammaproteobacteria | o__Pseudomonadales | f__Pseudomonadaceae | g__Pseudomonas |  |
| 04957621 | Bacteria | p__Proteobacteria | c__Gammaproteobacteria | o__Burkholderiales | f__Comamonadaceae |  |  |
| DQ372987 | Bacteria | p__Proteobacteria | c__Gammaproteobacteria | o__Burkholderiales | f__Comamonadaceae | g__Simplicispira |  |
| 02497021 | Bacteria | p__Chloroflexi | c__Chloroflexia | o__Thermomicrobiales | f__JG30-KF-CM45 | g__JG30-KF-CM45 | s__uncultured_bacterium |
| e20a74ecabdd811dafe3959a7f19aace | Bacteria | p__Acidobacteriota | c__Acidobacteriae | o__Subgroup_2 | f__Subgroup_2 | g__Subgroup_2 |  |
| MJ010-1-barcode47-umi19497bins-ubs-32 | Bacteria | p__Proteobacteria | c__Gammaproteobacteria | o__Pseudomonadales | f__Moraxellaceae | g__Acinetobacter |  |
| MJ034-1-barcode34-umi12429bins-ubs-27 | Bacteria | p__Firmicutes | c__Bacilli | o__Bacillales | f__Bacillaceae | g__Bacillus |  |
| 286bb41985cd67ca5041b3cc10a442e4 | Bacteria | p__Proteobacteria | c__Alphaproteobacteria | o__Acetobacterales | f__Acetobacteraceae |  |  |
| 35b7421496f928993cafb61e6ccd4b6d | Bacteria | p__Firmicutes | c__Bacilli | o__Bacillales | f__Bacillaceae | g__Bacillus |  |
| 0147c814914c930b9f8371129b7afd19 | Bacteria | p__Bacteroidota | c__Bacteroidia | o__Sphingobacteriales | f__Sphingobacteriaceae | g__Sphingobacterium |  |
| cb2e8d398c92f255c8f9548c765f1b5b | Bacteria | p__Actinobacteriota | c__Thermoleophilia | o__Solirubrobacterales | f__67-14 | g__67-14 |  |
| MJ010-2-barcode64-umi74179bins-ubs-8 | Bacteria | p__Proteobacteria | c__Gammaproteobacteria | o__Pseudomonadales | f__Moraxellaceae | g__Acinetobacter |  |
| 02354623 | Bacteria | p__Firmicutes | c__Clostridia | o__Peptostreptococcales-Tissierellales | f__Peptostreptococcaceae | g__Romboutsia |  |
| 56ac4403695628a501ce5c33e20c44a6 | Bacteria | p__Proteobacteria | c__Gammaproteobacteria | o__Pseudomonadales | f__Moraxellaceae | g__Acinetobacter |  |
| e7758bd64124fded0009f0d19750c884 | Bacteria | p__Actinobacteriota | c__Rubrobacteria | o__Rubrobacterales | f__Rubrobacteriaceae | g__Rubrobacter |  |
| 3caad774ce129989d7fe954e00a7ed49 | Bacteria | p__Acidobacteriota | c__Acidobacteriae | o__Subgroup_2 | f__Subgroup_2 | g__Subgroup_2 |  |
| f1d31df2fd1b4e8d9f00df04e0c5fc99 | Bacteria | p__Bacteroidota | c__Bacteroidia | o__Bacteroidales | f__Tannerellaceae | g__Parabacteroides |  |
| 04964761 | Bacteria | p__Firmicutes | c__Clostridia | o__Oscillospirales | f__Oscillospiraceae | g__uncultured |  |
| f1f5dea9926276f9198cf7d7953dced9 | Bacteria | p__Actinobacteriota | c__Actinobacteria | o__Catenulisporales | f__Actinospicaceae | g__Actinospica | s__uncultured_actinobacterium |
| MJ034-2-barcode71-umi1940bins-ubs-221 | Bacteria | p__Proteobacteria | c__Gammaproteobacteria | o__Burkholderiales | f__TRA3-20 | g__TRA3-20 |  |
| 05743033 | Bacteria | p__Chloroflexi | c__Chloroflexia | o__Chloroflexales | f__Roseiflexaceae | g__uncultured |  |
| MK253311 | Bacteria | p__Actinobacteriota | c__Actinobacteria | o__Actinomycetales | f__Actinomycetaceae | g__Flaviflexus |  |
| ad4aed54712addb69eb8333c51241528 | Bacteria | p__Proteobacteria | c__Gammaproteobacteria | o__Pseudomonadales | f__Moraxellaceae | g__Acinetobacter |  |
| 31ecde613defc08b2195e7c42bcbfc08 | Bacteria | p__Proteobacteria | c__Gammaproteobacteria | o__Alteromonadales | f__Shewanellaceae | g__Shewanella |  |
| 17652348 | Bacteria | p__Actinobacteriota | c__Coriobacteriia | o__Coriobacteriales | f__Atopobiaceae | g__Coriobacteriaceae_UCG-002 | s__uncultured_bacterium |
| 0fe2eaf45f81bbf5e8983c629a99100a | Bacteria | p__Proteobacteria | c__Alphaproteobacteria | o__Caulobacterales | f__Hyphomonadaceae | g__SWB02 |  |
| c0de9ee821ca9bbd88cd7f60c04c0e60 | Bacteria | p__Gemmatimonadota | c__Gemmatimonadetes | o__Gemmatimonadales | f__Gemmatimonadaceae | g__uncultured | s__uncultured_proteobacterium |
| e673aebcfa6988a4d8097b07b0eb9660 | Bacteria | p__Actinobacteriota | c__Thermoleophilia | o__Gaiellales | f__uncultured | g__uncultured |  |
| b914e750eca728902f5696347cca4262 | Bacteria | p__Chloroflexi | c__Ktedonobacteria | o__Ktedonobacterales | f__Ktedonobacteraceae | g__FCPS473 |  |
| e0f4f9c60404df1885094cc9dc2035ff | Bacteria | p__Acidobacteriota | c__Vicinamibacteria | o__Vicinamibacterales | f__Vicinamibacteraceae | g__Vicinamibacteraceae |  |
| 6102387339e60e62ac7a0280f0a916fc | Bacteria | p__Actinobacteriota | c__Actinobacteria | o__Micrococcales |  |  |  |
| MK480726 | Bacteria | p__Actinobacteriota | c__Actinobacteria | o__Micrococcales | f__Microbacteriaceae | g__Microbacterium |  |
| 570e4d744c383f4d8f9615d935a23711 | Bacteria | p__Gemmatimonadota | c__Gemmatimonadetes | o__Gemmatimonadales | f__Gemmatimonadaceae | g__Gemmatimonas | s__Gemmatimonadetes_bacterium |
| 05029825 | Bacteria | p__Actinobacteriota | c__Actinobacteria | o__Corynebacteriales | f__Mycobacteriaceae | g__Mycobacterium |  |
| DQ822566 | Bacteria | p__Actinobacteriota | c__Actinobacteria | o__Micrococcales | f__Dermabacteraceae | g__Brachybacterium |  |
| c2bf8a7d3e2b0c4d907fd485320a48e5 | Bacteria | p__Proteobacteria | c__Gammaproteobacteria | o__Enterobacterales |  |  |  |
| b043c60f8fa0e3491b062eb75c5725fe | Bacteria | p__Actinobacteriota | c__Actinobacteria | o__Micromonosporales | f__Micromonosporaceae |  |  |
| GU477780 | Bacteria | p__Firmicutes | c__Bacilli | o__Bacillales | f__Bacillaceae | g__Fictibacillus |  |
| b87cb7561016d77804d6704c2b9fbf5f | Bacteria | p__Proteobacteria | c__Gammaproteobacteria | o__Xanthomonadales | f__Rhodanobacteraceae | g__Ahniella |  |
| 6e232e8e270f8f5e1af8f49427acd1c2 | Bacteria | p__Proteobacteria | c__Gammaproteobacteria | o__Burkholderiales |  |  |  |
| ce3a1033b74aadee86e0e7de185b4198 | Bacteria | p__Bacteroidota | c__Bacteroidia | o__Sphingobacteriales | f__Sphingobacteriaceae | g__Mucilaginibacter |  |
| eebcf09511b97cfedb0145a4edcf3344 | Bacteria | p__Chloroflexi | c__Ktedonobacteria | o__Ktedonobacterales | f__Ktedonobacteraceae | g__HSB_OF53-F07 |  |
| 04963846 | Bacteria | p__Firmicutes | c__Bacilli | o__Bacillales | f__Bacillaceae | g__Fictibacillus |  |
| MJ009-2-barcode65-umi126bins-ubs-1541 | Bacteria | p__Verrucomicrobiota | c__Verrucomicrobiae | o__Pedosphaerales | f__Pedosphaeraceae |  |  |
| a3f59794f13425f3ba267c3028cc2c1f | Bacteria | p__Chloroflexi | c__Anaerolineae | o__Caldilineales | f__Caldilineaceae | g__uncultured | s__metagenome |
| f21a5acf6a99023067442033386061b8 | Bacteria | p__Firmicutes | c__Bacilli | o__Lactobacillales | f__Enterococcaceae | g__Enterococcus |  |
| 04909478 | Bacteria | p__Proteobacteria | c__Alphaproteobacteria | o__Azospirillales | f__Azospirillaceae | g__Azospirillum |  |
| 6670d173977f45c2f1918fa16f63ffe1 | Bacteria | p__Bacteroidota | c__Bacteroidia | o__Bacteroidales | f__Muribaculaceae | g__Muribaculaceae | s__uncultured_bacterium |
| 4d60249594136b8bf1411a7af2f4fb67 | Bacteria | p__Proteobacteria | c__Gammaproteobacteria | o__Burkholderiales | f__Comamonadaceae |  |  |
| RS-GCF-002906715.1-NZ-MIND01000014.1 | Bacteria | p__Proteobacteria | c__Gammaproteobacteria | o__Pseudomonadales | f__Pseudomonadaceae | g__Pseudomonas |  |
| 05032240 | Bacteria | p__Proteobacteria | c__Gammaproteobacteria | o__Burkholderiales | f__Comamonadaceae |  |  |
| f7d0e34bfd0ea995a9f5c7d5f311f34f | Bacteria | p__Acidobacteriota | c__Vicinamibacteria | o__Vicinamibacterales | f__uncultured | g__uncultured |  |
| 68748fd51f5e54342bde640d3d6f866b | Bacteria | p__Actinobacteriota | c__Actinobacteria | o__Micrococcales |  |  |  |
| 5565fcceaf9803f16b11987b3262eb69 | Bacteria | p__Proteobacteria | c__Gammaproteobacteria | o__Pseudomonadales | f__Moraxellaceae | g__Acinetobacter |  |
| 04950247 | Bacteria | p__Firmicutes | c__Clostridia | o__Lachnospirales | f__Lachnospiraceae |  |  |
| 05030656 | Bacteria | p__Proteobacteria | c__Gammaproteobacteria | o__Burkholderiales | f__Oxalobacteraceae | g__Massilia |  |
| 03617b8ff1c3085294217b9b2686a913 | Bacteria | p__Acidobacteriota | c__Blastocatellia | o__Pyrinomonadales | f__Pyrinomonadaceae | g__RB41 |  |
| 42bb66312b4c3b7e29f2bbae70d2c710 | Bacteria | p__Actinobacteriota | c__Thermoleophilia | o__Solirubrobacterales | f__67-14 | g__67-14 |  |
| 73797d559b18cabf605271489e78496b | Bacteria | p__Entotheonellaeota | c__Entotheonellia | o__Entotheonellales | f__Entotheonellaceae | g__Entotheonellaceae |  |
| 04960563 | Bacteria | p__Chloroflexi | c__TK10 | o__TK10 | f__TK10 | g__TK10 |  |
| 36d94c29bfb075d4a9e4684c4e6f2fcb | Bacteria |  |  |  |  |  |  |
| add117526716fb4bee711bef7aaf2e11 | Bacteria | p__Proteobacteria | c__Gammaproteobacteria |  |  |  |  |
| 9a419f64d54f310e104d5834242113df | Bacteria | p__Proteobacteria | c__Gammaproteobacteria | o__Enterobacterales |  |  |  |
| 05949597 | Bacteria | p__Acidobacteriota | c__Vicinamibacteria | o__Vicinamibacterales | f__Vicinamibacteraceae | g__Vicinamibacteraceae |  |
| 11053042 | Bacteria | p__Bacteroidota | c__Bacteroidia | o__Bacteroidales | f__Muribaculaceae | g__Muribaculum |  |
| RS-GCF-016526145.1-NZ-JADDUE010000027.1 | Bacteria | p__Actinobacteriota | c__Actinobacteria | o__Pseudonocardiales | f__Pseudonocardiaceae | g__Saccharopolyspora |  |
| 7bcbbcc98c80b0794f2cad4348c366fd | Bacteria | p__Proteobacteria | c__Gammaproteobacteria | o__Burkholderiales | f__A21b | g__A21b |  |
| 11726051 | Bacteria | p__Myxococcota | c__bacteriap25 | o__bacteriap25 | f__bacteriap25 | g__bacteriap25 | s__metagenome |
| 11077301 | Bacteria | p__Bacteroidota | c__Bacteroidia | o__Bacteroidales | f__Muribaculaceae | g__Muribaculaceae | s__uncultured_bacterium |
| 04990271 | Bacteria | p__Verrucomicrobiota | c__Verrucomicrobiae | o__Pedosphaerales | f__Pedosphaeraceae | g__Pedosphaeraceae |  |
| bfc98708dfb85436e5da8008f1624686 | Bacteria | p__Actinobacteriota | c__Thermoleophilia | o__Solirubrobacterales | f__67-14 | g__67-14 |  |
| 9b61cf58e23a0f929f88039e18b81b27 | Bacteria | p__Actinobacteriota | c__Actinobacteria | o__Frankiales | f__Acidothermaceae | g__Acidothermus |  |
| 5c1c5fcce4a521745efec4fb1be9dabc | Bacteria | p__Bacteroidota | c__Bacteroidia | o__Bacteroidales | f__Rikenellaceae | g__DMER64 |  |
| DQ462461 | Bacteria | p__Proteobacteria | c__Gammaproteobacteria | o__Xanthomonadales | f__Xanthomonadaceae | g__Lysobacter |  |
| 3b1b0f6c972767f1a0a7972e9d990a10 | Bacteria | p__Nitrospirota | c__Nitrospiria | o__Nitrospirales | f__Nitrospiraceae | g__Nitrospira | s__uncultured_Green |
| 04909787 | Bacteria | p__Proteobacteria | c__Alphaproteobacteria | o__Rhizobiales | f__Rhizobiaceae | g__Ochrobactrum |  |
| 85ec399683ca564dbf9f89938a33648b | Bacteria | p__Actinobacteriota | c__Actinobacteria | o__Micrococcales | f__Brevibacteriaceae | g__Brevibacterium |  |
| bb7403a4a6831f9578ecc0b9959b2053 | Bacteria | p__Proteobacteria | c__Alphaproteobacteria | o__Rickettsiales | f__Anaplasmataceae | g__Wolbachia |  |
| ede26ccbe6527f42ec892cd84ccd08dd | Bacteria | p__Actinobacteriota | c__Acidimicrobiia | o__IMCC26256 | f__IMCC26256 | g__IMCC26256 | s__uncultured_Ferrimicrobium |
| 04968357 | Bacteria | p__Firmicutes | c__Bacilli | o__Lactobacillales | f__Aerococcaceae | g__Aerococcus |  |
| 1ab5006fc6c7d9d40ef612e76cdff410 | Bacteria | p__Verrucomicrobiota | c__Verrucomicrobiae | o__Chthoniobacterales | f__Chthoniobacteraceae | g__Chthoniobacter | s__uncultured_bacterium |
| 04377421 | Bacteria | p__Armatimonadota | c__Fimbriimonadia | o__Fimbriimonadales | f__Fimbriimonadaceae | g__Fimbriimonadaceae |  |
| RS-GCF-001563285.1-NZ-CP005188.1 | Bacteria | p__Proteobacteria | c__Alphaproteobacteria | o__Sphingomonadales | f__Sphingomonadaceae | g__Sphingobium |  |
| 04957361 | Bacteria | p__Proteobacteria | c__Gammaproteobacteria | o__Burkholderiales | f__Comamonadaceae |  |  |
| d34a9f78247cc4c8c0387d061600a0a2 | Bacteria | p__Acidobacteriota | c__Blastocatellia | o__Blastocatellales | f__Blastocatellaceae |  |  |
| MJ032-2-barcode60-umi47048bins-ubs-11 | Bacteria | p__Proteobacteria | c__Gammaproteobacteria | o__Pseudomonadales | f__Moraxellaceae | g__Acinetobacter |  |
| 56ad5d9ab4637b4564a5767dbedc67b4 | Bacteria | p__Actinobacteriota | c__Actinobacteria | o__Frankiales | f__Sporichthyaceae | g__uncultured |  |
| 4604996ba9d947261141065cab54ab90 | Bacteria | p__Proteobacteria | c__Gammaproteobacteria | o__Burkholderiales | f__SC-I-84 | g__SC-I-84 |  |
| MJ006-2-barcode69-umi149253bins-ubs-3 | Bacteria | p__Bacteroidota | c__Bacteroidia | o__Bacteroidales | f__Tannerellaceae | g__Parabacteroides |  |
| f5e237821a08465eff84a0866f7f44d2 | Bacteria | p__Proteobacteria | c__Gammaproteobacteria | o__Pseudomonadales | f__Pseudomonadaceae | g__Pseudomonas |  |
| 99200612ea3385db0a235dcd15b43137 | Bacteria | p__Bacteroidota | c__Bacteroidia | o__Bacteroidales | f__Muribaculaceae | g__Muribaculaceae |  |
| cd63810e8bd9f2af4984a5ed245c52a7 | Bacteria | p__Acidobacteriota | c__Vicinamibacteria | o__Vicinamibacterales | f__uncultured | g__uncultured |  |
| 6d3e6d097b1afb9b2b8dba9c3d4c6519 | Bacteria | p__Proteobacteria | c__Gammaproteobacteria | o__Enterobacterales | f__Enterobacteriaceae |  |  |
| 18449819 | Bacteria | p__Proteobacteria | c__Gammaproteobacteria | o__Burkholderiales | f__Comamonadaceae |  |  |
| 85f000cc528c678fe1637299183baa2b | Bacteria |  |  |  |  |  |  |
| 04930741 | Bacteria | p__Proteobacteria | c__Gammaproteobacteria | o__Enterobacterales | f__Enterobacteriaceae |  |  |
| a08c8c06ef205953fcfb1acbbd4bca31 | Bacteria | p__Proteobacteria | c__Gammaproteobacteria | o__Pseudomonadales | f__Moraxellaceae | g__Acinetobacter |  |
| JQ898451 | Bacteria | p__Actinobacteriota | c__Actinobacteria | o__Corynebacteriales | f__Mycobacteriaceae | g__Mycobacterium |  |
| 011915addff33f892d931057646401e5 | Bacteria | p__Proteobacteria | c__Gammaproteobacteria | o__Cellvibrionales | f__Cellvibrionaceae | g__Cellvibrio |  |
| e1f81e18fe18361564ca891cbb7d89c5 | Bacteria | p__Proteobacteria | c__Gammaproteobacteria | o__Steroidobacterales | f__Steroidobacteraceae | g__uncultured |  |
| 05077813 | Bacteria | p__Proteobacteria | c__Gammaproteobacteria | o__Pseudomonadales | f__Moraxellaceae | g__Psychrobacter | s__Psychrobacter_alimentarius |
| 277bbaa29de7482733f8ef106d3295ab | Bacteria | p__Actinobacteriota | c__Actinobacteria | o__Frankiales | f__Acidothermaceae | g__Acidothermus |  |
| 04994193 | Bacteria | p__Proteobacteria | c__Alphaproteobacteria | o__Caulobacterales | f__Caulobacteraceae | g__Brevundimonas |  |
| 371b28346457dc623111533e193d1bef | Bacteria | p__Proteobacteria | c__Gammaproteobacteria | o__Burkholderiales | f__Burkholderiaceae | g__Ralstonia |  |
| MJ034-1-barcode30-umi26631bins-ubs-4 | Bacteria | p__Acidobacteriota | c__Acidobacteriae | o__Solibacterales | f__Solibacteraceae | g__Candidatus_Solibacter |  |
| 11438578 | Bacteria | p__Proteobacteria | c__Alphaproteobacteria | o__Sphingomonadales | f__Sphingomonadaceae | g__Sphingomonas |  |
| 154d867817c9084eb972b0e983fbebd7 | Bacteria | p__Firmicutes | c__Bacilli | o__Staphylococcales | f__Staphylococcaceae | g__Staphylococcus |  |
| 19706853 | Bacteria | p__Firmicutes | c__Bacilli | o__Erysipelotrichales | f__Erysipelotrichaceae | g__Ileibacterium | s__Ileibacterium_valens |
| 268a54f052fa564ae4ff4c9e7edd581a | Bacteria | p__Proteobacteria | c__Gammaproteobacteria | o__Thiotrichales | f__Thiotrichaceae | g__Thiothrix |  |
| f425a663f3edd3b442dbfd2a4377aaae | Bacteria | p__Actinobacteriota | c__Actinobacteria | o__Micrococcales | f__Intrasporangiaceae |  |  |
| AM422371 | Bacteria | p__Proteobacteria | c__Alphaproteobacteria | o__Rhizobiales | f__Rhizobiaceae |  |  |
| dd980c43a8bfccded44ab55406e79c79 | Bacteria | p__Proteobacteria | c__Gammaproteobacteria | o__Burkholderiales | f__Nitrosomonadaceae | g__MND1 |  |
| d68dd1d7c4b9a137f622d377f55fd1c6 | Bacteria | p__Proteobacteria | c__Gammaproteobacteria | o__Burkholderiales | f__Rhodocyclaceae |  |  |
| 05013394 | Bacteria | p__Bacteroidota | c__Bacteroidia | o__Chitinophagales | f__Chitinophagaceae | g__Terrimonas |  |
| MJ010-1-barcode29-umi129849bins-ubs-4 | Bacteria | p__Bacteroidota | c__Bacteroidia | o__Flavobacteriales | f__Crocinitomicaceae | g__Fluviicola |  |
| 2a14e20dbf37ede2684963f0332f050a | Bacteria | p__Firmicutes | c__Bacilli | o__Bacillales | f__Marinococcaceae | g__Alteribacillus |  |
| 18300442 | Bacteria | p__Chloroflexi | c__Chloroflexia | o__Thermomicrobiales | f__JG30-KF-CM45 | g__JG30-KF-CM45 | s__uncultured_Sphaerobacter |
| faae95c193539c88a28d52f7540cde65 | Bacteria | p__Actinobacteriota | c__Rubrobacteria | o__Rubrobacterales | f__Rubrobacteriaceae | g__Rubrobacter |  |
| KY425604 | Bacteria | p__Proteobacteria | c__Gammaproteobacteria | o__Burkholderiales | f__Comamonadaceae |  |  |
| f2f093104edd823946555814ee10a54f | Bacteria | p__Proteobacteria | c__Alphaproteobacteria | o__Rhodobacterales | f__Rhodobacteraceae |  |  |
| 26c0c299f622739dd6d66caa466949c5 | Bacteria | p__Bacteroidota | c__Bacteroidia | o__Flavobacteriales | f__Crocinitomicaceae | g__Fluviicola |  |
| MJ034-1-barcode30-umi23707bins-ubs-8 | Bacteria | p__Acidobacteriota | c__Acidobacteriae | o__Solibacterales | f__Solibacteraceae | g__Candidatus_Solibacter |  |
| 04909534 | Bacteria | p__Proteobacteria | c__Alphaproteobacteria | o__Azospirillales | f__Azospirillaceae | g__Niveispirillum |  |
| b49b904819d4d437e806d8fb7b96246a | Bacteria | p__Actinobacteriota | c__Actinobacteria | o__Propionibacteriales | f__Nocardioidaceae | g__Nocardioides |  |
| 540d36b00ff7a13da70f698a2df5f7ac | Bacteria | p__Proteobacteria | c__Gammaproteobacteria | o__Xanthomonadales | f__Xanthomonadaceae | g__Stenotrophomonas |  |
| 37850453b674de5b2256f1320e7fa8fc | Bacteria | p__Bacteroidota | c__Bacteroidia | o__Flavobacteriales | f__Flavobacteriaceae |  |  |
| G001428645_1 | Bacteria | p__Proteobacteria | c__Gammaproteobacteria | o__Burkholderiales | f__Comamonadaceae | g__Acidovorax |  |
| d90db3b6eb15c45e515b36953b10ef65 | Bacteria |  |  |  |  |  |  |
| MJ020-1-barcode31-umi117558bins-ubs-3 | Bacteria | p__Acidobacteriota | c__Blastocatellia | o__Pyrinomonadales | f__Pyrinomonadaceae | g__RB41 |  |
| d5416617145dc8133e0868ab13e6d911 | Bacteria | p__Firmicutes | c__Incertae_Sedis | o__DTU014 | f__DTU014 | g__DTU014 |  |
| ce2fcee25f374d0dfffea64e0f41a68d | Bacteria | p__Proteobacteria | c__Gammaproteobacteria | o__Xanthomonadales | f__Xanthomonadaceae | g__Pseudoxanthomonas |  |
| 905b00d9a75ea3843fe59c1db5550ee6 | Bacteria | p__Firmicutes | c__uncultured | o__uncultured | f__uncultured | g__uncultured | s__uncultured_bacterium |
| G000488255_8 | Bacteria | p__Proteobacteria | c__Gammaproteobacteria | o__Pseudomonadales | f__Moraxellaceae | g__Acinetobacter | s__Acinetobacter_indicus |
| 7e89c7836e977d84ac6b3097f3622d7f | Bacteria | p__Actinobacteriota | c__Thermoleophilia | o__Solirubrobacterales | f__Solirubrobacteraceae | g__Solirubrobacter |  |
| G000768675_1 | Bacteria | p__Actinobacteriota | c__Actinobacteria | o__Micrococcales | f__Intrasporangiaceae |  |  |
| 4cf69b5343716335d13b1cc138fc6a7e | Bacteria | p__Bacteroidota | c__Bacteroidia | o__Chitinophagales | f__Chitinophagaceae |  |  |
| 04912128 | Bacteria | p__Nitrospirota | c__Nitrospiria | o__Nitrospirales | f__Nitrospiraceae | g__Nitrospira | s__Nitrospira_japonica |
| e0504ae5440537b4b8b3f1b1dc15813f | Bacteria | p__Acidobacteriota | c__Vicinamibacteria | o__Vicinamibacterales | f__uncultured | g__uncultured |  |
| 04934726 | Bacteria | p__Bacteroidota | c__Bacteroidia | o__Flavobacteriales | f__Weeksellaceae | g__Empedobacter | s__Empedobacter_brevis |
| JN015213 | Bacteria | p__Bacteroidota | c__Bacteroidia | o__Sphingobacteriales | f__Sphingobacteriaceae | g__Sphingobacterium |  |
| c6353bd6b44f912e238e7dc12a47726e | Bacteria | p__Proteobacteria | c__Gammaproteobacteria | o__Burkholderiales | f__Rhodocyclaceae |  |  |
| 19345249 | Bacteria | p__Planctomycetota | c__Pla3_lineage | o__Pla3_lineage | f__Pla3_lineage | g__Pla3_lineage |  |
| 32eea72bc03d831fa74a47811d7569b9 | Bacteria | p__Proteobacteria | c__Gammaproteobacteria | o__Cellvibrionales | f__Cellvibrionaceae | g__Cellvibrio |  |
| 891333c4119fa041f696b7f8a442f8e3 | Bacteria | p__Bacteroidota | c__Bacteroidia | o__Sphingobacteriales | f__Sphingobacteriaceae | g__Sphingobacterium | s__Sphingobacterium_mizutaii |
| MJ031-2-barcode69-umi5848bins-ubs-70 | Bacteria | p__Bacteroidota | c__Bacteroidia | o__Sphingobacteriales | f__Sphingobacteriaceae | g__Sphingobacterium |  |
| 843599fa77faf436a1280127d1bc013f | Bacteria | p__Actinobacteriota | c__Actinobacteria | o__Micrococcales | f__Micrococcaceae |  |  |
| cdd6bc57a0b973cf5411141b69f8c5b9 | Bacteria | p__Proteobacteria | c__Gammaproteobacteria | o__Enterobacterales | f__Enterobacteriaceae |  |  |
| AF310217 | Bacteria | p__Proteobacteria | c__Gammaproteobacteria | o__Enterobacterales | f__Enterobacteriaceae |  |  |
| 17963823 | Bacteria | p__Chloroflexi | c__Ktedonobacteria | o__Ktedonobacterales | f__Ktedonobacteraceae | g__1921-3 | s__uncultured_Ktedonobacter |
| 04964376 | Bacteria | p__Firmicutes | c__Clostridia | o__Oscillospirales | f__Oscillospiraceae | g__Colidextribacter |  |
| 1862b05a2ac35f7e6a89ab6994e52c4e | Bacteria | p__Actinobacteriota | c__Actinobacteria | o__Micrococcales | f__Micrococcaceae |  |  |
| 103a8511398475e9b0579776cdc2fdc9 | Bacteria | p__Firmicutes | c__Bacilli | o__Lactobacillales | f__Lactobacillaceae | g__Lactobacillus |  |
| JX275858 | Bacteria | p__Proteobacteria | c__Gammaproteobacteria | o__Burkholderiales | f__Oxalobacteraceae | g__Noviherbaspirillum |  |
| 3e0454b0564d88735e691eb151b25a8b | Bacteria | p__Actinobacteriota | c__Actinobacteria | o__Frankiales | f__Geodermatophilaceae | g__Geodermatophilus |  |
| 47672a8f15437b6cbfb8ab8bec8fbbc8 | Bacteria | p__Firmicutes | c__Bacilli | o__Lactobacillales | f__Enterococcaceae | g__Enterococcus |  |
| 6b78eb5f32849bb4ecd53c3b6f503ca1 | Bacteria | p__Proteobacteria | c__Gammaproteobacteria | o__Enterobacterales | f__Enterobacteriaceae |  |  |
| 02247089 | Bacteria | p__Actinobacteriota | c__Actinobacteria | o__Propionibacteriales | f__Nocardioidaceae | g__Kribbella |  |
| 011d424859e268b3256dac3f654af544 | Bacteria | p__Proteobacteria | c__Alphaproteobacteria | o__Rhodospirillales | f__uncultured | g__uncultured |  |
| MJ010-2-barcode64-umi146967bins-ubs-3 | Bacteria | p__Bacteroidota | c__Bacteroidia | o__Flavobacteriales | f__Crocinitomicaceae | g__Fluviicola |  |
| 05035060 | Bacteria | p__Firmicutes | c__Bacilli | o__Erysipelotrichales | f__Erysipelotrichaceae | g__Ileibacterium | s__Ileibacterium_valens |
| d3b46c19697dd2591e307823d2259130 | Bacteria | p__Actinobacteriota | c__Actinobacteria | o__Micrococcales |  |  |  |
| c7c36293bcfae1d3129821c643b674c0 | Bacteria | p__Proteobacteria | c__Alphaproteobacteria | o__Rhizobiales | f__Rhizobiaceae |  |  |
| 0102871e5b96a7e8df808013d1c10207 | Bacteria | p__Firmicutes | c__Bacilli |  |  |  |  |
| a90f3d0fad560d943a69781a4ea8f257 | Bacteria | p__Acidobacteriota | c__Vicinamibacteria | o__Vicinamibacterales | f__uncultured | g__uncultured |  |
| KF934398 | Bacteria | p__Proteobacteria | c__Gammaproteobacteria | o__Xanthomonadales | f__Rhodanobacteraceae | g__Chujaibacter |  |
| 2d32be610c1c4c5333bb8d188c7edc41 | Bacteria | p__Myxococcota | c__Polyangia | o__Haliangiales | f__Haliangiaceae | g__Haliangium |  |
| 2792af052d2f51846959761102192539 | Bacteria | p__Cyanobacteria | c__Cyanobacteriia | o__Oxyphotobacteria_Incertae_Sedis | f__Unknown_Family | g__uncultured |  |
| 7e2f86a25120f59fe8b2ab367076bd0e | Bacteria | p__Proteobacteria | c__Gammaproteobacteria | o__Burkholderiales | f__SC-I-84 | g__SC-I-84 |  |
| 1d583483f131347e3e12d1178e98f648 | Bacteria | p__Proteobacteria | c__Gammaproteobacteria | o__Cellvibrionales | f__Spongiibacteraceae | g__BD1-7_clade | s__metagenome |
| AM230489 | Bacteria | p__Bacteroidota | c__Bacteroidia | o__Flavobacteriales | f__Flavobacteriaceae | g__Flavobacterium | s__Flavobacterium_johnsoniae |
| 04969424 | Bacteria | p__Firmicutes | c__Bacilli | o__Alicyclobacillales | f__Alicyclobacillaceae | g__Tumebacillus |  |
| MG852174 | Bacteria | p__Proteobacteria | c__Alphaproteobacteria | o__Rhizobiales | f__Devosiaceae | g__Devosia |  |
| 801146251ac2741a4c2507109ce92e2e | Bacteria |  |  |  |  |  |  |
| 2e343ac3ea087c5c883ab3c7e3d10a08 | Bacteria | p__Proteobacteria | c__Gammaproteobacteria |  |  |  |  |
| 5a9f1d285f8f753167a750f9851e6831 | Bacteria | p__Firmicutes | c__Bacilli | o__Erysipelotrichales | f__Erysipelotrichaceae | g__Turicibacter |  |
| 367e00e22320e3f23ead93f0a16f9ec7 | Bacteria | p__Bacteroidota | c__Bacteroidia | o__Bacteroidales | f__Muribaculaceae | g__Muribaculaceae | s__uncultured_bacterium |
| ade62f61d3fbbee435bb2a2571b08ae7 | Bacteria | p__Acidobacteriota | c__Holophagae | o__Holophagales | f__Holophagaceae |  |  |
| RS-GCF-000649515.1-NZ-KK308186.1 | Bacteria | p__Actinobacteriota | c__Actinobacteria | o__Corynebacteriales | f__Mycobacteriaceae | g__Mycobacterium |  |
| RS-GCF-015560775.1-NZ-JADMSM010000005.1 | Bacteria | p__Bacteroidota | c__Bacteroidia | o__Bacteroidales | f__Bacteroidaceae | g__Bacteroides | s__Bacteroides_eggerthii |
| 242d856eeb6582bd558aac3c199d814c | Bacteria | p__Proteobacteria | c__Gammaproteobacteria | o__Enterobacterales | f__Enterobacteriaceae |  |  |
| 05020583 | Bacteria | p__Firmicutes | c__Clostridia | o__Oscillospirales | f__Ruminococcaceae | g__Incertae_Sedis | s__uncultured_bacterium |
| dfaad37e302b901b468ccb6299cdefbf | Bacteria | p__Firmicutes | c__Bacilli | o__Lactobacillales | f__Lactobacillaceae | g__Lactobacillus |  |
| 56fa7b380b872f04eeaffb96d7ec395d | Bacteria | p__Bacteroidota | c__Bacteroidia | o__Bacteroidales | f__Bacteroidaceae | g__Bacteroides |  |
| e48fec3fdeb021759ae1538030f63e9c | Bacteria | p__Chloroflexi | c__Chloroflexia | o__Thermomicrobiales | f__AKYG1722 | g__AKYG1722 | s__uncultured_soil |
| MJ030-2-barcode74-umi98512bins-ubs-4 | Bacteria | p__Acidobacteriota | c__Acidobacteriae | o__Bryobacterales | f__Bryobacteraceae | g__Bryobacter |  |
| RS-GCF-003335165.1-NZ-CP031011.1 | Bacteria | p__Proteobacteria | c__Gammaproteobacteria | o__Pseudomonadales | f__Moraxellaceae | g__Acinetobacter |  |
| de350b1cafa42109b916117e2827a96c | Bacteria | p__Actinobacteriota | c__Thermoleophilia | o__Gaiellales | f__Gaiellaceae | g__Gaiella |  |
| G000576575_1 | Bacteria | p__Actinobacteriota | c__Actinobacteria | o__Micrococcales | f__Intrasporangiaceae |  |  |
| 02415739 | Bacteria | p__Proteobacteria | c__Alphaproteobacteria | o__Rhodobacterales | f__Rhodobacteraceae | g__Rubellimicrobium | s__uncultured_soil |
| 9ed1601b06a4261a0f054d68a8bd9557 | Bacteria | p__Proteobacteria | c__Gammaproteobacteria |  |  |  |  |
| 21199924 | Bacteria | p__Chloroflexi | c__Ktedonobacteria | o__C0119 | f__C0119 | g__C0119 | s__uncultured_bacterium |
| fb903feb1ad413e4b0082b5ba7ad4a70 | Bacteria | p__Actinobacteriota | c__Thermoleophilia | o__Solirubrobacterales | f__Solirubrobacteraceae | g__Conexibacter |  |
| 0a54ccc5583a490f5f9212ce62341444 | Bacteria | p__Chloroflexi | c__Anaerolineae | o__SBR1031 | f__A4b | g__A4b |  |
| MJ033-1-barcode36-umi41758bins-ubs-3 | Bacteria | p__Acidobacteriota | c__Blastocatellia | o__Pyrinomonadales | f__Pyrinomonadaceae | g__RB41 |  |
| 02360404 | Bacteria | p__Proteobacteria | c__Alphaproteobacteria | o__Rhizobiales | f__Rhizobiaceae |  |  |
| e0e8aacdda092f918dadc86d5929b9bb | Bacteria | p__Acidobacteriota | c__Acidobacteriae | o__Acidobacteriales | f__Acidobacteriales | g__Acidobacteriales | s__uncultured_bacterium |
| 1d824878fa765e6b4592039807a0bbcf | Bacteria | p__Actinobacteriota | c__MB-A2-108 | o__MB-A2-108 | f__MB-A2-108 | g__MB-A2-108 | s__uncultured_Actinomycetales |
| af666b82cbbaa6dfd35a2291b30a8e5f | Bacteria | p__MBNT15 | c__MBNT15 | o__MBNT15 | f__MBNT15 | g__MBNT15 |  |
| 0aca867c740b0d4389847b7cea2ad82b | Bacteria | p__Proteobacteria | c__Alphaproteobacteria | o__Rhizobiales | f__Rhizobiaceae | g__Aliihoeflea | s__uncultured_Aliihoeflea |
| 8fceb17032228e5e88e3b011d25ecd33 | Bacteria | p__Actinobacteriota | c__Thermoleophilia | o__Gaiellales |  |  |  |
| MJ030-1-barcode25-umi115336bins-ubs-3 | Bacteria | p__Actinobacteriota | c__Acidimicrobiia | o__Microtrichales | f__Ilumatobacteraceae | g__CL500-29_marine_group |  |
| DQ019167 | Bacteria | p__Firmicutes | c__Bacilli | o__Exiguobacterales | f__Exiguobacteraceae | g__Exiguobacterium |  |
| 04907717 | Bacteria | p__Proteobacteria | c__Alphaproteobacteria | o__Caulobacterales | f__Caulobacteraceae | g__Brevundimonas |  |
| 5fc4c2634a199067c1e468f8f6b60360 | Bacteria | p__Proteobacteria | c__Gammaproteobacteria | o__Burkholderiales | f__Comamonadaceae |  |  |
| 48086899e12d710d242d014dc1135759 | Bacteria | p__Acidobacteriota | c__Acidobacteriae | o__Subgroup_2 | f__Subgroup_2 | g__Subgroup_2 |  |
| f698a89d38cc430dccf9ef849d496011 | Bacteria | p__Proteobacteria | c__Gammaproteobacteria | o__Burkholderiales | f__Burkholderiaceae | g__Burkholderia-Caballeronia-Paraburkholderia |  |
| MJ030-2-barcode58-umi31509bins-ubs-22 | Bacteria | p__Firmicutes | c__Clostridia | o__Clostridiales | f__Clostridiaceae | g__Clostridium_sensu_stricto_8 | s__uncultured_bacterium |
| 1c2eab3bf8de367ad934dcbc0d5fb6b4 | Bacteria | p__Actinobacteriota | c__Actinobacteria |  |  |  |  |
| 05021561 | Bacteria | p__Actinobacteriota | c__Actinobacteria | o__Streptomycetales | f__Streptomycetaceae | g__Streptomyces |  |
| 34bdfce1a8c0ab1d0e38548da13035f4 | Bacteria | p__Firmicutes | c__Clostridia | o__Lachnospirales | f__Lachnospiraceae | g__Tyzzerella | s__uncultured_Clostridium |
| c13cc520a38958bc1a8bcc191f10722c | Bacteria | p__Proteobacteria | c__Gammaproteobacteria | o__Pseudomonadales | f__Pseudomonadaceae | g__Pseudomonas |  |
| AHAE01000085 | Bacteria | p__Actinobacteriota | c__Actinobacteria | o__Corynebacteriales | f__Corynebacteriaceae | g__Turicella |  |
| 1ee70f51d6b772d0b3f36cb665e54791 | Bacteria | p__Firmicutes | c__Clostridia | o__Lachnospirales | f__Lachnospiraceae |  |  |
| a6dfc4f7e65e21587efca107b3b8b33c | Bacteria | p__Firmicutes | c__Bacilli | o__Staphylococcales | f__Staphylococcaceae | g__Salinicoccus | s__Salinicoccus_albus |
| RS-GCF-013293385.1-NZ-JAAKAA010000059.1 | Bacteria | p__Firmicutes | c__Clostridia | o__Peptostreptococcales-Tissierellales | f__Peptostreptococcaceae | g__Romboutsia |  |
| 01102748 | Bacteria | p__Proteobacteria | c__Gammaproteobacteria | o__Pseudomonadales | f__Moraxellaceae | g__Acinetobacter |  |
| KC479803 | Bacteria | p__Proteobacteria | c__Alphaproteobacteria | o__Sphingomonadales | f__Sphingomonadaceae | g__Novosphingobium |  |
| 2e8441e95185109e0f4ae9537b794d53 | Bacteria | p__Proteobacteria | c__Gammaproteobacteria | o__Burkholderiales | f__SC-I-84 | g__SC-I-84 | s__uncultured_Nitrosomonadaceae |
| 7f9b4ef88b0cad0c5776e3c853ec105a | Bacteria | p__Proteobacteria | c__Alphaproteobacteria | o__Rhizobiales | f__Xanthobacteraceae |  |  |
| cbac16d5b8a3836ebca32432661fa475 | Bacteria | p__Bacteroidota | c__Bacteroidia | o__Flavobacteriales | f__Weeksellaceae | g__Chryseobacterium |  |
| G001476815_1 | Bacteria | p__Proteobacteria | c__Gammaproteobacteria | o__Burkholderiales | f__Comamonadaceae | g__Pseudacidovorax |  |
| 618cb68cc23698bfbaecd5f8f103375b | Bacteria | p__Firmicutes | c__Bacilli | o__Lactobacillales | f__Enterococcaceae | g__Enterococcus |  |
| 3ef52bd80d032d4508730bfa6bc79a14 | Bacteria | p__Actinobacteriota | c__Actinobacteria | o__Micrococcales | f__Intrasporangiaceae |  |  |
| 6e3e6ea27229207f28a5728b235f8655 | Bacteria | p__Actinobacteriota | c__Actinobacteria | o__Frankiales | f__Geodermatophilaceae | g__Geodermatophilus |  |
| 96666d4f5cf6258a3d41eaf933070505 | Bacteria | p__Bacteroidota | c__Ignavibacteria | o__Ignavibacteriales | f__PHOS-HE36 | g__PHOS-HE36 |  |
| c2bb998a25114bcd6c20f432f148e11b | Bacteria | p__Proteobacteria | c__Gammaproteobacteria | o__Enterobacterales | f__Enterobacteriaceae |  |  |
| 04903031 | Bacteria | p__Acidobacteriota | c__Vicinamibacteria | o__Vicinamibacterales | f__Vicinamibacteraceae | g__Vicinamibacteraceae |  |
| G001426485_1 | Bacteria | p__Actinobacteriota | c__Actinobacteria | o__Propionibacteriales | f__Nocardioidaceae | g__Aeromicrobium |  |
| HQ157187 | Bacteria | p__Actinobacteriota | c__Actinobacteria | o__Streptosporangiales | f__Thermomonosporaceae | g__Actinomadura |  |
| ac0f2dc664ed9d0d6cf06f35aab2a956 | Bacteria | p__Cyanobacteria | c__Cyanobacteriia | o__Leptolyngbyales | f__Leptolyngbyaceae | g__Leptolyngbya_Es-Yyy1000 | s__Leptolyngbya_cf. |
| 04879120 | Bacteria | p__Actinobacteriota | c__Actinobacteria | o__Propionibacteriales | f__Propionibacteriaceae | g__Cutibacterium |  |
| KF740333 | Bacteria | p__Proteobacteria | c__Gammaproteobacteria | o__Burkholderiales | f__Comamonadaceae |  |  |
| AB166881 | Bacteria | p__Proteobacteria | c__Alphaproteobacteria | o__Caulobacterales | f__Caulobacteraceae |  |  |
| 3132809b3261cc4f4e2a86786c87bcce | Bacteria | p__Actinobacteriota | c__Thermoleophilia | o__Solirubrobacterales | f__Solirubrobacteraceae | g__Solirubrobacter |  |
| fdbc0958ff3155649a3c51cf669b9b7b | Bacteria | p__Proteobacteria | c__Gammaproteobacteria | o__Alteromonadales | f__Alteromonadaceae | g__Rheinheimera |  |
| f017861e73d4d6356095b693174ea52a | Bacteria | p__Zixibacteria | c__Zixibacteria | o__Zixibacteria | f__Zixibacteria | g__Zixibacteria | s__uncultured_prokaryote |
| eb72bbf596b5d5945ab00d4e334777f9 | Bacteria | p__Proteobacteria | c__Gammaproteobacteria |  |  |  |  |
| bf5f645f2fc2527b22ef2348a88d3558 | Bacteria | p__Proteobacteria | c__Gammaproteobacteria | o__Burkholderiales |  |  |  |
| 00205121 | Bacteria | p__Chloroflexi | c__Anaerolineae | o__Anaerolineales | f__Anaerolineaceae | g__UTCFX1 |  |
| KT997475 | Bacteria | p__Proteobacteria | c__Gammaproteobacteria | o__Pseudomonadales | f__Moraxellaceae | g__Acinetobacter |  |
| 180281a3676a77238c3722adb1aef0b6 | Bacteria | p__Actinobacteriota | c__Actinobacteria | o__Micrococcales | f__Micrococcaceae |  |  |
| bcd44e3c555ebaff7cbd42d064cb65aa | Bacteria | p__Firmicutes | c__Clostridia | o__Clostridiales | f__Clostridiaceae | g__Clostridium_sensu_stricto_3 |  |
| MJ007-2-barcode74-umi33486bins-ubs-9 | Bacteria | p__Acidobacteriota | c__Acidobacteriae | o__Acidobacteriales |  |  |  |
| 7cd8732a54a703bece483124fe6adf12 | Bacteria | p__Proteobacteria | c__Gammaproteobacteria | o__Burkholderiales | f__Nitrosomonadaceae | g__mle1-7 |  |
| faf202378d35f9f59135df256b6f8ff8 | Bacteria | p__Chloroflexi | c__Ktedonobacteria | o__Ktedonobacterales | f__JG30-KF-AS9 | g__JG30-KF-AS9 | s__uncultured_bacterium |
| MJ007-2-barcode58-umi20777bins-ubs-16 | Bacteria | p__Proteobacteria | c__Gammaproteobacteria | o__Pseudomonadales | f__Moraxellaceae | g__Acinetobacter |  |
| c89d7d903f8eb29933396b665f43821d | Bacteria | p__Firmicutes | c__Bacilli | o__Acholeplasmatales | f__Acholeplasmataceae | g__Acholeplasma | s__uncultured_bacterium |
| MJ034-1-barcode48-umi5448bins-ubs-82 | Bacteria | p__Bacteroidota | c__Bacteroidia | o__Flavobacteriales | f__Flavobacteriaceae | g__Flavobacterium |  |
| RS-GCF-003950715.1-NZ-QLJK01000095.1 | Bacteria | p__Proteobacteria | c__Alphaproteobacteria | o__Sphingomonadales | f__Sphingomonadaceae |  |  |
| 5755589bcc1920af9b512c4059994f45 | Bacteria | p__Firmicutes | c__Negativicutes | o__Veillonellales-Selenomonadales | f__Veillonellaceae | g__Dialister | s__Dialister_succinatiphilus |
| 4cbdc69a0112eb4fd5036bc6cc9217ec | Bacteria | p__Proteobacteria | c__Alphaproteobacteria | o__Micropepsales | f__Micropepsaceae | g__uncultured |  |
| MJ030-2-barcode58-umi53282bins-ubs-13 | Bacteria | p__Proteobacteria | c__Gammaproteobacteria | o__Burkholderiales | f__Comamonadaceae |  |  |
| 5e01d04a94e45da47a85f22016a3d91c | Bacteria | p__Bacteroidota | c__Bacteroidia | o__Chitinophagales |  |  |  |
| e7ba92e5f8d41f9e9e80e90336dbeb3f | Bacteria |  |  |  |  |  |  |
| 1a9c2370edca38aeedb9ae0ffa96634c | Bacteria | p__Myxococcota | c__Polyangia | o__Polyangiales | f__Sandaracinaceae | g__uncultured |  |
| AJ243326 | Bacteria | p__Firmicutes | c__Bacilli | o__Lactobacillales | f__Vagococcaceae | g__Vagococcus |  |
| RS-GCF-013601105.1-NZ-JABFEE010000025.1 | Bacteria | p__Actinobacteriota | c__Actinobacteria | o__Corynebacteriales | f__Corynebacteriaceae | g__Corynebacterium |  |
| 202394ab2c247aea285c49f693769449 | Bacteria | p__Acidobacteriota | c__Acidobacteriae | o__Acidobacteriales | f__Acidobacteriaceae_(Subgroup_1) | g__Bryocella | s__uncultured_bacterium |
| a6851b1aa4ba39c2094432d69d355010 | Bacteria | p__Proteobacteria | c__Alphaproteobacteria | o__Acetobacterales | f__Acetobacteraceae |  |  |
| 8d7fbb1e0e6aa869a9cc841c4f2af9ba | Bacteria | p__Proteobacteria | c__Alphaproteobacteria | o__Rhizobiales | f__Xanthobacteraceae |  |  |
| 04946020 | Bacteria | p__Actinobacteriota | c__Actinobacteria | o__Streptomycetales | f__Streptomycetaceae | g__Streptomyces |  |
| 03176077 | Bacteria | p__Planctomycetota | c__Phycisphaerae | o__Tepidisphaerales | f__WD2101_soil_group | g__WD2101_soil_group | s__uncultured_planctomycete |
| MJ030-2-barcode58-umi76956bins-ubs-7 | Bacteria | p__Proteobacteria | c__Gammaproteobacteria | o__Burkholderiales | f__Methylophilaceae |  |  |
| MJ006-2-barcode62-umi175727bins-ubs-3 | Bacteria | p__Firmicutes | c__Clostridia | o__Oscillospirales | f__Oscillospiraceae | g__UCG-002 |  |
| 56c68e2b077b924a59cd6cb8b1099e62 | Bacteria | p__Proteobacteria | c__Gammaproteobacteria | o__Steroidobacterales | f__Steroidobacteraceae | g__uncultured |  |
| 04926113 | Bacteria | p__Proteobacteria | c__Alphaproteobacteria | o__Rhizobiales | f__Methyloligellaceae | g__uncultured |  |
| 14865087 | Bacteria | p__Chloroflexi | c__Ktedonobacteria | o__B12-WMSP1 | f__B12-WMSP1 | g__B12-WMSP1 | s__uncultured_Chloroflexi |
| KX815990 | Bacteria | p__Actinobacteriota | c__Actinobacteria | o__Propionibacteriales | f__Nocardioidaceae | g__Nocardioides |  |
| RS-GCF-003730795.1-NZ-MCKE01000014.1 | Bacteria | p__Proteobacteria | c__Gammaproteobacteria | o__Enterobacterales | f__Morganellaceae | g__Morganella |  |
| e0898e0f7d6c867599c52da5e70a3102 | Bacteria | p__Bdellovibrionota | c__Bdellovibrionia | o__Bacteriovoracales | f__Bacteriovoracaceae | g__Bacteriovorax |  |
| 04950185 | Bacteria | p__Firmicutes | c__Clostridia | o__Lachnospirales | f__Lachnospiraceae |  |  |
| 8ece3757bd6b36a9b7b0726cd4d551d5 | Bacteria | p__Patescibacteria | c__Saccharimonadia | o__Saccharimonadales | f__Saccharimonadales | g__Saccharimonadales |  |
| b1c58a6a9696bb763e684c8d4a5b1d39 | Bacteria | p__Bacteroidota | c__Bacteroidia | o__Bacteroidales | f__Bacteroidaceae | g__Bacteroides |  |
| 48a596693969ca9038d10db913a07611 | Bacteria | p__Firmicutes | c__Bacilli | o__Bacillales | f__Bacillaceae | g__Anoxybacillus |  |
| 9940b578e7de553a322a255c899b41a1 | Bacteria | p__Proteobacteria | c__Alphaproteobacteria | o__Sphingomonadales | f__Sphingomonadaceae |  |  |
| d22f4833127c6face4cb9aa909cd714d | Bacteria | p__Proteobacteria | c__Alphaproteobacteria | o__Sphingomonadales | f__Sphingomonadaceae |  |  |
| AY835924 | Bacteria | p__Actinobacteriota | c__Actinobacteria | o__Propionibacteriales | f__Nocardioidaceae | g__Nocardioides |  |
| MJ006-2-barcode69-umi51740bins-ubs-7 | Bacteria | p__Firmicutes | c__Negativicutes | o__Acidaminococcales | f__Acidaminococcaceae | g__Phascolarctobacterium |  |
| 248056698b5ef4cbd7749b8e23fc7480 | Bacteria | p__Gemmatimonadota | c__Gemmatimonadetes | o__Gemmatimonadales | f__Gemmatimonadaceae | g__uncultured |  |
| 8ec3ee1bd7cfbb35f3c988c9706dfd0e | Bacteria | p__Proteobacteria | c__Gammaproteobacteria | o__Enterobacterales | f__Enterobacteriaceae |  |  |
| b21d8456870789015b1ed024a8d65013 | Bacteria | p__Proteobacteria | c__Gammaproteobacteria | o__Burkholderiales | f__Comamonadaceae | g__Comamonas | s__Comamonas_aquatica |
| 4f266b78a0ba46b398e0368532e033dc | Bacteria | p__Actinobacteriota | c__Actinobacteria | o__Pseudonocardiales | f__Pseudonocardiaceae | g__Saccharopolyspora |  |
| f6ff7cf53b31c9907e11e5ccca9fb059 | Bacteria | p__Proteobacteria | c__Gammaproteobacteria | o__Burkholderiales | f__Comamonadaceae |  |  |
| 09993002 | Bacteria | p__Planctomycetota | c__Planctomycetes | o__Pirellulales | f__Pirellulaceae | g__Pirellula |  |
| d0dd2c749ceb0359e6ab3f39fb265ba5 | Bacteria | p__Proteobacteria | c__Alphaproteobacteria | o__Rhizobiales | f__Xanthobacteraceae |  |  |
| 04970919 | Bacteria | p__Firmicutes | c__Clostridia | o__Peptostreptococcales-Tissierellales | f__Peptostreptococcales-Tissierellales | g__Anaerococcus | s__Anaerococcus_vaginalis |
| 52e74e2709fcd93398549d8a7d6afc33 | Bacteria | p__Actinobacteriota | c__Thermoleophilia | o__Gaiellales |  |  |  |
| RS-GCF-900456855.1-NZ-UAUV01000012.1 | Bacteria | p__Proteobacteria | c__Gammaproteobacteria | o__Enterobacterales | f__Yersiniaceae | g__Serratia |  |
| 6357225f4791c118427829a1de9cba34 | Bacteria | p__Firmicutes | c__Bacilli | o__Lactobacillales | f__Enterococcaceae | g__Enterococcus |  |
| 014b1a4d72e1ba270a1b516c45d87e43 | Bacteria | p__Proteobacteria | c__Gammaproteobacteria | o__Pseudomonadales | f__Moraxellaceae | g__Acinetobacter |  |
| ae158dddbd5b3af9047b88bec7def975 | Bacteria | p__Actinobacteriota |  |  |  |  |  |
| 4a21f95e964fe25059ecf78772ce69b7 | Bacteria | p__Chloroflexi | c__Ktedonobacteria | o__Ktedonobacterales | f__Ktedonobacteraceae | g__uncultured | s__uncultured_Ktedobacteria |
| GB-GCA-000980335.1-LARC01000083.1 | Bacteria | p__Proteobacteria | c__Gammaproteobacteria | o__Burkholderiales | f__Sutterellaceae | g__Sutterella |  |
| 4323d32e5c7e3a7868642f6f18c3e771 | Bacteria | p__Bacteroidota | c__Bacteroidia | o__Bacteroidales | f__Bacteroidaceae | g__Bacteroides | s__uncultured_Bacteroidales |
| c4632f01b3c5d27818569bae9290274a | Bacteria | p__Actinobacteriota | c__Actinobacteria | o__Propionibacteriales | f__Propionibacteriaceae | g__Microlunatus |  |
| MJ006-1-barcode31-umi173022bins-ubs-3 | Bacteria | p__Firmicutes | c__Clostridia | o__Clostridia_UCG-014 | f__Clostridia_UCG-014 | g__Clostridia_UCG-014 |  |
| 07659409c39772fbc62872cbc27d184d | Bacteria | p__Proteobacteria | c__Gammaproteobacteria | o__Burkholderiales | f__Comamonadaceae |  |  |
| 8099596c59e745337fb5487dd2ec7f66 | Bacteria | p__Actinobacteriota | c__Thermoleophilia | o__Gaiellales | f__uncultured | g__uncultured | s__uncultured_Rubrobacterales |
| 04945862 | Bacteria | p__Actinobacteriota | c__Actinobacteria | o__Micrococcales | f__Micrococcaceae | g__Glutamicibacter |  |
| 4a4a21b6af2b08aeae21c4ea7a2da52c | Bacteria | p__Proteobacteria | c__Gammaproteobacteria | o__Pseudomonadales | f__Moraxellaceae | g__Acinetobacter |  |
| 04958072 | Bacteria | p__Actinobacteriota | c__Actinobacteria | o__Bifidobacteriales | f__Bifidobacteriaceae | g__Bifidobacterium |  |
| 1b81bbb99b373e797e8acf66592ef044 | Bacteria | p__Firmicutes | c__Clostridia | o__Peptostreptococcales-Tissierellales | f__Peptostreptococcaceae | g__Romboutsia |  |
| 768b2f431f2901dc7145c2575424547e | Bacteria | p__Actinobacteriota | c__Actinobacteria | o__Micrococcales | f__Promicromonosporaceae |  |  |
| 5b9d1ef4f599719e1e0db506d1b68e9d | Bacteria | p__Bacteroidota | c__Bacteroidia | o__Sphingobacteriales | f__AKYH767 | g__AKYH767 |  |
| e84159d9fd2cbc78019153209d631e1c | Bacteria | p__Methylomirabilota | c__Methylomirabilia | o__Rokubacteriales | f__Rokubacteriales | g__Rokubacteriales |  |
| 585b00d619301ad56bf11cacd5518482 | Bacteria | p__Proteobacteria | c__Gammaproteobacteria |  |  |  |  |
| 1dd522282cfb54691291de70b81a813f | Bacteria | p__Verrucomicrobiota | c__Verrucomicrobiae | o__Chthoniobacterales | f__Chthoniobacteraceae | g__Candidatus_Udaeobacter |  |
| 02397287 | Bacteria | p__Actinobacteriota | c__Acidimicrobiia | o__Microtrichales | f__Ilumatobacteraceae |  |  |
| 9c77fd520f95648596fbb3e2db4367a5 | Bacteria | p__Acidobacteriota | c__Holophagae | o__Subgroup_7 | f__Subgroup_7 | g__Subgroup_7 |  |
| cef17cab9fc876781976947962d55b46 | Bacteria | p__Firmicutes | c__Bacilli | o__Lactobacillales | f__Lactobacillaceae | g__Lactobacillus | s__Lactobacillus_rossiae |
| 05087246 | Bacteria | p__Actinobacteriota | c__Actinobacteria | o__Pseudonocardiales | f__Pseudonocardiaceae | g__Pseudonocardia |  |
| G000231445_1 | Bacteria | p__Proteobacteria | c__Alphaproteobacteria | o__Acetobacterales | f__Acetobacteraceae | g__Commensalibacter | s__Commensalibacter_intestini |
| 9322ef863a8915c1114aed3359f53ab0 | Bacteria | p__Bacteroidota | c__Bacteroidia | o__Bacteroidales | f__Muribaculaceae | g__Muribaculaceae |  |
| d8031b3c98f34e75da7d987854f89a8d | Bacteria | p__Chloroflexi | c__Anaerolineae | o__Ardenticatenales | f__uncultured | g__uncultured |  |
| MJ030-2-barcode65-umi49685bins-ubs-13 | Bacteria | p__Proteobacteria | c__Gammaproteobacteria | o__Burkholderiales | f__Burkholderiaceae | g__Burkholderia-Caballeronia-Paraburkholderia |  |
| 8e1d3d25c3930b9ae25b0c0114b65894 | Bacteria | p__Actinobacteriota | c__Thermoleophilia | o__Gaiellales | f__uncultured | g__uncultured |  |
| 6694f8972966fb09f1b89be77a281676 | Bacteria | p__Actinobacteriota | c__MB-A2-108 | o__MB-A2-108 | f__MB-A2-108 | g__MB-A2-108 | s__uncultured_Kineosporiaceae |
| fb65a5440560210d58ae35326d65edfd | Bacteria | p__Actinobacteriota | c__Actinobacteria | o__Frankiales | f__Sporichthyaceae | g__Longivirga |  |
| 19246353 | Bacteria | p__Actinobacteriota | c__Actinobacteria | o__Micrococcales | f__Intrasporangiaceae |  |  |
| d7965a9f14c94b42a7318c9ccbfa7b8b | Bacteria | p__Actinobacteriota | c__Coriobacteriia | o__Coriobacteriales | f__Atopobiaceae | g__Olsenella |  |
| 55deade633dd5c6ea100fb29b8597b93 | Bacteria | p__Bacteroidota | c__Bacteroidia | o__Bacteroidales | f__Muribaculaceae | g__Muribaculaceae |  |
| MJ033-1-barcode36-umi45321bins-ubs-3 | Bacteria | p__RCP2-54 | c__RCP2-54 | o__RCP2-54 | f__RCP2-54 | g__RCP2-54 |  |
| 05013063 | Bacteria | p__Bacteroidota | c__Bacteroidia | o__Chitinophagales | f__Chitinophagaceae | g__Parasegetibacter | s__Parasegetibacter_luojiensis |
| 075ccbda24140b1eb607e9c3218a1964 | Bacteria | p__Chloroflexi | c__Ktedonobacteria | o__Ktedonobacterales | f__Ktedonobacteraceae | g__Ktedonobacter | s__Ktedonobacterales_bacterium |
| 8da0930134618aae3fb6fb91de195b61 | Bacteria | p__Chloroflexi | c__Anaerolineae | o__SBR1031 | f__A4b | g__OLB13 |  |
| 05363865 | Bacteria | p__Bacteroidota | c__Bacteroidia | o__Bacteroidales | f__Tannerellaceae | g__Parabacteroides |  |
| a9bc3fee8cf6e50add76f4f5c5221f53 | Bacteria | p__Acidobacteriota | c__Acidobacteriae | o__Acidobacteriales |  |  |  |
| 60219663f194990c793a189b3e580dd8 | Bacteria | p__Firmicutes | c__Clostridia | o__Oscillospirales | f__[Eubacterium]_coprostanoligenes_group | g__[Eubacterium]_coprostanoligenes_group |  |
| f25fd1f5f06bb32ca1b2c891beeb63bd | Bacteria | p__Firmicutes | c__Bacilli | o__Lactobacillales | f__Aerococcaceae | g__Dolosicoccus | s__uncultured_bacterium |
| 9356026287608e7c0b090a078c05de59 | Bacteria | p__Proteobacteria | c__Gammaproteobacteria | o__Pseudomonadales | f__Moraxellaceae | g__Acinetobacter |  |
| 04970113 | Bacteria | p__Chloroflexi | c__KD4-96 | o__KD4-96 | f__KD4-96 | g__KD4-96 |  |
| a53a92c3f3314a2c01d53eecce4713fa | Bacteria | p__Actinobacteriota | c__Coriobacteriia | o__Coriobacteriales | f__Atopobiaceae |  |  |
| 27ccfa1932799e21dc637a4a190d5922 | Bacteria | p__Proteobacteria | c__Alphaproteobacteria | o__Acetobacterales | f__Acetobacteraceae | g__Roseomonas |  |
| d0acf940a8b69908095f60d56052407f | Bacteria | p__Firmicutes | c__Bacilli | o__Bacillales | f__Bacillaceae |  |  |
| 04666401 | Bacteria | p__Actinobacteriota | c__Actinobacteria | o__Micrococcales | f__Micrococcaceae | g__Rothia |  |
| AF011347 | Bacteria | p__Proteobacteria | c__Gammaproteobacteria | o__Burkholderiales | f__Rhodocyclaceae | g__Azospira |  |
| 7e85968252e83eb01e168675e9d74250 | Bacteria | p__Proteobacteria | c__Gammaproteobacteria | o__Burkholderiales | f__Burkholderiaceae | g__Burkholderia-Caballeronia-Paraburkholderia |  |
| e0778deebe8b13c145bd87523742eeb2 | Bacteria | p__Planctomycetota | c__Planctomycetes | o__Gemmatales | f__Gemmataceae | g__uncultured |  |
| 3e9e25ab93a3684435fc9ea6a447819b | Bacteria | p__Verrucomicrobiota | c__Verrucomicrobiae | o__Chthoniobacterales | f__Chthoniobacteraceae | g__Chthoniobacter | s__uncultured_bacterium |
| GU434268 | Bacteria | p__Actinobacteriota | c__Actinobacteria | o__Propionibacteriales | f__Nocardioidaceae | g__Kribbella |  |
| MJ007-1-barcode38-umi8970bins-ubs-32 | Bacteria | p__Proteobacteria | c__Gammaproteobacteria | o__Pseudomonadales | f__Moraxellaceae | g__Acinetobacter |  |
| JQ046313 | Bacteria | p__Proteobacteria | c__Alphaproteobacteria | o__Sphingomonadales | f__Sphingomonadaceae | g__Sphingobium |  |
| 04895942 | Bacteria | p__Cyanobacteria | c__Cyanobacteriia | o__Cyanobacteriales | f__Coleofasciculaceae | g__uncultured | s__uncultured_bacterium |
| 05127277 | Bacteria | p__Bacteroidota | c__Bacteroidia | o__Flavobacteriales | f__Weeksellaceae | g__Chryseobacterium |  |
| 5e06d0983609e9649346a90daa8bbe4b | Bacteria | p__Bacteroidota | c__Bacteroidia | o__Sphingobacteriales | f__Sphingobacteriaceae | g__Sphingobacterium |  |
| RS-GCF-900115115.1-NZ-FOVI01000068.1 | Bacteria | p__Bacteroidota | c__Bacteroidia | o__Flavobacteriales | f__Flavobacteriaceae | g__Flavobacterium | s__Flavobacterium_ummariense |
| a08f2d9f6cbeecd07c865d81e4b6d5ca | Bacteria | p__Bacteroidota | c__Bacteroidia | o__Sphingobacteriales | f__Sphingobacteriaceae |  |  |
| 7708cd6e4ea22e26c7f7655fcfe21e6a | Bacteria | p__Chloroflexi | c__Anaerolineae | o__Caldilineales | f__Caldilineaceae | g__uncultured | s__uncultured_bacterium |
| 04926885 | Bacteria | p__Acidobacteriota | c__Thermoanaerobaculia | o__Thermoanaerobaculales | f__Thermoanaerobaculaceae | g__Subgroup_10 |  |
| 493019d8c8e41ffb36baf3b9265a844b | Bacteria | p__Bacteroidota | c__Bacteroidia | o__Chitinophagales | f__Chitinophagaceae |  |  |
| 14952061 | Bacteria |  |  |  |  |  |  |
| MJ030-2-barcode65-umi9701bins-ubs-48 | Bacteria | p__Firmicutes | c__Clostridia | o__Lachnospirales | f__Lachnospiraceae | g__Tyzzerella | s__uncultured_Clostridium |
| MJ034-1-barcode34-umi1143bins-ubs-337 | Bacteria | p__Proteobacteria | c__Gammaproteobacteria | o__Pseudomonadales | f__Moraxellaceae | g__Acinetobacter |  |
| 14523466 | Bacteria | p__Gemmatimonadota | c__Gemmatimonadetes | o__Gemmatimonadales | f__Gemmatimonadaceae | g__uncultured |  |
| 04906719 | Bacteria | p__Proteobacteria | c__Alphaproteobacteria | o__Rhizobiales | f__Beijerinckiaceae | g__Methylobacterium-Methylorubrum |  |
| 05026069 | Bacteria | p__Actinobacteriota | c__Thermoleophilia | o__Solirubrobacterales | f__67-14 | g__67-14 | s__uncultured_Solirubrobacter |
| 628e6440bc3e2129196f4457ce29ca37 | Bacteria | p__Bacteroidota | c__Bacteroidia | o__Bacteroidales | f__Rikenellaceae | g__Rikenellaceae_RC9_gut_group | s__uncultured_organism |
| fde07b264c1e6f5e8622276e26f0ffb0 | Bacteria | p__Firmicutes | c__Clostridia | o__Clostridiales | f__Clostridiaceae | g__Clostridium_sensu_stricto_11 | s__Clostridium_sp. |
| RS-GCF-900168125.1-NZ-FUZF01000038.1 | Bacteria | p__Bacteroidota | c__Bacteroidia | o__Sphingobacteriales | f__Sphingobacteriaceae | g__Sphingobacterium | s__Sphingobacterium_nematocida |
| 720cf92446a6457dd87d85b5d204439e | Bacteria | p__Actinobacteriota | c__Actinobacteria | o__Streptomycetales | f__Streptomycetaceae | g__Streptomyces |  |
| 20f8aa73d01d1369d04d4b384c464f20 | Bacteria | p__Acidobacteriota | c__Blastocatellia | o__DS-100 | f__DS-100 | g__DS-100 | s__uncultured_bacterium |
| d742bd2477d6f3b0527a29e9eddf462a | Bacteria | p__Proteobacteria | c__Alphaproteobacteria | o__Sphingomonadales | f__Sphingomonadaceae | g__Sphingomonas |  |
| 3822ee2c2b20716a576520628f216e2f | Bacteria | p__Proteobacteria | c__Alphaproteobacteria | o__Sphingomonadales | f__Sphingomonadaceae | g__Sphingomonas |  |
| c5c5cbea711e3bdd1785e5dac72c64f5 | Bacteria | p__Bacteroidota | c__Bacteroidia | o__Chitinophagales | f__Chitinophagaceae | g__Segetibacter |  |
| MJ034-2-barcode60-umi17183bins-ubs-5 | Bacteria | p__Proteobacteria | c__Gammaproteobacteria | o__Burkholderiales | f__Comamonadaceae | g__Variovorax |  |
| c9290f1633a32fb8ccbdfc5033cbacea | Bacteria | p__Patescibacteria | c__Saccharimonadia | o__Saccharimonadales | f__Saccharimonadales | g__Saccharimonadales |  |
| 3e9abda274beaaca2592427032dd6923 | Bacteria | p__Actinobacteriota | c__Thermoleophilia | o__Solirubrobacterales | f__67-14 | g__67-14 |  |
| e74b9abf8dbfe1295f53c58f9e753c0f | Bacteria | p__Proteobacteria | c__Gammaproteobacteria | o__Burkholderiales | f__Comamonadaceae |  |  |
| ACHJ01000075 | Bacteria | p__Actinobacteriota | c__Actinobacteria | o__Corynebacteriales | f__Corynebacteriaceae | g__Corynebacterium | s__Corynebacterium_lipophiloflavum |
| 04961803 | Bacteria | p__Actinobacteriota | c__Actinobacteria | o__Micrococcales | f__Intrasporangiaceae |  |  |
| d84081ad232e05bafd3b46bea046c673 | Bacteria | p__Proteobacteria | c__Gammaproteobacteria | o__Enterobacterales | f__Morganellaceae | g__Providencia |  |
| 04946635 | Bacteria | p__Actinobacteriota | c__Thermoleophilia | o__Solirubrobacterales | f__Solirubrobacteraceae | g__Patulibacter |  |
| KC759435 | Bacteria | p__Bacteroidota | c__Bacteroidia | o__Chitinophagales | f__Chitinophagaceae | g__Lacibacter | s__Lacibacter_cauensis |
| 279ed49380d097928d8910e88e55c9e7 | Bacteria | p__Proteobacteria | c__Alphaproteobacteria |  |  |  |  |
| da56c42df1c2b1702df18fcf0d36a636 | Bacteria | p__Verrucomicrobiota | c__Verrucomicrobiae | o__Pedosphaerales | f__Pedosphaeraceae | g__Ellin516 | s__uncultured_Verrucomicrobia |
| 2000e0958b7900b0a1d388a4c34a28e0 | Bacteria | p__Actinobacteriota | c__Actinobacteria | o__Corynebacteriales | f__Corynebacteriaceae | g__Corynebacterium |  |
| 04927950 | Bacteria | p__Proteobacteria | c__Alphaproteobacteria | o__Sphingomonadales | f__Sphingomonadaceae | g__Sphingomonas | s__Sphingomonas_daechungensis |
| f5a5205cb9feb27efefcd676ac7377e8 | Bacteria | p__Deinococcota | c__Deinococci | o__Deinococcales | f__Trueperaceae | g__Truepera |  |
| cfe494081510045d4d0d1433ad88f9dd | Bacteria | p__Acidobacteriota | c__Acidobacteriae |  |  |  |  |
| eedf66263aea6a46211a672ef82559dd | Bacteria | p__Acidobacteriota | c__Vicinamibacteria | o__Vicinamibacterales | f__uncultured | g__uncultured |  |
| 9bd7fb8d3c12e1f8d8cfaa44167c8c3b | Bacteria | p__Actinobacteriota | c__Actinobacteria | o__Corynebacteriales | f__Corynebacteriaceae | g__Corynebacterium |  |
| RS-GCF-017163455.1-NZ-JAFJXK010000310.1 | Bacteria | p__Proteobacteria | c__Gammaproteobacteria | o__Enterobacterales | f__Morganellaceae | g__Providencia |  |
| b0e357bd106e1683fef27337fa31155b | Bacteria | p__Proteobacteria | c__Alphaproteobacteria | o__Sphingomonadales | f__Sphingomonadaceae | g__Sphingobium |  |
| DQ672568 | Bacteria | p__Proteobacteria | c__Alphaproteobacteria | o__Azospirillales | f__Azospirillaceae | g__Skermanella |  |
| 04900387 | Bacteria | p__Proteobacteria | c__Gammaproteobacteria | o__Pseudomonadales | f__Pseudomonadaceae | g__Pseudomonas |  |
| 11077010 | Bacteria | p__Bacteroidota | c__Bacteroidia | o__Bacteroidales | f__Muribaculaceae | g__Muribaculaceae | s__uncultured_bacterium |
| LT009500 | Bacteria | p__Proteobacteria | c__Gammaproteobacteria | o__Burkholderiales | f__Comamonadaceae | g__Comamonas | s__Comamonas_sediminis |
| 256231fa9644bc30a1baafa9322c192b | Bacteria | p__Proteobacteria | c__Alphaproteobacteria | o__Rhodobacterales | f__Rhodobacteraceae | g__Paracoccus |  |
| MJ010-1-barcode26-umi133140bins-ubs-4 | Bacteria | p__Bacteroidota | c__Bacteroidia | o__Chitinophagales | f__Chitinophagaceae |  |  |
| e2632f5eb77bdcca906c7f9f7362a4ae | Bacteria | p__Proteobacteria | c__Gammaproteobacteria | o__Legionellales | f__Legionellaceae | g__Legionella |  |
| 1624533323118936ce29e915ec5917b4 | Bacteria | p__Actinobacteriota | c__Thermoleophilia | o__Gaiellales |  |  |  |
| 64a02c9a1c572f44c8ee87fb181dcf84 | Bacteria | p__Bacteroidota | c__Bacteroidia | o__Bacteroidales | f__Muribaculaceae | g__Muribaculaceae | s__uncultured_bacterium |
| b42423165f490c3a220e6a3d81b5edbf | Bacteria | p__Proteobacteria | c__Gammaproteobacteria | o__Enterobacterales |  |  |  |
| RS-GCF-003144475.1-NZ-PGWU01000028.1 | Bacteria | p__Proteobacteria | c__Gammaproteobacteria | o__Enterobacterales | f__Morganellaceae | g__Proteus |  |
| 86114e8a56f4160f697d11c22657341b | Bacteria | p__Firmicutes | c__Bacilli | o__Lactobacillales | f__Enterococcaceae | g__Enterococcus |  |
| 04966254 | Bacteria | p__Firmicutes | c__Bacilli | o__Bacillales | f__Bacillaceae | g__Bacillus |  |
| 16259098 | Bacteria | p__Firmicutes | c__Clostridia | o__Lachnospirales | f__Lachnospiraceae | g__Lachnospiraceae_NK4A136_group |  |
| d34f6b6939c14ee37719cba60056f24b | Bacteria | p__Bacteroidota | c__Bacteroidia | o__Bacteroidales | f__Bacteroidaceae | g__Bacteroides | s__Bacteroides_graminisolvens |
| 02244268 | Bacteria | p__Actinobacteriota | c__Actinobacteria | o__Propionibacteriales | f__Propionibacteriaceae | g__Cutibacterium | s__Cutibacterium_granulosum |
| c742de8b1f0045d232f529bfa0f3ad71 | Bacteria | p__Actinobacteriota | c__Actinobacteria | o__Micrococcales | f__Microbacteriaceae | g__Microbacterium |  |
| 05032095 | Bacteria | p__Proteobacteria | c__Gammaproteobacteria | o__Burkholderiales | f__Comamonadaceae |  |  |
| 7c7bcf1f578edae04f2fabb6deeccbb1 | Bacteria | p__Chloroflexi | c__Anaerolineae | o__RBG-13-54-9 | f__RBG-13-54-9 | g__RBG-13-54-9 | s__metagenome |
| 1b1f5df345521177473910bae334f2df | Bacteria | p__Actinobacteriota | c__Actinobacteria | o__Propionibacteriales | f__Propionibacteriaceae | g__uncultured |  |
| a6ebaa6e4d7d9631c05c45dce5c76702 | Bacteria | p__Actinobacteriota | c__Actinobacteria | o__Micrococcales | f__Micrococcales_Incertae_Sedis | g__Timonella | s__uncultured_bacterium |
| 07450834 | Bacteria | p__Firmicutes | c__Clostridia | o__Clostridia_UCG-014 | f__Clostridia_UCG-014 | g__Clostridia_UCG-014 | s__unidentified |
| 3fda675f8608181a9cb8cd2b3bcddaf9 | Bacteria | p__Actinobacteriota | c__Actinobacteria | o__Frankiales | f__Frankiaceae | g__Jatrophihabitans |  |
| ac3fa1e9e26a504cb42cc71da872cb56 | Bacteria | p__Acidobacteriota | c__Vicinamibacteria | o__Vicinamibacterales | f__Vicinamibacteraceae |  |  |
| d101f22a06f1aa78888bb96b366e1084 | Bacteria | p__Firmicutes | c__Clostridia | o__Lachnospirales | f__Lachnospiraceae | g__Tyzzerella | s__uncultured_Clostridium |
| e4c5f916fe3df336673030687e683d4c | Bacteria | p__Firmicutes | c__Clostridia | o__Oscillospirales | f__Oscillospiraceae | g__Oscillibacter |  |
| d59fc6f9a05004c8a5a32bd64e80156d | Bacteria | p__Acidobacteriota | c__Acidobacteriae | o__Acidobacteriales | f__Acidobacteriaceae_(Subgroup_1) | g__uncultured | s__Acidobacteria_bacterium |
| 05001554 | Bacteria | p__Bacteroidota | c__Bacteroidia | o__Flavobacteriales | f__Flavobacteriaceae | g__Flavobacterium | s__Flavobacterium_ummariense |
| 2c939d8abe321925be9e3ada2c6fbcf3 | Bacteria | p__Chloroflexi | c__Anaerolineae | o__Caldilineales | f__Caldilineaceae | g__uncultured | s__metagenome |
| MJ034-1-barcode31-umi13475bins-ubs-25 | Bacteria | p__Acidobacteriota | c__Acidobacteriae | o__Acidobacteriales | f__uncultured | g__uncultured |  |
| 5c67f61786e20906ab4d755cd492a7f4 | Bacteria | p__Actinobacteriota | c__Thermoleophilia | o__Gaiellales | f__uncultured | g__uncultured |  |
| 94b896966fe6e6d332ea28ccc8ec4d3b | Bacteria | p__Actinobacteriota | c__Acidimicrobiia | o__IMCC26256 | f__IMCC26256 | g__IMCC26256 |  |
| RS-GCF-001054935.1-NZ-JVKR01000199.1 | Bacteria | p__Proteobacteria | c__Gammaproteobacteria | o__Enterobacterales |  |  |  |
| 8b384eb55bac4d790b066a9cd854bf2c | Bacteria | p__Chloroflexi | c__KD4-96 | o__KD4-96 | f__KD4-96 | g__KD4-96 | s__metagenome |
| 02244161 | Bacteria | p__Actinobacteriota | c__Actinobacteria | o__Micrococcales | f__Dermabacteraceae | g__Brachybacterium |  |
| 1b0e53117ab86a1ef762fd15f6a555d5 | Bacteria | p__Firmicutes | c__Clostridia | o__Oscillospirales | f__Ruminococcaceae | g__Subdoligranulum |  |
| 4e59cc8245ac2f6679425113aee385eb | Bacteria | p__Cyanobacteria | c__Cyanobacteriia | o__Cyanobacteriales | f__Coleofasciculaceae |  |  |
| 337d67a44d68bb4d7a956a9dbe66cca5 | Bacteria | p__Actinobacteriota | c__Actinobacteria | o__Micrococcales | f__Microbacteriaceae |  |  |
| c40b7ebc1113897b1cf0f02f493a7fe6 | Bacteria | p__Actinobacteriota | c__Rubrobacteria | o__Rubrobacterales | f__Rubrobacteriaceae | g__Rubrobacter | s__Rubrobacter_bracarensis |
| e1d1c044d8b10b521433b9b58dc2133d | Bacteria | p__Acidobacteriota | c__Acidobacteriae | o__Acidobacteriales | f__Acidobacteriaceae_(Subgroup_1) |  |  |
| 5e1c13a3498d9a13d3adb0fdcf2106d8 | Bacteria | p__Proteobacteria | c__Gammaproteobacteria | o__Pseudomonadales | f__Moraxellaceae | g__Acinetobacter |  |
| 8d8ab9f59ac8524ca9ffcb241a55f0fe | Bacteria | p__Firmicutes | c__Bacilli | o__Lactobacillales | f__Streptococcaceae | g__Lactococcus |  |
| fbfe8839ad93dfa6ffc11d92961f8698 | Bacteria | p__Bacteroidota | c__Bacteroidia | o__Bacteroidales | f__Bacteroidaceae | g__Bacteroides |  |
| 7c53410e96bcf35bb32c098d3de7d094 | Bacteria | p__Firmicutes | c__Bacilli | o__Lactobacillales | f__Enterococcaceae | g__Enterococcus |  |
| 0a3a9cf9af4a432badcd13f1244255af | Bacteria | p__Acidobacteriota | c__Vicinamibacteria | o__Vicinamibacterales | f__uncultured | g__uncultured |  |
| 11837702 | Bacteria | p__Proteobacteria | c__Alphaproteobacteria | o__Sphingomonadales | f__Sphingomonadaceae | g__Sphingoaurantiacus |  |
| b849216a7ff33e271bcc2142c6c47836 | Bacteria | p__Proteobacteria | c__Gammaproteobacteria | o__Burkholderiales | f__Comamonadaceae |  |  |
| G000964095_1 | Bacteria | p__Proteobacteria | c__Gammaproteobacteria | o__Pseudomonadales | f__Moraxellaceae | g__Acinetobacter | s__Acinetobacter_indicus |
| 04957449 | Bacteria | p__Proteobacteria | c__Gammaproteobacteria | o__Burkholderiales | f__Alcaligenaceae |  |  |
| f5494cb240bd221d49b70b75125ae66c | Bacteria | p__Chloroflexi | c__Anaerolineae | o__Caldilineales | f__Caldilineaceae | g__uncultured | s__metagenome |
| MJ031-1-barcode35-umi10775bins-ubs-53 | Bacteria | p__Proteobacteria | c__Gammaproteobacteria | o__Pseudomonadales | f__Moraxellaceae | g__Acinetobacter |  |
| f2e1f846c4db145cdb60cbcb8860ded6 | Bacteria | p__Actinobacteriota | c__Thermoleophilia | o__Solirubrobacterales | f__Solirubrobacteraceae |  |  |
| 7ee4890cafc8c4958de1d8fe1386a5f7 | Bacteria | p__Firmicutes | c__Clostridia | o__Peptostreptococcales-Tissierellales | f__Peptostreptococcales-Tissierellales | g__Anaerococcus | s__Anaerococcus_octavius |
| 05032190 | Bacteria | p__Proteobacteria | c__Gammaproteobacteria | o__Burkholderiales | f__Burkholderiaceae | g__Burkholderia-Caballeronia-Paraburkholderia |  |
| bddb6002877c2fd761dedf26a2a0b400 | Bacteria | p__Proteobacteria | c__Gammaproteobacteria | o__Burkholderiales | f__Nitrosomonadaceae | g__MND1 |  |
| a185682bebe06e87aac7062b1bde11b0 | Bacteria | p__Actinobacteriota | c__Actinobacteria | o__Frankiales | f__Geodermatophilaceae | g__Blastococcus |  |
| KF682221 | Bacteria | p__Actinobacteriota | c__Actinobacteria | o__Streptomycetales | f__Streptomycetaceae | g__Streptomyces |  |
| 834d36c410d4faa9ea8f745cecae3d2c | Bacteria | p__Chloroflexi | c__KD4-96 | o__KD4-96 | f__KD4-96 | g__KD4-96 |  |
| JQ032629 | Bacteria | p__Proteobacteria | c__Alphaproteobacteria | o__Rhizobiales | f__Rhizobiaceae | g__Allorhizobium-Neorhizobium-Pararhizobium-Rhizobium |  |
| 67f8a90e6031ecf1054694627e0a783e | Bacteria | p__Firmicutes | c__Bacilli | o__Lactobacillales | f__Enterococcaceae | g__Enterococcus |  |
| 05112052 | Bacteria | p__Firmicutes | c__Bacilli | o__Bacillales | f__Bacillaceae | g__Bacillus |  |
| 04957507 | Bacteria | p__Proteobacteria | c__Gammaproteobacteria | o__Burkholderiales | f__Oxalobacteraceae |  |  |
| 07f72dd0800ff962cc89430371e49fdc | Bacteria | p__Firmicutes | c__Clostridia | o__Clostridia_UCG-014 | f__Clostridia_UCG-014 | g__Clostridia_UCG-014 |  |
| 198bd7ea42abe679557498383358e62e | Bacteria | p__Proteobacteria | c__Alphaproteobacteria | o__Sphingomonadales | f__Sphingomonadaceae | g__Sphingomonas |  |
| 445f2c97facd2149eb2f34f6090fa77c | Bacteria | p__Proteobacteria | c__Gammaproteobacteria | o__Oceanospirillales | f__Halomonadaceae | g__Halomonas |  |
| 1f2df6efbc79f9887c4e023cf544af65 | Bacteria | p__Bdellovibrionota | c__Bdellovibrionia | o__Bdellovibrionales | f__Bdellovibrionaceae | g__Bdellovibrio |  |
| RS-GCF-000957415.1-NZ-LABD01000004.1 | Bacteria | p__Proteobacteria | c__Gammaproteobacteria | o__Enterobacterales | f__Enterobacteriaceae |  |  |
| 73edd4a3a6aed775e6a3461fced04de6 | Bacteria | p__Proteobacteria | c__Alphaproteobacteria | o__Rhizobiales | f__Devosiaceae | g__Devosia |  |
| 2b37d6b5adb6238734c6cf93c0257ede | Bacteria | p__Proteobacteria | c__Alphaproteobacteria | o__Rhizobiales | f__KF-JG30-B3 | g__KF-JG30-B3 |  |
| bbe5981ea1d5900fabad6346ccb913a8 | Bacteria | p__Actinobacteriota | c__Actinobacteria | o__Bifidobacteriales | f__Bifidobacteriaceae | g__Bifidobacterium | s__Bifidobacterium_bifidum |
| 871e024238bd15321a103ce485ed7aa9 | Bacteria | p__Verrucomicrobiota | c__Verrucomicrobiae | o__Verrucomicrobiales | f__Akkermansiaceae | g__Akkermansia |  |
| a61a00760744bdd0a375de83db23acf7 | Bacteria | p__Proteobacteria | c__Gammaproteobacteria | o__Pseudomonadales | f__Moraxellaceae | g__Acinetobacter |  |
| ab9c6039a3021c074a417ab448b14527 | Bacteria | p__Acidobacteriota | c__Blastocatellia | o__11-24 | f__11-24 | g__11-24 |  |
| 119d64ce9b64f6fc5fd879aabfdfc458 | Bacteria | p__Proteobacteria | c__Gammaproteobacteria | o__Pseudomonadales | f__Moraxellaceae | g__Acinetobacter |  |
| 8c7ab3659f8cfa319fa54579b4ebb60d | Bacteria | p__Actinobacteriota | c__Actinobacteria | o__Micrococcales | f__Microbacteriaceae |  |  |
| G900289045_1 | Bacteria | p__Actinobacteriota | c__Actinobacteria | o__Propionibacteriales | f__Propionibacteriaceae | g__Micropruina |  |
| c045ca96b9afd033ef097565e158ab46 | Bacteria | p__Proteobacteria | c__Alphaproteobacteria | o__Rhizobiales | f__uncultured | g__uncultured |  |
| e4102ad3cf2f7bf8339bcc0345fefd73 | Bacteria | p__Bacteroidota | c__Bacteroidia | o__Chitinophagales | f__Saprospiraceae | g__Lewinella |  |
| f878269c2ee5d63e170166388b20589a | Bacteria | p__Actinobacteriota | c__Acidimicrobiia |  |  |  |  |
| GB-GCA-902166655.1-LR607355.1 | Bacteria | p__Firmicutes | c__Bacilli | o__Lactobacillales | f__Enterococcaceae | g__Enterococcus |  |
| 17499022 | Bacteria | p__Chloroflexi |  |  |  |  |  |
| 0e20b89beeb185e6f132fc46fe3a8003 | Bacteria | p__Proteobacteria | c__Alphaproteobacteria | o__Rhizobiales |  |  |  |
| 2345da3d74506da97ed7bff9c33f2998 | Bacteria | p__Firmicutes | c__Bacilli | o__Lactobacillales | f__Lactobacillaceae | g__Lactobacillus | s__Lactobacillus_iners |
| ed7b6e77f691b7c4ad82ae79f1e65563 | Bacteria | p__Firmicutes | c__Bacilli | o__Erysipelotrichales | f__Erysipelotrichaceae | g__Faecalibaculum | s__uncultured_bacterium |
| 20588692a4cba6fa4fe38938fd527bc1 | Bacteria | p__Bacteroidota | c__Bacteroidia | o__Cytophagales | f__Hymenobacteraceae | g__Hymenobacter |  |
| KC569790 | Bacteria | p__Bacteroidota | c__Bacteroidia | o__Chitinophagales | f__Chitinophagaceae | g__Flavihumibacter |  |
| b85a4a75fb08af7b44b8c879c1f30b06 | Bacteria | p__Proteobacteria | c__Gammaproteobacteria | o__Alteromonadales | f__Alteromonadaceae | g__Rheinheimera |  |
| 49e06164d1211df504ae7acc260fa109 | Bacteria | p__Bacteroidota | c__Bacteroidia | o__Bacteroidales | f__Muribaculaceae | g__Muribaculaceae |  |
| 02267156 | Bacteria | p__Actinobacteriota | c__Actinobacteria | o__Propionibacteriales | f__Nocardioidaceae | g__Nocardioides |  |
| d54e227d24a1bb8c538b443fed9a3ba9 | Bacteria | p__Actinobacteriota | c__Acidimicrobiia | o__Microtrichales |  |  |  |
| EF373540 | Bacteria | p__Proteobacteria | c__Alphaproteobacteria | o__Rhizobiales | f__Rhizobiaceae | g__Aureimonas | s__Aureimonas_frigidaquae |
| 04912135 | Bacteria | p__Nitrospirota | c__Nitrospiria | o__Nitrospirales | f__Nitrospiraceae | g__Nitrospira |  |
| 04907450 | Bacteria | p__Proteobacteria | c__Alphaproteobacteria | o__Caulobacterales | f__Caulobacteraceae | g__Brevundimonas | s__Brevundimonas_bacteroides |
| e014a042552b1778c5c62a2c22fc7310 | Bacteria | p__Methylomirabilota | c__Methylomirabilia | o__Rokubacteriales | f__Rokubacteriales | g__Rokubacteriales | s__uncultured_bacterium |
| 78f2168b85647f7513da7b0b786c9b87 | Bacteria | p__Proteobacteria | c__Alphaproteobacteria | o__Rhodobacterales | f__Rhodobacteraceae | g__Gemmobacter |  |
| 1a75a3f0288d8703bd04ecb7c2b38f57 | Bacteria | p__Proteobacteria | c__Gammaproteobacteria | o__Burkholderiales | f__Alcaligenaceae | g__Achromobacter |  |
| b16f57454eb4c3205394f3c065eb95a0 | Bacteria | p__Proteobacteria | c__Alphaproteobacteria | o__Sphingomonadales | f__Sphingomonadaceae | g__Sphingomonas |  |
| 2a38a35093ffc2c5adef409144d17690 | Bacteria | p__Bacteroidota | c__Bacteroidia | o__Bacteroidales | f__Rikenellaceae | g__Rikenellaceae_RC9_gut_group |  |
| 2261d8509d29de54df47587701e7cb19 | Bacteria | p__Patescibacteria | c__Saccharimonadia | o__Saccharimonadales | f__LWQ8 | g__LWQ8 |  |
| 68a365725b19bcff71adcc71ec8f689f | Bacteria | p__Actinobacteriota | c__Actinobacteria | o__Corynebacteriales | f__Mycobacteriaceae | g__Mycobacterium |  |
| MJ006-2-barcode65-umi152766bins-ubs-3 | Bacteria | p__Bacteroidota | c__Bacteroidia | o__Bacteroidales | f__Tannerellaceae | g__Parabacteroides |  |
| RS-GCF-003474085.1-NZ-QRNS01000091.1 | Bacteria | p__Firmicutes | c__Clostridia | o__Lachnospirales | f__Lachnospiraceae | g__Dorea |  |
| RS-GCF-003626595.1-NZ-RBAK01000038.1 | Bacteria | p__Actinobacteriota | c__Actinobacteria | o__Micromonosporales | f__Micromonosporaceae | g__Micromonospora |  |
| JX986957 | Bacteria | p__Proteobacteria | c__Gammaproteobacteria | o__Burkholderiales | f__Burkholderiaceae | g__Robbsia | s__Robbsia_andropogonis |
| MJ030-1-barcode42-umi40732bins-ubs-13 | Bacteria | p__Proteobacteria | c__Gammaproteobacteria | o__Burkholderiales | f__TRA3-20 | g__TRA3-20 |  |
| MJ030-2-barcode65-umi10481bins-ubs-46 | Bacteria | p__Acidobacteriota | c__Acidobacteriae | o__Acidobacteriales |  |  |  |
| RS-GCF-019132155.1-NZ-JAHONJ010000125.1 | Bacteria | p__Bacteroidota | c__Bacteroidia | o__Bacteroidales | f__Bacteroidaceae | g__Bacteroides |  |
| KF709700 | Bacteria | p__Proteobacteria | c__Gammaproteobacteria | o__Xanthomonadales | f__Rhodanobacteraceae | g__Dyella |  |
| 17203029 | Bacteria | p__Actinobacteriota | c__Thermoleophilia | o__Solirubrobacterales | f__Solirubrobacteraceae | g__Conexibacter |  |
| 1e9b6d44c832add8263d6d6b21f48377 | Bacteria | p__Actinobacteriota | c__Actinobacteria | o__Propionibacteriales | f__Nocardioidaceae | g__Nocardioides |  |
| f7452b97ff256364b375d1918f132191 | Bacteria | p__Proteobacteria | c__Gammaproteobacteria | o__Xanthomonadales | f__Xanthomonadaceae | g__Stenotrophomonas |  |
| 3fd7760b9ea2d08211de4f9a808c28c1 | Bacteria | p__Chloroflexi | c__Anaerolineae | o__SBR1031 | f__A4b | g__A4b |  |
| 523ee371d6dcedaf59e0e03f3047fc7c | Bacteria | p__Bacteroidota | c__Bacteroidia | o__Flavobacteriales | f__Weeksellaceae | g__Chryseobacterium |  |
| 14834003 | Bacteria | p__Chloroflexi | c__Ktedonobacteria | o__C0119 | f__C0119 | g__C0119 | s__uncultured_bacterium |
| 37c472c4c9c939d9053e2fad3d5771df | Bacteria | p__Proteobacteria | c__Gammaproteobacteria | o__Burkholderiales | f__Comamonadaceae |  |  |
| efa3e06d7df4588e11406c138a2346be | Bacteria | p__Gemmatimonadota | c__Gemmatimonadetes | o__Gemmatimonadales | f__Gemmatimonadaceae | g__uncultured |  |
| f3ccbfc2fb638eb7d69ae2ea97ceeb2c | Bacteria | p__Bacteroidota | c__Bacteroidia | o__Chitinophagales | f__Chitinophagaceae | g__uncultured |  |
| 4f01840b2d95b6f919b148078e0f5c8c | Bacteria | p__Chloroflexi | c__Anaerolineae | o__Caldilineales | f__Caldilineaceae | g__uncultured | s__metagenome |
| 04955953 | Bacteria | p__Actinobacteriota | c__Thermoleophilia | o__Solirubrobacterales | f__Solirubrobacteraceae | g__Solirubrobacter |  |
| 11873459 | Bacteria | p__Proteobacteria | c__Alphaproteobacteria | o__Sphingomonadales | f__Sphingomonadaceae | g__Sphingomonas |  |
| 6b99cd24d8aaeced3f68521f654a337c | Bacteria | p__Firmicutes | c__Bacilli | o__Erysipelotrichales | f__Erysipelotrichaceae | g__Holdemanella |  |
| 7a81a598ffdc5b18bb669dfecee62120 | Bacteria | p__Methylomirabilota | c__Methylomirabilia | o__Rokubacteriales | f__Rokubacteriales | g__Rokubacteriales |  |
| 21393898 | Bacteria | p__Chloroflexi | c__OLB14 | o__OLB14 | f__OLB14 | g__OLB14 | s__uncultured_bacterium |
| 667089f0debf284d079bf080e30fb901 | Bacteria | p__Actinobacteriota | c__Actinobacteria | o__Propionibacteriales | f__Propionibacteriaceae | g__Microlunatus |  |
| 01000875 | Bacteria | p__Actinobacteriota | c__Actinobacteria | o__Corynebacteriales | f__Corynebacteriaceae | g__Corynebacterium |  |
| bafbc99be768c42b8dea188542898e93 | Bacteria | p__Actinobacteriota | c__Actinobacteria | o__Frankiales | f__Acidothermaceae | g__Acidothermus |  |
| 6aa7ec3b399b48f5e961f13463b4ea4d | Bacteria | p__Bacteroidota | c__Bacteroidia | o__Sphingobacteriales | f__Sphingobacteriaceae | g__Sphingobacterium |  |
| 07122730 | Bacteria | p__Verrucomicrobiota | c__Verrucomicrobiae | o__Chthoniobacterales | f__Chthoniobacteraceae | g__Chthoniobacter |  |
| 521451391df48599d0764e00c5fe49ed | Bacteria | p__Bacteroidota | c__Bacteroidia | o__Sphingobacteriales | f__Sphingobacteriaceae | g__Pedobacter |  |
| 46da53dcec2208e77ecbab8d75b385d6 | Bacteria | p__Chloroflexi | c__KD4-96 | o__KD4-96 | f__KD4-96 | g__KD4-96 |  |
| 4886048b447e86e98cef6ce0f2bfe403 | Bacteria | p__Acidobacteriota | c__Blastocatellia | o__11-24 | f__11-24 | g__11-24 |  |
| ea677b0faad591dee825f517acd0fdcb | Bacteria | p__Proteobacteria | c__Gammaproteobacteria | o__Xanthomonadales | f__Xanthomonadaceae |  |  |
| 3dcb620e33c2c007166dc154425db8b2 | Bacteria | p__Proteobacteria | c__Gammaproteobacteria | o__Pseudomonadales | f__Moraxellaceae | g__Acinetobacter | s__Ambystoma_mexicanum |
| d42f1d1b047e2659bcaace8af249585a | Bacteria | p__Dadabacteria | c__Dadabacteriia | o__Dadabacteriales | f__Dadabacteriales | g__Dadabacteriales | s__uncultured_bacterium |
| MJ006-1-barcode40-umi116523bins-ubs-5 | Bacteria | p__Firmicutes | c__Clostridia | o__Lachnospirales | f__Lachnospiraceae | g__Anaerostipes |  |
| b277f0bbdc4a99b393b5f2523a18edce | Bacteria | p__Firmicutes | c__Syntrophomonadia | o__Syntrophomonadales | f__Syntrophomonadaceae | g__Syntrophomonas | s__uncultured_bacterium |
| 6383e43cd8488cf5114b8354da22536e | Bacteria | p__Firmicutes | c__Bacilli | o__Staphylococcales | f__Staphylococcaceae | g__Staphylococcus |  |
| d8a1e7cc43d6db68e90c42d29547946f | Bacteria | p__Chloroflexi | c__Anaerolineae |  |  |  |  |
| 02411955 | Bacteria | p__Proteobacteria | c__Alphaproteobacteria | o__Rhizobiales | f__Rhizobiaceae |  |  |
| f9d5311ae5c8aa9f62ae03e42141c661 | Bacteria | p__Proteobacteria | c__Gammaproteobacteria | o__Burkholderiales | f__Burkholderiaceae | g__Burkholderia-Caballeronia-Paraburkholderia |  |
| 107dc689754e98bc76a9080f13f16ebd | Bacteria | p__Bacteroidota | c__Bacteroidia | o__Bacteroidales | f__Prevotellaceae | g__Prevotella |  |
| ecee75c29c7080804ed301a82471b0ab | Bacteria | p__Proteobacteria | c__Gammaproteobacteria | o__Enterobacterales | f__Enterobacteriaceae |  |  |
| 2d624e4a805a3504c0ecb0c71e59a4bb | Bacteria | p__Actinobacteriota | c__Actinobacteria | o__Micrococcales | f__Microbacteriaceae | g__Microbacterium |  |
| 5b548ea10b97cd4848af9d594424ac8b | Bacteria | p__Proteobacteria | c__Gammaproteobacteria | o__Burkholderiales | f__Comamonadaceae | g__Simplicispira |  |
| cbc0fafa15eb3c27ddcb3fc42e166bcb | Bacteria | p__Desulfobacterota | c__uncultured | o__uncultured | f__uncultured | g__uncultured |  |
| MJ006-1-barcode35-umi100404bins-ubs-5 | Bacteria | p__Proteobacteria | c__Gammaproteobacteria | o__Burkholderiales | f__Sutterellaceae | g__Sutterella | s__gut_metagenome |
| RS-GCF-016805365.1-NZ-JAAKGH010000025.1 | Bacteria | p__Proteobacteria | c__Gammaproteobacteria | o__Aeromonadales | f__Aeromonadaceae | g__Aeromonas |  |
| b32cb93c1d02920d5cb163e09144de44 | Bacteria | p__Chloroflexi | c__Ktedonobacteria | o__Ktedonobacterales | f__Ktedonobacteraceae |  |  |
| 16261011 | Bacteria | p__Firmicutes | c__Clostridia | o__Lachnospirales | f__Lachnospiraceae | g__Lachnospiraceae_NK4A136_group |  |
| f4e2c7f7928a43cc4f890f91a4056bcc | Bacteria | p__Bacteroidota | c__Bacteroidia | o__Flavobacteriales | f__Weeksellaceae | g__Empedobacter | s__Empedobacter_sp. |
| 1b34bebdfadd9ed3eb8e2f1cbc0a5077 | Bacteria | p__Firmicutes | c__Bacilli | o__Lactobacillales | f__Lactobacillaceae | g__Lactobacillus | s__Lactobacillus_aviarius |
| 6469acc9d037bbbf3a43da7dc2e14af8 | Bacteria | p__Firmicutes | c__Negativicutes | o__Veillonellales-Selenomonadales | f__Veillonellaceae | g__Veillonella |  |
| 207699627a7aae3abbced3b988dd017f | Bacteria | p__Firmicutes | c__Bacilli | o__Bacillales |  |  |  |
| FR714843 | Bacteria | p__Actinobacteriota | c__Actinobacteria | o__Corynebacteriales | f__Nocardiaceae | g__Rhodococcus |  |
| 04924902 | Bacteria | p__Bacteroidota | c__Bacteroidia | o__Bacteroidales | f__Rikenellaceae | g__Alistipes | s__uncultured_bacterium |
| 04959778 | Bacteria | p__Proteobacteria | c__Gammaproteobacteria | o__Burkholderiales | f__Burkholderiaceae | g__Burkholderia-Caballeronia-Paraburkholderia |  |
| 656b62a1d6d61e93fe3aa8db025f48de | Bacteria | p__Proteobacteria | c__Gammaproteobacteria | o__Alteromonadales | f__uncultured | g__uncultured | s__Idiomarina_sp. |
| DQ174151 | Bacteria | p__Campilobacterota | c__Campylobacteria | o__Campylobacterales | f__Campylobacteraceae | g__Campylobacter | s__Campylobacter_sputorum |
| 61e087b1d2b3ca5ed2ca541744663dda | Bacteria | p__Planctomycetota | c__Phycisphaerae | o__Tepidisphaerales | f__WD2101_soil_group | g__WD2101_soil_group | s__uncultured_Planctomycetia |
| 02325682 | Bacteria | p__Proteobacteria | c__Alphaproteobacteria | o__Rhodobacterales | f__Rhodobacteraceae | g__Rubellimicrobium | s__uncultured_bacterium |
| 4cf017723cd1fbfac185bc45d5577480 | Bacteria | p__Bacteroidota | c__Bacteroidia | o__Flavobacteriales | f__Flavobacteriaceae | g__Myroides | s__Myroides_phaeus |
| 13704587 | Bacteria | p__Gemmatimonadota | c__Gemmatimonadetes | o__Gemmatimonadales | f__Gemmatimonadaceae |  |  |
| 4affb86009118f029afbc10465ed2cc8 | Bacteria | p__Proteobacteria | c__Gammaproteobacteria | o__Xanthomonadales | f__Xanthomonadaceae | g__Stenotrophomonas |  |
| GQ988781 | Bacteria | p__Bacteroidota | c__Bacteroidia | o__Sphingobacteriales | f__Sphingobacteriaceae | g__Sphingobacterium | s__Sphingobacterium_wenxiniae |
| MG018989 | Bacteria | p__Proteobacteria | c__Alphaproteobacteria | o__Rhizobiales | f__Rhizobiaceae | g__Allorhizobium-Neorhizobium-Pararhizobium-Rhizobium |  |
| b62d82dc2d02acc23a421bdaa754b93e | Bacteria | p__Proteobacteria | c__Gammaproteobacteria | o__Xanthomonadales | f__Xanthomonadaceae | g__Lysobacter |  |
| MJ030-2-barcode58-umi42272bins-ubs-17 | Bacteria | p__Bacteroidota | c__Bacteroidia | o__Cytophagales | f__Microscillaceae | g__uncultured |  |
| 04950293 | Bacteria | p__Firmicutes | c__Clostridia | o__Lachnospirales | f__Lachnospiraceae | g__Dorea |  |
| d3f787c543cb9ec592deae2157de0387 | Bacteria | p__Proteobacteria | c__Alphaproteobacteria | o__Micropepsales | f__Micropepsaceae | g__uncultured |  |
| 43c7c7136d0c90a7d215610061c8e51d | Bacteria | p__Firmicutes | c__Clostridia | o__Lachnospirales | f__Lachnospiraceae |  |  |
| 45ed5e15dbb53490105de4f84ac591a1 | Bacteria | p__Acidobacteriota | c__Vicinamibacteria | o__Vicinamibacterales | f__Vicinamibacteraceae | g__Vicinamibacteraceae |  |
| MJ034-1-barcode31-umi21082bins-ubs-9 | Bacteria | p__Proteobacteria | c__Alphaproteobacteria | o__Rhizobiales | f__Xanthobacteraceae | g__uncultured |  |
| d5a16fa3d9a19ea49bf55666914ed93f | Bacteria | p__Actinobacteriota | c__Actinobacteria | o__Micrococcales | f__Intrasporangiaceae |  |  |
| 05025179 | Bacteria | p__Firmicutes | c__Clostridia | o__Lachnospirales | f__Lachnospiraceae |  |  |
| 04995539 | Bacteria | p__Proteobacteria | c__Alphaproteobacteria | o__Rhizobiales | f__Devosiaceae | g__Devosia |  |
| 5066cb5f52f1fdc09d69181b34de587f | Bacteria | p__Bacteroidota | c__Bacteroidia | o__Sphingobacteriales | f__Sphingobacteriaceae | g__Sphingobacterium |  |
| e65517067b33dbec28176500befbadbb | Bacteria | p__Patescibacteria | c__Saccharimonadia | o__Saccharimonadales |  |  |  |
| 5c75298473ca29ea8116564b863556a3 | Bacteria | p__Bacteroidota | c__Bacteroidia | o__Cytophagales | f__Spirosomaceae | g__Persicitalea | s__Ravibacter_arvi |
| debd16fbf5084ea7a6ff6484f4de4dcc | Bacteria | p__Bacteroidota | c__Bacteroidia | o__Flavobacteriales | f__NS9_marine_group | g__NS9_marine_group |  |
| 81860d1adec12ca579cd91029f2b5c1e | Bacteria | p__Proteobacteria | c__Alphaproteobacteria | o__Rhodobacterales | f__Rhodobacteraceae | g__Amaricoccus |  |
| G004138295_1 | Bacteria | p__Firmicutes | c__Bacilli | o__Bacillales | f__Bacillaceae | g__Bacillus | s__Bacillus_funiculus |
| AF367204 | Bacteria | p__Proteobacteria | c__Alphaproteobacteria | o__Sphingomonadales | f__Sphingomonadaceae | g__Sphingopyxis |  |
| c869d2d6d427c39767abe995ce7cf907 | Bacteria | p__Chloroflexi | c__Ktedonobacteria | o__C0119 | f__C0119 | g__C0119 | s__uncultured_Chloroflexi |
| d43bc03cd650e03b19c6b9ad1e8b441f | Bacteria | p__Proteobacteria | c__Gammaproteobacteria | o__Alteromonadales | f__uncultured | g__uncultured | s__Idiomarina_sp. |
| 9503ecb94c98c1177ebb81f1842a8a30 | Bacteria | p__Actinobacteriota | c__Acidimicrobiia | o__Microtrichales | f__Iamiaceae | g__Iamia |  |
| 5080c0e9a6b22f440b863d6e3c205882 | Bacteria | p__Verrucomicrobiota | c__Verrucomicrobiae | o__Chthoniobacterales | f__Chthoniobacteraceae | g__Candidatus_Udaeobacter | s__unidentified_eubacterium |
| eec020a6471bbb4b500cf05203ec3c40 | Bacteria | p__Firmicutes | c__Bacilli | o__Staphylococcales | f__Staphylococcaceae | g__Staphylococcus |  |
| 11312678 | Bacteria | p__Bacteroidota | c__Bacteroidia | o__Bacteroidales | f__Rikenellaceae | g__Alistipes |  |
| 70863260488d703425f5d7fbc3c02114 | Bacteria | p__Proteobacteria | c__Gammaproteobacteria | o__Pseudomonadales | f__Moraxellaceae | g__Acinetobacter |  |
| 01e88c7af5402d9e396227c973949f73 | Bacteria | p__Proteobacteria | c__Alphaproteobacteria | o__Rhodobacterales | f__Rhodobacteraceae | g__Gemmobacter |  |
| b4885757401cb30e81d03dff1a0e5512 | Bacteria | p__Verrucomicrobiota | c__Verrucomicrobiae | o__Chthoniobacterales | f__Chthoniobacteraceae | g__Candidatus_Udaeobacter |  |
| 79df42bd421a3de45058a0cb7fa0fb1f | Bacteria | p__Firmicutes | c__Clostridia | o__Lachnospirales | f__Lachnospiraceae | g__Blautia |  |
| 45b5f4c4234ae37a9da040148358b8fc | Bacteria | p__Proteobacteria | c__Gammaproteobacteria | o__Steroidobacterales | f__Steroidobacteraceae | g__uncultured |  |
| aa7743439969f9133527db69b8482a41 | Bacteria | p__Actinobacteriota | c__Actinobacteria | o__Frankiales | f__Acidothermaceae | g__Acidothermus |  |
| 8c3a786935172161241fa1618eababd8 | Bacteria | p__Methylomirabilota | c__Methylomirabilia | o__Rokubacteriales | f__Rokubacteriales | g__Rokubacteriales | s__uncultured_bacterium |
| 04544218 | Bacteria | p__Firmicutes | c__Bacilli | o__Lactobacillales | f__Streptococcaceae | g__Streptococcus |  |
| AB937774 | Bacteria | p__Proteobacteria | c__Alphaproteobacteria | o__Acetobacterales | f__Acetobacteraceae | g__Acetobacter |  |
| 26c48c16e83efa4f8d5a05f2cbbd4b7d | Bacteria | p__Firmicutes | c__Negativicutes | o__Veillonellales-Selenomonadales | f__Veillonellaceae | g__Dialister |  |
| 20881763 | Bacteria | p__Firmicutes | c__Bacilli | o__Erysipelotrichales | f__Erysipelotrichaceae | g__Ileibacterium | s__Ileibacterium_valens |
| RS-GCF-001552115.1-NZ-BCQO01000012.1 | Bacteria | p__Actinobacteriota | c__Actinobacteria | o__Micrococcales | f__Micrococcaceae | g__Glutamicibacter |  |
| a586998912b26696d5cb68b8976b098a | Bacteria | p__Proteobacteria | c__Alphaproteobacteria | o__Rhodospirillales | f__uncultured | g__uncultured |  |
| ca61e069d903e91c3af2821120c06919 | Bacteria |  |  |  |  |  |  |
| 11446311 | Bacteria | p__Proteobacteria | c__Alphaproteobacteria | o__Sphingomonadales | f__Sphingomonadaceae | g__Novosphingobium |  |
| MJ034-1-barcode31-umi31105bins-ubs-3 | Bacteria | p__Acidobacteriota | c__Vicinamibacteria | o__Vicinamibacterales | f__uncultured | g__uncultured |  |
| eaef5f87894a7ccc6900e65dc2229a25 | Bacteria | p__Chloroflexi | c__OLB14 | o__OLB14 | f__OLB14 | g__OLB14 |  |
| MJ007-2-barcode58-umi50148bins-ubs-3 | Bacteria | p__Firmicutes | c__Clostridia | o__Lachnospirales | f__Lachnospiraceae |  |  |
| 45d5cc3053b92a066e0a0b7abafc7577 | Bacteria | p__Proteobacteria | c__Alphaproteobacteria | o__Rhizobiales | f__Hyphomicrobiaceae | g__Hyphomicrobium |  |
| 28eb00af481419d5c3a3c8a64cced6ed | Bacteria | p__Proteobacteria | c__Gammaproteobacteria | o__Burkholderiales | f__Comamonadaceae | g__Acidovorax |  |
| a63a4d37a4b4a932bd8099bcd5c57783 | Bacteria | p__Chloroflexi | c__Anaerolineae |  |  |  |  |
| G000223375_1 | Bacteria | p__Proteobacteria | c__Alphaproteobacteria | o__Rhodobacterales | f__Rhodobacteraceae | g__Ketogulonicigenium | s__Ketogulonicigenium_vulgare |
| 2535e489dea2e3a483fdab2b01773aa4 | Bacteria | p__Proteobacteria | c__Alphaproteobacteria | o__Rhizobiales | f__Rhizobiales_Incertae_Sedis | g__uncultured |  |
| 04930906 | Bacteria | p__Proteobacteria | c__Gammaproteobacteria | o__Enterobacterales | f__Enterobacteriaceae | g__Escherichia-Shigella |  |
| ad0b45dc848897aab414e23fa0c57545 | Bacteria | p__Bacteroidota | c__Bacteroidia | o__Chitinophagales | f__Chitinophagaceae | g__Taibaiella |  |
| G003818285_1 | Bacteria | p__Myxococcota | c__Myxococcia | o__Myxococcales | f__Myxococcaceae |  |  |
| cd0fd1cfef8b87bdd79b5bc9b44a6087 | Bacteria | p__Bacteroidota | c__Bacteroidia | o__Flavobacteriales | f__Weeksellaceae | g__Chryseobacterium |  |
| c9677adaf47c4546bbe211d32aef7b5b | Bacteria | p__Acidobacteriota | c__Blastocatellia | o__Blastocatellales | f__Blastocatellaceae |  |  |
| 02315569 | Bacteria | p__Proteobacteria | c__Alphaproteobacteria | o__Sphingomonadales | f__Sphingomonadaceae | g__Sphingobium |  |
| 04958472 | Bacteria | p__Actinobacteriota | c__Actinobacteria | o__Frankiales | f__Geodermatophilaceae |  |  |
| 04958742 | Bacteria | p__Actinobacteriota | c__Actinobacteria | o__Frankiales | f__Geodermatophilaceae | g__Klenkia |  |
| FJ157976 | Bacteria | p__Proteobacteria | c__Alphaproteobacteria | o__Rhizobiales | f__Beijerinckiaceae | g__Methylobacterium-Methylorubrum |  |
| 819049e1a21633fd33459ee0e981dcfc | Bacteria | p__Actinobacteriota | c__Actinobacteria | o__Corynebacteriales | f__Mycobacteriaceae | g__Mycobacterium |  |
| 04966151 | Bacteria | p__Firmicutes | c__Bacilli | o__Bacillales | f__Bacillaceae | g__Bacillus |  |
| 740db34e7537bb2d9fdc949053c9c6bc | Bacteria | p__Proteobacteria | c__Gammaproteobacteria | o__Pseudomonadales | f__Moraxellaceae |  |  |
| 17182530 | Bacteria | p__Firmicutes | c__Bacilli | o__Alicyclobacillales | f__Alicyclobacillaceae | g__Tumebacillus |  |
| 05126236 | Bacteria | p__Proteobacteria | c__Gammaproteobacteria | o__Enterobacterales |  |  |  |
| 21725103 | Bacteria | p__Firmicutes | c__Clostridia | o__Lachnospirales | f__Lachnospiraceae | g__A2 | s__uncultured_bacterium |
| e6e6be7de9bee98296ce35a3842992ad | Bacteria | p__Cyanobacteria | c__Cyanobacteriia | o__Oxyphotobacteria_Incertae_Sedis | f__Unknown_Family | g__Leptolyngbya_EcFYyyy-00 |  |
| 2a9d282fe358aedf1f74d12d1f1a7bb3 | Bacteria | p__Bacteroidota | c__Bacteroidia | o__Bacteroidales | f__Muribaculaceae | g__Muribaculaceae | s__uncultured_bacterium |
| dcd1d461da244db191fea185adf3ef26 | Bacteria | p__Actinobacteriota | c__Actinobacteria | o__Micrococcales | f__Microbacteriaceae | g__Microbacterium |  |
| 0765fc1efd3913cb284c6aaa08fc7021 | Bacteria | p__Proteobacteria | c__Gammaproteobacteria | o__Pseudomonadales | f__Moraxellaceae | g__Acinetobacter |  |
| 95805577e6beeb8ad492dd23ea7ed338 | Bacteria | p__Actinobacteriota | c__Actinobacteria | o__Micrococcales | f__Microbacteriaceae | g__Microbacterium |  |
| 13948354 | Bacteria | p__Armatimonadota | c__Chthonomonadetes | o__Chthonomonadales | f__Chthonomonadales | g__Chthonomonadales | s__uncultured_bacterium |
| 623b9390dd8d8e27215582211693ecd8 | Bacteria | p__Actinobacteriota | c__Actinobacteria | o__Frankiales | f__Geodermatophilaceae |  |  |
| 15893159 | Bacteria | p__Actinobacteriota | c__Actinobacteria | o__Micrococcales | f__Microbacteriaceae | g__Microbacterium |  |
| b0a8886d78031a8a81917c8bb3442627 | Bacteria | p__Firmicutes | c__Clostridia | o__Oscillospirales | f__Oscillospiraceae | g__NK4A214_group |  |
| 1510142aa8b6940b2e7ddae1c6d6a1a8 | Bacteria | p__Methylomirabilota | c__Methylomirabilia | o__Rokubacteriales | f__Rokubacteriales | g__Rokubacteriales | s__uncultured_bacterium |
| 3deac0a41ac310ec3c7dc9bc12defc80 | Bacteria | p__Firmicutes | c__Bacilli | o__Lactobacillales | f__Enterococcaceae | g__Enterococcus |  |
| dfdf9dd556fd7b1cba83ccbd9f7cea7f | Bacteria | p__Firmicutes | c__Clostridia | o__Oscillospirales | f__Butyricicoccaceae | g__UCG-009 |  |
| 6a1af7ec80e25c7f48b0ee0d644d6eb2 | Bacteria | p__Actinobacteriota | c__MB-A2-108 | o__MB-A2-108 | f__MB-A2-108 | g__MB-A2-108 |  |
| 36c6633cc327532cdfc403944719575d | Bacteria | p__Proteobacteria | c__Gammaproteobacteria | o__Burkholderiales | f__B1-7BS | g__B1-7BS |  |
| 67be792999bb7c380e1feb85bd422295 | Bacteria | p__Firmicutes | c__Clostridia | o__Oscillospirales | f__Ruminococcaceae | g__Faecalibacterium | s__uncultured_bacterium |
| 39faf45612ca6d5056df7d16b372ec3e | Bacteria | p__Firmicutes | c__Bacilli | o__Erysipelotrichales | f__Erysipelatoclostridiaceae | g__Erysipelatoclostridium |  |
| 3630c18cc5cc0e1ff31d4e0828ab8630 | Bacteria | p__Acidobacteriota | c__Blastocatellia | o__Blastocatellales | f__Blastocatellaceae |  |  |
| 9eebfa06f61b2128c325c622a3f3998b | Bacteria | p__Bacteroidota | c__Bacteroidia | o__Bacteroidales | f__Bacteroidetes_vadinHA17 | g__Bacteroidetes_vadinHA17 | s__uncultured_Bacteroidetes |
| 97ee59d291fc8590809e5c12a7c3aa27 | Bacteria | p__Firmicutes | c__Bacilli | o__Paenibacillales | f__Paenibacillaceae | g__Paenibacillus |  |
| 235e6674731f39d157f3047073e57139 | Bacteria | p__Proteobacteria | c__Alphaproteobacteria | o__Rhizobiales | f__Hyphomicrobiaceae | g__Pedomicrobium |  |
| 7ec5b9a2b6212bdb8297c1e3c48002ed | Bacteria | p__Firmicutes | c__Bacilli | o__Alicyclobacillales | f__Alicyclobacillaceae | g__Tumebacillus |  |
| RS-GCF-014196195.1-NZ-JACIDF010000024.1 | Bacteria | p__Firmicutes | c__Bacilli | o__Bacillales | f__Bacillaceae | g__Anoxybacillus |  |
| f0bc6fbf2f8650e14682e2be768a7235 | Bacteria | p__Bacteroidota | c__Bacteroidia | o__Bacteroidales | f__Prevotellaceae | g__Prevotella |  |
| f4a11a9fa0877c3ab71d4d862f68a3ce | Bacteria | p__Proteobacteria | c__Gammaproteobacteria | o__Xanthomonadales | f__Rhodanobacteraceae | g__Dokdonella |  |
| 852062962d1ae9587c1d1fe9b4f4a377 | Bacteria | p__Actinobacteriota | c__Actinobacteria | o__Micrococcales | f__Micrococcaceae | g__Nesterenkonia |  |
| 41a3c06acc89fca5bc7dd4ecaf25a040 | Bacteria | p__Firmicutes | c__Bacilli | o__Lactobacillales | f__Streptococcaceae | g__Streptococcus |  |
| MJ006-2-barcode52-umi59436bins-ubs-7 | Bacteria | p__Firmicutes | c__Clostridia | o__Lachnospirales | f__Lachnospiraceae |  |  |
| 05363592 | Bacteria | p__Bacteroidota | c__Bacteroidia | o__Bacteroidales | f__Bacteroidaceae | g__Bacteroides | s__Bacteroides_sartorii |
| 5e046b44605a0a86b25d3ad42d7777d5 | Bacteria | p__Firmicutes | c__Clostridia | o__Lachnospirales | f__Lachnospiraceae |  |  |
| a6dba8096f2a5f382f5421c95ab9501b | Bacteria | p__Actinobacteriota | c__Actinobacteria | o__Corynebacteriales | f__Corynebacteriaceae | g__Corynebacterium | s__Corynebacterium_sputi |
| 397e5ff1edf85ada001d1cb9c0bed4a8 | Bacteria |  |  |  |  |  |  |
| 5908519fa73be507d31292143f9c5b86 | Bacteria |  |  |  |  |  |  |
| Y18098 | Bacteria | p__Firmicutes | c__Bacilli | o__Lactobacillales | f__Vagococcaceae | g__Vagococcus |  |
| a04faf6d3f9a03f5684e8a1daf6d3a18 | Bacteria | p__Chloroflexi | c__KD4-96 | o__KD4-96 | f__KD4-96 | g__KD4-96 |  |
| c9597e9d406466ab93c3eb2cf5e7b05a | Bacteria | p__Proteobacteria | c__Gammaproteobacteria | o__Xanthomonadales | f__Xanthomonadaceae | g__Xanthomonas |  |
| a6c103ed6996b6d25f378a3c7ae1d99d | Bacteria | p__Actinobacteriota | c__Actinobacteria | o__Corynebacteriales | f__Corynebacteriaceae | g__Corynebacterium |  |
| a123988ee6ff0a4e458ff0b1f800c4d0 | Bacteria | p__Proteobacteria | c__Gammaproteobacteria | o__Burkholderiales | f__Methylophilaceae |  |  |
| 7a189f47e6d513a31260931cbb3c7585 | Bacteria | p__Proteobacteria | c__Gammaproteobacteria | o__Burkholderiales | f__Nitrosomonadaceae | g__DSSD61 |  |
| 13720516 | Bacteria | p__Gemmatimonadota | c__Gemmatimonadetes | o__Gemmatimonadales | f__Gemmatimonadaceae | g__Gemmatimonas |  |
| 04340652 | Bacteria | p__Chloroflexi | c__Anaerolineae | o__SBR1031 | f__SBR1031 | g__SBR1031 |  |
| 1121a8f4a8496054cc7898db57805fe5 | Bacteria | p__Proteobacteria | c__Alphaproteobacteria | o__Rhizobiales | f__Xanthobacteraceae | g__uncultured |  |
| fd7d525fe2ca3046d78a5078f03b7d03 | Bacteria | p__Bacteroidota | c__Bacteroidia | o__Chitinophagales | f__Chitinophagaceae |  |  |
| c13f9cf2e8557373c8b313c2d815b14e | Bacteria | p__Proteobacteria | c__Gammaproteobacteria | o__Burkholderiales | f__Oxalobacteraceae |  |  |
| G902792045_1 | Bacteria | p__Firmicutes | c__Bacilli | o__Thermoactinomycetales | f__Thermoactinomycetaceae | g__Thermoactinomyces |  |
| 2e254792682ed588039f104cb5c6c695 | Bacteria | p__Proteobacteria | c__Gammaproteobacteria | o__Xanthomonadales | f__Xanthomonadaceae | g__Stenotrophomonas |  |
| RS-GCF-008633155.1-NZ-BHYQ01000011.1 | Bacteria | p__Firmicutes | c__Clostridia | o__Peptostreptococcales-Tissierellales | f__Peptostreptococcales-Tissierellales | g__Parvimonas |  |
| fa8007dd63247c627cc8433ec1519d86 | Bacteria | p__Chloroflexi | c__Ktedonobacteria | o__Ktedonobacterales | f__Ktedonobacteraceae | g__HSB_OF53-F07 | s__uncultured_bacterium |
| 02918510 | Bacteria | p__Planctomycetota | c__Phycisphaerae | o__Tepidisphaerales | f__WD2101_soil_group | g__WD2101_soil_group |  |
| c06c00eae9182e5e6fcf6b0093253650 | Bacteria | p__Proteobacteria | c__Gammaproteobacteria |  |  |  |  |
| G000762865_2 | Bacteria | p__Actinobacteriota | c__Actinobacteria | o__Corynebacteriales | f__Corynebacteriaceae | g__Corynebacterium |  |
| e2d9b5a731864d21eb7039cb655a9a2c | Bacteria | p__Proteobacteria | c__Gammaproteobacteria | o__Enterobacterales | f__Enterobacteriaceae |  |  |
| 04880462 | Bacteria | p__Proteobacteria | c__Gammaproteobacteria | o__Enterobacterales | f__Enterobacteriaceae |  |  |
| d7080e78f4769ea9d9ced8abf74447cd | Bacteria | p__Patescibacteria | c__Saccharimonadia | o__Saccharimonadales | f__Saccharimonadales | g__Saccharimonadales |  |
| MJ034-2-barcode60-umi15801bins-ubs-5 | Bacteria | p__Acidobacteriota | c__Acidobacteriae | o__Acidobacteriales | f__Acidobacteriaceae_(Subgroup_1) | g__Edaphobacter |  |
| 02325079 | Bacteria | p__Actinobacteriota | c__Actinobacteria | o__Propionibacteriales | f__Nocardioidaceae | g__Nocardioides |  |
| 05006789 | Bacteria | p__Bacteroidota | c__Bacteroidia | o__Bacteroidales | f__Muribaculaceae | g__Muribaculaceae |  |
| cba2fd81b0804136187d0f7e363994f0 | Bacteria | p__Firmicutes | c__Bacilli | o__Erysipelotrichales | f__Erysipelotrichaceae | g__Turicibacter | s__uncultured_bacterium |
| b48f9553b55f8121d33c360bb46ba0de | Bacteria | p__Proteobacteria | c__Alphaproteobacteria | o__Rhizobiales | f__Rhizobiaceae |  |  |
| 14333846 | Bacteria | p__Acidobacteriota | c__Vicinamibacteria | o__Vicinamibacterales | f__uncultured | g__uncultured |  |
| 04953464 | Bacteria | p__Firmicutes | c__Clostridia | o__Lachnospirales | f__Lachnospiraceae |  |  |
| b4e5cea8b90377b876508255782838d1 | Bacteria | p__Proteobacteria | c__Alphaproteobacteria | o__Rhizobiales |  |  |  |
| 04960299 | Bacteria | p__Proteobacteria | c__Gammaproteobacteria | o__Burkholderiales | f__Nitrosomonadaceae | g__MND1 |  |
| G000297395_4 | Bacteria | p__Actinobacteriota | c__Actinobacteria | o__Micromonosporales | f__Micromonosporaceae | g__Micromonospora |  |
| 19706817 | Bacteria | p__Firmicutes | c__Bacilli | o__Erysipelotrichales | f__Erysipelotrichaceae | g__Ileibacterium | s__Ileibacterium_valens |
| 67d76f730d1d73872493e3382502b7bf | Bacteria | p__Proteobacteria | c__Gammaproteobacteria | o__Burkholderiales | f__Burkholderiaceae | g__Burkholderia-Caballeronia-Paraburkholderia |  |
| 75c538ccf03d5ad156e95c46ad8e2f91 | Bacteria | p__Actinobacteriota | c__Thermoleophilia | o__Gaiellales |  |  |  |
| 02406433 | Bacteria | p__Actinobacteriota | c__Actinobacteria | o__Corynebacteriales | f__Corynebacteriaceae | g__Corynebacterium |  |
| MJ031-1-barcode42-umi32314bins-ubs-7 | Bacteria | p__Firmicutes | c__Bacilli | o__Bacillales | f__Bacillaceae | g__Bacillus |  |
| 05012916 | Bacteria | p__Bacteroidota | c__Bacteroidia | o__Chitinophagales | f__Chitinophagaceae |  |  |
| HM770864 | Bacteria | p__Actinobacteriota | c__Actinobacteria | o__Corynebacteriales | f__Mycobacteriaceae | g__Mycobacterium |  |
| 247934ae31f3a1a258f18309586b02b7 | Bacteria | p__Actinobacteriota | c__Actinobacteria | o__Micrococcales | f__Microbacteriaceae |  |  |
| f888f7b7942fd82565630b9db322992d | Bacteria | p__Proteobacteria | c__Gammaproteobacteria | o__Pseudomonadales | f__Pseudomonadaceae | g__Pseudomonas |  |
| 5bf936a2dff58f152026bff162c8603f | Bacteria | p__Firmicutes | c__Bacilli | o__Lactobacillales | f__Lactobacillaceae | g__Lactobacillus | s__Lactobacillus_brevis |
| 307a1393f3868c3761a6dfb9e05fa692 | Bacteria | p__Proteobacteria | c__Alphaproteobacteria | o__Elsterales | f__uncultured | g__uncultured |  |
| b17659e055ad60ef614458f8afdee59e | Bacteria | p__Proteobacteria | c__Gammaproteobacteria | o__Enterobacterales |  |  |  |
| 04957255 | Bacteria | p__Proteobacteria | c__Gammaproteobacteria | o__Burkholderiales | f__Burkholderiaceae | g__Burkholderia-Caballeronia-Paraburkholderia |  |
| 698ceab8545ac933f5dce45f9e487448 | Bacteria | p__Actinobacteriota | c__Actinobacteria | o__Corynebacteriales | f__Corynebacteriaceae | g__Corynebacterium |  |
| 4d91e306af445e548b171f1b9e40ba1a | Bacteria | p__Bacteroidota | c__Bacteroidia | o__Bacteroidales | f__Muribaculaceae | g__Muribaculaceae |  |
| 7f3a52dcf3e2474ef94ae4d04da0cd7c | Bacteria | p__Firmicutes | c__Bacilli | o__Erysipelotrichales | f__Erysipelatoclostridiaceae | g__Erysipelatoclostridium | s__uncultured_Clostridium |
| 04899787 | Bacteria | p__Acidobacteriota | c__Vicinamibacteria | o__Vicinamibacterales | f__Vicinamibacteraceae | g__Vicinamibacteraceae |  |
| 83b7f8beef352b280c150821ef6645f8 | Bacteria | p__Firmicutes | c__Bacilli | o__Lactobacillales | f__Lactobacillaceae | g__Lactobacillus | s__Lactobacillus_ruminis |
| 05031082 | Bacteria | p__Actinobacteriota | c__Actinobacteria | o__Frankiales | f__Frankiaceae | g__Jatrophihabitans |  |
| 05370641 | Bacteria | p__Proteobacteria | c__Gammaproteobacteria | o__Enterobacterales | f__Morganellaceae | g__Providencia |  |
| 15789727 | Bacteria | p__Firmicutes | c__Clostridia | o__Oscillospirales | f__Ruminococcaceae | g__Incertae_Sedis | s__uncultured_Clostridiales |
| f9b747bf9dd51e34d6ff8c2a0f5cb6b6 | Bacteria | p__Actinobacteriota | c__Thermoleophilia | o__Gaiellales | f__uncultured | g__uncultured |  |
| KF733462 | Bacteria | p__Proteobacteria | c__Gammaproteobacteria | o__Burkholderiales | f__Burkholderiaceae | g__Burkholderia-Caballeronia-Paraburkholderia |  |
| MJ032-2-barcode73-umi14736bins-ubs-34 | Bacteria | p__Proteobacteria | c__Gammaproteobacteria | o__Enterobacterales | f__Enterobacteriaceae |  |  |
| d970519bacda950403594fc6090f7d3a | Bacteria | p__Acidobacteriota | c__Acidobacteriae | o__Solibacterales | f__Solibacteraceae | g__Candidatus_Solibacter |  |
| 3fecb3681173d595a936e9dfe29807a7 | Bacteria | p__Actinobacteriota | c__Actinobacteria | o__Bifidobacteriales | f__Bifidobacteriaceae | g__Bifidobacterium |  |
| ee3b21a841bfc24be6ba293142e0304e | Bacteria | p__Desulfobacterota | c__Desulfuromonadia | o__Geobacterales | f__Geobacteraceae |  |  |
| AY588277 | Bacteria | p__Actinobacteriota | c__Actinobacteria | o__Micrococcales | f__Micrococcaceae | g__Nesterenkonia |  |
| AY264674 | Bacteria | p__Proteobacteria | c__Gammaproteobacteria | o__Enterobacterales | f__Morganellaceae | g__Arsenophonus |  |
| 4367ce224c77203deb56a3d2a6584082 | Bacteria | p__Cyanobacteria | c__Cyanobacteriia | o__Cyanobacteriales | f__uncultured | g__uncultured |  |
| d4e6479f169f4df0877bc6698da7a318 | Bacteria | p__Actinobacteriota | c__Actinobacteria | o__Pseudonocardiales | f__Pseudonocardiaceae | g__Prauserella |  |
| 02325080 | Bacteria | p__Actinobacteriota | c__Actinobacteria | o__Propionibacteriales | f__Nocardioidaceae | g__Nocardioides | s__Nocardioides_exalbidus |
| MJ007-1-barcode25-umi1766bins-ubs-168 | Bacteria | p__Bacteroidota | c__Bacteroidia | o__Sphingobacteriales | f__Sphingobacteriaceae | g__Mucilaginibacter |  |
| 06705760 | Bacteria | p__Gemmatimonadota | c__Gemmatimonadetes | o__Gemmatimonadales | f__Gemmatimonadaceae | g__uncultured |  |
| cdb3c476f82d99c7ff685a09e0fa4f49 | Bacteria | p__Firmicutes | c__Bacilli | o__Staphylococcales | f__Staphylococcaceae | g__Staphylococcus |  |
| 648d9c7325da4e18c68e7625710da6d4 | Bacteria | p__Gemmatimonadota | c__Gemmatimonadetes | o__Gemmatimonadales | f__Gemmatimonadaceae |  |  |
| JF824806 | Bacteria | p__Actinobacteriota | c__Actinobacteria | o__Micrococcales | f__Brevibacteriaceae | g__Brevibacterium |  |
| f8dbddb2df5f2775353e262d79f26acb | Bacteria | p__Proteobacteria | c__Gammaproteobacteria | o__Alteromonadales | f__Alteromonadaceae |  |  |
| 01029759 | Bacteria | p__Proteobacteria | c__Gammaproteobacteria | o__Burkholderiales | f__Comamonadaceae |  |  |
| df2dc1315d38b45f67acc35e525bb2f4 | Bacteria | p__Firmicutes | c__Bacilli | o__Bacillales | f__Bacillaceae | g__Pseudogracilibacillus | s__uncultured_bacterium |
| 04916881 | Bacteria | p__Cyanobacteria | c__Cyanobacteriia | o__Cyanobacteriales | f__Coleofasciculaceae | g__Microcoleus_PCC-7113 | s__Microcoleus_sp. |
| DQ244077 | Bacteria | p__Bacteroidota | c__Bacteroidia | o__Chitinophagales | f__Chitinophagaceae | g__Niastella | s__Niastella_koreensis |
| 36a034eea45bcc6259c1732afc181c01 | Bacteria | p__Gemmatimonadota | c__Gemmatimonadetes | o__Gemmatimonadales | f__Gemmatimonadaceae | g__Gemmatimonas |  |
| 16770453 | Bacteria | p__Firmicutes | c__Clostridia | o__Lachnospirales | f__Lachnospiraceae | g__Lachnoclostridium |  |
| 11105697 | Bacteria | p__Bacteroidota | c__Bacteroidia | o__Bacteroidales | f__Marinifilaceae | g__Odoribacter | s__uncultured_bacterium |
| 1ec87a650134cde1bcfafd3c9c3aa42a | Bacteria | p__Bacteroidota | c__Bacteroidia | o__Bacteroidales | f__Muribaculaceae | g__Muribaculaceae |  |
| f9bf76f8858fc6755e9b8c5470e964e7 | Bacteria | p__Bacteroidota | c__Bacteroidia | o__Chitinophagales | f__Chitinophagaceae | g__Terrimonas |  |
| 02428537 | Bacteria | p__Proteobacteria | c__Alphaproteobacteria | o__Caulobacterales | f__Caulobacteraceae | g__Brevundimonas |  |
| bf6cfce56487f6a6f71fb927ac3fc1b5 | Bacteria | p__Proteobacteria | c__Gammaproteobacteria | o__Burkholderiales | f__TRA3-20 | g__TRA3-20 | s__uncultured_Alcaligenaceae |
| b502155e022d22e5a2af4df542fa91b8 | Bacteria | p__Proteobacteria | c__Alphaproteobacteria | o__Acetobacterales | f__Acetobacteraceae |  |  |
| MJ030-1-barcode25-umi9584bins-ubs-43 | Bacteria | p__Firmicutes | c__Clostridia | o__Clostridiales | f__Clostridiaceae | g__Clostridium_sensu_stricto_9 |  |
| LC368136 | Bacteria | p__Proteobacteria | c__Gammaproteobacteria | o__Burkholderiales | f__Methylophilaceae | g__Methylotenera |  |
| 04943189 | Bacteria | p__Acidobacteriota | c__Acidobacteriae | o__Bryobacterales | f__Bryobacteraceae | g__Bryobacter |  |
| 05032775 | Bacteria | p__Actinobacteriota | c__Actinobacteria | o__Corynebacteriales | f__Corynebacteriaceae | g__Lawsonella |  |
| 99302b0d7518f2c15e9a3b162d848d70 | Bacteria | p__Proteobacteria | c__Alphaproteobacteria | o__Rhizobiales | f__Xanthobacteraceae |  |  |
| 0c91d06b3d2c37f879254dbd4e51d569 | Bacteria | p__Actinobacteriota | c__Actinobacteria | o__Pseudonocardiales | f__Pseudonocardiaceae | g__Prauserella |  |
| 693ffb6b4a3256d25337e2c35c1fdaf3 | Bacteria | p__Chloroflexi | c__AD3 | o__AD3 | f__AD3 | g__AD3 |  |
| 05031001 | Bacteria | p__Actinobacteriota | c__Actinobacteria | o__Corynebacteriales | f__Nocardiaceae | g__Rhodococcus |  |
| 20f813b36a1a9cb5aaecc39d6e9a7a34 | Bacteria | p__Myxococcota | c__Polyangia | o__Polyangiales | f__Polyangiaceae | g__Pajaroellobacter |  |
| 17183130 | Bacteria | p__Firmicutes | c__Bacilli | o__Alicyclobacillales | f__Alicyclobacillaceae | g__Tumebacillus | s__uncultured_Bacillus |
| fc5c41227ed19a08ce9e176d6e390067 | Bacteria | p__Bacteroidota | c__Bacteroidia | o__Bacteroidales | f__Rikenellaceae | g__Rikenellaceae_RC9_gut_group |  |
| 19cd667dd8796cbe9329a749bcb68a36 | Bacteria | p__Proteobacteria | c__Gammaproteobacteria | o__Xanthomonadales | f__Rhodanobacteraceae | g__Dyella |  |
| e5879b6ee23a957bd838618b9eaf8027 | Bacteria | p__Bacteroidota | c__Bacteroidia | o__Sphingobacteriales | f__env.OPS_17 | g__env.OPS_17 | s__uncultured_Sphingobacteriia |
| 453883c5a0f521af7baf79f76d4c91a4 | Bacteria | p__Firmicutes | c__Bacilli | o__Erysipelotrichales | f__Erysipelotrichaceae | g__Ileibacterium | s__Ileibacterium_valens |
| 9b8918923cbc1c110424fdb636f27b14 | Bacteria | p__Acidobacteriota | c__Vicinamibacteria | o__Subgroup_17 | f__Subgroup_17 | g__Subgroup_17 |  |
| 1b6bdd2f5b35939adeba9184ad0fb36c | Bacteria | p__Proteobacteria | c__Alphaproteobacteria | o__Reyranellales | f__Reyranellaceae | g__Reyranella |  |
| a52e65b352c67fe6aca36ebd0166d0b2 | Bacteria | p__Bacteroidota | c__Bacteroidia | o__Bacteroidales | f__Rikenellaceae | g__Alistipes |  |
| dffbdae6788f4f78d66619bc6c084371 | Bacteria | p__Bacteroidota | c__Bacteroidia | o__Chitinophagales | f__Chitinophagaceae | g__Sediminibacterium |  |
| f3a937b6464d4438f7483cd78fb15224 | Bacteria | p__Chloroflexi | c__Chloroflexia | o__Thermomicrobiales | f__JG30-KF-CM45 | g__JG30-KF-CM45 |  |
| GB-GCA-003762605.1-RIBA01000042.1 | Bacteria | p__Bacteroidota | c__Bacteroidia | o__Bacteroidales | f__Bacteroidaceae | g__Bacteroides |  |
| MJ006-2-barcode62-umi112167bins-ubs-4 | Bacteria | p__Firmicutes | c__Bacilli | o__Erysipelotrichales | f__Erysipelotrichaceae | g__Holdemanella |  |
| a5f52142834bd156ba0374efe84eee4f | Bacteria | p__Firmicutes | c__Bacilli | o__Bacillales | f__Bacillaceae | g__Bacillus |  |
| 17406299 | Bacteria | p__Armatimonadota | c__uncultured | o__uncultured | f__uncultured | g__uncultured |  |
| eba2ebeffce0d515b5816229cf739ace | Bacteria | p__Chloroflexi | c__Anaerolineae | o__Caldilineales | f__Caldilineaceae |  |  |
| RS-GCF-000419645.1-NZ-ATTK01000154.1 | Bacteria | p__Proteobacteria | c__Alphaproteobacteria | o__Rhizobiales | f__Rhizobiaceae | g__Ensifer |  |
| FJ859899 | Bacteria | p__Bacteroidota | c__Bacteroidia | o__Sphingobacteriales | f__Sphingobacteriaceae | g__Sphingobacterium | s__Sphingobacterium_hotanense |
| 236bc0986a0f799634f6b3e6e7ff4985 | Bacteria | p__Firmicutes | c__Clostridia | o__Lachnospirales | f__Lachnospiraceae |  |  |
| ac1295334ab41cde52b5aed24c495ca0 | Bacteria | p__Proteobacteria | c__Gammaproteobacteria | o__Burkholderiales | f__SC-I-84 | g__SC-I-84 |  |
| 739dab86106a719c82e1d200979407cb | Bacteria | p__Acidobacteriota | c__Vicinamibacteria | o__Vicinamibacterales | f__uncultured | g__uncultured |  |
| RS-GCF-003478645.1-NZ-QUFU01000071.1 | Bacteria | p__Firmicutes | c__Clostridia | o__Oscillospirales | f__Butyricicoccaceae | g__Butyricicoccus |  |
| AM403587 | Bacteria | p__Proteobacteria | c__Gammaproteobacteria | o__Xanthomonadales | f__Xanthomonadaceae |  |  |
| 04964601 | Bacteria | p__Firmicutes | c__Bacilli | o__Lactobacillales | f__Lactobacillaceae | g__Lactobacillus |  |
| 736964b54e510414baf23896caa5c07f | Bacteria | p__Proteobacteria | c__Alphaproteobacteria | o__Rhizobiales | f__Hyphomicrobiaceae | g__Hyphomicrobium |  |
| G007954425_1 | Bacteria | p__Proteobacteria | c__Alphaproteobacteria | o__Sphingomonadales | f__Sphingomonadaceae |  |  |
| RS-GCF-009496535.1-NZ-WIDL01000048.1 | Bacteria | p__Proteobacteria | c__Gammaproteobacteria | o__Xanthomonadales | f__Xanthomonadaceae | g__Stenotrophomonas |  |
| 494cc2f3f7c28b0b4852c55eccaea173 | Bacteria | p__Firmicutes | c__Bacilli | o__Erysipelotrichales | f__Erysipelotrichaceae | g__ZOR0006 |  |
| 15dd09c98f13a3402ef8def9e14d3368 | Bacteria | p__Proteobacteria | c__Gammaproteobacteria | o__Burkholderiales | f__Nitrosomonadaceae | g__Nitrosospira |  |
| G004803915_3 | Bacteria | p__Bacteroidota | c__Bacteroidia | o__Bacteroidales | f__Muribaculaceae | g__Muribaculaceae | s__uncultured_Bacteroidales |
| 0ec7915a948b2beb16c19587bef5aa95 | Bacteria | p__Bacteroidota | c__Bacteroidia | o__Chitinophagales | f__Saprospiraceae | g__uncultured |  |
| 97d98778996dc1a3ecb311d9c8d13057 | Bacteria | p__Proteobacteria | c__Alphaproteobacteria | o__Sphingomonadales | f__Sphingomonadaceae |  |  |
| d0ef4c13237361d0c6aeb19c2b7b4668 | Bacteria | p__Bacteroidota | c__Bacteroidia | o__Chitinophagales | f__Chitinophagaceae | g__uncultured |  |
| 58d42bae6cc76be396da0c5a1e0d2df0 | Bacteria | p__Bacteroidota | c__Bacteroidia | o__Sphingobacteriales | f__Sphingobacteriaceae | g__Parapedobacter |  |
| db8a4b347ff374e9d3ccd599bb93e139 | Bacteria | p__Acidobacteriota | c__Blastocatellia | o__Blastocatellales | f__Blastocatellaceae | g__JGI_0001001-H03 |  |
| 01779574 | Bacteria | p__Actinobacteriota | c__MB-A2-108 | o__MB-A2-108 | f__MB-A2-108 | g__MB-A2-108 |  |
| b99523268de3a69653745148f5c336d7 | Bacteria | p__Bacteroidota | c__Bacteroidia | o__Bacteroidales | f__Prolixibacteraceae | g__uncultured | s__uncultured_anaerobic |
| 4ab1b931cfbbe81a36d43715533573d0 | Bacteria | p__Bacteroidota | c__Bacteroidia | o__Bacteroidales | f__Tannerellaceae | g__Parabacteroides |  |
| 910e78eb49d327bc858f86d81d958512 | Bacteria | p__Proteobacteria | c__Gammaproteobacteria | o__Burkholderiales | f__Comamonadaceae |  |  |
| RS-GCF-016725325.1-NZ-CP068083.1 | Bacteria | p__Bacteroidota | c__Bacteroidia | o__Sphingobacteriales | f__Sphingobacteriaceae | g__Sphingobacterium | s__Sphingobacterium_spiritivorum |
| 046ee878166414749d617fa3ca496d13 | Bacteria | p__Proteobacteria | c__Gammaproteobacteria | o__Pseudomonadales | f__Moraxellaceae | g__Acinetobacter |  |
| 9b3cab749ede0c485a8bb174d0cc001c | Bacteria | p__Chloroflexi | c__KD4-96 | o__KD4-96 | f__KD4-96 | g__KD4-96 |  |
| LT746188 | Bacteria | p__Actinobacteriota | c__Actinobacteria | o__Frankiales | f__Geodermatophilaceae | g__Klenkia |  |
| 12979195 | Bacteria | p__Bacteroidota | c__Bacteroidia | o__Flavobacteriales | f__Weeksellaceae | g__Chryseobacterium |  |
| 6fbc2c3c5a04e39d706f69ee1f536995 | Bacteria | p__Bacteroidota | c__Bacteroidia | o__Chitinophagales | f__Chitinophagaceae | g__Flaviaesturariibacter | s__uncultured_bacterium |
| 1eea1411b3e035540d41b76a138184e9 | Bacteria | p__Proteobacteria | c__Gammaproteobacteria | o__Pseudomonadales | f__Pseudomonadaceae | g__Pseudomonas |  |
| ae5f1245f6076d7281dbaa597b480d58 | Bacteria | p__Firmicutes | c__Clostridia | o__Clostridia | f__Hungateiclostridiaceae |  |  |
| 25a9340e007a9e49f93acf2aecb69839 | Bacteria | p__Proteobacteria | c__Gammaproteobacteria | o__Steroidobacterales | f__Steroidobacteraceae | g__uncultured |  |
| 040319dc9bc3f7b67709135dd6253e54 | Bacteria | p__Proteobacteria | c__Gammaproteobacteria | o__Burkholderiales | f__Burkholderiaceae | g__Ralstonia |  |
| 100ec1647c2f67e37a56e56706709b87 | Bacteria |  |  |  |  |  |  |
| MJ033-1-barcode32-umi31167bins-ubs-7 | Bacteria | p__Acidobacteriota | c__Acidobacteriae | o__Solibacterales | f__Solibacteraceae | g__Candidatus_Solibacter | s__uncultured_forest |
| 631ca10922420f8065fbafe17cf7d636 | Bacteria |  |  |  |  |  |  |
| 58835e16296c90e1a532a5a48d72f948 | Bacteria | p__Actinobacteriota | c__Thermoleophilia | o__Gaiellales | f__uncultured | g__uncultured |  |
| f216da3e12d797b6d17a88dff7adea38 | Bacteria | p__Proteobacteria | c__Alphaproteobacteria | o__Rhizobiales | f__Devosiaceae | g__Devosia |  |
| c1e86c91d85d1a4c000925c075751597 | Bacteria | p__Chloroflexi | c__Ktedonobacteria | o__Ktedonobacterales | f__Ktedonobacteraceae | g__Ktedonobacter |  |
| 05131974 | Bacteria | p__Actinobacteriota | c__Actinobacteria | o__Micrococcales | f__Dermacoccaceae | g__Kytococcus |  |
| KM273177 | Bacteria | p__Proteobacteria | c__Alphaproteobacteria | o__Rhizobiales | f__Rhizobiaceae | g__Aureimonas |  |
| AB234027 | Bacteria | p__Actinobacteriota | c__Actinobacteria | o__Micrococcales | f__Microbacteriaceae |  |  |
| AY363245 | Bacteria | p__Proteobacteria | c__Gammaproteobacteria | o__Salinisphaerales | f__Solimonadaceae | g__Hydrocarboniphaga |  |
| 04958883 | Bacteria | p__Actinobacteriota | c__Actinobacteria | o__Frankiales | f__Geodermatophilaceae | g__Blastococcus |  |
| c12e9e72ed7c3e71b6af650cae78f3a4 | Bacteria | p__Proteobacteria | c__Alphaproteobacteria | o__Rhizobiales | f__Xanthobacteraceae | g__uncultured |  |
| 00d60151ad1660b4a9e1d51eb5aaa018 | Bacteria | p__Proteobacteria | c__Gammaproteobacteria | o__Methylococcales | f__Methylococcaceae |  |  |
| 02247882 | Bacteria | p__Proteobacteria | c__Alphaproteobacteria | o__Rhizobiales | f__Beijerinckiaceae | g__Methylobacterium-Methylorubrum |  |
| 8122a2ecd90682c0adf780496db32258 | Bacteria | p__Cyanobacteria | c__Cyanobacteriia | o__Oxyphotobacteria_Incertae_Sedis | f__Unknown_Family | g__Leptolyngbya_EcFYyyy-00 | s__uncultured_bacterium |
| 71799f0c0e95d8b3c139fd9b44ff9bc1 | Bacteria | p__Actinobacteriota | c__Actinobacteria | o__Propionibacteriales | f__Nocardioidaceae | g__Kribbella |  |
| 09fa4d11b579401ba1f53f7c7adc5fcd | Bacteria | p__Firmicutes | c__Bacilli | o__Bacillales | f__Bacillaceae | g__Geobacillus |  |
| 0e17d3e2668a744170a7c06d67905fc8 | Bacteria | p__Firmicutes | c__Incertae_Sedis | o__DTU014 | f__DTU014 | g__DTU014 |  |
| 02250933 | Bacteria | p__Proteobacteria | c__Alphaproteobacteria | o__Tistrellales | f__Geminicoccaceae | g__uncultured | s__uncultured_bacterium |
| 11a4f4b74f9309144a9bc267369787df | Bacteria | p__Proteobacteria | c__Alphaproteobacteria | o__Micropepsales | f__Micropepsaceae | g__Micropepsaceae | s__uncultured_proteobacterium |
| 03ba9e411316be8169876f29dd5a8c94 | Bacteria | p__Firmicutes | c__Bacilli | o__Erysipelotrichales | f__Erysipelatoclostridiaceae | g__Erysipelatoclostridium | s__uncultured_Clostridium |
| 920dfe9d1f9824d3b79bf5af7c2dde78 | Bacteria | p__Proteobacteria | c__Gammaproteobacteria | o__Enterobacterales | f__Enterobacteriaceae |  |  |
| 05041822 | Bacteria | p__Firmicutes | c__Bacilli | o__Lactobacillales | f__Leuconostocaceae | g__Leuconostoc |  |
| d468431d8788328a57b547c564645451 | Bacteria | p__Acidobacteriota | c__Vicinamibacteria | o__Vicinamibacterales | f__uncultured | g__uncultured |  |
| RS-GCF-015477615.1-NZ-JADLQW010000090.1 | Bacteria | p__Actinobacteriota | c__Actinobacteria | o__Corynebacteriales | f__Nocardiaceae | g__Nocardia |  |
| 2a6381987d389a06484968a9f1a7aae4 | Bacteria | p__Patescibacteria | c__Parcubacteria | o__Candidatus_Kaiserbacteria | f__Candidatus_Kaiserbacteria | g__Candidatus_Kaiserbacteria | s__groundwater_metagenome |
| 4204875a389bf74bf71b5ba0a5ab48a6 | Bacteria | p__Proteobacteria | c__Alphaproteobacteria | o__Sphingomonadales | f__Sphingomonadaceae |  |  |
| 606f508f65c79fb0c4665cc3a14f5aca | Bacteria | p__Proteobacteria | c__Gammaproteobacteria | o__Pseudomonadales | f__Moraxellaceae | g__Acinetobacter |  |
| 592141a05dc652b39cb1977cf026b204 | Bacteria | p__Proteobacteria | c__Gammaproteobacteria | o__Burkholderiales | f__Alcaligenaceae | g__Achromobacter |  |
| e7b4d4b084cc76479d7c250e9bbea6d5 | Bacteria | p__Chloroflexi |  |  |  |  |  |
| 8b118c9ee47aa281fe4b67fa8dbc721b | Bacteria | p__Actinobacteriota | c__Actinobacteria | o__Corynebacteriales | f__Mycobacteriaceae | g__Mycobacterium |  |
| 5fe53f47e78f0b0e655e1787914705d3 | Bacteria | p__Proteobacteria | c__Gammaproteobacteria | o__Pseudomonadales | f__Pseudomonadaceae | g__Pseudomonas |  |
| a37e3767a732d1b7e91fc14d54131cbd | Bacteria | p__Firmicutes | c__Bacilli | o__Lactobacillales | f__Lactobacillaceae | g__Lactobacillus |  |
| AJ316316 | Bacteria | p__Firmicutes | c__Bacilli | o__Bacillales | f__Bacillaceae | g__Bacillus |  |
| RS-GCF-003386735.1-NZ-QTTZ01000001.1 | Bacteria | p__Actinobacteriota | c__Actinobacteria | o__Micrococcales | f__Microbacteriaceae | g__Microbacterium |  |
| KM888184 | Bacteria | p__Proteobacteria | c__Gammaproteobacteria | o__Pseudomonadales | f__Pseudomonadaceae | g__Pseudomonas |  |
| cde769d2b7c6ec098185ddf7109fd59c | Bacteria | p__Proteobacteria | c__Gammaproteobacteria | o__Burkholderiales | f__Burkholderiaceae |  |  |
| c95914989fec51a1de6eae07c58a7d82 | Bacteria | p__Chloroflexi | c__Ktedonobacteria | o__Ktedonobacterales | f__Ktedonobacteraceae |  |  |
| 04909822 | Bacteria | p__Proteobacteria | c__Alphaproteobacteria | o__Rhizobiales | f__Devosiaceae | g__Devosia |  |
| 285b0351790a35847be2d1784906eaa7 | Bacteria | p__Proteobacteria | c__Gammaproteobacteria | o__Pseudomonadales | f__Moraxellaceae | g__Acinetobacter |  |
| 05896061 | Bacteria | p__Gemmatimonadota | c__Gemmatimonadetes | o__Gemmatimonadales | f__Gemmatimonadaceae |  |  |
| be783a90fdd36a41ae3d4bdb7194d2b3 | Bacteria | p__Acidobacteriota | c__Acidobacteriae | o__Bryobacterales | f__Bryobacteraceae | g__Bryobacter |  |
| RS-GCF-900171785.1-NZ-FWHD01000031.1 | Bacteria | p__Proteobacteria | c__Gammaproteobacteria | o__Pseudomonadales | f__Moraxellaceae | g__Acinetobacter |  |
| 11979502 | Bacteria | p__Proteobacteria | c__Alphaproteobacteria | o__Rhodobacterales | f__Rhodobacteraceae | g__Ketogulonicigenium |  |
| 04962120 | Bacteria | p__Actinobacteriota | c__Actinobacteria | o__Propionibacteriales | f__Nocardioidaceae | g__Nocardioides |  |
| 2f32606f4177cf8c452b13be5a4e6586 | Bacteria | p__Firmicutes | c__Bacilli | o__Lactobacillales | f__Enterococcaceae | g__Enterococcus |  |
| MJ033-1-barcode47-umi18350bins-ubs-14 | Bacteria | p__Acidobacteriota | c__Subgroup_11 | o__Subgroup_11 | f__Subgroup_11 | g__Subgroup_11 | s__uncultured_bacterium |
| 846ce84941bb21c0e7f88f16b98f36e2 | Bacteria | p__Nitrospirota | c__Nitrospiria | o__Nitrospirales | f__Nitrospiraceae | g__Nitrospira |  |
| GU459067 | Bacteria | p__Firmicutes | c__Bacilli | o__Lactobacillales | f__Carnobacteriaceae | g__Desemzia |  |
| a2e9220a8c066707592a83cf7b742b4e | Bacteria | p__Chloroflexi | c__Dehalococcoidia | o__S085 | f__S085 | g__S085 | s__uncultured_bacterium |
| 4a11a8e26c2cbcba534abbebbd3e841f | Bacteria | p__Bdellovibrionota | c__Bdellovibrionia | o__Bdellovibrionales | f__Bdellovibrionaceae | g__Bdellovibrio | s__Bdellovibrio_bacteriovorus |
| 56e71bf03917d35577d5816e2f782d27 | Bacteria | p__Bacteroidota | c__Bacteroidia | o__Sphingobacteriales | f__Sphingobacteriaceae | g__Sphingobacterium |  |
| 2bf8a034acfffc6ae30b72a945d5141c | Bacteria | p__Proteobacteria | c__Gammaproteobacteria | o__Burkholderiales | f__SC-I-84 | g__SC-I-84 |  |
| KT003984 | Bacteria | p__Bacteroidota | c__Bacteroidia | o__Cytophagales | f__Spirosomaceae | g__Dyadobacter |  |
| 5ff3e497517521c7e7c287c11182dcad | Bacteria | p__Myxococcota | c__Polyangia | o__Haliangiales | f__Haliangiaceae | g__Haliangium |  |
| MJ034-1-barcode44-umi31465bins-ubs-3 | Bacteria | p__Acidobacteriota | c__Acidobacteriae | o__Acidobacteriales | f__Acidobacteriaceae_(Subgroup_1) | g__Granulicella |  |
| 04959737 | Bacteria | p__Proteobacteria | c__Gammaproteobacteria | o__Burkholderiales | f__Comamonadaceae | g__Aquabacterium |  |
| 23047124 | Bacteria | p__Bacteroidota | c__Bacteroidia | o__Bacteroidales | f__Marinifilaceae | g__Odoribacter | s__unidentified |
| KC252615 | Bacteria | p__Proteobacteria | c__Alphaproteobacteria | o__Sphingomonadales | f__Sphingomonadaceae | g__Sphingomonas |  |
| MJ030-2-barcode58-umi39739bins-ubs-19 | Bacteria | p__Firmicutes | c__Clostridia | o__Clostridiales | f__Clostridiaceae | g__Clostridium_sensu_stricto_8 | s__uncultured_Firmicutes |
| a529409d8938da78d03598511d2577f7 | Bacteria | p__Chloroflexi | c__Ktedonobacteria | o__Ktedonobacterales | f__Ktedonobacteraceae | g__FCPS473 |  |
| MJ010-1-barcode29-umi103889bins-ubs-6 | Bacteria | p__Proteobacteria | c__Gammaproteobacteria | o__Pseudomonadales | f__Moraxellaceae | g__Acinetobacter | s__Acinetobacter_variabilis |
| 04786833 | Bacteria | p__Firmicutes | c__Bacilli | o__Erysipelotrichales | f__Erysipelotrichaceae | g__Turicibacter | s__uncultured_bacterium |
| 335d93efbc973cfee9e715c21ae7d75f | Bacteria | p__Proteobacteria | c__Gammaproteobacteria | o__Pseudomonadales | f__Moraxellaceae | g__Acinetobacter |  |
| GB-GCA-016713235.1-JADJPN010000004.1 | Bacteria | p__Proteobacteria | c__Alphaproteobacteria | o__Rhizobiales |  |  |  |
| 1aa6c4275a7a401cd6df11d0edb93034 | Bacteria | p__Actinobacteriota | c__Actinobacteria | o__Micrococcales | f__Intrasporangiaceae |  |  |
| U61298 | Bacteria | p__Actinobacteriota | c__Actinobacteria | o__Propionibacteriales | f__Nocardioidaceae | g__Nocardioides |  |
| RS-GCF-000334915.1-NZ-AMSF01000089.1 | Bacteria | p__Proteobacteria | c__Gammaproteobacteria | o__Xanthomonadales | f__Rhodanobacteraceae | g__Dyella |  |
| MJ005-barcode27-umi25984bins-ubs-9 | Bacteria | p__Fusobacteriota | c__Fusobacteriia | o__Fusobacteriales | f__Leptotrichiaceae | g__Leptotrichia |  |
| GB-GCA-009719285.1-WNCD01000184.1 | Bacteria | p__Verrucomicrobiota | c__Verrucomicrobiae | o__Verrucomicrobiales | f__Akkermansiaceae | g__Akkermansia |  |
| 58e4ff1e43bd0aae6dc40d2cf2ed46af | Bacteria | p__Proteobacteria | c__Gammaproteobacteria | o__Burkholderiales | f__Rhodocyclaceae | g__Thauera |  |
| d4870eddaabcd84ffb197694d7b4c1cd | Bacteria | p__Actinobacteriota | c__Actinobacteria | o__Frankiales | f__Sporichthyaceae | g__Longivirga |  |
| 9c49f2d2e677d6826182529e390436c6 | Bacteria | p__Actinobacteriota | c__Rubrobacteria | o__Rubrobacterales | f__Rubrobacteriaceae | g__Rubrobacter | s__Rubrobacter_bracarensis |
| 18676214 | Bacteria | p__Actinobacteriota | c__Actinobacteria | o__Kineosporiales | f__Kineosporiaceae | g__Pseudokineococcus | s__Pseudokineococcus_lusitanus |
| 39fcb96a5a3977464d5c38c04c4c65b7 | Bacteria | p__Proteobacteria | c__Alphaproteobacteria | o__SAR11_clade | f__Clade_III | g__Clade_III |  |
| f0bde26a7b4c3c580b882a53e8bbbfae | Bacteria | p__Bacteroidota | c__Bacteroidia | o__Chitinophagales | f__Chitinophagaceae |  |  |
| b0f835d49e14eb06596ca914c7695468 | Bacteria | p__Acidobacteriota | c__Acidobacteriae | o__Subgroup_2 | f__Subgroup_2 | g__Subgroup_2 |  |
| KC237397 | Bacteria | p__Proteobacteria | c__Alphaproteobacteria | o__Rhizobiales | f__Rhizobiaceae | g__Mesorhizobium |  |
| AF288309 | Bacteria | p__Proteobacteria | c__Alphaproteobacteria | o__Rhizobiales | f__Beijerinckiaceae | g__Bosea |  |
| 14801525 | Bacteria | p__Chloroflexi | c__Ktedonobacteria | o__Ktedonobacterales | f__JG30-KF-AS9 | g__JG30-KF-AS9 | s__uncultured_bacterium |
| 04959736 | Bacteria | p__Proteobacteria | c__Gammaproteobacteria | o__Burkholderiales | f__Comamonadaceae | g__Aquabacterium |  |
| 04994838 | Bacteria | p__Proteobacteria | c__Alphaproteobacteria | o__Rhizobiales | f__Rhizobiaceae | g__Aureimonas |  |
| 362509ac94bb678ebd0149970bd6e0d3 | Bacteria | p__Proteobacteria | c__Alphaproteobacteria | o__Rhizobiales | f__Rhizobiaceae | g__Allorhizobium-Neorhizobium-Pararhizobium-Rhizobium |  |
| 60808df6b98489a179acbd9ff9b77b99 | Bacteria | p__Proteobacteria | c__Gammaproteobacteria | o__Cellvibrionales | f__Cellvibrionaceae | g__Cellvibrio |  |
| GB-GCA-016703685.1-JADJBO010000002.1 | Bacteria | p__Bacteroidota | c__Bacteroidia | o__Flavobacteriales | f__NS9_marine_group | g__NS9_marine_group |  |
| 04665810 | Bacteria | p__Proteobacteria | c__Gammaproteobacteria | o__Pasteurellales | f__Pasteurellaceae |  |  |
| 5862c7c77824eab282d7a7d82f16eac5 | Bacteria | p__Proteobacteria | c__Alphaproteobacteria | o__Rhizobiales | f__Rhizobiaceae | g__Ensifer |  |
| 68708ca40463808c8ecfe8843b072324 | Bacteria | p__Actinobacteriota | c__Actinobacteria | o__Corynebacteriales | f__Mycobacteriaceae | g__Mycobacterium |  |
| 04995021 | Bacteria | p__Proteobacteria | c__Alphaproteobacteria | o__Rhizobiales | f__Rhizobiaceae |  |  |
| 977c852c24eca4e77edccb048b2084ba | Bacteria | p__Actinobacteriota | c__Actinobacteria | o__Bifidobacteriales | f__Bifidobacteriaceae | g__Bifidobacterium |  |
| 04919091 | Bacteria | p__Bacteroidota | c__Bacteroidia | o__Bacteroidales | f__Tannerellaceae | g__Parabacteroides |  |
| 9f1d8dfeec06af2fff700fbdcc520577 | Bacteria | p__Dadabacteria | c__Dadabacteriia | o__Dadabacteriales | f__Dadabacteriales | g__Dadabacteriales |  |
| GB-GCA-008669045.1-RQAJ01000001.1 | Bacteria | p__Bacteroidota | c__Bacteroidia | o__Bacteroidales | f__Bacteroidaceae | g__Bacteroides |  |
| MJ010-1-barcode29-umi125660bins-ubs-4 | Bacteria | p__Bacteroidota | c__Bacteroidia | o__Chitinophagales | f__Chitinophagaceae | g__Taibaiella | s__uncultured_bacterium |
| 04880997 | Bacteria | p__Actinobacteriota | c__Actinobacteria | o__Actinomycetales | f__Actinomycetaceae | g__Actinomyces |  |
| 62a4fe858a754f4eca97b8508e5b59d5 | Bacteria | p__Bacteroidota | c__Ignavibacteria | o__Ignavibacteriales | f__PHOS-HE36 | g__PHOS-HE36 |  |
| MJ006-1-barcode39-umi107659bins-ubs-4 | Bacteria | p__Firmicutes | c__Clostridia | o__Oscillospirales | f__Oscillospiraceae | g__UCG-005 |  |
| f8e0c7cbb133d6c717488ab80c0a5c9f | Bacteria | p__Proteobacteria | c__Alphaproteobacteria | o__Rhodobacterales | f__Rhodobacteraceae |  |  |
| 11142449 | Bacteria | p__Bacteroidota | c__Bacteroidia | o__Bacteroidales | f__Muribaculaceae |  |  |
| 4b52f3fd2ac6fb50d35a9031e25764b8 | Bacteria | p__Bacteroidota | c__Bacteroidia | o__Bacteroidales | f__Muribaculaceae | g__Muribaculaceae |  |
| 22856a1a430fe30b0e1bab9eeecde757 | Bacteria | p__Acidobacteriota | c__Acidobacteriae | o__Subgroup_2 | f__Subgroup_2 | g__Subgroup_2 |  |
| 97fb907d9514a9506dba6feeec21cd39 | Bacteria | p__Proteobacteria | c__Gammaproteobacteria | o__Pseudomonadales | f__Moraxellaceae | g__Acinetobacter |  |
| ed276fe77c81c9173ae08f82b00bc76f | Bacteria | p__Acidobacteriota | c__Subgroup_22 | o__Subgroup_22 | f__Subgroup_22 | g__Subgroup_22 |  |
| d103d510426baebcecf0e6005f56c7fb | Bacteria | p__Bacteroidota | c__Bacteroidia | o__Bacteroidales | f__Muribaculaceae | g__Muribaculaceae | s__uncultured_Bacteroidales |
| ea0f4ba8b40be8e980ea58e37b9703af | Bacteria | p__Firmicutes | c__Bacilli | o__Bacillales | f__Bacillaceae | g__Bacillus |  |
| da0ed34d1821abc893effa9ffe1a56a7 | Bacteria | p__Bacteroidota | c__Bacteroidia | o__Bacteroidales | f__Muribaculaceae | g__Muribaculaceae | s__uncultured_organism |
| b8edde35e0f76fb1c28087616f9c4ff2 | Bacteria | p__Actinobacteriota | c__Thermoleophilia | o__Gaiellales | f__uncultured | g__uncultured |  |
| FJ267649 | Bacteria | p__Proteobacteria | c__Gammaproteobacteria | o__Burkholderiales | f__Oxalobacteraceae | g__Herbaspirillum |  |
| 04834117 | Bacteria | p__Proteobacteria | c__Gammaproteobacteria | o__Pseudomonadales | f__Pseudomonadaceae | g__Pseudomonas |  |
| 3f8a13cab8f3babb0269f55844f311a5 | Bacteria | p__Firmicutes | c__Bacilli | o__Lactobacillales | f__Enterococcaceae | g__Enterococcus |  |
| a3a7681be2acb2e1209d6b87d7ac320e | Bacteria | p__Proteobacteria | c__Gammaproteobacteria | o__Burkholderiales | f__Comamonadaceae | g__Pelomonas |  |
| GB-GCA-902636615.1-CACPSF010000100.1 | Bacteria | p__Actinobacteriota | c__Actinobacteria | o__Micrococcales | f__Micrococcaceae |  |  |
| 21e55af13b5d825ca7faf8e5db22802c | Bacteria | p__Methylomirabilota | c__Methylomirabilia | o__Rokubacteriales | f__Rokubacteriales | g__Rokubacteriales | s__uncultured_bacterium |
| MJ006-2-barcode62-umi164579bins-ubs-4 | Bacteria | p__Firmicutes | c__Clostridia | o__Lachnospirales | f__Lachnospiraceae |  |  |
| b753e7af1caee7c5f0300ced6c1b0cef | Bacteria | p__Proteobacteria | c__Gammaproteobacteria | o__Xanthomonadales | f__Xanthomonadaceae | g__Lysobacter |  |
| bdaf31b7ee9ab185ae30ee605ae04e25 | Bacteria | p__Proteobacteria | c__Gammaproteobacteria | o__Burkholderiales | f__Burkholderiaceae | g__Burkholderia-Caballeronia-Paraburkholderia |  |
| 8b50c5339ce544c40111319efbdc066a | Bacteria | p__Verrucomicrobiota | c__Verrucomicrobiae | o__Chthoniobacterales |  |  |  |
| 9e52593bd8a2cff5ae1ca2b916a7cafb | Bacteria | p__Acidobacteriota | c__Acidobacteriae |  |  |  |  |
| 05019712 | Bacteria | p__Acidobacteriota | c__Acidobacteriae | o__Bryobacterales | f__Bryobacteraceae | g__Bryobacter |  |
| e923d9929c58ee3664a070a4ddc758f4 | Bacteria | p__Firmicutes | c__Bacilli | o__Exiguobacterales | f__Exiguobacteraceae | g__Exiguobacterium |  |
| KF906845 | Bacteria | p__Actinobacteriota | c__Actinobacteria | o__Micrococcales | f__Microbacteriaceae |  |  |
| 7311b95010f2a918b6cc9b596be98377 | Bacteria | p__Firmicutes | c__Bacilli | o__Exiguobacterales | f__Exiguobacteraceae | g__Exiguobacterium |  |
| 3f3d912dffb404e73d4fa64b308e37c6 | Bacteria | p__Acidobacteriota | c__Acidobacteriae | o__Acidobacteriales | f__Acidobacteriaceae_(Subgroup_1) | g__Occallatibacter |  |
| 64e7d6fd25de6790895afca0895051f8 | Bacteria | p__Actinobacteriota | c__Actinobacteria | o__Corynebacteriales | f__Nocardiaceae | g__Rhodococcus |  |
| 41f7aa22a7b1558abc3a0ce62c833568 | Bacteria | p__Bacteroidota | c__Bacteroidia | o__Sphingobacteriales | f__Sphingobacteriaceae | g__Sphingobacterium |  |
| e84ff1d560dac428cf1555e64561ea63 | Bacteria | p__Actinobacteriota | c__Thermoleophilia | o__Solirubrobacterales | f__Solirubrobacteraceae | g__Patulibacter | s__Patulibacter_minatonensis |
| 5a6050d2e008148b05c73169c1eca2f0 | Bacteria | p__Firmicutes | c__Syntrophomonadia | o__Syntrophomonadales | f__Syntrophomonadaceae | g__Syntrophomonas |  |
| MJ007-1-barcode25-umi1063bins-ubs-223 | Bacteria | p__Proteobacteria | c__Gammaproteobacteria | o__Enterobacterales |  |  |  |
| 68a1b38348c44b3df1f31c7a9aea84b6 | Bacteria | p__Firmicutes | c__Bacilli | o__Bacillales |  |  |  |
| c8615be2e45649a8e6c58df9226e3c4b | Bacteria | p__Chloroflexi | c__Chloroflexia | o__Thermomicrobiales | f__JG30-KF-CM45 | g__JG30-KF-CM45 |  |
| AB248285 | Bacteria | p__Proteobacteria | c__Alphaproteobacteria | o__Sphingomonadales | f__Sphingomonadaceae | g__Sphingomonas |  |
| 4bd5eb5d7a433bdb2ac107a106e57dd7 | Bacteria | p__Gemmatimonadota | c__Gemmatimonadetes | o__Gemmatimonadales | f__Gemmatimonadaceae | g__uncultured |  |
| 04958394 | Bacteria | p__Actinobacteriota | c__Actinobacteria | o__Micrococcales | f__Microbacteriaceae |  |  |
| AB594201 | Bacteria | p__Proteobacteria | c__Alphaproteobacteria | o__Acetobacterales | f__Acetobacteraceae | g__Roseomonas |  |
| c3deddb188f8fd6d704c23ceda791764 | Bacteria | p__Actinobacteriota | c__Actinobacteria | o__Catenulisporales | f__Catenulisporaceae | g__Catenulispora | s__Catenulispora_acidiphila |
| RS-GCF-001406355.1-NZ-CYYL01000043.1 | Bacteria | p__Firmicutes | c__Clostridia | o__Oscillospirales | f__Ruminococcaceae | g__Faecalibacterium |  |
| 916f41768fe485ba4e4bccec4d67a461 | Bacteria | p__Myxococcota | c__bacteriap25 | o__bacteriap25 | f__bacteriap25 | g__bacteriap25 | s__unidentified |
| 16396384 | Bacteria | p__Firmicutes | c__Clostridia | o__Lachnospirales | f__Lachnospiraceae |  |  |
| 225ef9ba1ea4a8e111f4f3d4418f71dd | Bacteria | p__Actinobacteriota | c__Actinobacteria | o__Propionibacteriales | f__Nocardioidaceae |  |  |
| MJ030-2-barcode58-umi85688bins-ubs-5 | Bacteria | p__Proteobacteria | c__Alphaproteobacteria | o__Rhizobiales | f__Rhizobiales_Incertae_Sedis | g__uncultured |  |
| 046edaab981e37c3dda75e3d40068cc2 | Bacteria | p__Proteobacteria | c__Gammaproteobacteria | o__Xanthomonadales | f__Xanthomonadaceae | g__Pseudoxanthomonas |  |
| 05041802 | Bacteria | p__Firmicutes | c__Bacilli | o__Lactobacillales | f__Leuconostocaceae | g__Leuconostoc |  |
| MJ009-1-barcode48-umi64870bins-ubs-5 | Bacteria | p__Firmicutes | c__Clostridia | o__Lachnospirales | f__Lachnospiraceae | g__[Eubacterium]_xylanophilum_group |  |
| 04934464 | Bacteria | p__Bacteroidota | c__Bacteroidia | o__Chitinophagales | f__Chitinophagaceae | g__Taibaiella | s__uncultured_bacterium |
| RS-GCF-001691555.1-NZ-MAYN01000008.1--2 | Bacteria | p__Proteobacteria | c__Gammaproteobacteria | o__Enterobacterales |  |  |  |
| MJ034-1-barcode39-umi5633bins-ubs-81 | Bacteria | p__Bacteroidota | c__Bacteroidia | o__Chitinophagales | f__Chitinophagaceae | g__Ferruginibacter |  |
| 04953167 | Bacteria | p__Actinobacteriota | c__Thermoleophilia | o__Gaiellales | f__uncultured | g__uncultured | s__uncultured_bacterium |
| 9b55a75b013870f80c88307c1c8665bb | Bacteria | p__Chloroflexi | c__Chloroflexia | o__Thermomicrobiales | f__JG30-KF-CM45 | g__JG30-KF-CM45 | s__metagenome |
| 1b38d9eee6b67770ea9f9fc9e7277e6f | Bacteria | p__Patescibacteria | c__Parcubacteria | o__Candidatus_Moranbacteria | f__Candidatus_Moranbacteria | g__Candidatus_Moranbacteria |  |
| 3b4b7849d67b8ac998da5b1aba613d1e | Bacteria | p__Acidobacteriota | c__Acidobacteriae | o__Acidobacteriales | f__Acidobacteriaceae_(Subgroup_1) | g__Occallatibacter |  |
| b18754f1ccbf055fe674b71439c28b40 | Bacteria | p__Firmicutes | c__Clostridia | o__Lachnospirales | f__Lachnospiraceae | g__Blautia |  |
| 844f4d6dbe42a3f1731e526609f7d63d | Bacteria | p__Actinobacteriota | c__Acidimicrobiia | o__IMCC26256 | f__IMCC26256 | g__IMCC26256 |  |
| 4d4dd51fef61d777a50c9a916284764e | Bacteria | p__Acidobacteriota | c__Vicinamibacteria | o__Vicinamibacterales | f__Vicinamibacteraceae | g__Vicinamibacteraceae |  |
| RS-GCF-004118035.1-NZ-MSXY01000096.1 | Bacteria | p__Firmicutes | c__Bacilli | o__Bacillales | f__Bacillaceae | g__Bacillus |  |
| MJ033-1-barcode26-umi31721bins-ubs-6 | Bacteria | p__Firmicutes | c__Clostridia | o__Lachnospirales | f__Lachnospiraceae | g__Lachnoclostridium |  |
| c2c5ece6e77f4e619924eb76139f9b1a | Bacteria | p__Proteobacteria | c__Gammaproteobacteria | o__Xanthomonadales | f__Rhodanobacteraceae | g__Dokdonella | s__uncultured_Dokdonella |
| 02471193 | Bacteria | p__Acidobacteriota | c__Vicinamibacteria | o__Vicinamibacterales | f__Vicinamibacteraceae | g__Vicinamibacteraceae |  |
| RS-GCF-002243155.1-NZ-NDYH01000035.1 | Bacteria | p__Firmicutes | c__Clostridia | o__Peptostreptococcales-Tissierellales | f__Peptostreptococcales-Tissierellales | g__Finegoldia |  |
| 4cc8751ba01ffcb67e317478846871eb | Bacteria | p__Actinobacteriota | c__Actinobacteria | o__Micrococcales | f__Microbacteriaceae |  |  |
| c277611d256acd2a71a4019ac239cb8a | Bacteria | p__Chloroflexi | c__Anaerolineae | o__Caldilineales | f__Caldilineaceae |  |  |
| 02387328 | Bacteria | p__Actinobacteriota | c__Actinobacteria | o__Propionibacteriales | f__Nocardioidaceae |  |  |
| 02323616 | Bacteria | p__Actinobacteriota | c__Actinobacteria | o__Corynebacteriales | f__Dietziaceae | g__Dietzia |  |
| GB-GCA-003241315.1-QFOZ01000022.1 | Bacteria | p__Actinobacteriota | c__Actinobacteria | o__Corynebacteriales | f__Corynebacteriaceae | g__Lawsonella |  |
| 2a2b7b36d6ce78329e3a39e5070fa063 | Bacteria | p__Patescibacteria | c__Saccharimonadia | o__Saccharimonadales | f__Saccharimonadaceae |  |  |
| 04960869 | Bacteria | p__Actinobacteriota | c__Actinobacteria | o__Corynebacteriales | f__Corynebacteriaceae | g__Corynebacterium |  |
| 080a0aab259bce0b92a9ec8b0761b5e1 | Bacteria | p__Bacteroidota | c__Bacteroidia | o__Flavobacteriales | f__Weeksellaceae | g__Chryseobacterium |  |
| f91df05ddf35e79a4d3f1917d44c76ef | Bacteria | p__Chloroflexi | c__Anaerolineae | o__SBR1031 | f__A4b | g__A4b |  |
| 00a34af243412516e239d39a8e06cc53 | Bacteria | p__Acidobacteriota | c__Holophagae | o__Subgroup_7 | f__Subgroup_7 | g__Subgroup_7 |  |
| MJ030-1-barcode42-umi41119bins-ubs-12 | Bacteria | p__Proteobacteria | c__Gammaproteobacteria | o__Steroidobacterales | f__Steroidobacteraceae | g__Steroidobacter |  |
| 4fdefea282a641c61ca62e63a388a0aa | Bacteria | p__Bacteroidota | c__Bacteroidia | o__Chitinophagales | f__Chitinophagaceae |  |  |
| 04945744 | Bacteria | p__Actinobacteriota | c__Actinobacteria | o__Micrococcales | f__Micrococcaceae |  |  |
| MJ034-1-barcode48-umi2395bins-ubs-183 | Bacteria | p__Bacteroidota | c__Bacteroidia | o__Sphingobacteriales | f__Sphingobacteriaceae |  |  |
| 04906806 | Bacteria | p__Proteobacteria | c__Alphaproteobacteria | o__Rhizobiales | f__Beijerinckiaceae | g__Psychroglaciecola | s__uncultured_bacterium |
| 20478299 | Bacteria | p__Chloroflexi | c__Dehalococcoidia | o__S085 | f__S085 | g__S085 | s__metagenome |
| 55b64a1f5b184f2606610c9433a261e0 | Bacteria | p__Proteobacteria | c__Alphaproteobacteria | o__Rhizobiales | f__Xanthobacteraceae |  |  |
| 58062ea628a88186ab45c3fdefc31bea | Bacteria | p__Proteobacteria | c__Gammaproteobacteria | o__Enterobacterales | f__Enterobacteriaceae |  |  |
| bc996af9bef1d57ea466f0d190db0890 | Bacteria | p__Actinobacteriota | c__Actinobacteria | o__Micromonosporales | f__Micromonosporaceae |  |  |
| 910a94d71fab33cd89f0afc9de381b10 | Bacteria | p__Firmicutes | c__Limnochordia | o__MBA03 | f__MBA03 | g__MBA03 | s__uncultured_bacterium |
| 07765769 | Bacteria |  |  |  |  |  |  |
| b9c7f3708cb083251339f11271b7b5f8 | Bacteria | p__Actinobacteriota | c__Actinobacteria | o__Frankiales | f__Geodermatophilaceae | g__Klenkia |  |
| 08627691 | Bacteria | p__Proteobacteria | c__Alphaproteobacteria | o__Rhizobiales | f__Rhizobiaceae |  |  |
| 1ed3f8e2fbfe0bde29997bea52ffd0b9 | Bacteria | p__Firmicutes | c__Bacilli | o__Lactobacillales | f__Enterococcaceae | g__Enterococcus |  |
| 3f6fabcfe94fb43507730648bf832e3d | Bacteria | p__Firmicutes | c__Bacilli | o__Staphylococcales | f__Staphylococcaceae | g__Staphylococcus |  |
| HQ123435 | Bacteria | p__Actinobacteriota | c__Actinobacteria | o__Micromonosporales | f__Micromonosporaceae |  |  |
| 66bd729e2f2f178fe75ed0d906c205c0 | Bacteria | p__Proteobacteria | c__Alphaproteobacteria | o__Azospirillales | f__Azospirillaceae | g__Skermanella |  |
| 6de2104f0582dd7737fdcfe30af5f65e | Bacteria | p__Acidobacteriota | c__Acidobacteriae | o__AKIW659 | f__AKIW659 | g__AKIW659 |  |
| 4a4f44f37926f74288018e5fba798b51 | Bacteria | p__Acidobacteriota | c__Vicinamibacteria | o__Vicinamibacterales | f__uncultured | g__uncultured | s__uncultured_bacterium |
| 04933430 | Bacteria | p__Bacteroidota | c__Bacteroidia | o__Chitinophagales | f__Chitinophagaceae |  |  |
| ecb692355b46d90569f9d946d20769aa | Bacteria | p__Actinobacteriota | c__Acidimicrobiia | o__IMCC26256 | f__IMCC26256 | g__IMCC26256 |  |
| 20952721 | Bacteria | p__Firmicutes | c__Bacilli | o__Erysipelotrichales | f__Erysipelotrichaceae | g__Dubosiella | s__uncultured_bacterium |
| 04956022 | Bacteria | p__Actinobacteriota | c__Thermoleophilia | o__Gaiellales | f__uncultured | g__uncultured | s__uncultured_actinobacterium |
| 585822cad202587d5bcabd154f4d6bfc | Bacteria | p__Proteobacteria | c__Alphaproteobacteria | o__Rhizobiales | f__Xanthobacteraceae |  |  |
| 14801761 | Bacteria | p__Chloroflexi | c__Ktedonobacteria | o__Ktedonobacterales | f__JG30-KF-AS9 | g__JG30-KF-AS9 | s__uncultured_bacterium |
| 897465dd9fb4dde3ab4a5a08d08ab51b | Bacteria | p__Firmicutes | c__Bacilli | o__Lactobacillales | f__Enterococcaceae | g__Enterococcus |  |
| c988155e6311ec031835f3ff7a9c19c9 | Bacteria |  |  |  |  |  |  |
| 05011460 | Bacteria | p__Proteobacteria | c__Gammaproteobacteria | o__Enterobacterales | f__Enterobacteriaceae | g__Escherichia-Shigella |  |
| 04934624 | Bacteria | p__Bacteroidota | c__Bacteroidia | o__Flavobacteriales | f__Weeksellaceae | g__Cloacibacterium |  |
| abafae31b7d57cda06f8571e4a4bd18c | Bacteria | p__Verrucomicrobiota | c__Verrucomicrobiae | o__Chthoniobacterales | f__Chthoniobacteraceae | g__Candidatus_Udaeobacter | s__Spartobacteria_bacterium |
| c3bcbd4b42166581345709d4f3a6ccfa | Bacteria | p__Planctomycetota | c__Planctomycetes | o__Isosphaerales | f__Isosphaeraceae | g__Singulisphaera |  |
| AB021366 | Bacteria | p__Proteobacteria | c__Gammaproteobacteria | o__Burkholderiales | f__Oxalobacteraceae | g__Herbaspirillum |  |
| MJ030-1-barcode25-umi104025bins-ubs-3 | Bacteria | p__Bacteroidota | c__Bacteroidia | o__Chitinophagales | f__Chitinophagaceae | g__Ferruginibacter |  |
| 993b307bd313b19585f017e752564b02 | Bacteria | p__Proteobacteria | c__Gammaproteobacteria | o__Pseudomonadales | f__Moraxellaceae | g__Acinetobacter |  |
| 021d9bd18bbc09c4b87222e4b34b1876 | Bacteria | p__Bacteroidota | c__Bacteroidia | o__Sphingobacteriales | f__Sphingobacteriaceae |  |  |
| 0b629016b91a1ca89893a79c1cf30562 | Bacteria | p__Proteobacteria | c__Gammaproteobacteria | o__Enterobacterales | f__Erwiniaceae |  |  |
| RS-GCF-001455265.1-NZ-LKPE01000028.1 | Bacteria | p__Firmicutes | c__Bacilli | o__Lactobacillales | f__Streptococcaceae | g__Lactococcus |  |
| 40110d8078b6c0b929b3a20721fd58b8 | Bacteria | p__Bacteroidota | c__Bacteroidia | o__Flavobacteriales | f__Weeksellaceae | g__Elizabethkingia | s__Elizabethkingia_meningoseptica |
| caa0b66ff78ff639554a31229d723522 | Bacteria | p__Actinobacteriota | c__Actinobacteria | o__Micrococcales | f__Microbacteriaceae | g__Microbacterium |  |
| d5728faeffe91ed19959482869e404b8 | Bacteria | p__Firmicutes | c__Clostridia | o__Oscillospirales | f__Ruminococcaceae |  |  |
| MJ020-1-barcode41-umi90550bins-ubs-4 | Bacteria | p__Firmicutes | c__Bacilli | o__Bacillales | f__Bacillaceae | g__Bacillus |  |
| MJ030-2-barcode65-umi93084bins-ubs-4 | Bacteria | p__Acidobacteriota | c__Acidobacteriae | o__Acidobacteriales | f__uncultured | g__uncultured |  |
| RS-GCF-001029205.1-NZ-LELF01000062.1--2 | Bacteria | p__Firmicutes | c__Bacilli | o__Paenibacillales | f__Paenibacillaceae | g__Paenibacillus |  |
| 2d353bac8c2072f3f6f5272785b0725f | Bacteria | p__Chloroflexi | c__KD4-96 | o__KD4-96 | f__KD4-96 | g__KD4-96 | s__metagenome |
| 94e1c331c977ad5f7da9b165a9dd35c4 | Bacteria | p__Proteobacteria | c__Alphaproteobacteria | o__Azospirillales | f__uncultured | g__uncultured | s__uncultured_bacterium |
| 083dd6664a689b2743eca0406c605a31 | Bacteria | p__Proteobacteria | c__Gammaproteobacteria | o__Pseudomonadales | f__Moraxellaceae | g__Acinetobacter |  |
| d617256e98834f9b0d0157651aa5616a | Bacteria | p__Bdellovibrionota | c__Oligoflexia | o__0319-6G20 | f__0319-6G20 | g__0319-6G20 |  |
| 04989861 | Bacteria | p__Proteobacteria | c__Gammaproteobacteria | o__Pseudomonadales | f__Moraxellaceae | g__Acinetobacter |  |
| MJ031-1-barcode28-umi7327bins-ubs-64 | Bacteria | p__Proteobacteria | c__Alphaproteobacteria | o__Rhizobiales | f__Rhizobiales_Incertae_Sedis | g__uncultured |  |
| RS-GCF-004167065.1-NZ-RCYP01000066.1 | Bacteria | p__Bacteroidota | c__Bacteroidia | o__Bacteroidales | f__Tannerellaceae | g__Parabacteroides |  |
| c5769824b175e747584e4e01aebf41a0 | Bacteria | p__Firmicutes | c__Clostridia | o__Lachnospirales | f__Lachnospiraceae | g__Lachnoanaerobaculum |  |
| 7d02e35a4f75bfcf447b4ae9d68fd88f | Bacteria | p__Proteobacteria | c__Gammaproteobacteria | o__Burkholderiales | f__Nitrosomonadaceae | g__MND1 |  |
| 19937047 | Bacteria | p__Firmicutes | c__Clostridia | o__Oscillospirales | f__Oscillospiraceae | g__Colidextribacter |  |
| 7f937d5d7a917fea0a3336449dbbf389 | Bacteria | p__Bacteroidota | c__Bacteroidia | o__Bacteroidales | f__Muribaculaceae | g__Muribaculaceae | s__uncultured_bacterium |
| MJ033-1-barcode39-umi40468bins-ubs-4 | Bacteria | p__Proteobacteria | c__Gammaproteobacteria | o__Gammaproteobacteria_Incertae_Sedis | f__Unknown_Family | g__Acidibacter |  |
| 208097fecba7e162f3608ad3e2d6b5d0 | Bacteria | p__Bacteroidota | c__Bacteroidia | o__Flavobacteriales | f__Weeksellaceae | g__Chryseobacterium |  |
| 4c38445c3e80dfa23ebe0f632b7a72e5 | Bacteria | p__Patescibacteria | c__Saccharimonadia | o__Saccharimonadales |  |  |  |
| X77436 | Bacteria | p__Actinobacteriota | c__Actinobacteria | o__Micrococcales | f__Microbacteriaceae | g__Curtobacterium |  |
| AB681719 | Bacteria | p__Proteobacteria | c__Alphaproteobacteria | o__Sphingomonadales | f__Sphingomonadaceae | g__Sphingomonas |  |
| b7add3dc0688b29f0459dd1d2a41ed1e | Bacteria | p__Firmicutes | c__Bacilli | o__Paenibacillales | f__Paenibacillaceae | g__Paenibacillus |  |
| 02247889 | Bacteria | p__Proteobacteria | c__Alphaproteobacteria | o__Rhizobiales | f__Beijerinckiaceae | g__Camelimonas | s__uncultured_bacterium |
| 82f4e08c8cf1242f080b91c632c4aa09 | Bacteria | p__MBNT15 | c__MBNT15 | o__MBNT15 | f__MBNT15 | g__MBNT15 |  |
| 431c1e52e26bfb041b74e5745ffcbe53 | Bacteria | p__Firmicutes | c__Clostridia | o__Lachnospirales | f__Lachnospiraceae |  |  |
| e8601cf01d07ddb0c084ebe4c895e516 | Bacteria | p__Firmicutes | c__Clostridia | o__Peptostreptococcales-Tissierellales | f__Peptostreptococcaceae |  |  |
| 02390715 | Bacteria | p__Actinobacteriota | c__Acidimicrobiia | o__IMCC26256 | f__IMCC26256 | g__IMCC26256 | s__uncultured_Ferrimicrobium |
| 04909654 | Bacteria | p__Proteobacteria | c__Alphaproteobacteria | o__Rhizobiales | f__Rhizobiaceae | g__Allorhizobium-Neorhizobium-Pararhizobium-Rhizobium |  |
| 18696290 | Bacteria | p__Actinobacteriota | c__Actinobacteria | o__Frankiales | f__Geodermatophilaceae | g__Geodermatophilus |  |
| 6c0d0dc875b42f324394d4108c1245a5 | Bacteria | p__Actinobacteriota | c__Actinobacteria | o__Micrococcales | f__Intrasporangiaceae |  |  |
| RS-GCF-013417345.1-NZ-VPEE01000069.1 | Bacteria | p__Proteobacteria | c__Gammaproteobacteria | o__Pseudomonadales | f__Moraxellaceae | g__Acinetobacter |  |
| 11355292 | Bacteria | p__Acidobacteriota | c__Subgroup_25 | o__Subgroup_25 | f__Subgroup_25 | g__Subgroup_25 | s__uncultured_Acidobacteria |
| RS-GCF-016505385.1-NZ-JADVJK010000040.1 | Bacteria | p__Proteobacteria | c__Gammaproteobacteria | o__Enterobacterales | f__Enterobacteriaceae |  |  |
| a95f45f5cc0e791b78e7a5108d06e2a8 | Bacteria | p__Verrucomicrobiota | c__Verrucomicrobiae | o__Chthoniobacterales | f__Chthoniobacteraceae | g__Candidatus_Udaeobacter |  |
| 171bbc68db6391028a190e163864c710 | Bacteria | p__Actinobacteriota | c__Thermoleophilia | o__Gaiellales |  |  |  |
| 08188012fdc09db38a10da2077e9951d | Bacteria | p__Chloroflexi | c__Ktedonobacteria | o__Ktedonobacterales | f__JG30-KF-AS9 | g__JG30-KF-AS9 | s__uncultured_bacterium |
| MJ006-1-barcode36-umi231131bins-ubs-3 | Bacteria | p__Firmicutes | c__Clostridia | o__Lachnospirales | f__Lachnospiraceae | g__Blautia |  |
| MJ034-1-barcode30-umi16034bins-ubs-19 | Bacteria | p__Acidobacteriota | c__Vicinamibacteria | o__Vicinamibacterales | f__uncultured | g__uncultured | s__uncultured_bacterium |
| b1302484f0589a6beae4dc71df940ddb | Bacteria | p__Actinobacteriota | c__Actinobacteria | o__Frankiales | f__Sporichthyaceae |  |  |
| bbbd4bda99fd72768812f7ee8290673b | Bacteria | p__Firmicutes | c__Bacilli | o__Lactobacillales | f__Enterococcaceae | g__Enterococcus |  |
| 69623af84f0ba1461ba3506b238337bf | Bacteria | p__Firmicutes | c__Bacilli | o__Erysipelotrichales | f__Erysipelatoclostridiaceae | g__Erysipelatoclostridium | s__uncultured_Clostridium |
| 04927960 | Bacteria | p__Proteobacteria | c__Alphaproteobacteria | o__Sphingomonadales | f__Sphingomonadaceae | g__Sphingomonas |  |
| c1b726c39f10f50978f194b69a65a19f | Bacteria | p__Actinobacteriota | c__Thermoleophilia | o__Solirubrobacterales | f__Solirubrobacteraceae | g__Conexibacter |  |
| 25bdcd6152d3b647feeb60a5a8dc53a7 | Bacteria | p__Proteobacteria | c__Gammaproteobacteria | o__Burkholderiales | f__Comamonadaceae | g__Delftia |  |
| 9584e4465d52d115763dc6da4de0654c | Bacteria | p__Bacteroidota | c__Bacteroidia | o__Chitinophagales | f__Chitinophagaceae |  |  |
| 494cb05320c9cb4ff1abdbf632f07533 | Bacteria | p__Firmicutes | c__Clostridia | o__Clostridiales | f__Clostridiaceae | g__Clostridium_sensu_stricto_12 |  |
| 17050648 | Bacteria | p__Firmicutes | c__Clostridia | o__Lachnospirales | f__Lachnospiraceae | g__[Eubacterium]_xylanophilum_group |  |
| a56a4fc39cb136d509d8bf5018b5df68 | Bacteria | p__Proteobacteria | c__Gammaproteobacteria | o__CCD24 | f__CCD24 | g__CCD24 |  |
| 04005735 | Bacteria | p__Gemmatimonadota | c__AKAU4049 | o__AKAU4049 | f__AKAU4049 | g__AKAU4049 |  |
| MJ020-2-barcode59-umi78991bins-ubs-3 | Bacteria | p__Proteobacteria | c__Gammaproteobacteria | o__Pseudomonadales | f__Moraxellaceae | g__Acinetobacter |  |
| 2cf72e93b640ea7134f4f98f51044381 | Bacteria | p__Actinobacteriota | c__Actinobacteria | o__Corynebacteriales | f__Corynebacteriaceae | g__Corynebacterium |  |
| KF735812 | Bacteria | p__Bacteroidota | c__Bacteroidia | o__Sphingobacteriales | f__Sphingobacteriaceae | g__Sphingobacterium |  |
| 87633107cbbf5f900f6fae03df3a3ade | Bacteria | p__Proteobacteria | c__Gammaproteobacteria | o__Beggiatoales | f__Beggiatoaceae | g__uncultured | s__uncultured_gamma |
| RS-GCF-002291145.1-NZ-NTGE01000050.1 | Bacteria | p__Actinobacteriota | c__Actinobacteria | o__Streptomycetales | f__Streptomycetaceae | g__Streptomyces |  |
| 02086217 | Bacteria | p__Actinobacteriota | c__Actinobacteria | o__Propionibacteriales | f__Nocardioidaceae | g__Nocardioides |  |
| 05021820 | Bacteria | p__Actinobacteriota | c__Actinobacteria | o__Micrococcales | f__Promicromonosporaceae | g__Cellulosimicrobium |  |
| 5fe73f40b17d536dce06ed99c4a0c2f0 | Bacteria | p__Firmicutes | c__Clostridia | o__Lachnospirales | f__Lachnospiraceae | g__CHKCI001 | s__uncultured_bacterium |
| 539719ee08faac0f20130bb1caa8f41a | Bacteria | p__Firmicutes | c__Bacilli | o__Lactobacillales | f__Enterococcaceae | g__Enterococcus |  |
| b5b72616476b4582446d645d5bfb3ed8 | Bacteria | p__Actinobacteriota | c__Acidimicrobiia | o__Microtrichales | f__uncultured | g__uncultured |  |
| MJ006-1-barcode38-umi171460bins-ubs-3 | Bacteria | p__Firmicutes | c__Clostridia | o__Clostridia_UCG-014 | f__Clostridia_UCG-014 | g__Clostridia_UCG-014 |  |
| 1a43d91b246d8900b2ab7d01994d5295 | Bacteria | p__Firmicutes | c__Bacilli | o__Staphylococcales | f__Staphylococcaceae | g__Staphylococcus |  |
| fb519c57a4325afbad92e2931e3c7938 | Bacteria | p__Actinobacteriota | c__Actinobacteria | o__Frankiales | f__Acidothermaceae | g__Acidothermus |  |
| 48a730cf4a495803eacd118b7a65ed0e | Bacteria | p__Actinobacteriota | c__Actinobacteria | o__Corynebacteriales | f__Corynebacteriaceae | g__Corynebacterium |  |
| 4d90900aef814ad28d655f115b369431 | Bacteria | p__Bacteroidota | c__Bacteroidia | o__Flavobacteriales | f__Weeksellaceae | g__Chryseobacterium |  |
| b608e70cfa0fb600becc1cc47ef74ab8 | Bacteria | p__Proteobacteria | c__Alphaproteobacteria | o__Sphingomonadales | f__Sphingomonadaceae |  |  |
| 05026881 | Bacteria | p__Actinobacteriota | c__Thermoleophilia | o__Gaiellales | f__uncultured | g__uncultured |  |
| 18417424 | Bacteria | p__Proteobacteria | c__Gammaproteobacteria | o__Burkholderiales | f__Comamonadaceae |  |  |
| 204f3fa949fc107eec9ef0e9bd5b2e23 | Bacteria | p__Proteobacteria | c__Gammaproteobacteria | o__Burkholderiales | f__Comamonadaceae |  |  |
| b4a55207e72ad7fe98cfa1d79ca9ddb1 | Bacteria | p__Proteobacteria | c__Gammaproteobacteria | o__Burkholderiales | f__Comamonadaceae |  |  |
| ce380685c90352c69b292dc5ae5d0d77 | Bacteria |  |  |  |  |  |  |
| afd54906174e5c4376840bddcc4a1f37 | Bacteria | p__Proteobacteria | c__Gammaproteobacteria | o__Enterobacterales | f__Enterobacteriaceae |  |  |
| a4b1b4981233dd75f8ad860a14d5bc26 | Bacteria | p__Actinobacteriota | c__Thermoleophilia | o__Gaiellales | f__uncultured | g__uncultured |  |
| 02238888 | Bacteria | p__Proteobacteria | c__Alphaproteobacteria | o__Sphingomonadales | f__Sphingomonadaceae | g__Sphingobium |  |
| 407bb9b9b59ebd9080384a41fffc0301 | Bacteria | p__Actinobacteriota | c__Actinobacteria |  |  |  |  |
| 109e6faecdaaf00b9c1737a7ff976c85 | Bacteria | p__Proteobacteria | c__Alphaproteobacteria | o__Rhizobiales | f__Rhizobiaceae | g__Allorhizobium-Neorhizobium-Pararhizobium-Rhizobium |  |
| e56553b9962463c17bfef216d5fa6294 | Bacteria | p__Bacteroidota | c__Bacteroidia | o__Flavobacteriales | f__NS9_marine_group | g__NS9_marine_group | s__metagenome |
| 9afad77292ee02549afa35be6a24301b | Bacteria | p__Proteobacteria | c__Alphaproteobacteria | o__Rhodobacterales | f__Rhodobacteraceae |  |  |
| GB-GCA-013316495.1-WSMP01000005.1 | Bacteria | p__Firmicutes | c__Clostridia | o__Oscillospirales | f__Ruminococcaceae |  |  |
| 238be6b3dfb3a3057961aa5e01bd81a1 | Bacteria | p__Bacteroidota | c__Bacteroidia | o__Sphingobacteriales | f__Sphingobacteriaceae |  |  |
| 04949454 | Bacteria | p__Firmicutes | c__Clostridia | o__Lachnospirales | f__Lachnospiraceae | g__Lachnospiraceae_UCG-006 | s__uncultured_bacterium |
| 07fddde1c42a53abb8f84387426e33e7 | Bacteria | p__Proteobacteria | c__Gammaproteobacteria | o__Burkholderiales | f__Oxalobacteraceae | g__[Aquaspirillum]_arcticum_group |  |
| JQ864240 | Bacteria | p__Proteobacteria | c__Alphaproteobacteria | o__Rhizobiales | f__Rhizobiaceae | g__Aureimonas |  |
| FN814307 | Bacteria | p__Proteobacteria | c__Gammaproteobacteria | o__Burkholderiales | f__Oxalobacteraceae | g__Massilia |  |
| b8ec17e275b17375b20906ea1aa17cbe | Bacteria | p__Proteobacteria | c__Gammaproteobacteria | o__Enterobacterales | f__Enterobacteriaceae |  |  |
| a751980815a87e279580070dee1a75f7 | Bacteria | p__Acidobacteriota | c__Subgroup_5 | o__Subgroup_5 | f__Subgroup_5 | g__Subgroup_5 |  |
| 1b83e95e7afdfb0f81c574c0c6a3d5be | Bacteria | p__Firmicutes | c__Bacilli | o__Bacillales |  |  |  |
| 16910212 | Bacteria | p__Firmicutes | c__Clostridia | o__Lachnospirales | f__Lachnospiraceae | g__GCA-900066575 | s__uncultured_bacterium |
| G005879655_1 | Bacteria |  |  |  |  |  |  |
| MJ031-1-barcode42-umi39028bins-ubs-3 | Bacteria | p__Proteobacteria | c__Gammaproteobacteria | o__Burkholderiales | f__Oxalobacteraceae | g__Massilia |  |
| MJ006-2-barcode54-umi104695bins-ubs-4 | Bacteria | p__Bacteroidota | c__Bacteroidia | o__Bacteroidales | f__Bacteroidaceae | g__Bacteroides | s__Bacteroides_coprocola |
| 07175096 | Bacteria | p__Planctomycetota | c__Phycisphaerae | o__Tepidisphaerales | f__WD2101_soil_group | g__WD2101_soil_group | s__uncultured_bacterium |
| 20113723 | Bacteria | p__Firmicutes | c__Bacilli | o__Alicyclobacillales | f__Alicyclobacillaceae | g__Tumebacillus |  |
| 1a1b11c2de2836f0a734a44bb0eb69bb | Bacteria | p__Bacteroidota | c__Bacteroidia | o__Flavobacteriales | f__Flavobacteriaceae |  |  |
| ef2ecab244d212bbca44f7e57b40cb5b | Bacteria | p__Acidobacteriota | c__Subgroup_22 | o__Subgroup_22 | f__Subgroup_22 | g__Subgroup_22 | s__uncultured_Holophaga |
| aec9d89f544f86c50ef82d23e685ead1 | Bacteria | p__Firmicutes | c__Bacilli | o__Lactobacillales | f__Enterococcaceae | g__Enterococcus |  |
| 89f06fdcae9e15a208a0dcdb2b26b1bb | Bacteria | p__Firmicutes | c__Clostridia | o__Lachnospirales | f__Lachnospiraceae |  |  |
| RS-GCF-014644475.1-NZ-BMKT01000177.1 | Bacteria | p__Actinobacteriota | c__Actinobacteria | o__Micrococcales | f__Micrococcaceae | g__Sinomonas |  |
| d82bf65b39118a16dfa9028a1129a02f | Bacteria | p__Firmicutes | c__Limnochordia | o__MBA03 | f__MBA03 | g__MBA03 | s__uncultured_compost |
| 3cce2a940221e35e6e40e12dfa9de9a2 | Bacteria | p__Proteobacteria | c__Gammaproteobacteria | o__Burkholderiales | f__Nitrosomonadaceae | g__DSSD61 |  |
| 76c136b8ed73e555fb11ee81596f6bab | Bacteria | p__Firmicutes | c__Bacilli | o__Erysipelotrichales | f__Erysipelotrichaceae | g__Turicibacter | s__uncultured_bacterium |
| 74523aa33f3f913b8a293585a5629f78 | Bacteria | p__Firmicutes | c__Clostridia | o__Monoglobales | f__Monoglobaceae | g__Monoglobus |  |
| 05129744 | Bacteria | p__Firmicutes | c__Clostridia | o__Peptostreptococcales-Tissierellales | f__Peptostreptococcales-Tissierellales | g__Anaerococcus |  |
| 04989788 | Bacteria | p__Proteobacteria | c__Gammaproteobacteria | o__Pseudomonadales | f__Moraxellaceae | g__Acinetobacter |  |
| b3f765229b7492223c4601ef47bf8fa8 | Bacteria | p__Actinobacteriota | c__Acidimicrobiia | o__IMCC26256 | f__IMCC26256 | g__IMCC26256 | s__uncultured_Ferrimicrobium |
| 3d15a59da55dd62ae92b56adfe7004c9 | Bacteria | p__Bacteroidota | c__Bacteroidia | o__Flavobacteriales | f__Flavobacteriaceae | g__Flavobacterium |  |
| 0e871044811d9cd95f14f8dba5de75f3 | Bacteria | p__Myxococcota | c__Myxococcia | o__Myxococcales | f__Anaeromyxobacteraceae | g__Anaeromyxobacter |  |
| 04989195 | Bacteria | p__Proteobacteria | c__Gammaproteobacteria | o__Pseudomonadales | f__Moraxellaceae | g__Acinetobacter |  |
| 4ae87c67816c42dfe70ccb5df942eb44 | Bacteria | p__Proteobacteria | c__Gammaproteobacteria | o__Pseudomonadales | f__Moraxellaceae | g__Acinetobacter | s__Ambystoma_mexicanum |
| MJ034-1-barcode31-umi29317bins-ubs-6 | Bacteria | p__Proteobacteria | c__Gammaproteobacteria | o__Gammaproteobacteria_Incertae_Sedis | f__Unknown_Family | g__Acidibacter |  |
| 325ab9c3418ee3d1d6c819d7f3ff0188 | Bacteria | p__Acidobacteriota | c__Vicinamibacteria | o__Vicinamibacterales | f__Vicinamibacteraceae | g__Vicinamibacter |  |
| KF751867 | Bacteria | p__Bacteroidota | c__Bacteroidia | o__Flavobacteriales | f__Weeksellaceae | g__Chryseobacterium |  |
| 36c9817631fe0df693f4fbeb51965025 | Bacteria | p__Acidobacteriota | c__Acidobacteriae | o__Subgroup_2 | f__Subgroup_2 | g__Subgroup_2 | s__uncultured_forest |
| e2277f203fa09ada291287202f95d3a3 | Bacteria | p__Acidobacteriota | c__Acidobacteriae | o__Subgroup_2 | f__Subgroup_2 | g__Subgroup_2 | s__uncultured_eubacterium |
| 07f96ed40d2f611a5fb8962263d50e6a | Bacteria | p__Acidobacteriota | c__Vicinamibacteria | o__Vicinamibacterales | f__uncultured | g__uncultured |  |
| 14b5c534f5406d580fc85e2418c25fd6 | Bacteria | p__Bacteroidota | c__Bacteroidia | o__Chitinophagales | f__Chitinophagaceae | g__Edaphobaculum | s__uncultured_bacterium |
| 04952800 | Bacteria | p__Actinobacteriota | c__Thermoleophilia | o__Gaiellales |  |  |  |
| 04994723 | Bacteria | p__Proteobacteria | c__Alphaproteobacteria | o__Rhizobiales | f__Beijerinckiaceae | g__Microvirga |  |
| 0447a387fd62f9655475f27273557fd8 | Bacteria | p__Firmicutes | c__Bacilli | o__Bacillales | f__Planococcaceae | g__Planococcus |  |
| 3d269e0e608d2b7b9ce168058c54cb90 | Bacteria | p__Actinobacteriota | c__Actinobacteria | o__Frankiales | f__Acidothermaceae | g__Acidothermus |  |
| MJ010-1-barcode47-umi17870bins-ubs-34 | Bacteria | p__Proteobacteria | c__Gammaproteobacteria | o__Pseudomonadales | f__Moraxellaceae | g__Acinetobacter |  |
| 53db24cc01e306b0f422f63e8256e122 | Bacteria | p__Patescibacteria | c__Saccharimonadia | o__Saccharimonadales | f__Saccharimonadaceae | g__TM7a | s__uncultured_bacterium |
| bed9e73c47a01dafeb03a6a69fbc1674 | Bacteria | p__Firmicutes | c__Bacilli | o__Lactobacillales | f__Enterococcaceae | g__Enterococcus |  |
| RS-GCF-014268755.2-NZ-CP077093.1--6 | Bacteria | p__Proteobacteria | c__Gammaproteobacteria | o__Pseudomonadales | f__Pseudomonadaceae | g__Pseudomonas |  |
| MJ030-2-barcode65-umi4561bins-ubs-69 | Bacteria | p__Acidobacteriota | c__Acidobacteriae | o__Acidobacteriales | f__uncultured | g__uncultured |  |
| 04950850 | Bacteria | p__Firmicutes | c__Clostridia | o__Lachnospirales | f__Lachnospiraceae | g__GCA-900066575 |  |
| 19706797 | Bacteria | p__Firmicutes | c__Bacilli | o__Erysipelotrichales | f__Erysipelotrichaceae | g__Ileibacterium | s__Ileibacterium_valens |
| 05011990 | Bacteria | p__Proteobacteria | c__Gammaproteobacteria | o__Xanthomonadales | f__Rhodanobacteraceae | g__Luteibacter |  |
| 14801644 | Bacteria | p__Chloroflexi | c__Ktedonobacteria | o__Ktedonobacterales | f__JG30-KF-AS9 | g__JG30-KF-AS9 | s__uncultured_bacterium |
| 3c1f58494dad281192ec631a996e649f | Bacteria | p__Proteobacteria | c__Alphaproteobacteria | o__Rhizobiales | f__Beijerinckiaceae |  |  |
| 15f60dadae477b274ed11ca6ef47a180 | Bacteria | p__Proteobacteria | c__Gammaproteobacteria | o__Burkholderiales | f__Nitrosomonadaceae | g__MND1 |  |
| 15f4bb351cd2ea6d47a25cdb1f022157 | Bacteria | p__Chloroflexi | c__JG30-KF-CM66 | o__JG30-KF-CM66 | f__JG30-KF-CM66 | g__JG30-KF-CM66 |  |
| bee97a5c44fb3fcb9fcc4063fd1beb95 | Bacteria | p__Actinobacteriota | c__Thermoleophilia | o__Gaiellales |  |  |  |
| 02990132 | Bacteria | p__Myxococcota | c__Polyangia | o__mle1-27 | f__mle1-27 | g__mle1-27 | s__uncultured_delta |
| e5f8dd0cdbc24b7e18be4b26db69657f | Bacteria | p__Bacteroidota | c__Bacteroidia | o__Flavobacteriales | f__Weeksellaceae | g__Empedobacter |  |
| 4bdb6f7d8ab178ad36e63bc2f5033054 | Bacteria | p__Acidobacteriota | c__Acidobacteriae |  |  |  |  |
| 7d064d63684af625650ccfa9cc4edb26 | Bacteria | p__Actinobacteriota | c__Actinobacteria | o__Corynebacteriales | f__Corynebacteriaceae | g__Corynebacterium |  |
| 76d3d1df6988b021d4e320c55dfa3056 | Bacteria | p__Chloroflexi | c__Anaerolineae | o__SBR1031 | f__SBR1031 | g__SBR1031 | s__uncultured_sludge |
| 4831aefe155af260b0f23d0aba6cb317 | Bacteria | p__Acidobacteriota | c__Vicinamibacteria | o__Vicinamibacterales | f__uncultured | g__uncultured | s__uncultured_bacterium |
| ea8dcc79eab3b5a94baf83969bb7879a | Bacteria | p__Proteobacteria | c__Gammaproteobacteria | o__Pseudomonadales | f__Pseudomonadaceae | g__Azotobacter |  |
| 04989559 | Bacteria | p__Proteobacteria | c__Gammaproteobacteria | o__Steroidobacterales | f__Steroidobacteraceae | g__Steroidobacter |  |
| MJ033-1-barcode26-umi10549bins-ubs-28 | Bacteria | p__Proteobacteria | c__Gammaproteobacteria | o__Burkholderiales | f__Comamonadaceae |  |  |
| 70e46f23c9f52ea985c3e53173a31c3e | Bacteria | p__Proteobacteria | c__Alphaproteobacteria | o__Rhizobiales | f__Hyphomicrobiaceae | g__Pedomicrobium |  |
| MJ006-2-barcode69-umi185171bins-ubs-3 | Bacteria | p__Firmicutes | c__Bacilli | o__Erysipelotrichales | f__Erysipelotrichaceae | g__Turicibacter |  |
| 9527d0f5126716edf833d40cbe61d337 | Bacteria | p__Actinobacteriota | c__Acidimicrobiia | o__IMCC26256 | f__IMCC26256 | g__IMCC26256 |  |
| 7d69746875888a9c5809d46b2bd40350 | Bacteria | p__Proteobacteria | c__Gammaproteobacteria | o__Burkholderiales | f__Comamonadaceae | g__Hydrogenophaga |  |
| 86f7167efe02e28812e569884fcaaad7 | Bacteria | p__Proteobacteria | c__Alphaproteobacteria | o__Acetobacterales | f__Acetobacteraceae |  |  |
| 2b7118e693eb50146121812cec8a891c | Bacteria | p__Firmicutes | c__Clostridia | o__Oscillospirales | f__Oscillospiraceae | g__UCG-003 |  |
| bc36f099942268bf7e4d74a11b305d9d | Bacteria | p__Chloroflexi | c__Anaerolineae | o__SBR1031 | f__SBR1031 | g__SBR1031 | s__uncultured_Caldilineaceae |
| 8b9aeee735f5c4bc5d544cb09afa950b | Bacteria | p__Proteobacteria | c__Gammaproteobacteria | o__Enterobacterales | f__Enterobacteriaceae |  |  |
| GB-GCA-902636385.1-CACPRL010000097.1 | Bacteria | p__Proteobacteria | c__Gammaproteobacteria | o__Pseudomonadales | f__Moraxellaceae | g__Acinetobacter |  |
| 3b5d0b1cff8022cbf3b84bc9490702a4 | Bacteria | p__Proteobacteria | c__Alphaproteobacteria | o__Sphingomonadales | f__Sphingomonadaceae |  |  |
| MW621474 | Bacteria | p__Actinobacteriota | c__Actinobacteria | o__Micrococcales | f__Microbacteriaceae |  |  |
| a6fe6ca967c936a99d452a0902414d2c | Bacteria | p__Firmicutes | c__Bacilli | o__Paenibacillales | f__Paenibacillaceae | g__Paenibacillus | s__Paenibacillus_validus |
| MG970328 | Bacteria | p__Bacteroidota | c__Bacteroidia | o__Bacteroidales | f__Muribaculaceae | g__Muribaculaceae |  |
| 4448429463ba8545e616d969d9712b59 | Bacteria | p__Firmicutes | c__Bacilli | o__Lactobacillales | f__Carnobacteriaceae | g__Atopostipes |  |
| 6c214d39baaed9a01cd6cdc8da852f7c | Bacteria | p__Proteobacteria | c__Alphaproteobacteria | o__Rhizobiales |  |  |  |
| G001447195_1 | Bacteria | p__Proteobacteria | c__Gammaproteobacteria | o__Burkholderiales | f__Comamonadaceae | g__Aquabacterium |  |
| 5a1176066cba5ef9979ffff6d2a4d128 | Bacteria | p__Proteobacteria | c__Gammaproteobacteria | o__Pseudomonadales | f__Moraxellaceae | g__Acinetobacter |  |
| MJ031-1-barcode32-umi1550bins-ubs-206 | Bacteria | p__Firmicutes | c__Clostridia | o__Clostridiales | f__Clostridiaceae | g__Clostridium_sensu_stricto_9 |  |
| 1128e1f40d56ff35ce6bf1f306deea92 | Bacteria | p__Chloroflexi | c__Ktedonobacteria | o__Ktedonobacterales | f__JG30-KF-AS9 | g__JG30-KF-AS9 | s__uncultured_bacterium |
| 88b20a52db55576a7a9d240ab050bbf7 | Bacteria | p__Chloroflexi | c__Chloroflexia | o__Chloroflexales | f__Roseiflexaceae | g__Kouleothrix | s__uncultured_bacterium |
| 856a761ec7435d1142ee3874c2e9f473 | Bacteria | p__Proteobacteria | c__Gammaproteobacteria | o__Pseudomonadales | f__Pseudomonadaceae | g__Pseudomonas |  |
| 79f811fe1284ea308ba896b4b141683b | Bacteria | p__Acidobacteriota | c__Vicinamibacteria | o__Vicinamibacterales | f__uncultured | g__uncultured |  |
| 04997461 | Bacteria | p__Acidobacteriota | c__Acidobacteriae | o__Acidobacteriales | f__Acidobacteriaceae_(Subgroup_1) | g__Bryocella | s__uncultured_bacterium |
| 9c019871385d4af4fd7702bdf1338cd4 | Bacteria | p__Proteobacteria | c__Gammaproteobacteria | o__Burkholderiales | f__Comamonadaceae | g__Ramlibacter |  |
| RS-GCF-000622225.1-NZ-JHWD01000071.1 | Bacteria | p__Proteobacteria | c__Alphaproteobacteria | o__Acetobacterales | f__Acetobacteraceae | g__Roseomonas |  |
| cbdc3bad4a2e646a9761ca03325c05a3 | Bacteria |  |  |  |  |  |  |
| 02918877 | Bacteria | p__Planctomycetota | c__Phycisphaerae | o__Tepidisphaerales | f__WD2101_soil_group | g__WD2101_soil_group |  |
| 37392bdab728ddfa73d3f6b5c24949c9 | Bacteria | p__Firmicutes | c__Bacilli | o__Lactobacillales | f__Lactobacillaceae | g__Lactobacillus |  |
| 05033249 | Bacteria | p__Actinobacteriota | c__Actinobacteria | o__Corynebacteriales | f__Corynebacteriaceae | g__Corynebacterium |  |
| 74c1bfaa1d97fe41fc08db0aec90aec6 | Bacteria | p__Chloroflexi | c__Anaerolineae | o__SJA-15 | f__SJA-15 | g__SJA-15 |  |
| e59dfff162609a34a6b369e695f44283 | Bacteria | p__Bacteroidota | c__Bacteroidia | o__Cytophagales | f__Microscillaceae | g__OLB12 |  |
| afb0fe9f9e4fc55885a8dbc1a805f1af | Bacteria | p__Firmicutes | c__Bacilli | o__Paenibacillales | f__Paenibacillaceae | g__Paenibacillus |  |
| GB-GCA-018326525.1-BJIB01000024.1 | Bacteria | p__Actinobacteriota | c__Actinobacteria | o__Bifidobacteriales | f__Bifidobacteriaceae | g__Bifidobacterium | s__Bifidobacterium_bifidum |
| MJ030-2-barcode65-umi89197bins-ubs-7 | Bacteria | p__Acidobacteriota | c__Acidobacteriae | o__Solibacterales | f__Solibacteraceae | g__Candidatus_Solibacter |  |
| 14335090 | Bacteria | p__Acidobacteriota | c__Vicinamibacteria | o__Vicinamibacterales | f__uncultured | g__uncultured |  |
| 04666519 | Bacteria | p__Firmicutes | c__Bacilli | o__Lactobacillales | f__Streptococcaceae | g__Streptococcus |  |
| 03345277 | Bacteria | p__Planctomycetota | c__Planctomycetes | o__Pirellulales | f__Pirellulaceae | g__uncultured |  |
| fb884a160ca0fa7c167c8466ee1ea3b5 | Bacteria | p__Actinobacteriota | c__Actinobacteria | o__Kineosporiales | f__Kineosporiaceae |  |  |
| bfa94de1084fb2f26db1b53cdbff8df8 | Bacteria | p__Actinobacteriota | c__Actinobacteria |  |  |  |  |
| 398726e470f146c4db933d375ed2f0a3 | Bacteria | p__Bacteroidota | c__Bacteroidia | o__Cytophagales | f__Hymenobacteraceae | g__Adhaeribacter |  |
| 23d220a2f126a8f15b93a438f8bdfe24 | Bacteria | p__Proteobacteria | c__Gammaproteobacteria | o__Enterobacterales | f__Enterobacteriaceae |  |  |
| MJ030-1-barcode42-umi12558bins-ubs-36 | Bacteria | p__Acidobacteriota | c__Blastocatellia | o__Blastocatellales | f__Blastocatellaceae |  |  |
| G000372185_1 | Bacteria | p__Actinobacteriota | c__Actinobacteria | o__Frankiales | f__Sporichthyaceae | g__hgcI_clade |  |
| MJ030-1-barcode42-umi89449bins-ubs-4 | Bacteria | p__Proteobacteria | c__Alphaproteobacteria | o__Rhizobiales | f__Xanthobacteraceae |  |  |
| bc92e438c8c725d8777cdd4ecd632eb6 | Bacteria | p__Actinobacteriota | c__Actinobacteria | o__Micrococcales | f__Microbacteriaceae |  |  |
| 05013896 | Bacteria | p__Bacteroidota | c__Bacteroidia | o__Flavobacteriales | f__Weeksellaceae | g__Chryseobacterium |  |
| MJ010-1-barcode25-umi89754bins-ubs-7 | Bacteria | p__Proteobacteria | c__Gammaproteobacteria | o__Xanthomonadales | f__Xanthomonadaceae | g__Pseudoxanthomonas |  |
| GB-GCA-013184635.1-JAAPAY010000061.1 | Bacteria | p__Proteobacteria | c__Alphaproteobacteria | o__Rhizobiales | f__Beijerinckiaceae | g__Methylobacterium-Methylorubrum |  |
| 11063375 | Bacteria | p__Bacteroidota | c__Bacteroidia | o__Bacteroidales | f__Muribaculaceae | g__Muribaculaceae |  |
| MJ006-1-barcode37-umi230976bins-ubs-3 | Bacteria | p__Firmicutes | c__Clostridia | o__Lachnospirales | f__Lachnospiraceae | g__Lachnoclostridium |  |
| KM083136 | Bacteria | p__Proteobacteria | c__Alphaproteobacteria | o__Rhizobiales | f__Rhizobiaceae | g__Allorhizobium-Neorhizobium-Pararhizobium-Rhizobium |  |
| a77bd2dba5771ce39ffdbd7e3be05c7d | Bacteria | p__Actinobacteriota | c__Actinobacteria | o__Frankiales | f__Acidothermaceae | g__Acidothermus |  |
| 02080691 | Bacteria | p__Actinobacteriota | c__Actinobacteria | o__Corynebacteriales | f__Corynebacteriaceae | g__Corynebacterium | s__Corynebacterium_variabile |
| RS-GCF-001556635.1-NZ-LSJF01000595.1 | Bacteria | p__Proteobacteria | c__Alphaproteobacteria | o__Sphingomonadales | f__Sphingomonadaceae | g__Sphingomonas |  |
| 3e365b13da05bfadc6b9d86e89a04055 | Bacteria | p__Patescibacteria | c__Saccharimonadia | o__Saccharimonadales | f__Saccharimonadaceae | g__TM7a |  |
| RS-GCF-003057695.2-NZ-QBJC02000047.1 | Bacteria | p__Proteobacteria | c__Gammaproteobacteria | o__Enterobacterales | f__Enterobacteriaceae | g__Pseudocitrobacter |  |
| X94198 | Bacteria | p__Proteobacteria | c__Alphaproteobacteria | o__Rhizobiales | f__Xanthobacteraceae | g__Xanthobacter |  |
| 05038736 | Bacteria | p__Firmicutes | c__Bacilli | o__Lactobacillales | f__Aerococcaceae | g__Aerococcus |  |
| 05015613 | Bacteria | p__Proteobacteria | c__Gammaproteobacteria | o__Pasteurellales | f__Pasteurellaceae | g__Haemophilus |  |
| ec77fec73823dbe6b3fd4732dc5636ac | Bacteria | p__Proteobacteria | c__Gammaproteobacteria | o__Enterobacterales |  |  |  |
| 02257277 | Bacteria | p__Chloroflexi | c__KD4-96 | o__KD4-96 | f__KD4-96 | g__KD4-96 | s__uncultured_bacterium |
| 91cd912ba6658ae81ca64dbc933464cd | Bacteria | p__Proteobacteria | c__Gammaproteobacteria | o__Enterobacterales | f__Enterobacteriaceae |  |  |
| 1e50713e40d33c275bcc58468771cbf8 | Bacteria | p__Chloroflexi | c__Gitt-GS-136 | o__Gitt-GS-136 | f__Gitt-GS-136 | g__Gitt-GS-136 | s__uncultured_bacterium |
| 773a133dcc139782bf9d4899c4dc8f9c | Bacteria | p__Proteobacteria | c__Gammaproteobacteria |  |  |  |  |
| 03197276 | Bacteria | p__Verrucomicrobiota | c__Omnitrophia | o__Omnitrophales | f__Omnitrophales | g__Omnitrophales |  |
| MJ034-1-barcode30-umi26918bins-ubs-4 | Bacteria | p__Proteobacteria | c__Gammaproteobacteria | o__Gammaproteobacteria_Incertae_Sedis | f__Unknown_Family | g__Acidibacter |  |
| aaab34d171566e280b4d7a2aa32aafb7 | Bacteria | p__Acidobacteriota | c__Acidobacteriae | o__Bryobacterales | f__Bryobacteraceae | g__Bryobacter |  |
| AB253435 | Bacteria | p__Proteobacteria | c__Alphaproteobacteria | o__Acetobacterales | f__Acetobacteraceae | g__Gluconobacter |  |
| 1e055bb55c51ad293c914f80dc0ed0b7 | Bacteria | p__Proteobacteria | c__Alphaproteobacteria | o__uncultured | f__uncultured | g__uncultured | s__uncultured_actinobacterium |
| RS-GCF-002161835.1-NZ-MXAR01000093.1 | Bacteria | p__Firmicutes | c__Bacilli | o__Brevibacillales | f__Brevibacillaceae | g__Brevibacillus |  |
| 03138094 | Bacteria | p__Gemmatimonadota | c__S0134_terrestrial_group | o__S0134_terrestrial_group | f__S0134_terrestrial_group | g__S0134_terrestrial_group | s__uncultured_bacterium |
| fd97e92a8896c6c2b781daa58b6a35d0 | Bacteria | p__Proteobacteria | c__Alphaproteobacteria | o__Rhizobiales | f__Rhizobiaceae |  |  |
| f28b0d784841a373830abc201f3639eb | Bacteria | p__Proteobacteria | c__Alphaproteobacteria | o__Sphingomonadales | f__Sphingomonadaceae | g__Sphingomonas |  |
| 04909283 | Bacteria | p__Proteobacteria | c__Alphaproteobacteria | o__Rhizobiales | f__D05-2 | g__D05-2 | s__alpha_proteobacterium |
| 392d3df557029f3fa925efbbe34add55 | Bacteria | p__Bacteroidota | c__Bacteroidia | o__Bacteroidales | f__Prolixibacteraceae | g__uncultured |  |
| 6d3cea3534b8bcc7a2a329e183b9ffd6 | Bacteria | p__Actinobacteriota | c__Rubrobacteria | o__Rubrobacterales | f__Rubrobacteriaceae | g__Rubrobacter | s__Rubrobacter_bracarensis |
| 585b800c2a3595bcb4394ac39f2ddc95 | Bacteria | p__Proteobacteria | c__Gammaproteobacteria | o__Xanthomonadales | f__Xanthomonadaceae | g__Stenotrophomonas |  |
| 3f343776b03923d8087c689eed2eb9c7 | Bacteria | p__Chloroflexi | c__TK10 | o__TK10 | f__TK10 | g__TK10 |  |
| 04946307 | Bacteria | p__Actinobacteriota | c__Actinobacteria | o__Micrococcales | f__Promicromonosporaceae | g__Cellulosimicrobium |  |
| 23475f0e3808d10d2642d87146b28761 | Bacteria | p__Proteobacteria | c__Alphaproteobacteria | o__Sphingomonadales | f__Sphingomonadaceae | g__Sphingobium |  |
| b00399533719a9b2f78b8527775933b6 | Bacteria | p__Proteobacteria | c__Alphaproteobacteria | o__Rhizobiales | f__Xanthobacteraceae | g__Pseudolabrys |  |
| d108038ea2dbafa6f4b05f351aa3a949 | Bacteria | p__Actinobacteriota | c__Actinobacteria | o__Corynebacteriales | f__Mycobacteriaceae | g__Mycobacterium |  |
| 15155c526256ad617e0d8801b4b8a039 | Bacteria | p__Firmicutes | c__Bacilli | o__Lactobacillales | f__Enterococcaceae | g__Enterococcus |  |
| 03171593 | Bacteria | p__Planctomycetota | c__Phycisphaerae | o__Tepidisphaerales | f__WD2101_soil_group | g__WD2101_soil_group | s__uncultured_planctomycete |
| 04665932 | Bacteria | p__Proteobacteria | c__Gammaproteobacteria | o__Burkholderiales | f__Neisseriaceae | g__Neisseria |  |
| RS-GCF-018784865.1-NZ-WQPA01000083.1 | Bacteria | p__Firmicutes | c__Clostridia | o__Lachnospirales | f__Lachnospiraceae | g__Dorea |  |
| 6748d29185d0986bb3b68f2846947a9b | Bacteria | p__Bacteroidota | c__Bacteroidia | o__Bacteroidales | f__Barnesiellaceae | g__Barnesiella |  |
| 03847b9db3d03362e1f193c86b10e943 | Bacteria | p__Acidobacteriota | c__Acidobacteriae | o__Acidobacteriales | f__uncultured | g__uncultured |  |
| 04957648 | Bacteria | p__Proteobacteria | c__Gammaproteobacteria | o__Burkholderiales | f__Comamonadaceae | g__Comamonas |  |
| G005881895_1 | Bacteria | p__Proteobacteria | c__Gammaproteobacteria | o__Burkholderiales | f__SC-I-84 | g__SC-I-84 |  |
| ed8827f343348ee930b632252af1b698 | Bacteria | p__Verrucomicrobiota | c__Verrucomicrobiae | o__Chthoniobacterales |  |  |  |
| RS-GCF-001553225.1-NZ-KQ960474.1 | Bacteria | p__Bacteroidota | c__Bacteroidia | o__Bacteroidales | f__Prevotellaceae | g__Prevotella | s__Prevotella_amnii |
| 817300b51e5976137c06f34329c5935f | Bacteria | p__Bacteroidota | c__Bacteroidia | o__Flavobacteriales | f__Crocinitomicaceae | g__Fluviicola |  |
| 2ca3435885d5ee48fe54dc8c2002d882 | Bacteria | p__Firmicutes | c__Clostridia | o__Lachnospirales | f__Lachnospiraceae |  |  |
| 05031425 | Bacteria | p__Actinobacteriota | c__Actinobacteria | o__Micrococcales | f__Microbacteriaceae |  |  |
| d6b2714669ce5856c2fb8dd95fabdfe6 | Bacteria | p__Actinobacteriota | c__Actinobacteria | o__Frankiales | f__Acidothermaceae | g__Acidothermus |  |
| 86a3a4e8c1eacfa13efb692e9a9507f8 | Bacteria | p__Proteobacteria | c__Gammaproteobacteria | o__Burkholderiales | f__Oxalobacteraceae |  |  |
| 02415675 | Bacteria | p__Proteobacteria | c__Alphaproteobacteria | o__Rhodobacterales | f__Rhodobacteraceae | g__Paracoccus |  |
| 45c58d71ad27cb5243ea8d46a3812cae | Bacteria | p__Methylomirabilota | c__Methylomirabilia | o__Rokubacteriales | f__Rokubacteriales | g__Rokubacteriales | s__uncultured_bacterium |
| f75b67eefb805d4f851608510902f15c | Bacteria | p__Proteobacteria | c__Alphaproteobacteria | o__Acetobacterales | f__Acetobacteraceae | g__Acidisphaera |  |
| cbbb23c7dc23cda9c0d74ef8d4a73432 | Bacteria | p__Actinobacteriota | c__Actinobacteria | o__Micrococcales | f__Microbacteriaceae | g__Microbacterium |  |
| fa69018ffac770583a52bc42febd162c | Bacteria | p__Chloroflexi | c__Anaerolineae | o__SBR1031 | f__SBR1031 | g__SBR1031 |  |
| 04994724 | Bacteria | p__Proteobacteria | c__Alphaproteobacteria | o__Rhizobiales | f__Beijerinckiaceae | g__Microvirga |  |
| 09cf04657f83a87c5e9dd75a875b73eb | Bacteria | p__Proteobacteria | c__Gammaproteobacteria | o__Pseudomonadales | f__Moraxellaceae | g__Acinetobacter | s__Acinetobacter_towneri |
| 5998335702d67df75dc8a6bc26eb9257 | Bacteria | p__Firmicutes | c__Clostridia | o__Oscillospirales | f__Ruminococcaceae |  |  |
| edad8b57044cd93cc8ebf69b0ec0bae2 | Bacteria | p__Firmicutes | c__Bacilli | o__Erysipelotrichales | f__Erysipelatoclostridiaceae | g__Erysipelatoclostridium | s__uncultured_Clostridium |
| RS-GCF-000742775.1-NZ-JPRQ01000048.1 | Bacteria | p__Proteobacteria | c__Gammaproteobacteria | o__Pseudomonadales | f__Pseudomonadaceae | g__Pseudomonas |  |
| KY194797 | Bacteria | p__Proteobacteria | c__Gammaproteobacteria | o__Xanthomonadales | f__Rhodanobacteraceae | g__Dyella | s__Dyella_halodurans |
| G001029695_1 | Bacteria | p__Patescibacteria | c__Saccharimonadia | o__Saccharimonadales | f__Saccharimonadales | g__Saccharimonadales |  |
| 51bf11f7d8c2d042f0bcaf35682d8802 | Bacteria | p__Planctomycetota | c__Phycisphaerae | o__Tepidisphaerales | f__WD2101_soil_group | g__WD2101_soil_group |  |
| G003574675_2 | Bacteria | p__Firmicutes | c__Bacilli | o__Bacillales | f__Bacillaceae | g__Bacillus |  |
| RS-GCF-000010225.1-NC-009342.1--6 | Bacteria | p__Actinobacteriota | c__Actinobacteria | o__Corynebacteriales | f__Corynebacteriaceae | g__Corynebacterium |  |
| RS-GCF-011022235.1-NZ-JAAIYP010000015.1 | Bacteria | p__Proteobacteria | c__Alphaproteobacteria | o__Rhodospirillales | f__Magnetospirillaceae | g__Magnetospirillum |  |
| e2decb1726d9f62f144cdbd2bb6a386f | Bacteria | p__Acidobacteriota | c__Thermoanaerobaculia | o__Thermoanaerobaculales | f__Thermoanaerobaculaceae | g__Subgroup_10 | s__uncultured_prokaryote |
| 861bb336b28022b54ba39b47e58dccc3 | Bacteria | p__Firmicutes | c__Clostridia | o__Lachnospirales | f__Lachnospiraceae |  |  |
| RS-GCF-001062925.1-NZ-JVQG01000014.1 | Bacteria | p__Proteobacteria | c__Gammaproteobacteria | o__Enterobacterales | f__Yersiniaceae | g__Serratia |  |
| 1124d67e25a67144c258c62e99eade8e | Bacteria | p__Chloroflexi | c__Anaerolineae | o__C10-SB1A | f__C10-SB1A | g__C10-SB1A | s__metagenome |
| GB-GCA-003833095.1-RIBN01000070.1 | Bacteria | p__Bacteroidota | c__Bacteroidia | o__Bacteroidales | f__Muribaculaceae | g__Muribaculaceae | s__mouse_gut |
| 4b118699df5f2aa1022bb238e662f9f0 | Bacteria | p__Chloroflexi | c__Ktedonobacteria | o__Ktedonobacterales | f__JG30-KF-AS9 | g__JG30-KF-AS9 | s__uncultured_bacterium |
| 33008b498bc56446da54f1e23bc4c7f2 | Bacteria | p__Firmicutes | c__Bacilli | o__Staphylococcales | f__Staphylococcaceae | g__Staphylococcus |  |
| ae07a440b2d536f3eb39702a39b48c16 | Bacteria | p__Actinobacteriota | c__Coriobacteriia | o__Coriobacteriales | f__Eggerthellaceae |  |  |
| AB514513 | Bacteria | p__Actinobacteriota | c__Actinobacteria | o__Pseudonocardiales | f__Pseudonocardiaceae | g__Actinomycetospora |  |
| 04912112 | Bacteria | p__Nitrospirota | c__Nitrospiria | o__Nitrospirales | f__Nitrospiraceae | g__Nitrospira |  |
| 04906284 | Bacteria | p__Proteobacteria | c__Alphaproteobacteria | o__Rhizobiales | f__Beijerinckiaceae | g__Bosea |  |
| AIEB01000124 | Bacteria | p__Proteobacteria | c__Gammaproteobacteria | o__Pseudomonadales | f__Moraxellaceae | g__Acinetobacter |  |
| c29f72c5d4372b836a23c61e77207f75 | Bacteria | p__Myxococcota | c__Polyangia | o__Polyangiales | f__Sandaracinaceae | g__uncultured |  |
| e284d5f843672fc79317233d27a88314 | Bacteria | p__Acidobacteriota | c__Acidobacteriae | o__Subgroup_2 | f__Subgroup_2 | g__Subgroup_2 |  |
| 4e54e7de1a2fabb869a67d4d353ab87a | Bacteria | p__Proteobacteria | c__Gammaproteobacteria | o__Xanthomonadales | f__Xanthomonadaceae |  |  |
| 148b79a3071a704bf7b0c826e5615c1a | Bacteria | p__Chloroflexi | c__Ktedonobacteria | o__C0119 | f__C0119 | g__C0119 | s__uncultured_bacterium |
| 4f11c953d7fb7c06736526ddd886479f | Bacteria | p__Acidobacteriota | c__Acidobacteriae | o__Subgroup_2 | f__Subgroup_2 | g__Subgroup_2 | s__uncultured_bacterium |
| MJ010-2-barcode64-umi70864bins-ubs-8 | Bacteria | p__Proteobacteria | c__Gammaproteobacteria | o__Pseudomonadales | f__Moraxellaceae | g__Acinetobacter |  |
| AM501439 | Bacteria | p__Proteobacteria | c__Gammaproteobacteria | o__Burkholderiales | f__Comamonadaceae | g__Pelomonas |  |
| 4b8a62dcc0ac5312d081415a79bb8180 | Bacteria | p__Proteobacteria | c__Gammaproteobacteria | o__Burkholderiales | f__Burkholderiaceae | g__Burkholderia-Caballeronia-Paraburkholderia |  |
| 04876097 | Bacteria | p__Proteobacteria | c__Alphaproteobacteria | o__Rhizobiales | f__Beijerinckiaceae | g__Methylobacterium-Methylorubrum |  |
| 277122bcd9fda2cc295741e821bd135e | Bacteria | p__Actinobacteriota | c__MB-A2-108 | o__MB-A2-108 | f__MB-A2-108 | g__MB-A2-108 |  |
| MJ031-1-barcode42-umi35639bins-ubs-5 | Bacteria | p__Bacteroidota | c__Bacteroidia | o__Chitinophagales | f__Chitinophagaceae | g__Puia |  |
| AY998119 | Bacteria | p__Firmicutes | c__Bacilli | o__Bacillales | f__Bacillaceae | g__Bacillus |  |
| de22792661ee272faca3704b28c54d27 | Bacteria | p__Proteobacteria | c__Alphaproteobacteria | o__Rhizobiales | f__Xanthobacteraceae | g__uncultured |  |
| 0ce430c1ada0023db78c8317f5f33f7d | Bacteria | p__Proteobacteria | c__Gammaproteobacteria | o__Pseudomonadales | f__Moraxellaceae | g__Acinetobacter |  |
| 7282e554ec4cfd5d7c5018be05773350 | Bacteria | p__Bacteroidota | c__Bacteroidia | o__Chitinophagales | f__Chitinophagaceae | g__Taibaiella | s__uncultured_bacterium |
| 7607fc7c5aab63ec9c307348846dbfe1 | Bacteria | p__Proteobacteria | c__Alphaproteobacteria | o__Rhodobacterales | f__Rhodobacteraceae | g__Paracoccus |  |
| 55be75e53913600f704b257b9759cafd | Bacteria | p__Patescibacteria | c__Parcubacteria | o__Candidatus_Kaiserbacteria | f__Candidatus_Kaiserbacteria | g__Candidatus_Kaiserbacteria |  |
| fcdefceeed42cdf4efa15371931e0bbe | Bacteria | p__Chloroflexi | c__Ktedonobacteria | o__Ktedonobacterales | f__Ktedonobacteraceae |  |  |
| 0a2f7652b363294cd8832da06fd02062 | Bacteria | p__Proteobacteria | c__Gammaproteobacteria | o__Burkholderiales | f__SC-I-84 | g__SC-I-84 |  |
| 577ec5ad27624b445189f9e7a5f90339 | Bacteria | p__WPS-2 | c__WPS-2 | o__WPS-2 | f__WPS-2 | g__WPS-2 | s__uncultured_bacterium |
| 0fb0f0ac70dab9a21a6efe2aa66773d0 | Bacteria | p__Proteobacteria | c__Gammaproteobacteria | o__Gammaproteobacteria_Incertae_Sedis | f__Unknown_Family | g__Acidibacter | s__metagenome |
| RS-GCF-000804465.1-NZ-AYPT01000053.1 | Bacteria | p__Firmicutes | c__Bacilli | o__Staphylococcales | f__Staphylococcaceae | g__Staphylococcus |  |
| 19abb5af932feec58b60f63c41a90578 | Bacteria | p__Bacteroidota | c__Bacteroidia | o__Bacteroidales | f__Muribaculaceae | g__Muribaculaceae |  |
| dacb0d0be9205115fa97d9c431becfc2 | Bacteria | p__Firmicutes | c__Bacilli | o__Lactobacillales | f__Enterococcaceae | g__Enterococcus |  |
| RS-GCF-003614925.1-NZ-QYLP01000008.1 | Bacteria | p__Actinobacteriota | c__Actinobacteria | o__Micrococcales | f__Micrococcaceae | g__Arthrobacter |  |
| e0c782a233f81da1e1beff89889338ab | Bacteria | p__Proteobacteria | c__Gammaproteobacteria | o__Burkholderiales | f__Rhodocyclaceae | g__Methyloversatilis |  |
| b05469e53fed8fce7081568dc1902cf5 | Bacteria | p__Chloroflexi | c__Chloroflexia | o__Thermomicrobiales | f__JG30-KF-CM45 | g__JG30-KF-CM45 | s__uncultured_Chloroflexi |
| 06b289b531de7d193f42c80522d82663 | Bacteria | p__Actinobacteriota | c__Actinobacteria | o__Micrococcales | f__Microbacteriaceae |  |  |
| AJ430344 | Bacteria | p__Proteobacteria | c__Gammaproteobacteria | o__Burkholderiales | f__Comamonadaceae | g__Comamonas | s__Comamonas_aquatica |
| 56ece2d04042ba9ff21620e83de51fcd | Bacteria | p__Chloroflexi | c__Chloroflexia | o__Chloroflexales | f__Roseiflexaceae | g__uncultured | s__uncultured_bacterium |
| Y14310 | Bacteria | p__Proteobacteria | c__Alphaproteobacteria | o__Rhizobiales | f__Hyphomicrobiaceae | g__Hyphomicrobium |  |
| 5895e64f1620cda2f9c0e8e73e61bb6d | Bacteria | p__Actinobacteriota | c__Actinobacteria | o__Propionibacteriales | f__Propionibacteriaceae | g__Cutibacterium |  |
| G902167945_1 | Bacteria | p__Firmicutes | c__Clostridia | o__Lachnospirales | f__Lachnospiraceae | g__Blautia |  |
| RS-GCF-001592875.1-NZ-LSOF01000029.1 | Bacteria | p__Proteobacteria | c__Gammaproteobacteria | o__Pseudomonadales | f__Pseudomonadaceae | g__Pseudomonas |  |
| DQ343754 | Bacteria | p__Firmicutes | c__Bacilli | o__Lactobacillales | f__Streptococcaceae | g__Lactococcus | s__Lactococcus_piscium |
| AY772089 | Bacteria | p__Proteobacteria | c__Gammaproteobacteria | o__Burkholderiales | f__Methylophilaceae | g__Methylophilus |  |
| 633acdbcabbb959e1ca2dbbd5b1bd445 | Bacteria | p__Actinobacteriota | c__Actinobacteria | o__Pseudonocardiales | f__Pseudonocardiaceae |  |  |
| 0eb2522a9b3ed8acb6a55055ac4688c3 | Bacteria | p__Proteobacteria | c__Gammaproteobacteria | o__Pseudomonadales | f__Moraxellaceae | g__Acinetobacter |  |
| GB-GCA-900066645.1-FMFR01000008.1 | Bacteria | p__Firmicutes | c__Clostridia | o__Oscillospirales | f__Oscillospiraceae | g__Colidextribacter |  |
| EU741036 | Bacteria | p__Firmicutes | c__Bacilli | o__Paenibacillales | f__Paenibacillaceae | g__Paenibacillus |  |
| 8f3d009dea485e9459cb984fa8ca4ee1 | Bacteria |  |  |  |  |  |  |
| 4d02b713fe269e8f87a1f6333f4ee4ac | Bacteria | p__Chloroflexi | c__AD3 | o__AD3 | f__AD3 | g__AD3 | s__uncultured_bacterium |
| 10a9f06378f21a59ad9d5fdc86bf7519 | Bacteria | p__Proteobacteria | c__Gammaproteobacteria | o__Burkholderiales | f__Nitrosomonadaceae | g__MND1 |  |
| 16958586 | Bacteria | p__Firmicutes | c__Clostridia | o__Lachnospirales | f__Lachnospiraceae | g__Lachnospiraceae_FCS020_group | s__uncultured_prokaryote |
| 0c0fc5b135fe64b131ab2f8dd88ddace | Bacteria | p__Proteobacteria | c__Gammaproteobacteria | o__Burkholderiales | f__Comamonadaceae |  |  |
| 04901490 | Bacteria | p__Proteobacteria | c__Gammaproteobacteria | o__Pseudomonadales | f__Moraxellaceae | g__Acinetobacter |  |
| baad0d451c97443301906bc56a065399 | Bacteria | p__Desulfobacterota | c__Desulfovibrionia | o__Desulfovibrionales | f__Desulfomicrobiaceae | g__Desulfomicrobium |  |
| RS-GCF-003339665.1-NZ-QNUW01000001.1 | Bacteria | p__Actinobacteriota | c__Actinobacteria | o__Micrococcales | f__Microbacteriaceae | g__Microbacterium |  |
| 14801207 | Bacteria | p__Chloroflexi | c__Ktedonobacteria | o__Ktedonobacterales | f__JG30-KF-AS9 | g__JG30-KF-AS9 | s__uncultured_soil |
| f60f3f466b45c47802578b77a82b3a91 | Bacteria | p__Proteobacteria | c__Gammaproteobacteria | o__Enterobacterales |  |  |  |
| MJ033-1-barcode26-umi21281bins-ubs-12 | Bacteria | p__Proteobacteria | c__Gammaproteobacteria | o__Enterobacterales | f__Morganellaceae | g__Morganella |  |
| GB-GCA-910575615.1-CAJTEX010000063.1 | Bacteria | p__Bacteroidota | c__Bacteroidia | o__Bacteroidales | f__Muribaculaceae | g__Muribaculaceae |  |
| AY268174 | Bacteria | p__Proteobacteria | c__Gammaproteobacteria | o__Burkholderiales | f__Burkholderiaceae | g__Pandoraea |  |
| fce3b4d019ca6022b00a6ed39a5395ee | Bacteria | p__Proteobacteria | c__Alphaproteobacteria | o__Rhizobiales |  |  |  |
| 02395829 | Bacteria | p__Proteobacteria | c__Alphaproteobacteria | o__Sphingomonadales | f__Sphingomonadaceae |  |  |
| abed377c083789b11da129a841a6f413 | Bacteria | p__Bacteroidota | c__Bacteroidia | o__Bacteroidales | f__Bacteroidetes_vadinHA17 | g__Bacteroidetes_vadinHA17 |  |
| 3f6994af968b89ac59d9f66710b46b33 | Bacteria | p__Proteobacteria | c__Gammaproteobacteria | o__Burkholderiales | f__TRA3-20 | g__TRA3-20 |  |
| b663cfdaec28433961a4ef053bc2527a | Bacteria | p__Acidobacteriota | c__Acidobacteriae | o__Acidobacteriales | f__Acidobacteriaceae_(Subgroup_1) |  |  |
| 02247205 | Bacteria | p__Actinobacteriota | c__Actinobacteria | o__Frankiales | f__Geodermatophilaceae | g__Blastococcus |  |
| a77d3bbbfc7337cc688688108a38b3f3 | Bacteria | p__Proteobacteria | c__Gammaproteobacteria |  |  |  |  |
| 04911183 | Bacteria | p__Proteobacteria | c__Gammaproteobacteria | o__Xanthomonadales | f__Xanthomonadaceae | g__Stenotrophomonas | s__Stenotrophomonas_rhizophila |
| 31a4af358b50615b5792c3d8a9ace5eb | Bacteria | p__Proteobacteria | c__Gammaproteobacteria | o__Pseudomonadales | f__Moraxellaceae | g__Acinetobacter | s__Ambystoma_mexicanum |
| 87c9fdcdba1fedb35da2e3d7c1aa21d3 | Bacteria | p__Firmicutes | c__Bacilli | o__Erysipelotrichales | f__Erysipelotrichaceae | g__Dubosiella | s__uncultured_bacterium |
| 2415d16b7e467e36400ae61ef0981724 | Bacteria | p__Proteobacteria | c__Gammaproteobacteria | o__Burkholderiales | f__Nitrosomonadaceae | g__mle1-7 |  |
| 950059a0c1d6d08fcce5af8971231843 | Bacteria | p__Actinobacteriota | c__Actinobacteria | o__Micrococcales | f__Brevibacteriaceae | g__Brevibacterium |  |
| 05126230 | Bacteria | p__Proteobacteria | c__Gammaproteobacteria | o__Enterobacterales | f__Yersiniaceae |  |  |
| 9bcb2a005b01641d69d46d9fef83f657 | Bacteria | p__Proteobacteria | c__Alphaproteobacteria |  |  |  |  |
| 16eb61d7d3e1958a9ef9976d2215b2bb | Bacteria | p__Bacteroidota | c__Bacteroidia | o__Flavobacteriales | f__Crocinitomicaceae | g__Fluviicola |  |
| 869271ab4711cc99aa795f71d5dc1c01 | Bacteria | p__Chloroflexi | c__Anaerolineae | o__SBR1031 | f__SBR1031 | g__SBR1031 |  |
| 25f89cdcfd885f65d340b93a36473609 | Bacteria | p__Firmicutes | c__Bacilli | o__Lactobacillales | f__Streptococcaceae | g__Streptococcus | s__Streptococcus_hyointestinalis |
| G000332995_1 | Bacteria | p__Actinobacteriota | c__Actinobacteria | o__Corynebacteriales | f__Nocardiaceae |  |  |
| eace9d6f0217407be4518589978dd5ff | Bacteria | p__Actinobacteriota | c__Actinobacteria | o__Pseudonocardiales | f__Pseudonocardiaceae | g__Pseudonocardia |  |
| GB-GCA-002903085.1-CP026197.1 | Bacteria | p__Proteobacteria | c__Gammaproteobacteria | o__Enterobacterales | f__Enterobacteriaceae |  |  |
| 7e24c84c3ee4f3c863b383710d181152 | Bacteria | p__Actinobacteriota | c__Acidimicrobiia | o__Microtrichales | f__Ilumatobacteraceae | g__Ilumatobacter |  |
| a3d373b8c560c3603bf57016cd3d7e8c | Bacteria | p__Actinobacteriota | c__Actinobacteria | o__Propionibacteriales | f__Propionibacteriaceae | g__Tessaracoccus |  |
| 882f43b5dc89a11d5590168a8391ce92 | Bacteria | p__Firmicutes | c__Clostridia | o__Lachnospirales | f__Lachnospiraceae |  |  |
| 6866e8781deba56b505544175d087a5e | Bacteria | p__Acidobacteriota | c__Vicinamibacteria | o__Vicinamibacterales | f__Vicinamibacteraceae | g__Vicinamibacteraceae |  |
| 03746785 | Bacteria | p__Planctomycetota | c__Planctomycetes | o__Pirellulales | f__Pirellulaceae | g__Pirellula |  |
| 04933320 | Bacteria | p__Bacteroidota | c__Bacteroidia | o__Chitinophagales | f__Chitinophagaceae | g__Flavisolibacter |  |
| 1b546745ef2285518d30a23ba0ab9a74 | Bacteria | p__Planctomycetota | c__Planctomycetes | o__Isosphaerales | f__Isosphaeraceae |  |  |
| RS-GCF-001036655.1-NZ-LEJW01000088.1 | Bacteria | p__Firmicutes | c__Bacilli | o__Lactobacillales | f__Streptococcaceae | g__Streptococcus |  |
| a544bdca29003afc9b2304cda2b01ca6 | Bacteria |  |  |  |  |  |  |
| 35910c7cad0139d8699bb47b2b005419 | Bacteria | p__Actinobacteriota | c__Actinobacteria | o__Micromonosporales | f__Micromonosporaceae |  |  |
| G003762715_1 | Bacteria | p__Bacteroidota | c__Bacteroidia | o__Bacteroidales | f__Muribaculaceae | g__Muribaculaceae | s__unidentified |
| 6ecb37d3e1f605165c47a09ef8e10669 | Bacteria | p__Proteobacteria | c__Alphaproteobacteria | o__Rhizobiales | f__Kaistiaceae | g__Kaistia |  |
| EF466117 | Bacteria | p__Actinobacteriota | c__Actinobacteria | o__Propionibacteriales | f__Nocardioidaceae |  |  |
| GB-GCA-910576865.1-CAJTKE010000008.1 | Bacteria | p__Firmicutes | c__Clostridia | o__Oscillospirales | f__Oscillospiraceae | g__Oscillibacter |  |
| fae437ce90a25630f79453349e2349f0 | Bacteria | p__Planctomycetota | c__Planctomycetes | o__Gemmatales | f__Gemmataceae | g__uncultured | s__uncultured_thermal |
| 05092717 | Bacteria | p__Proteobacteria | c__Alphaproteobacteria | o__Elsterales | f__uncultured | g__uncultured | s__uncultured_Stella |
| 30b89096bfa1754eea4191c2c6c84516 | Bacteria | p__Proteobacteria | c__Gammaproteobacteria | o__Pasteurellales | f__Pasteurellaceae | g__Haemophilus |  |
| 9b977d72758ae2678a25f72d88e585c4 | Bacteria | p__Actinobacteriota | c__Actinobacteria | o__Frankiales | f__Sporichthyaceae | g__Longivirga |  |
| 14521154 | Bacteria | p__Gemmatimonadota | c__Gemmatimonadetes | o__Gemmatimonadales | f__Gemmatimonadaceae | g__uncultured |  |
| RS-GCF-006351765.1-NZ-CP040903.1--4 | Bacteria | p__Proteobacteria | c__Gammaproteobacteria | o__Pseudomonadales | f__Moraxellaceae | g__Acinetobacter |  |
| a85f545097d66dafa26d38d49dc8c757 | Bacteria | p__Acidobacteriota | c__Acidobacteriae | o__Subgroup_2 | f__Subgroup_2 | g__Subgroup_2 |  |
| 5efc9c1dc5dd4015628fa05bcef3b825 | Bacteria | p__Proteobacteria | c__Alphaproteobacteria | o__Caulobacterales | f__Hyphomonadaceae | g__SWB02 | s__uncultured_bacterium |
| 04960401 | Bacteria | p__Actinobacteriota | c__Actinobacteria | o__Micrococcales | f__Microbacteriaceae |  |  |
| 0b89b2920c5e3e4a38566c4cd3a29a87 | Bacteria | p__Acidobacteriota | c__Acidobacteriae | o__Acidobacteriales | f__Acidobacteriaceae_(Subgroup_1) | g__uncultured | s__Acidobacteria_bacterium |
| 83ab31f8bcd5c2f59a3175fd9debcbb4 | Bacteria | p__Bacteroidota | c__Bacteroidia | o__Chitinophagales | f__Saprospiraceae | g__uncultured |  |
| 1331be8c0832cc74f104ea6891140458 | Bacteria | p__Cyanobacteria | c__Cyanobacteriia | o__Cyanobacteriales |  |  |  |
| 05031653 | Bacteria | p__Bacteroidota | c__Bacteroidia | o__Cytophagales | f__Cytophagaceae | g__Siphonobacter | s__Siphonobacter_sp. |
| FR733685 | Bacteria | p__Actinobacteriota | c__Actinobacteria | o__Micromonosporales | f__Micromonosporaceae | g__Actinoplanes |  |
| de3e26a17ac2104314650285f4565e99 | Bacteria | p__Proteobacteria | c__Gammaproteobacteria | o__Burkholderiales | f__Comamonadaceae |  |  |
| AF273081 | Bacteria | p__Proteobacteria | c__Alphaproteobacteria | o__Rhizobiales | f__Beijerinckiaceae | g__Bosea |  |
| 04959741 | Bacteria | p__Proteobacteria | c__Gammaproteobacteria | o__Burkholderiales | f__Comamonadaceae |  |  |
| 8302a5bdf8711edced0a02c1f1ebfa22 | Bacteria | p__Actinobacteriota | c__Actinobacteria | o__Corynebacteriales | f__Corynebacteriaceae | g__Corynebacterium |  |
| ab5179fdce3859cf19e80a97d65d9210 | Bacteria | p__Proteobacteria | c__Gammaproteobacteria | o__Burkholderiales | f__Comamonadaceae | g__Pelomonas |  |
| 0d62e28ca3144dd9647d6716bb0bbd94 | Bacteria | p__Proteobacteria | c__Alphaproteobacteria | o__Reyranellales | f__Reyranellaceae | g__Reyranella |  |
| 4ab0ca39460bfee47b3d8abb4a3d6501 | Bacteria | p__Verrucomicrobiota | c__Verrucomicrobiae | o__Opitutales | f__Opitutaceae | g__Lacunisphaera | s__Oleiharenicola_alkalitolerans |
| 04933806 | Bacteria | p__Bacteroidota | c__Bacteroidia | o__Chitinophagales | f__Chitinophagaceae | g__Ferruginibacter |  |
| 56ef7096bd54524ba4ea31040ce131a3 | Bacteria | p__Chloroflexi | c__Ktedonobacteria | o__Ktedonobacterales | f__Ktedonobacteraceae | g__HSB_OF53-F07 | s__uncultured_Ktedonobacter |
| 8b9cda47885aa4a7a9c29d55bfe0af47 | Bacteria | p__Firmicutes | c__Clostridia | o__Clostridia_UCG-014 | f__Clostridia_UCG-014 | g__Clostridia_UCG-014 |  |
| 3a5262979ec24bec46fad10b59b8bc35 | Bacteria | p__Proteobacteria | c__Gammaproteobacteria | o__Pseudomonadales | f__Pseudomonadaceae | g__Pseudomonas |  |
| RS-GCF-000426185.1-NZ-AUEW01000025.1 | Bacteria | p__Actinobacteriota | c__Actinobacteria | o__Micrococcales | f__Cellulomonadaceae |  |  |
| 39550eec915ae52c887262a748e199d3 | Bacteria | p__Firmicutes | c__Bacilli | o__Paenibacillales | f__Paenibacillaceae | g__Paenibacillus |  |
| MJ031-1-barcode30-umi37161bins-ubs-4 | Bacteria | p__Firmicutes | c__Clostridia | o__Clostridiales | f__Clostridiaceae | g__Clostridium_sensu_stricto_1 |  |
| 7206670179db991838424f782476b085 | Bacteria | p__Proteobacteria | c__Gammaproteobacteria | o__Xanthomonadales | f__Xanthomonadaceae |  |  |
| 52b9c23420444b13e775c9f5b9672bdf | Bacteria | p__NB1-j | c__NB1-j | o__NB1-j | f__NB1-j | g__NB1-j |  |
| 40a1431616135b0239c0c608f6ac75c3 | Bacteria | p__Firmicutes | c__Bacilli | o__Lactobacillales | f__Streptococcaceae | g__Streptococcus |  |
| 04943825 | Bacteria | p__Acidobacteriota | c__Blastocatellia | o__Blastocatellales | f__Blastocatellaceae |  |  |
| HE648174 | Bacteria | p__Proteobacteria | c__Gammaproteobacteria | o__Burkholderiales | f__Oxalobacteraceae | g__Undibacterium | s__Undibacterium_squillarum |
| a470bb11bdb88d7fae3e8f24a8cae9d3 | Bacteria | p__Firmicutes | c__Bacilli | o__Staphylococcales | f__Staphylococcaceae | g__Staphylococcus |  |
| 27379cdedbff3a4f93f0d995b10e6335 | Bacteria | p__Bacteroidota | c__Bacteroidia | o__Bacteroidales | f__Rikenellaceae | g__Rikenellaceae_RC9_gut_group | s__uncultured_prokaryote |
| MJ033-1-barcode36-umi7148bins-ubs-49 | Bacteria | p__Proteobacteria | c__Gammaproteobacteria | o__Burkholderiales | f__Rhodocyclaceae | g__uncultured |  |
| MJ033-1-barcode40-umi2190bins-ubs-166 | Bacteria | p__Bacteroidota | c__Bacteroidia | o__Chitinophagales | f__Chitinophagaceae | g__Flavisolibacter |  |
| 02863289 | Bacteria | p__Chloroflexi | c__Chloroflexia | o__Chloroflexales | f__Roseiflexaceae | g__uncultured | s__uncultured_Chloroflexi |
| AJ575430 | Bacteria | p__Bacteroidota | c__Bacteroidia | o__Flavobacteriales | f__Weeksellaceae | g__Cloacibacterium |  |
| 2de14c0f080b9146c766b7045651fc7c | Bacteria | p__Chloroflexi | c__Chloroflexia | o__Thermomicrobiales | f__JG30-KF-CM45 | g__JG30-KF-CM45 | s__uncultured_Chloroflexi |
| ec7552849c8d6afe16d1ded0dd9f34c7 | Bacteria | p__Bacteroidota | c__Bacteroidia | o__Bacteroidales | f__Dysgonomonadaceae | g__Proteiniphilum |  |
| 05036779 | Bacteria | p__Firmicutes | c__Bacilli | o__Bacillales | f__Planococcaceae | g__Sporosarcina | s__Sporosarcina_pasteurii |
| RS-GCF-004684265.1-NZ-CP038467.1 | Bacteria | p__Proteobacteria | c__Gammaproteobacteria | o__Enterobacterales | f__Yersiniaceae | g__Serratia |  |
| b29a7aba352d4fe37d333b8c41678cef | Bacteria | p__Actinobacteriota | c__Actinobacteria |  |  |  |  |
| 05021586 | Bacteria | p__Actinobacteriota | c__Actinobacteria | o__Streptomycetales | f__Streptomycetaceae | g__Streptomyces |  |
| 1b1a74018351ef57c323014db78cdf73 | Bacteria | p__Firmicutes | c__Clostridia | o__Peptostreptococcales-Tissierellales | f__Peptostreptococcales-Tissierellales | g__Finegoldia |  |
| 02360252 | Bacteria | p__Proteobacteria | c__Alphaproteobacteria | o__Sphingomonadales | f__Sphingomonadaceae | g__Sphingomonas |  |
| 941636a04c3deb40443d4f0b580194bd | Bacteria | p__Acidobacteriota | c__Subgroup_18 | o__Subgroup_18 | f__Subgroup_18 | g__Subgroup_18 | s__uncultured_bacterium |
| 29bc1c93a929f1e1e53ac3a08b9c563a | Bacteria | p__Proteobacteria | c__Alphaproteobacteria | o__Rhizobiales | f__Devosiaceae | g__Devosia |  |
| 17771243 | Bacteria | p__Armatimonadota | c__Chthonomonadetes | o__Chthonomonadales | f__Chthonomonadaceae | g__Chthonomonas | s__uncultured_bacterium |
| ec90760d8af6b6c35a9d4be5ff3777de | Bacteria | p__Firmicutes | c__Bacilli | o__Alicyclobacillales | f__Alicyclobacillaceae | g__Tumebacillus |  |
| 04995297 | Bacteria | p__Proteobacteria | c__Alphaproteobacteria | o__Azospirillales | f__Azospirillaceae | g__Azospirillum |  |
| GB-GCA-900066275.1-FMFG01000089.1 | Bacteria | p__Firmicutes | c__Clostridia | o__Oscillospirales | f__Ruminococcaceae | g__Faecalibacterium |  |
| 04881548 | Bacteria | p__Firmicutes | c__Bacilli | o__Bacillales | f__Bacillaceae | g__Bacillus |  |
| bb8a9abe38056360aa3d627d0ca0ffad | Bacteria | p__Proteobacteria | c__Gammaproteobacteria | o__Burkholderiales | f__SC-I-84 | g__SC-I-84 | s__uncultured_Nitrosomonadaceae |
| 10343221 | Bacteria | p__Bacteroidota | c__Bacteroidia | o__Flavobacteriales | f__Flavobacteriaceae | g__Myroides | s__Myroides_odoratus |
| 05089832 | Bacteria | p__Actinobacteriota | c__Actinobacteria | o__Micromonosporales | f__Micromonosporaceae | g__Dactylosporangium |  |
| f6a437a65bca6ade868ff311d7735c68 | Bacteria | p__Proteobacteria | c__Gammaproteobacteria |  |  |  |  |
| 04968106 | Bacteria | p__Firmicutes | c__Bacilli | o__Lactobacillales | f__Vagococcaceae | g__Vagococcus |  |
| 60d123516f2c89eca0350457813bb510 | Bacteria | p__Actinobacteriota | c__Thermoleophilia | o__Solirubrobacterales | f__Solirubrobacteraceae | g__Conexibacter |  |
| dcbfdc69a24252009d5b7d20a121ad3b | Bacteria | p__Proteobacteria | c__Gammaproteobacteria | o__Burkholderiales | f__Oxalobacteraceae |  |  |
| cb2fe02cfa165ecfd0f38d4f487dddd0 | Bacteria | p__Bacteroidota | c__Bacteroidia | o__Flavobacteriales | f__Blattabacteriaceae | g__Candidatus_Sulcia | s__Candidatus_Sulcia |
| RS-GCF-002205165.1-NZ-NIVS01000047.1 | Bacteria | p__Proteobacteria | c__Gammaproteobacteria | o__Xanthomonadales | f__Xanthomonadaceae | g__Stenotrophomonas |  |
| e4a3d21fe13de9cf0d1d5ac1fb095e65 | Bacteria | p__Actinobacteriota | c__Acidimicrobiia |  |  |  |  |
| ac02e696c04af1371201f43702c3426f | Bacteria | p__Firmicutes | c__Bacilli | o__Bacillales |  |  |  |
| 06740687 | Bacteria | p__Gemmatimonadota | c__Longimicrobia | o__Longimicrobiales | f__Longimicrobiaceae | g__YC-ZSS-LKJ147 |  |
| 08208797 | Bacteria | p__Proteobacteria | c__Alphaproteobacteria | o__Rhizobiales | f__Beijerinckiaceae | g__Methylobacterium-Methylorubrum |  |
| 5860914a432ffbc56a633fe8983e7a54 | Bacteria | p__Proteobacteria | c__Alphaproteobacteria | o__Rhizobiales | f__Beijerinckiaceae | g__Bosea |  |
| RS-GCF-001276505.1-NZ-LDWD01000163.1 | Bacteria | p__Proteobacteria | c__Gammaproteobacteria | o__Pseudomonadales | f__Moraxellaceae | g__Acinetobacter | s__Acinetobacter_soli |
| 541f377584f22de57db66bb501c20af4 | Bacteria | p__Bacteroidota | c__Bacteroidia | o__Sphingobacteriales | f__Sphingobacteriaceae | g__Sphingobacterium |  |
| 447da2ca205ca828d2c7a91508115bc3 | Bacteria | p__Actinobacteriota | c__Actinobacteria | o__Propionibacteriales | f__Propionibacteriaceae | g__Tessaracoccus |  |
| RS-GCF-003174995.1-NZ-QGIA01000131.1 | Bacteria | p__Firmicutes | c__Bacilli | o__Lactobacillales | f__Lactobacillaceae | g__Lactobacillus |  |
| 04900313 | Bacteria | p__Proteobacteria | c__Gammaproteobacteria | o__Pseudomonadales | f__Pseudomonadaceae | g__Pseudomonas |  |
| f26f21f0fb8dfa03a91bf68ba75f504e | Bacteria | p__Proteobacteria | c__Alphaproteobacteria | o__Rhizobiales | f__Beijerinckiaceae | g__Roseiarcus |  |
| MJ020-1-barcode31-umi6022bins-ubs-59 | Bacteria | p__Bacteroidota | c__Bacteroidia | o__Chitinophagales | f__Chitinophagaceae |  |  |
| MJ030-2-barcode58-umi110972bins-ubs-4 | Bacteria | p__Nitrospirota | c__Nitrospiria | o__Nitrospirales | f__Nitrospiraceae | g__Nitrospira |  |
| 1e9f4bebdcd999b8923381b553e62e4c | Bacteria | p__Proteobacteria | c__Alphaproteobacteria | o__uncultured | f__uncultured | g__uncultured | s__uncultured_actinobacterium |
| RS-GCF-016919385.1-NZ-CP070351.1 | Bacteria | p__Actinobacteriota | c__Actinobacteria | o__Corynebacteriales | f__Nocardiaceae | g__Gordonia |  |
| 47edf69e58c0ab4326394fcb61649eb3 | Bacteria | p__Firmicutes | c__Bacilli | o__Staphylococcales | f__Staphylococcaceae | g__Staphylococcus |  |
| 088b6b5b187328ff0676b962496b3207 | Bacteria | p__Proteobacteria | c__Gammaproteobacteria | o__Enterobacterales |  |  |  |
| 95cafd1cfdf4eb130f5b850fd8edd566 | Bacteria | p__Myxococcota | c__Polyangia | o__Polyangiales | f__Polyangiaceae | g__Pajaroellobacter |  |
| 06727434 | Bacteria | p__Gemmatimonadota | c__Gemmatimonadetes | o__Gemmatimonadales | f__Gemmatimonadaceae | g__uncultured | s__metagenome |
| 04909040 | Bacteria | p__Proteobacteria | c__Alphaproteobacteria | o__Rhizobiales | f__Rhizobiaceae | g__Aliihoeflea | s__uncultured_Aliihoeflea |
| c4bcb1d346ff231d958b27cb48f6519e | Bacteria | p__Proteobacteria | c__Alphaproteobacteria | o__Sphingomonadales | f__Sphingomonadaceae | g__Sphingopyxis |  |
| fb7d1d7ff193799cc1387d4d71437f9a | Bacteria | p__Firmicutes | c__Bacilli | o__Lactobacillales | f__Enterococcaceae | g__Enterococcus |  |
| d351fcad84de105d2337e1b0a54221fa | Bacteria | p__Proteobacteria | c__Gammaproteobacteria | o__Burkholderiales | f__A21b | g__A21b |  |
| MJ030-2-barcode59-umi61183bins-ubs-11 | Bacteria | p__Bacteroidota | c__Bacteroidia | o__Chitinophagales | f__Chitinophagaceae | g__Ferruginibacter |  |
| d7b0f3227293109fa9bfc430343b8b28 | Bacteria | p__Actinobacteriota | c__Thermoleophilia | o__Gaiellales | f__uncultured | g__uncultured |  |
| 96569089f20f53d277cf7a9b5c4ac47d | Bacteria | p__Firmicutes | c__Clostridia | o__Peptostreptococcales-Tissierellales | f__Peptostreptococcaceae |  |  |
| 16f142371b1a382a06539bef0ae5b457 | Bacteria | p__Proteobacteria | c__Gammaproteobacteria | o__Burkholderiales | f__uncultured | g__uncultured | s__beta_proteobacterium |
| 185f1dbca9524727afecd19b0bc5997a | Bacteria | p__Actinobacteriota | c__Actinobacteria | o__Propionibacteriales | f__Propionibacteriaceae | g__Microlunatus |  |
| 836fe4b074ddd93730714045764f4737 | Bacteria | p__Proteobacteria | c__Gammaproteobacteria | o__Pseudomonadales | f__Moraxellaceae | g__Acinetobacter |  |
| af1c61944c65c0a620c450f446990739 | Bacteria | p__Firmicutes | c__Clostridia | o__Oscillospirales | f__Butyricicoccaceae | g__Butyricicoccus |  |
| c213509a2812718fb1a4af17c2e5a93b | Bacteria | p__Firmicutes | c__Clostridia | o__Lachnospirales | f__Lachnospiraceae |  |  |
| KU973604 | Bacteria | p__Proteobacteria | c__Gammaproteobacteria | o__Burkholderiales | f__Comamonadaceae |  |  |
| 8dff0a2f2845b79a9628d54eca81290b | Bacteria | p__Proteobacteria | c__Gammaproteobacteria | o__Enterobacterales | f__Enterobacteriaceae | g__Escherichia-Shigella |  |
| 1027ff6792c8742c3d4403e0afc75e1e | Bacteria | p__Actinobacteriota | c__Thermoleophilia | o__Gaiellales |  |  |  |
| 3c4caf1f164861a09f68721e6963c4a1 | Bacteria | p__Chloroflexi | c__KD4-96 | o__KD4-96 | f__KD4-96 | g__KD4-96 |  |
| 2cab01190056b92a57d8bcfd636ec1b1 | Bacteria | p__Bacteroidota | c__Bacteroidia | o__Flavobacteriales | f__Weeksellaceae | g__Chryseobacterium |  |
| bec2224f62dde0f9dbea8812d1429e5e | Bacteria | p__Proteobacteria | c__Alphaproteobacteria | o__Rhodobacterales | f__Rhodobacteraceae |  |  |
| G900099705_1 | Bacteria | p__Actinobacteriota | c__Actinobacteria | o__Micrococcales | f__Microbacteriaceae | g__Agrococcus | s__Agrococcus_jejuensis |
| RS-GCF-900114755.1-NZ-FOUC01000047.1 | Bacteria | p__Firmicutes | c__Bacilli | o__Lactobacillales | f__Enterococcaceae | g__Enterococcus |  |
| e98b2ef8fc76a0b8a2e9089fc5cd700a | Bacteria | p__Firmicutes | c__Bacilli | o__Lactobacillales | f__Streptococcaceae | g__Lactococcus |  |
| 47ff9744cf29edb5c7c21f76556a4b97 | Bacteria | p__Proteobacteria | c__Alphaproteobacteria | o__Elsterales | f__uncultured | g__uncultured |  |
| 04893128 | Bacteria | p__Methylomirabilota | c__Methylomirabilia | o__Rokubacteriales | f__Rokubacteriales | g__Rokubacteriales |  |
| 4febbdd33808fff1f47dafa234f2b7dc | Bacteria | p__Proteobacteria | c__Gammaproteobacteria | o__Pseudomonadales | f__Moraxellaceae | g__Enhydrobacter |  |
| adb56f3c44bcb158bf38f96f3c98e003 | Bacteria | p__Proteobacteria | c__Alphaproteobacteria | o__Rhodobacterales | f__Rhodobacteraceae | g__Paracoccus |  |
| cab9b4b2e83910e119eb6a1adad1783b | Bacteria | p__Proteobacteria | c__Gammaproteobacteria | o__Burkholderiales | f__Rhodocyclaceae |  |  |
| AB110498 | Bacteria | p__Proteobacteria | c__Gammaproteobacteria | o__Xanthomonadales | f__Rhodanobacteraceae | g__Dyella |  |
| 04965856 | Bacteria | p__Firmicutes | c__Bacilli | o__Bacillales | f__Planococcaceae | g__Sporosarcina | s__Sporosarcina_pasteurii |
| RS-GCF-000805695.1-NZ-JSYQ01000060.1 | Bacteria | p__Bacteroidota | c__Bacteroidia | o__Flavobacteriales | f__Weeksellaceae | g__Empedobacter |  |
| fb5e721353e7664e228ed600308d7bdb | Bacteria | p__Proteobacteria | c__Gammaproteobacteria | o__Burkholderiales | f__Alcaligenaceae | g__Parapusillimonas |  |
| c285a4ff381f6825e71146a8f206182e | Bacteria | p__Firmicutes | c__Clostridia | o__Lachnospirales | f__Lachnospiraceae |  |  |
| FR851928 | Bacteria | p__Proteobacteria | c__Alphaproteobacteria | o__Rhizobiales | f__Xanthobacteraceae | g__Rhodopseudomonas |  |
| 08e6d1c18142d0399c25223b4efcc9bf | Bacteria | p__Proteobacteria | c__Alphaproteobacteria | o__Rhizobiales | f__Rhizobiaceae |  |  |
| 59fb8be9b27d692b3ffe976b03357000 | Bacteria | p__Proteobacteria | c__Alphaproteobacteria | o__Azospirillales | f__uncultured | g__uncultured | s__uncultured_bacterium |
| G000520775_1 | Bacteria | p__Firmicutes | c__Bacilli | o__Paenibacillales | f__Paenibacillaceae | g__Paenibacillus |  |
| MJ006-1-barcode38-umi102699bins-ubs-4 | Bacteria | p__Firmicutes | c__Clostridia | o__Lachnospirales | f__Lachnospiraceae | g__Lachnospiraceae_UCG-010 | s__uncultured_organism |
| HM362432 | Bacteria | p__Proteobacteria | c__Alphaproteobacteria | o__Rhizobiales | f__Beijerinckiaceae | g__Microvirga |  |
| e4a49c96f0c79e9934cb5373b61cd829 | Bacteria | p__Proteobacteria | c__Alphaproteobacteria | o__Elsterales |  |  |  |
| 9730c92169e95da7c8440ccf2fc1a6c8 | Bacteria | p__Firmicutes | c__Bacilli | o__Caldalkalibacillales | f__Caldalkalibacillaceae | g__Caldalkalibacillus | s__Caldalkalibacillus_thermarum |
| RS-GCF-011927635.1-NZ-JAATJB010000034.1 | Bacteria | p__Proteobacteria | c__Alphaproteobacteria | o__Sphingomonadales | f__Sphingomonadaceae | g__Sphingomonas |  |
| 3e11faeb8edc11fadddef6fcf73a0217 | Bacteria | p__Actinobacteriota | c__Actinobacteria | o__Micrococcales | f__Microbacteriaceae |  |  |
| 2291e59c39dade72a8724e941502a85d | Bacteria | p__Actinobacteriota | c__Acidimicrobiia | o__IMCC26256 | f__IMCC26256 | g__IMCC26256 |  |
| 98c1275c8793ec1e1f77980a0333347f | Bacteria | p__Actinobacteriota | c__Actinobacteria | o__Corynebacteriales | f__Mycobacteriaceae | g__Mycobacterium |  |
| MJ034-1-barcode31-umi28316bins-ubs-3 | Bacteria | p__Proteobacteria | c__Alphaproteobacteria | o__Rhizobiales | f__Xanthobacteraceae | g__uncultured |  |
| 2fbfce7716a9e3b0066830502436b4e6 | Bacteria | p__Proteobacteria | c__Gammaproteobacteria | o__Burkholderiales | f__Comamonadaceae | g__Lampropedia |  |
| 08491814 | Bacteria | p__Proteobacteria | c__Alphaproteobacteria | o__Rhizobiales | f__Rhizobiaceae | g__Allorhizobium-Neorhizobium-Pararhizobium-Rhizobium |  |
| a9e9782b203bba825239515b7aec6033 | Bacteria | p__Proteobacteria | c__Gammaproteobacteria | o__CCD24 | f__CCD24 | g__CCD24 |  |
| ff3bb41c7fa32d7d21e0c324981ba5f1 | Bacteria | p__Proteobacteria | c__Alphaproteobacteria | o__Tistrellales | f__Geminicoccaceae | g__uncultured |  |
| 7bb6e57fcdb9131ad1d00f4b9f7eccb5 | Bacteria | p__Proteobacteria | c__Gammaproteobacteria | o__Enterobacterales | f__Enterobacteriaceae |  |  |
| 3d8eefd9a21086063ac1570477df4bf7 | Bacteria | p__Actinobacteriota | c__Actinobacteria | o__Micromonosporales | f__Micromonosporaceae |  |  |
| 14812716 | Bacteria | p__Firmicutes | c__Clostridia | o__Clostridia_vadinBB60_group | f__Clostridia_vadinBB60_group | g__Clostridia_vadinBB60_group |  |
| 05131721 | Bacteria | p__Proteobacteria | c__Gammaproteobacteria | o__Burkholderiales | f__Burkholderiaceae | g__Pandoraea |  |
| 05021368 | Bacteria | p__Actinobacteriota | c__Actinobacteria | o__Micrococcales | f__Micrococcaceae |  |  |
| 04911170 | Bacteria | p__Proteobacteria | c__Gammaproteobacteria | o__Xanthomonadales | f__Xanthomonadaceae | g__Stenotrophomonas |  |
| AB193724 | Bacteria | p__Proteobacteria | c__Gammaproteobacteria | o__Burkholderiales | f__Methylophilaceae | g__Methylophilus |  |
| d02548b01045f019159322b239027b7a | Bacteria | p__Proteobacteria | c__Alphaproteobacteria | o__Sphingomonadales | f__Sphingomonadaceae | g__Novosphingobium |  |
| 967785babc6bda9a77fdb832ec98cac7 | Bacteria | p__Proteobacteria | c__Gammaproteobacteria | o__Xanthomonadales | f__Xanthomonadaceae | g__Stenotrophomonas |  |
| MF503624 | Bacteria | p__Proteobacteria | c__Alphaproteobacteria | o__Sphingomonadales | f__Sphingomonadaceae | g__Sphingomonas |  |
| bfc3117e18885defa5dfb896fa71ba6e | Bacteria | p__Proteobacteria | c__Gammaproteobacteria | o__Pseudomonadales | f__Pseudomonadaceae | g__Pseudomonas |  |
| 05025167 | Bacteria | p__Firmicutes | c__Clostridia | o__Lachnospirales | f__Lachnospiraceae |  |  |
| MJ032-2-barcode73-umi105535bins-ubs-3 | Bacteria | p__Proteobacteria | c__Gammaproteobacteria | o__Pseudomonadales | f__Pseudomonadaceae | g__Pseudomonas |  |
| 18317292 | Bacteria | p__Actinobacteriota | c__Actinobacteria | o__Propionibacteriales | f__Nocardioidaceae | g__Aeromicrobium |  |
| RS-GCF-014204055.1-NZ-JACHKH010000014.1 | Bacteria | p__Proteobacteria | c__Gammaproteobacteria | o__Pseudomonadales | f__Pseudomonadaceae | g__Pseudomonas |  |
| AY859683 | Bacteria | p__Actinobacteriota | c__Actinobacteria | o__Corynebacteriales | f__Mycobacteriaceae | g__Mycobacterium |  |
| MG271952 | Bacteria | p__Proteobacteria | c__Alphaproteobacteria | o__uncultured | f__uncultured | g__uncultured |  |
| d0133bfff916d526bf14b8e637ff2bb4 | Bacteria | p__Proteobacteria | c__Gammaproteobacteria | o__Gammaproteobacteria_Incertae_Sedis | f__Unknown_Family | g__Acidibacter |  |
| RS-GCF-001814125.1-NZ-KV814961.1 | Bacteria | p__Firmicutes | c__Bacilli | o__Staphylococcales | f__Staphylococcaceae | g__Staphylococcus |  |
| RS-GCF-001669335.1-NZ-LZLL01000080.1 | Bacteria | p__Actinobacteriota | c__Actinobacteria | o__Corynebacteriales | f__Mycobacteriaceae | g__Mycobacterium |  |
| 0a807149fbbbffd37a9be900547b55a4 | Bacteria | p__Acidobacteriota | c__Acidobacteriae | o__Bryobacterales | f__Bryobacteraceae | g__Bryobacter |  |
| 045bf1eb44e2bf234bfbd10f2d43dd72 | Bacteria | p__Proteobacteria | c__Gammaproteobacteria | o__Burkholderiales | f__Alcaligenaceae |  |  |
| 04881422 | Bacteria | p__Actinobacteriota | c__Actinobacteria | o__Pseudonocardiales | f__Pseudonocardiaceae | g__Actinomycetospora |  |
| G900114995_1 | Bacteria | p__Bacteroidota | c__Bacteroidia | o__Chitinophagales | f__Chitinophagaceae | g__Chitinophaga | s__Chitinophaga_pinensis |
| 02247625 | Bacteria | p__Actinobacteriota | c__Actinobacteria | o__Propionibacteriales | f__Nocardioidaceae | g__Marmoricola |  |
| 04956041 | Bacteria | p__Actinobacteriota | c__Thermoleophilia | o__Gaiellales | f__uncultured | g__uncultured |  |
| RS-GCF-000812065.1-NZ-JPZK01000022.1 | Bacteria | p__Actinobacteriota | c__Actinobacteria | o__Micrococcales | f__Micrococcaceae |  |  |
| 04318111 | Bacteria | p__WPS-2 | c__WPS-2 | o__WPS-2 | f__WPS-2 | g__WPS-2 |  |
| RS-GCF-009822395.1-NZ-VDHX01000006.1 | Bacteria | p__Proteobacteria | c__Gammaproteobacteria | o__Enterobacterales | f__Enterobacteriaceae |  |  |
| MJ009-2-barcode59-umi119203bins-ubs-3 | Bacteria | p__Proteobacteria | c__Gammaproteobacteria | o__Enterobacterales |  |  |  |
| b19713852d50276a00aed800f7d60773 | Bacteria | p__Actinobacteriota | c__Thermoleophilia | o__Gaiellales |  |  |  |
| 13df646376475ba01a77714cb9db7278 | Bacteria | p__Actinobacteriota | c__Actinobacteria | o__Corynebacteriales | f__Mycobacteriaceae | g__Mycobacterium |  |
| 7d00cb38379d5e8eaf578612ee3892e3 | Bacteria | p__Bacteroidota | c__Bacteroidia | o__Bacteroidales | f__Muribaculaceae | g__Muribaculum | s__uncultured_bacterium |
| f6ad421477363a931e07917c7c2f94f3 | Bacteria | p__Gemmatimonadota | c__Gemmatimonadetes | o__Gemmatimonadales | f__Gemmatimonadaceae | g__uncultured |  |
| 02244703 | Bacteria | p__Actinobacteriota | c__Actinobacteria | o__Corynebacteriales | f__Corynebacteriaceae | g__Corynebacterium |  |
| d7e05f609c411a5a46d0b03e43829dc5 | Bacteria | p__Chloroflexi | c__Chloroflexia | o__Thermomicrobiales | f__JG30-KF-CM45 | g__JG30-KF-CM45 |  |
| f345354afb37089a4f8b3b7014dc13f7 | Bacteria | p__Proteobacteria | c__Gammaproteobacteria | o__Pseudomonadales | f__Moraxellaceae | g__Acinetobacter |  |
| 868f836175e8aca33fa571b7444c7364 | Bacteria | p__Actinobacteriota | c__Actinobacteria | o__Streptomycetales | f__Streptomycetaceae | g__Streptomyces |  |
| RS-GCF-018861755.1-NZ-JAHLEF010000107.1 | Bacteria | p__Firmicutes | c__Clostridia | o__Clostridiales | f__Clostridiaceae | g__Clostridium_sensu_stricto_13 |  |
| 4884fe3074426731721dfad2dab37679 | Bacteria | p__Acidobacteriota | c__Vicinamibacteria | o__Vicinamibacterales | f__uncultured | g__uncultured |  |
| 356a8c3569c171e59f13369fe973900d | Bacteria | p__Bacteroidota | c__Bacteroidia | o__Bacteroidales | f__Rikenellaceae | g__Alistipes |  |
| c483d8668903344d0ce77dfde5996fb3 | Bacteria | p__Proteobacteria | c__Gammaproteobacteria | o__Pseudomonadales | f__Moraxellaceae | g__Acinetobacter |  |
| 00faf3e683b5dcee60a711e62d88cdb8 | Bacteria | p__Bdellovibrionota | c__Oligoflexia | o__0319-6G20 | f__0319-6G20 | g__0319-6G20 |  |
| 5a63dbc5ccf488bbb2a8316532fb3e8c | Bacteria | p__Bacteroidota | c__Bacteroidia | o__Sphingobacteriales | f__AKYH767 | g__AKYH767 |  |
| RS-GCF-019052575.1-NZ-JAHQVA010000078.1 | Bacteria | p__Bacteroidota | c__Bacteroidia | o__Bacteroidales | f__Bacteroidaceae | g__Bacteroides |  |
| 58406f37c3f67948bc7c8fe33a26f67d | Bacteria | p__Proteobacteria | c__Gammaproteobacteria | o__Pseudomonadales | f__Moraxellaceae | g__Acinetobacter |  |
| 2ce1840518f1bae2650e351c093e7c54 | Bacteria | p__Proteobacteria | c__Gammaproteobacteria | o__Steroidobacterales | f__Steroidobacteraceae | g__uncultured | s__Hypsibius_dujardini |
| 88a20c0458f552d0174caf41acc229f6 | Bacteria | p__Firmicutes | c__Bacilli | o__Lactobacillales | f__Enterococcaceae | g__Enterococcus |  |
| d0a232bb75cd0cb1b907771f65017c0e | Bacteria | p__Proteobacteria | c__Alphaproteobacteria | o__Caulobacterales | f__Caulobacteraceae |  |  |
| 05030566 | Bacteria | p__Proteobacteria | c__Gammaproteobacteria | o__Burkholderiales | f__Comamonadaceae | g__Comamonas |  |
| e0fbf6ac5319a0346f2dd5813dc9cdce | Bacteria | p__Bacteroidota | c__Bacteroidia | o__Bacteroidales | f__Prolixibacteraceae | g__uncultured | s__uncultured_anaerobic |
| b0179ffca5b1a9d4c2960523934d6b7b | Bacteria | p__Proteobacteria | c__Alphaproteobacteria | o__Rhizobiales | f__Xanthobacteraceae |  |  |
| 5c1ccd9a58d859981b3ff05113b6dc94 | Bacteria | p__Chloroflexi | c__Chloroflexia | o__Chloroflexales | f__Roseiflexaceae | g__uncultured | s__uncultured_Chloroflexaceae |
| 311bb4cc5d1840a13d89d0d1f18aecf0 | Bacteria | p__Chloroflexi | c__Anaerolineae | o__SBR1031 | f__A4b | g__A4b |  |
| dc7c7bd3fb2686c0b30990a082770322 | Bacteria | p__Proteobacteria | c__Gammaproteobacteria | o__Burkholderiales | f__Comamonadaceae | g__Rhizobacter |  |
| RS-GCF-009911595.1-NZ-QWKG01000143.1 | Bacteria | p__Firmicutes | c__Clostridia | o__Oscillospirales | f__Oscillospiraceae |  |  |
| 04909608 | Bacteria | p__Proteobacteria | c__Alphaproteobacteria | o__Rhizobiales | f__Devosiaceae | g__Devosia |  |
| 659dd1e26b79f59371e0d619b9a34d85 | Bacteria | p__Actinobacteriota | c__Acidimicrobiia | o__Microtrichales | f__Ilumatobacteraceae | g__Ilumatobacter |  |
| 44de97532ef3b6c8a53a3b7e278549e7 | Bacteria | p__Bacteroidota | c__Bacteroidia | o__Sphingobacteriales | f__Sphingobacteriaceae | g__Sphingobacterium |  |
| KC755038 | Bacteria | p__Proteobacteria | c__Gammaproteobacteria | o__Burkholderiales | f__Comamonadaceae |  |  |
| 0b18cac107bf8eceef606664191bbb3f | Bacteria | p__Proteobacteria | c__Gammaproteobacteria | o__Burkholderiales | f__Comamonadaceae | g__Aquabacterium |  |
| 0db784baf9defabd2ccdfeffa9e5394e | Bacteria | p__Actinobacteriota | c__Actinobacteria | o__Micrococcales | f__Micrococcaceae |  |  |
| G008271475_1 | Bacteria | p__Proteobacteria | c__Alphaproteobacteria | o__Rhodobacterales | f__Rhodobacteraceae | g__Rhodobacter |  |
| 76836964a097b79fc78102bd044b1193 | Bacteria | p__Proteobacteria | c__Gammaproteobacteria | o__Enterobacterales |  |  |  |
| GB-GCA-900542515.1-UQOA01000002.1 | Bacteria | p__Firmicutes | c__Negativicutes | o__Acidaminococcales | f__Acidaminococcaceae | g__Phascolarctobacterium |  |
| 19665315 | Bacteria | p__Firmicutes | c__Bacilli | o__Erysipelotrichales | f__Erysipelotrichaceae | g__Ileibacterium | s__Ileibacterium_valens |
| 04908924 | Bacteria | p__Proteobacteria | c__Alphaproteobacteria | o__Rhizobiales | f__Rhizobiaceae | g__Allorhizobium-Neorhizobium-Pararhizobium-Rhizobium |  |
| 46e149c30aefff40894652cfee5090e8 | Bacteria | p__Firmicutes | c__Clostridia | o__Oscillospirales | f__Ruminococcaceae | g__Ruminococcaceae | s__uncultured_bacterium |
| 03841604 | Bacteria | p__Cyanobacteria | c__Cyanobacteriia | o__Cyanobacteriales | f__Coleofasciculaceae | g__uncultured | s__uncultured_bacterium |
| 02403197 | Bacteria | p__Patescibacteria | c__Saccharimonadia | o__Saccharimonadales | f__Saccharimonadaceae | g__TM7x |  |
| RS-GCF-910574405.1-NZ-CAJTAQ010000093.1 | Bacteria | p__Firmicutes | c__Bacilli | o__Lactobacillales | f__Lactobacillaceae | g__Lactobacillus |  |
| MJ030-1-barcode42-umi33946bins-ubs-15 | Bacteria | p__Acidobacteriota | c__Subgroup_5 | o__Subgroup_5 | f__Subgroup_5 | g__Subgroup_5 | s__uncultured_bacterium |
| 02411940 | Bacteria | p__Proteobacteria | c__Alphaproteobacteria | o__Rhizobiales | f__Rhizobiaceae | g__Allorhizobium-Neorhizobium-Pararhizobium-Rhizobium |  |
| RS-GCF-003628305.1-NZ-RBZM01000028.1 | Bacteria | p__Firmicutes | c__Bacilli | o__Paenibacillales | f__Paenibacillaceae | g__Cohnella |  |
| 420c8596e862b2b5e0ad051f3e2fb7fc | Bacteria | p__Proteobacteria | c__Gammaproteobacteria | o__Pseudomonadales | f__Pseudomonadaceae | g__Pseudomonas |  |
| e5c71ef4bd27df333110206949476869 | Bacteria | p__Actinobacteriota | c__Actinobacteria | o__Bifidobacteriales | f__Bifidobacteriaceae | g__Bifidobacterium |  |
| feb10694e4e4e7ef93da3e451c785abc | Bacteria | p__Firmicutes | c__Bacilli | o__Lactobacillales | f__Enterococcaceae | g__Enterococcus |  |
| EF612765 | Bacteria | p__Proteobacteria | c__Alphaproteobacteria | o__Rhodospirillales | f__Rhodospirillaceae | g__Novispirillum | s__Novispirillum_itersonii |
| 414f0930a9e0e299ae819fa034af399b | Bacteria | p__Methylomirabilota | c__Methylomirabilia | o__Rokubacteriales | f__Rokubacteriales | g__Rokubacteriales | s__uncultured_bacterium |
| G003762875_1 | Bacteria | p__Bacteroidota | c__Bacteroidia | o__Bacteroidales | f__Muribaculaceae | g__Muribaculaceae | s__uncultured_bacterium |
| 5450335b90374b158100975e4d12ba25 | Bacteria | p__Proteobacteria | c__Gammaproteobacteria | o__Burkholderiales | f__Nitrosomonadaceae | g__mle1-7 |  |
| RS-GCF-000702865.1-NZ-JNKM01000001.1 | Bacteria | p__Actinobacteriota | c__Actinobacteria | o__Bifidobacteriales | f__Bifidobacteriaceae | g__Bifidobacterium |  |
| 1e896a6ed8e14bcbbfe97ad3d8685920 | Bacteria | p__Actinobacteriota | c__Actinobacteria | o__Actinomycetales | f__Actinomycetaceae | g__Flaviflexus |  |
| 15689210 | Bacteria | p__Firmicutes | c__Clostridia | o__Oscillospirales | f__Ruminococcaceae | g__Ruminococcus |  |
| KY012060 | Bacteria | p__Proteobacteria | c__Alphaproteobacteria | o__Rhodobacterales | f__Rhodobacteraceae | g__Roseobacter_clade_CHAB-I-5_lineage |  |
| 04911130 | Bacteria | p__Proteobacteria | c__Gammaproteobacteria | o__Xanthomonadales | f__Xanthomonadaceae | g__Arenimonas |  |
| MJ006-1-barcode38-umi101890bins-ubs-4 | Bacteria | p__Firmicutes | c__Clostridia | o__Oscillospirales | f__Ruminococcaceae | g__Incertae_Sedis | s__uncultured_bacterium |
| RS-GCF-013240295.1-NZ-JABETK010000001.1 | Bacteria | p__Proteobacteria | c__Alphaproteobacteria | o__Rhizobiales | f__Rhizobiaceae | g__Mesorhizobium |  |
| ef89b424fed1832d5d0070468f69147b | Bacteria | p__Proteobacteria | c__Gammaproteobacteria | o__Burkholderiales | f__Comamonadaceae |  |  |
| 521568e6abfa35120f4cc11fd33288c1 | Bacteria | p__Proteobacteria | c__Alphaproteobacteria | o__Sphingomonadales | f__Sphingomonadaceae |  |  |
| 04875838 | Bacteria | p__Proteobacteria | c__Gammaproteobacteria | o__Pseudomonadales | f__Pseudomonadaceae | g__Pseudomonas |  |
| 927933c9d8b4e26e2d2c93e7e638ba19 | Bacteria | p__Gemmatimonadota | c__Gemmatimonadetes | o__Gemmatimonadales | f__Gemmatimonadaceae | g__uncultured |  |
| HG793791 | Bacteria | p__Firmicutes | c__Bacilli | o__Lactobacillales | f__Streptococcaceae | g__Streptococcus |  |
| 023f8baee55e99868e34f3d190c13943 | Bacteria | p__Actinobacteriota | c__Actinobacteria | o__Propionibacteriales | f__Nocardioidaceae | g__Nocardioides |  |
| e7169bdf766139d822b42f2a7f983e53 | Bacteria | p__Actinobacteriota | c__Actinobacteria | o__Corynebacteriales | f__Nocardiaceae | g__Williamsia |  |
| 02168500 | Bacteria | p__Fusobacteriota | c__Fusobacteriia | o__Fusobacteriales | f__Leptotrichiaceae | g__Leptotrichia |  |
| db5167cc349d249f5111cd90fba3751f | Bacteria | p__Proteobacteria | c__Gammaproteobacteria | o__Burkholderiales | f__Methylophilaceae | g__Methylophilus | s__uncultured_Methylophilus |
| 52284dd7efd723b4dbc837e19616d8da | Bacteria | p__Bacteroidota | c__Bacteroidia | o__Bacteroidales | f__Bacteroidaceae | g__Bacteroides | s__Bacteroides_sartorii |
| MJ031-1-barcode38-umi35972bins-ubs-5 | Bacteria | p__Firmicutes | c__Clostridia | o__Oscillospirales | f__Ruminococcaceae | g__Ruminococcus | s__unidentified |
| 73f374e0665c2a29999e82bd4bb6f54f | Bacteria | p__Proteobacteria | c__Gammaproteobacteria | o__Alteromonadales | f__Alteromonadaceae | g__Rheinheimera |  |
| 29aa03a83191754f50e0853e0e2f810a | Bacteria | p__Proteobacteria | c__Alphaproteobacteria | o__Rhizobiales | f__Rhizobiaceae |  |  |
| 04833692 | Bacteria | p__Actinobacteriota | c__Actinobacteria | o__Corynebacteriales | f__Corynebacteriaceae | g__Corynebacterium |  |
| MJ030-2-barcode65-umi109260bins-ubs-4 | Bacteria | p__Proteobacteria | c__Gammaproteobacteria | o__Gammaproteobacteria_Incertae_Sedis | f__Unknown_Family | g__Acidibacter | s__uncultured_Steroidobacter |
| 12643332 | Bacteria | p__Campilobacterota | c__Campylobacteria | o__Campylobacterales | f__Helicobacteraceae | g__Helicobacter |  |
| ab073c188e360ccce05cda753e082ee3 | Bacteria | p__Firmicutes | c__Bacilli | o__Bacillales | f__Planococcaceae | g__Lysinibacillus |  |
| MJ030-1-barcode42-umi108532bins-ubs-3 | Bacteria | p__Acidobacteriota | c__Blastocatellia | o__Pyrinomonadales | f__Pyrinomonadaceae | g__RB41 | s__uncultured_Acidobacteriaceae |
| 2276d86fd877a8049afcaab7a578f868 | Bacteria | p__Chloroflexi | c__Chloroflexia | o__Chloroflexales | f__Roseiflexaceae | g__uncultured |  |
| 04902119 | Bacteria | p__Verrucomicrobiota | c__Verrucomicrobiae | o__Pedosphaerales | f__Pedosphaeraceae | g__Pedosphaeraceae |  |
| 05365109 | Bacteria | p__Bacteroidota | c__Bacteroidia | o__Bacteroidales | f__Muribaculaceae | g__Muribaculaceae |  |
| 6c4b8a4674111d182746cb78bbd000f1 | Bacteria | p__Firmicutes | c__Bacilli | o__Erysipelotrichales | f__Erysipelotrichaceae | g__ZOR0006 |  |
| 03483757e62e2ab70127de088e0f79cb | Bacteria | p__Firmicutes | c__Clostridia | o__Lachnospirales | f__Lachnospiraceae | g__Anaerostipes |  |
| 04987479 | Bacteria | p__Actinobacteriota | c__Actinobacteria | o__Micromonosporales | f__Micromonosporaceae | g__uncultured |  |
| MK621282 | Bacteria | p__Proteobacteria | c__Alphaproteobacteria | o__Rhizobiales | f__Rhizobiaceae |  |  |
| 121fbbc90a47f161951352a164ee0a7b | Bacteria | p__Chloroflexi | c__KD4-96 | o__KD4-96 | f__KD4-96 | g__KD4-96 |  |
| 082f86cb5c6c0bd623bead7926569ae4 | Bacteria | p__Firmicutes | c__Clostridia | o__Lachnospirales | f__Lachnospiraceae |  |  |
| 49f7f2edb27a9035246a2f3ecd1f0513 | Bacteria | p__Firmicutes | c__Bacilli | o__Staphylococcales | f__Staphylococcaceae | g__Corticicoccus | s__Corticicoccus_populi |
| 14311962 | Bacteria | p__Acidobacteriota | c__Vicinamibacteria | o__Vicinamibacterales | f__uncultured | g__uncultured |  |
| e0cdabef2cba7629e1c850c63b3e870a | Bacteria | p__Gemmatimonadota | c__Gemmatimonadetes | o__Gemmatimonadales | f__Gemmatimonadaceae | g__Gemmatimonas |  |
| RS-GCF-902160305.1-NZ-CABGND010000002.1 | Bacteria | p__Firmicutes | c__Bacilli | o__Lactobacillales | f__Enterococcaceae | g__Enterococcus |  |
| da27e740afc4d505902204fd19e66053 | Bacteria | p__Bacteroidota | c__Bacteroidia | o__Chitinophagales | f__Chitinophagaceae | g__Taibaiella |  |
| bd01b9c558e99c8970eda52a3b992a49 | Bacteria | p__Nitrospirota | c__Thermodesulfovibrionia | o__uncultured | f__uncultured | g__uncultured | s__uncultured_bacterium |
| 04957312 | Bacteria | p__Proteobacteria | c__Gammaproteobacteria | o__Burkholderiales | f__Comamonadaceae |  |  |
| 04941301 | Bacteria | p__Acidobacteriota | c__Vicinamibacteria | o__Vicinamibacterales | f__uncultured | g__uncultured |  |
| 2b71b3577de14320eb616e4e0bf76066 | Bacteria | p__Firmicutes | c__Bacilli | o__Lactobacillales | f__Leuconostocaceae | g__Weissella |  |
| 9767ae1347883be896fc7942a9a61435 | Bacteria | p__Proteobacteria | c__Alphaproteobacteria | o__Sphingomonadales | f__Sphingomonadaceae |  |  |
| G000972575_5 | Bacteria | p__Firmicutes | c__Bacilli | o__Staphylococcales | f__Staphylococcaceae | g__Staphylococcus |  |
| 73d60533b7b55441ba4a2c30a699b8fa | Bacteria | p__Proteobacteria | c__Gammaproteobacteria | o__Pseudomonadales | f__Pseudomonadaceae | g__Pseudomonas |  |
| X55598 | Bacteria | p__Actinobacteriota | c__Actinobacteria | o__Corynebacteriales | f__Mycobacteriaceae | g__Mycobacterium |  |
| KY363640 | Bacteria | p__Proteobacteria | c__Gammaproteobacteria | o__Salinisphaerales | f__Solimonadaceae | g__Nevskia |  |
| AJ430347 | Bacteria | p__Proteobacteria | c__Gammaproteobacteria | o__Burkholderiales | f__Comamonadaceae | g__Comamonas |  |
| FJ194436 | Bacteria | p__Proteobacteria | c__Alphaproteobacteria | o__Sphingomonadales | f__Sphingomonadaceae | g__Sphingomonas |  |
| G000403295_3 | Bacteria | p__Firmicutes | c__Clostridia | o__Lachnospirales | f__Lachnospiraceae | g__Lachnospiraceae_UCG-006 | s__uncultured_bacterium |
| 6ef42c6e7c49bf0a7b0226620ccdabc0 | Bacteria | p__Firmicutes | c__Bacilli | o__Lactobacillales | f__Lactobacillaceae | g__Lactobacillus |  |
| 130d57f630f48ea2ee7c4a7582a19a54 | Bacteria | p__Proteobacteria | c__Gammaproteobacteria | o__Xanthomonadales | f__Xanthomonadaceae |  |  |
| b27e1dbd11968bfd6714409881337060 | Bacteria | p__Chloroflexi | c__Chloroflexia | o__Thermomicrobiales | f__AKYG1722 | g__AKYG1722 |  |
| 8248c8677f621292538ea42dc86a1d4e | Bacteria | p__Chloroflexi | c__KD4-96 | o__KD4-96 | f__KD4-96 | g__KD4-96 | s__metagenome |
| 472633943a4af1ce9bf8e00962ed28d3 | Bacteria | p__Bacteroidota | c__Bacteroidia | o__Bacteroidales | f__Muribaculaceae | g__Muribaculaceae | s__uncultured_bacterium |
| b61cc9dd57a3d5bd5a7d4ad73e7f5a14 | Bacteria | p__Proteobacteria | c__Alphaproteobacteria | o__Rhizobiales | f__Rhizobiaceae |  |  |
| 2dad0f12446d1e7a1301c067a565b529 | Bacteria | p__Gemmatimonadota | c__Gemmatimonadetes | o__Gemmatimonadales | f__Gemmatimonadaceae | g__uncultured |  |
| af45163d4f94aef7a216e9396295a5e9 | Bacteria | p__Bacteroidota | c__Bacteroidia | o__Bacteroidales | f__Marinifilaceae | g__Marinifilum | s__Marinifilum_fragile |
| 02304412 | Bacteria | p__Proteobacteria | c__Alphaproteobacteria | o__Rhizobiales | f__Methyloligellaceae | g__uncultured |  |
| AJ247194 | Bacteria | p__Proteobacteria | c__Alphaproteobacteria | o__Caulobacterales | f__Caulobacteraceae | g__Asticcacaulis | s__Asticcacaulis_excentricus |
| 08021372 | Bacteria | p__Proteobacteria | c__Alphaproteobacteria | o__Rhizobiales | f__Beijerinckiaceae | g__Camelimonas | s__uncultured_bacterium |
| FM208261 | Bacteria | p__Actinobacteriota | c__Actinobacteria | o__Micromonosporales | f__Micromonosporaceae |  |  |
| c231ede101149ba94138198fb9f5aef5 | Bacteria | p__Firmicutes | c__Bacilli | o__Lactobacillales | f__Enterococcaceae | g__Enterococcus |  |
| 04342527 | Bacteria | p__WPS-2 | c__WPS-2 | o__WPS-2 | f__WPS-2 | g__WPS-2 | s__uncultured_forest |
| MJ031-2-barcode67-umi27720bins-ubs-15 | Bacteria | p__Verrucomicrobiota | c__Verrucomicrobiae | o__Pedosphaerales | f__Pedosphaeraceae | g__uncultured |  |
| 3077dddf2c47fc30f9c7539817d61927 | Bacteria | p__Proteobacteria | c__Gammaproteobacteria | o__Burkholderiales | f__Comamonadaceae | g__Aquabacterium |  |
| b3fd0a76da1834507019ba3d5bef59f9 | Bacteria | p__Chloroflexi | c__Ktedonobacteria | o__Ktedonobacterales | f__JG30-KF-AS9 | g__JG30-KF-AS9 | s__uncultured_bacterium |
| c8922161a01f4f13e28fa640665cdefa | Bacteria | p__Proteobacteria | c__Gammaproteobacteria | o__Burkholderiales | f__Nitrosomonadaceae | g__mle1-7 |  |
| 02360234 | Bacteria | p__Proteobacteria | c__Alphaproteobacteria | o__Defluviicoccales | f__Defluviicoccaceae | g__Defluviicoccus | s__uncultured_Defluviicoccus |
| 2a7dbe348d92241743fcfacf6bb844b3 | Bacteria | p__Proteobacteria | c__Alphaproteobacteria | o__Rhizobiales | f__Rhizobiales_Incertae_Sedis | g__Nordella |  |
| KC771032 | Bacteria | p__Deinococcota | c__Deinococci | o__Deinococcales | f__Deinococcaceae | g__Deinococcus |  |
| MJ030-2-barcode65-umi100900bins-ubs-4 | Bacteria | p__Acidobacteriota | c__Acidobacteriae | o__Solibacterales | f__Solibacteraceae | g__Candidatus_Solibacter | s__bacterium_Ellin7504 |
| f8c8d92d75bc0395856c21d0553a7e13 | Bacteria | p__Firmicutes | c__Bacilli | o__Paenibacillales | f__Paenibacillaceae | g__Saccharibacillus | s__Saccharibacillus_sp. |
| e1eeb78f54e4290b0b4f512cb7550dfd | Bacteria | p__Proteobacteria | c__Alphaproteobacteria | o__Rhizobiales | f__Rhizobiaceae |  |  |
| bc537d0d50e333a14949bd60d0ca1396 | Bacteria | p__Planctomycetota | c__Planctomycetes | o__Gemmatales | f__Gemmataceae | g__uncultured |  |
| d19af53c1439fa3876c30a6dc7630a99 | Bacteria | p__Proteobacteria | c__Gammaproteobacteria | o__Burkholderiales | f__Comamonadaceae |  |  |
| 7a11614e6e07a9f832136f83948bced1 | Bacteria | p__Proteobacteria | c__Alphaproteobacteria | o__Acetobacterales | f__Acetobacteraceae | g__Roseomonas |  |
| MJ006-2-barcode53-umi18634bins-ubs-16 | Bacteria | p__Firmicutes | c__Clostridia | o__Clostridia_UCG-014 | f__Clostridia_UCG-014 | g__Clostridia_UCG-014 |  |
| 1de6aa228ee8c48d8ee2465a69c12647 | Bacteria | p__Proteobacteria | c__Gammaproteobacteria | o__Pseudomonadales | f__Moraxellaceae | g__Alkanindiges |  |
| 8781e4ef8bf1d7a69c691efd1d630234 | Bacteria | p__Proteobacteria | c__Gammaproteobacteria | o__Burkholderiales | f__A21b | g__A21b |  |
| df6d1273e4a173626cef7b074a8e48ea | Bacteria | p__Proteobacteria | c__Alphaproteobacteria | o__Rhizobiales | f__Devosiaceae |  |  |
| RS-GCF-014651795.1-NZ-BMXO01000033.1 | Bacteria | p__Proteobacteria | c__Gammaproteobacteria | o__Oceanospirillales | f__Halomonadaceae | g__Halomonas |  |
| 04984488 | Bacteria | p__Myxococcota | c__Polyangia | o__Polyangiales | f__BIrii41 | g__BIrii41 |  |
| e303c4c2f727db900e09734dd88f2c22 | Bacteria | p__Bacteroidota | c__Bacteroidia | o__Sphingobacteriales | f__Sphingobacteriaceae | g__Pseudopedobacter | s__Pseudopedobacter_saltans |
| 6452a925ba62f42c449683745f2a3bdd | Bacteria | p__Actinobacteriota | c__MB-A2-108 | o__MB-A2-108 | f__MB-A2-108 | g__MB-A2-108 | s__uncultured_actinobacterium |
| MJ031-1-barcode42-umi37397bins-ubs-4 | Bacteria | p__Bacteroidota | c__Bacteroidia | o__Cytophagales | f__Microscillaceae | g__Ohtaekwangia |  |
| 999c3b33e758290ccc237a25c79bd5a5 | Bacteria | p__Actinobacteriota | c__Actinobacteria | o__Micrococcales | f__Microbacteriaceae |  |  |
| cbbc87efc4123f32decade2700943245 | Bacteria | p__Actinobacteriota | c__Thermoleophilia | o__Gaiellales | f__Gaiellaceae | g__Gaiella |  |
| fb6a6294228e2b355608082d90ba0f1a | Bacteria | p__Proteobacteria | c__Alphaproteobacteria | o__Rhizobiales | f__Xanthobacteraceae |  |  |
| 995e5c1e09df39ee3aaa087c1da86c84 | Bacteria | p__Firmicutes | c__Clostridia | o__Clostridiales | f__Clostridiaceae | g__Clostridium_sensu_stricto_11 | s__Clostridium_amazonense |
| 8f641fef844db9a67b861695cc52d7a5 | Bacteria | p__Acidobacteriota | c__Vicinamibacteria | o__Vicinamibacterales | f__uncultured | g__uncultured |  |
| 5cc4ec459e5f5be331c29bf0f01cc5ec | Bacteria | p__Firmicutes | c__Bacilli | o__Lactobacillales | f__Enterococcaceae | g__Enterococcus |  |
| MJ034-1-barcode26-umi17840bins-ubs-13 | Bacteria | p__Myxococcota | c__Myxococcia | o__Myxococcales | f__Myxococcaceae | g__uncultured | s__uncultured_delta |
| 6e023b993e17a50b2b888e07a157c054 | Bacteria | p__Firmicutes | c__Clostridia | o__Clostridia_vadinBB60_group | f__Clostridia_vadinBB60_group | g__Clostridia_vadinBB60_group | s__uncultured_Clostridia |
| ae7480bcda58d601aa3cefae734f59ed | Bacteria | p__Methylomirabilota | c__Methylomirabilia | o__Rokubacteriales | f__Rokubacteriales | g__Rokubacteriales | s__uncultured_bacterium |
| MJ031-2-barcode54-umi34441bins-ubs-9 | Bacteria | p__Proteobacteria | c__Gammaproteobacteria | o__Pseudomonadales | f__Moraxellaceae | g__Acinetobacter |  |
| 1767974fb29adc6e7dbe058b37ac45b6 | Bacteria | p__Actinobacteriota | c__Actinobacteria | o__Streptosporangiales | f__Thermomonosporaceae | g__Actinoallomurus |  |
| 9b3d2e55e07900e243950a79b31dce8a | Bacteria | p__Bacteroidota | c__Bacteroidia | o__Bacteroidales | f__Prevotellaceae | g__Prevotella | s__Prevotella_melaninogenica |
| 9727f250da6c0349f9793db511e72204 | Bacteria | p__Chloroflexi | c__Chloroflexia | o__Chloroflexales | f__Roseiflexaceae | g__uncultured | s__uncultured_bacterium |
| MJ010-2-barcode64-umi145020bins-ubs-3 | Bacteria | p__Proteobacteria | c__Gammaproteobacteria | o__Enterobacterales |  |  |  |
| KM670026 | Bacteria | p__Proteobacteria | c__Gammaproteobacteria | o__Burkholderiales | f__Comamonadaceae |  |  |
| 12512024 | Bacteria | p__Proteobacteria | c__Gammaproteobacteria | o__Xanthomonadales | f__Rhodanobacteraceae | g__Mizugakiibacter |  |
| 6b70d01f650df0d1961a283927b71cf8 | Bacteria | p__Bacteroidota | c__Bacteroidia | o__Flavobacteriales | f__Flavobacteriaceae |  |  |
| bcfe94a215e94d3a80e3a7d0b8734064 | Bacteria | p__Firmicutes | c__Bacilli | o__Bacillales | f__Bacillaceae | g__Bacillus |  |
| 66e7d05bde7f0bffba80f92559736ad3 | Bacteria | p__Verrucomicrobiota | c__Chlamydiae | o__Chlamydiales |  |  |  |
| db795f5843807fd60602b25dc02f6b3e | Bacteria | p__Chloroflexi | c__Anaerolineae | o__RBG-13-54-9 | f__RBG-13-54-9 | g__RBG-13-54-9 | s__uncultured_prokaryote |
| e77912bdc8c2d8d4e6d12794df7b6378 | Bacteria | p__Actinobacteriota | c__Actinobacteria | o__Micrococcales | f__Intrasporangiaceae |  |  |
| GB-GCA-003472845.1-QRMZ01000074.1 | Bacteria | p__Firmicutes | c__Bacilli | o__Lactobacillales | f__Enterococcaceae | g__Enterococcus |  |
| 9bf80b737d5b389f07fcbc542256f87b | Bacteria | p__Actinobacteriota | c__Acidimicrobiia | o__Microtrichales | f__uncultured | g__uncultured | s__wastewater_metagenome |
| 04878226 | Bacteria | p__Actinobacteriota | c__Actinobacteria | o__Kineosporiales | f__Kineosporiaceae | g__Quadrisphaera | s__Quadrisphaera_granulorum |
| cee094ebcee47c37c5cb9f9f0859da35 | Bacteria | p__Myxococcota | c__Myxococcia | o__Myxococcales | f__Myxococcaceae |  |  |
| 6d0c199835ed5af54e6e32c2c74d5729 | Bacteria | p__Proteobacteria | c__Gammaproteobacteria | o__Enterobacterales | f__Enterobacteriaceae | g__Klebsiella |  |
| fd3017cae484d6f61b6716d6ba3407fa | Bacteria | p__Actinobacteriota | c__Acidimicrobiia | o__Microtrichales | f__uncultured | g__uncultured |  |
| aaf2f2dbe470d55e25cc2c40b1b9daf8 | Bacteria | p__Desulfobacterota | c__uncultured | o__uncultured | f__uncultured | g__uncultured |  |
| 05011395 | Bacteria | p__Proteobacteria | c__Gammaproteobacteria | o__Enterobacterales | f__Erwiniaceae |  |  |
| MJ033-1-barcode40-umi13599bins-ubs-22 | Bacteria | p__Acidobacteriota | c__Blastocatellia | o__Pyrinomonadales | f__Pyrinomonadaceae | g__RB41 |  |
| adb0ebec7a7b48f27b99bd323355558c | Bacteria | p__Actinobacteriota | c__Actinobacteria | o__Kineosporiales | f__Kineosporiaceae |  |  |
| 640722f6e4b4c83a99ea0d3eee54bf8e | Bacteria | p__Firmicutes | c__Bacilli | o__Lactobacillales | f__Enterococcaceae | g__Enterococcus |  |
| 21e63da42d4e5d1c8b952a9beb76366c | Bacteria | p__Firmicutes | c__Bacilli | o__Exiguobacterales | f__Exiguobacteraceae | g__Exiguobacterium |  |
| a9c0a632f1f56d59495caa8bf4be667a | Bacteria | p__Chloroflexi | c__KD4-96 | o__KD4-96 | f__KD4-96 | g__KD4-96 |  |
| f79953076d19e94542b235e339f24528 | Bacteria | p__Acidobacteriota | c__Vicinamibacteria | o__Vicinamibacterales | f__Vicinamibacteraceae | g__Vicinamibacteraceae |  |
| 91d67ba4b798f99f2bfd80b594ceef1c | Bacteria | p__Actinobacteriota | c__Actinobacteria | o__Micrococcales | f__Microbacteriaceae |  |  |
| 084198871c89ecf5183d057a1f3df081 | Bacteria | p__Proteobacteria | c__Gammaproteobacteria | o__Burkholderiales | f__A21b | g__A21b | s__uncultured_beta |
| 06b6f6d02606b4e76a233d3d4ddaa36d | Bacteria | p__Proteobacteria | c__Alphaproteobacteria | o__Rhizobiales | f__Rhizobiaceae | g__Allorhizobium-Neorhizobium-Pararhizobium-Rhizobium |  |
| MJ006-1-barcode28-umi157746bins-ubs-3 | Bacteria | p__Firmicutes | c__Clostridia | o__Oscillospirales | f__Ruminococcaceae | g__Subdoligranulum |  |
| AY500143 | Bacteria | p__Actinobacteriota | c__Actinobacteria | o__Pseudonocardiales | f__Pseudonocardiaceae | g__Pseudonocardia |  |
| RS-GCF-000365385.1-NZ-KB846783.1 | Bacteria | p__Actinobacteriota | c__Actinobacteria | o__Streptomycetales | f__Streptomycetaceae | g__Streptomyces |  |
| 04876761 | Bacteria | p__Proteobacteria | c__Alphaproteobacteria | o__Sphingomonadales | f__Sphingomonadaceae |  |  |
| 04895574 | Bacteria | p__Proteobacteria | c__Alphaproteobacteria | o__Reyranellales | f__Reyranellaceae | g__Reyranella |  |
| 17388153 | Bacteria | p__Chloroflexi | c__Ktedonobacteria | o__Ktedonobacterales | f__Ktedonobacteraceae |  |  |
| 19cc36e9a45eadf15dff15a32dffa9fe | Bacteria | p__Proteobacteria | c__Alphaproteobacteria | o__Defluviicoccales | f__Defluviicoccaceae | g__Defluviicoccus |  |
| 999535b2edca2e31dba2770d885bde84 | Bacteria | p__Myxococcota | c__Polyangia | o__Polyangiales | f__Polyangiaceae | g__Minicystis |  |
| a2393a2e928fd6a7e9f0a3e9e9e19d06 | Bacteria | p__Proteobacteria | c__Alphaproteobacteria | o__Rhizobiales | f__Rhizobiaceae |  |  |
| 19022322 | Bacteria | p__Proteobacteria | c__Gammaproteobacteria | o__Burkholderiales | f__Sutterellaceae | g__Parasutterella | s__Burkholderiales_bacterium |
| 02267271 | Bacteria | p__Proteobacteria | c__Alphaproteobacteria | o__Dongiales | f__Dongiaceae | g__Dongia |  |
| f4ab05a428d9d156a3e9ba912a6e5dd4 | Bacteria | p__Proteobacteria | c__Alphaproteobacteria | o__Reyranellales | f__Reyranellaceae | g__Reyranella |  |
| 8402e22a7433517a77bcc48d9c42d82d | Bacteria | p__Bacteroidota | c__Bacteroidia | o__Chitinophagales | f__Saprospiraceae | g__uncultured |  |
| MJ010-2-barcode69-umi157044bins-ubs-3 | Bacteria | p__Firmicutes | c__Clostridia | o__Lachnospirales | f__Lachnospiraceae | g__Lachnospiraceae_NK4A136_group |  |
| MJ030-2-barcode65-umi19574bins-ubs-31 | Bacteria | p__Verrucomicrobiota | c__Verrucomicrobiae | o__Pedosphaerales | f__Pedosphaeraceae | g__Pedosphaeraceae |  |
| 17409055 | Bacteria | p__Actinobacteriota | c__Thermoleophilia | o__Gaiellales | f__Gaiellaceae | g__Gaiella |  |
| e6e3054f89e859ae43e821985753dd0c | Bacteria | p__Actinobacteriota | c__Actinobacteria | o__Propionibacteriales | f__Nocardioidaceae | g__Nocardioides |  |
| 45a19ecad0dc11f89a3672aa71f045df | Bacteria | p__Firmicutes | c__Clostridia | o__Peptostreptococcales-Tissierellales | f__Peptostreptococcaceae | g__Romboutsia |  |
| MJ034-1-barcode40-umi19533bins-ubs-11 | Bacteria | p__Acidobacteriota | c__Acidobacteriae | o__Acidobacteriales | f__uncultured | g__uncultured |  |
| 14340306 | Bacteria | p__Acidobacteriota | c__Vicinamibacteria | o__Vicinamibacterales | f__uncultured | g__uncultured |  |
| a581ee2e2e3f188d01593c3f73d6c159 | Bacteria | p__Bacteroidota | c__Bacteroidia | o__Chitinophagales | f__Chitinophagaceae |  |  |
| RS-GCF-001426825.1-NZ-LMFM01000005.1 | Bacteria | p__Proteobacteria | c__Alphaproteobacteria | o__Sphingomonadales | f__Sphingomonadaceae | g__Sphingomonas |  |
| 2afe44886e084d0fa0183cf7319e23af | Bacteria | p__Proteobacteria | c__Alphaproteobacteria | o__Rhizobiales | f__Methyloligellaceae | g__uncultured |  |
| c1bec0ca1dcd3ac8341460273bb0ca3e | Bacteria | p__Myxococcota | c__Polyangia | o__Haliangiales | f__Haliangiaceae | g__Haliangium |  |
| 04899811 | Bacteria | p__Acidobacteriota | c__Vicinamibacteria | o__Vicinamibacterales | f__Vicinamibacteraceae | g__Vicinamibacteraceae |  |
| MJ031-1-barcode30-umi37791bins-ubs-4 | Bacteria | p__Proteobacteria | c__Alphaproteobacteria | o__Rhizobiales | f__Rhizobiales_Incertae_Sedis | g__Nordella |  |
| MJ020-1-barcode29-umi15860bins-ubs-24 | Bacteria | p__Bacteroidota | c__Bacteroidia | o__Chitinophagales | f__Chitinophagaceae | g__Terrimonas |  |
| a73e890ae3a8ad37de7f97b54d3dbb6d | Bacteria | p__Planctomycetota | c__Planctomycetes | o__Isosphaerales | f__Isosphaeraceae |  |  |
| 2681ad3ff822953c65e5cb2937f4bccb | Bacteria | p__Acidobacteriota | c__Acidobacteriae | o__Subgroup_2 | f__Subgroup_2 | g__Subgroup_2 |  |
| ddfce48ff25bbb7f426f7b1147f6bd25 | Bacteria | p__Actinobacteriota | c__Coriobacteriia | o__Coriobacteriales | f__Coriobacteriaceae | g__Collinsella |  |
| MJ030-2-barcode65-umi103110bins-ubs-3 | Bacteria | p__Acidobacteriota | c__Acidobacteriae | o__Solibacterales | f__Solibacteraceae | g__Candidatus_Solibacter |  |
| 05131888 | Bacteria | p__Proteobacteria | c__Gammaproteobacteria | o__Burkholderiales | f__Comamonadaceae | g__Schlegelella |  |
| b45710a346ccdcaae511837af19eb134 | Bacteria | p__Gemmatimonadota | c__Gemmatimonadetes | o__Gemmatimonadales | f__Gemmatimonadaceae | g__uncultured |  |
| 04941312 | Bacteria | p__Acidobacteriota | c__Vicinamibacteria | o__Vicinamibacterales | f__uncultured | g__uncultured |  |
| b238f8b72be1e7dbbbd219a7b71e6376 | Bacteria |  |  |  |  |  |  |
| KM396265 | Bacteria | p__Actinobacteriota | c__Actinobacteria | o__Micromonosporales | f__Micromonosporaceae | g__Actinoplanes |  |
| eadb94093f41767edb5c06b5b58129ba | Bacteria | p__Cloacimonadota | c__Cloacimonadia | o__Cloacimonadales | f__W27 | g__W27 | s__uncultured_Cloacimonetes |
| 04993441 | Bacteria | p__Proteobacteria | c__Alphaproteobacteria | o__Rhizobiales | f__Beijerinckiaceae | g__Methylobacterium-Methylorubrum | s__Methylobacterium_aerolatum |
| 9a82bbad60e3819edc20b2c745861284 | Bacteria | p__Acidobacteriota | c__Acidobacteriae | o__AKIW659 | f__AKIW659 | g__AKIW659 |  |
| AY131212 | Bacteria | p__Proteobacteria | c__Gammaproteobacteria | o__Burkholderiales | f__Alcaligenaceae |  |  |
| 9d7621817db7dc43eac5fd5f5067f7f2 | Bacteria | p__Actinobacteriota | c__Actinobacteria | o__Frankiales | f__Acidothermaceae | g__Acidothermus | s__Thermomonosporaceae_bacterium |
| 0c653e60c04b2022054b47a740c9956e | Bacteria | p__Proteobacteria | c__Alphaproteobacteria | o__Sphingomonadales | f__Sphingomonadaceae | g__Sphingomonas |  |
| 501b76f48f00a10eff52e3a48a748b3a | Bacteria | p__Firmicutes | c__Clostridia | o__Peptostreptococcales-Tissierellales | f__Peptostreptococcales-Tissierellales | g__Guggenheimella | s__uncultured_bacterium |
| 04875892 | Bacteria | p__Proteobacteria | c__Gammaproteobacteria | o__Pseudomonadales | f__Moraxellaceae | g__Acinetobacter |  |
| 4269984cd31c6fb3f53c877b9ddd2b19 | Bacteria | p__Nitrospirota | c__Nitrospiria | o__Nitrospirales | f__Nitrospiraceae | g__Nitrospira |  |
| ca57b6244ae2d4c6ff23fc22655dbda7 | Bacteria | p__Firmicutes | c__Clostridia | o__Peptostreptococcales-Tissierellales | f__Sedimentibacteraceae | g__Sedimentibacter |  |
| 02250029 | Bacteria | p__Proteobacteria | c__Alphaproteobacteria | o__Rhodobacterales | f__Rhodobacteraceae | g__Rubellimicrobium | s__uncultured_bacterium |
| 1e80bd65ca122ea9baf423b4b593e1c1 | Bacteria | p__Proteobacteria | c__Alphaproteobacteria | o__Rhodobacterales | f__Rhodobacteraceae |  |  |
| 08509845 | Bacteria | p__Proteobacteria | c__Alphaproteobacteria | o__Rhizobiales | f__Rhizobiaceae | g__Shinella |  |
| 754829bde7f1252b96071f3b25bd7c81 | Bacteria | p__Proteobacteria | c__Gammaproteobacteria | o__Pseudomonadales | f__Moraxellaceae | g__Acinetobacter |  |
| 84c6e98659edce39a8863ebc1b1a22b8 | Bacteria | p__Proteobacteria | c__Gammaproteobacteria | o__Burkholderiales | f__Comamonadaceae |  |  |
| a8d0a845fdb95008f1cb3cb2edc06748 | Bacteria | p__Actinobacteriota | c__Actinobacteria | o__Corynebacteriales | f__Nocardiaceae | g__Rhodococcus |  |
| 18325020 | Bacteria | p__Chloroflexi | c__Chloroflexia | o__Thermomicrobiales | f__JG30-KF-CM45 | g__JG30-KF-CM45 |  |
| 95c050f9dba6af1268ae37120ca2e8a9 | Bacteria | p__Proteobacteria | c__Alphaproteobacteria | o__Sphingomonadales | f__Sphingomonadaceae |  |  |
| 19712127 | Bacteria | p__Firmicutes | c__Bacilli | o__Erysipelotrichales | f__Erysipelotrichaceae | g__Ileibacterium | s__Ileibacterium_valens |
| 04952404 | Bacteria | p__Firmicutes | c__Bacilli | o__Paenibacillales | f__Paenibacillaceae | g__Saccharibacillus |  |
| RS-GCF-014653695.1-NZ-BNAR01000037.1 | Bacteria | p__Actinobacteriota | c__Actinobacteria | o__Pseudonocardiales | f__Pseudonocardiaceae | g__Lechevalieria |  |
| 1a0b3b51578635cd4367d0c04c891fd8 | Bacteria | p__Actinobacteriota | c__Thermoleophilia | o__Gaiellales | f__uncultured | g__uncultured | s__bacterium_Ellin6515 |
| 04968550 | Bacteria | p__Bacteroidota | c__Bacteroidia | o__Cytophagales | f__Microscillaceae | g__uncultured | s__uncultured_soil |
| 9ae8ce23354d542f53439fd8b34eea08 | Bacteria | p__Actinobacteriota | c__Acidimicrobiia | o__Microtrichales | f__uncultured | g__uncultured |  |
| 3226bb609c8a21caed25c6620bfe0dd6 | Bacteria | p__Proteobacteria | c__Gammaproteobacteria | o__Burkholderiales | f__Comamonadaceae | g__Comamonas |  |
| d2aae8b55b567bc3fecf768f8fe39a75 | Bacteria | p__Proteobacteria | c__Gammaproteobacteria | o__Pseudomonadales | f__Moraxellaceae | g__Enhydrobacter |  |
| e1685b0bd262fc35375966bf35a9e712 | Bacteria | p__Proteobacteria | c__Alphaproteobacteria | o__Rhodobacterales | f__Rhodobacteraceae | g__Paracoccus |  |
| dc882b754ea6ce296a4ebb272bd2de6e | Bacteria | p__Proteobacteria | c__Gammaproteobacteria | o__Salinisphaerales | f__Solimonadaceae | g__Nevskia | s__uncultured_bacterium |
| 8b501e8f6f7ac3d83b11e8f922b162e2 | Bacteria | p__Firmicutes | c__Clostridia | o__Lachnospirales | f__Lachnospiraceae | g__Tyzzerella |  |
| 04989364 | Bacteria | p__Myxococcota | c__Myxococcia | o__Myxococcales | f__Myxococcaceae |  |  |
| MJ031-1-barcode27-umi37077bins-ubs-4 | Bacteria | p__Proteobacteria | c__Gammaproteobacteria | o__Burkholderiales | f__Rhodocyclaceae |  |  |
| 5d6066c6353ac3de9fbddf986ef0f75b | Bacteria | p__Firmicutes | c__Clostridia | o__Lachnospirales | f__Lachnospiraceae | g__Lachnospira |  |
| f753e5603099032e53fa9ebaebbf8ef2 | Bacteria | p__Actinobacteriota | c__Actinobacteria | o__Corynebacteriales | f__Corynebacteriaceae | g__Corynebacterium |  |
| 418d340dd1237ae82be9aa4b951c93a5 | Bacteria | p__Proteobacteria | c__Gammaproteobacteria |  |  |  |  |
| 72192bd3c542e41e6507384bd9c5d1af | Bacteria | p__Firmicutes | c__Bacilli | o__Staphylococcales | f__Staphylococcaceae | g__Staphylococcus |  |
| 93d20be8913b5e0c9bb0e749defaed3d | Bacteria | p__Desulfobacterota | c__Desulfuromonadia | o__Desulfuromonadia | f__Desulfuromonadaceae | g__Desulfuromonas |  |
| EF660757 | Bacteria | p__Proteobacteria | c__Alphaproteobacteria | o__Rhodobacterales | f__Rhodobacteraceae | g__Paracoccus |  |
| db723b16caa7b3b88fa0904f7cedba08 | Bacteria | p__Proteobacteria | c__Gammaproteobacteria | o__Burkholderiales | f__TRA3-20 | g__TRA3-20 | s__uncultured_gamma |
| a5af9a13c20f74d4b6448199812cafa3 | Bacteria | p__Actinobacteriota | c__Acidimicrobiia | o__Microtrichales | f__Microtrichaceae | g__uncultured |  |
| 08176630 | Bacteria | p__Proteobacteria | c__Alphaproteobacteria | o__Rhizobiales | f__Rhizobiales_Incertae_Sedis | g__Alsobacter |  |
| MJ033-1-barcode49-umi36794bins-ubs-4 | Bacteria | p__Proteobacteria | c__Gammaproteobacteria | o__Burkholderiales | f__Comamonadaceae |  |  |
| MF958452 | Bacteria | p__Proteobacteria | c__Alphaproteobacteria | o__Rhizobiales | f__Xanthobacteraceae |  |  |
| 416c34bf46548d641ce382d7b4c043eb | Bacteria | p__Proteobacteria | c__Gammaproteobacteria | o__Burkholderiales | f__Comamonadaceae |  |  |
| 0dadfdced3d8e60e60abd5a739664b52 | Bacteria | p__Proteobacteria | c__Gammaproteobacteria | o__Xanthomonadales | f__Xanthomonadaceae | g__Stenotrophomonas |  |
| 8a04f82aa300e2e8b83f6f1c146030e2 | Bacteria | p__Proteobacteria | c__Gammaproteobacteria | o__Pseudomonadales | f__Pseudomonadaceae | g__Pseudomonas |  |
| 86da381049c875119f271bd3554fda22 | Bacteria | p__Firmicutes | c__Bacilli | o__Lactobacillales | f__Lactobacillaceae | g__Lactobacillus |  |
| RS-GCF-013096895.1-NZ-SDLE01000084.1 | Bacteria | p__Firmicutes | c__Bacilli | o__Bacillales | f__Bacillaceae | g__Geobacillus |  |
| RS-GCF-002977295.1-NZ-NRKW01000026.1 | Bacteria | p__Proteobacteria | c__Gammaproteobacteria | o__Enterobacterales | f__Enterobacteriaceae |  |  |
| d848817030321a5e6ea29721bf5f9304 | Bacteria | p__Firmicutes | c__Bacilli | o__Erysipelotrichales | f__Erysipelotrichaceae | g__Erysipelothrix |  |
| 635f3f3aaea85cf1c4e26b29fe0e2c7f | Bacteria | p__Actinobacteriota | c__Coriobacteriia | o__Coriobacteriales | f__Atopobiaceae | g__Olsenella |  |
| 0e8c110fd15bb337a4931f50c3e55bcc | Bacteria | p__Proteobacteria | c__Gammaproteobacteria | o__Burkholderiales | f__Comamonadaceae | g__Comamonas |  |
| 04944351 | Bacteria | p__Actinobacteriota | c__Thermoleophilia | o__Solirubrobacterales | f__67-14 | g__67-14 |  |
| cbc20fd32758e812ca43ece379b1548d | Bacteria | p__Gemmatimonadota | c__Gemmatimonadetes | o__Gemmatimonadales | f__Gemmatimonadaceae | g__uncultured |  |
| 2b1c4c7ca3e51e1485e15fb31c984f92 | Bacteria | p__Proteobacteria | c__Alphaproteobacteria | o__Rhizobiales | f__Xanthobacteraceae | g__uncultured |  |
| be074b2874f31368bed6d730a89d69fe | Bacteria | p__Proteobacteria | c__Alphaproteobacteria | o__Rhizobiales | f__Beijerinckiaceae | g__Methylobacterium-Methylorubrum |  |
| fd9485bccc3ecf0330b033e0780acd5c | Bacteria | p__MBNT15 | c__MBNT15 | o__MBNT15 | f__MBNT15 | g__MBNT15 |  |
| 5021c87bd66203250564c0ab58dafd5e | Bacteria | p__Proteobacteria | c__Gammaproteobacteria | o__Enterobacterales | f__Enterobacteriaceae | g__Escherichia-Shigella |  |
| AB252201 | Bacteria | p__Proteobacteria | c__Alphaproteobacteria | o__Rhizobiales | f__Beijerinckiaceae | g__Methylobacterium-Methylorubrum |  |
| 64308f3b7e518f22da7bb4dfb25a6470 | Bacteria | p__Chloroflexi | c__Ktedonobacteria | o__Ktedonobacterales | f__JG30-KF-AS9 | g__JG30-KF-AS9 | s__uncultured_bacterium |
| 10292174 | Bacteria | p__Bacteroidota | c__Bacteroidia | o__Sphingobacteriales | f__Sphingobacteriaceae | g__Sphingobacterium | s__Sphingobacterium_corticis |
| MJ006-2-barcode63-umi134737bins-ubs-4 | Bacteria | p__Firmicutes | c__Clostridia | o__Oscillospirales | f__Oscillospiraceae |  |  |
| df56278b699c24cf2e5f1dc9828642d3 | Bacteria | p__Acidobacteriota | c__Acidobacteriae | o__Acidobacteriales | f__Acidobacteriaceae_(Subgroup_1) |  |  |
| 5d6d66243053ad7b923b6d28f80aa47d | Bacteria | p__Proteobacteria | c__Gammaproteobacteria | o__Burkholderiales | f__SC-I-84 | g__SC-I-84 | s__uncultured_Nitrosomonadaceae |
| MT878401 | Bacteria | p__Proteobacteria | c__Alphaproteobacteria | o__Sphingomonadales | f__Sphingomonadaceae | g__Sphingomonas |  |
| 02408900 | Bacteria | p__Firmicutes | c__Clostridia | o__Lachnospirales | f__Lachnospiraceae | g__[Ruminococcus]_gnavus_group |  |
| MJ033-1-barcode26-umi20911bins-ubs-12 | Bacteria | p__Proteobacteria | c__Gammaproteobacteria | o__Enterobacterales | f__Yersiniaceae | g__Serratia |  |
| 6f047956c9219a21af0649b9f58b0356 | Bacteria | p__Proteobacteria | c__Gammaproteobacteria | o__Burkholderiales | f__Nitrosomonadaceae | g__Ellin6067 |  |
| d7bbf8d2dc7578439f1c8aa9f35d3c7e | Bacteria | p__Gemmatimonadota | c__Gemmatimonadetes | o__Gemmatimonadales | f__Gemmatimonadaceae |  |  |
| RS-GCF-014902045.1-NZ-JACOJS010000015.1 | Bacteria | p__Proteobacteria | c__Gammaproteobacteria | o__Enterobacterales | f__Enterobacteriaceae |  |  |
| 13677923 | Bacteria | p__Gemmatimonadota | c__Gemmatimonadetes | o__Gemmatimonadales | f__Gemmatimonadaceae |  |  |
| b31af2842ee279be8bd17b2fdc5578fb | Bacteria | p__Proteobacteria | c__Gammaproteobacteria | o__Pseudomonadales | f__Pseudomonadaceae | g__Pseudomonas |  |
| dab2f47a17fed1f57aea44ca8419d52b | Bacteria | p__Actinobacteriota | c__Actinobacteria | o__Propionibacteriales | f__Nocardioidaceae | g__Aeromicrobium |  |
| 211d70c5aec8bde496a39f4144beef02 | Bacteria | p__Actinobacteriota | c__Coriobacteriia | o__Coriobacteriales | f__Eggerthellaceae |  |  |
| 06b42244396eb116177c5510c878683c | Bacteria | p__Proteobacteria | c__Gammaproteobacteria | o__Pseudomonadales | f__Moraxellaceae | g__Perlucidibaca | s__uncultured_bacterium |
| 95b3d0a663f42ed5f541f90157f0bfb6 | Bacteria | p__Firmicutes | c__Bacilli | o__Paenibacillales | f__Paenibacillaceae | g__Ammoniphilus |  |
| 44a4fa059d4f7eb9d3ea03d7d763c9ba | Bacteria | p__Verrucomicrobiota | c__Verrucomicrobiae | o__Chthoniobacterales | f__Chthoniobacteraceae | g__Candidatus_Udaeobacter |  |
| 02304220 | Bacteria | p__Proteobacteria | c__Alphaproteobacteria | o__Azospirillales | f__uncultured | g__uncultured |  |
| 93096ea33f6803335dbbd382146e8551 | Bacteria | p__Proteobacteria | c__Gammaproteobacteria | o__Pseudomonadales | f__Pseudomonadaceae | g__Pseudomonas |  |
| fb39b43695a69a47a47376a60f6332b9 | Bacteria | p__Verrucomicrobiota | c__Verrucomicrobiae | o__Opitutales | f__Opitutaceae |  |  |
| 04952159 | Bacteria | p__Firmicutes | c__Bacilli | o__Paenibacillales | f__Paenibacillaceae | g__Paenibacillus | s__Paenibacillus_turicensis |
| 80827193b55a1047460e57f012064f83 | Bacteria | p__Proteobacteria | c__Gammaproteobacteria | o__Burkholderiales | f__SC-I-84 | g__SC-I-84 |  |
| 02412495 | Bacteria | p__Proteobacteria | c__Alphaproteobacteria | o__Acetobacterales | f__Acetobacteraceae | g__Acetobacter |  |
| 02247877 | Bacteria | p__Proteobacteria | c__Alphaproteobacteria | o__Rhizobiales | f__Beijerinckiaceae | g__Methylobacterium-Methylorubrum |  |
| 771350c603f4ef78fc257ec7ad3ce02b | Bacteria | p__Proteobacteria | c__Gammaproteobacteria | o__Burkholderiales | f__Nitrosomonadaceae | g__MND1 |  |
| 481c70969f93f1b6e0d4e3de9450d21b | Bacteria | p__Firmicutes | c__Bacilli | o__Staphylococcales | f__Staphylococcaceae | g__Staphylococcus |  |
| 1670671a65d41e342c730432031db190 | Bacteria | p__Proteobacteria | c__Gammaproteobacteria | o__Pseudomonadales | f__Moraxellaceae | g__Acinetobacter |  |
| 03232323 | Bacteria |  |  |  |  |  |  |
| 21762928 | Bacteria | p__Firmicutes | c__Clostridia | o__Peptostreptococcales-Tissierellales | f__Peptostreptococcales-Tissierellales | g__Ezakiella |  |
| 08792559 | Bacteria | p__Proteobacteria | c__Gammaproteobacteria | o__Xanthomonadales | f__Xanthomonadaceae | g__Lysobacter |  |
| be9a5b32eed7fff4f2f60c6b70953fbb | Bacteria | p__Actinobacteriota | c__Actinobacteria | o__Propionibacteriales | f__Nocardioidaceae |  |  |
| 7fe6b6cd8969dc70c3585474392355cc | Bacteria | p__Proteobacteria | c__Gammaproteobacteria | o__Enterobacterales | f__Enterobacteriaceae |  |  |
| 04996514 | Bacteria | p__Proteobacteria | c__Gammaproteobacteria | o__Xanthomonadales | f__Xanthomonadaceae | g__Luteimonas |  |
| MJ006-1-barcode38-umi177664bins-ubs-3 | Bacteria | p__Firmicutes | c__Clostridia | o__Oscillospirales | f__Ruminococcaceae | g__Incertae_Sedis |  |
| c4efff4402866b68990466d1e3d2d5dd | Bacteria | p__Acidobacteriota | c__Acidobacteriae | o__Bryobacterales | f__Bryobacteraceae | g__Bryobacter |  |
| RS-GCF-001064485.1-NZ-JVLW01000023.1 | Bacteria | p__Actinobacteriota | c__Actinobacteria | o__Micrococcales | f__Micrococcaceae | g__Rothia |  |
| a79476b1e51fccae2a7686778427113c | Bacteria | p__Bacteroidota | c__Bacteroidia | o__Bacteroidales |  |  |  |
| 0764e1b41929dd8b3d2a2a94f96a5a4d | Bacteria | p__Bacteroidota | c__Bacteroidia | o__Bacteroidales | f__Rikenellaceae | g__Rikenellaceae_RC9_gut_group |  |
| MJ033-1-barcode34-umi1377bins-ubs-220 | Bacteria | p__Bdellovibrionota | c__Oligoflexia | o__0319-6G20 | f__0319-6G20 | g__0319-6G20 | s__uncultured_Roseobacter |
| 02307268 | Bacteria | p__Acidobacteriota | c__Acidobacteriae | o__Bryobacterales | f__Bryobacteraceae | g__Bryobacter |  |
| 20579750 | Bacteria | p__Firmicutes | c__Clostridia | o__Oscillospirales | f__Oscillospiraceae | g__Oscillibacter | s__uncultured_bacterium |
| 05035973 | Bacteria | p__Firmicutes | c__Clostridia | o__Oscillospirales | f__Oscillospiraceae | g__uncultured |  |
| d06f6e639901c5c79464533ff5a2d0f0 | Bacteria |  |  |  |  |  |  |
| dc04e8ca620896bc476a4b45d9b3f1e5 | Bacteria | p__Bacteroidota | c__Bacteroidia | o__Flavobacteriales | f__Flavobacteriaceae | g__Myroides | s__Myroides_odoratus |
| 822956f16515a1a94fb19e241fc21402 | Bacteria | p__Proteobacteria | c__Alphaproteobacteria | o__Caulobacterales | f__Hyphomonadaceae | g__SWB02 |  |
| 04961025 | Bacteria | p__Actinobacteriota | c__Actinobacteria | o__Corynebacteriales | f__Mycobacteriaceae | g__Mycobacterium |  |
| RS-GCF-014205685.1-NZ-JACIIU010000080.1 | Bacteria | p__Proteobacteria | c__Alphaproteobacteria | o__Rhizobiales | f__Rhizobiaceae |  |  |
| 49bb51f9befe11c26581b0dfd907c219 | Bacteria | p__Proteobacteria | c__Alphaproteobacteria | o__Rhizobiales | f__Rhizobiaceae | g__Allorhizobium-Neorhizobium-Pararhizobium-Rhizobium | s__[Pseudomonas]_geniculata |
| 01776620 | Bacteria | p__Chloroflexi | c__Anaerolineae | o__Anaerolineales | f__Anaerolineaceae | g__uncultured |  |
| 1b01ce6c88e3cfff3a322d04f9287f64 | Bacteria | p__Actinobacteriota | c__Thermoleophilia | o__Solirubrobacterales | f__67-14 | g__67-14 |  |
| 84c601918ab721899275451cb96b20c2 | Bacteria | p__Verrucomicrobiota | c__Verrucomicrobiae | o__Chthoniobacterales | f__Xiphinematobacteraceae | g__Candidatus_Xiphinematobacter | s__uncultured_soil |
| MJ033-1-barcode49-umi173bins-ubs-636 | Bacteria | p__Bacteroidota | c__Bacteroidia | o__Flavobacteriales | f__Flavobacteriaceae | g__Flavobacterium |  |
| 36371bb788584c3c455b095e71fbf231 | Bacteria | p__Proteobacteria | c__Gammaproteobacteria | o__Pseudomonadales | f__Moraxellaceae | g__Alkanindiges |  |
| 673b31a26ff785d7b7cc26012124e392 | Bacteria | p__Proteobacteria | c__Gammaproteobacteria | o__Burkholderiales | f__SC-I-84 | g__SC-I-84 |  |
| 40302a74fe9c7ce998acdf345d7ae58e | Bacteria | p__Proteobacteria | c__Gammaproteobacteria | o__Enterobacterales | f__Enterobacteriaceae |  |  |
| 04908522 | Bacteria | p__Proteobacteria | c__Alphaproteobacteria | o__Rhizobiales | f__Beijerinckiaceae | g__Microvirga |  |
| 2e6e7c9ed7235a2ce6baccd6ad9a74af | Bacteria | p__Acidobacteriota | c__Acidobacteriae | o__Bryobacterales | f__Bryobacteraceae | g__Bryobacter |  |
| 306da5568bed53378ba141a5b764dd13 | Bacteria | p__Chloroflexi | c__OLB14 | o__OLB14 | f__OLB14 | g__OLB14 |  |
| 11142160 | Bacteria | p__Bacteroidota | c__Bacteroidia | o__Bacteroidales | f__Muribaculaceae | g__Muribaculaceae |  |
| RS-GCF-000273275.1-NZ-JH724268.1--2 | Bacteria | p__Bacteroidota | c__Bacteroidia | o__Bacteroidales | f__Bacteroidaceae | g__Bacteroides |  |
| 1a51c430c7e5516fe495388f2fbe60ce | Bacteria | p__Bacteroidota | c__Bacteroidia | o__Bacteroidales | f__Bacteroidetes_vadinHA17 | g__Bacteroidetes_vadinHA17 |  |
| 55dc484daa3921cbfd9324da18746ce6 | Bacteria | p__Proteobacteria | c__Gammaproteobacteria | o__Enterobacterales | f__Morganellaceae | g__Morganella |  |
| MJ030-2-barcode58-umi114810bins-ubs-4 | Bacteria | p__Proteobacteria | c__Gammaproteobacteria | o__Burkholderiales | f__Comamonadaceae |  |  |
| fe1dfffecad2c0748aa0644ef979412c | Bacteria | p__Verrucomicrobiota | c__Verrucomicrobiae | o__Chthoniobacterales | f__Chthoniobacteraceae | g__Candidatus_Udaeobacter |  |
| 02694590 | Bacteria | p__Patescibacteria | c__Saccharimonadia | o__Saccharimonadales | f__Saccharimonadales | g__Saccharimonadales |  |
| ad34942b99d0c40cf10e0a100e9c796f | Bacteria | p__Proteobacteria | c__Gammaproteobacteria | o__Burkholderiales | f__TRA3-20 | g__TRA3-20 | s__uncultured_Nitrosomonadaceae |
| 02244819 | Bacteria | p__Actinobacteriota | c__Actinobacteria | o__Corynebacteriales | f__Corynebacteriaceae | g__Corynebacterium |  |
| KJ634465 | Bacteria | p__Bacteroidota | c__Bacteroidia | o__Chitinophagales | f__Chitinophagaceae | g__Parasegetibacter | s__Parasegetibacter_terrae |
| c41e2f4d0347588f1e615a2a5a0e14bd | Bacteria | p__Proteobacteria | c__Gammaproteobacteria | o__Pseudomonadales | f__Moraxellaceae | g__Acinetobacter |  |
| ba44dc82568d182731c4745e48480df0 | Bacteria | p__Actinobacteriota | c__Thermoleophilia | o__Solirubrobacterales | f__67-14 | g__67-14 |  |
| 8d8b71e687c17d461cd0e56c967e66a1 | Bacteria | p__Firmicutes | c__Clostridia | o__Peptostreptococcales-Tissierellales | f__Peptostreptococcales-Tissierellales | g__Peptoniphilus |  |
| 90e0b3442127bd256a1068cd7d4c7258 | Bacteria | p__Proteobacteria | c__Gammaproteobacteria | o__Pseudomonadales | f__Moraxellaceae | g__Acinetobacter |  |
| fea7cabed21751646730a7670c01a935 | Bacteria | p__Firmicutes | c__Bacilli | o__Staphylococcales | f__Staphylococcaceae | g__Staphylococcus |  |
| MJ010-2-barcode64-umi63028bins-ubs-8 | Bacteria | p__Bacteroidota | c__Bacteroidia | o__Flavobacteriales | f__Weeksellaceae | g__Chryseobacterium |  |
| 687b03d24f8125d9181a0f410960ff10 | Bacteria | p__Firmicutes | c__Symbiobacteriia | o__Symbiobacteriales | f__Symbiobacteraceae | g__Symbiobacterium | s__uncultured_bacterium |
| RS-GCF-013317225.1-NZ-JAALXU010000016.1 | Bacteria | p__Proteobacteria | c__Alphaproteobacteria | o__Rhizobiales | f__Rhizobiaceae |  |  |
| 5cc46ce28c716dd3cfd74137b48f1d20 | Bacteria | p__Patescibacteria | c__Parcubacteria | o__Candidatus_Moranbacteria | f__Candidatus_Moranbacteria | g__Candidatus_Moranbacteria | s__uncultured_bacterium |
| b83fadaa702d7da2b3baf84851209c65 | Bacteria | p__Proteobacteria | c__Gammaproteobacteria | o__Burkholderiales | f__SC-I-84 | g__SC-I-84 |  |
| f99dd29c5e18dccdc79b169405f81c4e | Bacteria | p__Proteobacteria | c__Alphaproteobacteria | o__Rhizobiales | f__Hyphomicrobiaceae |  |  |
| 04543872 | Bacteria | p__Firmicutes | c__Clostridia | o__Peptostreptococcales-Tissierellales | f__Peptostreptococcaceae | g__Romboutsia |  |
| MJ010-1-barcode30-umi161966bins-ubs-3 | Bacteria | p__Bacteroidota | c__Bacteroidia | o__Bacteroidales | f__Bacteroidaceae | g__Bacteroides | s__Bacteroides_sartorii |
| 04911115 | Bacteria | p__Proteobacteria | c__Gammaproteobacteria | o__Xanthomonadales | f__Xanthomonadaceae | g__Luteimonas |  |
| RS-GCF-002847425.1-NZ-CP025534.1--4 | Bacteria | p__Actinobacteriota | c__Actinobacteria | o__Corynebacteriales | f__Corynebacteriaceae | g__Corynebacterium |  |
| MJ010-1-barcode26-umi110311bins-ubs-5 | Bacteria | p__Proteobacteria | c__Gammaproteobacteria | o__Burkholderiales | f__Oxalobacteraceae |  |  |
| RS-GCF-000953675.1-NZ-LN679998.1 | Bacteria | p__Firmicutes | c__Clostridia | o__Peptostreptococcales-Tissierellales | f__Peptostreptococcaceae |  |  |
| b39f65db963a2ad7dd66bcef3cc9bfa2 | Bacteria | p__Firmicutes | c__Clostridia | o__Lachnospirales | f__Lachnospiraceae | g__Blautia |  |
| 32de701e09261e7c3e9e6f372d002b45 | Bacteria | p__Chloroflexi | c__Anaerolineae | o__SBR1031 | f__A4b | g__A4b |  |
| 02244716 | Bacteria | p__Actinobacteriota | c__Actinobacteria | o__Corynebacteriales | f__Corynebacteriaceae | g__Corynebacterium |  |
| 04898171 | Bacteria | p__Actinobacteriota | c__Actinobacteria | o__Micromonosporales | f__Micromonosporaceae | g__Micromonospora |  |
| 55dad6a960da36e84e3d38f78501d8a4 | Bacteria | p__Proteobacteria | c__Alphaproteobacteria | o__Rhizobiales | f__Rhizobiales_Incertae_Sedis | g__uncultured |  |
| 7761590663c052bf7a3724fd29d63229 | Bacteria | p__Acidobacteriota | c__Blastocatellia | o__Pyrinomonadales | f__Pyrinomonadaceae | g__RB41 |  |
| RS-GCF-014050425.1-NZ-JACGAM010000009.1 | Bacteria | p__Firmicutes | c__Bacilli | o__Lactobacillales | f__Aerococcaceae | g__Aerococcus |  |
| 04966032 | Bacteria | p__Firmicutes | c__Bacilli | o__Bacillales | f__Bacillaceae |  |  |
| 22794cdc7e84f95a2f43500ef9cd37e6 | Bacteria | p__Proteobacteria | c__Alphaproteobacteria | o__Rhizobiales | f__Hyphomicrobiaceae | g__Pedomicrobium |  |
| 4d9f606f5f735ee6763bb69799229d33 | Bacteria | p__Firmicutes | c__Bacilli | o__Lactobacillales | f__Enterococcaceae | g__Enterococcus |  |
| a8dfcb795c673f6737b4d9b11de64a51 | Bacteria | p__Proteobacteria | c__Gammaproteobacteria | o__Burkholderiales | f__Rhodocyclaceae |  |  |
| RS-GCF-006475605.1-NZ-SSBC01000007.1 | Bacteria | p__Proteobacteria | c__Alphaproteobacteria | o__Caulobacterales | f__Caulobacteraceae | g__Asticcacaulis |  |
| 4d9b19288f2da9e2dff1e7838b9cca81 | Bacteria | p__Firmicutes | c__Bacilli | o__Paenibacillales | f__Paenibacillaceae | g__Paenibacillus |  |
| b3281c3b7b4e8df5fe65df2fb1bbd228 | Bacteria | p__Proteobacteria | c__Gammaproteobacteria | o__Pseudomonadales | f__Moraxellaceae | g__Acinetobacter |  |
| MJ030-2-barcode58-umi60668bins-ubs-10 | Bacteria | p__Proteobacteria | c__Gammaproteobacteria | o__Burkholderiales | f__Comamonadaceae | g__Hydrogenophaga |  |
| 5306b8f380b9b73dfde274e16b0cfe03 | Bacteria | p__Firmicutes | c__Bacilli | o__Staphylococcales | f__Staphylococcaceae | g__Jeotgalicoccus |  |
| 04964483 | Bacteria | p__Firmicutes | c__Clostridia | o__Oscillospirales | f__Oscillospiraceae | g__Intestinimonas |  |
| 8e088e98068031ffb953076b8002b44b | Bacteria | p__Bacteroidota | c__Bacteroidia | o__Flavobacteriales | f__Flavobacteriaceae | g__Flavobacterium |  |
| 5c5d17c78261d7389bf475f7056c44ef | Bacteria | p__Firmicutes | c__Bacilli | o__Lactobacillales | f__Enterococcaceae | g__Enterococcus |  |
| 2bf3bba8ad0151afac5dd05c334a3945 | Bacteria | p__Proteobacteria | c__Alphaproteobacteria | o__Rhizobiales | f__Rhizobiaceae |  |  |
| 9727316b11c8a217d072176cf15407d2 | Bacteria | p__Firmicutes | c__Bacilli | o__Lactobacillales | f__Enterococcaceae | g__Enterococcus |  |
| 9ff295ffe195a4d767aa77b25bddc925 | Bacteria | p__Proteobacteria | c__Gammaproteobacteria | o__Burkholderiales | f__Burkholderiaceae | g__Burkholderia-Caballeronia-Paraburkholderia |  |
| RS-GCF-006546885.1-NZ-RYDQ01000063.1 | Bacteria | p__Firmicutes | c__Bacilli | o__Staphylococcales | f__Staphylococcaceae | g__Staphylococcus |  |
| 9110db87fb69e5491ffda70747446a17 | Bacteria | p__Firmicutes | c__Bacilli | o__Lactobacillales | f__Carnobacteriaceae | g__Atopostipes |  |
| 05026746 | Bacteria | p__Actinobacteriota | c__Thermoleophilia | o__Gaiellales | f__Gaiellaceae | g__Gaiella | s__actinobacterium_WWH12 |
| 04996493 | Bacteria | p__Proteobacteria | c__Gammaproteobacteria | o__Xanthomonadales | f__Xanthomonadaceae | g__Lysobacter |  |
| RS-GCF-000309115.1-NZ-AMFV01000001.1 | Bacteria | p__Proteobacteria | c__Gammaproteobacteria | o__Pseudomonadales | f__Moraxellaceae | g__Acinetobacter |  |
| GB-GCA-910574715.1-CAJTCH010000056.1 | Bacteria | p__Firmicutes | c__Clostridia | o__Lachnospirales | f__Lachnospiraceae | g__Lachnoclostridium |  |
| cc5dbf3f411b710551c1584924115c38 | Bacteria | p__Acidobacteriota | c__Blastocatellia | o__Blastocatellales | f__Blastocatellaceae |  |  |
| f8e15d4b6830508144566837517b2952 | Bacteria | p__Actinobacteriota | c__Acidimicrobiia | o__IMCC26256 | f__IMCC26256 | g__IMCC26256 | s__uncultured_Acidimicrobiia |
| 68d69ddd412fd6e106400c1e1dda688f | Bacteria | p__Proteobacteria | c__Gammaproteobacteria | o__Burkholderiales | f__Burkholderiaceae |  |  |
| GB-GCA-905197185.1-CAJJXW010000014.1 | Bacteria | p__Firmicutes | c__Negativicutes | o__Veillonellales-Selenomonadales | f__Selenomonadaceae | g__Megamonas | s__Megamonas_funiformis |
| a2c67c8ca231038c74b79e1b1d7fad7f | Bacteria | p__Actinobacteriota | c__Actinobacteria | o__Micrococcales | f__Microbacteriaceae |  |  |
| f47930885a2611109a24768d03a60c71 | Bacteria | p__Bdellovibrionota | c__Bdellovibrionia | o__Bdellovibrionales | f__Bdellovibrionaceae | g__Bdellovibrio | s__Bdellovibrio_sp. |
| 076cf9062c822a0f7889e0c155f99296 | Bacteria | p__Proteobacteria | c__Gammaproteobacteria | o__Xanthomonadales | f__Xanthomonadaceae | g__Thermomonas |  |
| a18014f584da6cff1c7c7aea27693347 | Bacteria | p__Proteobacteria | c__Gammaproteobacteria | o__Xanthomonadales | f__Xanthomonadaceae | g__Stenotrophomonas |  |
| 05032571 | Bacteria | p__Actinobacteriota | c__Actinobacteria | o__Micrococcales | f__Microbacteriaceae |  |  |
| G900184225_1 | Bacteria | p__Actinobacteriota | c__Actinobacteria | o__Micrococcales | f__Brevibacteriaceae | g__Brevibacterium |  |
| f1f313816aed9e91a30f6082b8ce5c00 | Bacteria | p__Actinobacteriota | c__Thermoleophilia | o__Solirubrobacterales | f__Solirubrobacteraceae | g__Patulibacter |  |
| 17f55000c180794e480cfa158d2453f9 | Bacteria |  |  |  |  |  |  |
| GQ406812 | Bacteria | p__Actinobacteriota | c__Actinobacteria | o__Micrococcales | f__Micrococcaceae |  |  |
| 01255354 | Bacteria | p__Firmicutes | c__Clostridia | o__Lachnospirales | f__Lachnospiraceae |  |  |
| RS-GCF-902167775.1-NZ-CABHND010000018.1 | Bacteria | p__Actinobacteriota | c__Actinobacteria | o__Bifidobacteriales | f__Bifidobacteriaceae | g__Bifidobacterium | s__Bifidobacterium_longum |
| 9fc33ed588f06222d9d71f3056457986 | Bacteria | p__Fibrobacterota | c__Fibrobacteria | o__Fibrobacterales | f__Fibrobacterales | g__BBMC-4 | s__uncultured_bacterium |
| 048bdcd57ef6d5f0936f38d22017aa04 | Bacteria | p__Proteobacteria | c__Gammaproteobacteria | o__Pseudomonadales | f__Moraxellaceae | g__Acinetobacter |  |
| MJ006-1-barcode43-umi205212bins-ubs-3 | Bacteria | p__Firmicutes | c__Clostridia | o__Monoglobales | f__Monoglobaceae | g__Monoglobus |  |
| 32c12e31bba7e5aea44ce8efc6f62606 | Bacteria | p__Actinobacteriota | c__Actinobacteria | o__Bifidobacteriales | f__Bifidobacteriaceae | g__Bifidobacterium |  |
| 8b14631097245e970869498261bf2f8c | Bacteria | p__Proteobacteria | c__Alphaproteobacteria | o__Sphingomonadales | f__Sphingomonadaceae | g__Sphingomonas |  |
| RS-GCF-002930535.1-NZ-PJDL01000022.1 | Bacteria | p__Proteobacteria | c__Gammaproteobacteria | o__Enterobacterales | f__Pectobacteriaceae |  |  |
| 1157888ddfdb4048fab87379ac61c841 | Bacteria | p__Proteobacteria | c__Gammaproteobacteria | o__Burkholderiales | f__Comamonadaceae | g__Comamonas |  |
| 2ac383f63fc5a7ae487f832bddb618ee | Bacteria | p__Gemmatimonadota | c__Gemmatimonadetes | o__Gemmatimonadales | f__Gemmatimonadaceae | g__uncultured |  |
| 543d3ee867f983f954f2a263c1ad2bca | Bacteria | p__Proteobacteria | c__Gammaproteobacteria | o__Pseudomonadales | f__Moraxellaceae | g__Acinetobacter |  |
| 63d3151bb2c81448b53df5bd93dae428 | Bacteria | p__Proteobacteria | c__Alphaproteobacteria | o__Rhodobacterales | f__Rhodobacteraceae |  |  |
| 5367d1295bf4a4a3ebfc030ce8167419 | Bacteria | p__Verrucomicrobiota | c__Verrucomicrobiae | o__Chthoniobacterales | f__Chthoniobacteraceae | g__Chthoniobacter |  |
| a36ca97b257eb7529dcd14c555fd5512 | Bacteria | p__Chloroflexi | c__TK10 | o__TK10 | f__TK10 | g__TK10 |  |
| 04958519 | Bacteria | p__Actinobacteriota | c__Actinobacteria | o__Kineosporiales | f__Kineosporiaceae | g__Angustibacter |  |
| MJ006-1-barcode39-umi142106bins-ubs-3 | Bacteria | p__Firmicutes | c__Clostridia | o__Oscillospirales | f__Ruminococcaceae | g__Faecalibacterium |  |
| a630e38bf55c21dcfc40da5060505a80 | Bacteria | p__Proteobacteria | c__Gammaproteobacteria | o__Pseudomonadales | f__Moraxellaceae | g__Acinetobacter |  |
| 04900486 | Bacteria | p__Proteobacteria | c__Gammaproteobacteria | o__Gammaproteobacteria_Incertae_Sedis | f__Unknown_Family | g__Acidibacter |  |
| d49e237ad9c91e08770354dd565e27f8 | Bacteria | p__Actinobacteriota | c__Actinobacteria | o__Micrococcales | f__Microbacteriaceae | g__Microbacterium |  |
| 19712483 | Bacteria | p__Firmicutes | c__Bacilli | o__Erysipelotrichales | f__Erysipelotrichaceae | g__Ileibacterium | s__Ileibacterium_valens |
| KM588957 | Bacteria | p__Proteobacteria | c__Alphaproteobacteria | o__Rhizobiales | f__Beijerinckiaceae | g__Microvirga |  |
| X81996 | Bacteria | p__Actinobacteriota | c__Actinobacteria | o__Corynebacteriales | f__Mycobacteriaceae | g__Mycobacterium |  |
| 5a7a828ad9a45f080ed5fcfa8c26a603 | Bacteria | p__Actinobacteriota | c__Thermoleophilia | o__Gaiellales |  |  |  |
| 83470d2979ec04164d1789a8a5ab4cf0 | Bacteria | p__Verrucomicrobiota | c__Verrucomicrobiae | o__Chthoniobacterales | f__Chthoniobacteraceae | g__Candidatus_Udaeobacter |  |
| 04930867 | Bacteria | p__Proteobacteria | c__Gammaproteobacteria | o__Aeromonadales | f__Aeromonadaceae | g__Aeromonas |  |
| 3ea6d1742664cbe5f24f788d53a79dba | Bacteria | p__Myxococcota | c__Polyangia | o__Polyangiales | f__Phaselicystidaceae | g__Phaselicystis | s__uncultured_Byssovorax |
| RS-GCF-900115705.1-NZ-FOXL01000003.1 | Bacteria | p__Proteobacteria | c__Gammaproteobacteria | o__Xanthomonadales | f__Rhodanobacteraceae |  |  |
| d18b27b470ba60431f204b605fb83d8b | Bacteria | p__Bacteroidota | c__Bacteroidia | o__Bacteroidales | f__Muribaculaceae | g__Muribaculaceae | s__uncultured_bacterium |
| e367e6dcc1e0cfa2360c59b1526f0167 | Bacteria | p__Firmicutes | c__Bacilli | o__Lactobacillales | f__Enterococcaceae | g__Enterococcus |  |
| f7bc3af4b97271272117ee4914887f32 | Bacteria | p__Proteobacteria | c__Gammaproteobacteria |  |  |  |  |
| MK601671 | Bacteria | p__Bacteroidota | c__Bacteroidia | o__Sphingobacteriales | f__Sphingobacteriaceae | g__Sphingobacterium |  |
| 14836032 | Bacteria | p__Chloroflexi | c__Ktedonobacteria | o__C0119 | f__C0119 | g__C0119 | s__uncultured_bacterium |
| b857fd016c53c78732c41a01c37627ff | Bacteria | p__Chloroflexi | c__Ktedonobacteria | o__Ktedonobacterales | f__Ktedonobacteraceae |  |  |
| 52b0e622bedc7ea3b89eb92568456f65 | Bacteria |  |  |  |  |  |  |
| f3d18531f2c461d13b728dde11e0eee7 | Bacteria | p__Firmicutes | c__Bacilli | o__Erysipelotrichales | f__Erysipelotrichaceae | g__Turicibacter | s__uncultured_bacterium |
| MJ033-1-barcode47-umi29398bins-ubs-7 | Bacteria | p__Bacteroidota | c__Bacteroidia | o__Sphingobacteriales | f__Sphingobacteriaceae | g__Solitalea | s__Solitalea_koreensis |
| 04961576 | Bacteria | p__Actinobacteriota | c__Actinobacteria | o__Pseudonocardiales | f__Pseudonocardiaceae |  |  |
| e4bcb2a71a6e92b90626faa3a739ef75 | Bacteria | p__Acidobacteriota | c__Acidobacteriae | o__Bryobacterales | f__Bryobacteraceae | g__Bryobacter |  |
| d09e8bb24d3f380b1ea39241473ceaa0 | Bacteria | p__Actinobacteriota | c__Actinobacteria | o__Streptosporangiales | f__Nocardiopsaceae | g__Nocardiopsis |  |
| ce78818f136eff61d485cfd88f47d228 | Bacteria | p__Methylomirabilota | c__Methylomirabilia | o__Rokubacteriales | f__Rokubacteriales | g__Rokubacteriales |  |
| EU427464 | Bacteria | p__Deinococcota | c__Deinococci | o__Deinococcales | f__Deinococcaceae | g__Deinococcus |  |
| 411211e414686300181844b48405ee13 | Bacteria | p__Proteobacteria | c__Gammaproteobacteria | o__Steroidobacterales | f__Steroidobacteraceae | g__uncultured |  |
| 3d84f1b43e8e03f0e10b8faa0034473e | Bacteria | p__Proteobacteria | c__Gammaproteobacteria | o__Xanthomonadales | f__Xanthomonadaceae | g__Xanthomonas |  |
| AB175644 | Bacteria | p__Proteobacteria | c__Alphaproteobacteria | o__Rhizobiales | f__Beijerinckiaceae | g__Methylobacterium-Methylorubrum |  |
| 54bc62bc359d37e6a90258ce527e1d6d | Bacteria | p__Proteobacteria | c__Gammaproteobacteria | o__Burkholderiales | f__Comamonadaceae |  |  |
| 229dde29448c276f07e251642a03801a | Bacteria | p__Actinobacteriota | c__Acidimicrobiia | o__Microtrichales | f__Ilumatobacteraceae | g__CL500-29_marine_group |  |
| 6634a0156c9edf8abae77f9aa0f8126b | Bacteria | p__Patescibacteria | c__Saccharimonadia | o__Saccharimonadales | f__Saccharimonadales | g__Saccharimonadales | s__metagenome |
| 04926923 | Bacteria | p__Armatimonadota | c__Chthonomonadetes | o__Chthonomonadales | f__Chthonomonadaceae | g__Chthonomonas | s__uncultured_Armatimonadetes |
| 04908938 | Bacteria | p__Proteobacteria | c__Alphaproteobacteria | o__Rhizobiales | f__Rhizobiaceae | g__Allorhizobium-Neorhizobium-Pararhizobium-Rhizobium |  |
| GQ504012 | Bacteria | p__Actinobacteriota | c__Actinobacteria | o__Micrococcales | f__Microbacteriaceae | g__Leucobacter |  |
| 163f9c6b5d1e71c5d843826a8fec0edf | Bacteria | p__Actinobacteriota | c__Actinobacteria |  |  |  |  |
| c84569b0be89447b0e64cf9219b17ed5 | Bacteria | p__Acidobacteriota | c__Acidobacteriae | o__Subgroup_2 | f__Subgroup_2 | g__Subgroup_2 | s__uncultured_bacterium |
| d809a07f12300a61eca455d5cdf39f72 | Bacteria | p__Actinobacteriota | c__Actinobacteria | o__Frankiales | f__Acidothermaceae | g__Acidothermus |  |
| 02859902 | Bacteria | p__Chloroflexi | c__Chloroflexia | o__Chloroflexales | f__Roseiflexaceae | g__uncultured |  |
| ca62baf1b85a4020263155d8651d165a | Bacteria | p__Actinobacteriota | c__Actinobacteria | o__Micrococcales | f__Microbacteriaceae | g__Microbacterium |  |
| KC835077 | Bacteria | p__Firmicutes | c__Bacilli | o__Paenibacillales | f__Paenibacillaceae | g__Paenibacillus |  |
| KT287072 | Bacteria | p__Acidobacteriota | c__Vicinamibacteria | o__Vicinamibacterales | f__Vicinamibacteraceae | g__Luteitalea |  |
| 53efef7371830bfa3a4bf20cfdfbc376 | Bacteria | p__Actinobacteriota | c__Actinobacteria | o__Micrococcales |  |  |  |
| 05863580 | Bacteria | p__Planctomycetota | c__Phycisphaerae | o__Tepidisphaerales | f__WD2101_soil_group | g__WD2101_soil_group | s__uncultured_eubacterium |
| 2381baabefaac2418649672942fe519e | Bacteria | p__Proteobacteria | c__Gammaproteobacteria | o__Cellvibrionales | f__Cellvibrionaceae | g__Cellvibrio | s__Cellvibrio_fontiphilus |
| 198e2f93ef4482c99a1789ef6fd33cec | Bacteria | p__Proteobacteria | c__Alphaproteobacteria | o__Caulobacterales | f__Hyphomonadaceae | g__SWB02 |  |
| FN665659 | Bacteria | p__Bacteroidota | c__Bacteroidia | o__Chitinophagales | f__Chitinophagaceae | g__Hydrotalea | s__uncultured_bacterium |
| 10292717 | Bacteria | p__Bacteroidota | c__Bacteroidia | o__Sphingobacteriales | f__Sphingobacteriaceae | g__Sphingobacterium | s__Sphingobacterium_populi |
| 04906685 | Bacteria | p__Proteobacteria | c__Alphaproteobacteria | o__Rhizobiales | f__Beijerinckiaceae | g__Methylobacterium-Methylorubrum | s__Methylobacterium_aerolatum |
| 5d93753e6e5b5335ac95c386dc847ac4 | Bacteria | p__Acidobacteriota | c__Acidobacteriae | o__Acidobacteriales | f__Acidobacteriaceae_(Subgroup_1) |  |  |
| 4dff68fa3a665c29e75882cf5fca290c | Bacteria | p__Actinobacteriota | c__Actinobacteria |  |  |  |  |
| 6f2fa6143c628fdf1301e79b2b72021e | Bacteria | p__Bacteroidota | c__Bacteroidia | o__Chitinophagales | f__Chitinophagaceae |  |  |
| G000800475_1 | Bacteria | p__Actinobacteriota | c__Actinobacteria | o__Bifidobacteriales | f__Bifidobacteriaceae | g__Bifidobacterium |  |
| 13cf7d1cc6049b747c2c7f14cb9a0835 | Bacteria | p__Actinobacteriota | c__Actinobacteria | o__Frankiales | f__Sporichthyaceae | g__Longivirga |  |
| 04879807 | Bacteria | p__Proteobacteria | c__Gammaproteobacteria | o__Pseudomonadales | f__Pseudomonadaceae | g__Pseudomonas |  |
| 1eca69d2a6c2f0f29d756885e5860a47 | Bacteria | p__Chloroflexi | c__Chloroflexia | o__Chloroflexales | f__Roseiflexaceae | g__uncultured | s__uncultured_bacterium |
| 8bd50ba2c4bfdd8bc9e085a8944aa87a | Bacteria | p__Proteobacteria | c__Gammaproteobacteria | o__Pseudomonadales | f__Pseudomonadaceae | g__Pseudomonas |  |
| 523f4cebcc56c451d06dc2219a2e9fa2 | Bacteria | p__Entotheonellaeota | c__Entotheonellia | o__Entotheonellales | f__Entotheonellaceae | g__Entotheonellaceae | s__uncultured_bacterium |
| 0ea042a7651060ac3f4fd6b639bfc16f | Bacteria | p__Actinobacteriota | c__Actinobacteria | o__Micrococcales | f__Microbacteriaceae |  |  |
| 42c25b2748693b6ec0fbea69f21e75c7 | Bacteria | p__Actinobacteriota | c__Actinobacteria | o__Corynebacteriales | f__Mycobacteriaceae | g__Mycobacterium |  |
| 22b4d07909c142c56b764a658289a734 | Bacteria | p__Actinobacteriota | c__Actinobacteria | o__Frankiales | f__Frankiaceae | g__Jatrophihabitans |  |
| G000488255_5 | Bacteria | p__Proteobacteria | c__Gammaproteobacteria | o__Pseudomonadales | f__Moraxellaceae | g__Acinetobacter | s__Acinetobacter_indicus |
| RS-GCF-019130915.1-NZ-JAHOLF010000125.1 | Bacteria | p__Bacteroidota | c__Bacteroidia | o__Bacteroidales | f__Bacteroidaceae | g__Bacteroides | s__Bacteroides_vulgatus |
| cfb2df09cc3bac3d2e5214f8cefd62f2 | Bacteria | p__Acidobacteriota | c__Acidobacteriae | o__Subgroup_2 | f__Subgroup_2 | g__Subgroup_2 |  |
| G001044245_1 | Bacteria | p__Actinobacteriota | c__Actinobacteria | o__Corynebacteriales | f__Mycobacteriaceae | g__Mycobacterium |  |
| D83374 | Bacteria | p__Firmicutes | c__Bacilli | o__Staphylococcales | f__Staphylococcaceae | g__Staphylococcus |  |
| 02416873 | Bacteria | p__Proteobacteria | c__Alphaproteobacteria | o__Rhizobiales | f__Hyphomicrobiaceae | g__Hyphomicrobium |  |
| 02247845 | Bacteria | p__Proteobacteria | c__Alphaproteobacteria | o__Rhizobiales | f__Rhizobiaceae |  |  |
| MJ006-1-barcode40-umi140330bins-ubs-3 | Bacteria | p__Firmicutes | c__Clostridia | o__Oscillospirales | f__Butyricicoccaceae | g__Butyricicoccus |  |
| 04967578 | Bacteria | p__Firmicutes | c__Clostridia | o__Oscillospirales | f__Oscillospiraceae |  |  |
| 5359a8611d57c83bcf6102f57a433a38 | Bacteria | p__Actinobacteriota | c__Actinobacteria | o__Micrococcales | f__Micrococcaceae |  |  |
| ae2c230b8511f1be8d0410d028ed277a | Bacteria | p__Proteobacteria | c__Gammaproteobacteria | o__Pseudomonadales | f__Moraxellaceae | g__Acinetobacter |  |
| 8e929a5845310eda565f1bcf938458dc | Bacteria | p__Planctomycetota | c__Planctomycetes | o__Gemmatales | f__Gemmataceae | g__uncultured |  |
| 5e165b262388425c1ac136548c1b0555 | Bacteria | p__Actinobacteriota | c__Actinobacteria | o__Micrococcales | f__Intrasporangiaceae |  |  |
| 42b79c099a3c90a8df178c3911e0e025 | Bacteria | p__Firmicutes | c__Bacilli | o__Lactobacillales |  |  |  |
| EF685359 | Bacteria | p__Bacteroidota | c__Bacteroidia | o__Flavobacteriales | f__Weeksellaceae | g__Chryseobacterium | s__Chryseobacterium_hungaricum |
| 6503690bdfe6d586a9ea4d4d6beb47d7 | Bacteria | p__Chloroflexi | c__Anaerolineae | o__Caldilineales | f__Caldilineaceae | g__uncultured | s__metagenome |
| f28d7e637d23dfbbe243bbfa4fc3b464 | Bacteria | p__Actinobacteriota | c__Acidimicrobiia | o__Microtrichales | f__Ilumatobacteraceae | g__CL500-29_marine_group |  |
| 4a562b723cdcdbd8e96c08d2c95c9e65 | Bacteria | p__Proteobacteria | c__Gammaproteobacteria | o__Burkholderiales | f__Neisseriaceae | g__uncultured | s__uncultured_bacterium |
| 04952611 | Bacteria | p__Actinobacteriota | c__Thermoleophilia | o__Gaiellales |  |  |  |
| 508f4c21e3a44ee96e0d6933d95666e6 | Bacteria | p__Proteobacteria | c__Gammaproteobacteria | o__Burkholderiales | f__Methylophilaceae | g__Methylophilus | s__uncultured_Methylophilus |
| c96d6d527475e624dedb945eafb6a0da | Bacteria | p__Firmicutes | c__Bacilli | o__Erysipelotrichales | f__Erysipelotrichaceae | g__Dubosiella |  |
| d323b9f680a80e76b67acfc601c6af0f | Bacteria | p__Proteobacteria | c__Alphaproteobacteria | o__Rhizobiales | f__Xanthobacteraceae | g__uncultured |  |
| MJ034-1-barcode30-umi26679bins-ubs-4 | Bacteria | p__Acidobacteriota | c__Acidobacteriae | o__Solibacterales | f__Solibacteraceae | g__Candidatus_Solibacter |  |
| 175d6f1fc42b9c30a9521e1028c42e75 | Bacteria | p__Proteobacteria | c__Gammaproteobacteria | o__Pseudomonadales | f__Moraxellaceae | g__Acinetobacter |  |
| 14875407 | Bacteria | p__Chloroflexi | c__Ktedonobacteria | o__C0119 | f__C0119 | g__C0119 | s__uncultured_soil |
| 3cfd06f56e5f2fcdf966ac180512ad04 | Bacteria | p__Proteobacteria | c__Alphaproteobacteria | o__Rhizobiales | f__Devosiaceae | g__Devosia |  |
| 14895380 | Bacteria | p__Bacteroidota | c__Kapabacteria | o__Kapabacteriales | f__Kapabacteriales | g__Kapabacteriales | s__uncultured_bacterium |
| 04950100 | Bacteria | p__Firmicutes | c__Clostridia | o__Lachnospirales | f__Lachnospiraceae |  |  |
| eede9fcfe841071147aa5ccc007fc9fd | Bacteria | p__Acidobacteriota | c__Acidobacteriae | o__Acidobacteriales | f__Acidobacteriaceae_(Subgroup_1) |  |  |
| AB537169 | Bacteria | p__Actinobacteriota | c__Actinobacteria | o__Micrococcales | f__Dermabacteraceae | g__Brachybacterium |  |
| 9bd10508e6f653523a866c5468d9655e | Bacteria | p__Firmicutes | c__Bacilli | o__Lactobacillales | f__Carnobacteriaceae | g__Atopostipes |  |
| 8aac1a2b2cae6801d957643ceb208c68 | Bacteria | p__Actinobacteriota | c__Actinobacteria |  |  |  |  |
| 0c3690f2505a881c3cca5b919edcc235 | Bacteria | p__Actinobacteriota | c__Actinobacteria | o__Propionibacteriales | f__Nocardioidaceae | g__Kribbella |  |
| c9bb6c97e956cda75963fb6c7b92e9d4 | Bacteria | p__Proteobacteria | c__Alphaproteobacteria | o__Elsterales | f__uncultured | g__uncultured |  |
| G009774765_1 | Bacteria | p__Bacteroidota | c__Bacteroidia | o__Bacteroidales | f__Prevotellaceae | g__Alloprevotella |  |
| G000312385_1 | Bacteria | p__Actinobacteriota | c__Actinobacteria | o__Corynebacteriales | f__Tsukamurellaceae | g__Tsukamurella |  |
| 7249852d57f8d10fa3e321a8d9dfae10 | Bacteria | p__Proteobacteria | c__Alphaproteobacteria | o__Rhizobiales | f__Rhizobiaceae | g__Allorhizobium-Neorhizobium-Pararhizobium-Rhizobium |  |
| RS-GCF-015845595.1-NZ-LDHW01000035.1 | Bacteria | p__Firmicutes | c__Bacilli | o__Bacillales | f__Planococcaceae | g__Lysinibacillus |  |
| 04956645 | Bacteria | p__Actinobacteriota | c__Actinobacteria | o__Micrococcales | f__Microbacteriaceae | g__Agromyces |  |
| 02858596 | Bacteria | p__Chloroflexi | c__Chloroflexia | o__Chloroflexales | f__Roseiflexaceae | g__uncultured | s__uncultured_bacterium |
| 1b29c617ec5db6009dce6166df5aab36 | Bacteria | p__Proteobacteria | c__Gammaproteobacteria | o__Xanthomonadales | f__Rhodanobacteraceae | g__Dyella |  |
| 2941730405553381d8803d3ca7b2af35 | Bacteria | p__Proteobacteria | c__Alphaproteobacteria | o__Azospirillales | f__Azospirillaceae | g__Niveispirillum | s__Niveispirillum_irakense |
| KT988032 | Bacteria | p__Bacteroidota | c__Bacteroidia | o__Sphingobacteriales | f__Sphingobacteriaceae | g__Sphingobacterium | s__Sphingobacterium_populi |
| c0a4fab4406e0bd4348eb17c3b47caf2 | Bacteria | p__Actinobacteriota | c__Actinobacteria | o__Micrococcales | f__Microbacteriaceae | g__Microbacterium |  |
| 04909744 | Bacteria | p__Proteobacteria | c__Alphaproteobacteria | o__Rhizobiales | f__Devosiaceae | g__Devosia |  |
| b5f037e3102be0202f93385d2f87badf | Bacteria | p__Proteobacteria | c__Gammaproteobacteria | o__Enterobacterales | f__Enterobacteriaceae |  |  |
| 59876d4c327aa007d77653216fdd59af | Bacteria | p__Verrucomicrobiota | c__Verrucomicrobiae | o__Chthoniobacterales | f__Chthoniobacteraceae | g__Chthoniobacter | s__uncultured_bacterium |
| fccf9eaf0e942bc4a8fd35c659015090 | Bacteria | p__Proteobacteria | c__Gammaproteobacteria | o__Pseudomonadales | f__Moraxellaceae | g__Acinetobacter |  |
| 8b86af6e1189a4477bf87d89959fd3ee | Bacteria | p__Actinobacteriota | c__Acidimicrobiia | o__Microtrichales | f__Ilumatobacteraceae | g__CL500-29_marine_group |  |
| 443fdb932e8a7215da9f46a2cc14eefb | Bacteria | p__Proteobacteria | c__Gammaproteobacteria | o__Gammaproteobacteria_Incertae_Sedis | f__Unknown_Family | g__Acidibacter |  |
| LT719153 | Bacteria | p__Proteobacteria | c__Alphaproteobacteria | o__Rhizobiales | f__Rhizobiales_Incertae_Sedis | g__Phreatobacter |  |
| 04930783 | Bacteria | p__Proteobacteria | c__Gammaproteobacteria | o__Enterobacterales |  |  |  |
| 06acf3f5fadd098002b2c48b5e736f4a | Bacteria | p__Firmicutes | c__Bacilli | o__Bacillales |  |  |  |
| afbf978c8474949c9a9e76b005d55192 | Bacteria | p__Acidobacteriota | c__Acidobacteriae | o__Subgroup_2 | f__Subgroup_2 | g__Subgroup_2 |  |
| f422c68dc22e5cb72b228e664dfafd4f | Bacteria | p__Acidobacteriota | c__Acidobacteriae | o__Acidobacteriales | f__Acidobacteriaceae_(Subgroup_1) | g__uncultured | s__Acidobacteria_bacterium |
| 78b66b9f7193a24bdd604a2b94ad91f2 | Bacteria | p__Bacteroidota | c__Bacteroidia | o__Sphingobacteriales | f__Sphingobacteriaceae | g__Mucilaginibacter |  |
| d3d53f886cf840aa597657d89b694155 | Bacteria | p__Proteobacteria | c__Gammaproteobacteria | o__Pseudomonadales | f__Moraxellaceae | g__Acinetobacter | s__Ambystoma_mexicanum |
| 04906848 | Bacteria | p__Proteobacteria | c__Alphaproteobacteria | o__Rhizobiales | f__Xanthobacteraceae | g__Bradyrhizobium |  |
| 1727fa18f1abc89d256f2fc199c39717 | Bacteria | p__Proteobacteria | c__Gammaproteobacteria | o__Xanthomonadales | f__Xanthomonadaceae | g__Thermomonas |  |
| 954ad4c1ec3b376f48e4cd91fd352c59 | Bacteria | p__Proteobacteria | c__Alphaproteobacteria | o__Rhodobacterales | f__Rhodobacteraceae |  |  |
| 8112fe05566d1d02262b5a883736d9e6 | Bacteria | p__Actinobacteriota | c__Actinobacteria | o__Streptosporangiales | f__Streptosporangiaceae | g__Streptosporangium |  |
| da0b6b4915e012c67df1204e2b13d088 | Bacteria | p__Armatimonadota | c__Chthonomonadetes | o__Chthonomonadales | f__Chthonomonadaceae | g__Chthonomonas | s__uncultured_bacterium |
| 16540900 | Bacteria | p__Firmicutes | c__Clostridia | o__Lachnospirales | f__Lachnospiraceae |  |  |
| RS-GCF-004793835.1-NZ-SRYJ01000091.1 | Bacteria | p__Bacteroidota | c__Bacteroidia | o__Bacteroidales | f__Bacteroidaceae | g__Bacteroides | s__Bacteroides_sartorii |
| a073a8209dd530eac1c0eb708c156ea2 | Bacteria | p__Acidobacteriota | c__Acidobacteriae | o__Solibacterales | f__Solibacteraceae | g__Candidatus_Solibacter |  |
| 056e6d9e5552168e60bc3f41b3307f91 | Bacteria | p__Actinobacteriota | c__Coriobacteriia | o__Coriobacteriales | f__Eggerthellaceae |  |  |
| MJ030-1-barcode42-umi106758bins-ubs-3 | Bacteria | p__Proteobacteria | c__Gammaproteobacteria | o__Pseudomonadales | f__Pseudomonadaceae | g__Pseudomonas |  |
| b6aa5e8dba32a493feb3ad1cfb7074c7 | Bacteria | p__Bacteroidota | c__Bacteroidia | o__Cytophagales | f__Spirosomaceae | g__Persicitalea |  |
| MJ031-1-barcode34-umi34359bins-ubs-6 | Bacteria | p__Firmicutes | c__Bacilli | o__Bacillales | f__Bacillaceae | g__Bacillus |  |
| 05009078 | Bacteria | p__Proteobacteria | c__Alphaproteobacteria | o__Rhodobacterales | f__Rhodobacteraceae | g__Paracoccus |  |
| f77d7ca4e3e9bd9d487ecdeb7dc59105 | Bacteria | p__Proteobacteria | c__Alphaproteobacteria | o__Rhodobacterales | f__Rhodobacteraceae |  |  |
| 30ad6987bb22228e7dc2636f5928d351 | Bacteria | p__Bacteroidota | c__Bacteroidia | o__Chitinophagales | f__Saprospiraceae | g__uncultured |  |
| G001953215_1 | Bacteria | p__Proteobacteria | c__Gammaproteobacteria | o__Burkholderiales | f__Nitrosomonadaceae | g__Ellin6067 |  |
| RS-GCF-000620805.1-NZ-KK211187.1--3 | Bacteria | p__Firmicutes | c__Bacilli | o__Exiguobacterales | f__Exiguobacteraceae | g__Exiguobacterium |  |
| 02266134 | Bacteria | p__Actinobacteriota | c__Actinobacteria | o__Kineosporiales | f__Kineosporiaceae | g__Pseudokineococcus |  |
| 79df18242a342288825f6d5dc509f55a | Bacteria | p__Acidobacteriota | c__Vicinamibacteria | o__Vicinamibacterales | f__uncultured | g__uncultured | s__uncultured_Acidobacteriales |
| 0851c9e8323221b541e730a01dc0f364 | Bacteria | p__Chloroflexi | c__TK10 | o__TK10 | f__TK10 | g__TK10 | s__uncultured_bacterium |
| MJ006-1-barcode45-umi135865bins-ubs-3 | Bacteria | p__Firmicutes | c__Clostridia | o__Lachnospirales | f__Lachnospiraceae | g__Coprococcus |  |
| ba9c97e1d28a55740dd26688134dcd46 | Bacteria | p__Proteobacteria | c__Gammaproteobacteria | o__Enterobacterales | f__Enterobacteriaceae |  |  |
| 321ce92a9cf72e2bc87e3b797f50cb72 | Bacteria | p__Firmicutes | c__Clostridia | o__Clostridia_UCG-014 | f__Clostridia_UCG-014 | g__Clostridia_UCG-014 |  |
| 1618e95a6b41374eb2562aef0ca696c9 | Bacteria | p__Planctomycetota | c__Planctomycetes | o__Gemmatales | f__Gemmataceae | g__uncultured |  |
| 21f05c24d453f1f1d662d2d4028d35e0 | Bacteria | p__Proteobacteria | c__Gammaproteobacteria |  |  |  |  |
| 02360025 | Bacteria | p__Proteobacteria | c__Alphaproteobacteria | o__Sphingomonadales | f__Sphingomonadaceae | g__Altererythrobacter |  |
| 19976009 | Bacteria | p__Firmicutes | c__Clostridia | o__Oscillospirales | f__Oscillospiraceae | g__Colidextribacter |  |
| b909ec36257585377ed46e12d85460a4 | Bacteria | p__Proteobacteria | c__Alphaproteobacteria | o__Caulobacterales | f__Caulobacteraceae | g__uncultured |  |
| 365eea2634f0958d786d192256d19398 | Bacteria | p__Proteobacteria | c__Alphaproteobacteria | o__Rhizobiales | f__Rhizobiaceae | g__Allorhizobium-Neorhizobium-Pararhizobium-Rhizobium |  |
| MJ010-1-barcode47-umi21155bins-ubs-29 | Bacteria | p__Bacteroidota | c__Bacteroidia | o__Sphingobacteriales | f__Sphingobacteriaceae | g__Sphingobacterium |  |
| 01252311 | Bacteria | p__Bacteroidota | c__Bacteroidia | o__Bacteroidales | f__Muribaculaceae | g__Muribaculaceae | s__uncultured_bacterium |
| c82139470a1a2c52bdb0f03af5192116 | Bacteria | p__Actinobacteriota | c__Acidimicrobiia | o__IMCC26256 | f__IMCC26256 | g__IMCC26256 |  |
| HM629444 | Bacteria | p__Proteobacteria | c__Alphaproteobacteria | o__Sphingomonadales | f__Sphingomonadaceae | g__Sphingomonas |  |
| 888141bac90281214a83a87d2317a9b1 | Bacteria | p__Proteobacteria | c__Alphaproteobacteria | o__Rhizobiales | f__uncultured | g__uncultured | s__uncultured_Alphaproteobacteria |
| 5fb1dc93f3f37a39502f4148ce8a1538 | Bacteria | p__Firmicutes | c__Bacilli | o__Erysipelotrichales | f__Erysipelotrichaceae | g__ZOR0006 |  |
| 0cc16ae7dceb38e58c0b5515cfe1998d | Bacteria | p__Proteobacteria | c__Gammaproteobacteria | o__Cellvibrionales | f__Cellvibrionaceae | g__Cellvibrio |  |
| 11057686 | Bacteria | p__Bacteroidota | c__Bacteroidia | o__Bacteroidales | f__Muribaculaceae | g__Muribaculaceae |  |
| 21292683 | Bacteria | p__Firmicutes | c__Clostridia | o__Clostridiales | f__Clostridiaceae | g__Clostridium_sensu_stricto_1 |  |
| a10db11bf2a880453c9d8e49084a9f7e | Bacteria | p__Planctomycetota | c__Phycisphaerae | o__Tepidisphaerales | f__WD2101_soil_group | g__WD2101_soil_group |  |
| baf3b0f36d4128c0782d3dd569094bb2 | Bacteria | p__Proteobacteria | c__Gammaproteobacteria |  |  |  |  |
| d2ba9e61fd204595b7ab415981727ab8 | Bacteria | p__Actinobacteriota | c__Actinobacteria | o__Frankiales |  |  |  |
| 05007665 | Bacteria | p__Proteobacteria | c__Alphaproteobacteria | o__Sphingomonadales | f__Sphingomonadaceae | g__Sphingomonas |  |
| 33ca5755633c1940dc38d7e2ba6e520d | Bacteria | p__Gemmatimonadota | c__Gemmatimonadetes | o__Gemmatimonadales | f__Gemmatimonadaceae | g__Gemmatimonas |  |
| 05863092 | Bacteria | p__Planctomycetota | c__Phycisphaerae | o__Tepidisphaerales | f__WD2101_soil_group | g__WD2101_soil_group |  |
| e31d834f2b665f1d7c97ad7e1bf8b1a8 | Bacteria | p__Proteobacteria | c__Alphaproteobacteria | o__Sphingomonadales | f__Sphingomonadaceae | g__Sphingomonas |  |
| 481c317d533f2e562d894e182994e8fc | Bacteria | p__Proteobacteria | c__Gammaproteobacteria | o__Burkholderiales | f__A21b | g__A21b | s__uncultured_beta |
| 6e0d1aba9b1c33b3a6b98371e807b698 | Bacteria | p__Acidobacteriota | c__Vicinamibacteria | o__Vicinamibacterales | f__uncultured | g__uncultured |  |
| 04b53dcee895cbfc133e4938362ae0a6 | Bacteria | p__Proteobacteria | c__Gammaproteobacteria | o__Alteromonadales | f__Alteromonadaceae | g__Alishewanella |  |
| c0613fa2b321dfcf56a56e68eac04559 | Bacteria | p__Proteobacteria | c__Gammaproteobacteria | o__Enterobacterales |  |  |  |
| a625461391edbcdff55de85ea30d4002 | Bacteria | p__Proteobacteria | c__Gammaproteobacteria | o__Burkholderiales | f__Hydrogenophilaceae | g__Hydrogenophilus |  |
| c023c8ba35eb8f3fed8c16434bc40585 | Bacteria | p__Bacteroidota | c__Bacteroidia | o__Chitinophagales | f__Chitinophagaceae | g__Sediminibacterium | s__Sediminibacterium_sp. |
| 1c4df0896d450cb2b061fe75df1c34b4 | Bacteria | p__Desulfobacterota | c__uncultured | o__uncultured | f__uncultured | g__uncultured | s__uncultured_proteobacterium |
| 19244878 | Bacteria | p__Actinobacteriota | c__Actinobacteria | o__Micrococcales | f__Dermacoccaceae |  |  |
| RS-GCF-000620285.1-NZ-JHVT01000030.1 | Bacteria | p__Proteobacteria | c__Gammaproteobacteria | o__Pseudomonadales | f__Pseudomonadaceae | g__Pseudomonas |  |
| 04946241 | Bacteria | p__Actinobacteriota | c__Actinobacteria | o__Micrococcales | f__Micrococcaceae | g__Kocuria |  |
| f4615bac344e91478d5db799a719bbf1 | Bacteria | p__Firmicutes | c__Bacilli | o__Paenibacillales | f__Paenibacillaceae | g__Paenibacillus |  |
| RS-GCF-010994075.1-NZ-WKLG01000090.1 | Bacteria | p__Proteobacteria | c__Gammaproteobacteria | o__Xanthomonadales | f__Xanthomonadaceae | g__Stenotrophomonas |  |
| 0bd1a47f08440a0627caf0d7eb3fdbd6 | Bacteria | p__Actinobacteriota | c__Actinobacteria | o__Micrococcales |  |  |  |
| b4cee45483e55be2a50880bb9c6b3208 | Bacteria | p__Actinobacteriota | c__Acidimicrobiia | o__Microtrichales | f__Ilumatobacteraceae | g__Ilumatobacter |  |
| 8c06703b578a0e5a510e9ca705118958 | Bacteria | p__Proteobacteria | c__Gammaproteobacteria | o__Pseudomonadales | f__Moraxellaceae | g__Acinetobacter |  |
| 03b081eea1cbac14dd9cbbc7d948ecd2 | Bacteria | p__Firmicutes | c__Bacilli | o__Lactobacillales | f__Enterococcaceae | g__Enterococcus |  |
| 15ad56c195980e031cad3a0484cd7aa1 | Bacteria | p__Firmicutes | c__Bacilli | o__Bacillales | f__Bacillaceae | g__Geobacillus |  |
| 00179828 | Bacteria | p__Chloroflexi | c__Anaerolineae | o__SBR1031 | f__A4b | g__A4b | s__wastewater_metagenome |
| G000719975_1 | Bacteria | p__Actinobacteriota | c__Actinobacteria | o__Pseudonocardiales | f__Pseudonocardiaceae | g__Prauserella | s__Prauserella_rugosa |
| 04879566 | Bacteria | p__Proteobacteria | c__Gammaproteobacteria | o__Pseudomonadales | f__Pseudomonadaceae | g__Pseudomonas |  |
| GB-GCA-910576435.1-CAJTIQ010000015.1 | Bacteria | p__Firmicutes | c__Clostridia | o__Lachnospirales | f__Lachnospiraceae | g__GCA-900066575 | s__uncultured_bacterium |
| RS-GCF-000410615.1-NZ-APCQ01000135.1 | Bacteria | p__Proteobacteria | c__Alphaproteobacteria | o__Sphingomonadales | f__Sphingomonadaceae | g__Novosphingobium |  |
| 05013890 | Bacteria | p__Bacteroidota | c__Bacteroidia | o__Flavobacteriales | f__Weeksellaceae | g__Chryseobacterium |  |
| a07b2bcd44b4f91940ed20be145052cd | Bacteria | p__Firmicutes | c__Clostridia | o__Oscillospirales | f__Oscillospiraceae |  |  |
| 04966201 | Bacteria | p__Firmicutes | c__Bacilli | o__Bacillales | f__Bacillaceae | g__Bacillus |  |
| 726efc856f1c680b84cd93ac6f3588ec | Bacteria | p__Cyanobacteria | c__Cyanobacteriia | o__Oxyphotobacteria_Incertae_Sedis | f__Unknown_Family | g__Phormidium_SAG_37.90 |  |
| dedc0c4396e2116ffdccbffd1cdc76ea | Bacteria | p__Firmicutes | c__Bacilli | o__Bacillales | f__Marinococcaceae |  |  |
| 86d0d6b785d6ea051d6510eea6ab045f | Bacteria | p__Bdellovibrionota |  |  |  |  |  |
| d86804467f1804ec44cce7b32c974a40 | Bacteria | p__Firmicutes | c__Bacilli | o__Erysipelotrichales | f__Erysipelotrichaceae | g__Turicibacter | s__uncultured_bacterium |
| 8c62975b5595a76e5e9ae63c0d91ae6f | Bacteria | p__Proteobacteria | c__Gammaproteobacteria | o__Burkholderiales | f__SC-I-84 | g__SC-I-84 |  |
| de4731582b093065c3334a7acd52706c | Bacteria | p__Actinobacteriota | c__Thermoleophilia | o__Gaiellales |  |  |  |
| 31097bc9ff3215fd6d5b8de6e6fd906a | Bacteria | p__Verrucomicrobiota | c__Chlamydiae | o__Chlamydiales | f__Parachlamydiaceae | g__Neochlamydia |  |
| 66d76b7398a7a6ca52a804c8b9212f66 | Bacteria | p__Bacteroidota | c__Bacteroidia | o__Sphingobacteriales | f__Sphingobacteriaceae | g__Mucilaginibacter |  |
| a4ae5f232a0697955f47bacb675a2c85 | Bacteria | p__Acidobacteriota | c__Blastocatellia | o__Pyrinomonadales | f__Pyrinomonadaceae | g__RB41 |  |
| a1cb8db26918b1068dc1fb34475277a2 | Bacteria | p__Bacteroidota | c__Bacteroidia | o__Chitinophagales | f__Chitinophagaceae | g__Ferruginibacter |  |
| 777a00d3824ee1a09fbceb0a85566fb7 | Bacteria | p__Proteobacteria | c__Alphaproteobacteria | o__Rhizobiales | f__Devosiaceae |  |  |
| 04889629 | Bacteria | p__Chloroflexi | c__Anaerolineae | o__Anaerolineales | f__Anaerolineaceae | g__UTCFX1 |  |
| AF480599 | Bacteria | p__Actinobacteriota | c__Actinobacteria | o__Corynebacteriales | f__Mycobacteriaceae | g__Mycobacterium |  |
| 261a6ce2aa41a90798758209d3ff70c6 | Bacteria | p__Myxococcota | c__Myxococcia | o__Myxococcales | f__Anaeromyxobacteraceae | g__Anaeromyxobacter |  |
| EF466123 | Bacteria | p__Actinobacteriota | c__Actinobacteria | o__Propionibacteriales | f__Nocardioidaceae | g__Nocardioides | s__Nocardioides_islandensis |
| f1db09327e1ab179749d98ebdf69874a | Bacteria | p__Actinobacteriota | c__Actinobacteria | o__Micrococcales | f__Microbacteriaceae | g__Microbacterium |  |
| KM670029 | Bacteria | p__Proteobacteria | c__Alphaproteobacteria | o__Sphingomonadales | f__Sphingomonadaceae |  |  |
| 13702425 | Bacteria | p__Gemmatimonadota | c__Gemmatimonadetes | o__Gemmatimonadales | f__Gemmatimonadaceae | g__uncultured | s__Gemmatimonadetes_bacterium |
| 0e7ef916da507e9fb8185e4179a875e9 | Bacteria | p__Proteobacteria | c__Gammaproteobacteria | o__Pseudomonadales | f__Pseudomonadaceae | g__Pseudomonas |  |
| 32a5899303c8db331abad4e952df9cce | Bacteria | p__Firmicutes | c__Bacilli | o__Bacillales | f__Bacillaceae | g__Bacillus |  |
| 12dc357d33581835233a719f9f96e44f | Bacteria | p__Actinobacteriota | c__Thermoleophilia | o__Gaiellales | f__uncultured | g__uncultured |  |
| 67a8a4546d577ca411cb9c50ccd314d4 | Bacteria | p__Actinobacteriota | c__Actinobacteria | o__Propionibacteriales | f__Nocardioidaceae | g__Nocardioides |  |
| 04968196 | Bacteria | p__Firmicutes | c__Bacilli | o__Lactobacillales | f__Lactobacillaceae | g__Lactobacillus |  |
| 62c4698c00d2596d50152175b2ae0b75 | Bacteria | p__Firmicutes | c__Clostridia | o__Oscillospirales | f__Ruminococcaceae | g__Faecalibacterium |  |
| e8504eb1985ef42ae2d92e2f9eaa266d | Bacteria | p__Firmicutes | c__Clostridia | o__Oscillospirales | f__Ruminococcaceae | g__Faecalibacterium |  |
| 24546a8fb4a5ceb9ea164d810d1a7d0e | Bacteria | p__Proteobacteria | c__Gammaproteobacteria | o__Burkholderiales | f__Comamonadaceae |  |  |
| 19272263 | Bacteria | p__Actinobacteriota | c__Actinobacteria | o__Propionibacteriales | f__Nocardioidaceae | g__Nocardioides |  |
| 04665984 | Bacteria | p__Firmicutes | c__Bacilli | o__Lactobacillales | f__Streptococcaceae | g__Streptococcus |  |
| 746f4ed3986ef9c4d0833834a65d1444 | Bacteria | p__Myxococcota | c__Myxococcia | o__Myxococcales | f__Vulgatibacteraceae | g__Vulgatibacter | s__uncultured_Myxococcales |
| d0cc27293e60574b6d45e86fd75c8f27 | Bacteria | p__Proteobacteria | c__Gammaproteobacteria | o__Xanthomonadales | f__Rhodanobacteraceae | g__Dokdonella | s__uncultured_soil |
| 000df2d20c40e6d828dae58efcdd29ce | Bacteria | p__Proteobacteria | c__Gammaproteobacteria | o__Burkholderiales | f__Burkholderiaceae | g__Burkholderia-Caballeronia-Paraburkholderia |  |
| AGEG01000003 | Bacteria | p__Firmicutes | c__Bacilli | o__Lactobacillales | f__Aerococcaceae | g__Facklamia | s__Facklamia_languida |
| 11531301 | Bacteria | p__Desulfobacterota | c__Desulfovibrionia | o__Desulfovibrionales | f__Desulfovibrionaceae | g__Desulfovibrio |  |
| 8b254650d2936ab761e3e99500db98e8 | Bacteria | p__Actinobacteriota | c__Thermoleophilia | o__Solirubrobacterales | f__67-14 | g__67-14 |  |
| 12801788 | Bacteria | p__Bacteroidota | c__Bacteroidia | o__Chitinophagales | f__Chitinophagaceae | g__Flavisolibacter |  |
| f7bf9685161328a0a94b639b82ea5b30 | Bacteria | p__Actinobacteriota | c__Actinobacteria | o__Bifidobacteriales | f__Bifidobacteriaceae | g__Bifidobacterium |  |
| e13d16f22685abceb518f7e9703618a7 | Bacteria | p__Proteobacteria | c__Gammaproteobacteria | o__Thiotrichales | f__Thiotrichaceae | g__Thiothrix |  |
| 660d909726dce1e4ccc768ef72b575a0 | Bacteria | p__Proteobacteria | c__Alphaproteobacteria | o__Reyranellales | f__Reyranellaceae | g__Reyranella |  |
| 07202205 | Bacteria | p__Acidobacteriota | c__Vicinamibacteria | o__Vicinamibacterales | f__Vicinamibacteraceae | g__Vicinamibacteraceae |  |
| AB178406 | Bacteria | p__Proteobacteria | c__Alphaproteobacteria | o__Acetobacterales | f__Acetobacteraceae | g__Gluconobacter |  |
| e76742c70ddf2fd43233622b1bdbd199 | Bacteria | p__Chloroflexi | c__Anaerolineae | o__1-20 | f__1-20 | g__1-20 | s__uncultured_Chloroflexia |
| 19706818 | Bacteria | p__Firmicutes | c__Bacilli | o__Erysipelotrichales | f__Erysipelotrichaceae | g__Ileibacterium | s__Ileibacterium_valens |
| MJ033-1-barcode26-umi31402bins-ubs-7 | Bacteria | p__Proteobacteria | c__Gammaproteobacteria | o__Burkholderiales | f__Burkholderiaceae | g__Burkholderia-Caballeronia-Paraburkholderia |  |
| f2babd8f957e0f5bd47514aa81388014 | Bacteria | p__Proteobacteria | c__Gammaproteobacteria | o__Pseudomonadales | f__Moraxellaceae | g__Acinetobacter |  |
| 1f4a0436ef14131403f2255119967274 | Bacteria | p__Planctomycetota | c__Planctomycetes | o__Gemmatales | f__Gemmataceae | g__Gemmata | s__uncultured_endolithic |
| ce4dc79a689f03dc15e2a81a1ac7062f | Bacteria | p__Actinobacteriota | c__Acidimicrobiia | o__Microtrichales | f__Microtrichaceae | g__IMCC26207 |  |
| RS-GCF-001909085.1-NZ-LVYY01000053.1 | Bacteria | p__Firmicutes | c__Bacilli | o__Paenibacillales | f__Paenibacillaceae | g__Paenibacillus |  |
| MG753794 | Bacteria | p__Proteobacteria | c__Alphaproteobacteria | o__Sphingomonadales | f__Sphingomonadaceae |  |  |
| 18306689 | Bacteria | p__Actinobacteriota | c__Actinobacteria | o__Micrococcales | f__Microbacteriaceae | g__Agromyces | s__Agromyces_ulmi |
| aea9af574964701af9581bc46427198e | Bacteria | p__Proteobacteria | c__Gammaproteobacteria | o__Burkholderiales | f__SC-I-84 | g__SC-I-84 |  |
| 800fb2786e119cdd18e6b62cabdfede0 | Bacteria | p__Firmicutes | c__Bacilli | o__Lactobacillales | f__Enterococcaceae | g__Enterococcus |  |
| 339e76e60cd9cd42f23c90a618bb73af | Bacteria | p__Proteobacteria | c__Gammaproteobacteria | o__Burkholderiales | f__SC-I-84 | g__SC-I-84 |  |
| d2119d7a772719b1539f93e952010c00 | Bacteria | p__Proteobacteria | c__Gammaproteobacteria | o__Burkholderiales | f__Comamonadaceae | g__Comamonas |  |
| MG269198 | Bacteria | p__Proteobacteria | c__Alphaproteobacteria | o__Rhodobacterales | f__Rhodobacteraceae | g__Paracoccus |  |
| 11844362 | Bacteria | p__Proteobacteria | c__Alphaproteobacteria | o__Sphingomonadales | f__Sphingomonadaceae | g__Sphingomonas |  |
| AB047364 | Bacteria | p__Proteobacteria | c__Alphaproteobacteria | o__Sphingomonadales | f__Sphingomonadaceae | g__Sphingobium |  |
| 04910987 | Bacteria | p__Proteobacteria | c__Gammaproteobacteria | o__Xanthomonadales | f__Xanthomonadaceae | g__Stenotrophomonas |  |
| e9df35c06dc9bf59a95fc95d78fc5637 | Bacteria | p__Proteobacteria | c__Alphaproteobacteria | o__Azospirillales | f__Azospirillaceae | g__Skermanella |  |
| 16907235 | Bacteria | p__Firmicutes | c__Clostridia | o__Lachnospirales | f__Lachnospiraceae |  |  |
| e33f124d848681add2662c36a6b2728a | Bacteria | p__Methylomirabilota | c__Methylomirabilia | o__Rokubacteriales | f__Rokubacteriales | g__Rokubacteriales |  |
| 05010053 | Bacteria | p__Proteobacteria | c__Alphaproteobacteria | o__Rhodobacterales | f__Rhodobacteraceae | g__Rubellimicrobium |  |
| 14801041 | Bacteria | p__Chloroflexi | c__Ktedonobacteria | o__Ktedonobacterales | f__JG30-KF-AS9 | g__JG30-KF-AS9 | s__uncultured_bacterium |
| 04354930 | Bacteria | p__Chloroflexi | c__Ktedonobacteria | o__Ktedonobacterales | f__Ktedonobacteraceae | g__HSB_OF53-F07 | s__uncultured_Chloroflexales |
| 02936181 | Bacteria | p__Methylomirabilota | c__Methylomirabilia | o__Rokubacteriales | f__Rokubacteriales | g__Rokubacteriales | s__uncultured_bacterium |
| 5c1539fb56b1d65159381bc8939f2726 | Bacteria | p__Bacteroidota | c__Bacteroidia | o__Sphingobacteriales | f__Sphingobacteriaceae | g__Olivibacter | s__Olivibacter_sitiensis |
| b4d848f33c30bb4218baf37b31396571 | Bacteria | p__Proteobacteria | c__Gammaproteobacteria | o__Xanthomonadales | f__Rhodanobacteraceae | g__uncultured |  |
| 04918571 | Bacteria | p__Bacteroidota | c__Bacteroidia | o__Flavobacteriales | f__Flavobacteriaceae | g__Myroides |  |
| 440cd4284f6f1036844da98e237bf116 | Bacteria | p__Proteobacteria | c__Gammaproteobacteria | o__Xanthomonadales | f__Xanthomonadaceae | g__Stenotrophomonas |  |
| ad42b1852abe2791d34cd917f11a2390 | Bacteria | p__Proteobacteria | c__Alphaproteobacteria | o__Sphingomonadales | f__Sphingomonadaceae |  |  |
| 72a18d86f74c1da57af469dd7a673c37 | Bacteria | p__Firmicutes | c__Desulfitobacteriia | o__Desulfitobacteriales | f__Heliobacteriaceae | g__uncultured | s__uncultured_bacterium |
| ca4e8f2b34d09039a5930c4021501abd | Bacteria | p__Proteobacteria | c__Gammaproteobacteria | o__Enterobacterales |  |  |  |
| 942800ed962794829b7b97cade527517 | Bacteria | p__Actinobacteriota | c__Actinobacteria | o__Pseudonocardiales | f__Pseudonocardiaceae | g__Saccharopolyspora |  |
| 04952980 | Bacteria | p__Chloroflexi | c__TK10 | o__TK10 | f__TK10 | g__TK10 | s__uncultured_Chloroflexus |
| GB-GCA-902636635.1-CACPRY010000089.1 | Bacteria | p__Proteobacteria | c__Gammaproteobacteria | o__Enterobacterales | f__Enterobacteriaceae |  |  |
| X97359 | Bacteria | p__Proteobacteria | c__Gammaproteobacteria | o__Legionellales | f__Legionellaceae | g__Legionella | s__Legionella_lytica |
| G900117415_1 | Bacteria | p__Proteobacteria | c__Alphaproteobacteria | o__Rhizobiales | f__Hyphomicrobiaceae | g__Pedomicrobium | s__uncultured_Hyphomicrobium |
| d9f08d22a0e97f559a2e645550cedeea | Bacteria | p__Chloroflexi | c__Anaerolineae | o__SBR1031 | f__A4b | g__A4b | s__wastewater_metagenome |
| 2ae79916dfcff7e1963e89199a79a298 | Bacteria | p__Proteobacteria | c__Gammaproteobacteria | o__Pseudomonadales | f__Pseudomonadaceae | g__Pseudomonas |  |
| ac29802ef963284be3fff79b0b0d29ec | Bacteria | p__Actinobacteriota | c__Actinobacteria | o__Micrococcales | f__Microbacteriaceae |  |  |
| 04941579 | Bacteria | p__Campilobacterota | c__Campylobacteria | o__Campylobacterales | f__Campylobacteraceae | g__Campylobacter | s__Campylobacter_ureolyticus |
| 0e534e0aeb36e6c16a9aaf347b650be5 | Bacteria | p__Actinobacteriota | c__Thermoleophilia | o__Gaiellales |  |  |  |
| e50242179b5251f7a856f80d15d107f5 | Bacteria | p__Proteobacteria | c__Alphaproteobacteria | o__Tistrellales | f__Geminicoccaceae | g__Candidatus_Alysiosphaera |  |
| 04990576 | Bacteria | p__Verrucomicrobiota | c__Verrucomicrobiae | o__Chthoniobacterales | f__Chthoniobacteraceae | g__Candidatus_Udaeobacter |  |
| ea0392130293f305ff13b4016df0f518 | Bacteria | p__Bacteroidota | c__Bacteroidia | o__Bacteroidales | f__Bacteroidaceae | g__Bacteroides |  |
| RS-GCF-001876475.1-NZ-LWJW01000053.1 | Bacteria | p__Firmicutes | c__Bacilli | o__Staphylococcales | f__Staphylococcaceae | g__Staphylococcus |  |
| 08309589 | Bacteria | p__Proteobacteria | c__Alphaproteobacteria | o__Rhizobiales | f__Xanthobacteraceae | g__Pseudolabrys |  |
| 8639bbab7b06ae776d1a8c316907ff9c | Bacteria | p__Actinobacteriota | c__Actinobacteria | o__Micrococcales | f__Micrococcaceae |  |  |
| 14870807 | Bacteria | p__Chloroflexi | c__Ktedonobacteria | o__Ktedonobacterales | f__JG30-KF-AS9 | g__JG30-KF-AS9 |  |
| RS-GCF-003063525.1-NZ-NERZ01000006.1--2 | Bacteria | p__Proteobacteria | c__Gammaproteobacteria | o__Burkholderiales | f__Comamonadaceae |  |  |
| 5f5b019e4ea3fbe10a4bddd349211e04 | Bacteria | p__Firmicutes | c__Bacilli | o__Erysipelotrichales | f__Erysipelatoclostridiaceae | g__Erysipelatoclostridium | s__uncultured_Clostridium |
| 25b491903bd185027f8a7f94bd5305cb | Bacteria | p__Firmicutes | c__Clostridia | o__Lachnospirales | f__Lachnospiraceae |  |  |
| b83bd6d6e55832760ea9e9d42a91d741 | Bacteria | p__Proteobacteria | c__Alphaproteobacteria | o__Elsterales | f__uncultured | g__uncultured |  |
| 4b4b4710108b4ba3923b3f54bc543e3d | Bacteria | p__Proteobacteria | c__Gammaproteobacteria | o__Burkholderiales | f__Comamonadaceae | g__Lampropedia |  |
| RS-GCF-000310165.2-NZ-AMXC01000063.1 | Bacteria | p__Proteobacteria | c__Gammaproteobacteria | o__Burkholderiales | f__Rhodocyclaceae | g__Thauera |  |
| 04994213 | Bacteria | p__Proteobacteria | c__Alphaproteobacteria | o__Caulobacterales | f__Caulobacteraceae | g__Brevundimonas | s__Brevundimonas_olei |
| 02926354 | Bacteria | p__Planctomycetota | c__Phycisphaerae | o__Tepidisphaerales | f__WD2101_soil_group | g__WD2101_soil_group |  |
| AY211516 | Bacteria | p__Proteobacteria | c__Alphaproteobacteria | o__Rhizobiales | f__Xanthobacteraceae | g__Ancylobacter |  |
| 34f9b7d13718c244eacce8ab58a85854 | Bacteria | p__Proteobacteria | c__Gammaproteobacteria | o__Xanthomonadales | f__Rhodanobacteraceae | g__Dokdonella |  |
| 1cbd0c7c97f432f841e496099ad050e5 | Bacteria | p__Firmicutes | c__Bacilli | o__Lactobacillales | f__Enterococcaceae | g__Enterococcus |  |
| 915acab545e1911458ade18651d9bb01 | Bacteria | p__Chloroflexi |  |  |  |  |  |
| MJ030-2-barcode73-umi83764bins-ubs-6 | Bacteria | p__Firmicutes | c__Clostridia | o__Lachnospirales | f__Lachnospiraceae |  |  |
| RS-GCF-016025255.1-NZ-CP065592.1 | Bacteria | p__Proteobacteria | c__Alphaproteobacteria | o__Sphingomonadales | f__Sphingomonadaceae |  |  |
| 04959900 | Bacteria | p__Proteobacteria | c__Gammaproteobacteria | o__Burkholderiales | f__Comamonadaceae |  |  |
| 8d0f988311539eb030d7114f5cbbcc44 | Bacteria | p__Actinobacteriota | c__Actinobacteria | o__Pseudonocardiales | f__Pseudonocardiaceae | g__Saccharopolyspora |  |
| 9c61f5b4d3015e568b0de518b4b8c321 | Bacteria | p__Proteobacteria | c__Alphaproteobacteria | o__Elsterales | f__uncultured | g__uncultured |  |
| FN386750 | Bacteria | p__Actinobacteriota | c__Actinobacteria | o__Propionibacteriales | f__Nocardioidaceae | g__Marmoricola |  |
| 5a312aa8d667fa7d4914a4e0308d688d | Bacteria | p__Proteobacteria | c__Alphaproteobacteria | o__Rhizobiales | f__KF-JG30-B3 | g__KF-JG30-B3 |  |
| f9f585bba0683d63c568296577ded57a | Bacteria | p__Firmicutes | c__Bacilli | o__Lactobacillales | f__Enterococcaceae | g__Enterococcus |  |
| AB032355 | Bacteria | p__Proteobacteria | c__Alphaproteobacteria | o__Acetobacterales | f__Acetobacteraceae | g__Acetobacter |  |
| d1624806ea260338c3dc1d9efa64cc9a | Bacteria | p__Bacteroidota | c__Bacteroidia | o__Bacteroidales | f__Tannerellaceae | g__Parabacteroides | s__uncultured_Bacteroidales |
| 998dc72cb28831de25350d51e155d680 | Bacteria | p__Bacteroidota | c__Bacteroidia | o__Bacteroidales | f__Muribaculaceae | g__Muribaculaceae | s__uncultured_bacterium |
| 0b55e7b45be8684d3baf8d8ae44b5deb | Bacteria | p__Chloroflexi | c__Anaerolineae | o__SBR1031 | f__A4b | g__A4b |  |
| 02250695 | Bacteria | p__Proteobacteria | c__Alphaproteobacteria | o__Caulobacterales | f__Caulobacteraceae | g__Brevundimonas |  |
| 9fc0a4c3e9b1eacc80f9925741b74591 | Bacteria |  |  |  |  |  |  |
| MJ033-1-barcode36-umi1689bins-ubs-200 | Bacteria | p__Bacteroidota | c__Bacteroidia | o__Chitinophagales | f__Chitinophagaceae | g__Niastella | s__uncultured_Niastella |
| 13170533 | Bacteria | p__Bacteroidota | c__Bacteroidia | o__Cytophagales | f__Hymenobacteraceae | g__Adhaeribacter |  |
| 05014576 | Bacteria | p__Bacteroidota | c__Bacteroidia | o__Cytophagales | f__Hymenobacteraceae | g__Pontibacter | s__Pontibacter_populi |
| RS-GCF-002261475.1-NZ-NEVN01000001.1 | Bacteria | p__Proteobacteria | c__Gammaproteobacteria | o__Burkholderiales | f__Alcaligenaceae |  |  |
| a27fe35ad5bc22175f02453a3ee2d013 | Bacteria | p__Proteobacteria | c__Alphaproteobacteria | o__uncultured | f__uncultured | g__uncultured |  |
| AJ011506 | Bacteria | p__Proteobacteria | c__Gammaproteobacteria | o__Burkholderiales | f__Rhodocyclaceae | g__Zoogloea |  |
| be40333313ff7fa73d8e88461d898662 | Bacteria | p__Bacteroidota | c__Bacteroidia | o__Bacteroidales | f__Bacteroidales_UCG-001 | g__Bacteroidales_UCG-001 |  |
| 862349cfcf8fce12335b2dcb182152cb | Bacteria | p__Actinobacteriota | c__Thermoleophilia | o__Gaiellales | f__uncultured | g__uncultured |  |
| MJ030-1-barcode25-umi105653bins-ubs-3 | Bacteria | p__Bacteroidota | c__Bacteroidia | o__Chitinophagales | f__Chitinophagaceae | g__Flavisolibacter |  |
| f2dd7d2b33bd6db6ee342a395c373f8c | Bacteria | p__Proteobacteria | c__Gammaproteobacteria | o__Methylococcales | f__Methylomonadaceae | g__Methylomicrobium | s__uncultured_bacterium |
| 1241a59f9bf47a2023f7736a9d569feb | Bacteria | p__Firmicutes | c__Clostridia | o__Oscillospirales | f__Oscillospiraceae |  |  |
| b6593b6953a5860ec048db3d671d2013 | Bacteria | p__Proteobacteria | c__Gammaproteobacteria | o__Burkholderiales | f__Methylophilaceae | g__Methylobacillus |  |
| 7373a0f4aa65f084833be7b5d437760e | Bacteria | p__Cyanobacteria | c__Cyanobacteriia | o__Pseudanabaenales | f__Pseudanabaenaceae | g__Pseudanabaena_PCC-7429 |  |
| MJ006-2-barcode62-umi148638bins-ubs-3 | Bacteria | p__Firmicutes | c__Clostridia | o__Lachnospirales | f__Lachnospiraceae | g__Tyzzerella | s__unidentified |
| KU201960 | Bacteria | p__Bacteroidota | c__Bacteroidia | o__Sphingobacteriales | f__Sphingobacteriaceae | g__Sphingobacterium | s__Sphingobacterium_zeae |
| 73a7515c04b4eca43be383b28a824031 | Bacteria | p__Actinobacteriota | c__Actinobacteria | o__Corynebacteriales | f__Corynebacteriaceae | g__Corynebacterium |  |
| 3ad54d63e66a5db62d039cf49555d891 | Bacteria | p__Gemmatimonadota | c__Gemmatimonadetes | o__Gemmatimonadales | f__Gemmatimonadaceae | g__uncultured |  |
| 04958772 | Bacteria | p__Actinobacteriota | c__Actinobacteria | o__Micrococcales | f__Microbacteriaceae |  |  |
| d410f63d0a9ea7cea73bb8336192e344 | Bacteria | p__Desulfobacterota | c__Desulfuromonadia | o__Geobacterales | f__Geobacteraceae |  |  |
| 08148744 | Bacteria | p__Proteobacteria | c__Alphaproteobacteria | o__Acetobacterales | f__Acetobacteraceae | g__Roseomonas |  |
| 02d2770b4c1dc43a90c113f9fd9f48bc | Bacteria | p__Firmicutes | c__Bacilli | o__Exiguobacterales | f__Exiguobacteraceae | g__Exiguobacterium |  |
| d7084c2c48739b6afd11f465cc66e52e | Bacteria | p__Proteobacteria | c__Gammaproteobacteria | o__Pseudomonadales | f__Moraxellaceae | g__Acinetobacter |  |
| 4a06e408fe2813bf0cfc054894c035e5 | Bacteria | p__Firmicutes | c__Bacilli | o__Caldalkalibacillales | f__Caldalkalibacillaceae | g__Caldalkalibacillus |  |
| RS-GCF-003481765.1-NZ-QULR01000098.1 | Bacteria | p__Firmicutes | c__Clostridia | o__Lachnospirales | f__Lachnospiraceae | g__Lachnoclostridium |  |
| 4f1eccc890adb2e3b89d53a156753a1f | Bacteria | p__Proteobacteria | c__Alphaproteobacteria | o__Rhodobacterales | f__Rhodobacteraceae |  |  |
| 04879738 | Bacteria | p__Proteobacteria | c__Alphaproteobacteria | o__Rhizobiales | f__Beijerinckiaceae | g__Methylobacterium-Methylorubrum |  |
| 3c7f5fddc12df638c3b0260cdcaf0f2b | Bacteria | p__Acidobacteriota | c__Vicinamibacteria | o__Vicinamibacterales | f__uncultured | g__uncultured |  |
| 04964374 | Bacteria | p__Firmicutes | c__Clostridia | o__Oscillospirales | f__Oscillospiraceae | g__Colidextribacter |  |
| 06869959 | Bacteria | p__Verrucomicrobiota | c__Verrucomicrobiae | o__Pedosphaerales | f__Pedosphaeraceae | g__Pedosphaeraceae | s__uncultured_Prosthecobacter |
| 15890470 | Bacteria | p__Actinobacteriota | c__Actinobacteria | o__Micrococcales | f__Micrococcaceae | g__Arthrobacter |  |
| c220e271f58c74afc02265ee55592573 | Bacteria | p__Proteobacteria | c__Alphaproteobacteria | o__uncultured | f__uncultured | g__uncultured | s__uncultured_actinobacterium |
| FR821260 | Bacteria | p__Actinobacteriota | c__Actinobacteria | o__Corynebacteriales | f__Dietziaceae | g__Dietzia |  |
| GB-GCA-002813375.1-PHUJ01000003.1--6 | Bacteria | p__Actinobacteriota | c__Actinobacteria | o__Pseudonocardiales | f__Pseudonocardiaceae | g__Pseudonocardia |  |
| 643492336229ebb94b38c0d8af7e8641 | Bacteria | p__Actinobacteriota | c__MB-A2-108 | o__MB-A2-108 | f__MB-A2-108 | g__MB-A2-108 | s__uncultured_bacterium |
| 04956857 | Bacteria | p__Chloroflexi | c__Chloroflexia | o__Thermomicrobiales | f__JG30-KF-CM45 | g__JG30-KF-CM45 | s__uncultured_soil |
| 3bedfddd4b2caae0b86389d33ee8f80a | Bacteria | p__Actinobacteriota | c__Actinobacteria |  |  |  |  |
| fc40201731a8dc3caf7c5f5058cb079b | Bacteria | p__Proteobacteria | c__Gammaproteobacteria |  |  |  |  |
| AM940158 | Bacteria | p__Actinobacteriota | c__Actinobacteria | o__Micrococcales | f__Microbacteriaceae |  |  |
| 15911553 | Bacteria | p__Actinobacteriota | c__Actinobacteria | o__Micrococcales | f__Micrococcaceae | g__Glutamicibacter |  |
| 3035f547fd6b1280fdc1cd7574535f9c | Bacteria | p__Proteobacteria | c__Gammaproteobacteria | o__Enterobacterales | f__Morganellaceae | g__endosymbionts |  |
| 04875858 | Bacteria | p__Proteobacteria | c__Gammaproteobacteria | o__Pseudomonadales | f__Moraxellaceae | g__Enhydrobacter |  |
| 32f3176bd851976f1eda5db4b2909acd | Bacteria | p__Proteobacteria | c__Gammaproteobacteria | o__Burkholderiales | f__SC-I-84 | g__SC-I-84 |  |
| a68721c6b546ea48c0869b73fcd54c51 | Bacteria | p__Proteobacteria | c__Gammaproteobacteria | o__Burkholderiales | f__Sutterellaceae | g__Parasutterella | s__uncultured_bacterium |
| RS-GCF-004302315.1-NZ-ML154982.1 | Bacteria | p__Firmicutes | c__Bacilli | o__Lactobacillales | f__Lactobacillaceae | g__Lactobacillus |  |
| G000179395_1 | Bacteria | p__Actinobacteriota | c__Actinobacteria | o__Corynebacteriales | f__Corynebacteriaceae | g__Corynebacterium | s__Corynebacterium_variabile |
| 11124666 | Bacteria | p__Bacteroidota | c__Bacteroidia | o__Bacteroidales | f__Muribaculaceae | g__Muribaculaceae |  |
| AB681337 | Bacteria | p__Proteobacteria | c__Gammaproteobacteria | o__Xanthomonadales | f__Xanthomonadaceae | g__Pseudoxanthomonas |  |
| 8cfb28fbec5eedc15de78636ed64757a | Bacteria | p__Proteobacteria | c__Gammaproteobacteria | o__Burkholderiales | f__Nitrosomonadaceae | g__Ellin6067 |  |
| 3a220481d828e2026547bf04bcb63b8b | Bacteria | p__Firmicutes | c__Bacilli | o__Erysipelotrichales | f__Erysipelatoclostridiaceae | g__Erysipelatoclostridium | s__uncultured_Clostridium |
| 2f845ea050043018268d507d86139120 | Bacteria | p__Proteobacteria | c__Alphaproteobacteria | o__Rhizobiales | f__Devosiaceae | g__Devosia |  |
| e6aa1acaaac55284db639258cbbaf735 | Bacteria | p__Bacteroidota | c__Bacteroidia | o__Flavobacteriales | f__Weeksellaceae | g__Empedobacter | s__Empedobacter_brevis |
| 14a4f92da70ab0f8bb52e04f03944ea0 | Bacteria | p__Proteobacteria | c__Alphaproteobacteria | o__Rhizobiales | f__Devosiaceae | g__Devosia |  |
| 04876122 | Bacteria | p__Proteobacteria | c__Alphaproteobacteria | o__Rhizobiales | f__Beijerinckiaceae | g__Microvirga |  |
| 18931798 | Bacteria | p__Proteobacteria | c__Gammaproteobacteria | o__Burkholderiales | f__Comamonadaceae |  |  |
| RS-GCF-000022665.1-NC-012971.2--4 | Bacteria | p__Proteobacteria | c__Gammaproteobacteria | o__Enterobacterales | f__Enterobacteriaceae | g__Escherichia-Shigella |  |
| 984a033b442b6e58fcfe55e4b1b186b4 | Bacteria | p__Actinobacteriota | c__Actinobacteria | o__Propionibacteriales | f__Nocardioidaceae | g__Nocardioides |  |
| 04917466 | Bacteria | p__Bacteroidota | c__Bacteroidia | o__Flavobacteriales | f__Flavobacteriaceae | g__Flavobacterium | s__Flavobacterium_ummariense |
| 1f4c8713fd7062a63e4055dd3c5f7cd5 | Bacteria | p__Proteobacteria | c__Gammaproteobacteria | o__Steroidobacterales | f__Steroidobacteraceae | g__uncultured |  |
| RS-GCF-003481175.1-NZ-QUKF01000070.1 | Bacteria | p__Firmicutes | c__Clostridia | o__Lachnospirales | f__Lachnospiraceae | g__Fusicatenibacter |  |
| MJ033-1-barcode47-umi12525bins-ubs-23 | Bacteria | p__Bacteroidota | c__Bacteroidia | o__Chitinophagales | f__Saprospiraceae | g__uncultured | s__uncultured_soil |
| 04959992 | Bacteria | p__Proteobacteria | c__Gammaproteobacteria | o__Burkholderiales | f__Comamonadaceae | g__Aquabacterium | s__Aquabacterium_citratiphilum |
| a70e8061afe2fe5c9a77424d0352e5b8 | Bacteria | p__Bacteroidota | c__Bacteroidia | o__Sphingobacteriales | f__Sphingobacteriaceae | g__Sphingobacterium |  |
| 1dfecbe19aacb578763b1349fb6f63ae | Bacteria | p__Proteobacteria | c__Gammaproteobacteria | o__Pasteurellales | f__Pasteurellaceae | g__Haemophilus |  |
| dd4729cb1e685bd397d189741aaadd42 | Bacteria | p__Firmicutes | c__Clostridia | o__Peptostreptococcales-Tissierellales | f__Sedimentibacteraceae | g__Sedimentibacter |  |
| 604e8500050ce1813dfb01a193c9925a | Bacteria | p__Actinobacteriota | c__Rubrobacteria | o__Rubrobacterales | f__Rubrobacteriaceae | g__Rubrobacter |  |
| 18446523 | Bacteria | p__Proteobacteria | c__Gammaproteobacteria | o__Burkholderiales | f__Alcaligenaceae | g__Verticiella |  |
| 688cb2d718caf1aa29a103103441ce7d | Bacteria | p__Proteobacteria | c__Alphaproteobacteria | o__Rhizobiales | f__Beijerinckiaceae |  |  |
| fe7345109787eef883743ca50a0bcee6 | Bacteria | p__Actinobacteriota | c__Actinobacteria | o__Propionibacteriales | f__Nocardioidaceae | g__Nocardioides |  |
| 72e7a5f2a87f50a23c79ee644d016f3f | Bacteria | p__Firmicutes | c__Bacilli | o__Erysipelotrichales | f__Erysipelatoclostridiaceae | g__Erysipelatoclostridium |  |
| AM941396 | Bacteria | p__Proteobacteria | c__Gammaproteobacteria | o__Oceanospirillales | f__Halomonadaceae | g__Halomonas |  |
| 9488a3c7a9535a1d5b8107d8651b8974 | Bacteria | p__Firmicutes | c__Bacilli | o__Lactobacillales | f__Lactobacillaceae | g__Lactobacillus |  |
| a64b2c744bcf7c5772492f8c163b60ab | Bacteria | p__Proteobacteria | c__Alphaproteobacteria | o__Caulobacterales | f__Caulobacteraceae |  |  |
| ada4446c122bd5bd07e9dd4c23d5cf8e | Bacteria | p__Proteobacteria | c__Gammaproteobacteria | o__Burkholderiales | f__Burkholderiaceae | g__Pandoraea |  |
| DQ515964 | Bacteria | p__Actinobacteriota | c__Actinobacteria | o__Micrococcales | f__Microbacteriaceae |  |  |
| G900248245_1 | Bacteria | p__Firmicutes | c__Clostridia | o__Lachnospirales | f__Lachnospiraceae | g__Acetatifactor |  |
| 59f7c926a894087da78d078d2df6996f | Bacteria | p__Proteobacteria | c__Gammaproteobacteria | o__Pseudomonadales | f__Pseudomonadaceae | g__Pseudomonas | s__Pseudomonas_luteola |
| MJ030-2-barcode65-umi5895bins-ubs-63 | Bacteria | p__Acidobacteriota | c__Acidobacteriae | o__Acidobacteriales | f__Koribacteraceae | g__Candidatus_Koribacter |  |
| G000196815_1 | Bacteria | p__Nitrospirota | c__Nitrospiria | o__Nitrospirales | f__Nitrospiraceae | g__Nitrospira |  |
| MF143629 | Bacteria | p__Proteobacteria | c__Gammaproteobacteria | o__Enterobacterales | f__Morganellaceae | g__Proteus |  |
| bf2b31ce7676b25b9b29dd06e4c389be | Bacteria | p__Acidobacteriota | c__Holophagae | o__Holophagales | f__Holophagaceae |  |  |
| 89f16dd93157f479c6e2a55d9cbb2dc9 | Bacteria | p__Firmicutes | c__Bacilli | o__Erysipelotrichales | f__Erysipelatoclostridiaceae | g__Erysipelatoclostridium |  |
| G902387315_1 | Bacteria | p__Firmicutes | c__Clostridia | o__Oscillospirales | f__Oscillospiraceae | g__uncultured |  |
| 22e83f6715940ba22c9127b71f282440 | Bacteria | p__Actinobacteriota | c__Acidimicrobiia | o__IMCC26256 | f__IMCC26256 | g__IMCC26256 |  |
| db827729879194fdf92ed5eeeeb64926 | Bacteria | p__Firmicutes | c__Bacilli | o__Lactobacillales | f__Carnobacteriaceae | g__Trichococcus |  |
| 7daf7024b7df019cbaefec872e8adc16 | Bacteria | p__Actinobacteriota | c__Actinobacteria |  |  |  |  |
| 4b11fb8eef27f510e65c862489d064b7 | Bacteria | p__Proteobacteria | c__Gammaproteobacteria | o__Xanthomonadales | f__Xanthomonadaceae | g__Stenotrophomonas |  |
| 19236273 | Bacteria | p__Actinobacteriota | c__Actinobacteria | o__Micrococcales | f__Intrasporangiaceae |  |  |
| MF407345 | Bacteria | p__Firmicutes | c__Bacilli | o__Lactobacillales | f__Carnobacteriaceae | g__Trichococcus |  |
| 1c13dbff7e2a245b6811c2f6593225ed | Bacteria | p__Proteobacteria | c__Alphaproteobacteria | o__uncultured | f__uncultured | g__uncultured |  |
| 92099881b4ecd9cc1500147a580e888d | Bacteria | p__Bacteroidota | c__Bacteroidia | o__Chitinophagales | f__Chitinophagaceae | g__Vibrionimonas |  |
| GB-GCA-003755025.1-RJVL01000011.1 | Bacteria | p__Proteobacteria | c__Gammaproteobacteria | o__Burkholderiales | f__Comamonadaceae | g__Diaphorobacter |  |
| 0774eca2874bc27dd33728e33b2c1159 | Bacteria | p__Desulfobacterota | c__Desulfovibrionia | o__Desulfovibrionales | f__Desulfovibrionaceae | g__Bilophila |  |
| cbcfbb34e09d100ee84b5a9112640062 | Bacteria | p__Proteobacteria | c__Gammaproteobacteria | o__Burkholderiales | f__Comamonadaceae |  |  |
| 05538170 | Bacteria | p__Firmicutes | c__Bacilli | o__Entomoplasmatales | f__Spiroplasmataceae | g__Spiroplasma |  |
| 04558138 | Bacteria | p__Firmicutes | c__Bacilli | o__Erysipelotrichales | f__Erysipelotrichaceae | g__Faecalibaculum | s__uncultured_bacterium |
| 2cfce6f63b15a33cbc61a063f2721c38 | Bacteria | p__Actinobacteriota | c__Thermoleophilia | o__Solirubrobacterales | f__67-14 | g__67-14 |  |
| ed2a9126db4d52d1d44113f7353fcae5 | Bacteria | p__Proteobacteria | c__Gammaproteobacteria | o__Burkholderiales | f__SC-I-84 | g__SC-I-84 |  |
| RS-GCF-000799595.1-NZ-JUGY01000106.1 | Bacteria | p__Firmicutes | c__Bacilli | o__Paenibacillales | f__Paenibacillaceae | g__Paenibacillus |  |
| bfe38c8af021a68e354169dd5fbe5b57 | Bacteria | p__Proteobacteria | c__Gammaproteobacteria | o__Burkholderiales | f__Rhodocyclaceae |  |  |
| 8608a5bae522b4ba74d3dec139eb439a | Bacteria | p__Chloroflexi | c__Anaerolineae | o__Caldilineales | f__Caldilineaceae | g__uncultured | s__uncultured_bacterium |
| MJ009-1-barcode27-umi50165bins-ubs-7 | Bacteria | p__Proteobacteria | c__Gammaproteobacteria | o__Burkholderiales | f__Alcaligenaceae | g__Achromobacter |  |
| e25e0bb6a87ff4243266a7b2638ae7d4 | Bacteria | p__Proteobacteria | c__Gammaproteobacteria | o__Burkholderiales | f__Burkholderiaceae | g__Burkholderia-Caballeronia-Paraburkholderia |  |
| MJ006-1-barcode45-umi138904bins-ubs-4 | Bacteria | p__Firmicutes | c__Clostridia | o__Clostridia_vadinBB60_group | f__Clostridia_vadinBB60_group | g__Clostridia_vadinBB60_group | s__gut_metagenome |
| 29995ba1d7f8ee08d3196707419c3995 | Bacteria | p__Proteobacteria | c__Gammaproteobacteria |  |  |  |  |
| MJ033-1-barcode49-umi11773bins-ubs-27 | Bacteria | p__Acidobacteriota | c__Blastocatellia | o__Blastocatellales | f__Blastocatellaceae | g__JGI_0001001-H03 |  |
| 05863084 | Bacteria | p__Planctomycetota | c__Phycisphaerae | o__Tepidisphaerales | f__WD2101_soil_group | g__WD2101_soil_group | s__uncultured_bacterium |
| beb1f3021c6a91dce83eaec7e2d59159 | Bacteria | p__Gemmatimonadota | c__Gemmatimonadetes | o__Gemmatimonadales | f__Gemmatimonadaceae |  |  |
| 00ddd24c46f38828616367650e12e868 | Bacteria | p__Firmicutes | c__Bacilli | o__Erysipelotrichales | f__Erysipelatoclostridiaceae | g__Erysipelatoclostridium | s__uncultured_Clostridium |
| 20fd44301524d1d1c9eb3c85e654e53c | Bacteria | p__Firmicutes | c__Bacilli | o__Erysipelotrichales | f__Erysipelotrichaceae | g__ZOR0006 | s__uncultured_organism |
| 06812767 | Bacteria | p__Verrucomicrobiota | c__Verrucomicrobiae | o__Chthoniobacterales | f__Chthoniobacteraceae | g__Chthoniobacter |  |
| MJ034-1-barcode31-umi25133bins-ubs-5 | Bacteria | p__Proteobacteria | c__Gammaproteobacteria | o__Gammaproteobacteria_Incertae_Sedis | f__Unknown_Family | g__Acidibacter |  |
| b28e78a1a47a7812ff289a491fd96ba7 | Bacteria | p__Proteobacteria | c__Gammaproteobacteria | o__Xanthomonadales | f__Xanthomonadaceae |  |  |
| 4fa7a266aff5d0a093e6e56db3c96a68 | Bacteria | p__Actinobacteriota | c__Thermoleophilia | o__Gaiellales | f__uncultured | g__uncultured |  |
| 00204286 | Bacteria | p__Chloroflexi | c__Anaerolineae | o__Anaerolineales | f__Anaerolineaceae |  |  |
| AF035054 | Bacteria | p__Proteobacteria | c__Gammaproteobacteria | o__Burkholderiales | f__Comamonadaceae | g__Aquabacterium |  |
| 3450c32438164c60a57b50e0484a3afb | Bacteria | p__Actinobacteriota | c__Thermoleophilia | o__Solirubrobacterales | f__Solirubrobacteraceae | g__Solirubrobacter |  |
| a3dcd6c211f34a8c2cf41c622029e646 | Bacteria | p__Chloroflexi | c__TK10 | o__TK10 | f__TK10 | g__TK10 |  |
| G000741215_2 | Bacteria | p__Actinobacteriota | c__Actinobacteria | o__Bifidobacteriales | f__Bifidobacteriaceae | g__Bifidobacterium | s__Bifidobacterium_pullorum |
| 04959840 | Bacteria | p__Proteobacteria | c__Gammaproteobacteria | o__Burkholderiales | f__Burkholderiaceae | g__Burkholderia-Caballeronia-Paraburkholderia |  |
| MJ010-2-barcode64-umi79073bins-ubs-6 | Bacteria | p__Bdellovibrionota | c__Bdellovibrionia | o__Bacteriovoracales | f__Bacteriovoracaceae | g__Peredibacter | s__microbial_mat |
| 04908295 | Bacteria | p__Proteobacteria | c__Alphaproteobacteria | o__Rhizobiales | f__Hyphomicrobiaceae | g__Pedomicrobium |  |
| e7047b6b53cf5bc7a110ea2a69c0fbd9 | Bacteria | p__Acidobacteriota | c__Vicinamibacteria | o__Vicinamibacterales | f__Vicinamibacteraceae | g__Vicinamibacteraceae |  |
| 66596a81c6ca6dd6e8c2cf4eb426d181 | Bacteria | p__Proteobacteria | c__Gammaproteobacteria | o__Burkholderiales | f__Comamonadaceae |  |  |
| 8d8b8fd508a4c8ee9e9f30b9d197c985 | Bacteria | p__Bacteroidota | c__Bacteroidia | o__Sphingobacteriales | f__env.OPS_17 | g__env.OPS_17 | s__uncultured_Bacteroidetes |
| 06993348 | Bacteria | p__Verrucomicrobiota | c__Verrucomicrobiae | o__Chthoniobacterales | f__Chthoniobacteraceae | g__Chthoniobacter | s__uncultured_Spartobacteria |
| MJ034-1-barcode46-umi4593bins-ubs-102 | Bacteria | p__Acidobacteriota | c__Blastocatellia | o__Blastocatellales | f__Blastocatellaceae |  |  |
| G000688095_1 | Bacteria | p__Actinobacteriota | c__Thermoleophilia | o__Solirubrobacterales | f__Solirubrobacteraceae | g__Conexibacter |  |
| a5ff5f1fc2543a7599770fb6580214d5 | Bacteria | p__Actinobacteriota | c__MB-A2-108 | o__MB-A2-108 | f__MB-A2-108 | g__MB-A2-108 |  |
| 28552de00fef08e776e70daf8f5b8064 | Bacteria | p__Proteobacteria | c__Gammaproteobacteria | o__Enterobacterales | f__Enterobacteriaceae |  |  |
| 14521678 | Bacteria | p__Gemmatimonadota | c__Gemmatimonadetes | o__Gemmatimonadales | f__Gemmatimonadaceae | g__uncultured |  |
| be159a0f6bb86bce71118b608d1fe8d9 | Bacteria | p__Bacteroidota | c__Bacteroidia | o__Chitinophagales | f__Chitinophagaceae |  |  |
| 89115c4ff4db5536c1b71ce92e23761c | Bacteria | p__Methylomirabilota | c__Methylomirabilia | o__Rokubacteriales | f__Rokubacteriales | g__Rokubacteriales |  |
| 528942f0c67dec75bbed4a6fd856f943 | Bacteria | p__Proteobacteria | c__Gammaproteobacteria | o__Burkholderiales | f__SC-I-84 | g__SC-I-84 |  |
| 04666512 | Bacteria | p__Firmicutes | c__Bacilli | o__Lactobacillales | f__Streptococcaceae | g__Streptococcus |  |
| 984b42667fd184c66a5b2450d68e9c50 | Bacteria | p__Actinobacteriota | c__Actinobacteria |  |  |  |  |
| 42428e56cfd7490ff5729eb9ff6a8ab7 | Bacteria | p__Cyanobacteria | c__Cyanobacteriia | o__Cyanobacteriales | f__Coleofasciculaceae |  |  |
| RS-GCF-000804305.1-NZ-JWHB01000055.1 | Bacteria | p__Proteobacteria | c__Gammaproteobacteria | o__Pseudomonadales | f__Moraxellaceae | g__Acinetobacter | s__Acinetobacter_variabilis |
| ab2d2e02b5c61270e90b19483fcf8da6 | Bacteria | p__Firmicutes | c__Negativicutes | o__Veillonellales-Selenomonadales | f__Veillonellaceae | g__Dialister | s__Dialister_succinatiphilus |
| d48e9fe143b930188a77ec6f988f2e58 | Bacteria | p__Firmicutes | c__Negativicutes | o__Veillonellales-Selenomonadales | f__Veillonellaceae | g__Dialister |  |
| GB-GCA-014896945.1-CP054719.1 | Bacteria | p__Proteobacteria | c__Alphaproteobacteria | o__Paracaedibacterales | f__Paracaedibacteraceae | g__uncultured | s__uncultured_prokaryote |
| a6bf8115dfc243f261270702f9d1e315 | Bacteria | p__Proteobacteria | c__Alphaproteobacteria | o__Azospirillales | f__Azospirillaceae | g__Azospirillum |  |
| 5143bac95d89515fe487101b02cf4625 | Bacteria | p__Proteobacteria | c__Gammaproteobacteria | o__Enterobacterales | f__Enterobacteriaceae |  |  |
| 3a7396aef30a1cc7415692f346c20a82 | Bacteria | p__Proteobacteria | c__Gammaproteobacteria | o__Pseudomonadales | f__Moraxellaceae | g__Enhydrobacter |  |
| 7c37f566f998d949cd65b5ce3af6288c | Bacteria | p__Bacteroidota | c__Bacteroidia | o__Bacteroidales | f__Rikenellaceae | g__Alistipes |  |
| 08549374 | Bacteria | p__Proteobacteria | c__Alphaproteobacteria | o__Rhizobiales | f__Beijerinckiaceae | g__Roseiarcus |  |
| 31554ff8c82dc6959afc80819e10d198 | Bacteria | p__Proteobacteria | c__Alphaproteobacteria | o__Elsterales | f__uncultured | g__uncultured |  |
| fb9d970af3cb74ae2122fa1815da255a | Bacteria | p__Bacteroidota | c__Bacteroidia | o__Flavobacteriales | f__Flavobacteriaceae | g__Flavobacterium |  |
| 4f6151096b2765041d5bdfb447e191fc | Bacteria | p__Proteobacteria | c__Gammaproteobacteria | o__Enterobacterales | f__Enterobacteriaceae |  |  |
| 80b6c7fd9aa1461ac81d60c5649dc49f | Bacteria | p__Actinobacteriota | c__Acidimicrobiia | o__Microtrichales | f__Iamiaceae | g__Iamia |  |
| 04930891 | Bacteria | p__Proteobacteria | c__Gammaproteobacteria | o__Enterobacterales | f__Yersiniaceae |  |  |
| RS-GCF-003951535.1-NZ-RPDN01000052.1 | Bacteria | p__Proteobacteria | c__Gammaproteobacteria | o__Enterobacterales | f__Enterobacteriaceae |  |  |
| GB-GCA-902636595.1-CACPSM010000097.1 | Bacteria | p__Firmicutes | c__Bacilli | o__Paenibacillales | f__Paenibacillaceae | g__Paenibacillus |  |
| 04966154 | Bacteria | p__Firmicutes | c__Bacilli | o__Bacillales | f__Bacillaceae | g__Bacillus |  |
| e7bd181f1a2361cc8fce97af789ec9d9 | Bacteria | p__Proteobacteria | c__Gammaproteobacteria | o__Pseudomonadales | f__Pseudomonadaceae | g__Pseudomonas |  |
| 26c4213099d9dd4dc60bd24dbd51f7a8 | Bacteria | p__Proteobacteria | c__Gammaproteobacteria | o__Pseudomonadales | f__Moraxellaceae | g__Acinetobacter |  |
| 48328811e6cac899c764b3d812408473 | Bacteria | p__Proteobacteria | c__Alphaproteobacteria | o__Reyranellales | f__Reyranellaceae | g__Reyranella |  |
| LR797591 | Bacteria | p__Proteobacteria | c__Gammaproteobacteria | o__Pseudomonadales | f__Pseudomonadaceae | g__Pseudomonas |  |
| 78f36a48dc63eebc208791d9732c7bbd | Bacteria | p__Proteobacteria | c__Alphaproteobacteria | o__Sphingomonadales | f__Sphingomonadaceae | g__Sphingomonas |  |
| G000764535_3 | Bacteria | p__Proteobacteria | c__Alphaproteobacteria | o__Sphingomonadales | f__Sphingomonadaceae | g__Sphingomonas |  |
| RS-GCF-900113835.1-NZ-FOQV01000007.1 | Bacteria | p__Proteobacteria | c__Alphaproteobacteria | o__Rhizobiales | f__Beijerinckiaceae | g__Bosea |  |
| 58e608d1be0b67887ba873308c5604be | Bacteria | p__Proteobacteria | c__Gammaproteobacteria | o__Burkholderiales | f__Rhodocyclaceae |  |  |
| 3f4ed1da091869d03e61b112620d569e | Bacteria | p__Proteobacteria | c__Alphaproteobacteria | o__Rhodobacterales | f__Rhodobacteraceae | g__Amaricoccus | s__uncultured_bacterium |
| d6279736acef30cc229056b992de477f | Bacteria | p__Proteobacteria | c__Alphaproteobacteria | o__Rhizobiales | f__Rhizobiaceae |  |  |
| 14801570 | Bacteria | p__Chloroflexi | c__Ktedonobacteria | o__Ktedonobacterales | f__JG30-KF-AS9 | g__JG30-KF-AS9 | s__uncultured_bacterium |
| 7ab427b4312c4d757c0d21776396e235 | Bacteria | p__Actinobacteriota | c__Actinobacteria |  |  |  |  |
| cf3004d6e18c29aadaa7c71a34861837 | Bacteria | p__Proteobacteria | c__Gammaproteobacteria | o__Burkholderiales | f__Oxalobacteraceae | g__Massilia |  |
| MJ034-1-barcode48-umi28513bins-ubs-3 | Bacteria | p__Acidobacteriota | c__Acidobacteriae | o__Bryobacterales | f__Bryobacteraceae | g__Bryobacter |  |
| 01e8888c2c1e421a47b6c235a397e6c3 | Bacteria | p__Proteobacteria | c__Gammaproteobacteria | o__Xanthomonadales | f__Xanthomonadaceae | g__Lysobacter |  |
| d71daaa0bad22aaa077c86c2a982fd51 | Bacteria | p__Firmicutes | c__Clostridia | o__Lachnospirales | f__Lachnospiraceae | g__Tyzzerella |  |
| f181070a9442cb7dbc06468f1eb21d16 | Bacteria | p__Proteobacteria | c__Gammaproteobacteria | o__Burkholderiales | f__Comamonadaceae |  |  |
| f7a0360e74bbb0d43ac9d017bcd2c03b | Bacteria | p__Chloroflexi | c__KD4-96 | o__KD4-96 | f__KD4-96 | g__KD4-96 | s__metagenome |
| 9896e8361f3f7d88a64863d1fb1e1868 | Bacteria | p__Firmicutes | c__Bacilli | o__Exiguobacterales | f__Exiguobacteraceae | g__Exiguobacterium |  |
| MG729590 | Bacteria | p__Actinobacteriota | c__Actinobacteria | o__Propionibacteriales | f__Nocardioidaceae | g__Kribbella |  |
| MJ031-1-barcode30-umi12776bins-ubs-41 | Bacteria | p__Bacteroidota | c__Bacteroidia | o__Cytophagales | f__Microscillaceae | g__uncultured |  |
| 04989568 | Bacteria | p__Proteobacteria | c__Gammaproteobacteria | o__Steroidobacterales | f__Steroidobacteraceae |  |  |
| f9d9f54d1cbbfd77fed064f2cef8e984 | Bacteria | p__Bacteroidota | c__Bacteroidia | o__Bacteroidales | f__Muribaculaceae | g__Muribaculaceae | s__uncultured_bacterium |
| abdfa95141b1bd4a8acc1f58faf78230 | Bacteria | p__Acidobacteriota | c__Acidobacteriae | o__Bryobacterales | f__Bryobacteraceae | g__Bryobacter |  |
| 3c01491559bab617e37991a9b9338f56 | Bacteria | p__Bacteroidota | c__Bacteroidia | o__Bacteroidales | f__Bacteroidaceae | g__Bacteroides | s__uncultured_Bacteroidales |
| f231ceaf684bcf86d64162d23b8c051e | Bacteria | p__Cyanobacteria | c__Cyanobacteriia | o__Oxyphotobacteria_Incertae_Sedis | f__Unknown_Family | g__Leptolyngbya_EcFYyyy-00 | s__uncultured_bacterium |
| eb8d9b0c5c54623d8f9f1eb895a4a173 | Bacteria | p__Bacteroidota | c__Bacteroidia | o__Flavobacteriales | f__Weeksellaceae | g__Chryseobacterium |  |
| 237e23c9dfc308f7b84d60565f368bba | Bacteria | p__Acidobacteriota | c__Blastocatellia | o__Blastocatellales | f__Blastocatellaceae | g__Tellurimicrobium | s__Tellurimicrobium_multivorans |
| 01803739 | Bacteria | p__Actinobacteriota | c__Acidimicrobiia | o__Microtrichales | f__Iamiaceae | g__Iamia |  |
| G001618685_1 | Bacteria | p__Firmicutes | c__Bacilli | o__Bacillales | f__Bacillaceae | g__Geobacillus |  |
| RS-GCF-019067065.1-NZ-JAGIXT010000050.1 | Bacteria | p__Actinobacteriota | c__Actinobacteria | o__Micrococcales | f__Micrococcaceae | g__Paenarthrobacter |  |
| RS-GCF-001807075.1-NZ-KV787400.1 | Bacteria | p__Actinobacteriota | c__Actinobacteria | o__Corynebacteriales | f__Corynebacteriaceae | g__Corynebacterium |  |
| 02266152 | Bacteria | p__Actinobacteriota | c__Actinobacteria | o__Pseudonocardiales | f__Pseudonocardiaceae | g__Pseudonocardia |  |
| e1d7250ed3130f820c5aee8b7fcf8b1d | Bacteria | p__Actinobacteriota | c__Coriobacteriia | o__Coriobacteriales | f__Coriobacteriaceae | g__Collinsella |  |
| 67fdbe017447a56cff702784350a0ca9 | Bacteria | p__Actinobacteriota | c__Actinobacteria | o__Pseudonocardiales | f__Pseudonocardiaceae | g__Saccharopolyspora |  |
| f379857e7abec6a74391cea8b2c3d7b3 | Bacteria | p__Proteobacteria | c__Gammaproteobacteria | o__Cellvibrionales | f__Cellvibrionaceae | g__Cellvibrio |  |
| FJ156081 | Bacteria | p__Bacteroidota | c__Bacteroidia | o__Sphingobacteriales | f__Sphingobacteriaceae | g__Sphingobacterium | s__Sphingobacterium_psychroaquaticum |
| dd16a3fdd3061c12c22dcd8ceb0d092c | Bacteria | p__Planctomycetota | c__Phycisphaerae | o__Tepidisphaerales | f__WD2101_soil_group | g__WD2101_soil_group | s__uncultured_bacterium |
| 17459763 | Bacteria | p__Actinobacteriota | c__Thermoleophilia | o__Gaiellales | f__uncultured | g__uncultured |  |
| 5e1aa97720ee13d06b11d83288d86b7f | Bacteria | p__Proteobacteria | c__Gammaproteobacteria | o__Burkholderiales | f__Comamonadaceae |  |  |
| d67c9c9fd4f5a427c707427c039c9017 | Bacteria | p__Proteobacteria | c__Alphaproteobacteria | o__Rhizobiales | f__Xanthobacteraceae | g__uncultured |  |
| 16efb9c1a1e3f1b39a42051ed82e1be1 | Bacteria | p__Actinobacteriota | c__Thermoleophilia | o__Gaiellales | f__uncultured | g__uncultured |  |
| RS-GCF-015351275.1-NZ-JADKSL010000041.1 | Bacteria | p__Actinobacteriota | c__Actinobacteria | o__Micrococcales | f__Microbacteriaceae |  |  |
| G001006805_1 | Bacteria | p__Proteobacteria | c__Alphaproteobacteria | o__Rhizobiales | f__Beijerinckiaceae | g__Microvirga |  |
| 4ba4cbba4e97cbd7170b67e112d248cf | Bacteria | p__Actinobacteriota | c__Actinobacteria | o__Corynebacteriales | f__Corynebacteriaceae | g__Corynebacterium |  |
| AJ277886 | Bacteria | p__Desulfobacterota | c__Desulfovibrionia | o__Desulfovibrionales | f__Desulfomicrobiaceae | g__Desulfomicrobium |  |
| FM995533 | Bacteria | p__Actinobacteriota | c__Actinobacteria | o__Corynebacteriales | f__Dietziaceae | g__Dietzia |  |
| MJ030-1-barcode25-umi45360bins-ubs-10 | Bacteria | p__Firmicutes | c__Bacilli | o__Bacillales | f__Bacillaceae | g__Bacillus |  |
| 397eda1c642e7bd34896924bb5d224b2 | Bacteria | p__Firmicutes | c__Clostridia | o__Oscillospirales | f__Ruminococcaceae | g__Subdoligranulum |  |
| 391cdf04222c7e83335a8553e9b61515 | Bacteria | p__Chloroflexi | c__Anaerolineae | o__SBR1031 | f__A4b | g__A4b |  |
| 05110885 | Bacteria | p__Proteobacteria | c__Gammaproteobacteria | o__Burkholderiales | f__Burkholderiaceae | g__Pandoraea |  |
| b143ce01d6025f6cb28e12e6bfa51b69 | Bacteria | p__Proteobacteria | c__Gammaproteobacteria | o__Burkholderiales | f__Nitrosomonadaceae | g__mle1-7 | s__uncultured_Nitrosomonadaceae |
| G003438075_1 | Bacteria | p__Firmicutes | c__Clostridia | o__Oscillospirales | f__Ruminococcaceae | g__CAG-352 | s__uncultured_bacterium |
| 03177548 | Bacteria | p__Planctomycetota | c__Phycisphaerae | o__Tepidisphaerales | f__WD2101_soil_group | g__WD2101_soil_group | s__uncultured_bacterium |
| 0b28b0bdce53ef9fbab2a51063cc1ee2 | Bacteria | p__Actinobacteriota | c__Actinobacteria | o__Pseudonocardiales | f__Pseudonocardiaceae | g__Saccharopolyspora | s__Saccharopolyspora_rectivirgula |
| b8f4d8935136ffa1e246dc8ccb9fcc89 | Bacteria | p__Chloroflexi | c__KD4-96 | o__KD4-96 | f__KD4-96 | g__KD4-96 |  |
| a35305fbcde67d02e51260319bb5ded0 | Bacteria | p__Actinobacteriota | c__Acidimicrobiia | o__Microtrichales | f__Ilumatobacteraceae | g__CL500-29_marine_group |  |
| MJ030-2-barcode65-umi28499bins-ubs-24 | Bacteria | p__Acidobacteriota | c__Acidobacteriae | o__Solibacterales | f__Solibacteraceae | g__Candidatus_Solibacter | s__uncultured_Acidobacteriaceae |
| f6f87f95479cbcb3100a80f58b1ff9de | Bacteria | p__Bacteroidota | c__Bacteroidia | o__Sphingobacteriales | f__AKYH767 | g__AKYH767 | s__uncultured_bacterium |
| 1a943cc7fcd6411b034837ba17bdb8ae | Bacteria | p__Proteobacteria | c__Alphaproteobacteria | o__Sphingomonadales | f__Sphingomonadaceae |  |  |
| c1a523633429243eae0c233058bac0c0 | Bacteria | p__Proteobacteria | c__Alphaproteobacteria | o__Paracaedibacterales | f__Paracaedibacteraceae | g__Candidatus_Paracaedibacter |  |
| b00c465f256baeb6cb46b5f762f12b76 | Bacteria | p__Actinobacteriota | c__Actinobacteria | o__Pseudonocardiales | f__Pseudonocardiaceae | g__Pseudonocardia |  |
| 04877703 | Bacteria | p__Firmicutes | c__Clostridia | o__Oscillospirales | f__[Eubacterium]_coprostanoligenes_group | g__[Eubacterium]_coprostanoligenes_group | s__uncultured_bacterium |
| d63e32f2014bc00fd2c0de01623eeadf | Bacteria | p__Proteobacteria | c__Gammaproteobacteria | o__Alteromonadales | f__Alteromonadaceae | g__Rheinheimera |  |
| X80633 | Bacteria | p__Actinobacteriota | c__Actinobacteria | o__Corynebacteriales | f__Nocardiaceae | g__Gordonia |  |
| 05037939 | Bacteria | p__Firmicutes | c__Clostridia | o__Oscillospirales | f__Oscillospiraceae | g__Oscillibacter | s__uncultured_bacterium |
| 4ecba5be307cb4a924301d35be7aac0d | Bacteria | p__Firmicutes | c__Bacilli | o__Bacillales | f__Planococcaceae | g__Domibacillus |  |
| 04665992 | Bacteria | p__Firmicutes | c__Bacilli | o__Lactobacillales | f__Streptococcaceae | g__Streptococcus |  |
| 06376721 | Bacteria | p__Gemmatimonadota | c__S0134_terrestrial_group | o__S0134_terrestrial_group | f__S0134_terrestrial_group | g__S0134_terrestrial_group | s__uncultured_Gemmatimonadetes |
| 05037488 | Bacteria | p__Chloroflexi | c__Gitt-GS-136 | o__Gitt-GS-136 | f__Gitt-GS-136 | g__Gitt-GS-136 |  |
| FR774994 | Bacteria | p__Proteobacteria | c__Alphaproteobacteria | o__Rhizobiales | f__Beijerinckiaceae | g__Bosea |  |
| GB-GCA-003006655.1-PXIN01000324.1 | Bacteria | p__Proteobacteria | c__Gammaproteobacteria | o__Xanthomonadales | f__Xanthomonadaceae | g__Stenotrophomonas |  |
| 82c7aedf7d856fd847c359acc3e419fa | Bacteria | p__Proteobacteria | c__Alphaproteobacteria | o__Caulobacterales | f__Caulobacteraceae | g__Brevundimonas |  |
| RS-GCF-007923255.1-NZ-VOHJ01000066.1 | Bacteria | p__Proteobacteria | c__Gammaproteobacteria | o__Xanthomonadales | f__Xanthomonadaceae | g__Vulcaniibacterium | s__uncultured_bacterium |
| 05029457 | Bacteria | p__Chloroflexi | c__Chloroflexia | o__Chloroflexales | f__Herpetosiphonaceae | g__Herpetosiphon | s__uncultured_bacterium |
| 7ce73d64d4aa5f05a71fff7a2ac0ae3a | Bacteria | p__Bacteroidota | c__Bacteroidia | o__Bacteroidales | f__Dysgonomonadaceae | g__Proteiniphilum |  |
| 19677587 | Bacteria | p__Elusimicrobiota | c__Lineage_IIa | o__Lineage_IIa | f__Lineage_IIa | g__Lineage_IIa |  |
| ace595c42c5fb08ba2ddc417e73fb7b4 | Bacteria | p__Actinobacteriota | c__Actinobacteria | o__Frankiales | f__Acidothermaceae | g__Acidothermus |  |
| a2e4a32173434e669bd8443f84191628 | Bacteria | p__Actinobacteriota | c__Thermoleophilia | o__Solirubrobacterales | f__67-14 | g__67-14 |  |
| KT997477 | Bacteria | p__Proteobacteria | c__Gammaproteobacteria | o__Pseudomonadales | f__Moraxellaceae | g__Acinetobacter |  |
| 74e7ea34460cfb02aabeb292cf839abe | Bacteria | p__Actinobacteriota | c__Actinobacteria | o__Pseudonocardiales | f__Pseudonocardiaceae | g__Pseudonocardiaceae | s__Bounagaea_algeriensis |
| 9cf80eacbbed35693221f9f07e25a208 | Bacteria | p__Proteobacteria | c__Gammaproteobacteria | o__Aeromonadales | f__Aeromonadaceae | g__Aeromonas |  |
| d8d1be8db33bdc8eb0c0610e8580e604 | Bacteria | p__Proteobacteria | c__Gammaproteobacteria | o__Pseudomonadales | f__Moraxellaceae | g__Acinetobacter |  |
| MJ006-2-barcode58-umi167044bins-ubs-3 | Bacteria | p__Firmicutes | c__Clostridia | o__Oscillospirales | f__Ruminococcaceae | g__Incertae_Sedis |  |
| 2c2bb08b3bc33bfe00de4fe2fa4b79ee | Bacteria | p__Actinobacteriota | c__Actinobacteria | o__Micrococcales | f__Microbacteriaceae |  |  |
| MJ034-1-barcode31-umi12101bins-ubs-28 | Bacteria | p__Proteobacteria | c__Gammaproteobacteria | o__Burkholderiales | f__Oxalobacteraceae | g__Undibacterium | s__Undibacterium_arcticum |
| 378f715f9d722b6d546ca4b83e3b07b2 | Bacteria | p__Actinobacteriota | c__MB-A2-108 | o__MB-A2-108 | f__MB-A2-108 | g__MB-A2-108 | s__uncultured_actinobacterium |
| 1dc7608cc06dff247cb4317c6a4bab86 | Bacteria | p__Firmicutes | c__Clostridia | o__Lachnospirales | f__Lachnospiraceae |  |  |
| 05132328 | Bacteria | p__Firmicutes | c__Bacilli | o__Bacillales | f__Bacillaceae | g__Bacillus |  |
| cb48307d80d5786fa18e677e7767a189 | Bacteria | p__Proteobacteria | c__Alphaproteobacteria | o__Rhizobiales | f__Hyphomicrobiaceae | g__Hyphomicrobium |  |
| f1129ac2af15d1894e5e2447efd49365 | Bacteria | p__Chloroflexi | c__Anaerolineae |  |  |  |  |
| GB-GCA-000304595.1-AMQQ01000088.1 | Bacteria | p__Proteobacteria | c__Alphaproteobacteria | o__Rhizobiales | f__Rhizobiaceae | g__Allorhizobium-Neorhizobium-Pararhizobium-Rhizobium |  |
| bc8344b532e2f6b652a0042db35071ee | Bacteria | p__Proteobacteria | c__Gammaproteobacteria | o__Pseudomonadales | f__Moraxellaceae | g__Acinetobacter |  |
| 01255366 | Bacteria | p__Firmicutes | c__Clostridia | o__Lachnospirales | f__Lachnospiraceae |  |  |
| 92624ec80f7392e6b5a8d9b95300bfa7 | Bacteria | p__Proteobacteria | c__Alphaproteobacteria | o__Rhizobiales |  |  |  |
| 9295287f172432094af622fce8ef5db9 | Bacteria | p__Proteobacteria | c__Alphaproteobacteria | o__Acetobacterales | f__Acetobacteraceae | g__Acidisphaera | s__uncultured_Alphaproteobacteria |
| MJ034-1-barcode48-umi13133bins-ubs-24 | Bacteria | p__Bacteroidota | c__Bacteroidia | o__Chitinophagales | f__Chitinophagaceae | g__Flavisolibacter |  |
| 0a7551bcb58a74e3f27db0c53aea13d1 | Bacteria | p__Bacteroidota | c__Bacteroidia | o__Chitinophagales | f__Chitinophagaceae |  |  |
| 05009548 | Bacteria | p__Proteobacteria | c__Alphaproteobacteria | o__Sphingomonadales | f__Sphingomonadaceae | g__Sphingomonas |  |
| 31d678058dbc621f44927a1c86f8871b | Bacteria | p__Proteobacteria | c__Gammaproteobacteria | o__Burkholderiales | f__Comamonadaceae | g__Comamonas |  |
| MJ020-2-barcode70-umi36588bins-ubs-13 | Bacteria | p__Proteobacteria | c__Gammaproteobacteria | o__Pseudomonadales | f__Pseudomonadaceae | g__Pseudomonas |  |
| 233dbdbd67603352c0ecac41bccafb1a | Bacteria | p__Actinobacteriota | c__Actinobacteria | o__Corynebacteriales | f__Corynebacteriaceae | g__Corynebacterium |  |
| 04897240 | Bacteria | p__Cyanobacteria | c__Cyanobacteriia |  |  |  |  |
| MJ034-2-barcode71-umi16722bins-ubs-4 | Bacteria | p__Myxococcota | c__bacteriap25 | o__bacteriap25 | f__bacteriap25 | g__bacteriap25 |  |
| KP001330 | Bacteria | p__Firmicutes | c__Bacilli | o__Lactobacillales | f__Streptococcaceae | g__Streptococcus |  |
| 14801536 | Bacteria | p__Chloroflexi | c__Ktedonobacteria | o__Ktedonobacterales | f__JG30-KF-AS9 | g__JG30-KF-AS9 | s__uncultured_bacterium |
| 7f3bbc034376242e8ab0014579f4f930 | Bacteria |  |  |  |  |  |  |
| 7848d54352ebad241ce7c7824dd40fc8 | Bacteria | p__Actinobacteriota | c__Actinobacteria | o__Bifidobacteriales | f__Bifidobacteriaceae | g__Bifidobacterium | s__Bifidobacterium_animalis |
| 73b89f29df28320f6a2644864f265f46 | Bacteria | p__Actinobacteriota | c__Actinobacteria | o__Corynebacteriales | f__Corynebacteriaceae | g__Corynebacterium |  |
| MH094643 | Bacteria | p__Proteobacteria | c__Alphaproteobacteria | o__Caulobacterales | f__Caulobacteraceae | g__Brevundimonas |  |
| RS-GCF-017798005.1-NZ-CP072548.1--2 | Bacteria | p__Proteobacteria | c__Gammaproteobacteria | o__Pasteurellales | f__Pasteurellaceae | g__Aggregatibacter | s__uncultured_bacterium |
| 45d584b17081f058f6dfb22664dd1bf1 | Bacteria | p__Firmicutes | c__Bacilli | o__Lactobacillales | f__Aerococcaceae | g__Dolosicoccus | s__Dolosicoccus_paucivorans |
| X80739 | Bacteria | p__Actinobacteriota | c__Actinobacteria | o__Micrococcales | f__Micrococcaceae | g__Glutamicibacter |  |
| d8ff1280881d9c8648514585b9ee5699 | Bacteria | p__Firmicutes | c__Bacilli | o__Lactobacillales | f__Enterococcaceae | g__Enterococcus |  |
| GB-GCA-016717205.1-JADKGC010000006.1 | Bacteria | p__Chloroflexi | c__Anaerolineae | o__SBR1031 | f__A4b | g__A4b |  |
| RS-GCF-001466835.1-NZ-LKEF01000083.1 | Bacteria | p__Proteobacteria | c__Gammaproteobacteria | o__Pseudomonadales | f__Pseudomonadaceae | g__Pseudomonas |  |
| 927b82de71a21626614b65ee251c1060 | Bacteria | p__Patescibacteria | c__Saccharimonadia | o__Saccharimonadales |  |  |  |
| c5c8287e92e6295eafd149b254283eac | Bacteria | p__Actinobacteriota | c__Actinobacteria | o__Corynebacteriales | f__Corynebacteriaceae | g__Corynebacterium |  |
| RS-GCF-000469425.1-NZ-KI271465.1 | Bacteria | p__Firmicutes | c__Clostridia | o__Oscillospirales | f__Oscillospiraceae | g__Oscillibacter |  |
| KF900163 | Bacteria | p__Actinobacteriota | c__Actinobacteria | o__Propionibacteriales | f__Nocardioidaceae | g__Nocardioides | s__Nocardioides_zeae |
| 1da64dc9185592700da72e9829f975ab | Bacteria | p__Acidobacteriota | c__Vicinamibacteria | o__Vicinamibacterales | f__Vicinamibacteraceae | g__Vicinamibacteraceae |  |
| 52735aa63bc13bbb5c165d52923d7f14 | Bacteria | p__Actinobacteriota | c__Acidimicrobiia | o__IMCC26256 | f__IMCC26256 | g__IMCC26256 | s__uncultured_Ferrimicrobium |
| 04877057 | Bacteria | p__Proteobacteria | c__Gammaproteobacteria | o__Enterobacterales | f__Erwiniaceae |  |  |
| 7212088ed7eb489ce20f9e2779c3ba08 | Bacteria | p__Proteobacteria | c__Gammaproteobacteria | o__Burkholderiales | f__Comamonadaceae |  |  |
| 02364475 | Bacteria | p__Proteobacteria | c__Alphaproteobacteria | o__Rhizobiales | f__KF-JG30-B3 | g__KF-JG30-B3 |  |
| 8bbc0701e906327c58b5337a6df48dc0 | Bacteria | p__Firmicutes | c__Bacilli | o__Paenibacillales | f__Paenibacillaceae | g__Paenibacillus |  |
| 33c0cc430319904d0853406dcaab3ebb | Bacteria | p__Proteobacteria | c__Alphaproteobacteria | o__Rhizobiales | f__Xanthobacteraceae |  |  |
| 04943491 | Bacteria | p__Acidobacteriota | c__Acidobacteriae | o__Bryobacterales | f__Bryobacteraceae | g__Bryobacter |  |
| G009708035_1 | Bacteria | p__Proteobacteria | c__Alphaproteobacteria | o__Rhizobiales | f__Hyphomicrobiaceae | g__Hyphomicrobium |  |
| dfa53406de2930ff823a61c40aa41540 | Bacteria | p__Proteobacteria | c__Gammaproteobacteria | o__Burkholderiales | f__Comamonadaceae |  |  |
| 77a85dbea0fc3f10b2835623f4d6f0be | Bacteria | p__Proteobacteria | c__Gammaproteobacteria | o__Xanthomonadales | f__Rhodanobacteraceae | g__Dokdonella | s__uncultured_Dokdonella |
| 04912142 | Bacteria | p__Proteobacteria | c__Alphaproteobacteria | o__Rhodospirillales | f__uncultured | g__uncultured | s__metagenome |
| 682aac44b9bc9ee260b56b94eb80dc34 | Bacteria | p__Desulfobacterota | c__Desulfovibrionia | o__Desulfovibrionales | f__Desulfovibrionaceae | g__uncultured | s__uncultured_bacterium |
| d800d16f2ae10d691970dcfa90f409df | Bacteria | p__Firmicutes | c__Bacilli | o__Alicyclobacillales | f__Alicyclobacillaceae | g__Tumebacillus |  |
| 3944108cda4862629d92777113c5b843 | Bacteria | p__Actinobacteriota | c__Actinobacteria | o__Micrococcales | f__Microbacteriaceae | g__Microbacterium |  |
| 08479837 | Bacteria | p__Proteobacteria | c__Alphaproteobacteria | o__Rhizobiales | f__Rhizobiaceae |  |  |
| 4335b9dad36771125c48e09f280f8d11 | Bacteria | p__Acidobacteriota | c__Holophagae | o__Subgroup_7 | f__Subgroup_7 | g__Subgroup_7 |  |
| 24627a65af2613c8a99e5f909b986f77 | Bacteria | p__Acidobacteriota | c__Acidobacteriae | o__Acidobacteriales | f__uncultured | g__uncultured |  |
| 1a95e650d176684879d29b2275dd50ba | Bacteria | p__Proteobacteria | c__Gammaproteobacteria | o__Pseudomonadales | f__Pseudomonadaceae | g__Pseudomonas |  |
| GB-GCA-018006385.1-JAGMYM010000089.1 | Bacteria | p__Actinobacteriota | c__Acidimicrobiia | o__Microtrichales | f__Ilumatobacteraceae | g__CL500-29_marine_group |  |
| MJ030-2-barcode65-umi28502bins-ubs-23 | Bacteria | p__Proteobacteria | c__Gammaproteobacteria | o__Burkholderiales | f__Comamonadaceae |  |  |
| HM777012 | Bacteria | p__Proteobacteria | c__Alphaproteobacteria | o__Caulobacterales | f__Caulobacteraceae | g__Brevundimonas |  |
| 502c309ec6aaac9dad967ed305d31b5f | Bacteria | p__Proteobacteria | c__Gammaproteobacteria | o__Enterobacterales | f__Enterobacteriaceae | g__Escherichia-Shigella |  |
| 3d2ade02bdff7d2c6088ed9e3d5d4891 | Bacteria | p__Proteobacteria | c__Alphaproteobacteria | o__Rhizobiales | f__Rhizobiaceae |  |  |
| b7c2857fe9fa44696061fc2a28bbffec | Bacteria | p__Proteobacteria | c__Gammaproteobacteria | o__Enterobacterales | f__Enterobacteriaceae |  |  |
| MJ030-2-barcode58-umi75999bins-ubs-7 | Bacteria | p__Proteobacteria | c__Alphaproteobacteria | o__Rhizobiales | f__Rhizobiales_Incertae_Sedis | g__uncultured | s__metagenome |
| 23a84fb4e10fc84eccc02744bb2fc7c4 | Bacteria | p__Chloroflexi | c__Anaerolineae | o__SBR1031 | f__SBR1031 | g__SBR1031 |  |
| 48581e0a05d53bfedc6c3d8c57d1ddcb | Bacteria | p__Acidobacteriota | c__Vicinamibacteria | o__Subgroup_17 | f__Subgroup_17 | g__Subgroup_17 |  |
| RS-GCF-003471295.1-NZ-QRIU01000015.1 | Bacteria | p__Actinobacteriota | c__Actinobacteria | o__Bifidobacteriales | f__Bifidobacteriaceae | g__Bifidobacterium |  |
| 7a5779d86040b0291cf9866b50ea5f8e | Bacteria | p__Proteobacteria | c__Alphaproteobacteria | o__Rhizobiales | f__Rhizobiales_Incertae_Sedis | g__uncultured |  |
| RS-GCF-009677535.1-NZ-WKNF01000241.1 | Bacteria | p__Proteobacteria | c__Gammaproteobacteria | o__Burkholderiales | f__Sutterellaceae | g__Parasutterella |  |
| HQ612241 | Bacteria | p__Actinobacteriota | c__Actinobacteria | o__Corynebacteriales | f__Corynebacteriaceae | g__Corynebacterium | s__Corynebacterium_deserti |
| 70353f59889626afa638e8047d3608dc | Bacteria | p__Firmicutes | c__Bacilli | o__Lactobacillales | f__Streptococcaceae | g__Streptococcus |  |
| 2d69e97a181ba6e74d664e1952ffcb74 | Bacteria | p__Proteobacteria | c__Alphaproteobacteria | o__Rhizobiales | f__Xanthobacteraceae | g__Bradyrhizobium |  |
| 05034105 | Bacteria | p__Actinobacteriota | c__Actinobacteria | o__Corynebacteriales | f__Corynebacteriaceae | g__Turicella | s__uncultured_bacterium |
| 228c81603d34bf73c4e26f87f77fd6ed | Bacteria | p__Actinobacteriota | c__Thermoleophilia | o__Solirubrobacterales | f__67-14 | g__67-14 | s__metagenome |
| f8f9787e229cbf12b9d6c1a76b8d72fd | Bacteria | p__Proteobacteria | c__Gammaproteobacteria | o__Steroidobacterales | f__Steroidobacteraceae | g__uncultured |  |
| 30f5e57f5195124eaf5d77fce82d1f0e | Bacteria | p__Proteobacteria | c__Gammaproteobacteria | o__Xanthomonadales | f__Xanthomonadaceae | g__Pseudoxanthomonas |  |
| 04908745 | Bacteria | p__Proteobacteria | c__Alphaproteobacteria | o__Rhizobiales | f__Rhizobiaceae |  |  |
| 4bfd809030d65696d84eb6e8c3c2a2e7 | Bacteria | p__Proteobacteria | c__Gammaproteobacteria | o__Burkholderiales | f__A21b | g__A21b | s__uncultured_beta |
| f965009ac09a60bebf8ca1a6a9aa42e8 | Bacteria | p__Proteobacteria | c__Alphaproteobacteria | o__Rhizobiales | f__Beijerinckiaceae | g__Microvirga |  |
| 62364d9be5a357c628b92833093c9601 | Bacteria | p__Proteobacteria | c__Alphaproteobacteria | o__Rhizobiales | f__Hyphomicrobiaceae | g__Hyphomicrobium |  |
| 9be1918943392a28cd4cfabceecadecc | Bacteria | p__Proteobacteria | c__Gammaproteobacteria | o__Burkholderiales | f__SC-I-84 | g__SC-I-84 |  |
| RS-GCF-007990735.1-NZ-BJWZ01000370.1 | Bacteria | p__Actinobacteriota | c__Actinobacteria | o__Corynebacteriales | f__Nocardiaceae | g__Nocardia |  |
| 04949279 | Bacteria | p__Firmicutes | c__Clostridia | o__Lachnospirales | f__Lachnospiraceae |  |  |
| 02266597 | Bacteria | p__Actinobacteriota | c__Actinobacteria | o__Propionibacteriales | f__Nocardioidaceae | g__Nocardioides | s__Nocardioides_zeae |
| X89934 | Bacteria | p__Actinobacteriota | c__Actinobacteria | o__Streptosporangiales | f__Streptosporangiaceae | g__Streptosporangium |  |
| aa7e7e7f3604fcf1d3e7481b59a67872 | Bacteria | p__Gemmatimonadota | c__Gemmatimonadetes | o__Gemmatimonadales | f__Gemmatimonadaceae | g__uncultured |  |
| cf26e427eed2b005a89b56dd681c7121 | Bacteria | p__Proteobacteria | c__Gammaproteobacteria | o__Burkholderiales | f__B1-7BS | g__B1-7BS |  |
| 3b98049c77967f315def1d2a55f3f06b | Bacteria | p__Actinobacteriota | c__MB-A2-108 | o__MB-A2-108 | f__MB-A2-108 | g__MB-A2-108 |  |
| 74edbf7a774802335c457f78e6023f6b | Bacteria | p__Acidobacteriota | c__Acidobacteriae | o__Acidobacteriales | f__Acidobacteriaceae_(Subgroup_1) | g__Granulicella | s__bacterium_enrichment |
| 30201fed428562e2633d3b98ef5eb2f1 | Bacteria | p__Proteobacteria | c__Gammaproteobacteria | o__Burkholderiales | f__SC-I-84 | g__SC-I-84 |  |
| MJ033-1-barcode36-umi39153bins-ubs-4 | Bacteria | p__Bacteroidota | c__Bacteroidia | o__Chitinophagales | f__Chitinophagaceae | g__Puia | s__uncultured_soil |
| 03177522 | Bacteria | p__Planctomycetota | c__Phycisphaerae | o__Tepidisphaerales | f__WD2101_soil_group | g__WD2101_soil_group |  |
| 1b20da7aecd60a55015400a858686796 | Bacteria | p__Firmicutes | c__Clostridia | o__Peptostreptococcales-Tissierellales | f__Peptostreptococcaceae | g__Peptoclostridium | s__Peptoclostridium_acidaminophilum |
| MJ020-1-barcode31-umi112580bins-ubs-3 | Bacteria | p__Bacteroidota | c__Bacteroidia | o__Cytophagales | f__Microscillaceae | g__uncultured |  |
| 14c138038be71cd0a73f99ad19504277 | Bacteria | p__Bacteroidota | c__Bacteroidia | o__Chitinophagales | f__Chitinophagaceae | g__Terrimonas |  |
| RS-GCF-900110155.1-NZ-FOCS01000073.1 | Bacteria | p__Proteobacteria | c__Alphaproteobacteria | o__Rhizobiales | f__Beijerinckiaceae | g__Methylobacterium-Methylorubrum |  |
| b0c7294f33edb70baeba14cc048c0022 | Bacteria | p__Gemmatimonadota | c__Gemmatimonadetes | o__Gemmatimonadales | f__Gemmatimonadaceae | g__Gemmatimonas |  |
| 04950438 | Bacteria | p__Firmicutes | c__Clostridia | o__Lachnospirales | f__Lachnospiraceae |  |  |
| MJ010-2-barcode69-umi56084bins-ubs-10 | Bacteria | p__Firmicutes | c__Clostridia | o__Clostridia_UCG-014 | f__Clostridia_UCG-014 | g__Clostridia_UCG-014 |  |
| a9ea4bedb12a90ea25dc7e14ec4a5cba | Bacteria | p__Bacteroidota | c__Bacteroidia | o__Bacteroidales | f__Prevotellaceae |  |  |
| 8a02ec4cfc65cfe7aa9f45efed97ba4c | Bacteria | p__Proteobacteria | c__Gammaproteobacteria | o__Burkholderiales | f__Comamonadaceae | g__Comamonas |  |
| 2b4f606bbd8dbdf7665dd49c1b971ff5 | Bacteria | p__Myxococcota | c__bacteriap25 | o__bacteriap25 | f__bacteriap25 | g__bacteriap25 | s__uncultured_Desulfovibrionales |
| 18cf8c60d56bc1825ae66c62191577c3 | Bacteria | p__Acidobacteriota | c__Acidobacteriae | o__Subgroup_2 | f__Subgroup_2 | g__Subgroup_2 | s__metagenome |
| 00225219 | Bacteria | p__Chloroflexi | c__Anaerolineae | o__Anaerolineales | f__Anaerolineaceae | g__UTCFX1 |  |
| AJ315980 | Bacteria | p__Firmicutes | c__Clostridia | o__Oscillospirales | f__Ruminococcaceae | g__Anaerotruncus |  |
| 682c2aba62a437c13def934dcd76ca54 | Bacteria | p__Firmicutes | c__Clostridia | o__Lachnospirales | f__Lachnospiraceae |  |  |
| 05038855 | Bacteria | p__Bacteroidota | c__Bacteroidia | o__Cytophagales | f__Microscillaceae | g__Chryseolinea |  |
| 01831856 | Bacteria | p__Actinobacteriota | c__MB-A2-108 | o__MB-A2-108 | f__MB-A2-108 | g__MB-A2-108 |  |
| 04963990 | Bacteria | p__Firmicutes | c__Bacilli | o__Staphylococcales | f__Staphylococcaceae | g__Staphylococcus |  |
| 7333450fac4e8c750bdf28c3ee87bee1 | Bacteria | p__NB1-j | c__NB1-j | o__NB1-j | f__NB1-j | g__NB1-j | s__uncultured_soil |
| 94a54196ba4a9b66ea03865a569fffb8 | Bacteria | p__Proteobacteria | c__Alphaproteobacteria | o__Rhizobiales | f__Beijerinckiaceae | g__Methylobacterium-Methylorubrum |  |
| 93bf91482e3c90f2d60f17cd42c9aeab | Bacteria | p__Actinobacteriota | c__Actinobacteria | o__Pseudonocardiales | f__Pseudonocardiaceae | g__Saccharopolyspora |  |
| 18316115 | Bacteria | p__Actinobacteriota | c__Actinobacteria | o__Propionibacteriales | f__Nocardioidaceae | g__Nocardioides |  |
| 28dad015ef2d80ee582dc10300759291 | Bacteria | p__Patescibacteria | c__Saccharimonadia | o__Saccharimonadales | f__Saccharimonadaceae | g__TM7a |  |
| 35a66806ff5501af871f6dc848996d22 | Bacteria | p__Bacteroidota | c__Bacteroidia | o__Flavobacteriales | f__Flavobacteriaceae | g__Flavobacterium | s__Flavobacterium_johnsoniae |
| 58bf77b74e8cdc5bbbf911f27181748e | Bacteria | p__Nitrospirota | c__Nitrospiria | o__Nitrospirales | f__Nitrospiraceae | g__Nitrospira |  |
| 16635888 | Bacteria | p__Firmicutes | c__Clostridia | o__Lachnospirales | f__Lachnospiraceae |  |  |
| dc3dafcce1ad64c295157048d0afb318 | Bacteria | p__Proteobacteria | c__Alphaproteobacteria | o__Azospirillales | f__uncultured | g__uncultured | s__uncultured_bacterium |
| 28aa544ea7fd3c33ee120507b94ff73a | Bacteria | p__Proteobacteria | c__Alphaproteobacteria | o__Sphingomonadales | f__Sphingomonadaceae | g__Sphingomonas |  |
| 63fa2891e30c67ec09b8e5c5218f4890 | Bacteria | p__Chloroflexi | c__Ktedonobacteria | o__Ktedonobacterales | f__Ktedonobacteraceae | g__Ktedonobacter | s__Ktedonobacterales_bacterium |
| 607add6546c10d854c06c6730a31b71c | Bacteria | p__Actinobacteriota | c__Actinobacteria | o__Actinomycetales | f__Actinomycetaceae | g__Trueperella | s__uncultured_bacterium |
| MJ020-2-barcode70-umi54668bins-ubs-7 | Bacteria | p__Bacteroidota | c__Bacteroidia | o__Chitinophagales | f__Chitinophagaceae | g__Segetibacter |  |
| 2ec8181489d5fe925c143d7c340fa30a | Bacteria | p__Actinobacteriota | c__Actinobacteria | o__Micrococcales | f__Micrococcaceae |  |  |
| 515a244f21cf7ba0f97ceeb72b696d80 | Bacteria | p__Proteobacteria | c__Alphaproteobacteria | o__Acetobacterales | f__Acetobacteraceae | g__Asaia |  |
| MJ031-1-barcode34-umi9574bins-ubs-50 | Bacteria | p__Firmicutes | c__Clostridia | o__Clostridiales | f__Clostridiaceae | g__Clostridium_sensu_stricto_1 | s__Clostridium_gasigenes |
| 04994057 | Bacteria | p__Proteobacteria | c__Alphaproteobacteria | o__Caulobacterales | f__Caulobacteraceae | g__Caulobacter |  |
| ccb1ce2e273c8dcd66d1956ab0218e8d | Bacteria | p__Firmicutes | c__Bacilli | o__Lactobacillales | f__Enterococcaceae | g__Enterococcus |  |
| 02918808 | Bacteria | p__Planctomycetota | c__Phycisphaerae | o__Tepidisphaerales | f__WD2101_soil_group | g__WD2101_soil_group |  |
| G000431455_1 | Bacteria | p__Proteobacteria | c__Gammaproteobacteria | o__Burkholderiales | f__Sutterellaceae | g__Parasutterella | s__uncultured_bacterium |
| 78d0d682837ce481180ecc3c2064e7cf | Bacteria | p__Bacteroidota | c__Bacteroidia | o__Cytophagales | f__Hymenobacteraceae | g__Hymenobacter | s__Hymenobacter_rubidus |
| 8d0a329191a8a0da708f9be922ea0f29 | Bacteria | p__Proteobacteria | c__Alphaproteobacteria | o__Caulobacterales | f__Caulobacteraceae | g__Caulobacter |  |
| 17968831 | Bacteria | p__Chloroflexi | c__Ktedonobacteria | o__Ktedonobacterales | f__Ktedonobacteraceae | g__HSB_OF53-F07 | s__uncultured_soil |
| 2745696a21c9de360e5e1f1efe709249 | Bacteria | p__Proteobacteria | c__Gammaproteobacteria | o__Enterobacterales | f__Enterobacteriaceae |  |  |
| e88a1ef7a4eae6943f91f090a401c41e | Bacteria | p__Bacteroidota | c__Bacteroidia | o__Cytophagales | f__Hymenobacteraceae | g__Hymenobacter |  |
| 9cd33d78bf463e87328b92b47f0802bd | Bacteria | p__RCP2-54 | c__RCP2-54 | o__RCP2-54 | f__RCP2-54 | g__RCP2-54 |  |
| 17409597 | Bacteria | p__Actinobacteriota | c__Thermoleophilia | o__Gaiellales | f__Gaiellaceae | g__Gaiella |  |
| 19950740 | Bacteria | p__Firmicutes | c__Clostridia | o__Oscillospirales | f__Oscillospiraceae | g__Intestinimonas |  |
| 2a7acc7343333896720870f4283a64cd | Bacteria | p__Proteobacteria | c__Alphaproteobacteria | o__Rhizobiales | f__Rhizobiaceae |  |  |
| 975bc7de21a571f357d03c8b1cd4928e | Bacteria | p__Actinobacteriota | c__Thermoleophilia | o__Gaiellales | f__uncultured | g__uncultured |  |
| e0feeb646f77f6f28cb5bf2efd194e54 | Bacteria | p__Chloroflexi | c__Anaerolineae | o__RBG-13-54-9 | f__RBG-13-54-9 | g__RBG-13-54-9 |  |
| 05036924 | Bacteria | p__Firmicutes | c__Bacilli | o__Bacillales | f__Planococcaceae |  |  |
| 6eac5328163622d5c3bdfff806997c22 | Bacteria | p__Bacteroidota | c__Bacteroidia | o__Sphingobacteriales | f__AKYH767 | g__AKYH767 |  |
| MF153474 | Bacteria | p__Proteobacteria | c__Gammaproteobacteria | o__Xanthomonadales | f__Xanthomonadaceae | g__Lysobacter |  |
| RS-GCF-002891015.1-NZ-POUK01000011.1 | Bacteria | p__Proteobacteria | c__Gammaproteobacteria | o__Pseudomonadales | f__Pseudomonadaceae | g__Pseudomonas |  |
| 04948814 | Bacteria | p__Firmicutes | c__Clostridia | o__Lachnospirales | f__Lachnospiraceae | g__uncultured |  |
| RS-GCF-003461005.1-NZ-QTWG01000138.1 | Bacteria | p__Firmicutes | c__Clostridia | o__Lachnospirales | f__Lachnospiraceae | g__[Ruminococcus]_torques_group |  |
| 17cbabe6202c6e336f188bf35509d745 | Bacteria | p__Myxococcota | c__Polyangia | o__Polyangiales |  |  |  |
| 1955a9e3f52516044d9ef9905cff4cfa | Bacteria | p__Bacteroidota | c__Bacteroidia | o__Bacteroidales | f__Prevotellaceae | g__Prevotella |  |
| 04666211 | Bacteria | p__Proteobacteria | c__Gammaproteobacteria | o__Pseudomonadales | f__Pseudomonadaceae | g__Pseudomonas |  |
| 05032828 | Bacteria | p__Actinobacteriota | c__Actinobacteria | o__Corynebacteriales | f__Nocardiaceae | g__Gordonia |  |
| 2bee91524636fb36144f3d713a934bd2 | Bacteria | p__Bacteroidota | c__Bacteroidia | o__Bacteroidales | f__Bacteroidetes_vadinHA17 | g__Bacteroidetes_vadinHA17 |  |
| 70ac6161d64935382902b566f73ecc92 | Bacteria | p__Proteobacteria | c__Alphaproteobacteria | o__Sphingomonadales | f__Sphingomonadaceae | g__Sphingobium |  |
| 16132039 | Bacteria | p__Chloroflexi | c__Chloroflexia | o__Thermomicrobiales | f__JG30-KF-CM45 | g__JG30-KF-CM45 |  |
| 7edb60c9b2920159299191c81ea3366f | Bacteria | p__Proteobacteria | c__Alphaproteobacteria | o__Rhodobacterales | f__Rhodobacteraceae |  |  |
| 07210849 | Bacteria | p__Acidobacteriota | c__Vicinamibacteria | o__Vicinamibacterales | f__Vicinamibacteraceae | g__Vicinamibacteraceae |  |
| ecb4e4c78a79da408b05c845598b97b4 | Bacteria | p__Proteobacteria | c__Alphaproteobacteria | o__Sphingomonadales | f__Sphingomonadaceae |  |  |
| 4c9fe068b9940a12e3e2feb985f6f601 | Bacteria | p__Proteobacteria | c__Gammaproteobacteria | o__Pseudomonadales | f__Moraxellaceae | g__Acinetobacter |  |
| eb4f29b7c614c15661c527428010db34 | Bacteria | p__Firmicutes | c__Clostridia | o__Lachnospirales | f__Lachnospiraceae | g__Lachnospiraceae_NK4A136_group |  |
| dde6ceec5143a58f58fe7c5826791d3f | Bacteria | p__Chloroflexi | c__Gitt-GS-136 | o__Gitt-GS-136 | f__Gitt-GS-136 | g__Gitt-GS-136 | s__uncultured_bacterium |
| 02922891 | Bacteria | p__Planctomycetota | c__Phycisphaerae | o__Tepidisphaerales | f__WD2101_soil_group | g__WD2101_soil_group |  |
| 1238f937f6600ba77d3151849affd7f6 | Bacteria | p__Acidobacteriota | c__Vicinamibacteria | o__Vicinamibacterales | f__Vicinamibacteraceae | g__Vicinamibacteraceae |  |
| 06ed368c2c2a118f9029b2ada79b1097 | Bacteria | p__Acidobacteriota | c__Acidobacteriae | o__Bryobacterales | f__Bryobacteraceae | g__Bryobacter |  |
| G000463665_1 | Bacteria | p__Actinobacteriota | c__Actinobacteria | o__Propionibacteriales | f__Propionibacteriaceae | g__Cutibacterium | s__Cutibacterium_granulosum |
| MJ033-1-barcode36-umi18232bins-ubs-15 | Bacteria | p__Proteobacteria | c__Gammaproteobacteria | o__Steroidobacterales | f__Steroidobacteraceae | g__Steroidobacter |  |
| RS-GCF-000179935.1-NZ-AEKH01000023.1 | Bacteria | p__Firmicutes | c__Bacilli | o__Lactobacillales | f__Lactobacillaceae | g__Lactobacillus | s__Lactobacillus_iners |
| 7811d451db9e052512cc1a868491b312 | Bacteria | p__Proteobacteria | c__Gammaproteobacteria | o__Enterobacterales | f__Enterobacteriaceae |  |  |
| cbb5ae4325ce35ce291cab30d86358f9 | Bacteria | p__Chloroflexi | c__Anaerolineae | o__Anaerolineales | f__Anaerolineaceae | g__uncultured | s__uncultured_Chloroflexi |
| RS-GCF-001461705.1-NZ-AUTA01000035.1 | Bacteria | p__Proteobacteria | c__Alphaproteobacteria | o__Rhizobiales | f__Rhizobiaceae |  |  |
| GB-GCA-002364745.1-DEZG01000009.1 | Bacteria | p__Proteobacteria | c__Alphaproteobacteria | o__Rhizobiales | f__Rhizobiaceae | g__Shinella |  |
| e2e0cd00bbb2cb68d652d6d6b5745193 | Bacteria | p__Cyanobacteria | c__Cyanobacteriia | o__Cyanobacteriales |  |  |  |
| 5a00435a99489e181e565101f08a1ccf | Bacteria | p__Methylomirabilota | c__Methylomirabilia | o__Rokubacteriales | f__Rokubacteriales | g__Rokubacteriales |  |
| GB-GCA-013816855.1-JACDDT010000121.1 | Bacteria | p__Gemmatimonadota | c__Gemmatimonadetes | o__Gemmatimonadales | f__Gemmatimonadaceae | g__uncultured |  |
| 17970018 | Bacteria | p__Chloroflexi | c__Ktedonobacteria | o__Ktedonobacterales | f__Ktedonobacteraceae | g__HSB_OF53-F07 | s__uncultured_bacterium |
| RS-GCF-006094275.1-NZ-CP034412.1 | Bacteria | p__Actinobacteriota | c__Actinobacteria | o__Micrococcales | f__Micrococcaceae |  |  |
| fc607f7f6ace7a3b455cbbd8e8c84fe4 | Bacteria | p__Verrucomicrobiota | c__Verrucomicrobiae | o__Chthoniobacterales | f__Chthoniobacteraceae | g__Chthoniobacter | s__uncultured_bacterium |
| MJ020-1-barcode31-umi22614bins-ubs-20 | Bacteria | p__Proteobacteria | c__Gammaproteobacteria | o__Burkholderiales | f__Comamonadaceae |  |  |
| 05097524 | Bacteria | p__Proteobacteria | c__Alphaproteobacteria | o__Sphingomonadales | f__Sphingomonadaceae | g__Sphingomonas |  |
| G004168625_1 | Bacteria | p__Actinobacteriota | c__Actinobacteria | o__Micrococcales |  |  |  |
| b0fae4535743c4a6b71a2ab2f874b607 | Bacteria | p__Acidobacteriota | c__Acidobacteriae | o__Subgroup_2 | f__Subgroup_2 | g__Subgroup_2 |  |
| 612e7c02d351dfbbd09092c989f52c59 | Bacteria | p__Proteobacteria | c__Alphaproteobacteria | o__Dongiales | f__Dongiaceae | g__Dongia |  |
| 14800994 | Bacteria | p__Chloroflexi | c__Ktedonobacteria | o__Ktedonobacterales | f__JG30-KF-AS9 | g__JG30-KF-AS9 | s__uncultured_bacterium |
| 04957563 | Bacteria | p__Proteobacteria | c__Gammaproteobacteria | o__Burkholderiales | f__Oxalobacteraceae | g__Herbaspirillum |  |
| RS-GCF-003474435.1-NZ-QRNF01000126.1 | Bacteria | p__Firmicutes | c__Clostridia | o__Lachnospirales | f__Lachnospiraceae | g__Blautia |  |
| 35c77ec60fe697185d7bdb832f46c971 | Bacteria | p__Acidobacteriota | c__Vicinamibacteria | o__Vicinamibacterales | f__Vicinamibacteraceae | g__Vicinamibacteraceae |  |
| 03538657 | Bacteria | p__Planctomycetota | c__Planctomycetes | o__Gemmatales | f__Gemmataceae | g__uncultured |  |
| MJ006-1-barcode48-umi100463bins-ubs-5 | Bacteria | p__Proteobacteria | c__Gammaproteobacteria | o__Burkholderiales | f__Sutterellaceae | g__Parasutterella | s__Burkholderiales_bacterium |
| 32ce57047fc0d1b8cadbd5dbd52ae9a7 | Bacteria | p__Gemmatimonadota | c__Gemmatimonadetes | o__Gemmatimonadales | f__Gemmatimonadaceae | g__uncultured | s__Gemmatimonas_sp. |
| 568b7b65d95975e905955dc75c148140 | Bacteria | p__Bacteroidota | c__Bacteroidia | o__Flavobacteriales | f__Flavobacteriaceae | g__Flavobacterium | s__Flavobacterium_ceti |
| MJ007-1-barcode25-umi23028bins-ubs-9 | Bacteria | p__Proteobacteria | c__Alphaproteobacteria | o__Rhizobiales | f__Rhizobiales_Incertae_Sedis | g__uncultured |  |
| c7c6e220a8257f6f72eff4764478c85f | Bacteria | p__Proteobacteria | c__Gammaproteobacteria | o__Burkholderiales | f__Oxalobacteraceae |  |  |
| 08635268 | Bacteria | p__Proteobacteria | c__Alphaproteobacteria | o__Rhizobiales | f__Devosiaceae | g__Devosia |  |
| 39a0f44cbedccff7b6fc202ee37e8cf1 | Bacteria | p__Chloroflexi | c__Chloroflexia | o__Thermomicrobiales | f__JG30-KF-CM45 | g__JG30-KF-CM45 |  |
| ceea84ebf48c8078434523d679b6d12a | Bacteria | p__Bacteroidota | c__Bacteroidia | o__Chitinophagales | f__Chitinophagaceae | g__Flavihumibacter |  |
| 02359427 | Bacteria | p__Actinobacteriota | c__Actinobacteria | o__Frankiales | f__Geodermatophilaceae | g__Modestobacter |  |
| MJ031-1-barcode42-umi5821bins-ubs-77 | Bacteria | p__Acidobacteriota | c__Blastocatellia | o__Blastocatellales | f__Blastocatellaceae |  |  |
| 05007913 | Bacteria | p__Proteobacteria | c__Alphaproteobacteria | o__Sphingomonadales | f__Sphingomonadaceae | g__Sphingomonas |  |
| AJ000684 | Bacteria | p__Actinobacteriota | c__Actinobacteria | o__Corynebacteriales | f__Mycobacteriaceae | g__Mycobacterium |  |
| 604c57358ca79e1b760e0efba558b398 | Bacteria | p__Bacteroidota | c__Bacteroidia | o__Chitinophagales | f__Saprospiraceae | g__uncultured |  |
| 02247870 | Bacteria | p__Proteobacteria | c__Alphaproteobacteria | o__Rhizobiales | f__Beijerinckiaceae | g__Microvirga |  |
| fd368450d201a19b5de82b92a86269fb | Bacteria | p__Proteobacteria | c__Alphaproteobacteria | o__Rhizobiales | f__Rhizobiaceae |  |  |
| GB-GCA-007095845.1-SFBI01000217.1 | Bacteria | p__Proteobacteria | c__Alphaproteobacteria | o__Rhodobacterales | f__Rhodobacteraceae | g__Rubellimicrobium |  |
| MJ005-barcode33-umi12779bins-ubs-15 | Bacteria | p__Firmicutes | c__Clostridia | o__Oscillospirales | f__Ruminococcaceae | g__Ruminococcus | s__Ruminococcus_bicirculans |
| 14334220 | Bacteria | p__Acidobacteriota | c__Vicinamibacteria | o__Vicinamibacterales | f__uncultured | g__uncultured | s__uncultured_Acidobacteriales |
| 100ac61a1dcaaae011ca2107c3a03fff | Bacteria | p__Actinobacteriota | c__Actinobacteria | o__Micrococcales | f__Micrococcaceae |  |  |
| cea62dcccac3b3f397c989ecb36f1b5f | Bacteria | p__Acidobacteriota | c__Thermoanaerobaculia | o__Thermoanaerobaculales | f__Thermoanaerobaculaceae | g__Subgroup_10 |  |
| 04958387 | Bacteria | p__Actinobacteriota | c__Actinobacteria | o__Micrococcales | f__Microbacteriaceae |  |  |
| 78dd410bc859b7fbba531ec8194c0fd4 | Bacteria | p__Acidobacteriota | c__Vicinamibacteria | o__Vicinamibacterales | f__uncultured | g__uncultured |  |
| 04966025 | Bacteria | p__Firmicutes | c__Bacilli | o__Bacillales | f__Bacillaceae | g__Bacillus |  |
| 55ffb2f3390cf082b540969c47bd714f | Bacteria | p__Bacteroidota | c__Bacteroidia | o__Bacteroidales | f__Bacteroidetes_vadinHA17 | g__Bacteroidetes_vadinHA17 |  |
| LC438515 | Bacteria | p__Firmicutes | c__Bacilli | o__Lactobacillales | f__Enterococcaceae | g__Enterococcus |  |
| 04905824 | Bacteria | p__Proteobacteria | c__Alphaproteobacteria | o__Acetobacterales | f__Acetobacteraceae | g__Roseomonas |  |
| 20934361 | Bacteria | p__Firmicutes | c__Bacilli | o__Erysipelotrichales | f__Erysipelatoclostridiaceae | g__Erysipelatoclostridium | s__unidentified |
| 57880da51ed08068a0ce2f95403f2773 | Bacteria | p__Actinobacteriota | c__Thermoleophilia | o__Gaiellales | f__Gaiellaceae | g__Gaiella |  |
| G003865405_1 | Bacteria | p__Bacteroidota | c__Bacteroidia | o__Flavobacteriales | f__Flavobacteriaceae | g__Flavobacterium |  |
| EU305672 | Bacteria | p__Actinobacteriota | c__Actinobacteria | o__Micrococcales | f__Micrococcaceae |  |  |
| 02360463 | Bacteria | p__Proteobacteria | c__Alphaproteobacteria | o__Rhizobiales | f__Beijerinckiaceae | g__Methylobacterium-Methylorubrum |  |
| MJ032-1-barcode26-umi63728bins-ubs-4 | Bacteria | p__Proteobacteria | c__Gammaproteobacteria | o__Burkholderiales | f__TRA3-20 | g__TRA3-20 |  |
| 04941365 | Bacteria | p__Acidobacteriota | c__Vicinamibacteria | o__Vicinamibacterales | f__uncultured | g__uncultured |  |
| 17388157 | Bacteria | p__Chloroflexi | c__Ktedonobacteria | o__Ktedonobacterales | f__Ktedonobacteraceae |  |  |
| b689791f1119fc937a4d804c031923cf | Bacteria | p__Proteobacteria | c__Gammaproteobacteria | o__Burkholderiales | f__Comamonadaceae | g__Acidovorax |  |
| GB-GCA-013823855.1-PNMW01000007.1 | Bacteria | p__Proteobacteria | c__Gammaproteobacteria | o__Salinisphaerales | f__Solimonadaceae | g__Nevskia |  |
| c36d7c9f78e543dd15cb06200ebb26ff | Bacteria | p__Firmicutes | c__Bacilli | o__Paenibacillales | f__Paenibacillaceae | g__Paenibacillus |  |
| be0a14f22f934fe4c174d8e51f20ceac | Bacteria | p__Bacteroidota | c__Bacteroidia | o__Chitinophagales | f__Saprospiraceae | g__uncultured |  |
| 4a8ddabe33f73ee343eeeca1f1f70562 | Bacteria | p__Planctomycetota | c__Phycisphaerae | o__Tepidisphaerales | f__WD2101_soil_group | g__WD2101_soil_group | s__uncultured_bacterium |
| ad3d41e3490e0dc9f7077e6647069b65 | Bacteria | p__Firmicutes | c__Bacilli | o__Bacillales | f__Bacillaceae | g__Bacillus |  |
| 01696535 | Bacteria | p__Proteobacteria | c__Alphaproteobacteria | o__Caulobacterales | f__Caulobacteraceae | g__Brevundimonas |  |
| RS-GCF-001578585.1-NZ-KQ968490.1 | Bacteria | p__Proteobacteria | c__Gammaproteobacteria | o__Burkholderiales | f__Sutterellaceae | g__Sutterella |  |
| 53316792bb87b8b1b6844a7cc9733dec | Bacteria | p__Proteobacteria | c__Gammaproteobacteria | o__Burkholderiales | f__Methylophilaceae | g__Methylotenera |  |
| 0ce53790291d6339b76bbf542f8c091a | Bacteria | p__Actinobacteriota | c__Actinobacteria |  |  |  |  |
| c8fc8663c6b5f491c8f171e61f87cfcb | Bacteria | p__Firmicutes | c__Bacilli | o__Lactobacillales | f__Enterococcaceae | g__Enterococcus |  |
| 15439332 | Bacteria | p__Firmicutes | c__Clostridia | o__Oscillospirales | f__Ruminococcaceae | g__Incertae_Sedis | s__uncultured_bacterium |
| dd60dcedb30a8a7a3c7161cd7c3241dc | Bacteria | p__Proteobacteria | c__Alphaproteobacteria | o__Rhizobiales | f__Beijerinckiaceae | g__Methylobacterium-Methylorubrum |  |
| a42a1817bf6488d7c6a0bc71811c27b7 | Bacteria | p__Proteobacteria | c__Gammaproteobacteria | o__JG36-TzT-191 | f__JG36-TzT-191 | g__JG36-TzT-191 | s__uncultured_proteobacterium |
| 0d3d5a725a92008b38ee21a80897223a | Bacteria | p__Planctomycetota | c__Planctomycetes | o__Isosphaerales | f__Isosphaeraceae |  |  |
| 4ed1fba064182275dd1f274a723750da | Bacteria | p__Acidobacteriota | c__Acidobacteriae | o__Acidobacteriales | f__uncultured | g__uncultured |  |
| 04996448 | Bacteria | p__Proteobacteria | c__Gammaproteobacteria | o__Xanthomonadales | f__Xanthomonadaceae | g__Pseudoxanthomonas |  |
| 43548bd43713d4706e0c43ab4b0a178e | Bacteria | p__Proteobacteria | c__Gammaproteobacteria | o__Burkholderiales | f__Nitrosomonadaceae | g__mle1-7 |  |
| 1b0fb7da32f1ced1af2e28d317d1c50b | Bacteria | p__Proteobacteria | c__Gammaproteobacteria | o__Pseudomonadales | f__Moraxellaceae | g__Acinetobacter |  |
| 9e72e2b9cb725c2aab6fd8b8c02fe571 | Bacteria | p__Proteobacteria | c__Alphaproteobacteria | o__Sphingomonadales | f__Sphingomonadaceae | g__Sphingopyxis |  |
| 152fe49591e32afd0e329039fd1fe606 | Bacteria | p__Proteobacteria | c__Gammaproteobacteria | o__Burkholderiales | f__Burkholderiaceae | g__Burkholderia-Caballeronia-Paraburkholderia |  |
| MJ010-1-barcode26-umi132805bins-ubs-5 | Bacteria | p__Firmicutes | c__Bacilli | o__Paenibacillales | f__Paenibacillaceae | g__Paenibacillus |  |
| GB-GCA-015260565.1-JABZWK010000152.1 | Bacteria | p__Proteobacteria | c__Gammaproteobacteria | o__Pasteurellales | f__Pasteurellaceae | g__Haemophilus |  |
| RS-GCF-013301525.1-NZ-JAAIOL010000051.1 | Bacteria | p__Firmicutes | c__Clostridia | o__Lachnospirales | f__Lachnospiraceae | g__Coprococcus |  |
| b9585c1c1de2e33990921eb8a2095f8d | Bacteria | p__Proteobacteria | c__Gammaproteobacteria | o__Pseudomonadales | f__Moraxellaceae | g__Acinetobacter |  |
| 921cd285eb52d93d3067a27d0a1c7138 | Bacteria |  |  |  |  |  |  |
| 38640e88f5cda1970e4bd9a5fe0de845 | Bacteria | p__Firmicutes | c__Bacilli | o__Lactobacillales | f__Enterococcaceae | g__Enterococcus |  |
| d0b30f9d561b5e4e183fe217b005b5c6 | Bacteria | p__Acidobacteriota | c__Acidobacteriae | o__Subgroup_2 | f__Subgroup_2 | g__Subgroup_2 |  |
| G000152705_2 | Bacteria | p__Actinobacteriota | c__Actinobacteria | o__Micrococcales | f__Intrasporangiaceae |  |  |
| 1d5a7301d483a13f14a17e07cd339a6a | Bacteria | p__Proteobacteria | c__Gammaproteobacteria | o__Cellvibrionales | f__Cellvibrionaceae | g__Cellvibrio |  |
| a392acc2c2fcd3ec84c5d06384beab44 | Bacteria | p__Firmicutes | c__Bacilli | o__Lactobacillales | f__Lactobacillaceae | g__Lactobacillus | s__Lactobacillus_brevis |
| 42ba0d900d6c98c0ba955db61946bc78 | Bacteria | p__Proteobacteria | c__Alphaproteobacteria | o__Micropepsales | f__Micropepsaceae | g__uncultured | s__uncultured_Hyphomicrobiaceae |
| ab142d7f6aa95a1785d528f510c50160 | Bacteria | p__Bacteroidota | c__Bacteroidia | o__Bacteroidales | f__Muribaculaceae | g__Muribaculaceae |  |
| 05025451 | Bacteria | p__Firmicutes | c__Clostridia | o__Lachnospirales | f__Lachnospiraceae |  |  |
| RS-GCF-001662715.1-NZ-LULD01000035.1 | Bacteria | p__Proteobacteria | c__Gammaproteobacteria | o__Enterobacterales | f__Enterobacteriaceae |  |  |
| 52942f0c30c4b6b22a39f38a8165b802 | Bacteria | p__Actinobacteriota | c__Acidimicrobiia | o__Microtrichales | f__Microtrichaceae | g__uncultured |  |
| 724b98bbb3734c37c35110084507d7f3 | Bacteria | p__Proteobacteria | c__Gammaproteobacteria | o__Burkholderiales | f__Oxalobacteraceae | g__Massilia |  |
| 3264ceb67f909c85519d2ce939d15934 | Bacteria | p__Proteobacteria | c__Gammaproteobacteria |  |  |  |  |
| 6282e46850eba54bb155a05008292181 | Bacteria | p__Proteobacteria | c__Gammaproteobacteria | o__Burkholderiales | f__Burkholderiaceae | g__Burkholderia-Caballeronia-Paraburkholderia |  |
| MJ030-2-barcode65-umi11431bins-ubs-42 | Bacteria | p__Bacteroidota | c__Bacteroidia | o__Chitinophagales | f__Chitinophagaceae |  |  |
| 28933500c7b493166363f2ae5dabf806 | Bacteria | p__Proteobacteria | c__Alphaproteobacteria | o__Caulobacterales | f__Caulobacteraceae | g__Caulobacter |  |
| 04968527 | Bacteria | p__Bacteroidota | c__Bacteroidia | o__Cytophagales | f__Microscillaceae | g__Chryseolinea |  |
| 9de509399bcc1dab3da76ca1a3d71986 | Bacteria | p__Myxococcota | c__Polyangia | o__Polyangiales | f__Polyangiaceae | g__Pajaroellobacter |  |
| 04924058 | Bacteria | p__Bacteroidota | c__Bacteroidia | o__Bacteroidales | f__Muribaculaceae | g__Muribaculum | s__uncultured_bacterium |
| RS-GCF-003470215.1-NZ-QSIS01000045.1 | Bacteria | p__Firmicutes | c__Clostridia | o__Lachnospirales | f__Lachnospiraceae | g__[Eubacterium]_eligens_group |  |
| CP002122 | Bacteria | p__Bacteroidota | c__Bacteroidia | o__Bacteroidales | f__Prevotellaceae | g__Prevotella | s__Prevotella_melaninogenica |
| e7fd655d49c8115f0c7260e360d6c394 | Bacteria | p__Firmicutes | c__Bacilli | o__Lactobacillales | f__Lactobacillaceae | g__Lactobacillus |  |
| RS-GCF-001807485.1-NZ-KV789177.1 | Bacteria | p__Actinobacteriota | c__Actinobacteria | o__Corynebacteriales | f__Corynebacteriaceae | g__Corynebacterium |  |
| MJ010-1-barcode29-umi148842bins-ubs-3 | Bacteria | p__Proteobacteria | c__Gammaproteobacteria | o__Pseudomonadales | f__Moraxellaceae | g__Acinetobacter |  |
| e746e5c8ea5b70dd4208693e41d37104 | Bacteria | p__Myxococcota | c__Polyangia | o__Haliangiales | f__Haliangiaceae | g__Haliangium |  |
| 6fcd02283d81e544c9bcefecdeabae37 | Bacteria | p__Actinobacteriota | c__Actinobacteria | o__Micrococcales |  |  |  |
| 13703222 | Bacteria | p__Gemmatimonadota | c__Gemmatimonadetes | o__Gemmatimonadales | f__Gemmatimonadaceae | g__Gemmatimonas |  |
| 38071818f4e97c04c505734f063243a3 | Bacteria | p__Firmicutes | c__Clostridia | o__Lachnospirales | f__Lachnospiraceae |  |  |
| 05009300 | Bacteria | p__Proteobacteria | c__Alphaproteobacteria | o__Sphingomonadales | f__Sphingomonadaceae | g__Sphingobium |  |
| f057795fb339608118839c3739183fdb | Bacteria | p__Myxococcota | c__Myxococcia | o__Myxococcales | f__Myxococcaceae |  |  |
| ff678da90f1d7e805484c1dc487c5ca9 | Bacteria | p__Acidobacteriota | c__Holophagae | o__Subgroup_7 | f__Subgroup_7 | g__Subgroup_7 |  |
| 0b2a248c9c310dfe619112b906dc1382 | Bacteria | p__Actinobacteriota | c__Actinobacteria | o__Micromonosporales | f__Micromonosporaceae | g__Hamadaea |  |
| 7ade67199e77dd7c9df72d203832939d | Bacteria | p__Actinobacteriota | c__Actinobacteria | o__Corynebacteriales | f__Corynebacteriaceae | g__Corynebacterium |  |
| 04959245 | Bacteria | p__Proteobacteria | c__Gammaproteobacteria | o__Burkholderiales | f__TRA3-20 | g__TRA3-20 |  |
| AM501435 | Bacteria | p__Proteobacteria | c__Gammaproteobacteria | o__Burkholderiales | f__Comamonadaceae | g__Pelomonas |  |
| 05024852 | Bacteria | p__Firmicutes | c__Clostridia | o__Lachnospirales | f__Lachnospiraceae |  |  |
| 0f4b32580e9b3e270b47db4e5d602ce9 | Bacteria | p__Bacteroidota | c__Bacteroidia | o__Bacteroidales | f__Muribaculaceae | g__Muribaculaceae | s__uncultured_bacterium |
| 15c2455ff59540db6a5bf5011a8c872b | Bacteria | p__Proteobacteria | c__Gammaproteobacteria | o__Enterobacterales |  |  |  |
| efae3620741a3800d8bef3691ad0dd3e | Bacteria | p__Proteobacteria | c__Gammaproteobacteria | o__Burkholderiales | f__TRA3-20 | g__TRA3-20 |  |
| 02245486 | Bacteria | p__Actinobacteriota | c__Actinobacteria | o__Micrococcales | f__Brevibacteriaceae | g__Brevibacterium |  |
| aeef6d3a0c4b2d59f3b35ad0b5cf1251 | Bacteria | p__Chloroflexi | c__TK10 | o__TK10 | f__TK10 | g__TK10 | s__uncultured_Dehalococcoidia |
| MJ031-1-barcode30-umi11682bins-ubs-39 | Bacteria | p__Bacteroidota | c__Bacteroidia | o__Cytophagales | f__Microscillaceae |  |  |
| d0f0bec2f436fc8b2d0635cd789de672 | Bacteria | p__Actinobacteriota | c__Thermoleophilia | o__Gaiellales | f__Gaiellaceae | g__Gaiella |  |
| 41ceebcf85213b7293c0053f4a696df7 | Bacteria | p__Actinobacteriota | c__Acidimicrobiia | o__Microtrichales | f__Microtrichaceae | g__uncultured |  |
| 356ab2f167c5f2830ad9bb569c7c8e74 | Bacteria | p__Proteobacteria | c__Alphaproteobacteria | o__Rhodobacterales | f__Rhodobacteraceae |  |  |
| RS-GCF-000407145.1-NZ-KE136478.1 | Bacteria | p__Firmicutes | c__Bacilli | o__Lactobacillales | f__Enterococcaceae | g__Enterococcus |  |
| MJ031-2-barcode61-umi15903bins-ubs-30 | Bacteria | p__Bacteroidota | c__Bacteroidia | o__Chitinophagales | f__Chitinophagaceae | g__Flavisolibacter |  |
| 04993169 | Bacteria | p__Proteobacteria | c__Alphaproteobacteria | o__Rhizobiales | f__Beijerinckiaceae | g__Bosea |  |
| 19707094 | Bacteria | p__Firmicutes | c__Bacilli | o__Erysipelotrichales | f__Erysipelotrichaceae | g__Ileibacterium | s__Ileibacterium_valens |
| 13702559 | Bacteria | p__Gemmatimonadota | c__Gemmatimonadetes | o__Gemmatimonadales | f__Gemmatimonadaceae |  |  |
| 71c19fddbfa80e00e55077be8e40a3f0 | Bacteria | p__Actinobacteriota | c__Acidimicrobiia | o__Microtrichales | f__uncultured | g__uncultured |  |
| HQ839787 | Bacteria | p__Actinobacteriota | c__Actinobacteria | o__Propionibacteriales | f__Propionibacteriaceae | g__Friedmanniella |  |
| 4891ae4322acd033f38576a30105e41f | Bacteria | p__Proteobacteria | c__Gammaproteobacteria | o__Burkholderiales | f__Oxalobacteraceae |  |  |
| RS-GCF-003432145.1-NZ-CP031789.1--4 | Bacteria | p__Proteobacteria | c__Gammaproteobacteria | o__Enterobacterales | f__Enterobacteriaceae |  |  |
| bff406c37fa69152b8cbba59b886139d | Bacteria | p__Proteobacteria | c__Alphaproteobacteria | o__Rhizobiales | f__Xanthobacteraceae | g__uncultured |  |
| AM942444 | Bacteria | p__Actinobacteriota | c__Actinobacteria | o__Corynebacteriales | f__Corynebacteriaceae | g__Corynebacterium | s__Corynebacterium_urealyticum |
| 5fb75920a7bf995402fb3609a19412d2 | Bacteria | p__Acidobacteriota | c__Vicinamibacteria | o__Vicinamibacterales | f__Vicinamibacteraceae | g__Vicinamibacteraceae | s__uncultured_Saccharibacillus |
| RS-GCF-000738175.1-NZ-JFCH01000010.1 | Bacteria | p__Actinobacteriota | c__Actinobacteria | o__Corynebacteriales | f__Corynebacteriaceae | g__Corynebacterium |  |
| 04957239 | Bacteria | p__Proteobacteria | c__Gammaproteobacteria | o__Burkholderiales | f__Comamonadaceae |  |  |
| 89a7eb4ff93c537783706802f9b5fb42 | Bacteria | p__Proteobacteria | c__Gammaproteobacteria | o__Burkholderiales | f__Comamonadaceae | g__Delftia |  |
| dad4242d4ea38f93eb6472bc273a8ad7 | Bacteria | p__Firmicutes | c__Bacilli | o__Erysipelotrichales | f__Erysipelotrichaceae | g__Turicibacter | s__uncultured_bacterium |
| a43aa375c793d594dde1f789354c6294 | Bacteria | p__Proteobacteria | c__Gammaproteobacteria | o__Burkholderiales | f__Alcaligenaceae | g__Alcaligenes |  |
| f5b6f20369472675f45fcdd24b7f129b | Bacteria | p__Verrucomicrobiota | c__Chlamydiae | o__Chlamydiales | f__Simkaniaceae | g__uncultured |  |
| 5135d20df7d3be5c16b6de4d86f1e3e5 | Bacteria | p__Bacteroidota | c__Bacteroidia | o__Chitinophagales | f__Saprospiraceae | g__uncultured |  |
| U49757 | Bacteria | p__Proteobacteria | c__Gammaproteobacteria | o__Burkholderiales | f__Oxalobacteraceae | g__Oxalobacter | s__Oxalobacter_formigenes |
| 04934706 | Bacteria | p__Bacteroidota | c__Bacteroidia | o__Flavobacteriales | f__Weeksellaceae | g__Chryseobacterium |  |
| 2cf6b31a15451bb9e6b1a23a57dff7e7 | Bacteria | p__Actinobacteriota | c__Acidimicrobiia | o__Microtrichales | f__Iamiaceae | g__Iamia |  |
| c2c3f6b3eb7ecf1c55fac0f928c1d911 | Bacteria | p__Proteobacteria | c__Alphaproteobacteria | o__Rhodospirillales | f__uncultured | g__uncultured | s__unidentified_marine |
| 04989800 | Bacteria | p__Proteobacteria | c__Gammaproteobacteria | o__Pseudomonadales | f__Moraxellaceae | g__Acinetobacter |  |
| 20d15ccef8de9af18361c38b1abb6b36 | Bacteria | p__Actinobacteriota | c__Actinobacteria | o__Micrococcales | f__Dermabacteraceae | g__Brachybacterium |  |
| 1768ae4da6f20bb8a94f7ee48537ca5d | Bacteria | p__Planctomycetota | c__Planctomycetes | o__Pirellulales | f__Pirellulaceae | g__uncultured |  |
| c1c113b203315f314182400f1e10a8fc | Bacteria | p__Bdellovibrionota | c__Bdellovibrionia | o__Bacteriovoracales | f__Bacteriovoracaceae | g__Peredibacter |  |
| 608d22512e3b00f4df790909a103d461 | Bacteria | p__Acidobacteriota | c__Thermoanaerobaculia | o__Thermoanaerobaculales | f__Thermoanaerobaculaceae | g__Subgroup_10 | s__metagenome |
| 04952624 | Bacteria | p__Actinobacteriota | c__Thermoleophilia | o__Gaiellales | f__Gaiellaceae | g__Gaiella | s__actinobacterium_WWH12 |
| 07202174 | Bacteria | p__Acidobacteriota | c__Vicinamibacteria | o__Vicinamibacterales | f__Vicinamibacteraceae | g__Vicinamibacteraceae |  |
| 02354729 | Bacteria | p__Actinobacteriota | c__Actinobacteria | o__Micrococcales | f__Microbacteriaceae |  |  |
| 04954946 | Bacteria | p__Firmicutes | c__Bacilli | o__Bacillales | f__Bacillaceae | g__Geobacillus |  |
| 11844473 | Bacteria | p__Proteobacteria | c__Alphaproteobacteria | o__Sphingomonadales | f__Sphingomonadaceae | g__Sphingomonas |  |
| MJ010-2-barcode71-umi180220bins-ubs-3 | Bacteria | p__Bacteroidota | c__Bacteroidia | o__Flavobacteriales | f__Flavobacteriaceae | g__Flavobacterium |  |
| a046eb43e4022bb2316a7b5bcccccb71 | Bacteria | p__Actinobacteriota | c__Acidimicrobiia | o__IMCC26256 | f__IMCC26256 | g__IMCC26256 | s__uncultured_Ferrimicrobium |
| 12495579 | Bacteria | p__Proteobacteria | c__Gammaproteobacteria | o__Xanthomonadales | f__Rhodanobacteraceae | g__Chujaibacter |  |
| fe0dfb17ea71e67af42542f08a743df7 | Bacteria | p__Campilobacterota | c__Campylobacteria | o__Campylobacterales | f__Arcobacteraceae |  |  |
| abad23373ead40c0576ae41ad8facadd | Bacteria | p__Actinobacteriota | c__Actinobacteria | o__Corynebacteriales | f__Corynebacteriaceae | g__Corynebacterium |  |
| G011751895_1 | Bacteria | p__Actinobacteriota | c__Actinobacteria | o__Micrococcales | f__Microbacteriaceae |  |  |
| 3c5e28718131c79abf846aaabbd1c72c | Bacteria | p__Firmicutes | c__Bacilli | o__Alicyclobacillales | f__Alicyclobacillaceae | g__Tumebacillus | s__uncultured_Alicyclobacillaceae |
| G000712035_1 | Bacteria | p__Firmicutes | c__Bacilli | o__Staphylococcales | f__Staphylococcaceae | g__Salinicoccus |  |
| 1a329f30283e2b152b0cbc0d23833970 | Bacteria | p__Bacteroidota | c__Bacteroidia | o__Flavobacteriales | f__Flavobacteriaceae | g__Flavobacterium | s__Flavobacterium_ummariense |
| 26f54b1c80d6b8aa90733c32d275d948 | Bacteria | p__Firmicutes | c__Bacilli | o__Bacillales | f__Bacillaceae |  |  |
| b26db174d9b3a871a6e3bf725815f8d6 | Bacteria | p__Proteobacteria | c__Gammaproteobacteria | o__Xanthomonadales | f__Rhodanobacteraceae | g__Ahniella |  |
| RS-GCF-013322225.1-NZ-JAAMFB010000048.1 | Bacteria | p__Proteobacteria | c__Alphaproteobacteria | o__Rhizobiales | f__Rhizobiaceae | g__Allorhizobium-Neorhizobium-Pararhizobium-Rhizobium | s__[Pseudomonas]_geniculata |
| MJ031-1-barcode42-umi8663bins-ubs-57 | Bacteria | p__Bacteroidota | c__Bacteroidia | o__Sphingobacteriales | f__Sphingobacteriaceae |  |  |
| 2224d9da32dae27eb9ad4405f3e16d69 | Bacteria | p__Actinobacteriota | c__Thermoleophilia | o__Solirubrobacterales | f__67-14 | g__67-14 |  |
| 02918512 | Bacteria | p__Planctomycetota | c__Phycisphaerae | o__Tepidisphaerales | f__WD2101_soil_group | g__WD2101_soil_group |  |
| 0b733631434d6931905800509a39836c | Bacteria | p__Hydrogenedentes | c__Hydrogenedentia | o__Hydrogenedentiales | f__Hydrogenedensaceae | g__Hydrogenedensaceae | s__uncultured_bacterium |
| 8ca71f6b8c03e3bf18e5a779ed4993d4 | Bacteria | p__Proteobacteria | c__Gammaproteobacteria | o__Xanthomonadales | f__Xanthomonadaceae | g__Stenotrophomonas |  |
| MJ006-1-barcode45-umi40866bins-ubs-8 | Bacteria | p__Firmicutes | c__Clostridia | o__Oscillospirales | f__Ruminococcaceae | g__Ruminococcus |  |
| MJ006-2-barcode51-umi163156bins-ubs-3 | Bacteria | p__Firmicutes | c__Clostridia | o__Lachnospirales | f__Lachnospiraceae |  |  |
| G000154805_2 | Bacteria | p__Firmicutes | c__Bacilli | o__Erysipelotrichales | f__Erysipelatoclostridiaceae | g__Erysipelatoclostridium | s__[Clostridium]_spiroforme |
| 684803d64687ee0f5b87ecd535db74cc | Bacteria | p__Actinobacteriota | c__Actinobacteria | o__Actinomycetales | f__Actinomycetaceae | g__Actinomyces | s__Actinomyces_graevenitzii |
| G000154845_5 | Bacteria | p__Bacteroidota | c__Bacteroidia | o__Bacteroidales | f__Bacteroidaceae | g__Bacteroides | s__Bacteroides_coprocola |
| MJ006-1-barcode38-umi157215bins-ubs-3 | Bacteria | p__Firmicutes | c__Clostridia | o__Lachnospirales | f__Lachnospiraceae |  |  |
| c176c03af1fef9636affdd909e3da016 | Bacteria | p__Actinobacteriota | c__MB-A2-108 | o__MB-A2-108 | f__MB-A2-108 | g__MB-A2-108 |  |
| KP899563 | Bacteria | p__Proteobacteria | c__Alphaproteobacteria | o__Rhizobiales | f__Xanthobacteraceae | g__Bradyrhizobium |  |
| 7e43996fc69d2d7cb5f6dea3eb1fe75f | Bacteria | p__Proteobacteria | c__Gammaproteobacteria | o__Burkholderiales | f__SC-I-84 | g__SC-I-84 |  |
| 722d065d51aa159e6a0ee024a0294dda | Bacteria | p__Actinobacteriota | c__Thermoleophilia | o__Solirubrobacterales | f__67-14 | g__67-14 | s__metagenome |
| 9fa6d989bdceb2f614543ef40205704f | Bacteria | p__Firmicutes | c__Clostridia | o__Lachnospirales | f__Lachnospiraceae | g__A2 | s__uncultured_bacterium |
| b3a41c9c58832e74a33921c00ac4f703 | Bacteria | p__Actinobacteriota | c__Thermoleophilia | o__Gaiellales | f__Gaiellaceae | g__Gaiella | s__uncultured_Rubrobacteria |
| 400af62e08033a102c14b1ab956d915a | Bacteria | p__Actinobacteriota | c__Actinobacteria | o__Propionibacteriales | f__Nocardioidaceae | g__Nocardioides |  |
| MJ033-1-barcode37-umi42540bins-ubs-3 | Bacteria | p__Bdellovibrionota | c__Bdellovibrionia | o__Bdellovibrionales | f__Bdellovibrionaceae | g__Bdellovibrio |  |
| RS-GCF-004345285.1-NZ-SLUJ01000094.1 | Bacteria | p__Proteobacteria | c__Gammaproteobacteria | o__Enterobacterales |  |  |  |
| bac0f1a0c82c790ae2c1a60d7adc548b | Bacteria | p__Chloroflexi | c__Chloroflexia | o__Thermomicrobiales | f__JG30-KF-CM45 | g__JG30-KF-CM45 | s__metagenome |
| 4ffd6a9ffe6113afc109f36490436d78 | Bacteria | p__Proteobacteria | c__Alphaproteobacteria | o__Rhizobiales | f__Xanthobacteraceae | g__Bradyrhizobium |  |
| MJ005-barcode57-umi73096bins-ubs-3 | Bacteria | p__Proteobacteria | c__Gammaproteobacteria | o__Burkholderiales | f__Sutterellaceae | g__Parasutterella |  |
| 018f4c9e9f5606a3642b28798583ac82 | Bacteria | p__Actinobacteriota | c__Thermoleophilia | o__Solirubrobacterales | f__Solirubrobacteraceae | g__Conexibacter |  |
| MJ006-1-barcode39-umi145585bins-ubs-3 | Bacteria | p__Firmicutes | c__Clostridia | o__Christensenellales | f__Christensenellaceae | g__Christensenellaceae_R-7_group | s__uncultured_prokaryote |
| b562f951e0e06da70c6d4df36a2f88f9 | Bacteria | p__Verrucomicrobiota | c__Verrucomicrobiae | o__Chthoniobacterales | f__Chthoniobacteraceae | g__Candidatus_Udaeobacter |  |
| b8404fd32508c81078410a53f240ba33 | Bacteria | p__Firmicutes | c__Bacilli | o__Erysipelotrichales | f__Erysipelotrichaceae | g__Turicibacter |  |
| 2cc9561fe8dad59f5d4c835a1f16f1df | Bacteria | p__Verrucomicrobiota | c__Verrucomicrobiae | o__Opitutales | f__Opitutaceae | g__Lacunisphaera | s__uncultured_Verrucomicrobia |
| 52d899db626e550c573b994e5348afa7 | Bacteria | p__Proteobacteria | c__Alphaproteobacteria | o__Sphingomonadales | f__Sphingomonadaceae | g__Sphingomonas |  |
| 9a13b6a5bfa0978aadc58c7fa4d85ad6 | Bacteria | p__Actinobacteriota | c__Actinobacteria | o__Micromonosporales | f__Micromonosporaceae | g__Virgisporangium |  |
| a43de165875f046ea36ac5bccf6b7257 | Bacteria | p__Bacteroidota | c__Bacteroidia | o__Bacteroidales | f__Prevotellaceae | g__Prevotella |  |
| G000786655_1 | Bacteria | p__Actinobacteriota | c__Actinobacteria | o__Micrococcales | f__Micrococcaceae | g__Kocuria |  |
| b68ef7b3e07459ed35182b4b85a141f1 | Bacteria | p__Proteobacteria | c__Gammaproteobacteria | o__Burkholderiales | f__Burkholderiaceae | g__Pandoraea |  |
| f878803e8a12db7bc6bbd4efda4029d8 | Bacteria | p__Proteobacteria | c__Gammaproteobacteria | o__Xanthomonadales | f__Xanthomonadaceae | g__Pseudoxanthomonas |  |
| 4ca570f2e5a85da47c54701329611a4f | Bacteria | p__Proteobacteria | c__Gammaproteobacteria | o__Pseudomonadales | f__Pseudomonadaceae | g__Pseudomonas |  |
| 14409247 | Bacteria |  |  |  |  |  |  |
| 85ba78eab8d10261e88aeb021bedcc6e | Bacteria | p__Proteobacteria | c__Gammaproteobacteria | o__Pseudomonadales | f__Moraxellaceae | g__Acinetobacter |  |
| 89f57a2677c2bae7fc05a93a427bfb61 | Bacteria | p__Proteobacteria | c__Gammaproteobacteria | o__Xanthomonadales | f__Xanthomonadaceae | g__Lysobacter |  |
| 04906824 | Bacteria | p__Proteobacteria | c__Alphaproteobacteria | o__Rhizobiales | f__Xanthobacteraceae | g__Afipia |  |
| b6d4c6a966262e6f74dec5ea32278ff1 | Bacteria | p__Acidobacteriota | c__Acidobacteriae | o__Acidobacteriales | f__uncultured | g__uncultured |  |
| f0115d20a3229232779e0bc471921c1b | Bacteria | p__Actinobacteriota | c__Actinobacteria | o__Streptomycetales | f__Streptomycetaceae | g__Streptomyces |  |
| MJ006-1-barcode45-umi123176bins-ubs-5 | Bacteria | p__Firmicutes | c__Clostridia | o__Oscillospirales | f__UCG-010 | g__UCG-010 |  |
| 0033d75280774731ba155abfebc84b76 | Bacteria | p__Bacteroidota | c__Bacteroidia | o__Bacteroidales | f__Bacteroidaceae | g__Bacteroides |  |
| 0190ce18f830f0a0c1b7764c42a29e4f | Bacteria | p__Firmicutes | c__Bacilli | o__Lactobacillales | f__Carnobacteriaceae | g__Atopostipes |  |
| 7744d798a9a61f10174b7f6597a5f048 | Bacteria | p__Proteobacteria | c__Alphaproteobacteria | o__Rhodospirillales | f__uncultured | g__uncultured | s__uncultured_Magnetospirillum |
| d5c8f58c4382ae010851dfcddf9464f2 | Bacteria | p__Proteobacteria | c__Alphaproteobacteria | o__Rhizobiales | f__Xanthobacteraceae | g__uncultured |  |
| f062bfed289fab4be19338bee2126290 | Bacteria | p__Chloroflexi | c__Ktedonobacteria | o__Ktedonobacterales | f__Ktedonobacteraceae | g__Ktedonobacter | s__Dictyobacter_aurantiacus |
| RS-GCF-018618925.1-NZ-JACSNA010000009.1 | Bacteria | p__Firmicutes | c__Bacilli | o__Exiguobacterales | f__Exiguobacteraceae | g__Exiguobacterium |  |
| G000829415_1 | Bacteria | p__Proteobacteria | c__Gammaproteobacteria | o__Pseudomonadales | f__Pseudomonadaceae | g__Pseudomonas |  |
| 3140f03bbe2e7626f75096eafbe5f3f1 | Bacteria | p__Proteobacteria | c__Gammaproteobacteria | o__Pseudomonadales | f__Pseudomonadaceae | g__Pseudomonas |  |
| 3647ae43004af061b0cc03bc502ada0c | Bacteria | p__Chloroflexi | c__Chloroflexia | o__Chloroflexales | f__Roseiflexaceae | g__Kouleothrix | s__uncultured_bacterium |
| b1b145f7fd705840621cd8f902bfc551 | Bacteria | p__Firmicutes | c__Bacilli | o__Staphylococcales | f__Staphylococcaceae | g__Salinicoccus |  |
| 8b85de40eaeba1715926b9e8de5022e7 | Bacteria | p__Firmicutes | c__Clostridia | o__Peptostreptococcales-Tissierellales | f__Anaerovoracaceae |  |  |
| 6f79ad8e2ca5f58a5805b4b1d8fe9c3c | Bacteria | p__Proteobacteria | c__Gammaproteobacteria | o__Pseudomonadales | f__Pseudomonadaceae | g__Pseudomonas |  |
| MJ005-barcode49-umi421bins-ubs-136 | Bacteria | p__Proteobacteria | c__Gammaproteobacteria | o__Burkholderiales | f__Neisseriaceae | g__Neisseria |  |
| 4840b125a6daa74c3186ea65e353ce21 | Bacteria | p__Proteobacteria | c__Alphaproteobacteria | o__Sphingomonadales | f__Sphingomonadaceae | g__Sphingobium |  |
| 10095877 | Bacteria | p__Cyanobacteria | c__Cyanobacteriia | o__Cyanobacteriales | f__uncultured | g__uncultured | s__uncultured_cyanobacterium |
| MJ030-2-barcode50-umi31304bins-ubs-22 | Bacteria | p__Bacteroidota | c__Bacteroidia | o__Cytophagales | f__Hymenobacteraceae | g__Pontibacter |  |
| 19d4e49e568c3cd35d2d6a09cc4c1215 | Bacteria | p__Proteobacteria | c__Gammaproteobacteria | o__Alteromonadales |  |  |  |
| 2d011a6568c433f8f3b09af4b00ec4b6 | Bacteria | p__Firmicutes | c__Bacilli | o__Bacillales | f__Planococcaceae |  |  |
| MJ009-1-barcode47-umi16874bins-ubs-25 | Bacteria | p__Bacteroidota | c__Bacteroidia | o__Sphingobacteriales | f__Sphingobacteriaceae | g__Sphingobacterium | s__Sphingobacterium_spiritivorum |
| 78ddc45d1a0560068ed9db325d62808c | Bacteria | p__Proteobacteria | c__Alphaproteobacteria | o__Sphingomonadales | f__Sphingomonadaceae | g__Sphingomonas |  |
| 04665631 | Bacteria | p__Proteobacteria | c__Gammaproteobacteria | o__Pseudomonadales | f__Pseudomonadaceae | g__Pseudomonas |  |
| 401f08ce858d8099fda04dd2bd2e081b | Bacteria | p__Bacteroidota | c__Bacteroidia | o__Bacteroidales | f__F082 | g__F082 |  |
| 11548721 | Bacteria |  |  |  |  |  |  |
| 04959987 | Bacteria | p__Proteobacteria | c__Gammaproteobacteria | o__Burkholderiales | f__Oxalobacteraceae |  |  |
| 8406e243b1ddaf7772dc2377c530da42 | Bacteria | p__Proteobacteria | c__Gammaproteobacteria | o__Enterobacterales |  |  |  |
| 8887b0ea6b65120b4af54b93b2e63791 | Bacteria | p__Proteobacteria | c__Alphaproteobacteria | o__Rhodobacterales | f__Rhodobacteraceae |  |  |
| f3841134927f7e5aa5d84c5af6f5f36c | Bacteria | p__Patescibacteria | c__Saccharimonadia | o__Saccharimonadales |  |  |  |
| dbd244dd6bcb88ac180be742090b3253 | Bacteria | p__Proteobacteria | c__Gammaproteobacteria | o__Burkholderiales |  |  |  |
| 02411980 | Bacteria | p__Proteobacteria | c__Alphaproteobacteria | o__Rhizobiales | f__Rhizobiaceae | g__Allorhizobium-Neorhizobium-Pararhizobium-Rhizobium | s__[Pseudomonas]_geniculata |
| RS-GCF-003058325.1-NZ-CP028844.1--3 | Bacteria | p__Proteobacteria | c__Alphaproteobacteria | o__Rhizobiales | f__Beijerinckiaceae | g__Methylobacterium-Methylorubrum |  |
| 654ed90e89fa0ec36b00e8f011da34d8 | Bacteria | p__Bdellovibrionota | c__Oligoflexia | o__0319-6G20 | f__0319-6G20 | g__0319-6G20 |  |
| KT988031 | Bacteria | p__Firmicutes | c__Bacilli | o__Staphylococcales | f__Staphylococcaceae | g__Corticicoccus | s__Corticicoccus_populi |
| 3afebfe5ff5f46d36c63592ee7bde69d | Bacteria | p__Actinobacteriota | c__Actinobacteria | o__Micrococcales | f__Micrococcaceae |  |  |
| aaf370b92574f25505f2b51124c5c476 | Bacteria | p__Chloroflexi | c__Ktedonobacteria | o__Ktedonobacterales | f__Ktedonobacteraceae |  |  |
| 17334957 | Bacteria | p__Firmicutes | c__Bacilli | o__Paenibacillales | f__Paenibacillaceae | g__Paenibacillus |  |
| e6574dafcc6806b136898fc07380e5f4 | Bacteria | p__Firmicutes | c__Bacilli | o__Bacillales | f__Marinococcaceae | g__Alteribacillus |  |
| EF104116 | Bacteria | p__Actinobacteriota | c__Actinobacteria | o__Pseudonocardiales | f__Pseudonocardiaceae | g__Saccharopolyspora |  |
| 787732cd9504ea082cb124bad6bf88ce | Bacteria | p__Actinobacteriota | c__Actinobacteria | o__Corynebacteriales | f__Nocardiaceae | g__Rhodococcus |  |
| 19706714 | Bacteria | p__Firmicutes | c__Bacilli | o__Erysipelotrichales | f__Erysipelotrichaceae | g__Ileibacterium | s__Ileibacterium_valens |
| 915e40599dc0a409f1327c197ceb07e5 | Bacteria | p__Chloroflexi | c__Anaerolineae | o__Caldilineales | f__Caldilineaceae | g__uncultured | s__metagenome |
| 61123c3aa6437ceec4ca57eacd04c24c | Bacteria | p__Proteobacteria | c__Gammaproteobacteria | o__Burkholderiales | f__Comamonadaceae | g__Simplicispira |  |
| GB-GCA-000318405.1-KB291796.1 | Bacteria | p__Proteobacteria | c__Alphaproteobacteria | o__Caulobacterales | f__Caulobacteraceae | g__Brevundimonas | s__Brevundimonas_olei |
| ee23b900cfb3543c9d7fa15131074c50 | Bacteria | p__Bacteroidota | c__Bacteroidia | o__Flavobacteriales | f__Flavobacteriaceae | g__Flavobacterium | s__Flavobacterium_ummariense |
| f5b9102184afaf543dcec15cde2023a2 | Bacteria | p__Actinobacteriota | c__Actinobacteria | o__Micrococcales | f__Microbacteriaceae |  |  |
| 05132551 | Bacteria | p__Firmicutes | c__Bacilli | o__Lactobacillales | f__Carnobacteriaceae | g__Carnobacterium |  |
| b5662148c05995b1dcb659e022ce749d | Bacteria | p__Proteobacteria | c__Gammaproteobacteria | o__Xanthomonadales | f__Rhodanobacteraceae | g__Ahniella |  |
| f2f5ec61123e0662cc5948e916a49161 | Bacteria | p__Actinobacteriota | c__Actinobacteria | o__Micrococcales | f__Dermabacteraceae | g__Helcobacillus | s__Helcobacillus_massiliensis |
| GB-GCA-902166465.1-LR607350.1 | Bacteria | p__Proteobacteria | c__Gammaproteobacteria | o__Enterobacterales | f__Enterobacteriaceae |  |  |
| MJ031-1-barcode38-umi31967bins-ubs-7 | Bacteria | p__Firmicutes | c__Clostridia | o__Oscillospirales | f__Ruminococcaceae | g__[Eubacterium]_siraeum_group | s__uncultured_bacterium |
| 15054902 | Bacteria | p__Acidobacteriota | c__Acidobacteriae | o__Bryobacterales | f__Bryobacteraceae | g__Bryobacter |  |
| RS-GCF-018067805.1-NZ-JAGKMP010000005.1 | Bacteria | p__Proteobacteria | c__Gammaproteobacteria | o__Enterobacterales | f__Morganellaceae | g__Providencia |  |
| 0620af0f011099829b1125d57230c5d6 | Bacteria | p__Proteobacteria | c__Gammaproteobacteria | o__Burkholderiales | f__Burkholderiaceae | g__Ralstonia |  |
| G001421785_2 | Bacteria | p__Proteobacteria | c__Alphaproteobacteria | o__Sphingomonadales | f__Sphingomonadaceae | g__Sphingomonas |  |
| 0160aff6266060dd04ec6b96a24ee1c1 | Bacteria | p__Firmicutes | c__Clostridia | o__Peptostreptococcales-Tissierellales | f__Peptostreptococcaceae |  |  |
| 14801515 | Bacteria | p__Chloroflexi | c__Ktedonobacteria | o__Ktedonobacterales | f__JG30-KF-AS9 | g__JG30-KF-AS9 | s__uncultured_bacterium |
| MJ020-1-barcode44-umi162792bins-ubs-3 | Bacteria | p__Firmicutes | c__Clostridia | o__Lachnospirales | f__Lachnospiraceae |  |  |
| EU817500 | Bacteria | p__Firmicutes | c__Bacilli | o__Lactobacillales | f__Carnobacteriaceae | g__Carnobacterium |  |
| 0ba628633caa7924008bc51dad8e7484 | Bacteria | p__Proteobacteria | c__Alphaproteobacteria | o__Sphingomonadales | f__Sphingomonadaceae |  |  |
| KF483861 | Bacteria | p__Proteobacteria | c__Gammaproteobacteria | o__Xanthomonadales | f__Xanthomonadaceae | g__Lysobacter |  |
| 04957236 | Bacteria | p__Proteobacteria | c__Gammaproteobacteria | o__Burkholderiales | f__Comamonadaceae |  |  |
| b0627ce1e2a1a3a1cb4a2347cee5ce73 | Bacteria | p__Proteobacteria | c__Gammaproteobacteria | o__Burkholderiales | f__Comamonadaceae |  |  |
| MN365231 | Bacteria | p__Proteobacteria | c__Alphaproteobacteria | o__Rhodobacterales | f__Rhodobacteraceae |  |  |
| 0f8ef8945c028dfcadcf4c364ed1ce48 | Bacteria | p__Proteobacteria | c__Alphaproteobacteria | o__Caulobacterales | f__Caulobacteraceae | g__Brevundimonas |  |
| RS-GCF-014207835.1-NZ-JACHKS010000006.1 | Bacteria | p__Bacteroidota | c__Bacteroidia | o__Flavobacteriales | f__Weeksellaceae | g__Chryseobacterium |  |
| 04909739 | Bacteria | p__Proteobacteria | c__Alphaproteobacteria | o__Rhizobiales | f__Rhizobiaceae | g__Paenochrobactrum |  |
| b3c6f32f0a5409386e4b87db1dbff206 | Bacteria | p__Chloroflexi | c__Chloroflexia | o__Chloroflexales | f__Roseiflexaceae | g__uncultured | s__uncultured_bacterium |
| 5064f673161701c4add5ffe11fa502fc | Bacteria | p__Proteobacteria | c__Gammaproteobacteria | o__Burkholderiales | f__Rhodocyclaceae |  |  |
| ff43c1f43eb36d0e52b0e01608ead1d5 | Bacteria | p__Chloroflexi | c__Anaerolineae | o__SBR1031 | f__A4b | g__A4b | s__wastewater_metagenome |
| cbc95bb698af43798a16c3e98958933b | Bacteria |  |  |  |  |  |  |
| RS-GCF-009759525.1-NZ-WGGT01000066.1 | Bacteria | p__Firmicutes | c__Clostridia | o__Lachnospirales | f__Lachnospiraceae | g__Roseburia |  |
| ea4c6371c136134292fa847ee2432443 | Bacteria | p__Bacteroidota | c__Bacteroidia | o__Bacteroidales | f__Tannerellaceae | g__Macellibacteroides |  |
| f13c553fdfe0ada339c8dddbfb30aeb2 | Bacteria | p__Proteobacteria | c__Gammaproteobacteria | o__Gammaproteobacteria_Incertae_Sedis | f__Unknown_Family | g__Acidibacter |  |
| 00d1c58c138a233a18b3e99af9892216 | Bacteria | p__Proteobacteria | c__Gammaproteobacteria | o__Pseudomonadales | f__Pseudomonadaceae | g__Pseudomonas |  |
| 1fda2e0dce83afa894fe45c4a483e3ff | Bacteria | p__Actinobacteriota | c__Actinobacteria |  |  |  |  |
| 1fca21b705562eb55e7c7767c0cdbe8e | Bacteria | p__Actinobacteriota | c__Actinobacteria | o__Corynebacteriales | f__Mycobacteriaceae | g__Mycobacterium |  |
| 04958837 | Bacteria | p__Actinobacteriota | c__Actinobacteria | o__Micrococcales | f__Microbacteriaceae |  |  |
| e309c98b50d924a1639e411b11629cc9 | Bacteria | p__Firmicutes | c__Clostridia | o__Christensenellales | f__Christensenellaceae | g__Christensenellaceae_R-7_group |  |
| 05033063 | Bacteria | p__Actinobacteriota | c__Actinobacteria | o__Corynebacteriales | f__Mycobacteriaceae | g__Mycobacterium |  |
| RS-GCF-003576535.1-NZ-QXCQ01000108.1 | Bacteria | p__Proteobacteria | c__Gammaproteobacteria | o__Pseudomonadales | f__Moraxellaceae | g__Acinetobacter |  |
| a74ca0e4dcccc9f95dc65285512eed39 | Bacteria | p__Myxococcota | c__Polyangia | o__Polyangiales | f__Polyangiaceae |  |  |
| 788a8164eeedefb18283b29f3378a8c7 | Bacteria | p__Proteobacteria | c__Alphaproteobacteria | o__Rhizobiales | f__Rhizobiaceae |  |  |
| EF465534 | Bacteria | p__Proteobacteria | c__Alphaproteobacteria | o__Sphingomonadales | f__Sphingomonadaceae | g__Sphingobium |  |
| 5c2f8cdbd79bc3823aeb891171174539 | Bacteria | p__Actinobacteriota | c__Actinobacteria | o__Propionibacteriales | f__Nocardioidaceae | g__Nocardioides |  |
| 58407f12ac6cbaa98a879b28a636f6ad | Bacteria | p__Firmicutes | c__Bacilli | o__Bacillales | f__Bacillaceae | g__Bacillus |  |
| 9437344e721c22156e07836ae9457ff6 | Bacteria | p__Gemmatimonadota | c__Gemmatimonadetes | o__Gemmatimonadales | f__Gemmatimonadaceae | g__uncultured | s__metagenome |
| 02250706 | Bacteria | p__Proteobacteria | c__Alphaproteobacteria | o__Caulobacterales | f__Caulobacteraceae | g__uncultured |  |
| 8a1a237f22a5a1eb1659338c44b0cdd1 | Bacteria | p__Proteobacteria | c__Gammaproteobacteria | o__Burkholderiales | f__Comamonadaceae |  |  |
| 0495a8b6555a0dd8e3150a870e6f0cf9 | Bacteria | p__Proteobacteria | c__Gammaproteobacteria | o__Burkholderiales | f__Sutterellaceae | g__Parasutterella |  |
| 08088c79214691d6eed2f7ecb5022a9c | Bacteria | p__Proteobacteria | c__Alphaproteobacteria | o__Rhizobiales | f__Xanthobacteraceae |  |  |
| b68b326bb91ebcb79e716513f79e49b8 | Bacteria | p__Firmicutes | c__Bacilli | o__Lactobacillales | f__Streptococcaceae | g__Lactococcus |  |
| 098e55e3a2aa32a6e47782454c2283ca | Bacteria | p__Armatimonadota | c__Armatimonadia | o__Armatimonadales | f__Armatimonadaceae | g__Armatimonas | s__uncultured_beta |
| cdfd87f24d79535eede292b9eadadb08 | Bacteria | p__Proteobacteria | c__Gammaproteobacteria | o__Enterobacterales | f__Enterobacteriaceae |  |  |
| 9d395da05753cad17175f56f184d57f4 | Bacteria | p__Firmicutes | c__Bacilli | o__Lactobacillales | f__Streptococcaceae | g__Lactococcus |  |
| 1e3c0554dce8d5e0a65947ce08aaa86c | Bacteria | p__Actinobacteriota | c__Acidimicrobiia | o__Microtrichales | f__Microtrichaceae | g__uncultured |  |
| 868c3441f3f76ad7d27c99e27a867b45 | Bacteria | p__Proteobacteria | c__Alphaproteobacteria | o__Rhizobiales | f__Xanthobacteraceae |  |  |
| b57e936429ec941648fda76d504621db | Bacteria | p__Cyanobacteria | c__Cyanobacteriia | o__Cyanobacteriales |  |  |  |
| 85e3481196988b0edad1e6a8a2a1aa55 | Bacteria | p__Proteobacteria | c__Gammaproteobacteria | o__Burkholderiales | f__Comamonadaceae |  |  |
| 04934716 | Bacteria | p__Bacteroidota | c__Bacteroidia | o__Flavobacteriales | f__Weeksellaceae | g__Chishuiella | s__Chishuiella_changwenlii |
| 1327bcba37e44c0f1c0efc0a62fb812a | Bacteria | p__Actinobacteriota | c__Thermoleophilia | o__Solirubrobacterales | f__Solirubrobacteraceae |  |  |
| 228f8460275d76212351aff705aa2ccd | Bacteria | p__Proteobacteria | c__Alphaproteobacteria | o__Caulobacterales | f__Caulobacteraceae | g__Brevundimonas |  |
| 04909634 | Bacteria | p__Proteobacteria | c__Alphaproteobacteria | o__Rhizobiales | f__Rhizobiaceae |  |  |
| 66661887167a72b5481d5a771eb63d91 | Bacteria | p__Chloroflexi | c__Ktedonobacteria | o__Ktedonobacterales | f__JG30-KF-AS9 | g__JG30-KF-AS9 |  |
| f698f115dcc423243c58f8b609b4788e | Bacteria | p__Proteobacteria | c__Alphaproteobacteria | o__Rhodobacterales | f__Rhodobacteraceae | g__Paracoccus |  |
| GB-GCA-004793885.1-SRYG01000051.1 | Bacteria | p__Firmicutes | c__Bacilli | o__Erysipelotrichales | f__Erysipelotrichaceae | g__Dubosiella | s__uncultured_bacterium |
| G000488855_1 | Bacteria | p__Firmicutes | c__Clostridia | o__Peptostreptococcales-Tissierellales | f__Anaerovoracaceae | g__[Eubacterium]_brachy_group |  |
| f42e517dc7916986c8393fd9c957fe5d | Bacteria | p__Acidobacteriota | c__Blastocatellia | o__Pyrinomonadales | f__Pyrinomonadaceae | g__RB41 |  |
| 56d1560720cf9530b032ba9bbe57f1b3 | Bacteria | p__Bacteroidota | c__Bacteroidia | o__Bacteroidales | f__Prevotellaceae | g__Prevotellaceae_UCG-001 |  |
| f19b9d5464986c8b86b60f1b0b7d130a | Bacteria | p__Bacteroidota | c__Bacteroidia |  |  |  |  |
| 33a4185ca04244934515b562c9690eb3 | Bacteria | p__Proteobacteria | c__Gammaproteobacteria | o__Pseudomonadales | f__Moraxellaceae | g__Acinetobacter |  |
| RS-GCF-014268925.1-NZ-JABWRK010000058.1 | Bacteria | p__Proteobacteria | c__Gammaproteobacteria | o__Pseudomonadales | f__Pseudomonadaceae | g__Pseudomonas |  |
| 04a91ef6d07d4267ef4edef26fd48fee | Bacteria | p__Proteobacteria | c__Gammaproteobacteria | o__Burkholderiales | f__SC-I-84 | g__SC-I-84 |  |
| KX865139 | Bacteria | p__Firmicutes | c__Bacilli | o__Bacillales | f__Bacillaceae | g__Bacillus |  |
| GB-GCA-900758135.1-CAAEQM010000063.1 | Bacteria | p__Bacteroidota | c__Bacteroidia | o__Bacteroidales | f__Rikenellaceae | g__Rikenellaceae_RC9_gut_group |  |
| 08582635 | Bacteria | p__Proteobacteria | c__Alphaproteobacteria | o__Azospirillales | f__Azospirillaceae | g__Skermanella |  |
| RS-GCF-002967455.1-NZ-PUGJ01000494.1 | Bacteria | p__Proteobacteria | c__Gammaproteobacteria | o__Enterobacterales | f__Enterobacteriaceae | g__Escherichia-Shigella |  |
| 23055547 | Bacteria | p__Bacteroidota | c__Bacteroidia | o__Bacteroidales | f__Muribaculaceae | g__Muribaculaceae |  |
| 5d89367efb5ca6f6b12ce531b905f7b5 | Bacteria | p__Cyanobacteria | c__Cyanobacteriia | o__Oxyphotobacteria_Incertae_Sedis | f__Unknown_Family | g__Leptolyngbya_ANT.L52.2 |  |
| 19268657 | Bacteria | p__Actinobacteriota | c__Actinobacteria | o__Propionibacteriales | f__Nocardioidaceae | g__Nocardioides |  |
| 857e2f8b8e1f3e0bfbf14b5ef8ac7757 | Bacteria | p__Proteobacteria | c__Gammaproteobacteria | o__Burkholderiales | f__Hydrogenophilaceae | g__Hydrogenophilus |  |
| 10853201ceae5643aeae61231ab928e5 | Bacteria | p__Proteobacteria | c__Gammaproteobacteria | o__Burkholderiales | f__Nitrosomonadaceae | g__mle1-7 | s__uncultured_Burkholderiales |
| d63e06f83bf44edb013ea279fdd647be | Bacteria | p__Proteobacteria | c__Gammaproteobacteria | o__Pseudomonadales | f__Moraxellaceae | g__Acinetobacter |  |
| 285eee5903a48cc1c456180746e81e46 | Bacteria | p__Myxococcota | c__Polyangia | o__Polyangiales |  |  |  |
| 56f063b00ed50e3ec6f042717c99b7fa | Bacteria | p__Verrucomicrobiota | c__Verrucomicrobiae | o__Chthoniobacterales | f__Chthoniobacteraceae | g__Candidatus_Udaeobacter |  |
| 07207569 | Bacteria | p__Acidobacteriota | c__Vicinamibacteria | o__Subgroup_17 | f__Subgroup_17 | g__Subgroup_17 |  |
| 05029086 | Bacteria | p__Firmicutes | c__Clostridia | o__Peptostreptococcales-Tissierellales | f__Peptostreptococcaceae | g__Romboutsia |  |
| 04951257 | Bacteria | p__Firmicutes | c__Bacilli | o__Alicyclobacillales | f__Alicyclobacillaceae | g__Tumebacillus |  |
| 11531303 | Bacteria | p__Desulfobacterota | c__Desulfovibrionia | o__Desulfovibrionales | f__Desulfovibrionaceae | g__Desulfovibrio |  |
| 9203fb10d31b43042e50b1768d1d6807 | Bacteria | p__Actinobacteriota | c__Actinobacteria | o__Propionibacteriales | f__Propionibacteriaceae |  |  |
| 15341645 | Bacteria | p__Acidobacteriota | c__Acidobacteriae | o__Bryobacterales | f__Bryobacteraceae | g__Bryobacter | s__uncultured_actinobacterium |
| e1571b492964790c4c14ac164a806a3f | Bacteria | p__Campilobacterota | c__Campylobacteria | o__Campylobacterales | f__Arcobacteraceae | g__Arcobacter |  |
| RS-GCF-002212305.1-NZ-LYNU01000453.1 | Bacteria | p__Proteobacteria | c__Gammaproteobacteria | o__Pseudomonadales | f__Moraxellaceae | g__Acinetobacter |  |
| b00392502d8ddf98c30f32b1fb0b5490 | Bacteria | p__Firmicutes | c__Bacilli | o__Bacillales | f__Marinococcaceae | g__Alteribacillus |  |
| c5056fc406a0444054e3125b9d809673 | Bacteria | p__Acidobacteriota | c__Vicinamibacteria | o__Vicinamibacterales | f__uncultured | g__uncultured |  |
| a172032f7957561057026a768e58ee8a | Bacteria | p__Firmicutes | c__Bacilli | o__Bacillales | f__Bacillaceae | g__Geobacillus |  |
| a4b331d9f6715b1203b86d1ed28bc200 | Bacteria |  |  |  |  |  |  |
| e2c399fee2d803acc6b87ea3aa58e362 | Bacteria | p__Firmicutes | c__Bacilli | o__Bacillales |  |  |  |
| dca3db992cf40692a7d967b84ee1e931 | Bacteria | p__Proteobacteria | c__Gammaproteobacteria | o__Cellvibrionales | f__Spongiibacteraceae | g__BD1-7_clade |  |
| RS-GCF-003290055.1-NZ-QMKS01000021.1 | Bacteria | p__Proteobacteria | c__Gammaproteobacteria | o__Burkholderiales | f__Burkholderiaceae | g__Ralstonia |  |
| MJ032-2-barcode60-umi53778bins-ubs-9 | Bacteria | p__Bacteroidota | c__Bacteroidia | o__Chitinophagales | f__Chitinophagaceae | g__Taibaiella |  |
| 13634027 | Bacteria | p__Bacteroidota | c__Bacteroidia | o__Chitinophagales | f__Saprospiraceae | g__uncultured | s__uncultured_soil |
| 3c282fd142876d907701cdf23f0cecc0 | Bacteria | p__Actinobacteriota | c__Actinobacteria | o__Bifidobacteriales | f__Bifidobacteriaceae | g__Bifidobacterium |  |
| GB-GCA-902646515.1-CACRCQ010000098.1 | Bacteria | p__Bacteroidota | c__Bacteroidia | o__Bacteroidales | f__Rikenellaceae | g__Alistipes |  |
| 91ae75692901265b1c5077c9440fc766 | Bacteria | p__Myxococcota | c__Polyangia | o__Polyangiales | f__Polyangiaceae |  |  |
| 8f3dc3f66e315ae28bcd3214546ecc23 | Bacteria | p__Actinobacteriota | c__Actinobacteria |  |  |  |  |
| 5db79923c7c3b57ff6201cc7b837666d | Bacteria | p__Actinobacteriota | c__Actinobacteria | o__Micrococcales | f__Microbacteriaceae |  |  |
| 7866e58ab45b414107de6588c009dd77 | Bacteria | p__Proteobacteria | c__Gammaproteobacteria | o__Burkholderiales | f__Burkholderiaceae | g__Burkholderia-Caballeronia-Paraburkholderia |  |
| MJ034-2-barcode54-umi3691bins-ubs-92 | Bacteria | p__Proteobacteria | c__Alphaproteobacteria | o__Rhizobiales | f__Rhizobiales_Incertae_Sedis | g__uncultured |  |
| AM501446 | Bacteria | p__Proteobacteria | c__Gammaproteobacteria | o__Burkholderiales | f__Comamonadaceae |  |  |
| G001945525_1 | Bacteria | p__Firmicutes | c__Bacilli | o__Erysipelotrichales | f__Erysipelotrichaceae | g__Ileibacterium | s__Ileibacterium_valens |
| 9555aa981567bac0874e461577855213 | Bacteria | p__Chloroflexi | c__Chloroflexia | o__Kallotenuales | f__AKIW781 | g__AKIW781 | s__uncultured_bacterium |
| 16597432 | Bacteria | p__Firmicutes | c__Clostridia | o__Lachnospirales | f__Lachnospiraceae |  |  |
| 665d544acbf08b6cd6e7a2b53f232617 | Bacteria | p__Firmicutes | c__Bacilli | o__Erysipelotrichales | f__Erysipelotrichaceae | g__Erysipelothrix |  |
| RS-GCF-017167705.1-NZ-JAFDPH010000138.1 | Bacteria | p__Actinobacteriota | c__Actinobacteria | o__Micrococcales | f__Micrococcaceae | g__Micrococcus |  |
| G001591445_1 | Bacteria | p__Firmicutes | c__Bacilli | o__Bacillales | f__Bacillaceae | g__Bacillus |  |
| 03958887 | Bacteria | p__Sumerlaeota | c__Sumerlaeia | o__Sumerlaeales | f__Sumerlaeaceae | g__Sumerlaea | s__uncultured_Acidobacteria |
| 917bcb110fc21366efc7c6af2845a11a | Bacteria | p__Actinobacteriota | c__Thermoleophilia | o__Solirubrobacterales | f__67-14 | g__67-14 | s__uncultured_Conexibacteraceae |
| 02239596 | Bacteria | p__Proteobacteria | c__Alphaproteobacteria | o__Caulobacterales | f__Caulobacteraceae |  |  |
| 79bcc281e74b20185b358f5f9a7a5ee1 | Bacteria | p__Proteobacteria | c__Gammaproteobacteria | o__Burkholderiales | f__Comamonadaceae |  |  |
| JN713899 | Bacteria | p__Proteobacteria | c__Gammaproteobacteria | o__Burkholderiales | f__Comamonadaceae | g__Tepidicella | s__uncultured_Tepidicella |
| 17214710 | Bacteria | p__Actinobacteriota | c__Thermoleophilia | o__Solirubrobacterales | f__67-14 | g__67-14 |  |
| 3d2d7992a9a93ba27982f405f6d520d4 | Bacteria | p__Proteobacteria | c__Gammaproteobacteria | o__PLTA13 | f__PLTA13 | g__PLTA13 | s__uncultured_sludge |
| DQ417336 | Bacteria | p__Proteobacteria | c__Gammaproteobacteria | o__Burkholderiales | f__Comamonadaceae |  |  |
| KC508867 | Bacteria | p__Proteobacteria | c__Alphaproteobacteria | o__Rhizobiales | f__Xanthobacteraceae | g__Bradyrhizobium |  |
| ddc59f343a9339180332c4991c10d2d9 | Bacteria | p__Firmicutes | c__Bacilli | o__Caldalkalibacillales | f__Caldalkalibacillaceae | g__Caldalkalibacillus |  |
| GB-GCA-018919725.1-JAHLRK010000301.1 | Bacteria | p__Proteobacteria | c__Gammaproteobacteria | o__Enterobacterales | f__Enterobacteriaceae |  |  |
| 21292682 | Bacteria | p__Firmicutes | c__Clostridia | o__Clostridiales | f__Clostridiaceae | g__Clostridium_sensu_stricto_1 |  |
| 22d56e7fddb75af860fbeddbcc25e381 | Bacteria | p__Chloroflexi | c__Anaerolineae | o__Anaerolineales | f__Anaerolineaceae |  |  |
| ba853f8edb77c9106ed3f2ee9bc16a9a | Bacteria | p__Proteobacteria | c__Alphaproteobacteria | o__Rhodobacterales | f__Rhodobacteraceae |  |  |
| 5ab13246f7eaa1af649057d615230e0f | Bacteria | p__Proteobacteria | c__Gammaproteobacteria | o__Gammaproteobacteria_Incertae_Sedis | f__Unknown_Family | g__Acidibacter |  |
| 89816c87a2dd32ec21f0b8d1064bd0c2 | Bacteria | p__Proteobacteria | c__Alphaproteobacteria | o__Rickettsiales | f__SM2D12 | g__SM2D12 |  |
| aa2e4e1e0a62c6ed41f24bbb05c10149 | Bacteria | p__Proteobacteria | c__Alphaproteobacteria | o__Acetobacterales | f__Acetobacteraceae |  |  |
| MJ030-2-barcode59-umi72693bins-ubs-8 | Bacteria | p__Proteobacteria | c__Gammaproteobacteria | o__Burkholderiales | f__Comamonadaceae | g__Comamonas |  |
| 04666531 | Bacteria | p__Firmicutes | c__Bacilli | o__Staphylococcales | f__Staphylococcaceae | g__Staphylococcus |  |
| 04909622 | Bacteria | p__Proteobacteria | c__Alphaproteobacteria | o__Rhizobiales | f__Rhizobiaceae | g__Phyllobacterium |  |
| 05021468 | Bacteria | p__Actinobacteriota | c__Actinobacteria | o__Micrococcales | f__Micrococcaceae | g__Glutamicibacter |  |
| 892ee6845f6f8b6698fe85772cc625c4 | Bacteria | p__Bacteroidota | c__Bacteroidia | o__Sphingobacteriales | f__Sphingobacteriaceae | g__Mucilaginibacter |  |
| 7a6ad425046a6bd6508d321ec7d60b25 | Bacteria | p__Planctomycetota | c__Phycisphaerae | o__Tepidisphaerales | f__WD2101_soil_group | g__WD2101_soil_group | s__uncultured_eubacterium |
| KY117470 | Bacteria | p__Proteobacteria | c__Alphaproteobacteria | o__Sphingomonadales | f__Sphingomonadaceae |  |  |
| 12187479 | Bacteria | p__Verrucomicrobiota | c__Chlamydiae | o__Chlamydiales | f__Simkaniaceae | g__uncultured |  |
| 610788c07b4be9ba639fa38acd935e8c | Bacteria | p__Actinobacteriota | c__Actinobacteria | o__Micrococcales | f__Microbacteriaceae | g__Leucobacter |  |
| b87fa3d92ae9304e9984d62bdf2b8cec | Bacteria | p__Firmicutes | c__Bacilli | o__Exiguobacterales | f__Exiguobacteraceae | g__Exiguobacterium |  |
| FR714938 | Bacteria | p__Proteobacteria | c__Alphaproteobacteria | o__Rhizobiales | f__Rhizobiaceae |  |  |
| 50031fdbbac754aa5deeb5fa09605186 | Bacteria | p__Patescibacteria | c__Saccharimonadia | o__Saccharimonadales | f__Saccharimonadales | g__Saccharimonadales |  |
| 6cb3393aa90908315b4652b123deec48 | Bacteria | p__Proteobacteria | c__Gammaproteobacteria | o__Burkholderiales | f__Comamonadaceae | g__Ottowia |  |
| RS-GCF-900111375.1-NZ-FOHB01000007.1 | Bacteria | p__Actinobacteriota | c__Actinobacteria | o__Micrococcales | f__Intrasporangiaceae |  |  |
| 02406359 | Bacteria | p__Actinobacteriota | c__Actinobacteria | o__Bifidobacteriales | f__Bifidobacteriaceae | g__Bifidobacterium |  |
| 11903515 | Bacteria | p__Proteobacteria | c__Alphaproteobacteria | o__Azospirillales | f__uncultured | g__uncultured | s__uncultured_bacterium |
| 19294894 | Bacteria | p__Actinobacteriota | c__Actinobacteria | o__Corynebacteriales | f__Tsukamurellaceae | g__Tsukamurella |  |
| b17bd26fcb705dbd3297956d1b4727a5 | Bacteria | p__Proteobacteria | c__Alphaproteobacteria | o__Rhizobiales | f__Xanthobacteraceae | g__uncultured |  |
| c16ee1d3b4e7cf6931b7cb7a92768ec6 | Bacteria | p__Firmicutes | c__Bacilli | o__Paenibacillales | f__Paenibacillaceae | g__Paenibacillus |  |
| 09992521 | Bacteria | p__Planctomycetota | c__Planctomycetes | o__Pirellulales | f__Pirellulaceae | g__Pirellula |  |
| e0ebf907b635f876969d3ad3beb2cff6 | Bacteria | p__Firmicutes | c__Bacilli | o__Lactobacillales | f__Enterococcaceae | g__Enterococcus |  |
| 08219800 | Bacteria | p__Proteobacteria | c__Alphaproteobacteria | o__Rhizobiales | f__Beijerinckiaceae | g__Methylobacterium-Methylorubrum |  |
| 9045932c8e2efe4f2a6ed0c190524aa0 | Bacteria | p__Proteobacteria | c__Gammaproteobacteria | o__Pseudomonadales | f__Pseudomonadaceae | g__Pseudomonas |  |
| MJ034-1-barcode45-umi108bins-ubs-1668 | Bacteria | p__Bacteroidota | c__Bacteroidia | o__Flavobacteriales | f__Weeksellaceae | g__Chryseobacterium |  |
| fdd31da7d9286ed1708154d148f13f18 | Bacteria | p__Proteobacteria | c__Alphaproteobacteria | o__Sphingomonadales | f__Sphingomonadaceae | g__Sphingomonas |  |
| MJ006-2-barcode71-umi136802bins-ubs-4 | Bacteria | p__Firmicutes | c__Clostridia | o__Lachnospirales | f__Lachnospiraceae | g__Coprococcus |  |
| 36218436ab0f06ef3f3db845ac748c7f | Bacteria | p__Proteobacteria | c__Gammaproteobacteria | o__Burkholderiales | f__Comamonadaceae | g__Comamonas |  |
| 5b1e0d263983cddf72f58c6efca021cd | Bacteria | p__Bacteroidota | c__Bacteroidia | o__Cytophagales | f__Microscillaceae | g__OLB12 |  |
| 14800122 | Bacteria |  |  |  |  |  |  |
| RS-GCF-016107705.1-NZ-JAEHGC010000041.1 | Bacteria | p__Firmicutes | c__Bacilli | o__Bacillales | f__Bacillaceae | g__Bacillus |  |
| 02406175 | Bacteria | p__Actinobacteriota | c__Actinobacteria | o__Corynebacteriales | f__Mycobacteriaceae | g__Mycobacterium |  |
| a6063816d129b46f6ead2a21a43e0c7e | Bacteria | p__Proteobacteria | c__Gammaproteobacteria | o__Xanthomonadales | f__Rhodanobacteraceae |  |  |
| RS-GCF-003590715.1-NZ-QQVY01000005.1 | Bacteria | p__Proteobacteria | c__Alphaproteobacteria | o__Rhodobacterales | f__Rhodobacteraceae | g__Paracoccus |  |
| 7c90d250118e20c0bead043fd9505b6c | Bacteria | p__Proteobacteria | c__Gammaproteobacteria | o__Burkholderiales | f__SC-I-84 | g__SC-I-84 |  |
| 6f2e7e876aef2d82b31d57bc8dbf9ea5 | Bacteria | p__Actinobacteriota | c__Rubrobacteria | o__Rubrobacterales | f__Rubrobacteriaceae | g__Rubrobacter |  |
| G000332875_1 | Bacteria | p__Firmicutes | c__Clostridia | o__Lachnospirales | f__Lachnospiraceae | g__Anaerostipes |  |
| 05028672 | Bacteria | p__Chloroflexi | c__Ktedonobacteria | o__Ktedonobacterales | f__Ktedonobacteraceae | g__HSB_OF53-F07 | s__uncultured_soil |
| KF308409 | Bacteria | p__Proteobacteria | c__Gammaproteobacteria | o__Enterobacterales | f__Yersiniaceae | g__Serratia |  |
| 0ea338cf17404457c9c9f159b2f6a2cb | Bacteria | p__Actinobacteriota | c__Acidimicrobiia |  |  |  |  |
| b79bbe14f4563678344b01fffec38d43 | Bacteria | p__Proteobacteria | c__Alphaproteobacteria | o__Acetobacterales | f__Acetobacteraceae |  |  |
| f9d22524de0baa0e4f9428740117beeb | Bacteria | p__Acidobacteriota | c__Acidobacteriae | o__Subgroup_2 | f__Subgroup_2 | g__Subgroup_2 | s__uncultured_Holophaga |
| 7aa9ebc39d25a96c62e3e47b7e1c53af | Bacteria | p__Chloroflexi | c__TK10 | o__TK10 | f__TK10 | g__TK10 |  |
| 2052a8a108df1a1f6e324de60650fe2e | Bacteria | p__Proteobacteria | c__Gammaproteobacteria | o__Burkholderiales | f__Comamonadaceae |  |  |
| ec1a65d50b62b57ceab3d2c9b2b0ab35 | Bacteria | p__Bacteroidota | c__Bacteroidia | o__Sphingobacteriales | f__Sphingobacteriaceae | g__Sphingobacterium |  |
| 07960cd06bcfb8397b9d012b12ab0e32 | Bacteria | p__Proteobacteria | c__Gammaproteobacteria | o__Burkholderiales | f__Alcaligenaceae | g__Parapusillimonas |  |
| RS-GCF-008633215.1-NZ-BHYR01000037.1 | Bacteria | p__Fusobacteriota | c__Fusobacteriia | o__Fusobacteriales | f__Fusobacteriaceae | g__Fusobacterium |  |
| RS-GCF-013409575.1-NZ-JACCCY010000002.1--2 | Bacteria | p__Bacteroidota | c__Bacteroidia | o__Bacteroidales | f__Tannerellaceae | g__Macellibacteroides |  |
| 19706816 | Bacteria | p__Firmicutes | c__Bacilli | o__Erysipelotrichales | f__Erysipelotrichaceae | g__Ileibacterium | s__Ileibacterium_valens |
| 04962332 | Bacteria | p__Actinobacteriota | c__Actinobacteria | o__Corynebacteriales | f__Corynebacteriaceae | g__Turicella | s__uncultured_bacterium |
| 01252041 | Bacteria | p__Bacteroidota | c__Bacteroidia | o__Bacteroidales | f__Rikenellaceae | g__Rikenella | s__uncultured_bacterium |
| c80833164ce8782083fda49465bc6e18 | Bacteria | p__Firmicutes | c__Bacilli | o__Erysipelotrichales | f__Erysipelatoclostridiaceae | g__Erysipelatoclostridium | s__uncultured_Clostridium |
| d1fbd80080e280f66297722e3f785fdf | Bacteria | p__Bacteroidota | c__Bacteroidia | o__Sphingobacteriales | f__Sphingobacteriaceae | g__Sphingobacterium | s__Sphingobacterium_mizutaii |
| eda4f1d78d56b66011533fa6f7c04b94 | Bacteria | p__Proteobacteria | c__Alphaproteobacteria | o__Rhizobiales | f__Rhizobiales_Incertae_Sedis | g__uncultured | s__uncultured_bacterium |
| eb9bac3481a132f2e77719ddea37bc57 | Bacteria | p__Bacteroidota | c__Bacteroidia | o__Sphingobacteriales | f__Sphingobacteriaceae | g__Sphingobacterium | s__Sphingobacterium_thalpophilum |
| RS-GCF-009734465.1-NZ-CP046446.1--5 | Bacteria | p__Proteobacteria | c__Gammaproteobacteria | o__Enterobacterales | f__Enterobacteriaceae |  |  |
| a7364c177f64c7ff22ba5fe182d4f2b8 | Bacteria | p__Proteobacteria | c__Gammaproteobacteria | o__Burkholderiales | f__Rhodocyclaceae |  |  |
| 10a16b4abbfc42fb4185ddb7b6b8d5b3 | Bacteria | p__Proteobacteria | c__Gammaproteobacteria | o__Xanthomonadales | f__Xanthomonadaceae | g__Stenotrophomonas |  |
| RS-GCF-902831875.1-NZ-CADEZC010000051.1 | Bacteria | p__Proteobacteria | c__Gammaproteobacteria | o__Burkholderiales | f__Burkholderiaceae | g__Burkholderia-Caballeronia-Paraburkholderia |  |
| cb3cf96d1a1048d644e424be4fbf7ef9 | Bacteria | p__Actinobacteriota | c__Actinobacteria | o__Micrococcales | f__Microbacteriaceae | g__Microbacterium |  |
| 07989329 | Bacteria | p__Proteobacteria | c__Alphaproteobacteria | o__Rhizobiales | f__Hyphomicrobiaceae | g__Hyphomicrobium |  |
| 05002357 | Bacteria | p__Bacteroidota | c__Bacteroidia | o__Sphingobacteriales | f__Sphingobacteriaceae | g__Olivibacter |  |
| MJ032-2-barcode74-umi56200bins-ubs-8 | Bacteria | p__Proteobacteria | c__Gammaproteobacteria | o__Burkholderiales | f__Comamonadaceae | g__Comamonas |  |
| 9f2279a1bc02f9cbb58e7637097a46bc | Bacteria | p__Acidobacteriota | c__Vicinamibacteria | o__Vicinamibacterales | f__Vicinamibacteraceae | g__Luteitalea | s__bacterium_enrichment |
| d11c28720a3360cf60dabdc3eaa34791 | Bacteria | p__Patescibacteria | c__Saccharimonadia | o__Saccharimonadales |  |  |  |
| 0e3190256d45a8347ecbafca4ffc9026 | Bacteria | p__Proteobacteria | c__Gammaproteobacteria | o__Burkholderiales | f__A21b | g__A21b |  |
| 4f6c971a64d6ea67b50b0d2ac442ec52 | Bacteria | p__Firmicutes | c__Clostridia | o__Clostridia_UCG-014 | f__Clostridia_UCG-014 | g__Clostridia_UCG-014 |  |
| RS-GCF-003670205.1-NZ-RAYS01000200.1 | Bacteria | p__Bacteroidota | c__Bacteroidia | o__Bacteroidales | f__Bacteroidaceae | g__Bacteroides |  |
| 89a3910ddceb24c62c323e0d441b1561 | Bacteria | p__Proteobacteria | c__Gammaproteobacteria | o__Enterobacterales |  |  |  |
| 25d1f9a9c0489305171683ba8894ddf6 | Bacteria | p__Deinococcota | c__Deinococci | o__Deinococcales | f__Deinococcaceae | g__Deinococcus |  |
| AB109209 | Bacteria | p__Firmicutes | c__Bacilli | o__Bacillales | f__Bacillaceae | g__Bacillus |  |
| 4928c395ee07eac167f6363e8225d32a | Bacteria | p__Firmicutes | c__Clostridia | o__Lachnospirales | f__Lachnospiraceae | g__Roseburia |  |
| 8584d5d36fc340735b5d974dad3eb1a6 | Bacteria | p__MBNT15 | c__MBNT15 | o__MBNT15 | f__MBNT15 | g__MBNT15 |  |
| KC237387 | Bacteria | p__Proteobacteria | c__Alphaproteobacteria | o__Rhizobiales | f__Rhizobiaceae | g__Mesorhizobium |  |
| MJ031-1-barcode30-umi37185bins-ubs-4 | Bacteria | p__Proteobacteria | c__Gammaproteobacteria | o__Burkholderiales | f__Comamonadaceae |  |  |
| c7554c441cd490acbce47252bbd27bf9 | Bacteria | p__Actinobacteriota | c__Actinobacteria | o__Micrococcales | f__Microbacteriaceae | g__Microbacterium |  |
| 6caa04e8ddfe20d000b56e03fd952828 | Bacteria | p__Firmicutes | c__Negativicutes | o__Veillonellales-Selenomonadales | f__Veillonellaceae | g__Dialister | s__Dialister_succinatiphilus |
| 04545616 | Bacteria | p__Firmicutes | c__Clostridia | o__Oscillospirales | f__[Eubacterium]_coprostanoligenes_group | g__[Eubacterium]_coprostanoligenes_group | s__uncultured_bacterium |
| 8917ccb28f2b83aa7c6170e4c55fcfee | Bacteria | p__Proteobacteria | c__Alphaproteobacteria | o__Reyranellales | f__Reyranellaceae | g__Reyranella |  |
| dc170d7330c3253182913cce1dd10f80 | Bacteria | p__Proteobacteria | c__Gammaproteobacteria | o__Enterobacterales | f__Enterobacteriaceae | g__Escherichia-Shigella |  |
| 274fa228f3fb3f1639a459b50f6d8bfb | Bacteria | p__Proteobacteria | c__Gammaproteobacteria | o__Burkholderiales | f__Comamonadaceae | g__Delftia |  |
| 4ed1b93952c4e358c83142019394810c | Bacteria | p__Proteobacteria | c__Alphaproteobacteria | o__Elsterales | f__uncultured | g__uncultured |  |
| 79a9002e027a8dc380f2e6e2f34f0272 | Bacteria | p__Chloroflexi | c__KD4-96 | o__KD4-96 | f__KD4-96 | g__KD4-96 | s__uncultured_bacterium |
| 1fd14dc783c526b1123a2291389fb80c | Bacteria | p__Proteobacteria | c__Gammaproteobacteria | o__Burkholderiales | f__Comamonadaceae | g__Hydrogenophaga |  |
| 04945756 | Bacteria | p__Actinobacteriota | c__Actinobacteria | o__Micrococcales | f__Microbacteriaceae |  |  |
| bd923bc7309cf8b486552d526cfe5cbc | Bacteria | p__Gemmatimonadota | c__Gemmatimonadetes | o__Gemmatimonadales | f__Gemmatimonadaceae | g__uncultured |  |
| a61d454f0308e195a8001fab76ae5f22 | Bacteria | p__Firmicutes | c__Bacilli | o__Lactobacillales | f__Streptococcaceae | g__Streptococcus | s__Streptococcus_danieliae |
| RS-GCF-000266345.1-NZ-AKEK01000250.1 | Bacteria | p__Proteobacteria | c__Gammaproteobacteria | o__Xanthomonadales | f__Xanthomonadaceae | g__Xanthomonas |  |
| fa7bd4714bfd6b8d4c58d33579f75c16 | Bacteria | p__Gemmatimonadota | c__Gemmatimonadetes | o__Gemmatimonadales | f__Gemmatimonadaceae | g__Gemmatimonas |  |
| MJ034-1-barcode40-umi5630bins-ubs-79 | Bacteria | p__Bacteroidota | c__Bacteroidia | o__Chitinophagales | f__Chitinophagaceae | g__Flavitalea |  |
| 3aaea13b6a8147dc6b6505d4f85a9479 | Bacteria | p__Firmicutes | c__Bacilli | o__Alicyclobacillales | f__Alicyclobacillaceae | g__Tumebacillus |  |
| 1e5d611761ffde3ffecb364af2005484 | Bacteria |  |  |  |  |  |  |
| 04930830 | Bacteria | p__Proteobacteria | c__Gammaproteobacteria | o__Enterobacterales | f__Erwiniaceae |  |  |
| 142adcd66ae5a7740512f1429a8ba9a9 | Bacteria | p__Myxococcota | c__Polyangia | o__Haliangiales | f__Haliangiaceae | g__Haliangium |  |
| ab9ba84bd5d7d46b0442970ca62008a8 | Bacteria | p__Proteobacteria | c__Gammaproteobacteria | o__Burkholderiales | f__Burkholderiaceae | g__Ralstonia |  |
| 8682f9b0bdb951ce9ceeb2a4b3feed99 | Bacteria | p__Actinobacteriota | c__Acidimicrobiia | o__Microtrichales | f__Iamiaceae | g__Iamia |  |
| b445048b660872ab85e3adb4a22f64e8 | Bacteria | p__Actinobacteriota | c__Thermoleophilia | o__Gaiellales | f__uncultured | g__uncultured |  |
| 8d13c93678921a6c53a1297a183a2042 | Bacteria | p__Chloroflexi | c__Chloroflexia | o__Thermomicrobiales | f__AKYG1722 | g__AKYG1722 |  |
| ac5af28c6ea0eb0546a03852f3d055e4 | Bacteria | p__Firmicutes | c__Bacilli | o__Staphylococcales | f__Staphylococcaceae | g__Staphylococcus |  |
| 13686646 | Bacteria | p__Gemmatimonadota | c__Gemmatimonadetes | o__Gemmatimonadales | f__Gemmatimonadaceae | g__Gemmatimonas |  |
| 1accc788f94d2af4a3edf60a7b490457 | Bacteria | p__Proteobacteria | c__Gammaproteobacteria | o__Pseudomonadales | f__Moraxellaceae | g__Acinetobacter |  |
| 3b972512d129c2e309e6724145699763 | Bacteria | p__Firmicutes | c__Clostridia | o__Oscillospirales | f__Ruminococcaceae | g__Subdoligranulum |  |
| 3f88bb52e87776040a3d2bd207a660f9 | Bacteria | p__Methylomirabilota | c__Methylomirabilia | o__Rokubacteriales | f__Rokubacteriales | g__Rokubacteriales |  |
| 80a85f916c204b075254f5e1e8119288 | Bacteria | p__Proteobacteria | c__Gammaproteobacteria | o__Burkholderiales | f__TRA3-20 | g__TRA3-20 |  |
| RS-GCF-016907255.1-NZ-JAFBBQ010000001.1 | Bacteria | p__Actinobacteriota | c__Actinobacteria | o__Micrococcales | f__Microbacteriaceae | g__Microbacterium |  |
| MJ030-2-barcode58-umi39250bins-ubs-17 | Bacteria | p__Proteobacteria | c__Gammaproteobacteria | o__Burkholderiales | f__Comamonadaceae | g__Hydrogenophaga |  |
| 7e67c191eb0ce892094f89b4569d40b1 | Bacteria | p__Planctomycetota | c__Planctomycetes | o__Pirellulales | f__Pirellulaceae | g__Pir4_lineage |  |
| 3aaaefa31dfff197d3ff38c0753ac92d | Bacteria | p__Chloroflexi | c__Ktedonobacteria | o__C0119 | f__C0119 | g__C0119 | s__uncultured_Chloroflexi |
| 943d7b2a115c9f8392bfa202c8695293 | Bacteria | p__Myxococcota | c__Polyangia | o__Polyangiales | f__Polyangiaceae | g__Pajaroellobacter | s__uncultured_Chondromyces |
| 5feb03192fcee1fba86412ab705fd1c5 | Bacteria | p__Bacteroidota | c__Bacteroidia | o__Cytophagales | f__Microscillaceae | g__Ohtaekwangia |  |
| 18261064 | Bacteria | p__Patescibacteria | c__Saccharimonadia | o__Saccharimonadales | f__Saccharimonadales | g__Saccharimonadales |  |
| MJ030-2-barcode65-umi17008bins-ubs-35 | Bacteria | p__Acidobacteriota | c__Acidobacteriae | o__Acidobacteriales | f__uncultured | g__uncultured | s__uncultured_forest |
| 2e5e72fd4679856636531fdce5ba091a | Bacteria | p__Proteobacteria | c__Gammaproteobacteria | o__Enterobacterales |  |  |  |
| 03151101 | Bacteria | p__Planctomycetota | c__Phycisphaerae | o__Tepidisphaerales | f__WD2101_soil_group | g__WD2101_soil_group | s__uncultured_bacterium |
| 05030330 | Bacteria | p__Proteobacteria | c__Gammaproteobacteria | o__Burkholderiales | f__Comamonadaceae |  |  |
| 17219630 | Bacteria | p__Actinobacteriota | c__Thermoleophilia | o__Gaiellales | f__uncultured | g__uncultured |  |
| ec063750d34398be733d97b8567db374 | Bacteria | p__Chloroflexi | c__Anaerolineae | o__Caldilineales | f__Caldilineaceae | g__uncultured | s__uncultured_bacterium |
| RS-GCF-019052595.1-NZ-JAHQUW010000259.1 | Bacteria | p__Firmicutes | c__Bacilli | o__Erysipelotrichales | f__Erysipelatoclostridiaceae | g__Erysipelatoclostridium |  |
| 01873434 | Bacteria | p__Actinobacteriota | c__Coriobacteriia | o__Coriobacteriales | f__Eggerthellaceae | g__DNF00809 | s__uncultured_bacterium |
| 88a94feeaf61b4ae9abbd26b201e5a6b | Bacteria | p__Acidobacteriota | c__Acidobacteriae | o__Bryobacterales | f__Bryobacteraceae | g__Bryobacter |  |
| 4699fef0fe333507c0f3c977d9958f9e | Bacteria | p__Bacteroidota | c__Bacteroidia | o__Flavobacteriales | f__Weeksellaceae | g__Chryseobacterium |  |
| MJ030-1-barcode42-umi23369bins-ubs-24 | Bacteria | p__Verrucomicrobiota | c__Verrucomicrobiae | o__Pedosphaerales | f__Pedosphaeraceae | g__Ellin517 |  |
| 83ab8c2ac29b62dcfc060dc28051c397 | Bacteria | p__Proteobacteria | c__Gammaproteobacteria | o__Gammaproteobacteria_Incertae_Sedis | f__Unknown_Family | g__Acidibacter |  |
| 05024637 | Bacteria | p__Firmicutes | c__Clostridia | o__Lachnospirales | f__Lachnospiraceae |  |  |
| 5bd2af312e82cdc9b8d4ddb32cc0b122 | Bacteria | p__Proteobacteria | c__Gammaproteobacteria | o__Burkholderiales | f__Sutterellaceae | g__uncultured |  |
| 640a9ee94158e6820855143857052bf0 | Bacteria | p__Cyanobacteria | c__Cyanobacteriia | o__Oxyphotobacteria_Incertae_Sedis | f__Unknown_Family | g__Leptolyngbya_EcFYyyy-00 | s__uncultured_soil |
| 00176909 | Bacteria | p__Chloroflexi | c__Anaerolineae | o__SBR1031 | f__A4b | g__A4b |  |
| 7c181442d8132c6ae93eea36ba062a37 | Bacteria | p__Proteobacteria | c__Gammaproteobacteria | o__Pseudomonadales | f__Moraxellaceae | g__Acinetobacter |  |
| 735b15d64f900c910cfe2d7d74c63fea | Bacteria | p__Actinobacteriota | c__Actinobacteria | o__Streptomycetales | f__Streptomycetaceae | g__Streptomyces |  |
| 332dd9c26673b3f528bc8878dc4eed9b | Bacteria | p__Myxococcota | c__Polyangia | o__mle1-27 | f__mle1-27 | g__mle1-27 | s__Myxococcales_bacterium |
| fa5b7c1bf5fabcb05223ac2fdd6efb20 | Bacteria | p__Firmicutes | c__Bacilli | o__Lactobacillales | f__Lactobacillaceae | g__Lactobacillus |  |
| 2cc8a91e6bd2b84a8b69c5c135df81be | Bacteria | p__Firmicutes | c__Bacilli | o__Lactobacillales | f__Enterococcaceae | g__Enterococcus |  |
| 19706920 | Bacteria | p__Firmicutes | c__Bacilli | o__Erysipelotrichales | f__Erysipelotrichaceae | g__Ileibacterium | s__Ileibacterium_valens |
| 35e1ff54fcd655524194559ea8099181 | Bacteria | p__Proteobacteria | c__Gammaproteobacteria | o__Pseudomonadales | f__Pseudomonadaceae | g__Pseudomonas |  |
| RS-GCF-006333905.1-NZ-PJQB01000245.1 | Bacteria | p__Proteobacteria | c__Gammaproteobacteria | o__Enterobacterales | f__Enterobacteriaceae |  |  |
| AY501364 | Bacteria | p__Actinobacteriota | c__Actinobacteria | o__Micrococcales | f__Promicromonosporaceae |  |  |
| cfef31c3534fe859397c9aca32cfa05c | Bacteria | p__Proteobacteria | c__Gammaproteobacteria | o__PLTA13 | f__PLTA13 | g__PLTA13 |  |
| ebfb5bca8dbb55e7868ab24ba7ce352f | Bacteria | p__Proteobacteria | c__Gammaproteobacteria | o__Burkholderiales | f__Nitrosomonadaceae | g__MND1 |  |
| 7e062f6e28a236adc811619bcb8387ef | Bacteria | p__Firmicutes | c__Clostridia | o__Oscillospirales | f__Butyricicoccaceae | g__Butyricicoccus | s__uncultured_Firmicutes |
| 78b5d9b6febfbf25979720266f848747 | Bacteria | p__Actinobacteriota | c__Acidimicrobiia | o__Microtrichales | f__Microtrichaceae | g__IMCC26207 |  |
| 51e6ed46f57a608dea2e2dcff086aecd | Bacteria | p__Firmicutes | c__Clostridia | o__Peptostreptococcales-Tissierellales | f__Anaerovoracaceae |  |  |
| 218442f1f37275a89af9bffc88524dd9 | Bacteria | p__Proteobacteria | c__Alphaproteobacteria | o__Caulobacterales | f__Caulobacteraceae | g__Brevundimonas |  |
| d41ef7d30bcd628923f40dff8cd9b5f2 | Bacteria | p__Proteobacteria | c__Alphaproteobacteria | o__Rhodobacterales | f__Rhodobacteraceae | g__Amaricoccus |  |
| f91fc28ddfeaa3441b06ed7f8a800f75 | Bacteria | p__Actinobacteriota | c__Actinobacteria | o__Frankiales | f__Geodermatophilaceae | g__Blastococcus |  |
| RS-GCF-014650275.1-NZ-BMUQ01000073.1 | Bacteria | p__Actinobacteriota | c__Actinobacteria | o__Streptomycetales | f__Streptomycetaceae | g__Streptomyces |  |
| AB231946 | Bacteria | p__Proteobacteria | c__Alphaproteobacteria | o__Rhizobiales | f__Rhizobiales_Incertae_Sedis | g__Alsobacter |  |
| 0514bb425a2de7095fa8ed8ab3c1feb2 | Bacteria | p__Actinobacteriota | c__Actinobacteria | o__Corynebacteriales | f__Corynebacteriaceae | g__Corynebacterium | s__Corynebacterium_variabile |
| 6d070a7b1536e5fed2b99e0e6523f478 | Bacteria | p__Firmicutes | c__Clostridia | o__Lachnospirales | f__Lachnospiraceae | g__Tyzzerella | s__uncultured_Clostridium |
| ec30ed8b077105f5391bb6a2d3c9bc9c | Bacteria | p__Firmicutes | c__Bacilli | o__Bacillales | f__Bacillaceae | g__Bacillus |  |
| 283eafb3523f810aff1ab94bec13e331 | Bacteria | p__Nitrospirota | c__Nitrospiria | o__Nitrospirales | f__Nitrospiraceae | g__Nitrospira |  |
| GB-GCA-000980475.1-LARM01000066.1 | Bacteria | p__Bacteroidota | c__Bacteroidia | o__Bacteroidales | f__Bacteroidaceae | g__Bacteroides |  |
| a48c3af980c269e3e0e7a361b4fa9be6 | Bacteria | p__Proteobacteria | c__Gammaproteobacteria | o__Pseudomonadales | f__Pseudomonadaceae | g__Pseudomonas |  |
| MJ034-1-barcode37-umi24bins-ubs-3394 | Bacteria | p__Bacteroidota | c__Bacteroidia | o__Chitinophagales | f__Chitinophagaceae |  |  |
| b735a78bda6e64a67284e6673ecc46ef | Bacteria | p__Patescibacteria | c__Saccharimonadia | o__Saccharimonadales |  |  |  |
| 24fdda602314c5103f55997163f6d9f2 | Bacteria | p__Proteobacteria | c__Alphaproteobacteria | o__Rhizobiales | f__Devosiaceae | g__Devosia |  |
| 11138323 | Bacteria | p__Bacteroidota | c__Bacteroidia | o__Bacteroidales | f__Muribaculaceae | g__Muribaculaceae | s__uncultured_Bacteroidales |
| 04972331 | Bacteria | p__Firmicutes | c__Bacilli | o__Lactobacillales | f__Leuconostocaceae | g__Leuconostoc |  |
| b9feac10b8c8c22e7d987aaa0174f58a | Bacteria | p__Chloroflexi | c__Anaerolineae | o__C10-SB1A | f__C10-SB1A | g__C10-SB1A | s__uncultured_bacterium |
| da0a6aee6c2a3e051bf88dc7dacae5a5 | Bacteria | p__Actinobacteriota | c__Coriobacteriia | o__Coriobacteriales | f__Eggerthellaceae | g__Gordonibacter | s__uncultured_bacterium |
| 02956157 | Bacteria | p__Chloroflexi | c__Chloroflexia | o__Kallotenuales | f__AKIW781 | g__AKIW781 |  |
| 644112c1bfd5b2407bf375cb22f53aea | Bacteria | p__Planctomycetota | c__Planctomycetes | o__Gemmatales | f__Gemmataceae | g__uncultured |  |
| MJ030-1-barcode42-umi11639bins-ubs-36 | Bacteria | p__Firmicutes | c__Clostridia | o__Clostridiales | f__Clostridiaceae | g__Clostridium_sensu_stricto_9 |  |
| MJ006-1-barcode44-umi159020bins-ubs-3 | Bacteria | p__Firmicutes | c__Bacilli | o__RF39 | f__RF39 | g__RF39 | s__human_gut |
| 13105553 | Bacteria | p__Gemmatimonadota | c__Longimicrobia | o__Longimicrobiales | f__Longimicrobiaceae | g__Longimicrobiaceae | s__uncultured_bacterium |
| RS-GCF-016908395.1-NZ-JAFBDU010000024.1 | Bacteria | p__Firmicutes | c__Bacilli | o__Bacillales | f__Bacillaceae | g__Bacillus |  |
| 7545fb74ee09e69b1ee8fd5097cec940 | Bacteria | p__Proteobacteria | c__Alphaproteobacteria | o__Tistrellales | f__Geminicoccaceae | g__Candidatus_Alysiosphaera | s__uncultured_bacterium |
| LT719160 | Bacteria | p__Actinobacteriota | c__Actinobacteria | o__Kineosporiales | f__Kineosporiaceae |  |  |
| MJ032-2-barcode74-umi88647bins-ubs-3 | Bacteria | p__Firmicutes | c__Clostridia | o__Clostridiales | f__Clostridiaceae | g__Clostridium_sensu_stricto_12 | s__Clostridium_magnum |
| 45c70363fbff3c194aa08e0a8b9ef575 | Bacteria | p__Actinobacteriota | c__Acidimicrobiia | o__IMCC26256 | f__IMCC26256 | g__IMCC26256 |  |
| f57f5fc4276598fab7b3dcf425c94d77 | Bacteria | p__Firmicutes | c__Clostridia | o__Clostridiales | f__Clostridiaceae | g__Clostridium_sensu_stricto_1 |  |
| RS-GCF-001190985.1-NZ-JPWX02000090.1 | Bacteria | p__Actinobacteriota | c__Actinobacteria | o__Micrococcales | f__Micrococcaceae | g__Kocuria |  |
| 18313001 | Bacteria | p__Actinobacteriota | c__Actinobacteria | o__Propionibacteriales | f__Nocardioidaceae | g__Kribbella |  |
| 04876229 | Bacteria | p__Acidobacteriota | c__Acidobacteriae | o__Acidobacteriales | f__Acidobacteriaceae_(Subgroup_1) | g__Terriglobus |  |
| 154229d406bc8dcabd1f715896d606df | Bacteria | p__Acidobacteriota | c__Vicinamibacteria | o__Vicinamibacterales | f__Vicinamibacteraceae | g__uncultured |  |
| fae3188472107ae7c62a8860969b9672 | Bacteria | p__Actinobacteriota | c__Actinobacteria | o__Pseudonocardiales | f__Pseudonocardiaceae | g__Saccharopolyspora | s__Saccharopolyspora_qijiaojingensis |
| 44a8a84497c31bebd5814da586ff1dac | Bacteria | p__Proteobacteria | c__Gammaproteobacteria | o__Burkholderiales | f__Nitrosomonadaceae | g__Ellin6067 |  |
| MJ005-barcode42-umi87138bins-ubs-3 | Bacteria | p__Firmicutes | c__Negativicutes | o__Veillonellales-Selenomonadales | f__Selenomonadaceae | g__Megamonas |  |
| 04911068 | Bacteria | p__Proteobacteria | c__Gammaproteobacteria | o__Xanthomonadales | f__Xanthomonadaceae | g__Stenotrophomonas |  |
| 10948b01b85a080e53b3b8b72d113970 | Bacteria | p__Myxococcota | c__bacteriap25 | o__bacteriap25 | f__bacteriap25 | g__bacteriap25 | s__uncultured_Desulfovibrionales |
| 794b5dc0ddb3d81881078552ab7bb907 | Bacteria | p__Firmicutes | c__Clostridia | o__Lachnospirales | f__Lachnospiraceae | g__Lachnoclostridium |  |
| MJ009-1-barcode40-umi41361bins-ubs-8 | Bacteria | p__Firmicutes | c__Bacilli | o__Lactobacillales | f__Enterococcaceae | g__Enterococcus |  |
| KC843944 | Bacteria | p__Bacteroidota | c__Bacteroidia | o__Sphingobacteriales | f__Sphingobacteriaceae | g__Sphingobacterium |  |
| RS-GCF-016735675.1-NZ-JAERSG010000009.1 | Bacteria | p__Actinobacteriota | c__Actinobacteria | o__Propionibacteriales | f__Nocardioidaceae | g__Nocardioides |  |
| 11079978 | Bacteria | p__Bacteroidota | c__Bacteroidia | o__Bacteroidales | f__Muribaculaceae | g__Muribaculaceae |  |
| 94f09f3f50d8ea29a741e9372961f90d | Bacteria | p__Proteobacteria | c__Gammaproteobacteria | o__Burkholderiales | f__Burkholderiaceae | g__Ralstonia |  |
| 05033696 | Bacteria | p__Actinobacteriota | c__Actinobacteria | o__Micrococcales | f__Intrasporangiaceae |  |  |
| HQ315828 | Bacteria | p__Proteobacteria | c__Alphaproteobacteria | o__Azospirillales | f__Azospirillaceae | g__Skermanella |  |
| b9701b456087331defe159d2281fee57 | Bacteria | p__Acidobacteriota | c__Acidobacteriae | o__Acidobacteriales | f__Koribacteraceae | g__Candidatus_Koribacter |  |
| MJ034-1-barcode33-umi16050bins-ubs-17 | Bacteria | p__Proteobacteria | c__Gammaproteobacteria | o__Burkholderiales | f__Sutterellaceae | g__uncultured |  |
| 6212bb27ed5609e1344b6edb16146147 | Bacteria | p__Proteobacteria | c__Gammaproteobacteria |  |  |  |  |
| 8d423e001624edc2b0b7637f7316c197 | Bacteria | p__Proteobacteria | c__Gammaproteobacteria | o__Xanthomonadales | f__Xanthomonadaceae | g__Thermomonas |  |
| RS-GCF-001433975.1-NZ-AZCY01000019.1 | Bacteria | p__Firmicutes | c__Bacilli | o__Lactobacillales | f__Lactobacillaceae | g__Lactobacillus |  |
| 5626a23d71c4a1420870d66b54f1e418 | Bacteria | p__Actinobacteriota | c__Actinobacteria | o__Micrococcales | f__Microbacteriaceae | g__Microbacterium |  |
| MJ006-1-barcode40-umi135136bins-ubs-3 | Bacteria | p__Firmicutes | c__Clostridia | o__Lachnospirales | f__Lachnospiraceae |  |  |
| 66c9c2461942868f591a13c50dbaf802 | Bacteria | p__Acidobacteriota | c__Acidobacteriae | o__Subgroup_2 | f__Subgroup_2 | g__Subgroup_2 | s__metagenome |
| 67060f823da9c3cc7994d7259902d2ff | Bacteria | p__Proteobacteria | c__Gammaproteobacteria | o__Pseudomonadales | f__Moraxellaceae | g__Acinetobacter |  |
| 27f813539f98ab60deb89bd694969495 | Bacteria | p__Proteobacteria | c__Gammaproteobacteria | o__Xanthomonadales | f__Rhodanobacteraceae | g__Ahniella |  |
| 06701021 | Bacteria | p__Gemmatimonadota | c__Gemmatimonadetes | o__Gemmatimonadales | f__Gemmatimonadaceae | g__uncultured |  |
| 295f22f0605e50ea34439b45053d58bf | Bacteria | p__Proteobacteria | c__Gammaproteobacteria | o__Burkholderiales | f__Burkholderiaceae | g__Ralstonia |  |
| 93b36a7adccdbbae0c19c24c634cbd87 | Bacteria | p__Actinobacteriota | c__Actinobacteria | o__Propionibacteriales | f__Propionibacteriaceae | g__Brooklawnia |  |
| 8f17a37402994abf66beaedd2dd4f9a2 | Bacteria | p__Chloroflexi | c__Anaerolineae | o__SBR1031 | f__A4b | g__A4b |  |
| a1b43a2ea34daffc684930ba00e4db5a | Bacteria | p__Bacteroidota | c__Bacteroidia | o__Sphingobacteriales | f__Sphingobacteriaceae | g__Mucilaginibacter |  |
| ee00b5f80f79807d2b3028602baacdff | Bacteria | p__Proteobacteria | c__Gammaproteobacteria | o__Pseudomonadales | f__Moraxellaceae | g__Acinetobacter |  |
| MJ034-1-barcode41-umi3441bins-ubs-129 | Bacteria | p__Proteobacteria | c__Gammaproteobacteria | o__Burkholderiales | f__Oxalobacteraceae | g__Massilia |  |
| 4045bfc3d934a7906ba5bd7dd512cb66 | Bacteria | p__Bacteroidota | c__Bacteroidia | o__Chitinophagales | f__Chitinophagaceae |  |  |
| 8ac095c6efd6687c92edcbc998d8605f | Bacteria | p__Proteobacteria | c__Alphaproteobacteria | o__Sphingomonadales | f__Sphingomonadaceae |  |  |
| RS-GCF-018397215.1-NZ-JAFLJF010000001.1 | Bacteria | p__Firmicutes | c__Bacilli | o__Lactobacillales | f__Enterococcaceae | g__Enterococcus |  |
| 7f5e400d59829a106fe26582963c8587 | Bacteria | p__Acidobacteriota | c__Vicinamibacteria | o__Vicinamibacterales | f__uncultured | g__uncultured |  |
| 19b61a18df20180534593e5d5e39bc37 | Bacteria | p__Actinobacteriota | c__Actinobacteria | o__Pseudonocardiales | f__Pseudonocardiaceae | g__Prauserella |  |
| 517b12edb6a0165ffd8649eaa6c72553 | Bacteria | p__Firmicutes | c__Clostridia | o__Eubacteriales | f__Eubacteriaceae | g__Acetobacterium |  |
| 12c4a192b6c648cb4b6b96e9b0077b65 | Bacteria | p__Myxococcota | c__Polyangia | o__Polyangiales | f__Polyangiaceae | g__Minicystis |  |
| d39930987facb5176f9a82484bd40be7 | Bacteria | p__Actinobacteriota | c__Acidimicrobiia | o__Microtrichales | f__Ilumatobacteraceae | g__uncultured | s__uncultured_Acidimicrobiales |
| 07835635 | Bacteria | p__Planctomycetota | c__Planctomycetes | o__Planctomycetales | f__Rubinisphaeraceae | g__SH-PL14 | s__uncultured_bacterium |
| 09354e91b0f2b732eb323feeccba2355 | Bacteria | p__Bacteroidota | c__Bacteroidia | o__Chitinophagales | f__Chitinophagaceae | g__Puia | s__uncultured_soil |
| d8fed5af055e034ac4ef7bd2208de941 | Bacteria | p__Actinobacteriota | c__Actinobacteria | o__Propionibacteriales | f__Nocardioidaceae | g__Nocardioides |  |
| RS-GCF-013487985.1-NZ-CP047982.1--6 | Bacteria | p__Proteobacteria | c__Gammaproteobacteria | o__Aeromonadales | f__Aeromonadaceae | g__Aeromonas |  |
| e9cced2e8fd5601df8c75664d1177fb5 | Bacteria | p__Proteobacteria | c__Gammaproteobacteria | o__Burkholderiales | f__Nitrosomonadaceae | g__mle1-7 | s__uncultured_bacterium |
| GB-GCA-900762515.1-CAAFHA010000156.1 | Bacteria | p__Firmicutes | c__Bacilli | o__Erysipelotrichales | f__Erysipelatoclostridiaceae | g__Erysipelotrichaceae_UCG-003 |  |
| a2f0dba7e84a6f29ddd51737475e5251 | Bacteria | p__Proteobacteria | c__Alphaproteobacteria |  |  |  |  |
| b2992c6051c40e6293bef645858dce33 | Bacteria | p__Firmicutes | c__Clostridia | o__Lachnospirales | f__Lachnospiraceae |  |  |
| MJ010-2-barcode64-umi126940bins-ubs-5 | Bacteria | p__Bacteroidota | c__Bacteroidia | o__Sphingobacteriales | f__Sphingobacteriaceae | g__Sphingobacterium |  |
| 3b6aff9d9171bf267bb73a1a02121071 | Bacteria | p__Proteobacteria | c__Alphaproteobacteria | o__Elsterales | f__uncultured | g__uncultured | s__uncultured_Alphaproteobacteria |
| 590e5e278c994926b0ea8116b44f4600 | Bacteria | p__Proteobacteria | c__Gammaproteobacteria | o__Burkholderiales |  |  |  |
| MJ030-1-barcode42-umi34650bins-ubs-15 | Bacteria | p__Proteobacteria | c__Gammaproteobacteria | o__Burkholderiales | f__Comamonadaceae | g__Ramlibacter |  |
| 04875895 | Bacteria | p__Proteobacteria | c__Gammaproteobacteria | o__Pseudomonadales | f__Moraxellaceae | g__Acinetobacter |  |
| HF674688 | Bacteria | p__Proteobacteria | c__Gammaproteobacteria | o__Burkholderiales | f__Burkholderiaceae | g__Burkholderia-Caballeronia-Paraburkholderia |  |
| 04930815 | Bacteria | p__Proteobacteria | c__Gammaproteobacteria | o__Enterobacterales | f__Enterobacteriaceae |  |  |
| 02247646 | Bacteria | p__Proteobacteria | c__Alphaproteobacteria | o__Sphingomonadales | f__Sphingomonadaceae |  |  |
| 722b72750eb28f54aec6a0c7bff5717d | Bacteria | p__Actinobacteriota | c__Thermoleophilia | o__Solirubrobacterales | f__67-14 | g__67-14 |  |
| 13ffe49e511a9ca1c82bfd0372e8fe49 | Bacteria | p__Proteobacteria | c__Alphaproteobacteria | o__Sphingomonadales | f__Sphingomonadaceae | g__Sphingobium |  |
| 61445e0b5a196974f375d4f2ee3b837c | Bacteria | p__Firmicutes | c__Clostridia | o__Oscillospirales | f__UCG-010 | g__UCG-010 | s__uncultured_Clostridia |
| 19269830 | Bacteria | p__Actinobacteriota | c__Actinobacteria | o__Propionibacteriales | f__Nocardioidaceae | g__Nocardioides |  |
| 02948009 | Bacteria | p__Chloroflexi | c__Chloroflexia | o__Chloroflexales | f__Roseiflexaceae | g__uncultured | s__uncultured_bacterium |
| 2ba9a47a66f9524f89ce2b3e2e06f881 | Bacteria | p__Proteobacteria | c__Gammaproteobacteria | o__Burkholderiales | f__Comamonadaceae | g__Pelomonas |  |
| RS-GCF-014851465.1-NZ-JACUZS010000010.1 | Bacteria | p__Proteobacteria | c__Gammaproteobacteria | o__Burkholderiales | f__Alcaligenaceae | g__Achromobacter |  |
| 888448caeb2561979216397deda6a073 | Bacteria | p__Bacteroidota | c__Bacteroidia | o__Chitinophagales | f__Chitinophagaceae |  |  |
| 17974189 | Bacteria | p__Chloroflexi | c__Ktedonobacteria | o__Ktedonobacterales | f__Ktedonobacteraceae |  |  |
| fcd4d73226f79d9d408278fb0de5b8c2 | Bacteria | p__Actinobacteriota | c__Actinobacteria | o__Actinomycetales | f__Actinomycetaceae | g__uncultured | s__Actinomycetaceae_bacterium |
| 447f850288423fea1548de661682ab1b | Bacteria | p__Proteobacteria | c__Gammaproteobacteria | o__Burkholderiales | f__SC-I-84 | g__SC-I-84 |  |
| 6bd1a1b292bc2a213ac081b63f520893 | Bacteria | p__Proteobacteria | c__Gammaproteobacteria | o__Burkholderiales | f__Comamonadaceae |  |  |
| 666d67a2ea0add0ef3ec1c6e90ed3694 | Bacteria | p__Chloroflexi | c__Chloroflexia | o__Thermomicrobiales | f__JG30-KF-CM45 | g__JG30-KF-CM45 |  |
| 295da0604a4bd25bfbe2b136d181f518 | Bacteria | p__Actinobacteriota | c__Actinobacteria | o__Micrococcales | f__Microbacteriaceae | g__Leucobacter |  |
| dadcae535029609f844295797a9bf7e9 | Bacteria | p__Actinobacteriota | c__Thermoleophilia | o__Solirubrobacterales | f__67-14 | g__67-14 | s__uncultured_Conexibacteraceae |
| 08173705 | Bacteria | p__Proteobacteria | c__Alphaproteobacteria | o__Rhizobiales | f__Xanthobacteraceae | g__uncultured |  |
| 20952725 | Bacteria | p__Firmicutes | c__Bacilli | o__Erysipelotrichales | f__Erysipelotrichaceae | g__Dubosiella | s__uncultured_bacterium |
| c812fe95df8e400242411c19a32d8d00 | Bacteria | p__Acidobacteriota | c__Acidobacteriae | o__Solibacterales | f__Solibacteraceae | g__Candidatus_Solibacter |  |
| a2e9b9de7c8bd210d31cb81e8ac30527 | Bacteria | p__Proteobacteria | c__Gammaproteobacteria | o__Thiotrichales | f__Thiotrichaceae | g__Thiothrix |  |
| GQ339892 | Bacteria | p__Actinobacteriota | c__Actinobacteria | o__Propionibacteriales | f__Nocardioidaceae |  |  |
| MJ010-1-barcode29-umi151650bins-ubs-3 | Bacteria | p__Bacteroidota | c__Bacteroidia | o__Flavobacteriales | f__Flavobacteriaceae | g__Flavobacterium |  |
| 2abd962a42ad1e144351bebb89693c88 | Bacteria | p__Actinobacteriota | c__Actinobacteria | o__Corynebacteriales | f__Corynebacteriaceae | g__Corynebacterium |  |
| 3090ed0eae487670cf6407ca3e0571bd | Bacteria | p__Bacteroidota | c__Bacteroidia | o__Chitinophagales | f__Chitinophagaceae | g__Edaphobaculum |  |
| 19243440 | Bacteria | p__Actinobacteriota | c__Actinobacteria | o__Micrococcales | f__Intrasporangiaceae |  |  |
| 91ea708b8ba8125de68c9b36a409f9c1 | Bacteria | p__Desulfobacterota | c__uncultured | o__uncultured | f__uncultured | g__uncultured |  |
| 08531516 | Bacteria | p__Proteobacteria | c__Alphaproteobacteria | o__Rhizobiales | f__Rhizobiaceae | g__Chelativorans | s__uncultured_Alphaproteobacteria |
| 10844244 | Bacteria | p__Bacteroidota | c__Bacteroidia | o__Bacteroidales | f__Muribaculaceae | g__Muribaculaceae |  |
| dc9e8bc222e106d4806fdacb982bfaf6 | Bacteria | p__MBNT15 | c__MBNT15 | o__MBNT15 | f__MBNT15 | g__MBNT15 | s__uncultured_Syntrophaceae |
| MJ033-1-barcode32-umi27837bins-ubs-8 | Bacteria | p__Acidobacteriota | c__Acidobacteriae | o__Acidobacteriales |  |  |  |
| f1abee0cd0902320f676c87e698114b7 | Bacteria | p__Actinobacteriota | c__Actinobacteria | o__Corynebacteriales | f__Nocardiaceae | g__Gordonia |  |
| 3cb06468fd682b89343d284b29c20fbf | Bacteria | p__Proteobacteria | c__Gammaproteobacteria | o__Burkholderiales | f__SC-I-84 | g__SC-I-84 |  |
| 04945866 | Bacteria | p__Actinobacteriota | c__Actinobacteria | o__Micrococcales | f__Micrococcaceae |  |  |
| b0210cc88d72c435f4fc59a98ef92cf6 | Bacteria | p__Acidobacteriota | c__Acidobacteriae | o__Acidobacteriales | f__Acidobacteriaceae_(Subgroup_1) | g__Granulicella | s__bacterium_enrichment |
| AB854065 | Bacteria | p__Proteobacteria | c__Alphaproteobacteria | o__Rhizobiales | f__Rhizobiaceae | g__Allorhizobium-Neorhizobium-Pararhizobium-Rhizobium |  |
| 04903623 | Bacteria | p__Firmicutes | c__Clostridia | o__Clostridia_UCG-014 | f__Clostridia_UCG-014 | g__Clostridia_UCG-014 | s__unidentified |
| 04994132 | Bacteria | p__Proteobacteria | c__Alphaproteobacteria | o__Caulobacterales | f__Caulobacteraceae | g__Phenylobacterium |  |
| 55148ec71a18444538a4903a53dcf82e | Bacteria | p__Bacteroidota | c__Bacteroidia | o__Chitinophagales | f__Chitinophagaceae | g__Ferruginibacter |  |
| 168feca1c9e188df37cb0c8f94b2ee1b | Bacteria | p__Proteobacteria | c__Gammaproteobacteria | o__Alteromonadales | f__Pseudoalteromonadaceae | g__Pseudoalteromonas |  |
| 8f719aa7fa02823dd31c62fb26cc7ee9 | Bacteria | p__Acidobacteriota | c__Acidobacteriae | o__Acidobacteriales | f__Acidobacteriaceae_(Subgroup_1) | g__Granulicella |  |
| da06629d3beaa4e2dc2148037b8ebf38 | Bacteria | p__Actinobacteriota | c__Actinobacteria | o__Micrococcales | f__Micrococcaceae | g__Glutamicibacter |  |
| 8b1af11a3ac64e01dc8975a84f944343 | Bacteria | p__Proteobacteria | c__Gammaproteobacteria | o__Pseudomonadales | f__Pseudomonadaceae | g__Pseudomonas |  |
| ddad4cd3c4669a887a975eaa93da4d88 | Bacteria | p__Actinobacteriota | c__Actinobacteria | o__Frankiales | f__Nakamurellaceae | g__Nakamurella |  |
| 02432154 | Bacteria | p__Proteobacteria | c__Alphaproteobacteria | o__Rhizobiales | f__Methyloligellaceae |  |  |
| MJ006-2-barcode72-umi161506bins-ubs-3 | Bacteria | p__Bacteroidota | c__Bacteroidia | o__Bacteroidales | f__Bacteroidaceae | g__Bacteroides | s__Bacteroides_massiliensis |
| 0cc19bdd9d073500510a64edf6908bc2 | Bacteria | p__Chloroflexi | c__KD4-96 | o__KD4-96 | f__KD4-96 | g__KD4-96 |  |
| d9748074ee6f3861a7e87c43b24b3f3d | Bacteria | p__Acidobacteriota | c__Acidobacteriae | o__Subgroup_2 | f__Subgroup_2 | g__Subgroup_2 |  |
| 1563a014a6d1727e0cc8ab1ec16ab162 | Bacteria | p__Actinobacteriota | c__Acidimicrobiia | o__Microtrichales | f__Microtrichaceae | g__uncultured | s__metagenome |
| 928a4e0739ea3670e28ceeeeeb7488ef | Bacteria | p__Acidobacteriota | c__Holophagae | o__Subgroup_7 | f__Subgroup_7 | g__Subgroup_7 |  |
| 17a7f7749d7f4e2611fe830203c20ed2 | Bacteria | p__Gemmatimonadota | c__Gemmatimonadetes | o__Gemmatimonadales | f__Gemmatimonadaceae | g__uncultured |  |
| 0e77e928d7024a4120a3ee581a151c91 | Bacteria | p__Verrucomicrobiota | c__Verrucomicrobiae | o__Chthoniobacterales |  |  |  |
| 8c2e51d917115abf94fbfabf99b56162 | Bacteria | p__Proteobacteria | c__Gammaproteobacteria | o__Pseudomonadales | f__Moraxellaceae | g__Acinetobacter |  |
| f9fdb42aa6f206725488912e7f6a5b3c | Bacteria | p__Proteobacteria | c__Gammaproteobacteria | o__Pseudomonadales | f__Moraxellaceae | g__Acinetobacter |  |
| AF005024 | Bacteria | p__Actinobacteriota | c__Actinobacteria | o__Propionibacteriales | f__Nocardioidaceae | g__Nocardioides |  |
| MJ030-2-barcode65-umi4692bins-ubs-68 | Bacteria | p__Acidobacteriota | c__Acidobacteriae | o__Acidobacteriales | f__Acidobacteriaceae_(Subgroup_1) | g__Granulicella |  |
| AB621358 | Bacteria | p__Spirochaetota | c__Spirochaetia | o__Spirochaetales | f__Spirochaetaceae | g__Treponema | s__Treponema_denticola |
| GB-GCA-003463955.1-QSEG01000126.1 | Bacteria | p__Bacteroidota | c__Bacteroidia | o__Bacteroidales | f__Bacteroidaceae | g__Bacteroides |  |
| fb0257ccf7f1bb0b4e0810849bf6e1fe | Bacteria | p__Chloroflexi | c__Anaerolineae | o__SBR1031 | f__A4b | g__A4b | s__wastewater_metagenome |
| GB-GCA-000980395.1-LARI01000057.1 | Bacteria | p__Firmicutes | c__Clostridia | o__Oscillospirales | f__Ruminococcaceae | g__Ruminococcus | s__Ruminococcus_callidus |
| 513666c1cf2c0013d61e13bf36fee3a2 | Bacteria | p__Proteobacteria | c__Gammaproteobacteria | o__Burkholderiales | f__Comamonadaceae | g__Aquabacterium |  |
| ac47f026f3c84fc533a104e8192fb936 | Bacteria | p__Proteobacteria | c__Gammaproteobacteria | o__Pseudomonadales | f__Pseudomonadaceae | g__Pseudomonas |  |
| 08412382 | Bacteria | p__Proteobacteria | c__Alphaproteobacteria | o__Rhizobiales | f__Rhodomicrobiaceae | g__Rhodomicrobium |  |
| b44d4f5b4d5ab7eeb61d74a018bd7b2c | Bacteria | p__Proteobacteria | c__Alphaproteobacteria | o__Rhizobiales | f__Hyphomicrobiaceae |  |  |
| 811f35a935953629df524508b9e3de1b | Bacteria | p__Firmicutes | c__Bacilli |  |  |  |  |
| G001418245_1 | Bacteria | p__Proteobacteria | c__Gammaproteobacteria | o__Burkholderiales | f__Hydrogenophilaceae | g__Tepidiphilus |  |
| 04199723 | Bacteria | p__Chloroflexi | c__Anaerolineae | o__RBG-13-54-9 | f__RBG-13-54-9 | g__RBG-13-54-9 | s__uncultured_Caldilineaceae |
| e5acd9144bf3dfc4fcb639c41e046bea | Bacteria | p__Verrucomicrobiota | c__Verrucomicrobiae | o__Chthoniobacterales | f__Chthoniobacteraceae | g__Chthoniobacter | s__uncultured_bacterium |
| 03c1f154200041ee6d99d660bc636748 | Bacteria | p__Actinobacteriota | c__MB-A2-108 | o__MB-A2-108 | f__MB-A2-108 | g__MB-A2-108 | s__uncultured_bacterium |
| GB-GCA-014763725.1-JACXUX010000430.1 | Bacteria | p__Proteobacteria | c__Gammaproteobacteria | o__Xanthomonadales | f__Xanthomonadaceae | g__Pseudoxanthomonas |  |
| b786932ec90b3743e3ff09c0943c0546 | Bacteria | p__Actinobacteriota | c__Thermoleophilia | o__Gaiellales | f__Gaiellaceae | g__Gaiella |  |
| MJ010-1-barcode42-umi107085bins-ubs-5 | Bacteria | p__Proteobacteria | c__Gammaproteobacteria | o__Burkholderiales | f__Comamonadaceae | g__Comamonas |  |
| 063bdc12449b6464e5b9dc87434e6aba | Bacteria | p__Firmicutes | c__Clostridia | o__Peptostreptococcales-Tissierellales | f__Fusibacteraceae | g__Fusibacter |  |
| e9e45412239676f1c7ef39af2630344d | Bacteria | p__Firmicutes | c__Bacilli | o__Lactobacillales | f__Enterococcaceae | g__Enterococcus |  |
| RS-GCF-015557345.1-NZ-JADNOC010000089.1 | Bacteria | p__Firmicutes | c__Clostridia | o__Lachnospirales | f__Lachnospiraceae | g__[Ruminococcus]_gnavus_group |  |
| 05002563 | Bacteria | p__Bacteroidota | c__Bacteroidia | o__Bacteroidales | f__Bacteroidaceae | g__Bacteroides |  |
| 06c242e872cc3c0771d54aa313f1275e | Bacteria | p__Actinobacteriota | c__MB-A2-108 | o__MB-A2-108 | f__MB-A2-108 | g__MB-A2-108 | s__uncultured_bacterium |
| 5bd1ce82db061d2e3688152896c23b0b | Bacteria | p__Actinobacteriota | c__Acidimicrobiia | o__Microtrichales | f__Ilumatobacteraceae | g__Ilumatobacter |  |
| dadf04ac21236e85301c1f8aebcc3d0d | Bacteria | p__Actinobacteriota | c__Actinobacteria | o__Micrococcales | f__Microbacteriaceae |  |  |
| G001436475_1 | Bacteria | p__Firmicutes | c__Bacilli | o__Lactobacillales | f__Lactobacillaceae | g__Lactobacillus | s__Lactobacillus_ruminis |
| G004101785_1 | Bacteria | p__Firmicutes | c__Clostridia | o__Peptostreptococcales-Tissierellales | f__Peptostreptococcales-Tissierellales | g__Soehngenia |  |
| 33ff6c51ff27529899730bcd6569dead | Bacteria | p__Actinobacteriota | c__Thermoleophilia | o__Gaiellales | f__uncultured | g__uncultured |  |
| 577f5d38b15e43325796c05b3c327a48 | Bacteria | p__Proteobacteria | c__Gammaproteobacteria | o__Burkholderiales | f__A21b | g__A21b |  |
| AB018439 | Bacteria | p__Proteobacteria | c__Alphaproteobacteria | o__Sphingomonadales | f__Sphingomonadaceae | g__Sphingomonas |  |
| c2b9e7b80e7868d118e64306364fbac8 | Bacteria | p__Proteobacteria | c__Gammaproteobacteria | o__Burkholderiales | f__Nitrosomonadaceae | g__mle1-7 |  |
| AB681845 | Bacteria | p__Proteobacteria | c__Gammaproteobacteria | o__Burkholderiales | f__Comamonadaceae | g__Hydrogenophaga |  |
| 9f1d68b5df59732c35a6aa125ee0f997 | Bacteria | p__Chloroflexi | c__Dehalococcoidia | o__S085 | f__S085 | g__S085 | s__uncultured_bacterium |
| d106499cd30350504f725a63b1992284 | Bacteria | p__Acidobacteriota | c__Acidobacteriae | o__Acidobacteriales | f__uncultured | g__uncultured |  |
| GB-GCA-902642655.1-CACQNY010000087.1 | Bacteria | p__Proteobacteria | c__Alphaproteobacteria | o__Acetobacterales | f__Acetobacteraceae | g__Acetobacter |  |
| 175fd1da4ca80d3168c07cca95ce4529 | Bacteria | p__Firmicutes | c__Bacilli | o__Staphylococcales | f__Staphylococcaceae | g__Staphylococcus |  |
| c525caf3271c953e329bfcd5a3e0a48f | Bacteria | p__Actinobacteriota | c__Acidimicrobiia | o__Microtrichales | f__uncultured | g__uncultured |  |
| GB-GCA-016793355.1-JAEUMG010000297.1 | Bacteria | p__Proteobacteria | c__Alphaproteobacteria | o__Rhizobiales | f__Rhizobiales_Incertae_Sedis |  |  |
| 71b9a8919176606e0ec59f6bf08eb691 | Bacteria | p__Firmicutes | c__Bacilli | o__Lactobacillales | f__Lactobacillaceae | g__Lactobacillus | s__Lactobacillus_taiwanensis |
| 1edc06703b32772a4cd4ea289949fa48 | Bacteria | p__Campilobacterota | c__Campylobacteria | o__Campylobacterales | f__Helicobacteraceae | g__Helicobacter |  |
| 02281678 | Bacteria | p__Proteobacteria | c__Alphaproteobacteria | o__Dongiales | f__Dongiaceae | g__Dongia |  |
| LR760817 | Bacteria | p__Proteobacteria | c__Gammaproteobacteria | o__Burkholderiales | f__Burkholderiaceae | g__Burkholderia-Caballeronia-Paraburkholderia |  |
| b9bee41179c05a6587c81a5f299355aa | Bacteria | p__Proteobacteria | c__Gammaproteobacteria | o__Burkholderiales | f__SC-I-84 | g__SC-I-84 |  |
| 04117de922f0aad37eb908c9877ee18b | Bacteria | p__Chloroflexi | c__Anaerolineae | o__Caldilineales | f__Caldilineaceae | g__uncultured |  |
| KP966093 | Bacteria | p__Firmicutes | c__Clostridia | o__Lachnospirales | f__Lachnospiraceae |  |  |
| 25e155eb42fdf222dbf64a1b3346dcc7 | Bacteria | p__Proteobacteria | c__Gammaproteobacteria | o__Burkholderiales | f__Burkholderiaceae | g__Burkholderia-Caballeronia-Paraburkholderia |  |
| 30b063a738f2232eca356f075077c2bc | Bacteria | p__Firmicutes | c__Bacilli | o__Erysipelotrichales | f__Erysipelotrichaceae |  |  |
| ac3e01f9f6cc2f08f4c4e6a0f4c8d944 | Bacteria | p__Chloroflexi | c__Anaerolineae | o__SBR1031 | f__A4b | g__A4b |  |
| e52180fd581933714cb4c1da29b7b816 | Bacteria | p__Actinobacteriota | c__Thermoleophilia | o__Gaiellales |  |  |  |
| fcf527f2646a6664b6844162acfd7a5b | Bacteria | p__Firmicutes | c__Clostridia | o__Oscillospirales | f__UCG-010 | g__UCG-010 |  |
| RS-GCF-004523415.1-NZ-SONK01000098.1 | Bacteria | p__Bacteroidota | c__Bacteroidia | o__Bacteroidales | f__Prevotellaceae | g__Prevotella | s__Prevotella_copri |
| ac73190cefa58852555a0c5b58904781 | Bacteria | p__Proteobacteria | c__Gammaproteobacteria | o__Xanthomonadales | f__Xanthomonadaceae |  |  |
| 0ce7b1bbdbe5967547448965e9814976 | Bacteria | p__Proteobacteria | c__Alphaproteobacteria | o__Elsterales | f__uncultured | g__uncultured | s__uncultured_Stella |
| c5db20ed745e3da2fa9f4b76419cee2a | Bacteria | p__Chloroflexi | c__KD4-96 | o__KD4-96 | f__KD4-96 | g__KD4-96 |  |
| aa763dc977fd8f4e98bb02e7c82e7b58 | Bacteria | p__Acidobacteriota | c__Vicinamibacteria | o__Vicinamibacterales | f__uncultured | g__uncultured |  |
| 0d35f8fe543b9db4e947657907a296c2 | Bacteria | p__Proteobacteria | c__Gammaproteobacteria | o__Burkholderiales | f__Oxalobacteraceae | g__Massilia |  |
| 18458116 | Bacteria | p__Proteobacteria | c__Gammaproteobacteria | o__Burkholderiales | f__Comamonadaceae |  |  |
| 13447510 | Bacteria | p__Proteobacteria | c__Gammaproteobacteria | o__Pasteurellales | f__Pasteurellaceae | g__Muribacter | s__Muribacter_muris |
| a765e33f69907018eb37cf6981887ba8 | Bacteria | p__Proteobacteria | c__Gammaproteobacteria | o__Burkholderiales | f__Comamonadaceae |  |  |
| 05032610 | Bacteria | p__Actinobacteriota | c__Actinobacteria | o__Micrococcales | f__Micrococcaceae | g__Micrococcus |  |
| c380e35bcb42b93e01517b6db4596826 | Bacteria | p__Proteobacteria | c__Gammaproteobacteria | o__Enterobacterales | f__Enterobacteriaceae |  |  |
| RS-GCF-002160145.1-NZ-NFKA01000023.1 | Bacteria | p__Firmicutes | c__Clostridia | o__Oscillospirales | f__Ruminococcaceae | g__Fournierella |  |
| 4a1c2cbc58c885dd5a6f850bf2c08d3c | Bacteria | p__Actinobacteriota | c__Actinobacteria | o__Micrococcales |  |  |  |
| ae06e8214bba61686436a7037a8ff1df | Bacteria | p__Gemmatimonadota | c__Gemmatimonadetes | o__Gemmatimonadales | f__Gemmatimonadaceae | g__Gemmatimonas |  |
| LT222224 | Bacteria | p__Proteobacteria | c__Gammaproteobacteria | o__Xanthomonadales | f__Xanthomonadaceae | g__Stenotrophomonas |  |
| KY053202 | Bacteria | p__Bacteroidota | c__Bacteroidia | o__Sphingobacteriales | f__Sphingobacteriaceae | g__Sphingobacterium | s__Sphingobacterium_corticis |
| 3770ae722af39b77279f36c652078314 | Bacteria | p__Bacteroidota | c__Bacteroidia | o__Sphingobacteriales | f__AKYH767 | g__AKYH767 | s__uncultured_Sphingobacteriales |
| MJ033-1-barcode36-umi16019bins-ubs-19 | Bacteria | p__Acidobacteriota | c__Blastocatellia | o__11-24 | f__11-24 | g__11-24 |  |
| 74b7adc4d1cc448244f1af0762e78e86 | Bacteria | p__Actinobacteriota | c__Actinobacteria | o__Corynebacteriales | f__Mycobacteriaceae | g__Mycobacterium |  |
| f36104051af82481a21925bc1177f325 | Bacteria | p__Proteobacteria | c__Alphaproteobacteria | o__Rhodobacterales | f__Rhodobacteraceae | g__Amaricoccus |  |
| G000725405_1 | Bacteria | p__Actinobacteriota | c__Actinobacteria | o__Micrococcales | f__Dermacoccaceae |  |  |
| 08479901 | Bacteria | p__Proteobacteria | c__Alphaproteobacteria | o__Rhizobiales | f__Rhizobiaceae |  |  |
| aa92fad2a7785a06e2630eeaadc715d7 | Bacteria | p__Firmicutes | c__Bacilli | o__Lactobacillales | f__Enterococcaceae | g__Enterococcus |  |
| ed81109da1629f971afef4bcf8a19ad3 | Bacteria | p__Bacteroidota | c__Bacteroidia | o__Sphingobacteriales | f__Sphingobacteriaceae | g__Sphingobacterium | s__Sphingobacterium_thalpophilum |
| d1d7bdc346904d42bd32f126437ddb98 | Bacteria | p__Firmicutes | c__Bacilli | o__Erysipelotrichales | f__Erysipelotrichaceae | g__Turicibacter |  |
| MJ007-1-barcode42-umi33406bins-ubs-5 | Bacteria | p__Proteobacteria | c__Gammaproteobacteria | o__Pseudomonadales | f__Pseudomonadaceae | g__Pseudomonas | s__Pseudomonas_caeni |
| 95ee125e6ea467d8057acfc487dd1001 | Bacteria |  |  |  |  |  |  |
| 9f5ace83175caf800f267ad061cc7925 | Bacteria | p__Acidobacteriota | c__Vicinamibacteria | o__Subgroup_17 | f__Subgroup_17 | g__Subgroup_17 |  |
| 8167ecea5b7dccb1959cd7fbf7919b73 | Bacteria | p__Bacteroidota | c__Bacteroidia | o__Flavobacteriales | f__Crocinitomicaceae | g__Fluviicola |  |
| 01253323 | Bacteria | p__Bacteroidota | c__Bacteroidia | o__Bacteroidales | f__Rikenellaceae | g__Rikenellaceae_RC9_gut_group |  |
| a90825f873ae2363ff779aff05ee3639 | Bacteria | p__Proteobacteria | c__Gammaproteobacteria | o__Burkholderiales | f__Oxalobacteraceae | g__Massilia |  |
| a16feea748e60c5989cb3ae6ccec4d73 | Bacteria | p__Cyanobacteria | c__Cyanobacteriia |  |  |  |  |
| 05035824 | Bacteria | p__Firmicutes | c__Bacilli | o__Lactobacillales | f__Lactobacillaceae | g__Lactobacillus |  |
| RS-GCF-015910515.1-NZ-JACWNC010000019.1--2 | Bacteria | p__Proteobacteria | c__Gammaproteobacteria | o__Burkholderiales | f__Burkholderiaceae | g__Ralstonia |  |
| 46e0b8a4263b52c4146b0ee605b772a6 | Bacteria | p__Actinobacteriota | c__Thermoleophilia | o__Solirubrobacterales | f__Solirubrobacteraceae |  |  |
| 04900317 | Bacteria | p__Proteobacteria | c__Gammaproteobacteria | o__Pseudomonadales | f__Pseudomonadaceae | g__Pseudomonas |  |
| 02412055 | Bacteria | p__Proteobacteria | c__Alphaproteobacteria | o__Rhizobiales | f__Beijerinckiaceae | g__Bosea |  |
| 17217499 | Bacteria | p__Actinobacteriota | c__Thermoleophilia | o__Solirubrobacterales | f__67-14 | g__67-14 |  |
| 19425594 | Bacteria | p__Firmicutes | c__Bacilli | o__Erysipelotrichales | f__Erysipelotrichaceae | g__ZOR0006 | s__uncultured_organism |
| 8cd627297b8174ba9a55898202e34df9 | Bacteria | p__Chloroflexi | c__Anaerolineae | o__SBR1031 | f__SBR1031 | g__SBR1031 |  |
| 6ac7c13c88da1c896db1a9196bc47d09 | Bacteria | p__Proteobacteria | c__Alphaproteobacteria | o__Sphingomonadales | f__Sphingomonadaceae | g__Novosphingobium |  |
| a6e0a0ec04bc2c2353f29f19c69f5efc | Bacteria | p__Spirochaetota | c__Spirochaetia | o__Spirochaetales | f__Spirochaetaceae | g__Treponema |  |
| 04918149 | Bacteria | p__Bacteroidota | c__Bacteroidia | o__Sphingobacteriales | f__Sphingobacteriaceae | g__Pedobacter |  |
| 02298475 | Bacteria | p__Patescibacteria | c__Saccharimonadia | o__Saccharimonadales | f__Saccharimonadales | g__Saccharimonadales |  |
| 118a830951bcef12d6198d77967b56b1 | Bacteria | p__Actinobacteriota | c__Actinobacteria | o__PeM15 | f__PeM15 | g__PeM15 |  |
| cc7dd173b7a8d45863dcaea85584cf2e | Bacteria | p__Actinobacteriota | c__Actinobacteria | o__Propionibacteriales | f__Nocardioidaceae | g__Nocardioides |  |
| 05271425 | Bacteria | p__Chloroflexi | c__Ktedonobacteria | o__Ktedonobacterales | f__Ktedonobacteraceae | g__FCPS473 | s__uncultured_bacterium |
| 46b1e333cb98bdfc67138b0315abfb69 | Bacteria | p__Actinobacteriota | c__Actinobacteria | o__Frankiales | f__Sporichthyaceae | g__hgcI_clade |  |
| 649d4ca4a3ef3da9c3c600b3684f0e61 | Bacteria | p__Actinobacteriota | c__Actinobacteria | o__Micromonosporales | f__Micromonosporaceae |  |  |
| 818f7a1abe65ad3e42a96e3e465a712d | Bacteria | p__Firmicutes | c__Clostridia | o__Oscillospirales | f__Oscillospiraceae | g__UCG-002 |  |
| 5064e4ae47c5def5bff38acf389e1f7b | Bacteria | p__Actinobacteriota | c__Thermoleophilia | o__Gaiellales |  |  |  |
| 16d4a7fe3399002a111dc9ecc82d862e | Bacteria | p__Chloroflexi | c__Anaerolineae | o__SBR1031 | f__SBR1031 | g__SBR1031 |  |
| 0f675a22455c27865f4414558a09703f | Bacteria | p__Bacteroidota | c__Bacteroidia | o__Bacteroidales | f__Tannerellaceae | g__Macellibacteroides |  |
| def0eb8f0dd204fd015bc6cefccaa2cd | Bacteria | p__Actinobacteriota | c__Actinobacteria | o__Glycomycetales | f__Glycomycetaceae | g__Glycomyces |  |
| 6e745dea34a05d50e694c74e87c2ec97 | Bacteria | p__Firmicutes | c__Clostridia | o__Oscillospirales | f__Ruminococcaceae | g__Faecalibacterium |  |
| d8ea8bc89530141fd720282d9ed57223 | Bacteria | p__Bacteroidota | c__Bacteroidia | o__Chitinophagales | f__Chitinophagaceae | g__Pseudoflavitalea | s__Flavitalea_sp. |
| 2c22092a9aab6fa48bf0250e242f12e4 | Bacteria | p__Bacteroidota | c__Bacteroidia | o__Flavobacteriales | f__Weeksellaceae | g__Elizabethkingia |  |
| aeccea598ef2cf05a4ec4a9c5c034bb9 | Bacteria | p__Proteobacteria | c__Alphaproteobacteria | o__Elsterales | f__uncultured | g__uncultured |  |
| MJ007-2-barcode58-umi1285bins-ubs-352 | Bacteria | p__Firmicutes | c__Clostridia | o__Clostridia_UCG-014 | f__Clostridia_UCG-014 | g__Clostridia_UCG-014 |  |
| 7255d679c3f6fbe846f1cd925c86c579 | Bacteria | p__Acidobacteriota | c__Vicinamibacteria | o__Vicinamibacterales | f__uncultured | g__uncultured |  |
| 04959860 | Bacteria | p__Proteobacteria | c__Gammaproteobacteria | o__Burkholderiales | f__Burkholderiaceae | g__Burkholderia-Caballeronia-Paraburkholderia |  |
| ecde87b6c36035aa3fae5f8dabebd8c3 | Bacteria | p__Actinobacteriota | c__Actinobacteria | o__Streptosporangiales | f__Nocardiopsaceae |  |  |
| AB251884 | Bacteria | p__Bacteroidota | c__Bacteroidia | o__Cytophagales | f__Hymenobacteraceae | g__Hymenobacter |  |
| EF154516 | Bacteria | p__Bacteroidota | c__Bacteroidia | o__Flavobacteriales | f__Weeksellaceae | g__Chryseobacterium |  |
| RS-GCF-009678675.1-NZ-WKMO01000065.1 | Bacteria | p__Bacteroidota | c__Bacteroidia | o__Bacteroidales | f__Tannerellaceae | g__Parabacteroides |  |
| MJ030-1-barcode42-umi82292bins-ubs-6 | Bacteria | p__Verrucomicrobiota | c__Verrucomicrobiae | o__Chthoniobacterales | f__Chthoniobacteraceae | g__Chthoniobacter |  |
| 2c30460526e86e6f3f5ef41f1b10ba6f | Bacteria | p__Actinobacteriota | c__Acidimicrobiia | o__Microtrichales | f__Ilumatobacteraceae | g__uncultured |  |
| 04876525 | Bacteria | p__Bacteroidota | c__Bacteroidia | o__Flavobacteriales | f__Flavobacteriaceae | g__Myroides | s__Myroides_odoratus |
| 11205893 | Bacteria | p__Bacteroidota | c__Bacteroidia | o__Bacteroidales | f__Tannerellaceae | g__Parabacteroides |  |
| MJ007-1-barcode42-umi4441bins-ubs-62 | Bacteria | p__Firmicutes | c__Clostridia | o__Peptostreptococcales-Tissierellales | f__Sedimentibacteraceae | g__Sedimentibacter |  |
| 0ce4fb870d8ac0d547c3f844ab5d2e03 | Bacteria | p__Firmicutes | c__Bacilli | o__Lactobacillales | f__Carnobacteriaceae | g__Trichococcus |  |
| ec9a740fc697390e4fe91d6baab387eb | Bacteria | p__Proteobacteria | c__Alphaproteobacteria | o__Sphingomonadales | f__Sphingomonadaceae | g__Sphingomonas |  |
| f32038d332804af5ab5d40a9ee855ecd | Bacteria | p__Chloroflexi | c__Dehalococcoidia | o__S085 | f__S085 | g__S085 | s__metagenome |
| fe832b733ed155bfc775d02f4a9ea21b | Bacteria | p__Firmicutes | c__Bacilli | o__Erysipelotrichales | f__Erysipelatoclostridiaceae | g__Erysipelatoclostridium | s__uncultured_Clostridium |
| 18930853 | Bacteria | p__Proteobacteria | c__Gammaproteobacteria | o__Burkholderiales | f__Comamonadaceae |  |  |
| 3a84eed954e184abb4fe4c7c04553f80 | Bacteria | p__Actinobacteriota | c__Actinobacteria | o__Pseudonocardiales | f__Pseudonocardiaceae | g__Pseudonocardia |  |
| 252bb3af0347f8a96d5f176cdcd36e8f | Bacteria | p__Firmicutes | c__Limnochordia | o__Limnochordia | f__Limnochordia | g__Hydrogenispora |  |
| b964079237f3acb6f0c0d985ab1e152c | Bacteria | p__Thermotogota | c__Thermotogae | o__Thermotogales | f__Fervidobacteriaceae | g__Fervidobacterium | s__Fervidobacterium_nodosum |
| 09632423 | Bacteria | p__Bacteroidota | c__Bacteroidia | o__Bacteroidales | f__Prevotellaceae |  |  |
| MJ020-1-barcode34-umi54826bins-ubs-9 | Bacteria | p__Chloroflexi | c__Ktedonobacteria | o__C0119 | f__C0119 | g__C0119 | s__uncultured_Chloroflexi |
| d5385434b7b7f408e1c12a0424aeffa2 | Bacteria | p__Firmicutes | c__Bacilli | o__Lactobacillales | f__Enterococcaceae | g__Enterococcus |  |
| f93c57e6050a71d591935d5f6f16eba3 | Bacteria | p__Acidobacteriota | c__Acidobacteriae | o__Acidobacteriales | f__uncultured | g__uncultured |  |
| 4b2e82f982a50a5af411400d7369f925 | Bacteria | p__Proteobacteria | c__Gammaproteobacteria | o__Steroidobacterales | f__Steroidobacteraceae | g__uncultured |  |
| 14899155 | Bacteria | p__Patescibacteria | c__Saccharimonadia | o__Saccharimonadales | f__Saccharimonadaceae | g__Candidatus_Saccharimonas | s__uncultured_bacterium |
| b0d12598027fef5f2bc58bd11aed6f57 | Bacteria | p__Firmicutes | c__Bacilli | o__Bacillales | f__Bacillaceae | g__Bacillus |  |
| AB109887 | Bacteria | p__Proteobacteria | c__Gammaproteobacteria | o__Pseudomonadales | f__Pseudomonadaceae | g__Pseudomonas |  |
| a0e10c5edcfc702c10e6daa2900119de | Bacteria | p__Bacteroidota | c__Bacteroidia | o__Chitinophagales | f__Chitinophagaceae | g__Hydrotalea |  |
| 02247093 | Bacteria | p__Firmicutes | c__Clostridia | o__Eubacteriales | f__Eubacteriaceae | g__uncultured | s__uncultured_bacterium |
| 0214c5e3da71d9f159a877d294736e87 | Bacteria | p__Actinobacteriota | c__Actinobacteria | o__Streptosporangiales | f__Nocardiopsaceae |  |  |
| RS-GCF-003086875.1-NZ-CP025996.1 | Bacteria | p__Proteobacteria | c__Gammaproteobacteria | o__Xanthomonadales | f__Xanthomonadaceae | g__Stenotrophomonas |  |
| a7bf45a1766f722cc1ac5ad37db7057b | Bacteria | p__Proteobacteria | c__Gammaproteobacteria | o__Burkholderiales | f__Alcaligenaceae |  |  |
| MJ034-1-barcode46-umi14503bins-ubs-21 | Bacteria | p__Bacteroidota | c__Bacteroidia | o__Chitinophagales | f__Chitinophagaceae |  |  |
| MJ032-2-barcode66-umi83820bins-ubs-4 | Bacteria | p__Firmicutes | c__Clostridia | o__Clostridia_vadinBB60_group | f__Clostridia_vadinBB60_group | g__Clostridia_vadinBB60_group |  |
| d0be28df7eaf125af09017074d1e24a0 | Bacteria | p__Proteobacteria | c__Alphaproteobacteria | o__Rhizobiales | f__A0839 | g__A0839 |  |
| 35961ff3ae01dc984102b92fd6e69707 | Bacteria | p__Acidobacteriota | c__Acidobacteriae | o__Solibacterales | f__Solibacteraceae | g__Candidatus_Solibacter | s__uncultured_eubacterium |
| b4943a3d9ecf46d693561f919d3b3982 | Bacteria | p__Actinobacteriota | c__Rubrobacteria | o__Rubrobacterales | f__Rubrobacteriaceae | g__Rubrobacter | s__Rubrobacter_bracarensis |
| dfb35dc34a5fea1a2df09592e2a37a18 | Bacteria | p__Proteobacteria | c__Gammaproteobacteria | o__Burkholderiales | f__Nitrosomonadaceae | g__Ellin6067 |  |
| 03fa2ce312f2b289912102ba80796c42 | Bacteria | p__Firmicutes | c__Clostridia | o__Clostridiales | f__Clostridiaceae | g__Clostridium_sensu_stricto_11 | s__Clostridium_senegalense |
| 05038529 | Bacteria | p__Firmicutes | c__Bacilli | o__Lactobacillales | f__Vagococcaceae | g__Vagococcus |  |
| MJ032-2-barcode70-umi77065bins-ubs-5 | Bacteria | p__Bacteroidota | c__Bacteroidia | o__Chitinophagales | f__Saprospiraceae | g__uncultured |  |
| 05037191 | Bacteria | p__Firmicutes | c__Bacilli | o__Bacillales | f__Bacillaceae | g__Bacillus |  |
| KX664462 | Bacteria | p__Proteobacteria | c__Alphaproteobacteria | o__Rhodobacterales | f__Rhodobacteraceae | g__Paracoccus |  |
| AJ227779 | Bacteria | p__Proteobacteria | c__Alphaproteobacteria | o__Caulobacterales | f__Caulobacteraceae | g__Brevundimonas |  |
| 6564cd2c306f8453f757479f61488fe0 | Bacteria | p__Bacteroidota | c__Bacteroidia | o__Sphingobacteriales | f__Sphingobacteriaceae | g__Sphingobacterium |  |
| cea1a3a7c573a6f4aa0b816c5a99b9ed | Bacteria | p__Desulfobacterota | c__Desulfovibrionia | o__Desulfovibrionales | f__Desulfovibrionaceae | g__Desulfovibrio | s__uncultured_bacterium |
| 982405a110302a38b6fb91ab9b19c03f | Bacteria | p__Firmicutes | c__Bacilli | o__Exiguobacterales | f__Exiguobacteraceae | g__Exiguobacterium |  |
| 05007782 | Bacteria | p__Proteobacteria | c__Alphaproteobacteria | o__Sphingomonadales | f__Sphingomonadaceae |  |  |
| 04989595 | Bacteria | p__Proteobacteria | c__Gammaproteobacteria | o__Pseudomonadales | f__Pseudomonadaceae | g__Pseudomonas |  |
| e028f2fb0b246de50192decd1784830c | Bacteria | p__Actinobacteriota | c__Actinobacteria | o__Propionibacteriales | f__Nocardioidaceae | g__Nocardioides |  |
| 88ff823a7410c705347c5845b32471c1 | Bacteria | p__Proteobacteria | c__Gammaproteobacteria | o__Burkholderiales | f__Comamonadaceae | g__Comamonas |  |
| a71fa52537ceb25f822798c12284451e | Bacteria | p__Acidobacteriota | c__Acidobacteriae | o__Subgroup_2 | f__Subgroup_2 | g__Subgroup_2 |  |
| 03ced6d29fb96be0d46ff03b11a0f788 | Bacteria | p__Actinobacteriota | c__Actinobacteria | o__Micrococcales | f__Microbacteriaceae | g__Microbacterium |  |
| G900540175_1 | Bacteria | p__Firmicutes | c__Bacilli | o__Acholeplasmatales | f__Acholeplasmataceae | g__Anaeroplasma | s__uncultured_bacterium |
| 9d1597e36f003b31117da61eab8787b9 | Bacteria | p__Proteobacteria | c__Alphaproteobacteria | o__Elsterales | f__uncultured | g__uncultured |  |
| 59e3d92b2dc61fb80dceb84de5b39bcc | Bacteria | p__Gemmatimonadota | c__Gemmatimonadetes | o__Gemmatimonadales | f__Gemmatimonadaceae |  |  |
| ff71a65abce1c0d7b51d10a8a0e3d16c | Bacteria | p__Proteobacteria | c__Gammaproteobacteria | o__Oceanospirillales | f__Halomonadaceae | g__Halomonas |  |
| 6030be9889f7d9f98e6aaa0212202db3 | Bacteria | p__Bacteroidota | c__Bacteroidia | o__Bacteroidales | f__Tannerellaceae | g__Parabacteroides |  |
| MJ020-1-barcode44-umi28623bins-ubs-15 | Bacteria | p__Bacteroidota | c__Bacteroidia | o__Chitinophagales | f__Chitinophagaceae |  |  |
| RS-GCF-900221015.1-NZ-OBQK01000045.1 | Bacteria | p__Actinobacteriota | c__Actinobacteria | o__Micrococcales | f__Intrasporangiaceae | g__Ornithinimicrobium |  |
| 04950105 | Bacteria | p__Firmicutes | c__Clostridia | o__Lachnospirales | f__Lachnospiraceae | g__Lachnospiraceae_NK4A136_group |  |
| 1873b1deb49872879f8ee39b5a5ae529 | Bacteria | p__Chloroflexi | c__Anaerolineae | o__SJA-15 | f__SJA-15 | g__SJA-15 |  |
| 12ceaa6c6c80e78b1052677412f5e59e | Bacteria | p__Firmicutes | c__Bacilli | o__Lactobacillales | f__Streptococcaceae | g__Streptococcus |  |
| MJ033-1-barcode44-umi11042bins-ubs-27 | Bacteria | p__Bacteroidota | c__Bacteroidia | o__Chitinophagales | f__Chitinophagaceae |  |  |
| MJ006-2-barcode62-umi136306bins-ubs-4 | Bacteria | p__Firmicutes | c__Clostridia | o__Lachnospirales | f__Lachnospiraceae |  |  |
| 04934616 | Bacteria | p__Bacteroidota | c__Bacteroidia | o__Flavobacteriales | f__Weeksellaceae | g__Elizabethkingia |  |
| b2201abf3e43e5c5a44b1d6e76f082d9 | Bacteria | p__Actinobacteriota | c__Acidimicrobiia | o__IMCC26256 | f__IMCC26256 | g__IMCC26256 |  |
| 78801b52b3beda256663d9667b191c0a | Bacteria | p__Proteobacteria | c__Alphaproteobacteria | o__Rhizobiales | f__Beijerinckiaceae |  |  |
| 27f1db173a03ad23d329bb2cc0bc57b0 | Bacteria | p__Proteobacteria | c__Gammaproteobacteria | o__Enterobacterales | f__Enterobacteriaceae |  |  |
| 691e265e441dc9e6b82b43ce125e4d17 | Bacteria | p__Patescibacteria | c__Dojkabacteria | o__Dojkabacteria | f__Dojkabacteria | g__Dojkabacteria |  |
| 04966036 | Bacteria | p__Firmicutes | c__Bacilli | o__Bacillales | f__Bacillaceae | g__Bacillus |  |
| G001945605_1 | Bacteria | p__Firmicutes | c__Bacilli | o__Erysipelotrichales | f__Erysipelotrichaceae | g__Dubosiella | s__Dubosiella_newyorkensis |
| 903f70e9a5a7b609b1ac1a9591461961 | Bacteria | p__Actinobacteriota | c__Actinobacteria |  |  |  |  |
| 1c20d4e2e04364236b570437059b71f6 | Bacteria | p__Firmicutes | c__Bacilli | o__Lactobacillales | f__Enterococcaceae | g__Enterococcus |  |
| 65b2602c20bc208ef2d4fb13a15b145e | Bacteria | p__Proteobacteria | c__Alphaproteobacteria | o__Rhizobiales | f__Hyphomicrobiaceae | g__Pedomicrobium |  |
| 1eb5fc8dced742aabfd49ef326f58959 | Bacteria | p__Actinobacteriota | c__Actinobacteria | o__Frankiales | f__Acidothermaceae | g__Acidothermus |  |
| de0e16ac333eecdf68ea3a8d101f011f | Bacteria | p__Proteobacteria | c__Alphaproteobacteria | o__Rhizobiales | f__Hyphomicrobiaceae | g__Pedomicrobium | s__uncultured_Alphaproteobacteria |
| MJ007-1-barcode25-umi4003bins-ubs-67 | Bacteria | p__Proteobacteria | c__Gammaproteobacteria | o__Burkholderiales | f__Oxalobacteraceae |  |  |
| JX275860 | Bacteria | p__Proteobacteria | c__Alphaproteobacteria | o__Acetobacterales | f__Acetobacteraceae | g__Roseomonas |  |
| 18688359 | Bacteria | p__Actinobacteriota | c__Actinobacteria | o__Pseudonocardiales | f__Pseudonocardiaceae | g__Crossiella | s__uncultured_bacterium |
| 6fc2ec94fd6d21480ce48353aa450d9a | Bacteria | p__Proteobacteria | c__Gammaproteobacteria | o__Pasteurellales | f__Pasteurellaceae |  |  |
| RS-GCF-000474255.1-NZ-BATO01000155.1 | Bacteria | p__Proteobacteria | c__Gammaproteobacteria | o__Pseudomonadales | f__Pseudomonadaceae | g__Pseudomonas |  |
| G000504185_2 | Bacteria | p__Proteobacteria | c__Gammaproteobacteria | o__Pseudomonadales | f__Pseudomonadaceae | g__Pseudomonas |  |
| 884e00213514c847bd5f26e46fd9dac7 | Bacteria | p__Bacteroidota | c__Bacteroidia | o__Flavobacteriales | f__Weeksellaceae | g__Cloacibacterium |  |
| fa3ac6d152ab31d85286b19ea3e9b652 | Bacteria | p__Methylomirabilota | c__Methylomirabilia | o__Rokubacteriales | f__Rokubacteriales | g__Rokubacteriales | s__uncultured_bacterium |
| 9f995305e1abefde8327aa8a02815f89 | Bacteria | p__Proteobacteria | c__Gammaproteobacteria | o__Pseudomonadales | f__Moraxellaceae | g__Acinetobacter |  |
| 72022e0561c1bc17b3b0a8eeced91d44 | Bacteria | p__Planctomycetota | c__Planctomycetes | o__Gemmatales | f__Gemmataceae | g__uncultured |  |
| 05872a572c2e2a6d4196381b9f41bd31 | Bacteria | p__Proteobacteria | c__Gammaproteobacteria | o__Burkholderiales | f__Burkholderiaceae | g__Burkholderia-Caballeronia-Paraburkholderia |  |
| 3eeaa7db35f91be0f53734a80379742e | Bacteria | p__Firmicutes | c__Bacilli | o__Erysipelotrichales | f__Erysipelotrichaceae | g__Turicibacter | s__uncultured_bacterium |
| G000145515_1 | Bacteria | p__Actinobacteriota | c__Actinobacteria | o__Propionibacteriales | f__Propionibacteriaceae | g__Cutibacterium |  |
| MJ009-1-barcode35-umi105850bins-ubs-3 | Bacteria | p__Proteobacteria | c__Alphaproteobacteria | o__Rhizobiales | f__Rhizobiaceae | g__Mesorhizobium |  |
| RS-GCF-013166535.1-NZ-JABKKK010000031.1 | Bacteria | p__Bacteroidota | c__Bacteroidia | o__Bacteroidales | f__Prevotellaceae | g__Prevotellaceae_UCG-001 |  |
| 05039531 | Bacteria | p__Firmicutes | c__Bacilli | o__Alicyclobacillales | f__Alicyclobacillaceae | g__Tumebacillus |  |
| 319cdeecf1dc10134ceacf08e2a34474 | Bacteria | p__Actinobacteriota | c__Actinobacteria | o__Propionibacteriales | f__Nocardioidaceae |  |  |
| 04994418 | Bacteria | p__Proteobacteria | c__Alphaproteobacteria | o__Acetobacterales | f__Acetobacteraceae |  |  |
| AY305310 | Bacteria | p__Firmicutes | c__Clostridia | o__Lachnospirales | f__Lachnospiraceae | g__Roseburia |  |
| c34048ef6d41d92bc6acba1ec2b38427 | Bacteria | p__Myxococcota | c__Polyangia | o__Polyangiales | f__Sandaracinaceae | g__uncultured |  |
| 19103239 | Bacteria | p__Chloroflexi | c__TK10 | o__TK10 | f__TK10 | g__TK10 | s__bacterium_Ellin6519 |
| 04875840 | Bacteria | p__Proteobacteria | c__Gammaproteobacteria | o__Pseudomonadales | f__Moraxellaceae | g__Acinetobacter |  |
| 0a9319c07b58fe8ba12822100ed85183 | Bacteria | p__Proteobacteria | c__Gammaproteobacteria | o__Enterobacterales | f__Enterobacteriaceae |  |  |
| ba7a036ab571ba36329a94c46516a765 | Bacteria | p__NB1-j | c__NB1-j | o__NB1-j | f__NB1-j | g__NB1-j |  |
| 8f9a1804515b2d6f384a3c59764a6312 | Bacteria | p__Proteobacteria | c__Alphaproteobacteria | o__Sphingomonadales | f__Sphingomonadaceae |  |  |
| eb11c63e637cc7195abc49e2396cc5cf | Bacteria | p__Proteobacteria | c__Gammaproteobacteria | o__Thiotrichales | f__Thiotrichaceae | g__Thiothrix |  |
| bb964637aea97a1af57f4b293bd81544 | Bacteria | p__Actinobacteriota | c__Actinobacteria | o__Corynebacteriales | f__Mycobacteriaceae | g__Mycobacterium |  |
| GQ406810 | Bacteria | p__Actinobacteriota | c__Actinobacteria | o__Micrococcales | f__Microbacteriaceae |  |  |
| 9d45c7ae88aafacf4b4d3ba37d9fe0e4 | Bacteria | p__Chloroflexi | c__Chloroflexia | o__Thermomicrobiales | f__JG30-KF-CM45 | g__JG30-KF-CM45 | s__uncultured_bacterium |
| 19cd7406b05cd0518b09dc27ab65e0db | Bacteria | p__Proteobacteria | c__Gammaproteobacteria | o__Enterobacterales | f__Enterobacteriaceae | g__Escherichia-Shigella |  |
| MJ030-2-barcode62-umi36672bins-ubs-18 | Bacteria | p__Verrucomicrobiota | c__Verrucomicrobiae | o__Verrucomicrobiales | f__Rubritaleaceae | g__Luteolibacter |  |
| 04938409 | Bacteria | p__Proteobacteria | c__Gammaproteobacteria | o__Alteromonadales | f__Shewanellaceae | g__Shewanella |  |
| 04958476 | Bacteria | p__Actinobacteriota | c__Actinobacteria | o__Frankiales | f__Geodermatophilaceae | g__Geodermatophilus |  |
| 05060779 | Bacteria | p__Firmicutes | c__Bacilli | o__Staphylococcales | f__Staphylococcaceae | g__Staphylococcus |  |
| aab815ad0a98ed62c2b1b9b560cec0e8 | Bacteria | p__Hydrogenedentes | c__Hydrogenedentia | o__Hydrogenedentiales | f__Hydrogenedensaceae | g__Hydrogenedensaceae | s__uncultured_bacterium |
| ac6a0e577fedca23df19fdd56c4e4920 | Bacteria | p__Proteobacteria | c__Alphaproteobacteria | o__Rhizobiales | f__Rhizobiales_Incertae_Sedis | g__uncultured |  |
| AF000221 | Bacteria | p__Campilobacterota | c__Campylobacteria | o__Campylobacterales | f__Helicobacteraceae | g__Helicobacter |  |
| G000304395_2 | Bacteria | p__Proteobacteria | c__Alphaproteobacteria | o__Rhizobiales | f__Rhizobiaceae |  |  |
| EF174498 | Bacteria | p__Proteobacteria | c__Alphaproteobacteria | o__Rhizobiales | f__Beijerinckiaceae | g__Methylobacterium-Methylorubrum |  |
| 5f3776fe084038a9f6a3447324ea29a8 | Bacteria | p__Proteobacteria | c__Alphaproteobacteria | o__Rhizobiales | f__Xanthobacteraceae | g__Rhodoplanes |  |
| 04956364 | Bacteria | p__Proteobacteria | c__Gammaproteobacteria | o__Burkholderiales | f__Burkholderiaceae | g__Ralstonia |  |
| ec10bc842991fac0d8e719150d1a3aab | Bacteria | p__Bacteroidota | c__Bacteroidia | o__Bacteroidales | f__Muribaculaceae | g__Muribaculaceae | s__uncultured_bacterium |
| 6d85cb0be969e0231befc6472a1c35a8 | Bacteria | p__Proteobacteria | c__Gammaproteobacteria | o__Burkholderiales | f__Alcaligenaceae |  |  |
| a072c554c029f4979b267be74d24aa73 | Bacteria | p__Proteobacteria | c__Gammaproteobacteria | o__Pseudomonadales | f__Moraxellaceae | g__Acinetobacter |  |
| 3daa023a81eeb9a09eedf5cc864106b5 | Bacteria | p__Proteobacteria | c__Gammaproteobacteria | o__Pseudomonadales | f__Moraxellaceae | g__Acinetobacter |  |
| 86f121497ac700fc57bf5f4a3d0d7c48 | Bacteria | p__Acidobacteriota | c__Acidobacteriae | o__Acidobacteriales | f__Koribacteraceae | g__Candidatus_Koribacter |  |
| cb60e67f20490a325b20d7832dd6205e | Bacteria | p__Proteobacteria | c__Gammaproteobacteria | o__Pseudomonadales | f__Moraxellaceae | g__Acinetobacter | s__Acinetobacter_soli |
| f752d84d7a58af469ad131105b4f7da4 | Bacteria | p__Bacteroidota | c__Bacteroidia | o__Flavobacteriales | f__Flavobacteriaceae |  |  |
| 04960655 | Bacteria | p__Actinobacteriota | c__Actinobacteria | o__Corynebacteriales | f__Corynebacteriaceae | g__Lawsonella |  |
| ff3dc839d1432e141e199c62463cad3f | Bacteria | p__Acidobacteriota | c__Acidobacteriae | o__Acidobacteriales | f__Koribacteraceae | g__Candidatus_Koribacter |  |
| 724bfcd7b21a615ea0fbcfdc4b49548c | Bacteria | p__Chloroflexi | c__KD4-96 | o__KD4-96 | f__KD4-96 | g__KD4-96 | s__metagenome |
| MJ010-1-barcode26-umi53998bins-ubs-12 | Bacteria | p__Bacteroidota | c__Bacteroidia | o__Chitinophagales | f__Chitinophagaceae | g__Segetibacter |  |
| 02251023 | Bacteria | p__Cyanobacteria | c__Cyanobacteriia | o__Cyanobacteriales | f__Chroococcidiopsaceae | g__Chroococcidiopsis_PCC_7203 |  |
| 1a872422bea3dc19519697d3628d6c82 | Bacteria | p__Bacteroidota | c__Bacteroidia | o__Bacteroidales | f__Muribaculaceae | g__Muribaculaceae |  |
| 16752333 | Bacteria | p__Firmicutes | c__Clostridia | o__Lachnospirales | f__Lachnospiraceae | g__Lachnoclostridium |  |
| fd0401ca614ef8a07dfc7d1334bc1e6b | Bacteria | p__Bacteroidota | c__Bacteroidia | o__Bacteroidales | f__Bacteroidaceae | g__Bacteroides | s__Bacteroides_salanitronis |
| 40d3974d9d5325b7946273426e45ad93 | Bacteria | p__Proteobacteria | c__Alphaproteobacteria | o__Dongiales | f__Dongiaceae | g__Dongia |  |
| 3fedd52ee1675da95c13de92298b4b05 | Bacteria | p__Proteobacteria | c__Gammaproteobacteria | o__Pseudomonadales | f__Moraxellaceae | g__Acinetobacter |  |
| RS-GCF-016724845.1-NZ-CP068082.1 | Bacteria | p__Bacteroidota | c__Bacteroidia | o__Sphingobacteriales | f__Sphingobacteriaceae | g__Sphingobacterium | s__Sphingobacterium_spiritivorum |
| 04912791 | Bacteria | p__Acidobacteriota | c__Acidobacteriae | o__Acidobacteriales | f__Acidobacteriaceae_(Subgroup_1) | g__uncultured | s__Acidobacteria_bacterium |
| 05002765 | Bacteria | p__Bacteroidota | c__Bacteroidia | o__Sphingobacteriales | f__Sphingobacteriaceae | g__Sphingobacterium |  |
| b86438c5471aeb8fd073569f19e52922 | Bacteria | p__Acidobacteriota | c__Vicinamibacteria | o__Subgroup_17 | f__Subgroup_17 | g__Subgroup_17 |  |
| 02416496 | Bacteria | p__Actinobacteriota | c__Acidimicrobiia | o__Microtrichales | f__Ilumatobacteraceae | g__uncultured | s__uncultured_Acidimicrobiaceae |
| 00f912eccbf200730488e7c5a1822b8f | Bacteria | p__Proteobacteria | c__Alphaproteobacteria | o__Sphingomonadales | f__Sphingomonadaceae |  |  |
| 02243879 | Bacteria | p__Firmicutes | c__Clostridia | o__Clostridiales |  |  |  |
| 7a4ef91636ed3fc34b814dc3e6437b94 | Bacteria | p__Actinobacteriota | c__Acidimicrobiia | o__Microtrichales | f__Microtrichaceae | g__uncultured |  |
| 310fbfbccfb42c94b6a55731973b20a1 | Bacteria | p__Proteobacteria | c__Gammaproteobacteria | o__Burkholderiales | f__SC-I-84 | g__SC-I-84 |  |
| 82e6fc0d0f0fe898bb60469b88e0bda9 | Bacteria | p__Bacteroidota | c__Bacteroidia | o__Flavobacteriales | f__Weeksellaceae | g__Chryseobacterium | s__Chryseobacterium_taeanense |
| afa3d32b9e694d6193f585e8f52bda96 | Bacteria | p__Proteobacteria | c__Alphaproteobacteria | o__Sphingomonadales | f__Sphingomonadaceae | g__Sphingobium |  |
| 01223330bb8e2834dedad12a732801cb | Bacteria | p__Actinobacteriota | c__Actinobacteria | o__Streptosporangiales | f__Thermomonosporaceae | g__Actinoallomurus |  |
| 01254900 | Bacteria | p__Firmicutes | c__Clostridia | o__Lachnospirales | f__Lachnospiraceae | g__Lachnospiraceae_NK4A136_group | s__Clostridiales_bacterium |
| MJ006-2-barcode69-umi170627bins-ubs-3 | Bacteria | p__Firmicutes | c__Clostridia | o__Oscillospirales | f__Ruminococcaceae |  |  |
| 8cb26c9cc5aef9ed2b6142bf5dfeeb0a | Bacteria | p__Proteobacteria | c__Gammaproteobacteria | o__Burkholderiales | f__Rhodocyclaceae |  |  |
| 868da32e4f79f0101759dd6eaea14066 | Bacteria | p__Firmicutes | c__Bacilli | o__Lactobacillales | f__Enterococcaceae | g__Enterococcus |  |
| 16c2ca3ec0f573ed9bdf51c19ddf7565 | Bacteria | p__Verrucomicrobiota | c__Chlamydiae | o__Chlamydiales | f__Parachlamydiaceae |  |  |
| 13948224 | Bacteria | p__Armatimonadota | c__Chthonomonadetes | o__Chthonomonadales | f__Chthonomonadales | g__Chthonomonadales | s__uncultured_bacterium |
| cfc8925c2e563f44b184892ae2841d68 | Bacteria | p__Firmicutes | c__Clostridia | o__Oscillospirales | f__Ruminococcaceae | g__Faecalibacterium | s__uncultured_bacterium |
| b9d8451785a11b5afe3fd49ccf5f366a | Bacteria | p__Actinobacteriota | c__Actinobacteria | o__Propionibacteriales | f__Nocardioidaceae |  |  |
| 1e2b17579ef7cc1d2092b99490e15fab | Bacteria | p__Bacteroidota | c__Bacteroidia | o__Bacteroidales | f__Muribaculaceae | g__Muribaculaceae | s__mouse_gut |
| 921ba2f2f720e9ae84abcf88cb0e88ea | Bacteria | p__Actinobacteriota | c__Acidimicrobiia |  |  |  |  |
| 9c82b95db8b6cdecdbdaff7314ca7fa3 | Bacteria | p__Proteobacteria | c__Alphaproteobacteria | o__Caulobacterales | f__Caulobacteraceae | g__Phenylobacterium | s__Phenylobacterium_hankyongense |
| 1bdc4ae94c4eaa51a57a1831876242fa | Bacteria | p__Chloroflexi | c__TK10 | o__TK10 | f__TK10 | g__TK10 |  |
| e86eb479b1a3edb605aa0aa00ff2984c | Bacteria | p__Proteobacteria | c__Gammaproteobacteria | o__Burkholderiales | f__Comamonadaceae |  |  |
| 02858463 | Bacteria | p__Chloroflexi | c__Chloroflexia | o__Chloroflexales | f__Roseiflexaceae | g__uncultured | s__uncultured_bacterium |
| 04909400 | Bacteria | p__Proteobacteria | c__Alphaproteobacteria | o__Rhizobiales | f__Kaistiaceae | g__Kaistia |  |
| fcce62f5f0efdd28a8c7b9bb607fbc59 | Bacteria | p__Firmicutes | c__Bacilli | o__Erysipelotrichales | f__Erysipelatoclostridiaceae | g__Erysipelatoclostridium | s__uncultured_Clostridium |
| 06814840 | Bacteria | p__Planctomycetota | c__Phycisphaerae | o__Tepidisphaerales | f__CPla-3_termite_group | g__CPla-3_termite_group | s__metagenome |
| MJ033-1-barcode32-umi10292bins-ubs-28 | Bacteria | p__Acidobacteriota | c__Acidobacteriae | o__Solibacterales | f__Solibacteraceae | g__Candidatus_Solibacter |  |
| dc5a6c8e24515a4198f2ca4e6bc9f572 | Bacteria | p__Bacteroidota | c__Bacteroidia | o__Bacteroidales | f__Muribaculaceae | g__Muribaculaceae |  |
| MJ006-2-barcode69-umi153123bins-ubs-3 | Bacteria | p__Bacteroidota | c__Bacteroidia | o__Bacteroidales | f__Bacteroidaceae | g__Bacteroides |  |
| ffa314ff65e99272b5c34f346f2517be | Bacteria | p__Proteobacteria | c__Gammaproteobacteria | o__Steroidobacterales | f__Steroidobacteraceae | g__uncultured |  |
| RS-GCF-900104255.1-NZ-FNKZ01000001.1--2 | Bacteria | p__Proteobacteria | c__Gammaproteobacteria | o__Xanthomonadales | f__Xanthomonadaceae | g__Pseudoxanthomonas |  |
| f6ce64ec9fe5c3d63fa2ed7310bb630e | Bacteria | p__Proteobacteria | c__Alphaproteobacteria | o__Caulobacterales | f__Caulobacteraceae |  |  |
| c228abda728469457a19627ea22f1633 | Bacteria | p__Proteobacteria | c__Alphaproteobacteria | o__Sphingomonadales | f__Sphingomonadaceae |  |  |
| 09489ae4ef70d4b2864732d0ee9c9de6 | Bacteria | p__Bacteroidota | c__Bacteroidia | o__Chitinophagales | f__Chitinophagaceae | g__Edaphobaculum | s__uncultured_Sphingobacterium |
| af7d3bec183f0085a48fd481c1a54301 | Bacteria | p__Proteobacteria | c__Gammaproteobacteria | o__Steroidobacterales | f__Steroidobacteraceae | g__Steroidobacter |  |
| 92b216e73f3b33a1db45cef4932273f7 | Bacteria | p__Chloroflexi | c__Anaerolineae | o__1-20 | f__1-20 | g__1-20 | s__uncultured_bacterium |
| 06701016 | Bacteria | p__Gemmatimonadota | c__Gemmatimonadetes | o__Gemmatimonadales | f__Gemmatimonadaceae | g__uncultured |  |
| c70b8a7f8e6b6840468626d76617c208 | Bacteria | p__Bacteroidota | c__Bacteroidia | o__Flavobacteriales | f__Weeksellaceae | g__Chryseobacterium |  |
| ca1dcbbfea260d49a3697828d8d11233 | Bacteria | p__Firmicutes | c__Clostridia | o__Oscillospirales | f__Ruminococcaceae | g__Faecalibacterium |  |
| b618b762131e0b14ac6aa1740d0b5ac2 | Bacteria | p__Proteobacteria | c__Gammaproteobacteria | o__Pseudomonadales | f__Moraxellaceae | g__Acinetobacter |  |
| KC806052 | Bacteria | p__Actinobacteriota | c__Actinobacteria | o__Micrococcales | f__Micrococcaceae | g__Rothia |  |
| 2c28a89423bcb383947fc956fab8946e | Bacteria | p__Proteobacteria | c__Alphaproteobacteria | o__Sphingomonadales | f__Sphingomonadaceae | g__Sphingomonas |  |
| f3c2b57c7d36d94ec5fc5b3c326905c9 | Bacteria | p__Chloroflexi | c__KD4-96 | o__KD4-96 | f__KD4-96 | g__KD4-96 | s__uncultured_Anaerolineaceae |
| 6d405bca4320b4da01c0245f7f3c7712 | Bacteria | p__Proteobacteria | c__Alphaproteobacteria | o__Rhizobiales | f__Xanthobacteraceae |  |  |
| 205a110bfa951fd755ad392ceb2fb8d5 | Bacteria | p__Actinobacteriota | c__Actinobacteria | o__Corynebacteriales | f__Mycobacteriaceae | g__Mycobacterium |  |
| 5fea4f19dd34ce276eaa7c18c365f3e1 | Bacteria | p__Actinobacteriota | c__Thermoleophilia | o__uncultured | f__uncultured | g__uncultured | s__uncultured_bacterium |
| bc12cd6945dc0b6bc4801d766f923076 | Bacteria | p__Firmicutes | c__Bacilli | o__Bacillales | f__Marinococcaceae | g__Alteribacillus |  |
| 04945765 | Bacteria | p__Actinobacteriota | c__Actinobacteria | o__Micrococcales | f__Micrococcaceae | g__Paenarthrobacter |  |
| 12828420 | Bacteria | p__Bacteroidota | c__Bacteroidia | o__Chitinophagales | f__Chitinophagaceae | g__Hydrotalea | s__uncultured_bacterium |
| f1452bb94634822a8aedd9d49796f4d9 | Bacteria | p__Bacteroidota | c__Bacteroidia | o__Bacteroidales | f__Bacteroidaceae | g__Bacteroides |  |
| a65bccb43ccbe43c3dff5d69200d8e2d | Bacteria | p__Proteobacteria | c__Gammaproteobacteria | o__Enterobacterales | f__Enterobacteriaceae | g__Klebsiella |  |
| cd86620f42cad2b7dc304eda05921031 | Bacteria | p__Proteobacteria | c__Alphaproteobacteria | o__Rhodobacterales | f__Rhodobacteraceae | g__Rhodobacter |  |
| 7fd5655b80baa67ba36e73463c4b2872 | Bacteria | p__Actinobacteriota | c__MB-A2-108 | o__MB-A2-108 | f__MB-A2-108 | g__MB-A2-108 |  |
| 5cf2bc3ca8550b6940f08ae7898ebb77 | Bacteria | p__Firmicutes | c__Bacilli | o__Staphylococcales | f__Staphylococcaceae | g__Staphylococcus |  |
| 03739182 | Bacteria | p__Planctomycetota | c__Planctomycetes | o__Pirellulales | f__Pirellulaceae | g__uncultured |  |
| f449b5c3a2f4ce876c7525018e34bf79 | Bacteria | p__Proteobacteria | c__Alphaproteobacteria | o__Rhizobiales | f__Rhizobiaceae | g__Allorhizobium-Neorhizobium-Pararhizobium-Rhizobium |  |
| a8200a573262ce2b3995472f43b4a5fb | Bacteria | p__Proteobacteria | c__Gammaproteobacteria | o__Burkholderiales | f__Comamonadaceae | g__Lampropedia | s__Lampropedia_sp. |
| 4c16357b9c6f367c21fa5151bdea8d51 | Bacteria | p__Proteobacteria | c__Alphaproteobacteria | o__Caulobacterales | f__Parvularculaceae | g__Amphiplicatus |  |
| c44f6ceeba45834d3265c3bd71d88e5f | Bacteria | p__Proteobacteria | c__Alphaproteobacteria | o__Acetobacterales | f__Acetobacteraceae |  |  |
| Z78212 | Bacteria | p__Actinobacteriota | c__Actinobacteria | o__Propionibacteriales | f__Nocardioidaceae | g__Nocardioides |  |
| HM587321 | Bacteria | p__Firmicutes | c__Clostridia | o__Peptostreptococcales-Tissierellales | f__Peptostreptococcales-Tissierellales | g__Fenollaria |  |
| 46affea945c71630bf07664c519b9aae | Bacteria | p__Bacteroidota | c__Bacteroidia | o__Flavobacteriales | f__Blattabacteriaceae | g__Candidatus_Sulcia | s__Candidatus_Sulcia |
| 07175727 | Bacteria | p__Planctomycetota | c__Phycisphaerae | o__Tepidisphaerales | f__WD2101_soil_group | g__WD2101_soil_group | s__uncultured_bacterium |
| 8abd6ef9e47b4a598c5a63bd8a944254 | Bacteria | p__Proteobacteria | c__Gammaproteobacteria | o__Burkholderiales | f__Comamonadaceae | g__Delftia |  |
| 04318158 | Bacteria | p__WPS-2 | c__WPS-2 | o__WPS-2 | f__WPS-2 | g__WPS-2 |  |
| MJ030-2-barcode65-umi31483bins-ubs-21 | Bacteria | p__Acidobacteriota | c__Acidobacteriae | o__Solibacterales | f__Solibacteraceae | g__Candidatus_Solibacter |  |
| 7f3660caef7efb12eb234d30dbc68da2 | Bacteria | p__Bacteroidota | c__Bacteroidia | o__Chitinophagales | f__Chitinophagaceae | g__Puia |  |
| MJ032-2-barcode60-umi38130bins-ubs-14 | Bacteria | p__Proteobacteria | c__Gammaproteobacteria | o__Pseudomonadales | f__Moraxellaceae | g__Acinetobacter |  |
| 04899059 | Bacteria | p__Verrucomicrobiota | c__Verrucomicrobiae | o__Opitutales | f__Opitutaceae |  |  |
| 8bd8f7087d47c8ba0a15e3e88d015b0a | Bacteria | p__Firmicutes | c__Bacilli | o__Lactobacillales | f__Enterococcaceae | g__Enterococcus |  |
| 8b62a13bab81637cf4d197a6c47fd73d | Bacteria | p__Gemmatimonadota | c__Gemmatimonadetes | o__Gemmatimonadales | f__Gemmatimonadaceae | g__uncultured |  |
| 68e42528b0e8f4d6c927da53f4182d19 | Bacteria | p__Firmicutes | c__Clostridia | o__Oscillospirales | f__Oscillospiraceae |  |  |
| 14944822 | Bacteria | p__Chloroflexi |  |  |  |  |  |
| RS-GCF-900291485.1-NZ-OLKO01000008.1 | Bacteria | p__Firmicutes | c__Clostridia | o__Oscillospirales | f__Ruminococcaceae | g__Ruminococcus |  |
| 1f8abba43148f6291ff6ad441762cd2c | Bacteria | p__WPS-2 | c__WPS-2 | o__WPS-2 | f__WPS-2 | g__WPS-2 | s__uncultured_bacterium |
| edfe10221dc0d791fd9dfec2c3489cfa | Bacteria | p__Proteobacteria | c__Alphaproteobacteria | o__Sphingomonadales | f__Sphingomonadaceae | g__Sphingomonas |  |
| RS-GCF-001883325.1-NZ-MEAC01000038.1 | Bacteria | p__Proteobacteria | c__Gammaproteobacteria | o__Enterobacterales | f__Yersiniaceae |  |  |
| 17309288 | Bacteria | p__Actinobacteriota | c__Thermoleophilia | o__Solirubrobacterales | f__67-14 | g__67-14 |  |
| 05013849 | Bacteria | p__Bacteroidota | c__Bacteroidia | o__Flavobacteriales | f__Weeksellaceae | g__Elizabethkingia |  |
| 04995328 | Bacteria | p__Proteobacteria | c__Alphaproteobacteria | o__Azospirillales | f__Azospirillaceae | g__Skermanella |  |
| 2d654878b416e3903426f5fd96354a9f | Bacteria | p__Actinobacteriota | c__Actinobacteria | o__Corynebacteriales | f__Nocardiaceae | g__Rhodococcus |  |
| 9aa729b195be56e8bd697b690c14056b | Bacteria | p__Bacteroidota | c__Bacteroidia | o__Flavobacteriales | f__Weeksellaceae | g__Chryseobacterium |  |
| b17025b7dd7d53a04eed4dae7011f6b8 | Bacteria | p__Actinobacteriota | c__Acidimicrobiia | o__Microtrichales | f__uncultured | g__uncultured |  |
| RS-GCF-011019565.1-NZ-SXFL01000145.1 | Bacteria | p__Firmicutes | c__Clostridia | o__Clostridiales | f__Clostridiaceae | g__Clostridium_sensu_stricto_1 |  |
| feaf5c0f527df57bf7adad91b27e7de6 | Bacteria | p__Proteobacteria | c__Alphaproteobacteria | o__Rhizobiales | f__Xanthobacteraceae | g__Rhodoplanes |  |
| 739c11358963d4f0082076ea95f140b0 | Bacteria | p__Acidobacteriota | c__Acidobacteriae | o__Acidobacteriales |  |  |  |
| 06760398 | Bacteria | p__Myxococcota | c__Myxococcia | o__Myxococcales | f__Anaeromyxobacteraceae | g__Anaeromyxobacter | s__uncultured_delta |
| eb5695ecb27f153f91e7398362d710f2 | Bacteria | p__Acidobacteriota | c__Vicinamibacteria | o__Vicinamibacterales | f__uncultured | g__uncultured |  |
| 72bda1ec17a49db43ed4d7df95c4412e | Bacteria | p__Firmicutes | c__Bacilli | o__Lactobacillales | f__Enterococcaceae | g__Enterococcus |  |
| 2c9cf1b22f6a9a9871b221743f8b81c7 | Bacteria | p__Planctomycetota | c__Planctomycetes | o__Gemmatales | f__Gemmataceae | g__uncultured |  |
| edd1e733131fa7b49715b292a02b9ec3 | Bacteria | p__Firmicutes | c__Bacilli | o__Lactobacillales | f__Lactobacillaceae | g__Lactobacillus |  |
| e6d55b79510f6d8306814f61fb6c4282 | Bacteria | p__Proteobacteria | c__Gammaproteobacteria | o__Enterobacterales |  |  |  |
| LC438514 | Bacteria | p__Firmicutes | c__Bacilli | o__Lactobacillales | f__Enterococcaceae | g__Enterococcus |  |
| 141c5b266ca8c01765fc2fc9db3848a1 | Bacteria | p__Firmicutes | c__Bacilli | o__Lactobacillales | f__Enterococcaceae | g__Enterococcus |  |
| e846c5dac4167e1592f12fe00507c309 | Bacteria | p__Proteobacteria | c__Alphaproteobacteria | o__Rhizobiales | f__Devosiaceae | g__Devosia |  |
| EF472955 | Bacteria | p__Actinobacteriota | c__Actinobacteria | o__Propionibacteriales | f__Nocardioidaceae | g__Kribbella |  |
| 345f5c2e05a2d87d68aa856bc6886efa | Bacteria | p__Actinobacteriota | c__Rubrobacteria | o__Rubrobacterales | f__Rubrobacteriaceae | g__Rubrobacter | s__metagenome |
| a688b7cfdb5558864be35eb8970929f9 | Bacteria | p__Proteobacteria | c__Alphaproteobacteria | o__Acetobacterales | f__Acetobacteraceae |  |  |
| MJ010-1-barcode30-umi116319bins-ubs-5 | Bacteria | p__Bacteroidota | c__Bacteroidia | o__Bacteroidales | f__Prevotellaceae | g__Prevotella |  |
| RS-GCF-002902045.1-NZ-PPQP01000093.1 | Bacteria | p__Firmicutes | c__Bacilli | o__Staphylococcales | f__Staphylococcaceae | g__Staphylococcus | s__Staphylococcus_succinus |
| KY114945 | Bacteria | p__Proteobacteria | c__Gammaproteobacteria | o__Burkholderiales | f__Alcaligenaceae | g__Parapusillimonas | s__Paracandidimonas_soli |
| ae4e71a2c3a194967cc83041d57e28af | Bacteria | p__Proteobacteria | c__Alphaproteobacteria | o__Rhodobacterales | f__Rhodobacteraceae | g__Paracoccus |  |
| c53c584d96f6c6bf56067f38806f7c18 | Bacteria | p__Proteobacteria | c__Alphaproteobacteria | o__Rhizobiales | f__Rhizobiaceae |  |  |
| 430fda30a4e6288a289b38c9995a0982 | Bacteria | p__Actinobacteriota | c__Actinobacteria |  |  |  |  |
| db17b4949def90aa64d1a6539b1ba439 | Bacteria | p__Proteobacteria | c__Alphaproteobacteria | o__Rhizobiales | f__Methyloligellaceae | g__uncultured |  |
| 15b3809697c0528e5a4f968cb9413ce9 | Bacteria | p__Acidobacteriota | c__Acidobacteriae | o__Bryobacterales | f__Bryobacteraceae | g__Bryobacter |  |
| 06759090ca8ad7664721e673ce57c69b | Bacteria | p__Actinobacteriota | c__Actinobacteria | o__Micrococcales | f__Micrococcaceae |  |  |
| MJ006-2-barcode72-umi112070bins-ubs-5 | Bacteria | p__Bacteroidota | c__Bacteroidia | o__Bacteroidales | f__Bacteroidaceae | g__Bacteroides | s__Bacteroides_massiliensis |
| 02307359 | Bacteria | p__Proteobacteria | c__Alphaproteobacteria | o__Caulobacterales | f__Caulobacteraceae | g__Caulobacter |  |
| d6452aa0fe83b22b9c61a6f6922f5e15 | Bacteria | p__Proteobacteria | c__Alphaproteobacteria | o__Sphingomonadales | f__Sphingomonadaceae | g__Sphingomonas |  |
| 484b80ca44453f57f0fb3ad253f85861 | Bacteria | p__Proteobacteria | c__Alphaproteobacteria | o__Reyranellales | f__Reyranellaceae | g__Reyranella |  |
| f3b709acc89dcce299c9307c12020752 | Bacteria | p__Chloroflexi | c__Ktedonobacteria | o__Ktedonobacterales | f__JG30-KF-AS9 | g__JG30-KF-AS9 | s__uncultured_bacterium |
| 874801a3c23de4fb039857ef25e7920e | Bacteria | p__Actinobacteriota | c__Thermoleophilia | o__Gaiellales | f__Gaiellaceae | g__Gaiella |  |
| EU604272 | Bacteria | p__Proteobacteria | c__Gammaproteobacteria | o__Xanthomonadales | f__Rhodanobacteraceae |  |  |
| 070eea3161747c8f9af641cfe0a33ae8 | Bacteria | p__Actinobacteriota | c__Actinobacteria | o__Glycomycetales | f__Glycomycetaceae | g__Glycomyces | s__Glycomyces_mongolensis |
| 05741415 | Bacteria | p__Chloroflexi | c__Chloroflexia | o__Chloroflexales | f__Roseiflexaceae | g__uncultured |  |
| a9ffda15c8e8cfd7d77e9cd9a74e41fd | Bacteria | p__Proteobacteria | c__Alphaproteobacteria | o__Sphingomonadales | f__Sphingomonadaceae | g__Sphingomonas |  |
| 206f4ec9a2d0d41113e0c7910ed0ecbc | Bacteria | p__Bacteroidota | c__Bacteroidia | o__Flavobacteriales | f__Weeksellaceae | g__Chryseobacterium |  |
| MJ034-1-barcode41-umi25347bins-ubs-5 | Bacteria | p__Cyanobacteria | c__Cyanobacteriia | o__Cyanobacteriales | f__Coleofasciculaceae | g__Microcoleus_PCC-7113 | s__Microcoleus_sp. |
| daf0722a29692a46640a6176c9a4cc24 | Bacteria | p__Proteobacteria | c__Alphaproteobacteria | o__Elsterales | f__uncultured | g__uncultured |  |
| RS-GCF-901212445.1-NZ-CAAKNW010000110.1 | Bacteria | p__Bacteroidota | c__Bacteroidia | o__Bacteroidales | f__Bacteroidaceae | g__Bacteroides |  |
| b3416c1d89183590dd3eaba1453f8865 | Bacteria | p__Actinobacteriota | c__Actinobacteria | o__Corynebacteriales | f__Corynebacteriaceae | g__Corynebacterium | s__Corynebacterium_sputi |
| dc6290b3b73c78e5605bc79d0cdb9533 | Bacteria | p__Firmicutes | c__Bacilli | o__Lactobacillales | f__Streptococcaceae | g__Streptococcus |  |
| f0ae1ff23d1274caf0d90e44ed618ff8 | Bacteria | p__Gemmatimonadota | c__Gemmatimonadetes | o__Gemmatimonadales | f__Gemmatimonadaceae | g__uncultured |  |
| 4c80ce2590eb0a4c9ba84b5a7fc2b905 | Bacteria | p__Bacteroidota | c__Bacteroidia | o__Bacteroidales | f__Muribaculaceae | g__Muribaculaceae |  |
| 04902629 | Bacteria | p__Verrucomicrobiota | c__Verrucomicrobiae | o__Chthoniobacterales | f__Chthoniobacteraceae | g__Chthoniobacter | s__uncultured_bacterium |
| RS-GCF-000257765.1-NZ-AICP01000064.1 | Bacteria | p__Actinobacteriota | c__Actinobacteria | o__Micrococcales | f__Micrococcaceae | g__Micrococcus |  |
| e3d90c5df4a504688ad29543c731e947 | Bacteria | p__Firmicutes | c__Clostridia | o__Oscillospirales | f__Ruminococcaceae | g__Ruminococcus |  |
| fa842427c97920117122c72c209c4492 | Bacteria | p__Firmicutes | c__Bacilli | o__Erysipelotrichales | f__Erysipelatoclostridiaceae | g__Erysipelatoclostridium | s__uncultured_Clostridium |
| a4a01066da760b800f5baba01c82a57b | Bacteria | p__Proteobacteria | c__Gammaproteobacteria | o__Legionellales | f__Legionellaceae | g__Legionella |  |
| c166c90b9fe77b729e5ae297eae52b6c | Bacteria | p__Chloroflexi | c__TK10 | o__TK10 | f__TK10 | g__TK10 |  |
| 11876694 | Bacteria | p__Proteobacteria | c__Alphaproteobacteria | o__Sphingomonadales | f__Sphingomonadaceae | g__Sphingomonas |  |
| G001439965_1 | Bacteria | p__Firmicutes | c__Bacilli | o__Bacillales | f__Bacillaceae | g__Bacillus | s__Bacillus_oleronius |
| 02923468 | Bacteria | p__Planctomycetota | c__Phycisphaerae | o__Tepidisphaerales | f__WD2101_soil_group | g__WD2101_soil_group |  |
| bf3564ac65212c5c39f3d0255e2bf7ce | Bacteria | p__Synergistota | c__Synergistia | o__Synergistales | f__Synergistaceae | g__uncultured | s__uncultured_Synergistes |
| 09661696 | Bacteria | p__Entotheonellaeota | c__Entotheonellia | o__Entotheonellales | f__Entotheonellaceae | g__Candidatus_Entotheonella | s__uncultured_delta |
| 250f81a33ee15466affee3b6fa6cfd4c | Bacteria | p__Bacteroidota | c__Bacteroidia | o__Sphingobacteriales | f__Sphingobacteriaceae |  |  |
| 9396e1f9ecb89dd09706153e2d5c9dc9 | Bacteria | p__Firmicutes | c__Bacilli | o__Lactobacillales | f__Streptococcaceae | g__Streptococcus |  |
| 02411497 | Bacteria | p__Proteobacteria | c__Alphaproteobacteria | o__Rhizobiales | f__Devosiaceae |  |  |
| AJ005447 | Bacteria | p__Proteobacteria | c__Gammaproteobacteria | o__Burkholderiales | f__Alcaligenaceae | g__Castellaniella | s__Castellaniella_defragrans |
| c06604e56624086da8ac74abf84371ca | Bacteria | p__Bacteroidota | c__Bacteroidia | o__Bacteroidales | f__Muribaculaceae | g__Muribaculaceae |  |
| ed6eb34358d768e0de3dfc32f2756b09 | Bacteria | p__Acidobacteriota | c__Acidobacteriae | o__Acidobacteriales | f__uncultured | g__uncultured |  |
| 04904763 | Bacteria | p__Planctomycetota | c__Planctomycetes | o__Planctomycetales | f__Rubinisphaeraceae | g__SH-PL14 |  |
| e148c8d656026db21202f27a44975c8d | Bacteria | p__Bacteroidota | c__Bacteroidia | o__Bacteroidales | f__Muribaculaceae | g__Muribaculaceae | s__uncultured_bacterium |
| 08796317 | Bacteria | p__Proteobacteria | c__Gammaproteobacteria | o__Xanthomonadales | f__Xanthomonadaceae | g__Stenotrophomonas | s__Stenotrophomonas_acidaminiphila |
| MJ006-1-barcode27-umi145382bins-ubs-3 | Bacteria | p__Firmicutes | c__Clostridia | o__Oscillospirales | f__Ruminococcaceae |  |  |
| e28ddc5f9dda018b5c48689c9309101c | Bacteria | p__Proteobacteria | c__Alphaproteobacteria | o__Elsterales | f__uncultured | g__uncultured |  |
| ee4c6446479a090cab89d91390b49130 | Bacteria | p__Actinobacteriota | c__Thermoleophilia | o__Gaiellales | f__Gaiellaceae | g__Gaiella |  |
| a2973402f9390bdcca1540a8abf291eb | Bacteria | p__Actinobacteriota | c__Thermoleophilia | o__Solirubrobacterales | f__Solirubrobacteraceae |  |  |
| RS-GCF-003392415.1-NZ-QSXC01000285.1 | Bacteria | p__Proteobacteria | c__Gammaproteobacteria | o__Xanthomonadales | f__Xanthomonadaceae | g__Stenotrophomonas | s__Stenotrophomonas_rhizophila |
| 412ce04529b4f9476d26f3b358d9591d | Bacteria | p__Chloroflexi | c__Anaerolineae | o__Caldilineales | f__Caldilineaceae | g__uncultured | s__Chloroflexi_bacterium |
| 04945769 | Bacteria | p__Actinobacteriota | c__Actinobacteria | o__Micrococcales | f__Micrococcaceae |  |  |
| 16135901 | Bacteria | p__Actinobacteriota | c__Acidimicrobiia | o__IMCC26256 | f__IMCC26256 | g__IMCC26256 | s__uncultured_Ferrimicrobium |
| 5e81c85a47970077e5aeba59d40a351b | Bacteria | p__Firmicutes | c__Bacilli | o__Lactobacillales | f__Enterococcaceae | g__Enterococcus |  |
| 555476fd669423a572b18e6763c7a3a1 | Bacteria | p__Bacteroidota | c__Bacteroidia | o__Chitinophagales | f__Chitinophagaceae |  |  |
| db947ae0ac49bc00b2336477b5398ce9 | Bacteria | p__Firmicutes | c__Bacilli | o__Paenibacillales | f__Paenibacillaceae | g__Paenibacillus |  |
| 05742147 | Bacteria | p__Chloroflexi | c__Chloroflexia | o__Chloroflexales | f__Roseiflexaceae | g__uncultured |  |
| e1994435f1e2f444e3989ada4c66c8e8 | Bacteria | p__Firmicutes | c__Clostridia | o__Lachnospirales | f__Lachnospiraceae | g__Blautia |  |
| 09653083 | Bacteria | p__Entotheonellaeota | c__Entotheonellia | o__Entotheonellales | f__Entotheonellaceae | g__Entotheonellaceae | s__uncultured_bacterium |
| MJ030-2-barcode58-umi89384bins-ubs-5 | Bacteria | p__Proteobacteria | c__Gammaproteobacteria | o__Burkholderiales | f__Comamonadaceae |  |  |
| 06436906 | Bacteria | p__Acidobacteriota | c__Acidobacteriae | o__Acidobacteriales | f__Koribacteraceae | g__Candidatus_Koribacter |  |
| DQ442494 | Bacteria | p__Actinobacteriota | c__Actinobacteria | o__Streptomycetales | f__Streptomycetaceae | g__Streptomyces |  |
| MF078077 | Bacteria | p__Proteobacteria | c__Gammaproteobacteria | o__Pseudomonadales | f__Pseudomonadaceae |  |  |
| ca3a478147f252ffbc2431404e4ef9ae | Bacteria | p__Firmicutes | c__Bacilli | o__Lactobacillales | f__Enterococcaceae | g__Enterococcus |  |
| MJ010-2-barcode64-umi85975bins-ubs-6 | Bacteria | p__Proteobacteria | c__Gammaproteobacteria | o__Enterobacterales | f__Enterobacteriaceae |  |  |
| AB544079 | Bacteria | p__Actinobacteriota | c__Actinobacteria | o__Propionibacteriales | f__Nocardioidaceae | g__Nocardioides |  |
| 64809078d155198b3b16e171f316ac20 | Bacteria | p__Acidobacteriota | c__Vicinamibacteria | o__Vicinamibacterales | f__Vicinamibacteraceae | g__Vicinamibacteraceae |  |
| 9ab1fcf948c57af566f8bfa7f3f2598b | Bacteria | p__Bacteroidota | c__Bacteroidia | o__Bacteroidales | f__Bacteroidaceae | g__Bacteroides | s__Bacteroides_graminisolvens |
| 66e2f90156652b7149e09c487782af39 | Bacteria | p__Actinobacteriota | c__Actinobacteria | o__Pseudonocardiales | f__Pseudonocardiaceae | g__Prauserella |  |
| beee7a198b2b0381c40ee528890fd64c | Bacteria | p__Proteobacteria | c__Gammaproteobacteria | o__Burkholderiales | f__Comamonadaceae |  |  |
| e36591708f368a0291e8f3521262cb95 | Bacteria | p__Actinobacteriota | c__Acidimicrobiia | o__Acidimicrobiales | f__Acidimicrobiaceae | g__uncultured | s__uncultured_bacterium |
| 10275804 | Bacteria | p__Bacteroidota | c__Bacteroidia | o__Sphingobacteriales | f__Sphingobacteriaceae | g__Sphingobacterium | s__Sphingobacterium_spiritivorum |
| e5c801bca95f257b675aa23b2de6139b | Bacteria | p__Proteobacteria | c__Gammaproteobacteria | o__Burkholderiales | f__Comamonadaceae |  |  |
| RS-GCF-017315785.1-NZ-JAFLRE010000044.1 | Bacteria | p__Proteobacteria | c__Gammaproteobacteria | o__Burkholderiales | f__Chromobacteriaceae | g__Chromobacterium |  |
| CP001726 | Bacteria | p__Actinobacteriota | c__Coriobacteriia | o__Coriobacteriales | f__Eggerthellaceae | g__Eggerthella |  |
| 43cc8d3e1f32a9366df43d0fa56cef9f | Bacteria | p__Actinobacteriota | c__Acidimicrobiia | o__Microtrichales | f__Ilumatobacteraceae | g__CL500-29_marine_group |  |
| 03362631 | Bacteria | p__Planctomycetota | c__Planctomycetes | o__Pirellulales | f__Pirellulaceae | g__Pirellula | s__uncultured_Planctomycetales |
| G006740705_1 | Bacteria | p__Firmicutes | c__Bacilli | o__Bacillales | f__Marinococcaceae | g__Natribacillus | s__Ruegeria_sp. |
| 974b8347707160de912eda346023b717 | Bacteria | p__Firmicutes | c__Clostridia | o__Clostridia_UCG-014 | f__Clostridia_UCG-014 | g__Clostridia_UCG-014 |  |
| MJ006-2-barcode63-umi115073bins-ubs-5 | Bacteria | p__Bacteroidota | c__Bacteroidia | o__Bacteroidales | f__Rikenellaceae | g__Alistipes |  |
| dbec18a968ae6d541153a14970e24a67 | Bacteria | p__Proteobacteria | c__Gammaproteobacteria | o__Xanthomonadales | f__Xanthomonadaceae | g__Pseudoxanthomonas |  |
| 04960996 | Bacteria | p__Actinobacteriota | c__Actinobacteria | o__Corynebacteriales | f__Mycobacteriaceae | g__Mycobacterium |  |
| 29e35d2d3cff1dc4d6a96f294c20548c | Bacteria | p__Chloroflexi | c__Chloroflexia | o__Thermomicrobiales | f__JG30-KF-CM45 | g__JG30-KF-CM45 |  |
| 20e147a67fad5dd8f52047c7b2d401b3 | Bacteria | p__Bacteroidota | c__Bacteroidia | o__Flavobacteriales | f__NS9_marine_group | g__NS9_marine_group |  |
| b30c4dcba65279c5a1b378447263d424 | Bacteria | p__Proteobacteria | c__Alphaproteobacteria | o__Rhodospirillales | f__uncultured | g__uncultured | s__uncultured_Magnetospirillum |
| 12846605 | Bacteria | p__Bacteroidota | c__Bacteroidia | o__Chitinophagales | f__Chitinophagaceae |  |  |
| c52a31dfa36dbd8f86775d0186294a93 | Bacteria | p__Actinobacteriota | c__Acidimicrobiia | o__Microtrichales | f__Microtrichaceae | g__uncultured |  |
| 83eb8972fa06232becec43654672f5a3 | Bacteria | p__Proteobacteria | c__Gammaproteobacteria | o__Pseudomonadales | f__Moraxellaceae | g__Acinetobacter |  |
| MJ006-2-barcode61-umi1042bins-ubs-74 | Bacteria | p__Firmicutes | c__Bacilli | o__RF39 | f__RF39 | g__RF39 | s__gut_metagenome |
| RS-GCF-001949725.1-NZ-MASC01000067.1 | Bacteria | p__Firmicutes | c__Clostridia | o__Clostridiales | f__Clostridiaceae |  |  |
| 764d9871b47ba2d1e1d2b7d5d5d27ff7 | Bacteria | p__Firmicutes | c__Bacilli | o__Erysipelotrichales | f__Erysipelatoclostridiaceae | g__Erysipelatoclostridium | s__uncultured_Clostridium |
| 04989830 | Bacteria | p__Proteobacteria | c__Gammaproteobacteria | o__Pseudomonadales | f__Moraxellaceae | g__Acinetobacter |  |
| 487f015472dc7a1ab155008e431529ff | Bacteria | p__Firmicutes | c__Bacilli | o__Bacillales | f__Bacillaceae | g__Geobacillus |  |
| 87ed69afaef46ec92fcb6d70271014da | Bacteria | p__Desulfobacterota | c__uncultured | o__uncultured | f__uncultured | g__uncultured |  |
| 20332538 | Bacteria | p__Firmicutes | c__Bacilli | o__Bacillales | f__Bacillaceae | g__Bacillus |  |
| d89a8252d25ef6ae2988e695d1e82aa9 | Bacteria | p__Armatimonadota | c__Armatimonadia | o__Armatimonadales | f__Armatimonadaceae | g__Armatimonas | s__uncultured_beta |
| 04919077 | Bacteria | p__Bacteroidota | c__Bacteroidia | o__Sphingobacteriales | f__Sphingobacteriaceae | g__Sphingobacterium |  |
| 39f423407b7e7598f3de98e13fe10d9a | Bacteria | p__Proteobacteria | c__Gammaproteobacteria | o__Burkholderiales | f__Oxalobacteraceae | g__Candidatus_Vidania | s__Candidatus_Vidania |
| 54a3ac4a1ec3dd91dd7ead77323d7dff | Bacteria | p__Firmicutes | c__Bacilli | o__Staphylococcales | f__Staphylococcaceae | g__Staphylococcus |  |
| c5d39e8b427f566f4bda38145bf9eeeb | Bacteria | p__Actinobacteriota | c__Actinobacteria | o__Corynebacteriales | f__Mycobacteriaceae | g__Mycobacterium |  |
| 02416797 | Bacteria | p__Actinobacteriota | c__Coriobacteriia | o__Coriobacteriales | f__Coriobacteriaceae | g__Collinsella |  |
| 02361030 | Bacteria | p__Firmicutes | c__Clostridia | o__Oscillospirales | f__Oscillospiraceae | g__Oscillibacter |  |
| AY126616 | Bacteria | p__Bacteroidota | c__Bacteroidia | o__Bacteroidales | f__Bacteroidaceae | g__Bacteroides | s__Bacteroides_massiliensis |
| 09194304 | Bacteria | p__Planctomycetota | c__Planctomycetes | o__Pirellulales | f__Pirellulaceae | g__Pirellula |  |
| 9cf1ab4fe2d2354f53fe7ac3b316bda1 | Bacteria | p__Proteobacteria | c__Alphaproteobacteria | o__Rhizobiales | f__A0839 | g__A0839 | s__uncultured_proteobacterium |
| RS-GCF-001274725.1-NZ-LGUF01000011.1 | Bacteria | p__Firmicutes | c__Bacilli | o__Bacillales | f__Bacillaceae | g__Bacillus |  |
| HM037994 | Bacteria | p__Proteobacteria | c__Alphaproteobacteria | o__Rhizobiales | f__Pleomorphomonadaceae | g__Prosthecomicrobium |  |
| fbe105905b9603be8719165c791d754a | Bacteria | p__Actinobacteriota | c__Thermoleophilia | o__Gaiellales | f__uncultured | g__uncultured |  |
| 04909490 | Bacteria | p__Proteobacteria | c__Alphaproteobacteria | o__Azospirillales | f__Azospirillaceae | g__Azospirillaceae | s__Rhodocista_sp. |
| 02247627 | Bacteria | p__Actinobacteriota | c__Actinobacteria | o__Propionibacteriales | f__Nocardioidaceae | g__Nocardioides |  |
| c744eb3bcfcee79060d6d8cd73003568 | Bacteria | p__Proteobacteria | c__Alphaproteobacteria | o__Rhodobacterales | f__Rhodobacteraceae |  |  |
| ae7a1fecdf9bc4f7509bc48459b53ebd | Bacteria | p__Actinobacteriota | c__Actinobacteria | o__Corynebacteriales | f__Corynebacteriaceae | g__Corynebacterium |  |
| RS-GCF-000693835.1-NZ-KK737140.1--4 | Bacteria | p__Proteobacteria | c__Gammaproteobacteria | o__Enterobacterales | f__Enterobacteriaceae | g__Klebsiella |  |
| MJ030-2-barcode59-umi110917bins-ubs-3 | Bacteria | p__Actinobacteriota | c__Actinobacteria | o__Corynebacteriales | f__Corynebacteriaceae | g__Lawsonella |  |
| 7fcdc7d3a9544cc4604c45606b9471dc | Bacteria | p__Bacteroidota | c__Bacteroidia | o__Flavobacteriales | f__Flavobacteriaceae | g__Flavobacterium | s__Flavobacterium_haoranii |
| GB-GCA-018000395.1-JAGNJY010000040.1 | Bacteria | p__Bacteroidota | c__Bacteroidia | o__Sphingobacteriales | f__AKYH767 | g__AKYH767 |  |
| 0d29f73d88b446c5670dec58a45c7835 | Bacteria | p__Proteobacteria | c__Gammaproteobacteria |  |  |  |  |
| 04993467 | Bacteria | p__Proteobacteria | c__Alphaproteobacteria | o__Rhizobiales | f__Beijerinckiaceae | g__Methylobacterium-Methylorubrum |  |
| a3d968280ca6dfd03042bc5638614ee7 | Bacteria | p__Proteobacteria | c__Gammaproteobacteria | o__Alteromonadales | f__Alteromonadaceae | g__Rheinheimera |  |
| 27c1444abf540485345f326c59e122a6 | Bacteria | p__Acidobacteriota | c__Vicinamibacteria | o__Vicinamibacterales | f__uncultured | g__uncultured |  |
| JF806526 | Bacteria | p__Actinobacteriota | c__Actinobacteria | o__Corynebacteriales | f__Nocardiaceae | g__Gordonia |  |
| 2eb8c6a1a738f5ed54bba110ca5d09b1 | Bacteria | p__Chloroflexi | c__Anaerolineae |  |  |  |  |
| 11440312 | Bacteria | p__Proteobacteria | c__Alphaproteobacteria | o__Sphingomonadales | f__Sphingomonadaceae |  |  |
| 23e9990942babc9a77ed56b5c0c9b34a | Bacteria | p__Actinobacteriota | c__MB-A2-108 | o__MB-A2-108 | f__MB-A2-108 | g__MB-A2-108 | s__uncultured_bacterium |
| 078c34c8e40fa54397a7393db9b956e3 | Bacteria | p__Proteobacteria | c__Gammaproteobacteria | o__Burkholderiales | f__Nitrosomonadaceae | g__MND1 |  |
| 9a50859e19e19523960aba62de2a4887 | Bacteria | p__Firmicutes | c__Clostridia | o__Lachnospirales | f__Lachnospiraceae | g__[Eubacterium]_hallii_group |  |
| MJ034-1-barcode29-umi2014bins-ubs-218 | Bacteria | p__Proteobacteria | c__Gammaproteobacteria | o__Burkholderiales | f__Oxalobacteraceae |  |  |
| RS-GCF-001682865.1-NZ-MAQX01000033.1 | Bacteria | p__Firmicutes | c__Bacilli | o__Paenibacillales | f__Paenibacillaceae | g__Paenibacillus |  |
| 6b73bff92de63b8c3a228d7a16926d87 | Bacteria | p__Proteobacteria | c__Gammaproteobacteria | o__Burkholderiales | f__Oxalobacteraceae | g__Herbaspirillum |  |
| d77ac7952b47e90412215621aadf8241 | Bacteria | p__Actinobacteriota | c__Acidimicrobiia | o__Microtrichales | f__Ilumatobacteraceae | g__CL500-29_marine_group |  |
| 7808b440f87c917e40f3c7a2477b92da | Bacteria | p__Actinobacteriota | c__Actinobacteria | o__Pseudonocardiales | f__Pseudonocardiaceae | g__Pseudonocardia |  |
| 576619e6d35f9bd2fa4061557c50ad8a | Bacteria | p__Firmicutes | c__Bacilli | o__Lactobacillales | f__Enterococcaceae | g__Enterococcus |  |
| f290e381db541f75360169eab3b417af | Bacteria | p__Firmicutes | c__Limnochordia | o__M55-D21 | f__M55-D21 | g__M55-D21 | s__uncultured_bacterium |
| 2e2e9b11c9c483443b40e7dd8b993611 | Bacteria | p__Firmicutes | c__Desulfitobacteriia | o__Desulfitobacteriales | f__Heliobacteriaceae | g__uncultured | s__uncultured_bacterium |
| 4c072cd39f62abdd0f31e995e7c4eeb9 | Bacteria | p__Bacteroidota | c__Bacteroidia | o__Sphingobacteriales | f__Sphingobacteriaceae | g__Sphingobacterium | s__Sphingobacterium_multivorum |
| 6c17eff9e0dff98507e8f9612b85c3ab | Bacteria | p__Actinobacteriota | c__Actinobacteria | o__Corynebacteriales | f__Mycobacteriaceae | g__Mycobacterium |  |
| G000988325_1 | Bacteria | p__Bacteroidota | c__Bacteroidia | o__Sphingobacteriales | f__Sphingobacteriaceae | g__Sphingobacterium | s__Sphingobacterium_multivorum |
| 04956371 | Bacteria | p__Proteobacteria | c__Gammaproteobacteria | o__Burkholderiales | f__Burkholderiaceae | g__Ralstonia |  |
| 5c2f3cfc9247e30d466c37e1e5e16a73 | Bacteria | p__Cyanobacteria | c__Cyanobacteriia | o__Leptolyngbyales | f__Leptolyngbyaceae | g__Leptolyngbya_Es-Yyy1000 | s__Leptolyngbya_cf. |
| 2662e0d3a385a20aff3cfbca7835fa8c | Bacteria | p__Proteobacteria | c__Alphaproteobacteria | o__uncultured | f__uncultured | g__uncultured | s__Rhodospirillaceae_bacterium |
| 0d5e0a540b2726e9cf92ec8fba075a01 | Bacteria | p__Proteobacteria | c__Gammaproteobacteria | o__Pseudomonadales | f__Pseudomonadaceae | g__Pseudomonas |  |
| AJ535710 | Bacteria | p__Proteobacteria | c__Alphaproteobacteria | o__Azospirillales | f__Azospirillales_Incertae_Sedis | g__Stella |  |
| 17787929 | Bacteria |  |  |  |  |  |  |
| 04963004 | Bacteria | p__Firmicutes | c__Bacilli | o__Lactobacillales | f__Streptococcaceae | g__Lactococcus |  |
| c3b6a8d159a464deecf8e30f77b3c799 | Bacteria | p__Firmicutes | c__Bacilli | o__Bacillales | f__Planococcaceae | g__Chryseomicrobium |  |
| 2d365dfb31f418b997e2020659c1899b | Bacteria | p__Proteobacteria | c__Alphaproteobacteria | o__Rhizobiales | f__Xanthobacteraceae |  |  |
| 04876101 | Bacteria | p__Proteobacteria | c__Alphaproteobacteria | o__Rhizobiales | f__Xanthobacteraceae | g__Bradyrhizobium |  |
| 5b24e4494f0f6781cb4f808b3f1b72fb | Bacteria | p__Proteobacteria | c__Gammaproteobacteria | o__Burkholderiales | f__Burkholderiaceae | g__Burkholderia-Caballeronia-Paraburkholderia | s__Paraburkholderia_caledonica |
| b18b6a8de6b3069ed388c3b275d1a040 | Bacteria | p__Bacteroidota | c__Bacteroidia | o__Bacteroidales | f__Muribaculaceae | g__Muribaculaceae |  |
| 6b243cf761ddfe16309618e7fb1a6b5f | Bacteria |  |  |  |  |  |  |
| 08325646 | Bacteria | p__Proteobacteria | c__Alphaproteobacteria | o__Caulobacterales | f__Caulobacteraceae | g__Caulobacter |  |
| 697fc68e024a8ea1004f8f370259f673 | Bacteria | p__Chloroflexi | c__Anaerolineae | o__Caldilineales | f__Caldilineaceae | g__uncultured |  |
| 4c490ce2614429ae879ba6b469899575 | Bacteria | p__Verrucomicrobiota | c__Verrucomicrobiae | o__Chthoniobacterales | f__Chthoniobacteraceae | g__Candidatus_Udaeobacter |  |
| KM199639 | Bacteria | p__Actinobacteriota | c__Actinobacteria | o__Propionibacteriales | f__Nocardioidaceae | g__Marmoricola |  |
| 865ffd35e987172f40fdad85aaa51187 | Bacteria | p__Proteobacteria | c__Gammaproteobacteria | o__Enterobacterales | f__Erwiniaceae |  |  |
| 05132150 | Bacteria | p__Chloroflexi | c__KD4-96 | o__KD4-96 | f__KD4-96 | g__KD4-96 |  |
| 01309cc398adff36f3e04dfeb01f8b1b | Bacteria | p__Proteobacteria | c__Gammaproteobacteria | o__Steroidobacterales | f__Woeseiaceae | g__JTB255_marine_benthic_group | s__uncultured_bacterium |
| 3f79e52237f06474b6488612bb8e4227 | Bacteria | p__Firmicutes | c__Clostridia | o__Lachnospirales | f__Lachnospiraceae | g__Blautia |  |
| GB-GCA-003158025.1-PMGD01000030.1 | Bacteria | p__Actinobacteriota | c__Actinobacteria | o__Corynebacteriales | f__Mycobacteriaceae | g__Mycobacterium |  |
| 19712294 | Bacteria | p__Firmicutes | c__Bacilli | o__Erysipelotrichales | f__Erysipelotrichaceae | g__Ileibacterium | s__Ileibacterium_valens |
| ce8f3ff3840ae408f67a70c1e1020250 | Bacteria | p__Acidobacteriota | c__Holophagae | o__Subgroup_7 | f__Subgroup_7 | g__Subgroup_7 | s__uncultured_bacterium |
| 17963785 | Bacteria | p__Chloroflexi | c__Ktedonobacteria | o__Ktedonobacterales | f__Ktedonobacteraceae | g__1959-1 | s__uncultured_bacterium |
| LC522510 | Bacteria | p__Firmicutes | c__Clostridia | o__Oscillospirales | f__Oscillospiraceae | g__UCG-002 |  |
| 05124641 | Bacteria | p__Proteobacteria | c__Alphaproteobacteria | o__Sphingomonadales | f__Sphingomonadaceae | g__Sphingomonas |  |
| 0d2f26cc45606b768f4cc4d320a50cb8 | Bacteria | p__Firmicutes | c__Bacilli | o__Bacillales | f__Bacillaceae | g__Bacillus |  |
| 1ae5b2cd5949488f383148827ae53711 | Bacteria | p__Proteobacteria | c__Gammaproteobacteria | o__Pseudomonadales | f__Moraxellaceae | g__Acinetobacter |  |
| 08173959 | Bacteria | p__Proteobacteria | c__Alphaproteobacteria | o__Rhizobiales | f__Xanthobacteraceae | g__uncultured |  |
| 761a49e13c41995b7c8c5bdab33f0349 | Bacteria | p__Proteobacteria | c__Gammaproteobacteria | o__Burkholderiales |  |  |  |
| RS-GCF-014138775.1-NZ-JACJHT010000014.1 | Bacteria | p__Firmicutes | c__Bacilli | o__Bacillales | f__Bacillaceae | g__Bacillus |  |
| 1c5412b29a005daf4c40a0df589303d6 | Bacteria | p__Proteobacteria | c__Gammaproteobacteria | o__Burkholderiales | f__Comamonadaceae |  |  |
| 06415322 | Bacteria | p__Myxococcota | c__Myxococcia | o__Myxococcales | f__Myxococcaceae |  |  |
| 1968e70c2f8db88720744f96d0a19c33 | Bacteria | p__NB1-j | c__NB1-j | o__NB1-j | f__NB1-j | g__NB1-j | s__uncultured_Desulfuromonadales |
| fd4d3a18fba4dd58ace0c4fcda8e4e83 | Bacteria | p__Firmicutes | c__Bacilli | o__Paenibacillales | f__Paenibacillaceae | g__Saccharibacillus |  |
| 0211ebae762e457d9f522e1b3602476b | Bacteria | p__Actinobacteriota | c__Thermoleophilia | o__Solirubrobacterales | f__Solirubrobacteraceae | g__Solirubrobacter |  |
| 04925430 | Bacteria | p__Proteobacteria | c__Alphaproteobacteria | o__Sphingomonadales | f__Sphingomonadaceae | g__Sphingobium |  |
| bdb8424e838fc5ddfb8b9409a5570d1f | Bacteria | p__Actinobacteriota | c__Actinobacteria | o__Actinomycetales | f__Actinomycetaceae | g__Actinomyces |  |
| G003479885_1 | Bacteria | p__Firmicutes | c__Clostridia | o__Lachnospirales | f__Lachnospiraceae |  |  |
| 46da1324a552a777cae8a2a09cdcf0a0 | Bacteria | p__Actinobacteriota | c__Actinobacteria |  |  |  |  |
| 88b58140f5c0b1c8c67e11a8eafa7a8a | Bacteria | p__Proteobacteria | c__Alphaproteobacteria | o__Rhizobiales | f__Beijerinckiaceae |  |  |
| 10acde8401a6da3943c2df9daa6c19be | Bacteria | p__Myxococcota | c__bacteriap25 | o__bacteriap25 | f__bacteriap25 | g__bacteriap25 |  |
| f977fd51a0fbd1b0e785c2485e6a1283 | Bacteria | p__Actinobacteriota | c__Actinobacteria |  |  |  |  |
| EF424391 | Bacteria | p__Proteobacteria | c__Alphaproteobacteria | o__Sphingomonadales | f__Sphingomonadaceae | g__Sphingopyxis |  |
| 05197375 | Bacteria | p__Gemmatimonadota | c__S0134_terrestrial_group | o__S0134_terrestrial_group | f__S0134_terrestrial_group | g__S0134_terrestrial_group | s__uncultured_Gemmatimonadetes |
| 197e922f1556aba5dc5cf0a4c7cb7480 | Bacteria | p__Actinobacteriota | c__Actinobacteria | o__Pseudonocardiales | f__Pseudonocardiaceae | g__Actinomycetospora |  |
| 02695322 | Bacteria | p__Actinobacteriota | c__Actinobacteria | o__Propionibacteriales | f__Nocardioidaceae | g__Marmoricola |  |
| KT724336 | Bacteria | p__Firmicutes | c__Bacilli | o__Bacillales | f__Planococcaceae | g__Domibacillus |  |
| 9ef6874294b0aa5ab3bbb446fae12cb3 | Bacteria | p__Firmicutes | c__Bacilli | o__Lactobacillales | f__Vagococcaceae | g__Vagococcus |  |
| MJ034-1-barcode41-umi4903bins-ubs-91 | Bacteria | p__Proteobacteria | c__Gammaproteobacteria | o__Burkholderiales | f__Comamonadaceae | g__Variovorax |  |
| 8aafdc2c568714c8f68ab7daa3aa9bab | Bacteria | p__Proteobacteria | c__Alphaproteobacteria | o__Rhizobiales | f__Beijerinckiaceae | g__Methylobacterium-Methylorubrum |  |
| d45ed3358cbdd061a70b838a03f0d67f | Bacteria | p__Gemmatimonadota | c__Gemmatimonadetes | o__Gemmatimonadales | f__Gemmatimonadaceae | g__uncultured | s__uncultured_Alphaproteobacteria |
| cdb9be3ae90ef7605d031e5e4b0ad3df | Bacteria | p__Actinobacteriota | c__Actinobacteria | o__Micrococcales | f__Microbacteriaceae | g__Leucobacter |  |
| MJ030-1-barcode25-umi43912bins-ubs-10 | Bacteria | p__Firmicutes | c__Bacilli | o__Bacillales | f__Bacillaceae | g__Bacillus |  |
| MJ006-2-barcode71-umi265477bins-ubs-3 | Bacteria | p__Firmicutes | c__Clostridia | o__Lachnospirales | f__Lachnospiraceae | g__Blautia |  |
| EU204978 | Bacteria | p__Bacteroidota | c__Bacteroidia | o__Flavobacteriales | f__Flavobacteriaceae | g__Myroides |  |
| 03827416 | Bacteria | p__Myxococcota | c__bacteriap25 | o__bacteriap25 | f__bacteriap25 | g__bacteriap25 |  |
| ad5172d7fbfd258dc1d865f79b3ad6a3 | Bacteria | p__Proteobacteria | c__Gammaproteobacteria | o__Xanthomonadales | f__Xanthomonadaceae | g__Luteimonas |  |
| 18973080 | Bacteria | p__Proteobacteria | c__Gammaproteobacteria | o__Burkholderiales | f__Nitrosomonadaceae | g__Ellin6067 |  |
| c5f4e10fb1a8c162471e6be49921ae4f | Bacteria | p__Proteobacteria | c__Alphaproteobacteria | o__Rhizobiales | f__Beijerinckiaceae |  |  |
| 5e8c591558ac6cd7305504a46e29ff83 | Bacteria | p__Firmicutes | c__Bacilli | o__Lactobacillales | f__Enterococcaceae | g__Enterococcus |  |
| 0db09a87e00eb8c347d86ddd09980b4b | Bacteria | p__Bacteroidota | c__Bacteroidia | o__Flavobacteriales | f__Weeksellaceae | g__Chryseobacterium |  |
| aca64b702c0cb20f55ee149c58b185fd | Bacteria | p__Planctomycetota | c__Phycisphaerae | o__Tepidisphaerales | f__WD2101_soil_group | g__WD2101_soil_group | s__uncultured_bacterium |
| 69b89b90421ca1eec18be0f372632f7d | Bacteria | p__Proteobacteria | c__Gammaproteobacteria | o__Burkholderiales | f__Comamonadaceae |  |  |
| 62f34710c67db84a86699a3d5929a153 | Bacteria | p__Proteobacteria | c__Gammaproteobacteria |  |  |  |  |
| 15e19555f0324479bff14b4036dbba78 | Bacteria | p__Proteobacteria | c__Gammaproteobacteria | o__Xanthomonadales | f__Rhodanobacteraceae |  |  |
| FR733689 | Bacteria | p__Firmicutes | c__Bacilli | o__Bacillales | f__Bacillaceae | g__Bacillus |  |
| 036399aa6c96f0a0335fb41c19b4d8b7 | Bacteria | p__Acidobacteriota | c__Vicinamibacteria | o__Vicinamibacterales | f__Vicinamibacteraceae |  |  |
| ce0deb10191465464c22a5585ee477c1 | Bacteria | p__Firmicutes | c__Clostridia | o__Lachnospirales | f__Lachnospiraceae | g__Dorea |  |
| 7527cc770e5821da5c12fe9101f34203 | Bacteria | p__Firmicutes | c__Bacilli | o__Erysipelotrichales | f__Erysipelotrichaceae | g__ZOR0006 |  |
| 763975b9a362839ffb6ea676e0c29a8f | Bacteria | p__Chloroflexi | c__Anaerolineae | o__Caldilineales | f__Caldilineaceae | g__uncultured | s__metagenome |
| MJ034-1-barcode41-umi23296bins-ubs-6 | Bacteria | p__Acidobacteriota | c__Holophagae | o__Subgroup_7 | f__Subgroup_7 | g__Subgroup_7 |  |
| 02247127 | Bacteria | p__Actinobacteriota | c__Actinobacteria | o__Propionibacteriales | f__Propionibacteriaceae | g__Micropruina |  |
| 0dd529751df03bc7b97657ddc1b36add | Bacteria | p__Proteobacteria | c__Gammaproteobacteria | o__Thiotrichales | f__Thiotrichaceae | g__Thiothrix |  |
| KJ769177 | Bacteria | p__Proteobacteria | c__Gammaproteobacteria | o__Xanthomonadales | f__Xanthomonadaceae | g__Luteimonas |  |
| MJ010-1-barcode47-umi114444bins-ubs-5 | Bacteria | p__Proteobacteria | c__Gammaproteobacteria | o__Burkholderiales | f__Comamonadaceae |  |  |
| 3bc7c471442a6aac66885a4d9b0aaddb | Bacteria | p__Proteobacteria | c__Alphaproteobacteria | o__Rhizobiales | f__Rhizobiales_Incertae_Sedis |  |  |
| a640817d72633cc1bab4914b9b4e1dc6 | Bacteria | p__Acidobacteriota | c__Vicinamibacteria | o__Vicinamibacterales | f__uncultured | g__uncultured |  |
| MJ010-1-barcode29-umi121271bins-ubs-4 | Bacteria | p__Proteobacteria | c__Gammaproteobacteria | o__Pseudomonadales | f__Moraxellaceae | g__Acinetobacter |  |
| 3bf8c89af55596437580b9fcd17bae03 | Bacteria | p__Chloroflexi | c__Anaerolineae | o__SBR1031 | f__A4b | g__A4b |  |
| d9462d2e36c9b5a0e34a2209ce01254c | Bacteria | p__Proteobacteria | c__Gammaproteobacteria | o__Burkholderiales | f__Nitrosomonadaceae | g__Ellin6067 |  |
| f510312a6c2f47912cf9e7f1e47d4f70 | Bacteria | p__Bacteroidota | c__Bacteroidia | o__Chitinophagales | f__Chitinophagaceae | g__Ferruginibacter |  |
| 73693173a1f0bdccacf1ac139ff247e9 | Bacteria | p__Chloroflexi | c__Anaerolineae | o__Caldilineales | f__Caldilineaceae |  |  |
| JQ659512 | Bacteria | p__Proteobacteria | c__Alphaproteobacteria | o__Sphingomonadales | f__Sphingomonadaceae | g__Sphingomonas |  |
| RS-GCF-003935825.1-NZ-RHPZ01000002.1 | Bacteria | p__Proteobacteria | c__Gammaproteobacteria | o__Xanthomonadales | f__Xanthomonadaceae | g__Stenotrophomonas |  |
| AY884571 | Bacteria | p__Proteobacteria | c__Gammaproteobacteria | o__Xanthomonadales | f__Rhodanobacteraceae |  |  |
| 08491286 | Bacteria | p__Proteobacteria | c__Alphaproteobacteria | o__Rhizobiales | f__Rhizobiaceae | g__Aureimonas |  |
| b5ddf6becc79f8aa84ad3634a22f5dde | Bacteria | p__Chloroflexi | c__Gitt-GS-136 | o__Gitt-GS-136 | f__Gitt-GS-136 | g__Gitt-GS-136 |  |
| 6d24c0e6ca2aa42aa909596be3062e0d | Bacteria | p__Proteobacteria | c__Gammaproteobacteria | o__Burkholderiales | f__Comamonadaceae | g__Ottowia | s__uncultured_bacterium |
| AY676115 | Bacteria | p__Proteobacteria | c__Alphaproteobacteria | o__Sphingomonadales | f__Sphingomonadaceae |  |  |
| 0db94b29410478bfe698fb2970522456 | Bacteria | p__Proteobacteria | c__Gammaproteobacteria | o__Gammaproteobacteria_Incertae_Sedis | f__Unknown_Family | g__Acidibacter |  |
| 2fd7383ff9968523c976fa79b4b86538 | Bacteria | p__Myxococcota | c__Polyangia | o__Haliangiales | f__Haliangiaceae | g__Haliangium | s__uncultured_Myxococcales |
| 110144c980c68077c66d6ff1b9c95b06 | Bacteria | p__Firmicutes | c__Clostridia | o__Clostridia_UCG-014 | f__Clostridia_UCG-014 | g__Clostridia_UCG-014 |  |
| G900316605_1 | Bacteria | p__Firmicutes | c__Clostridia | o__Oscillospirales | f__[Eubacterium]_coprostanoligenes_group | g__[Eubacterium]_coprostanoligenes_group |  |
| 218dc0d011310a35a045f4862443e3b5 | Bacteria | p__Proteobacteria | c__Alphaproteobacteria | o__Caulobacterales | f__Caulobacteraceae | g__Brevundimonas |  |
| f3f3ef7684ef91e3de2bbd5d6e2c1d4e | Bacteria | p__Firmicutes | c__Bacilli | o__Lactobacillales | f__Leuconostocaceae | g__Weissella |  |
| 82cc3bb919165e28b4dfc6e0086c74e5 | Bacteria | p__Firmicutes | c__Bacilli | o__Exiguobacterales | f__Exiguobacteraceae | g__Exiguobacterium |  |
| 14833948 | Bacteria | p__Chloroflexi | c__Ktedonobacteria | o__C0119 | f__C0119 | g__C0119 | s__uncultured_bacterium |
| 8efa1e28a42a15adb3212597265a0a0b | Bacteria | p__Firmicutes | c__Bacilli | o__Lactobacillales | f__Lactobacillaceae | g__Lactobacillus | s__Lactobacillus_iners |
| 470f337b43e46635d63e876792dfe909 | Bacteria | p__Proteobacteria | c__Alphaproteobacteria | o__Rhizobiales | f__Methyloligellaceae |  |  |
| 68b3757d8ec5719c69d40ac913dc634b | Bacteria | p__Proteobacteria | c__Gammaproteobacteria | o__Xanthomonadales | f__Xanthomonadaceae |  |  |
| 3e2bba300620ca49a48f27f873da786b | Bacteria | p__Actinobacteriota | c__Actinobacteria | o__Frankiales |  |  |  |
| 7bfa2d9dfe320a6e59e35306ff86f4f3 | Bacteria | p__Myxococcota | c__Polyangia | o__Polyangiales | f__Polyangiaceae | g__Pajaroellobacter |  |
| 02360733 | Bacteria | p__Proteobacteria | c__Alphaproteobacteria | o__Dongiales | f__Dongiaceae | g__Dongia |  |
| c969d8b8b397f3560e3675272bc4eb82 | Bacteria | p__Firmicutes | c__Bacilli | o__Lactobacillales | f__Lactobacillaceae | g__Lactobacillus |  |
| bca42b9ec9acf8169e6fd1fd987628f5 | Bacteria | p__Proteobacteria | c__Gammaproteobacteria | o__Burkholderiales | f__Nitrosomonadaceae | g__MND1 |  |
| 08512344 | Bacteria | p__Proteobacteria | c__Alphaproteobacteria | o__Rhizobiales | f__Rhizobiales_Incertae_Sedis | g__Nordella | s__uncultured_bacterium |
| 253a079e5f60250ee1c49d803e3ff2ed | Bacteria | p__Actinobacteriota | c__Thermoleophilia | o__Gaiellales | f__uncultured | g__uncultured |  |
| fd574ab91eeceef43a76c1d647fa1d3c | Bacteria | p__Actinobacteriota | c__Actinobacteria |  |  |  |  |
| 04901125 | Bacteria | p__Proteobacteria | c__Gammaproteobacteria | o__Steroidobacterales | f__Steroidobacteraceae | g__uncultured | s__uncultured_Methylococcaceae |
| 04665650 | Bacteria | p__Bacteroidota | c__Bacteroidia | o__Bacteroidales | f__Prevotellaceae | g__Alloprevotella |  |
| MJ030-2-barcode50-umi113695bins-ubs-3 | Bacteria | p__Proteobacteria | c__Gammaproteobacteria | o__WD260 | f__WD260 | g__WD260 |  |
| MJ020-1-barcode44-umi15890bins-ubs-24 | Bacteria | p__Bacteroidota | c__Bacteroidia | o__Chitinophagales | f__Chitinophagaceae |  |  |
| 14799524 | Bacteria | p__Chloroflexi | c__Ktedonobacteria | o__C0119 | f__C0119 | g__C0119 | s__uncultured_bacterium |
| MJ030-2-barcode59-umi88611bins-ubs-6 | Bacteria | p__Bacteroidota | c__Bacteroidia | o__Flavobacteriales | f__NS9_marine_group | g__NS9_marine_group |  |
| 02408366 | Bacteria | p__Firmicutes | c__Clostridia | o__Lachnospirales | f__Lachnospiraceae | g__Blautia |  |
| 05129652 | Bacteria | p__Acidobacteriota | c__Vicinamibacteria | o__Vicinamibacterales | f__uncultured | g__uncultured |  |
| 5f0ea21ff3c60f1de39d16d838c570d9 | Bacteria | p__Proteobacteria | c__Gammaproteobacteria | o__Pseudomonadales | f__Moraxellaceae | g__Acinetobacter |  |
| RS-GCF-003325755.1-NZ-QORI02000245.1 | Bacteria | p__Proteobacteria | c__Gammaproteobacteria | o__Pseudomonadales | f__Pseudomonadaceae | g__Pseudomonas |  |
| 6651d19102acccadcc895f76f344c01b | Bacteria | p__Actinobacteriota | c__Actinobacteria | o__Pseudonocardiales | f__Pseudonocardiaceae | g__Saccharopolyspora |  |
| G000620685_1 | Bacteria | p__Proteobacteria | c__Alphaproteobacteria | o__Dongiales | f__Dongiaceae | g__Dongia |  |
| 9e9f570323be932078e6676dc314a649 | Bacteria | p__Firmicutes | c__Bacilli | o__Bacillales | f__Bacillaceae | g__Bacillus |  |
| 38c5e8f70df47198d1bdb903e1438902 | Bacteria | p__Actinobacteriota | c__Actinobacteria | o__Micrococcales | f__Microbacteriaceae |  |  |
| 8bff894c8d6c8ca901cf4b40b29ecfe0 | Bacteria | p__Proteobacteria | c__Gammaproteobacteria | o__Pseudomonadales | f__Moraxellaceae | g__Acinetobacter |  |
| c2fdc56ff0ad5b1b50a1553b7a5511b3 | Bacteria | p__Planctomycetota | c__Planctomycetes | o__Pirellulales | f__Pirellulaceae | g__Pir4_lineage |  |
| 849c02e9697e4f90ee42d0917f09a8e3 | Bacteria | p__Myxococcota | c__bacteriap25 | o__bacteriap25 | f__bacteriap25 | g__bacteriap25 | s__uncultured_proteobacterium |
| 04958679 | Bacteria | p__Actinobacteriota | c__Actinobacteria | o__Frankiales | f__Geodermatophilaceae |  |  |
| 04898112 | Bacteria | p__Actinobacteriota | c__Actinobacteria | o__Micromonosporales | f__Micromonosporaceae | g__uncultured |  |
| 06427278 | Bacteria | p__Myxococcota | c__Myxococcia | o__Myxococcales | f__Myxococcaceae | g__KD3-10 | s__metagenome |
| b095e169ea1bfc752721881c5afc709b | Bacteria | p__Proteobacteria | c__Gammaproteobacteria | o__Thiotrichales | f__Thiotrichaceae | g__Thiothrix |  |
| MJ031-2-barcode69-umi51122bins-ubs-3 | Bacteria | p__Firmicutes | c__Bacilli | o__Lactobacillales | f__Enterococcaceae | g__Enterococcus |  |
| 4b9376fc2b4142003bfe58aecf7e9d78 | Bacteria | p__Bacteroidota | c__Bacteroidia | o__Sphingobacteriales | f__Sphingobacteriaceae | g__Pedobacter |  |
| MJ033-1-barcode47-umi6635bins-ubs-54 | Bacteria | p__Firmicutes | c__Clostridia | o__Peptostreptococcales-Tissierellales | f__Sedimentibacteraceae | g__Sedimentibacter | s__uncultured_bacterium |
| MN319555 | Bacteria | p__Actinobacteriota | c__Actinobacteria | o__Streptomycetales | f__Streptomycetaceae | g__Streptomyces |  |
| 5c5c9f20a85f04702a3976d1abc10500 | Bacteria | p__Actinobacteriota | c__Actinobacteria | o__Frankiales | f__Frankiaceae | g__Jatrophihabitans |  |
| ba143d6b8b203bdf80c0eb87cb1c14f0 | Bacteria | p__Proteobacteria | c__Gammaproteobacteria | o__Competibacterales | f__Competibacteraceae | g__Candidatus_Competibacter | s__uncultured_bacterium |
| 02917463 | Bacteria | p__Planctomycetota | c__Phycisphaerae | o__Tepidisphaerales | f__WD2101_soil_group | g__WD2101_soil_group | s__uncultured_planctomycete |
| RS-GCF-003947885.1-NZ-RFEF01000053.1 | Bacteria | p__Proteobacteria | c__Gammaproteobacteria | o__Pseudomonadales | f__Moraxellaceae | g__Acinetobacter |  |
| MJ034-1-barcode30-umi26762bins-ubs-5 | Bacteria |  |  |  |  |  |  |
| 34a169135e6b82214023654379733d8e | Bacteria | p__Bacteroidota | c__Bacteroidia | o__Bacteroidales | f__Prevotellaceae | g__Prevotella | s__Prevotella_stercorea |
| 04958435 | Bacteria | p__Actinobacteriota | c__Actinobacteria | o__Frankiales | f__Geodermatophilaceae |  |  |
| 860d492c1de8899155b2846c82b7f468 | Bacteria | p__Actinobacteriota | c__Acidimicrobiia | o__Microtrichales | f__Ilumatobacteraceae | g__CL500-29_marine_group |  |
| 05026371 | Bacteria | p__Firmicutes | c__Bacilli | o__Paenibacillales | f__Paenibacillaceae | g__Paenibacillus |  |
| 600b1aaa94d554f312f85582d6bf8c07 | Bacteria | p__Acidobacteriota | c__Acidobacteriae | o__Solibacterales | f__Solibacteraceae | g__Candidatus_Solibacter |  |
| AB006167 | Bacteria | p__Actinobacteriota | c__Actinobacteria | o__Micromonosporales | f__Micromonosporaceae | g__Virgisporangium |  |
| MJ010-1-barcode47-umi18691bins-ubs-33 | Bacteria | p__Proteobacteria | c__Gammaproteobacteria | o__Pseudomonadales | f__Moraxellaceae | g__Acinetobacter |  |
| 02248041 | Bacteria | p__Proteobacteria | c__Alphaproteobacteria | o__Reyranellales | f__Reyranellaceae | g__Reyranella |  |
| 900b0f14823511b248d8768ffb4a512d | Bacteria | p__Proteobacteria | c__Alphaproteobacteria | o__Rhizobiales | f__Beijerinckiaceae | g__Methylobacterium-Methylorubrum |  |
| KT962171 | Bacteria | p__Proteobacteria | c__Gammaproteobacteria | o__Xanthomonadales | f__Xanthomonadaceae | g__Lysobacter |  |
| f59729d7ad2dfd88120c9ff0866377e5 | Bacteria | p__Proteobacteria | c__Gammaproteobacteria | o__Burkholderiales | f__Comamonadaceae | g__Acidovorax |  |
| b83a027e41e478905a953d5b8b690d0d | Bacteria | p__Proteobacteria | c__Alphaproteobacteria | o__Caulobacterales | f__Hyphomonadaceae | g__SWB02 |  |
| 4f25d6699ddf4b33e91eb0850c72a00e | Bacteria | p__Proteobacteria | c__Gammaproteobacteria | o__CCD24 | f__CCD24 | g__CCD24 |  |
| 16295067 | Bacteria | p__Firmicutes | c__Clostridia | o__Lachnospirales | f__Lachnospiraceae | g__Tyzzerella | s__uncultured_Clostridium |
| 4f0752a473dcbd7cb24a3efd07545598 | Bacteria | p__Firmicutes | c__Bacilli | o__Paenibacillales | f__Paenibacillaceae | g__Ammoniphilus |  |
| MJ030-1-barcode25-umi118521bins-ubs-3 | Bacteria | p__Proteobacteria | c__Gammaproteobacteria | o__Burkholderiales | f__Comamonadaceae |  |  |
| bfa4689a0d27015d1ce4676d497c84a4 | Bacteria | p__Proteobacteria | c__Gammaproteobacteria | o__Alteromonadales | f__Alteromonadaceae | g__Rheinheimera |  |
| KY077157 | Bacteria | p__Bacteroidota | c__Bacteroidia | o__Flavobacteriales | f__Flavobacteriaceae | g__Flavobacterium | s__Flavobacterium_hauense |
| 1a7a83f68c0f4864dedb3ec30cd75cf7 | Bacteria | p__Proteobacteria | c__Alphaproteobacteria | o__Rhizobiales | f__Methyloligellaceae | g__uncultured |  |
| d2dc45f912782f1473990376e8331b9d | Bacteria | p__Actinobacteriota | c__Acidimicrobiia |  |  |  |  |
| 7840bc6cee89b0ba38e871c31e9a5a77 | Bacteria | p__Bacteroidota | c__Bacteroidia | o__Bacteroidales | f__Bacteroidetes_vadinHA17 | g__Bacteroidetes_vadinHA17 | s__metagenome |
| 4b22bfd5b81b1f4ef82d007be1240499 | Bacteria | p__Actinobacteriota | c__Actinobacteria | o__Corynebacteriales | f__Corynebacteriaceae | g__Corynebacterium |  |
| 83afd63d0e74aaf9e41bd6a28f36129d | Bacteria | p__Myxococcota | c__Polyangia | o__Polyangiales | f__Polyangiaceae | g__Pajaroellobacter |  |
| 04972358 | Bacteria | p__Firmicutes | c__Bacilli | o__Lactobacillales | f__Leuconostocaceae | g__Leuconostoc |  |
| 04964400 | Bacteria | p__Firmicutes | c__Clostridia | o__Oscillospirales | f__Oscillospiraceae | g__Colidextribacter |  |
| 14346481 | Bacteria | p__Acidobacteriota | c__Vicinamibacteria | o__Vicinamibacterales | f__uncultured | g__uncultured |  |
| 13583971e07f63f8d250f4be02f2c516 | Bacteria | p__Actinobacteriota | c__Acidimicrobiia | o__Microtrichales | f__Microtrichaceae | g__IMCC26207 |  |
| 22651de4dc2f909d42f8d97c52989394 | Bacteria | p__Proteobacteria | c__Alphaproteobacteria | o__Caulobacterales | f__Caulobacteraceae | g__Brevundimonas |  |
| MJ030-1-barcode25-umi29504bins-ubs-18 | Bacteria | p__Bacteroidota | c__Bacteroidia | o__Chitinophagales | f__Saprospiraceae | g__uncultured | s__uncultured_Saprospiraceae |
| 15726653 | Bacteria | p__Firmicutes | c__Clostridia | o__Oscillospirales | f__Ruminococcaceae | g__Ruminococcus | s__unidentified |
| c2acf7b3467acefb5e84d874afa333b5 | Bacteria | p__Chloroflexi | c__Anaerolineae | o__SJA-15 | f__SJA-15 | g__SJA-15 |  |
| 54702e581a8b79710c3690ebb38cab3a | Bacteria | p__Proteobacteria | c__Gammaproteobacteria | o__Burkholderiales | f__Comamonadaceae | g__Comamonas |  |
| 070dc41930a40b9e904b328b26dc0cc8 | Bacteria | p__Actinobacteriota | c__Thermoleophilia | o__Gaiellales | f__uncultured | g__uncultured |  |
| b524f2424771fba1e58c3999ccd64a91 | Bacteria | p__Proteobacteria | c__Alphaproteobacteria | o__Rhizobiales | f__Rhizobiaceae | g__Allorhizobium-Neorhizobium-Pararhizobium-Rhizobium |  |
| 80b3595fcc962d4751b7493c629ad328 | Bacteria | p__Actinobacteriota | c__Actinobacteria | o__Pseudonocardiales | f__Pseudonocardiaceae | g__Pseudonocardia |  |
| 04906287 | Bacteria | p__Proteobacteria | c__Alphaproteobacteria | o__Rhizobiales | f__Beijerinckiaceae | g__Bosea |  |
| 45a7a478c268967de8035af1567495f1 | Bacteria | p__Proteobacteria | c__Gammaproteobacteria | o__Methylococcales | f__Methylomonadaceae | g__Methylomonas |  |
| fe28398dbce457c7a9fe8dcb3fcfa869 | Bacteria | p__Planctomycetota | c__Planctomycetes | o__Isosphaerales | f__Isosphaeraceae |  |  |
| 8479881f7f3428161a6608400b1dcec6 | Bacteria | p__Proteobacteria | c__Gammaproteobacteria | o__Burkholderiales | f__Nitrosomonadaceae | g__Nitrosomonas |  |
| GB-GCA-003521065.1-DOTO01000001.1 | Bacteria | p__Proteobacteria | c__Gammaproteobacteria | o__Burkholderiales | f__Alcaligenaceae | g__Alcaligenes |  |
| 04905973 | Bacteria | p__Proteobacteria | c__Alphaproteobacteria | o__Rhizobiales | f__Xanthobacteraceae | g__uncultured |  |
| 783ba032c050ae8dc21dbe359929d359 | Bacteria | p__Gemmatimonadota | c__Gemmatimonadetes | o__Gemmatimonadales | f__Gemmatimonadaceae | g__uncultured |  |
| KY214292 | Bacteria | p__Actinobacteriota | c__Actinobacteria | o__Micrococcales | f__Microbacteriaceae | g__Leucobacter |  |
| 67e42bee1cd99cd18b5a3147d15fe1bd | Bacteria | p__Planctomycetota | c__Planctomycetes | o__Gemmatales | f__Gemmataceae | g__uncultured | s__uncultured_thermal |
| cf57dc448653f28b4c2d954eee9ad386 | Bacteria | p__Chloroflexi | c__Chloroflexia | o__Chloroflexales | f__Roseiflexaceae | g__uncultured |  |
| c322b9cb8d20612ce0d12f609c0a7c63 | Bacteria | p__Bacteroidota | c__Bacteroidia | o__Flavobacteriales | f__Flavobacteriaceae | g__Flavobacterium |  |
| b470aae01cf93f9f8aaa31bf44edb116 | Bacteria | p__Bacteroidota | c__Bacteroidia | o__Sphingobacteriales | f__NS11-12_marine_group | g__NS11-12_marine_group | s__uncultured_Flexibacteraceae |
| 3965834d8e7cf56673c8ce6c1e9113c2 | Bacteria | p__Bacteroidota | c__Bacteroidia | o__Bacteroidales | f__Rikenellaceae | g__Alistipes |  |
| 05040942 | Bacteria | p__Firmicutes | c__Clostridia | o__Peptostreptococcales-Tissierellales | f__Peptostreptococcales-Tissierellales | g__Finegoldia | s__uncultured_bacterium |
| 203c49e568da3e81e8789c876c0b63e2 | Bacteria | p__Proteobacteria | c__Gammaproteobacteria | o__Pseudomonadales | f__Moraxellaceae | g__Acinetobacter |  |
| bf6904595f155ebcee650cf347200985 | Bacteria | p__Acidobacteriota | c__Acidobacteriae | o__Subgroup_2 | f__Subgroup_2 | g__Subgroup_2 | s__uncultured_Holophaga |
| RS-GCF-014191245.1-NZ-JACHWG010000026.1 | Bacteria | p__Proteobacteria | c__Gammaproteobacteria | o__Burkholderiales | f__Burkholderiaceae | g__Cupriavidus |  |
| dc6de9dee299b741398f2fed4db872ab | Bacteria | p__Chloroflexi | c__Ktedonobacteria | o__Ktedonobacterales | f__Ktedonobacteraceae | g__Ktedonobacter |  |
| 43c6ed87caaa2b5f8b00b86157d1b2d8 | Bacteria | p__Firmicutes | c__Bacilli | o__Lactobacillales | f__Enterococcaceae | g__Enterococcus |  |
| HE616165 | Bacteria | p__Proteobacteria | c__Alphaproteobacteria | o__Rhizobiales | f__Rhizobiales_Incertae_Sedis | g__Phreatobacter |  |
| cd6d54c3e8d020e65690c13cac249d55 | Bacteria | p__Firmicutes | c__Clostridia | o__Clostridiales | f__Clostridiaceae | g__Clostridium_sensu_stricto_1 |  |
| 3a9e51bdf0ad29b2034e2bf7d8207e20 | Bacteria | p__Firmicutes | c__Clostridia | o__Lachnospirales | f__Lachnospiraceae | g__[Ruminococcus]_torques_group |  |
| 6bc1a73be11fe3c7ac02f5615156c4a4 | Bacteria | p__Proteobacteria | c__Gammaproteobacteria | o__Pseudomonadales | f__Moraxellaceae | g__Acinetobacter |  |
| d9eeb060a7b54148a4cfb865f381a420 | Bacteria | p__Proteobacteria | c__Alphaproteobacteria | o__Rhizobiales | f__Xanthobacteraceae | g__uncultured |  |
| AY457074 | Bacteria | p__Actinobacteriota | c__Actinobacteria | o__Corynebacteriales | f__Mycobacteriaceae | g__Mycobacterium |  |
| 17927940 | Bacteria | p__Actinobacteriota | c__Rubrobacteria | o__Rubrobacterales | f__Rubrobacteriaceae | g__Rubrobacter |  |
| 39404334c817403d1acd5880ca7eabf7 | Bacteria | p__Firmicutes | c__Clostridia | o__Lachnospirales | f__Lachnospiraceae |  |  |
| G000179255_1 | Bacteria | p__Cyanobacteria | c__Cyanobacteriia | o__Synechococcales | f__Cyanobiaceae | g__Cyanobium_PCC-6307 |  |
| e3c1d0e07a68380c9534969652c685b2 | Bacteria | p__Actinobacteriota | c__Actinobacteria | o__Propionibacteriales | f__Propionibacteriaceae | g__Propioniciclava | s__uncultured_bacterium |
| 1dc20636cdade28fd00458840295a99b | Bacteria | p__Actinobacteriota | c__Actinobacteria | o__Pseudonocardiales | f__Pseudonocardiaceae | g__Pseudonocardia |  |
| RS-GCF-016501935.1-NZ-JADVFR010000053.1 | Bacteria | p__Proteobacteria | c__Gammaproteobacteria | o__Enterobacterales | f__Enterobacteriaceae |  |  |
| 66150c792d1df065d07f0880ea8a9eca | Bacteria | p__Proteobacteria | c__Alphaproteobacteria | o__Rhodobacterales | f__Rhodobacteraceae | g__Falsirhodobacter |  |
| 04665616 | Bacteria | p__Proteobacteria | c__Alphaproteobacteria | o__Rhizobiales | f__Beijerinckiaceae | g__Methylobacterium-Methylorubrum |  |
| e4a9ee0823fa5c31d3f927d0994f70a3 | Bacteria | p__Firmicutes | c__Clostridia | o__Clostridia | f__Hungateiclostridiaceae | g__Ruminiclostridium | s__uncultured_prokaryote |
| 04962155 | Bacteria | p__Actinobacteriota | c__Actinobacteria | o__Propionibacteriales | f__Nocardioidaceae | g__Nocardioides |  |
| MJ010-1-barcode26-umi114957bins-ubs-5 | Bacteria | p__Acidobacteriota | c__Blastocatellia | o__Pyrinomonadales | f__Pyrinomonadaceae | g__RB41 |  |
| MJ031-1-barcode42-umi32157bins-ubs-8 | Bacteria | p__Bacteroidota | c__Bacteroidia | o__Chitinophagales | f__Chitinophagaceae | g__Flavisolibacter |  |
| GB-GCA-000468795.1-AWPJ01000352.1 | Bacteria | p__Actinobacteriota | c__Actinobacteria | o__Bifidobacteriales | f__Bifidobacteriaceae | g__Bifidobacterium |  |
| 05022675 | Bacteria | p__Actinobacteriota | c__Acidimicrobiia | o__IMCC26256 | f__IMCC26256 | g__IMCC26256 | s__uncultured_Ferrimicrobium |
| 2a373c4c10b6b4b3f518edc506326ff7 | Bacteria | p__Bacteroidota | c__Bacteroidia | o__Sphingobacteriales | f__Sphingobacteriaceae | g__Sphingobacterium |  |
| KJ810584 | Bacteria | p__Proteobacteria | c__Gammaproteobacteria | o__Burkholderiales | f__Oxalobacteraceae | g__Massilia |  |
| 20336835 | Bacteria | p__Firmicutes | c__Bacilli | o__Bacillales | f__Bacillaceae | g__Bacillus | s__Bacillus_oleronius |
| 04e97227d8678a67c7ab8d051e34f76c | Bacteria | p__Firmicutes | c__Bacilli | o__Erysipelotrichales | f__Erysipelotrichaceae | g__Turicibacter |  |
| 02917578 | Bacteria | p__Planctomycetota | c__Phycisphaerae | o__Tepidisphaerales | f__WD2101_soil_group | g__WD2101_soil_group |  |
| 04918579 | Bacteria | p__Bacteroidota | c__Bacteroidia | o__Flavobacteriales | f__Flavobacteriaceae | g__Myroides | s__Myroides_marinus |
| b20a6cd287edab9f5bfd0a2171e8b84d | Bacteria | p__Proteobacteria | c__Gammaproteobacteria | o__Steroidobacterales | f__Steroidobacteraceae | g__Steroidobacter |  |
| f8d29dc276b0b545df3115cfb94baa58 | Bacteria | p__Proteobacteria | c__Alphaproteobacteria | o__Rhodobacterales | f__Rhodobacteraceae | g__Paracoccus |  |
| 6d31324b057626847123237c7ea35938 | Bacteria | p__Myxococcota | c__Polyangia | o__Polyangiales | f__Polyangiaceae | g__uncultured |  |
| f18801503899f9fa63f71f6a61b1aa81 | Bacteria | p__Verrucomicrobiota | c__Verrucomicrobiae | o__Chthoniobacterales | f__Chthoniobacteraceae | g__Candidatus_Udaeobacter | s__uncultured_Prosthecobacter |
| AM177497 | Bacteria | p__Bacteroidota | c__Bacteroidia | o__Flavobacteriales | f__Weeksellaceae | g__Empedobacter |  |
| G000434535_1 | Bacteria | p__Actinobacteriota | c__Coriobacteriia | o__Coriobacteriales | f__Coriobacteriaceae | g__Collinsella |  |
| 0b3d764be943cc31341854199ea9ef4d | Bacteria | p__Actinobacteriota | c__Actinobacteria | o__Micrococcales | f__Microbacteriaceae | g__Microbacterium |  |
| 013fe4a791073b1e98269d9ccb194c32 | Bacteria | p__Proteobacteria | c__Gammaproteobacteria | o__Burkholderiales | f__Nitrosomonadaceae | g__Ellin6067 |  |
| 17182109 | Bacteria | p__Firmicutes | c__Bacilli | o__Alicyclobacillales | f__Alicyclobacillaceae | g__Tumebacillus | s__bacterium_S1(2012) |
| a82c29d6fb728f0ab801666dd15585ac | Bacteria | p__Firmicutes | c__Bacilli | o__Erysipelotrichales | f__Erysipelotrichaceae | g__Holdemanella | s__uncultured_bacterium |
| 02304539 | Bacteria | p__Proteobacteria | c__Alphaproteobacteria | o__Defluviicoccales | f__Defluviicoccaceae | g__Defluviicoccus |  |
| 535d6a3875fe73a9a51d2362179f722a | Bacteria | p__Proteobacteria | c__Alphaproteobacteria | o__Sphingomonadales | f__Sphingomonadaceae |  |  |
| d6b5af4643de7abf2e088980bfa7b4ce | Bacteria | p__Proteobacteria | c__Alphaproteobacteria | o__Rhizobiales | f__Beijerinckiaceae | g__Methylobacterium-Methylorubrum |  |
| MJ031-1-barcode30-umi13732bins-ubs-33 | Bacteria | p__Proteobacteria | c__Gammaproteobacteria | o__Burkholderiales | f__Comamonadaceae |  |  |
| 04959780 | Bacteria | p__Proteobacteria | c__Gammaproteobacteria | o__Burkholderiales | f__Burkholderiaceae | g__Burkholderia-Caballeronia-Paraburkholderia |  |
| GB-GCA-902366885.1-CABJNK010000117.1 | Bacteria | p__Firmicutes | c__Clostridia | o__Lachnospirales | f__Lachnospiraceae |  |  |
| AB200231 | Bacteria | p__Actinobacteriota | c__Actinobacteria | o__Micromonosporales | f__Micromonosporaceae | g__Catellatospora |  |
| e4376b1cd8fa608d573bcc59874f6c2e | Bacteria | p__Proteobacteria | c__Alphaproteobacteria | o__Rhizobiales | f__Xanthobacteraceae | g__Rhodoplanes |  |
| bdf47b011495211e843b19649387ff1f | Bacteria | p__Cyanobacteria | c__Cyanobacteriia | o__Oxyphotobacteria_Incertae_Sedis | f__Unknown_Family | g__Leptolyngbya_EcFYyyy-00 | s__uncultured_bacterium |
| ed25cb1938670fe000b57f009f162c3f | Bacteria | p__Proteobacteria | c__Gammaproteobacteria | o__Pseudomonadales | f__Pseudomonadaceae | g__Pseudomonas |  |
| 1bf1a4f5986af051409bd52a5cad2d65 | Bacteria | p__Bacteroidota | c__Bacteroidia | o__Sphingobacteriales | f__Sphingobacteriaceae |  |  |
| MT137387 | Bacteria | p__Proteobacteria | c__Alphaproteobacteria | o__Rhodobacterales | f__Rhodobacteraceae | g__Paracoccus |  |
| 10286062 | Bacteria | p__Bacteroidota | c__Bacteroidia | o__Bacteroidales | f__Rikenellaceae | g__Alistipes | s__uncultured_bacterium |
| 100abd7c73e38ab6f530ec8281670000 | Bacteria | p__Proteobacteria | c__Gammaproteobacteria | o__Burkholderiales | f__Methylophilaceae | g__Methylophilus |  |
| b87abc7e504ced7d11db537bae243204 | Bacteria | p__Chloroflexi | c__Ktedonobacteria | o__Ktedonobacterales | f__Ktedonobacteraceae | g__uncultured |  |
| aa780d37c2ab30ab820b6f71b6981212 | Bacteria | p__Chloroflexi | c__KD4-96 | o__KD4-96 | f__KD4-96 | g__KD4-96 |  |
| G004144185_1 | Bacteria | p__Proteobacteria | c__Alphaproteobacteria | o__Rhizobiales | f__Xanthobacteraceae |  |  |
| APPT01000006 | Bacteria | p__Proteobacteria | c__Gammaproteobacteria | o__Pseudomonadales | f__Moraxellaceae | g__Acinetobacter | s__Acinetobacter_baylyi |
| a212efca64d3b5f191ca3cc7eaff176f | Bacteria | p__NB1-j | c__NB1-j | o__NB1-j | f__NB1-j | g__NB1-j | s__uncultured_Desulfoglaeba |
| MJ006-2-barcode61-umi190207bins-ubs-3 | Bacteria | p__Firmicutes | c__Clostridia | o__Lachnospirales | f__Lachnospiraceae | g__Roseburia | s__gut_metagenome |
| 13719893 | Bacteria | p__Gemmatimonadota | c__Gemmatimonadetes | o__Gemmatimonadales | f__Gemmatimonadaceae | g__Gemmatimonas |  |
| 18696347 | Bacteria | p__Actinobacteriota | c__Actinobacteria | o__Frankiales | f__Geodermatophilaceae |  |  |
| 2bc6269f7447b65c0ae15ba3e4f43175 | Bacteria | p__Proteobacteria | c__Alphaproteobacteria | o__Caulobacterales | f__Caulobacteraceae | g__Phenylobacterium |  |
| 08939180 | Bacteria | p__Proteobacteria | c__Alphaproteobacteria | o__Rhodospirillales | f__uncultured | g__uncultured |  |
| MJ009-2-barcode59-umi93523bins-ubs-5 | Bacteria | p__Proteobacteria | c__Gammaproteobacteria | o__Enterobacterales | f__Erwiniaceae |  |  |
| KT950747 | Bacteria | p__Proteobacteria | c__Alphaproteobacteria | o__Sphingomonadales | f__Sphingomonadaceae | g__Sphingomonas |  |
| MJ006-1-barcode29-umi105197bins-ubs-4 | Bacteria | p__Bacteroidota | c__Bacteroidia | o__Bacteroidales | f__Barnesiellaceae | g__Barnesiella |  |
| RS-GCF-018784645.1-NZ-WQOM01000090.1 | Bacteria | p__Firmicutes | c__Clostridia | o__Oscillospirales | f__Ruminococcaceae | g__Faecalibacterium |  |
| fca99ed3795a692097ba0c860dcfcb49 | Bacteria | p__Actinobacteriota | c__Actinobacteria | o__Corynebacteriales | f__Corynebacteriaceae | g__Corynebacterium |  |
| GB-GCA-004801755.1-SSTL01000077.1 | Bacteria | p__Bacteroidota | c__Bacteroidia | o__Bacteroidales | f__Muribaculaceae | g__Muribaculaceae |  |
| RS-GCF-014268815.2-NZ-JABWRE020000001.1 | Bacteria | p__Proteobacteria | c__Gammaproteobacteria | o__Pseudomonadales | f__Pseudomonadaceae | g__Pseudomonas |  |
| LC487980 | Bacteria | p__Firmicutes | c__Negativicutes | o__Veillonellales-Selenomonadales | f__Veillonellaceae | g__Dialister | s__gut_metagenome |
| c0e1f03ea164458cc1c28c0f21d38e18 | Bacteria | p__Actinobacteriota | c__Actinobacteria | o__Bifidobacteriales | f__Bifidobacteriaceae | g__Bifidobacterium |  |
| RS-GCF-014397025.1-NZ-JACJUO010000001.1 | Bacteria | p__Proteobacteria | c__Alphaproteobacteria | o__Rhizobiales | f__Rhizobiaceae | g__Falsochrobactrum |  |
| MJ007-2-barcode74-umi20788bins-ubs-16 | Bacteria | p__Acidobacteriota | c__Acidobacteriae | o__Bryobacterales | f__Bryobacteraceae | g__Bryobacter |  |
| GQ241324 | Bacteria | p__Bacteroidota | c__Bacteroidia | o__Cytophagales | f__Spirosomaceae | g__Dyadobacter |  |
| de4266ac45caf089718d607b70298212 | Bacteria | p__Bacteroidota | c__Bacteroidia | o__Bacteroidales | f__Bacteroidaceae | g__Bacteroides |  |
| 05009479 | Bacteria | p__Proteobacteria | c__Alphaproteobacteria | o__Sphingomonadales | f__Sphingomonadaceae | g__Sphingomonas | s__Sphingomonas_daechungensis |
| 7320d6055a66370b0bfeaf05251282b2 | Bacteria | p__Bacteroidota | c__Bacteroidia | o__Bacteroidales | f__Bacteroidaceae | g__Bacteroides |  |
| 48392e86abc7ce351c3a1faa97c20eed | Bacteria | p__Proteobacteria | c__Gammaproteobacteria | o__Xanthomonadales | f__Xanthomonadaceae |  |  |
| MH161601 | Bacteria | p__Actinobacteriota | c__Actinobacteria | o__Micrococcales | f__Microbacteriaceae |  |  |
| f0bab26073b533ee4a55cf2302ebe9c8 | Bacteria | p__Chloroflexi | c__Anaerolineae | o__Caldilineales | f__Caldilineaceae | g__uncultured |  |
| MJ005-barcode74-umi19810bins-ubs-10 | Bacteria | p__Proteobacteria | c__Gammaproteobacteria | o__Enterobacterales | f__Enterobacteriaceae |  |  |
| cc27802ca9b32459fb662b197fd7366a | Bacteria | p__Proteobacteria | c__Gammaproteobacteria | o__Burkholderiales | f__Comamonadaceae |  |  |
| 47f1366776cdca4b322d172321a652ac | Bacteria | p__Bacteroidota | c__Bacteroidia | o__Chitinophagales | f__Chitinophagaceae | g__Flavisolibacter |  |
| RS-GCF-014646955.1-NZ-BMOG01000032.1 | Bacteria | p__Proteobacteria | c__Gammaproteobacteria | o__Pseudomonadales | f__Pseudomonadaceae | g__Pseudomonas |  |
| 8761ef1c2857750a049e51b4e03d2d3a | Bacteria | p__Proteobacteria | c__Gammaproteobacteria | o__Pseudomonadales | f__Moraxellaceae | g__Acinetobacter |  |
| b3d0e698d06050545c0f282af73f1c6b | Bacteria | p__Acidobacteriota | c__Acidobacteriae | o__Acidobacteriales |  |  |  |
| 66dcb675cb544ab95319daeb232f62cd | Bacteria | p__Gemmatimonadota | c__Gemmatimonadetes | o__Gemmatimonadales | f__Gemmatimonadaceae | g__Roseisolibacter | s__uncultured_bacterium |
| 195be9652137998e973b5d2bc1757336 | Bacteria | p__Actinobacteriota | c__Thermoleophilia | o__Solirubrobacterales | f__Solirubrobacteraceae | g__Patulibacter | s__Patulibacter_minatonensis |
| c2837c37ec3270d38f08ad9f9f129195 | Bacteria | p__Firmicutes | c__Symbiobacteriia | o__Symbiobacteriales | f__Symbiobacteraceae | g__Symbiobacterium |  |
| d8ecaeeac1c75d0b27bdcd63daf6c6c5 | Bacteria | p__Firmicutes | c__Clostridia | o__Lachnospirales | f__Lachnospiraceae | g__[Eubacterium]_hallii_group |  |
| 16222941 | Bacteria | p__Firmicutes | c__Bacilli | o__Erysipelotrichales | f__Erysipelotrichaceae | g__Ileibacterium | s__Ileibacterium_valens |
| MJ030-2-barcode63-umi109272bins-ubs-3 | Bacteria | p__Firmicutes | c__Bacilli | o__Erysipelotrichales | f__Erysipelotrichaceae | g__Turicibacter | s__uncultured_bacterium |
| c814984451915a6f319ceea28e141235 | Bacteria | p__Actinobacteriota | c__Actinobacteria | o__Frankiales | f__Acidothermaceae | g__Acidothermus |  |
| 332550a9b5a15fcb3c72eb16b2caae31 | Bacteria | p__Proteobacteria | c__Gammaproteobacteria | o__Xanthomonadales | f__Xanthomonadaceae | g__Lysobacter |  |
| 7e7687477cdd7f0358a8a1816412ef95 | Bacteria | p__Actinobacteriota | c__Acidimicrobiia | o__Actinomarinales | f__uncultured | g__uncultured | s__uncultured_Actinomycetales |
| MJ020-1-barcode47-umi58bins-ubs-1804 | Bacteria | p__Proteobacteria | c__Gammaproteobacteria | o__Pseudomonadales | f__Moraxellaceae | g__Acinetobacter |  |
| 17973428 | Bacteria | p__Chloroflexi | c__Ktedonobacteria | o__Ktedonobacterales | f__Ktedonobacteraceae | g__uncultured | s__uncultured_soil |
| KT900247 | Bacteria | p__Proteobacteria | c__Gammaproteobacteria | o__Alteromonadales | f__Alteromonadaceae | g__Rheinheimera |  |
| GQ339904 | Bacteria | p__Actinobacteriota | c__Actinobacteria | o__Propionibacteriales | f__Nocardioidaceae | g__Nocardioides |  |
| MJ006-1-barcode40-umi54882bins-ubs-7 | Bacteria | p__Bacteroidota | c__Bacteroidia | o__Bacteroidales | f__Bacteroidaceae | g__Bacteroides | s__Bacteroides_kribbi |
| ab049a553ab7078fb82fbeb83cf4370d | Bacteria | p__Actinobacteriota | c__Actinobacteria | o__Corynebacteriales | f__Corynebacteriaceae | g__Corynebacterium |  |
| 73faea9c77946d83f897c28835e9aca1 | Bacteria | p__Myxococcota | c__bacteriap25 | o__bacteriap25 | f__bacteriap25 | g__bacteriap25 |  |
| 47632b9d284d162943e23701bdb8d96e | Bacteria | p__Proteobacteria | c__Gammaproteobacteria | o__Aeromonadales |  |  |  |
| MJ030-2-barcode74-umi26852bins-ubs-25 | Bacteria | p__Bacteroidota | c__Bacteroidia | o__Chitinophagales | f__Chitinophagaceae |  |  |
| 04946127 | Bacteria | p__Actinobacteriota | c__Actinobacteria | o__Micrococcales | f__Promicromonosporaceae | g__Promicromonospora |  |
| f2c1fcb08dc2fa030849944db2d9297c | Bacteria |  |  |  |  |  |  |
| e0bb17fea113ce48e8e7fd55a0308a75 | Bacteria | p__Firmicutes | c__Bacilli | o__Lactobacillales | f__Streptococcaceae | g__Lactococcus |  |
| RS-GCF-014894875.1-NZ-JACXYT010000012.1 | Bacteria | p__Proteobacteria | c__Gammaproteobacteria | o__Enterobacterales | f__Yersiniaceae | g__Serratia |  |
| GU721027 | Bacteria | p__Proteobacteria | c__Gammaproteobacteria | o__Burkholderiales | f__Comamonadaceae | g__Delftia |  |
| 04943797 | Bacteria | p__Acidobacteriota | c__Blastocatellia | o__Blastocatellales | f__Blastocatellaceae | g__uncultured |  |
| a0f4db0ba5f3561759c0fcfcb8e77ff3 | Bacteria | p__Proteobacteria | c__Gammaproteobacteria | o__Xanthomonadales | f__Rhodanobacteraceae | g__uncultured |  |
| 02244722 | Bacteria | p__Actinobacteriota | c__Actinobacteria | o__Corynebacteriales | f__Corynebacteriaceae | g__Corynebacterium |  |
| ae41b8c68872aa87bead5c45d517af43 | Bacteria | p__Actinobacteriota | c__Actinobacteria | o__Propionibacteriales | f__Nocardioidaceae | g__Nocardioides |  |
| 46db96eac57be1708fcce7374603bddf | Bacteria | p__Proteobacteria | c__Gammaproteobacteria | o__Enterobacterales | f__Enterobacteriaceae |  |  |
| G000526595_1 | Bacteria | p__Actinobacteriota | c__Actinobacteria | o__Micrococcales |  |  |  |
| cd7ae15c5d9e01f3b3713220d1a487f6 | Bacteria | p__Firmicutes | c__Clostridia | o__Lachnospirales | f__Lachnospiraceae | g__[Ruminococcus]_torques_group |  |
| 20946070 | Bacteria | p__Firmicutes | c__Bacilli | o__Erysipelotrichales | f__Erysipelotrichaceae | g__Ileibacterium | s__Ileibacterium_valens |
| 68fdbcf5c6c0026f9da8b716855eae30 | Bacteria | p__Gemmatimonadota | c__Gemmatimonadetes | o__Gemmatimonadales | f__Gemmatimonadaceae | g__uncultured |  |
| MJ009-2-barcode59-umi14575bins-ubs-38 | Bacteria | p__Proteobacteria | c__Gammaproteobacteria | o__Enterobacterales | f__Erwiniaceae |  |  |
| 33c2ebc99a46d8626e8528e9521c4be5 | Bacteria | p__Proteobacteria | c__Alphaproteobacteria |  |  |  |  |
| 04665846 | Bacteria | p__Actinobacteriota | c__Actinobacteria | o__Actinomycetales | f__Actinomycetaceae | g__Actinomyces |  |
| 3292f8f3502aa330c9b93ca1b11b07e5 | Bacteria | p__Myxococcota | c__Polyangia | o__Haliangiales | f__Haliangiaceae | g__Haliangium |  |
| MJ030-2-barcode63-umi112006bins-ubs-3 | Bacteria | p__Firmicutes | c__Bacilli | o__Lactobacillales | f__Streptococcaceae | g__Streptococcus |  |
| RS-GCF-004348115.1-NZ-QPFB01000001.1 | Bacteria | p__Proteobacteria | c__Gammaproteobacteria | o__Xanthomonadales | f__Xanthomonadaceae | g__Stenotrophomonas |  |
| 05030602 | Bacteria | p__Proteobacteria | c__Gammaproteobacteria | o__Burkholderiales | f__Oxalobacteraceae | g__Massilia |  |
| 02407883 | Bacteria | p__Firmicutes | c__Clostridia | o__Clostridiales | f__Clostridiaceae | g__Clostridium_sensu_stricto_1 |  |
| 04992726 | Bacteria | p__Proteobacteria | c__Alphaproteobacteria | o__Azospirillales | f__Azospirillaceae | g__Skermanella |  |
| 3e921282cdbd2d7b939d7952221cfb65 | Bacteria | p__Acidobacteriota | c__Holophagae | o__Subgroup_7 | f__Subgroup_7 | g__Subgroup_7 | s__uncultured_Desulfovirga |
| 7952d75e619d4caeb22f704ef27844b1 | Bacteria | p__Actinobacteriota | c__Actinobacteria | o__Corynebacteriales | f__Tsukamurellaceae | g__Tsukamurella |  |
| 05029350 | Bacteria | p__Actinobacteriota | c__Thermoleophilia | o__Solirubrobacterales | f__Solirubrobacteraceae | g__Solirubrobacter |  |
| MN685328 | Bacteria | p__Proteobacteria | c__Gammaproteobacteria | o__Burkholderiales | f__Oxalobacteraceae |  |  |
| 04898438 | Bacteria | p__Acidobacteriota | c__Acidobacteriae | o__Subgroup_2 | f__Subgroup_2 | g__Subgroup_2 |  |
| 5c11d9c97d115d31afa93c67ad2faf59 | Bacteria | p__Bacteroidota | c__Bacteroidia | o__Sphingobacteriales | f__Sphingobacteriaceae | g__Sphingobacterium |  |
| 8cb04690e8169fe646805dc7f4851ece | Bacteria | p__Acidobacteriota | c__Acidobacteriae | o__Solibacterales | f__Solibacteraceae | g__Candidatus_Solibacter |  |
| 0f714939c892f96e2fe42b98c40380d0 | Bacteria | p__Proteobacteria | c__Gammaproteobacteria | o__Burkholderiales | f__A21b | g__A21b |  |
| 05031094 | Bacteria | p__Actinobacteriota | c__Actinobacteria | o__Micrococcales | f__Microbacteriaceae |  |  |
| d5d66f5c9fb6de1148d08c79102d2967 | Bacteria | p__Proteobacteria | c__Gammaproteobacteria | o__Burkholderiales | f__Comamonadaceae | g__Lampropedia |  |
| 84ff773625a57ded753ea42a579bb3b2 | Bacteria | p__Proteobacteria | c__Gammaproteobacteria | o__JG36-GS-52 | f__JG36-GS-52 | g__JG36-GS-52 | s__uncultured_bacterium |
| 5efce3deaab773798e87f9b13eee6ce6 | Bacteria | p__Methylomirabilota | c__Methylomirabilia | o__Rokubacteriales | f__Rokubacteriales | g__Rokubacteriales |  |
| KT962169 | Bacteria | p__Bacteroidota | c__Bacteroidia | o__Chitinophagales | f__Chitinophagaceae | g__Niastella |  |
| 724bad59c1b2224e6165b49e10dce0f9 | Bacteria | p__Proteobacteria | c__Alphaproteobacteria | o__Rhizobiales | f__Methyloligellaceae |  |  |
| 04967372 | Bacteria | p__Firmicutes | c__Clostridia | o__Oscillospirales | f__Oscillospiraceae | g__Oscillibacter |  |
| Y17591 | Bacteria | p__Proteobacteria | c__Gammaproteobacteria | o__Burkholderiales | f__Rhodocyclaceae | g__Thauera |  |
| RS-GCF-000410515.1-NC-021500.1--10 | Bacteria | p__Proteobacteria | c__Gammaproteobacteria | o__Enterobacterales | f__Enterobacteriaceae |  |  |
| G900115805_1 | Bacteria | p__Firmicutes | c__Bacilli | o__Bacillales | f__Planococcaceae | g__Psychrobacillus |  |
| a596e1ce88223a35fbd63b8a859d1ba0 | Bacteria | p__Verrucomicrobiota | c__Verrucomicrobiae | o__Chthoniobacterales | f__Chthoniobacteraceae | g__Candidatus_Udaeobacter |  |
| 1a930d1b275c14d84e1cd24d24c1bf2c | Bacteria | p__Proteobacteria | c__Alphaproteobacteria | o__Dongiales | f__Dongiaceae | g__Dongia |  |
| abccfaa737c3120ae1c2e67b7588f343 | Bacteria | p__Proteobacteria | c__Alphaproteobacteria | o__Elsterales | f__uncultured | g__uncultured |  |
| 4de3559d16d2a8a4458d9261a2738ce9 | Bacteria | p__Firmicutes | c__Bacilli | o__Paenibacillales | f__Paenibacillaceae | g__Saccharibacillus |  |
| e054180a96396ed021ac2a307f5faa8a | Bacteria | p__Firmicutes | c__Clostridia | o__Caldicoprobacterales | f__Caldicoprobacteraceae | g__Caldicoprobacter |  |
| 78b347736379e46c55c9c2503420dd71 | Bacteria | p__NB1-j | c__NB1-j | o__NB1-j | f__NB1-j | g__NB1-j |  |
| 46c176223a71ed90467715881c1831a3 | Bacteria | p__Acidobacteriota | c__Acidobacteriae | o__Bryobacterales | f__Bryobacteraceae | g__Bryobacter |  |
| 74954a510f9068ce50591e61b2a4ba8f | Bacteria | p__Proteobacteria | c__Gammaproteobacteria | o__Xanthomonadales | f__Xanthomonadaceae | g__Thermomonas |  |
| 08442017 | Bacteria | p__Proteobacteria | c__Alphaproteobacteria | o__Rhizobiales | f__Beijerinckiaceae | g__Microvirga |  |
| 44f4673202a4e3a9cd8288efa1859c33 | Bacteria | p__Actinobacteriota | c__Actinobacteria | o__Corynebacteriales | f__Mycobacteriaceae | g__Mycobacterium |  |
| 7d6b0d0ba7eb5d7cc81fcf6490cdf3ba | Bacteria | p__Chloroflexi | c__AD3 | o__AD3 | f__AD3 | g__AD3 |  |
| 3023324d82b5b70c22d30e9291bd7ae3 | Bacteria | p__Proteobacteria | c__Alphaproteobacteria | o__uncultured | f__uncultured | g__uncultured |  |
| G900177325_1 | Bacteria | p__Proteobacteria | c__Alphaproteobacteria | o__Rhizobiales | f__Rhizobiaceae |  |  |
| EF212039 | Bacteria | p__Actinobacteriota | c__Actinobacteria | o__Micrococcales | f__Intrasporangiaceae | g__Terrabacter |  |
| 749b480ea445880c392939ddd561baec | Bacteria | p__Proteobacteria | c__Gammaproteobacteria | o__Burkholderiales | f__TRA3-20 | g__TRA3-20 |  |
| 0d9b3827c4958ab95ad81395ad0ef9b8 | Bacteria | p__Proteobacteria | c__Gammaproteobacteria | o__Burkholderiales | f__Nitrosomonadaceae | g__MND1 |  |
| MJ030-2-barcode66-umi10824bins-ubs-47 | Bacteria | p__Firmicutes | c__Clostridia | o__Clostridia_UCG-014 | f__Clostridia_UCG-014 | g__Clostridia_UCG-014 |  |
| MJ034-1-barcode31-umi14683bins-ubs-21 | Bacteria | p__Acidobacteriota | c__Acidobacteriae | o__Acidobacteriales | f__uncultured | g__uncultured |  |
| 05013214 | Bacteria | p__Bacteroidota | c__Bacteroidia | o__Chitinophagales | f__Chitinophagaceae | g__Flavitalea |  |
| MJ007-1-barcode36-umi41898bins-ubs-3 | Bacteria | p__Bacteroidota | c__Bacteroidia | o__Flavobacteriales | f__Flavobacteriaceae | g__Myroides | s__Myroides_odoratus |
| 19c1eca367e0eb3bd9df9cc414d5e0ca | Bacteria | p__Bacteroidota | c__Bacteroidia | o__Chitinophagales | f__Chitinophagaceae | g__Terrimonas |  |
| a49243b9260f4ff2afacfa3da2b6d6e1 | Bacteria | p__Firmicutes | c__Bacilli | o__Bacillales | f__Bacillaceae | g__Bacillus |  |
| 95bc3ac6db2063c77553c5c2816978dd | Bacteria | p__Bacteroidota | c__Bacteroidia | o__Chitinophagales | f__Chitinophagaceae |  |  |
| GB-GCA-902636675.1-CACPSQ010000105.1 | Bacteria | p__Bacteroidota | c__Bacteroidia | o__Sphingobacteriales | f__Sphingobacteriaceae | g__Pedobacter |  |
| G007581405_1 | Bacteria | p__Proteobacteria | c__Gammaproteobacteria | o__Enterobacterales | f__Enterobacteriaceae |  |  |
| 535db91531dd5542ed17ed57804a2a8a | Bacteria | p__Actinobacteriota | c__Thermoleophilia | o__Solirubrobacterales | f__Solirubrobacteraceae | g__Solirubrobacter |  |
| 08553767 | Bacteria | p__Proteobacteria | c__Alphaproteobacteria | o__Rhizobiales | f__Kaistiaceae | g__Kaistia |  |
| 2b8b77ce33668c0eeede135fad449ebd | Bacteria | p__Firmicutes | c__Symbiobacteriia | o__Symbiobacteriales | f__Symbiobacteraceae | g__Symbiobacterium | s__uncultured_bacterium |
| 17ffc625fac922f9940e840aac7d5929 | Bacteria | p__Firmicutes | c__Bacilli | o__Staphylococcales | f__Staphylococcaceae | g__Staphylococcus |  |
| KT309087 | Bacteria | p__Proteobacteria | c__Alphaproteobacteria | o__Caulobacterales | f__Caulobacteraceae |  |  |
| d305e1cd36a9fa41fd902d26391e51a6 | Bacteria | p__Proteobacteria | c__Gammaproteobacteria | o__Enterobacterales | f__Enterobacteriaceae |  |  |
| 14705029 | Bacteria | p__Chloroflexi |  |  |  |  |  |
| 24018007931a5c87b603c84945b46610 | Bacteria | p__Proteobacteria | c__Alphaproteobacteria | o__Rhizobiales | f__A0839 | g__A0839 | s__uncultured_Phyllobacteriaceae |
| 366482680ef528265c832510764b52a5 | Bacteria | p__Proteobacteria | c__Alphaproteobacteria | o__Rhizobiales | f__Devosiaceae |  |  |
| MJ030-2-barcode50-umi92296bins-ubs-5 | Bacteria | p__Proteobacteria | c__Alphaproteobacteria | o__Rhizobiales | f__Xanthobacteraceae |  |  |
| e1f6765408c1ed2b67b5fa26cced7b43 | Bacteria | p__Verrucomicrobiota | c__Verrucomicrobiae | o__Chthoniobacterales | f__Chthoniobacteraceae | g__Candidatus_Udaeobacter |  |
| RS-GCF-004337025.1-NZ-SJUP01000030.1 | Bacteria | p__Firmicutes | c__Bacilli | o__Exiguobacterales | f__Exiguobacteraceae | g__Exiguobacterium |  |
| 7b6d916c02bf246d9b310a4105bd97a4 | Bacteria | p__Firmicutes | c__Bacilli | o__Lactobacillales | f__Enterococcaceae | g__Enterococcus |  |
| dd373930746af60d62bc3ac06a5fffc0 | Bacteria | p__Acidobacteriota | c__Holophagae | o__Subgroup_7 | f__Subgroup_7 | g__Subgroup_7 |  |
| 04928054 | Bacteria | p__Proteobacteria | c__Alphaproteobacteria | o__Sphingomonadales | f__Sphingomonadaceae | g__Sphingomonas |  |
| 4791dea26720aff11c2db288a265c0cd | Bacteria | p__Proteobacteria | c__Gammaproteobacteria | o__Burkholderiales | f__Comamonadaceae | g__Lampropedia | s__Lampropedia_sp. |
| b0ee83dcb96f71cbb8f724b01f0b2c60 | Bacteria | p__Firmicutes | c__Bacilli | o__Lactobacillales | f__Enterococcaceae | g__Enterococcus |  |
| a1f7363baf6cf884a681ef3e7257cca5 | Bacteria | p__Proteobacteria | c__Gammaproteobacteria | o__Enterobacterales | f__Enterobacteriaceae |  |  |
| 26be9c348e845a89fcb4727be8b088ac | Bacteria | p__Proteobacteria | c__Gammaproteobacteria | o__Burkholderiales | f__Comamonadaceae | g__Comamonas |  |
| a95f29c2096005032501ffa526379f44 | Bacteria | p__Proteobacteria | c__Gammaproteobacteria | o__Aeromonadales | f__Aeromonadaceae | g__Aeromonas |  |
| MJ010-1-barcode25-umi115232bins-ubs-5 | Bacteria | p__Bacteroidota | c__Bacteroidia | o__Chitinophagales | f__Chitinophagaceae | g__Ferruginibacter |  |
| RS-GCF-000613205.1-NZ-CBXW010000058.1 | Bacteria | p__Proteobacteria | c__Gammaproteobacteria | o__Xanthomonadales | f__Xanthomonadaceae | g__Stenotrophomonas |  |
| 034364576382c511a2e446bcaebcd1e4 | Bacteria | p__Bacteroidota | c__Bacteroidia | o__Flavobacteriales | f__Flavobacteriaceae | g__Flavobacterium | s__Flavobacterium_ceti |
| e9de61b663c5a78973ac84d01a5db4f2 | Bacteria | p__Actinobacteriota | c__Acidimicrobiia | o__Microtrichales | f__Ilumatobacteraceae | g__CL500-29_marine_group |  |
| 15dded9ecd838fb513e8725b65a518af | Bacteria | p__Firmicutes | c__Bacilli | o__Paenibacillales | f__Paenibacillaceae | g__Paenibacillus |  |
| 03faded19ffa19dfaea5cce752d6c0b7 | Bacteria | p__Proteobacteria | c__Gammaproteobacteria | o__Burkholderiales | f__Burkholderiaceae | g__Ralstonia |  |
| 05013038 | Bacteria | p__Bacteroidota | c__Bacteroidia | o__Chitinophagales | f__Chitinophagaceae |  |  |
| 19782d6cab7573bccc61e373d079717d | Bacteria | p__Proteobacteria | c__Alphaproteobacteria | o__Sphingomonadales | f__Sphingomonadaceae |  |  |
| 781ffcc992e2b94271946e5ae7b8c53f | Bacteria | p__Firmicutes | c__Bacilli | o__Staphylococcales | f__Staphylococcaceae | g__Staphylococcus |  |
| 08623386 | Bacteria | p__Proteobacteria | c__Alphaproteobacteria | o__Rhizobiales | f__Devosiaceae | g__Devosia |  |
| MJ020-2-barcode71-umi36798bins-ubs-12 | Bacteria | p__Acidobacteriota | c__Acidobacteriae | o__Acidobacteriales | f__Acidobacteriaceae_(Subgroup_1) | g__Terriglobus |  |
| b1d165018f7ba70911302a0541561fe3 | Bacteria | p__Actinobacteriota | c__Actinobacteria | o__Micrococcales | f__Intrasporangiaceae |  |  |
| G003457795_2 | Bacteria | p__Firmicutes | c__Clostridia | o__Lachnospirales | f__Lachnospiraceae | g__Roseburia |  |
| 7c42275a2dfe7394fbe0e6b10c3e8f09 | Bacteria | p__NB1-j | c__NB1-j | o__NB1-j | f__NB1-j | g__NB1-j |  |
| bd12838cd4d9ac0badc9d6ffc107e2a5 | Bacteria | p__Chloroflexi | c__TK10 | o__TK10 | f__TK10 | g__TK10 | s__bacterium_Ellin6519 |
| MJ010-2-barcode64-umi55581bins-ubs-14 | Bacteria | p__Proteobacteria | c__Gammaproteobacteria | o__Cellvibrionales | f__Cellvibrionaceae | g__Cellvibrio | s__Cellvibrio_fontiphilus |
| ca65a89e166bdfc31a93ef903cf0c511 | Bacteria | p__Firmicutes | c__Bacilli | o__Bacillales | f__Bacillaceae | g__Bacillus |  |
| d0bc54c48844401bff1d24d4c42139cb | Bacteria | p__Proteobacteria | c__Gammaproteobacteria | o__Burkholderiales | f__Comamonadaceae |  |  |
| 975d24a6410edb500c63ae160d5940d7 | Bacteria | p__Proteobacteria | c__Alphaproteobacteria | o__Caulobacterales | f__Caulobacteraceae |  |  |
| 04925203 | Bacteria | p__Proteobacteria | c__Alphaproteobacteria | o__Sphingomonadales | f__Sphingomonadaceae | g__Sphingomonas |  |
| 2efc5be0fb11698a227121b72e06ff5a | Bacteria | p__Proteobacteria | c__Gammaproteobacteria | o__Burkholderiales | f__Comamonadaceae | g__Aquabacterium |  |
| c90092e07fc916639536cde09aed75cf | Bacteria | p__Firmicutes | c__Bacilli | o__Alicyclobacillales | f__Alicyclobacillaceae | g__Tumebacillus |  |
| 05949133 | Bacteria | p__Acidobacteriota | c__Vicinamibacteria | o__Vicinamibacterales | f__Vicinamibacteraceae | g__Luteitalea |  |
| AJ415384 | Bacteria | p__Actinobacteriota | c__Actinobacteria | o__Micrococcales | f__Dermabacteraceae | g__Brachybacterium |  |
| MJ010-2-barcode64-umi87275bins-ubs-6 | Bacteria | p__Bacteroidota | c__Bacteroidia | o__Sphingobacteriales | f__Sphingobacteriaceae | g__Sphingobacterium |  |
| GB-GCA-015554585.1-JADNIT010000076.1 | Bacteria | p__Firmicutes | c__Clostridia | o__Lachnospirales | f__Lachnospiraceae | g__Agathobacter |  |
| e10aa8400430fb62a87c3399efdffe0e | Bacteria | p__Acidobacteriota | c__Acidobacteriae | o__Subgroup_2 | f__Subgroup_2 | g__Subgroup_2 |  |
| MJ007-2-barcode68-umi32871bins-ubs-8 | Bacteria | p__Acidobacteriota | c__Blastocatellia | o__Blastocatellales | f__Blastocatellaceae |  |  |
| 04946270 | Bacteria | p__Actinobacteriota | c__Actinobacteria | o__Micrococcales | f__Beutenbergiaceae |  |  |
| 00bbc5508b224d5e4a013adbc72621c6 | Bacteria | p__Bacteroidota | c__Bacteroidia | o__Sphingobacteriales | f__Sphingobacteriaceae | g__Sphingobacterium |  |
| RS-GCF-001425725.1-NZ-LMDD01000002.1 | Bacteria | p__Proteobacteria | c__Alphaproteobacteria | o__Caulobacterales | f__Caulobacteraceae | g__Caulobacter |  |
| MJ009-1-barcode27-umi15617bins-ubs-28 | Bacteria | p__Firmicutes | c__Clostridia | o__Clostridiales | f__Clostridiaceae | g__Clostridium_sensu_stricto_1 |  |
| 0b93e3563d67e57c0bac99b5fcad60e2 | Bacteria | p__Acidobacteriota | c__Vicinamibacteria | o__Vicinamibacterales | f__Vicinamibacteraceae | g__Vicinamibacter |  |
| L76596 | Bacteria | p__Firmicutes | c__Clostridia | o__Oscillospirales | f__Ruminococcaceae | g__Ruminococcus | s__Ruminococcus_callidus |
| MJ007-1-barcode36-umi32764bins-ubs-4 | Bacteria | p__Proteobacteria | c__Gammaproteobacteria | o__Burkholderiales | f__Comamonadaceae |  |  |
| 04994024 | Bacteria | p__Proteobacteria | c__Alphaproteobacteria | o__Caulobacterales | f__Caulobacteraceae | g__Brevundimonas |  |
| 04966030 | Bacteria | p__Firmicutes | c__Bacilli | o__Bacillales |  |  |  |
| b39aac6d0753acb4f4cd8055a9926140 | Bacteria | p__Myxococcota | c__Myxococcia | o__Myxococcales | f__Anaeromyxobacteraceae | g__Anaeromyxobacter |  |
| EF100698 | Bacteria | p__Proteobacteria | c__Gammaproteobacteria | o__Xanthomonadales | f__Xanthomonadaceae | g__Lysobacter |  |
| 04940983 | Bacteria | p__Acidobacteriota | c__Blastocatellia | o__Pyrinomonadales | f__Pyrinomonadaceae | g__RB41 | s__uncultured_Acidobacteriaceae |
| 31b2f88c2ea07b5591beeb4edf6357f3 | Bacteria | p__Bacteroidota | c__Bacteroidia | o__Bacteroidales | f__Muribaculaceae | g__Muribaculaceae | s__uncultured_bacterium |
| 3bfa4fc475180c4fe46ffeb24681ecdd | Bacteria | p__Proteobacteria | c__Gammaproteobacteria | o__Enterobacterales |  |  |  |
| 115ce89ae73b216466e58c067f599faa | Bacteria | p__Proteobacteria | c__Gammaproteobacteria | o__Pseudomonadales | f__Moraxellaceae | g__Acinetobacter |  |
| 590496c94441fdf68a1ce07743bbaa05 | Bacteria | p__Proteobacteria | c__Alphaproteobacteria | o__Rhodobacterales | f__Rhodobacteraceae | g__Gemmobacter |  |
| f4e0d2fcb1678a85b9aaae3f8b1ecd13 | Bacteria | p__Proteobacteria | c__Gammaproteobacteria | o__Pseudomonadales | f__Pseudomonadaceae | g__Pseudomonas |  |
| KJ461685 | Bacteria | p__Bacteroidota | c__Bacteroidia | o__Flavobacteriales | f__Flavobacteriaceae | g__Flavobacterium | s__Flavobacterium_seoulense |
| G000382165_1 | Bacteria | p__Proteobacteria | c__Gammaproteobacteria | o__Alteromonadales | f__Alteromonadaceae | g__Rheinheimera |  |
| RS-GCF-011603095.1-NZ-JAANAW010000007.1 | Bacteria | p__Proteobacteria | c__Gammaproteobacteria | o__Aeromonadales | f__Aeromonadaceae | g__Aeromonas |  |
| 64f470e080e0108ae771e1ed73c1fd18 | Bacteria | p__Firmicutes | c__Bacilli | o__Erysipelotrichales | f__Erysipelotrichaceae | g__Dubosiella | s__uncultured_bacterium |
| 6d4cfd5e78d45dc680f396900870f3b0 | Bacteria | p__Actinobacteriota | c__Actinobacteria | o__Corynebacteriales | f__Mycobacteriaceae | g__Mycobacterium |  |
| RS-GCF-016902445.1-NZ-JACSNX010000046.1 | Bacteria | p__Firmicutes | c__Clostridia | o__Oscillospirales | f__Oscillospiraceae | g__Oscillibacter | s__uncultured_organism |
| 02251099 | Bacteria | p__Firmicutes | c__Clostridia | o__Peptostreptococcales-Tissierellales | f__Peptostreptococcales-Tissierellales | g__Anaerococcus |  |
| 514a0d9cf30647af4c7596de89219bb1 | Bacteria | p__Proteobacteria | c__Gammaproteobacteria | o__Enterobacterales | f__Enterobacteriaceae |  |  |
| 72580c0a8f79d3d0af1945b47782ad6d | Bacteria | p__Proteobacteria | c__Gammaproteobacteria | o__Burkholderiales | f__SC-I-84 | g__SC-I-84 |  |
| 16403003 | Bacteria | p__Actinobacteriota | c__Coriobacteriia | o__Coriobacteriales | f__Eggerthellaceae | g__Enterorhabdus | s__uncultured_bacterium |
| 4cd17670fc371efea5e599e93b1dabc9 | Bacteria | p__Proteobacteria | c__Gammaproteobacteria | o__Burkholderiales | f__Nitrosomonadaceae | g__MND1 |  |
| MJ034-1-barcode34-umi24949bins-ubs-5 | Bacteria | p__Proteobacteria | c__Gammaproteobacteria | o__Burkholderiales | f__Nitrosomonadaceae | g__mle1-7 | s__uncultured_Burkholderiales |
| 2c41473ec4394c56521e42e530e744a2 | Bacteria | p__Proteobacteria | c__Alphaproteobacteria | o__Caulobacterales | f__Caulobacteraceae | g__uncultured |  |
| d33321655cdf976c4b78c0d0a1804cfa | Bacteria | p__Firmicutes | c__Bacilli | o__Lactobacillales | f__Lactobacillaceae | g__Lactobacillus |  |
| aa7685cc9dfcd06c74d58be861ea0da5 | Bacteria | p__Firmicutes | c__Bacilli | o__Bacillales | f__Marinococcaceae | g__Alteribacillus |  |
| 0e007777d02f1a682821139ca1eee316 | Bacteria | p__Proteobacteria | c__Gammaproteobacteria | o__Burkholderiales | f__Comamonadaceae |  |  |
| MJ031-1-barcode30-umi19573bins-ubs-20 | Bacteria | p__Proteobacteria | c__Gammaproteobacteria | o__Burkholderiales | f__Rhodocyclaceae |  |  |
| adcb2f5561ca47511dda392ddb3dae07 | Bacteria | p__Actinobacteriota | c__Actinobacteria | o__Micrococcales | f__Micrococcaceae | g__Glutamicibacter | s__uncultured_bacterium |
| MJ006-2-barcode73-umi187671bins-ubs-3 | Bacteria | p__Bacteroidota | c__Bacteroidia | o__Bacteroidales | f__Prevotellaceae | g__Prevotella |  |
| G000702305_1 | Bacteria | p__Proteobacteria | c__Alphaproteobacteria | o__Rhizobiales | f__Xanthobacteraceae |  |  |
| 8436e413a9f35d0594d882f1bddfd97a | Bacteria | p__Firmicutes | c__Clostridia | o__Oscillospirales | f__Oscillospiraceae | g__uncultured |  |
| MJ010-1-barcode47-umi116660bins-ubs-5 | Bacteria | p__Proteobacteria | c__Gammaproteobacteria | o__Pseudomonadales | f__Moraxellaceae | g__Acinetobacter |  |
| 04924098 | Bacteria | p__Bacteroidota | c__Bacteroidia | o__Bacteroidales | f__Muribaculaceae | g__Muribaculaceae |  |
| 04959168 | Bacteria | p__Bacteroidota | c__Bacteroidia | o__Cytophagales | f__Cytophagaceae | g__Siphonobacter | s__Siphonobacter_sp. |
| 5f5e0466d727935c3b2bdeab8b8339e7 | Bacteria | p__Proteobacteria | c__Alphaproteobacteria | o__Rhodobacterales | f__Rhodobacteraceae |  |  |
| ab9b67548a4bc3385bd05c682544bb16 | Bacteria | p__Bacteroidota | c__Bacteroidia | o__Flavobacteriales | f__Flavobacteriaceae | g__Myroides |  |
| e7ef738d000fcaec6c8225ed17855b67 | Bacteria | p__Chloroflexi |  |  |  |  |  |
| b1576b205819d872068b969076362b61 | Bacteria | p__Proteobacteria | c__Gammaproteobacteria | o__Burkholderiales | f__Rhodocyclaceae |  |  |
| RS-GCF-013387485.1-NZ-JABBXB010000001.1 | Bacteria | p__Proteobacteria | c__Gammaproteobacteria | o__Xanthomonadales | f__Xanthomonadaceae | g__Stenotrophomonas |  |
| 1db5cdd65ac26a2c1f109f42542f0687 | Bacteria | p__Methylomirabilota | c__Methylomirabilia | o__Rokubacteriales | f__Rokubacteriales | g__Rokubacteriales |  |
| 21cb133a5249855262b89e91ce82aed9 | Bacteria | p__Proteobacteria | c__Gammaproteobacteria | o__Alteromonadales |  |  |  |
| Z78211 | Bacteria | p__Actinobacteriota | c__Actinobacteria | o__Propionibacteriales | f__Nocardioidaceae | g__Nocardioides |  |
| f2d1c29c4f5b17a7643cb41fffac1a6e | Bacteria | p__Firmicutes | c__Bacilli | o__Lactobacillales | f__Carnobacteriaceae | g__Atopostipes |  |
| MJ010-1-barcode36-umi134287bins-ubs-4 | Bacteria | p__Firmicutes | c__Clostridia | o__Lachnospirales | f__Lachnospiraceae | g__Lachnospiraceae_FCS020_group | s__uncultured_prokaryote |
| 24697168179fd61da24afcc3565f2fb9 | Bacteria | p__Bacteroidota | c__Kapabacteria | o__Kapabacteriales | f__Kapabacteriales | g__Kapabacteriales | s__metagenome |
| 1e85af412442a0de4c6e9fa7b63fb221 | Bacteria | p__Desulfobacterota | c__uncultured | o__uncultured | f__uncultured | g__uncultured |  |
| e467a125de12793d44241dc28b1adc47 | Bacteria | p__Proteobacteria | c__Gammaproteobacteria | o__Xanthomonadales | f__Rhodanobacteraceae | g__Ahniella |  |
| 02355920 | Bacteria | p__Actinobacteriota | c__Actinobacteria | o__Bifidobacteriales | f__Bifidobacteriaceae | g__Bifidobacterium |  |
| f1b0cca6fc47b53d7599c935a9436596 | Bacteria | p__Actinobacteriota | c__Actinobacteria | o__Pseudonocardiales | f__Pseudonocardiaceae | g__Prauserella |  |
| RS-GCF-003970855.1-NZ-RWKO01000042.1 | Bacteria | p__Firmicutes | c__Bacilli | o__Lactobacillales | f__Lactobacillaceae | g__Lactobacillus |  |
| 03158521 | Bacteria | p__Planctomycetota | c__Phycisphaerae | o__Tepidisphaerales | f__WD2101_soil_group | g__WD2101_soil_group |  |
| 04206226 | Bacteria | p__Chloroflexi | c__Chloroflexia | o__Thermomicrobiales | f__JG30-KF-CM45 | g__JG30-KF-CM45 |  |
| RS-GCF-000369585.1-NZ-KB850094.1--2 | Bacteria | p__Proteobacteria | c__Gammaproteobacteria | o__Pseudomonadales | f__Moraxellaceae | g__Acinetobacter |  |
| f5d55360ac13c333157b0159906a34bc | Bacteria | p__Bacteroidota | c__Bacteroidia | o__Sphingobacteriales | f__Sphingobacteriaceae | g__Sphingobacterium |  |
| 540602541cfe5bdf909b3876e0bb6713 | Bacteria | p__Gemmatimonadota | c__Gemmatimonadetes | o__Gemmatimonadales | f__Gemmatimonadaceae | g__Gemmatimonas |  |
| 5d172eb9c6079f0f54ba7da439bd743d | Bacteria | p__Gemmatimonadota | c__Gemmatimonadetes | o__Gemmatimonadales | f__Gemmatimonadaceae | g__uncultured |  |
| 257ca6bf68928aa8bca2a4264ce6c91d | Bacteria | p__Bacteroidota | c__Bacteroidia | o__Bacteroidales | f__Rikenellaceae | g__uncultured |  |
| a40e517086db74efc70bca956b6f7151 | Bacteria | p__Actinobacteriota | c__Acidimicrobiia |  |  |  |  |
| 17968211 | Bacteria | p__Chloroflexi | c__Ktedonobacteria | o__Ktedonobacterales | f__Ktedonobacteraceae | g__uncultured | s__uncultured_soil |
| a99d1b359c6b6d5298dcaf004683ea7d | Bacteria | p__Firmicutes | c__Clostridia | o__Lachnospirales | f__Lachnospiraceae | g__Tyzzerella | s__uncultured_Clostridium |
| MJ032-2-barcode72-umi56642bins-ubs-8 | Bacteria | p__Proteobacteria | c__Gammaproteobacteria | o__Cellvibrionales | f__Halieaceae | g__OM60(NOR5)_clade |  |
| b7e2423a3d0c654df627915575010720 | Bacteria | p__Proteobacteria | c__Gammaproteobacteria | o__Xanthomonadales | f__Xanthomonadaceae | g__Stenotrophomonas |  |
| ec02769eb19cf686c5ca3873e826fc65 | Bacteria | p__Actinobacteriota | c__Coriobacteriia | o__Coriobacteriales | f__Coriobacteriaceae | g__Collinsella |  |
| 929f26d2765d65432fc903231293fba6 | Bacteria | p__Actinobacteriota | c__Thermoleophilia | o__Gaiellales | f__uncultured | g__uncultured |  |
| 353e554f6e62e19a186df9a0b8cfc881 | Bacteria |  |  |  |  |  |  |
| 028a8849b104fe364600b9a22944b13d | Bacteria | p__Proteobacteria | c__Gammaproteobacteria | o__PLTA13 | f__PLTA13 | g__PLTA13 |  |
| 909ede0996d8ab1dfe05a6db1b8e6ff7 | Bacteria | p__Bacteroidota | c__Bacteroidia | o__Bacteroidales | f__Muribaculaceae | g__Muribaculaceae |  |
| DQ322069 | Bacteria | p__Proteobacteria | c__Gammaproteobacteria | o__Burkholderiales | f__Comamonadaceae | g__Comamonas |  |
| 8d6805d1c3f3edcc4f4abab49cfe2df8 | Bacteria | p__Proteobacteria | c__Gammaproteobacteria |  |  |  |  |
| b7fe525b521873586d33986447c28a81 | Bacteria | p__Proteobacteria | c__Alphaproteobacteria | o__Sphingomonadales | f__Sphingomonadaceae | g__Sphingomonas |  |
| d716eaf18bfe19276adacacf899e4730 | Bacteria | p__Bacteroidota | c__Bacteroidia | o__Bacteroidales |  |  |  |
| 85b662598b1f483b9b40e37b8b0f7065 | Bacteria | p__Acidobacteriota | c__Thermoanaerobaculia | o__Thermoanaerobaculales | f__Thermoanaerobaculaceae | g__Subgroup_10 | s__uncultured_Geoalkalibacter |
| MJ032-1-barcode26-umi55680bins-ubs-6 | Bacteria | p__Cyanobacteria | c__Cyanobacteriia | o__Cyanobacteriales | f__Cyanobacteriales_Incertae_Sedis | g__Microseira_Carmichael-Alabama | s__uncultured_bacterium |
| 427dac522ee873378669e70645c11b07 | Bacteria | p__Firmicutes | c__Clostridia | o__Lachnospirales | f__Lachnospiraceae | g__Anaerostipes |  |
| 6f720af9ee8eb483bdce742838cd3eef | Bacteria | p__Bacteroidota | c__Bacteroidia | o__Bacteroidales | f__Bacteroidaceae | g__Bacteroides | s__Bacteroides_barnesiae |
| RS-GCF-016862955.1-NZ-BONY01000175.1 | Bacteria | p__Actinobacteriota | c__Actinobacteria | o__Micromonosporales | f__Micromonosporaceae | g__Rhizocola |  |
| 924202214dcc5dc9789b9cdd6405b2ba | Bacteria | p__Firmicutes | c__Bacilli | o__Staphylococcales | f__Staphylococcaceae | g__Staphylococcus |  |
| 07caa06b1d9edeae8e5ed2a4d9784662 | Bacteria | p__Proteobacteria | c__Gammaproteobacteria | o__Enterobacterales | f__Enterobacteriaceae |  |  |
| 08808023 | Bacteria | p__Proteobacteria | c__Gammaproteobacteria | o__Xanthomonadales | f__Rhodanobacteraceae | g__Rhodanobacter | s__uncultured_Xanthomonadaceae |
| 77513cc9f764040a6b8c80bc88dc5092 | Bacteria | p__Firmicutes | c__Bacilli | o__Lactobacillales | f__Enterococcaceae | g__Enterococcus |  |
| b1d74bcdcf1cf8f19a11c307a196965c | Bacteria | p__Acidobacteriota | c__Vicinamibacteria | o__Vicinamibacterales | f__Vicinamibacteraceae | g__Vicinamibacteraceae |  |
| 3d9c4a72f7cf05d02cf036c016cbd4aa | Bacteria | p__Campilobacterota | c__Campylobacteria | o__Campylobacterales | f__Sulfurimonadaceae | g__Sulfuricurvum |  |
| 584a2b197a24e5a9d4416723f3b535d9 | Bacteria | p__Proteobacteria | c__Gammaproteobacteria | o__Burkholderiales | f__Comamonadaceae | g__Aquabacterium |  |
| 059f3f4b2e6f9a903c166964f1ba5ac3 | Bacteria | p__Proteobacteria | c__Gammaproteobacteria | o__Burkholderiales | f__Burkholderiaceae |  |  |
| MJ034-1-barcode30-umi25209bins-ubs-5 | Bacteria | p__Acidobacteriota | c__Acidobacteriae | o__Solibacterales | f__Solibacteraceae | g__Candidatus_Solibacter |  |
| 61d95b864f654884ac4e61abde7a4e62 | Bacteria | p__Verrucomicrobiota | c__Verrucomicrobiae | o__Chthoniobacterales | f__Chthoniobacteraceae | g__Candidatus_Udaeobacter | s__uncultured_Spartobacteria |
| MJ006-1-barcode28-umi123590bins-ubs-4 | Bacteria | p__Firmicutes | c__Clostridia | o__Oscillospirales | f__Oscillospiraceae | g__UCG-002 |  |
| 08792562 | Bacteria | p__Proteobacteria | c__Gammaproteobacteria | o__Xanthomonadales | f__Xanthomonadaceae | g__Lysobacter |  |
| MJ006-1-barcode34-umi121582bins-ubs-4 | Bacteria | p__Firmicutes | c__Clostridia | o__Oscillospirales | f__Oscillospiraceae | g__UCG-005 | s__uncultured_organism |
| MJ034-1-barcode31-umi11561bins-ubs-33 | Bacteria | p__Acidobacteriota | c__Acidobacteriae | o__Acidobacteriales | f__uncultured | g__uncultured |  |
| LC405931 | Bacteria | p__Actinobacteriota | c__Actinobacteria | o__Kineosporiales | f__Kineosporiaceae | g__Quadrisphaera | s__Quadrisphaera_granulorum |
| 02360386 | Bacteria | p__Proteobacteria | c__Alphaproteobacteria | o__Rhizobiales | f__Hyphomicrobiaceae | g__Pedomicrobium |  |
| 05006731 | Bacteria | p__Bacteroidota | c__Bacteroidia | o__Bacteroidales | f__Muribaculaceae | g__Muribaculaceae | s__uncultured_Bacteroidales |
| 56e75bc6639d0e6423f06b6375cb6740 | Bacteria | p__Verrucomicrobiota | c__Verrucomicrobiae | o__Chthoniobacterales | f__Chthoniobacteraceae | g__Candidatus_Udaeobacter |  |
| FR733696 | Bacteria | p__Proteobacteria | c__Alphaproteobacteria | o__Rhizobiales | f__Beijerinckiaceae | g__Rhodoblastus |  |
| a1afb0a4957d2afaad281df6c27ec223 | Bacteria | p__Proteobacteria | c__Alphaproteobacteria | o__Sphingomonadales | f__Sphingomonadaceae | g__Sphingomonas |  |
| 13720358 | Bacteria | p__Gemmatimonadota | c__Gemmatimonadetes | o__Gemmatimonadales | f__Gemmatimonadaceae | g__Gemmatimonas |  |
| 14836037 | Bacteria | p__Chloroflexi | c__Ktedonobacteria | o__C0119 | f__C0119 | g__C0119 | s__uncultured_bacterium |
| MJ034-1-barcode30-umi23015bins-ubs-7 | Bacteria | p__Nitrospirota | c__Nitrospiria | o__Nitrospirales | f__Nitrospiraceae | g__Nitrospira |  |
| 25eae459691c0cbb401adec032c3446a | Bacteria | p__Chloroflexi | c__KD4-96 | o__KD4-96 | f__KD4-96 | g__KD4-96 | s__uncultured_Anaerolineaceae |
| 8b85bf7dba0950d793e7beb00a8c9c58 | Bacteria | p__Proteobacteria | c__Gammaproteobacteria | o__Burkholderiales | f__Oxalobacteraceae | g__Massilia |  |
| 04919010 | Bacteria | p__Bacteroidota | c__Bacteroidia | o__Sphingobacteriales | f__Sphingobacteriaceae | g__Sphingobacterium |  |
| 2f97d46c4ed60a58e527d62c44bef3a0 | Bacteria | p__Actinobacteriota | c__Acidimicrobiia | o__Microtrichales | f__uncultured | g__uncultured |  |
| 698165c434c4d3f835d4194a3ebea1d2 | Bacteria | p__Proteobacteria | c__Gammaproteobacteria | o__Burkholderiales | f__A21b | g__A21b |  |
| 01831661 | Bacteria | p__Actinobacteriota | c__MB-A2-108 | o__MB-A2-108 | f__MB-A2-108 | g__MB-A2-108 | s__metagenome |
| MJ006-1-barcode43-umi109112bins-ubs-4 | Bacteria | p__Proteobacteria | c__Gammaproteobacteria | o__Burkholderiales | f__Sutterellaceae | g__Sutterella | s__Sutturella_timonensis |
| adf006e3091262953df83143c046297b | Bacteria | p__Actinobacteriota | c__Actinobacteria | o__Micrococcales | f__Brevibacteriaceae | g__Brevibacterium |  |
| G900232865_1 | Bacteria | p__Actinobacteriota | c__Actinobacteria | o__Corynebacteriales | f__Corynebacteriaceae | g__Corynebacterium | s__Corynebacterium_falsenii |
| f057725ff15f64234870031f30c3445e | Bacteria | p__NB1-j | c__NB1-j | o__NB1-j | f__NB1-j | g__NB1-j |  |
| 05002862 | Bacteria | p__Bacteroidota | c__Bacteroidia | o__Sphingobacteriales | f__Sphingobacteriaceae | g__Mucilaginibacter |  |
| 938d45ed69c88d942fc041160fcd8ad3 | Bacteria | p__Proteobacteria | c__Alphaproteobacteria | o__Acetobacterales | f__Acetobacteraceae | g__Roseomonas |  |
| d561daf71c0cb064b3a74447b0360355 | Bacteria | p__Bdellovibrionota | c__Bdellovibrionia | o__Bdellovibrionales | f__Bdellovibrionaceae | g__Bdellovibrio | s__uncultured_bacterium |
| 5bb604bfc7b342c26e1d05336b1db33a | Bacteria | p__Firmicutes | c__Bacilli | o__Lactobacillales | f__Enterococcaceae | g__Enterococcus |  |
| 8f21292e7da84a6509a9843ff40f19fa | Bacteria | p__Firmicutes | c__Bacilli | o__Lactobacillales | f__Enterococcaceae | g__Enterococcus |  |
| a2c633a3b0b808f85851b5c8f609899f | Bacteria | p__Acidobacteriota | c__Acidobacteriae | o__Subgroup_2 | f__Subgroup_2 | g__Subgroup_2 | s__uncultured_forest |
| 58cb08269251ed7ef1b6a78cbb9ced4b | Bacteria | p__Proteobacteria | c__Alphaproteobacteria | o__Caulobacterales | f__Hyphomonadaceae | g__SWB02 | s__uncultured_bacterium |
| 81ac2cef42586632c9caee10d4f87a7f | Bacteria | p__Bacteroidota | c__Bacteroidia | o__Sphingobacteriales | f__Sphingobacteriaceae |  |  |
| MJ010-1-barcode25-umi110055bins-ubs-5 | Bacteria | p__Bacteroidota | c__Bacteroidia | o__Chitinophagales | f__Chitinophagaceae | g__Sediminibacterium |  |
| MH801891 | Bacteria | p__Bacteroidota | c__Bacteroidia | o__Sphingobacteriales | f__Sphingobacteriaceae | g__Mucilaginibacter |  |
| MN328031 | Bacteria | p__Bacteroidota | c__Bacteroidia | o__Flavobacteriales | f__Weeksellaceae | g__Chryseobacterium |  |
| 497dabc07b3b447e837bbc105100f3ae | Bacteria |  |  |  |  |  |  |
| fd69f648ee640fac49fe8dbb976e30a6 | Bacteria | p__Proteobacteria | c__Alphaproteobacteria | o__Caulobacterales | f__Caulobacteraceae | g__uncultured |  |
| MJ005-barcode35-umi81300bins-ubs-3 | Bacteria | p__Proteobacteria | c__Gammaproteobacteria | o__Pasteurellales | f__Pasteurellaceae | g__Haemophilus |  |
| RS-GCF-009866445.1-NZ-ML956146.1 | Bacteria | p__Proteobacteria | c__Gammaproteobacteria | o__Enterobacterales | f__Erwiniaceae |  |  |
| MJ006-1-barcode30-umi59078bins-ubs-6 | Bacteria | p__Firmicutes | c__Bacilli | o__Erysipelotrichales | f__Erysipelotrichaceae | g__Turicibacter |  |
| JF495126 | Bacteria | p__Proteobacteria | c__Gammaproteobacteria | o__Burkholderiales | f__Comamonadaceae | g__Variovorax |  |
| 90422a76eb7704830956589efaba20dd | Bacteria | p__Actinobacteriota | c__Actinobacteria | o__Micrococcales | f__Microbacteriaceae | g__Microbacterium |  |
| 0ecb710c60cbb91791b03218840ed4d6 | Bacteria | p__Firmicutes | c__Bacilli | o__Caldalkalibacillales | f__Caldalkalibacillaceae | g__Caldalkalibacillus | s__Caldalkalibacillus_thermarum |
| 05092844 | Bacteria | p__Proteobacteria | c__Alphaproteobacteria | o__Rhizobiales | f__Xanthobacteraceae | g__Pseudolabrys |  |
| 769e223f8ca23b6b95afa8c2def992fb | Bacteria | p__Firmicutes | c__Bacilli | o__Bacillales | f__Bacillaceae | g__Bacillus |  |
| b7d664f62fa8ababa2b44219e04b9936 | Bacteria | p__Proteobacteria | c__Gammaproteobacteria | o__Enterobacterales | f__Enterobacteriaceae |  |  |
| RS-GCF-000210015.1-NC-021022.1 | Bacteria | p__Firmicutes | c__Clostridia | o__Lachnospirales | f__Lachnospiraceae | g__Blautia |  |
| fa59c6ccac820a03c3a6ea5b7473a74d | Bacteria | p__Chloroflexi | c__Anaerolineae | o__SBR1031 | f__A4b | g__A4b |  |
| 8a275a734b2e265639aabb8d499724f1 | Bacteria | p__Chloroflexi | c__Anaerolineae | o__Caldilineales | f__Caldilineaceae | g__uncultured | s__metagenome |
| 7764844e65a89d5fb9616a4ff3c4ecf0 | Bacteria |  |  |  |  |  |  |
| 8b3aca4710154b79866cd80f2c9310d3 | Bacteria | p__Bacteroidota | c__Bacteroidia | o__Bacteroidales | f__Tannerellaceae | g__Macellibacteroides |  |
| 3914c2c02ec5036dfe0e6f36cc3fe3d3 | Bacteria | p__Firmicutes | c__Clostridia | o__Lachnospirales | f__Lachnospiraceae |  |  |
| 484e79eb2096377ac2a6c682ba9505dc | Bacteria | p__Proteobacteria | c__Gammaproteobacteria | o__Burkholderiales | f__Alcaligenaceae | g__Bordetella |  |
| 95ae00d0347651d4466dd9547899654f | Bacteria | p__Bacteroidota | c__Bacteroidia | o__Bacteroidales | f__Muribaculaceae | g__Muribaculaceae |  |
| 1b15e8aeff73461347769cfef8442bce | Bacteria | p__Bacteroidota | c__Bacteroidia | o__Flavobacteriales | f__Weeksellaceae |  |  |
| 66df69ea93b054ab0689b6114bde5c0d | Bacteria | p__Firmicutes | c__Bacilli | o__Lactobacillales | f__Lactobacillaceae | g__Lactobacillus |  |
| 04961321 | Bacteria | p__Actinobacteriota | c__Actinobacteria | o__Streptomycetales | f__Streptomycetaceae |  |  |
| 08489373 | Bacteria | p__Proteobacteria | c__Alphaproteobacteria | o__Rhizobiales | f__Rhizobiaceae | g__Allorhizobium-Neorhizobium-Pararhizobium-Rhizobium |  |
| 04960536 | Bacteria | p__Chloroflexi | c__TK10 | o__TK10 | f__TK10 | g__TK10 |  |
| 31697845837e4c646434587dda5d0f21 | Bacteria | p__Bdellovibrionota | c__Bdellovibrionia | o__Bacteriovoracales | f__Bacteriovoracaceae | g__Peredibacter |  |
| 05038523 | Bacteria | p__Firmicutes | c__Bacilli | o__Lactobacillales | f__Enterococcaceae | g__Enterococcus |  |
| GB-GCA-002083355.1-LVWU01000135.1 | Bacteria | p__Nitrospirota | c__Nitrospiria | o__Nitrospirales | f__Nitrospiraceae | g__Nitrospira |  |
| d27fe0a8b3a4a40507cc004393781039 | Bacteria | p__Actinobacteriota | c__Actinobacteria | o__Micrococcales | f__Micrococcaceae |  |  |
| dd8d09109c0d7dea3fb83f3c4128379a | Bacteria | p__Firmicutes | c__Bacilli | o__Erysipelotrichales | f__Erysipelatoclostridiaceae | g__Erysipelatoclostridium | s__uncultured_Clostridium |
| 34c10309a1cfcebb92ba5a97b474a23e | Bacteria | p__Proteobacteria | c__Gammaproteobacteria | o__Burkholderiales | f__Comamonadaceae |  |  |
| 05016243 | Bacteria | p__Proteobacteria | c__Gammaproteobacteria | o__Oceanospirillales | f__Halomonadaceae | g__Halomonas |  |
| de537a32ad3a69f0330bfc0fddadc05f | Bacteria | p__Acidobacteriota | c__Acidobacteriae | o__Bryobacterales | f__Bryobacteraceae | g__Bryobacter |  |
| 19e344f84071c14a74e6b3b7ac628972 | Bacteria | p__Proteobacteria | c__Gammaproteobacteria | o__Alteromonadales | f__Alteromonadaceae | g__Rheinheimera |  |
| G011959405_1 | Bacteria | p__Bacteroidota | c__Bacteroidia | o__Bacteroidales | f__Muribaculaceae | g__Muribaculaceae | s__uncultured_bacterium |
| RS-GCF-009765645.1-NZ-WSFS01000004.1 | Bacteria | p__Proteobacteria | c__Gammaproteobacteria | o__Aeromonadales | f__Aeromonadaceae | g__Aeromonas |  |
| RS-GCF-003633655.1-NZ-RBLF01000001.1--4 | Bacteria | p__Actinobacteriota | c__Actinobacteria | o__Corynebacteriales | f__Nocardiaceae | g__Rhodococcus |  |
| 04927450 | Bacteria | p__Proteobacteria | c__Alphaproteobacteria | o__Rhodobacterales | f__Rhodobacteraceae | g__Paracoccus |  |
| 24388f2996228fb7b239f870d2112bdf | Bacteria | p__Proteobacteria | c__Alphaproteobacteria | o__Rhodobacterales | f__Rhodobacteraceae | g__Paracoccus |  |
| MJ033-1-barcode36-umi37350bins-ubs-4 | Bacteria | p__Firmicutes | c__Bacilli | o__Paenibacillales | f__Paenibacillaceae | g__Ammoniphilus |  |
| 45826b3d51c0795b24626f7030217225 | Bacteria | p__Proteobacteria | c__Gammaproteobacteria | o__Enterobacterales | f__Enterobacteriaceae |  |  |
| 06708565 | Bacteria | p__Gemmatimonadota | c__Gemmatimonadetes | o__Gemmatimonadales | f__Gemmatimonadaceae | g__Gemmatimonas | s__Gemmatimonas_sp. |
| 05031002 | Bacteria | p__Actinobacteriota | c__Actinobacteria | o__Corynebacteriales | f__Nocardiaceae | g__Rhodococcus |  |
| 04878157 | Bacteria | p__Actinobacteriota | c__Actinobacteria | o__Micrococcales | f__Brevibacteriaceae | g__Brevibacterium |  |
| 9221ffe6e2b0556c2a4f4831f2b2fa9e | Bacteria | p__Actinobacteriota | c__Thermoleophilia | o__Gaiellales |  |  |  |
| 14acd05d2a91f047b0f2f3b349bfc93a | Bacteria | p__Actinobacteriota | c__Actinobacteria | o__Frankiales | f__Geodermatophilaceae |  |  |
| da042badbcfcfc3fe62d912ab52861af | Bacteria | p__Actinobacteriota | c__Actinobacteria | o__Micrococcales | f__Microbacteriaceae | g__Microbacterium |  |
| 05021761 | Bacteria | p__Actinobacteriota | c__Actinobacteria | o__Micrococcales | f__Micrococcaceae | g__Kocuria |  |
| 111c203db401164f506a9456e7846902 | Bacteria | p__Bacteroidota | c__Bacteroidia | o__Sphingobacteriales | f__Sphingobacteriaceae | g__Sphingobacterium |  |
| RS-GCF-013004255.1-NZ-JABERL010000015.1 | Bacteria | p__Proteobacteria | c__Gammaproteobacteria | o__Pseudomonadales | f__Moraxellaceae | g__Acinetobacter |  |
| 04876855 | Bacteria | p__Proteobacteria | c__Alphaproteobacteria | o__Rhizobiales | f__Methyloligellaceae |  |  |
| c2c3eca9b0b625ffbca16ff9c38b9726 | Bacteria | p__Proteobacteria | c__Gammaproteobacteria | o__Enterobacterales | f__Enterobacteriaceae |  |  |
| EU873314 | Bacteria | p__Actinobacteriota | c__Actinobacteria | o__Micrococcales | f__Microbacteriaceae | g__Microbacterium |  |
| EF174497 | Bacteria | p__Proteobacteria | c__Alphaproteobacteria | o__Rhizobiales | f__Beijerinckiaceae | g__Methylobacterium-Methylorubrum |  |
| G001010185_4 | Bacteria | p__Firmicutes | c__Bacilli | o__Lactobacillales | f__Lactobacillaceae | g__Lactobacillus | s__Lactobacillus_fermentum |
| 52f5b1416d3e70a52bb3878cead5bf8b | Bacteria | p__Proteobacteria | c__Gammaproteobacteria | o__Pseudomonadales | f__Pseudomonadaceae | g__Pseudomonas |  |
| 33b521f09cf1446c063d2f269590de74 | Bacteria | p__Bacteroidota | c__Bacteroidia | o__Bacteroidales | f__Rikenellaceae | g__DMER64 |  |
| 08858929 | Bacteria | p__Bdellovibrionota | c__Bdellovibrionia | o__Bacteriovoracales | f__Bacteriovoracaceae | g__Peredibacter |  |
| CP002042 | Bacteria | p__Deinococcota | c__Deinococci | o__Thermales | f__Thermaceae | g__Meiothermus | s__Meiothermus_silvanus |
| 2eedd656eea928c025f84e9270692a84 | Bacteria | p__Bacteroidota | c__Bacteroidia |  |  |  |  |
| 04909812 | Bacteria | p__Proteobacteria | c__Alphaproteobacteria | o__Rhizobiales | f__Devosiaceae | g__Devosia |  |
| MJ006-2-barcode53-umi82289bins-ubs-5 | Bacteria | p__Firmicutes | c__Clostridia | o__Oscillospirales | f__Oscillospiraceae |  |  |
| 04956558 | Bacteria | p__Actinobacteriota | c__Actinobacteria | o__Corynebacteriales | f__Mycobacteriaceae | g__Mycobacterium |  |
| GQ339906 | Bacteria | p__Actinobacteriota | c__Actinobacteria | o__Propionibacteriales | f__Nocardioidaceae |  |  |
| MJ031-2-barcode61-umi48969bins-ubs-3 | Bacteria | p__Bacteroidota | c__Bacteroidia | o__Chitinophagales | f__Chitinophagaceae |  |  |
| 375378e4938e3a5ae489b25a045d1d7e | Bacteria | p__Proteobacteria | c__Alphaproteobacteria | o__Caulobacterales | f__Hyphomonadaceae | g__SWB02 |  |
| RS-GCF-001730005.1-NZ-MINM01000003.1 | Bacteria | p__Firmicutes | c__Bacilli | o__Bacillales | f__Planococcaceae | g__Planococcus |  |
| 1aa4a1d3e7d978337fafde0d9185c491 | Bacteria | p__Chloroflexi | c__Anaerolineae | o__Anaerolineae | f__Anaerolineae | g__Anaerolineae | s__uncultured_Bellilinea |
| 04958391 | Bacteria | p__Actinobacteriota | c__Actinobacteria | o__Micrococcales | f__Microbacteriaceae |  |  |
| 40ebc6a3b2e280c9bfd355feff0d328f | Bacteria | p__Firmicutes | c__Bacilli | o__Erysipelotrichales | f__Erysipelotrichaceae | g__Turicibacter | s__uncultured_bacterium |
| 12842d95df769710a82980015726c57e | Bacteria | p__Actinobacteriota | c__Actinobacteria | o__Propionibacteriales | f__Propionibacteriaceae | g__Cutibacterium |  |
| c44628034989b0443871280340a1259f | Bacteria | p__Chloroflexi | c__Dehalococcoidia | o__S085 | f__S085 | g__S085 |  |
| DQ768118 | Bacteria | p__Myxococcota | c__Myxococcia | o__Myxococcales | f__Myxococcaceae | g__Myxococcus |  |
| 87441a27e787c675cdf94a54453ee136 | Bacteria | p__Verrucomicrobiota | c__Verrucomicrobiae | o__Chthoniobacterales | f__Chthoniobacteraceae | g__Candidatus_Udaeobacter |  |
| RS-GCF-900100995.1-NZ-FMUI01000001.1 | Bacteria | p__Proteobacteria | c__Gammaproteobacteria | o__Enterobacterales | f__Enterobacteriaceae |  |  |
| 18965748 | Bacteria | p__Proteobacteria | c__Gammaproteobacteria | o__Burkholderiales | f__Oxalobacteraceae |  |  |
| 04994849 | Bacteria | p__Proteobacteria | c__Alphaproteobacteria | o__Rhizobiales | f__Rhizobiaceae | g__Ensifer |  |
| bdba3390311fa23374684437708adb7f | Bacteria | p__Proteobacteria | c__Alphaproteobacteria | o__Rhizobiales | f__Xanthobacteraceae |  |  |
| c67d4740e5248d614e6fa146ec2dc152 | Bacteria | p__Firmicutes | c__Incertae_Sedis | o__DTU014 | f__DTU014 | g__DTU014 |  |
| e55fa2138293a810955443d00b7bb09b | Bacteria | p__Proteobacteria | c__Gammaproteobacteria | o__Burkholderiales | f__Burkholderiaceae | g__Burkholderia-Caballeronia-Paraburkholderia |  |
| 199b7c3c1bd032274ce3024a117b6e95 | Bacteria | p__Firmicutes | c__Bacilli | o__Bacillales | f__Marinococcaceae | g__Alteribacillus |  |
| 29412461f821e50d8d4fb3cfe79598a2 | Bacteria | p__Bacteroidota | c__Bacteroidia | o__Chitinophagales | f__Chitinophagaceae | g__Sediminibacterium |  |
| MJ031-1-barcode28-umi10514bins-ubs-47 | Bacteria | p__Proteobacteria | c__Gammaproteobacteria | o__Burkholderiales | f__Nitrosomonadaceae | g__Nitrosospira |  |
| dd6ec6fee3c39eb242460da097f3aa3a | Bacteria | p__Desulfobacterota | c__Desulfobulbia | o__Desulfobulbales | f__Desulfobulbaceae | g__Desulfobulbus |  |
| 09867805 | Bacteria | p__Bacteroidota | c__Bacteroidia | o__Bacteroidales | f__Prevotellaceae | g__Alloprevotella |  |
| ab3db1c77465ccc0454db6256741d852 | Bacteria | p__Proteobacteria | c__Alphaproteobacteria | o__Rhizobiales | f__Beijerinckiaceae |  |  |
| e22c4fe930c600a69162d5a85e750432 | Bacteria | p__Proteobacteria | c__Gammaproteobacteria | o__Burkholderiales | f__Comamonadaceae |  |  |
| 2257c39d728e03d512cb003b789023b8 | Bacteria | p__Proteobacteria | c__Gammaproteobacteria |  |  |  |  |
| 6492eab6315ad74137741bc260f46cdd | Bacteria | p__Firmicutes | c__Bacilli | o__Bacillales | f__Planococcaceae | g__Lysinibacillus |  |
| 1982fa563cc3255fcceb80c02091e24a | Bacteria | p__Proteobacteria | c__Gammaproteobacteria | o__Pseudomonadales | f__Moraxellaceae | g__Acinetobacter |  |
| 4ac82b433c17980cc05818561cf99bcb | Bacteria | p__Actinobacteriota | c__Thermoleophilia | o__Solirubrobacterales | f__Solirubrobacteraceae |  |  |
| G001421995_1 | Bacteria | p__Proteobacteria | c__Alphaproteobacteria | o__Sphingomonadales | f__Sphingomonadaceae | g__Sphingomonas |  |
| MJ032-1-barcode26-umi72908bins-ubs-3 | Bacteria | p__Bacteroidota | c__Bacteroidia | o__Chitinophagales | f__Saprospiraceae | g__uncultured | s__uncultured_soil |
| G000768065_1 | Bacteria | p__Proteobacteria | c__Gammaproteobacteria | o__Burkholderiales | f__Comamonadaceae |  |  |
| RS-GCF-012584445.1-NZ-JABAHI010000010.1 | Bacteria | p__Actinobacteriota | c__Coriobacteriia | o__Coriobacteriales | f__Atopobiaceae | g__Coriobacteriaceae_UCG-002 | s__uncultured_bacterium |
| GU944672 | Bacteria | p__Proteobacteria | c__Gammaproteobacteria | o__Alteromonadales | f__Shewanellaceae | g__Shewanella |  |
| 7b4ccdb9890148309985302e0ab46466 | Bacteria | p__Bacteroidota | c__Bacteroidia | o__Bacteroidales | f__Bacteroidetes_vadinHA17 | g__Bacteroidetes_vadinHA17 | s__uncultured_bacterium |
| 59d5c0bbab364f87a1b32160b9a14044 | Bacteria | p__Planctomycetota | c__Planctomycetes | o__Gemmatales | f__Gemmataceae | g__uncultured | s__uncultured_thermal |
| 46f09bfb096377cde2a101f54cba00a7 | Bacteria | p__Chloroflexi | c__KD4-96 | o__KD4-96 | f__KD4-96 | g__KD4-96 |  |
| e32d9e893ed7983aaaea3abcd4a425b8 | Bacteria | p__Firmicutes | c__Bacilli | o__Bacillales | f__Bacillaceae | g__Geobacillus |  |
| 9fa4d85f94c9ae191d493419046bb551 | Bacteria | p__Proteobacteria | c__Gammaproteobacteria | o__Pseudomonadales | f__Moraxellaceae | g__Acinetobacter |  |
| 14522330 | Bacteria | p__Gemmatimonadota | c__Gemmatimonadetes | o__Gemmatimonadales | f__Gemmatimonadaceae | g__uncultured |  |
| 04996557 | Bacteria | p__Proteobacteria | c__Gammaproteobacteria | o__Xanthomonadales | f__Xanthomonadaceae | g__Stenotrophomonas |  |
| 9c83b0e8b4b023a5ef4438e1c8ce87e1 | Bacteria | p__Proteobacteria | c__Gammaproteobacteria | o__Burkholderiales | f__Nitrosomonadaceae | g__mle1-7 |  |
| RS-GCF-902497365.1-NZ-LR699993.1 | Bacteria | p__Firmicutes | c__Clostridia | o__Oscillospirales | f__Ruminococcaceae | g__Faecalibacterium |  |
| f43f274616f4bbc48605ae1e4f89a0e8 | Bacteria | p__Firmicutes | c__Clostridia | o__Lachnospirales | f__Lachnospiraceae |  |  |
| 11843491 | Bacteria | p__Proteobacteria | c__Alphaproteobacteria | o__Sphingomonadales | f__Sphingomonadaceae | g__Sphingomonas |  |
| 9d13c9efa60b7e2243e1e42b750f2853 | Bacteria | p__Verrucomicrobiota | c__Verrucomicrobiae | o__Verrucomicrobiales | f__Rubritaleaceae | g__Luteolibacter |  |
| 20daa4173782aeae7138e3792eb772cc | Bacteria | p__Firmicutes | c__Clostridia | o__Oscillospirales | f__Ruminococcaceae |  |  |
| 80a2dfd4c4c9c1dcc93d4f70431b3e90 | Bacteria | p__Bacteroidota | c__Bacteroidia | o__Chitinophagales | f__Chitinophagaceae |  |  |
| 34e46d52e69da4eee84b6fee41a76553 | Bacteria | p__Actinobacteriota | c__Actinobacteria | o__Catenulisporales | f__Actinospicaceae | g__Actinospica | s__bacterium_Ellin5012 |
| 04941748 | Bacteria | p__Gemmatimonadota | c__Gemmatimonadetes | o__Gemmatimonadales | f__Gemmatimonadaceae | g__uncultured |  |
| JX077130 | Bacteria | p__Bacteroidota | c__Bacteroidia | o__Flavobacteriales | f__Crocinitomicaceae | g__Fluviicola | s__Bacteroidetes_bacterium |
| GB-GCA-016742415.1-JAEPEL010000544.1 | Bacteria | p__Firmicutes | c__Clostridia | o__Clostridiales | f__Clostridiaceae | g__Clostridium_sensu_stricto_1 |  |
| 324287264106cdd38e73672a426da0ab | Bacteria | p__Actinobacteriota | c__Thermoleophilia | o__Gaiellales |  |  |  |
| RS-GCF-009827015.1-NZ-WUMG01000089.1 | Bacteria | p__Proteobacteria | c__Gammaproteobacteria | o__Burkholderiales | f__Comamonadaceae | g__Delftia |  |
| a2a16b4f247713d6e0edc1d7744be89e | Bacteria | p__Proteobacteria | c__Gammaproteobacteria | o__Pseudomonadales | f__Moraxellaceae | g__Acinetobacter |  |
| 2fda1181c508cfdd14618f9864a3b672 | Bacteria | p__Planctomycetota | c__Phycisphaerae | o__Tepidisphaerales | f__WD2101_soil_group | g__WD2101_soil_group | s__uncultured_bacterium |
| 72d03df937bb7c74e1edabb9bdec88cf | Bacteria | p__Actinobacteriota | c__Actinobacteria | o__Micrococcales |  |  |  |
| 05032308 | Bacteria | p__Proteobacteria | c__Gammaproteobacteria | o__Burkholderiales | f__Oxalobacteraceae |  |  |
| e24df1e45aefa648c6ea3169ed89ed72 | Bacteria | p__Actinobacteriota | c__Actinobacteria | o__Micrococcales |  |  |  |
| MJ030-2-barcode65-umi27658bins-ubs-25 | Bacteria | p__Acidobacteriota | c__Acidobacteriae | o__Solibacterales | f__Solibacteraceae | g__Candidatus_Solibacter |  |
| KP017569 | Bacteria | p__Actinobacteriota | c__Actinobacteria | o__Frankiales | f__Frankiaceae | g__Jatrophihabitans |  |
| 3a8bc4c6099a29bf252c2335040d24bc | Bacteria | p__Proteobacteria | c__Gammaproteobacteria | o__Burkholderiales | f__Alcaligenaceae | g__Castellaniella |  |
| 15c9592058b8dada1062501122b5eead | Bacteria | p__Firmicutes | c__Bacilli | o__Erysipelotrichales | f__Erysipelotrichaceae | g__Turicibacter |  |
| 3d7b32694c3831a0755ff44d7f4f20c3 | Bacteria | p__Proteobacteria | c__Gammaproteobacteria | o__Burkholderiales | f__Comamonadaceae |  |  |
| f6fc4a3f0130f3798f19d4c14148d073 | Bacteria | p__Firmicutes | c__Bacilli | o__Lactobacillales | f__Enterococcaceae | g__Enterococcus |  |
| b081c9716c2ba6ede2d0466d252b2e8b | Bacteria | p__Actinobacteriota | c__Actinobacteria | o__Micrococcales | f__Microbacteriaceae |  |  |
| 45c2fb19ec322e2488106dc5fb853382 | Bacteria | p__Firmicutes | c__Bacilli | o__Paenibacillales | f__Paenibacillaceae | g__Paenibacillus | s__Paenibacillus_validus |
| 09a78a38d7ef7e8973a33094c7fd07b7 | Bacteria | p__Firmicutes | c__Clostridia | o__Oscillospirales | f__Ruminococcaceae | g__uncultured | s__uncultured_Clostridium |
| 89d2b3de676aeaebc46d259345dc6e9d | Bacteria | p__Proteobacteria | c__Gammaproteobacteria | o__Burkholderiales | f__Comamonadaceae |  |  |
| eb591c93a70c303b183f147e66ea7b3e | Bacteria | p__Proteobacteria | c__Alphaproteobacteria | o__Caulobacterales | f__Caulobacteraceae | g__uncultured |  |
| f94e8bff963b40527cced2d4fb96775a | Bacteria | p__Actinobacteriota | c__Acidimicrobiia | o__Microtrichales | f__Iamiaceae | g__Iamia |  |
| 02247834 | Bacteria | p__Proteobacteria | c__Alphaproteobacteria | o__Rhizobiales | f__Hyphomicrobiaceae | g__Hyphomicrobium |  |
| 0f38390439f87adf9890430893048a11 | Bacteria | p__Firmicutes | c__Clostridia | o__Lachnospirales | f__Lachnospiraceae |  |  |
| 49e468d01f4098fc08b6e0b9999c6b4a | Bacteria | p__Bacteroidota | c__Bacteroidia | o__Cytophagales | f__Hymenobacteraceae | g__Hymenobacter |  |
| GB-GCA-004365535.1-SOEQ01000052.1 | Bacteria | p__Firmicutes | c__Bacilli | o__Bacillales | f__Planococcaceae | g__Lysinibacillus |  |
| 05036764 | Bacteria | p__Firmicutes | c__Bacilli | o__Exiguobacterales | f__Exiguobacteraceae | g__Exiguobacterium |  |
| ad2b1484c52d635c096309f320843da5 | Bacteria | p__Actinobacteriota | c__MB-A2-108 | o__MB-A2-108 | f__MB-A2-108 | g__MB-A2-108 |  |
| db60bba5fdd8dc4368275928278d7ad3 | Bacteria | p__Proteobacteria | c__Gammaproteobacteria | o__Cellvibrionales | f__Spongiibacteraceae | g__BD1-7_clade |  |
| 2e345106031e6319ebc5519766c663ac | Bacteria | p__Firmicutes | c__Bacilli | o__Erysipelotrichales | f__Erysipelatoclostridiaceae | g__Erysipelatoclostridium | s__uncultured_Clostridium |
| 659de9a2cad8187319242b2e285c68ea | Bacteria | p__Proteobacteria | c__Gammaproteobacteria | o__Burkholderiales | f__Comamonadaceae |  |  |
| 13ad256ca6b9a165890b39f8ba4f95c2 | Bacteria |  |  |  |  |  |  |
| 16920174 | Bacteria | p__Firmicutes | c__Clostridia | o__Lachnospirales | f__Lachnospiraceae | g__GCA-900066575 | s__uncultured_bacterium |
| 5976753c0ecaf761f2c351f54d49938d | Bacteria | p__Chloroflexi | c__AD3 | o__AD3 | f__AD3 | g__AD3 |  |
| f49e1b0695b3bcf768849bce1c1ee2c5 | Bacteria | p__Proteobacteria | c__Alphaproteobacteria | o__Rhodobacterales | f__Rhodobacteraceae |  |  |
| GB-GCA-014763825.1-JACXUR010000157.1 | Bacteria | p__Firmicutes | c__Bacilli | o__Bacillales | f__Planococcaceae | g__Planococcus |  |
| 3b9506dc012d706738bf4399df5fc951 | Bacteria | p__Chloroflexi | c__Ktedonobacteria | o__Ktedonobacterales | f__Ktedonobacteraceae |  |  |
| e3fc699c45a875c735658869500b5990 | Bacteria | p__Actinobacteriota | c__Actinobacteria | o__Propionibacteriales | f__Nocardioidaceae | g__Nocardioides |  |
| G000160775_1 | Bacteria | p__Actinobacteriota | c__Actinobacteria | o__Propionibacteriales | f__Nocardioidaceae | g__Aeromicrobium |  |
| 8fdb0722fd4e766fe3123f4e8c01dccd | Bacteria | p__Actinobacteriota | c__Acidimicrobiia | o__IMCC26256 | f__IMCC26256 | g__IMCC26256 |  |
| RS-GCF-008017545.1-NZ-VRTZ01000001.1 | Bacteria | p__Actinobacteriota | c__Actinobacteria | o__Micrococcales | f__Microbacteriaceae |  |  |
| 471ab2b2d9cf3047002d1224df3110de | Bacteria | p__Firmicutes | c__Bacilli | o__Paenibacillales | f__Paenibacillaceae | g__Paenibacillus |  |
| d76937096cb000eb8db64f140b6ba507 | Bacteria | p__Acidobacteriota | c__Acidobacteriae | o__Acidobacteriales |  |  |  |
| RS-GCF-015547935.1-NZ-JADMWC010000052.1 | Bacteria | p__Bacteroidota | c__Bacteroidia | o__Bacteroidales | f__Tannerellaceae | g__Parabacteroides |  |
| 15765044 | Bacteria | p__Actinobacteriota | c__Acidimicrobiia | o__IMCC26256 | f__IMCC26256 | g__IMCC26256 | s__actinobacterium_BGR |
| 5e181cae4db0c7e3cff7c12e64d51be8 | Bacteria | p__Firmicutes | c__Bacilli | o__Acholeplasmatales | f__Acholeplasmataceae | g__Anaeroplasma | s__uncultured_bacterium |
| AM747631 | Bacteria | p__Proteobacteria | c__Gammaproteobacteria | o__Burkholderiales | f__Burkholderiaceae | g__Burkholderia-Caballeronia-Paraburkholderia |  |
| abfa7f518340e5330be6a8b019d09b99 | Bacteria | p__Proteobacteria | c__Alphaproteobacteria | o__Rhizobiales | f__Devosiaceae |  |  |
| a89db122ea20939de8584f4e18d0a358 | Bacteria | p__Proteobacteria | c__Alphaproteobacteria | o__Rhizobiales | f__Beijerinckiaceae | g__Microvirga |  |
| 652f3096518866fdbf466d9f95445542 | Bacteria | p__Bacteroidota | c__Bacteroidia | o__Bacteroidales | f__Bacteroidaceae | g__Bacteroides | s__Bacteroides_coprocola |
| 3823e106a3070480571cc96589837f65 | Bacteria | p__Bacteroidota | c__Bacteroidia | o__Chitinophagales | f__Chitinophagaceae |  |  |
| RS-GCF-007665635.1-NZ-VDNQ01000010.1 | Bacteria | p__Proteobacteria | c__Gammaproteobacteria | o__Pseudomonadales | f__Pseudomonadaceae | g__Pseudomonas |  |
| 2775db9d482a18d54d72f2991e659a80 | Bacteria | p__Proteobacteria | c__Alphaproteobacteria | o__Sphingomonadales | f__Sphingomonadaceae | g__Sphingomonas |  |
| 04994967 | Bacteria | p__Proteobacteria | c__Alphaproteobacteria | o__Rhizobiales | f__Rhizobiaceae | g__Allorhizobium-Neorhizobium-Pararhizobium-Rhizobium |  |
| ed07a5045d0a30f445781ceb2b16fade | Bacteria | p__Actinobacteriota | c__Acidimicrobiia |  |  |  |  |
| fa124c5527ff7025135cb4b334a763b0 | Bacteria | p__Proteobacteria | c__Alphaproteobacteria | o__Rhizobiales | f__Methyloligellaceae | g__uncultured |  |
| AB012594 | Bacteria | p__Actinobacteriota | c__Actinobacteria | o__Micrococcales | f__Microbacteriaceae | g__Leucobacter |  |
| 17445657 | Bacteria | p__Chloroflexi | c__TK10 | o__TK10 | f__TK10 | g__TK10 |  |
| 083dc065efce99420084579f6ec27531 | Bacteria | p__Bdellovibrionota | c__Bdellovibrionia | o__Bacteriovoracales | f__Bacteriovoracaceae | g__Peredibacter |  |
| 5e286260555f51f2ce1d1bebaa810ee3 | Bacteria | p__Actinobacteriota | c__Actinobacteria | o__Pseudonocardiales | f__Pseudonocardiaceae | g__Pseudonocardia |  |
| MJ006-1-barcode40-umi116237bins-ubs-5 | Bacteria | p__Firmicutes | c__Clostridia | o__Lachnospirales | f__Lachnospiraceae |  |  |
| 7f08ba8337225f1c8adac4806aed6efd | Bacteria | p__Firmicutes | c__Bacilli | o__Lactobacillales | f__Streptococcaceae | g__Lactococcus |  |
| e697827cf4e6d20dfb94205282bfbd3b | Bacteria | p__Gemmatimonadota |  |  |  |  |  |
| 11072500 | Bacteria | p__Bacteroidota | c__Bacteroidia | o__Bacteroidales | f__Muribaculaceae | g__Muribaculaceae | s__uncultured_organism |
| RS-GCF-014873475.1-NZ-JADBDX010000001.1 | Bacteria | p__Proteobacteria | c__Alphaproteobacteria | o__Sphingomonadales | f__Sphingomonadaceae | g__Sphingomonas |  |
| MJ032-2-barcode70-umi80909bins-ubs-4 | Bacteria | p__Acidobacteriota | c__Blastocatellia | o__Blastocatellales | f__Blastocatellaceae | g__JGI_0001001-H03 |  |
| 8c2698cf147142a0a9c9fd15d3271d7a | Bacteria | p__Firmicutes | c__Bacilli | o__Staphylococcales | f__Staphylococcaceae | g__Staphylococcus | s__Staphylococcus_succinus |
| 02360053 | Bacteria | p__Proteobacteria | c__Alphaproteobacteria | o__Sphingomonadales | f__Sphingomonadaceae | g__Sphingomonas |  |
| RS-GCF-006716455.1-NZ-VFPC01000007.1 | Bacteria | p__Proteobacteria | c__Gammaproteobacteria | o__Xanthomonadales | f__Xanthomonadaceae | g__Stenotrophomonas |  |
| MJ031-1-barcode42-umi39085bins-ubs-3 | Bacteria | p__Bacteroidota | c__Bacteroidia | o__Chitinophagales | f__Chitinophagaceae | g__Flavisolibacter |  |
| 6e0e45b5e95aa6db0287689d9d0359b3 | Bacteria | p__Proteobacteria | c__Gammaproteobacteria | o__Enterobacterales | f__Enterobacteriaceae |  |  |
| 2e7ef323958e25215bbbe64da4415417 | Bacteria | p__Acidobacteriota | c__Acidobacteriae | o__Solibacterales | f__Solibacteraceae | g__Candidatus_Solibacter |  |
| e51a89932d866b7ce72482b22dd537a9 | Bacteria | p__Proteobacteria | c__Gammaproteobacteria | o__Burkholderiales | f__Comamonadaceae |  |  |
| 16700a89a5e4b15399f5e932e90ae2c4 | Bacteria | p__Firmicutes | c__Clostridia | o__Clostridia | f__Hungateiclostridiaceae | g__Fastidiosipila |  |
| MJ006-2-barcode54-umi268101bins-ubs-3 | Bacteria | p__Bacteroidota | c__Bacteroidia | o__Bacteroidales | f__Tannerellaceae | g__Parabacteroides |  |
| 1f143582fafba0745ed0eb82efcce619 | Bacteria | p__Proteobacteria | c__Alphaproteobacteria | o__Rhodobacterales | f__Rhodobacteraceae |  |  |
| 5089f7deabc6a39d02bbc860c6766d4d | Bacteria | p__Actinobacteriota | c__Actinobacteria | o__Corynebacteriales | f__Corynebacteriaceae | g__Corynebacterium | s__Corynebacterium_matruchotii |
| 93d25b62cd45ed2ed66adb5f2739aaea | Bacteria | p__Proteobacteria | c__Gammaproteobacteria | o__Burkholderiales | f__Alcaligenaceae |  |  |
| d680dc9ed2679cf89350c185fd9cfa8e | Bacteria | p__Actinobacteriota | c__Actinobacteria | o__Propionibacteriales | f__Nocardioidaceae | g__Nocardioides |  |
| b0775bc957bdc34647b151bfccc03b8d | Bacteria | p__Actinobacteriota | c__Thermoleophilia | o__Gaiellales | f__Gaiellaceae | g__Gaiella | s__metagenome |
| 343f7445759a01fed730806f99c95b97 | Bacteria | p__Gemmatimonadota | c__Gemmatimonadetes | o__Gemmatimonadales | f__Gemmatimonadaceae | g__uncultured |  |
| 8b268f20a0745af19559ddb976f5c36d | Bacteria | p__Firmicutes | c__Bacilli | o__Paenibacillales | f__Paenibacillaceae | g__Paenibacillus |  |
| f866410011e8a2ab4f691bb55f6d91d7 | Bacteria | p__Proteobacteria | c__Alphaproteobacteria | o__Rhizobiales | f__Xanthobacteraceae |  |  |
| 019010ad1558582108553e29ca0f82dd | Bacteria | p__Acidobacteriota | c__Vicinamibacteria | o__Subgroup_17 | f__Subgroup_17 | g__Subgroup_17 |  |
| 4da358341125df629dd35217e81822c2 | Bacteria | p__Planctomycetota | c__Planctomycetes | o__Gemmatales | f__Gemmataceae | g__Gemmata |  |
| aa349e4c013f3e42377aa3ea05ae0602 | Bacteria | p__Acidobacteriota | c__Acidobacteriae | o__Acidobacteriales |  |  |  |
| 03cbf3d88f529519e03457c74c6ed397 | Bacteria | p__Acidobacteriota | c__Vicinamibacteria | o__Vicinamibacterales | f__Vicinamibacteraceae | g__Vicinamibacteraceae |  |
| EF204258 | Bacteria | p__Proteobacteria | c__Gammaproteobacteria | o__Pseudomonadales | f__Moraxellaceae | g__Acinetobacter |  |
| AB033328 | Bacteria | p__Proteobacteria | c__Alphaproteobacteria | o__Sphingomonadales | f__Sphingomonadaceae | g__Porphyrobacter |  |
| 05033951 | Bacteria | p__Actinobacteriota | c__Actinobacteria | o__Propionibacteriales | f__Nocardioidaceae | g__Nocardioides |  |
| AY423719 | Bacteria | p__Actinobacteriota | c__Actinobacteria | o__Propionibacteriales | f__Nocardioidaceae | g__Nocardioides |  |
| MJ007-1-barcode36-umi22195bins-ubs-8 | Bacteria | p__Proteobacteria | c__Gammaproteobacteria | o__Burkholderiales | f__Comamonadaceae |  |  |
| MJ034-1-barcode26-umi29091bins-ubs-3 | Bacteria | p__Proteobacteria | c__Gammaproteobacteria | o__Burkholderiales | f__A21b | g__A21b |  |
| 6d7337f61ad0905d92c5117daa262d98 | Bacteria | p__Synergistota | c__Synergistia | o__Synergistales | f__Synergistaceae | g__Syner-01 | s__uncultured_bacterium |
| RS-GCF-016504725.1-NZ-JADVIC010000029.1 | Bacteria | p__Proteobacteria | c__Gammaproteobacteria | o__Enterobacterales | f__Enterobacteriaceae |  |  |
| MJ020-1-barcode31-umi74742bins-ubs-5 | Bacteria | p__Proteobacteria | c__Gammaproteobacteria | o__Burkholderiales | f__Comamonadaceae |  |  |
| 2101714b9e6ca3edaf2d504d5a3b5008 | Bacteria | p__Firmicutes | c__Clostridia | o__Peptostreptococcales-Tissierellales | f__Peptostreptococcales-Tissierellales | g__Anaerococcus |  |
| 08339903 | Bacteria | p__Proteobacteria | c__Alphaproteobacteria | o__Caulobacterales | f__Caulobacteraceae | g__Brevundimonas |  |
| 109b894bfa2343eb66f71dacb4f5c5f1 | Bacteria | p__Actinobacteriota | c__Acidimicrobiia | o__IMCC26256 | f__IMCC26256 | g__IMCC26256 |  |
| d398cb3f6b808c51c64b12e037fcbaae | Bacteria | p__Bacteroidota | c__Bacteroidia | o__Flavobacteriales | f__Flavobacteriaceae | g__Flavobacterium | s__Flavobacterium_ummariense |
| a479c48a9245740da5b6790d76c0adfd | Bacteria | p__Bacteroidota | c__Bacteroidia | o__Sphingobacteriales | f__Sphingobacteriaceae | g__Pedobacter |  |
| 04318038 | Bacteria | p__WPS-2 | c__WPS-2 | o__WPS-2 | f__WPS-2 | g__WPS-2 | s__uncultured_forest |
| 9705a6d78496f7b6a201a66eb1062f7e | Bacteria | p__Firmicutes | c__Bacilli | o__Lactobacillales | f__Enterococcaceae | g__Enterococcus |  |
| MJ030-2-barcode65-umi104332bins-ubs-3 | Bacteria | p__Acidobacteriota | c__Acidobacteriae | o__Acidobacteriales | f__uncultured | g__uncultured |  |
| DQ673672 | Bacteria | p__Bacteroidota | c__Bacteroidia | o__Flavobacteriales | f__Weeksellaceae | g__Chryseobacterium |  |
| 15997970 | Bacteria | p__Actinobacteriota | c__Actinobacteria | o__Micrococcales | f__Micrococcaceae | g__Sinomonas |  |
| 8150242df41a4071c5dc2e5524d91f33 | Bacteria | p__Acidobacteriota | c__Vicinamibacteria | o__Subgroup_17 | f__Subgroup_17 | g__Subgroup_17 |  |
| 0035dec9deb57749a10792e097a5c234 | Bacteria | p__Firmicutes | c__Clostridia | o__Lachnospirales | f__Lachnospiraceae |  |  |
| 730686f78591aa248c3df992347e1b3b | Bacteria | p__Acidobacteriota | c__Acidobacteriae | o__Bryobacterales | f__Bryobacteraceae | g__Bryobacter |  |
| EF469609 | Bacteria | p__Proteobacteria | c__Gammaproteobacteria | o__Burkholderiales | f__Comamonadaceae | g__Pseudacidovorax |  |
| 17388531 | Bacteria | p__Chloroflexi | c__Ktedonobacteria | o__Ktedonobacterales | f__Ktedonobacteraceae | g__FCPS473 | s__uncultured_bacterium |
| 214e21e5d969cee79aee3ad9c30df08d | Bacteria | p__Bacteroidota | c__Bacteroidia | o__Chitinophagales | f__Chitinophagaceae | g__Taibaiella |  |
| G001899805_1 | Bacteria | p__Proteobacteria | c__Gammaproteobacteria | o__Xanthomonadales | f__Rhodanobacteraceae | g__Chujaibacter |  |
| RS-GCF-000477975.1-NZ-KI515719.1--4 | Bacteria | p__Actinobacteriota | c__Actinobacteria | o__Corynebacteriales | f__Corynebacteriaceae | g__Corynebacterium |  |
| 13283504 | Bacteria | p__Gemmatimonadota | c__Longimicrobia | o__Longimicrobiales | f__Longimicrobiaceae | g__Longimicrobiaceae | s__uncultured_bacterium |
| 1c4c37ceba4c9dc73be10319fca124d6 | Bacteria | p__Proteobacteria | c__Alphaproteobacteria | o__Rhizobiales | f__Rhizobiaceae | g__Mesorhizobium |  |
| KY797643 | Bacteria | p__Proteobacteria | c__Gammaproteobacteria | o__Enterobacterales | f__Enterobacteriaceae |  |  |
| a9b550dffaf2548292535700502d1888 | Bacteria | p__Actinobacteriota | c__Actinobacteria | o__Micrococcales | f__Microbacteriaceae |  |  |
| JX260424 | Bacteria | p__Proteobacteria | c__Alphaproteobacteria | o__Reyranellales | f__Reyranellaceae | g__Reyranella |  |
| 21e35117adfbe68e05e4fa6311f70291 | Bacteria | p__Proteobacteria | c__Gammaproteobacteria | o__Pseudomonadales | f__Pseudomonadaceae | g__Pseudomonas |  |
| 68b180b4bce1215ab342987772164a95 | Bacteria | p__Bacteroidota | c__Bacteroidia | o__Sphingobacteriales | f__Sphingobacteriaceae | g__Pseudopedobacter | s__Pseudopedobacter_saltans |
| 39bd8a2eab73242d79fd7c85031ff03d | Bacteria | p__Proteobacteria | c__Gammaproteobacteria | o__Steroidobacterales | f__Steroidobacteraceae | g__uncultured |  |
| e51c1cefa1651f4e6ef0895307b45ddb | Bacteria | p__Proteobacteria | c__Gammaproteobacteria | o__Enterobacterales | f__Enterobacteriaceae |  |  |
| 5d76164214577a29ec2e7b1e298f7cfd | Bacteria | p__Firmicutes | c__Clostridia | o__Lachnospirales | f__Lachnospiraceae | g__Butyrivibrio | s__uncultured_organism |
| 70f22630dc88a54d9ffd730d94f40bd8 | Bacteria | p__Acidobacteriota | c__Acidobacteriae | o__Subgroup_2 | f__Subgroup_2 | g__Subgroup_2 |  |
| 17b876b6f23a270a15f015df88bb1c43 | Bacteria | p__Actinobacteriota | c__Actinobacteria | o__Corynebacteriales | f__Corynebacteriaceae | g__Corynebacterium |  |
| 1493ddaa55a2c8388bd54d5c73d94b03 | Bacteria | p__Actinobacteriota | c__Acidimicrobiia | o__Microtrichales | f__Ilumatobacteraceae |  |  |
| 705462b4176ce051d4c562ef07be85e0 | Bacteria | p__NB1-j | c__NB1-j | o__NB1-j | f__NB1-j | g__NB1-j |  |
| 7c20518a5cc85cdc408135f628b75107 | Bacteria | p__Methylomirabilota | c__Methylomirabilia | o__Rokubacteriales | f__Rokubacteriales | g__Rokubacteriales | s__uncultured_Candidatus |
| MJ006-1-barcode26-umi204238bins-ubs-3 | Bacteria | p__Firmicutes | c__Clostridia | o__Clostridia_UCG-014 | f__Clostridia_UCG-014 | g__Clostridia_UCG-014 |  |
| 04960821 | Bacteria | p__Actinobacteriota | c__Actinobacteria | o__Corynebacteriales | f__Corynebacteriaceae | g__Corynebacteriaceae | s__uncultured_Corynebacterium |
| GB-GCA-009911555.1-QWKE01000141.1 | Bacteria | p__Bacteroidota | c__Bacteroidia | o__Bacteroidales | f__Muribaculaceae | g__Muribaculaceae |  |
| JQ346805 | Bacteria | p__Proteobacteria | c__Alphaproteobacteria | o__Rhizobiales | f__Rhizobiaceae | g__Aureimonas |  |
| 9bd37c0fcc3fca9e12bb5b1ea3214d48 | Bacteria | p__Actinobacteriota | c__Actinobacteria | o__Propionibacteriales | f__Nocardioidaceae | g__Nocardioides |  |
| 48aafaa975d7fe112025161ec9eb2ce4 | Bacteria | p__Proteobacteria | c__Gammaproteobacteria | o__Steroidobacterales | f__Steroidobacteraceae | g__uncultured |  |
| f3d17fb05471717a20d8316c3433bf12 | Bacteria | p__Proteobacteria | c__Alphaproteobacteria | o__Rhizobiales | f__uncultured | g__uncultured | s__uncultured_Hyphomicrobiaceae |
| MJ006-2-barcode69-umi27046bins-ubs-11 | Bacteria | p__Proteobacteria | c__Gammaproteobacteria | o__Enterobacterales | f__Enterobacteriaceae |  |  |
| MJ009-1-barcode40-umi73217bins-ubs-4 | Bacteria | p__Proteobacteria | c__Gammaproteobacteria | o__Burkholderiales | f__Sutterellaceae | g__Sutterella | s__uncultured_bacterium |
| RS-GCF-018784285.1-NZ-WQOB01000007.1 | Bacteria | p__Bacteroidota | c__Bacteroidia | o__Bacteroidales | f__Marinifilaceae | g__Odoribacter |  |
| 4f2caf097832ce68f3d8b987f4377515 | Bacteria | p__Chloroflexi | c__Anaerolineae | o__Caldilineales | f__Caldilineaceae | g__uncultured | s__Chloroflexi_bacterium |
| 532b60ea8fef2a036225a93e39f44561 | Bacteria | p__Firmicutes | c__Clostridia | o__Clostridiales | f__Clostridiaceae | g__Proteiniclasticum |  |
| RS-GCF-000559065.1-NZ-AZRT01000118.1 | Bacteria | p__Actinobacteriota | c__Actinobacteria | o__Micrococcales | f__Brevibacteriaceae | g__Brevibacterium |  |
| d2eb7b3e52eb47f2afbf54d6dca3751b | Bacteria | p__Proteobacteria | c__Gammaproteobacteria | o__Burkholderiales | f__Comamonadaceae | g__Piscinibacter |  |
| MJ009-1-barcode47-umi13244bins-ubs-34 | Bacteria | p__Proteobacteria | c__Gammaproteobacteria | o__Pseudomonadales | f__Pseudomonadaceae | g__Pseudomonas |  |
| 50290910d9dfd3e55eb5b44c37010398 | Bacteria | p__Proteobacteria | c__Gammaproteobacteria | o__Xanthomonadales | f__Xanthomonadaceae | g__Silanimonas | s__uncultured_bacterium |
| MJ030-2-barcode67-umi74220bins-ubs-7 | Bacteria | p__Firmicutes | c__Bacilli | o__Lactobacillales | f__Enterococcaceae | g__Enterococcus |  |
| 02429869 | Bacteria | p__Proteobacteria | c__Alphaproteobacteria | o__Sphingomonadales | f__Sphingomonadaceae |  |  |
| e6b847624a0e665df80b47c3489f000d | Bacteria | p__Bacteroidota | c__Bacteroidia | o__Bacteroidales | f__Muribaculaceae | g__Muribaculaceae |  |
| 1df4cbb2ea8fa80f655d6983e7e02ad6 | Bacteria | p__Actinobacteriota | c__Actinobacteria | o__Pseudonocardiales | f__Pseudonocardiaceae | g__Saccharopolyspora |  |
| 2a7dc532a2068ee08abd0da360add163 | Bacteria | p__Actinobacteriota | c__Actinobacteria | o__Propionibacteriales | f__Propionibacteriaceae | g__Cutibacterium |  |
| f6d6394895b0dff15ba8d57bd51666f5 | Bacteria | p__Firmicutes | c__Clostridia | o__Oscillospirales | f__Ruminococcaceae | g__Ruminococcus | s__unidentified |
| G001063195_1 | Bacteria | p__Actinobacteriota | c__Actinobacteria | o__Micrococcales | f__Micrococcaceae | g__Kocuria |  |
| c72b4714d3fa562d8df076791f694e5f | Bacteria | p__Actinobacteriota | c__Rubrobacteria | o__Rubrobacterales | f__Rubrobacteriaceae | g__Rubrobacter | s__uncultured_Actinomycetales |
| ea18d854248ddeb364fab2c3b50733ae | Bacteria | p__Verrucomicrobiota | c__Chlamydiae | o__Chlamydiales | f__Parachlamydiaceae |  |  |
| 065697d8fcf140acb966fa53f9890aeb | Bacteria | p__Actinobacteriota | c__Thermoleophilia | o__Solirubrobacterales | f__67-14 | g__67-14 | s__metagenome |
| RS-GCF-004745805.1-NZ-SRLQ01000037.1 | Bacteria | p__Actinobacteriota | c__Actinobacteria | o__Corynebacteriales | f__Mycobacteriaceae | g__Mycobacterium |  |
| 08177291 | Bacteria | p__Proteobacteria | c__Alphaproteobacteria | o__Rhizobiales | f__Xanthobacteraceae | g__Rhodoplanes |  |
| 52c81d5985295bb60ed1f6fbddf62174 | Bacteria | p__Acidobacteriota | c__Acidobacteriae | o__Subgroup_2 | f__Subgroup_2 | g__Subgroup_2 |  |
| 00673199 | Bacteria | p__Proteobacteria | c__Alphaproteobacteria | o__Rhizobiales | f__Rhizobiaceae | g__Phyllobacterium |  |
| 04892027 | Bacteria | p__Actinobacteriota | c__Actinobacteria | o__Frankiales | f__Acidothermaceae | g__Acidothermus |  |
| 820740bbaa70a4c6ad40d757e08bda6a | Bacteria |  |  |  |  |  |  |
| 8468ec3af49cad9f5bad7ae1ef392b2a | Bacteria | p__Firmicutes | c__Clostridia | o__Lachnospirales | f__Lachnospiraceae | g__[Eubacterium]_xylanophilum_group |  |
| 82f781dbe75f22435b4299c512be7b5e | Bacteria | p__Actinobacteriota | c__Acidimicrobiia | o__IMCC26256 | f__IMCC26256 | g__IMCC26256 |  |
| 04956735 | Bacteria | p__Actinobacteriota | c__Actinobacteria | o__Propionibacteriales | f__Nocardioidaceae | g__Nocardioides |  |
| d08700aefdd76d215cd2c078eb3b1c71 | Bacteria | p__Proteobacteria | c__Alphaproteobacteria | o__Caulobacterales | f__Hyphomonadaceae | g__SWB02 | s__uncultured_Alphaproteobacteria |
| 17361647 | Bacteria | p__Firmicutes | c__Bacilli | o__Paenibacillales | f__Paenibacillaceae | g__Saccharibacillus |  |
| MT379648 | Bacteria | p__Proteobacteria | c__Alphaproteobacteria | o__Acetobacterales | f__Acetobacteraceae | g__Gluconacetobacter |  |
| RS-GCF-012843865.1-NZ-JABAFZ010000016.1 | Bacteria | p__Actinobacteriota | c__Actinobacteria | o__Corynebacteriales | f__Corynebacteriaceae | g__Corynebacterium |  |
| DQ768107 | Bacteria | p__Myxococcota | c__Myxococcia | o__Myxococcales | f__Myxococcaceae | g__Cystobacter |  |
| G000969445_1 | Bacteria | p__Proteobacteria | c__Alphaproteobacteria | o__Rhizobiales | f__Devosiaceae | g__Devosia |  |
| 53aa9acf0f384a3506f85791a4c05134 | Bacteria | p__Nitrospirota | c__Nitrospiria | o__Nitrospirales | f__Nitrospiraceae | g__Nitrospira |  |
| 04919184 | Bacteria | p__Bacteroidota | c__Bacteroidia | o__Sphingobacteriales | f__Sphingobacteriaceae | g__Mucilaginibacter |  |
| AB023236 | Bacteria | p__Firmicutes | c__Bacilli | o__Lactobacillales | f__Leuconostocaceae | g__Weissella | s__Weissella_viridescens |
| 5d3a9f75faf6354aad8a1b4b6ee632d1 | Bacteria | p__Actinobacteriota | c__Acidimicrobiia | o__Microtrichales | f__Microtrichaceae | g__uncultured |  |
| b422c1e2c7205c81399e7b3a38d8bd49 | Bacteria | p__Myxococcota | c__Polyangia | o__Haliangiales | f__Haliangiaceae | g__Haliangium |  |
| KF360052 | Bacteria | p__Proteobacteria | c__Alphaproteobacteria | o__Caulobacterales | f__Caulobacteraceae | g__Caulobacter |  |
| a45695e40bbdb68a8770b8098dbbafe7 | Bacteria | p__Actinobacteriota | c__Actinobacteria | o__Corynebacteriales | f__Corynebacteriaceae | g__Corynebacterium |  |
| 8d8b81669ffa61d6b47e54c7905e4fa6 | Bacteria | p__Actinobacteriota | c__Actinobacteria | o__Micrococcales | f__Micrococcaceae | g__Micrococcus |  |
| bb8d5b791f791b2b4626610a52e1742b | Bacteria | p__Firmicutes | c__Negativicutes | o__Veillonellales-Selenomonadales | f__Selenomonadaceae | g__Megamonas |  |
| 02269788 | Bacteria | p__Proteobacteria | c__Alphaproteobacteria | o__Rhodobacterales | f__Rhodobacteraceae |  |  |
| b21476cff6463dab80f548d71eff7629 | Bacteria | p__Actinobacteriota | c__Actinobacteria | o__Micrococcales | f__Microbacteriaceae |  |  |
| 1644ff4d9c7739a9983185158816c75e | Bacteria | p__Chloroflexi | c__Anaerolineae | o__SBR1031 | f__A4b | g__A4b |  |
| b60abb48f38a709722f224c9083d26d2 | Bacteria | p__Firmicutes | c__Clostridia | o__Lachnospirales | f__Lachnospiraceae |  |  |
| 04901263 | Bacteria | p__Proteobacteria | c__Gammaproteobacteria | o__Gammaproteobacteria_Incertae_Sedis | f__Unknown_Family | g__Acidibacter |  |
| AB126621 | Bacteria | p__Proteobacteria | c__Gammaproteobacteria | o__Pseudomonadales | f__Pseudomonadaceae | g__Pseudomonas |  |
| a7e118e4e0582d76296b0da5f55a242c | Bacteria | p__Actinobacteriota | c__Actinobacteria | o__Propionibacteriales | f__Propionibacteriaceae |  |  |
| 1d691cd738542b3d9ce04aa650613f85 | Bacteria | p__Acidobacteriota | c__Vicinamibacteria | o__Vicinamibacterales | f__Vicinamibacteraceae | g__Vicinamibacteraceae |  |
| MJ005-barcode42-umi85849bins-ubs-3 | Bacteria | p__Bacteroidota | c__Bacteroidia | o__Bacteroidales | f__Bacteroidaceae | g__Bacteroides |  |
| 04910373 | Bacteria | p__Proteobacteria | c__Gammaproteobacteria | o__Pseudomonadales | f__Pseudomonadaceae | g__Pseudomonas |  |
| 34fe6dc15bb6ddf54d549bfbef6c277b | Bacteria | p__Actinobacteriota | c__Actinobacteria | o__Corynebacteriales | f__Mycobacteriaceae | g__Mycobacterium |  |
| 8a3f97a97fd7adcf991bc9957c60dd59 | Bacteria | p__Firmicutes | c__Bacilli | o__Bacillales | f__Bacillaceae | g__Bacillus |  |
| d987e9475924670ed6ce19597d372448 | Bacteria | p__Actinobacteriota | c__Actinobacteria | o__Corynebacteriales | f__Nocardiaceae | g__Rhodococcus |  |
| RS-GCF-009494045.1-NZ-VZCT01000026.1 | Bacteria | p__Bacteroidota | c__Bacteroidia | o__Bacteroidales | f__Prevotellaceae | g__Prevotella |  |
| G001885655_1 | Bacteria | p__Cyanobacteria | c__Cyanobacteriia | o__Cyanobacteriales | f__Microcystaceae | g__Microcystis_PCC-7914 |  |
| 04878193 | Bacteria | p__Proteobacteria | c__Gammaproteobacteria | o__Burkholderiales | f__Oxalobacteraceae | g__Massilia |  |
| 074c37af688926966469e477d4d344c7 | Bacteria | p__Actinobacteriota | c__Actinobacteria | o__Bifidobacteriales | f__Bifidobacteriaceae | g__Bifidobacterium |  |
| RS-GCF-017829895.1-NZ-JAGJUD010000077.1 | Bacteria | p__Proteobacteria | c__Gammaproteobacteria | o__Enterobacterales |  |  |  |
| 04005661 | Bacteria | p__Gemmatimonadota | c__AKAU4049 | o__AKAU4049 | f__AKAU4049 | g__AKAU4049 | s__uncultured_soil |
| 9e2224a27c0edbe0c01b427da5ce9a74 | Bacteria | p__Bacteroidota | c__Bacteroidia | o__Bacteroidales | f__Muribaculaceae | g__Muribaculaceae |  |
| RS-GCF-900105535.1-NZ-LT629779.1--3 | Bacteria | p__Actinobacteriota | c__Actinobacteria | o__Micrococcales | f__Micrococcaceae | g__Pseudarthrobacter |  |
| f62b3b1d3bcd44438c497ce378b3fce5 | Bacteria | p__Bacteroidota | c__Bacteroidia | o__Chitinophagales | f__Chitinophagaceae | g__Flavisolibacter |  |
| ARTG01000014 | Bacteria | p__Proteobacteria | c__Alphaproteobacteria | o__Rhizobiales | f__Hyphomicrobiaceae | g__Hyphomicrobium |  |
| 04927529 | Bacteria | p__Proteobacteria | c__Alphaproteobacteria | o__uncultured | f__uncultured | g__uncultured | s__bacterium_enrichment |
| ffa80f6a3a62b0fd2113ee6cd2f6d263 | Bacteria | p__Firmicutes | c__Bacilli | o__Lactobacillales | f__Enterococcaceae | g__Enterococcus |  |
| 08204919 | Bacteria | p__Proteobacteria | c__Alphaproteobacteria | o__Rhizobiales | f__Beijerinckiaceae |  |  |
| 54b457f691ea01990d04ffaa2f74053b | Bacteria | p__Proteobacteria | c__Alphaproteobacteria | o__Azospirillales | f__uncultured | g__uncultured | s__uncultured_bacterium |
| cbd1679f42e8471b03ef2613b56e4576 | Bacteria | p__Proteobacteria | c__Alphaproteobacteria | o__Rhizobiales | f__Xanthobacteraceae |  |  |
| HE995762 | Bacteria | p__Bacteroidota | c__Bacteroidia | o__Flavobacteriales | f__Flavobacteriaceae | g__Flavobacterium |  |
| 04912676 | Bacteria | p__Acidobacteriota | c__Acidobacteriae | o__Acidobacteriales | f__Acidobacteriaceae_(Subgroup_1) | g__Bryocella | s__uncultured_bacterium |
| MJ032-2-barcode71-umi9390bins-ubs-48 | Bacteria | p__Bacteroidota | c__Bacteroidia | o__Chitinophagales | f__Chitinophagaceae |  |  |
| 746ef5cbbdc5ad0dec368d27bc274db5 | Bacteria | p__Firmicutes | c__Bacilli | o__Erysipelotrichales | f__Erysipelotrichaceae | g__Turicibacter |  |
| 14520983 | Bacteria | p__Gemmatimonadota | c__Gemmatimonadetes | o__Gemmatimonadales | f__Gemmatimonadaceae | g__uncultured |  |
| 287c06dfd2db6f7cd868a70ddab20392 | Bacteria | p__Chloroflexi | c__KD4-96 | o__KD4-96 | f__KD4-96 | g__KD4-96 | s__metagenome |
| 11628181 | Bacteria | p__Myxococcota | c__bacteriap25 | o__bacteriap25 | f__bacteriap25 | g__bacteriap25 | s__metagenome |
| 04996399 | Bacteria | p__Proteobacteria | c__Gammaproteobacteria | o__Xanthomonadales | f__Xanthomonadaceae | g__Stenotrophomonas |  |
| 04925213 | Bacteria | p__Proteobacteria | c__Alphaproteobacteria | o__Sphingomonadales | f__Sphingomonadaceae | g__Sphingomonas |  |
| 6fe072276127a47bb1008d4db279c624 | Bacteria | p__Proteobacteria | c__Gammaproteobacteria | o__Enterobacterales | f__Enterobacteriaceae |  |  |
| f07c7e9589f74a5af2df60edb08b5c5f | Bacteria | p__Actinobacteriota | c__Actinobacteria | o__Corynebacteriales | f__Mycobacteriaceae | g__Mycobacterium |  |
| d74701f7e015f18a764e40359e79e9b9 | Bacteria | p__Methylomirabilota | c__Methylomirabilia | o__Rokubacteriales | f__Rokubacteriales | g__Rokubacteriales | s__uncultured_bacterium |
| RS-GCF-019048865.1-NZ-CP077604.1 | Bacteria | p__Actinobacteriota | c__Acidimicrobiia | o__Microtrichales | f__Iamiaceae | g__Iamia |  |
| f2932da171f528859268f228da51e71b | Bacteria | p__Chloroflexi | c__Chloroflexia | o__Thermomicrobiales | f__AKYG1722 | g__AKYG1722 | s__uncultured_soil |
| 04918300 | Bacteria | p__Bacteroidota | c__Bacteroidia | o__Sphingobacteriales | f__Sphingobacteriaceae | g__Sphingobacterium | s__Sphingobacterium_spiritivorum |
| 8800c373a7204a5937c795edeaa538ea | Bacteria | p__Firmicutes | c__Bacilli | o__Lactobacillales | f__Streptococcaceae | g__Streptococcus |  |
| 53e6db7fd4a3f2898381e393a3b20f75 | Bacteria | p__Chloroflexi | c__AD3 | o__AD3 | f__AD3 | g__AD3 | s__uncultured_bacterium |
| 08438298 | Bacteria | p__Proteobacteria | c__Alphaproteobacteria | o__Rhizobiales | f__Beijerinckiaceae | g__Microvirga |  |
| 02250692 | Bacteria | p__Proteobacteria | c__Alphaproteobacteria | o__Caulobacterales | f__Caulobacteraceae | g__Brevundimonas |  |
| 81613026ce4f327ddcf09c2074664067 | Bacteria | p__NB1-j | c__NB1-j | o__NB1-j | f__NB1-j | g__NB1-j | s__uncultured_proteobacterium |
| 86579f98f02eacd48b29cffead5a5ab4 | Bacteria | p__Nitrospirota | c__Nitrospiria | o__Nitrospirales | f__Nitrospiraceae | g__Nitrospira |  |
| 05102328 | Bacteria | p__Bacteroidota | c__Bacteroidia | o__Chitinophagales | f__Chitinophagaceae |  |  |
| 18974129 | Bacteria | p__Proteobacteria | c__Gammaproteobacteria | o__Burkholderiales | f__Nitrosomonadaceae | g__Ellin6067 |  |
| 02247761 | Bacteria | p__Proteobacteria | c__Alphaproteobacteria | o__Sphingomonadales | f__Sphingomonadaceae |  |  |
| 02354944 | Bacteria | p__Firmicutes | c__Clostridia | o__Lachnospirales | f__Lachnospiraceae | g__Agathobacter |  |
| d8e5b7f69907bb9d748221978a814cbb | Bacteria | p__Actinobacteriota | c__Actinobacteria |  |  |  |  |
| 9950fd4fdbaaa686e70e5ee62f54d58c | Bacteria | p__Actinobacteriota | c__Acidimicrobiia | o__IMCC26256 | f__IMCC26256 | g__IMCC26256 |  |
| MJ006-2-barcode73-umi101434bins-ubs-5 | Bacteria | p__Firmicutes | c__Clostridia | o__Lachnospirales | f__Lachnospiraceae | g__Agathobacter |  |
| 6a1c5c1187a804d414bbac8f7842a858 | Bacteria | p__Campilobacterota | c__Campylobacteria | o__Campylobacterales | f__Campylobacteraceae | g__Campylobacter | s__Campylobacter_avium |
| MJ034-1-barcode33-umi3001bins-ubs-150 | Bacteria | p__Proteobacteria | c__Gammaproteobacteria | o__Cellvibrionales | f__Cellvibrionaceae | g__Cellvibrio |  |
| 16bd0492f2b7a14ecf77efa0cd908ed1 | Bacteria | p__Actinobacteriota | c__Actinobacteria | o__Propionibacteriales | f__Nocardioidaceae | g__Nocardioides |  |
| MJ032-1-barcode26-umi44292bins-ubs-7 | Bacteria | p__Firmicutes | c__Clostridia | o__Peptostreptococcales-Tissierellales | f__Anaerovoracaceae | g__Anaerovorax |  |
| 04907733 | Bacteria | p__Proteobacteria | c__Alphaproteobacteria | o__Caulobacterales | f__Caulobacteraceae | g__Brevundimonas | s__Brevundimonas_olei |
| 57eae9e90ce07584c937bcb405ff249b | Bacteria | p__Actinobacteriota | c__Actinobacteria |  |  |  |  |
| 97b1e2331615bbf40ca1ebef60fd94a4 | Bacteria | p__Desulfobacterota | c__uncultured | o__uncultured | f__uncultured | g__uncultured |  |
| f2441e744a2d87ffa320ce343d9cad3f | Bacteria | p__Firmicutes | c__Clostridia | o__Lachnospirales | f__Lachnospiraceae | g__[Ruminococcus]_torques_group |  |
| MJ034-1-barcode34-umi5265bins-ubs-87 | Bacteria | p__Acidobacteriota | c__Blastocatellia | o__Blastocatellales | f__Blastocatellaceae |  |  |
| 08509614 | Bacteria | p__Proteobacteria | c__Alphaproteobacteria | o__Rhizobiales | f__Rhizobiaceae | g__Shinella |  |
| HE573918 | Bacteria | p__Firmicutes | c__Bacilli | o__Lactobacillales | f__Lactobacillaceae | g__Lactobacillus | s__Lactobacillus_taiwanensis |
| 02407809 | Bacteria | p__Firmicutes | c__Clostridia | o__Clostridiales | f__Clostridiaceae | g__Clostridium_sensu_stricto_3 | s__iron-reducing_bacterium |
| KJ556384 | Bacteria | p__Proteobacteria | c__Alphaproteobacteria | o__Rhizobiales | f__Rhizobiaceae |  |  |
| 01696435 | Bacteria | p__Actinobacteriota | c__Actinobacteria | o__Propionibacteriales | f__Propionibacteriaceae | g__Cutibacterium |  |
| 2444f4c136d701af24d5ecd15c1eca28 | Bacteria | p__Acidobacteriota | c__Vicinamibacteria | o__Vicinamibacterales | f__Vicinamibacteraceae | g__Vicinamibacteraceae |  |
| 56ec9b411185cc3d82d92d125d81d5cb | Bacteria | p__Proteobacteria | c__Alphaproteobacteria | o__Rhizobiales | f__Devosiaceae | g__Devosia |  |
| 03565258 | Bacteria | p__Planctomycetota | c__Planctomycetes | o__Gemmatales | f__Gemmataceae | g__uncultured |  |
| ee000e59ec5a256a6a26cfea071df591 | Bacteria | p__Planctomycetota | c__Planctomycetes | o__Gemmatales | f__Gemmataceae | g__Gemmata | s__uncultured_bacterium |
| eaee3fabcfc6e30cfe59f324981b9489 | Bacteria | p__Proteobacteria | c__Gammaproteobacteria | o__Burkholderiales | f__SC-I-84 | g__SC-I-84 |  |
| b0facabd0a535b0630d65c7aae5ee31b | Bacteria | p__Proteobacteria | c__Gammaproteobacteria | o__Alteromonadales | f__Alteromonadaceae | g__Rheinheimera |  |
| MJ006-1-barcode45-umi38159bins-ubs-10 | Bacteria | p__Firmicutes | c__Clostridia | o__Oscillospirales | f__[Eubacterium]_coprostanoligenes_group | g__[Eubacterium]_coprostanoligenes_group | s__gut_metagenome |
| f19adec303a010b1d50e8b2a061117df | Bacteria | p__Gemmatimonadota | c__Gemmatimonadetes | o__Gemmatimonadales | f__Gemmatimonadaceae | g__Gemmatimonas | s__uncultured_Gemmatimonadaceae |
| 02267002 | Bacteria | p__Proteobacteria | c__Alphaproteobacteria | o__Rhizobiales | f__Rhizobiaceae | g__Allorhizobium-Neorhizobium-Pararhizobium-Rhizobium | s__[Pseudomonas]_geniculata |
| de3728af9993b5bbca1bb3e0c401d7ae | Bacteria | p__Bacteroidota | c__Bacteroidia | o__Chitinophagales | f__Chitinophagaceae | g__Terrimonas |  |
| 18713466 | Bacteria | p__Actinobacteriota | c__Actinobacteria | o__Frankiales | f__Geodermatophilaceae | g__Blastococcus |  |
| MJ030-1-barcode42-umi115584bins-ubs-3 | Bacteria | p__Proteobacteria | c__Gammaproteobacteria | o__Burkholderiales | f__Rhodocyclaceae |  |  |
| VOUX01000001 | Bacteria | p__Proteobacteria | c__Gammaproteobacteria | o__Enterobacterales |  |  |  |
| 0719dc9482cb8eaa5addaec26795ef00 | Bacteria | p__Proteobacteria | c__Gammaproteobacteria | o__Burkholderiales | f__Burkholderiaceae | g__Ralstonia |  |
| ed9cd9a15e2b9a0b5f0a887d324de6dc | Bacteria | p__Firmicutes | c__Bacilli | o__Lactobacillales | f__Streptococcaceae | g__Streptococcus |  |
| 076c74abbd97055b11137d648ffa63d9 | Bacteria | p__Proteobacteria | c__Alphaproteobacteria | o__Caulobacterales | f__Hyphomonadaceae | g__SWB02 |  |
| aacaf6a6ff416de905c5b4b390f0fa16 | Bacteria | p__Proteobacteria | c__Gammaproteobacteria | o__Pseudomonadales | f__Moraxellaceae | g__Acinetobacter |  |
| 16618302 | Bacteria | p__Firmicutes | c__Clostridia | o__Lachnospirales | f__Lachnospiraceae |  |  |
| 04941315 | Bacteria | p__Acidobacteriota | c__Vicinamibacteria | o__Vicinamibacterales | f__uncultured | g__uncultured |  |
| 07941082 | Bacteria | p__Proteobacteria | c__Alphaproteobacteria | o__Rhizobiales | f__Hyphomicrobiaceae | g__Pedomicrobium |  |
| 1af2e4b5d6c6a6976f3d9d48a36991cc | Bacteria | p__Gemmatimonadota | c__Gemmatimonadetes | o__Gemmatimonadales | f__Gemmatimonadaceae | g__uncultured |  |
| RS-GCF-002764055.1-NZ-CP024749.1--4 | Bacteria | p__Fusobacteriota | c__Fusobacteriia | o__Fusobacteriales | f__Fusobacteriaceae | g__Fusobacterium |  |
| RS-GCF-002115725.1-NZ-JFKD01000077.1 | Bacteria | p__Proteobacteria | c__Gammaproteobacteria | o__Pseudomonadales | f__Pseudomonadaceae | g__Pseudomonas |  |
| 2a9e6e10f497a8475fa61acb308d1eb0 | Bacteria | p__Proteobacteria | c__Gammaproteobacteria | o__Alteromonadales | f__Shewanellaceae | g__Shewanella |  |
| 73fc0db41177c7f9ac2d09e15b609866 | Bacteria | p__Myxococcota | c__Polyangia | o__Haliangiales | f__Haliangiaceae | g__Haliangium | s__uncultured_Myxococcales |
| AJ575818 | Bacteria | p__Proteobacteria | c__Alphaproteobacteria | o__Sphingomonadales | f__Sphingomonadaceae | g__Sphingomonas | s__Sphingomonas_panni |
| 6e3f114eb1c7d26fd9b0a3e1db2a0036 | Bacteria | p__Actinobacteriota | c__Actinobacteria | o__Micromonosporales | f__Micromonosporaceae |  |  |
| c33a7f5ea399062e47df96fe3787be04 | Bacteria | p__Firmicutes | c__Bacilli | o__Erysipelotrichales | f__Erysipelatoclostridiaceae | g__Erysipelatoclostridium | s__uncultured_Clostridium |
| 014e71e8836971c450f0cbf3feedec8e | Bacteria | p__Firmicutes | c__Bacilli | o__Erysipelotrichales | f__Erysipelotrichaceae | g__Dubosiella | s__uncultured_bacterium |
| 11136045 | Bacteria | p__Bacteroidota | c__Bacteroidia | o__Bacteroidales | f__Muribaculaceae | g__Muribaculaceae | s__uncultured_organism |
| 46efd1608c554976c1bf27cd982d3c6e | Bacteria | p__Firmicutes | c__Clostridia | o__Lachnospirales | f__Lachnospiraceae | g__Lachnospiraceae_UCG-001 | s__uncultured_bacterium |
| 04931062 | Bacteria | p__Proteobacteria | c__Gammaproteobacteria | o__Cellvibrionales | f__Cellvibrionaceae | g__Cellvibrio |  |
| bd071e23efef3ca6a90be824e1ad677e | Bacteria | p__Proteobacteria | c__Gammaproteobacteria | o__Xanthomonadales | f__Xanthomonadaceae | g__Stenotrophomonas |  |
| GU129567 | Bacteria | p__Proteobacteria | c__Alphaproteobacteria | o__Rhodobacterales | f__Rhodobacteraceae | g__Paracoccus |  |
| 0a9fc8a9c9a84ded23f4cc82ea295189 | Bacteria | p__Acidobacteriota | c__Acidobacteriae | o__Acidobacteriales | f__uncultured | g__uncultured |  |
| MJ034-1-barcode34-umi21940bins-ubs-9 | Bacteria | p__Acidobacteriota | c__Blastocatellia | o__Pyrinomonadales | f__Pyrinomonadaceae | g__RB41 |  |
| 04875834 | Bacteria | p__Proteobacteria | c__Gammaproteobacteria | o__Pseudomonadales | f__Pseudomonadaceae | g__Pseudomonas |  |
| c4f84d1af9d525dbd68ac6675966ab2c | Bacteria | p__Proteobacteria | c__Gammaproteobacteria | o__Pseudomonadales | f__Moraxellaceae | g__Acinetobacter |  |
| 7d6a31fc9040bf850883c25e3ba89026 | Bacteria | p__Firmicutes | c__Clostridia | o__Lachnospirales | f__Lachnospiraceae | g__Roseburia |  |
| e4a74513bdfd2b49c064890dddf116b6 | Bacteria | p__Proteobacteria | c__Gammaproteobacteria | o__Burkholderiales |  |  |  |
| 1d16ccb7d0b2c48ab57fc3fd37a458e1 | Bacteria | p__Proteobacteria | c__Alphaproteobacteria | o__Rhizobiales | f__Devosiaceae | g__Devosia |  |
| 04934664 | Bacteria | p__Bacteroidota | c__Bacteroidia | o__Flavobacteriales | f__Weeksellaceae | g__Chryseobacterium |  |
| 15765272 | Bacteria | p__Actinobacteriota | c__Acidimicrobiia | o__IMCC26256 | f__IMCC26256 | g__IMCC26256 |  |
| c3a2132b9b63bcbf4f379e8c2436f268 | Bacteria | p__Firmicutes | c__Clostridia | o__Lachnospirales | f__Lachnospiraceae | g__Lachnospiraceae_NK4A136_group | s__Clostridiales_bacterium |
| f41297d7a9e4a9b76566deb78b7398f7 | Bacteria | p__Proteobacteria | c__Gammaproteobacteria | o__Pseudomonadales | f__Pseudomonadaceae | g__Pseudomonas |  |
| faf7436f7297e66e6915682b18dce0ed | Bacteria | p__Acidobacteriota | c__Acidobacteriae | o__Acidobacteriales | f__uncultured | g__uncultured |  |
| G001068545_1 | Bacteria | p__Firmicutes | c__Bacilli | o__Staphylococcales | f__Staphylococcaceae | g__Staphylococcus |  |
| 78e2c028fc4e63c5138c0541f77c3793 | Bacteria | p__Actinobacteriota | c__Actinobacteria | o__Frankiales | f__Sporichthyaceae |  |  |
| bb0b96668b7de0d35928373c50eb8bc5 | Bacteria | p__Actinobacteriota | c__Actinobacteria | o__Micrococcales | f__Intrasporangiaceae |  |  |
| 04901097 | Bacteria | p__Proteobacteria | c__Gammaproteobacteria | o__Steroidobacterales | f__Steroidobacteraceae | g__uncultured |  |
| 63fe8b6c32b476c58cb9aeb767fdafdf | Bacteria | p__Firmicutes | c__Clostridia | o__Oscillospirales | f__[Eubacterium]_coprostanoligenes_group | g__[Eubacterium]_coprostanoligenes_group |  |
| DQ411542 | Bacteria | p__Actinobacteriota | c__Actinobacteria | o__Propionibacteriales | f__Nocardioidaceae | g__Nocardioides |  |
| 05040313 | Bacteria | p__Firmicutes | c__Bacilli | o__Lactobacillales | f__Aerococcaceae | g__Abiotrophia | s__uncultured_bacterium |
| fd4239b7e34d1702a6a39e15af98a0f8 | Bacteria | p__Bacteroidota | c__Bacteroidia | o__Bacteroidales | f__Muribaculaceae | g__Muribaculaceae |  |
| 30b392bda0a386e6987b079f0caa6ab5 | Bacteria | p__Actinobacteriota | c__Actinobacteria | o__Glycomycetales | f__Glycomycetaceae | g__Glycomyces |  |
| 6141390651f4f2d9e4969e53f68eec89 | Bacteria | p__Actinobacteriota | c__Actinobacteria | o__Propionibacteriales | f__Propionibacteriaceae | g__Cutibacterium |  |
| 11839038 | Bacteria | p__Proteobacteria | c__Alphaproteobacteria | o__Sphingomonadales | f__Sphingomonadaceae | g__Sphingomonas |  |
| cc4190743b93d0822052b8971694902e | Bacteria | p__Acidobacteriota | c__Acidobacteriae | o__Acidobacteriales | f__uncultured | g__uncultured |  |
| MJ030-2-barcode65-umi88949bins-ubs-6 | Bacteria | p__Firmicutes | c__Bacilli | o__Alicyclobacillales | f__Alicyclobacillaceae | g__Tumebacillus |  |
| 11426067 | Bacteria | p__Proteobacteria | c__Alphaproteobacteria | o__Sphingomonadales | f__Sphingomonadaceae | g__Blastomonas |  |
| 06153547 | Bacteria | p__Proteobacteria | c__Gammaproteobacteria | o__Gammaproteobacteria_Incertae_Sedis | f__Unknown_Family | g__Acidibacter |  |
| 02244747 | Bacteria | p__Actinobacteriota | c__Actinobacteria | o__Micrococcales | f__Microbacteriaceae | g__Microbacterium |  |
| 04917666 | Bacteria | p__Bacteroidota | c__Bacteroidia | o__Flavobacteriales | f__Flavobacteriaceae | g__Flavobacterium |  |
| 5c7eabecba09398200684d9a7fd9b16a | Bacteria | p__Acidobacteriota | c__Acidobacteriae | o__Acidobacteriales | f__Acidobacteriaceae_(Subgroup_1) | g__uncultured |  |
| f0d8a19db837887d08bec4bcee549551 | Bacteria | p__Bacteroidota | c__Bacteroidia | o__Bacteroidales | f__Dysgonomonadaceae | g__Petrimonas |  |
| FR775448 | Bacteria | p__Proteobacteria | c__Alphaproteobacteria | o__Caulobacterales | f__Caulobacteraceae | g__Brevundimonas |  |
| b088304bebc83b9e5ef2eed6ca3bdddb | Bacteria | p__Proteobacteria | c__Gammaproteobacteria | o__Xanthomonadales | f__Xanthomonadaceae | g__Stenotrophomonas |  |
| d5cc3cec0606153a29b6947750e10f56 | Bacteria | p__Proteobacteria | c__Gammaproteobacteria | o__Pseudomonadales | f__Moraxellaceae | g__Acinetobacter |  |
| a5773010465d14be5fa432e49473fceb | Bacteria | p__Proteobacteria | c__Gammaproteobacteria | o__Burkholderiales | f__Burkholderiaceae | g__Lautropia | s__metagenome |
| 04966006 | Bacteria | p__Firmicutes | c__Bacilli | o__Bacillales | f__Planococcaceae | g__Sporosarcina |  |
| 04994984 | Bacteria | p__Proteobacteria | c__Alphaproteobacteria | o__Rhizobiales | f__Rhizobiaceae | g__Allorhizobium-Neorhizobium-Pararhizobium-Rhizobium |  |
| 2717dc855a9df52c031c33ddaa7bd1a2 | Bacteria | p__Bacteroidota | c__Bacteroidia | o__Bacteroidales | f__Bacteroidaceae | g__Bacteroides | s__Bacteroides_plebeius |
| MJ034-1-barcode25-umi17353bins-ubs-17 | Bacteria | p__Proteobacteria | c__Alphaproteobacteria | o__Rhizobiales | f__Devosiaceae | g__Devosia |  |
| 175da00dfb734a29c6793bc5a6e00737 | Bacteria | p__Proteobacteria | c__Gammaproteobacteria | o__Pseudomonadales | f__Moraxellaceae | g__Acinetobacter |  |
| G001406515_2 | Bacteria | p__Firmicutes | c__Clostridia | o__Clostridiales | f__Clostridiaceae | g__Clostridium_sensu_stricto_1 |  |
| ca8119dd7961c5f640c9635f27d60250 | Bacteria | p__Firmicutes | c__Bacilli | o__RF39 | f__RF39 | g__RF39 |  |
| 08585570 | Bacteria | p__Proteobacteria | c__Alphaproteobacteria | o__Azospirillales | f__Azospirillaceae | g__Niveispirillum |  |
| G009767915_1 | Bacteria | p__Proteobacteria | c__Gammaproteobacteria | o__Burkholderiales | f__Sutterellaceae | g__Parasutterella | s__uncultured_bacterium |
| 60c5b2fb85df93a9ebf891129ee5f212 | Bacteria | p__Actinobacteriota | c__Actinobacteria | o__Propionibacteriales | f__Nocardioidaceae |  |  |
| G000403475_1 | Bacteria | p__Firmicutes | c__Clostridia | o__Lachnospirales | f__Lachnospiraceae | g__Lachnoclostridium |  |
| 01253146 | Bacteria | p__Desulfobacterota | c__Desulfovibrionia | o__Desulfovibrionales | f__Desulfovibrionaceae | g__uncultured | s__uncultured_Desulfovibrionales |
| AJ635304 | Bacteria | p__Proteobacteria | c__Alphaproteobacteria | o__Rhizobiales | f__Beijerinckiaceae | g__Methylobacterium-Methylorubrum |  |
| MH553383 | Bacteria | p__Actinobacteriota | c__Actinobacteria | o__Frankiales | f__Geodermatophilaceae | g__Blastococcus |  |
| 514d0af4bd389475689d1cde53f8b33b | Bacteria | p__Proteobacteria | c__Gammaproteobacteria | o__Burkholderiales | f__Nitrosomonadaceae | g__Ellin6067 |  |
| 08636338 | Bacteria | p__Proteobacteria | c__Alphaproteobacteria | o__Rhizobiales | f__Devosiaceae | g__Devosia |  |
| a973e1f4a9b50297089bd686b77524ae | Bacteria | p__Actinobacteriota | c__Thermoleophilia | o__Gaiellales |  |  |  |
| G000759675_1 | Bacteria | p__Firmicutes | c__Bacilli | o__Bacillales | f__Bacillaceae | g__Bacillus |  |
| ebfdb2776a61d3557aa414b4dacb7f67 | Bacteria | p__Bdellovibrionota | c__Bdellovibrionia | o__Bdellovibrionales | f__Bdellovibrionaceae | g__Bdellovibrio |  |
| ea38ad006107eccb9da988ca77ab5616 | Bacteria | p__Proteobacteria | c__Gammaproteobacteria | o__PLTA13 | f__PLTA13 | g__PLTA13 |  |
| MJ006-1-barcode26-umi112828bins-ubs-4 | Bacteria | p__Firmicutes | c__Clostridia | o__Oscillospirales | f__[Eubacterium]_coprostanoligenes_group | g__[Eubacterium]_coprostanoligenes_group | s__human_gut |
| a87812711fda4426148a739d8e812df8 | Bacteria | p__Actinobacteriota | c__Actinobacteria | o__Pseudonocardiales | f__Pseudonocardiaceae | g__Prauserella |  |
| 2d4bc8f4496de0e3d619fe0d41412454 | Bacteria | p__Proteobacteria | c__Gammaproteobacteria | o__Enterobacterales | f__Enterobacteriaceae | g__Escherichia-Shigella |  |
| 04905367 | Bacteria | p__Proteobacteria | c__Alphaproteobacteria | o__Dongiales | f__Dongiaceae | g__Dongia |  |
| MJ006-1-barcode28-umi74131bins-ubs-5 | Bacteria | p__Firmicutes | c__Clostridia | o__Lachnospirales | f__Lachnospiraceae | g__Lachnospiraceae_NK4A136_group |  |
| f19ac2ec42bcfef22b57203b0706a379 | Bacteria | p__Proteobacteria | c__Gammaproteobacteria | o__Burkholderiales | f__Alcaligenaceae |  |  |
| 16f46ae5e4500bdd3993bf827f1773a4 | Bacteria | p__Verrucomicrobiota | c__Chlamydiae | o__Chlamydiales | f__Parachlamydiaceae | g__Neochlamydia | s__metagenome |
| 479d3561798551b7f33adf8880969716 | Bacteria | p__Proteobacteria | c__Alphaproteobacteria | o__Rhizobiales | f__Rhizobiaceae |  |  |
| 2413ebbe3e6cdcfe4ed79b3f8d1268d1 | Bacteria | p__Proteobacteria | c__Alphaproteobacteria | o__Rhizobiales | f__Rhizobiaceae |  |  |
| c6d79c46f63fa7d560e42863087212bd | Bacteria | p__Proteobacteria | c__Alphaproteobacteria | o__Caulobacterales | f__Caulobacteraceae |  |  |
| 052147631c44afd3242c9f0d6ef7a6d7 | Bacteria | p__Proteobacteria | c__Gammaproteobacteria | o__Pseudomonadales | f__Moraxellaceae | g__Acinetobacter |  |
| JN033775 | Bacteria | p__Actinobacteriota | c__Acidimicrobiia | o__Microtrichales | f__Iamiaceae | g__Iamia |  |
| RS-GCF-018619375.1-NZ-JAHHFT010000043.1 | Bacteria | p__Firmicutes | c__Bacilli | o__Lactobacillales | f__Streptococcaceae | g__Streptococcus |  |
| 7426204b645f8b4eaa1be6522a5cefe2 | Bacteria | p__Proteobacteria | c__Alphaproteobacteria | o__Acetobacterales | f__Acetobacteraceae |  |  |
| 241d86344ab5e21f7579d0415e6ec33b | Bacteria | p__Proteobacteria | c__Gammaproteobacteria | o__Pseudomonadales | f__Pseudomonadaceae | g__Pseudomonas |  |
| c07661b3f9141db25615890760f06c66 | Bacteria | p__Proteobacteria | c__Alphaproteobacteria | o__Rickettsiales | f__Anaplasmataceae | g__Wolbachia |  |
| MJ006-1-barcode37-umi152549bins-ubs-3 | Bacteria | p__Bacteroidota | c__Bacteroidia | o__Bacteroidales | f__Bacteroidaceae | g__Bacteroides |  |
| b5635a67c098a8199ac6000aaed3f656 | Bacteria | p__Actinobacteriota | c__Actinobacteria | o__Micrococcales | f__Microbacteriaceae | g__Agromyces |  |
| MJ006-1-barcode42-umi101368bins-ubs-5 | Bacteria | p__Firmicutes | c__Clostridia | o__Lachnospirales | f__Lachnospiraceae | g__[Eubacterium]_ventriosum_group |  |
| AB600999 | Bacteria | p__Bacteroidota | c__Bacteroidia | o__Bacteroidales | f__Rikenellaceae | g__Alistipes | s__Alistipes_indistinctus |
| 331ac785855543100baed4b894c09348 | Bacteria | p__Proteobacteria | c__Gammaproteobacteria | o__Burkholderiales | f__B1-7BS | g__B1-7BS |  |
| 6f93d9cd32a635de6a2389dde6d65504 | Bacteria | p__Acidobacteriota | c__Acidobacteriae | o__Subgroup_2 | f__Subgroup_2 | g__Subgroup_2 |  |
| d8b7778ac1b994428a405858811d2e1f | Bacteria | p__Actinobacteriota | c__Actinobacteria | o__Corynebacteriales | f__Corynebacteriaceae | g__Corynebacterium |  |
| 2fbce24c5a5f68f8cd2d9e9abcd1dcba | Bacteria | p__Actinobacteriota | c__Actinobacteria | o__Micrococcales | f__Micrococcaceae |  |  |
| KU296959 | Bacteria | p__Proteobacteria | c__Gammaproteobacteria | o__Xanthomonadales | f__Rhodanobacteraceae | g__Dyella |  |
| 5e6952410ba5ce5e0b1a6050b0bfd63b | Bacteria | p__Proteobacteria | c__Gammaproteobacteria | o__Pseudomonadales | f__Moraxellaceae | g__Acinetobacter |  |
| a8837a1f08511fb2ab6c6b333264b08b | Bacteria | p__Chloroflexi | c__KD4-96 | o__KD4-96 | f__KD4-96 | g__KD4-96 |  |
| 5b544f7042fd6f21f4f0e100cc577e75 | Bacteria | p__Acidobacteriota | c__Acidobacteriae | o__Acidobacteriales | f__uncultured | g__uncultured |  |
| a07a71ed98dff0ec3ac48a1d9cc48267 | Bacteria | p__Actinobacteriota | c__Actinobacteria | o__Micrococcales |  |  |  |
| 2935ba54cc887552fa885e0da79ab059 | Bacteria | p__Actinobacteriota | c__Actinobacteria | o__Corynebacteriales | f__Nocardiaceae |  |  |
| 783c875901e4c06f077c68dbd662d6d4 | Bacteria | p__Proteobacteria | c__Gammaproteobacteria | o__Burkholderiales | f__Comamonadaceae | g__Comamonas |  |
| 165822db6db637d9496cbf7036567b1f | Bacteria | p__Firmicutes | c__Bacilli | o__Paenibacillales | f__Paenibacillaceae | g__Paenibacillus |  |
| 04925553 | Bacteria | p__Proteobacteria | c__Alphaproteobacteria | o__Sphingomonadales | f__Sphingomonadaceae | g__Sphingomonas |  |
| MJ009-2-barcode67-umi128463bins-ubs-4 | Bacteria | p__Actinobacteriota | c__Actinobacteria | o__Micrococcales | f__Microbacteriaceae |  |  |
| 0d490dd4483a5cdfe9f1423594b4b242 | Bacteria | p__Proteobacteria | c__Alphaproteobacteria | o__Rhizobiales | f__Xanthobacteraceae |  |  |
| 08033821 | Bacteria | p__Proteobacteria | c__Alphaproteobacteria | o__Dongiales | f__Dongiaceae | g__Dongia |  |
| G000452245_10 | Bacteria | p__Firmicutes | c__Clostridia | o__Peptostreptococcales-Tissierellales | f__Peptostreptococcaceae | g__Paraclostridium |  |
| LT601575 | Bacteria | p__Actinobacteriota | c__Actinobacteria | o__Micrococcales | f__Micrococcaceae | g__Nesterenkonia |  |
| 67e79cb3df2c132ccfbf234fc260ff25 | Bacteria | p__Firmicutes | c__Bacilli | o__Bacillales | f__Planococcaceae | g__Rummeliibacillus |  |
| e51fe64dd7475da111d2fe7904e9e864 | Bacteria | p__Proteobacteria | c__Gammaproteobacteria | o__Xanthomonadales | f__Rhodanobacteraceae | g__Ahniella |  |
| 233ad52dff7eea91d323cca117530f70 | Bacteria | p__Bacteroidota | c__Bacteroidia | o__Sphingobacteriales | f__Sphingobacteriaceae | g__Sphingobacterium |  |
| 05109954 | Bacteria | p__Actinobacteriota | c__Thermoleophilia | o__Solirubrobacterales | f__Solirubrobacteraceae | g__Conexibacter |  |
| MJ006-1-barcode45-umi167744bins-ubs-3 | Bacteria | p__Proteobacteria | c__Gammaproteobacteria | o__Pasteurellales | f__Pasteurellaceae | g__Haemophilus |  |
| MK248116 | Bacteria | p__Proteobacteria | c__Gammaproteobacteria | o__Pseudomonadales | f__Pseudomonadaceae | g__Pseudomonas |  |
| GB-GCA-016742095.1-JACVCJ010000046.1 | Bacteria | p__Bacteroidota | c__Bacteroidia |  |  |  |  |
| d4a7a8d50c36c2302c29f551d06de00f | Bacteria | p__Proteobacteria | c__Gammaproteobacteria | o__Enterobacterales | f__Enterobacteriaceae |  |  |
| 4096503c4befa8dd4793a3721d65e698 | Bacteria | p__Proteobacteria | c__Gammaproteobacteria | o__Pseudomonadales | f__Pseudomonadaceae | g__Pseudomonas |  |
| 5f5e133e2fb430765915fd0b85a6e7ca | Bacteria | p__Bacteroidota | c__Bacteroidia | o__Chitinophagales | f__Chitinophagaceae |  |  |
| 688cdf6d542553c81f33dd4c7ce6c8d4 | Bacteria | p__Bacteroidota | c__Bacteroidia | o__Sphingobacteriales | f__Sphingobacteriaceae | g__Mucilaginibacter |  |
| 7fe7c920816aee7c8804ff9973fa2f9b | Bacteria | p__Actinobacteriota | c__Actinobacteria | o__Micrococcales | f__Microbacteriaceae |  |  |
| f88b53fb6aaf7590d4fe339fdc27a4b8 | Bacteria | p__Planctomycetota | c__Phycisphaerae | o__Tepidisphaerales | f__WD2101_soil_group | g__WD2101_soil_group |  |
| cc0341bac9f8e5f2ce895ab589c7ed4f | Bacteria | p__Proteobacteria | c__Gammaproteobacteria | o__Pseudomonadales | f__Moraxellaceae | g__Acinetobacter |  |
| 05011552 | Bacteria | p__Proteobacteria | c__Gammaproteobacteria | o__Cellvibrionales | f__Cellvibrionaceae | g__Cellvibrio |  |
| bb45d1f5dab0aa34949570d3e4762021 | Bacteria | p__Proteobacteria | c__Alphaproteobacteria | o__Rhizobiales | f__Xanthobacteraceae | g__uncultured |  |
| fadbfadd2ad220908465226ed9b6abe1 | Bacteria | p__Chloroflexi | c__Chloroflexia | o__Thermomicrobiales | f__JG30-KF-CM45 | g__JG30-KF-CM45 | s__metagenome |
| c7b52d8624f3d847d34af546ddb4276e | Bacteria | p__Proteobacteria | c__Alphaproteobacteria | o__Sphingomonadales | f__Sphingomonadaceae | g__Sphingomonas |  |
| 130f9725a428462cc00d2e81ac338508 | Bacteria | p__Proteobacteria | c__Alphaproteobacteria | o__Rhizobiales | f__Rhizobiaceae |  |  |
| 574b3314cb424063b26a14578021e6b1 | Bacteria | p__Nitrospirota | c__Nitrospiria | o__Nitrospirales | f__Nitrospiraceae | g__Nitrospira |  |
| MJ031-2-barcode62-umi45863bins-ubs-4 | Bacteria | p__Firmicutes | c__Bacilli | o__Bacillales | f__Bacillaceae | g__Bacillus |  |
| d862902c8a569bd307c6bf3791e304f9 | Bacteria | p__Actinobacteriota | c__Acidimicrobiia | o__IMCC26256 | f__IMCC26256 | g__IMCC26256 |  |
| 0af81343216471cddee0703157147b56 | Bacteria | p__Proteobacteria | c__Alphaproteobacteria | o__Sphingomonadales | f__Sphingomonadaceae |  |  |
| c6d39d38900a2087b441189efcaa3a90 | Bacteria | p__Gemmatimonadota | c__Gemmatimonadetes | o__Gemmatimonadales | f__Gemmatimonadaceae | g__uncultured |  |
| 0811417e9275e57e61b6225fd6c6364d | Bacteria | p__Proteobacteria | c__Alphaproteobacteria | o__Defluviicoccales | f__Defluviicoccaceae | g__Defluviicoccus | s__uncultured_Defluviicoccus |
| 04933202 | Bacteria | p__Bacteroidota | c__Bacteroidia | o__Chitinophagales | f__Chitinophagaceae | g__Flavisolibacter |  |
| 52fd09f7eb01a82261f8515166bb4ee9 | Bacteria | p__Bacteroidota | c__Bacteroidia | o__Chitinophagales | f__Saprospiraceae | g__uncultured |  |
| MH277174 | Bacteria | p__Bacteroidota | c__Bacteroidia | o__Chitinophagales | f__Chitinophagaceae | g__Lacibacter | s__Lacibacter_cauensis |
| f1dc30c7224faf95a87eb02bf1539604 | Bacteria | p__Bacteroidota | c__Bacteroidia | o__Sphingobacteriales | f__Sphingobacteriaceae | g__Sphingobacterium |  |
| c4f75d7b073eb40a0d54531792e871c7 | Bacteria | p__Firmicutes | c__Clostridia | o__Clostridia_UCG-014 | f__Clostridia_UCG-014 | g__Clostridia_UCG-014 |  |
| RS-GCF-019127415.1-NZ-JAHOEK010000122.1 | Bacteria | p__Bacteroidota | c__Bacteroidia | o__Bacteroidales | f__Prevotellaceae | g__Prevotella |  |
| 7288d0849c0b04f222dd5ad4a52809b1 | Bacteria | p__Chloroflexi | c__Chloroflexia | o__Thermomicrobiales | f__JG30-KF-CM45 | g__JG30-KF-CM45 | s__uncultured_Chloroflexi |
| 08509630 | Bacteria | p__Proteobacteria | c__Alphaproteobacteria | o__Rhizobiales | f__Rhizobiaceae |  |  |
| G000344785_3 | Bacteria | p__Actinobacteriota | c__Actinobacteria | o__Corynebacteriales | f__Corynebacteriaceae | g__Corynebacterium |  |
| 64a36891b3722cdf1e8ebd564e575adc | Bacteria | p__Actinobacteriota | c__Thermoleophilia | o__Gaiellales | f__Gaiellaceae | g__Gaiella |  |
| 737c454d56cc2cd9c534833a86cc0c0e | Bacteria | p__Proteobacteria | c__Gammaproteobacteria | o__Pseudomonadales | f__Moraxellaceae | g__Acinetobacter |  |
| d008af1e152d92a6e20bd656b345d540 | Bacteria | p__Firmicutes | c__Clostridia | o__Peptostreptococcales-Tissierellales | f__Peptostreptococcaceae | g__Romboutsia |  |
| 552db082a4b6697948391676666e4288 | Bacteria | p__Actinobacteriota | c__Actinobacteria | o__Corynebacteriales | f__Mycobacteriaceae | g__Mycobacterium |  |
| 04212051 | Bacteria | p__Chloroflexi | c__Chloroflexia | o__Thermomicrobiales | f__AKYG1722 | g__AKYG1722 | s__uncultured_Alphaproteobacteria |
| 15342060 | Bacteria | p__Actinobacteriota | c__Thermoleophilia | o__Solirubrobacterales | f__67-14 | g__67-14 | s__metagenome |
| AB778526 | Bacteria | p__Proteobacteria | c__Alphaproteobacteria | o__Acetobacterales | f__Acetobacteraceae | g__Gluconacetobacter |  |
| GB-GCA-014868765.1-WHEU01000011.1 | Bacteria | p__Firmicutes | c__Clostridia | o__Oscillospirales | f__Ruminococcaceae | g__Faecalibacterium |  |
| 14800836 | Bacteria | p__Chloroflexi | c__Ktedonobacteria | o__Ktedonobacterales | f__JG30-KF-AS9 | g__JG30-KF-AS9 | s__uncultured_bacterium |
| adef1e78994be58081c1f19d774234da | Bacteria | p__Bacteroidota | c__Kapabacteria | o__Kapabacteriales | f__Kapabacteriales | g__Kapabacteriales | s__uncultured_bacterium |
| e0e04adfcbce8cdb9e11eb613ee0729a | Bacteria | p__Acidobacteriota | c__Acidobacteriae | o__Bryobacterales | f__Bryobacteraceae | g__Bryobacter |  |
| 44200e96c42ba59e2bbd465f9e7f8756 | Bacteria | p__Actinobacteriota | c__Actinobacteria | o__Corynebacteriales | f__Mycobacteriaceae | g__Mycobacterium |  |
| c71dc7ab6c9f0b6ee14c512f99032c6d | Bacteria | p__Firmicutes | c__Bacilli | o__Lactobacillales | f__Enterococcaceae | g__Enterococcus |  |
| GB-GCA-910575825.1-CAJTET010000077.1 | Bacteria | p__Firmicutes | c__Clostridia | o__Lachnospirales | f__Lachnospiraceae | g__GCA-900066575 | s__uncultured_bacterium |
| f27a265987fe7568ffc0e37c9a9d4f1d | Bacteria | p__Bacteroidota | c__Bacteroidia | o__Bacteroidales | f__Muribaculaceae | g__Muribaculaceae |  |
| G000963965_1 | Bacteria | p__Proteobacteria | c__Alphaproteobacteria | o__Acetobacterales | f__Acetobacteraceae | g__Acetobacter |  |
| AJ132378 | Bacteria | p__Proteobacteria | c__Alphaproteobacteria | o__Rhizobiales | f__Rhizobiaceae |  |  |
| 18422836 | Bacteria | p__Proteobacteria | c__Gammaproteobacteria | o__Burkholderiales | f__Comamonadaceae |  |  |
| MJ034-1-barcode26-umi24205bins-ubs-6 | Bacteria | p__Proteobacteria | c__Gammaproteobacteria | o__Burkholderiales | f__TRA3-20 | g__TRA3-20 |  |
| ab5e1f4d87355d39e72783912be1e842 | Bacteria | p__Acidobacteriota | c__Acidobacteriae | o__Solibacterales | f__Solibacteraceae | g__Candidatus_Solibacter |  |
| 51b2455af63ca5458c0d11d21a1083f9 | Bacteria | p__Actinobacteriota | c__Actinobacteria | o__Micromonosporales | f__Micromonosporaceae |  |  |
| e829f425d29c85f57e464e2312a6e7f9 | Bacteria | p__Desulfobacterota | c__Desulfovibrionia | o__Desulfovibrionales | f__Desulfomicrobiaceae | g__Desulfomicrobium | s__uncultured_bacterium |
| KF056993 | Bacteria | p__Proteobacteria | c__Alphaproteobacteria | o__Caulobacterales | f__Hyphomonadaceae | g__Hirschia |  |
| f7c41de3d5dd48486c0a8d8b90500ce1 | Bacteria | p__Actinobacteriota | c__Actinobacteria | o__Frankiales | f__Frankiales | g__Frankiales | s__uncultured_bacterium |
| 02266860 | Bacteria | p__Proteobacteria | c__Alphaproteobacteria | o__Sphingomonadales | f__Sphingomonadaceae | g__Sphingomonas |  |
| RS-GCF-000383555.1-NZ-KB911577.1 | Bacteria | p__Actinobacteriota | c__Actinobacteria | o__Corynebacteriales | f__Nocardiaceae | g__Rhodococcus |  |
| 35a66116268343aec27b1be3bea7e097 | Bacteria | p__Chloroflexi | c__Ktedonobacteria | o__Ktedonobacterales | f__Ktedonobacteraceae | g__Ktedonobacter |  |
| 640b7b40ea05b2d5a3b2d7e1c8a8b03f | Bacteria | p__Actinobacteriota | c__Acidimicrobiia | o__Microtrichales | f__Ilumatobacteraceae | g__CL500-29_marine_group |  |
| eecf1f2b143e74f0b8482474c6cb50ed | Bacteria | p__Acidobacteriota | c__Acidobacteriae | o__Solibacterales | f__Solibacteraceae | g__Candidatus_Solibacter |  |
| HQ263024 | Bacteria | p__Firmicutes | c__Bacilli | o__Lactobacillales | f__Leuconostocaceae | g__Leuconostoc |  |
| 04699980 | Bacteria | p__Proteobacteria | c__Gammaproteobacteria | o__Pseudomonadales | f__Pseudomonadaceae | g__Pseudomonas |  |
| f01446993f45aca352b1faa2860f6ad0 | Bacteria | p__Proteobacteria | c__Alphaproteobacteria | o__Rhizobiales | f__Devosiaceae | g__Devosia |  |
| 17773225 | Bacteria | p__Armatimonadota | c__Armatimonadia | o__Armatimonadales | f__Armatimonadales | g__Armatimonadales |  |
| 0a924b943ba937803f8ac29d0ecab730 | Bacteria | p__Acidobacteriota | c__Acidobacteriae | o__Solibacterales | f__Solibacteraceae | g__Candidatus_Solibacter |  |
| 9ab1241416e5a44341800db16289cb89 | Bacteria | p__Bacteroidota | c__Bacteroidia | o__Chitinophagales | f__Chitinophagaceae | g__Ferruginibacter |  |
| 05002722 | Bacteria | p__Bacteroidota | c__Bacteroidia | o__Sphingobacteriales | f__Sphingobacteriaceae | g__Sphingobacterium |  |
| LT009497 | Bacteria | p__Proteobacteria | c__Alphaproteobacteria | o__Acetobacterales | f__Acetobacteraceae | g__Roseomonas |  |
| c090a3b256698e1d57af41eb3146b713 | Bacteria | p__Actinobacteriota | c__Actinobacteria | o__Micrococcales | f__Brevibacteriaceae | g__Brevibacterium |  |
| 07c071598060ec7fcd59c1f665295b06 | Bacteria | p__Firmicutes | c__Bacilli | o__Lactobacillales | f__Carnobacteriaceae | g__Atopostipes |  |
| RS-GCF-004569355.1-NZ-SPPR01000066.1 | Bacteria | p__Bacteroidota | c__Bacteroidia | o__Bacteroidales | f__Tannerellaceae |  |  |
| 02407747 | Bacteria | p__Firmicutes | c__Clostridia | o__Lachnospirales | f__Lachnospiraceae | g__[Eubacterium]_hallii_group |  |
| 902a5250ee021114b3054a4c89771cc2 | Bacteria | p__Chloroflexi | c__Dehalococcoidia | o__S085 | f__S085 | g__S085 | s__metagenome |
| G000245075_1 | Bacteria | p__Proteobacteria | c__Alphaproteobacteria | o__Acetobacterales | f__Acetobacteraceae | g__Roseomonas |  |
| 6e3d6b139138d7f8c7d256da58c408bf | Bacteria | p__Actinobacteriota | c__Actinobacteria | o__Frankiales | f__Acidothermaceae | g__Acidothermus | s__uncultured_Thermomonosporaceae |
| c32ef98f3dbfc22965131afa8b626136 | Bacteria | p__Bacteroidota | c__Bacteroidia | o__Chitinophagales | f__Saprospiraceae | g__uncultured |  |
| 14357626 | Bacteria | p__Acidobacteriota | c__Vicinamibacteria | o__Vicinamibacterales | f__uncultured | g__uncultured |  |
| 521f576acdec802c57a474ba701f7932 | Bacteria | p__Actinobacteriota | c__Actinobacteria | o__Micromonosporales | f__Micromonosporaceae |  |  |
| 05041805 | Bacteria | p__Firmicutes | c__Bacilli | o__Lactobacillales | f__Leuconostocaceae | g__Leuconostoc |  |
| d4e7be1938a7a816960a0b59a6abf3b6 | Bacteria | p__Myxococcota | c__Myxococcia | o__Myxococcales | f__Myxococcaceae |  |  |
| 29528fef4045b620db04b64be1ce8d93 | Bacteria | p__Proteobacteria | c__Gammaproteobacteria | o__Burkholderiales | f__Methylophilaceae | g__Methylobacillus |  |
| 87664d0deec5d8f411d23c0945721f5f | Bacteria | p__Patescibacteria | c__Saccharimonadia | o__Saccharimonadales | f__Saccharimonadaceae | g__TM7a | s__uncultured_compost |
| KT191026 | Bacteria | p__Proteobacteria | c__Alphaproteobacteria | o__Caulobacterales | f__Caulobacteraceae | g__Phenylobacterium |  |
| 04989896 | Bacteria | p__Proteobacteria | c__Gammaproteobacteria | o__Pseudomonadales | f__Moraxellaceae | g__Acinetobacter |  |
| 04900890 | Bacteria | p__Myxococcota | c__Myxococcia | o__Myxococcales | f__Myxococcaceae |  |  |
| f3050ced9a453998129b4a0e90f3fdf1 | Bacteria | p__Verrucomicrobiota | c__Verrucomicrobiae | o__Chthoniobacterales | f__Chthoniobacteraceae | g__Candidatus_Udaeobacter |  |
| MJ006-1-barcode28-umi162137bins-ubs-3 | Bacteria | p__Bacteroidota | c__Bacteroidia | o__Bacteroidales | f__Bacteroidaceae | g__Bacteroides |  |
| 7943310966ea57af1bbd09655db5c18c | Bacteria | p__Proteobacteria | c__Alphaproteobacteria | o__Acetobacterales | f__Acetobacteraceae | g__Rhodovastum | s__uncultured_bacterium |
| 05007436 | Bacteria | p__Bacteroidota | c__Bacteroidia | o__Bacteroidales | f__Rikenellaceae | g__Alistipes | s__uncultured_bacterium |
| RS-GCF-001954425.1-NZ-MPTC01000001.1 | Bacteria | p__Firmicutes | c__Bacilli | o__Paenibacillales | f__Paenibacillaceae | g__Paenibacillus |  |
| 04910980 | Bacteria | p__Proteobacteria | c__Gammaproteobacteria | o__Xanthomonadales | f__Xanthomonadaceae | g__Stenotrophomonas |  |
| 02250709 | Bacteria | p__Proteobacteria | c__Alphaproteobacteria | o__Caulobacterales | f__Caulobacteraceae | g__Brevundimonas |  |
| 136aef7b5f51878a467ee74ad7aa5d7b | Bacteria | p__Actinobacteriota | c__Acidimicrobiia | o__IMCC26256 | f__IMCC26256 | g__IMCC26256 |  |
| dcd1b8702c0a70acf7323faacdc6e848 | Bacteria | p__Verrucomicrobiota | c__Verrucomicrobiae | o__Chthoniobacterales | f__Chthoniobacteraceae | g__Candidatus_Udaeobacter |  |
| 6cd649ed5479db335ac31976cb4aec4d | Bacteria | p__Chloroflexi | c__Anaerolineae | o__Caldilineales | f__Caldilineaceae |  |  |
| ec15331b7b622728815921d0de345c47 | Bacteria | p__Proteobacteria | c__Alphaproteobacteria | o__Tistrellales | f__Geminicoccaceae | g__uncultured | s__uncultured_bacterium |
| MJ010-2-barcode64-umi101327bins-ubs-6 | Bacteria | p__Proteobacteria | c__Gammaproteobacteria | o__Enterobacterales | f__Enterobacteriaceae |  |  |
| 03930564 | Bacteria | p__Gemmatimonadota | c__BD2-11_terrestrial_group | o__BD2-11_terrestrial_group | f__BD2-11_terrestrial_group | g__BD2-11_terrestrial_group |  |
| 52310577b3b9ef961c73ce3b2cc1bef0 | Bacteria | p__Actinobacteriota | c__Actinobacteria | o__Propionibacteriales | f__Nocardioidaceae | g__Nocardioides |  |
| 50eac9680f6a58519e799b5ba10ee8af | Bacteria | p__Acidobacteriota | c__Acidobacteriae | o__Acidobacteriales | f__uncultured | g__uncultured |  |
| 7d7c10a44db72e36dc9823817f81aa15 | Bacteria | p__Proteobacteria | c__Gammaproteobacteria | o__Xanthomonadales | f__Rhodanobacteraceae | g__Ahniella |  |
| 08335133 | Bacteria | p__Proteobacteria | c__Alphaproteobacteria | o__Caulobacterales | f__Caulobacteraceae | g__Phenylobacterium |  |
| b22ef95346479bef070637f9de3801dd | Bacteria | p__Firmicutes | c__Clostridia | o__Clostridiales | f__Clostridiaceae | g__Clostridium_sensu_stricto_1 |  |
| G002798305_1 | Bacteria | p__Firmicutes | c__Bacilli | o__Bacillales | f__Planococcaceae | g__Chryseomicrobium |  |
| 6bddde99a01eda7314f4fc088094e654 | Bacteria | p__Actinobacteriota | c__Thermoleophilia | o__Gaiellales | f__uncultured | g__uncultured |  |
| ac65eb5e88d5bae1589921b725d89d75 | Bacteria | p__Actinobacteriota | c__Thermoleophilia | o__Gaiellales |  |  |  |
| 1615ea9ee389fea63f064fe7ad028656 | Bacteria | p__Firmicutes | c__Clostridia | o__Lachnospirales | f__Lachnospiraceae | g__Lachnospiraceae_NK3A20_group |  |
| b9a54ec7210f61140f0384f81e9cd63d | Bacteria | p__Acidobacteriota | c__Acidobacteriae | o__Acidobacteriales | f__Acidobacteriaceae_(Subgroup_1) | g__Edaphobacter |  |
| 01683269 | Bacteria | p__Actinobacteriota | c__Actinobacteria | o__Propionibacteriales | f__Propionibacteriaceae | g__Cutibacterium |  |
| 33c6ceb0e6fd7f86c5189bf3416b3066 | Bacteria | p__Acidobacteriota | c__Acidobacteriae | o__Bryobacterales | f__Bryobacteraceae | g__Bryobacter |  |
| EF035064 | Bacteria | p__Proteobacteria | c__Alphaproteobacteria | o__Rhizobiales | f__Rhizobiaceae | g__Mesorhizobium |  |
| AB249372 | Bacteria | p__Bacteroidota | c__Bacteroidia | o__Sphingobacteriales | f__Sphingobacteriaceae | g__Sphingobacterium | s__Sphingobacterium_daejeonense |
| a600b43cb076265ba0f754b3db0e834a | Bacteria | p__Proteobacteria | c__Gammaproteobacteria | o__Burkholderiales | f__Comamonadaceae | g__Delftia |  |
| a431fec81e3e38dc84704a6fa3171ef1 | Bacteria | p__Firmicutes | c__Bacilli | o__Staphylococcales | f__Staphylococcaceae | g__Staphylococcus |  |
| MJ006-1-barcode37-umi110861bins-ubs-4 | Bacteria | p__Firmicutes | c__Clostridia | o__Peptostreptococcales-Tissierellales | f__Peptostreptococcaceae |  |  |
| RS-GCF-003289665.1-NZ-QMBM01000001.1--2 | Bacteria | p__Proteobacteria | c__Gammaproteobacteria | o__Pseudomonadales | f__Moraxellaceae | g__Acinetobacter |  |
| 041f0277a69e666c973d2654d43bff7a | Bacteria | p__Actinobacteriota | c__Actinobacteria | o__Actinomycetales | f__Actinomycetaceae | g__Flaviflexus |  |
| 33454f9373c2d6c22e057e034eb773b0 | Bacteria | p__Proteobacteria | c__Alphaproteobacteria | o__Rhizobiales | f__Rhizobiales_Incertae_Sedis | g__Bauldia |  |
| 7887f2b00595a07b2c78df9d2d01a8e6 | Bacteria | p__Proteobacteria | c__Gammaproteobacteria | o__Burkholderiales | f__Burkholderiaceae | g__Burkholderia-Caballeronia-Paraburkholderia |  |
| 81ec64ba0904a023707e16ea5d072fb8 | Bacteria | p__Proteobacteria | c__Gammaproteobacteria | o__Burkholderiales | f__SC-I-84 | g__SC-I-84 |  |
| 18679527 | Bacteria | p__Actinobacteriota | c__Actinobacteria | o__Micrococcales | f__Microbacteriaceae |  |  |
| 04934667 | Bacteria | p__Bacteroidota | c__Bacteroidia | o__Flavobacteriales | f__Weeksellaceae | g__Chryseobacterium |  |
| 276aaa56ceb8dbd34f48e51637abad95 | Bacteria | p__Proteobacteria | c__Alphaproteobacteria | o__Elsterales | f__uncultured | g__uncultured |  |
| 2785c9ebc98afb3cfa4136585c557d30 | Bacteria | p__Actinobacteriota | c__Actinobacteria | o__Streptomycetales | f__Streptomycetaceae | g__Streptomyces |  |
| 04950880 | Bacteria | p__Firmicutes | c__Clostridia | o__Lachnospirales | f__Lachnospiraceae |  |  |
| ba341d3624e7a93dc1e273be4518c0f4 | Bacteria | p__Firmicutes | c__Clostridia | o__Clostridiales | f__Clostridiaceae |  |  |
| 3caa297a9c40ca1610a74e6e2dc04a64 | Bacteria | p__Firmicutes | c__Bacilli | o__Paenibacillales | f__Paenibacillaceae | g__Paenibacillus |  |
| X76446 | Bacteria | p__Firmicutes | c__Bacilli | o__Bacillales | f__Bacillaceae | g__Bacillus |  |
| 04962126 | Bacteria | p__Actinobacteriota | c__Actinobacteria | o__Propionibacteriales | f__Nocardioidaceae | g__Nocardioides |  |
| 1e178698f950b36fb33d887c17f0bdfc | Bacteria | p__Chloroflexi | c__Ktedonobacteria | o__Ktedonobacterales | f__Ktedonobacteraceae |  |  |
| 15164376 | Bacteria | p__Actinobacteriota | c__Thermoleophilia | o__Gaiellales | f__uncultured | g__uncultured | s__bacterium_Ellin6517 |
| MJ006-2-barcode66-umi97810bins-ubs-5 | Bacteria | p__Firmicutes | c__Clostridia | o__Oscillospirales | f__Butyricicoccaceae | g__Butyricicoccus | s__uncultured_bacterium |
| 04910402 | Bacteria | p__Proteobacteria | c__Gammaproteobacteria | o__Pseudomonadales | f__Pseudomonadaceae | g__Pseudomonas |  |
| d76e561c51d3d36168d0b953bfe79e55 | Bacteria | p__Firmicutes | c__Clostridia | o__Lachnospirales | f__Lachnospiraceae |  |  |
| fe98247719a9b5e6ffa3c33a7063c07e | Bacteria | p__Bacteroidota | c__Bacteroidia | o__Chitinophagales | f__Chitinophagaceae | g__Segetibacter |  |
| GB-GCA-000529385.1-CBVR010000030.1 | Bacteria | p__Firmicutes | c__Bacilli | o__Lactobacillales | f__Lactobacillaceae | g__Lactobacillus |  |
| e5ab4c74b15e4dc07467d6eb7b0ad999 | Bacteria | p__Actinobacteriota | c__Actinobacteria | o__Propionibacteriales | f__Nocardioidaceae | g__Aeromicrobium |  |
| 2a448d07f336b41204bb1a15b662aab7 | Bacteria | p__Actinobacteriota | c__Acidimicrobiia | o__Microtrichales | f__Iamiaceae | g__Iamia |  |
| a9c828cec30fa2578205e868a812799e | Bacteria | p__Bdellovibrionota | c__Bdellovibrionia | o__Bacteriovoracales | f__Bacteriovoracaceae | g__Bacteriovorax | s__uncultured_bacterium |
| 04933924 | Bacteria | p__Bacteroidota | c__Bacteroidia | o__Chitinophagales | f__Chitinophagaceae | g__Terrimonas |  |
| a296519154c1922d6408f5d16ea355eb | Bacteria | p__Acidobacteriota | c__Acidobacteriae | o__Subgroup_2 | f__Subgroup_2 | g__Subgroup_2 | s__uncultured_forest |
| b59753b4ccdb2ec9c091b62e45696ec4 | Bacteria | p__Proteobacteria | c__Gammaproteobacteria | o__Burkholderiales | f__SC-I-84 | g__SC-I-84 |  |
| AY566291 | Bacteria | p__Actinobacteriota | c__Actinobacteria | o__Micrococcales | f__Microbacteriaceae | g__Microbacterium |  |
| 01e98c39fa174ea8502be714b470db53 | Bacteria | p__Actinobacteriota | c__Actinobacteria | o__Propionibacteriales | f__Nocardioidaceae | g__Nocardioides |  |
| 04943505 | Bacteria | p__Acidobacteriota | c__Acidobacteriae | o__Bryobacterales | f__Bryobacteraceae | g__Bryobacter |  |
| 6a9415bb88ebe999729f43104b9bb60a | Bacteria | p__Bacteroidota | c__Bacteroidia | o__Sphingobacteriales | f__Sphingobacteriaceae | g__Sphingobacterium |  |
| RS-GCF-904846165.1-NZ-CAJGZI010000145.1 | Bacteria | p__Proteobacteria | c__Gammaproteobacteria | o__Pseudomonadales | f__Moraxellaceae | g__Psychrobacter |  |
| 0f223ccac8eff3837e3f3bdfe52d01a8 | Bacteria | p__Proteobacteria | c__Alphaproteobacteria | o__Rhizobiales | f__uncultured | g__uncultured | s__uncultured_Hyphomicrobiaceae |
| 05036785 | Bacteria | p__Firmicutes | c__Bacilli | o__Exiguobacterales | f__Exiguobacteraceae | g__Exiguobacterium |  |
| ac0e09f034605fa7dda6945fff39471f | Bacteria | p__Chloroflexi | c__Chloroflexia | o__Thermomicrobiales | f__JG30-KF-CM45 | g__JG30-KF-CM45 | s__uncultured_Thermomicrobium |
| 16129430 | Bacteria | p__Chloroflexi | c__Chloroflexia | o__Thermomicrobiales | f__JG30-KF-CM45 | g__JG30-KF-CM45 |  |
| f9be036b0a264347b58c8ff049961957 | Bacteria | p__Bacteroidota | c__Bacteroidia | o__Flavobacteriales | f__NS9_marine_group | g__NS9_marine_group |  |
| f1de48436e2a26e1a6f55eb61141a0e5 | Bacteria | p__Actinobacteriota | c__Actinobacteria |  |  |  |  |
| 04185339 | Bacteria | p__Planctomycetota | c__Planctomycetes | o__Planctomycetales | f__uncultured | g__uncultured |  |
| MJ006-2-barcode69-umi34671bins-ubs-9 | Bacteria | p__Firmicutes | c__Bacilli | o__Lactobacillales | f__Aerococcaceae | g__Abiotrophia | s__uncultured_bacterium |
| d60eb7a042dd9661ce3b9f6089222ac6 | Bacteria | p__Proteobacteria | c__Gammaproteobacteria | o__Burkholderiales | f__Comamonadaceae | g__Tepidicella |  |
| 7283ee9e222f4ed5cf681e9df5e896b3 | Bacteria | p__Bacteroidota | c__Bacteroidia | o__Sphingobacteriales | f__Sphingobacteriaceae | g__Sphingobacterium |  |
| 741926e4160f1889f12e002afaed1d41 | Bacteria | p__Chloroflexi | c__OLB14 | o__OLB14 | f__OLB14 | g__OLB14 | s__uncultured_bacterium |
| a3d75d76070adc4492d3c8796332f00f | Bacteria | p__Proteobacteria | c__Alphaproteobacteria | o__Rhizobiales | f__Xanthobacteraceae | g__uncultured |  |
| 76ec575beb7bd00a5f98e7855c4393aa | Bacteria | p__Proteobacteria | c__Gammaproteobacteria | o__Pseudomonadales | f__Moraxellaceae | g__Acinetobacter |  |
| b9ca3e60520d3795e84916642a708d7a | Bacteria | p__Proteobacteria | c__Gammaproteobacteria | o__Pseudomonadales | f__Moraxellaceae | g__Acinetobacter |  |
| DQ100063 | Bacteria | p__Proteobacteria | c__Alphaproteobacteria | o__Rhizobiales | f__Rhizobiaceae | g__Allorhizobium-Neorhizobium-Pararhizobium-Rhizobium |  |
| 8b5e87ee4948f47fceff22311db7760f | Bacteria | p__Firmicutes | c__Bacilli | o__Lactobacillales | f__Enterococcaceae | g__Enterococcus |  |
| 01922951 | Bacteria | p__Actinobacteriota | c__Actinobacteria | o__Pseudonocardiales | f__Pseudonocardiaceae | g__Pseudonocardia |  |
| 4f1a35b1727addd950ee180bc114675f | Bacteria | p__Acidobacteriota | c__Vicinamibacteria | o__Vicinamibacterales | f__Vicinamibacteraceae | g__Vicinamibacteraceae |  |
| G000263355_1 | Bacteria | p__Proteobacteria | c__Gammaproteobacteria | o__Cellvibrionales | f__Cellvibrionaceae | g__Cellvibrio |  |
| 13722919 | Bacteria | p__Gemmatimonadota | c__Gemmatimonadetes | o__Gemmatimonadales | f__Gemmatimonadaceae | g__Gemmatimonas |  |
| cc095ac7ba8ffe2cd31a9cfeb318b2f5 | Bacteria | p__Firmicutes | c__Bacilli | o__Lactobacillales | f__Enterococcaceae | g__Enterococcus |  |
| GB-GCA-016712885.1-JADJQF010000003.1 | Bacteria | p__Chloroflexi | c__Anaerolineae | o__SBR1031 | f__A4b | g__OLB13 |  |
| a2ecd1108a642fe55b527d0dc7a97a2c | Bacteria | p__Gemmatimonadota | c__Gemmatimonadetes | o__Gemmatimonadales | f__Gemmatimonadaceae | g__Gemmatimonas |  |
| 10591ba13d2b94df2b84e3c5d73f9bd3 | Bacteria | p__Firmicutes | c__Clostridia | o__Clostridiales | f__Clostridiaceae | g__Clostridium_sensu_stricto_1 |  |
| 9e7c7f0b3991a90a12bfa5ff3cd3eafc | Bacteria |  |  |  |  |  |  |
| MJ030-2-barcode65-umi23206bins-ubs-29 | Bacteria | p__Verrucomicrobiota | c__Verrucomicrobiae | o__Pedosphaerales | f__Pedosphaeraceae | g__Pedosphaeraceae | s__uncultured_Prosthecobacter |
| MJ006-1-barcode38-umi147565bins-ubs-3 | Bacteria | p__Firmicutes | c__Clostridia | o__Oscillospirales | f__Oscillospiraceae | g__UCG-003 |  |
| GB-GCA-003612395.1-RAYQ01000068.1 | Bacteria | p__Firmicutes | c__Clostridia | o__Lachnospirales | f__Lachnospiraceae |  |  |
| f673202c45e87ee050b833e208bfddaf | Bacteria | p__Proteobacteria | c__Gammaproteobacteria | o__Burkholderiales |  |  |  |
| e2b45a95161519ab3c694ab117cdb9cc | Bacteria | p__Chloroflexi | c__Anaerolineae | o__RBG-13-54-9 | f__RBG-13-54-9 | g__RBG-13-54-9 |  |
| KC505174 | Bacteria | p__Actinobacteriota | c__Actinobacteria | o__Micrococcales | f__Microbacteriaceae |  |  |
| AJ853910 | Bacteria | p__Actinobacteriota | c__Actinobacteria | o__Micrococcales | f__Microbacteriaceae | g__Microbacterium |  |
| G000328565_1 | Bacteria | p__Actinobacteriota | c__Actinobacteria | o__Corynebacteriales | f__Mycobacteriaceae | g__Mycobacterium |  |
| 1a0a12f2470bdbfb1b99e2dde842a267 | Bacteria | p__Chloroflexi | c__Anaerolineae | o__Caldilineales | f__Caldilineaceae | g__uncultured | s__metagenome |
| 04908507 | Bacteria | p__Proteobacteria | c__Alphaproteobacteria | o__Rhizobiales | f__Beijerinckiaceae | g__Microvirga |  |
| 93ae7384f7ccc70753a63921afc62ab9 | Bacteria |  |  |  |  |  |  |
| 05037530 | Bacteria | p__Chloroflexi | c__KD4-96 | o__KD4-96 | f__KD4-96 | g__KD4-96 |  |
| JX986958 | Bacteria | p__Proteobacteria | c__Alphaproteobacteria | o__Zavarziniales | f__Zavarziniaceae | g__Zavarzinia | s__Zavarzinia_compransoris |
| 523c0aaeec526f7f9b41a54facd1ceba | Bacteria | p__Actinobacteriota | c__Actinobacteria | o__Micrococcales | f__Microbacteriaceae | g__Microbacterium |  |
| 11023007 | Bacteria | p__Bacteroidota | c__Bacteroidia | o__Bacteroidales | f__Muribaculaceae | g__Muribaculaceae | s__uncultured_bacterium |
| 93f52dcd00cbbc7cd8fe3543dec932ac | Bacteria | p__Proteobacteria | c__Alphaproteobacteria | o__Rhizobiales | f__Beijerinckiaceae |  |  |
| RS-GCF-015554445.1-NZ-JADNIN010000006.1 | Bacteria | p__Bacteroidota | c__Bacteroidia | o__Bacteroidales | f__Bacteroidaceae | g__Bacteroides |  |
| 2ff499d2d9b6a83b533a085e0933aa4b | Bacteria | p__Actinobacteriota | c__Actinobacteria | o__Corynebacteriales | f__Nocardiaceae | g__Rhodococcus |  |
| b9c3cf26fb70b65d0c1d61d9387beb71 | Bacteria | p__Actinobacteriota | c__Actinobacteria | o__Frankiales | f__Acidothermaceae | g__Acidothermus | s__Thermomonosporaceae_bacterium |
| ec5f64a72fb88332d0855251c67e5582 | Bacteria | p__Firmicutes | c__Bacilli | o__Bacillales | f__Bacillaceae | g__Bacillus |  |
| 871a9c81fc5c999a6650473aa9805052 | Bacteria | p__Bacteroidota | c__Bacteroidia | o__Sphingobacteriales | f__AKYH767 | g__AKYH767 | s__uncultured_bacterium |
| ba2c32e3ef183eceec3587fa7c79f2e3 | Bacteria | p__Chloroflexi | c__Chloroflexia | o__Chloroflexales | f__Roseiflexaceae | g__Kouleothrix | s__uncultured_bacterium |
| 962156f707e0d92acbd0c0beee408666 | Bacteria | p__Proteobacteria | c__Gammaproteobacteria | o__Burkholderiales | f__Oxalobacteraceae |  |  |
| f52d3eb4ff67587fba904c0290c14e91 | Bacteria | p__MBNT15 | c__MBNT15 | o__MBNT15 | f__MBNT15 | g__MBNT15 | s__uncultured_Syntrophaceae |
| 2e31ee1199768821faf06664fb49bafd | Bacteria | p__Proteobacteria | c__Gammaproteobacteria | o__Burkholderiales | f__Burkholderiaceae | g__Ralstonia |  |
| ea62c4c20dd28efbe0cde05dafa1085e | Bacteria | p__Actinobacteriota | c__Thermoleophilia | o__Gaiellales | f__Gaiellaceae | g__Gaiella |  |
| f9dd0fddd44b1d8f2fab19e6e11d240d | Bacteria | p__Proteobacteria | c__Gammaproteobacteria | o__Pseudomonadales | f__Pseudomonadaceae | g__Pseudomonas |  |
| 2f76f6ad8ff2bff7365d66f8cf67db0f | Bacteria | p__Acidobacteriota | c__Acidobacteriae | o__Acidobacteriales | f__Acidobacteriaceae_(Subgroup_1) | g__Granulicella | s__bacterium_enrichment |
| 1b66ba777b54fb627d6c3ac5e90b6c61 | Bacteria | p__Actinobacteriota | c__Actinobacteria | o__Propionibacteriales | f__Nocardioidaceae | g__Aeromicrobium |  |
| MJ034-2-barcode71-umi4558bins-ubs-65 | Bacteria | p__Bacteroidota | c__Bacteroidia | o__Chitinophagales | f__Chitinophagaceae | g__Terrimonas |  |
| a7d21f03bd22d101793d6ec1867a7662 | Bacteria | p__Acidobacteriota | c__Acidobacteriae | o__Acidobacteriales | f__Acidobacteriaceae_(Subgroup_1) | g__Granulicella |  |
| 14800455 | Bacteria | p__Chloroflexi | c__Ktedonobacteria | o__Ktedonobacterales | f__JG30-KF-AS9 | g__JG30-KF-AS9 | s__uncultured_bacterium |
| 04957533 | Bacteria | p__Proteobacteria | c__Gammaproteobacteria | o__Burkholderiales | f__Oxalobacteraceae | g__Noviherbaspirillum |  |
| 689d1c4d79c3bb58f6a2d4ce81fbbe2a | Bacteria | p__Firmicutes | c__Bacilli | o__Lactobacillales | f__Lactobacillaceae | g__Lactobacillus |  |
| 1a65f581c64b267e1ee49fc1f456b90b | Bacteria | p__Actinobacteriota | c__Actinobacteria | o__Propionibacteriales | f__Propionibacteriaceae | g__Microlunatus |  |
| bc9e6eb0ca9902fdaae6ad73a88569f2 | Bacteria | p__Proteobacteria | c__Gammaproteobacteria | o__Pseudomonadales | f__Pseudomonadaceae | g__Pseudomonas |  |
| f669c61e91ed5d34db0e2fc6e6ecc7af | Bacteria | p__Proteobacteria | c__Gammaproteobacteria | o__Enterobacterales |  |  |  |
| RS-GCF-001034005.1-NZ-KQ089628.1--5 | Bacteria | p__Proteobacteria | c__Gammaproteobacteria | o__Enterobacterales | f__Enterobacteriaceae | g__Klebsiella |  |
| 00286561 | Bacteria | p__Acidobacteriota | c__Acidobacteriae | o__Subgroup_2 | f__Subgroup_2 | g__Subgroup_2 |  |
| e646538b63e86a52ee57f3469d3779ad | Bacteria | p__Actinobacteriota | c__Actinobacteria | o__Micrococcales | f__Dermabacteraceae | g__Brachybacterium |  |
| 08445908 | Bacteria | p__Proteobacteria | c__Alphaproteobacteria | o__Rhizobiales | f__Beijerinckiaceae | g__1174-901-12 |  |
| a9fa9e9858a6b2174b39170d4ce19e53 | Bacteria | p__Proteobacteria | c__Gammaproteobacteria | o__Burkholderiales | f__Nitrosomonadaceae | g__Ellin6067 |  |
| e3307817241be4ea4d6c045ecfc7a461 | Bacteria | p__Bacteroidota | c__Bacteroidia | o__Bacteroidales | f__Bacteroidaceae | g__Bacteroides |  |
| 24449a19c37a0b9399fb0c0dcd259eaa | Bacteria | p__Acidobacteriota | c__Acidobacteriae | o__Acidobacteriales | f__Acidobacteriaceae_(Subgroup_1) | g__Bryocella | s__uncultured_bacterium |
| 14958526 | Bacteria |  |  |  |  |  |  |
| 74b8aae009f62a3c59cc4439cdfa743b | Bacteria | p__Proteobacteria | c__Gammaproteobacteria | o__Burkholderiales | f__Nitrosomonadaceae | g__Ellin6067 |  |
| GQ456228 | Bacteria | p__Actinobacteriota | c__Coriobacteriia | o__Coriobacteriales | f__Eggerthellaceae | g__Parvibacter |  |
| RS-GCF-002165335.1-NZ-NHRQ01000040.1 | Bacteria | p__Proteobacteria | c__Gammaproteobacteria | o__Pseudomonadales | f__Moraxellaceae | g__Acinetobacter |  |
| GB-GCA-902636645.1-CACPSA010000106.1 | Bacteria | p__Proteobacteria | c__Gammaproteobacteria | o__Pseudomonadales | f__Pseudomonadaceae | g__Pseudomonas |  |
| G004801375_1 | Bacteria | p__Bacteroidota | c__Bacteroidia | o__Flavobacteriales | f__Flavobacteriaceae | g__Flavobacterium |  |
| ba7b4d469e7e1d1fc03f63633c1fd4ee | Bacteria | p__Actinobacteriota | c__Actinobacteria | o__Corynebacteriales | f__Tsukamurellaceae | g__Tsukamurella |  |
| MJ034-1-barcode39-umi23729bins-ubs-6 | Bacteria | p__Acidobacteriota | c__Acidobacteriae | o__Acidobacteriales | f__Koribacteraceae | g__Candidatus_Koribacter |  |
| 4ea104d14cec81695c865433a9ab994c | Bacteria | p__Proteobacteria | c__Gammaproteobacteria | o__Enterobacterales | f__Enterobacteriaceae |  |  |
| AB260026 | Bacteria | p__Bacteroidota | c__Bacteroidia | o__Bacteroidales | f__Bacteroidaceae | g__Bacteroides | s__Bacteroides_coprophilus |
| df90b6487b93d3d2e1af90ab48f6cdaa | Bacteria | p__Proteobacteria | c__Gammaproteobacteria |  |  |  |  |
| 08177295 | Bacteria | p__Proteobacteria | c__Alphaproteobacteria | o__Rhizobiales | f__Xanthobacteraceae |  |  |
| 16181675 | Bacteria | p__Chloroflexi | c__AD3 | o__AD3 | f__AD3 | g__AD3 |  |
| RS-GCF-900078315.1-NZ-FKBS01000024.1 | Bacteria | p__Proteobacteria | c__Gammaproteobacteria | o__Burkholderiales | f__Alcaligenaceae |  |  |
| e862718c68aa3b8753da30c233bea75c | Bacteria | p__Proteobacteria | c__Alphaproteobacteria |  |  |  |  |
| e04e67705ab936afa72f93e46025da73 | Bacteria | p__Firmicutes | c__Clostridia | o__Lachnospirales | f__Lachnospiraceae |  |  |
| 03052375 | Bacteria | p__Myxococcota | c__Polyangia | o__Polyangiales | f__Polyangiaceae | g__Pajaroellobacter | s__uncultured_proteobacterium |
| 18689232 | Bacteria | p__Actinobacteriota | c__Actinobacteria | o__Frankiales | f__Geodermatophilaceae |  |  |
| 2c98f7c59fd84b0bbff9dcd553b7c009 | Bacteria | p__Firmicutes | c__Bacilli | o__Staphylococcales | f__Staphylococcaceae | g__Staphylococcus | s__Staphylococcus_succinus |
| a7ab8a479c729d4e5f0a1aa55d2728ca | Bacteria | p__Acidobacteriota | c__Vicinamibacteria | o__Vicinamibacterales | f__Vicinamibacteraceae | g__Vicinamibacteraceae |  |
| 04958098 | Bacteria | p__Chloroflexi | c__TK10 | o__TK10 | f__TK10 | g__TK10 |  |
| 08795200 | Bacteria | p__Proteobacteria | c__Gammaproteobacteria | o__Xanthomonadales | f__Xanthomonadaceae |  |  |
| HM103934 | Bacteria | p__Firmicutes | c__Bacilli | o__Staphylococcales | f__Gemellaceae | g__Gemella |  |
| 2f347864c6eeaca195527b6d4d59ad70 | Bacteria | p__Firmicutes | c__Bacilli | o__Bacillales | f__Marinococcaceae | g__Alteribacillus |  |
| b1a84b9c5293ac239bbde44649d2f49a | Bacteria | p__Actinobacteriota | c__Thermoleophilia | o__Solirubrobacterales | f__Solirubrobacteraceae |  |  |
| RS-GCF-002161595.1-NZ-NFID01000035.1 | Bacteria | p__Firmicutes | c__Clostridia | o__Oscillospirales | f__Ruminococcaceae | g__Fournierella | s__uncultured_organism |
| df9ad9b26cd7bc82b66a7e897b8dcc18 | Bacteria | p__Bacteroidota | c__Bacteroidia | o__Bacteroidales | f__Prevotellaceae | g__Prevotellaceae_UCG-003 |  |
| 08203388 | Bacteria | p__Proteobacteria | c__Alphaproteobacteria | o__Rhizobiales | f__Beijerinckiaceae | g__Bosea |  |
| d2d0cdc3d69dc8c7dbfda16a601d53d3 | Bacteria | p__Firmicutes | c__Bacilli | o__Erysipelotrichales | f__Erysipelotrichaceae | g__Faecalibaculum | s__uncultured_bacterium |
| e395ed5fac66ab580065e754a1c58106 | Bacteria | p__Acidobacteriota | c__Acidobacteriae | o__Acidobacteriales | f__Acidobacteriaceae_(Subgroup_1) | g__Acidipila |  |
| 04935883 | Bacteria | p__Bacteroidota | c__Bacteroidia | o__Cytophagales | f__Hymenobacteraceae | g__Adhaeribacter |  |
| 05035061 | Bacteria | p__Firmicutes | c__Bacilli | o__Erysipelotrichales | f__Erysipelotrichaceae | g__Ileibacterium | s__Ileibacterium_valens |
| ff156883b5d4376fa56130947e11d3ec | Bacteria | p__Proteobacteria | c__Gammaproteobacteria | o__Enterobacterales |  |  |  |
| EU169581 | Bacteria | p__Proteobacteria | c__Alphaproteobacteria | o__Rhizobiales | f__Rhizobiaceae |  |  |
| DQ471331 | Bacteria | p__Proteobacteria | c__Alphaproteobacteria | o__Rhizobiales | f__Beijerinckiaceae | g__Methylobacterium-Methylorubrum | s__Methylobacterium_jeotgali |
| 1ded623fd65f0e9958b7a1ac547be81a | Bacteria | p__Proteobacteria | c__Alphaproteobacteria | o__Sphingomonadales | f__Sphingomonadaceae | g__Novosphingobium |  |
| 0884937a8ee75bb93a71718a72bd0d92 | Bacteria | p__Bacteroidota | c__Bacteroidia | o__Bacteroidales | f__Dysgonomonadaceae | g__Proteiniphilum |  |
| MJ020-1-barcode31-umi109023bins-ubs-3 | Bacteria | p__Bacteroidota | c__Bacteroidia | o__Chitinophagales | f__Chitinophagaceae | g__Segetibacter | s__Segetibacter_aerophilus |
| 08448056 | Bacteria | p__Proteobacteria | c__Alphaproteobacteria | o__Rhizobiales | f__Beijerinckiaceae | g__1174-901-12 | s__uncultured_bacterium |
| 8aa33c4e36282d9b90804af34cf80b6e | Bacteria | p__Proteobacteria | c__Gammaproteobacteria | o__Xanthomonadales | f__Rhodanobacteraceae | g__Rhodanobacter |  |
| 8229665b1ff07d90efc9e55e511bed27 | Bacteria | p__Proteobacteria | c__Gammaproteobacteria | o__Burkholderiales | f__Nitrosomonadaceae | g__Ellin6067 |  |
| caa1c45dacd2b80d492b655b27eaff62 | Bacteria | p__Actinobacteriota | c__Actinobacteria | o__Propionibacteriales | f__Propionibacteriaceae | g__Cutibacterium |  |
| MJ006-1-barcode28-umi92548bins-ubs-5 | Bacteria | p__Firmicutes | c__Bacilli | o__Staphylococcales | f__Staphylococcaceae | g__Staphylococcus |  |
| 04875812 | Bacteria | p__Proteobacteria | c__Gammaproteobacteria | o__Pseudomonadales | f__Pseudomonadaceae | g__Pseudomonas |  |
| 3d4c7ab05ae6be1e1a28777782dfce45 | Bacteria | p__Proteobacteria | c__Gammaproteobacteria | o__Enterobacterales | f__Enterobacteriaceae |  |  |
| G000779535_1 | Bacteria | p__Proteobacteria | c__Gammaproteobacteria | o__Enterobacterales | f__Enterobacteriaceae | g__Escherichia-Shigella |  |
| MJ030-2-barcode65-umi33329bins-ubs-21 | Bacteria | p__Proteobacteria | c__Gammaproteobacteria | o__Burkholderiales | f__Comamonadaceae |  |  |
| 16922200 | Bacteria | p__Firmicutes | c__Clostridia | o__Lachnospirales | f__Lachnospiraceae | g__GCA-900066575 | s__uncultured_bacterium |
| a0ba692483cd45d9a3d05f2e47eef374 | Bacteria | p__Proteobacteria | c__Alphaproteobacteria | o__Reyranellales | f__Reyranellaceae | g__Reyranella |  |
| MJ030-2-barcode59-umi75942bins-ubs-8 | Bacteria | p__Bacteroidota | c__Bacteroidia | o__Cytophagales | f__Microscillaceae | g__OLB12 |  |
| 0da5ae3778cd03e173335794b036e364 | Bacteria | p__Firmicutes | c__Clostridia | o__Oscillospirales | f__Oscillospiraceae | g__Colidextribacter |  |
| 346e0883cc4ec693ba287fee8dcea312 | Bacteria | p__Actinobacteriota | c__Thermoleophilia | o__Solirubrobacterales |  |  |  |
| MJ031-1-barcode30-umi27067bins-ubs-12 | Bacteria | p__Acidobacteriota | c__Acidobacteriae | o__Acidobacteriales |  |  |  |
| 498eaf64fd2ae4b413b8ea4bf8658c62 | Bacteria | p__Actinobacteriota | c__Acidimicrobiia | o__IMCC26256 | f__IMCC26256 | g__IMCC26256 | s__uncultured_Cellulomonadaceae |
| 6d59790899176581d1d6631007c5c884 | Bacteria | p__Proteobacteria | c__Gammaproteobacteria | o__Xanthomonadales | f__Rhodanobacteraceae |  |  |
| AM292800 | Bacteria | p__Bacteroidota | c__Bacteroidia | o__Flavobacteriales | f__Flavobacteriaceae | g__Flavobacterium | s__Flavobacterium_ceti |
| 16699640 | Bacteria | p__Firmicutes | c__Clostridia | o__Lachnospirales | f__Lachnospiraceae |  |  |
| MJ031-1-barcode42-umi20695bins-ubs-18 | Bacteria | p__Proteobacteria | c__Gammaproteobacteria | o__Burkholderiales | f__Methylophilaceae | g__Methylotenera |  |
| GB-GCA-018262715.1-JADFDS010000042.1 | Bacteria | p__Bacteroidota | c__Bacteroidia | o__Bacteroidales | f__Bacteroidetes_vadinHA17 | g__Bacteroidetes_vadinHA17 | s__uncultured_bacterium |
| RS-GCF-000474175.2-NZ-BATN01000197.1 | Bacteria | p__Proteobacteria | c__Alphaproteobacteria | o__Sphingomonadales | f__Sphingomonadaceae | g__Sphingobium |  |
| 7cd333bd55784e2be10faa67c998a3a4 | Bacteria | p__Actinobacteriota | c__Actinobacteria | o__Micrococcales | f__Micrococcaceae | g__Glutamicibacter |  |
| 02240669 | Bacteria | p__Chloroflexi | c__KD4-96 | o__KD4-96 | f__KD4-96 | g__KD4-96 |  |
| a45a5664a017de1db9c55f7f91a56129 | Bacteria | p__Proteobacteria | c__Gammaproteobacteria | o__Burkholderiales | f__Comamonadaceae |  |  |
| b24c067663a1965fede17b9de15f63b0 | Bacteria | p__Firmicutes | c__Clostridia | o__Lachnospirales | f__Lachnospiraceae |  |  |
| GB-GCA-000466885.2-AVFH02000268.1 | Bacteria | p__Firmicutes | c__Bacilli | o__Lactobacillales | f__Lactobacillaceae | g__Lactobacillus |  |
| DQ870754 | Bacteria | p__Firmicutes | c__Bacilli | o__Bacillales | f__Planococcaceae |  |  |
| KR780024 | Bacteria | p__Proteobacteria | c__Alphaproteobacteria | o__Sphingomonadales | f__Sphingomonadaceae | g__Sphingomonas |  |
| 38d557850fc5da3ad51f657b7837bc04 | Bacteria | p__Bacteroidota | c__Bacteroidia | o__Sphingobacteriales | f__Sphingobacteriaceae | g__Sphingobacterium |  |
| f388ae1b3c45e1abb482a1a880ce0f27 | Bacteria | p__Actinobacteriota | c__Rubrobacteria | o__Rubrobacterales | f__Rubrobacteriaceae | g__Rubrobacter |  |
| 04196978 | Bacteria |  |  |  |  |  |  |
| cbe36ddc7405269313b57d0a1fea6c75 | Bacteria | p__Actinobacteriota | c__Actinobacteria | o__Corynebacteriales | f__Corynebacteriaceae | g__Corynebacterium |  |
| MJ030-2-barcode58-umi111613bins-ubs-4 | Bacteria | p__Bacteroidota | c__Bacteroidia | o__Cytophagales | f__Microscillaceae | g__OLB12 |  |
| G900539055_1 | Bacteria | p__Firmicutes | c__Clostridia | o__Lachnospirales | f__Lachnospiraceae | g__[Eubacterium]_ruminantium_group | s__uncultured_bacterium |
| 0dfaa311e9a0b3a75471c7df48d11941 | Bacteria | p__Actinobacteriota | c__Actinobacteria | o__Corynebacteriales | f__Corynebacteriaceae | g__Corynebacterium |  |
| fa13ca4b1e88d05d2ee936f964b469a0 | Bacteria | p__Planctomycetota | c__Planctomycetes | o__Gemmatales | f__Gemmataceae | g__uncultured |  |
| 02307366 | Bacteria | p__Proteobacteria | c__Alphaproteobacteria | o__Rhizobiales | f__A0839 | g__A0839 |  |
| 6dad79e274daf7ac8703d540e89ad11b | Bacteria | p__Acidobacteriota | c__Vicinamibacteria | o__Vicinamibacterales | f__uncultured | g__uncultured |  |
| ANAC01000044 | Bacteria | p__Actinobacteriota | c__Actinobacteria | o__Streptosporangiales | f__Nocardiopsaceae | g__Nocardiopsis |  |
| 1751ecce7ab99824fa30e5c56f959d68 | Bacteria | p__Acidobacteriota | c__Blastocatellia | o__Blastocatellales | f__Blastocatellaceae | g__Tellurimicrobium | s__Tellurimicrobium_multivorans |
| MJ030-2-barcode50-umi90957bins-ubs-5 | Bacteria | p__Firmicutes | c__Bacilli | o__Bacillales | f__Bacillaceae | g__Bacillus |  |
| 5f1f12ca89162b005c21f823bfce420d | Bacteria | p__Proteobacteria | c__Gammaproteobacteria | o__Burkholderiales | f__Comamonadaceae | g__Piscinibacter |  |
| KC508872 | Bacteria | p__Proteobacteria | c__Alphaproteobacteria | o__Rhizobiales | f__Xanthobacteraceae | g__Bradyrhizobium |  |
| CP006842 | Bacteria | p__Actinobacteriota | c__Actinobacteria | o__Corynebacteriales | f__Corynebacteriaceae | g__Corynebacterium | s__Corynebacterium_glyciniphilum |
| 1d56100307900e1fd0be8b3bdf7b3b92 | Bacteria | p__Acidobacteriota | c__Vicinamibacteria | o__Vicinamibacterales | f__Vicinamibacteraceae | g__Vicinamibacteraceae |  |
| 66822408cff07013706c330665dabd46 | Bacteria | p__Acidobacteriota | c__Holophagae | o__Subgroup_7 | f__Subgroup_7 | g__Subgroup_7 |  |
| b6232339b1f6b12f492d23214efec456 | Bacteria | p__Chloroflexi | c__Chloroflexia | o__Thermomicrobiales | f__JG30-KF-CM45 | g__JG30-KF-CM45 |  |
| RS-GCF-009857505.1-NZ-WWCP01000028.1 | Bacteria | p__Proteobacteria | c__Gammaproteobacteria | o__Burkholderiales | f__Oxalobacteraceae | g__Duganella |  |
| 04882087 | Bacteria | p__Actinobacteriota | c__Actinobacteria | o__Propionibacteriales | f__Propionibacteriaceae | g__Cutibacterium |  |
| 299cbbc2b9b1bfa3570c8f85acf2556b | Bacteria | p__Actinobacteriota | c__Acidimicrobiia | o__Microtrichales | f__Ilumatobacteraceae | g__uncultured |  |
| af8afc6e1ca5a3d1c46649bf3572277d | Bacteria | p__Patescibacteria | c__Saccharimonadia | o__Saccharimonadales |  |  |  |
| 11015797 | Bacteria | p__Bacteroidota | c__Bacteroidia | o__Bacteroidales | f__Muribaculaceae | g__Muribaculaceae |  |
| RS-GCF-001706115.1-NZ-MDHU01000122.1 | Bacteria | p__Proteobacteria | c__Gammaproteobacteria | o__Pseudomonadales | f__Moraxellaceae | g__Acinetobacter |  |
| 82476ec2822b1d7a7a2da907836030e6 | Bacteria | p__Proteobacteria | c__Alphaproteobacteria | o__Rhizobiales | f__Hyphomicrobiaceae |  |  |
| 310ccc11c68e62a4f67d8456f8c71015 | Bacteria | p__Firmicutes | c__Bacilli | o__Lactobacillales | f__Streptococcaceae | g__Streptococcus |  |
| 26064bd57a55886134d3ad1b3dde654f | Bacteria | p__Proteobacteria | c__Gammaproteobacteria | o__Burkholderiales | f__Oxalobacteraceae | g__Candidatus_Vidania | s__Candidatus_Vidania |
| 065d2ceaa82d3bc3e71c642488e78dc4 | Bacteria | p__Planctomycetota | c__Planctomycetes | o__Gemmatales | f__Gemmataceae | g__uncultured |  |
| 3a73a8d73f9a2d6ba00bfc6d39b84692 | Bacteria | p__Proteobacteria | c__Gammaproteobacteria | o__PLTA13 | f__PLTA13 | g__PLTA13 | s__uncultured_sludge |
| MJ030-2-barcode58-umi63528bins-ubs-8 | Bacteria | p__Proteobacteria | c__Gammaproteobacteria | o__Burkholderiales | f__Comamonadaceae | g__Acidovorax |  |
| 2dfb3712200170a625dc4046eee95f09 | Bacteria | p__Proteobacteria | c__Gammaproteobacteria | o__Burkholderiales | f__Comamonadaceae | g__Delftia |  |
| KX247636 | Bacteria | p__Proteobacteria | c__Alphaproteobacteria | o__Rhizobiales | f__Beijerinckiaceae | g__Microvirga |  |
| 5bb7ecfc84edff08f08d137a47e7d2a9 | Bacteria | p__Acidobacteriota | c__Acidobacteriae | o__Acidobacteriales | f__Acidobacteriaceae_(Subgroup_1) |  |  |
| d11ee077950d20d99238b92e525af9e6 | Bacteria | p__Bacteroidota | c__Bacteroidia | o__Bacteroidales | f__Bacteroidaceae | g__Bacteroides |  |
| e480ced7d6ef9235ee0d3125bd135817 | Bacteria | p__Proteobacteria | c__Gammaproteobacteria | o__Enterobacterales | f__Enterobacteriaceae |  |  |
| RS-GCF-003631695.1-NZ-RAAK01000657.1 | Bacteria | p__Proteobacteria | c__Gammaproteobacteria | o__Pseudomonadales | f__Pseudomonadaceae | g__Pseudomonas |  |
| 96a036675e1bc3e518597b7e73b2ea22 | Bacteria | p__Proteobacteria | c__Alphaproteobacteria | o__Rhizobiales | f__Methyloligellaceae |  |  |
| 22387f25b358502a205a284bd2c80f16 | Bacteria | p__Actinobacteriota | c__Actinobacteria | o__Corynebacteriales | f__Nocardiaceae |  |  |
| 04952770 | Bacteria | p__Actinobacteriota | c__Thermoleophilia | o__Gaiellales | f__Gaiellaceae | g__Gaiella |  |
| GB-GCA-900112995.1-FONU01000001.1 | Bacteria | p__Firmicutes | c__Clostridia | o__Lachnospirales | f__Lachnospiraceae | g__Lachnospira |  |
| 36367fcd887b2c9d7a9491e708381ada | Bacteria | p__Bacteroidota | c__Bacteroidia | o__Bacteroidales | f__Muribaculaceae | g__Muribaculaceae | s__uncultured_bacterium |
| MJ034-1-barcode31-umi3652bins-ubs-125 | Bacteria | p__Proteobacteria | c__Gammaproteobacteria | o__Burkholderiales | f__Comamonadaceae | g__Variovorax |  |
| a64a514dfbb9eb1d11e093394991b7ba | Bacteria | p__Bacteroidota | c__Bacteroidia | o__Sphingobacteriales | f__Sphingobacteriaceae | g__Sphingobacterium |  |
| 59483977a1e95fba719cde412c295b5f | Bacteria | p__Proteobacteria | c__Alphaproteobacteria | o__Rhizobiales | f__Xanthobacteraceae |  |  |
| MJ020-2-barcode59-umi17353bins-ubs-30 | Bacteria | p__Proteobacteria | c__Gammaproteobacteria | o__Burkholderiales | f__Oxalobacteraceae |  |  |
| MJ034-1-barcode40-umi4388bins-ubs-105 | Bacteria | p__Bacteroidota | c__Bacteroidia | o__Chitinophagales | f__Chitinophagaceae |  |  |
| 5901be6099d3b50614bd484e6230399f | Bacteria | p__Proteobacteria | c__Alphaproteobacteria | o__Rhizobiales | f__Xanthobacteraceae |  |  |
| 798c12407f3e5fe64ecba9cea52d281d | Bacteria | p__Gemmatimonadota | c__Gemmatimonadetes | o__Gemmatimonadales | f__Gemmatimonadaceae | g__Gemmatimonas |  |
| ec8682edfed9ef9033da87eae8e8d02b | Bacteria | p__Proteobacteria | c__Alphaproteobacteria | o__Rhizobiales | f__Rhizobiaceae |  |  |
| MK512363 | Bacteria | p__Proteobacteria | c__Gammaproteobacteria | o__Alteromonadales | f__Alteromonadaceae | g__Rheinheimera |  |
| c6b232f0edfbdbc631b569c33daa492f | Bacteria | p__Bacteroidota | c__Bacteroidia | o__Chitinophagales | f__Chitinophagaceae | g__Sediminibacterium |  |
| MJ032-1-barcode26-umi67883bins-ubs-3 | Bacteria | p__Bacteroidota | c__Bacteroidia | o__Flavobacteriales | f__NS9_marine_group | g__NS9_marine_group | s__uncultured_soil |
| adf333611a37b274746d1b2eec40bb3d | Bacteria | p__Firmicutes | c__Bacilli | o__Erysipelotrichales | f__Erysipelotrichaceae | g__Turicibacter |  |
| MJ031-2-barcode69-umi4885bins-ubs-82 | Bacteria | p__Proteobacteria | c__Gammaproteobacteria | o__Enterobacterales | f__Enterobacteriaceae |  |  |
| 54637222bf7398c39bbe2abb9d6eb194 | Bacteria | p__Proteobacteria | c__Gammaproteobacteria | o__Pseudomonadales | f__Moraxellaceae | g__Acinetobacter | s__Acinetobacter_indicus |
| 02efbd038b78cb1fcdc335e40469cddc | Bacteria | p__Firmicutes | c__Bacilli | o__Bacillales |  |  |  |
| 127b70facfbcba787624ecc3c8e71cc3 | Bacteria | p__Proteobacteria | c__Alphaproteobacteria | o__Rhizobiales | f__Beijerinckiaceae | g__Microvirga |  |
| 095a2e6d544d4294e56e714a23ce5b05 | Bacteria | p__Proteobacteria | c__Gammaproteobacteria | o__Alteromonadales | f__uncultured | g__uncultured | s__Idiomarina_sp. |
| 5b4e021af7a77e2e47dfd7e83d5f5b4f | Bacteria | p__Actinobacteriota | c__Actinobacteria | o__Micromonosporales | f__Micromonosporaceae |  |  |
| b4830bb4b3cc8aed1132138caadd94cb | Bacteria | p__Proteobacteria | c__Gammaproteobacteria | o__Burkholderiales | f__Burkholderiaceae | g__Burkholderia-Caballeronia-Paraburkholderia |  |
| c1701403a1bfcdb5aca32d452b326c44 | Bacteria | p__Actinobacteriota | c__Actinobacteria | o__Frankiales | f__Acidothermaceae | g__Acidothermus |  |
| 0bd6957754d5bf8b1ce63e9e27adb61f | Bacteria | p__Proteobacteria | c__Alphaproteobacteria | o__Sphingomonadales | f__Sphingomonadaceae | g__Sphingomonas |  |
| RS-GCF-001429855.1-NZ-LMTA01000001.1 | Bacteria | p__Firmicutes | c__Bacilli | o__Bacillales | f__Bacillaceae | g__Bacillus |  |
| d9d77c413af89c64ca43862c76bcb701 | Bacteria |  |  |  |  |  |  |
| LT622838 | Bacteria | p__Proteobacteria | c__Gammaproteobacteria | o__Xanthomonadales | f__Xanthomonadaceae | g__Stenotrophomonas |  |
| AY162221 | Bacteria | p__Fusobacteriota | c__Fusobacteriia | o__Fusobacteriales | f__Fusobacteriaceae | g__Fusobacterium |  |
| 89a388729197aab3d0fa6244466ada01 | Bacteria | p__Firmicutes | c__Clostridia | o__Clostridia_UCG-014 | f__Clostridia_UCG-014 | g__Clostridia_UCG-014 |  |
| 39c0331f983f365f7d18126f6e56bb0c | Bacteria | p__Proteobacteria | c__Alphaproteobacteria | o__Acetobacterales | f__Acetobacteraceae | g__Roseomonas |  |
| 22c3aee49cbde80fca53326e1a0c6dcb | Bacteria | p__Bacteroidota | c__Bacteroidia | o__Sphingobacteriales | f__Sphingobacteriaceae | g__Sphingobacterium | s__Sphingobacterium_kyonggiense |
| df94ee8baa331d5f6889802503796822 | Bacteria | p__Proteobacteria | c__Gammaproteobacteria | o__Oceanospirillales | f__Hahellaceae | g__Hahella | s__uncultured_bacterium |
| 7ba037625784ffc44d09849e3e9ece92 | Bacteria | p__Firmicutes | c__Negativicutes | o__Veillonellales-Selenomonadales | f__Selenomonadaceae | g__Mitsuokella | s__metagenome |
| AB021392 | Bacteria | p__Proteobacteria | c__Gammaproteobacteria | o__Xanthomonadales | f__Xanthomonadaceae | g__Stenotrophomonas |  |
| 17017223 | Bacteria | p__Firmicutes | c__Clostridia | o__Lachnospirales | f__Lachnospiraceae |  |  |
| 6272675bdb782fd8da2478e681ad216d | Bacteria | p__Proteobacteria | c__Gammaproteobacteria | o__Burkholderiales | f__Nitrosomonadaceae | g__MND1 |  |
| fe09c4126e4750c58830e52944e29a69 | Bacteria | p__Actinobacteriota | c__Actinobacteria | o__Corynebacteriales | f__Corynebacteriaceae | g__Corynebacterium |  |
| 15588000 | Bacteria | p__Firmicutes | c__Clostridia | o__Clostridia_UCG-014 | f__Clostridia_UCG-014 | g__Clostridia_UCG-014 |  |
| 043602db357a90c9b54b793b8838293c | Bacteria | p__Actinobacteriota | c__Actinobacteria | o__Pseudonocardiales | f__Pseudonocardiaceae | g__Pseudonocardia |  |
| AY839869 | Bacteria | p__Proteobacteria | c__Gammaproteobacteria | o__Cellvibrionales | f__Cellvibrionaceae | g__Marinimicrobium |  |
| c04a08d58821630373f46e1cb2763da3 | Bacteria | p__Proteobacteria | c__Alphaproteobacteria | o__Sphingomonadales | f__Sphingomonadaceae | g__Sphingomonas |  |
| 7ceb4b538623350afd0f4a41957a33ef | Bacteria | p__Bacteroidota | c__Bacteroidia | o__Flavobacteriales | f__Weeksellaceae |  |  |
| 960ffcca743cd272645a31f5a1e8b13e | Bacteria | p__Proteobacteria | c__Gammaproteobacteria | o__Pseudomonadales | f__Moraxellaceae | g__Acinetobacter |  |
| cb8f64ffa7a924f48127625a3ef04126 | Bacteria | p__Actinobacteriota | c__Actinobacteria | o__Propionibacteriales | f__Propionibacteriaceae |  |  |
| 8dbf43d2284c60c3444355c9ac03cb05 | Bacteria | p__Proteobacteria | c__Gammaproteobacteria | o__Enterobacterales | f__Enterobacteriaceae |  |  |
| d00bb49497b292fb948b001995355d65 | Bacteria | p__Actinobacteriota | c__Coriobacteriia | o__Coriobacteriales | f__Atopobiaceae | g__Olsenella |  |
| 4f7316e84f641cd2c9e0252739de59e2 | Bacteria | p__Proteobacteria | c__Gammaproteobacteria | o__Burkholderiales | f__Comamonadaceae |  |  |
| 17320729 | Bacteria | p__Chloroflexi | c__Ktedonobacteria | o__Ktedonobacterales | f__Ktedonobacteraceae | g__FCPS473 | s__uncultured_bacterium |
| 86964552f5706a0a2f9c3331074dc544 | Bacteria | p__Proteobacteria | c__Alphaproteobacteria | o__Rhizobiales | f__Rhizobiaceae | g__Allorhizobium-Neorhizobium-Pararhizobium-Rhizobium |  |
| e1597d714fdd5cc950100f4e8444f5e3 | Bacteria | p__Actinobacteriota | c__Thermoleophilia | o__Gaiellales | f__uncultured | g__uncultured |  |
| 2a4315b824658acdec30f7999f57a2ab | Bacteria | p__Bacteroidota | c__Bacteroidia | o__Bacteroidales | f__Bacteroidaceae | g__Bacteroides | s__Bacteroides_graminisolvens |
| 1b45b93a3e1a68fa92c85c26f476e601 | Bacteria | p__Proteobacteria | c__Gammaproteobacteria | o__Burkholderiales | f__Nitrosomonadaceae | g__mle1-7 |  |
| 08733891 | Bacteria | p__Proteobacteria | c__Gammaproteobacteria | o__Pseudomonadales | f__Pseudomonadaceae | g__Pseudomonas |  |
| MJ030-1-barcode42-umi38882bins-ubs-13 | Bacteria | p__Firmicutes | c__Bacilli | o__Bacillales | f__Planococcaceae | g__Sporosarcina |  |
| 0c241a11f0d2465cdbc5416671c150bb | Bacteria | p__Firmicutes | c__Bacilli | o__Lactobacillales | f__Lactobacillaceae | g__Lactobacillus |  |
| bb030e1d1b90a186f5a15fc49eda7070 | Bacteria | p__Firmicutes | c__Clostridia | o__Lachnospirales | f__Lachnospiraceae | g__Blautia |  |
| 05033026 | Bacteria | p__Actinobacteriota | c__Actinobacteria | o__Corynebacteriales | f__Corynebacteriaceae | g__Corynebacterium |  |
| 73366e9cbe459b42f25e85472b7c3fb9 | Bacteria | p__Verrucomicrobiota | c__Verrucomicrobiae | o__Chthoniobacterales | f__Chthoniobacteraceae | g__Chthoniobacter |  |
| 816b4bc1901f81e2752aa006860162bc | Bacteria | p__Acidobacteriota | c__Vicinamibacteria | o__Vicinamibacterales | f__Vicinamibacteraceae | g__Vicinamibacteraceae |  |
| 05030875 | Bacteria | p__Actinobacteriota | c__Actinobacteria | o__Bifidobacteriales | f__Bifidobacteriaceae | g__Bifidobacterium |  |
| MJ009-1-barcode35-umi97798bins-ubs-3 | Bacteria | p__Firmicutes | c__Clostridia | o__Lachnospirales | f__Lachnospiraceae |  |  |
| 11840875 | Bacteria | p__Proteobacteria | c__Alphaproteobacteria | o__Sphingomonadales | f__Sphingomonadaceae | g__Sphingomonas |  |
| RS-GCF-018423175.1-NZ-JAFHEI010000011.1 | Bacteria | p__Proteobacteria | c__Gammaproteobacteria | o__Enterobacterales | f__Enterobacteriaceae | g__Enterobacter |  |
| 04900932 | Bacteria | p__Acidobacteriota | c__Acidobacteriae | o__Acidobacteriales | f__Koribacteraceae | g__Candidatus_Koribacter |  |
| 5dd102728d94bb6e3f26fedd6a36efd8 | Bacteria | p__Actinobacteriota | c__Acidimicrobiia | o__Microtrichales |  |  |  |
| 02948132 | Bacteria | p__Chloroflexi | c__Chloroflexia | o__Chloroflexales | f__Roseiflexaceae | g__uncultured | s__uncultured_bacterium |
| G000403455_1 | Bacteria | p__Firmicutes | c__Clostridia | o__Lachnospirales | f__Lachnospiraceae | g__Lachnoclostridium |  |
| 7321fcbb7fed644db284434a3a1808b4 | Bacteria | p__Acidobacteriota | c__Vicinamibacteria | o__Vicinamibacterales | f__uncultured | g__uncultured | s__uncultured_bacterium |
| 6d93b7bb4fcd365db6d813ac083b4a7a | Bacteria | p__Proteobacteria | c__Gammaproteobacteria | o__Alteromonadales | f__Alteromonadaceae | g__Rheinheimera |  |
| GB-GCA-003426415.1-NNRV01000013.1 | Bacteria | p__Actinobacteriota | c__Actinobacteria | o__Bifidobacteriales | f__Bifidobacteriaceae | g__Gardnerella |  |
| 6a14860495055d11f985e34b7f990b08 | Bacteria | p__Firmicutes | c__Bacilli | o__Erysipelotrichales | f__Erysipelotrichaceae | g__Turicibacter |  |
| 17495826 | Bacteria | p__Chloroflexi | c__TK10 | o__TK10 | f__TK10 | g__TK10 | s__uncultured_Chloroflexus |
| 9664015530253d060c4def994c7320e2 | Bacteria | p__Firmicutes | c__Clostridia | o__Lachnospirales | f__Lachnospiraceae |  |  |
| EF108443 | Bacteria | p__Firmicutes | c__Negativicutes | o__Veillonellales-Selenomonadales | f__Veillonellaceae | g__Veillonella |  |
| 12643330 | Bacteria | p__Campilobacterota | c__Campylobacteria | o__Campylobacterales | f__Helicobacteraceae | g__Helicobacter |  |
| 13680680 | Bacteria | p__Gemmatimonadota | c__Gemmatimonadetes | o__Gemmatimonadales | f__Gemmatimonadaceae |  |  |
| 9db1391672dacdbc2bcd9a35396c5d8b | Bacteria | p__Proteobacteria | c__Gammaproteobacteria | o__Burkholderiales | f__Burkholderiaceae | g__Burkholderia-Caballeronia-Paraburkholderia |  |
| 9a1fd8304d301cd2b677afa1eec84879 | Bacteria | p__Chloroflexi | c__Chloroflexia | o__Thermomicrobiales | f__JG30-KF-CM45 | g__JG30-KF-CM45 |  |
| a0868b53b6e48c3afdea3cbce7c0d69a | Bacteria | p__Proteobacteria | c__Gammaproteobacteria | o__Burkholderiales | f__Comamonadaceae |  |  |
| MJ006-1-barcode34-umi104577bins-ubs-4 | Bacteria | p__Firmicutes | c__Clostridia | o__Oscillospirales | f__Butyricicoccaceae | g__Butyricicoccus |  |
| GB-GCA-910574865.1-CAJTDN010000040.1 | Bacteria | p__Firmicutes | c__Clostridia | o__Lachnospirales | f__Lachnospiraceae |  |  |
| c840e518c198d41aa3f695f4f41237bd | Bacteria | p__Proteobacteria | c__Gammaproteobacteria |  |  |  |  |
| 880d7abbcda4128669cbc2785c863229 | Bacteria | p__Chloroflexi | c__Chloroflexia | o__Thermomicrobiales | f__JG30-KF-CM45 | g__JG30-KF-CM45 |  |
| 02260146 | Bacteria | p__Patescibacteria | c__Saccharimonadia | o__Saccharimonadales | f__Saccharimonadaceae | g__TM7a | s__uncultured_bacterium |
| AJ315677 | Bacteria | p__Proteobacteria | c__Gammaproteobacteria | o__Burkholderiales | f__Rhodocyclaceae | g__Thauera |  |
| 47b6b205a5b6a23cc389fc0159877d4b | Bacteria | p__Myxococcota | c__bacteriap25 | o__bacteriap25 | f__bacteriap25 | g__bacteriap25 |  |
| 39acefa4e4908a869fe6a746711cbaaa | Bacteria | p__Myxococcota | c__Polyangia | o__Polyangiales | f__BIrii41 | g__BIrii41 |  |
| c7fba28d03a5729b27d5c08742a3e895 | Bacteria | p__Planctomycetota | c__Phycisphaerae | o__Tepidisphaerales | f__WD2101_soil_group | g__WD2101_soil_group |  |
| f388e4009264c993b6b66683ba8957a0 | Bacteria | p__Actinobacteriota | c__Actinobacteria | o__Micrococcales |  |  |  |
| 394e94ec7f74e22d8e45d6a3b0264897 | Bacteria | p__Proteobacteria | c__Gammaproteobacteria | o__Pseudomonadales | f__Moraxellaceae | g__Acinetobacter |  |
| MJ034-1-barcode30-umi6643bins-ubs-65 | Bacteria | p__Acidobacteriota | c__Acidobacteriae | o__Acidobacteriales |  |  |  |
| 89085339c2dc0f113a2d364fe662b724 | Bacteria | p__Acidobacteriota | c__Vicinamibacteria | o__Vicinamibacterales | f__Vicinamibacteraceae | g__Vicinamibacteraceae |  |
| RS-GCF-014145665.1-NZ-JACHTD010000037.1 | Bacteria | p__Bacteroidota | c__Bacteroidia | o__Flavobacteriales | f__Flavobacteriaceae | g__Myroides | s__Myroides_odoratus |
| MJ006-1-barcode39-umi233032bins-ubs-3 | Bacteria | p__Firmicutes | c__Clostridia | o__Oscillospirales | f__Ruminococcaceae | g__Subdoligranulum |  |
| 4017ba1ebe0c313866128ab2f1d4a70d | Bacteria | p__Chloroflexi | c__KD4-96 | o__KD4-96 | f__KD4-96 | g__KD4-96 | s__uncultured_Anaerolineaceae |
| 02269326 | Bacteria | p__Firmicutes | c__Clostridia | o__Oscillospirales | f__Oscillospiraceae | g__UCG-005 |  |
| d112b0a3a2900c33538bff7d0e1e0a67 | Bacteria | p__Actinobacteriota | c__Actinobacteria | o__Micrococcales | f__Intrasporangiaceae |  |  |
| 8646d016baa88938ee04ac32f1dbd752 | Bacteria | p__Proteobacteria | c__Alphaproteobacteria | o__Rhizobiales | f__Xanthobacteraceae | g__Rhodoplanes |  |
| 6f35be8721c3b9e7cf7b24a7b1bac533 | Bacteria | p__Firmicutes | c__Clostridia | o__Lachnospirales | f__Lachnospiraceae | g__Acetitomaculum | s__uncultured_rumen |
| 02357120 | Bacteria | p__Firmicutes | c__Clostridia | o__Clostridiales | f__Clostridiaceae | g__Clostridium_sensu_stricto_1 |  |
| ae0275a0369791e1af85d48d30e2c705 | Bacteria | p__Proteobacteria | c__Alphaproteobacteria | o__Azospirillales |  |  |  |
| G900129295_1 | Bacteria | p__Bacteroidota | c__Bacteroidia | o__Chitinophagales | f__Chitinophagaceae | g__Flavisolibacter |  |
| d0ad84b7a19e755d404411440901dcfb | Bacteria | p__Actinobacteriota | c__MB-A2-108 | o__MB-A2-108 | f__MB-A2-108 | g__MB-A2-108 | s__uncultured_Kineosporiaceae |
| 05031437 | Bacteria | p__Actinobacteriota | c__Actinobacteria | o__Micrococcales | f__Microbacteriaceae |  |  |
| 63d5c771f1246a697c96bf8f00a8962e | Bacteria | p__Proteobacteria | c__Gammaproteobacteria | o__Burkholderiales | f__Nitrosomonadaceae | g__Ellin6067 |  |
| c9b0103bd31b51ab7ba1f9889eb6d803 | Bacteria | p__Bacteroidota | c__Bacteroidia | o__Bacteroidales | f__Bacteroidaceae | g__Bacteroides | s__Bacteroides_plebeius |
| 2d200ef116f6fe4e44c6dffaeeb1da28 | Bacteria | p__Firmicutes | c__Bacilli | o__Lactobacillales | f__Enterococcaceae | g__Enterococcus |  |
| ecd7261b6767b51830b3b59e5d23cb47 | Bacteria | p__Proteobacteria | c__Gammaproteobacteria | o__Enterobacterales | f__Enterobacteriaceae |  |  |
| MJ030-1-barcode42-umi37699bins-ubs-14 | Bacteria | p__Proteobacteria | c__Gammaproteobacteria | o__Burkholderiales | f__Oxalobacteraceae |  |  |
| 4a459653f4797e67f3dcd428e70fde5d | Bacteria | p__Firmicutes | c__Bacilli | o__Erysipelotrichales | f__Erysipelotrichaceae | g__Ileibacterium | s__Ileibacterium_valens |
| 14338221 | Bacteria | p__Acidobacteriota | c__Vicinamibacteria | o__Vicinamibacterales | f__uncultured | g__uncultured |  |
| G011959565_1 | Bacteria | p__Bacteroidota | c__Bacteroidia | o__Bacteroidales | f__Muribaculaceae | g__Muribaculaceae | s__uncultured_Bacteroidales |
| b22816c4b4ec72538304ba5a3dc51978 | Bacteria | p__Bacteroidota | c__Bacteroidia | o__Cytophagales | f__Hymenobacteraceae | g__Adhaeribacter |  |
| b3fd0b5027b037198c26e16a005652fc | Bacteria | p__Proteobacteria | c__Gammaproteobacteria |  |  |  |  |
| ee3eb38d0d93f977b410cf20218a87f4 | Bacteria | p__Verrucomicrobiota | c__Verrucomicrobiae | o__Chthoniobacterales | f__Chthoniobacteraceae | g__Candidatus_Udaeobacter |  |
| 9d811aa782c1179920d71e84dc0a938a | Bacteria | p__Chloroflexi | c__Anaerolineae | o__SBR1031 | f__SBR1031 | g__SBR1031 |  |
| G004558915_1 | Bacteria | p__Firmicutes | c__Clostridia | o__Christensenellales | f__Christensenellaceae | g__Christensenellaceae_R-7_group | s__human_gut |
| EF076757 | Bacteria | p__Proteobacteria | c__Gammaproteobacteria | o__Alteromonadales | f__Alteromonadaceae | g__Rheinheimera |  |
| a18cf05529103afb5f3d9f9f1abcaa7b | Bacteria | p__Myxococcota | c__Polyangia | o__Polyangiales | f__Phaselicystidaceae | g__Phaselicystis |  |
| 54bcc6662a0d47c2b1a1c7c393e9dc47 | Bacteria | p__Proteobacteria | c__Alphaproteobacteria | o__Rhizobiales | f__Methyloligellaceae | g__uncultured | s__metagenome |
| 04924060 | Bacteria | p__Bacteroidota | c__Bacteroidia | o__Bacteroidales | f__Muribaculaceae | g__Muribaculaceae |  |
| 17456912 | Bacteria | p__Actinobacteriota | c__Thermoleophilia | o__Gaiellales | f__Gaiellaceae | g__Gaiella |  |
| 18147855 | Bacteria | p__Actinobacteriota | c__Thermoleophilia | o__uncultured | f__uncultured | g__uncultured | s__uncultured_bacterium |
| GB-GCA-902646465.1-CACRCV010000084.1 | Bacteria | p__Proteobacteria | c__Gammaproteobacteria | o__Burkholderiales | f__Rhodocyclaceae | g__Methyloversatilis |  |
| 8bb1ecdabc55bebfa27d88b34c5d9679 | Bacteria | p__Firmicutes | c__Clostridia | o__Clostridia | f__Hungateiclostridiaceae | g__Fastidiosipila |  |
| d579f85b14690bec889609db27087a0b | Bacteria |  |  |  |  |  |  |
| 205c97364c443a03962afa8bde26b35f | Bacteria | p__Acidobacteriota | c__Thermoanaerobaculia | o__Thermoanaerobaculales | f__Thermoanaerobaculaceae | g__Subgroup_10 | s__uncultured_prokaryote |
| c8ea027926146786eb60c297d6695040 | Bacteria | p__Actinobacteriota | c__MB-A2-108 | o__MB-A2-108 | f__MB-A2-108 | g__MB-A2-108 | s__uncultured_Catenuloplanes |
| 1d559a826dee13cb2731d449df1705ec | Bacteria | p__Firmicutes | c__Clostridia | o__Lachnospirales | f__Lachnospiraceae | g__Blautia |  |
| d1685eea49b0b3c5fd6c365f52a72984 | Bacteria | p__Proteobacteria | c__Gammaproteobacteria | o__Burkholderiales | f__Comamonadaceae | g__Pelomonas |  |
| 1a629a58d64ab12583dbe3b6208b629d | Bacteria | p__Actinobacteriota | c__Thermoleophilia | o__Solirubrobacterales | f__Solirubrobacteraceae | g__Conexibacter |  |
| 872f0554bb09cbe86254d099ca9bb243 | Bacteria | p__Proteobacteria | c__Gammaproteobacteria | o__Enterobacterales | f__Enterobacteriaceae |  |  |
| 04901113 | Bacteria | p__Proteobacteria | c__Gammaproteobacteria | o__PLTA13 | f__PLTA13 | g__PLTA13 |  |
| RS-GCF-000354695.1-NZ-AOBP01000062.1 | Bacteria | p__Proteobacteria | c__Gammaproteobacteria | o__Aeromonadales | f__Aeromonadaceae | g__Aeromonas |  |
| 05863469 | Bacteria | p__Planctomycetota | c__Phycisphaerae | o__Tepidisphaerales | f__WD2101_soil_group | g__WD2101_soil_group | s__uncultured_eubacterium |
| 16818567 | Bacteria | p__Firmicutes | c__Clostridia | o__Lachnospirales | f__Lachnospiraceae |  |  |
| 392181fc1c6931ee9bb70f764f1ccb04 | Bacteria | p__Proteobacteria | c__Gammaproteobacteria | o__Burkholderiales | f__Nitrosomonadaceae | g__mle1-7 | s__uncultured_Burkholderiales |
| 3316cb66480b6946771e550413fee60a | Bacteria | p__Verrucomicrobiota | c__Verrucomicrobiae | o__Chthoniobacterales | f__Chthoniobacteraceae | g__Chthoniobacter |  |
| fc65168de993b7ef22276ec465133c9f | Bacteria | p__Proteobacteria | c__Gammaproteobacteria | o__Pseudomonadales | f__Pseudomonadaceae | g__Pseudomonas |  |
| 495803663375dfc980764f58ef3f730b | Bacteria | p__Acidobacteriota | c__Vicinamibacteria | o__Vicinamibacterales | f__uncultured | g__uncultured | s__uncultured_Acidobacteria |
| 648b3044668d29b8648f5772e84b8246 | Bacteria | p__Firmicutes | c__Bacilli | o__Erysipelotrichales | f__Erysipelotrichaceae | g__Dubosiella | s__uncultured_bacterium |
| 05030375 | Bacteria | p__Proteobacteria | c__Gammaproteobacteria | o__Burkholderiales | f__Comamonadaceae |  |  |
| 3420ff86978f328d58db6ecc8184f8ab | Bacteria | p__Firmicutes | c__Bacilli | o__Paenibacillales | f__Paenibacillaceae |  |  |
| de6d0d632a489010fb214bae3312cdfd | Bacteria | p__Firmicutes | c__Clostridia | o__Oscillospirales | f__[Eubacterium]_coprostanoligenes_group | g__[Eubacterium]_coprostanoligenes_group |  |
| MJ033-1-barcode47-umi30898bins-ubs-7 | Bacteria | p__Bacteroidota | c__Bacteroidia | o__Chitinophagales | f__Chitinophagaceae |  |  |
| 044992f1361aee6210a9101c55724192 | Bacteria | p__Nitrospirota | c__Nitrospiria | o__Nitrospirales | f__Nitrospiraceae | g__Nitrospira | s__uncultured_Nitrospiraceae |
| 8917669339c30d6df36d35e71baf1dab | Bacteria | p__Actinobacteriota | c__Actinobacteria | o__Micrococcales | f__Microbacteriaceae | g__Microbacterium |  |
| b31456891cf29d7c8e1d85ef1a41a486 | Bacteria | p__Acidobacteriota | c__Thermoanaerobaculia | o__Thermoanaerobaculales | f__Thermoanaerobaculaceae | g__Subgroup_10 | s__metagenome |
| 04947987 | Bacteria | p__Firmicutes | c__Clostridia | o__Lachnospirales | f__Lachnospiraceae | g__Tuzzerella |  |
| LN997852.2 | Bacteria | p__Proteobacteria | c__Gammaproteobacteria | o__Xanthomonadales | f__Xanthomonadales | g__Coralloluteibacterium | s__Coralloluteibacterium_stylophorae |
| RS-GCF-000655275.1-NZ-KK322516.1 | Bacteria | p__Actinobacteriota | c__Actinobacteria | o__Corynebacteriales | f__Mycobacteriaceae | g__Mycobacterium |  |
| 7a6d345ffc19ff54a678d628051aa8d8 | Bacteria | p__Proteobacteria |  |  |  |  |  |
| 65864ddf0a23b4cf7b36fe4e0134c5fe | Bacteria | p__Nitrospirota | c__Nitrospiria | o__Nitrospirales | f__Nitrospiraceae | g__Nitrospira | s__uncultured_Green |
| 0ae9b248a727fdf638763d7e993b6f08 | Bacteria | p__Firmicutes | c__Negativicutes | o__Veillonellales-Selenomonadales | f__Selenomonadaceae | g__Megamonas |  |
| b6d60bc472ff84b8bd197e9239facbf8 | Bacteria | p__Bacteroidota | c__Bacteroidia | o__Flavobacteriales | f__Weeksellaceae | g__Chryseobacterium |  |
| b12e0b919e305a73b831b6b87f11419d | Bacteria | p__Proteobacteria | c__Gammaproteobacteria | o__JG36-TzT-191 | f__JG36-TzT-191 | g__JG36-TzT-191 | s__uncultured_proteobacterium |
| 04909802 | Bacteria | p__Proteobacteria | c__Alphaproteobacteria | o__Rhizobiales | f__Devosiaceae | g__Devosia |  |
| 04952860 | Bacteria | p__Actinobacteriota | c__Thermoleophilia | o__Gaiellales |  |  |  |
| 402b007c68885c8383ab08e06ba07eed | Bacteria |  |  |  |  |  |  |
| b4d9b7aea703c29f4d5a8cc888522b8d | Bacteria | p__Proteobacteria | c__Gammaproteobacteria | o__Enterobacterales | f__Enterobacteriaceae | g__Escherichia-Shigella |  |
| 4ef68f5c3c9cb1aefdf0435de433c9b7 | Bacteria | p__Proteobacteria | c__Gammaproteobacteria | o__Pseudomonadales | f__Moraxellaceae | g__Acinetobacter |  |
| 293a2c6589098ce84f3eae2d645f91e1 | Bacteria | p__Bacteroidota | c__Bacteroidia | o__Bacteroidales | f__Prevotellaceae | g__Prevotella |  |
| LC203064 | Bacteria | p__Actinobacteriota | c__Actinobacteria | o__Micrococcales | f__Microbacteriaceae | g__Schumannella | s__Schumannella_sp. |
| feb0fa733614c627a5a6949e8003c4c2 | Bacteria | p__Actinobacteriota | c__Thermoleophilia | o__Solirubrobacterales | f__Solirubrobacteraceae |  |  |
| 10166140 | Bacteria | p__Bacteroidota | c__Bacteroidia | o__Flavobacteriales | f__Flavobacteriaceae | g__Flavobacterium |  |
| ce2b6ff76ac1bdda8df249043911919e | Bacteria | p__Proteobacteria | c__Gammaproteobacteria | o__R7C24 | f__R7C24 | g__R7C24 | s__uncultured_soil |
| MJ010-2-barcode64-umi104209bins-ubs-6 | Bacteria | p__Proteobacteria | c__Gammaproteobacteria | o__Pseudomonadales | f__Moraxellaceae | g__Acinetobacter |  |
| a147aac5ae53c4bc18c39b69fe963fba | Bacteria | p__Firmicutes | c__Bacilli | o__Alicyclobacillales | f__Alicyclobacillaceae | g__Tumebacillus |  |
| b5daa871ed40ba8ccba4d89a7e41ac5f | Bacteria | p__Firmicutes | c__Bacilli | o__Lactobacillales | f__Enterococcaceae | g__Enterococcus |  |
| dee98794b340da1439451e1fa5cd4f0c | Bacteria | p__Desulfobacterota | c__uncultured | o__uncultured | f__uncultured | g__uncultured |  |
| c240c91a79b057ecce7a33176f3f9d09 | Bacteria | p__Acidobacteriota | c__Blastocatellia | o__Pyrinomonadales | f__Pyrinomonadaceae | g__RB41 |  |
| 050bcd9db6056e7c09a383c1ebada658 | Bacteria | p__Proteobacteria | c__Gammaproteobacteria | o__Xanthomonadales | f__Rhodanobacteraceae | g__Dyella |  |
| MJ006-1-barcode34-umi165105bins-ubs-3 | Bacteria | p__Firmicutes | c__Clostridia | o__Oscillospirales | f__Ruminococcaceae | g__Subdoligranulum |  |
| RS-GCF-014647815.1-NZ-BMPX01000104.1 | Bacteria | p__Actinobacteriota | c__Actinobacteria | o__Micromonosporales | f__Micromonosporaceae |  |  |
| dbcc5254ca1b141109d2e655736281f2 | Bacteria | p__Planctomycetota | c__Phycisphaerae | o__Tepidisphaerales | f__WD2101_soil_group | g__WD2101_soil_group |  |
| RS-GCF-018474885.1-NZ-JADIBL010000030.1 | Bacteria | p__Proteobacteria | c__Gammaproteobacteria | o__Enterobacterales | f__Morganellaceae | g__Morganella |  |
| 07ebffd7740f326363d1f8c552126549 | Bacteria | p__Actinobacteriota | c__Actinobacteria | o__Propionibacteriales | f__Nocardioidaceae | g__Nocardioides |  |
| 04971130 | Bacteria | p__Firmicutes | c__Clostridia | o__Lachnospirales | f__Lachnospiraceae | g__A2 |  |
| 565d461858b55c8253dc8d57e0e8357e | Bacteria | p__Actinobacteriota | c__Rubrobacteria | o__Rubrobacterales | f__Rubrobacteriaceae | g__Rubrobacter | s__metagenome |
| MJ030-1-barcode49-umi40771bins-ubs-13 | Bacteria | p__Firmicutes | c__Bacilli | o__Lactobacillales | f__Enterococcaceae | g__Enterococcus |  |
| 04911087 | Bacteria | p__Proteobacteria | c__Gammaproteobacteria | o__Xanthomonadales | f__Xanthomonadaceae | g__Lysobacter |  |
| MH206611 | Bacteria | p__Bacteroidota | c__Bacteroidia | o__Sphingobacteriales | f__Sphingobacteriaceae |  |  |
| MJ006-2-barcode60-umi204079bins-ubs-3 | Bacteria | p__Firmicutes | c__Clostridia | o__Lachnospirales | f__Lachnospiraceae | g__Lachnospira |  |
| 04957757 | Bacteria | p__Proteobacteria | c__Gammaproteobacteria | o__Burkholderiales | f__Oxalobacteraceae | g__Massilia |  |
| MJ007-1-barcode38-umi14611bins-ubs-17 | Bacteria | p__Bacteroidota | c__Bacteroidia | o__Sphingobacteriales | f__Sphingobacteriaceae | g__Sphingobacterium | s__Sphingobacterium_mizutaii |
| 77e9bf020080948de222dc9595f12c35 | Bacteria | p__Actinobacteriota | c__Thermoleophilia | o__Gaiellales |  |  |  |
| 04963424 | Bacteria | p__Firmicutes | c__Bacilli | o__Lactobacillales | f__Carnobacteriaceae | g__Dolosigranulum |  |
| 59683a352d738c9e3e036ac7c4ef9274 | Bacteria | p__Firmicutes | c__Bacilli | o__Lactobacillales | f__Streptococcaceae | g__Streptococcus |  |
| 04895975 | Bacteria | p__Cyanobacteria | c__Cyanobacteriia | o__Cyanobacteriales | f__Phormidiaceae | g__Tychonema_CCAP_1459-11B |  |
| RS-GCF-008867985.1-NZ-VXLD01000026.1 | Bacteria | p__Proteobacteria | c__Gammaproteobacteria | o__Pseudomonadales | f__Moraxellaceae | g__Acinetobacter |  |
| dd4e931d6bd5436865289976d1a250d9 | Bacteria | p__Actinobacteriota | c__Actinobacteria | o__Micrococcales | f__Micrococcaceae |  |  |
| e86958d5c6549f5edb56d735195e9f18 | Bacteria | p__Proteobacteria | c__Gammaproteobacteria | o__Pseudomonadales | f__Moraxellaceae | g__Acinetobacter |  |
| b220047e1bac7cb60fed25adc602a467 | Bacteria | p__Proteobacteria | c__Gammaproteobacteria | o__Alteromonadales | f__Alteromonadaceae | g__Rheinheimera |  |
| b614bceb3b90e9bcc03591a7d428104a | Bacteria | p__Proteobacteria | c__Alphaproteobacteria | o__Rhizobiales | f__Xanthobacteraceae |  |  |
| RS-GCF-016770975.1-NZ-JAENMS010000030.1 | Bacteria | p__Proteobacteria | c__Gammaproteobacteria | o__Enterobacterales | f__Enterobacteriaceae |  |  |
| 0d4e95a95de7999f2d4ca23e21604b85 | Bacteria | p__Actinobacteriota | c__Coriobacteriia | o__OPB41 | f__OPB41 | g__OPB41 | s__uncultured_soil |
| RS-GCF-900637825.1-NZ-LR134408.1--3 | Bacteria | p__Actinobacteriota | c__Actinobacteria | o__Corynebacteriales | f__Corynebacteriaceae | g__Corynebacterium |  |
| MJ010-1-barcode47-umi82537bins-ubs-8 | Bacteria | p__Bacteroidota | c__Bacteroidia | o__Sphingobacteriales | f__Sphingobacteriaceae | g__Sphingobacterium |  |
| c119f9477494f83a0e5f5de7eb12791c | Bacteria | p__Firmicutes | c__Clostridia | o__Oscillospirales | f__Ruminococcaceae | g__Subdoligranulum |  |
| 16722813 | Bacteria | p__Firmicutes | c__Clostridia | o__Lachnospirales | f__Lachnospiraceae |  |  |
| 2d3648f84ac14afaf4a8a6eecaaac138 | Bacteria | p__Proteobacteria | c__Gammaproteobacteria | o__Pseudomonadales | f__Moraxellaceae | g__Acinetobacter | s__Ambystoma_mexicanum |
| 7a9adf7539e9bc1bcbbc03c0b8cd1156 | Bacteria | p__Actinobacteriota | c__Actinobacteria | o__Micrococcales |  |  |  |
| aa347959a5fa22c6cea999d724e07d88 | Bacteria | p__Firmicutes | c__Bacilli | o__Paenibacillales | f__Paenibacillaceae | g__Paenibacillus |  |
| 55f149f260b2ac944d55bf1ff4fa8814 | Bacteria | p__Proteobacteria | c__Alphaproteobacteria | o__Micropepsales | f__Micropepsaceae | g__uncultured |  |
| 5000ea14e7a5daccfb1814f7f211e34d | Bacteria | p__Proteobacteria | c__Alphaproteobacteria | o__uncultured | f__uncultured | g__uncultured |  |
| 05008715 | Bacteria | p__Acidobacteriota | c__Thermoanaerobaculia | o__Thermoanaerobaculales | f__Thermoanaerobaculaceae | g__Subgroup_10 |  |
| cde36fbe1fcfb944b16f5b92dfeaa9c2 | Bacteria | p__Myxococcota | c__Polyangia | o__Blfdi19 | f__Blfdi19 | g__Blfdi19 | s__uncultured_bacterium |
| ebf6ccb1a5d30f671669c9be3ad23a35 | Bacteria | p__Proteobacteria | c__Gammaproteobacteria | o__Burkholderiales | f__Comamonadaceae | g__Delftia |  |
| 04917639 | Bacteria | p__Bacteroidota | c__Bacteroidia | o__Flavobacteriales | f__Flavobacteriaceae | g__Flavobacterium |  |
| 04902116 | Bacteria | p__Verrucomicrobiota | c__Verrucomicrobiae | o__Pedosphaerales | f__Pedosphaeraceae | g__Pedosphaeraceae |  |
| 8403b9a25fb7286690d3b89dd43e3d70 | Bacteria | p__Bacteroidota | c__Bacteroidia | o__Chitinophagales | f__Chitinophagaceae | g__uncultured |  |
| 7cfd55dd3aad6f97aff78bd813e32c9a | Bacteria | p__Proteobacteria | c__Alphaproteobacteria | o__Rhizobiales | f__Xanthobacteraceae | g__uncultured |  |
| 04944072 | Bacteria | p__Firmicutes | c__Clostridia | o__Oscillospirales | f__Oscillospiraceae | g__NK4A214_group | s__unidentified |
| b74a981a54d9f7003ca3ced03a49c938 | Bacteria | p__Proteobacteria | c__Gammaproteobacteria | o__Burkholderiales | f__Burkholderiaceae |  |  |
| dacbdc336543ecdf74ce43db63878e42 | Bacteria | p__Chloroflexi | c__Chloroflexia | o__Thermomicrobiales | f__JG30-KF-CM45 | g__JG30-KF-CM45 | s__metagenome |
| 70380fbc5a248bdd52792cf4c8289325 | Bacteria | p__Proteobacteria | c__Alphaproteobacteria | o__Rhizobiales | f__Rhizobiaceae | g__Allorhizobium-Neorhizobium-Pararhizobium-Rhizobium |  |
| 02244765 | Bacteria | p__Actinobacteriota | c__Actinobacteria | o__Propionibacteriales | f__Propionibacteriaceae | g__Cutibacterium |  |
| 5dc51a53eeb8627a3ffd66c239c9a962 | Bacteria | p__Actinobacteriota | c__Thermoleophilia | o__Gaiellales | f__uncultured | g__uncultured |  |
| 11b3457d1ee829a245f203fdc1b654a0 | Bacteria | p__Proteobacteria | c__Gammaproteobacteria | o__Burkholderiales | f__Comamonadaceae |  |  |
| 5bba1f57fe223bee26384f05712b9f54 | Bacteria | p__Proteobacteria | c__Gammaproteobacteria | o__Pseudomonadales | f__Pseudomonadaceae | g__Pseudomonas |  |
| 05013932 | Bacteria | p__Bacteroidota | c__Bacteroidia | o__Flavobacteriales | f__Weeksellaceae | g__Chishuiella | s__Empedobacter_sp. |
| 4fa1561246c6a6df81c5ac3f4f39c8be | Bacteria | p__Proteobacteria | c__Alphaproteobacteria | o__uncultured | f__uncultured | g__uncultured |  |
| 0b1df18e4dd4c8fdecebd9990d076db7 | Bacteria | p__Firmicutes | c__Clostridia | o__Lachnospirales | f__Lachnospiraceae | g__A2 | s__uncultured_bacterium |
| 04955766 | Bacteria | p__Firmicutes | c__Clostridia | o__Peptostreptococcales-Tissierellales | f__Peptostreptococcales-Tissierellales | g__Peptoniphilus |  |
| RS-GCF-003010495.1-NZ-CP027570.1--2 | Bacteria | p__Firmicutes | c__Negativicutes | o__Veillonellales-Selenomonadales | f__Veillonellaceae | g__Megasphaera |  |
| 58c428619e92229ac4d36e44b4e84ce2 | Bacteria | p__Bacteroidota | c__Bacteroidia | o__Bacteroidales | f__Marinifilaceae | g__Odoribacter | s__unidentified |
| MJ005-barcode41-umi90208bins-ubs-3 | Bacteria | p__Firmicutes | c__Clostridia | o__Lachnospirales | f__Lachnospiraceae | g__Anaerostipes |  |
| MJ032-1-barcode26-umi44855bins-ubs-7 | Bacteria | p__Proteobacteria | c__Gammaproteobacteria | o__Burkholderiales | f__Oxalobacteraceae | g__Massilia |  |
| 00205075 | Bacteria | p__Chloroflexi | c__Anaerolineae | o__Anaerolineales | f__Anaerolineaceae | g__UTCFX1 |  |
| ebd945058e6e013027f74dad0b074da4 | Bacteria | p__Actinobacteriota | c__Acidimicrobiia | o__Microtrichales | f__Microtrichaceae | g__IMCC26207 |  |
| 514ac01040de16829b98d13c06c90a33 | Bacteria | p__Acidobacteriota | c__Acidobacteriae | o__Acidobacteriales | f__uncultured | g__uncultured |  |
| LN832063 | Bacteria | p__Proteobacteria | c__Alphaproteobacteria | o__Rhizobiales | f__Rhizobiaceae | g__Allorhizobium-Neorhizobium-Pararhizobium-Rhizobium |  |
| 19947471 | Bacteria | p__Firmicutes | c__Clostridia | o__Oscillospirales | f__Oscillospiraceae |  |  |
| 05013281 | Bacteria | p__Bacteroidota | c__Bacteroidia | o__Chitinophagales | f__Chitinophagaceae |  |  |
| MJ010-1-barcode26-umi110768bins-ubs-5 | Bacteria | p__Proteobacteria | c__Alphaproteobacteria | o__Rhizobiales | f__Rhizobiales_Incertae_Sedis | g__Nordella | s__uncultured_Phyllobacteriaceae |
| e8743d928102868c64df38827c63ddf5 | Bacteria | p__Actinobacteriota | c__Actinobacteria | o__Propionibacteriales | f__Propionibacteriaceae | g__Cutibacterium |  |
| 1c3d9f4cd4fc665ec9855ffce3d382d2 | Bacteria | p__Proteobacteria | c__Alphaproteobacteria | o__Rhodobacterales | f__Rhodobacteraceae |  |  |
| 431d3ce74d0c05804f5e9ca0a43ef5ef | Bacteria | p__Actinobacteriota | c__Thermoleophilia | o__Solirubrobacterales | f__Solirubrobacteraceae | g__Conexibacter |  |
| f3d2cbf385453a39512a8b38344da00b | Bacteria | p__Bacteroidota | c__Bacteroidia | o__Sphingobacteriales | f__Sphingobacteriaceae | g__Sphingobacterium | s__Sphingobacterium_spiritivorum |
| cdb568ed9070b0df83dd34ec51a03cf8 | Bacteria | p__Firmicutes | c__Bacilli | o__Staphylococcales | f__Staphylococcaceae | g__Staphylococcus |  |
| 04932067 | Bacteria | p__Deinococcota | c__Deinococci | o__Deinococcales | f__Deinococcaceae | g__Deinococcus |  |
| 7af43438122eab1e2b3be054d03989bb | Bacteria | p__Acidobacteriota | c__Acidobacteriae | o__Acidobacteriales | f__uncultured | g__uncultured | s__bacterium_Ellin6527 |
| ebcf91dbc70d1b6423c3cc06f60f6e7b | Bacteria | p__Acidobacteriota | c__Acidobacteriae | o__Acidobacteriales |  |  |  |
| 5dce968b5e044a7810180026cf9068b9 | Bacteria | p__Bacteroidota | c__Bacteroidia | o__Chitinophagales | f__Chitinophagaceae | g__Terrimonas |  |
| JX504804 | Bacteria | p__Proteobacteria | c__Alphaproteobacteria | o__Rhizobiales | f__Beijerinckiaceae | g__Microvirga |  |
| 25369b23de50f3425d38a0195d904480 | Bacteria | p__Actinobacteriota | c__Thermoleophilia | o__Solirubrobacterales | f__67-14 | g__67-14 |  |
| 0ac86dce52048c9fa9a4bef02c24e5d4 | Bacteria | p__Acidobacteriota | c__Blastocatellia | o__DS-100 | f__DS-100 | g__DS-100 | s__uncultured_bacterium |
| fd01121a3b85d6e5d27f89c3b7a5b1a2 | Bacteria | p__Proteobacteria | c__Alphaproteobacteria | o__Sphingomonadales | f__Sphingomonadaceae |  |  |
| 7a64361e40d18f8fa903c91a9b8396d6 | Bacteria | p__Proteobacteria | c__Gammaproteobacteria | o__Xanthomonadales | f__Xanthomonadaceae | g__Stenotrophomonas |  |
| 91d38bdedeb798869b8837396979698d | Bacteria | p__Bacteroidota | c__Bacteroidia | o__Sphingobacteriales | f__Sphingobacteriaceae | g__Sphingobacterium |  |
| 832eaa665f17557448ffd2166489f62b | Bacteria | p__Fusobacteriota | c__Fusobacteriia | o__Fusobacteriales | f__Fusobacteriaceae | g__Cetobacterium | s__uncultured_bacterium |
| KT003983 | Bacteria | p__Actinobacteriota | c__Actinobacteria | o__Micrococcales | f__Microbacteriaceae | g__Microbacterium |  |
| 6abffef299316a4e5c86645e6c81821e | Bacteria | p__Actinobacteriota | c__Actinobacteria |  |  |  |  |
| G004102775_1 | Bacteria | p__Bacteroidota | c__Bacteroidia | o__Bacteroidales | f__Muribaculaceae | g__Muribaculaceae |  |
| 1e783f0750d02244cc8a75512b7a5f67 | Bacteria | p__Firmicutes | c__Clostridia | o__Peptostreptococcales-Tissierellales | f__Peptostreptococcaceae |  |  |
| 30eb0d3100402e3d09b9c04b0ecad56a | Bacteria | p__Proteobacteria | c__Gammaproteobacteria | o__Xanthomonadales | f__Xanthomonadaceae |  |  |
| 37afbb7d3a0d073d682f0162eb65cdaf | Bacteria | p__Bacteroidota | c__Bacteroidia | o__Bacteroidales | f__Bacteroidetes_vadinHA17 | g__Bacteroidetes_vadinHA17 |  |
| 73000fd0682ad65f7eee6115e3946372 | Bacteria | p__Proteobacteria | c__Gammaproteobacteria | o__Pseudomonadales | f__Moraxellaceae | g__Acinetobacter |  |
| d9b6d6603d4e911bab24f297be09832c | Bacteria |  |  |  |  |  |  |
| 955e495cf0dd1002585a8ddda6633d07 | Bacteria | p__Firmicutes | c__Bacilli | o__Erysipelotrichales | f__Erysipelotrichaceae | g__uncultured | s__uncultured_bacterium |
| MJ034-1-barcode30-umi16217bins-ubs-16 | Bacteria | p__Acidobacteriota | c__Acidobacteriae | o__Solibacterales | f__Solibacteraceae | g__Candidatus_Solibacter |  |
| 04911093 | Bacteria | p__Proteobacteria | c__Gammaproteobacteria | o__Xanthomonadales | f__Xanthomonadaceae | g__Lysobacter |  |
| 04956738 | Bacteria | p__Actinobacteriota | c__Actinobacteria | o__Propionibacteriales | f__Nocardioidaceae | g__Nocardioides |  |
| JX843771 | Bacteria | p__Bacteroidota | c__Bacteroidia | o__Flavobacteriales | f__Weeksellaceae | g__Chryseobacterium |  |
| 04901548 | Bacteria | p__Proteobacteria | c__Gammaproteobacteria | o__Pseudomonadales | f__Moraxellaceae | g__Acinetobacter |  |
| c92db5f4ffb40997d1af94874aeac901 | Bacteria | p__Actinobacteriota | c__Thermoleophilia | o__Gaiellales | f__uncultured | g__uncultured |  |
| fa4f428f7dcdf987d9c36ea8aeeef6c6 | Bacteria | p__Proteobacteria | c__Gammaproteobacteria | o__Enterobacterales | f__Enterobacteriaceae |  |  |
| MK101438 | Bacteria | p__Bacteroidota | c__Bacteroidia | o__Sphingobacteriales | f__Sphingobacteriaceae | g__Sphingobacterium |  |
| 04927978 | Bacteria | p__Proteobacteria | c__Alphaproteobacteria | o__Sphingomonadales | f__Sphingomonadaceae | g__Sphingomonas |  |
| d18aea7ec0b0c741404dad4898e19edd | Bacteria | p__Firmicutes | c__Negativicutes | o__Veillonellales-Selenomonadales | f__Veillonellaceae | g__Dialister |  |
| GB-GCA-000980475.1-LARM01000062.1 | Bacteria | p__Bacteroidota | c__Bacteroidia | o__Bacteroidales | f__Rikenellaceae | g__Alistipes | s__Alistipes_shahii |
| 26663ff64a42068c091a2f653f4db25c | Bacteria | p__Proteobacteria | c__Alphaproteobacteria | o__Acetobacterales | f__Acetobacteraceae | g__uncultured |  |
| RS-GCF-002407065.1-NZ-CP023564.1 | Bacteria | p__Actinobacteriota | c__Actinobacteria | o__Micrococcales |  |  |  |
| e87eb64fbc7e64b7f7672b74f9bb403c | Bacteria | p__Verrucomicrobiota | c__Verrucomicrobiae | o__Opitutales | f__Opitutaceae |  |  |
| fc9ec3375015fd12207cb5a1f093cdd5 | Bacteria | p__Proteobacteria | c__Alphaproteobacteria | o__Rhizobiales |  |  |  |
| 9b37fa36b9884a0cb6e4f93250d70247 | Bacteria | p__Bdellovibrionota | c__Bdellovibrionia | o__Bdellovibrionales | f__Bdellovibrionaceae | g__Bdellovibrio | s__Bdellovibrio_bacteriovorus |
| G000955975_1 | Bacteria | p__Actinobacteriota | c__Actinobacteria | o__Streptomycetales | f__Streptomycetaceae | g__Kitasatospora |  |
| 04993047 | Bacteria | p__Proteobacteria | c__Alphaproteobacteria | o__Rhizobiales | f__Xanthobacteraceae |  |  |
| e41d88594a32cd13ed4a8417bb5ffb67 | Bacteria | p__Chloroflexi | c__Ktedonobacteria | o__Ktedonobacterales | f__Ktedonobacteraceae | g__Ktedonobacter | s__Ktedonobacterales_bacterium |
| edc6f3f3746929e6f487cc727fe28467 | Bacteria | p__Chloroflexi | c__Anaerolineae | o__Caldilineales | f__Caldilineaceae | g__uncultured | s__Chloroflexi_bacterium |
| 14865016 | Bacteria | p__Chloroflexi | c__Chloroflexia | o__Elev-1554 | f__Elev-1554 | g__Elev-1554 | s__uncultured_bacterium |
| 718b29f14b8c6527f18d3a2ec2897e71 | Bacteria | p__Chloroflexi | c__KD4-96 | o__KD4-96 | f__KD4-96 | g__KD4-96 |  |
| f06a705ec8c48a70a20cdf4299e12742 | Bacteria | p__Chloroflexi | c__KD4-96 | o__KD4-96 | f__KD4-96 | g__KD4-96 | s__metagenome |
| AB018046 | Bacteria | p__Actinobacteriota | c__Actinobacteria | o__Streptosporangiales | f__Streptosporangiaceae |  |  |
| MK940276 | Bacteria | p__Proteobacteria | c__Alphaproteobacteria | o__Rhizobiales | f__Rhizobiaceae | g__Allorhizobium-Neorhizobium-Pararhizobium-Rhizobium |  |
| 7e88424ac50205f5ac28cc389869cf33 | Bacteria | p__Actinobacteriota | c__Actinobacteria | o__Streptosporangiales | f__Nocardiopsaceae |  |  |
| 3d691fccfc4de1a36186d6a0a316b3a6 | Bacteria | p__Proteobacteria | c__Gammaproteobacteria | o__Burkholderiales | f__Burkholderiaceae | g__Lautropia | s__metagenome |
| JX502622 | Bacteria | p__Proteobacteria | c__Alphaproteobacteria | o__Rhodospirillales | f__Magnetospirillaceae | g__Magnetospirillum |  |
| RS-GCF-013302025.1-NZ-JAAIPK010000186.1 | Bacteria | p__Firmicutes | c__Clostridia | o__Lachnospirales | f__Lachnospiraceae | g__Blautia |  |
| MJ032-2-barcode60-umi64476bins-ubs-6 | Bacteria | p__Bacteroidota | c__Bacteroidia | o__Flavobacteriales | f__Flavobacteriaceae | g__Flavobacterium |  |
| 5338f2a321fb09922e4f4c0400654129 | Bacteria | p__Proteobacteria | c__Alphaproteobacteria | o__Acetobacterales | f__Acetobacteraceae | g__Roseomonas |  |
| G000242195_5 | Bacteria | p__Firmicutes | c__Bacilli | o__Erysipelotrichales | f__Erysipelotrichaceae | g__[Clostridium]_innocuum_group |  |
| 195cd2dfc8ceaf5b0a561cbd314c06fa | Bacteria | p__Proteobacteria | c__Gammaproteobacteria | o__Burkholderiales | f__Comamonadaceae | g__Alicycliphilus | s__Melaminivora_alkalimesophila |
| a227fc7fb9c0b3ff3443f6538e6ee076 | Bacteria | p__Thermotogota | c__Thermotogae | o__Thermotogales | f__Fervidobacteriaceae | g__Fervidobacterium | s__Fervidobacterium_nodosum |
| 20579754 | Bacteria | p__Firmicutes | c__Clostridia | o__Oscillospirales | f__Oscillospiraceae | g__Oscillibacter | s__uncultured_bacterium |
| cf2087c03af71e9b6b04e4a1e0d2c577 | Bacteria | p__Acidobacteriota | c__Acidobacteriae | o__Acidobacteriales | f__Acidobacteriaceae_(Subgroup_1) |  |  |
| a8135d51a26a3967525ad6b392202c63 | Bacteria | p__Firmicutes | c__Clostridia | o__Oscillospirales | f__Butyricicoccaceae | g__Butyricicoccus |  |
| cac34f64f45c324e1fadd26d5374b4cc | Bacteria | p__Proteobacteria | c__Alphaproteobacteria | o__Rhizobiales | f__Rhizobiaceae |  |  |
| MJ006-2-barcode53-umi27446bins-ubs-11 | Bacteria | p__Firmicutes | c__Bacilli | o__RF39 | f__RF39 | g__RF39 | s__uncultured_bacterium |
| 04918807 | Bacteria | p__Bacteroidota | c__Bacteroidia | o__Bacteroidales | f__Bacteroidaceae | g__Bacteroides |  |
| 0e5f7c866316bac0d184bff05e8b6493 | Bacteria | p__Bacteroidota | c__Bacteroidia | o__Chitinophagales | f__Saprospiraceae | g__uncultured | s__uncultured_soil |
| 48df6a1a97a7049d807d4d0cc61bf00c | Bacteria | p__Actinobacteriota | c__Actinobacteria | o__Micromonosporales | f__Micromonosporaceae | g__Micromonospora |  |
| GB-GCA-016741945.1-JACVCQ010000500.1 | Bacteria | p__Bacteroidota | c__Bacteroidia | o__Chitinophagales | f__Chitinophagaceae | g__Sediminibacterium |  |
| 17328645 | Bacteria | p__Firmicutes | c__Bacilli | o__Paenibacillales | f__Paenibacillaceae | g__Paenibacillus | s__Paenibacillus_turicensis |
| RS-GCF-000582575.1-NZ-AZTE01000018.1 | Bacteria | p__Firmicutes | c__Bacilli | o__Paenibacillales | f__Paenibacillaceae | g__Paenibacillus |  |
| AB245380 | Bacteria | p__Firmicutes | c__Bacilli | o__Bacillales | f__Bacillaceae | g__Bacillus | s__Bacillus_panaciterrae |
| 04971673 | Bacteria | p__Firmicutes | c__Clostridia | o__Lachnospirales | f__Lachnospiraceae |  |  |
| 6ed53e8a771a29aff2f3bac9f778a197 | Bacteria | p__Bacteroidota | c__Bacteroidia | o__Chitinophagales | f__Saprospiraceae | g__uncultured | s__uncultured_Saprospiraceae |
| ACLI01000121 | Bacteria | p__Actinobacteriota | c__Actinobacteria | o__Corynebacteriales | f__Corynebacteriaceae | g__Corynebacterium |  |
| c0e66e566d5a6a2bf505cfaa9f3f5dd7 | Bacteria | p__Proteobacteria | c__Alphaproteobacteria | o__Rhizobiales | f__Beijerinckiaceae | g__Microvirga |  |
| GQ421848 | Bacteria | p__Proteobacteria | c__Alphaproteobacteria | o__Rhizobiales | f__Beijerinckiaceae | g__Microvirga |  |
| 02250914 | Bacteria | p__Proteobacteria | c__Alphaproteobacteria | o__Rhodobacterales | f__Rhodobacteraceae | g__Amaricoccus |  |
| G003150645_1 | Bacteria | p__Firmicutes | c__Clostridia | o__Oscillospirales | f__[Eubacterium]_coprostanoligenes_group | g__[Eubacterium]_coprostanoligenes_group | s__metagenome |
| 05674291 | Bacteria | p__Verrucomicrobiota | c__Verrucomicrobiae | o__Opitutales | f__Opitutaceae | g__Opitutus |  |
| MJ006-2-barcode66-umi156743bins-ubs-3 | Bacteria | p__Firmicutes | c__Clostridia | o__Oscillospirales | f__Oscillospiraceae | g__UCG-005 |  |
| 9a60f30be7e9bda2c26f8690ca9ed498 | Bacteria | p__Bacteroidota | c__Bacteroidia | o__Bacteroidales | f__Bacteroidaceae | g__Bacteroides | s__Bacteroides_coprocola |
| ac01dd2ad5a107423ab1748ef7afaafd | Bacteria | p__Proteobacteria | c__Alphaproteobacteria | o__Rhizobiales | f__Rhizobiaceae |  |  |
| 1808f976a266f5427486c443d9399cf7 | Bacteria | p__Chloroflexi | c__OLB14 | o__OLB14 | f__OLB14 | g__OLB14 |  |
| b0219507d45bc576041f89a88357e636 | Bacteria |  |  |  |  |  |  |
| c9bb145450ca74e5d4f41e6ca30871ad | Bacteria | p__Actinobacteriota | c__Acidimicrobiia | o__uncultured | f__uncultured | g__uncultured | s__uncultured_forest |
| ece56e5de3acb6e10ab7d34f62c44c2c | Bacteria | p__Actinobacteriota | c__Actinobacteria | o__Micrococcales | f__Brevibacteriaceae | g__Brevibacterium |  |
| 11128164 | Bacteria | p__Bacteroidota | c__Bacteroidia | o__Bacteroidales | f__Muribaculaceae | g__Muribaculaceae |  |
| 79dc82127fc9c567fa995ebaa9775cb0 | Bacteria | p__Proteobacteria | c__Alphaproteobacteria | o__Rhizobiales | f__Beijerinckiaceae |  |  |
| 13a5a1189ffe3df03edf97f2a8eaec34 | Bacteria | p__Proteobacteria | c__Alphaproteobacteria | o__Caulobacterales | f__Parvularculaceae | g__Amphiplicatus |  |
| MJ010-2-barcode64-umi127646bins-ubs-3 | Bacteria | p__Firmicutes | c__Bacilli | o__Lactobacillales | f__Enterococcaceae | g__Enterococcus |  |
| 18315441 | Bacteria | p__Actinobacteriota | c__Actinobacteria | o__Propionibacteriales | f__Nocardioidaceae | g__Nocardioides |  |
| RS-GCF-002029255.1-NZ-MZGT01000173.1 | Bacteria | p__Firmicutes | c__Clostridia | o__Clostridiales | f__Clostridiaceae | g__Clostridium_sensu_stricto_1 |  |
| G900112535_1 | Bacteria | p__Firmicutes | c__Bacilli | o__Bacillales | f__Bacillaceae | g__Bacillus |  |
| EF012357 | Bacteria | p__Proteobacteria | c__Alphaproteobacteria | o__Rhizobiales | f__Devosiaceae | g__Devosia |  |
| 17970455 | Bacteria | p__Chloroflexi | c__Ktedonobacteria | o__Ktedonobacterales | f__Ktedonobacteraceae | g__HSB_OF53-F07 |  |
| G000438965_1 | Bacteria | p__Actinobacteriota | c__Actinobacteria | o__Frankiales | f__Sporichthyaceae |  |  |
| MJ010-1-barcode30-umi28431bins-ubs-23 | Bacteria | p__Firmicutes | c__Bacilli | o__Lactobacillales | f__Streptococcaceae | g__Lactococcus |  |
| 63cb6955b759249141c7ec0d4ef559a9 | Bacteria | p__Firmicutes | c__Incertae_Sedis | o__DTU014 | f__DTU014 | g__DTU014 |  |
| a439ee80e1bdd407f7c8478a6b292b00 | Bacteria | p__Acidobacteriota | c__Vicinamibacteria | o__Vicinamibacterales | f__Vicinamibacteraceae | g__Vicinamibacteraceae |  |
| 05036908 | Bacteria | p__Firmicutes | c__Bacilli | o__Bacillales | f__Planococcaceae | g__Sporosarcina |  |
| f7d08a60c08ca258ca3e339587f1253a | Bacteria | p__Actinobacteriota | c__Rubrobacteria | o__Rubrobacterales | f__Rubrobacteriaceae | g__Rubrobacter |  |
| 2e0649db8a14ea2aaa50b1e8369d17b9 | Bacteria | p__Proteobacteria | c__Gammaproteobacteria | o__Pseudomonadales | f__Moraxellaceae | g__Acinetobacter |  |
| e76009a60a560114e0b6286787260675 | Bacteria | p__Actinobacteriota | c__Actinobacteria | o__Bifidobacteriales | f__Bifidobacteriaceae | g__Bifidobacterium |  |
| 75e97972ef24e5423a0f62003856dbf5 | Bacteria | p__Proteobacteria | c__Gammaproteobacteria | o__Enterobacterales |  |  |  |
| 81771b3680aed7763d05e775734d6bf0 | Bacteria | p__Proteobacteria | c__Alphaproteobacteria | o__Rhizobiales | f__Devosiaceae | g__Devosia |  |
| 844611f65ebe14c7901bce45efca3d5d | Bacteria | p__Proteobacteria | c__Gammaproteobacteria | o__Burkholderiales |  |  |  |
| 381e9ed26f55d25c04f4e54d383eef63 | Bacteria | p__Gemmatimonadota | c__Gemmatimonadetes | o__Gemmatimonadales | f__Gemmatimonadaceae |  |  |
| 17526773 | Bacteria | p__Acidobacteriota | c__Acidobacteriae | o__Subgroup_13 | f__Subgroup_13 | g__Subgroup_13 | s__uncultured_bacterium |
| 8549bb319a6183b39dadba7172d76325 | Bacteria | p__Actinobacteriota | c__Coriobacteriia | o__Coriobacteriales | f__Atopobiaceae |  |  |
| ad6b549de6dc93223c2b477f829ae26d | Bacteria | p__Proteobacteria | c__Gammaproteobacteria | o__Burkholderiales | f__Comamonadaceae | g__Delftia |  |
| 33df710468f789f17f3dd286d3bb0c45 | Bacteria | p__Firmicutes | c__Bacilli | o__Erysipelotrichales | f__Erysipelotrichaceae | g__Turicibacter | s__uncultured_bacterium |
| 52c90a9cc619027349f148740557fe72 | Bacteria | p__Proteobacteria | c__Gammaproteobacteria | o__Burkholderiales | f__Rhodocyclaceae | g__Thauera |  |
| 04907628 | Bacteria | p__Proteobacteria | c__Alphaproteobacteria | o__Caulobacterales | f__Caulobacteraceae | g__Phenylobacterium |  |
| f7e00098eb02b3693dfaab61b70f9f47 | Bacteria | p__Proteobacteria | c__Gammaproteobacteria | o__Enterobacterales | f__Enterobacteriaceae | g__Escherichia-Shigella |  |
| 04966347 | Bacteria | p__Firmicutes | c__Bacilli | o__Bacillales | f__Planococcaceae | g__Lysinibacillus |  |
| 04957679 | Bacteria | p__Proteobacteria | c__Gammaproteobacteria | o__Burkholderiales | f__Oxalobacteraceae | g__Massilia |  |
| MJ033-1-barcode47-umi10547bins-ubs-28 | Bacteria | p__Bacteroidota | c__Bacteroidia | o__Chitinophagales | f__Chitinophagaceae |  |  |
| KJ150597 | Bacteria | p__Proteobacteria | c__Alphaproteobacteria | o__Sphingomonadales | f__Sphingomonadaceae | g__Altererythrobacter |  |
| 8e348742419e9a4187ac751c22b8ad98 | Bacteria | p__Bacteroidota | c__Bacteroidia | o__Chitinophagales | f__Chitinophagaceae | g__Sediminibacterium | s__Sediminibacterium_sp. |
| 77bf32045d4cb0fcbacdcb8645e3ffc7 | Bacteria | p__Actinobacteriota | c__Thermoleophilia | o__Gaiellales | f__Gaiellaceae | g__Gaiella |  |
| 33b1c2bf8e360e8affc359ae25332947 | Bacteria | p__Acidobacteriota | c__Vicinamibacteria | o__Vicinamibacterales | f__uncultured | g__uncultured |  |
| RS-GCF-002160495.1-NZ-NFKR01000094.1 | Bacteria | p__Firmicutes | c__Bacilli | o__Erysipelotrichales | f__Erysipelatoclostridiaceae | g__Erysipelatoclostridium | s__bacterium_ic1391 |
| a254a713c5402c5db1f87ddbc97b1e74 | Bacteria | p__Proteobacteria | c__Alphaproteobacteria | o__Sphingomonadales | f__Sphingomonadaceae | g__Sphingomonas |  |
| KR349468 | Bacteria | p__Proteobacteria | c__Gammaproteobacteria | o__Burkholderiales | f__Comamonadaceae | g__Variovorax |  |
| KP334257 | Bacteria | p__Acidobacteriota | c__Blastocatellia | o__Blastocatellales | f__Blastocatellaceae | g__Tellurimicrobium | s__Tellurimicrobium_multivorans |
| 04928205 | Bacteria | p__Proteobacteria | c__Alphaproteobacteria | o__Rhizobiales | f__Methyloligellaceae | g__Methyloligellaceae | s__uncultured_bacterium |
| 13248453 | Bacteria | p__Desulfobacterota | c__uncultured | o__uncultured | f__uncultured | g__uncultured |  |
| e3e7b57836305c177c6752bddaa3f531 | Bacteria | p__Cyanobacteria | c__Cyanobacteriia | o__Cyanobacteriales |  |  |  |
| 05035356 | Bacteria | p__Firmicutes | c__Bacilli | o__Staphylococcales | f__Staphylococcaceae | g__Staphylococcus |  |
| 04902572 | Bacteria | p__Verrucomicrobiota | c__Verrucomicrobiae | o__Chthoniobacterales | f__Chthoniobacteraceae | g__Candidatus_Udaeobacter |  |
| 40dd6d0639709526e1d0237707a22276 | Bacteria | p__Actinobacteriota | c__Actinobacteria | o__Frankiales | f__Nakamurellaceae | g__Nakamurella | s__metagenome |
| AB775175 | Bacteria | p__Firmicutes | c__Bacilli | o__Lactobacillales | f__Enterococcaceae | g__Enterococcus |  |
| f758e5802b133578800c49ed50daf60c | Bacteria | p__Acidobacteriota | c__Acidobacteriae | o__Solibacterales | f__Solibacteraceae | g__Candidatus_Solibacter |  |
| 05031167 | Bacteria | p__Actinobacteriota | c__Actinobacteria | o__Frankiales | f__uncultured | g__uncultured |  |
| 08323125 | Bacteria | p__Proteobacteria | c__Alphaproteobacteria | o__Caulobacterales | f__Caulobacteraceae | g__Brevundimonas |  |
| 9f7ad152642d97b03db2eb1a45c47ab8 | Bacteria | p__Bacteroidota | c__Bacteroidia | o__Bacteroidales | f__Prevotellaceae |  |  |
| 08176629 | Bacteria | p__Proteobacteria | c__Alphaproteobacteria | o__Rhizobiales | f__Rhizobiales_Incertae_Sedis | g__Alsobacter |  |
| MJ020-2-barcode52-umi29665bins-ubs-19 | Bacteria | p__Bacteroidota | c__Bacteroidia | o__Cytophagales | f__Microscillaceae | g__OLB12 | s__uncultured_bacterium |
| MJ034-1-barcode30-umi26730bins-ubs-4 | Bacteria | p__Myxococcota | c__bacteriap25 | o__bacteriap25 | f__bacteriap25 | g__bacteriap25 |  |
| 14207767 | Bacteria | p__Acidobacteriota | c__Blastocatellia | o__Pyrinomonadales | f__Pyrinomonadaceae | g__RB41 |  |
| 1b552630bba6057ea8b1dd191d8e7eee | Bacteria | p__Proteobacteria | c__Gammaproteobacteria | o__Pseudomonadales | f__Moraxellaceae | g__Acinetobacter |  |
| 7dabe7d19e4686cdcbb0afef9d9f47d0 | Bacteria | p__Acidobacteriota | c__Blastocatellia | o__DS-100 | f__DS-100 | g__DS-100 |  |
| 01574575 | Bacteria | p__Planctomycetota | c__Planctomycetes | o__Pirellulales | f__Pirellulaceae | g__Pir4_lineage | s__uncultured_Planctomycetaceae |
| 049450047851da7f567441ffcc5c0b18 | Bacteria | p__Actinobacteriota | c__Thermoleophilia | o__Gaiellales | f__uncultured | g__uncultured | s__uncultured_microorganism |
| 282c6bf63ae2c19990676ac12544334f | Bacteria | p__Proteobacteria | c__Gammaproteobacteria | o__Pseudomonadales | f__Moraxellaceae | g__Acinetobacter |  |
| ba3a28b5211a8b2e48cf5cef13503377 | Bacteria | p__Firmicutes | c__Clostridia | o__Lachnospirales | f__Lachnospiraceae |  |  |
| 5f5dcc372ba3ca72d7e44cd45e26ffe1 | Bacteria | p__Actinobacteriota | c__Thermoleophilia | o__Gaiellales | f__uncultured | g__uncultured |  |
| ed502d0552f4e64acaac5c56791478ee | Bacteria | p__Firmicutes | c__Clostridia | o__Clostridiales | f__Clostridiaceae | g__Clostridium_sensu_stricto_1 |  |
| 902a7b36e8947af25cf4b5d9b4718789 | Bacteria | p__Patescibacteria | c__Dojkabacteria | o__Dojkabacteria | f__Dojkabacteria | g__Dojkabacteria |  |
| 5920e8b4facae013992eeb0005a4e807 | Bacteria | p__Bacteroidota | c__Bacteroidia | o__Flavobacteriales | f__Weeksellaceae | g__Chryseobacterium |  |
| 08792725 | Bacteria | p__Proteobacteria | c__Gammaproteobacteria | o__Xanthomonadales | f__Xanthomonadaceae |  |  |
| AY753654 | Bacteria | p__Firmicutes | c__Bacilli | o__Caldalkalibacillales | f__Caldalkalibacillaceae | g__Caldalkalibacillus | s__Caldalkalibacillus_thermarum |
| 947d79d84e18356c1d0ffee5945b37f7 | Bacteria | p__Proteobacteria | c__Alphaproteobacteria | o__Rhizobiales | f__Xanthobacteraceae | g__uncultured |  |
| aadbe8e81340613448e4f55a2765302f | Bacteria | p__Bacteroidota | c__Bacteroidia | o__Bacteroidales | f__Muribaculaceae | g__Muribaculaceae | s__uncultured_Bacteroidales |
| RS-GCF-013166515.1-NZ-JABKKJ010000049.1 | Bacteria | p__Bacteroidota | c__Bacteroidia | o__Bacteroidales | f__Prevotellaceae |  |  |
| 05030490 | Bacteria | p__Proteobacteria | c__Gammaproteobacteria | o__Burkholderiales | f__Oxalobacteraceae | g__Noviherbaspirillum |  |
| 4572787e47c31a2f1b9d83eb8aa77575 | Bacteria | p__Cyanobacteria | c__Cyanobacteriia | o__Cyanobacteriales | f__Phormidiaceae | g__Tychonema_CCAP_1459-11B |  |
| 02360466 | Bacteria | p__Proteobacteria | c__Alphaproteobacteria | o__Rhizobiales | f__Beijerinckiaceae | g__Bosea |  |
| 8b8eb4c08db69e18a0250fdc72d41f9d | Bacteria | p__Proteobacteria | c__Gammaproteobacteria | o__Pseudomonadales | f__Pseudomonadaceae | g__Pseudomonas |  |
| 04911205 | Bacteria | p__Proteobacteria | c__Gammaproteobacteria | o__Xanthomonadales | f__Xanthomonadaceae | g__Lysobacter |  |
| 16129436 | Bacteria | p__Chloroflexi | c__Chloroflexia | o__Thermomicrobiales | f__JG30-KF-CM45 | g__JG30-KF-CM45 | s__uncultured_anaerobic |
| 25fe72e3337eb014c28876ddd8cd879b | Bacteria | p__Proteobacteria | c__Gammaproteobacteria | o__Pseudomonadales | f__Moraxellaceae | g__Acinetobacter | s__Acinetobacter_soli |
| 04908922 | Bacteria | p__Proteobacteria | c__Alphaproteobacteria | o__Rhizobiales | f__Rhizobiaceae |  |  |
| fe847f285bcbc297b0d40cd52cd15589 | Bacteria | p__Firmicutes | c__Bacilli | o__Erysipelotrichales | f__Erysipelotrichaceae | g__Ileibacterium | s__Ileibacterium_valens |
| d2326f5ed019dfee19ec8efe396cdc25 | Bacteria | p__Gemmatimonadota | c__Gemmatimonadetes | o__Gemmatimonadales | f__Gemmatimonadaceae | g__Gemmatimonas | s__uncultured_Gemmatimonas |
| 09d9cbaaa3570bf965a29db96983252a | Bacteria | p__Actinobacteriota | c__Actinobacteria | o__Propionibacteriales | f__Nocardioidaceae | g__Nocardioides |  |
| MJ034-1-barcode40-umi28230bins-ubs-3 | Bacteria | p__Acidobacteriota | c__Acidobacteriae | o__Acidobacteriales | f__uncultured | g__uncultured | s__uncultured_Acidobacteriaceae |
| 26f70074acb731b0963d9795de677d1e | Bacteria | p__Firmicutes | c__Clostridia | o__Clostridiales | f__Clostridiaceae | g__Clostridium_sensu_stricto_1 |  |
| MJ034-1-barcode32-umi26915bins-ubs-5 | Bacteria | p__Proteobacteria | c__Gammaproteobacteria | o__Xanthomonadales | f__Xanthomonadaceae |  |  |
| MJ010-2-barcode71-umi90630bins-ubs-5 | Bacteria | p__Bacteroidota | c__Bacteroidia | o__Chitinophagales | f__Chitinophagaceae | g__Terrimonas |  |
| 3dd77716b0784a0237524afa62e5d260 | Bacteria | p__Acidobacteriota | c__Acidobacteriae | o__Subgroup_2 | f__Subgroup_2 | g__Subgroup_2 | s__uncultured_Holophaga |
| 183644b2c2311a03949d8aa502e09ae1 | Bacteria | p__Proteobacteria | c__Alphaproteobacteria | o__Rhizobiales | f__Hyphomicrobiaceae |  |  |
| MJ020-1-barcode34-umi8675bins-ubs-42 | Bacteria | p__Proteobacteria | c__Gammaproteobacteria | o__Pseudomonadales | f__Moraxellaceae | g__Acinetobacter |  |
| 59a82b19f0d8d4bbf57d3092da93ef45 | Bacteria | p__Firmicutes | c__Bacilli | o__Bacillales | f__Planococcaceae |  |  |
| 05131729 | Bacteria | p__Proteobacteria | c__Gammaproteobacteria | o__Burkholderiales | f__Alcaligenaceae | g__Alcaligenes |  |
| 0c7bc0a249744d0c2bd4a25f6696bd85 | Bacteria | p__Bacteroidota | c__Ignavibacteria | o__Ignavibacteriales | f__PHOS-HE36 | g__PHOS-HE36 |  |
| AJ458473 | Bacteria | p__Proteobacteria | c__Alphaproteobacteria | o__Rhizobiales | f__Beijerinckiaceae |  |  |
| 04958403 | Bacteria | p__Actinobacteriota | c__Actinobacteria | o__Micrococcales | f__Microbacteriaceae |  |  |
| 04960567 | Bacteria | p__Chloroflexi | c__TK10 | o__TK10 | f__TK10 | g__TK10 |  |
| fedbfdedd2d849e28a8ea7053590874e | Bacteria | p__Proteobacteria | c__Gammaproteobacteria | o__Pasteurellales | f__Pasteurellaceae | g__Rodentibacter |  |
| 24dea4784ce58f1be192bf3875588539 | Bacteria | p__Actinobacteriota | c__Actinobacteria | o__Corynebacteriales | f__Nocardiaceae | g__Rhodococcus |  |
| 0f290de1f214201d1fe637cded97a485 | Bacteria | p__Firmicutes | c__Bacilli | o__Lactobacillales | f__Enterococcaceae | g__Enterococcus |  |
| MF363155 | Bacteria | p__Gemmatimonadota | c__Gemmatimonadetes | o__Gemmatimonadales | f__Gemmatimonadaceae | g__Roseisolibacter | s__Roseisolibacter_agri |
| 19295554 | Bacteria | p__Actinobacteriota | c__Actinobacteria | o__Corynebacteriales | f__Corynebacteriaceae | g__Turicella | s__uncultured_bacterium |
| RS-GCF-003610905.1-NZ-MCAQ01000003.1 | Bacteria | p__Bacteroidota | c__Bacteroidia | o__Sphingobacteriales | f__Sphingobacteriaceae | g__Sphingobacterium |  |
| 04957672 | Bacteria | p__Proteobacteria | c__Gammaproteobacteria | o__Burkholderiales | f__Nitrosomonadaceae | g__Nitrosospira |  |
| 04959798 | Bacteria | p__Proteobacteria | c__Gammaproteobacteria | o__Burkholderiales | f__Comamonadaceae | g__Tepidimonas |  |
| f2304a8cbad0494b8f6b30b3bf1329a4 | Bacteria | p__Chloroflexi | c__Anaerolineae | o__Anaerolineales | f__Anaerolineaceae |  |  |
| 14227591 | Bacteria | p__Latescibacterota | c__Latescibacterota | o__Latescibacterota | f__Latescibacterota | g__Latescibacterota | s__metagenome |
| MJ010-1-barcode25-umi54974bins-ubs-12 | Bacteria | p__Proteobacteria | c__Gammaproteobacteria | o__Alteromonadales | f__Alteromonadaceae | g__Rheinheimera | s__uncultured_Rheinheimera |
| 0e35c584c373ed33260e3c455ba6f4dc | Bacteria | p__Firmicutes | c__Bacilli | o__Erysipelotrichales | f__Erysipelatoclostridiaceae | g__Erysipelatoclostridium | s__uncultured_Clostridium |
| DQ191179 | Bacteria | p__Proteobacteria | c__Gammaproteobacteria | o__Xanthomonadales | f__Xanthomonadaceae | g__Lysobacter |  |
| 04994069 | Bacteria | p__Proteobacteria | c__Alphaproteobacteria | o__Caulobacterales | f__Caulobacteraceae |  |  |
| 516a5625bd402e817012c427a100edba | Bacteria | p__Bacteroidota | c__Bacteroidia | o__Bacteroidales | f__Muribaculaceae | g__Muribaculaceae | s__unidentified |
| dc373699eda195f85c53cab4e0bf5cb6 | Bacteria | p__Firmicutes | c__Bacilli | o__Exiguobacterales | f__Exiguobacteraceae | g__Exiguobacterium |  |
| 84ae446423b60b7bc26d17b6ecb679fe | Bacteria | p__Proteobacteria | c__Alphaproteobacteria | o__Rhizobiales | f__Hyphomicrobiaceae |  |  |
| 02247622 | Bacteria | p__Actinobacteriota | c__Actinobacteria | o__Propionibacteriales | f__Nocardioidaceae | g__Nocardioides |  |
| AB681294 | Bacteria | p__Firmicutes | c__Bacilli | o__Lactobacillales | f__Streptococcaceae | g__Lactococcus |  |
| 0ee9bd619edd2580e82fcee3eb903dcd | Bacteria | p__Actinobacteriota | c__Thermoleophilia | o__Gaiellales |  |  |  |
| AJ292759 | Bacteria | p__Bdellovibrionota | c__Bdellovibrionia | o__Bdellovibrionales | f__Bdellovibrionaceae | g__Bdellovibrio | s__Bdellovibrio_bacteriovorus |
| 40c1ae64a80e4c4c2fc4f4a540478593 | Bacteria | p__Chloroflexi | c__KD4-96 | o__KD4-96 | f__KD4-96 | g__KD4-96 |  |
| 04878698 | Bacteria | p__Firmicutes | c__Bacilli | o__Bacillales | f__Bacillaceae | g__Bacillus |  |
| EF591303 | Bacteria | p__Bacteroidota | c__Bacteroidia | o__Flavobacteriales | f__Weeksellaceae | g__Chryseobacterium |  |
| 80aefbccc8705612381a42ec3336adc5 | Bacteria | p__Firmicutes | c__Bacilli | o__Lactobacillales | f__Enterococcaceae | g__Enterococcus |  |
| c57cfa03304e04a16924e1e048c2c731 | Bacteria | p__Proteobacteria | c__Gammaproteobacteria | o__Burkholderiales | f__uncultured | g__uncultured | s__beta_proteobacterium |
| MJ006-1-barcode34-umi21823bins-ubs-12 | Bacteria | p__Firmicutes | c__Bacilli | o__Staphylococcales | f__Staphylococcaceae | g__Staphylococcus |  |
| 99ccfc649153f8814004af3745fa443c | Bacteria | p__Proteobacteria | c__Gammaproteobacteria | o__Legionellales | f__Legionellaceae | g__Legionella |  |
| 4d708a8bdad2c23197789306ca7f915e | Bacteria | p__Firmicutes | c__Clostridia | o__Lachnospirales | f__Lachnospiraceae |  |  |
| 4d7cb2f1ea1ccd1e998ab577bcc9de1a | Bacteria | p__Proteobacteria | c__Gammaproteobacteria | o__Burkholderiales | f__SC-I-84 | g__SC-I-84 |  |
| MJ020-1-barcode31-umi33663bins-ubs-14 | Bacteria | p__Bacteroidota | c__Bacteroidia | o__Chitinophagales | f__Chitinophagaceae |  |  |
| MJ010-1-barcode26-umi153755bins-ubs-3 | Bacteria | p__Firmicutes | c__Clostridia | o__Oscillospirales | f__[Eubacterium]_coprostanoligenes_group | g__[Eubacterium]_coprostanoligenes_group |  |
| 081e12ed9797dd676e9ac800c92a2d3b | Bacteria | p__Firmicutes | c__Bacilli | o__Lactobacillales | f__Lactobacillaceae | g__Lactobacillus |  |
| 14521516 | Bacteria | p__Gemmatimonadota | c__Gemmatimonadetes | o__Gemmatimonadales | f__Gemmatimonadaceae |  |  |
| RS-GCF-003124035.1-NZ-QFBJ01000046.1 | Bacteria | p__Actinobacteriota | c__Actinobacteria | o__Micrococcales | f__Micrococcaceae | g__Kocuria |  |
| MJ006-1-barcode43-umi183932bins-ubs-3 | Bacteria | p__Bacteroidota | c__Bacteroidia | o__Bacteroidales | f__Tannerellaceae | g__Parabacteroides |  |
| 601e676d5a2c6ac5b3030c5642d469f9 | Bacteria | p__Proteobacteria | c__Gammaproteobacteria |  |  |  |  |
| 303837162ab464866890f5979f7e33e6 | Bacteria | p__Firmicutes | c__Clostridia | o__Lachnospirales | f__Lachnospiraceae |  |  |
| 9c8a6dbd0eb6ffd4523626a4822490ed | Bacteria | p__Actinobacteriota | c__Actinobacteria | o__Propionibacteriales | f__Propionibacteriaceae | g__Tessaracoccus |  |
| 7d470c26d4be9516e9d1901dd060f805 | Bacteria | p__Bdellovibrionota | c__Bdellovibrionia | o__Bacteriovoracales | f__Bacteriovoracaceae | g__Peredibacter |  |
| 31d00fd2eb8bc4cdb8d1ca02e42bec80 | Bacteria | p__Proteobacteria | c__Alphaproteobacteria | o__Rhizobiales | f__Xanthobacteraceae |  |  |
| 79b080ffedd3e65451b80ea79b6ef84e | Bacteria | p__Bacteroidota | c__Bacteroidia | o__Chitinophagales | f__Chitinophagaceae | g__Taibaiella | s__uncultured_bacterium |
| c291a83fd7821b0cf9fab998bb972864 | Bacteria | p__Proteobacteria | c__Alphaproteobacteria | o__Rhizobiales | f__Rhizobiaceae |  |  |
| RS-GCF-016765655.1-NZ-JAETGP010000120.1 | Bacteria | p__Proteobacteria | c__Alphaproteobacteria | o__Azospirillales | f__Azospirillaceae | g__Skermanella |  |
| KU055631 | Bacteria | p__Actinobacteriota | c__Actinobacteria | o__Frankiales | f__Sporichthyaceae | g__Longivirga |  |
| 800f982b93ee8619d73f5cb0d33ebb45 | Bacteria | p__Firmicutes | c__Symbiobacteriia | o__Symbiobacteriales | f__Symbiobacteraceae | g__Symbiobacterium | s__uncultured_bacterium |
| d32962928400761e12d481f5c9db15f3 | Bacteria | p__Proteobacteria | c__Alphaproteobacteria | o__Sphingomonadales | f__Sphingomonadaceae |  |  |
| fa1e82a81a08ff50b54c309748546861 | Bacteria | p__Bacteroidota | c__Bacteroidia | o__Bacteroidales |  |  |  |
| 0ab9456723c8092d452ad1481004b621 | Bacteria | p__Proteobacteria | c__Gammaproteobacteria | o__Burkholderiales | f__Oxalobacteraceae | g__Massilia |  |
| 517984b083eb8134755a5a6a52d1bd01 | Bacteria |  |  |  |  |  |  |
| 59d44877ca7c27cd95e83a16a99adb8d | Bacteria | p__Bacteroidota | c__Bacteroidia | o__Chitinophagales | f__Saprospiraceae | g__uncultured |  |
| 19265944 | Bacteria | p__Actinobacteriota | c__Actinobacteria | o__Propionibacteriales | f__Nocardioidaceae |  |  |
| bc47634e0667d91e6ddd2d9299652fe8 | Bacteria | p__Proteobacteria | c__Gammaproteobacteria | o__Enterobacterales | f__Enterobacteriaceae |  |  |
| 114694da49f5749095a8eb1d7a7120dd | Bacteria | p__Proteobacteria | c__Alphaproteobacteria | o__Rhodospirillales | f__Magnetospirillaceae | g__Magnetospirillaceae |  |
| RS-GCF-013408445.1-NZ-JACBZL010000001.1--4 | Bacteria | p__Actinobacteriota | c__Actinobacteria | o__Corynebacteriales | f__Corynebacteriaceae | g__Corynebacterium |  |
| 02247653 | Bacteria | p__Proteobacteria | c__Alphaproteobacteria | o__Sphingomonadales | f__Sphingomonadaceae | g__Porphyrobacter |  |
| 04994753 | Bacteria | p__Proteobacteria | c__Alphaproteobacteria | o__Rhizobiales | f__Beijerinckiaceae | g__1174-901-12 |  |
| ec8f83bfec70ff96e24b0fa6f69d3008 | Bacteria | p__Proteobacteria | c__Alphaproteobacteria | o__Sphingomonadales | f__Sphingomonadaceae |  |  |
| 05037368 | Bacteria | p__Firmicutes | c__Bacilli | o__Bacillales | f__Bacillaceae | g__Bacillus |  |
| MJ034-2-barcode71-umi756bins-ubs-474 | Bacteria | p__Bacteroidota | c__Bacteroidia | o__Cytophagales | f__Microscillaceae | g__uncultured |  |
| 8cefb6b1d41e84e9e6d2ae2e08692352 | Bacteria | p__Actinobacteriota | c__Actinobacteria | o__Micrococcales | f__Microbacteriaceae | g__Microbacterium |  |
| f67f98903edb940f12e8d0d38312129b | Bacteria | p__Firmicutes | c__Bacilli | o__Lactobacillales | f__Streptococcaceae | g__Streptococcus |  |
| b54ca0d0a744cee477262e12eb176195 | Bacteria | p__Chloroflexi | c__Chloroflexia | o__Thermomicrobiales | f__JG30-KF-CM45 | g__JG30-KF-CM45 | s__metagenome |
| d33ffe5f0d09f5ffe5d1250b605ac90b | Bacteria | p__Chloroflexi | c__Anaerolineae |  |  |  |  |
| 04833926 | Bacteria | p__Fusobacteriota | c__Fusobacteriia | o__Fusobacteriales | f__Fusobacteriaceae | g__Fusobacterium |  |
| 8def0737ecebfebe1691b5acc9ab5c4e | Bacteria | p__Proteobacteria | c__Alphaproteobacteria | o__Rhodobacterales | f__Rhodobacteraceae | g__Rhodobacter |  |
| 19951293 | Bacteria | p__Firmicutes | c__Clostridia | o__Oscillospirales | f__Oscillospiraceae |  |  |
| 05018275 | Bacteria | p__Acidobacteriota | c__Vicinamibacteria | o__Vicinamibacterales | f__uncultured | g__uncultured |  |
| 63aee52a76d9458fc053570aa48639fc | Bacteria | p__Proteobacteria | c__Alphaproteobacteria | o__Elsterales | f__uncultured | g__uncultured |  |
| RS-GCF-900465415.1-NZ-UICD01000001.1 | Bacteria | p__Proteobacteria | c__Gammaproteobacteria | o__Pseudomonadales | f__Moraxellaceae | g__Acinetobacter |  |
| MJ031-2-barcode69-umi29534bins-ubs-12 | Bacteria | p__Bacteroidota | c__Bacteroidia | o__Cytophagales | f__Spirosomaceae | g__Dyadobacter |  |
| 8382ddc8d1df939220113af406b1aaa0 | Bacteria | p__Proteobacteria | c__Gammaproteobacteria | o__Enterobacterales | f__Morganellaceae | g__Providencia |  |
| 43b9b028e29fdeffd049404c37195cc5 | Bacteria | p__Proteobacteria | c__Gammaproteobacteria | o__Cellvibrionales | f__Spongiibacteraceae | g__BD1-7_clade | s__metagenome |
| 88a7a68943aa84edeb3dd64474d6eedb | Bacteria | p__Firmicutes | c__Bacilli | o__Erysipelotrichales | f__Erysipelotrichaceae | g__Turicibacter |  |
| 95558e4d4357e4a5137e2b0b20705ca2 | Bacteria | p__Firmicutes | c__Incertae_Sedis | o__DTU014 | f__DTU014 | g__DTU014 |  |
| 02248043 | Bacteria | p__Proteobacteria | c__Alphaproteobacteria | o__Rhizobiales | f__Beijerinckiaceae | g__Methylobacterium-Methylorubrum |  |
| e362e45d79c36eb540d15c8f21f15eef | Bacteria | p__Acidobacteriota | c__Blastocatellia | o__Blastocatellales | f__Blastocatellaceae |  |  |
| 883e1b044150818e0aca10cc5a504fc9 | Bacteria | p__Proteobacteria | c__Alphaproteobacteria | o__Sphingomonadales | f__Sphingomonadaceae | g__Sphingopyxis |  |
| 01715aa5fe2b7bd83fa5bd117a534e9d | Bacteria | p__Proteobacteria | c__Alphaproteobacteria | o__Elsterales | f__uncultured | g__uncultured |  |
| ea03a9305752a192c096dfacb169bb9a | Bacteria | p__Proteobacteria | c__Gammaproteobacteria | o__Enterobacterales | f__Enterobacteriaceae |  |  |
| 04747360 | Bacteria | p__Desulfobacterota | c__Desulfovibrionia | o__Desulfovibrionales | f__Desulfovibrionaceae | g__uncultured | s__uncultured_bacterium |
| f3fce1462d35ade9ffc50860d18d28ff | Bacteria | p__Bacteroidota | c__Bacteroidia | o__Flavobacteriales | f__Flavobacteriaceae | g__Myroides | s__Myroides_odoratus |
| 17984384 | Bacteria | p__Chloroflexi | c__TK10 | o__TK10 | f__TK10 | g__TK10 | s__uncultured_bacterium |
| 04393325 | Bacteria | p__Armatimonadota | c__Fimbriimonadia | o__Fimbriimonadales | f__Fimbriimonadaceae | g__Fimbriimonadaceae |  |
| b938ce61245362113313ad75c6096751 | Bacteria | p__Firmicutes | c__Bacilli | o__Erysipelotrichales | f__Erysipelotrichaceae | g__Turicibacter |  |
| 04908968 | Bacteria | p__Proteobacteria | c__Alphaproteobacteria | o__Rhizobiales | f__Rhizobiaceae | g__Aureimonas |  |
| d85390096c98156430d2f701aef3cbdb | Bacteria | p__Proteobacteria | c__Gammaproteobacteria | o__Burkholderiales | f__Alcaligenaceae |  |  |
| e9782779368e7a828fc4a7ef29810eea | Bacteria | p__Firmicutes | c__Bacilli | o__Bacillales | f__Bacillaceae | g__Anoxybacillus |  |
| 6d368428eaff9f7371087c2320acba4b | Bacteria | p__Gemmatimonadota | c__Gemmatimonadetes | o__Gemmatimonadales | f__Gemmatimonadaceae | g__uncultured |  |
| 4c2eb16b7411a488462200a894ad85b0 | Bacteria | p__Firmicutes | c__Bacilli | o__Alicyclobacillales | f__Alicyclobacillaceae | g__Tumebacillus |  |
| abd2a691b4f4df7caf1be308f76bdbd5 | Bacteria | p__Bacteroidota | c__Bacteroidia | o__Chitinophagales | f__Chitinophagaceae |  |  |
| c780f1aed905b18a70fa7490446aec13 | Bacteria | p__Cloacimonadota | c__Cloacimonadia | o__Cloacimonadales | f__W27 | g__W27 | s__uncultured_Cloacimonetes |
| ffa3b8c3a3b50f16d76fefa0eb5cb19a | Bacteria | p__Proteobacteria | c__Gammaproteobacteria | o__Xanthomonadales | f__Rhodanobacteraceae |  |  |
| MJ030-2-barcode58-umi45220bins-ubs-15 | Bacteria | p__Firmicutes | c__Bacilli | o__Bacillales | f__Bacillaceae | g__Bacillus |  |
| 3e555cfb3e0bdbb066aed0d9f0f2be76 | Bacteria | p__Firmicutes | c__Bacilli | o__Bacillales | f__Bacillaceae | g__Geobacillus |  |
| c9fb5a95d2b02deeee9b3a44d403ecb5 | Bacteria | p__Proteobacteria | c__Gammaproteobacteria | o__Burkholderiales | f__Alcaligenaceae |  |  |
| 95d4800f0235fffb443a26611a455119 | Bacteria | p__Proteobacteria | c__Gammaproteobacteria | o__Burkholderiales | f__Comamonadaceae |  |  |
| e16dd7829ac6aed34c5fe4d7fd3d5a7d | Bacteria | p__Proteobacteria | c__Gammaproteobacteria | o__Burkholderiales | f__TRA3-20 | g__TRA3-20 |  |
| 05021759 | Bacteria | p__Actinobacteriota | c__Actinobacteria | o__Micrococcales | f__Micrococcaceae | g__Kocuria |  |
| 0d28d1aeb5495a4022f991e2dd2303c1 | Bacteria | p__Acidobacteriota | c__Vicinamibacteria | o__Subgroup_17 | f__Subgroup_17 | g__Subgroup_17 |  |
| AGBA01000019 | Bacteria | p__Actinobacteriota | c__Actinobacteria | o__Propionibacteriales | f__Propionibacteriaceae | g__Cutibacterium | s__Cutibacterium_avidum |
| 04879743 | Bacteria | p__Proteobacteria | c__Alphaproteobacteria | o__Rhizobiales | f__Beijerinckiaceae | g__Methylobacterium-Methylorubrum |  |
| 04909628 | Bacteria | p__Proteobacteria | c__Alphaproteobacteria | o__Rhizobiales | f__Rhizobiaceae | g__Ochrobactrum |  |
| 93ab2b4bb6dfe5f8deaab9a2a27fc5fb | Bacteria | p__Chloroflexi | c__KD4-96 | o__KD4-96 | f__KD4-96 | g__KD4-96 |  |
| f0de005c8c640025e027048953f03620 | Bacteria | p__Proteobacteria | c__Gammaproteobacteria | o__Xanthomonadales | f__Rhodanobacteraceae | g__Ahniella |  |
| 05012943 | Bacteria | p__Bacteroidota | c__Bacteroidia | o__Chitinophagales | f__Chitinophagaceae | g__Flavisolibacter |  |
| b1241334c2c0876eb2a88785b62aa1ea | Bacteria | p__Proteobacteria | c__Alphaproteobacteria | o__Rhizobiales | f__Xanthobacteraceae |  |  |
| d859dd8ccd2b0bbd69b9363275b422e0 | Bacteria | p__Firmicutes | c__Clostridia | o__Lachnospirales | f__Lachnospiraceae |  |  |
| b20431369dfc8648759b4b695865fd28 | Bacteria | p__Gemmatimonadota | c__Gemmatimonadetes | o__Gemmatimonadales | f__Gemmatimonadaceae | g__uncultured | s__uncultured_Nitrospira |
| 052c90cc8b8b58b0c4ab72da6b833705 | Bacteria | p__Proteobacteria | c__Gammaproteobacteria | o__Burkholderiales | f__Burkholderiaceae | g__Burkholderia-Caballeronia-Paraburkholderia |  |
| MJ030-2-barcode65-umi102734bins-ubs-3 | Bacteria | p__Acidobacteriota | c__Acidobacteriae | o__Acidobacteriales | f__uncultured | g__uncultured |  |
| a673d4d9a5d3296f88bddac23dcf8c3e | Bacteria | p__Proteobacteria | c__Gammaproteobacteria | o__Enterobacterales |  |  |  |
| 12412176 | Bacteria | p__Proteobacteria | c__Gammaproteobacteria | o__Cellvibrionales | f__Cellvibrionaceae | g__Gilvimarinus |  |
| 05030483 | Bacteria | p__Proteobacteria | c__Gammaproteobacteria | o__Burkholderiales | f__Oxalobacteraceae |  |  |
| EF660748 | Bacteria | p__Actinobacteriota | c__Actinobacteria | o__Micrococcales | f__Micrococcaceae |  |  |
| 43f7c12056477999dabcc1a6f977158e | Bacteria | p__Acidobacteriota | c__Acidobacteriae | o__Bryobacterales | f__Bryobacteraceae | g__Bryobacter |  |
| 59b12fcc02c91f7e3b5ccfdd4553dbf7 | Bacteria | p__Chloroflexi | c__Anaerolineae | o__SJA-15 | f__SJA-15 | g__SJA-15 | s__uncultured_bacterium |
| 0b1d230e6718104de4d1b9b080a68d8e | Bacteria | p__Proteobacteria | c__Alphaproteobacteria | o__Caulobacterales | f__Caulobacteraceae | g__Brevundimonas |  |
| 21955362 | Bacteria | p__Firmicutes | c__Clostridia | o__Lachnospirales | f__Lachnospiraceae |  |  |
| 6c62d98b2eefd6c50dda150cb55c5a80 | Bacteria | p__Firmicutes | c__Bacilli | o__Staphylococcales | f__Staphylococcaceae | g__Staphylococcus |  |
| 3786744cd73afb72c0af2bc8dad03019 | Bacteria | p__Proteobacteria | c__Alphaproteobacteria | o__Rhizobiales | f__Methyloligellaceae | g__uncultured | s__metagenome |
| 6711184de486858dbf0b130ccc785cd8 | Bacteria | p__Acidobacteriota | c__Vicinamibacteria | o__Vicinamibacterales | f__uncultured | g__uncultured |  |
| a7a10da162b46f4535916c68c06a0eb1 | Bacteria | p__Proteobacteria | c__Gammaproteobacteria | o__Burkholderiales | f__Burkholderiaceae | g__Ralstonia |  |
| MJ020-2-barcode50-umi8424bins-ubs-65 | Bacteria | p__Firmicutes | c__Clostridia | o__Clostridia_UCG-014 | f__Clostridia_UCG-014 | g__Clostridia_UCG-014 |  |
| MJ030-2-barcode59-umi64324bins-ubs-9 | Bacteria | p__Bacteroidota | c__Bacteroidia | o__Cytophagales | f__Microscillaceae | g__uncultured |  |
| RS-GCF-904848645.1-NZ-CAJHCS010000127.1 | Bacteria | p__Proteobacteria | c__Gammaproteobacteria | o__Burkholderiales | f__Burkholderiaceae | g__Burkholderia-Caballeronia-Paraburkholderia |  |
| 039048eaf623338e11c8fb0ad5ed09cc | Bacteria | p__Actinobacteriota | c__Actinobacteria | o__Bifidobacteriales | f__Bifidobacteriaceae | g__Gardnerella | s__Gardnerella_vaginalis |
| e7f123ad91a3e965454903be8ff4aae2 | Bacteria | p__Proteobacteria | c__Gammaproteobacteria | o__Burkholderiales | f__Comamonadaceae |  |  |
| 3c124ea3d83804ecb8b37eafd7cb14b3 | Bacteria | p__Actinobacteriota | c__Actinobacteria | o__Micrococcales | f__Microbacteriaceae | g__Microbacterium |  |
| 02946860 | Bacteria | p__Chloroflexi | c__Chloroflexia | o__Chloroflexales | f__Roseiflexaceae | g__uncultured | s__uncultured_Chloroflexi |
| 575b5b2c720563013a10ca95e010a2e2 | Bacteria | p__Patescibacteria | c__Dojkabacteria | o__Dojkabacteria | f__Dojkabacteria | g__Dojkabacteria | s__uncultured_anaerobic |
| ac8539a45030a025c58e21dcc338ee7f | Bacteria | p__Proteobacteria | c__Gammaproteobacteria | o__Burkholderiales | f__Comamonadaceae |  |  |
| b511e31a97d95acd625c865de2c19388 | Bacteria | p__Proteobacteria | c__Alphaproteobacteria | o__Rhizobiales | f__Rhizobiaceae | g__Allorhizobium-Neorhizobium-Pararhizobium-Rhizobium | s__[Pseudomonas]_geniculata |
| d5586881e08941c55678c65d1f3334d9 | Bacteria | p__Actinobacteriota | c__Actinobacteria | o__Micrococcales |  |  |  |
| 0552825cd29471a0ea8e8fcb9f5a02a2 | Bacteria | p__Bacteroidota | c__Bacteroidia | o__Chitinophagales | f__Chitinophagaceae | g__Flaviaesturariibacter | s__Flaviaesturariibacter_sp. |
| G000224715_1 | Bacteria | p__Actinobacteriota | c__Actinobacteria | o__Micrococcales | f__Intrasporangiaceae |  |  |
| 8d72dd94d299c7c390454d7e09d5748f | Bacteria |  |  |  |  |  |  |
| AB238928 | Bacteria | p__Bacteroidota | c__Bacteroidia | o__Bacteroidales | f__Tannerellaceae | g__Parabacteroides | s__Parabacteroides_merdae |
| 3b00d0affc96e8c1d995333e78e0d37c | Bacteria | p__Proteobacteria | c__Gammaproteobacteria | o__Burkholderiales | f__Nitrosomonadaceae |  |  |
| KF309176 | Bacteria | p__Bacteroidota | c__Bacteroidia | o__Chitinophagales | f__Chitinophagaceae | g__Flavihumibacter |  |
| 445fe962d3077a6eb086a59403f89392 | Bacteria | p__Actinobacteriota | c__Thermoleophilia | o__Solirubrobacterales | f__67-14 | g__67-14 | s__uncultured_Conexibacteraceae |
| b096904a99a590b047baf1ba63192b76 | Bacteria | p__Gemmatimonadota |  |  |  |  |  |
| MJ034-1-barcode40-umi3598bins-ubs-126 | Bacteria | p__Bacteroidota | c__Bacteroidia | o__Cytophagales | f__Microscillaceae |  |  |
| cef8ed9d8ae3b4ae55c12163b8983755 | Bacteria | p__Firmicutes | c__Clostridia | o__Clostridia | f__Hungateiclostridiaceae |  |  |
| 89cd782dbe8b4440e9f00d71ccc4701c | Bacteria | p__Actinobacteriota | c__MB-A2-108 | o__MB-A2-108 | f__MB-A2-108 | g__MB-A2-108 | s__uncultured_Catenuloplanes |
| d66b7fc3728e9d64530740177408f8dd | Bacteria | p__Verrucomicrobiota | c__Chlamydiae | o__Chlamydiales | f__Parachlamydiaceae | g__Neochlamydia | s__metagenome |
| 05032315 | Bacteria | p__Proteobacteria | c__Gammaproteobacteria | o__Burkholderiales | f__Comamonadaceae | g__Aquabacterium | s__Aquabacterium_citratiphilum |
| 86c992a0a3c7e453a525972cca299b4d | Bacteria | p__Firmicutes | c__Bacilli | o__Lactobacillales | f__Enterococcaceae | g__Enterococcus |  |
| MJ030-2-barcode59-umi77948bins-ubs-6 | Bacteria | p__Proteobacteria | c__Gammaproteobacteria | o__Burkholderiales | f__Comamonadaceae |  |  |
| bf63549ae3342598976567845d4ba449 | Bacteria | p__Actinobacteriota | c__Actinobacteria | o__Frankiales | f__Acidothermaceae | g__Acidothermus | s__uncultured_soil |
| RS-GCF-009734705.1-NZ-WOGT01000002.1 | Bacteria | p__Actinobacteriota | c__Actinobacteria | o__Micrococcales | f__Micrococcaceae | g__Kocuria |  |
| MJ031-1-barcode42-umi40089bins-ubs-3 | Bacteria | p__Firmicutes | c__Clostridia | o__Peptostreptococcales-Tissierellales | f__Peptostreptococcaceae | g__Sporacetigenium |  |
| RS-GCF-002204165.1-NZ-NBNP01000057.1 | Bacteria | p__Proteobacteria | c__Gammaproteobacteria | o__Pseudomonadales | f__Moraxellaceae | g__Acinetobacter |  |
| MJ030-1-barcode42-umi77403bins-ubs-5 | Bacteria | p__Bacteroidota | c__Bacteroidia | o__Cytophagales | f__Microscillaceae | g__uncultured |  |
| MJ032-2-barcode60-umi81298bins-ubs-4 | Bacteria | p__Bacteroidota | c__Bacteroidia | o__Cytophagales | f__Cytophagaceae | g__Siphonobacter | s__Siphonobacter_sp. |
| 04952042 | Bacteria | p__Actinobacteriota | c__Thermoleophilia | o__Solirubrobacterales | f__67-14 | g__67-14 |  |
| 6cbbbf867b56460cbb85af2fe97125fa | Bacteria | p__Acidobacteriota | c__Blastocatellia | o__Pyrinomonadales | f__Pyrinomonadaceae | g__RB41 |  |
| 05012932 | Bacteria | p__Bacteroidota | c__Bacteroidia | o__Chitinophagales | f__Chitinophagaceae |  |  |
| AY170848 | Bacteria | p__Proteobacteria | c__Gammaproteobacteria | o__Burkholderiales | f__Alcaligenaceae | g__Achromobacter |  |
| 43fb27b806e21b369bb40742733caab8 | Bacteria | p__Desulfobacterota | c__Desulfovibrionia | o__Desulfovibrionales | f__Desulfomicrobiaceae | g__Desulfomicrobium |  |
| 56f12d890f6d7ee7c7b0671a9d39721c | Bacteria | p__Chloroflexi | c__Anaerolineae | o__RBG-13-54-9 | f__RBG-13-54-9 | g__RBG-13-54-9 |  |
| 04305779 | Bacteria | p__WPS-2 | c__WPS-2 | o__WPS-2 | f__WPS-2 | g__WPS-2 | s__uncultured_bacterium |
| 02412552 | Bacteria | p__Proteobacteria | c__Alphaproteobacteria | o__Rhizobiales | f__Xanthobacteraceae | g__Bradyrhizobium |  |
| d2ce8b882069fd9c7916bbfc77589b74 | Bacteria | p__Chloroflexi | c__Anaerolineae | o__Caldilineales | f__Caldilineaceae | g__uncultured | s__uncultured_bacterium |
| 040b9d77caeac725ca3cba322ab9a24f | Bacteria | p__Patescibacteria | c__Saccharimonadia | o__Saccharimonadales | f__Saccharimonadales | g__Saccharimonadales | s__metagenome |
| GB-GCA-016874295.1-VGUH01000017.1 | Bacteria | p__Proteobacteria | c__Gammaproteobacteria | o__PLTA13 | f__PLTA13 | g__PLTA13 |  |
| d0ca75c30eb598b684d656f3e8dbe2f2 | Bacteria | p__Proteobacteria | c__Gammaproteobacteria | o__Alteromonadales | f__Alteromonadaceae | g__Rheinheimera |  |
| 8957b5aea2b859d6414ef6176a415022 | Bacteria | p__Actinobacteriota | c__Thermoleophilia | o__Solirubrobacterales | f__67-14 | g__67-14 |  |
| aaf9288e3127cfad185e1f8df9f35964 | Bacteria | p__Proteobacteria | c__Alphaproteobacteria | o__Sphingomonadales | f__Sphingomonadaceae |  |  |
| fe6c02161f22774668eea0300b281814 | Bacteria | p__Proteobacteria | c__Alphaproteobacteria | o__uncultured | f__uncultured | g__uncultured |  |
| 04959814 | Bacteria | p__Proteobacteria | c__Gammaproteobacteria | o__Burkholderiales | f__Comamonadaceae |  |  |
| b51f4f9cdbd07f0851f3f75761f79a66 | Bacteria | p__Chloroflexi | c__Anaerolineae | o__SBR1031 | f__SBR1031 | g__SBR1031 | s__uncultured_Caldilineaceae |
| cc306ea7ab5b2cdfa5f3d02cc3bcccb8 | Bacteria | p__Proteobacteria | c__Gammaproteobacteria | o__Pseudomonadales | f__Moraxellaceae | g__Acinetobacter |  |
| 11029916 | Bacteria | p__Bacteroidota | c__Bacteroidia | o__Bacteroidales | f__Muribaculaceae | g__Muribaculaceae | s__unidentified |
| c879c43a433289947a6e5779f78c9993 | Bacteria | p__Bacteroidota | c__Bacteroidia | o__Sphingobacteriales | f__Sphingobacteriaceae | g__Sphingobacterium |  |
| MJ010-1-barcode47-umi10570bins-ubs-50 | Bacteria | p__Bacteroidota | c__Bacteroidia | o__Sphingobacteriales | f__Sphingobacteriaceae | g__Sphingobacterium |  |
| a33bb47953c120e0e1abe4c7f1df882b | Bacteria | p__Proteobacteria | c__Gammaproteobacteria | o__Burkholderiales | f__Nitrosomonadaceae | g__Ellin6067 |  |
| MJ031-1-barcode38-umi37203bins-ubs-4 | Bacteria | p__Firmicutes | c__Clostridia | o__Oscillospirales | f__Ruminococcaceae | g__Ruminococcus | s__unidentified |
| de38b75704d33dfbb1f038c9a1ea9e60 | Bacteria | p__Proteobacteria | c__Alphaproteobacteria | o__Rhizobiales | f__Xanthobacteraceae |  |  |
| ba66c90481d30c8c6dfb074e998c99f9 | Bacteria | p__Proteobacteria | c__Alphaproteobacteria | o__Rhizobiales | f__Beijerinckiaceae |  |  |
| KM507333 | Bacteria | p__Actinobacteriota | c__Actinobacteria | o__Micrococcales | f__Micrococcaceae |  |  |
| MJ006-1-barcode37-umi144579bins-ubs-3 | Bacteria | p__Firmicutes | c__Clostridia | o__Oscillospirales | f__Ruminococcaceae | g__uncultured | s__gut_metagenome |
| 561bfb1ecda2256b639f019262a7023c | Bacteria | p__Chloroflexi | c__Anaerolineae | o__SBR1031 | f__A4b | g__A4b |  |
| 07788373 | Bacteria | p__Planctomycetota | c__Planctomycetes | o__Pirellulales | f__Pirellulaceae | g__Pir4_lineage |  |
| 5a942c8070a14e7be52d46adfcd5f6fd | Bacteria | p__Actinobacteriota | c__Actinobacteria | o__Streptomycetales | f__Streptomycetaceae | g__Streptomyces |  |
| 6a31a358554150a12594f1f26bc74de3 | Bacteria | p__Bacteroidota | c__Bacteroidia | o__Bacteroidales | f__Prevotellaceae |  |  |
| 4faab5dc4dfbd34d49b2fb42abf856ef | Bacteria | p__Acidobacteriota | c__Vicinamibacteria | o__Vicinamibacterales | f__Vicinamibacteraceae |  |  |
| 02247881 | Bacteria | p__Proteobacteria | c__Alphaproteobacteria | o__Rhizobiales | f__Rhizobiales_Incertae_Sedis | g__Phreatobacter |  |
| 794d9e4cdc87a47bf883721dcab0b890 | Bacteria | p__Bacteroidota | c__Bacteroidia | o__Bacteroidales | f__Prevotellaceae | g__Prevotella |  |
| 432daa4bb6cc83c73e0fa0bf26fe6060 | Bacteria | p__Firmicutes | c__Bacilli | o__Bacillales | f__Bacillaceae | g__Anoxybacillus |  |
| 08187873 | Bacteria | p__Proteobacteria | c__Alphaproteobacteria | o__Rhizobiales | f__Xanthobacteraceae | g__uncultured |  |
| RS-GCF-018785055.1-NZ-WQPE01000063.1 | Bacteria | p__Firmicutes | c__Clostridia | o__Lachnospirales | f__Lachnospiraceae | g__Coprococcus |  |
| 4db995e9cbc484c01c2716b2721a0721 | Bacteria | p__Verrucomicrobiota | c__Chlamydiae | o__Chlamydiales | f__Parachlamydiaceae | g__Neochlamydia |  |
| KT345712 | Bacteria | p__Proteobacteria | c__Alphaproteobacteria | o__Rhizobiales | f__Devosiaceae | g__Devosia |  |
| 05105832 | Bacteria | p__Bacteroidota | c__Bacteroidia | o__Chitinophagales | f__Saprospiraceae | g__uncultured |  |
| 02303380 | Bacteria | p__Firmicutes | c__Clostridia | o__Lachnospirales | f__Lachnospiraceae |  |  |
| 21849007 | Bacteria | p__Firmicutes | c__Clostridia | o__Lachnospirales | f__Lachnospiraceae |  |  |
| d6a3072af5293a9fcf9a65b5d81f92b3 | Bacteria |  |  |  |  |  |  |
| f763d10152c230d95bd78ddefea0b580 | Bacteria | p__Firmicutes | c__Bacilli | o__Lactobacillales | f__Leuconostocaceae | g__Weissella |  |
| 03178767 | Bacteria | p__Planctomycetota | c__Phycisphaerae | o__Tepidisphaerales | f__WD2101_soil_group | g__WD2101_soil_group |  |
| 543c185769e37dcd5589d4225ac83383 | Bacteria | p__Firmicutes | c__Clostridia | o__Peptostreptococcales-Tissierellales | f__Peptostreptococcales-Tissierellales | g__W5053 | s__uncultured_bacterium |
| 18631408 | Bacteria | p__Chloroflexi | c__TK10 | o__TK10 | f__TK10 | g__TK10 | s__bacterium_Ellin6519 |
| 32d7db72a36ed9e47dd1a6ef662d2113 | Bacteria | p__Proteobacteria | c__Alphaproteobacteria | o__Rhizobiales | f__Rhizobiaceae | g__Mesorhizobium |  |
| 50b5a2a6238ba242614f1e83d881ba7e | Bacteria | p__Proteobacteria | c__Gammaproteobacteria | o__PLTA13 | f__PLTA13 | g__PLTA13 |  |
| 7c4ea2dbe30f9686545c21d5adccd304 | Bacteria | p__Acidobacteriota | c__Acidobacteriae | o__Acidobacteriales | f__Acidobacteriaceae_(Subgroup_1) | g__Granulicella |  |
| c1a6380622ed2405577281b1aeff2b40 | Bacteria | p__Proteobacteria | c__Alphaproteobacteria | o__Acetobacterales | f__Acetobacteraceae | g__uncultured |  |
| b5a3a7dc4295cd92ed2daeafa604be4b | Bacteria | p__Proteobacteria | c__Alphaproteobacteria | o__Sphingomonadales | f__Sphingomonadaceae | g__Sphingobium |  |
| 0479443456fcf93da94889df8a0ab933 | Bacteria | p__Proteobacteria | c__Gammaproteobacteria | o__Enterobacterales |  |  |  |
| 39954e4aa0a5b2b188dde76bb63ad139 | Bacteria | p__Actinobacteriota | c__Actinobacteria | o__Pseudonocardiales | f__Pseudonocardiaceae | g__Pseudonocardia |  |
| 80936a037af8e8514eeb3a8bbe585d38 | Bacteria | p__Actinobacteriota | c__Thermoleophilia | o__Solirubrobacterales | f__Solirubrobacteraceae |  |  |
| 05012928 | Bacteria | p__Bacteroidota | c__Bacteroidia | o__Chitinophagales | f__Chitinophagaceae | g__Flavisolibacter |  |
| 5be7a7e668c1ec13c6b8e1e711f95af6 | Bacteria |  |  |  |  |  |  |
| 02951247 | Bacteria | p__Chloroflexi | c__Chloroflexia | o__Chloroflexales | f__Roseiflexaceae | g__uncultured | s__uncultured_bacterium |
| 00047c180a302bc52fbb832781826ed9 | Bacteria | p__Proteobacteria | c__Alphaproteobacteria | o__Rhizobiales | f__Rhizobiales_Incertae_Sedis | g__Bauldia |  |
| AJ420326 | Bacteria | p__Proteobacteria | c__Gammaproteobacteria | o__Burkholderiales | f__Comamonadaceae | g__Comamonas |  |
| DQ239766 | Bacteria | p__Proteobacteria | c__Gammaproteobacteria | o__Xanthomonadales | f__Xanthomonadaceae |  |  |
| 04957436 | Bacteria | p__Proteobacteria | c__Gammaproteobacteria | o__Burkholderiales | f__Alcaligenaceae | g__Achromobacter |  |
| a0eb16aea078323b8683218bb2659961 | Bacteria | p__Proteobacteria | c__Alphaproteobacteria | o__Rhizobiales | f__Rhizobiaceae |  |  |
| 812b384925ec8cc05a727b8173cf251e | Bacteria | p__Proteobacteria | c__Gammaproteobacteria | o__Pseudomonadales | f__Pseudomonadaceae | g__Pseudomonas |  |
| 5d8e0d6880995d6aa1215cdb9293d177 | Bacteria | p__Proteobacteria | c__Alphaproteobacteria | o__Rhizobiales | f__Rhizobiales_Incertae_Sedis | g__uncultured |  |
| G003538775_1 | Bacteria |  |  |  |  |  |  |
| 11129769 | Bacteria | p__Bacteroidota | c__Bacteroidia | o__Bacteroidales | f__Muribaculaceae | g__Muribaculaceae | s__uncultured_bacterium |
| RS-GCF-000577615.1-NZ-CBXX010000086.1 | Bacteria | p__Proteobacteria | c__Gammaproteobacteria | o__Burkholderiales | f__Burkholderiaceae | g__Burkholderia-Caballeronia-Paraburkholderia |  |
| RS-GCF-901482695.1-NZ-LR590484.1--4 | Bacteria | p__Bacteroidota | c__Bacteroidia | o__Sphingobacteriales | f__Sphingobacteriaceae | g__Sphingobacterium | s__Sphingobacterium_thalpophilum |
| MJ010-2-barcode72-umi7204bins-ubs-47 | Bacteria | p__Bacteroidota | c__Ignavibacteria | o__Ignavibacteriales | f__PHOS-HE36 | g__PHOS-HE36 |  |
| 04972306 | Bacteria | p__Firmicutes | c__Bacilli | o__Lactobacillales | f__Leuconostocaceae | g__Weissella |  |
| 451eed3044bd988c5a3c318ca37a7353 | Bacteria | p__Proteobacteria | c__Gammaproteobacteria | o__Xanthomonadales | f__Xanthomonadaceae | g__Lysobacter |  |
| 733df66a8f792b7964a8a3cd5967b7fb | Bacteria | p__Proteobacteria | c__Gammaproteobacteria | o__Methylococcales | f__Methylococcaceae |  |  |
| 188cf47bd13081bc038ed60efe5e0a99 | Bacteria | p__Proteobacteria | c__Alphaproteobacteria | o__Dongiales | f__Dongiaceae | g__Dongia |  |
| MJ010-1-barcode29-umi151430bins-ubs-4 | Bacteria | p__Bacteroidota | c__Bacteroidia | o__Sphingobacteriales | f__Sphingobacteriaceae | g__Sphingobacterium |  |
| 16558f4669ce7ef09ccae5a2a7907f55 | Bacteria | p__Chloroflexi | c__Chloroflexia | o__Chloroflexales | f__Roseiflexaceae | g__uncultured |  |
| 19485546 | Bacteria | p__Firmicutes | c__Bacilli | o__Lactobacillales | f__Streptococcaceae | g__Streptococcus |  |
| e3025a0232efb9016c25eb4b457fe73d | Bacteria | p__Proteobacteria | c__Alphaproteobacteria | o__Acetobacterales | f__Acetobacteraceae |  |  |
| 8c47d53b4a979feb426873619f2dee2f | Bacteria | p__Actinobacteriota | c__Coriobacteriia | o__Coriobacteriales | f__Eggerthellaceae | g__Enterorhabdus | s__uncultured_bacterium |
| RS-GCF-014287955.1-NZ-JACOPD010000022.1 | Bacteria | p__Firmicutes | c__Clostridia | o__Lachnospirales | f__Lachnospiraceae | g__Lachnospira |  |
| 602df105c3f7f321471f23b059112ab4 | Bacteria | p__Chloroflexi | c__Anaerolineae | o__SBR1031 | f__A4b | g__A4b |  |
| 79e5813ca37c6aca2e317d5f0d88c039 | Bacteria | p__Proteobacteria | c__Gammaproteobacteria | o__Pseudomonadales | f__Pseudomonadaceae | g__Pseudomonas |  |
| MJ006-2-barcode69-umi175247bins-ubs-3 | Bacteria | p__Proteobacteria | c__Gammaproteobacteria | o__Burkholderiales | f__Sutterellaceae | g__Parasutterella |  |
| 391b8275591fb57d2cc1ef76d62ed9f8 | Bacteria | p__Proteobacteria | c__Gammaproteobacteria | o__Burkholderiales | f__Burkholderiaceae | g__Burkholderia-Caballeronia-Paraburkholderia |  |
| X82598 | Bacteria | p__Actinobacteriota | c__Actinobacteria | o__Micrococcales | f__Cellulomonadaceae |  |  |
| 984d2bf22aeeab6eab08d7e9c9c43d55 | Bacteria | p__Proteobacteria | c__Gammaproteobacteria | o__Enterobacterales | f__Enterobacteriaceae |  |  |
| 02412111 | Bacteria | p__Proteobacteria | c__Alphaproteobacteria | o__Rhizobiales | f__Xanthobacteraceae |  |  |
| 8e5ecf43a16d251d4782a1630aa10836 | Bacteria | p__Proteobacteria | c__Gammaproteobacteria | o__Gammaproteobacteria_Incertae_Sedis | f__Unknown_Family | g__Acidibacter |  |
| 355d1afd340130107e7fd642f73defe4 | Bacteria | p__Firmicutes | c__Negativicutes | o__Veillonellales-Selenomonadales | f__Veillonellaceae | g__Dialister | s__Dialister_succinatiphilus |
| 8dc5bb31647e143bccb6655a3a57ed54 | Bacteria | p__Firmicutes | c__Bacilli | o__Bacillales | f__Planococcaceae | g__Chryseomicrobium |  |
| 05011421 | Bacteria | p__Proteobacteria | c__Gammaproteobacteria | o__Aeromonadales | f__Aeromonadaceae | g__Aeromonas |  |
| 5ad4d3ae9cebf8b07ec634e955f3c5dd | Bacteria | p__Actinobacteriota | c__MB-A2-108 | o__MB-A2-108 | f__MB-A2-108 | g__MB-A2-108 | s__Actinobacteria_bacterium |
| 01683268 | Bacteria | p__Actinobacteriota | c__Actinobacteria | o__Corynebacteriales | f__Corynebacteriaceae | g__Corynebacterium |  |
| MJ010-1-barcode25-umi77945bins-ubs-9 | Bacteria | p__Proteobacteria | c__Gammaproteobacteria | o__Pseudomonadales | f__Moraxellaceae | g__Acinetobacter |  |
| EU364817 | Bacteria | p__Bacteroidota | c__Bacteroidia | o__Sphingobacteriales | f__Sphingobacteriaceae | g__Sphingobacterium |  |
| 77997d0ec864733dcf3caca2ed2417f6 | Bacteria | p__Proteobacteria | c__Gammaproteobacteria | o__Burkholderiales | f__Rhodocyclaceae | g__uncultured | s__uncultured_organism |
| 05917669 | Bacteria | p__Acidobacteriota | c__Holophagae | o__Subgroup_7 | f__Subgroup_7 | g__Subgroup_7 |  |
| KC107829 | Bacteria | p__Actinobacteriota | c__Actinobacteria | o__Micrococcales | f__Microbacteriaceae | g__Leucobacter |  |
| 04907337 | Bacteria | p__Proteobacteria | c__Alphaproteobacteria | o__Rhizobiales | f__Xanthobacteraceae | g__Pseudolabrys |  |
| 8d507a51862a0f529057480dccccacd3 | Bacteria | p__Bacteroidota | c__Bacteroidia | o__Flavobacteriales | f__Flavobacteriaceae | g__Flavobacterium |  |
| 590af6a99a9d03cbd173360e25793de0 | Bacteria | p__Verrucomicrobiota | c__Verrucomicrobiae | o__Chthoniobacterales | f__Chthoniobacteraceae | g__Candidatus_Udaeobacter |  |
| f7e58f5b98069c02937b1c7671b9fe4e | Bacteria | p__Acidobacteriota | c__Vicinamibacteria | o__Vicinamibacterales | f__uncultured | g__uncultured |  |
| RS-GCF-007115085.1-NZ-SULK01000061.1 | Bacteria | p__Firmicutes | c__Clostridia | o__Clostridiales | f__Clostridiaceae | g__Clostridium_sensu_stricto_1 |  |
| 01189822 | Bacteria | p__Actinobacteriota | c__Coriobacteriia | o__Coriobacteriales | f__Atopobiaceae | g__Coriobacteriaceae_UCG-002 | s__uncultured_bacterium |
| G004127505_2 | Bacteria | p__Firmicutes | c__Bacilli | o__Staphylococcales | f__Staphylococcaceae | g__Staphylococcus |  |
| 96098fc6813883a1809740d39c7bd3fc | Bacteria | p__Firmicutes | c__Bacilli | o__Erysipelotrichales | f__Erysipelotrichaceae | g__ZOR0006 |  |
| ee84c18d0d15c7633dea0e4082819b2a | Bacteria | p__Proteobacteria | c__Gammaproteobacteria | o__Thiotrichales | f__Thiotrichaceae | g__Thiothrix |  |
| MJ006-1-barcode47-umi78729bins-ubs-6 | Bacteria | p__Proteobacteria | c__Gammaproteobacteria | o__Pasteurellales | f__Pasteurellaceae | g__Haemophilus |  |
| b53713883dc8ecf84668e50efcc4ea8e | Bacteria | p__Acidobacteriota | c__Acidobacteriae | o__Acidobacteriales | f__Koribacteraceae | g__Candidatus_Koribacter |  |
| 05033095 | Bacteria | p__Actinobacteriota | c__Actinobacteria | o__Corynebacteriales | f__Mycobacteriaceae | g__Mycobacterium |  |
| MT500785 | Bacteria | p__Firmicutes | c__Bacilli | o__Erysipelotrichales | f__Erysipelotrichaceae | g__Turicibacter |  |
| 11125909 | Bacteria | p__Bacteroidota | c__Bacteroidia | o__Bacteroidales | f__Muribaculaceae | g__Muribaculaceae |  |
| 382594db946b6b1154289f23cec319c1 | Bacteria | p__Proteobacteria | c__Alphaproteobacteria | o__Rhizobiales | f__Hyphomicrobiaceae | g__Pedomicrobium |  |
| bc647f63e005a9898a8bbd33fc6413b3 | Bacteria | p__Bacteroidota | c__Bacteroidia | o__Sphingobacteriales | f__Sphingobacteriaceae | g__Sphingobacterium |  |
| aecb959fcddbb8957e0d09cc082c905a | Bacteria | p__Gemmatimonadota | c__Gemmatimonadetes | o__Gemmatimonadales | f__Gemmatimonadaceae |  |  |
| 2dbd0832ce38132c5ce5370899922562 | Bacteria | p__Proteobacteria | c__Gammaproteobacteria | o__Burkholderiales | f__Comamonadaceae |  |  |
| 02b61020909a7311c34a9245242d17b7 | Bacteria | p__Proteobacteria | c__Gammaproteobacteria | o__Pseudomonadales | f__Pseudomonadaceae | g__Pseudomonas |  |
| 04965833 | Bacteria | p__Firmicutes | c__Bacilli | o__Exiguobacterales | f__Exiguobacteraceae | g__Exiguobacterium |  |
| MJ034-1-barcode33-umi6166bins-ubs-73 | Bacteria | p__Bacteroidota | c__Bacteroidia | o__Chitinophagales | f__Chitinophagaceae | g__Niastella | s__uncultured_Niastella |
| 21720661 | Bacteria | p__Firmicutes | c__Clostridia | o__Lachnospirales | f__Lachnospiraceae | g__A2 | s__uncultured_bacterium |
| 715fa92f8d8e68c19d04ff1fb6224171 | Bacteria | p__Acidobacteriota | c__Vicinamibacteria | o__Vicinamibacterales | f__uncultured | g__uncultured |  |
| KF444510 | Bacteria | p__Proteobacteria | c__Alphaproteobacteria | o__Rhizobiales | f__Rhizobiaceae |  |  |
| DQ335215 | Bacteria | p__Proteobacteria | c__Alphaproteobacteria | o__Caulobacterales | f__Caulobacteraceae | g__Brevundimonas |  |
| eaa8ef356e2630f4a7dd1ced9b9615f4 | Bacteria | p__Actinobacteriota | c__Thermoleophilia | o__Gaiellales | f__uncultured | g__uncultured |  |
| 1bad16f1fb0249d179f2428234be1ac0 | Bacteria | p__Firmicutes | c__Bacilli | o__Lactobacillales | f__Enterococcaceae | g__Enterococcus |  |
| MJ006-1-barcode37-umi59724bins-ubs-6 | Bacteria | p__Firmicutes | c__Clostridia | o__Oscillospirales | f__Oscillospiraceae | g__NK4A214_group |  |
| MJ031-1-barcode42-umi34147bins-ubs-7 | Bacteria | p__Bacteroidota | c__Bacteroidia | o__Chitinophagales | f__Chitinophagaceae |  |  |
| 05012915 | Bacteria | p__Bacteroidota | c__Bacteroidia | o__Chitinophagales | f__Chitinophagaceae | g__Flavisolibacter | s__Flavisolibacter_tropicus |
| MH665405 | Bacteria | p__Proteobacteria | c__Alphaproteobacteria | o__Rhodobacterales | f__Rhodobacteraceae | g__Paracoccus |  |
| 02262764 | Bacteria | p__Actinobacteriota | c__Actinobacteria | o__Corynebacteriales | f__Corynebacteriaceae | g__Corynebacterium |  |
| 7077373651490d775a94ebc9d9a5bd08 | Bacteria | p__Acidobacteriota | c__Acidobacteriae | o__Acidobacteriales | f__uncultured | g__uncultured |  |
| 04941012 | Bacteria | p__Acidobacteriota | c__Blastocatellia | o__Pyrinomonadales | f__Pyrinomonadaceae | g__RB41 |  |
| a5e627410f3e78d74cec30b738ce4cbb | Bacteria | p__Proteobacteria | c__Alphaproteobacteria | o__Rhodobacterales | f__Rhodobacteraceae | g__Rhodobacter |  |
| 0fa4dcce4c03eae196af0484850d26db | Bacteria | p__Proteobacteria | c__Alphaproteobacteria | o__Caulobacterales | f__Parvularculaceae | g__Amphiplicatus |  |
| RS-GCF-001550375.1-NZ-LRTR01000508.1 | Bacteria | p__Actinobacteriota | c__Actinobacteria | o__Streptomycetales | f__Streptomycetaceae | g__Streptomyces |  |
| 022eb1ca1c2c99e59d38c3f6be65f7c1 | Bacteria | p__Patescibacteria | c__Saccharimonadia | o__Saccharimonadales | f__Saccharimonadaceae |  |  |
| 6614d05a7167912f193806026eaca73e | Bacteria | p__Actinobacteriota | c__Actinobacteria | o__Frankiales | f__Sporichthyaceae | g__uncultured |  |
| 08f1083a328b57a28924a30523d46a7c | Bacteria | p__Acidobacteriota | c__Vicinamibacteria | o__Vicinamibacterales | f__Vicinamibacteraceae | g__Vicinamibacteraceae |  |
| 3986155d33fa6c1a83ddeaa4f62e6cee | Bacteria | p__Actinobacteriota | c__Acidimicrobiia | o__Microtrichales | f__Ilumatobacteraceae | g__CL500-29_marine_group |  |
| 05002741 | Bacteria | p__Bacteroidota | c__Bacteroidia | o__Sphingobacteriales | f__Sphingobacteriaceae | g__Sphingobacterium |  |
| EF688011 | Bacteria | p__Proteobacteria | c__Gammaproteobacteria | o__Enterobacterales |  |  |  |
| a53c2b1657807ac55cd24b67651a2124 | Bacteria | p__Proteobacteria | c__Alphaproteobacteria | o__Rhizobiales | f__Rhizobiaceae | g__Ciceribacter |  |
| 04958200 | Bacteria | p__Actinobacteriota | c__Actinobacteria | o__Corynebacteriales | f__Nocardiaceae | g__Rhodococcus |  |
| 02244479 | Bacteria | p__Actinobacteriota | c__Actinobacteria | o__Micrococcales | f__Micrococcaceae | g__Micrococcus |  |
| f241250e4975ea43abad3db882814321 | Bacteria | p__Firmicutes | c__Bacilli | o__Lactobacillales | f__Lactobacillaceae | g__Lactobacillus |  |
| ccfa3a5cc8c3149c0fb607ce7991bf6b | Bacteria | p__Proteobacteria | c__Gammaproteobacteria | o__Burkholderiales | f__SC-I-84 | g__SC-I-84 |  |
| 0a2b44668bc3a62c9d985367b04e7eef | Bacteria | p__Firmicutes | c__Clostridia | o__Lachnospirales | f__Lachnospiraceae |  |  |
| RS-GCF-002161355.1-NZ-NFHL01000016.1 | Bacteria | p__Firmicutes | c__Clostridia | o__Lachnospirales | f__Lachnospiraceae | g__[Ruminococcus]_torques_group |  |
| MJ030-2-barcode65-umi42674bins-ubs-15 | Bacteria | p__Acidobacteriota | c__Acidobacteriae | o__Acidobacteriales | f__Koribacteraceae | g__Candidatus_Koribacter |  |
| b592ca1636955119509b2f2bac0c4926 | Bacteria | p__Proteobacteria | c__Gammaproteobacteria | o__Xanthomonadales | f__Xanthomonadaceae | g__Thermomonas |  |
| eb060286cdd53b6a933dcae68e9a8451 | Bacteria | p__Proteobacteria | c__Gammaproteobacteria | o__Enterobacterales |  |  |  |
| 05011385 | Bacteria | p__Proteobacteria | c__Gammaproteobacteria | o__Enterobacterales | f__Enterobacteriaceae |  |  |
| ea2bf6a6cee8e92eca61b52afbfa1d4e | Bacteria | p__Actinobacteriota | c__Actinobacteria | o__Propionibacteriales | f__Nocardioidaceae | g__Marmoricola |  |
| 04995433 | Bacteria | p__Proteobacteria | c__Alphaproteobacteria | o__Rhizobiales | f__Devosiaceae | g__Devosia |  |
| 820ee09cc8cbfd2d334b14059e1ca439 | Bacteria |  |  |  |  |  |  |
| 228f4ff7ee34ae266ee5a259091a3767 | Bacteria | p__Verrucomicrobiota | c__Verrucomicrobiae | o__Pedosphaerales | f__Pedosphaeraceae | g__ADurb.Bin063-1 |  |
| c8745812cf37086febaa66ef19eff0e7 | Bacteria | p__Firmicutes | c__Bacilli | o__Staphylococcales | f__Staphylococcaceae | g__Staphylococcus | s__Staphylococcus_succinus |
| d663be7220d104ad9a87003ed88b279e | Bacteria | p__Desulfobacterota | c__uncultured | o__uncultured | f__uncultured | g__uncultured |  |
| GB-GCA-900767615.1-CAAFZU010000193.1 | Bacteria | p__Firmicutes | c__Bacilli | o__Lactobacillales | f__Streptococcaceae | g__Streptococcus |  |
| 02342789 | Bacteria | p__Proteobacteria | c__Alphaproteobacteria | o__Caulobacterales | f__Caulobacteraceae | g__Phenylobacterium |  |
| MJ032-2-barcode62-umi36435bins-ubs-16 | Bacteria | p__Firmicutes | c__Clostridia | o__Clostridiales | f__Clostridiaceae |  |  |
| 04958504 | Bacteria | p__Actinobacteriota | c__Actinobacteria | o__Pseudonocardiales | f__Pseudonocardiaceae | g__Crossiella | s__uncultured_bacterium |
| 04944898 | Bacteria | p__Acidobacteriota | c__Blastocatellia | o__Blastocatellales | f__Blastocatellaceae | g__Stenotrophobacter | s__uncultured_soil |
| 955d1af8dd5ee8cebd23b58c45df76b4 | Bacteria | p__Actinobacteriota | c__Actinobacteria | o__Corynebacteriales | f__Corynebacteriaceae | g__Corynebacterium | s__Corynebacterium_riegelii |
| RS-GCF-013205055.1-NZ-JABMLF010000008.1 | Bacteria | p__Actinobacteriota | c__Actinobacteria | o__Micrococcales | f__Microbacteriaceae | g__Rathayibacter |  |
| RS-GCF-017776345.1-NZ-JAFIQO010000082.1 | Bacteria | p__Firmicutes | c__Clostridia | o__Lachnospirales | f__Lachnospiraceae | g__[Eubacterium]_hallii_group |  |
| aca8a7e54b58bdf5be31aaedd86ffffd | Bacteria | p__Actinobacteriota | c__Actinobacteria | o__Frankiales | f__Acidothermaceae | g__Acidothermus |  |
| 05030654 | Bacteria | p__Proteobacteria | c__Gammaproteobacteria | o__Burkholderiales | f__Oxalobacteraceae | g__Massilia |  |
| 97524378e10f831ef30cebfdbd9233cf | Bacteria | p__Actinobacteriota | c__Thermoleophilia | o__Gaiellales | f__uncultured | g__uncultured |  |
| 43004b551b30bdfc96bdaa1347153779 | Bacteria | p__Chloroflexi | c__OLB14 | o__OLB14 | f__OLB14 | g__OLB14 |  |
| a8316842ac348d9e575b90fb1d9b1504 | Bacteria | p__Cyanobacteria | c__Cyanobacteriia | o__Leptolyngbyales | f__Leptolyngbyaceae | g__Leptolyngbya_Es-Yyy1000 | s__Leptolyngbya_cf. |
| 05037027 | Bacteria | p__Firmicutes | c__Bacilli | o__Bacillales | f__Bacillaceae | g__Anoxybacillus |  |
| 9ac44f1b216636411c4dc68f3439c4fb | Bacteria | p__Firmicutes | c__Bacilli | o__Bacillales | f__Planococcaceae |  |  |
| a797f7f99962f73b05fe2b3713b60f7e | Bacteria | p__Proteobacteria | c__Alphaproteobacteria | o__Rhizobiales | f__Xanthobacteraceae | g__uncultured |  |
| 532533349c95b9bf438a0b6c6a34de20 | Bacteria | p__Firmicutes | c__Clostridia | o__Peptostreptococcales-Tissierellales | f__Peptostreptococcales-Tissierellales | g__Tissierella |  |
| 3c5e3ed4a5162ad5b4c6da79b3991ec2 | Bacteria | p__Proteobacteria | c__Alphaproteobacteria | o__Rhizobiales | f__Rhizobiaceae | g__Allorhizobium-Neorhizobium-Pararhizobium-Rhizobium |  |
| dff773bfdd8e703ddfedb56fdc4301d0 | Bacteria | p__Proteobacteria | c__Gammaproteobacteria | o__Pseudomonadales | f__Moraxellaceae | g__Acinetobacter |  |
| RS-GCF-013103535.1-NZ-WJEP01000018.1 | Bacteria | p__Firmicutes | c__Bacilli | o__Lactobacillales | f__Enterococcaceae | g__Enterococcus |  |
| 7ecc31e7a7c095b43781fad161c9260f | Bacteria | p__Proteobacteria | c__Alphaproteobacteria | o__Sphingomonadales | f__Sphingomonadaceae | g__Sphingobium |  |
| 15780644 | Bacteria | p__Firmicutes | c__Clostridia | o__Oscillospirales |  |  |  |
| 111499359127dfd760f2a7e6d056d03c | Bacteria | p__Actinobacteriota | c__Thermoleophilia | o__Solirubrobacterales | f__67-14 | g__67-14 |  |
| ca9096f354fa2b4f42f2a9b92f588ec2 | Bacteria | p__Proteobacteria | c__Gammaproteobacteria | o__Pseudomonadales | f__Moraxellaceae | g__Acinetobacter |  |
| ffadec3f6ae62940a3a0e69bc948dd87 | Bacteria | p__Proteobacteria | c__Gammaproteobacteria | o__Burkholderiales | f__Alcaligenaceae |  |  |
| 11725649 | Bacteria | p__Myxococcota | c__bacteriap25 | o__bacteriap25 | f__bacteriap25 | g__bacteriap25 | s__metagenome |
| a8f2dd0d446a638fc8be3ea750da9642 | Bacteria | p__Bacteroidota | c__Bacteroidia | o__Cytophagales | f__Microscillaceae | g__Chryseolinea |  |
| 16a693833cf7548234dcc2c9a3f6139d | Bacteria | p__Bacteroidota | c__Bacteroidia | o__Sphingobacteriales | f__Sphingobacteriaceae | g__Sphingobacterium | s__Sphingobacterium_bambusae |
| 038240da52b727f1b42d7f0fc37b51e1 | Bacteria | p__Proteobacteria | c__Alphaproteobacteria | o__Rhizobiales | f__Xanthobacteraceae |  |  |
| d93d1d9fef1589613b451aea142c4705 | Bacteria | p__Firmicutes | c__Bacilli | o__Lactobacillales | f__Leuconostocaceae | g__Weissella |  |
| d2122827f4ea912de2f481efd9e056c6 | Bacteria | p__Cyanobacteria | c__Cyanobacteriia | o__Leptolyngbyales | f__Leptolyngbyaceae | g__Leptolyngbya_Es-Yyy1000 | s__Leptolyngbya_cf. |
| RS-GCF-001078375.1-NZ-BBXI01000047.1 | Bacteria | p__Firmicutes | c__Negativicutes | o__Veillonellales-Selenomonadales | f__Veillonellaceae | g__Veillonella |  |
| 0598244f44828da31a408a985964f811 | Bacteria | p__Proteobacteria | c__Gammaproteobacteria | o__Burkholderiales | f__Burkholderiaceae | g__Burkholderia-Caballeronia-Paraburkholderia |  |
| EU626561 | Bacteria | p__Deinococcota | c__Deinococci | o__Deinococcales | f__Deinococcaceae | g__Deinococcus | s__Deinococcus_xinjiangensis |
| 54a92a0880cb769f442d21b6b3e934f0 | Bacteria | p__Acidobacteriota | c__Acidobacteriae | o__Acidobacteriales | f__uncultured | g__uncultured | s__uncultured_forest |
| 0295c4ab0254e43abc30429c53118805 | Bacteria | p__Gemmatimonadota | c__Gemmatimonadetes | o__Gemmatimonadales | f__Gemmatimonadaceae |  |  |
| cbd2af5686aad9a4a177c699ba02b421 | Bacteria | p__Proteobacteria | c__Alphaproteobacteria | o__Rhizobiales | f__Rhizobiales_Incertae_Sedis | g__uncultured |  |
| 05034141 | Bacteria | p__Actinobacteriota | c__Actinobacteria | o__Pseudonocardiales | f__Pseudonocardiaceae | g__Amycolatopsis |  |
| AF268968 | Bacteria | p__Proteobacteria | c__Gammaproteobacteria | o__Pseudomonadales | f__Pseudomonadaceae | g__Pseudomonas |  |
| DQ372921 | Bacteria | p__Proteobacteria | c__Alphaproteobacteria | o__Rhizobiales | f__Rhizobiaceae |  |  |
| d56dfab2f7b1a76f9cdcc9d84c717402 | Bacteria | p__Firmicutes | c__Bacilli | o__Bacillales | f__Bacillaceae |  |  |
| 58dba88ab145edbf18d771bb94d2c277 | Bacteria | p__Bacteroidota | c__Bacteroidia | o__Cytophagales | f__Hymenobacteraceae | g__Hymenobacter |  |
| 96f2cec734042dd6d160c102f0a17dc8 | Bacteria | p__Proteobacteria | c__Alphaproteobacteria | o__Rhizobiales | f__Rhizobiaceae |  |  |
| 9b406d26e3b49a6f02940436abc7e2ca | Bacteria | p__Actinobacteriota | c__Actinobacteria | o__Micrococcales | f__Microbacteriaceae |  |  |
| RS-GCF-900176865.1-NZ-LT839632.1 | Bacteria | p__Actinobacteriota | c__Actinobacteria | o__Corynebacteriales | f__Corynebacteriaceae | g__Corynebacterium |  |
| 8025ae0af569f9c6d4486f72aa76ca1b | Bacteria | p__Proteobacteria | c__Gammaproteobacteria | o__Xanthomonadales | f__Xanthomonadaceae | g__Stenotrophomonas |  |
| AF480590 | Bacteria | p__Actinobacteriota | c__Actinobacteria | o__Corynebacteriales | f__Mycobacteriaceae | g__Mycobacterium |  |
| e1e7be5a5729a61a818c6434a79f5a42 | Bacteria | p__Actinobacteriota | c__Actinobacteria | o__Micrococcales | f__Microbacteriaceae |  |  |
| 44ee2d902c736a5c1a2b528ee5bfa2d9 | Bacteria | p__Acidobacteriota | c__Vicinamibacteria | o__Vicinamibacterales | f__Vicinamibacteraceae | g__Vicinamibacter |  |
| 4cd983daca3d701ee82a4f7d78891918 | Bacteria | p__Proteobacteria | c__Gammaproteobacteria | o__Burkholderiales | f__uncultured | g__uncultured | s__uncultured_bacterium |
| 37d26732801ae4790411b08c41e0dbcd | Bacteria | p__Chloroflexi | c__Anaerolineae | o__Caldilineales | f__Caldilineaceae | g__uncultured |  |
| 08148046 | Bacteria | p__Proteobacteria | c__Alphaproteobacteria | o__Acetobacterales | f__Acetobacteraceae | g__Roseomonas |  |
| MN620432 | Bacteria | p__Proteobacteria | c__Alphaproteobacteria | o__Rhizobiales | f__Devosiaceae | g__Devosia |  |
| 05033654 | Bacteria | p__Actinobacteriota | c__Actinobacteria | o__Micrococcales | f__Intrasporangiaceae |  |  |
| MJ032-2-barcode56-umi2182bins-ubs-128 | Bacteria | p__Firmicutes | c__Clostridia | o__Clostridiales | f__Clostridiaceae |  |  |
| 05093297 | Bacteria | p__Proteobacteria | c__Alphaproteobacteria | o__Rhizobiales | f__Rhizobiaceae | g__Pseudochrobactrum |  |
| 05119377 | Bacteria | p__Methylomirabilota | c__Methylomirabilia | o__Rokubacteriales | f__Rokubacteriales | g__Rokubacteriales |  |
| 6659782b4d31a2fc867238e66e12ef7f | Bacteria | p__Bdellovibrionota | c__Bdellovibrionia | o__Bacteriovoracales | f__Bacteriovoracaceae | g__Peredibacter |  |
| MJ032-2-barcode56-umi1176bins-ubs-189 | Bacteria | p__Firmicutes | c__Clostridia | o__Clostridiales | f__Clostridiaceae |  |  |
| 9042ffbe353d6361a42c2f2f48dc2b1e | Bacteria | p__Proteobacteria | c__Gammaproteobacteria | o__Burkholderiales | f__Alcaligenaceae | g__Achromobacter |  |
| 82bbdcd60d913e4a4996cc8bce30c8b0 | Bacteria | p__Proteobacteria | c__Gammaproteobacteria | o__Pseudomonadales | f__Moraxellaceae | g__Acinetobacter |  |
| 03055929 | Bacteria | p__Myxococcota | c__Polyangia | o__Polyangiales | f__Polyangiaceae | g__Pajaroellobacter | s__uncultured_proteobacterium |
| 447b2ddd198f217bd73135f4ab130383 | Bacteria | p__Bacteroidota | c__Bacteroidia | o__Bacteroidales |  |  |  |
| EU912445 | Bacteria | p__Proteobacteria | c__Alphaproteobacteria | o__Rhizobiales | f__Beijerinckiaceae | g__Methylobacterium-Methylorubrum | s__Methylobacterium_gossipiicola |
| MJ032-2-barcode73-umi9961bins-ubs-47 | Bacteria | p__Proteobacteria | c__Gammaproteobacteria | o__Enterobacterales |  |  |  |
| MJ010-2-barcode64-umi102667bins-ubs-5 | Bacteria | p__Proteobacteria | c__Gammaproteobacteria | o__Pseudomonadales | f__Moraxellaceae | g__Acinetobacter |  |
| RS-GCF-011320095.1-NZ-JAANOY010000006.1 | Bacteria | p__Proteobacteria | c__Gammaproteobacteria | o__Xanthomonadales | f__Xanthomonadaceae |  |  |
| RS-GCF-018784445.1-NZ-WQOH01000081.1 | Bacteria | p__Firmicutes | c__Clostridia | o__Oscillospirales | f__Ruminococcaceae | g__Subdoligranulum |  |
| b02f35d93a150092b5b804edde5dadde | Bacteria | p__Proteobacteria | c__Gammaproteobacteria | o__Burkholderiales | f__Comamonadaceae |  |  |
| 1396d25a8d462c2f76ad2412bc488d91 | Bacteria | p__Firmicutes | c__Bacilli | o__Staphylococcales | f__Staphylococcaceae | g__Staphylococcus |  |
| G001908185_1 | Bacteria | p__Proteobacteria | c__Alphaproteobacteria | o__Rhizobiales | f__Xanthobacteraceae |  |  |
| G011960185_1 | Bacteria | p__Bacteroidota | c__Bacteroidia | o__Bacteroidales | f__Prevotellaceae | g__Alloprevotella | s__uncultured_Bacteroidales |
| 09d8c65d8556d7c067ab0cec805c9e0e | Bacteria | p__Firmicutes | c__Negativicutes | o__Veillonellales-Selenomonadales | f__Veillonellaceae | g__Megasphaera |  |
| 3199e460899cb8572243767c22673375 | Bacteria | p__Gemmatimonadota | c__Gemmatimonadetes | o__Gemmatimonadales | f__Gemmatimonadaceae | g__uncultured |  |
| 9666a6e7d5b1463cf2e8be830ac8828e | Bacteria | p__Proteobacteria | c__Alphaproteobacteria | o__Rhodobacterales | f__Rhodobacteraceae |  |  |
| 6e2ac20946a65d3744821afcef9dac13 | Bacteria | p__Firmicutes | c__Symbiobacteriia | o__Symbiobacteriales | f__Symbiobacteraceae | g__Symbiobacterium |  |
| 871584c07aebe76a706d96f68a395d92 | Bacteria | p__Proteobacteria | c__Alphaproteobacteria | o__Rhizobiales | f__Devosiaceae |  |  |
| 04962204 | Bacteria | p__Actinobacteriota | c__Actinobacteria | o__Propionibacteriales | f__Nocardioidaceae | g__Nocardioides |  |
| 6beb403d71dc3f550288d0cb55010230 | Bacteria | p__Proteobacteria | c__Gammaproteobacteria | o__Vibrionales | f__Vibrionaceae | g__Photobacterium |  |
| 84e05179737ec67c7086c8705e3943df | Bacteria | p__Firmicutes | c__Clostridia | o__Clostridia | f__Hungateiclostridiaceae |  |  |
| 807a7bf239d20c011c63c2c010a036b7 | Bacteria | p__Actinobacteriota | c__Actinobacteria | o__Pseudonocardiales | f__Pseudonocardiaceae | g__Prauserella |  |
| 02267088 | Bacteria | p__Proteobacteria | c__Alphaproteobacteria | o__Rhizobiales | f__Xanthobacteraceae |  |  |
| RS-GCF-008041935.1-NZ-VKAC01000026.1 | Bacteria | p__Actinobacteriota | c__Actinobacteria | o__Kineosporiales | f__Kineosporiaceae | g__Quadrisphaera |  |
| c55d7c9ce6ef32306dcb4d066c3d3e1f | Bacteria | p__Acidobacteriota | c__Acidobacteriae | o__Subgroup_2 | f__Subgroup_2 | g__Subgroup_2 |  |
| c12576a2ac44ac95a9396510bdc22174 | Bacteria | p__Firmicutes | c__Bacilli | o__Lactobacillales | f__Aerococcaceae | g__Aerococcus |  |
| 9c172d83dbdaa1acf6aeaad7f0ad5792 | Bacteria | p__Proteobacteria | c__Gammaproteobacteria | o__Enterobacterales |  |  |  |
| c051f1d7053e5c585320b097c18c8ea0 | Bacteria | p__Proteobacteria | c__Alphaproteobacteria | o__Sphingomonadales | f__Sphingomonadaceae |  |  |
| 85f4f06491bd13053605641ebd853aca | Bacteria | p__Firmicutes | c__Clostridia | o__Clostridiales | f__Clostridiaceae | g__Clostridium_sensu_stricto_1 |  |
| 69e8da2b66958df806e504af24421416 | Bacteria | p__Firmicutes | c__Bacilli | o__Erysipelotrichales | f__Erysipelotrichaceae | g__ZOR0006 |  |
| 85b50cf7ccb5b26e761c8b809023c3bd | Bacteria | p__Firmicutes | c__Bacilli | o__Lactobacillales | f__Aerococcaceae | g__Facklamia | s__uncultured_bacterium |
| HQ844973 | Bacteria | p__Actinobacteriota | c__Actinobacteria | o__Micrococcales | f__Microbacteriaceae | g__Microbacterium |  |
| MN099416 | Bacteria | p__Proteobacteria | c__Alphaproteobacteria | o__Sphingomonadales | f__Sphingomonadaceae | g__Sphingomonas |  |
| 4748940b96dcce979ad2b74e7bc31b60 | Bacteria | p__Myxococcota | c__Polyangia | o__Haliangiales | f__Haliangiaceae | g__Haliangium |  |
| MJ033-1-barcode32-umi41292bins-ubs-3 | Bacteria | p__WPS-2 | c__WPS-2 | o__WPS-2 | f__WPS-2 | g__WPS-2 | s__uncultured_forest |
| 2d68a72fd02d1f6b543a6dfa7c4962e8 | Bacteria | p__Chloroflexi | c__Chloroflexia | o__Thermomicrobiales | f__JG30-KF-CM45 | g__JG30-KF-CM45 |  |
| 36d9374a5f0fa8ad7f95e3f93288c9a8 | Bacteria | p__Firmicutes | c__Clostridia | o__Clostridiales | f__Clostridiaceae | g__Clostridium_sensu_stricto_1 |  |
| f3afbe0f982bc58ba2e3b1f1a6890985 | Bacteria | p__Proteobacteria | c__Gammaproteobacteria | o__Pseudomonadales | f__Moraxellaceae | g__Perlucidibaca | s__uncultured_bacterium |
| 368301d3025abde13012fb7a6608d250 | Bacteria | p__Proteobacteria | c__Alphaproteobacteria | o__Defluviicoccales | f__Defluviicoccaceae | g__Defluviicoccus | s__uncultured_Defluviicoccus |
| eb68249c3b09dac52eb6190da3ee70b0 | Bacteria | p__Firmicutes | c__Clostridia | o__Lachnospirales | f__Lachnospiraceae |  |  |
| 05032943 | Bacteria | p__Actinobacteriota | c__Actinobacteria | o__Corynebacteriales | f__Corynebacteriaceae | g__Corynebacterium |  |
| GB-GCA-008673455.1-RPUK01000107.1 | Bacteria | p__Firmicutes | c__Clostridia | o__Christensenellales | f__Christensenellaceae | g__Christensenellaceae_R-7_group |  |
| 6ce527a56d06df09fe2efdb16ab3a57c | Bacteria | p__Firmicutes | c__Bacilli | o__Lactobacillales | f__Enterococcaceae | g__Enterococcus |  |
| RS-GCF-900114015.1-NZ-FORN01000089.1 | Bacteria | p__Proteobacteria | c__Alphaproteobacteria | o__Caulobacterales | f__Caulobacteraceae | g__Caulobacter |  |
| 04876083 | Bacteria | p__Proteobacteria | c__Alphaproteobacteria | o__Rhizobiales | f__Xanthobacteraceae | g__uncultured |  |
| 1abb94d3dd065bf76464b2d67d9b99db | Bacteria | p__Proteobacteria | c__Gammaproteobacteria | o__Enterobacterales |  |  |  |
| 88e1dcc1f0a9b8a740becde72ced2943 | Bacteria | p__Proteobacteria | c__Alphaproteobacteria | o__Rhizobiales | f__Rhizobiaceae |  |  |
| a6cde76f03b303abc10724b3e288e642 | Bacteria | p__Proteobacteria | c__Gammaproteobacteria | o__Burkholderiales | f__Comamonadaceae |  |  |
| d532dba41c48cd2d14c2b4e851a2fb3a | Bacteria | p__Bacteroidota | c__Bacteroidia | o__Chitinophagales | f__Chitinophagaceae |  |  |
| 4861b2b30fe5ec23afc56bc72912daa5 | Bacteria | p__Proteobacteria | c__Alphaproteobacteria | o__Rhizobiales | f__Hyphomicrobiaceae | g__Pedomicrobium |  |
| 04992933 | Bacteria | p__Proteobacteria | c__Alphaproteobacteria | o__Rhizobiales | f__Xanthobacteraceae | g__uncultured |  |
| 0606299d587b33b8c25984cd3fb05803 | Bacteria | p__Desulfobacterota | c__uncultured | o__uncultured | f__uncultured | g__uncultured |  |
| 57ef71504c13f3d28214668145b44af5 | Bacteria | p__Proteobacteria | c__Gammaproteobacteria | o__Pseudomonadales | f__Moraxellaceae | g__Perlucidibaca | s__uncultured_bacterium |
| ff3b73c4b0a80e224b77e7e7af7827a6 | Bacteria | p__Acidobacteriota | c__Acidobacteriae | o__Bryobacterales | f__Bryobacteraceae | g__Bryobacter |  |
| 09ccf129fc7eaf5de9a59dc6b457defb | Bacteria | p__Proteobacteria | c__Gammaproteobacteria | o__Enterobacterales |  |  |  |
| MJ031-1-barcode42-umi25447bins-ubs-14 | Bacteria | p__Proteobacteria | c__Gammaproteobacteria | o__Cellvibrionales | f__Cellvibrionaceae | g__Cellvibrio |  |
| 04875870 | Bacteria | p__Proteobacteria | c__Gammaproteobacteria | o__Pseudomonadales | f__Moraxellaceae | g__Acinetobacter |  |
| RS-GCF-015704955.1-NZ-JADRJH010000001.1 | Bacteria | p__Bacteroidota | c__Bacteroidia | o__Flavobacteriales | f__Weeksellaceae | g__Elizabethkingia | s__Elizabethkingia_meningoseptica |
| 18560883 | Bacteria | p__Actinobacteriota | c__Actinobacteria | o__Micrococcales | f__Microbacteriaceae |  |  |
| 02356229 | Bacteria | p__Actinobacteriota | c__Actinobacteria | o__Propionibacteriales | f__Propionibacteriaceae | g__Cutibacterium |  |
| 20f48676e384c5fe3e32420a84933a66 | Bacteria | p__Methylomirabilota | c__Methylomirabilia | o__Rokubacteriales | f__Rokubacteriales | g__Rokubacteriales |  |
| AY362894 | Bacteria | p__Proteobacteria | c__Gammaproteobacteria | o__Pasteurellales | f__Pasteurellaceae | g__Muribacter | s__Muribacter_muris |
| RS-GCF-014873545.1-NZ-JADBDW010000001.1 | Bacteria | p__Proteobacteria | c__Alphaproteobacteria | o__Rhizobiales | f__Rhizobiaceae |  |  |
| d007b67bffd05898873e40297a8e2a41 | Bacteria | p__Actinobacteriota | c__Actinobacteria | o__Micrococcales | f__Microbacteriaceae | g__Microbacterium |  |
| 322a92ed20a065345fe0a05f4a7e8964 | Bacteria | p__Actinobacteriota |  |  |  |  |  |
| 3e86d25f41b5e588b8c873762f27ffd3 | Bacteria | p__Planctomycetota | c__Planctomycetes | o__Gemmatales | f__Gemmataceae | g__uncultured | s__uncultured_thermal |
| 4802b7d3ffe5590549b948abcf382fbd | Bacteria | p__Proteobacteria | c__Gammaproteobacteria | o__Pseudomonadales | f__Pseudomonadaceae | g__Pseudomonas |  |
| 3ca98f30e40ff4789d3b921be27d0132 | Bacteria | p__Chloroflexi | c__Ktedonobacteria | o__Ktedonobacterales | f__JG30-KF-AS9 | g__JG30-KF-AS9 | s__uncultured_bacterium |
| 80b4dbb2d597dca2f911568b66749550 | Bacteria | p__Verrucomicrobiota | c__Verrucomicrobiae | o__Chthoniobacterales |  |  |  |
| 02857443 | Bacteria | p__Chloroflexi | c__Chloroflexia | o__Chloroflexales | f__Roseiflexaceae | g__uncultured | s__uncultured_bacterium |
| 15096244 | Bacteria | p__Firmicutes | c__Bacilli | o__Erysipelotrichales | f__Erysipelotrichaceae | g__Ileibacterium | s__Ileibacterium_valens |
| c80a1dadfebd358f5d4090c26115a4f7 | Bacteria | p__Chloroflexi | c__Anaerolineae | o__1-20 | f__1-20 | g__1-20 | s__uncultured_Chloroflexia |
| 48c63af097fe31709f34f62b57f9827e | Bacteria | p__Proteobacteria | c__Gammaproteobacteria | o__Pseudomonadales | f__Moraxellaceae | g__Acinetobacter |  |
| MJ030-2-barcode65-umi48231bins-ubs-14 | Bacteria | p__Acidobacteriota | c__Acidobacteriae | o__Bryobacterales | f__Bryobacteraceae | g__Bryobacter |  |
| 4eb179becaf05de6012fe7ab4c8c4641 | Bacteria | p__Proteobacteria | c__Gammaproteobacteria | o__Pseudomonadales | f__Moraxellaceae |  |  |
| 19273347 | Bacteria | p__Actinobacteriota | c__Actinobacteria | o__Propionibacteriales | f__Nocardioidaceae |  |  |
| 18906383 | Bacteria | p__Proteobacteria | c__Gammaproteobacteria | o__Burkholderiales | f__Nitrosomonadaceae | g__MND1 |  |
| f821ea5c94b75d34f7b5d7548dad03aa | Bacteria | p__Proteobacteria | c__Gammaproteobacteria | o__Burkholderiales | f__Comamonadaceae |  |  |
| 8734c1e204e7c60d2bc6150fbfa03948 | Bacteria | p__Firmicutes | c__Negativicutes | o__Veillonellales-Selenomonadales | f__Veillonellaceae | g__Dialister |  |
| 02412065 | Bacteria | p__Proteobacteria | c__Alphaproteobacteria | o__Rhizobiales | f__Beijerinckiaceae | g__Methylobacterium-Methylorubrum |  |
| e1d09db5b9ea99f103fe250dafda9ec2 | Bacteria | p__Actinobacteriota | c__Acidimicrobiia | o__Microtrichales | f__Ilumatobacteraceae | g__CL500-29_marine_group |  |
| dbad21f3b04f768ec219df5ab3a52090 | Bacteria | p__Proteobacteria | c__Gammaproteobacteria | o__Steroidobacterales | f__Steroidobacteraceae | g__uncultured |  |
| 03fe0002671f3856b8e44c792c16339f | Bacteria | p__Firmicutes | c__Clostridia | o__Peptostreptococcales-Tissierellales | f__Peptostreptococcales-Tissierellales | g__Soehngenia |  |
| 04958497 | Bacteria | p__Actinobacteriota | c__Actinobacteria | o__Frankiales | f__uncultured | g__uncultured |  |
| RS-GCF-002575105.1-NZ-NUHI01000018.1 | Bacteria | p__Firmicutes | c__Bacilli | o__Bacillales | f__Bacillaceae | g__Bacillus |  |
| fe5913f5519cd332aa0ad3119241c986 | Bacteria | p__Proteobacteria | c__Alphaproteobacteria | o__Caulobacterales | f__Caulobacteraceae | g__Caulobacter |  |
| 04959755 | Bacteria | p__Proteobacteria | c__Gammaproteobacteria | o__Burkholderiales | f__Comamonadaceae |  |  |
| RS-GCF-000230975.2-NC-021172.1 | Bacteria | p__Proteobacteria | c__Alphaproteobacteria | o__Rhizobiales | f__Hyphomicrobiaceae | g__Hyphomicrobium | s__Hyphomicrobium_denitrificans |
| 5dbffc9329d341687dde4e5767f4bb4d | Bacteria | p__Bacteroidota | c__Bacteroidia | o__Flavobacteriales | f__Weeksellaceae | g__Cloacibacterium |  |
| f45269fb5f20f14e88c159354ac0dbba | Bacteria | p__Planctomycetota | c__Planctomycetes | o__Gemmatales | f__Gemmataceae | g__uncultured |  |
| a0f3b01311fbf90e4b92100527388816 | Bacteria | p__Firmicutes | c__Clostridia | o__Lachnospirales | f__Lachnospiraceae | g__Blautia |  |
| 20dd9c27fd684fa1206455dca814aacb | Bacteria | p__Nitrospirota | c__Nitrospiria | o__Nitrospirales | f__Nitrospiraceae | g__Nitrospira |  |
| 8f6603d9a0381a567fbe4d161f00fbf7 | Bacteria | p__Proteobacteria | c__Alphaproteobacteria | o__Rhizobiales | f__Hyphomicrobiaceae | g__Hyphomicrobium |  |
| 14872148 | Bacteria | p__Chloroflexi | c__Ktedonobacteria | o__Ktedonobacterales | f__JG30-KF-AS9 | g__JG30-KF-AS9 |  |
| 991bbb56a9ebcbf666dbca86e679f049 | Bacteria | p__Actinobacteriota | c__Actinobacteria | o__Micrococcales | f__Micrococcaceae |  |  |
| e8e97f76bed3a09d1e6fd6555e9441b9 | Bacteria | p__Bacteroidota | c__Bacteroidia | o__Chitinophagales | f__Saprospiraceae | g__uncultured |  |
| 552c19abc15f092afcee4107ec87fc1e | Bacteria | p__Actinobacteriota | c__Acidimicrobiia |  |  |  |  |
| 14cb2fda9784de3ed38a4d5778dfe88d | Bacteria | p__Actinobacteriota | c__Rubrobacteria | o__Rubrobacterales | f__Rubrobacteriaceae | g__Rubrobacter | s__Rubrobacter_bracarensis |
| 6f645b1a95dabc980d90f69622fc1fa5 | Bacteria | p__Actinobacteriota | c__Thermoleophilia |  |  |  |  |
| 4b9f5e6f13ad1e3ecb4f876d018489dd | Bacteria | p__Firmicutes | c__Bacilli | o__Erysipelotrichales | f__Erysipelotrichaceae | g__ZOR0006 |  |
| c893bd3cfff6dfc14094ca653f9f3de0 | Bacteria | p__Firmicutes | c__Bacilli | o__Staphylococcales | f__Staphylococcaceae |  |  |
| 08337543 | Bacteria | p__Proteobacteria | c__Alphaproteobacteria | o__Caulobacterales | f__Caulobacteraceae | g__Phenylobacterium |  |
| 190da33f7abd21ad202ad3334ef6b344 | Bacteria | p__Actinobacteriota | c__Actinobacteria | o__Micrococcales | f__Dermabacteraceae | g__Brachybacterium |  |
| 508b43058a764613b6a8d4aa621fe099 | Bacteria | p__Cyanobacteria | c__Vampirivibrionia | o__Gastranaerophilales | f__Gastranaerophilales | g__Gastranaerophilales | s__uncultured_bacterium |
| 04990724 | Bacteria | p__Verrucomicrobiota | c__Verrucomicrobiae | o__Chthoniobacterales | f__Chthoniobacteraceae | g__Candidatus_Udaeobacter |  |
| 23d80835818acd465781ec69ef35136c | Bacteria | p__Methylomirabilota | c__Methylomirabilia | o__Rokubacteriales | f__Rokubacteriales | g__Rokubacteriales |  |
| c1997607059db92f21d5ce14fa41465b | Bacteria | p__Myxococcota | c__Myxococcia | o__Myxococcales | f__Myxococcaceae |  |  |
| 04963853 | Bacteria | p__Firmicutes | c__Bacilli | o__Bacillales | f__Bacillaceae | g__Bacillus |  |
| 05012880 | Bacteria | p__Bacteroidota | c__Bacteroidia | o__Chitinophagales | f__Chitinophagaceae | g__Lacibacter | s__Lacibacter_cauensis |
| 17493986 | Bacteria | p__Chloroflexi | c__TK10 | o__TK10 | f__TK10 | g__TK10 | s__uncultured_Chloroflexus |
| 99b9dc16b46f3443cc0d93716d9d94ab | Bacteria | p__Proteobacteria | c__Alphaproteobacteria | o__Rhizobiales | f__Beijerinckiaceae | g__1174-901-12 | s__uncultured_bacterium |
| MJ034-1-barcode34-umi13355bins-ubs-25 | Bacteria | p__Proteobacteria | c__Gammaproteobacteria | o__Burkholderiales | f__Comamonadaceae |  |  |
| bd35ccc61b16078e5134f57ae286dc58 | Bacteria | p__Actinobacteriota | c__Actinobacteria | o__Micrococcales | f__Micrococcaceae |  |  |
| 13720334 | Bacteria | p__Gemmatimonadota | c__Gemmatimonadetes | o__Gemmatimonadales | f__Gemmatimonadaceae | g__Gemmatimonas |  |
| 05036793 | Bacteria | p__Firmicutes | c__Bacilli | o__Bacillales | f__Bacillaceae | g__Bacillus |  |
| 04907708 | Bacteria | p__Proteobacteria | c__Alphaproteobacteria | o__Caulobacterales | f__Caulobacteraceae | g__Brevundimonas |  |
| 7468dcdbf8ea603b4392fe15c045f2c5 | Bacteria | p__Actinobacteriota | c__Thermoleophilia | o__Solirubrobacterales | f__67-14 | g__67-14 |  |
| MJ030-1-barcode25-umi45931bins-ubs-12 | Bacteria | p__Firmicutes | c__Bacilli | o__Paenibacillales | f__Paenibacillaceae | g__Ammoniphilus |  |
| 91794aba58fcc43c23bec6aaa74f74ad | Bacteria | p__Acidobacteriota | c__Vicinamibacteria | o__Vicinamibacterales | f__uncultured | g__uncultured |  |
| 58133a6c56341011c3d9903345c2b461 | Bacteria | p__Acidobacteriota | c__Holophagae | o__Subgroup_7 | f__Subgroup_7 | g__Subgroup_7 |  |
| MH119720 | Bacteria | p__Actinobacteriota | c__Actinobacteria | o__Micrococcales | f__Micrococcaceae | g__Micrococcus |  |
| 15071048 | Bacteria | p__Acidobacteriota | c__Acidobacteriae | o__Bryobacterales | f__Bryobacteraceae | g__Bryobacter |  |
| RS-GCF-019075875.1-NZ-JAHOOZ010000001.1 | Bacteria | p__Proteobacteria | c__Alphaproteobacteria | o__Sphingomonadales | f__Sphingomonadaceae |  |  |
| 939ce517bad61e3c5a41f894155fb1ac | Bacteria | p__Actinobacteriota | c__Acidimicrobiia | o__Microtrichales | f__Ilumatobacteraceae |  |  |
| 6fd34af0efaf9ffaceec6b91bd58bb8e | Bacteria | p__Bacteroidota | c__Bacteroidia | o__Sphingobacteriales | f__Sphingobacteriaceae | g__Sphingobacterium |  |
| 058d350d797c2e956ac74893a26420af | Bacteria | p__Actinobacteriota | c__Acidimicrobiia | o__Microtrichales | f__uncultured | g__uncultured |  |
| 02350346 | Bacteria | p__Chloroflexi | c__KD4-96 | o__KD4-96 | f__KD4-96 | g__KD4-96 | s__uncultured_bacterium |
| dfba429ea8577cb19c35b8402ef5f1e5 | Bacteria | p__Firmicutes | c__Bacilli | o__Lactobacillales | f__Enterococcaceae | g__Enterococcus |  |
| 23503b77121b5c61cb990cc622313e21 | Bacteria | p__Acidobacteriota | c__Acidobacteriae | o__Bryobacterales | f__Bryobacteraceae | g__Bryobacter |  |
| db088114e3056952381ac01a869ed218 | Bacteria | p__Proteobacteria | c__Gammaproteobacteria | o__Xanthomonadales | f__Rhodanobacteraceae | g__Dyella |  |
| 9086de4c9320a01f36d7dc028f2664cb | Bacteria | p__Bacteroidota | c__Bacteroidia | o__Chitinophagales | f__Saprospiraceae | g__uncultured |  |
| HM027578 | Bacteria | p__Proteobacteria | c__Gammaproteobacteria | o__Burkholderiales | f__Comamonadaceae |  |  |
| a53c083dbf6da33d1557de324375be06 | Bacteria | p__Firmicutes | c__Clostridia | o__Lachnospirales | f__Lachnospiraceae |  |  |
| 19706901 | Bacteria | p__Firmicutes | c__Bacilli | o__Erysipelotrichales | f__Erysipelotrichaceae | g__Ileibacterium | s__Ileibacterium_valens |
| cbca6fcad3481f3ca76911b70bfbeaf0 | Bacteria | p__Bacteroidota | c__Bacteroidia | o__Chitinophagales | f__Saprospiraceae | g__uncultured |  |
| 05120837 | Bacteria | p__Acidobacteriota | c__Vicinamibacteria | o__Vicinamibacterales | f__Vicinamibacteraceae | g__Vicinamibacteraceae |  |
| 006145e65faec1ba359d48ff200f035f | Bacteria | p__Bacteroidota | c__Bacteroidia | o__Bacteroidales | f__Prevotellaceae | g__Prevotellaceae_UCG-003 |  |
| GB-GCA-002391865.1-DGFV01000010.1 | Bacteria | p__Bacteroidota | c__Bacteroidia | o__Flavobacteriales | f__Weeksellaceae | g__Chryseobacterium |  |
| 13708322 | Bacteria | p__Gemmatimonadota | c__Gemmatimonadetes | o__Gemmatimonadales | f__Gemmatimonadaceae |  |  |
| MJ006-1-barcode26-umi118135bins-ubs-5 | Bacteria | p__Firmicutes | c__Clostridia | o__Lachnospirales | f__Lachnospiraceae | g__Agathobacter |  |
| 04964812 | Bacteria | p__Firmicutes | c__Bacilli | o__Alicyclobacillales | f__Alicyclobacillaceae | g__Tumebacillus | s__uncultured_Alicyclobacillaceae |
| d78c6d94ff9c1fbc8d300eded885568e | Bacteria | p__Acidobacteriota | c__Vicinamibacteria | o__Vicinamibacterales | f__uncultured | g__uncultured |  |
| MJ007-1-barcode42-umi7931bins-ubs-38 | Bacteria | p__Proteobacteria | c__Gammaproteobacteria | o__Pseudomonadales | f__Moraxellaceae | g__Acinetobacter |  |
| b3820ac236b614c0c7aea1dfdf6e1a68 | Bacteria | p__Bacteroidota | c__Bacteroidia | o__Bacteroidales | f__Tannerellaceae | g__Parabacteroides |  |
| MJ006-2-barcode58-umi74372bins-ubs-7 | Bacteria | p__Bacteroidota | c__Bacteroidia | o__Bacteroidales | f__Rikenellaceae | g__Alistipes | s__Alistipes_finegoldii |
| MJ006-1-barcode38-umi93891bins-ubs-4 | Bacteria | p__Firmicutes | c__Clostridia | o__Lachnospirales | f__Lachnospiraceae |  |  |
| MJ032-2-barcode60-umi86027bins-ubs-4 | Bacteria | p__Proteobacteria | c__Gammaproteobacteria | o__Burkholderiales | f__Methylophilaceae | g__Methylophilus |  |
| RS-GCF-900457365.1-NZ-UGYW01000001.1 | Bacteria | p__Bacteroidota | c__Bacteroidia | o__Sphingobacteriales | f__Sphingobacteriaceae | g__Sphingobacterium | s__Sphingobacterium_spiritivorum |
| RS-GCF-013282575.1-NZ-JAAAYE010000118.1 | Bacteria | p__Proteobacteria | c__Gammaproteobacteria | o__Burkholderiales | f__Burkholderiaceae | g__Burkholderia-Caballeronia-Paraburkholderia |  |
| c7813cf8950f504ea365fcf9ccd1c586 | Bacteria | p__Bacteroidota | c__Bacteroidia | o__Flavobacteriales | f__NS9_marine_group | g__NS9_marine_group |  |
| KF447772 | Bacteria | p__Firmicutes | c__Clostridia | o__Oscillospirales | f__Oscillospiraceae | g__Colidextribacter |  |
| 04966832 | Bacteria | p__Chloroflexi | c__KD4-96 | o__KD4-96 | f__KD4-96 | g__KD4-96 |  |
| MJ020-1-barcode31-umi47239bins-ubs-9 | Bacteria | p__Acidobacteriota | c__Blastocatellia | o__Pyrinomonadales | f__Pyrinomonadaceae | g__RB41 |  |
| 17663945 | Bacteria | p__Actinobacteriota | c__Coriobacteriia | o__Coriobacteriales | f__Eggerthellaceae |  |  |
| e5efd8dc43014c666f8e1b5241bc5d53 | Bacteria | p__Chloroflexi | c__KD4-96 | o__KD4-96 | f__KD4-96 | g__KD4-96 |  |
| MJ033-1-barcode26-umi40540bins-ubs-3 | Bacteria | p__Proteobacteria | c__Gammaproteobacteria | o__Enterobacterales | f__Morganellaceae | g__Morganella |  |
| MJ006-2-barcode50-umi239810bins-ubs-3 | Bacteria | p__Proteobacteria | c__Gammaproteobacteria | o__Pasteurellales | f__Pasteurellaceae | g__Haemophilus |  |
| 0c4be7341b6c7d3e54a53abb94d68df4 | Bacteria | p__Acidobacteriota | c__Blastocatellia | o__Blastocatellales | f__Blastocatellaceae |  |  |
| d41e141a7cc5b27363740d09fb6026ee | Bacteria | p__Actinobacteriota | c__Actinobacteria | o__Corynebacteriales |  |  |  |
| 216135ab6d8b892fae09826e7c0cfb3a | Bacteria | p__Bacteroidota | c__Bacteroidia | o__Bacteroidales | f__Bacteroidaceae | g__Bacteroides |  |
| 04989057 | Bacteria | p__Proteobacteria | c__Gammaproteobacteria | o__Gammaproteobacteria_Incertae_Sedis | f__Unknown_Family | g__Acidibacter | s__uncultured_Steroidobacter |
| 10157143 | Bacteria | p__Bacteroidota | c__Bacteroidia | o__Flavobacteriales | f__Flavobacteriaceae | g__Flavobacterium | s__Flavobacterium_ceti |
| 1e451ef9f5a177e9f4818b0e3f89dcf5 | Bacteria | p__Proteobacteria | c__Alphaproteobacteria | o__Rhizobiales | f__Xanthobacteraceae | g__uncultured |  |
| GB-GCA-018376935.1-JAHAAJ010000118.1 | Bacteria | p__Firmicutes | c__Clostridia | o__Peptostreptococcales-Tissierellales | f__Peptostreptococcaceae | g__Intestinibacter |  |
| 047588783ccb3152c7f3f6d4f2e3ad3d | Bacteria | p__Firmicutes | c__Syntrophomonadia | o__Syntrophomonadales | f__Syntrophomonadaceae | g__Syntrophomonas | s__anaerobic_digester |
| 2bd2ee6147f0f6a927748b06fb71871d | Bacteria | p__Proteobacteria | c__Alphaproteobacteria | o__Rhizobiales | f__Rhizobiaceae |  |  |
| RS-GCF-004795405.1-NZ-QXNK01000067.1 | Bacteria | p__Proteobacteria | c__Gammaproteobacteria | o__Pasteurellales | f__Pasteurellaceae | g__Rodentibacter |  |
| 7c6ecd3c1366fc9ccaad85ebd526120c | Bacteria | p__Actinobacteriota | c__Actinobacteria | o__Micrococcales | f__Micrococcaceae |  |  |
| AJ430193 | Bacteria | p__Actinobacteriota | c__Actinobacteria | o__Frankiales | f__Geodermatophilaceae | g__Blastococcus |  |
| d478302bb2fe3fd43656315eb5d0b0fc | Bacteria | p__Myxococcota | c__Myxococcia | o__Myxococcales | f__Myxococcaceae | g__uncultured |  |
| 08704839 | Bacteria | p__Myxococcota | c__Polyangia | o__Haliangiales | f__Haliangiaceae | g__Haliangium |  |
| ef7ed454a2ff3972b1f11108eb9d6358 | Bacteria | p__Actinobacteriota | c__Acidimicrobiia | o__IMCC26256 | f__IMCC26256 | g__IMCC26256 |  |
| 207992d7ea68faa2ad63fd750d210332 | Bacteria | p__Actinobacteriota | c__Actinobacteria | o__Bifidobacteriales | f__Bifidobacteriaceae | g__Bifidobacterium |  |
| 3fb7eb77b74a837491f8b310033add73 | Bacteria | p__Actinobacteriota | c__Actinobacteria | o__Micrococcales | f__Microbacteriaceae | g__Microbacterium |  |
| MJ030-1-barcode25-umi76005bins-ubs-5 | Bacteria | p__Firmicutes | c__Bacilli | o__Bacillales | f__Bacillaceae | g__Bacillus |  |
| RS-GCF-002141235.1-NZ-NGLK01000001.1--4 | Bacteria | p__Firmicutes | c__Bacilli | o__Lactobacillales | f__Enterococcaceae | g__Enterococcus |  |
| 4db9fbb33b8172dc5944059a32fb3acb | Bacteria | p__Proteobacteria | c__Gammaproteobacteria | o__Enterobacterales |  |  |  |
| AF389342 | Bacteria | p__Actinobacteriota | c__Actinobacteria | o__Micrococcales | f__Microbacteriaceae | g__Agromyces | s__Agromyces_aurantiacus |
| GB-GCA-006228165.1-CP040827.1--2 | Bacteria | p__Proteobacteria | c__Gammaproteobacteria | o__Enterobacterales | f__Enterobacteriaceae |  |  |
| G000826105_1 | Bacteria | p__Proteobacteria | c__Gammaproteobacteria | o__Pseudomonadales | f__Pseudomonadaceae | g__Pseudomonas |  |
| MJ020-1-barcode29-umi101025bins-ubs-3 | Bacteria | p__Proteobacteria | c__Alphaproteobacteria | o__Rhodobacterales | f__Rhodobacteraceae |  |  |
| 04988917 | Bacteria | p__Proteobacteria | c__Gammaproteobacteria | o__Pseudomonadales | f__Pseudomonadaceae | g__Pseudomonas |  |
| a4f498ee02b5a27089ce6ddce505577e | Bacteria | p__Proteobacteria | c__Alphaproteobacteria | o__Rhizobiales | f__Rhizobiaceae | g__Allorhizobium-Neorhizobium-Pararhizobium-Rhizobium |  |
| 412099b1b847c08af7f271dd0847fb4f | Bacteria | p__Actinobacteriota | c__Thermoleophilia | o__Gaiellales |  |  |  |
| 884fb651ad3c6e7d5082b15ab7d1436a | Bacteria | p__Firmicutes | c__Incertae_Sedis | o__DTU014 | f__DTU014 | g__DTU014 |  |
| 4269251fba877108149d30e4cdc4f638 | Bacteria | p__Firmicutes | c__Bacilli | o__Alicyclobacillales | f__Alicyclobacillaceae | g__Alicyclobacillus |  |
| RS-GCF-010119385.1-NZ-VFIE01000045.1 | Bacteria | p__Proteobacteria | c__Gammaproteobacteria | o__Xanthomonadales | f__Xanthomonadaceae | g__Stenotrophomonas |  |
| f8bb79e186b95d79460062df5d8caedd | Bacteria | p__Firmicutes | c__Bacilli | o__Lactobacillales | f__Carnobacteriaceae | g__Atopostipes |  |
| 04911091 | Bacteria | p__Proteobacteria | c__Gammaproteobacteria | o__Xanthomonadales | f__Xanthomonadaceae | g__Lysobacter |  |
| c5eeb647f3d3fb34a610e40b3e367e7b | Bacteria | p__Actinobacteriota | c__Actinobacteria | o__Frankiales |  |  |  |
| 03711097 | Bacteria | p__Planctomycetota | c__BD7-11 | o__BD7-11 | f__BD7-11 | g__BD7-11 | s__uncultured_bacterium |
| RS-GCF-009696265.1-NZ-VUMH01000026.1 | Bacteria | p__Desulfobacterota | c__Desulfovibrionia | o__Desulfovibrionales | f__Desulfovibrionaceae | g__Desulfovibrio | s__Brachyspira_sp. |
| RS-GCF-002165145.1-NZ-NFZP01000154.1 | Bacteria | p__Actinobacteriota | c__Actinobacteria | o__Micrococcales | f__Cellulomonadaceae | g__Cellulomonas |  |
| RS-GCF-003024675.1-NZ-PYLP01000049.1 | Bacteria | p__Firmicutes | c__Bacilli | o__Erysipelotrichales | f__Erysipelatoclostridiaceae | g__Erysipelotrichaceae_UCG-003 |  |
| 5002f9c62f207aa7d2e964f1ad91acbd | Bacteria | p__Chloroflexi | c__Anaerolineae | o__Caldilineales | f__Caldilineaceae | g__uncultured |  |
| 03173476 | Bacteria | p__Planctomycetota | c__Phycisphaerae | o__Tepidisphaerales | f__WD2101_soil_group | g__WD2101_soil_group | s__uncultured_bacterium |
| 84c21f17bb509237d3299110843d1a2a | Bacteria | p__Actinobacteriota | c__Actinobacteria | o__Corynebacteriales | f__Mycobacteriaceae | g__Mycobacterium |  |
| dfb3a6423aee9f23357e1e3ea53a8702 | Bacteria | p__Proteobacteria | c__Alphaproteobacteria | o__Rhizobiales |  |  |  |
| 04922138 | Bacteria | p__Cyanobacteria | c__Cyanobacteriia | o__Cyanobacteriales | f__Phormidiaceae | g__Phormidium_IAM_M-71 | s__uncultured_bacterium |
| 7c8eaef1e21bd539d4b6f94fe281b0a5 | Bacteria | p__Acidobacteriota | c__Acidobacteriae | o__Bryobacterales | f__Bryobacteraceae | g__Bryobacter | s__uncultured_actinobacterium |
| GB-GCA-014384785.1-JACRSU010000009.1 | Bacteria | p__Firmicutes | c__Clostridia |  |  |  |  |
| 04767cbbc4ff9b9a2ff031222f122072 | Bacteria | p__Proteobacteria | c__Gammaproteobacteria | o__Pseudomonadales | f__Moraxellaceae | g__Acinetobacter |  |
| ee4bdbdc0af1da88e4f1ec1e1964930d | Bacteria | p__Bacteroidota | c__Bacteroidia | o__Bacteroidales | f__Dysgonomonadaceae | g__Petrimonas |  |
| a19b13c675ae9d1fcb2e7a17d2eed67a | Bacteria | p__Actinobacteriota | c__Actinobacteria | o__Micrococcales | f__Microbacteriaceae | g__Microbacterium |  |
| 11ffb34f656c408fdcdaf94fe63c539c | Bacteria | p__Bacteroidota | c__Bacteroidia | o__Chitinophagales | f__Chitinophagaceae | g__Sediminibacterium |  |
| 56748531766bc2b63d988f505c3157dc | Bacteria | p__Planctomycetota | c__Planctomycetes | o__Gemmatales | f__Gemmataceae |  |  |
| ab695d56a0eec68b8a5f596296be328a | Bacteria | p__Chloroflexi | c__Ktedonobacteria | o__Ktedonobacterales | f__JG30-KF-AS9 | g__JG30-KF-AS9 | s__uncultured_bacterium |
| 04903000 | Bacteria | p__Acidobacteriota | c__Vicinamibacteria | o__Vicinamibacterales | f__Vicinamibacteraceae | g__Vicinamibacteraceae |  |
| 08208799 | Bacteria | p__Proteobacteria | c__Alphaproteobacteria | o__Rhizobiales | f__Beijerinckiaceae | g__Methylobacterium-Methylorubrum |  |
| 24ae97d1a92f181caa0eead94e3762b7 | Bacteria | p__Proteobacteria | c__Gammaproteobacteria | o__Burkholderiales | f__Neisseriaceae | g__uncultured | s__uncultured_bacterium |
| ecfeda3d4bed82fd5eb6dca2d34d5a13 | Bacteria | p__Bacteroidota | c__Bacteroidia | o__Bacteroidales | f__Rikenellaceae | g__Rikenellaceae_RC9_gut_group | s__uncultured_bacterium |
| MJ020-1-barcode41-umi1272bins-ubs-182 | Bacteria | p__Firmicutes | c__Clostridia | o__Clostridiales | f__Clostridiaceae | g__Clostridium_sensu_stricto_12 | s__Clostridium_magnum |
| 949405090d61dd7b4642dfd53acec8d3 | Bacteria | p__Proteobacteria | c__Gammaproteobacteria | o__Enterobacterales | f__Erwiniaceae |  |  |
| GB-GCA-001672155.1-LJOS01000152.1 | Bacteria | p__Cyanobacteria | c__Cyanobacteriia | o__Cyanobacteriales | f__Nostocaceae | g__Aphanizomenon_MDT14a | s__Aphanizomenon_flos-aquae |
| MJ006-1-barcode49-umi168641bins-ubs-3 | Bacteria | p__Bacteroidota | c__Bacteroidia | o__Bacteroidales | f__Bacteroidaceae | g__Bacteroides | s__Bacteroides_sartorii |
| 14870604 | Bacteria | p__Chloroflexi | c__Ktedonobacteria | o__Ktedonobacterales | f__JG30-KF-AS9 | g__JG30-KF-AS9 | s__uncultured_bacterium |
| RS-GCF-014930775.1-NZ-JADBRS010000011.1 | Bacteria | p__Proteobacteria | c__Gammaproteobacteria | o__Enterobacterales |  |  |  |
| e51cb202db08b93c5d1686e01c0a5e4c | Bacteria | p__Actinobacteriota | c__Thermoleophilia | o__Gaiellales |  |  |  |
| bf7ba57a541797401a07f7b83c0af307 | Bacteria | p__Firmicutes | c__Negativicutes | o__Acidaminococcales | f__Acidaminococcaceae | g__Acidaminococcus | s__Acidaminococcus_sp. |
| RS-GCF-014203735.1-NZ-JACHJQ010000021.1 | Bacteria | p__Actinobacteriota | c__Actinobacteria | o__Pseudonocardiales | f__Pseudonocardiaceae | g__Actinophytocola |  |
| RS-GCF-000319305.2-NZ-AMWJ02000004.1 | Bacteria | p__Proteobacteria | c__Gammaproteobacteria | o__Pseudomonadales | f__Pseudomonadaceae | g__Pseudomonas |  |
| 14c33c3f07d0f790e8f187c68f02df7d | Bacteria | p__Bacteroidota | c__Bacteroidia | o__Bacteroidales | f__Prolixibacteraceae |  |  |
| GB-GCA-000962895.1-KQ031358.1 | Bacteria | p__Proteobacteria | c__Gammaproteobacteria | o__Pseudomonadales | f__Pseudomonadaceae | g__Pseudomonas |  |
| f963504261f5208f7e343c27382fe3a1 | Bacteria | p__Gemmatimonadota | c__Gemmatimonadetes | o__Gemmatimonadales | f__Gemmatimonadaceae |  |  |
| 127efbc0be0f0900737b567d59d49e88 | Bacteria | p__Proteobacteria | c__Alphaproteobacteria |  |  |  |  |
| RS-GCF-009866805.1-NZ-SIZW01000030.1 | Bacteria | p__Proteobacteria | c__Gammaproteobacteria | o__Burkholderiales | f__Alcaligenaceae | g__Verticiella |  |
| 1433ef93ad808341daf2593d31d575e1 | Bacteria | p__Proteobacteria | c__Gammaproteobacteria | o__Burkholderiales | f__SC-I-84 | g__SC-I-84 |  |
| a913f755d3c46a61a89225c7b7ee10de | Bacteria | p__Verrucomicrobiota | c__Chlamydiae | o__Chlamydiales | f__Parachlamydiaceae | g__Neochlamydia | s__metagenome |
| f5da9909073cf25e20ac510708f26deb | Bacteria | p__Firmicutes | c__Bacilli | o__Erysipelotrichales | f__Erysipelotrichaceae | g__Ileibacterium | s__Ileibacterium_valens |
| c0d1ead00b5796f23a50650a6e41df4f | Bacteria | p__Bacteroidota | c__Bacteroidia | o__Bacteroidales | f__Rikenellaceae | g__DMER64 |  |
| 05029821 | Bacteria | p__Actinobacteriota | c__Actinobacteria | o__Corynebacteriales | f__Mycobacteriaceae | g__Mycobacterium |  |
| MJ032-1-barcode26-umi67278bins-ubs-3 | Bacteria | p__Proteobacteria | c__Gammaproteobacteria | o__Burkholderiales | f__TRA3-20 | g__TRA3-20 |  |
| 2541b19f5260c7b0d2276597eff23630 | Bacteria | p__Acidobacteriota | c__Vicinamibacteria | o__Vicinamibacterales | f__uncultured | g__uncultured |  |
| 7d89fcb83e8e80a17aecbe62ef6ad9a2 | Bacteria | p__Proteobacteria | c__Gammaproteobacteria | o__Burkholderiales | f__Alcaligenaceae | g__Achromobacter |  |
| MJ030-1-barcode25-umi107063bins-ubs-3 | Bacteria | p__Chloroflexi | c__Anaerolineae | o__Anaerolineales | f__Anaerolineaceae | g__UTCFX1 |  |
| 58f87f573d9b84b2d1ba9bce7e6f0c73 | Bacteria | p__Fusobacteriota | c__Fusobacteriia | o__Fusobacteriales | f__Fusobacteriaceae | g__Fusobacterium |  |
| 11182503 | Bacteria | p__Bacteroidota | c__Bacteroidia | o__Bacteroidales | f__Rikenellaceae | g__Rikenellaceae_RC9_gut_group | s__unidentified |
| 02339582 | Bacteria | p__Proteobacteria | c__Alphaproteobacteria | o__Rhizobiales | f__Rhizobiaceae | g__Allorhizobium-Neorhizobium-Pararhizobium-Rhizobium | s__[Pseudomonas]_geniculata |
| 03862167 | Bacteria | p__Cyanobacteria | c__Cyanobacteriia | o__Thermosynechococcales | f__Thermosynechococcaceae | g__Synechococcus_IR11 | s__Pseudanabaena_galeata |
| 09aae3da9aee53bfb3f9b6497cf37b9f | Bacteria | p__Proteobacteria | c__Gammaproteobacteria | o__Burkholderiales | f__Nitrosomonadaceae | g__mle1-7 | s__uncultured_beta |
| 04911150 | Bacteria | p__Proteobacteria | c__Gammaproteobacteria | o__Xanthomonadales | f__Xanthomonadaceae | g__Stenotrophomonas | s__Stenotrophomonas_acidaminiphila |
| 90ff9e6168ea0fc50d92fa3641687867 | Bacteria | p__Firmicutes | c__Clostridia | o__Clostridiales | f__Clostridiaceae | g__Clostridium_sensu_stricto_1 |  |
| 18175019 | Bacteria | p__Actinobacteriota | c__Thermoleophilia | o__Gaiellales | f__uncultured | g__uncultured | s__uncultured_Rubrobacteria |
| 79453ced78fa98fb1cf87025987b21ab | Bacteria | p__Proteobacteria | c__Alphaproteobacteria | o__Sphingomonadales | f__Sphingomonadaceae | g__Sphingomonas |  |
| 6940ae1b5c982146a3eb15d37a0a0a14 | Bacteria | p__Proteobacteria | c__Gammaproteobacteria | o__Burkholderiales | f__Nitrosomonadaceae | g__Ellin6067 |  |
| 15341373 | Bacteria | p__Actinobacteriota | c__Thermoleophilia | o__Solirubrobacterales | f__67-14 | g__67-14 |  |
| RS-GCF-002847925.1-NZ-PKHW01000010.1 | Bacteria | p__Firmicutes | c__Negativicutes | o__Veillonellales-Selenomonadales | f__Veillonellaceae | g__Veillonella |  |
| MJ030-1-barcode25-umi18090bins-ubs-26 | Bacteria | p__Firmicutes | c__Clostridia | o__Clostridiales | f__Clostridiaceae | g__Clostridium_sensu_stricto_8 | s__uncultured_bacterium |
| 71bfd5b616339332f9ecd5b8448fb625 | Bacteria | p__Proteobacteria | c__Gammaproteobacteria | o__Burkholderiales | f__Burkholderiaceae | g__Ralstonia |  |
| 04943727 | Bacteria | p__Acidobacteriota | c__Blastocatellia | o__Blastocatellales | f__Blastocatellaceae |  |  |
| f35e789050140487cf5e8ddd55c93223 | Bacteria | p__Proteobacteria | c__Alphaproteobacteria | o__Rhizobiales | f__Beijerinckiaceae |  |  |
| fac2246ce46bc153684931664a526436 | Bacteria | p__Proteobacteria | c__Gammaproteobacteria | o__Enterobacterales | f__Enterobacteriaceae |  |  |
| cc74ce58e212bcb3f2f7963912c3f8e0 | Bacteria | p__Chloroflexi | c__Anaerolineae | o__SBR1031 | f__SBR1031 | g__SBR1031 | s__anaerobic_bacterium |
| 09094520 | Bacteria | p__Nitrospirota | c__Nitrospiria | o__Nitrospirales | f__Nitrospiraceae | g__Nitrospira |  |
| FN178400 | Bacteria | p__Actinobacteriota | c__Actinobacteria | o__Propionibacteriales | f__Nocardioidaceae | g__Nocardioides |  |
| KY194796 | Bacteria | p__Proteobacteria | c__Gammaproteobacteria | o__Xanthomonadales | f__Rhodanobacteraceae | g__Dyella |  |
| 7b036928dd109365c3592188b15be227 | Bacteria | p__Actinobacteriota | c__Thermoleophilia | o__Solirubrobacterales | f__67-14 | g__67-14 |  |
| AB703239 | Bacteria | p__Proteobacteria | c__Alphaproteobacteria | o__Rhizobiales | f__Beijerinckiaceae | g__Methylobacterium-Methylorubrum |  |
| 28a2952d157cc3838cc4843be7ff11f6 | Bacteria | p__Proteobacteria | c__Gammaproteobacteria | o__Xanthomonadales | f__Xanthomonadaceae |  |  |
| AB245347 | Bacteria | p__Proteobacteria | c__Alphaproteobacteria | o__Sphingomonadales | f__Sphingomonadaceae | g__Sphingomonas |  |
| 17258661 | Bacteria | p__Firmicutes | c__Clostridia | o__Clostridia_vadinBB60_group | f__Clostridia_vadinBB60_group | g__Clostridia_vadinBB60_group |  |
| 26b93b8c9266b135822d190e10e34824 | Bacteria | p__Firmicutes | c__Bacilli | o__Paenibacillales | f__Paenibacillaceae | g__Paenibacillus |  |
| 7901a3f4c624fff822f06588b3bfa849 | Bacteria | p__Proteobacteria | c__Alphaproteobacteria | o__Rhizobiales | f__Hyphomicrobiaceae | g__Pedomicrobium |  |
| 198bb3a25cb705c17ed5c04698842a98 | Bacteria | p__Proteobacteria | c__Gammaproteobacteria | o__Pseudomonadales | f__Moraxellaceae | g__Acinetobacter |  |
| 04907463 | Bacteria | p__Proteobacteria | c__Alphaproteobacteria | o__Caulobacterales | f__Caulobacteraceae | g__Brevundimonas |  |
| f650775d7253c7adf93a0a105a57be29 | Bacteria | p__Proteobacteria | c__Gammaproteobacteria | o__Enterobacterales | f__Enterobacteriaceae |  |  |
| 17769962 | Bacteria | p__Armatimonadota | c__Chthonomonadetes | o__Chthonomonadales | f__Chthonomonadales | g__Chthonomonadales | s__uncultured_bacterium |
| 7fadd439718b0087bd73d89f019b1401 | Bacteria | p__Proteobacteria | c__Gammaproteobacteria | o__Alteromonadales | f__Alteromonadaceae | g__Rheinheimera |  |
| d87b9569b43697d5c3efa08daf2293b4 | Bacteria | p__Firmicutes | c__Bacilli | o__Lactobacillales | f__Lactobacillaceae | g__Lactobacillus |  |
| 401839a34dc609651211973b60ff052a | Bacteria | p__Firmicutes | c__Bacilli | o__Paenibacillales | f__Paenibacillaceae | g__Paenibacillus | s__Paenibacillus_cucumis |
| dc1e6a7d56faa32b646a4ce79663f077 | Bacteria | p__Firmicutes | c__Bacilli | o__Paenibacillales | f__Paenibacillaceae | g__Paenibacillus |  |
| 65dc2823ecf51aafda2de40c84db4490 | Bacteria | p__Firmicutes | c__Clostridia | o__Lachnospirales | f__Lachnospiraceae | g__[Ruminococcus]_torques_group |  |
| 601062312dbffead2f73828113aad715 | Bacteria | p__Verrucomicrobiota | c__Verrucomicrobiae | o__Chthoniobacterales | f__Chthoniobacteraceae | g__Candidatus_Udaeobacter |  |
| 207c64a4f4a9359698be864e2d6a677b | Bacteria | p__Firmicutes | c__Clostridia | o__Peptostreptococcales-Tissierellales | f__Sedimentibacteraceae | g__Sedimentibacter | s__uncultured_bacterium |
| 02936971 | Bacteria | p__Methylomirabilota | c__Methylomirabilia | o__Rokubacteriales | f__Rokubacteriales | g__Rokubacteriales | s__metagenome |
| 04994871 | Bacteria | p__Proteobacteria | c__Alphaproteobacteria | o__Rhizobiales | f__Rhizobiaceae | g__Aureimonas |  |
| 3046829e85d35c75b42a2bc5c93a481d | Bacteria | p__Chloroflexi | c__Anaerolineae | o__Caldilineales | f__Caldilineaceae | g__uncultured |  |
| 02850893 | Bacteria |  |  |  |  |  |  |
| 11017961 | Bacteria | p__Bacteroidota | c__Bacteroidia | o__Bacteroidales | f__Muribaculaceae | g__Muribaculaceae |  |
| RS-GCF-015844595.1-NZ-LDFY01000100.1 | Bacteria | p__Firmicutes | c__Bacilli | o__Brevibacillales | f__Brevibacillaceae | g__Brevibacillus |  |
| MJ030-2-barcode58-umi38922bins-ubs-18 | Bacteria | p__Bacteroidota | c__Bacteroidia |  |  |  |  |
| f4a261549488a455057b1d0f8e568e36 | Bacteria | p__Myxococcota | c__Polyangia | o__Haliangiales | f__Haliangiaceae | g__Haliangium |  |
| 6e577bb3a5d3c346a45d8899bd3c8bb9 | Bacteria | p__Firmicutes | c__Clostridia | o__Lachnospirales | f__Lachnospiraceae | g__Lachnoclostridium |  |
| 03dbc22e6fdc48ae1ff00deec6bd6907 | Bacteria | p__Acidobacteriota | c__Blastocatellia | o__Blastocatellales | f__Blastocatellaceae |  |  |
| 794b4352c7a7e5f0a9b0ce0184c9eee0 | Bacteria | p__Proteobacteria | c__Gammaproteobacteria | o__Burkholderiales | f__SC-I-84 | g__SC-I-84 | s__uncultured_Nitrosomonadaceae |
| RS-GCF-013374995.1-NZ-CP056030.1--3 | Bacteria | p__Proteobacteria | c__Gammaproteobacteria | o__Pseudomonadales | f__Pseudomonadaceae | g__Pseudomonas |  |
| MJ020-1-barcode31-umi13444bins-ubs-28 | Bacteria | p__Firmicutes | c__Clostridia | o__Clostridiales | f__Clostridiaceae | g__Clostridium_sensu_stricto_10 |  |
| d1677fa9f78a2e1b64d90040339ba544 | Bacteria | p__Proteobacteria | c__Gammaproteobacteria | o__Pseudomonadales | f__Moraxellaceae | g__Acinetobacter |  |
| JN029805 | Bacteria | p__Bacteroidota | c__Bacteroidia | o__Bacteroidales | f__Tannerellaceae | g__Macellibacteroides |  |
| 19706839 | Bacteria | p__Firmicutes | c__Bacilli | o__Erysipelotrichales | f__Erysipelotrichaceae | g__Ileibacterium | s__Ileibacterium_valens |
| FJ225426 | Bacteria | p__Desulfobacterota | c__Desulfovibrionia | o__Desulfovibrionales | f__Desulfovibrionaceae | g__Desulfovibrio | s__Desulfovibrio_legallii |
| 05030272 | Bacteria | p__Proteobacteria | c__Gammaproteobacteria | o__Burkholderiales | f__Comamonadaceae |  |  |
| EU781656 | Bacteria | p__Proteobacteria | c__Alphaproteobacteria | o__Rhizobiales | f__Rhizobiaceae | g__Allorhizobium-Neorhizobium-Pararhizobium-Rhizobium |  |
| aa6d93a22695ec16abf6a6c4043700d4 | Bacteria | p__Bacteroidota | c__Bacteroidia | o__Chitinophagales | f__Saprospiraceae | g__uncultured | s__uncultured_Saprospiraceae |
| 04949974 | Bacteria | p__Firmicutes | c__Clostridia | o__Lachnospirales | f__Lachnospiraceae |  |  |
| AY971368 | Bacteria | p__Proteobacteria | c__Alphaproteobacteria | o__Caulobacterales | f__Caulobacteraceae | g__Brevundimonas |  |
| 30bf13810dfb3ba530366afbd4ce3b1b | Bacteria | p__Proteobacteria | c__Alphaproteobacteria | o__Rhizobiales | f__Rhizobiaceae |  |  |
| 4bd5b3c20afd346c10802212995a32e3 | Bacteria | p__Proteobacteria | c__Alphaproteobacteria | o__Rhizobiales | f__Hyphomicrobiaceae |  |  |
| 00204412 | Bacteria | p__Chloroflexi | c__Anaerolineae | o__Anaerolineales | f__Anaerolineaceae | g__uncultured | s__uncultured_soil |
| 022c09f9acb1095eeb1a37380f19e9e3 | Bacteria | p__Proteobacteria | c__Gammaproteobacteria | o__Pseudomonadales | f__Moraxellaceae | g__Acinetobacter |  |
| 6d3f113a8cabb392a78da8ae8f6601ec | Bacteria | p__Proteobacteria | c__Alphaproteobacteria | o__Rhizobiales | f__Xanthobacteraceae | g__Bradyrhizobium |  |
| 05029682 | Bacteria | p__Proteobacteria | c__Gammaproteobacteria | o__Burkholderiales | f__Burkholderiaceae | g__Ralstonia |  |
| LT617055 | Bacteria | p__Firmicutes | c__Bacilli | o__Bacillales | f__Bacillaceae | g__Bacillus |  |
| 4fcea6c3ac5e39f2fe29d50f9d3e1e78 | Bacteria | p__Verrucomicrobiota | c__Verrucomicrobiae | o__Chthoniobacterales | f__Chthoniobacteraceae | g__Candidatus_Udaeobacter |  |
| e79a4de387f987a31c6376da82fac13b | Bacteria | p__Firmicutes | c__Bacilli | o__Bacillales | f__Bacillaceae | g__Oceanobacillus |  |
| 22229760 | Bacteria | p__Chloroflexi | c__Chloroflexia | o__Thermomicrobiales | f__JG30-KF-CM45 | g__JG30-KF-CM45 | s__uncultured_Chloroflexi |
| 3a6dde3d374882aeef16468bb29c4cee | Bacteria | p__MBNT15 | c__MBNT15 | o__MBNT15 | f__MBNT15 | g__MBNT15 | s__uncultured_proteobacterium |
| 5395bff993b432456cf60203bd0d783b | Bacteria | p__Proteobacteria | c__Alphaproteobacteria | o__Rhizobiales | f__Hyphomicrobiaceae | g__Pedomicrobium |  |
| ef65644e4b5c57d7bd1816f8971fc614 | Bacteria |  |  |  |  |  |  |
| G001956915_1 | Bacteria | p__Actinobacteriota | c__Actinobacteria | o__Micrococcales | f__Intrasporangiaceae |  |  |
| c6cb8826f415412d7f22d97aba17ca38 | Bacteria | p__Proteobacteria | c__Gammaproteobacteria | o__Enterobacterales |  |  |  |
| b3e2217c0e2a35ad9f525f16cf87e794 | Bacteria | p__Proteobacteria | c__Gammaproteobacteria | o__Alteromonadales | f__uncultured | g__uncultured | s__Idiomarina_sp. |
| d0306a1126b26796e2163fa9d651a968 | Bacteria | p__Proteobacteria | c__Gammaproteobacteria | o__Pseudomonadales | f__Moraxellaceae | g__Acinetobacter |  |
| 56c633d46478c6a545797c11e4d536e3 | Bacteria | p__Actinobacteriota | c__MB-A2-108 | o__MB-A2-108 | f__MB-A2-108 | g__MB-A2-108 | s__uncultured_Firmicutes |
| 02244449 | Bacteria | p__Actinobacteriota | c__Actinobacteria | o__Corynebacteriales | f__Nocardiaceae | g__Gordonia |  |
| MJ031-1-barcode28-umi5313bins-ubs-88 | Bacteria | p__Proteobacteria | c__Gammaproteobacteria | o__Methylococcales | f__Methylomonadaceae | g__Methylobacter |  |
| MJ033-1-barcode40-umi24815bins-ubs-11 | Bacteria | p__Acidobacteriota | c__Blastocatellia | o__Pyrinomonadales | f__Pyrinomonadaceae | g__RB41 |  |
| 0f79db98ec9255164d39ed11065fb6fe | Bacteria | p__Nitrospirota | c__Nitrospiria | o__Nitrospirales | f__Nitrospiraceae | g__Nitrospira |  |
| KU043522 | Bacteria | p__Firmicutes | c__Clostridia | o__Peptostreptococcales-Tissierellales | f__Peptostreptococcales-Tissierellales | g__Anaerococcus | s__Anaerococcus_nagyae |
| 02244791 | Bacteria | p__Actinobacteriota | c__Actinobacteria | o__Corynebacteriales |  |  |  |
| 05020769 | Bacteria | p__Firmicutes | c__Clostridia | o__Clostridia_UCG-014 | f__Clostridia_UCG-014 | g__Clostridia_UCG-014 |  |
| bbc5c4a1626b294589d63dc0236d012e | Bacteria | p__Proteobacteria | c__Gammaproteobacteria | o__Xanthomonadales | f__Xanthomonadaceae |  |  |
| 05033691 | Bacteria | p__Actinobacteriota | c__Actinobacteria | o__Micrococcales | f__Intrasporangiaceae |  |  |
| 21955e30148c5ad787c62ab603b54df8 | Bacteria | p__Chloroflexi | c__Chloroflexia | o__Chloroflexales | f__Roseiflexaceae | g__uncultured |  |
| 04906525 | Bacteria | p__Proteobacteria | c__Alphaproteobacteria | o__Rhizobiales | f__Beijerinckiaceae | g__Microvirga | s__Microvirga_massiliensis |
| d84a5605ad0d8b478c2733a0027ae286 | Bacteria | p__Chloroflexi | c__Anaerolineae | o__1-20 | f__1-20 | g__1-20 | s__uncultured_bacterium |
| HM776705 | Bacteria | p__Bacteroidota | c__Bacteroidia | o__Flavobacteriales | f__Flavobacteriaceae | g__Flavobacterium |  |
| cecfd83ebed4bc866f09ae0dc57e4b4f | Bacteria | p__Planctomycetota | c__Planctomycetes | o__Gemmatales | f__Gemmataceae | g__uncultured |  |
| aacec78f503744ea7a7dd47f4b2e2919 | Bacteria | p__Proteobacteria | c__Alphaproteobacteria | o__Rhizobiales | f__Xanthobacteraceae | g__uncultured |  |
| MJ031-2-barcode67-umi315bins-ubs-626 | Bacteria | p__Proteobacteria | c__Alphaproteobacteria | o__SAR11_clade | f__Clade_III | g__Clade_III | s__Candidatus_Fonsibacter |
| G001423245_1 | Bacteria | p__Proteobacteria | c__Alphaproteobacteria | o__Rhizobiales | f__Rhizobiaceae | g__Aureimonas |  |
| d4481eade281c2c97fd6e9b4009bcde9 | Bacteria | p__Bacteroidota | c__Bacteroidia | o__Chitinophagales | f__Chitinophagaceae |  |  |
| RS-GCF-004793675.1-NZ-SRYX01000135.1 | Bacteria | p__Bacteroidota | c__Bacteroidia | o__Bacteroidales | f__Bacteroidaceae | g__Bacteroides | s__Bacteroides_caecimuris |
| RS-GCF-001758725.1-NZ-LRIC01000049.1 | Bacteria | p__Proteobacteria | c__Gammaproteobacteria | o__Burkholderiales | f__Oxalobacteraceae |  |  |
| 3d91c94d9f3847519c582e3da5987452 | Bacteria | p__Proteobacteria | c__Alphaproteobacteria | o__Elsterales | f__uncultured | g__uncultured |  |
| FJ177534 | Bacteria | p__Proteobacteria | c__Alphaproteobacteria | o__Sphingomonadales | f__Sphingomonadaceae | g__Novosphingobium |  |
| f91ec2ea989faedc12259e3cdbfe9e62 | Bacteria | p__Chloroflexi | c__KD4-96 | o__KD4-96 | f__KD4-96 | g__KD4-96 | s__uncultured_bacterium |
| 5a7879be34f835765d8797cffd96d23d | Bacteria | p__Firmicutes | c__Bacilli | o__Lactobacillales | f__Lactobacillaceae | g__Lactobacillus |  |
| 2afecfd038f08efe3c4fd9a10dc301e7 | Bacteria | p__Acidobacteriota | c__Acidobacteriae | o__Subgroup_2 | f__Subgroup_2 | g__Subgroup_2 |  |
| 4e05b67e9bc8ed21e72a5deb2ec64249 | Bacteria | p__Fusobacteriota | c__Fusobacteriia | o__Fusobacteriales | f__Fusobacteriaceae | g__Cetobacterium | s__uncultured_bacterium |
| 3f28baa8dcb5491fdb942b0cc7f191f6 | Bacteria | p__Acidobacteriota | c__Vicinamibacteria | o__Vicinamibacterales | f__uncultured | g__uncultured |  |
| 04911155 | Bacteria | p__Proteobacteria | c__Gammaproteobacteria | o__Xanthomonadales | f__Xanthomonadaceae | g__Stenotrophomonas |  |
| 04956562 | Bacteria | p__Actinobacteriota | c__Actinobacteria | o__Corynebacteriales | f__Mycobacteriaceae | g__Mycobacterium |  |
| 8dfa639642d7439b8ebf26d82ebb75a4 | Bacteria | p__Firmicutes | c__Bacilli | o__Staphylococcales | f__Staphylococcaceae | g__Staphylococcus |  |
| 04907486 | Bacteria | p__Proteobacteria | c__Alphaproteobacteria | o__Caulobacterales | f__Caulobacteraceae | g__Caulobacter |  |
| 05951393 | Bacteria | p__Acidobacteriota | c__Vicinamibacteria | o__Vicinamibacterales | f__Vicinamibacteraceae | g__Vicinamibacteraceae |  |
| RS-GCF-002850055.1-NZ-CP018180.1--2 | Bacteria | p__Firmicutes | c__Bacilli | o__Lactobacillales | f__Lactobacillaceae | g__Lactobacillus | s__Lactobacillus_nagelii |
| 7c1b1127df368f25eb9ce41597852764 | Bacteria | p__Firmicutes | c__Bacilli | o__Erysipelotrichales | f__Erysipelatoclostridiaceae | g__Erysipelotrichaceae_UCG-003 |  |
| GB-GCA-000413335.1-KE340299.1 | Bacteria | p__Actinobacteriota | c__Actinobacteria | o__Propionibacteriales | f__Propionibacteriaceae | g__Cutibacterium |  |
| 06438e6efc4d77aca011b6cb03e6d347 | Bacteria | p__Actinobacteriota | c__Acidimicrobiia | o__Microtrichales | f__uncultured | g__uncultured | s__wastewater_metagenome |
| b75f66653898665a60310411b73aa089 | Bacteria | p__Proteobacteria | c__Gammaproteobacteria | o__Steroidobacterales | f__Steroidobacteraceae | g__Steroidobacter |  |
| MJ006-1-barcode46-umi207799bins-ubs-3 | Bacteria | p__Firmicutes | c__Clostridia | o__Lachnospirales | f__Lachnospiraceae |  |  |
| 7aa9c4f6fa8a619c9446563d652a2d44 | Bacteria | p__Chloroflexi | c__OLB14 | o__OLB14 | f__OLB14 | g__OLB14 | s__uncultured_bacterium |
| 04960093 | Bacteria | p__Proteobacteria | c__Gammaproteobacteria | o__Burkholderiales | f__Comamonadaceae |  |  |
| 02859899 | Bacteria | p__Chloroflexi | c__Chloroflexia | o__Chloroflexales | f__Roseiflexaceae | g__uncultured | s__uncultured_Chloroflexaceae |
| 034ef0383db76ed38a595864309528ce | Bacteria |  |  |  |  |  |  |
| a97d353e922e65263e189bf14825e6f6 | Bacteria | p__Bacteroidota | c__Bacteroidia | o__Bacteroidales | f__Bacteroidetes_vadinHA17 | g__Bacteroidetes_vadinHA17 |  |
| RS-GCF-009004095.1-NZ-BKSS01000201.1 | Bacteria | p__Proteobacteria | c__Gammaproteobacteria | o__Pseudomonadales | f__Moraxellaceae | g__Acinetobacter |  |
| 1a1717f6c4c9628b7b13c5f45eda65e3 | Bacteria | p__Firmicutes | c__Clostridia | o__Peptostreptococcales-Tissierellales | f__Anaerovoracaceae | g__Mogibacterium | s__uncultured_rumen |
| 8dfbcdbfc62dcad3342644108616f0ce | Bacteria | p__Actinobacteriota | c__Actinobacteria | o__Corynebacteriales | f__Nocardiaceae | g__Rhodococcus |  |
| 7a535f3f5992188d761d0bdd003f3d65 | Bacteria | p__Actinobacteriota | c__Actinobacteria | o__Propionibacteriales | f__Nocardioidaceae | g__Nocardioides |  |
| 666b9397e0463f05c1d06a9a65229245 | Bacteria | p__Bacteroidota | c__Bacteroidia | o__Bacteroidales | f__Muribaculaceae | g__Muribaculaceae |  |
| GB-GCA-007096665.1-SFAL01000225.1 | Bacteria | p__Proteobacteria | c__Alphaproteobacteria | o__Rhizobiales |  |  |  |
| 4bd21d71a786eda7df97c0c62cd1afd3 | Bacteria | p__Proteobacteria | c__Gammaproteobacteria | o__Enterobacterales | f__Enterobacteriaceae |  |  |
| 01936126a0bc74110c962607c05c3298 | Bacteria | p__Actinobacteriota | c__Actinobacteria | o__Frankiales | f__Sporichthyaceae | g__Candidatus_Planktophila |  |
| MJ006-2-barcode74-umi167704bins-ubs-3 | Bacteria | p__Bacteroidota | c__Bacteroidia | o__Bacteroidales | f__Bacteroidaceae | g__Bacteroides |  |
| GB-GCA-017832085.1-JAFDSV010000031.1 | Bacteria | p__Proteobacteria | c__Gammaproteobacteria | o__Enterobacterales | f__Yersiniaceae |  |  |
| 3c2a3dd63267e67bab39e30253345faf | Bacteria | p__Bacteroidota | c__Bacteroidia | o__Bacteroidales | f__Prevotellaceae | g__Alloprevotella | s__Prevotellaceae_bacterium |
| 04909683 | Bacteria | p__Proteobacteria | c__Alphaproteobacteria | o__Rhizobiales | f__Devosiaceae | g__Devosia |  |
| 02247951 | Bacteria | p__Actinobacteriota | c__Actinobacteria | o__Propionibacteriales | f__Nocardioidaceae | g__Nocardioides |  |
| GB-GCA-018370675.1-JAGYZX010000010.1 | Bacteria | p__Firmicutes | c__Clostridia | o__Clostridia_UCG-014 | f__Clostridia_UCG-014 | g__Clostridia_UCG-014 |  |
| 04964628 | Bacteria | p__Firmicutes | c__Bacilli | o__Lactobacillales | f__Lactobacillaceae | g__Pediococcus |  |
| d14ec4e4f29b26c4001efa4ef84a7c06 | Bacteria | p__Actinobacteriota | c__Acidimicrobiia | o__Actinomarinales | f__uncultured | g__uncultured |  |
| ce7e3c5921b5518c095e5495e9866c30 | Bacteria | p__Proteobacteria | c__Gammaproteobacteria | o__Burkholderiales | f__Comamonadaceae | g__Pelomonas |  |
| 468eae303428c916af3f6ef8d4f7d246 | Bacteria | p__Actinobacteriota | c__Thermoleophilia | o__Gaiellales | f__uncultured | g__uncultured |  |
| X79286 | Bacteria | p__Actinobacteriota | c__Actinobacteria | o__Corynebacteriales | f__Nocardiaceae | g__Gordonia |  |
| 18436856 | Bacteria | p__Proteobacteria | c__Gammaproteobacteria | o__Burkholderiales | f__Comamonadaceae |  |  |
| 8024330b521b722697701a4f75d5e045 | Bacteria | p__Proteobacteria | c__Gammaproteobacteria | o__Enterobacterales | f__Enterobacteriaceae |  |  |
| MJ031-2-barcode64-umi7828bins-ubs-59 | Bacteria | p__Firmicutes | c__Bacilli | o__Bacillales | f__Bacillaceae | g__Bacillus |  |
| U07574 | Bacteria | p__Campilobacterota | c__Campylobacteria | o__Campylobacterales | f__Helicobacteraceae | g__Helicobacter | s__Helicobacter_hepaticus |
| 44758f3d7168e466008e9561db1a39d4 | Bacteria | p__Acidobacteriota | c__Acidobacteriae | o__Bryobacterales | f__Bryobacteraceae | g__Bryobacter |  |
| 11805265 | Bacteria | p__Proteobacteria | c__Alphaproteobacteria | o__Rhodobacterales | f__Rhodobacteraceae | g__Paracoccus |  |
| f9b2c120463c6058f3c1d3e3d7c8ee1f | Bacteria | p__Actinobacteriota | c__Thermoleophilia | o__Solirubrobacterales | f__Solirubrobacteraceae |  |  |
| d9eac0c5f5fff031bbd0ef228a5ecf48 | Bacteria | p__Actinobacteriota | c__Actinobacteria | o__Frankiales |  |  |  |
| 04665635 | Bacteria | p__Proteobacteria | c__Gammaproteobacteria | o__Pseudomonadales | f__Pseudomonadaceae | g__Pseudomonas |  |
| 4589b2df8a21d5db35d1c7fd83644cd7 | Bacteria | p__Bacteroidota | c__Bacteroidia | o__Bacteroidales | f__Prevotellaceae | g__Prevotella |  |
| 92edd79826b21a9eaf10194b70da3c52 | Bacteria | p__Proteobacteria | c__Gammaproteobacteria | o__Xanthomonadales | f__Xanthomonadaceae | g__Thermomonas |  |
| acadb7310cfc94a5f87d48edab204ad6 | Bacteria | p__Patescibacteria | c__Saccharimonadia | o__Saccharimonadales | f__LWQ8 | g__LWQ8 |  |
| bbb21e79507333b6535e1d135536b15e | Bacteria | p__Proteobacteria | c__Gammaproteobacteria | o__Enterobacterales | f__Enterobacteriaceae |  |  |
| MJ007-2-barcode68-umi31923bins-ubs-10 | Bacteria | p__Bacteroidota | c__Bacteroidia | o__Bacteroidales | f__Bacteroidetes_vadinHA17 | g__Bacteroidetes_vadinHA17 | s__uncultured_bacterium |
| 4213eb4e688a86201dd9697b21f0dc38 | Bacteria | p__Firmicutes | c__Bacilli | o__Alicyclobacillales | f__Alicyclobacillaceae | g__Tumebacillus |  |
| RS-GCF-014650955.1-NZ-BMVY01000099.1 | Bacteria | p__Actinobacteriota | c__Actinobacteria | o__Streptomycetales | f__Streptomycetaceae | g__Streptomyces |  |
| 5dbb68ff02ef0dc7092d6659ed18b96c | Bacteria | p__Firmicutes | c__Bacilli |  |  |  |  |
| ff9d8c6b0180dc087ca708ede429713c | Bacteria | p__Chloroflexi | c__Anaerolineae | o__Anaerolineales | f__Anaerolineaceae | g__uncultured |  |
| f3913a31aebebe2b292bb40e31a6dfed | Bacteria | p__Proteobacteria | c__Gammaproteobacteria | o__Burkholderiales | f__Comamonadaceae | g__Comamonas |  |
| 4909b2361d1e8a3fb874913de36a56ff | Bacteria | p__Actinobacteriota | c__Actinobacteria | o__Bifidobacteriales | f__Bifidobacteriaceae | g__Bifidobacterium | s__Bifidobacterium_bifidum |
| 05012862 | Bacteria | p__Bacteroidota | c__Bacteroidia | o__Chitinophagales | f__Chitinophagaceae | g__Flavisolibacter |  |
| KP722591 | Bacteria | p__Actinobacteriota | c__Actinobacteria | o__Micrococcales | f__Microbacteriaceae | g__Microbacterium |  |
| 2a509c23e33a6060613e7904466fab08 | Bacteria | p__Desulfobacterota | c__uncultured | o__uncultured | f__uncultured | g__uncultured |  |
| 6ffbee9478ac1f69a8700b01697cbcbb | Bacteria | p__Proteobacteria | c__Gammaproteobacteria | o__Xanthomonadales | f__Xanthomonadaceae | g__Vulcaniibacterium | s__uncultured_bacterium |
| 08792684 | Bacteria | p__Proteobacteria | c__Gammaproteobacteria | o__Xanthomonadales | f__Xanthomonadaceae | g__Lysobacter |  |
| ddfa79da38a1e093d895f4c430bf8444 | Bacteria | p__Proteobacteria | c__Gammaproteobacteria | o__Enterobacterales | f__Erwiniaceae |  |  |
| 85f5716355edc7faa1e1501fbb065a07 | Bacteria | p__Proteobacteria | c__Alphaproteobacteria | o__Sphingomonadales | f__Sphingomonadaceae | g__Sandaracinobacter |  |
| fa6605afc61d2682d608bc16c8e8e64e | Bacteria | p__Proteobacteria | c__Gammaproteobacteria | o__Pseudomonadales | f__Pseudomonadaceae | g__Pseudomonas |  |
| MJ006-2-barcode66-umi80613bins-ubs-5 | Bacteria | p__Firmicutes | c__Bacilli | o__Erysipelotrichales | f__Erysipelotrichaceae | g__Holdemanella | s__uncultured_bacterium |
| RS-GCF-000264255.1-NZ-AJWW01000044.1 | Bacteria | p__Firmicutes | c__Bacilli | o__Bacillales | f__Bacillaceae | g__Bacillus |  |
| 2728b26cbf3a155c121e16df9bd447ad | Bacteria | p__Myxococcota | c__bacteriap25 | o__bacteriap25 | f__bacteriap25 | g__bacteriap25 | s__uncultured_Desulfovibrionales |
| 04545303 | Bacteria | p__Bacteroidota | c__Bacteroidia | o__Bacteroidales | f__Tannerellaceae | g__Parabacteroides |  |
| befc72931f24b919641295c35a963049 | Bacteria | p__Proteobacteria | c__Gammaproteobacteria | o__Cellvibrionales | f__Cellvibrionaceae | g__Cellvibrio | s__uncultured_bacterium |
| MH665407 | Bacteria | p__Proteobacteria | c__Alphaproteobacteria | o__Rhizobiales | f__Devosiaceae | g__Devosia |  |
| RS-GCF-002165305.2-NZ-CP032286.1 | Bacteria | p__Proteobacteria | c__Gammaproteobacteria | o__Pseudomonadales | f__Moraxellaceae | g__Acinetobacter |  |
| 8b302af8b0d3b3543ebd108180637686 | Bacteria | p__Patescibacteria | c__Saccharimonadia | o__Saccharimonadales | f__Saccharimonadaceae | g__TM7a |  |
| 2932bdbf3656d4c9b15e1522830ec63a | Bacteria | p__Acidobacteriota | c__Vicinamibacteria | o__Vicinamibacterales | f__Vicinamibacteraceae | g__Vicinamibacteraceae |  |
| 1ca28def3d077d747e09f90a2221a992 | Bacteria | p__Bacteroidota | c__Bacteroidia | o__Bacteroidales | f__Rikenellaceae | g__Alistipes |  |
| c326149c6d5cb9bf51259eb135195ba8 | Bacteria | p__Proteobacteria | c__Alphaproteobacteria | o__Rhizobiales | f__Xanthobacteraceae |  |  |
| ba8afb8e1685ecf1a1d041bcf0e3202c | Bacteria | p__Proteobacteria | c__Alphaproteobacteria | o__Caulobacterales | f__Caulobacteraceae | g__Brevundimonas |  |
| 16210589 | Bacteria | p__Firmicutes | c__Clostridia | o__Lachnospirales | f__Lachnospiraceae |  |  |
| 37dede21b4fda5576b6b5a41082bc682 | Bacteria | p__Actinobacteriota | c__Thermoleophilia | o__Solirubrobacterales | f__Solirubrobacteraceae | g__Conexibacter | s__uncultured_bacterium |
| 04908976 | Bacteria | p__Proteobacteria | c__Alphaproteobacteria | o__Rhizobiales | f__Rhizobiaceae | g__Mesorhizobium |  |
| GB-GCA-910575335.1-CAJTEB010000008.1 | Bacteria | p__Actinobacteriota | c__Coriobacteriia | o__Coriobacteriales | f__Eggerthellaceae | g__Enterorhabdus |  |
| MJ009-1-barcode40-umi22393bins-ubs-16 | Bacteria | p__Bacteroidota | c__Bacteroidia | o__Chitinophagales | f__Chitinophagaceae | g__uncultured |  |
| MJ010-2-barcode71-umi94744bins-ubs-5 | Bacteria | p__Proteobacteria | c__Gammaproteobacteria | o__Burkholderiales | f__Comamonadaceae | g__Rhizobacter |  |
| 2c9507c5154602e9fb4dc80006c300ec | Bacteria | p__Proteobacteria | c__Gammaproteobacteria | o__JG36-TzT-191 | f__JG36-TzT-191 | g__JG36-TzT-191 |  |
| 3f58bbe832d2ccdb95f090fe69c6d0ef | Bacteria | p__Proteobacteria | c__Alphaproteobacteria | o__Rhodobacterales | f__Rhodobacteraceae | g__Rhodobacter |  |
| aeb0351422e19d018caf33894a0fc3a7 | Bacteria | p__Bacteroidota | c__Bacteroidia | o__Flavobacteriales | f__NS9_marine_group | g__NS9_marine_group | s__uncultured_soil |
| RS-GCF-002215315.1-NZ-NETI01000389.1 | Bacteria | p__Proteobacteria | c__Gammaproteobacteria | o__Xanthomonadales | f__Xanthomonadaceae | g__Stenotrophomonas |  |
| 01255653 | Bacteria | p__Firmicutes | c__Clostridia | o__Lachnospirales | f__Lachnospiraceae |  |  |
| 8144b876d90ff0e13d038e5bda62930a | Bacteria | p__Chloroflexi | c__Anaerolineae | o__SBR1031 | f__A4b | g__A4b |  |
| 0c8d754fb14a22facf467b12e59310a4 | Bacteria | p__Actinobacteriota | c__Acidimicrobiia | o__Microtrichales | f__uncultured | g__uncultured |  |
| 3fbe8dc3742ddc83bc808e4bcc556d82 | Bacteria | p__Proteobacteria | c__Alphaproteobacteria | o__Rhizobiales | f__Beijerinckiaceae | g__1174-901-12 | s__uncultured_bacterium |
| cc1c74d178fddcfa2503410cff65b8a3 | Bacteria | p__Proteobacteria | c__Gammaproteobacteria | o__Burkholderiales | f__Nitrosomonadaceae | g__Ellin6067 |  |
| 6fad9b4895a13b5d4215799548c6960e | Bacteria | p__Actinobacteriota | c__Actinobacteria | o__Micrococcales | f__Brevibacteriaceae | g__Brevibacterium |  |
| e6758666a21fb401b287522cc5b6bec8 | Bacteria | p__Proteobacteria | c__Gammaproteobacteria | o__Xanthomonadales | f__Rhodanobacteraceae | g__Ahniella | s__uncultured_bacterium |
| f10b83a67c5d5099935c5b2e2b087537 | Bacteria | p__Proteobacteria | c__Alphaproteobacteria | o__Rhizobiales | f__Xanthobacteraceae | g__Rhodopseudomonas |  |
| 02409608 | Bacteria | p__Firmicutes | c__Clostridia | o__Oscillospirales | f__Ruminococcaceae | g__Faecalibacterium |  |
| 4bafd042ba7e59aec2d19bef56bb8dd8 | Bacteria | p__Acidobacteriota | c__Blastocatellia | o__Blastocatellales | f__Blastocatellaceae |  |  |
| RS-GCF-015235485.1-NZ-JADGLP010000315.1 | Bacteria | p__Bacteroidota | c__Bacteroidia | o__Bacteroidales | f__Bacteroidaceae | g__Bacteroides |  |
| f5f680a4b52ae392dbc1c673a6590c2c | Bacteria | p__RCP2-54 | c__RCP2-54 | o__RCP2-54 | f__RCP2-54 | g__RCP2-54 |  |
| fb86507fa82158957776a9d97cd7cca6 | Bacteria | p__Proteobacteria | c__Gammaproteobacteria | o__Burkholderiales | f__SC-I-84 | g__SC-I-84 |  |
| 379d99d769388e221c4fdb8b7d24d927 | Bacteria | p__Acidobacteriota | c__Acidobacteriae | o__Acidobacteriales | f__Acidobacteriaceae_(Subgroup_1) | g__Granulicella |  |
| 4e27419511ebd960f2dd69e6a451788a | Bacteria | p__Proteobacteria | c__Gammaproteobacteria | o__PLTA13 | f__PLTA13 | g__PLTA13 | s__uncultured_bacterium |
| 2f6583ba17eff727b5a76b32c3709f17 | Bacteria | p__Cyanobacteria | c__Cyanobacteriia | o__Cyanobacteriales | f__Nostocaceae |  |  |
| c961091c359a61afbabf3eac5021a2cc | Bacteria | p__Bacteroidota | c__Bacteroidia | o__Chitinophagales | f__Chitinophagaceae | g__Flavisolibacter |  |
| RS-GCF-003458915.1-NZ-QRTR01000015.1 | Bacteria | p__Bacteroidota | c__Bacteroidia | o__Bacteroidales | f__Bacteroidaceae | g__Bacteroides | s__Bacteroides_plebeius |
| e5e6ea0797a70d96e3e39ab53798e439 | Bacteria | p__Proteobacteria | c__Gammaproteobacteria | o__Burkholderiales | f__Rhodocyclaceae |  |  |
| ef88a02e46cbbaa4c1dfd7a8aca7c4fc | Bacteria | p__Proteobacteria | c__Gammaproteobacteria | o__Burkholderiales | f__SC-I-84 | g__SC-I-84 |  |
| c85e70f9cee4884182d221e735555702 | Bacteria | p__Firmicutes | c__Bacilli | o__Bacillales | f__Marinococcaceae | g__Alteribacillus |  |
| 90444a4c690a4731d48ae86c89aed931 | Bacteria | p__Bacteroidota | c__Bacteroidia | o__Flavobacteriales | f__Weeksellaceae | g__Chryseobacterium |  |
| c65c3fd58e71201d6f1325874d601428 | Bacteria | p__Firmicutes | c__Clostridia | o__Christensenellales | f__Christensenellaceae | g__Christensenellaceae_R-7_group |  |
| f10a0753a1e6501e389962f708777b06 | Bacteria | p__Chloroflexi | c__Anaerolineae | o__1-20 | f__1-20 | g__1-20 | s__uncultured_bacterium |
| RS-GCF-001591645.1-NZ-BCVH01000052.1 | Bacteria | p__Firmicutes | c__Bacilli | o__Bacillales | f__Bacillaceae | g__Bacillus |  |
| Y07841 | Bacteria | p__Firmicutes | c__Clostridia | o__Peptostreptococcales-Tissierellales | f__Peptostreptococcales-Tissierellales | g__Anaerococcus |  |
| 5cdf17587b3a5656998fd2d033899391 | Bacteria | p__Proteobacteria | c__Gammaproteobacteria | o__Burkholderiales | f__SC-I-84 | g__SC-I-84 |  |
| 01085284 | Bacteria | p__Actinobacteriota | c__Actinobacteria | o__Micrococcales | f__Dermabacteraceae | g__Brachybacterium |  |
| GB-GCA-018383075.1-JAGZOU010000022.1 | Bacteria | p__Bacteroidota | c__Bacteroidia | o__Bacteroidales |  |  |  |
| 4df7e17128809d582cc2874c506bfd60 | Bacteria | p__Verrucomicrobiota | c__Verrucomicrobiae | o__Chthoniobacterales | f__Chthoniobacteraceae | g__Candidatus_Udaeobacter | s__uncultured_Prosthecobacter |
| RS-GCF-002754135.1-NZ-CP024598.1--3 | Bacteria | p__Bacteroidota | c__Bacteroidia | o__Bacteroidales | f__Porphyromonadaceae | g__Porphyromonas | s__Porphyromonas_gingivalis |
| KJ721000 | Bacteria | p__Proteobacteria | c__Alphaproteobacteria | o__Rhizobiales | f__Beijerinckiaceae | g__Bosea |  |
| 3e25a26abb865c54d8381e4d9e603ca8 | Bacteria | p__Bacteroidota | c__Bacteroidia | o__Bacteroidales | f__Bacteroidaceae | g__Bacteroides |  |
| 74987d4129a666a04d020821e9956a11 | Bacteria | p__Firmicutes | c__Clostridia | o__Peptostreptococcales-Tissierellales | f__Peptostreptococcaceae |  |  |
| a135b2cea2e63e18a18eade71ee03bf0 | Bacteria | p__Proteobacteria | c__Alphaproteobacteria | o__Rhodobacterales | f__Rhodobacteraceae | g__Amaricoccus |  |
| 6f86613d5b63adb5ec496def7650966c | Bacteria | p__Firmicutes | c__BRH-c20a | o__BRH-c20a | f__BRH-c20a | g__BRH-c20a |  |
| 04961002 | Bacteria | p__Actinobacteriota | c__Actinobacteria | o__Corynebacteriales | f__Mycobacteriaceae | g__Mycobacterium |  |
| 8ccd551e6757b454ac4c22727b07cbcc | Bacteria | p__Actinobacteriota | c__Acidimicrobiia | o__Microtrichales | f__Ilumatobacteraceae | g__uncultured | s__uncultured_Acidimicrobiaceae |
| GB-GCA-019048765.1-CP077415.1 | Bacteria | p__Proteobacteria | c__Gammaproteobacteria | o__Pseudomonadales | f__Moraxellaceae | g__Acinetobacter |  |
| ccbe67051f82d9135df9ff48a4844167 | Bacteria | p__Cyanobacteria | c__Cyanobacteriia | o__Cyanobacteriales |  |  |  |
| 289846b01b556b3d0453789e0915aff3 | Bacteria | p__Firmicutes | c__Bacilli | o__Erysipelotrichales | f__Erysipelotrichaceae | g__ZOR0006 |  |
| RS-GCF-013296575.1-NZ-JABTAQ010000001.1--13 | Bacteria | p__Firmicutes | c__Clostridia | o__Clostridiales | f__Clostridiaceae | g__Clostridium_sensu_stricto_1 |  |
| DQ883810 | Bacteria | p__Proteobacteria | c__Alphaproteobacteria | o__Rhizobiales | f__Rhizobiaceae |  |  |
| 7734a473aef183e1121b1c26e215e31a | Bacteria | p__Actinobacteriota | c__Thermoleophilia | o__Solirubrobacterales | f__Solirubrobacteraceae |  |  |
| 7955f9959cd0db724e8e485053e80a21 | Bacteria | p__Firmicutes | c__Clostridia | o__Clostridia_vadinBB60_group | f__Clostridia_vadinBB60_group | g__Clostridia_vadinBB60_group | s__unidentified |
| KJ504173 | Bacteria | p__Actinobacteriota | c__Actinobacteria | o__Pseudonocardiales | f__Pseudonocardiaceae | g__Prauserella |  |
| 04918972 | Bacteria | p__Bacteroidota | c__Bacteroidia | o__Sphingobacteriales | f__Sphingobacteriaceae | g__Sphingobacterium |  |
| e368a0221c9fa2285d69512447854e4c | Bacteria | p__Proteobacteria | c__Alphaproteobacteria | o__Rhizobiales | f__Rhizobiaceae |  |  |
| c0088dbdd9de6975f37c2fe32be5ad4d | Bacteria | p__Bacteroidota | c__Bacteroidia | o__Bacteroidales | f__Bacteroidaceae | g__Bacteroides | s__Bacteroides_finegoldii |
| RS-GCF-014853615.1-NZ-JACZAP010000041.1 | Bacteria | p__Firmicutes | c__Bacilli | o__Lactobacillales | f__Enterococcaceae | g__Enterococcus |  |
| a14dafe75c3b5e43f198cf187d905a0e | Bacteria | p__Firmicutes | c__Bacilli | o__Bacillales | f__Bacillaceae | g__Bacillus |  |
| 1e5cda9133cb98afe1b4c023d79acfd7 | Bacteria | p__Bacteroidota | c__Bacteroidia | o__Cytophagales | f__Hymenobacteraceae | g__Hymenobacter |  |
| 03038018 | Bacteria | p__Myxococcota | c__Polyangia | o__mle1-27 | f__mle1-27 | g__mle1-27 | s__uncultured_Polyangiaceae |
| 612306e8e3202396e88a2333c2b283ad | Bacteria | p__Proteobacteria | c__Gammaproteobacteria | o__Burkholderiales |  |  |  |
| 04960888 | Bacteria | p__Actinobacteriota | c__Actinobacteria | o__Corynebacteriales | f__Corynebacteriaceae | g__Corynebacterium |  |
| 9184296310c04352dc7d0f05b203aa07 | Bacteria | p__Proteobacteria | c__Gammaproteobacteria | o__Xanthomonadales | f__Xanthomonadaceae |  |  |
| 8dbf19af9ef56b68ce7010fed056342b | Bacteria | p__Proteobacteria | c__Gammaproteobacteria | o__Burkholderiales |  |  |  |
| 04237906 | Bacteria | p__Cyanobacteria | c__Sericytochromatia | o__Sericytochromatia | f__Sericytochromatia | g__Sericytochromatia |  |
| 2c838352043453caf916196166a7cda0 | Bacteria | p__Actinobacteriota | c__Actinobacteria | o__Micrococcales | f__Micrococcaceae |  |  |
| ae75ddb0332a661baf2d971989774d59 | Bacteria | p__Nitrospirota | c__Nitrospiria | o__Nitrospirales | f__Nitrospiraceae | g__Nitrospira |  |
| b3da892a532a21e6a39d1623498c144e | Bacteria | p__Chloroflexi | c__Anaerolineae | o__SBR1031 | f__SBR1031 | g__SBR1031 | s__uncultured_Caldilineaceae |
| 16959454 | Bacteria | p__Firmicutes | c__Clostridia | o__Lachnospirales | f__Lachnospiraceae |  |  |
| 6cbea9b2959a01020312e1926249a42f | Bacteria | p__Actinobacteriota | c__Actinobacteria | o__Corynebacteriales | f__Corynebacteriaceae | g__Corynebacterium | s__Corynebacterium_sputi |
| AY014175 | Bacteria | p__Proteobacteria | c__Alphaproteobacteria | o__Rhodobacterales | f__Rhodobacteraceae | g__Paracoccus |  |
| MJ006-1-barcode38-umi213440bins-ubs-3 | Bacteria | p__Firmicutes | c__Negativicutes | o__Veillonellales-Selenomonadales | f__Veillonellaceae | g__Veillonella |  |
| MG183676 | Bacteria | p__Proteobacteria | c__Alphaproteobacteria | o__Sphingomonadales | f__Sphingomonadaceae |  |  |
| 02339821 | Bacteria | p__Proteobacteria | c__Alphaproteobacteria | o__Rhizobiales | f__Beijerinckiaceae | g__Methylobacterium-Methylorubrum |  |
| 17684207 | Bacteria | p__Firmicutes | c__Clostridia | o__Lachnospirales | f__Lachnospiraceae |  |  |
| 53f9b46820d5d3a49b486bd1413c914b | Bacteria | p__Proteobacteria | c__Gammaproteobacteria | o__KI89A_clade | f__KI89A_clade | g__KI89A_clade |  |
| 04944434 | Bacteria | p__Firmicutes | c__Clostridia | o__Peptococcales | f__Peptococcaceae | g__uncultured | s__uncultured_bacterium |
| e529c39ea9d96a4de8e7950dd2b3b8d5 | Bacteria | p__Proteobacteria | c__Alphaproteobacteria | o__Sphingomonadales | f__Sphingomonadaceae | g__Sphingomonas |  |
| 8ee583fd210994ab3607cea061eb13fd | Bacteria | p__Bacteroidota | c__Bacteroidia | o__Bacteroidales | f__Williamwhitmaniaceae | g__Acetobacteroides | s__uncultured_bacterium |
| 41d350dcd3f5d215e2a92527b01aa6e9 | Bacteria | p__Bacteroidota | c__Bacteroidia | o__Flavobacteriales | f__Flavobacteriaceae |  |  |
| 04906103 | Bacteria | p__Proteobacteria | c__Alphaproteobacteria | o__Rhizobiales | f__Xanthobacteraceae | g__uncultured |  |
| 34d8e05354ea24a911e8e6b0db1b4427 | Bacteria | p__MBNT15 | c__MBNT15 | o__MBNT15 | f__MBNT15 | g__MBNT15 | s__uncultured_proteobacterium |
| 613be25506c0ad5267e28fe3b0be59da | Bacteria | p__Bacteroidota | c__Bacteroidia | o__Flavobacteriales | f__NS9_marine_group | g__NS9_marine_group | s__Bacteroidetes_bacterium |
| RS-GCF-000313875.1-NZ-AMXV01000004.1 | Bacteria | p__Proteobacteria | c__Gammaproteobacteria | o__Burkholderiales | f__Alcaligenaceae | g__Alcaligenes |  |
| 5b1ab8a1b472df922e6f69435ead2df0 | Bacteria | p__Proteobacteria | c__Alphaproteobacteria | o__Rhizobiales | f__Xanthobacteraceae | g__uncultured |  |
| 0925cdf4c98f3937906092e4d3dace5d | Bacteria | p__Proteobacteria | c__Gammaproteobacteria | o__Competibacterales | f__Competibacteraceae | g__Candidatus_Competibacter |  |
| 5ba764bf98ee87f143e1bda2f0acbe8f | Bacteria | p__Actinobacteriota | c__Thermoleophilia |  |  |  |  |
| b1fced8399b8af841ea58ed821520c58 | Bacteria | p__Planctomycetota | c__Phycisphaerae | o__Tepidisphaerales | f__WD2101_soil_group | g__WD2101_soil_group | s__uncultured_bacterium |
| 03072496 | Bacteria | p__Myxococcota | c__Polyangia | o__Polyangiales | f__BIrii41 | g__BIrii41 | s__uncultured_delta |
| 5e3f6a0298ec992dfa1d130238ccd954 | Bacteria | p__Bacteroidota | c__Bacteroidia | o__Bacteroidales | f__Dysgonomonadaceae | g__Proteiniphilum |  |
| 8e274e333d4e7bfc2a0c472f82840dba | Bacteria | p__Chloroflexi | c__Chloroflexia | o__Chloroflexales | f__Roseiflexaceae | g__uncultured | s__uncultured_bacterium |
| JN591313 | Bacteria | p__Proteobacteria | c__Alphaproteobacteria | o__Sphingomonadales | f__Sphingomonadaceae | g__Sphingobium |  |
| MK177529 | Bacteria | p__Proteobacteria | c__Gammaproteobacteria | o__Xanthomonadales | f__Xanthomonadaceae |  |  |
| 59d6842cef43815a191df2ef30c80166 | Bacteria | p__Bacteroidota | c__Bacteroidia | o__Bacteroidales | f__Bacteroidaceae | g__Bacteroides | s__Bacteroides_plebeius |
| G900101065_1 | Bacteria | p__Firmicutes | c__Bacilli | o__Staphylococcales | f__Staphylococcaceae | g__Jeotgalicoccus |  |
| 0b1fac1f786dbe94260074cf7212a238 | Bacteria | p__Myxococcota |  |  |  |  |  |
| 04956026 | Bacteria | p__Actinobacteriota | c__Thermoleophilia | o__Gaiellales | f__uncultured | g__uncultured |  |
| 6e3a8fd08537bf7df7204a0170021b16 | Bacteria | p__Bacteroidota | c__Bacteroidia | o__Bacteroidales |  |  |  |
| 14522352 | Bacteria | p__Gemmatimonadota | c__Gemmatimonadetes | o__Gemmatimonadales | f__Gemmatimonadaceae | g__uncultured |  |
| d0b0fff45e724726173d86c8d547e98b | Bacteria | p__Actinobacteriota | c__Actinobacteria | o__Frankiales | f__Geodermatophilaceae |  |  |
| 6d3ca4da75301140a325d74604e6f505 | Bacteria | p__Actinobacteriota | c__Actinobacteria | o__Micrococcales | f__Microbacteriaceae | g__Microbacterium |  |
| 02242795 | Bacteria | p__Patescibacteria | c__Saccharimonadia | o__Saccharimonadales | f__Saccharimonadaceae | g__TM7x |  |
| 11e1cf24e3768a901380c9e2ac2827c4 | Bacteria | p__Bacteroidota | c__Bacteroidia | o__Sphingobacteriales | f__Sphingobacteriaceae | g__Sphingobacterium |  |
| 05032961 | Bacteria | p__Actinobacteriota | c__Actinobacteria | o__Corynebacteriales | f__Corynebacteriaceae | g__Corynebacterium |  |
| c62b11fe9feb7dfd7a5bc7593e039535 | Bacteria | p__Proteobacteria | c__Gammaproteobacteria | o__Burkholderiales | f__Rhodocyclaceae |  |  |
| 8661fd5b100dffd75a5fa548bbe22627 | Bacteria | p__Verrucomicrobiota | c__Verrucomicrobiae | o__Chthoniobacterales | f__Chthoniobacteraceae | g__Candidatus_Udaeobacter |  |
| 8b9a027a4ebee07150c43021311a027d | Bacteria | p__Acidobacteriota | c__Acidobacteriae | o__Bryobacterales | f__Bryobacteraceae | g__Bryobacter | s__uncultured_Holophaga |
| 629f76ff739171c8d70de1b882772283 | Bacteria | p__Proteobacteria | c__Gammaproteobacteria | o__Burkholderiales | f__Rhodocyclaceae | g__Thauera |  |
| G000294575_1 | Bacteria | p__Actinobacteriota | c__Actinobacteria | o__Frankiales | f__Sporichthyaceae | g__hgcI_clade |  |
| 09edd38b491656f78dd3b63ea1145258 | Bacteria | p__Firmicutes | c__Clostridia | o__Oscillospirales | f__Ruminococcaceae | g__Faecalibacterium | s__uncultured_bacterium |
| MJ034-1-barcode31-umi22901bins-ubs-7 | Bacteria | p__RCP2-54 | c__RCP2-54 | o__RCP2-54 | f__RCP2-54 | g__RCP2-54 |  |
| be39d6aee969cb1edbdc18d94eec7117 | Bacteria | p__Acidobacteriota | c__Blastocatellia | o__Pyrinomonadales | f__Pyrinomonadaceae | g__RB41 |  |
| 64e4b6bb7d5aa62eb0a49da39151b5f5 | Bacteria | p__Bacteroidota | c__Bacteroidia | o__Sphingobacteriales | f__Sphingobacteriaceae | g__Pedobacter |  |
| 10577216 | Bacteria | p__Bacteroidota | c__Bacteroidia | o__Sphingobacteriales | f__Sphingobacteriaceae | g__Sphingobacterium |  |
| 2691af2f453525c77fddcdb53ad51d58 | Bacteria | p__Actinobacteriota | c__Actinobacteria | o__Micromonosporales | f__Micromonosporaceae |  |  |
| 21961184 | Bacteria | p__Firmicutes | c__Clostridia | o__Lachnospirales | f__Lachnospiraceae |  |  |
| 675a68c5202d9602e889ba27ee14dcc1 | Bacteria | p__Proteobacteria | c__Gammaproteobacteria | o__Enterobacterales | f__Enterobacteriaceae |  |  |
| 34684dd8e24fe6315fc4ab284e3e5ab5 | Bacteria | p__Firmicutes | c__Bacilli | o__Paenibacillales | f__Paenibacillaceae | g__Paenibacillus |  |
| 49e922de41989a94d759c23fcbf85c85 | Bacteria | p__Proteobacteria | c__Gammaproteobacteria | o__Burkholderiales | f__Oxalobacteraceae |  |  |
| a0a2fd42dcdfcb4445b0ba01e305b27d | Bacteria | p__Chloroflexi | c__KD4-96 | o__KD4-96 | f__KD4-96 | g__KD4-96 | s__uncultured_bacterium |
| 81b062d0d6a43dd33ab5836ebbf3d848 | Bacteria | p__Proteobacteria | c__Gammaproteobacteria | o__Alteromonadales | f__Shewanellaceae | g__Shewanella |  |
| 03165354 | Bacteria | p__Planctomycetota | c__Phycisphaerae | o__Tepidisphaerales | f__WD2101_soil_group | g__WD2101_soil_group |  |
| 1cf97507ee8d11255ee92f925c9ec0d7 | Bacteria | p__Actinobacteriota | c__Thermoleophilia | o__Gaiellales | f__Gaiellaceae | g__Gaiella |  |
| 90268de9a48ac00f41bd241c58aabb20 | Bacteria | p__Proteobacteria | c__Alphaproteobacteria | o__Sphingomonadales | f__Sphingomonadaceae | g__Sphingomonas |  |
| 9e18f5f21e93cab6e302e252d3322c74 | Bacteria | p__Actinobacteriota | c__Actinobacteria | o__Micrococcales | f__Microbacteriaceae | g__Microbacterium |  |
| bd5b4a6774c160ac590b129c33c76b67 | Bacteria | p__Firmicutes | c__Clostridia | o__Lachnospirales | f__Lachnospiraceae |  |  |
| RS-GCF-014207955.1-NZ-JACHLK010000040.1 | Bacteria | p__Proteobacteria | c__Gammaproteobacteria | o__Burkholderiales | f__Comamonadaceae | g__Acidovorax |  |
| X89939 | Bacteria | p__Actinobacteriota | c__Actinobacteria | o__Streptosporangiales | f__Streptosporangiaceae | g__Sphaerisporangium |  |
| MJ031-1-barcode30-umi18151bins-ubs-24 | Bacteria | p__Bacteroidota | c__Bacteroidia | o__Cytophagales | f__Microscillaceae | g__uncultured |  |
| 643488102e600f88fbdc06d43dcb2818 | Bacteria | p__Actinobacteriota | c__Actinobacteria | o__Streptomycetales | f__Streptomycetaceae | g__Streptomyces |  |
| 3376a78ec58e3ab0e6f6218af70f036f | Bacteria | p__Bacteroidota | c__Bacteroidia | o__Sphingobacteriales | f__Sphingobacteriaceae | g__Sphingobacterium |  |
| bcfe8dcd1ff5c285e60b53ba0d3a560f | Bacteria | p__Firmicutes | c__Bacilli | o__Bacillales | f__Bacillaceae | g__Bacillus |  |
| 5a37c341d0883a5e7723e0f52702550e | Bacteria | p__Bacteroidota | c__Bacteroidia | o__Sphingobacteriales | f__Sphingobacteriaceae | g__Mucilaginibacter |  |
| 04954845 | Bacteria | p__Actinobacteriota | c__Rubrobacteria | o__Rubrobacterales | f__Rubrobacteriaceae | g__Rubrobacter | s__uncultured_bacterium |
| c9dde8732241292bb2d1f61f8d267723 | Bacteria | p__Proteobacteria | c__Alphaproteobacteria | o__Reyranellales | f__Reyranellaceae | g__Reyranella |  |
| 4d1816be62ff889af648a0f210095c89 | Bacteria | p__Proteobacteria | c__Alphaproteobacteria | o__Rhizobiales | f__A0839 | g__A0839 |  |
| 38213614b7de68fe466173b3ca419778 | Bacteria | p__Proteobacteria | c__Alphaproteobacteria | o__Tistrellales | f__Geminicoccaceae | g__Candidatus_Alysiosphaera |  |
| beb034a73f3be14f86e5127a80e07bee | Bacteria | p__Gemmatimonadota | c__Gemmatimonadetes | o__Gemmatimonadales | f__Gemmatimonadaceae | g__uncultured | s__uncultured_proteobacterium |
| a0ef556a8210c38c43519c3b79fa247a | Bacteria | p__Proteobacteria | c__Gammaproteobacteria | o__Xanthomonadales | f__Xanthomonadaceae | g__Stenotrophomonas |  |
| 20d206e59394c13f6106eef65c86a4e6 | Bacteria | p__Actinobacteriota | c__Actinobacteria | o__Frankiales | f__Frankiaceae | g__Jatrophihabitans |  |
| 05004ce38035b6f91b970d56713acdf3 | Bacteria | p__Proteobacteria | c__Alphaproteobacteria | o__Sphingomonadales | f__Sphingomonadaceae |  |  |
| f7f876bfbfd0913284ca3462c5021670 | Bacteria | p__Proteobacteria | c__Gammaproteobacteria | o__Burkholderiales | f__Alcaligenaceae |  |  |
| 22320acb4f75454c7f5547843d415b72 | Bacteria | p__Bacteroidota | c__Bacteroidia | o__Sphingobacteriales | f__Sphingobacteriaceae | g__Mucilaginibacter |  |
| 05034614 | Bacteria | p__Firmicutes | c__Bacilli | o__Lactobacillales | f__Streptococcaceae | g__Lactococcus |  |
| G002420045_1 | Bacteria | p__Nitrospirota | c__Nitrospiria | o__Nitrospirales | f__Nitrospiraceae | g__Nitrospira |  |
| 02405653 | Bacteria | p__Actinobacteriota | c__Actinobacteria | o__Propionibacteriales | f__Propionibacteriaceae | g__Microlunatus |  |
| 57775516161b8b8f28cf889bee97adc9 | Bacteria | p__Chloroflexi | c__Ktedonobacteria | o__Ktedonobacterales | f__Ktedonobacteraceae | g__Ktedonobacter |  |
| 30fbd6a45250b7e468b13fcbab63c717 | Bacteria | p__Chloroflexi | c__KD4-96 | o__KD4-96 | f__KD4-96 | g__KD4-96 |  |
| X79287 | Bacteria | p__Actinobacteriota | c__Actinobacteria | o__Corynebacteriales | f__Nocardiaceae | g__Gordonia |  |
| 02858590 | Bacteria | p__Chloroflexi | c__Chloroflexia | o__Chloroflexales | f__Roseiflexaceae | g__uncultured | s__uncultured_bacterium |
| RS-GCF-000620265.1-NZ-JHVS01000030.1 | Bacteria | p__Firmicutes | c__Bacilli | o__Staphylococcales | f__Staphylococcaceae | g__Staphylococcus |  |
| 13717965 | Bacteria | p__Gemmatimonadota | c__Gemmatimonadetes | o__Gemmatimonadales | f__Gemmatimonadaceae | g__Gemmatimonas |  |
| f42f3cc02e58344eb59ee4e0b67233c5 | Bacteria | p__Acidobacteriota | c__Acidobacteriae | o__Acidobacteriales | f__Acidobacteriaceae_(Subgroup_1) |  |  |
| 32214f74de477832dcefc6d8d3f317e7 | Bacteria | p__Proteobacteria | c__Gammaproteobacteria | o__Pseudomonadales | f__Moraxellaceae | g__Acinetobacter |  |
| e1dda6717c2f040ef06913889fcec781 | Bacteria | p__NB1-j | c__NB1-j | o__NB1-j | f__NB1-j | g__NB1-j |  |
| 2822ed5244b8513cde1a774b6286410e | Bacteria | p__Proteobacteria | c__Alphaproteobacteria | o__Rhizobiales | f__Xanthobacteraceae | g__uncultured |  |
| b85d69976f74f1f4578ade1e55b4b4ef | Bacteria | p__Gemmatimonadota | c__Gemmatimonadetes | o__Gemmatimonadales | f__Gemmatimonadaceae | g__uncultured |  |
| RS-GCF-000368985.1-NZ-KB849760.1--2 | Bacteria | p__Proteobacteria | c__Gammaproteobacteria | o__Pseudomonadales | f__Moraxellaceae | g__Acinetobacter |  |
| db6abf15875bb8c9365b1d75e72a8846 | Bacteria | p__Proteobacteria | c__Alphaproteobacteria |  |  |  |  |
| 007e2dfdf46126f9982a352a597aee49 | Bacteria | p__Proteobacteria | c__Gammaproteobacteria | o__Burkholderiales | f__Oxalobacteraceae |  |  |
| EU707560 | Bacteria | p__Proteobacteria | c__Alphaproteobacteria | o__Sphingomonadales | f__Sphingomonadaceae | g__Sphingomonas |  |
| RS-GCF-015471265.1-NZ-BLVS01000028.1 | Bacteria | p__Proteobacteria | c__Gammaproteobacteria | o__Pseudomonadales | f__Pseudomonadaceae | g__Pseudomonas |  |
| MJ031-1-barcode38-umi21486bins-ubs-17 | Bacteria | p__Firmicutes | c__Clostridia | o__Clostridia_UCG-014 | f__Clostridia_UCG-014 | g__Clostridia_UCG-014 |  |
| 1872215e69dd560a2c309164dd92ce20 | Bacteria | p__Firmicutes | c__Bacilli | o__Lactobacillales | f__Lactobacillaceae | g__Lactobacillus |  |
| MJ034-2-barcode50-umi3999bins-ubs-82 | Bacteria | p__Bacteroidota | c__Bacteroidia | o__Chitinophagales | f__Chitinophagaceae |  |  |
| 92ede805bf5cee1f05fbbb77ad89e163 | Bacteria | p__Proteobacteria | c__Gammaproteobacteria | o__Pseudomonadales | f__Moraxellaceae | g__Acinetobacter |  |
| MJ009-1-barcode40-umi76421bins-ubs-4 | Bacteria | p__Firmicutes | c__Bacilli | o__Lactobacillales | f__Carnobacteriaceae | g__Atopostipes |  |
| RS-GCF-003480145.1-NZ-QUIO01000097.1 | Bacteria | p__Firmicutes | c__Clostridia | o__Lachnospirales | f__Lachnospiraceae | g__Blautia |  |
| fe9620d4334add7087e824131c2f0e4e | Bacteria | p__Bacteroidota | c__Bacteroidia | o__Bacteroidales | f__Rikenellaceae | g__DMER64 |  |
| 22253368 | Bacteria |  |  |  |  |  |  |
| 09441b864954f47911e032ed0f1b26ba | Bacteria | p__Proteobacteria | c__Gammaproteobacteria | o__Pseudomonadales | f__Moraxellaceae | g__Acinetobacter | s__Acinetobacter_towneri |
| ec5045801b166399c8d94d74a0b4a6dc | Bacteria | p__Proteobacteria | c__Alphaproteobacteria | o__Rhizobiales | f__Xanthobacteraceae | g__uncultured |  |
| 04938259 | Bacteria | p__Proteobacteria | c__Gammaproteobacteria | o__Oceanospirillales | f__Halomonadaceae | g__Halomonas |  |
| 05013902 | Bacteria | p__Bacteroidota | c__Bacteroidia | o__Flavobacteriales | f__Weeksellaceae | g__Chryseobacterium |  |
| 58ce7a574290dbce6843eea028ff8df3 | Bacteria | p__Proteobacteria | c__Gammaproteobacteria | o__Enterobacterales | f__Enterobacteriaceae |  |  |
| 85b53445a618cc764bcd84da4c080c5a | Bacteria | p__Firmicutes | c__Bacilli | o__Lactobacillales | f__Lactobacillaceae | g__Lactobacillus |  |
| MJ010-1-barcode29-umi105014bins-ubs-6 | Bacteria | p__Bacteroidota | c__Bacteroidia | o__Sphingobacteriales | f__Sphingobacteriaceae | g__Sphingobacterium |  |
| MJ020-1-barcode44-umi10522bins-ubs-36 | Bacteria | p__Firmicutes | c__Clostridia | o__Lachnospirales | f__Lachnospiraceae | g__[Eubacterium]_ventriosum_group |  |
| 30f60f46b1587b0c378be8c331416bc6 | Bacteria | p__Firmicutes | c__Bacilli | o__Erysipelotrichales | f__Erysipelotrichaceae | g__Allobaculum |  |
| 6ffe5eb74d29cc7c24296c6d64790164 | Bacteria | p__Proteobacteria | c__Gammaproteobacteria | o__Pseudomonadales | f__Pseudomonadaceae | g__Pseudomonas |  |
| MJ032-1-barcode33-umi105172bins-ubs-3 | Bacteria | p__Firmicutes | c__Clostridia | o__Oscillospirales | f__Butyricicoccaceae | g__Butyricicoccus |  |
| 39bf0068d472dca80dcc871d300cdecd | Bacteria | p__Proteobacteria | c__Alphaproteobacteria | o__Rhizobiales | f__KF-JG30-B3 | g__KF-JG30-B3 |  |
| 87916f9b84aef4090cdc43da21f95b3c | Bacteria | p__Bacteroidota | c__Bacteroidia | o__Sphingobacteriales | f__AKYH767 | g__AKYH767 | s__uncultured_Sphingobacteriales |
| 1a683546a0454067f3f77f2932cc4682 | Bacteria | p__Proteobacteria | c__Gammaproteobacteria | o__Pseudomonadales | f__Moraxellaceae | g__Acinetobacter |  |
| 078a8d38ad4f4b46fe97709669d1cecf | Bacteria | p__Bacteroidota | c__Bacteroidia | o__Sphingobacteriales | f__Sphingobacteriaceae | g__Sphingobacterium |  |
| ffc0a099cb4be1b4908f9c8406175319 | Bacteria | p__Actinobacteriota | c__Thermoleophilia | o__Solirubrobacterales | f__Solirubrobacteraceae | g__Solirubrobacter | s__uncultured_bacterium |
| bd54c5e71278519247acb828ce357263 | Bacteria | p__Chloroflexi | c__Ktedonobacteria | o__C0119 | f__C0119 | g__C0119 | s__uncultured_Chloroflexi |
| MJ010-1-barcode41-umi141737bins-ubs-3 | Bacteria | p__Bacteroidota | c__Bacteroidia | o__Bacteroidales | f__Bacteroidaceae | g__Bacteroides |  |
| 1e3ccde977e2b8b781ff4a92bf5876b9 | Bacteria | p__Proteobacteria | c__Gammaproteobacteria | o__Alteromonadales |  |  |  |
| MJ030-2-barcode57-umi810bins-ubs-152 | Bacteria | p__Firmicutes | c__Clostridia | o__Clostridiales | f__Clostridiaceae |  |  |
| f5b2643f83eb399b11308f3fc443dc10 | Bacteria | p__Proteobacteria | c__Gammaproteobacteria | o__Burkholderiales | f__SC-I-84 | g__SC-I-84 |  |
| 11561113 | Bacteria | p__Proteobacteria | c__Alphaproteobacteria | o__Rhizobiales | f__Methyloligellaceae |  |  |
| GQ505294 | Bacteria | p__Proteobacteria | c__Gammaproteobacteria | o__Alteromonadales | f__Alteromonadaceae | g__Alishewanella |  |
| 80024a9015e16bb838a46730edde3ad3 | Bacteria | p__Proteobacteria | c__Alphaproteobacteria | o__Paracaedibacterales | f__Paracaedibacteraceae | g__Candidatus_Paracaedibacter |  |
| RS-GCF-017916455.1-NZ-JAFMSY010000006.1 | Bacteria | p__Actinobacteriota | c__Actinobacteria | o__Propionibacteriales | f__Nocardioidaceae | g__Nocardioides |  |
| 347163495a561626b6b452dc9b10cfc4 | Bacteria | p__Bacteroidota | c__Bacteroidia | o__Bacteroidales | f__Muribaculaceae | g__Muribaculaceae |  |
| 4244911769eb13adbfd05be9dc0d3212 | Bacteria | p__Proteobacteria | c__Alphaproteobacteria | o__Rhizobiales | f__Beijerinckiaceae | g__Microvirga |  |
| 03e69adba76a0a9ec9bb722e2103abac | Bacteria | p__Bacteroidota | c__Bacteroidia | o__Bacteroidales | f__Prevotellaceae | g__Prevotella |  |
| G000980235_1 | Bacteria | p__Firmicutes | c__Clostridia | o__Clostridiales | f__Clostridiaceae | g__Clostridium_sensu_stricto_1 |  |
| KU201963 | Bacteria | p__Actinobacteriota | c__Actinobacteria | o__Propionibacteriales | f__Nocardioidaceae | g__Nocardioides |  |
| GB-GCA-013817065.1-JACDDJ010000002.1 | Bacteria | p__Acidobacteriota | c__Vicinamibacteria | o__Vicinamibacterales | f__uncultured | g__uncultured | s__uncultured_Acidobacteriales |
| MJ030-1-barcode36-umi16398bins-ubs-29 | Bacteria | p__Firmicutes | c__Bacilli | o__Paenibacillales | f__Paenibacillaceae | g__Paenibacillus |  |
| 5f347843c74a9102a7f30b7a369fe342 | Bacteria | p__Proteobacteria | c__Gammaproteobacteria |  |  |  |  |
| GB-GCA-910573865.1-CAJTBA010000041.1 | Bacteria | p__Bacteroidota | c__Bacteroidia | o__Bacteroidales | f__Bacteroidaceae | g__Bacteroides |  |
| EU009209 | Bacteria | p__Proteobacteria | c__Alphaproteobacteria | o__Sphingomonadales | f__Sphingomonadaceae |  |  |
| RS-GCF-018785515.1-NZ-WQPY01000015.1 | Bacteria | p__Firmicutes | c__Clostridia | o__Oscillospirales | f__Butyricicoccaceae | g__Butyricicoccus | s__Butyricicoccus_sp. |
| 04908556 | Bacteria | p__Proteobacteria | c__Alphaproteobacteria | o__Rhizobiales | f__Beijerinckiaceae | g__1174-901-12 | s__uncultured_bacterium |
| 10f943879565b68ed2d8a1709d54f8aa | Bacteria | p__Planctomycetota | c__Planctomycetes | o__Gemmatales | f__Gemmataceae | g__uncultured |  |
| 0dbb261aa579320ad34547fe2b3e1a77 | Bacteria | p__Firmicutes | c__Bacilli | o__Lactobacillales | f__Lactobacillaceae | g__Pediococcus |  |
| 16612524 | Bacteria | p__Firmicutes | c__Clostridia | o__Lachnospirales | f__Lachnospiraceae |  |  |
| b79989d0e6b643947d3138cc59badced | Bacteria | p__Actinobacteriota | c__MB-A2-108 | o__MB-A2-108 | f__MB-A2-108 | g__MB-A2-108 | s__uncultured_Kineosporiaceae |
| 13184505 | Bacteria | p__Bacteroidota | c__Bacteroidia | o__Cytophagales | f__Hymenobacteraceae | g__Hymenobacter |  |
| 32413e1f50b19469ee6ff5be2f72ce9f | Bacteria | p__Proteobacteria | c__Gammaproteobacteria | o__Burkholderiales | f__Nitrosomonadaceae | g__Ellin6067 |  |
| G000403275_1 | Bacteria | p__Firmicutes | c__Clostridia | o__Lachnospirales | f__Lachnospiraceae | g__Blautia |  |
| AB122751 | Bacteria | p__Actinobacteriota | c__Actinobacteria | o__Streptomycetales | f__Streptomycetaceae |  |  |
| MJ006-2-barcode58-umi133299bins-ubs-4 | Bacteria | p__Firmicutes | c__Clostridia | o__Lachnospirales | f__Lachnospiraceae | g__GCA-900066575 |  |
| 5a0ce7ac8cca1bd17556e09a571cf4c1 | Bacteria | p__Firmicutes | c__Bacilli | o__Alicyclobacillales | f__Alicyclobacillaceae | g__Alicyclobacillus |  |
| 1abc196085419a1e8f4021cbc1e3425b | Bacteria | p__Proteobacteria | c__Alphaproteobacteria | o__Acetobacterales | f__Acetobacteraceae | g__Gluconobacter |  |
| c738ca3ccf64bd61ce12afd1e270fffc | Bacteria | p__Proteobacteria | c__Gammaproteobacteria | o__Alteromonadales | f__Shewanellaceae | g__Shewanella |  |
| 02360385 | Bacteria | p__Proteobacteria | c__Alphaproteobacteria | o__Rhizobiales | f__Hyphomicrobiaceae | g__Pedomicrobium | s__uncultured_Alphaproteobacteria |
| ef9a8080f5a354b0b989af75813ce94d | Bacteria | p__Firmicutes | c__Clostridia | o__Peptostreptococcales-Tissierellales | f__Fusibacteraceae | g__Fusibacter |  |
| GB-GCA-018436805.1-JAHDKS010000001.1 | Bacteria | p__Bacteroidota | c__Bacteroidia | o__Bacteroidales | f__Rikenellaceae | g__DMER64 |  |
| fd58d18ebee6ba6559b6cb87834df508 | Bacteria | p__Actinobacteriota | c__Actinobacteria | o__Bifidobacteriales | f__Bifidobacteriaceae | g__Bifidobacterium |  |
| MJ034-2-barcode50-umi15922bins-ubs-5 | Bacteria | p__Bacteroidota | c__Bacteroidia | o__Chitinophagales | f__Chitinophagaceae |  |  |
| 4d9e7e8fe9288a4b1b7ddd8eb18674ad | Bacteria | p__Firmicutes | c__Clostridia | o__Lachnospirales | f__Lachnospiraceae |  |  |
| 3dbeb900e1c21f3d5b43e68d13f3fae0 | Bacteria | p__Bacteroidota | c__Bacteroidia | o__Flavobacteriales | f__NS9_marine_group | g__NS9_marine_group |  |
| 10246995 | Bacteria | p__Bacteroidota | c__Bacteroidia | o__Bacteroidales | f__Rikenellaceae | g__Alistipes | s__uncultured_bacterium |
| G000091545_1 | Bacteria | p__Deinococcota | c__Deinococci | o__Thermales | f__Thermaceae | g__Thermus | s__Thermus_thermophilus |
| 04206887 | Bacteria | p__Chloroflexi | c__Ktedonobacteria | o__Ktedonobacterales | f__JG30-KF-AS9 | g__JG30-KF-AS9 | s__uncultured_bacterium |
| 04911188 | Bacteria | p__Proteobacteria | c__Gammaproteobacteria | o__Xanthomonadales | f__Xanthomonadaceae | g__Stenotrophomonas |  |
| 9b3050bce694a966cf67abd51ad82adb | Bacteria | p__Proteobacteria | c__Alphaproteobacteria | o__Caulobacterales | f__Caulobacteraceae |  |  |
| RS-GCF-006716945.1-NZ-VFPX01000010.1 | Bacteria | p__Actinobacteriota | c__Actinobacteria | o__Micrococcales | f__Intrasporangiaceae | g__Terrabacter |  |
| 497db244df7b77f1f1dc94d2ecff30a5 | Bacteria |  |  |  |  |  |  |
| 4299fa77389be6f2f1b859c417a68b0c | Bacteria | p__Firmicutes | c__Negativicutes | o__Veillonellales-Selenomonadales | f__Veillonellaceae | g__Allisonella | s__uncultured_bacterium |
| G004555055_3 | Bacteria | p__Desulfobacterota | c__Desulfovibrionia | o__Desulfovibrionales | f__Desulfovibrionaceae | g__Desulfovibrio | s__bacterium |
| RS-GCF-002144475.1-NZ-MBOU01000032.1 | Bacteria | p__Proteobacteria | c__Gammaproteobacteria | o__Enterobacterales | f__Enterobacteriaceae |  |  |
| bfeffedcd2f11827c0e175ae07fe1254 | Bacteria | p__Firmicutes | c__Clostridia | o__Clostridiales | f__Clostridiaceae | g__Clostridium_sensu_stricto_12 |  |
| 621f33c02c5ebd06d2dba974d989fedd | Bacteria | p__Bacteroidota | c__Bacteroidia | o__Cytophagales | f__Spirosomaceae | g__Leadbetterella | s__Leadbetterella_sp. |
| MJ030-2-barcode65-umi105203bins-ubs-3 | Bacteria | p__Proteobacteria | c__Gammaproteobacteria | o__Burkholderiales | f__Oxalobacteraceae |  |  |
| eb7b3828c2398f69013dd70cc2b5fc1e | Bacteria | p__Chloroflexi | c__Anaerolineae | o__Caldilineales | f__Caldilineaceae | g__uncultured |  |
| 4eb270e3af9d584acb00f5e4d093940c | Bacteria | p__Chloroflexi | c__Anaerolineae | o__SJA-15 | f__SJA-15 | g__SJA-15 |  |
| dab82b27707f9f175a125dcb07c0b7e5 | Bacteria | p__Proteobacteria | c__Gammaproteobacteria | o__Xanthomonadales | f__Rhodanobacteraceae | g__Ahniella |  |
| 04918280 | Bacteria | p__Bacteroidota | c__Bacteroidia | o__Sphingobacteriales | f__Sphingobacteriaceae | g__Sphingobacterium |  |
| b4f4072c46ae444921e926e9402b57f0 | Bacteria | p__Proteobacteria | c__Alphaproteobacteria | o__Caulobacterales | f__Caulobacteraceae |  |  |
| AM232810 | Bacteria | p__Bacteroidota | c__Bacteroidia | o__Flavobacteriales | f__Weeksellaceae | g__Chryseobacterium |  |
| 02247844 | Bacteria | p__Proteobacteria | c__Alphaproteobacteria | o__Rhizobiales | f__Rhizobiaceae |  |  |
| 91d8ec763e0bd21cdfaa248412f7c198 | Bacteria | p__Firmicutes | c__Bacilli | o__Lactobacillales | f__Enterococcaceae | g__Enterococcus |  |
| AB023970 | Bacteria | p__Firmicutes | c__Clostridia | o__Peptostreptococcales-Tissierellales | f__Peptostreptococcaceae | g__Peptoclostridium |  |
| 5c44e84136bc583aca2b5589a1d32ac9 | Bacteria | p__Actinobacteriota | c__Actinobacteria | o__Frankiales |  |  |  |
| 535b579746ea16559f309b265c1ed5e8 | Bacteria | p__Firmicutes | c__Clostridia | o__Peptococcales | f__Peptococcaceae | g__uncultured | s__uncultured_bacterium |
| 666cd20ccc4e7b92ec8583581c868879 | Bacteria | p__Verrucomicrobiota | c__Verrucomicrobiae | o__Chthoniobacterales | f__Chthoniobacteraceae |  |  |
| 9efedcdfa4095e9a5a5394beb7216d96 | Bacteria | p__Actinobacteriota | c__Actinobacteria | o__Pseudonocardiales | f__Pseudonocardiaceae | g__Lechevalieria |  |
| 3f210cc25b027f90a59f209c9f12047d | Bacteria |  |  |  |  |  |  |
| MJ007-1-barcode42-umi12956bins-ubs-23 | Bacteria | p__Proteobacteria | c__Gammaproteobacteria | o__Pseudomonadales | f__Moraxellaceae | g__Acinetobacter | s__Acinetobacter_variabilis |
| HM165487 | Bacteria | p__Actinobacteriota | c__Actinobacteria | o__Corynebacteriales | f__Corynebacteriaceae | g__Corynebacterium | s__Corynebacterium_nuruki |
| DQ411541 | Bacteria | p__Actinobacteriota | c__Actinobacteria | o__Propionibacteriales | f__Nocardioidaceae | g__Aeromicrobium |  |
| 021235a8d0c61307cc359ee409b12efd | Bacteria | p__Acidobacteriota | c__Acidobacteriae | o__Acidobacteriales | f__Acidobacteriaceae_(Subgroup_1) |  |  |
| 04893402 | Bacteria | p__Myxococcota | c__Polyangia | o__Polyangiales | f__Sandaracinaceae | g__uncultured |  |
| 76be4061ecb28752ddf6aa48a501374e | Bacteria | p__Proteobacteria | c__Gammaproteobacteria | o__Alteromonadales | f__Shewanellaceae | g__Shewanella |  |
| daea0334e177d997bf5a005f950ab014 | Bacteria | p__Actinobacteriota | c__Actinobacteria | o__Micrococcales | f__Intrasporangiaceae |  |  |
| 2d11571c39a3fd33410f9f4021a044ad | Bacteria | p__Proteobacteria | c__Alphaproteobacteria | o__Rhizobiales | f__Xanthobacteraceae | g__uncultured |  |
| ba55959d2465294c599ec92705c6d2ea | Bacteria | p__Bacteroidota | c__Bacteroidia | o__Flavobacteriales | f__Weeksellaceae | g__Chryseobacterium | s__Chryseobacterium_hungaricum |
| 6fd501cb38a931b84eec6e9b214ebb3b | Bacteria | p__Bacteroidota | c__Bacteroidia | o__Sphingobacteriales | f__Sphingobacteriaceae | g__Sphingobacterium | s__Sphingobacterium_humi |
| 4a2d9cbbc7b4a62d3e6c17d6e878af7b | Bacteria | p__Acidobacteriota | c__Vicinamibacteria | o__Vicinamibacterales | f__uncultured | g__uncultured |  |
| MJ034-1-barcode37-umi20902bins-ubs-10 | Bacteria | p__Proteobacteria | c__Alphaproteobacteria | o__Rhizobiales | f__Hyphomicrobiaceae | g__Pedomicrobium |  |
| b7264f3d4e16a824fe51851fbbbc3396 | Bacteria | p__Proteobacteria | c__Alphaproteobacteria | o__Azospirillales | f__uncultured | g__uncultured | s__uncultured_bacterium |
| 12843557 | Bacteria | p__Bacteroidota | c__Bacteroidia | o__Chitinophagales | f__Chitinophagaceae | g__Segetibacter |  |
| 2bdc0d419df0f9a60a22824133309698 | Bacteria | p__Proteobacteria | c__Gammaproteobacteria | o__Burkholderiales | f__Nitrosomonadaceae | g__Ellin6067 |  |
| c42f940130e62be92221c4de4982189d | Bacteria | p__Chloroflexi | c__Chloroflexia | o__Chloroflexales | f__Roseiflexaceae | g__uncultured | s__uncultured_bacterium |
| 20406312 | Bacteria | p__Chloroflexi | c__JG30-KF-CM66 | o__JG30-KF-CM66 | f__JG30-KF-CM66 | g__JG30-KF-CM66 | s__uncultured_Caldilinea |
| 0541426be153cbd7a480cc2578924e9a | Bacteria |  |  |  |  |  |  |
| c59d658e9677b57c6122675239a19472 | Bacteria | p__Acidobacteriota | c__Vicinamibacteria | o__Vicinamibacterales | f__Vicinamibacteraceae | g__Vicinamibacteraceae |  |
| 91f095f15db35026b45f879bcd7a4893 | Bacteria | p__Firmicutes | c__Bacilli | o__Erysipelotrichales | f__Erysipelotrichaceae | g__ZOR0006 |  |
| c768c695d4467b2b7bb0d8ad271dc721 | Bacteria | p__Firmicutes | c__Bacilli | o__Bacillales | f__Bacillaceae | g__Bacillus |  |
| ea1be0b5620f8eb5db94b5e7c085f6c5 | Bacteria | p__Proteobacteria | c__Alphaproteobacteria | o__Rhizobiales | f__Rhizobiales_Incertae_Sedis | g__Phreatobacter |  |
| fd3094feb1d24a3f40259b44d92e3376 | Bacteria | p__Proteobacteria | c__Alphaproteobacteria | o__Sphingomonadales | f__Sphingomonadaceae | g__Sphingomonas |  |
| 05030339 | Bacteria | p__Proteobacteria | c__Gammaproteobacteria | o__Burkholderiales | f__Comamonadaceae |  |  |
| RS-GCF-000474415.1-NZ-AUQF01000057.1 | Bacteria | p__Proteobacteria | c__Gammaproteobacteria | o__Enterobacterales | f__Enterobacteriaceae |  |  |
| DQ454123 | Bacteria | p__Proteobacteria | c__Alphaproteobacteria | o__Rhizobiales | f__Rhizobiaceae | g__Allorhizobium-Neorhizobium-Pararhizobium-Rhizobium |  |
| 83c50001bb0c104633f718bed2821976 | Bacteria | p__Firmicutes | c__Bacilli | o__Bacillales |  |  |  |
| AB558487 | Bacteria | p__Firmicutes | c__Bacilli | o__Erysipelotrichales | f__Erysipelotrichaceae | g__Faecalitalea | s__Faecalitalea_cylindroides |
| G000437635_1 | Bacteria | p__Proteobacteria | c__Gammaproteobacteria | o__Burkholderiales | f__Sutterellaceae | g__Parasutterella | s__Parasutterella_secunda |
| f30ddf360baad24e6e2cb52170be4b72 | Bacteria | p__Proteobacteria | c__Gammaproteobacteria | o__Burkholderiales | f__Nitrosomonadaceae | g__Ellin6067 |  |
| 8f73d0950973ffdbb1c2c1de4d28832b | Bacteria | p__Chloroflexi | c__Chloroflexia | o__Thermomicrobiales | f__JG30-KF-CM45 | g__JG30-KF-CM45 | s__metagenome |
| 405527190f1817b57e9f6ffb7d0ad4b2 | Bacteria | p__Proteobacteria | c__Gammaproteobacteria | o__Pseudomonadales | f__Moraxellaceae | g__Acinetobacter |  |
| 14840275 | Bacteria | p__Chloroflexi | c__Ktedonobacteria | o__Ktedonobacterales | f__JG30-KF-AS9 | g__JG30-KF-AS9 | s__uncultured_bacterium |
| 04257518 | Bacteria | p__Actinobacteriota | c__MB-A2-108 | o__MB-A2-108 | f__MB-A2-108 | g__MB-A2-108 |  |
| 8c37f1780c1791be3b578a383c0eb6db | Bacteria | p__Proteobacteria | c__Gammaproteobacteria | o__Pseudomonadales | f__Moraxellaceae | g__Acinetobacter |  |
| a3cdbf2f744c6ddfd4847f30611c1017 | Bacteria | p__Proteobacteria | c__Alphaproteobacteria | o__uncultured | f__uncultured | g__uncultured | s__uncultured_Bradyrhizobiaceae |
| 1a7e39452581a5a4b5cebdf04fbe0702 | Bacteria | p__Bacteroidota | c__Bacteroidia | o__Flavobacteriales | f__Crocinitomicaceae | g__Fluviicola |  |
| 842f28a03fe01a5947144e910c0fe93e | Bacteria | p__Proteobacteria | c__Gammaproteobacteria | o__Pseudomonadales | f__Pseudomonadaceae | g__Pseudomonas |  |
| MJ030-2-barcode58-umi70582bins-ubs-8 | Bacteria | p__Bacteroidota | c__Bacteroidia | o__Chitinophagales | f__Saprospiraceae | g__uncultured |  |
| d26fa67daef9b78f12fdf155df9f8fa4 | Bacteria | p__Nitrospirota | c__Nitrospiria | o__Nitrospirales | f__Nitrospiraceae | g__Nitrospira | s__uncultured_Green |
| MN883860 | Bacteria | p__Actinobacteriota | c__Actinobacteria | o__Propionibacteriales | f__Nocardioidaceae | g__Nocardioides |  |
| f754b5a95e11a60955f9bd8a235026e2 | Bacteria | p__Proteobacteria | c__Gammaproteobacteria | o__Burkholderiales | f__Nitrosomonadaceae | g__Ellin6067 |  |
| 20b2922ddd3bcc62720504ed0aef87e6 | Bacteria | p__Verrucomicrobiota | c__Chlamydiae | o__Chlamydiales | f__Simkaniaceae | g__uncultured |  |
| f21e02e15576d689f31d8f26086e71fe | Bacteria | p__Acidobacteriota | c__Subgroup_25 | o__Subgroup_25 | f__Subgroup_25 | g__Subgroup_25 | s__uncultured_Acidobacteria |
| 04994152 | Bacteria | p__Proteobacteria | c__Alphaproteobacteria | o__Caulobacterales | f__Caulobacteraceae | g__Brevundimonas |  |
| 0867a339cfa07c9277ac77e4cd18a896 | Bacteria | p__Actinobacteriota | c__Actinobacteria | o__Corynebacteriales | f__Corynebacteriaceae | g__Corynebacterium | s__Corynebacterium_sputi |
| cf47d74ab28582ac8db5989a7d3af467 | Bacteria | p__Acidobacteriota | c__Acidobacteriae | o__Solibacterales | f__Solibacteraceae | g__Candidatus_Solibacter | s__uncultured_forest |
| 3afe32cfb279a496a6811678d69bbad3 | Bacteria | p__Methylomirabilota | c__Methylomirabilia | o__Rokubacteriales | f__Rokubacteriales | g__Rokubacteriales |  |
| GB-GCA-001422565.1-LMLI01000012.1 | Bacteria | p__Proteobacteria | c__Gammaproteobacteria | o__Enterobacterales | f__Yersiniaceae | g__Serratia |  |
| 18709489 | Bacteria | p__Actinobacteriota | c__Actinobacteria | o__Micrococcales | f__Microbacteriaceae |  |  |
| 9095e27b219e81264e07c09c9464f265 | Bacteria | p__Proteobacteria | c__Gammaproteobacteria | o__Pseudomonadales | f__Moraxellaceae | g__Acinetobacter |  |
| 2fd6b81c36e68d87dbe0908780c9a126 | Bacteria | p__Firmicutes | c__Clostridia | o__Lachnospirales | f__Lachnospiraceae | g__Blautia |  |
| 45ee5645a78d594ea66213831a2dcd9f | Bacteria | p__Proteobacteria | c__Gammaproteobacteria | o__Enterobacterales | f__Enterobacteriaceae |  |  |
| f5e9455c731681d3a4e6ca054b4bcc0d | Bacteria | p__Actinobacteriota | c__Acidimicrobiia | o__Microtrichales | f__Microtrichaceae | g__IMCC26207 |  |
| 80d185eef89b309e7fae2f75f6009939 | Bacteria | p__Desulfobacterota | c__Desulfovibrionia | o__Desulfovibrionales | f__Desulfovibrionaceae | g__uncultured | s__uncultured_bacterium |
| c9397154ff8021815078aa9f0cc9e5df | Bacteria | p__Actinobacteriota | c__Actinobacteria | o__Corynebacteriales | f__Mycobacteriaceae | g__Mycobacterium |  |
| 05030313 | Bacteria | p__Proteobacteria | c__Gammaproteobacteria | o__Burkholderiales | f__Comamonadaceae |  |  |
| 13746801 | Bacteria | p__Bacteroidota | c__Bacteroidia | o__Cytophagales | f__Hymenobacteraceae | g__Hymenobacter |  |
| 96e8ebb3c43991773f70a7554bf5562e | Bacteria | p__Proteobacteria | c__Gammaproteobacteria | o__Burkholderiales |  |  |  |
| GB-GCA-004769095.1-DUDR01000022.1 | Bacteria | p__Cyanobacteria | c__Vampirivibrionia | o__Obscuribacterales | f__Obscuribacteraceae | g__Candidatus_Obscuribacter |  |
| 6eafc7d2ab229a2b9ec555b2b4460d09 | Bacteria | p__Myxococcota | c__Polyangia | o__Polyangiales | f__Polyangiaceae | g__Pajaroellobacter |  |
| ad2f62384ff095235597bda08752ef9f | Bacteria | p__Methylomirabilota | c__Methylomirabilia | o__Rokubacteriales | f__Rokubacteriales | g__Rokubacteriales | s__uncultured_bacterium |
| 9e70f9f96b4b18b57a04ade8d059b24e | Bacteria | p__Proteobacteria | c__Gammaproteobacteria | o__Pseudomonadales | f__Pseudomonadaceae | g__Pseudomonas |  |
| 5e5c9c8fa25c765d2e698bd5f55a0175 | Bacteria | p__Proteobacteria | c__Gammaproteobacteria | o__Burkholderiales | f__Oxalobacteraceae | g__Massilia |  |
| 2251607ebb915de3ec35fda566ea3a8d | Bacteria | p__Planctomycetota | c__Planctomycetes | o__Pirellulales | f__Pirellulaceae | g__Pir4_lineage | s__metagenome |
| MJ034-2-barcode60-umi4480bins-ubs-64 | Bacteria | p__Acidobacteriota | c__Blastocatellia | o__Blastocatellales | f__Blastocatellaceae |  |  |
| 90b254a26d76958f10d5777c42e33176 | Bacteria | p__Proteobacteria | c__Gammaproteobacteria | o__Burkholderiales | f__Burkholderiaceae | g__Burkholderia-Caballeronia-Paraburkholderia |  |
| 58b26fe4d1af0525a12de2198e7fc7ce | Bacteria | p__Acidobacteriota | c__Thermoanaerobaculia | o__Thermoanaerobaculales | f__Thermoanaerobaculaceae | g__Subgroup_10 |  |
| 6522e2dc07f9794d3b6e34bc1c897e75 | Bacteria | p__Chloroflexi | c__KD4-96 | o__KD4-96 | f__KD4-96 | g__KD4-96 |  |
| 02923456 | Bacteria | p__Planctomycetota | c__Phycisphaerae | o__Tepidisphaerales | f__WD2101_soil_group | g__WD2101_soil_group |  |
| 04925177 | Bacteria | p__Proteobacteria | c__Alphaproteobacteria | o__Sphingomonadales | f__Sphingomonadaceae | g__Sphingomonas |  |
| c5c5b91776c09be1b18e82d78e231ce4 | Bacteria | p__Proteobacteria | c__Alphaproteobacteria | o__uncultured | f__uncultured | g__uncultured |  |
| 623a27f25670df44b9a1c0b5e89a4842 | Bacteria | p__Proteobacteria | c__Gammaproteobacteria | o__Burkholderiales | f__Burkholderiaceae | g__Ralstonia |  |
| c032062da59608b5743d97bab1147e01 | Bacteria | p__Proteobacteria | c__Gammaproteobacteria | o__Burkholderiales | f__Comamonadaceae |  |  |
| a6ce7a1377e40947b03989f2de01b750 | Bacteria | p__Bacteroidota | c__Bacteroidia | o__Cytophagales | f__Hymenobacteraceae | g__Pontibacter |  |
| RS-GCF-018146015.1-NZ-JAGSWL010000013.1 | Bacteria | p__Proteobacteria | c__Gammaproteobacteria | o__Burkholderiales | f__Burkholderiaceae | g__Burkholderia-Caballeronia-Paraburkholderia |  |
| G003223075_1 | Bacteria | p__Acidobacteriota | c__Acidobacteriae | o__Solibacterales | f__Solibacteraceae | g__Candidatus_Solibacter |  |
| 04927894 | Bacteria | p__Proteobacteria | c__Alphaproteobacteria | o__Sphingomonadales | f__Sphingomonadaceae | g__Sphingomonas |  |
| f9b3eabddefcfa8610517b22d5457613 | Bacteria | p__Acidobacteriota | c__Vicinamibacteria | o__Vicinamibacterales | f__uncultured | g__uncultured |  |
| 04312021 | Bacteria | p__WPS-2 | c__WPS-2 | o__WPS-2 | f__WPS-2 | g__WPS-2 | s__uncultured_bacterium |
| MJ005-barcode74-umi597bins-ubs-114 | Bacteria | p__Firmicutes | c__Bacilli | o__Lactobacillales | f__Lactobacillaceae | g__Lactobacillus | s__Lactobacillus_iners |
| 3579086399148fe8abd9db55d4bd57a5 | Bacteria | p__Bacteroidota | c__Bacteroidia | o__Bacteroidales | f__Bacteroidaceae | g__Bacteroides |  |
| e2a34ee60f9676882c9640440d9e3a13 | Bacteria | p__Bacteroidota | c__Bacteroidia | o__Bacteroidales | f__Prevotellaceae |  |  |
| 78ac9a4aa8a9a896ad5fb79077e37bd5 | Bacteria | p__Proteobacteria | c__Gammaproteobacteria | o__Burkholderiales | f__Neisseriaceae | g__Snodgrassella |  |
| d0e6a562804ae96b1b8bc92e02c7ff4b | Bacteria | p__Proteobacteria | c__Gammaproteobacteria | o__Burkholderiales | f__SC-I-84 | g__SC-I-84 |  |
| MG857114 | Bacteria | p__Proteobacteria | c__Alphaproteobacteria | o__Rhizobiales | f__Rhizobiaceae | g__Allorhizobium-Neorhizobium-Pararhizobium-Rhizobium |  |
| MJ031-1-barcode42-umi30848bins-ubs-8 | Bacteria | p__Bacteroidota | c__Bacteroidia | o__Chitinophagales | f__Saprospiraceae | g__uncultured | s__uncultured_Devosia |
| JX843738 | Bacteria | p__Proteobacteria | c__Alphaproteobacteria | o__Rhizobiales | f__Hyphomicrobiaceae | g__Hyphomicrobium |  |
| 7ff65b976f0ba6cebac8f67774b5e63e | Bacteria | p__Proteobacteria | c__Gammaproteobacteria | o__Pseudomonadales | f__Moraxellaceae | g__Acinetobacter |  |
| 59764454095b017b5f99afdacf1adb98 | Bacteria |  |  |  |  |  |  |
| 04993606 | Bacteria | p__Proteobacteria | c__Alphaproteobacteria | o__Rhizobiales | f__Xanthobacteraceae | g__Bradyrhizobium |  |
| MJ034-1-barcode25-umi19654bins-ubs-13 | Bacteria | p__Acidobacteriota | c__Acidobacteriae | o__Acidobacteriales | f__uncultured | g__uncultured |  |
| d18a823ff282499aa9b5dbb030875bec | Bacteria | p__Firmicutes | c__Negativicutes | o__Veillonellales-Selenomonadales | f__Veillonellaceae | g__Dialister | s__gut_metagenome |
| 033b67b72cfd60cb3b036dd85f50f5f3 | Bacteria | p__Proteobacteria | c__Gammaproteobacteria | o__Steroidobacterales | f__Steroidobacteraceae | g__Steroidobacter |  |
| 0e1a33bb587bb1a464929d0ce8cce14b | Bacteria | p__Proteobacteria | c__Gammaproteobacteria | o__Burkholderiales | f__Hydrogenophilaceae | g__Hydrogenophilus |  |
| e5cdd117c840ee0feab16fcd97ae2bea | Bacteria |  |  |  |  |  |  |
| 5f51005080a914a965261b96d9e641bc | Bacteria | p__Actinobacteriota | c__Actinobacteria | o__Frankiales | f__Acidothermaceae | g__Acidothermus |  |
| a35d355d9e123abc32ed482966ee4d03 | Bacteria | p__Firmicutes | c__Bacilli | o__Bacillales | f__Bacillaceae | g__Bacillus |  |
| MJ031-1-barcode42-umi19800bins-ubs-21 | Bacteria | p__Bacteroidota | c__Bacteroidia | o__Chitinophagales | f__Chitinophagaceae |  |  |
| 060e64b5c581cbb077b228e27a7df0f2 | Bacteria | p__Bacteroidota | c__Bacteroidia | o__Sphingobacteriales | f__Sphingobacteriaceae |  |  |
| RS-GCF-006539505.1-NZ-BJND01000183.1 | Bacteria | p__Actinobacteriota | c__Actinobacteria | o__Streptomycetales | f__Streptomycetaceae | g__Streptomyces |  |
| 01e352ff664e0a66de42d28d5ece3605 | Bacteria | p__Firmicutes | c__Bacilli | o__Lactobacillales | f__Lactobacillaceae | g__Lactobacillus |  |
| RS-GCF-002009685.1-NZ-KV917367.1--7 | Bacteria | p__Firmicutes | c__Bacilli | o__Exiguobacterales | f__Exiguobacteraceae | g__Exiguobacterium |  |
| 5e077c2dc9a48034b76b07c886440742 | Bacteria | p__Firmicutes | c__Bacilli | o__Staphylococcales | f__Staphylococcaceae | g__Staphylococcus |  |
| AB245363 | Bacteria | p__Proteobacteria | c__Gammaproteobacteria | o__Xanthomonadales | f__Xanthomonadaceae |  |  |
| KX263320 | Bacteria | p__Proteobacteria | c__Alphaproteobacteria | o__Caulobacterales | f__Caulobacteraceae | g__Caulobacter |  |
| fab840623208b20310d0d6740c732845 | Bacteria | p__Firmicutes | c__Clostridia | o__Lachnospirales | f__Lachnospiraceae | g__[Ruminococcus]_torques_group |  |
| 04381808 | Bacteria | p__Chloroflexi | c__Chloroflexia | o__Thermomicrobiales | f__Thermomicrobiaceae | g__Nitrolancea | s__uncultured_bacterium |
| 04957850 | Bacteria | p__Proteobacteria | c__Gammaproteobacteria | o__Burkholderiales | f__Alcaligenaceae | g__Parapusillimonas |  |
| 1560f157992c0fc93773a8971a757041 | Bacteria | p__Proteobacteria | c__Gammaproteobacteria | o__Burkholderiales | f__Comamonadaceae |  |  |
| 8e1dcd6331a92633f463c528657b105a | Bacteria | p__Proteobacteria | c__Gammaproteobacteria | o__Pseudomonadales | f__Moraxellaceae | g__Acinetobacter |  |
| MJ034-1-barcode40-umi28817bins-ubs-3 | Bacteria | p__Bacteroidota | c__Bacteroidia | o__Chitinophagales | f__Chitinophagaceae | g__uncultured | s__uncultured_Ferruginibacter |
| AY785319 | Bacteria | p__Proteobacteria | c__Alphaproteobacteria | o__Rhizobiales | f__Rhizobiaceae |  |  |
| e1f533374fc2f4247c43cc046909d47f | Bacteria | p__Gemmatimonadota | c__Gemmatimonadetes | o__Gemmatimonadales | f__Gemmatimonadaceae |  |  |
| e486cd73079eebc38c8d16820addcb87 | Bacteria | p__Firmicutes | c__Bacilli | o__Staphylococcales | f__Staphylococcaceae | g__Staphylococcus |  |
| HQ888847 | Bacteria | p__Proteobacteria | c__Gammaproteobacteria | o__Enterobacterales | f__Morganellaceae | g__Providencia |  |
| 02404734 | Bacteria | p__Firmicutes | c__Clostridia | o__Peptostreptococcales-Tissierellales | f__Peptostreptococcaceae |  |  |
| 42416b50219ad50f5632e968cc97c684 | Bacteria | p__Proteobacteria | c__Alphaproteobacteria | o__Tistrellales | f__Geminicoccaceae | g__Candidatus_Alysiosphaera | s__uncultured_bacterium |
| 9477df4280daa11e96e8fdb7b5f5011a | Bacteria | p__Proteobacteria | c__Gammaproteobacteria | o__Burkholderiales | f__Oxalobacteraceae |  |  |
| 3dad83318e68e593996da1717398ca23 | Bacteria | p__Proteobacteria | c__Gammaproteobacteria | o__Burkholderiales | f__Neisseriaceae | g__Vitreoscilla | s__uncultured_bacterium |
| f30981677fa49bc782de8d1e6d9e2c99 | Bacteria | p__Bacteroidota | c__Bacteroidia | o__Bacteroidales | f__Muribaculaceae | g__Muribaculaceae |  |
| MJ031-1-barcode30-umi30507bins-ubs-10 | Bacteria | p__Bacteroidota | c__Bacteroidia | o__Chitinophagales | f__Chitinophagaceae | g__Flavisolibacter |  |
| f3f1bb9ee1cfe4998487274b2f65445e | Bacteria | p__Firmicutes | c__Bacilli | o__Erysipelotrichales | f__Erysipelotrichaceae | g__ZOR0006 |  |
| 11838540 | Bacteria | p__Proteobacteria | c__Alphaproteobacteria | o__Sphingomonadales | f__Sphingomonadaceae | g__Sphingomonas |  |
| AF064702 | Bacteria | p__Actinobacteriota | c__Actinobacteria | o__Micrococcales | f__Cellulomonadaceae | g__Cellulomonas |  |
| f2329d4c03bb2a4c10d06c122773fb19 | Bacteria | p__Proteobacteria | c__Alphaproteobacteria | o__Sphingomonadales | f__Sphingomonadaceae | g__Sphingomonas |  |
| MK733277 | Bacteria | p__Proteobacteria | c__Gammaproteobacteria | o__Burkholderiales | f__Comamonadaceae |  |  |
| 8b6b23ea14a5aa75b3e13902a27ae50b | Bacteria | p__Actinobacteriota | c__Actinobacteria | o__Micrococcales | f__Microbacteriaceae |  |  |
| f847aba91ecd124245f53de4ca6034ff | Bacteria | p__Actinobacteriota | c__Actinobacteria | o__Corynebacteriales | f__Mycobacteriaceae | g__Mycobacterium |  |
| 16b4e565c027f8922e7fe094e0ec5d01 | Bacteria | p__Firmicutes | c__Clostridia | o__Lachnospirales | f__Lachnospiraceae |  |  |
| 04900645 | Bacteria | p__Proteobacteria | c__Gammaproteobacteria | o__Pseudomonadales | f__Moraxellaceae | g__Acinetobacter |  |
| MG654667 | Bacteria | p__Proteobacteria | c__Alphaproteobacteria | o__Rhizobiales | f__Rhizobiales_Incertae_Sedis | g__Alsobacter |  |
| fa2eaf3d1573f37ed2bcc6a69a386632 | Bacteria | p__Firmicutes | c__Bacilli | o__Lactobacillales | f__Carnobacteriaceae | g__Trichococcus |  |
| GB-GCA-900066145.1-FMEX01000041.1 | Bacteria | p__Firmicutes | c__Clostridia | o__Lachnospirales | f__Lachnospiraceae | g__Blautia |  |
| ce147fec4119be45043e552d20befc7f | Bacteria | p__Bacteroidota | c__Bacteroidia | o__Sphingobacteriales | f__Sphingobacteriaceae | g__Sphingobacterium |  |
| 04964703 | Bacteria | p__Firmicutes | c__Bacilli | o__Lactobacillales | f__Lactobacillaceae | g__Lactobacillus |  |
| d975f2a7bcc3b485fca3f34fb24ef293 | Bacteria | p__Firmicutes | c__Clostridia | o__Clostridia | f__Gracilibacteraceae |  |  |
| 04996568 | Bacteria | p__Proteobacteria | c__Gammaproteobacteria | o__Xanthomonadales | f__Xanthomonadaceae | g__Stenotrophomonas |  |
| MJ010-2-barcode64-umi100374bins-ubs-5 | Bacteria | p__Bacteroidota | c__Bacteroidia | o__Sphingobacteriales | f__Sphingobacteriaceae | g__Sphingobacterium |  |
| 82b7954b0803150c39ad20fd6039ba76 | Bacteria | p__Firmicutes | c__Bacilli | o__Acholeplasmatales | f__Acholeplasmataceae | g__Anaeroplasma | s__uncultured_bacterium |
| 11482fdcd1679287abcb85eb778411a6 | Bacteria | p__Proteobacteria | c__Gammaproteobacteria | o__Burkholderiales | f__Comamonadaceae |  |  |
| 0d516c8d7e9f31be42351bf628baca9b | Bacteria | p__Proteobacteria | c__Gammaproteobacteria | o__Oceanospirillales | f__Halomonadaceae | g__Halomonas | s__Halomonas_alimentaria |
| 04961095 | Bacteria | p__Actinobacteriota | c__Actinobacteria | o__Corynebacteriales | f__Nocardiaceae | g__Rhodococcus |  |
| 04833417 | Bacteria | p__Proteobacteria | c__Gammaproteobacteria | o__Enterobacterales | f__Enterobacteriaceae |  |  |
| 3119f5d592ff717f93f0228ddc963abc | Bacteria | p__Actinobacteriota | c__Thermoleophilia | o__Gaiellales | f__uncultured | g__uncultured |  |
| MJ034-1-barcode40-umi16110bins-ubs-18 | Bacteria | p__Verrucomicrobiota | c__Verrucomicrobiae | o__Chthoniobacterales | f__Chthoniobacteraceae | g__Chthoniobacter | s__Verrucomicrobia_bacterium |
| 02360397 | Bacteria | p__Proteobacteria | c__Alphaproteobacteria | o__Rhizobiales | f__Rhizobiaceae |  |  |
| G001427865_1 | Bacteria | p__Proteobacteria | c__Alphaproteobacteria | o__Sphingomonadales | f__Sphingomonadaceae |  |  |
| 5c69fcc1389598ed72e76a93243f2315 | Bacteria | p__Proteobacteria | c__Alphaproteobacteria | o__Rhizobiales | f__Devosiaceae | g__Devosia |  |
| RS-GCF-002370925.1-NZ-NQNI01000034.1 | Bacteria | p__Firmicutes | c__Bacilli | o__Lactobacillales | f__Lactobacillaceae | g__Lactobacillus |  |
| MJ030-2-barcode74-umi58760bins-ubs-11 | Bacteria | p__Acidobacteriota | c__Acidobacteriae | o__Acidobacteriales | f__uncultured | g__uncultured |  |
| 04957770 | Bacteria | p__Proteobacteria | c__Gammaproteobacteria | o__Burkholderiales | f__Oxalobacteraceae | g__Massilia |  |
| e3eb3415895d14d1dea47371af130f53 | Bacteria | p__Proteobacteria | c__Gammaproteobacteria | o__Pseudomonadales | f__Moraxellaceae | g__Acinetobacter |  |
| d2e31599678944b298a2276e63208175 | Bacteria | p__Acidobacteriota | c__Vicinamibacteria | o__Subgroup_17 | f__Subgroup_17 | g__Subgroup_17 | s__uncultured_prokaryote |
| 02280919 | Bacteria | p__Cyanobacteria | c__Vampirivibrionia | o__Obscuribacterales | f__Obscuribacteraceae | g__Candidatus_Obscuribacter | s__uncultured_bacterium |
| 6eb48f9cd84e417203c2556aac755b03 | Bacteria | p__Actinobacteriota | c__Actinobacteria | o__Micrococcales | f__Micrococcaceae |  |  |
| 58f973f9386c22c0e105210373df12a7 | Bacteria | p__Firmicutes | c__Bacilli | o__Erysipelotrichales | f__Erysipelotrichaceae | g__Holdemanella | s__uncultured_bacterium |
| b01229d3c22961e64ce4d77e717af2b8 | Bacteria | p__Proteobacteria | c__Gammaproteobacteria | o__Burkholderiales | f__Comamonadaceae | g__Ottowia |  |
| 311ccf163214bc4f5c439f18ec57a882 | Bacteria | p__Firmicutes | c__Clostridia | o__Peptostreptococcales-Tissierellales | f__Sedimentibacteraceae | g__Sedimentibacter | s__uncultured_bacterium |
| 11174932 | Bacteria | p__Bacteroidota | c__Bacteroidia | o__Bacteroidales | f__Rikenellaceae | g__Alistipes |  |
| be751de25785ee236a9f92c9a8956fca | Bacteria | p__Proteobacteria | c__Alphaproteobacteria | o__Rhizobiales | f__Xanthobacteraceae |  |  |
| 05031386 | Bacteria | p__Actinobacteriota | c__Actinobacteria | o__Kineosporiales | f__Kineosporiaceae | g__Quadrisphaera | s__Quadrisphaera_granulorum |
| d18ac710dea2d646e639ea7a33999c0f | Bacteria | p__Proteobacteria | c__Gammaproteobacteria | o__Steroidobacterales | f__Steroidobacteraceae | g__Steroidobacter |  |
| f6f6ace6ac33806b3533d028cf86fbba | Bacteria | p__Actinobacteriota | c__Actinobacteria | o__Micrococcales | f__Microbacteriaceae | g__Microbacterium |  |
| 04997401 | Bacteria | p__Acidobacteriota | c__Acidobacteriae | o__Acidobacteriales | f__Acidobacteriaceae_(Subgroup_1) | g__Terriglobus |  |
| 199f2e4e5c53aa2f6b6e886f56c0f334 | Bacteria | p__Acidobacteriota | c__Vicinamibacteria | o__Vicinamibacterales | f__uncultured | g__uncultured |  |
| MJ020-1-barcode44-umi1857bins-ubs-135 | Bacteria | p__Bacteroidota | c__Bacteroidia | o__Chitinophagales | f__Chitinophagaceae | g__Flavisolibacter |  |
| a2775d90186a20d41a6b39394697d935 | Bacteria | p__Actinobacteriota | c__Actinobacteria | o__Corynebacteriales | f__Corynebacteriaceae | g__Corynebacterium |  |
| G000177075_1 | Bacteria | p__Bacteroidota | c__Bacteroidia | o__Bacteroidales | f__Prevotellaceae | g__Prevotella | s__Prevotella_buccalis |
| 61001cf89de225f49f839a2fc84d0150 | Bacteria | p__Actinobacteriota | c__Actinobacteria | o__Streptosporangiales | f__Nocardiopsaceae |  |  |
| c853c7f2b16a78ce0b6085581487caf1 | Bacteria | p__Actinobacteriota | c__Actinobacteria | o__Corynebacteriales | f__Mycobacteriaceae | g__Mycobacterium |  |
| 04930943 | Bacteria | p__Proteobacteria | c__Gammaproteobacteria | o__CCD24 | f__CCD24 | g__CCD24 |  |
| 021b58e161b37e3dd3f247f92ed80c43 | Bacteria | p__Proteobacteria | c__Gammaproteobacteria | o__Burkholderiales | f__Oxalobacteraceae | g__Noviherbaspirillum |  |
| 13680355 | Bacteria | p__Gemmatimonadota | c__Gemmatimonadetes | o__Gemmatimonadales | f__Gemmatimonadaceae |  |  |
| 2beff10dc5a2e59f663bad0bf47af79e | Bacteria | p__Proteobacteria | c__Gammaproteobacteria | o__Pseudomonadales | f__Moraxellaceae | g__Acinetobacter |  |
| fd73283a9a671cf63f7cc0e888e1b7fd | Bacteria | p__Acidobacteriota | c__Vicinamibacteria | o__Vicinamibacterales | f__uncultured | g__uncultured |  |
| 08939182 | Bacteria | p__Proteobacteria | c__Alphaproteobacteria | o__Rhodospirillales | f__uncultured | g__uncultured |  |
| 19600087 | Bacteria | p__Chloroflexi | c__Anaerolineae | o__Caldilineales | f__Caldilineaceae | g__Caldilineaceae | s__uncultured_bacterium |
| 8cda25be43ca5b99bc72123d69408449 | Bacteria | p__Bacteroidota | c__Bacteroidia | o__Chitinophagales | f__Chitinophagaceae | g__Flavisolibacter |  |
| MJ033-1-barcode36-umi22548bins-ubs-11 | Bacteria | p__Acidobacteriota | c__Subgroup_5 | o__Subgroup_5 | f__Subgroup_5 | g__Subgroup_5 | s__uncultured_bacterium |
| 85f223ef92fe278d6117d0f55f4ce546 | Bacteria | p__Proteobacteria | c__Alphaproteobacteria | o__Micropepsales | f__Micropepsaceae | g__Micropepsis |  |
| 3afc3bf70965a2f73673860d478d681a | Bacteria | p__Proteobacteria | c__Gammaproteobacteria | o__Burkholderiales | f__MWH-UniP1_aquatic_group | g__MWH-UniP1_aquatic_group |  |
| MJ030-2-barcode59-umi85768bins-ubs-5 | Bacteria | p__Proteobacteria | c__Gammaproteobacteria | o__Burkholderiales | f__TRA3-20 | g__TRA3-20 |  |
| c4a881944c35b491338ae0046a301fdb | Bacteria | p__Proteobacteria | c__Gammaproteobacteria | o__Enterobacterales | f__Enterobacteriaceae |  |  |
| RS-GCF-001263945.1-NZ-AWFK01000023.1 | Bacteria | p__Actinobacteriota | c__Actinobacteria | o__Bifidobacteriales | f__Bifidobacteriaceae | g__Bifidobacterium |  |
| RS-GCF-014395975.1-NZ-CP060714.1 | Bacteria | p__Proteobacteria | c__Gammaproteobacteria | o__Burkholderiales | f__Comamonadaceae |  |  |
| 17341874 | Bacteria | p__Firmicutes | c__Bacilli | o__Paenibacillales | f__Paenibacillaceae | g__Paenibacillus | s__Paenibacillus_camelliae |
| 4fc4ed13a855cc1022eddf1ea50978f2 | Bacteria | p__Proteobacteria | c__Alphaproteobacteria | o__Rhodobacterales | f__Rhodobacteraceae | g__Paracoccus |  |
| 04912105 | Bacteria | p__Nitrospirota | c__Nitrospiria | o__Nitrospirales | f__Nitrospiraceae | g__Nitrospira |  |
| MJ010-2-barcode64-umi122176bins-ubs-4 | Bacteria | p__Bacteroidota | c__Bacteroidia | o__Sphingobacteriales | f__Sphingobacteriaceae | g__Pedobacter |  |
| 05037884 | Bacteria | p__Firmicutes | c__Clostridia | o__Oscillospirales | f__Oscillospiraceae | g__Oscillibacter |  |
| facfb85b1edc95c26e7be52835ad4925 | Bacteria | p__Acidobacteriota | c__Acidobacteriae | o__Bryobacterales | f__Bryobacteraceae | g__Bryobacter |  |
| e8eac1634cee2976838cf9493f2e6ab5 | Bacteria | p__Firmicutes | c__Bacilli | o__Lactobacillales | f__Lactobacillaceae | g__Lactobacillus |  |
| 0511e9b32d71eff499a3ed84d1cbd10f | Bacteria | p__Actinobacteriota | c__Actinobacteria | o__Pseudonocardiales | f__Pseudonocardiaceae | g__Pseudonocardia |  |
| 6c2619aae7f9c67790eceaa2a83ac2fc | Bacteria | p__Acidobacteriota | c__Holophagae | o__Subgroup_7 | f__Subgroup_7 | g__Subgroup_7 |  |
| 04924380 | Bacteria | p__Bacteroidota | c__Bacteroidia | o__Bacteroidales | f__Muribaculaceae | g__Muribaculaceae | s__uncultured_Bacteroidales |
| 6cf57557fe5ac4a26a39963f81302665 | Bacteria | p__Actinobacteriota | c__Actinobacteria | o__Frankiales |  |  |  |
| DQ386635 | Bacteria | p__Firmicutes | c__Bacilli | o__Bacillales | f__Bacillaceae |  |  |
| c40ab61aec405fa0531cc356841bdca0 | Bacteria | p__Cyanobacteria | c__Vampirivibrionia | o__Obscuribacterales | f__Obscuribacteraceae | g__Obscuribacteraceae | s__uncultured_bacterium |
| 4f50ae0ef52c3045b376cf4347155912 | Bacteria | p__Firmicutes | c__Bacilli | o__Lactobacillales | f__Enterococcaceae | g__Enterococcus |  |
| 05111150 | Bacteria | p__Proteobacteria | c__Gammaproteobacteria | o__Burkholderiales | f__SC-I-84 | g__SC-I-84 |  |
| 0f7e563401d8d7fc0ead4222dc6e372c | Bacteria | p__Firmicutes | c__Bacilli | o__Bacillales | f__Bacillaceae | g__Geobacillus |  |
| MJ007-2-barcode62-umi15456bins-ubs-22 | Bacteria | p__Firmicutes | c__Bacilli | o__Bacillales | f__Bacillaceae | g__Bacillus |  |
| 04963977 | Bacteria | p__Firmicutes | c__Bacilli | o__Staphylococcales | f__Staphylococcaceae | g__Staphylococcus |  |
| 654f0e8b4c327c292327f6fdb8190872 | Bacteria | p__Actinobacteriota | c__Actinobacteria | o__Micrococcales | f__Microbacteriaceae |  |  |
| 1bbe9ff339a3f73cfb9f94a9fd8f3fce | Bacteria | p__Acidobacteriota | c__Vicinamibacteria | o__Vicinamibacterales | f__Vicinamibacteraceae | g__Vicinamibacteraceae |  |
| 0a51c556492af9b08cd9989489cfb574 | Bacteria | p__Verrucomicrobiota | c__Chlamydiae | o__Chlamydiales | f__cvE6 | g__cvE6 | s__uncultured_bacterium |
| 230fd1cb3e201a2b5cdf55daca7e8af9 | Bacteria | p__Firmicutes | c__Incertae_Sedis | o__DTU014 | f__DTU014 | g__DTU014 | s__metagenome |
| dbd95333aebfeaf66856ebef097ea54d | Bacteria | p__Proteobacteria | c__Alphaproteobacteria | o__Holosporales | f__Holosporaceae | g__uncultured | s__uncultured_bacterium |
| RS-GCF-902385375.1-NZ-CABMEW010000017.1 | Bacteria | p__Firmicutes | c__Clostridia | o__Lachnospirales | f__Lachnospiraceae |  |  |
| G004570525_1 | Bacteria | p__Firmicutes | c__Bacilli | o__Lactobacillales | f__Streptococcaceae | g__Streptococcus | s__Streptococcus_danieliae |
| e151274254d653899fc58ae9f46c2a5b | Bacteria | p__Bacteroidota | c__Bacteroidia | o__Chitinophagales | f__Chitinophagaceae | g__Flaviaesturariibacter | s__uncultured_bacterium |
| 02303591 | Bacteria | p__Actinobacteriota | c__Actinobacteria | o__Pseudonocardiales | f__Pseudonocardiaceae | g__Pseudonocardia |  |
| 07ebc7c8f19feffc3ebbf443cb669918 | Bacteria | p__Firmicutes | c__Bacilli | o__Lactobacillales | f__Streptococcaceae | g__Streptococcus |  |
| 02412098 | Bacteria | p__Proteobacteria | c__Alphaproteobacteria | o__Rhizobiales | f__Beijerinckiaceae | g__Methylobacterium-Methylorubrum |  |
| GB-GCA-900545115.1-UQXN01000021.1 | Bacteria | p__Firmicutes | c__Clostridia | o__Oscillospirales | f__Oscillospiraceae | g__UCG-002 |  |
| 3282898d26a56166e1a935cac90c3915 | Bacteria | p__Firmicutes | c__Clostridia | o__Oscillospirales | f__Ruminococcaceae | g__[Eubacterium]_siraeum_group | s__uncultured_bacterium |
| 41c21b57ee05068103efe123fe001d6d | Bacteria | p__Actinobacteriota | c__Actinobacteria | o__Frankiales | f__Frankiaceae | g__Jatrophihabitans |  |
| 30e17c387ea4b5d85b6310a3b8470cf7 | Bacteria | p__Proteobacteria | c__Gammaproteobacteria | o__Burkholderiales | f__Comamonadaceae | g__Pelomonas |  |
| 17408378 | Bacteria | p__Actinobacteriota | c__Thermoleophilia | o__Gaiellales | f__Gaiellaceae | g__Gaiella |  |
| 2dfd4dc13abc22851b5c338710b1b067 | Bacteria | p__Proteobacteria | c__Gammaproteobacteria | o__Xanthomonadales | f__Rhodanobacteraceae |  |  |
| fa68be5ab6ee300f8ca31709c2ed92a3 | Bacteria | p__Acidobacteriota | c__Vicinamibacteria | o__Vicinamibacterales | f__uncultured | g__uncultured |  |
| RS-GCF-012030615.1-NZ-JAAUWA010000037.1 | Bacteria | p__Actinobacteriota | c__Actinobacteria | o__Corynebacteriales | f__Corynebacteriaceae | g__Corynebacterium |  |
| 11812855 | Bacteria | p__Proteobacteria | c__Alphaproteobacteria | o__Rhodobacterales | f__Rhodobacteraceae |  |  |
| 9495a8d0729334f87ef58956168e5dbf | Bacteria | p__Myxococcota | c__Polyangia | o__Haliangiales | f__Haliangiaceae | g__Haliangium |  |
| 548c22e24977103f0d4ea0416d446a3a | Bacteria | p__Proteobacteria | c__Alphaproteobacteria | o__Rhodobacterales | f__Rhodobacteraceae | g__Rhodobacter |  |
| 317e4ebdd56c392590b961b00c71ee55 | Bacteria | p__Proteobacteria | c__Alphaproteobacteria | o__Rhizobiales | f__A0839 | g__A0839 |  |
| d9b462a15efbfedc4c59ac60843fa590 | Bacteria | p__Proteobacteria | c__Gammaproteobacteria | o__Burkholderiales | f__MWH-UniP1_aquatic_group | g__MWH-UniP1_aquatic_group | s__uncultured_bacterium |
| 88f5f816b7ca405f0b94f7f979a785bf | Bacteria | p__Proteobacteria | c__Gammaproteobacteria | o__Burkholderiales | f__Nitrosomonadaceae | g__Ellin6067 |  |
| KC337335 | Bacteria | p__Proteobacteria | c__Gammaproteobacteria | o__Enterobacterales | f__Pectobacteriaceae |  |  |
| f6471cb246d385dc50e60b500fcf256d | Bacteria | p__Proteobacteria | c__Alphaproteobacteria | o__Sphingomonadales | f__Sphingomonadaceae | g__Sphingomonas |  |
| 417a960eeecaa23f88532e59a62c0c63 | Bacteria | p__Actinobacteriota | c__Actinobacteria | o__Corynebacteriales | f__Mycobacteriaceae | g__Mycobacterium |  |
| 2c3fd505b9de1d498f1f5a4673d4ee42 | Bacteria | p__Bacteroidota | c__Bacteroidia | o__Sphingobacteriales | f__Sphingobacteriaceae | g__Sphingobacterium |  |
| HM212668 | Bacteria | p__Proteobacteria | c__Gammaproteobacteria | o__Pseudomonadales | f__Moraxellaceae | g__Psychrobacter |  |
| 722df7ee8fb6dad72986cdceeba39699 | Bacteria | p__Proteobacteria | c__Gammaproteobacteria | o__Burkholderiales | f__Comamonadaceae | g__Comamonas |  |
| GB-GCA-004173515.1-SECN01000736.1 | Bacteria | p__Proteobacteria | c__Gammaproteobacteria | o__Xanthomonadales | f__Xanthomonadaceae |  |  |
| 10853f5f64301dda74560c72e466818c | Bacteria | p__Proteobacteria | c__Gammaproteobacteria | o__Pseudomonadales | f__Moraxellaceae | g__Acinetobacter |  |
| 04957519 | Bacteria | p__Proteobacteria | c__Gammaproteobacteria | o__Burkholderiales | f__Oxalobacteraceae |  |  |
| 2d17d72c040d8b5ce70c7ad99947b474 | Bacteria | p__Firmicutes | c__Bacilli | o__Bacillales | f__Bacillaceae | g__Bacillus |  |
| 45e9a38556bf76fe34c0d08c9c0a6dfd | Bacteria | p__Proteobacteria | c__Gammaproteobacteria | o__Burkholderiales | f__Comamonadaceae |  |  |
| e329d3007bd05401f1b5108c2f35b17b | Bacteria | p__Actinobacteriota | c__Actinobacteria | o__Propionibacteriales | f__Propionibacteriaceae | g__uncultured |  |
| d3e48550da74a716fa18b82c16aeb2c3 | Bacteria | p__Bacteroidota | c__Bacteroidia | o__Bacteroidales | f__Muribaculaceae | g__Muribaculaceae |  |
| G000828085_1 | Bacteria | p__Proteobacteria | c__Alphaproteobacteria | o__Rhizobiales | f__Rhizobiaceae |  |  |
| 3b95037bcbc4ed0f550945712e3f44f8 | Bacteria | p__Firmicutes | c__Bacilli | o__Lactobacillales | f__Enterococcaceae | g__Enterococcus |  |
| a0e71d20ee4f395e019f72d6311b8d9e | Bacteria | p__Bacteroidota | c__Bacteroidia | o__Chitinophagales | f__Chitinophagaceae | g__Flavitalea |  |
| f156bbb3d9ad3f571abc5f8e1731d953 | Bacteria | p__Proteobacteria | c__Gammaproteobacteria | o__PLTA13 | f__PLTA13 | g__PLTA13 | s__uncultured_sludge |
| 04973249 | Bacteria | p__Firmicutes | c__Bacilli | o__Lactobacillales | f__Leuconostocaceae | g__Weissella |  |
| 1d8dff9788e526fea9149d6698d27c67 | Bacteria | p__Proteobacteria | c__Alphaproteobacteria | o__Rhizobiales | f__Hyphomicrobiaceae | g__Pedomicrobium |  |
| 971e8cded3eae2438dfcc30c475ad7c1 | Bacteria | p__Firmicutes | c__Bacilli | o__Bacillales | f__Bacillaceae | g__Bacillus |  |
| MJ034-1-barcode33-umi21073bins-ubs-9 | Bacteria | p__Proteobacteria | c__Gammaproteobacteria | o__CCD24 | f__CCD24 | g__CCD24 |  |
| eb3d898f940118a6eaab530a6cf9e53c | Bacteria | p__Proteobacteria | c__Alphaproteobacteria | o__Acetobacterales | f__Acetobacteraceae |  |  |
| G900545585_1 | Bacteria | p__Firmicutes | c__Clostridia | o__Oscillospirales | f__Oscillospiraceae | g__UCG-002 |  |
| MH974806 | Bacteria | p__Proteobacteria | c__Alphaproteobacteria | o__Acetobacterales | f__Acetobacteraceae | g__Roseomonas |  |
| d6f9901be539909ccba394a107dd9c39 | Bacteria | p__Actinobacteriota | c__Actinobacteria | o__Micrococcales | f__Micrococcaceae |  |  |
| MJ033-1-barcode34-umi43304bins-ubs-3 | Bacteria | p__Bdellovibrionota | c__Oligoflexia | o__0319-6G20 | f__0319-6G20 | g__0319-6G20 |  |
| 1d5de9a2a47a6a37639fd780613935fe | Bacteria | p__Proteobacteria | c__Alphaproteobacteria | o__uncultured | f__uncultured | g__uncultured |  |
| fed7a298495b562b0cee3a5550bb9433 | Bacteria | p__Verrucomicrobiota | c__Verrucomicrobiae | o__Pedosphaerales | f__Pedosphaeraceae | g__ADurb.Bin063-1 |  |
| 05031107 | Bacteria | p__Actinobacteriota | c__Actinobacteria | o__Micrococcales | f__Microbacteriaceae |  |  |
| fa5a2ca22fc0edde2e6e181ab4a70c6c | Bacteria | p__Proteobacteria | c__Gammaproteobacteria | o__Burkholderiales | f__Rhodocyclaceae |  |  |
| d9b003704df470e251eb18287e58d394 | Bacteria | p__Proteobacteria | c__Gammaproteobacteria | o__Pseudomonadales | f__Pseudomonadaceae | g__Pseudomonas |  |
| 7d6fda02698aa0b70fc17ed1a7a8d531 | Bacteria | p__Firmicutes | c__Clostridia | o__Oscillospirales | f__Ruminococcaceae |  |  |
| d1196703c309cdc6dd09e6745357039c | Bacteria | p__Myxococcota | c__Polyangia | o__Haliangiales | f__Haliangiaceae | g__Haliangium |  |
| 02250049 | Bacteria | p__Proteobacteria | c__Alphaproteobacteria | o__Rhodobacterales | f__Rhodobacteraceae | g__Paracoccus |  |
| 0fd5f194f3bccb88e11dff054e819e33 | Bacteria | p__Proteobacteria | c__Gammaproteobacteria | o__CCD24 | f__CCD24 | g__CCD24 |  |
| 17691963 | Bacteria | p__Firmicutes | c__Clostridia | o__Lachnospirales | f__Lachnospiraceae |  |  |
| 02301637 | Bacteria | p__Firmicutes | c__Clostridia | o__Clostridiales | f__Clostridiaceae | g__Clostridium_sensu_stricto_1 |  |
| fa2cc796fdb50e29d8769293d2bb163f | Bacteria | p__Proteobacteria | c__Gammaproteobacteria | o__Xanthomonadales |  |  |  |
| c12ccc78ae89c28389dcacab15451ef8 | Bacteria | p__Proteobacteria | c__Gammaproteobacteria | o__Enterobacterales | f__Enterobacteriaceae | g__Escherichia-Shigella |  |
| 4f6f18111fc283699d88b01b81919fe9 | Bacteria | p__Firmicutes | c__Bacilli | o__Lactobacillales | f__Enterococcaceae | g__Enterococcus |  |
| 359493bd68c5a36f91e330e1da3b0846 | Bacteria | p__Acidobacteriota | c__Subgroup_5 | o__Subgroup_5 | f__Subgroup_5 | g__Subgroup_5 | s__uncultured_bacterium |
| MT776695 | Bacteria | p__Proteobacteria | c__Gammaproteobacteria | o__Burkholderiales | f__Oxalobacteraceae |  |  |
| 25068e517e4038daa4782ec96da1541b | Bacteria | p__Firmicutes | c__Bacilli | o__Erysipelotrichales | f__Erysipelatoclostridiaceae | g__Erysipelatoclostridium | s__uncultured_Clostridium |
| RS-GCF-004793735.1-NZ-SRYY01000027.1 | Bacteria | p__Bacteroidota | c__Bacteroidia | o__Bacteroidales | f__Muribaculaceae | g__Muribaculaceae |  |
| 06647045 | Bacteria | p__Proteobacteria | c__Gammaproteobacteria | o__Pseudomonadales | f__Moraxellaceae | g__Acinetobacter |  |
| MJ010-1-barcode47-umi44721bins-ubs-15 | Bacteria | p__Proteobacteria | c__Gammaproteobacteria | o__Pseudomonadales | f__Moraxellaceae | g__Acinetobacter |  |
| 413f6f83d534210802dc01c46a254031 | Bacteria | p__Actinobacteriota | c__Actinobacteria | o__Streptomycetales | f__Streptomycetaceae | g__Streptomyces |  |
| EU290155 | Bacteria | p__Proteobacteria | c__Gammaproteobacteria | o__Pseudomonadales | f__Moraxellaceae | g__Acinetobacter | s__Acinetobacter_soli |
| AB266102 | Bacteria | p__Actinobacteriota | c__Coriobacteriia | o__Coriobacteriales | f__Eggerthellaceae | g__Adlercreutzia |  |
| 709cb558d481b43e7e077ffed7fa01e8 | Bacteria | p__Proteobacteria | c__Gammaproteobacteria | o__Xanthomonadales | f__Rhodanobacteraceae | g__Pseudofulvimonas |  |
| X80745 | Bacteria | p__Actinobacteriota | c__Actinobacteria | o__Micrococcales | f__Micrococcaceae | g__Glutamicibacter |  |
| 0018bb7cfc7e9ba8c4ed5ce6302c7e7b | Bacteria | p__Proteobacteria | c__Alphaproteobacteria | o__Rhizobiales | f__Beijerinckiaceae | g__Methylobacterium-Methylorubrum |  |
| 05008659 | Bacteria | p__Deferribacterota | c__Deferribacteres | o__Deferribacterales | f__Deferribacteraceae | g__Mucispirillum | s__Mucispirillum_schaedleri |
| RS-GCF-016899775.1-NZ-JACJJE010000125.1 | Bacteria | p__Firmicutes | c__Clostridia | o__Oscillospirales | f__Oscillospiraceae |  |  |
| 04996472 | Bacteria | p__Proteobacteria | c__Gammaproteobacteria | o__Xanthomonadales | f__Xanthomonadaceae | g__Stenotrophomonas |  |
| GB-GCA-016794585.1-JAEUJM010000047.1 | Bacteria | p__Actinobacteriota | c__Acidimicrobiia | o__Microtrichales | f__uncultured | g__uncultured | s__wastewater_metagenome |
| 04949809 | Bacteria | p__Firmicutes | c__Clostridia | o__Lachnospirales | f__Lachnospiraceae |  |  |
| KF308333 | Bacteria | p__Proteobacteria | c__Gammaproteobacteria | o__Enterobacterales |  |  |  |
| 0bd7b92b33566336c4465044671ad71b | Bacteria | p__Bacteroidota | c__Bacteroidia | o__Flavobacteriales | f__Weeksellaceae | g__Empedobacter |  |
| 6b669ada9c7b885ab84ff8817036f4b1 | Bacteria | p__Proteobacteria | c__Gammaproteobacteria | o__Pseudomonadales | f__Moraxellaceae | g__Acinetobacter |  |
| b021cdb03e2e992f1061a2e006689fea | Bacteria | p__Firmicutes | c__Bacilli | o__Lactobacillales | f__Enterococcaceae | g__Enterococcus |  |
| 9eb8f85c5c059c21fd802eac6e212621 | Bacteria | p__Firmicutes | c__Clostridia | o__Peptostreptococcales-Tissierellales | f__Anaerovoracaceae | g__uncultured | s__anaerobic_digester |
| RS-GCF-016516575.1-NZ-JACVUL010000008.1 | Bacteria | p__Proteobacteria | c__Gammaproteobacteria | o__Enterobacterales |  |  |  |
| a5a71019f58ef38587e29e3ff3cc8929 | Bacteria | p__Deferribacterota | c__Deferribacteres | o__Deferribacterales | f__Deferribacteraceae | g__Mucispirillum | s__Mucispirillum_sp. |
| 0a843613169fa749c3c7e1e3140d128e | Bacteria | p__Bacteroidota | c__Bacteroidia | o__Bacteroidales | f__Dysgonomonadaceae | g__uncultured | s__uncultured_prokaryote |
| b46f9beac808303bbbb05fe13110485d | Bacteria | p__Actinobacteriota | c__Actinobacteria | o__Micrococcales | f__Microbacteriaceae |  |  |
| 02247664 | Bacteria | p__Proteobacteria | c__Alphaproteobacteria | o__Sphingomonadales | f__Sphingomonadaceae |  |  |
| MJ031-1-barcode27-umi38760bins-ubs-3 | Bacteria | p__Proteobacteria | c__Gammaproteobacteria | o__Burkholderiales | f__Comamonadaceae |  |  |
| RS-GCF-009938535.1-NZ-RPBS01000019.1 | Bacteria | p__Proteobacteria | c__Gammaproteobacteria | o__Enterobacterales | f__Enterobacteriaceae |  |  |
| f5eb86fb34aada84f933a0373eeeab70 | Bacteria | p__Proteobacteria | c__Gammaproteobacteria | o__Burkholderiales | f__Comamonadaceae |  |  |
| X97887 | Bacteria | p__Actinobacteriota | c__Actinobacteria | o__Streptosporangiales | f__Nocardiopsaceae | g__Nocardiopsis |  |
| G003933735_1 | Bacteria | p__Proteobacteria | c__Gammaproteobacteria | o__Burkholderiales | f__Comamonadaceae | g__Aquabacterium | s__Aquabacterium_citratiphilum |
| 17338604 | Bacteria | p__Firmicutes | c__Bacilli | o__Paenibacillales | f__Paenibacillaceae | g__Paenibacillus |  |
| 04966028 | Bacteria | p__Firmicutes | c__Bacilli | o__Bacillales | f__Planococcaceae |  |  |
| G900106785_1 | Bacteria | p__Bacteroidota | c__Bacteroidia | o__Bacteroidales | f__Prevotellaceae | g__Alloprevotella | s__Prevotellaceae_bacterium |
| 3920bcc7703ee90b8488aaba638962ba | Bacteria | p__Bacteroidota | c__Bacteroidia | o__Bacteroidales | f__Muribaculaceae | g__Muribaculaceae |  |
| 846ce5fd5f472010f40e8a80386782c2 | Bacteria | p__Firmicutes | c__Limnochordia | o__MBA03 | f__MBA03 | g__MBA03 |  |
| b944b776049f16d9846fb3c9c3e42eb9 | Bacteria | p__Proteobacteria | c__Gammaproteobacteria | o__Xanthomonadales | f__Rhodanobacteraceae | g__uncultured |  |
| KM873051 | Bacteria | p__Proteobacteria | c__Gammaproteobacteria | o__Burkholderiales | f__Oxalobacteraceae | g__Massilia |  |
| 95f3f6b276625ab134c042bc290c6f8f | Bacteria | p__Proteobacteria | c__Alphaproteobacteria | o__Rhodobacterales | f__Rhodobacteraceae | g__Rhodobacter |  |
| a17e77361aeb4e9984234ee20a0cb8d7 | Bacteria | p__Methylomirabilota | c__Methylomirabilia | o__Rokubacteriales | f__Rokubacteriales | g__Rokubacteriales | s__uncultured_bacterium |
| 04989027 | Bacteria | p__Proteobacteria | c__Gammaproteobacteria | o__Pseudomonadales | f__Pseudomonadaceae | g__Pseudomonas |  |
| 9757a5b95d5a264c6c74ae80ceb21e5e | Bacteria | p__Chloroflexi | c__Anaerolineae | o__SJA-15 | f__SJA-15 | g__SJA-15 |  |
| 08094393 | Bacteria | p__Proteobacteria | c__Alphaproteobacteria | o__Dongiales | f__Dongiaceae | g__Dongia | s__bacterium_Ellin314 |
| 9b40a4561109183102619ff5bb4031b5 | Bacteria | p__Actinobacteriota | c__Actinobacteria | o__Micrococcales | f__Micrococcaceae |  |  |
| fb654fb92b0131b69bb323856410220d | Bacteria | p__Proteobacteria | c__Gammaproteobacteria | o__Pseudomonadales | f__Pseudomonadaceae | g__Pseudomonas |  |
| DQ845457 | Bacteria | p__Actinobacteriota | c__Actinobacteria | o__Micrococcales | f__Microbacteriaceae |  |  |
| 04957345 | Bacteria | p__Proteobacteria | c__Gammaproteobacteria | o__Burkholderiales | f__Comamonadaceae | g__Acidovorax |  |
| 04958621 | Bacteria | p__Actinobacteriota | c__Actinobacteria | o__Kineosporiales | f__Kineosporiaceae | g__Kineococcus |  |
| f26bfb51357d1f4273e63cf48a50e3f9 | Bacteria | p__Bacteroidota | c__Bacteroidia | o__Cytophagales | f__Spirosomaceae | g__Leadbetterella | s__Leadbetterella_sp. |
| 976182432b6de4a80d5c8e27b0c64e29 | Bacteria | p__Actinobacteriota | c__Rubrobacteria | o__Rubrobacterales | f__Rubrobacteriaceae | g__Rubrobacter | s__metagenome |
| MJ030-2-barcode59-umi94512bins-ubs-4 | Bacteria | p__Bacteroidota | c__Bacteroidia | o__Chitinophagales | f__Chitinophagaceae | g__Flavitalea | s__uncultured_bacterium |
| 71db6f9ed3bec875f06c9e4c065c3362 | Bacteria | p__Proteobacteria | c__Gammaproteobacteria | o__Burkholderiales | f__Burkholderiaceae | g__Ralstonia |  |
| 02403947 | Bacteria | p__Firmicutes | c__Clostridia | o__Lachnospirales | f__Lachnospiraceae | g__Dorea |  |
| 04950669 | Bacteria | p__Firmicutes | c__Clostridia | o__Lachnospirales | f__Lachnospiraceae | g__Acetatifactor |  |
| 04996395 | Bacteria | p__Proteobacteria | c__Gammaproteobacteria | o__Xanthomonadales | f__Xanthomonadaceae | g__Stenotrophomonas |  |
| RS-GCF-008824165.1-NZ-CP044409.1--6 | Bacteria | p__Proteobacteria | c__Gammaproteobacteria | o__Pseudomonadales | f__Pseudomonadaceae | g__Pseudomonas |  |
| b06550890b39040fdb4e7e52ed3d87ac | Bacteria | p__Bacteroidota | c__Bacteroidia | o__Flavobacteriales | f__Flavobacteriaceae | g__Flavobacterium |  |
| 2235d8e97b923ffe4aca433cec3b4d9a | Bacteria | p__Gemmatimonadota | c__Gemmatimonadetes | o__Gemmatimonadales | f__Gemmatimonadaceae |  |  |
| cbca9427342ef26a40c164d97cd5fc52 | Bacteria | p__Proteobacteria | c__Gammaproteobacteria | o__Pseudomonadales | f__Moraxellaceae | g__Acinetobacter |  |
| 74ec713197de94575ca67bb0cf4ecc5a | Bacteria | p__Firmicutes | c__Bacilli | o__Staphylococcales | f__Staphylococcaceae | g__Jeotgalicoccus |  |
| b91f825a5c4f18ae3b426c9ffd7a08d5 | Bacteria | p__Firmicutes | c__Bacilli | o__Staphylococcales | f__Gemellaceae | g__Gemella |  |
| 04878502 | Bacteria | p__Firmicutes | c__Bacilli | o__Staphylococcales | f__Staphylococcaceae | g__Staphylococcus |  |
| 14801717 | Bacteria | p__Chloroflexi | c__Ktedonobacteria | o__Ktedonobacterales | f__JG30-KF-AS9 | g__JG30-KF-AS9 | s__uncultured_bacterium |
| 826574f64c2069ab14c1169d89fa4dc7 | Bacteria | p__Bacteroidota | c__Bacteroidia | o__Chitinophagales | f__Chitinophagaceae | g__Segetibacter | s__Segetibacter_aerophilus |
| 19e4d00d6ce011b33d40278e041635c6 | Bacteria | p__Proteobacteria | c__Gammaproteobacteria | o__Steroidobacterales | f__Steroidobacteraceae | g__uncultured |  |
| 03318232 | Bacteria | p__Planctomycetota | c__Planctomycetes | o__Gemmatales | f__Gemmataceae | g__Zavarzinella | s__uncultured_Zavarzinella |
| 9761e788ba2cf7ef4c93495a5c9939cb | Bacteria | p__Actinobacteriota | c__Actinobacteria |  |  |  |  |
| cf3e0dc23f9c9d46e1e316c0e166e903 | Bacteria | p__Firmicutes | c__Clostridia | o__Oscillospirales | f__Oscillospiraceae | g__UCG-005 | s__uncultured_Clostridiales |
| MJ006-2-barcode52-umi145850bins-ubs-3 | Bacteria | p__Bacteroidota | c__Bacteroidia | o__Bacteroidales | f__Barnesiellaceae | g__Barnesiella |  |
| AJ314919 | Bacteria | p__Actinobacteriota | c__Actinobacteria | o__Corynebacteriales | f__Corynebacteriaceae | g__Corynebacterium | s__Corynebacterium_appendicis |
| 04901171 | Bacteria | p__Proteobacteria | c__Gammaproteobacteria | o__Steroidobacterales | f__Steroidobacteraceae |  |  |
| RS-GCF-000719855.1-NZ-JOIB01000087.1 | Bacteria | p__Actinobacteriota | c__Actinobacteria | o__Streptomycetales | f__Streptomycetaceae | g__Streptomyces |  |
| 27bee41a27b4726fb87b6f83d1d4a82e | Bacteria | p__Acidobacteriota | c__Vicinamibacteria | o__Vicinamibacterales | f__uncultured | g__uncultured |  |
| b4f68eb500730f073f9935c8133e7f86 | Bacteria | p__Proteobacteria | c__Gammaproteobacteria | o__Enterobacterales | f__Enterobacteriaceae | g__Klebsiella |  |
| 0cce2b469c6021c67816cb62abdd4082 | Bacteria | p__Bacteroidota | c__Bacteroidia | o__Chitinophagales | f__Chitinophagaceae | g__Flavisolibacter |  |
| 04965096 | Bacteria | p__Firmicutes | c__Clostridia | o__Oscillospirales | f__Ruminococcaceae |  |  |
| ef4fd6e66478507028f09f59ff6411f9 | Bacteria | p__Firmicutes | c__Bacilli | o__Erysipelotrichales | f__Erysipelotrichaceae | g__Dubosiella | s__uncultured_bacterium |
| 227de52a394e7e6c95a87b0338919af9 | Bacteria | p__Bacteroidota | c__Bacteroidia | o__Sphingobacteriales | f__Sphingobacteriaceae |  |  |
| RS-GCF-000694115.1-NZ-KK737323.1--5 | Bacteria | p__Proteobacteria | c__Gammaproteobacteria | o__Enterobacterales | f__Enterobacteriaceae |  |  |
| 4a4ab061fb64c46fd82df868f99ef28f | Bacteria | p__Proteobacteria | c__Alphaproteobacteria | o__Acetobacterales | f__Acetobacteraceae |  |  |
| 43999759269d95fb4aa5e6f5483d6305 | Bacteria | p__Actinobacteriota | c__Thermoleophilia | o__Solirubrobacterales | f__67-14 | g__67-14 |  |
| 79d63c635d875dc8a624e3dafd42b78f | Bacteria | p__Proteobacteria | c__Gammaproteobacteria | o__Burkholderiales | f__Nitrosomonadaceae | g__MND1 |  |
| 04908571 | Bacteria | p__Proteobacteria | c__Alphaproteobacteria | o__Rhizobiales | f__Beijerinckiaceae | g__1174-901-12 | s__uncultured_bacterium |
| RS-GCF-003043175.1-NZ-PZES01000204.1 | Bacteria | p__Firmicutes | c__Bacilli | o__Staphylococcales | f__Staphylococcaceae | g__Staphylococcus |  |
| 05462738 | Bacteria | p__Proteobacteria | c__Gammaproteobacteria | o__Pseudomonadales | f__Moraxellaceae | g__Acinetobacter |  |
| 05030506 | Bacteria | p__Proteobacteria | c__Gammaproteobacteria | o__Burkholderiales | f__Oxalobacteraceae | g__Herbaspirillum |  |
| 11147089 | Bacteria | p__Bacteroidota | c__Bacteroidia | o__Bacteroidales | f__Muribaculaceae | g__Muribaculaceae | s__unidentified |
| 05370626 | Bacteria | p__Proteobacteria | c__Gammaproteobacteria | o__Enterobacterales | f__Morganellaceae | g__Providencia |  |
| b9b0083d05ff745ed0ec326c21d56fa0 | Bacteria | p__Myxococcota | c__Myxococcia | o__Myxococcales | f__Myxococcaceae | g__Melittangium | s__Melittangium_lichenicola |
| 04666034 | Bacteria | p__Firmicutes | c__Bacilli | o__Staphylococcales | f__Gemellaceae | g__Gemella |  |
| 4c90367289b6b981378a794d6220b1fe | Bacteria | p__Proteobacteria | c__Alphaproteobacteria | o__Rhodobacterales | f__Rhodobacteraceae |  |  |
| AB370249 | Bacteria | p__Firmicutes | c__Negativicutes | o__Veillonellales-Selenomonadales | f__Veillonellaceae | g__Dialister | s__Dialister_succinatiphilus |
| 02244708 | Bacteria | p__Actinobacteriota | c__Actinobacteria | o__Corynebacteriales | f__Corynebacteriaceae | g__Corynebacterium |  |
| 649ed8fbd9051b3e63a93b92574f4aab | Bacteria | p__Firmicutes | c__Bacilli | o__Lactobacillales | f__Enterococcaceae | g__Enterococcus |  |
| 18249855 | Bacteria | p__Proteobacteria | c__Gammaproteobacteria | o__Burkholderiales | f__Burkholderiaceae | g__Ralstonia |  |
| 50bed2e9394707c7acc5bbecaba8a643 | Bacteria | p__Bacteroidota | c__Bacteroidia | o__Bacteroidales | f__Bacteroidaceae | g__Bacteroides | s__Bacteroides_plebeius |
| 5548d50e2fec43e14048a59cc1b1d68c | Bacteria | p__Actinobacteriota | c__Acidimicrobiia | o__Microtrichales |  |  |  |
| bfe5d69d030294285776f1228377d686 | Bacteria | p__Proteobacteria | c__Gammaproteobacteria | o__Burkholderiales | f__Comamonadaceae | g__Acidovorax | s__uncultured_Xylophilus |
| b25159e5a786baa75d2147c093ba9f1c | Bacteria | p__Firmicutes | c__Clostridia | o__Clostridia_vadinBB60_group | f__Clostridia_vadinBB60_group | g__Clostridia_vadinBB60_group | s__unidentified |
| d1308e0d6fdbfa6ab7f067d2f218b7d8 | Bacteria | p__Acidobacteriota | c__Subgroup_5 | o__Subgroup_5 | f__Subgroup_5 | g__Subgroup_5 | s__uncultured_bacterium |
| MJ030-2-barcode58-umi38204bins-ubs-17 | Bacteria | p__Proteobacteria | c__Gammaproteobacteria | o__Burkholderiales | f__Comamonadaceae |  |  |
| a2642bca0a2933e9163e50d50d483e01 | Bacteria | p__Gemmatimonadota | c__Gemmatimonadetes | o__Gemmatimonadales | f__Gemmatimonadaceae | g__uncultured |  |
| a4a2d903c4dc4db981cc14e37262dbd5 | Bacteria | p__Firmicutes | c__Bacilli | o__Erysipelotrichales | f__Erysipelatoclostridiaceae | g__Erysipelatoclostridium | s__uncultured_Clostridium |
| 17663721 | Bacteria | p__Actinobacteriota | c__Coriobacteriia | o__Coriobacteriales | f__Eggerthellaceae | g__Enterorhabdus | s__uncultured_bacterium |
| X97693 | Bacteria | p__Proteobacteria | c__Alphaproteobacteria | o__Rhizobiales | f__Hyphomicrobiaceae | g__Pedomicrobium |  |
| GB-GCA-910575495.1-CAJTFS010000013.1 | Bacteria | p__Bacteroidota | c__Bacteroidia | o__Bacteroidales | f__Rikenellaceae | g__Alistipes |  |
| EF424395 | Bacteria | p__Bacteroidota | c__Bacteroidia | o__Flavobacteriales | f__Flavobacteriaceae | g__Flavobacterium | s__Flavobacterium_lindanitolerans |
| 24bc46b3b6f72d4e749ec325bdccf7e7 | Bacteria | p__Bacteroidota | c__Bacteroidia | o__Sphingobacteriales | f__Sphingobacteriaceae |  |  |
| 04971300 | Bacteria | p__Firmicutes | c__Clostridia | o__Peptostreptococcales-Tissierellales | f__Peptostreptococcales-Tissierellales | g__Finegoldia |  |
| 04995398 | Bacteria | p__Proteobacteria | c__Alphaproteobacteria | o__Rhizobiales | f__Rhizobiaceae | g__Ochrobactrum |  |
| 9a165b3155503946b62d84ee9cd64417 | Bacteria | p__Proteobacteria | c__Gammaproteobacteria | o__Burkholderiales | f__Oxalobacteraceae |  |  |
| RS-GCF-005844755.1-NZ-SPHB01000048.1 | Bacteria | p__Bacteroidota | c__Bacteroidia | o__Bacteroidales | f__Bacteroidaceae | g__Bacteroides |  |
| 9061992705e62db81fffee451ca906d2 | Bacteria | p__Proteobacteria | c__Gammaproteobacteria | o__Pseudomonadales | f__Moraxellaceae | g__Acinetobacter |  |
| 1e61c8eb5c0c64e333a1af163a047153 | Bacteria | p__Gemmatimonadota | c__Gemmatimonadetes | o__Gemmatimonadales | f__Gemmatimonadaceae | g__Gemmatimonas |  |
| dff57539590a9d228443e731ed1be80c | Bacteria | p__Actinobacteriota | c__Acidimicrobiia | o__Microtrichales | f__uncultured | g__uncultured | s__wastewater_metagenome |
| 11136044 | Bacteria | p__Bacteroidota | c__Bacteroidia | o__Bacteroidales | f__Muribaculaceae | g__Muribaculaceae |  |
| 4167f9d838bbdaad757d601ceb4bd61f | Bacteria | p__Verrucomicrobiota | c__Verrucomicrobiae | o__Pedosphaerales | f__Pedosphaeraceae | g__Pedosphaeraceae |  |
| 04933711 | Bacteria | p__Bacteroidota | c__Bacteroidia | o__Chitinophagales | f__Chitinophagaceae | g__Flavitalea |  |
| 04934679 | Bacteria | p__Bacteroidota | c__Bacteroidia | o__Flavobacteriales | f__Weeksellaceae | g__Chryseobacterium |  |
| 720a9a842a7cc786b048247063ca422d | Bacteria | p__Firmicutes | c__Bacilli | o__Lactobacillales | f__Leuconostocaceae | g__Weissella |  |
| 14800933 | Bacteria | p__Chloroflexi | c__Ktedonobacteria | o__Ktedonobacterales | f__JG30-KF-AS9 | g__JG30-KF-AS9 | s__uncultured_bacterium |
| RS-GCF-002355155.1-NZ-AP017369.1--6 | Bacteria | p__Actinobacteriota | c__Actinobacteria | o__Corynebacteriales | f__Corynebacteriaceae | g__Corynebacterium |  |
| 02387869 | Bacteria | p__Proteobacteria | c__Alphaproteobacteria | o__Reyranellales | f__Reyranellaceae | g__Reyranella |  |
| 7cef7a12924a1bd9c70ecfd808c23435 | Bacteria | p__Actinobacteriota | c__Actinobacteria | o__Micrococcales | f__Micrococcaceae |  |  |
| 1e62d103ff5a3fb6f4cd4130d031e19c | Bacteria | p__Actinobacteriota | c__Actinobacteria | o__Frankiales |  |  |  |
| 98345fb3aaec955aeabd786b95295ea7 | Bacteria | p__Bacteroidota | c__Bacteroidia | o__Bacteroidales | f__Bacteroidaceae | g__Bacteroides |  |
| 7a959a10c5a737699d615e162d7458c7 | Bacteria | p__Planctomycetota | c__Phycisphaerae | o__Tepidisphaerales | f__WD2101_soil_group | g__WD2101_soil_group |  |
| e4efd067e38d69a94d67604fae870241 | Bacteria | p__Proteobacteria | c__Alphaproteobacteria | o__Sphingomonadales | f__Sphingomonadaceae |  |  |
| 416a36defe09bed991d5ab3cbb91720b | Bacteria | p__Proteobacteria | c__Gammaproteobacteria | o__Burkholderiales | f__Hydrogenophilaceae | g__Thiobacillus |  |
| 1bb38ceb768815172c0f91781d3ad3be | Bacteria | p__Proteobacteria | c__Alphaproteobacteria | o__Rhizobiales | f__Beijerinckiaceae |  |  |
| 5e32202cf9fc14c831b01ec2ff4db188 | Bacteria | p__Chloroflexi | c__Anaerolineae | o__Caldilineales | f__Caldilineaceae | g__uncultured |  |
| RS-GCF-004804275.1-NZ-STGI01000028.1 | Bacteria | p__Proteobacteria | c__Gammaproteobacteria | o__Burkholderiales | f__Oxalobacteraceae |  |  |
| 03779eebe0b4f7e82381c2cbed529065 | Bacteria | p__Firmicutes | c__Bacilli | o__Lactobacillales | f__Enterococcaceae | g__Enterococcus |  |
| 2da398dd66d72208fac9a9f100b5dce4 | Bacteria | p__Myxococcota | c__Myxococcia | o__Myxococcales | f__Myxococcaceae |  |  |
| c1a13e34dcf78d9d8ccbe711d54aad79 | Bacteria | p__Actinobacteriota | c__Acidimicrobiia | o__Microtrichales | f__Microtrichaceae | g__IMCC26207 |  |
| 6063ce3192ff28ecad783e02a23d5fc0 | Bacteria | p__Bacteroidota | c__Bacteroidia | o__Bacteroidales | f__Prevotellaceae | g__Prevotella |  |
| MJ030-2-barcode65-umi43951bins-ubs-16 | Bacteria | p__Acidobacteriota | c__Acidobacteriae | o__Solibacterales | f__Solibacteraceae | g__Candidatus_Solibacter |  |
| 05945059 | Bacteria | p__Acidobacteriota | c__Vicinamibacteria | o__Vicinamibacterales | f__Vicinamibacteraceae | g__Vicinamibacteraceae |  |
| 44dbe2a91cad0dcf2cec471b5f10c960 | Bacteria | p__Bacteroidota | c__Bacteroidia | o__Chitinophagales | f__Chitinophagaceae | g__Terrimonas |  |
| MJ006-2-barcode66-umi158413bins-ubs-3 | Bacteria | p__Firmicutes | c__Clostridia | o__Oscillospirales | f__Ruminococcaceae | g__Faecalibacterium |  |
| 02347454 | Bacteria | p__Proteobacteria | c__Alphaproteobacteria | o__Sphingomonadales | f__Sphingomonadaceae | g__Sphingobium |  |
| 93068332ddc966f8b9013b2fd1763f05 | Bacteria | p__Proteobacteria | c__Alphaproteobacteria | o__Rhizobiales | f__Xanthobacteraceae | g__uncultured |  |
| LABY01000145 | Bacteria | p__Proteobacteria | c__Alphaproteobacteria | o__Rhizobiales | f__Beijerinckiaceae | g__Methylobacterium-Methylorubrum | s__Methylobacterium_variabile |
| 7af49b0f165c803f878ba4d13a01801e | Bacteria | p__Firmicutes | c__Clostridia | o__Clostridiales | f__Clostridiaceae | g__Clostridium_sensu_stricto_1 |  |
| 03662970 | Bacteria | p__Proteobacteria | c__Alphaproteobacteria | o__Rhodospirillales | f__uncultured | g__uncultured |  |
| 04943965 | Bacteria | p__Actinobacteriota | c__Rubrobacteria | o__Rubrobacterales | f__Rubrobacteriaceae | g__Rubrobacter | s__metagenome |
| d78c23478aa3388767d108856b269025 | Bacteria | p__Proteobacteria | c__Alphaproteobacteria | o__Acetobacterales | f__Acetobacteraceae | g__uncultured |  |
| 0b0fe26d7131c7dc90df7343fc87431b | Bacteria | p__Firmicutes | c__Bacilli | o__Bacillales |  |  |  |
| 76ded889ab553ebef87f21c74284ef68 | Bacteria | p__Proteobacteria | c__Gammaproteobacteria | o__Pseudomonadales | f__Pseudomonadaceae | g__Pseudomonas |  |
| 2323b0c8c739942ec546ca9981a0e129 | Bacteria | p__Bacteroidota | c__Bacteroidia | o__Flavobacteriales | f__Flavobacteriaceae | g__Flavobacterium |  |
| 313325f7edda5a9385164be0b1003d65 | Bacteria | p__Entotheonellaeota | c__Entotheonellia | o__Entotheonellales | f__Entotheonellaceae | g__Entotheonellaceae |  |
| 05977659 | Bacteria | p__Acidobacteriota | c__Vicinamibacteria | o__Vicinamibacterales | f__Vicinamibacteraceae | g__Vicinamibacteraceae |  |
| 04934686 | Bacteria | p__Bacteroidota | c__Bacteroidia | o__Flavobacteriales | f__Weeksellaceae | g__Chryseobacterium |  |
| RS-GCF-900500875.1-NZ-UISC01000078.1 | Bacteria | p__Proteobacteria | c__Gammaproteobacteria | o__Enterobacterales | f__Enterobacteriaceae |  |  |
| ab6923fbb47e17a8060cb08cad256584 | Bacteria | p__Actinobacteriota | c__Acidimicrobiia | o__IMCC26256 | f__IMCC26256 | g__IMCC26256 |  |
| 99b6b660d65b14a487fe713503dd321b | Bacteria | p__Cyanobacteria | c__Cyanobacteriia | o__Oxyphotobacteria_Incertae_Sedis | f__Unknown_Family | g__Leptolyngbya_EcFYyyy-00 | s__uncultured_bacterium |
| MJ010-1-barcode25-umi117205bins-ubs-6 | Bacteria | p__Firmicutes | c__Clostridia | o__Peptostreptococcales-Tissierellales | f__Fusibacteraceae | g__Fusibacter |  |
| f0b41d24b3e99031d145abb6a61da708 | Bacteria | p__Bacteroidota | c__Bacteroidia | o__Bacteroidales | f__Prevotellaceae | g__Prevotella | s__Prevotella_melaninogenica |
| 04972336 | Bacteria | p__Firmicutes | c__Bacilli | o__Lactobacillales | f__Leuconostocaceae | g__Leuconostoc |  |
| c046f66e27ba9cacc41bbf25c0419eeb | Bacteria | p__Chloroflexi | c__Anaerolineae | o__SBR1031 | f__SBR1031 | g__SBR1031 |  |
| MJ034-1-barcode33-umi14953bins-ubs-22 | Bacteria | p__Bacteroidota | c__Bacteroidia | o__Chitinophagales | f__Saprospiraceae | g__uncultured | s__uncultured_soil |
| acaa517a417a762b467ec3aec798606c | Bacteria | p__Proteobacteria | c__Alphaproteobacteria | o__Azospirillales | f__Azospirillaceae | g__Skermanella |  |
| d38b876af7630416f937a8a73234899c | Bacteria | p__Proteobacteria | c__Gammaproteobacteria | o__Burkholderiales |  |  |  |
| dfa00d5c86fca18c1a95326e611a1ce5 | Bacteria | p__Proteobacteria | c__Gammaproteobacteria | o__Oceanospirillales | f__Hahellaceae | g__Hahella | s__uncultured_bacterium |
| 05132362 | Bacteria | p__Firmicutes | c__Bacilli | o__Bacillales | f__Planococcaceae | g__Lysinibacillus |  |
| G001945535_1 | Bacteria | p__Firmicutes | c__Bacilli | o__Erysipelotrichales | f__Erysipelotrichaceae | g__Faecalibaculum | s__uncultured_bacterium |
| 04946005 | Bacteria | p__Actinobacteriota | c__Actinobacteria | o__Streptomycetales | f__Streptomycetaceae | g__Streptomyces |  |
| 4a631869a409fcce331eabd65bf2ddb7 | Bacteria | p__Proteobacteria | c__Gammaproteobacteria | o__Burkholderiales | f__Comamonadaceae | g__Aquabacterium |  |
| GB-GCA-003585715.1-NQOQ01000004.1 | Bacteria | p__Actinobacteriota | c__Actinobacteria | o__Bifidobacteriales | f__Bifidobacteriaceae | g__Gardnerella | s__Gardnerella_vaginalis |
| 4504d2acb8a7a4d771c2dbd6e917bf92 | Bacteria | p__Proteobacteria | c__Gammaproteobacteria | o__Burkholderiales | f__Burkholderiaceae | g__Burkholderia-Caballeronia-Paraburkholderia |  |
| 412e944a8d70e8ee07ed8990636f5ea7 | Bacteria | p__Bacteroidota | c__Bacteroidia | o__Cytophagales | f__Hymenobacteraceae | g__Adhaeribacter | s__metagenome |
| 02266608 | Bacteria | p__Actinobacteriota | c__Actinobacteria | o__Propionibacteriales | f__Nocardioidaceae |  |  |
| 693d3be5e949d094c90cc27dbeb72808 | Bacteria | p__Actinobacteriota | c__Actinobacteria | o__Micrococcales | f__Micrococcaceae |  |  |
| 3123f94213f6f2d3eb58757068dd5590 | Bacteria | p__Actinobacteriota | c__Actinobacteria | o__Micrococcales | f__Microbacteriaceae | g__Leucobacter |  |
| 682c78c1138c533a0f267464faf2584a | Bacteria | p__Proteobacteria | c__Alphaproteobacteria | o__Elsterales | f__uncultured | g__uncultured |  |
| 02250050 | Bacteria | p__Proteobacteria | c__Alphaproteobacteria | o__Rhodobacterales | f__Rhodobacteraceae | g__Paracoccus |  |
| 93e987b5c09360b9acada8bea7c9771c | Bacteria | p__Chloroflexi | c__Anaerolineae | o__Caldilineales | f__Caldilineaceae | g__uncultured |  |
| 6aeb510459d7dc76e010e40738b85fe8 | Bacteria | p__Proteobacteria | c__Gammaproteobacteria | o__Nitrosococcales | f__Nitrosococcaceae | g__CI75cm.2.12 | s__uncultured_bacterium |
| 4fb3cd73233862d64af642821d18c05d | Bacteria | p__Desulfobacterota | c__uncultured | o__uncultured | f__uncultured | g__uncultured |  |
| GB-GCA-018052775.1-JAGPMS010000005.1 | Bacteria | p__Firmicutes | c__Clostridia | o__Oscillospirales | f__[Eubacterium]_coprostanoligenes_group | g__[Eubacterium]_coprostanoligenes_group | s__gut_metagenome |
| ed972504e9f9a916fad16af439eaaabf | Bacteria | p__Actinobacteriota | c__Actinobacteria | o__Micrococcales | f__Microbacteriaceae | g__Microbacterium |  |
| bac5ea80e66b0b3a3351e98a336a54f3 | Bacteria | p__Bacteroidota | c__Bacteroidia | o__Chitinophagales | f__Chitinophagaceae |  |  |
| 15b4894930a5bd1687b24c8ba1c6f904 | Bacteria | p__Firmicutes | c__Bacilli | o__Erysipelotrichales | f__Erysipelotrichaceae | g__Ileibacterium | s__Ileibacterium_valens |
| 5773432bfb110c776e68e6e28dee99ab | Bacteria | p__Proteobacteria | c__Gammaproteobacteria | o__Pseudomonadales | f__Pseudomonadaceae | g__Pseudomonas |  |
| ae3a8a3c1e5b011e02ab7209af957518 | Bacteria | p__Bacteroidota | c__Bacteroidia | o__Chitinophagales | f__Chitinophagaceae | g__Taibaiella |  |
| 63f26916fcd841813a9117a99ca33f68 | Bacteria | p__Acidobacteriota | c__Vicinamibacteria | o__Vicinamibacterales | f__uncultured | g__uncultured |  |
| AY660685 | Bacteria | p__Proteobacteria | c__Gammaproteobacteria | o__Pseudomonadales | f__Moraxellaceae | g__Psychrobacter |  |
| MJ033-1-barcode45-umi11755bins-ubs-24 | Bacteria | p__Proteobacteria | c__Gammaproteobacteria | o__Gammaproteobacteria_Incertae_Sedis | f__Unknown_Family | g__Candidatus_Ovatusbacter | s__uncultured_proteobacterium |
| cba6d30a446e5aab321af0baf022ed0d | Bacteria | p__Actinobacteriota | c__Actinobacteria | o__Corynebacteriales | f__Dietziaceae | g__Dietzia |  |
| c970db9b393adae2eb9428f710162b0f | Bacteria | p__Proteobacteria | c__Alphaproteobacteria | o__Rhodobacterales | f__Rhodobacteraceae | g__Rhodobacter |  |
| a10ee2e134833677d8763c0093b5460f | Bacteria | p__Actinobacteriota | c__Actinobacteria |  |  |  |  |
| 04997369 | Bacteria | p__Acidobacteriota | c__Acidobacteriae | o__Acidobacteriales |  |  |  |
| 02339583 | Bacteria | p__Proteobacteria | c__Alphaproteobacteria | o__Rhizobiales | f__Rhizobiaceae | g__Allorhizobium-Neorhizobium-Pararhizobium-Rhizobium |  |
| G000434815_1 | Bacteria | p__Proteobacteria | c__Gammaproteobacteria | o__Burkholderiales | f__Sutterellaceae | g__Parasutterella |  |
| 04933519 | Bacteria | p__Bacteroidota | c__Bacteroidia | o__Chitinophagales | f__Chitinophagaceae | g__Parasegetibacter | s__Parasegetibacter_luojiensis |
| MJ034-1-barcode33-umi19319bins-ubs-12 | Bacteria | p__Proteobacteria | c__Alphaproteobacteria | o__Rhizobiales | f__Devosiaceae | g__Devosia |  |
| 018b1b3a493a569c579ac5ffb8ac3043 | Bacteria | p__Verrucomicrobiota | c__Verrucomicrobiae | o__Chthoniobacterales |  |  |  |
| 252a7546a0a788a3be20fa498fb9a7d1 | Bacteria | p__Acidobacteriota | c__Vicinamibacteria | o__Vicinamibacterales | f__uncultured | g__uncultured | s__uncultured_bacterium |
| 383b686f9a30561d3798ce9e05ca5caa | Bacteria | p__Proteobacteria | c__Alphaproteobacteria | o__Rhizobiales | f__Rhizobiaceae |  |  |
| 877fdaf9360536921dfe82f1d8c373bc | Bacteria | p__Proteobacteria | c__Gammaproteobacteria | o__Burkholderiales | f__Comamonadaceae |  |  |
| 11129774 | Bacteria | p__Bacteroidota | c__Bacteroidia | o__Bacteroidales | f__Muribaculaceae | g__Muribaculaceae | s__uncultured_bacterium |
| RS-GCF-003226135.1-NZ-QKNQ01000009.1 | Bacteria | p__Proteobacteria | c__Gammaproteobacteria | o__Enterobacterales | f__Morganellaceae | g__Providencia |  |
| 8764bb25ef3504658c7675ac84f90cdb | Bacteria | p__Actinobacteriota | c__Actinobacteria |  |  |  |  |
| 16770731 | Bacteria | p__Firmicutes | c__Clostridia | o__Lachnospirales | f__Lachnospiraceae | g__Lachnoclostridium |  |
| eb4ade45e6e2f89ea42e070e31b3a772 | Bacteria | p__Proteobacteria | c__Alphaproteobacteria | o__Rhizobiales | f__Beijerinckiaceae |  |  |
| MJ032-2-barcode70-umi97039bins-ubs-3 | Bacteria | p__Acidobacteriota | c__Blastocatellia | o__Pyrinomonadales | f__Pyrinomonadaceae | g__RB41 |  |
| d6d625d43d7d4e493e56e8a2f828e263 | Bacteria | p__Actinobacteriota | c__Thermoleophilia | o__Solirubrobacterales | f__Solirubrobacteraceae | g__Solirubrobacter |  |
| f3916c93e97d60e55ea27e763b35a587 | Bacteria | p__Proteobacteria | c__Gammaproteobacteria | o__Burkholderiales | f__Comamonadaceae |  |  |
| ba1a2a6fe373c8518e8f5bb2fcef2260 | Bacteria | p__Proteobacteria | c__Alphaproteobacteria | o__Caulobacterales | f__Caulobacteraceae | g__Phenylobacterium |  |
| 61c6368554df951eeff75801a3b3d06c | Bacteria | p__Proteobacteria | c__Alphaproteobacteria | o__Rhizobiales | f__Beijerinckiaceae | g__Microvirga |  |
| cdc85c974d6b360a67e2c50f39630103 | Bacteria | p__Bacteroidota | c__Bacteroidia | o__Chitinophagales | f__Chitinophagaceae | g__Sediminibacterium | s__Sediminibacterium_sp. |
| RS-GCF-017829785.1-NZ-JAGJTY010000056.1 | Bacteria | p__Proteobacteria | c__Gammaproteobacteria | o__Enterobacterales | f__Yersiniaceae | g__Serratia |  |
| 12f9e2a083fdbd1931c48f5de895a7d4 | Bacteria | p__Actinobacteriota | c__Acidimicrobiia | o__Microtrichales | f__Microtrichaceae | g__IMCC26207 |  |
| 05951411 | Bacteria | p__Acidobacteriota | c__Vicinamibacteria | o__Vicinamibacterales | f__Vicinamibacteraceae | g__Vicinamibacteraceae |  |
| 997340a47c11c833b17effa2b112e868 | Bacteria | p__Gemmatimonadota | c__Gemmatimonadetes | o__Gemmatimonadales | f__Gemmatimonadaceae | g__uncultured |  |
| 04955630 | Bacteria | p__Firmicutes | c__Clostridia | o__Peptostreptococcales-Tissierellales | f__Peptostreptococcaceae | g__Romboutsia |  |
| ca23c2ea0050503caadb7d2f010d4803 | Bacteria | p__Chloroflexi | c__Dehalococcoidia | o__S085 | f__S085 | g__S085 | s__metagenome |
| EF567306 | Bacteria | p__Actinobacteriota | c__Actinobacteria | o__Micrococcales | f__Microbacteriaceae | g__Microbacterium |  |
| a7c36eab13c8dc1e0d307c16700ef816 | Bacteria | p__Proteobacteria | c__Alphaproteobacteria | o__Sphingomonadales | f__Sphingomonadaceae | g__Ellin6055 |  |
| a794e73f6e931694a0065856cf67ee31 | Bacteria | p__Actinobacteriota | c__Thermoleophilia | o__Gaiellales |  |  |  |
| 91d29a1fd504e4379c5ca3d862c76d21 | Bacteria | p__Proteobacteria | c__Alphaproteobacteria | o__Rhodobacterales | f__Rhodobacteraceae | g__Gemmobacter |  |
| 594250dae69ab90c73e6380dad9c8d1d | Bacteria | p__Actinobacteriota | c__Actinobacteria | o__Propionibacteriales | f__Nocardioidaceae | g__Nocardioides |  |
| 728a2dcba30b1c10987323b0a41af73a | Bacteria | p__Actinobacteriota | c__Actinobacteria | o__Frankiales | f__Sporichthyaceae | g__Longivirga |  |
| 04989169 | Bacteria | p__Proteobacteria | c__Gammaproteobacteria | o__Pseudomonadales | f__Moraxellaceae | g__Acinetobacter |  |
| G009781045_1 | Bacteria | p__Proteobacteria | c__Alphaproteobacteria | o__Rhizobiales | f__Xanthobacteraceae |  |  |
| KC788572 | Bacteria | p__Actinobacteriota | c__Actinobacteria | o__Corynebacteriales | f__Nocardiaceae | g__Rhodococcus | s__Rhodococcus_hoagii |
| 0d99b24206cacdce7e6d6cfa83902be8 | Bacteria | p__Actinobacteriota | c__Actinobacteria |  |  |  |  |
| 02409609 | Bacteria | p__Firmicutes | c__Clostridia | o__Oscillospirales | f__Ruminococcaceae | g__Faecalibacterium |  |
| 2972655ff6bdaa6a56da9d6d6d858428 | Bacteria | p__Actinobacteriota | c__Acidimicrobiia | o__Actinomarinales | f__uncultured | g__uncultured |  |
| MJ010-2-barcode64-umi154030bins-ubs-3 | Bacteria | p__Proteobacteria | c__Gammaproteobacteria | o__Enterobacterales | f__Enterobacteriaceae |  |  |
| 7cbe1e3f75edee77bdf0fc4cfc7a03ae | Bacteria | p__Firmicutes | c__Bacilli | o__Lactobacillales | f__Enterococcaceae | g__Enterococcus |  |
| d676902d3510403dcf8bc62bf517ec79 | Bacteria | p__Chloroflexi | c__Anaerolineae |  |  |  |  |
| 484376b459f8da83bd64fb205b57657a | Bacteria | p__Chloroflexi | c__Ktedonobacteria | o__C0119 | f__C0119 | g__C0119 | s__uncultured_Chloroflexi |
| 04833128 | Bacteria | p__Proteobacteria | c__Gammaproteobacteria | o__Pseudomonadales | f__Moraxellaceae | g__Alkanindiges |  |
| 240ec0a37a9f0a2b784de429e34d4cc9 | Bacteria | p__Proteobacteria | c__Alphaproteobacteria | o__Azospirillales | f__Azospirillales_Incertae_Sedis | g__Stella |  |
| 1f182dc54dee49a26aedb216a9dcd1c9 | Bacteria | p__Planctomycetota | c__Phycisphaerae | o__Tepidisphaerales | f__WD2101_soil_group | g__WD2101_soil_group |  |
| 02250899 | Bacteria | p__Proteobacteria | c__Alphaproteobacteria | o__Rhizobiales | f__Rhizobiales_Incertae_Sedis | g__uncultured |  |
| RS-GCF-005502275.1-NZ-SWLZ01000064.1 | Bacteria | p__Firmicutes | c__Bacilli | o__Bacillales | f__Bacillaceae | g__Bacillus |  |
| 44c05dcf23581c32eedfc265bb4f4dc9 | Bacteria | p__Proteobacteria | c__Alphaproteobacteria |  |  |  |  |
| 163eee8d9e54769c019288d7ddb3587d | Bacteria | p__Spirochaetota | c__Spirochaetia | o__Spirochaetales | f__Spirochaetaceae | g__uncultured |  |
| 98fe04e7e66f61b8d9d8fe2caa539d23 | Bacteria | p__Bacteroidota | c__Bacteroidia | o__Chitinophagales | f__Chitinophagaceae | g__Terrimonas |  |
| MJ032-2-barcode60-umi11998bins-ubs-41 | Bacteria | p__Bacteroidota | c__Bacteroidia | o__Flavobacteriales | f__Weeksellaceae | g__Moheibacter | s__Empedobacter_sp. |
| KU321297 | Bacteria | p__Proteobacteria | c__Gammaproteobacteria | o__Burkholderiales | f__Oxalobacteraceae |  |  |
| FJ214659 | Bacteria | p__Firmicutes | c__Bacilli | o__Bacillales | f__Marinococcaceae | g__Marinococcus | s__Marinococcus_sp. |
| RS-GCF-009789205.1-NZ-WSRY01000076.1 | Bacteria | p__Proteobacteria | c__Gammaproteobacteria | o__Pseudomonadales | f__Moraxellaceae | g__Acinetobacter |  |
| c57e9190570ffb7ff5485ff1e11b6787 | Bacteria | p__Actinobacteriota | c__Acidimicrobiia | o__Microtrichales | f__uncultured | g__uncultured |  |
| 4671fa4c48d001f083e892c64bb571b0 | Bacteria | p__Actinobacteriota | c__Actinobacteria | o__Micrococcales | f__Microbacteriaceae | g__Agromyces |  |
| 862a1043c1ef76bb1beb47d942d1df0b | Bacteria |  |  |  |  |  |  |
| a5d55b283a1abd27d0dc25e502c783bb | Bacteria | p__Bacteroidota | c__Bacteroidia | o__Bacteroidales | f__Prevotellaceae | g__Prevotellaceae_UCG-001 |  |
| 8b2c16986bb2c71163b587a07b6b155a | Bacteria | p__Actinobacteriota | c__Actinobacteria | o__Frankiales | f__Acidothermaceae | g__Acidothermus |  |
| 04879573 | Bacteria | p__Proteobacteria | c__Gammaproteobacteria | o__Pseudomonadales | f__Moraxellaceae | g__Acinetobacter |  |
| 04907526 | Bacteria | p__Proteobacteria | c__Alphaproteobacteria | o__Caulobacterales | f__Caulobacteraceae |  |  |
| 75844639093dd9af15bf156863878c2f | Bacteria | p__Chloroflexi | c__Anaerolineae | o__SJA-15 | f__SJA-15 | g__SJA-15 |  |
| 140ca7e247c0a658d8286bf1c1db9bc1 | Bacteria | p__Firmicutes | c__Bacilli | o__Bacillales | f__Marinococcaceae | g__Alteribacillus |  |
| 04935731 | Bacteria | p__Bacteroidota | c__Bacteroidia | o__Cytophagales | f__Hymenobacteraceae | g__Adhaeribacter | s__uncultured_soil |
| 67c454f2b5f2371aa015d7a3722a6f51 | Bacteria | p__Verrucomicrobiota | c__Verrucomicrobiae | o__Pedosphaerales | f__Pedosphaeraceae | g__Ellin517 |  |
| 04940715 | Bacteria | p__Bacteroidota | c__Bacteroidia | o__Bacteroidales | f__Rikenellaceae | g__Rikenellaceae_RC9_gut_group | s__uncultured_organism |
| MJ034-2-barcode51-umi2402bins-ubs-179 | Bacteria | p__Acidobacteriota | c__Blastocatellia | o__Blastocatellales | f__Blastocatellaceae | g__uncultured |  |
| KX426256 | Bacteria | p__Proteobacteria | c__Alphaproteobacteria | o__Rhizobiales | f__Rhizobiaceae | g__Phyllobacterium |  |
| dfdeae7167303e7811297a2db4c28bd6 | Bacteria | p__Bacteroidota | c__Bacteroidia | o__Bacteroidales | f__Rikenellaceae |  |  |
| 05122583 | Bacteria | p__Proteobacteria | c__Alphaproteobacteria | o__Rhizobiales | f__Rhizobiaceae | g__Pseudochrobactrum |  |
| RS-GCF-003579765.1-NZ-QYJS01000149.1 | Bacteria | p__Firmicutes | c__Bacilli | o__Staphylococcales | f__Staphylococcaceae | g__Staphylococcus |  |
| 07132418 | Bacteria | p__Verrucomicrobiota | c__Verrucomicrobiae | o__Chthoniobacterales | f__Xiphinematobacteraceae | g__Candidatus_Xiphinematobacter | s__uncultured_bacterium |
| b32e78ad85523fa74efcc12895f9cd0e | Bacteria | p__Firmicutes | c__Bacilli | o__Lactobacillales | f__Enterococcaceae | g__Enterococcus |  |
| 04900442 | Bacteria | p__Proteobacteria | c__Gammaproteobacteria | o__Pseudomonadales | f__Pseudomonadaceae | g__Pseudomonas |  |
| 04b130474b4d3efb0e97eeaa9e0488c8 | Bacteria | p__Actinobacteriota | c__Thermoleophilia | o__Gaiellales | f__uncultured | g__uncultured |  |
| 04898532 | Bacteria | p__Acidobacteriota | c__Acidobacteriae | o__Subgroup_2 | f__Subgroup_2 | g__Subgroup_2 |  |
| FM992368 | Bacteria | p__Actinobacteriota | c__Actinobacteria | o__Micrococcales | f__Dermacoccaceae | g__Kytococcus |  |
| JF824807 | Bacteria | p__Firmicutes | c__Bacilli | o__Erysipelotrichales | f__Erysipelotrichaceae | g__Dielma | s__Dielma_fastidiosa |
| e9f99c63f771669b333b072df6528f1b | Bacteria | p__Bacteroidota | c__Bacteroidia | o__Chitinophagales | f__Chitinophagaceae |  |  |
| RS-GCF-003052605.1-NZ-QASO01000077.1 | Bacteria | p__Proteobacteria | c__Gammaproteobacteria | o__Pseudomonadales | f__Pseudomonadaceae | g__Pseudomonas |  |
| RS-GCF-009679795.1-NZ-WKPZ01000065.1 | Bacteria | p__Firmicutes | c__Bacilli | o__Lactobacillales | f__Lactobacillaceae | g__Lactobacillus |  |
| 15780652 | Bacteria | p__Firmicutes | c__Clostridia | o__Oscillospirales | f__Oscillospiraceae |  |  |
| 04318204 | Bacteria | p__WPS-2 | c__WPS-2 | o__WPS-2 | f__WPS-2 | g__WPS-2 |  |
| 04946321 | Bacteria | p__Actinobacteriota | c__Actinobacteria | o__Streptosporangiales | f__Thermomonosporaceae |  |  |
| 04959850 | Bacteria | p__Proteobacteria | c__Gammaproteobacteria | o__Burkholderiales | f__Nitrosomonadaceae | g__mle1-7 |  |
| 2f58fcae8257503d73f158f5f304e598 | Bacteria | p__Myxococcota | c__Polyangia | o__Polyangiales | f__Polyangiaceae | g__Pajaroellobacter |  |
| 16fe0eec91478098b26b083b4d765aa8 | Bacteria | p__Bacteroidota | c__Bacteroidia | o__Chitinophagales | f__Chitinophagaceae | g__Terrimonas |  |
| MJ033-1-barcode40-umi11595bins-ubs-27 | Bacteria | p__Bacteroidota | c__Bacteroidia | o__Chitinophagales | f__Chitinophagaceae | g__Flavisolibacter | s__uncultured_Flavobacterium |
| RS-GCF-017315705.1-NZ-JAFLRF010000090.1 | Bacteria | p__Proteobacteria | c__Gammaproteobacteria | o__Burkholderiales | f__Burkholderiaceae | g__Burkholderia-Caballeronia-Paraburkholderia |  |
| c137e1351c2ba765526631ed8cd1325c | Bacteria | p__Proteobacteria | c__Alphaproteobacteria | o__Acetobacterales | f__Acetobacteraceae | g__Acidocella |  |
| RS-GCF-001653035.1-NZ-BCWH01000025.1 | Bacteria | p__Actinobacteriota | c__Actinobacteria | o__Corynebacteriales | f__Nocardiaceae | g__Rhodococcus |  |
| MJ010-1-barcode47-umi143200bins-ubs-3 | Bacteria | p__Bacteroidota | c__Bacteroidia | o__Sphingobacteriales | f__Sphingobacteriaceae | g__Sphingobacterium |  |
| afa3211f3079e5714000ccc932d4eb47 | Bacteria | p__Proteobacteria | c__Gammaproteobacteria | o__Burkholderiales |  |  |  |
| df2735070f6d006b44cc0d865bd8da59 | Bacteria | p__Proteobacteria | c__Gammaproteobacteria | o__Burkholderiales | f__Comamonadaceae |  |  |
| 440615227f5bd2a5147b4bfeb802c165 | Bacteria | p__Acidobacteriota | c__Holophagae | o__Subgroup_7 | f__Subgroup_7 | g__Subgroup_7 |  |
| 15164092 | Bacteria | p__Actinobacteriota | c__Thermoleophilia | o__Gaiellales | f__uncultured | g__uncultured | s__bacterium_Ellin6517 |
| 24a51d5b921ecde753d4a10e2f756652 | Bacteria | p__Proteobacteria | c__Gammaproteobacteria | o__Pseudomonadales | f__Pseudomonadaceae | g__Pseudomonas |  |
| MJ010-1-barcode47-umi20389bins-ubs-32 | Bacteria | p__Bacteroidota | c__Bacteroidia | o__Flavobacteriales | f__Flavobacteriaceae | g__Flavobacterium | s__Flavobacterium_ummariense |
| bc1f043cd41ef48b50126d4131c60182 | Bacteria | p__Acidobacteriota | c__Thermoanaerobaculia | o__Thermoanaerobaculales | f__Thermoanaerobaculaceae | g__Subgroup_10 |  |
| 02247673 | Bacteria | p__Proteobacteria | c__Alphaproteobacteria | o__Acetobacterales | f__Acetobacteraceae | g__Roseomonas |  |
| RS-GCF-003401015.1-NZ-PXLK01000057.1 | Bacteria | p__Proteobacteria | c__Gammaproteobacteria | o__Enterobacterales | f__Enterobacteriaceae |  |  |
| 04907327 | Bacteria | p__Proteobacteria | c__Alphaproteobacteria | o__Rhizobiales | f__Xanthobacteraceae |  |  |
| 948193fc6f016cfe4106e9d510160acf | Bacteria | p__Proteobacteria | c__Alphaproteobacteria | o__Rhizobiales | f__Beijerinckiaceae | g__Methylobacterium-Methylorubrum |  |
| MN069869 | Bacteria | p__Actinobacteriota | c__Actinobacteria | o__Kineosporiales | f__Kineosporiaceae | g__Kineococcus |  |
| 345b42f5a9c11ad3a35930493abbb530 | Bacteria | p__Proteobacteria | c__Alphaproteobacteria | o__Rhizobiales | f__Rhizobiaceae |  |  |
| 6efef244fdd213b14ff5499a2784e398 | Bacteria | p__Chloroflexi | c__Ktedonobacteria | o__Ktedonobacterales | f__JG30-KF-AS9 | g__JG30-KF-AS9 | s__uncultured_bacterium |
| 5efc15c10d997a635d895fa17c85d03d | Bacteria | p__Proteobacteria | c__Gammaproteobacteria | o__Burkholderiales | f__Nitrosomonadaceae | g__MND1 |  |
| 05030436 | Bacteria | p__Proteobacteria | c__Gammaproteobacteria | o__Burkholderiales | f__Alcaligenaceae | g__Achromobacter |  |
| eac180548d0f429277358a7929ff5559 | Bacteria | p__Actinobacteriota | c__Actinobacteria | o__Corynebacteriales | f__Dietziaceae | g__Dietzia |  |
| 31f0f1bb807e87984bcab49ba09f62af | Bacteria | p__Firmicutes | c__Bacilli | o__Erysipelotrichales | f__Erysipelatoclostridiaceae | g__Erysipelatoclostridium | s__uncultured_Clostridium |
| f03f0c2b3c673cf3f90dd425bc387d27 | Bacteria | p__Proteobacteria | c__Alphaproteobacteria | o__Rhodospirillales | f__uncultured | g__uncultured | s__Azospirillum_sp. |
| 18970547 | Bacteria | p__Proteobacteria | c__Gammaproteobacteria | o__Burkholderiales | f__Burkholderiaceae | g__Cupriavidus |  |
| RS-GCF-902377535.1-NZ-CABLBY010000023.1 | Bacteria | p__Proteobacteria | c__Gammaproteobacteria | o__Enterobacterales | f__Enterobacteriaceae |  |  |
| RS-GCF-004005905.1-NZ-SACO01000033.1 | Bacteria | p__Proteobacteria | c__Alphaproteobacteria | o__Sphingomonadales | f__Sphingomonadaceae | g__Novosphingobium |  |
| 04877206 | Bacteria | p__Bacteroidota | c__Bacteroidia | o__Flavobacteriales | f__Weeksellaceae | g__Chryseobacterium |  |
| 4efc882604bf809df4206919072994ea | Bacteria | p__Firmicutes | c__Bacilli | o__Erysipelotrichales | f__Erysipelatoclostridiaceae | g__Erysipelatoclostridium |  |
| 5988007c79910daa9d34b8fee241fe27 | Bacteria | p__Proteobacteria | c__Alphaproteobacteria | o__Rhizobiales | f__KF-JG30-B3 | g__KF-JG30-B3 |  |
| c0110d7dd60c4cb35a0aa7d00b44cade | Bacteria | p__Verrucomicrobiota | c__Verrucomicrobiae | o__Opitutales | f__Opitutaceae | g__Lacunisphaera | s__Oleiharenicola_alkalitolerans |
| d972c28ea21885020b09b0d383d2bf37 | Bacteria | p__Proteobacteria | c__Gammaproteobacteria | o__Burkholderiales | f__Comamonadaceae | g__Comamonas |  |
| 02247767 | Bacteria | p__Proteobacteria | c__Alphaproteobacteria | o__Sphingomonadales | f__Sphingomonadaceae | g__Sphingomonas | s__Sphingomonas_panni |
| 8f6541fc182046165e470800e0b5fc97 | Bacteria | p__Actinobacteriota | c__Acidimicrobiia | o__IMCC26256 | f__IMCC26256 | g__IMCC26256 |  |
| 5aa910972110aafe045b8985d70f9eca | Bacteria | p__Cyanobacteria | c__Cyanobacteriia | o__Cyanobacteriales |  |  |  |
| 910000c8605275bd2bd54486f48243d2 | Bacteria | p__Proteobacteria | c__Alphaproteobacteria | o__Rhizobiales | f__Rhizobiales_Incertae_Sedis | g__uncultured |  |
| 93df6254858fedbe7109050f38125074 | Bacteria | p__Proteobacteria | c__Gammaproteobacteria | o__Pseudomonadales | f__Moraxellaceae | g__Acinetobacter |  |
| 0829feeec0c9832a6ca14065b2633a32 | Bacteria | p__Firmicutes | c__Bacilli | o__Lactobacillales | f__Enterococcaceae | g__Enterococcus |  |
| 995282bc3a886b509e80c9775ae3f71b | Bacteria | p__Proteobacteria | c__Gammaproteobacteria | o__Burkholderiales | f__Oxalobacteraceae | g__Oxalobacter |  |
| 05034006 | Bacteria | p__Actinobacteriota | c__Actinobacteria | o__Propionibacteriales | f__Nocardioidaceae | g__Nocardioides |  |
| b75b6125bafa959156b65aebc8e74060 | Bacteria | p__Proteobacteria | c__Gammaproteobacteria | o__Pseudomonadales | f__Moraxellaceae | g__Acinetobacter |  |
| 04941919 | Bacteria | p__Firmicutes | c__Clostridia | o__Peptostreptococcales-Tissierellales | f__Peptostreptococcales-Tissierellales | g__Anaerococcus |  |
| ce59072886a338e9017110104baf7c27 | Bacteria | p__Proteobacteria | c__Alphaproteobacteria | o__Sphingomonadales | f__Sphingomonadaceae | g__Sphingomonas |  |
| KY117468 | Bacteria | p__Bacteroidota | c__Bacteroidia | o__Chitinophagales | f__Chitinophagaceae | g__Chitinophaga |  |
| 6d9d9d0fd8c7cb4043659c1f34dbed41 | Bacteria | p__Proteobacteria | c__Alphaproteobacteria | o__Rhizobiales |  |  |  |
| 156386e7cc430bb0ffb6c2ba7bbf164b | Bacteria | p__Planctomycetota | c__Planctomycetes | o__Gemmatales | f__Gemmataceae | g__uncultured |  |
| 3d2e1ed461464890f05942f909fd2ad5 | Bacteria | p__Actinobacteriota | c__Actinobacteria | o__Catenulisporales | f__Actinospicaceae | g__Actinospica | s__uncultured_actinobacterium |
| MJ031-1-barcode42-umi38989bins-ubs-3 | Bacteria | p__Verrucomicrobiota | c__Verrucomicrobiae | o__Pedosphaerales | f__Pedosphaeraceae | g__ADurb.Bin063-1 |  |
| 04876144 | Bacteria | p__Proteobacteria | c__Alphaproteobacteria | o__Rhizobiales | f__Rhizobiaceae | g__Allorhizobium-Neorhizobium-Pararhizobium-Rhizobium |  |
| d638d461d02df2d567d77007c3f65da1 | Bacteria | p__Firmicutes | c__Bacilli | o__Erysipelotrichales | f__Erysipelatoclostridiaceae | g__Erysipelatoclostridium |  |
| 05037878 | Bacteria | p__Firmicutes | c__Clostridia | o__Oscillospirales | f__Oscillospiraceae | g__Oscillibacter |  |
| MJ006-2-barcode70-umi144281bins-ubs-3 | Bacteria | p__Firmicutes | c__Clostridia | o__Lachnospirales | f__Lachnospiraceae | g__Roseburia |  |
| a57d7447655fc31834f84983cf7de1ed | Bacteria | p__Bacteroidota | c__Bacteroidia | o__Sphingobacteriales | f__Sphingobacteriaceae | g__Mucilaginibacter |  |
| 48c5966422b8fd926db8f9fb038d73e3 | Bacteria | p__Proteobacteria | c__Gammaproteobacteria | o__Pseudomonadales | f__Moraxellaceae | g__Acinetobacter |  |
| e64f5267fc4967212772c4b01a52fee0 | Bacteria | p__Firmicutes | c__Bacilli | o__Erysipelotrichales | f__Erysipelotrichaceae | g__Dubosiella | s__uncultured_bacterium |
| 01256191 | Bacteria | p__Firmicutes | c__Clostridia | o__Clostridia_vadinBB60_group | f__Clostridia_vadinBB60_group | g__Clostridia_vadinBB60_group |  |
| G900102835_1 | Bacteria | p__Bacteroidota | c__Bacteroidia | o__Sphingobacteriales | f__Sphingobacteriaceae | g__Sphingobacterium | s__Sphingobacterium_mizutaii |
| a273967c12138eb6dba94bd704852eb2 | Bacteria | p__Acidobacteriota | c__Vicinamibacteria | o__Vicinamibacterales | f__uncultured | g__uncultured |  |
| 19f87929743f414b99ed90f45bb730e1 | Bacteria | p__Chloroflexi | c__Anaerolineae | o__SBR1031 | f__A4b | g__A4b |  |
| 1c3a6885d5a3545aac2f88c3b1c5ba74 | Bacteria | p__Acidobacteriota | c__Vicinamibacteria | o__Vicinamibacterales | f__Vicinamibacteraceae | g__Vicinamibacteraceae |  |
| 02467241 | Bacteria | p__Acidobacteriota | c__Vicinamibacteria | o__Vicinamibacterales | f__uncultured | g__uncultured |  |
| MJ034-1-barcode31-umi30799bins-ubs-3 | Bacteria | p__Acidobacteriota | c__Acidobacteriae | o__Acidobacteriales | f__uncultured | g__uncultured |  |
| KF676669 | Bacteria | p__Proteobacteria | c__Alphaproteobacteria | o__Sphingomonadales | f__Sphingomonadaceae |  |  |
| 176c203eb55a228558b247f2c6c5b9a2 | Bacteria | p__Proteobacteria | c__Gammaproteobacteria | o__Burkholderiales | f__Comamonadaceae |  |  |
| 5a6bd59e3e7f7250e419cb58e5f788a8 | Bacteria | p__Actinobacteriota | c__Thermoleophilia | o__Gaiellales | f__uncultured | g__uncultured |  |
| G900148505_1 | Bacteria | p__Proteobacteria | c__Alphaproteobacteria | o__Rhizobiales |  |  |  |
| G002158865_1 | Bacteria | p__Proteobacteria | c__Gammaproteobacteria | o__Burkholderiales | f__Comamonadaceae | g__Ottowia |  |
| 917f32e5d486c48a5df129a6ccebee7d | Bacteria | p__Actinobacteriota | c__Thermoleophilia |  |  |  |  |
| AJ251463 | Bacteria | p__Actinobacteriota | c__Actinobacteria | o__Micrococcales | f__Brevibacteriaceae | g__Brevibacterium |  |
| 03408817 | Bacteria | p__Planctomycetota | c__Planctomycetes | o__Gemmatales | f__Gemmataceae | g__uncultured |  |
| G001896575_5 | Bacteria | p__Bacteroidota | c__Bacteroidia | o__Bacteroidales | f__Dysgonomonadaceae | g__Fermentimonas |  |
| 04946041 | Bacteria | p__Actinobacteriota | c__Actinobacteria | o__Streptomycetales | f__Streptomycetaceae | g__Streptomyces |  |
| 843b5bada51b41f722660337af425b9c | Bacteria | p__Patescibacteria | c__Dojkabacteria | o__Dojkabacteria | f__Dojkabacteria | g__Dojkabacteria |  |
| 51db29dae5595461e40c1b85df889cc9 | Bacteria | p__Acidobacteriota | c__Vicinamibacteria | o__Vicinamibacterales | f__Vicinamibacteraceae | g__Vicinamibacteraceae |  |
| 04944777 | Bacteria | p__Firmicutes | c__Clostridia | o__Oscillospirales | f__Ruminococcaceae | g__Incertae_Sedis | s__uncultured_bacterium |
| d610c7e23a34222b466dba35daf6e83e | Bacteria | p__Desulfobacterota | c__Desulfuromonadia | o__Geobacterales | f__Geobacteraceae |  |  |
| e90131c429ec8069cfa7eb3d47468d96 | Bacteria | p__Proteobacteria | c__Alphaproteobacteria | o__Elsterales | f__uncultured | g__uncultured |  |
| AJ746070 | Bacteria | p__Actinobacteriota | c__Actinobacteria | o__Corynebacteriales | f__Mycobacteriaceae | g__Mycobacterium |  |
| MJ030-2-barcode65-umi13223bins-ubs-41 | Bacteria | p__Acidobacteriota | c__Acidobacteriae | o__Acidobacteriales | f__Acidobacteriaceae_(Subgroup_1) | g__Granulicella |  |
| 795c8191e91cbc2cbb9bf8df4c10cec8 | Bacteria | p__Firmicutes | c__Clostridia | o__Peptostreptococcales-Tissierellales | f__Peptostreptococcaceae |  |  |
| AJ580498 | Bacteria | p__Proteobacteria | c__Gammaproteobacteria | o__Xanthomonadales | f__Rhodanobacteraceae | g__Luteibacter |  |
| MJ034-1-barcode25-umi755bins-ubs-479 | Bacteria | p__Proteobacteria | c__Gammaproteobacteria | o__Burkholderiales | f__Comamonadaceae |  |  |
| 10fbd78d68006ba51f642c01b50f2afb | Bacteria | p__Proteobacteria | c__Gammaproteobacteria | o__Pseudomonadales | f__Moraxellaceae | g__Acinetobacter |  |
| 04968133 | Bacteria | p__Firmicutes | c__Bacilli | o__Lactobacillales | f__Lactobacillaceae | g__Lactobacillus |  |
| GB-GCA-016721845.1-JADKHK010000005.1 | Bacteria | p__Proteobacteria | c__Gammaproteobacteria | o__Xanthomonadales | f__Rhodanobacteraceae | g__Ahniella |  |
| RS-GCF-009856545.1-NZ-CP047218.1 | Bacteria | p__Proteobacteria | c__Alphaproteobacteria | o__Sphingomonadales | f__Sphingomonadaceae | g__Sphingobium |  |
| 04941383 | Bacteria | p__Acidobacteriota | c__Vicinamibacteria | o__Vicinamibacterales | f__uncultured | g__uncultured |  |
| MJ020-1-barcode29-umi103006bins-ubs-3 | Bacteria | p__Proteobacteria | c__Alphaproteobacteria | o__Rhodobacterales | f__Rhodobacteraceae | g__Paracoccus |  |
| 99f7aa12e8df025312649fb25d36d3d4 | Bacteria | p__Acidobacteriota | c__Subgroup_25 | o__Subgroup_25 | f__Subgroup_25 | g__Subgroup_25 | s__uncultured_bacterium |
| 06354430 | Bacteria | p__Gemmatimonadota | c__S0134_terrestrial_group | o__S0134_terrestrial_group | f__S0134_terrestrial_group | g__S0134_terrestrial_group | s__uncultured_bacterium |
| 04877829 | Bacteria | p__Actinobacteriota | c__Actinobacteria | o__Micrococcales | f__Micrococcaceae | g__Kocuria |  |
| G000598965_1 | Bacteria | p__Bacteroidota | c__Bacteroidia | o__Bacteroidales | f__Bacteroidaceae | g__Bacteroides | s__Bacteroides_fragilis |
| ef2fac5c7c6ee464f3f5ee8ea220d885 | Bacteria | p__Bacteroidota | c__Bacteroidia | o__Bacteroidales | f__Prevotellaceae | g__Prevotella |  |
| MJ010-1-barcode26-umi106781bins-ubs-5 | Bacteria | p__Proteobacteria | c__Alphaproteobacteria | o__Rhizobiales | f__Xanthobacteraceae | g__uncultured |  |
| 26a04ab0502790697acc8e506136915b | Bacteria | p__Acidobacteriota | c__Acidobacteriae | o__Acidobacteriales |  |  |  |
| MJ034-1-barcode41-umi15836bins-ubs-17 | Bacteria | p__Proteobacteria | c__Gammaproteobacteria | o__Gammaproteobacteria_Incertae_Sedis | f__Unknown_Family | g__Acidibacter |  |
| fdd3c2cec7e0c32afece608bcab1c856 | Bacteria | p__Firmicutes | c__Bacilli | o__Bacillales | f__Marinococcaceae | g__Alteribacillus |  |
| b2f84c6b85badded787bfcdd2ab778b0 | Bacteria | p__RCP2-54 | c__RCP2-54 | o__RCP2-54 | f__RCP2-54 | g__RCP2-54 | s__uncultured_Desulfuromonadales |
| 02300691 | Bacteria | p__Actinobacteriota | c__Actinobacteria | o__Corynebacteriales | f__Corynebacteriaceae | g__Corynebacterium |  |
| MJ030-2-barcode65-umi19519bins-ubs-31 | Bacteria | p__Proteobacteria | c__Gammaproteobacteria | o__Gammaproteobacteria_Incertae_Sedis | f__Unknown_Family | g__Acidibacter | s__uncultured_proteobacterium |
| 442e88cfa038d521f34dfef4339ec29c | Bacteria | p__Proteobacteria | c__Gammaproteobacteria | o__Burkholderiales | f__Nitrosomonadaceae | g__mle1-7 |  |
| 9e3e3b2cd9b4b2f6536f4b21ad6afac1 | Bacteria | p__Proteobacteria | c__Gammaproteobacteria | o__Burkholderiales | f__Nitrosomonadaceae | g__Nitrosomonas |  |
| GB-GCA-900539315.1-UQBL01000026.1 | Bacteria | p__Firmicutes | c__Bacilli | o__Lactobacillales | f__Carnobacteriaceae | g__Granulicatella |  |
| 398928f7fb40695b4ead3260b5afe8c4 | Bacteria | p__Proteobacteria | c__Gammaproteobacteria | o__Pseudomonadales | f__Moraxellaceae | g__Acinetobacter |  |
| 04c82fb8b993ba3021a44c6070045edf | Bacteria | p__Actinobacteriota | c__Thermoleophilia | o__Gaiellales | f__uncultured | g__uncultured |  |
| ac9817a2a2a98889f62230979e7b3e4b | Bacteria | p__Acidobacteriota | c__Vicinamibacteria | o__Vicinamibacterales | f__Vicinamibacteraceae | g__Vicinamibacteraceae |  |
| f973a09b8ff36f33ca406b005362ed96 | Bacteria | p__Bacteroidota | c__Bacteroidia | o__Flavobacteriales | f__NS9_marine_group | g__NS9_marine_group |  |
| 54982165344c9a10d38b1db824966ee9 | Bacteria | p__Bacteroidota | c__Bacteroidia | o__Sphingobacteriales | f__AKYH767 | g__AKYH767 |  |
| RS-GCF-007829595.1-NZ-VLJR01000055.1 | Bacteria | p__Proteobacteria | c__Alphaproteobacteria | o__Rhizobiales | f__Rhizobiaceae |  |  |
| ea8b62f56459eb194c33c79824821881 | Bacteria | p__Proteobacteria | c__Gammaproteobacteria | o__Burkholderiales | f__Burkholderiaceae | g__Lautropia | s__metagenome |
| MJ006-1-barcode33-umi150609bins-ubs-3 | Bacteria | p__Firmicutes | c__Clostridia | o__Lachnospirales | f__Lachnospiraceae | g__[Ruminococcus]_torques_group |  |
| RS-GCF-014287855.1-NZ-JACOOV010000039.1 | Bacteria | p__Firmicutes | c__Clostridia | o__Clostridiales | f__Clostridiaceae | g__Clostridium_sensu_stricto_1 |  |
| RS-GCF-015549505.1-NZ-JADMYZ010000028.1 | Bacteria | p__Bacteroidota | c__Bacteroidia | o__Bacteroidales | f__Barnesiellaceae | g__Barnesiella |  |
| a70e6f38c6ef3cc4ef6853e0376c615f | Bacteria | p__Proteobacteria | c__Alphaproteobacteria | o__Sphingomonadales | f__Sphingomonadaceae | g__Sphingomonas |  |
| ee1d5271a2763640c5720ad3323457ed | Bacteria | p__Firmicutes | c__Bacilli | o__RF39 | f__RF39 | g__RF39 |  |
| 55d816edd588fd8608f18230d4acbd17 | Bacteria | p__Acidobacteriota | c__Acidobacteriae | o__Acidobacteriales | f__uncultured | g__uncultured |  |
| c8d84bc4dff6745a0eeae711bbfa21d5 | Bacteria | p__Chloroflexi | c__Ktedonobacteria | o__Ktedonobacterales | f__Ktedonobacteraceae | g__Ktedonobacter | s__bacterium_SOSP1-79 |
| 472eb67eac04a9dd22d393c3d3d226c8 | Bacteria | p__Actinobacteriota | c__Actinobacteria | o__Frankiales | f__Geodermatophilaceae | g__Klenkia |  |
| MJ030-2-barcode50-umi10907bins-ubs-46 | Bacteria | p__Acidobacteriota | c__Acidobacteriae | o__Acidobacteriales | f__Acidobacteriaceae_(Subgroup_1) | g__Granulicella |  |
| 71381b8ac2624f461c4a9523b00ab7a6 | Bacteria | p__Proteobacteria | c__Gammaproteobacteria | o__Pseudomonadales | f__Moraxellaceae | g__Acinetobacter |  |
| MJ006-1-barcode40-umi135342bins-ubs-3 | Bacteria | p__Firmicutes | c__Clostridia | o__Christensenellales | f__Christensenellaceae | g__Christensenellaceae_R-7_group |  |
| af18b34344968497d1612314c6967f94 | Bacteria | p__Chloroflexi | c__Anaerolineae | o__SJA-15 | f__SJA-15 | g__SJA-15 |  |
| 921c4110eb94b81794a3d50c65d1a306 | Bacteria | p__Proteobacteria | c__Alphaproteobacteria | o__Elsterales | f__uncultured | g__uncultured |  |
| JQ762289 | Bacteria | p__Proteobacteria | c__Gammaproteobacteria | o__Oceanospirillales | f__Halomonadaceae | g__Halomonas |  |
| MJ030-2-barcode73-umi98497bins-ubs-4 | Bacteria | p__Firmicutes | c__Clostridia | o__Lachnospirales | f__Lachnospiraceae | g__Lachnospiraceae_NK4A136_group |  |
| JQ946365 | Bacteria | p__Proteobacteria | c__Gammaproteobacteria | o__Burkholderiales | f__Comamonadaceae |  |  |
| 04933473 | Bacteria | p__Bacteroidota | c__Bacteroidia | o__Chitinophagales | f__Chitinophagaceae |  |  |
| 05863692 | Bacteria | p__Planctomycetota | c__Phycisphaerae | o__Tepidisphaerales | f__WD2101_soil_group | g__WD2101_soil_group | s__uncultured_bacterium |
| 956b29674628292e692d1e24ccd79b0f | Bacteria | p__Verrucomicrobiota | c__Verrucomicrobiae | o__Chthoniobacterales |  |  |  |
| MJ034-1-barcode49-umi24545bins-ubs-5 | Bacteria | p__Acidobacteriota | c__Acidobacteriae | o__Acidobacteriales | f__Acidobacteriaceae_(Subgroup_1) |  |  |
| MW478902 | Bacteria | p__Firmicutes | c__Bacilli | o__Exiguobacterales | f__Exiguobacteraceae | g__Exiguobacterium |  |
| GQ339910 | Bacteria | p__Bacteroidota | c__Bacteroidia | o__Sphingobacteriales | f__Sphingobacteriaceae | g__Sphingobacterium | s__Sphingobacterium_bambusae |
| GB-GCA-018223365.1-JAGTTP010000001.1 | Bacteria | p__Firmicutes | c__Clostridia | o__Lachnospirales | f__Lachnospiraceae |  |  |
| RS-GCF-902162685.1-NZ-CABGWE010000038.1 | Bacteria | p__Proteobacteria | c__Gammaproteobacteria | o__Enterobacterales | f__Enterobacteriaceae |  |  |
| RS-GCF-003460985.1-NZ-QTWF01000009.1 | Bacteria | p__Firmicutes | c__Clostridia | o__Oscillospirales | f__Ruminococcaceae | g__Ruminococcus | s__Ruminococcus_bicirculans |
| 02339567 | Bacteria | p__Proteobacteria | c__Alphaproteobacteria | o__Rhizobiales | f__Rhizobiaceae |  |  |
| 1bee2954f1b82069fd74c14ac816e830 | Bacteria | p__Firmicutes | c__Bacilli | o__Paenibacillales | f__Paenibacillaceae | g__Ammoniphilus |  |
| 3a57fd20c79a58665c1fa9d8482628e1 | Bacteria | p__Proteobacteria | c__Gammaproteobacteria | o__Burkholderiales | f__Burkholderiaceae | g__Burkholderia-Caballeronia-Paraburkholderia |  |
| 97ac787c4693a17ad3281791f88271bf | Bacteria | p__Proteobacteria | c__Alphaproteobacteria | o__Rhizobiales | f__Rhizobiaceae |  |  |
| c36833ed9d0d26a343448d3b0ba8f76d | Bacteria | p__Gemmatimonadota | c__Gemmatimonadetes | o__Gemmatimonadales | f__Gemmatimonadaceae | g__uncultured |  |
| f025104e54973d39257d7ff70a46e851 | Bacteria | p__Actinobacteriota | c__Actinobacteria | o__Corynebacteriales | f__Mycobacteriaceae | g__Mycobacterium |  |
| KF876184 | Bacteria | p__Proteobacteria | c__Gammaproteobacteria | o__Enterobacterales | f__Erwiniaceae | g__Rosenbergiella |  |
| 968d6bc2da96dba8d66378036ff2a664 | Bacteria | p__Firmicutes | c__Bacilli | o__Bacillales | f__Bacillaceae | g__Bacillus |  |
| 00930060 | Bacteria | p__Firmicutes | c__Clostridia | o__Peptostreptococcales-Tissierellales | f__Anaerovoracaceae | g__Family_XIII_UCG-001 | s__uncultured_bacterium |
| 1275acb83b47205fce54d98fabda620f | Bacteria | p__Firmicutes | c__Bacilli | o__Lactobacillales | f__Carnobacteriaceae | g__Atopostipes |  |
| 338195669dacabdfdb209d462c2ab50a | Bacteria | p__Chloroflexi | c__Anaerolineae | o__Caldilineales | f__Caldilineaceae | g__uncultured | s__uncultured_bacterium |
| 04901870 | Bacteria | p__Gemmatimonadota | c__Longimicrobia | o__Longimicrobiales | f__Longimicrobiaceae | g__Longimicrobium | s__uncultured_bacterium |
| 21289983 | Bacteria | p__Firmicutes | c__Clostridia | o__Clostridiales | f__Clostridiaceae | g__Clostridium_sensu_stricto_1 |  |
| RS-GCF-003943785.1-NZ-RJMZ01000014.1 | Bacteria | p__Firmicutes | c__Bacilli | o__Lactobacillales | f__Streptococcaceae | g__Streptococcus |  |
| d4d82ab96cdfd1906413fe80aec24fce | Bacteria | p__Actinobacteriota | c__Actinobacteria | o__Corynebacteriales | f__Mycobacteriaceae | g__Mycobacterium |  |
| e1f40b1a0a02294b95c619a1be97cbbf | Bacteria | p__Proteobacteria | c__Gammaproteobacteria | o__Enterobacterales | f__Enterobacteriaceae | g__Escherichia-Shigella |  |
| Y16263 | Bacteria | p__Actinobacteriota | c__Actinobacteria | o__Micrococcales | f__Micrococcaceae | g__Kocuria |  |
| MJ020-1-barcode29-umi72164bins-ubs-5 | Bacteria | p__Firmicutes | c__Bacilli | o__Lactobacillales | f__Enterococcaceae | g__Enterococcus |  |
| b5ecbf04b35186356975eac09faf5a5c | Bacteria | p__Firmicutes | c__Clostridia | o__Clostridiales | f__Clostridiaceae | g__Clostridium_sensu_stricto_10 |  |
| 325fcd11c4980e777836364378c5105a | Bacteria | p__Proteobacteria | c__Gammaproteobacteria | o__Xanthomonadales | f__Xanthomonadaceae | g__Stenotrophomonas |  |
| 02282387 | Bacteria | p__Bdellovibrionota | c__Bdellovibrionia | o__Bdellovibrionales | f__Bdellovibrionaceae | g__Bdellovibrio |  |
| 498ba476051f3023e4e1de67cc877ca7 | Bacteria | p__Firmicutes | c__Clostridia | o__Oscillospirales | f__[Eubacterium]_coprostanoligenes_group | g__[Eubacterium]_coprostanoligenes_group |  |
| 02938275 | Bacteria | p__Methylomirabilota | c__Methylomirabilia | o__Rokubacteriales | f__Rokubacteriales | g__Rokubacteriales | s__uncultured_bacterium |
| G000368025_3 | Bacteria | p__Proteobacteria | c__Gammaproteobacteria | o__Pseudomonadales | f__Moraxellaceae | g__Acinetobacter |  |
| 7dcbb913f15914ca3902d679d8496a3d | Bacteria | p__Chloroflexi | c__Chloroflexia | o__Chloroflexales | f__Roseiflexaceae | g__Kouleothrix | s__uncultured_bacterium |
| 63d59a8d69000435ae2ed7475ee092eb | Bacteria | p__Desulfobacterota | c__Syntrophobacteria | o__Syntrophobacterales | f__Syntrophobacteraceae | g__Desulforhabdus |  |
| 06db7cd7b2523d7e59f472e594d1289d | Bacteria | p__Acidobacteriota | c__Vicinamibacteria | o__Vicinamibacterales | f__Vicinamibacteraceae | g__Vicinamibacteraceae |  |
| a6edbba7a769d6cdb7ba460c6076abab | Bacteria | p__Proteobacteria | c__Gammaproteobacteria | o__Diplorickettsiales | f__Diplorickettsiaceae | g__Aquicella | s__uncultured_bacterium |
| MH077556 | Bacteria | p__Proteobacteria | c__Gammaproteobacteria | o__Burkholderiales | f__Comamonadaceae |  |  |
| 05012913 | Bacteria | p__Bacteroidota | c__Bacteroidia | o__Chitinophagales | f__Chitinophagaceae | g__Chitinophagaceae | s__Flavisolibacter_sp. |
| 1f3ed9bed7578ae0cd0eef1d9775182a | Bacteria | p__Actinobacteriota | c__MB-A2-108 | o__MB-A2-108 | f__MB-A2-108 | g__MB-A2-108 | s__uncultured_Actinomycetales |
| 10148297 | Bacteria | p__Bacteroidota | c__Bacteroidia | o__Flavobacteriales | f__Flavobacteriaceae | g__Flavobacterium |  |
| MJ031-1-barcode42-umi14741bins-ubs-41 | Bacteria | p__Proteobacteria | c__Gammaproteobacteria | o__Pseudomonadales | f__Moraxellaceae | g__Acinetobacter |  |
| 3007ffe715bc8994349d56ea757db4e9 | Bacteria | p__Cyanobacteria | c__Cyanobacteriia | o__Oxyphotobacteria_Incertae_Sedis | f__Unknown_Family | g__Leptolyngbya_ANT.L52.2 |  |
| 7d66f38a5b85e617d31373426056662f | Bacteria | p__Chloroflexi | c__Anaerolineae | o__SBR1031 | f__A4b | g__OLB13 |  |
| 88fab1d68a46c776e117b23e71ddb56f | Bacteria | p__Proteobacteria | c__Gammaproteobacteria | o__Enterobacterales | f__Enterobacteriaceae |  |  |
| 35146ac275d6f0e4d461c39139df0a46 | Bacteria | p__Actinobacteriota | c__Rubrobacteria | o__Rubrobacterales | f__Rubrobacteriaceae | g__Rubrobacter | s__Rubrobacter_xylanophilus |
| 05037130 | Bacteria | p__Firmicutes | c__Bacilli | o__Bacillales | f__Planococcaceae | g__Solibacillus |  |
| b5ec4c2ebf802bd10942aca49ddb5cea | Bacteria | p__Acidobacteriota | c__Acidobacteriae | o__Bryobacterales | f__Bryobacteraceae | g__Bryobacter |  |
| 4722bf6832f48df4783a34294ed3af47 | Bacteria | p__Proteobacteria | c__Alphaproteobacteria | o__Tistrellales | f__Geminicoccaceae | g__Candidatus_Alysiosphaera |  |
| e5d90a7f02a0d1146226dd5019abd021 | Bacteria | p__Firmicutes | c__Bacilli | o__Lactobacillales | f__Streptococcaceae | g__Lactococcus |  |
| RS-GCF-016495725.1-NZ-CP054705.1 | Bacteria | p__Firmicutes | c__Bacilli | o__Bacillales | f__Marinococcaceae | g__Natribacillus | s__uncultured_bacterium |
| 246adab845915553194d6682201c76b2 | Bacteria | p__Actinobacteriota | c__Actinobacteria | o__Kineosporiales | f__Kineosporiaceae | g__Kineococcus |  |
| 22c02d52cbda870a43a90c18d8a27d3b | Bacteria | p__Proteobacteria | c__Gammaproteobacteria | o__Pseudomonadales | f__Pseudomonadaceae | g__Pseudomonas | s__Pseudomonas_caeni |
| FJ969487 | Bacteria | p__Proteobacteria | c__Gammaproteobacteria | o__Burkholderiales | f__Oxalobacteraceae |  |  |
| b8b97ff88811bd043c5d8da9851dbc98 | Bacteria | p__Bacteroidota | c__Bacteroidia | o__Bacteroidales | f__Bacteroidetes_vadinHA17 | g__Bacteroidetes_vadinHA17 | s__uncultured_bacterium |
| 6933184341f5009451123c7750f157a9 | Bacteria | p__Proteobacteria | c__Alphaproteobacteria | o__Rhizobiales | f__Xanthobacteraceae | g__uncultured |  |
| MJ010-1-barcode47-umi152413bins-ubs-4 | Bacteria | p__Proteobacteria | c__Gammaproteobacteria | o__Burkholderiales | f__Comamonadaceae |  |  |
| KT946774 | Bacteria | p__Actinobacteriota | c__Actinobacteria | o__Micromonosporales | f__Micromonosporaceae | g__Hamadaea |  |
| dbf3a2cd62ccb5b003a5a7dc61e779db | Bacteria | p__Proteobacteria | c__Gammaproteobacteria | o__Pseudomonadales | f__Moraxellaceae | g__Acinetobacter |  |
| fb3d27b296caa459e74fd31cc1c2cc20 | Bacteria | p__Acidobacteriota | c__Vicinamibacteria | o__Subgroup_17 | f__Subgroup_17 | g__Subgroup_17 |  |
| GQ241325 | Bacteria | p__Actinobacteriota | c__Actinobacteria | o__Micrococcales | f__Microbacteriaceae | g__Agromyces |  |
| RS-GCF-000368045.1-NZ-KB849238.1 | Bacteria | p__Proteobacteria | c__Gammaproteobacteria | o__Pseudomonadales | f__Moraxellaceae | g__Acinetobacter |  |
| 2187d400a926df3e7de75b48fd5e31e5 | Bacteria | p__Proteobacteria | c__Gammaproteobacteria | o__Pseudomonadales | f__Moraxellaceae | g__Acinetobacter |  |
| 04957295 | Bacteria | p__Proteobacteria | c__Gammaproteobacteria | o__Burkholderiales | f__Comamonadaceae |  |  |
| 64aafa9afc08b52bf1ea6947eab52d70 | Bacteria | p__Proteobacteria | c__Alphaproteobacteria | o__Rhizobiales | f__Xanthobacteraceae |  |  |
| 01ebecf07fa4d258fe0901c8ad76ae23 | Bacteria | p__Proteobacteria | c__Gammaproteobacteria | o__Alteromonadales | f__Alteromonadaceae | g__Rheinheimera | s__uncultured_Rheinheimera |
| 920f80fef4c9ed0b34266b6089610d30 | Bacteria | p__Bacteroidota | c__Bacteroidia | o__Chitinophagales | f__Chitinophagaceae |  |  |
| 39e2f7ba41fe89468c73897dc4b2bdcb | Bacteria | p__Proteobacteria | c__Gammaproteobacteria | o__Pseudomonadales | f__Moraxellaceae | g__Acinetobacter |  |
| 14800837 | Bacteria | p__Chloroflexi | c__Ktedonobacteria | o__Ktedonobacterales | f__JG30-KF-AS9 | g__JG30-KF-AS9 | s__uncultured_bacterium |
| 14409216 | Bacteria |  |  |  |  |  |  |
| 827704c3b0701c4968a652de3fc09bd8 | Bacteria | p__Proteobacteria | c__Gammaproteobacteria | o__Burkholderiales | f__Comamonadaceae |  |  |
| 9596deebbdccb167fd27a8bfa0d55de5 | Bacteria | p__Proteobacteria | c__Gammaproteobacteria | o__Burkholderiales | f__Nitrosomonadaceae | g__MND1 |  |
| ab53f0f8dc5ec30faf2306f2b6f44d7c | Bacteria | p__Acidobacteriota | c__Vicinamibacteria | o__Subgroup_17 | f__Subgroup_17 | g__Subgroup_17 |  |
| 004468f0b6ee347c715688ee31f67a2b | Bacteria | p__Actinobacteriota | c__Thermoleophilia | o__Gaiellales |  |  |  |
| AJ010478 | Bacteria | p__Firmicutes | c__Bacilli | o__Bacillales | f__Bacillaceae | g__Anoxybacillus |  |
| 11105696 | Bacteria | p__Bacteroidota | c__Bacteroidia | o__Bacteroidales | f__Marinifilaceae | g__Odoribacter | s__uncultured_bacterium |
| MJ010-1-barcode29-umi143004bins-ubs-3 | Bacteria | p__Proteobacteria | c__Gammaproteobacteria | o__Pseudomonadales | f__Moraxellaceae | g__Acinetobacter | s__Acinetobacter_towneri |
| 128b1a0fee28862912f54efeb648902e | Bacteria | p__Proteobacteria | c__Gammaproteobacteria | o__Burkholderiales | f__Comamonadaceae | g__Azohydromonas |  |
| G011046735_1 | Bacteria | p__Proteobacteria | c__Alphaproteobacteria | o__Rhizobiales | f__Rhizobiales_Incertae_Sedis | g__Nordella | s__Nordella_oligomobilis |
| 04910971 | Bacteria | p__Proteobacteria | c__Gammaproteobacteria | o__Xanthomonadales | f__Xanthomonadaceae | g__Stenotrophomonas |  |
| 3e7c4a5aa4aecd395649169d1f42b293 | Bacteria | p__Firmicutes | c__Negativicutes | o__Veillonellales-Selenomonadales | f__Selenomonadaceae | g__Megamonas |  |
| MJ006-2-barcode68-umi137620bins-ubs-4 | Bacteria | p__Proteobacteria | c__Gammaproteobacteria | o__Enterobacterales | f__Enterobacteriaceae | g__Escherichia-Shigella |  |
| 16566951 | Bacteria | p__Firmicutes | c__Clostridia | o__Lachnospirales | f__Lachnospiraceae | g__Lachnospiraceae_UCG-006 | s__uncultured_bacterium |
| 8315641c27bb5602e51b6de0330c0d39 | Bacteria | p__Proteobacteria | c__Alphaproteobacteria |  |  |  |  |
| e6fe9ab9bd5f46989029bb9360ae5f5a | Bacteria |  |  |  |  |  |  |
| G001428865_1 | Bacteria | p__Proteobacteria | c__Alphaproteobacteria | o__Sphingomonadales | f__Sphingomonadaceae | g__Sphingomonas |  |
| 0b04b01f447ac8f8888bbb2d674b4c50 | Bacteria | p__Proteobacteria | c__Alphaproteobacteria | o__Sphingomonadales | f__Sphingomonadaceae | g__Sphingomonas |  |
| 04958800 | Bacteria | p__Actinobacteriota | c__Actinobacteria | o__Micrococcales | f__Microbacteriaceae |  |  |
| 10574685 | Bacteria | p__Bacteroidota | c__Bacteroidia | o__Bacteroidales | f__Rikenellaceae | g__Alistipes |  |
| 88155d65b86c3d10debd9e1f698d321c | Bacteria |  |  |  |  |  |  |
| 05013230 | Bacteria | p__Bacteroidota | c__Bacteroidia | o__Chitinophagales | f__Chitinophagaceae | g__Lacibacter | s__uncultured_Chitinophagaceae |
| 14841738 | Bacteria | p__Chloroflexi | c__Ktedonobacteria | o__C0119 | f__C0119 | g__C0119 | s__uncultured_bacterium |
| 82d990e7b70793bce3cdf04a469bad0d | Bacteria | p__Proteobacteria | c__Gammaproteobacteria | o__Burkholderiales | f__Hydrogenophilaceae | g__Hydrogenophilus |  |
| 14153800 | Bacteria | p__Bacteroidota | c__Bacteroidia | o__Bacteroidales | f__Rikenellaceae | g__Rikenellaceae_RC9_gut_group | s__uncultured_bacterium |
| 6a5c3eaa47a538356feb147724fd6fb1 | Bacteria | p__Actinobacteriota | c__MB-A2-108 | o__MB-A2-108 | f__MB-A2-108 | g__MB-A2-108 |  |
| 04995404 | Bacteria | p__Proteobacteria | c__Alphaproteobacteria | o__Rhizobiales | f__Rhizobiaceae |  |  |
| G003121705_1 | Bacteria | p__Actinobacteriota | c__Actinobacteria | o__Micrococcales |  |  |  |
| KF779477 | Bacteria | p__Actinobacteriota | c__Actinobacteria | o__Pseudonocardiales | f__Pseudonocardiaceae | g__Amycolatopsis |  |
| MJ030-2-barcode69-umi73716bins-ubs-6 | Bacteria | p__Firmicutes | c__Bacilli | o__Bacillales | f__Bacillaceae | g__Bacillus |  |
| MJ006-1-barcode46-umi129937bins-ubs-3 | Bacteria | p__Bacteroidota | c__Bacteroidia | o__Bacteroidales | f__Bacteroidaceae | g__Bacteroides | s__Bacteroides_massiliensis |
| 5fbf48b7134f9740890b883206720ceb | Bacteria | p__Proteobacteria | c__Gammaproteobacteria | o__Burkholderiales | f__Comamonadaceae | g__Comamonas |  |
| ffc4c613884451b46b862836dd926db2 | Bacteria | p__Actinobacteriota | c__Actinobacteria |  |  |  |  |
| 86e6b20198c19baa38cf23915847de5c | Bacteria | p__Actinobacteriota | c__Actinobacteria | o__Corynebacteriales | f__Corynebacteriaceae | g__Corynebacterium |  |
| 9d4348f34d26881a8a634fa46b9de6b0 | Bacteria | p__Proteobacteria | c__Alphaproteobacteria | o__Rhizobiales |  |  |  |
| GU138380 | Bacteria | p__Bacteroidota | c__Bacteroidia | o__Flavobacteriales | f__Weeksellaceae | g__Chryseobacterium |  |
| 04900300 | Bacteria | p__Proteobacteria | c__Gammaproteobacteria | o__Pseudomonadales | f__Pseudomonadaceae | g__Pseudomonas |  |
| ace843fff8363b54c8d6ce14f7ca26f5 | Bacteria | p__Firmicutes | c__Bacilli | o__Bacillales | f__Planococcaceae | g__Lysinibacillus |  |
| GB-GCA-902636365.1-CACPRG010000096.1 | Bacteria | p__Proteobacteria | c__Gammaproteobacteria | o__Pseudomonadales | f__Pseudomonadaceae | g__Pseudomonas |  |
| 09850664 | Bacteria | p__Bacteroidota | c__Bacteroidia | o__Bacteroidales | f__Prevotellaceae | g__Prevotellaceae_UCG-001 |  |
| 00204425 | Bacteria | p__Chloroflexi | c__Anaerolineae | o__Anaerolineales | f__Anaerolineaceae | g__uncultured | s__uncultured_soil |
| a2e738f07eb7620fdb7d75fb0ae0f88e | Bacteria | p__Actinobacteriota | c__Thermoleophilia | o__Solirubrobacterales | f__67-14 | g__67-14 |  |
| 13fc25aee4fc78fcce93c4cb3f9707cb | Bacteria | p__Proteobacteria | c__Gammaproteobacteria | o__Pseudomonadales | f__Moraxellaceae | g__Acinetobacter |  |
| 15dc69fa07c7a606f34335be0b91c637 | Bacteria | p__Chloroflexi | c__Anaerolineae | o__1-20 | f__1-20 | g__1-20 |  |
| 80c8f231044d37378766b8a48775111c | Bacteria | p__Bacteroidota | c__Bacteroidia | o__Flavobacteriales | f__Weeksellaceae | g__Chryseobacterium |  |
| a9eaf8f5a0875eb424df76e54bc5d889 | Bacteria | p__Chloroflexi | c__Ktedonobacteria | o__C0119 | f__C0119 | g__C0119 |  |
| 04941307 | Bacteria | p__Acidobacteriota | c__Vicinamibacteria | o__Vicinamibacterales | f__uncultured | g__uncultured |  |
| 4c68d0e8f7a22fa83f8cfe544183b555 | Bacteria | p__Actinobacteriota | c__MB-A2-108 | o__MB-A2-108 | f__MB-A2-108 | g__MB-A2-108 |  |
| a8a192a3d6cb6928405cf375c2b308f3 | Bacteria | p__Chloroflexi | c__KD4-96 | o__KD4-96 | f__KD4-96 | g__KD4-96 |  |
| 08858415 | Bacteria | p__Bdellovibrionota | c__Bdellovibrionia | o__Bacteriovoracales | f__Bacteriovoracaceae | g__Peredibacter | s__Peredibacter_starrii |
| 05011340 | Bacteria | p__Proteobacteria | c__Gammaproteobacteria | o__Enterobacterales | f__Enterobacteriaceae |  |  |
| AJ493583 | Bacteria | p__Proteobacteria | c__Gammaproteobacteria | o__Cellvibrionales | f__Cellvibrionaceae | g__Cellvibrio |  |
| RS-GCF-017834005.1-NZ-JAFICS010000025.1 | Bacteria | p__Proteobacteria | c__Gammaproteobacteria | o__Burkholderiales | f__Oxalobacteraceae | g__Herbaspirillum |  |
| 788de77ae5c6512193cb99036b2ff3a1 | Bacteria | p__Bdellovibrionota | c__Bdellovibrionia | o__Bdellovibrionales | f__Bdellovibrionaceae | g__Bdellovibrio |  |
| 08797474 | Bacteria | p__Proteobacteria | c__Gammaproteobacteria | o__Xanthomonadales | f__Xanthomonadaceae | g__Stenotrophomonas |  |
| MJ007-1-barcode36-umi14966bins-ubs-17 | Bacteria | p__Proteobacteria | c__Gammaproteobacteria | o__Pseudomonadales | f__Moraxellaceae | g__Acinetobacter |  |
| 05948211 | Bacteria | p__Acidobacteriota | c__Vicinamibacteria | o__Vicinamibacterales | f__Vicinamibacteraceae | g__Vicinamibacteraceae |  |
| 4b00bb14790f881b0995cf4e11ee5ee5 | Bacteria | p__Proteobacteria | c__Gammaproteobacteria | o__Burkholderiales | f__Comamonadaceae |  |  |
| KM598239 | Bacteria | p__Proteobacteria | c__Gammaproteobacteria | o__Burkholderiales | f__Comamonadaceae | g__Hydrogenophaga |  |
| 8d6a8fadfb8eb086ca94b933c0f02132 | Bacteria | p__Actinobacteriota | c__Actinobacteria | o__Micrococcales | f__Micrococcaceae |  |  |
| 04912107 | Bacteria | p__Nitrospirota | c__Nitrospiria | o__Nitrospirales | f__Nitrospiraceae | g__Nitrospira |  |
| f8b4377a32a68aab6998de2750044e7d | Bacteria | p__Proteobacteria | c__Alphaproteobacteria | o__Elsterales | f__URHD0088 | g__URHD0088 |  |
| 50718b9bc569f9983c42eb34dce21b60 | Bacteria | p__Proteobacteria | c__Gammaproteobacteria | o__Burkholderiales | f__Nitrosomonadaceae | g__mle1-7 |  |
| 42c123225d52b54dea2a7c47bbadce9b | Bacteria | p__Firmicutes | c__Bacilli | o__Lactobacillales | f__Enterococcaceae | g__Enterococcus |  |
| 2e817d30df16d86efed17c20d45edb92 | Bacteria | p__Proteobacteria | c__Alphaproteobacteria | o__Rhizobiales | f__Beijerinckiaceae |  |  |
| 36b7e5d9f10ccf5887de4614a87cdaf3 | Bacteria | p__Proteobacteria | c__Gammaproteobacteria | o__Pseudomonadales | f__Moraxellaceae |  |  |
| 05008083 | Bacteria | p__Proteobacteria | c__Alphaproteobacteria | o__Sphingomonadales | f__Sphingomonadaceae | g__Novosphingobium |  |
| 04900259 | Bacteria | p__Proteobacteria | c__Gammaproteobacteria | o__Pseudomonadales | f__Pseudomonadaceae | g__Pseudomonas |  |
| d214c28a5d7069689efd9b9f03afdfd1 | Bacteria | p__Actinobacteriota | c__Actinobacteria | o__Frankiales | f__Geodermatophilaceae | g__Geodermatophilus |  |
| 6866e400340184a4151993aa47b1dace | Bacteria | p__Acidobacteriota | c__Vicinamibacteria | o__Vicinamibacterales | f__uncultured | g__uncultured |  |
| KT988034 | Bacteria | p__Proteobacteria | c__Gammaproteobacteria | o__Cardiobacteriales | f__Wohlfahrtiimonadaceae |  |  |
| 147d00b496138e8b8fd7bba98214a329 | Bacteria | p__Actinobacteriota | c__Actinobacteria | o__Micrococcales | f__Micrococcaceae |  |  |
| AB546308 | Bacteria | p__Actinobacteriota | c__Actinobacteria | o__Micrococcales | f__Microbacteriaceae | g__Agromyces |  |
| d2a0b02a32df82c17124d820387b5b9c | Bacteria | p__Acidobacteriota | c__Vicinamibacteria | o__Vicinamibacterales | f__uncultured | g__uncultured |  |
| b3cd63b68e7a8283d5142bab132db170 | Bacteria | p__Acidobacteriota | c__Acidobacteriae | o__Subgroup_2 | f__Subgroup_2 | g__Subgroup_2 |  |
| 02360472 | Bacteria | p__Proteobacteria | c__Alphaproteobacteria | o__Rhizobiales | f__Beijerinckiaceae | g__Methylobacterium-Methylorubrum |  |
| MJ032-1-barcode26-umi78877bins-ubs-3 | Bacteria | p__Firmicutes | c__Bacilli | o__Paenibacillales | f__Paenibacillaceae | g__Ammoniphilus |  |
| 555af88e13ca0218c0b853687b555849 | Bacteria | p__Proteobacteria | c__Gammaproteobacteria | o__Burkholderiales | f__Comamonadaceae | g__Aquabacterium |  |
| MJ020-1-barcode41-umi100235bins-ubs-5 | Bacteria | p__Firmicutes | c__Clostridia | o__Clostridiales | f__Clostridiaceae | g__Clostridium_sensu_stricto_1 |  |
| 91f459defa227182da90b58510afa321 | Bacteria | p__Actinobacteriota | c__Actinobacteria | o__Propionibacteriales | f__Nocardioidaceae | g__Nocardioides |  |
| 31614b2dbb4e7c89a725b320ee7dda14 | Bacteria | p__Acidobacteriota | c__Blastocatellia | o__Pyrinomonadales | f__Pyrinomonadaceae | g__RB41 |  |
| 2c9c9892ac47e995cb3c55a44d42d310 | Bacteria | p__Actinobacteriota | c__Thermoleophilia | o__Gaiellales | f__uncultured | g__uncultured |  |
| d605f5b6ee0f0463312c09e8fa82e195 | Bacteria | p__Firmicutes | c__Clostridia | o__Peptostreptococcales-Tissierellales | f__Peptostreptococcaceae |  |  |
| G000445125_1 | Bacteria | p__Proteobacteria | c__Alphaproteobacteria | o__Sphingomonadales | f__Sphingomonadaceae |  |  |
| 11048187 | Bacteria | p__Bacteroidota | c__Bacteroidia | o__Bacteroidales | f__Muribaculaceae | g__Muribaculaceae |  |
| 8ba4c5faec336eddee11e4edc8e712fe | Bacteria | p__Chloroflexi | c__Anaerolineae |  |  |  |  |
| G003762615_1 | Bacteria | p__Bacteroidota | c__Bacteroidia | o__Bacteroidales | f__Muribaculaceae | g__Muribaculaceae | s__uncultured_bacterium |
| DQ822568 | Bacteria | p__Actinobacteriota | c__Actinobacteria | o__Micrococcales | f__Micrococcaceae | g__Rothia |  |
| GB-GCA-003585735.1-NQOH01000016.1 | Bacteria | p__Actinobacteriota | c__Actinobacteria | o__Bifidobacteriales | f__Bifidobacteriaceae | g__Gardnerella |  |
| MJ030-2-barcode62-umi44968bins-ubs-14 | Bacteria | p__Proteobacteria | c__Gammaproteobacteria | o__Burkholderiales | f__Comamonadaceae | g__Delftia |  |
| 866889b1a57079f8b543406646179563 | Bacteria | p__Proteobacteria | c__Alphaproteobacteria | o__Rhizobiales | f__Xanthobacteraceae |  |  |
| MJ010-1-barcode26-umi144652bins-ubs-3 | Bacteria | p__Proteobacteria | c__Alphaproteobacteria | o__Rhodobacterales | f__Rhodobacteraceae | g__Paracoccus |  |
| bfc86c4a1c6ec4833a94e20a308e997b | Bacteria | p__Firmicutes | c__Clostridia | o__Lachnospirales | f__Lachnospiraceae |  |  |
| 21f71b7b74fc0a7ba5a30eecc7255b81 | Bacteria | p__Proteobacteria | c__Gammaproteobacteria | o__Pseudomonadales | f__Moraxellaceae | g__Acinetobacter |  |
| 04951001 | Bacteria | p__Firmicutes | c__Clostridia | o__Lachnospirales | f__Lachnospiraceae | g__Blautia | s__Lachnospiraceae_bacterium |
| 909c98012a3b76ebd965b30eb23a7aba | Bacteria | p__Proteobacteria | c__Alphaproteobacteria | o__Rhizobiales | f__Rhizobiales_Incertae_Sedis | g__Bauldia | s__uncultured_Methylocystaceae |
| 3231cfc3fe31fe07f188b8a78166d4af | Bacteria | p__Acidobacteriota | c__Acidobacteriae | o__Acidobacteriales | f__Acidobacteriaceae_(Subgroup_1) | g__Acidipila |  |
| MJ006-1-barcode38-umi163149bins-ubs-3 | Bacteria | p__Firmicutes | c__Clostridia | o__Christensenellales | f__Christensenellaceae | g__Christensenellaceae_R-7_group | s__uncultured_prokaryote |
| 09e2cad34b7afde260432bb74e279c65 | Bacteria | p__Proteobacteria | c__Alphaproteobacteria | o__Rhizobiales | f__Xanthobacteraceae |  |  |
| RS-GCF-007666805.1-NZ-VDPX01000022.1 | Bacteria | p__Firmicutes | c__Bacilli | o__Staphylococcales | f__Staphylococcaceae | g__Staphylococcus |  |
| 8dcc14b5f16aae64881da40cedc3da22 | Bacteria | p__Proteobacteria | c__Gammaproteobacteria | o__Gammaproteobacteria_Incertae_Sedis | f__Unknown_Family | g__Acidibacter |  |
| f1b7ecddd44a503c1225b14bcc699324 | Bacteria | p__Firmicutes | c__Clostridia | o__Peptostreptococcales-Tissierellales | f__Peptostreptococcales-Tissierellales | g__Tissierella |  |
| 04899771 | Bacteria | p__Acidobacteriota | c__Vicinamibacteria | o__Vicinamibacterales | f__Vicinamibacteraceae | g__uncultured |  |
| 7b953cf2337a680690341df7f7b58545 | Bacteria | p__Actinobacteriota | c__Thermoleophilia | o__Solirubrobacterales | f__67-14 | g__67-14 |  |
| 336e5a4ae95dd395dccf5d1d7d8a81e3 | Bacteria | p__Proteobacteria | c__Gammaproteobacteria | o__Enterobacterales | f__Enterobacteriaceae |  |  |
| ec542f996ceaa7cc327de51707e6b622 | Bacteria | p__Actinobacteriota | c__Actinobacteria | o__Micromonosporales | f__Micromonosporaceae |  |  |
| 05029986 | Bacteria | p__Actinobacteriota | c__Actinobacteria | o__Propionibacteriales | f__Nocardioidaceae | g__Nocardioides |  |
| 7aa94fe307881a71cfd47766a067cff1 | Bacteria | p__Proteobacteria | c__Gammaproteobacteria |  |  |  |  |
| 7d9ed6def3e00c90f46bfb360088a1c5 | Bacteria | p__Proteobacteria | c__Gammaproteobacteria | o__Burkholderiales | f__Burkholderiaceae | g__Ralstonia |  |
| 9a5e87436d7ec67e99c5c59cb7b16b8f | Bacteria | p__Chloroflexi | c__KD4-96 | o__KD4-96 | f__KD4-96 | g__KD4-96 |  |
| fcc08c90b4c2ce7a969d5a5ccb5e403b | Bacteria | p__Proteobacteria | c__Gammaproteobacteria | o__Burkholderiales | f__Methylophilaceae | g__Methylophilus |  |
| 02250032 | Bacteria | p__Proteobacteria | c__Alphaproteobacteria | o__Rhodobacterales | f__Rhodobacteraceae | g__Rubellimicrobium |  |
| G000368625_1 | Bacteria | p__Proteobacteria | c__Gammaproteobacteria | o__Pseudomonadales | f__Moraxellaceae | g__Acinetobacter |  |
| G001476195_1 | Bacteria | p__Actinobacteriota | c__Actinobacteria |  |  |  |  |
| 2ce399de55a89687b665dc9c4108231f | Bacteria | p__Acidobacteriota | c__Vicinamibacteria | o__Vicinamibacterales | f__Vicinamibacteraceae | g__Vicinamibacteraceae |  |
| 02240078 | Bacteria | p__Verrucomicrobiota | c__Verrucomicrobiae | o__Verrucomicrobiales | f__Akkermansiaceae | g__Akkermansia | s__uncultured_bacterium |
| 02947978 | Bacteria | p__Chloroflexi | c__Chloroflexia | o__Chloroflexales | f__Roseiflexaceae | g__uncultured | s__uncultured_bacterium |
| G000612625_1 | Bacteria | p__Firmicutes | c__Bacilli | o__Bacillales | f__Bacillaceae | g__Bacillus |  |
| 4156b10e996fd247e5b485681357bfb5 | Bacteria | p__Actinobacteriota | c__Actinobacteria | o__Corynebacteriales | f__Corynebacteriaceae | g__Corynebacterium |  |
| MJ034-1-barcode31-umi9839bins-ubs-39 | Bacteria | p__Acidobacteriota | c__Acidobacteriae | o__Acidobacteriales | f__uncultured | g__uncultured |  |
| MJ010-1-barcode36-umi7060bins-ubs-60 | Bacteria | p__Firmicutes | c__Clostridia | o__Clostridia_UCG-014 | f__Clostridia_UCG-014 | g__Clostridia_UCG-014 | s__unidentified |
| b0387a59e0a80a338bb1e9373dd2566e | Bacteria | p__Chloroflexi | c__Anaerolineae | o__Caldilineales | f__Caldilineaceae | g__uncultured |  |
| a94c230b630c51ce7799d423a98a00d2 | Bacteria | p__Actinobacteriota | c__Actinobacteria | o__Bifidobacteriales | f__Bifidobacteriaceae | g__Bifidobacterium |  |
| 28d8ecb360b5827301e4825324799a6c | Bacteria | p__Acidobacteriota | c__Holophagae | o__Subgroup_7 | f__Subgroup_7 | g__Subgroup_7 |  |
| 02336148 | Bacteria | p__Actinobacteriota | c__Actinobacteria | o__Micrococcales | f__Microbacteriaceae | g__Microbacterium |  |
| bea0ad5961e5569de86f6d6e0d98fc85 | Bacteria | p__Proteobacteria | c__Alphaproteobacteria | o__Rhizobiales | f__Xanthobacteraceae |  |  |
| RS-GCF-009663755.1-NZ-RDBS01000044.1 | Bacteria | p__Bacteroidota | c__Bacteroidia | o__Bacteroidales | f__Rikenellaceae | g__Alistipes |  |
| 11168272 | Bacteria | p__Bacteroidota | c__Bacteroidia | o__Bacteroidales | f__Muribaculaceae | g__Muribaculaceae |  |
| RS-GCF-007625075.1-NZ-QMIE01000006.1 | Bacteria | p__Desulfobacterota | c__Desulfovibrionia | o__Desulfovibrionales | f__Desulfovibrionaceae | g__Desulfovibrio | s__Desulfovibrio_marinus |
| 17971590 | Bacteria | p__Chloroflexi | c__Ktedonobacteria | o__Ktedonobacterales | f__Ktedonobacteraceae | g__HSB_OF53-F07 |  |
| 6a71aa9971530814a631fb6fbdd14bab | Bacteria | p__Acidobacteriota | c__Subgroup_22 | o__Subgroup_22 | f__Subgroup_22 | g__Subgroup_22 |  |
| GB-GCA-016702615.1-JADJDR010000015.1 | Bacteria | p__Actinobacteriota | c__Actinobacteria | o__Actinomycetales | f__Actinomycetaceae | g__uncultured | s__metagenome |
| 8c8a04d1d87074bc0788c69bc26f65a3 | Bacteria | p__Proteobacteria | c__Gammaproteobacteria |  |  |  |  |
| f9cfa7eb197f25d9b41f9b8840db046b | Bacteria | p__Bacteroidota | c__Bacteroidia | o__Cytophagales | f__Spirosomaceae | g__Dyadobacter |  |
| eab703006b98a36824303a924fe3bb7a | Bacteria | p__Actinobacteriota | c__Actinobacteria | o__Micrococcales | f__Micrococcaceae |  |  |
| d3848b45ed72dca93add7db26df0c0a3 | Bacteria | p__Chloroflexi | c__Chloroflexia | o__Thermomicrobiales | f__JG30-KF-CM45 | g__JG30-KF-CM45 |  |
| JQ390507 | Bacteria | p__Firmicutes | c__Bacilli | o__Lactobacillales | f__Streptococcaceae | g__Streptococcus |  |
| 1b47f9461a71bb416209296d59ff2e6d | Bacteria | p__Firmicutes | c__Symbiobacteriia | o__Symbiobacteriales | f__Symbiobacteraceae | g__Symbiobacterium | s__uncultured_bacterium |
| RS-GCF-900188385.1-NZ-FZOI01000046.1 | Bacteria | p__Proteobacteria | c__Alphaproteobacteria | o__Azospirillales | f__Azospirillaceae | g__Niveispirillum |  |
| c7faf4c925db4f1860deb88abf00c1e9 | Bacteria | p__Chloroflexi | c__Chloroflexia | o__Chloroflexales | f__Roseiflexaceae | g__uncultured | s__uncultured_bacterium |
| 0c44447e9add0f8ad71a12c548c1c185 | Bacteria | p__Proteobacteria | c__Alphaproteobacteria | o__Rhizobiales | f__Xanthobacteraceae | g__Bradyrhizobium |  |
| 07182494 | Bacteria | p__Planctomycetota | c__Phycisphaerae | o__Tepidisphaerales | f__WD2101_soil_group | g__WD2101_soil_group | s__uncultured_bacterium |
| e7c3681dc530f9ce0b0fd30b23a6c875 | Bacteria | p__Proteobacteria | c__Alphaproteobacteria | o__Rhizobiales |  |  |  |
| MJ034-2-barcode72-umi2297bins-ubs-184 | Bacteria | p__Bacteroidota | c__Bacteroidia | o__Chitinophagales | f__Chitinophagaceae | g__Flavisolibacter |  |
| d2469151ee285f84b733478a749915d4 | Bacteria | p__Bacteroidota | c__Bacteroidia | o__Bacteroidales | f__Dysgonomonadaceae | g__Petrimonas |  |
| 367b7c4a2f2eed8c2910f248d5567ef6 | Bacteria | p__Gemmatimonadota | c__Gemmatimonadetes | o__Gemmatimonadales | f__Gemmatimonadaceae |  |  |
| b4359e1ac7f9ddcfa8780c18327d951f | Bacteria | p__Proteobacteria | c__Alphaproteobacteria | o__Rhizobiales | f__Beijerinckiaceae | g__Microvirga |  |
| 271d23547666e421b692517dcecaa4f4 | Bacteria | p__Firmicutes | c__Clostridia | o__Peptostreptococcales-Tissierellales | f__Peptostreptococcaceae |  |  |
| 63c97489c94050c76c41d39285cc08bf | Bacteria | p__Gemmatimonadota | c__Gemmatimonadetes | o__Gemmatimonadales | f__Gemmatimonadaceae | g__uncultured |  |
| bbf2363c1887eba32787f8a2d03b9f43 | Bacteria | p__Patescibacteria | c__Saccharimonadia | o__Saccharimonadales |  |  |  |
| 04901501 | Bacteria | p__Proteobacteria | c__Gammaproteobacteria | o__Pseudomonadales | f__Moraxellaceae | g__Acinetobacter |  |
| 0104ace10e7eaf67eb5099ab34c98c01 | Bacteria | p__Actinobacteriota | c__Thermoleophilia | o__Gaiellales | f__uncultured | g__uncultured |  |
| AB682173 | Bacteria | p__Proteobacteria | c__Alphaproteobacteria | o__Rhodobacterales | f__Rhodobacteraceae | g__Gemmobacter |  |
| e900e61fbaee75b63ec6699ecb362c87 | Bacteria | p__Actinobacteriota | c__Actinobacteria | o__Bifidobacteriales | f__Bifidobacteriaceae | g__Bifidobacterium |  |
| cc6230e2b85d622cb266caed5b36748b | Bacteria | p__Myxococcota | c__Polyangia | o__Polyangiales |  |  |  |
| 08148108 | Bacteria | p__Proteobacteria | c__Alphaproteobacteria | o__Acetobacterales | f__Acetobacteraceae | g__Roseomonas |  |
| G001427875_1 | Bacteria | p__Proteobacteria | c__Alphaproteobacteria | o__Rhizobiales |  |  |  |
| 797e535f2170cfb4c14f10cccdcb3485 | Bacteria | p__Actinobacteriota | c__Coriobacteriia | o__Coriobacteriales | f__Eggerthellaceae | g__Adlercreutzia |  |
| c954fa97a8d734f038944c307f54b838 | Bacteria | p__Proteobacteria | c__Alphaproteobacteria | o__Sphingomonadales | f__Sphingomonadaceae | g__Sphingobium |  |
| fc99a08b2217143a28f413ac66db0fdc | Bacteria | p__Proteobacteria | c__Alphaproteobacteria | o__Rhizobiales | f__Xanthobacteraceae | g__Pseudorhodoplanes |  |
| bd15b87128c2a70902a1b0c01b766b4e | Bacteria | p__Acidobacteriota | c__Acidobacteriae | o__Acidobacteriales | f__uncultured | g__uncultured |  |
| MT374271 | Bacteria | p__Proteobacteria | c__Gammaproteobacteria | o__Burkholderiales | f__Nitrosomonadaceae | g__Ellin6067 |  |
| 835aac0e5529306d5b5baafb1eab5154 | Bacteria | p__Methylomirabilota | c__Methylomirabilia | o__Rokubacteriales | f__Rokubacteriales | g__Rokubacteriales | s__uncultured_bacterium |
| 21524fd281916e15b5016bf9c680f775 | Bacteria | p__Gemmatimonadota | c__Gemmatimonadetes | o__Gemmatimonadales | f__Gemmatimonadaceae | g__uncultured |  |
| 940909f3a3cf8f41db48d26373470468 | Bacteria | p__Proteobacteria | c__Alphaproteobacteria | o__Rhizobiales | f__Xanthobacteraceae | g__Pseudolabrys |  |
| 98ebcb04e9577bee2e2ae80b62780e8c | Bacteria | p__Proteobacteria | c__Alphaproteobacteria | o__Rhizobiales | f__Hyphomicrobiaceae | g__Hyphomicrobium | s__Hyphomicrobium_denitrificans |
| d259dd23670d5eb15eab3d319af647fc | Bacteria | p__Firmicutes | c__Clostridia | o__Lachnospirales | f__Lachnospiraceae | g__Lachnospiraceae_NK4A136_group |  |
| 34792d2294f6a63cc51332a718926478 | Bacteria | p__Proteobacteria | c__Alphaproteobacteria | o__Rhizobiales | f__Hyphomicrobiaceae | g__Hyphomicrobium |  |
| 04956646 | Bacteria | p__Actinobacteriota | c__Actinobacteria | o__Micrococcales | f__Microbacteriaceae | g__Agromyces |  |
| ee5b8e20a71a38fb22a31a1f1dbdfd2c | Bacteria | p__Gemmatimonadota | c__Gemmatimonadetes | o__Gemmatimonadales | f__Gemmatimonadaceae | g__uncultured |  |
| 5813d423798d00f44fbe900ae919a6fe | Bacteria | p__Acidobacteriota | c__Vicinamibacteria | o__Vicinamibacterales | f__Vicinamibacteraceae | g__Vicinamibacteraceae |  |
| MJ030-2-barcode58-umi121343bins-ubs-3 | Bacteria | p__Proteobacteria | c__Gammaproteobacteria | o__Burkholderiales | f__Comamonadaceae | g__Hydrogenophaga |  |
| RS-GCF-014839505.1-NZ-JACYNV010000007.1 | Bacteria | p__Actinobacteriota | c__Actinobacteria | o__Micrococcales | f__Microbacteriaceae |  |  |
| 7c244fa4ad6319105458aa87b2b8b699 | Bacteria | p__Proteobacteria | c__Gammaproteobacteria | o__Burkholderiales | f__Alcaligenaceae |  |  |
| 04062969 | Bacteria | p__Proteobacteria | c__Alphaproteobacteria | o__Tistrellales | f__Geminicoccaceae | g__uncultured | s__uncultured_bacterium |
| c3d36c08097e6dfc113f14dbeee1bca5 | Bacteria | p__Proteobacteria | c__Gammaproteobacteria | o__Burkholderiales | f__Alcaligenaceae | g__Verticiella |  |
| G001431535_1 | Bacteria | p__Proteobacteria | c__Gammaproteobacteria | o__Xanthomonadales | f__Xanthomonadaceae | g__Stenotrophomonas |  |
| ef35e7cdb2bddc7f2cc52acf26589fe8 | Bacteria | p__Cyanobacteria | c__Cyanobacteriia | o__Cyanobacteriales |  |  |  |
| f2bddc9a8b536ddd6ab3a997b3fd428b | Bacteria | p__Proteobacteria | c__Gammaproteobacteria | o__Pseudomonadales | f__Moraxellaceae | g__Enhydrobacter |  |
| 709b42ef7b79c8f96e1e038a73c2d787 | Bacteria | p__Proteobacteria | c__Gammaproteobacteria | o__Xanthomonadales | f__Xanthomonadaceae | g__Luteimonas |  |
| eb10c971f6aaafdb61a266431176943a | Bacteria | p__Bacteroidota | c__Bacteroidia | o__Sphingobacteriales | f__Sphingobacteriaceae | g__Sphingobacterium |  |
| 789c45c3b3e9b9ec73819c37331f5964 | Bacteria | p__Chloroflexi | c__Ktedonobacteria | o__Ktedonobacterales | f__Ktedonobacteraceae | g__FCPS473 |  |
| 6f3f8246a890370ce46bed83868a1148 | Bacteria | p__Chloroflexi | c__Chloroflexia | o__Chloroflexales | f__Roseiflexaceae | g__uncultured |  |
| d8675a2c1e3cccbd0e9efc6d66276c6a | Bacteria | p__Desulfobacterota | c__Desulfovibrionia | o__Desulfovibrionales | f__Desulfomicrobiaceae | g__Desulfomicrobium |  |
| MJ033-1-barcode31-umi33430bins-ubs-6 | Bacteria | p__Acidobacteriota | c__Acidobacteriae | o__Solibacterales | f__Solibacteraceae | g__Candidatus_Solibacter |  |
| 8310cdf14b439e86c16d6eb318df6dc9 | Bacteria | p__Cyanobacteria | c__Cyanobacteriia | o__Oxyphotobacteria_Incertae_Sedis | f__Unknown_Family | g__Leptolyngbya_EcFYyyy-00 | s__uncultured_bacterium |
| DQ401093 | Bacteria | p__Actinobacteriota | c__Actinobacteria | o__Propionibacteriales | f__Nocardioidaceae | g__Nocardioides |  |
| 1cd3fe27b4694451dae5fc6519c74b6c | Bacteria | p__Proteobacteria | c__Gammaproteobacteria | o__R7C24 | f__R7C24 | g__R7C24 |  |
| 14801524 | Bacteria | p__Chloroflexi | c__Ktedonobacteria | o__Ktedonobacterales | f__JG30-KF-AS9 | g__JG30-KF-AS9 | s__uncultured_bacterium |
| 8efe3406826fda5a3f6f565436d97716 | Bacteria | p__Proteobacteria | c__Gammaproteobacteria | o__Burkholderiales | f__SC-I-84 | g__SC-I-84 |  |
| FJ042789 | Bacteria | p__Actinobacteriota | c__Actinobacteria | o__Propionibacteriales | f__Nocardioidaceae | g__Aeromicrobium |  |
| 05008603 | Bacteria | p__Acidobacteriota | c__Thermoanaerobaculia | o__Thermoanaerobaculales | f__Thermoanaerobaculaceae | g__Subgroup_10 |  |
| 43dba4ee60acc365c918a911a66e6f9d | Bacteria | p__Actinobacteriota | c__Thermoleophilia | o__Gaiellales | f__Gaiellaceae | g__Gaiella |  |
| 4b950707d139774852000f7a80d1d1b7 | Bacteria | p__Firmicutes | c__Bacilli | o__Lactobacillales | f__Streptococcaceae | g__Lactococcus | s__Lactococcus_garvieae |
| RS-GCF-003462425.1-NZ-QTYZ01000042.1 | Bacteria | p__Firmicutes | c__Clostridia | o__Lachnospirales | f__Lachnospiraceae | g__Blautia |  |
| 4e80f6481d2bd84304f3e80294bf7d00 | Bacteria | p__Actinobacteriota | c__Actinobacteria | o__Micrococcales | f__Micrococcaceae |  |  |
| 02247593 | Bacteria | p__Fusobacteriota | c__Fusobacteriia | o__Fusobacteriales | f__Fusobacteriaceae | g__Fusobacterium |  |
| MJ007-1-barcode49-umi10979bins-ubs-25 | Bacteria | p__Firmicutes | c__Clostridia | o__Caldicoprobacterales | f__Caldicoprobacteraceae | g__Caldicoprobacter | s__uncultured_prokaryote |
| 86e76f77ca51c44cd64f4f80dd2d4806 | Bacteria | p__Actinobacteriota | c__Thermoleophilia | o__Gaiellales | f__Gaiellaceae | g__Gaiella |  |
| d577a8861c5c30632486571560c4c483 | Bacteria | p__Proteobacteria | c__Alphaproteobacteria | o__Rhodobacterales | f__Rhodobacteraceae | g__Rhodobacter |  |
| MJ030-2-barcode50-umi43198bins-ubs-15 | Bacteria | p__Acidobacteriota | c__Acidobacteriae | o__Acidobacteriales | f__Acidobacteriaceae_(Subgroup_1) | g__Granulicella |  |
| KY363639 | Bacteria | p__Proteobacteria | c__Alphaproteobacteria | o__Reyranellales | f__Reyranellaceae | g__Reyranella |  |
| 4bdd43e5ed0172c99942a7de8af216d8 | Bacteria | p__Proteobacteria | c__Gammaproteobacteria | o__Cellvibrionales | f__Microbulbiferaceae | g__Microbulbifer |  |
| fd9d4f46cf3d7f50e45d6e94337bc2d5 | Bacteria | p__Actinobacteriota | c__Thermoleophilia | o__Solirubrobacterales | f__Solirubrobacteraceae |  |  |
| 14840250 | Bacteria | p__Chloroflexi | c__Ktedonobacteria | o__Ktedonobacterales | f__JG30-KF-AS9 | g__JG30-KF-AS9 | s__uncultured_bacterium |
| 02262110 | Bacteria | p__Actinobacteriota | c__Actinobacteria | o__Propionibacteriales | f__Propionibacteriaceae | g__Friedmanniella |  |
| 02411972 | Bacteria | p__Proteobacteria | c__Alphaproteobacteria | o__Rhizobiales | f__Xanthobacteraceae |  |  |
| 5b41eda5d0823ceef31d0f3f17854538 | Bacteria | p__Firmicutes | c__Bacilli | o__Erysipelotrichales | f__Erysipelatoclostridiaceae | g__Erysipelatoclostridium | s__uncultured_Clostridium |
| MJ010-1-barcode26-umi130429bins-ubs-4 | Bacteria | p__Proteobacteria | c__Alphaproteobacteria | o__Rhizobiales | f__Devosiaceae | g__Devosia |  |
| 02262721 | Bacteria | p__Actinobacteriota | c__Actinobacteria | o__Corynebacteriales | f__Corynebacteriaceae | g__Corynebacterium |  |
| 6a15dde4430bb4d2addea7d1c6380989 | Bacteria | p__Proteobacteria | c__Alphaproteobacteria | o__Rhizobiales | f__Hyphomicrobiaceae | g__Pedomicrobium | s__uncultured_bacterium |
| 6823d5af36d3acf0ad35736b751b6ccb | Bacteria | p__Proteobacteria | c__Alphaproteobacteria | o__Caulobacterales | f__Caulobacteraceae |  |  |
| 14844923 | Bacteria | p__Chloroflexi |  |  |  |  |  |
| 03165945 | Bacteria | p__Planctomycetota | c__Phycisphaerae | o__Tepidisphaerales | f__WD2101_soil_group | g__WD2101_soil_group |  |
| GB-GCA-002905915.1-MLEP01000147.1 | Bacteria | p__Proteobacteria | c__Gammaproteobacteria | o__Burkholderiales | f__Comamonadaceae | g__Delftia |  |
| fe8d4d575a0a65c6a153e4338a6a2316 | Bacteria | p__Planctomycetota | c__Phycisphaerae | o__Tepidisphaerales | f__WD2101_soil_group | g__WD2101_soil_group | s__uncultured_bacterium |
| f556a3573ac6343e03624a39fb62ee4f | Bacteria | p__Chloroflexi | c__Anaerolineae | o__Ardenticatenales | f__uncultured | g__uncultured |  |
| 04908985 | Bacteria | p__Proteobacteria | c__Alphaproteobacteria | o__Rhizobiales | f__Rhizobiaceae |  |  |
| 02416618 | Bacteria | p__Proteobacteria | c__Alphaproteobacteria | o__Caulobacterales | f__Caulobacteraceae | g__Brevundimonas |  |
| d4ec34dbe67dba119fd3c08575e5df38 | Bacteria | p__Proteobacteria | c__Alphaproteobacteria | o__Rhizobiales | f__Beijerinckiaceae | g__Methylobacterium-Methylorubrum |  |
| abaaf89b4b0405db9523ec9568998d20 | Bacteria | p__Proteobacteria | c__Gammaproteobacteria | o__Pseudomonadales | f__Moraxellaceae | g__Acinetobacter |  |
| 04901430 | Bacteria | p__Proteobacteria | c__Gammaproteobacteria | o__Pseudomonadales | f__Moraxellaceae | g__Acinetobacter |  |
| e609c2e03099ee483d585337a209d227 | Bacteria | p__Proteobacteria | c__Gammaproteobacteria | o__Burkholderiales | f__Oxalobacteraceae |  |  |
| 04965862 | Bacteria | p__Firmicutes | c__Bacilli | o__Exiguobacterales | f__Exiguobacteraceae | g__Exiguobacterium |  |
| 808a4173ace1a8b1d4f650054cc7cde5 | Bacteria | p__Myxococcota | c__Polyangia | o__Polyangiales | f__Sandaracinaceae | g__uncultured |  |
| 20328977 | Bacteria | p__Firmicutes | c__Bacilli | o__Bacillales | f__Bacillaceae | g__Bacillus |  |
| 8f054684f7151724088884955e491a3a | Bacteria | p__Actinobacteriota | c__Actinobacteria | o__Micrococcales | f__Microbacteriaceae | g__Leucobacter |  |
| 4ce585973c41390acddbfcfbb39d26a2 | Bacteria | p__Firmicutes | c__Clostridia | o__Peptostreptococcales-Tissierellales | f__Peptostreptococcaceae |  |  |
| AB682230 | Bacteria | p__Proteobacteria | c__Alphaproteobacteria | o__Rhodobacterales | f__Rhodobacteraceae | g__Paracoccus |  |
| AB894335 | Bacteria | p__Actinobacteriota | c__Actinobacteria | o__Streptomycetales | f__Streptomycetaceae |  |  |
| 87aa0441a09803b08fd1e83d2ac7c25a | Bacteria | p__Proteobacteria | c__Gammaproteobacteria | o__Pseudomonadales | f__Pseudomonadaceae | g__Pseudomonas |  |
| RS-GCF-000368845.1-NZ-KB849720.1--2 | Bacteria | p__Proteobacteria | c__Gammaproteobacteria | o__Pseudomonadales | f__Moraxellaceae | g__Acinetobacter |  |
| 6e17ac83d97bdfcf86f4dd8c6ded5b53 | Bacteria | p__Proteobacteria | c__Gammaproteobacteria | o__Pseudomonadales | f__Moraxellaceae | g__Acinetobacter |  |
| abd2b1dca967cfeca1854b84c3ff3952 | Bacteria | p__Proteobacteria | c__Alphaproteobacteria | o__Rhizobiales | f__Xanthobacteraceae | g__uncultured |  |
| d4dc5702c79383aa73fabe0e60de6c39 | Bacteria | p__Proteobacteria | c__Alphaproteobacteria | o__Holosporales | f__Holosporaceae | g__uncultured | s__uncultured_bacterium |
| 2c9bcebee5525bcf3b49ca70b78f5a55 | Bacteria | p__Firmicutes | c__Clostridia | o__Peptostreptococcales-Tissierellales | f__Sedimentibacteraceae | g__Sedimentibacter |  |
| 134925e8818a096d315a744c26d3e22c | Bacteria | p__Proteobacteria | c__Alphaproteobacteria | o__Rhizobiales | f__Beijerinckiaceae | g__Methylobacterium-Methylorubrum |  |
| 0205103751f9791dc216af9ad76b38df | Bacteria | p__Proteobacteria | c__Gammaproteobacteria | o__Pseudomonadales | f__Pseudomonadaceae | g__Pseudomonas |  |
| 02260199 | Bacteria | p__Patescibacteria | c__Gracilibacteria | o__JGI_0000069-P22 | f__JGI_0000069-P22 | g__JGI_0000069-P22 |  |
| ae4ded65d5531cf9e22cb51f5ef5ec42 | Bacteria | p__Proteobacteria | c__Alphaproteobacteria | o__Azospirillales | f__Azospirillaceae | g__Skermanella |  |
| G000154245_4 | Bacteria | p__Firmicutes | c__Clostridia | o__Lachnospirales | f__Lachnospiraceae | g__Coprococcus |  |
| f16396a66353239da984975d139547c6 | Bacteria | p__Bacteroidota | c__Bacteroidia | o__Bacteroidales | f__Rikenellaceae | g__DMER64 |  |
| 03123271 | Bacteria | p__Myxococcota | c__Polyangia | o__mle1-27 | f__mle1-27 | g__mle1-27 |  |
| 13702498 | Bacteria | p__Gemmatimonadota | c__Gemmatimonadetes | o__Gemmatimonadales | f__Gemmatimonadaceae |  |  |
| d07bf1664df0258f0b1f0c9145267848 | Bacteria | p__Proteobacteria | c__Alphaproteobacteria | o__Rhizobiales | f__Beijerinckiaceae |  |  |
| c910d15076e32e637d276dad30a5a1c4 | Bacteria | p__Actinobacteriota | c__Thermoleophilia | o__Gaiellales | f__uncultured | g__uncultured |  |
| 20332067 | Bacteria | p__Firmicutes | c__Bacilli | o__Bacillales | f__Bacillaceae |  |  |
| MJ006-2-barcode60-umi189791bins-ubs-4 | Bacteria | p__Firmicutes | c__Clostridia | o__Lachnospirales | f__Lachnospiraceae | g__Lachnospiraceae_ND3007_group |  |
| 08f3977a120bf281558439ebc08515e8 | Bacteria | p__Acidobacteriota | c__Vicinamibacteria | o__Vicinamibacterales | f__Vicinamibacteraceae | g__Vicinamibacteraceae |  |
| 204c1e7f9593e6d8e4631bcdddc83c8a | Bacteria | p__Firmicutes | c__Clostridia | o__Oscillospirales | f__Butyricicoccaceae | g__Butyricicoccus |  |
| MJ006-2-barcode56-umi52838bins-ubs-7 | Bacteria | p__Firmicutes | c__Clostridia | o__Clostridia_UCG-014 | f__Clostridia_UCG-014 | g__Clostridia_UCG-014 |  |
| LT840125 | Bacteria | p__Proteobacteria | c__Alphaproteobacteria | o__Acetobacterales | f__Acetobacteraceae | g__Rhodovarius | s__Rhodovarius_sp. |
| 53596730e91c762643e630b0acf1ef13 | Bacteria | p__Proteobacteria | c__Gammaproteobacteria | o__Burkholderiales | f__Comamonadaceae |  |  |
| 04878838 | Bacteria | p__Firmicutes | c__Clostridia | o__Clostridiales | f__Clostridiaceae | g__Clostridium_sensu_stricto_1 |  |
| a554153b0857d3efeb891cf2a10b1de2 | Bacteria | p__Proteobacteria | c__Alphaproteobacteria | o__Rhizobiales | f__Hyphomicrobiaceae | g__Hyphomicrobium |  |
| 12913296 | Bacteria | p__Bacteroidota | c__Bacteroidia | o__Chitinophagales |  |  |  |
| c83d00617e7ae4205cea9e52f0a2d116 | Bacteria | p__Acidobacteriota | c__Thermoanaerobaculia | o__Thermoanaerobaculales | f__Thermoanaerobaculaceae | g__Subgroup_10 | s__uncultured_prokaryote |
| GB-GCA-019137195.1-JAGVSR010000003.1 | Bacteria | p__Proteobacteria | c__Alphaproteobacteria | o__Rhizobiales | f__Hyphomicrobiaceae | g__Hyphomicrobium |  |
| fe3f5783db9ea5ccb9e8fb0652bb8a43 | Bacteria | p__Firmicutes | c__Clostridia | o__Lachnospirales | f__Lachnospiraceae | g__[Eubacterium]_xylanophilum_group | s__uncultured_bacterium |
| a76de5d58730206bacab9a11e724def7 | Bacteria | p__Bacteroidota | c__Bacteroidia | o__Bacteroidales | f__Bacteroidaceae | g__Bacteroides | s__Bacteroides_massiliensis |
| ebf81eacee11474051686c4067107856 | Bacteria | p__Bacteroidota | c__Bacteroidia | o__Sphingobacteriales | f__AKYH767 | g__AKYH767 |  |
| DQ417335 | Bacteria | p__Proteobacteria | c__Alphaproteobacteria | o__Rhizobiales | f__Labraceae | g__Labrys |  |
| G009911695_1 | Bacteria | p__Actinobacteriota | c__Coriobacteriia | o__Coriobacteriales | f__Eggerthellaceae | g__Enterorhabdus | s__mouse_gut |
| 96f25b0921dcf3dab2f81d409d3961d8 | Bacteria | p__Proteobacteria | c__Alphaproteobacteria | o__Acetobacterales | f__Acetobacteraceae |  |  |
| 04901459 | Bacteria | p__Proteobacteria | c__Gammaproteobacteria | o__Pseudomonadales | f__Moraxellaceae | g__Acinetobacter |  |
| 9f446d135c0e8409818503b3f90dd759 | Bacteria | p__Chloroflexi | c__Ktedonobacteria | o__Ktedonobacterales | f__JG30-KF-AS9 | g__JG30-KF-AS9 | s__uncultured_bacterium |
| RS-GCF-000620385.1-NZ-JIBJ01000033.1 | Bacteria | p__Actinobacteriota | c__Actinobacteria | o__Micrococcales | f__Micrococcaceae |  |  |
| MJ033-1-barcode40-umi2573bins-ubs-147 | Bacteria | p__Bacteroidota | c__Bacteroidia | o__Chitinophagales | f__Chitinophagaceae | g__Flavisolibacter |  |
| 7fb5bf01213511f81e10fbf465455843 | Bacteria | p__Actinobacteriota | c__Thermoleophilia | o__Solirubrobacterales | f__67-14 | g__67-14 | s__uncultured_Conexibacteraceae |
| f6f1317d4a090fc13540838c0099ec10 | Bacteria | p__Firmicutes | c__Bacilli | o__Bacillales | f__Bacillaceae | g__Bacillus |  |
| f2facfeb1df05de9d702c2a974fcc23e | Bacteria | p__Actinobacteriota | c__Actinobacteria | o__Pseudonocardiales | f__Pseudonocardiaceae | g__Amycolatopsis |  |
| f461a3515f1f69c6b61b39e51b80c133 | Bacteria | p__Actinobacteriota | c__Acidimicrobiia | o__IMCC26256 | f__IMCC26256 | g__IMCC26256 |  |
| 04898480 | Bacteria | p__Acidobacteriota | c__Acidobacteriae | o__Subgroup_2 | f__Subgroup_2 | g__Subgroup_2 | s__uncultured_eubacterium |
| 17463346 | Bacteria | p__Actinobacteriota | c__Thermoleophilia | o__Gaiellales | f__uncultured | g__uncultured | s__uncultured_Conexibacter |
| 12a3e36c983285d4d4c0e7de7ebf495f | Bacteria | p__Verrucomicrobiota | c__Verrucomicrobiae | o__Chthoniobacterales | f__Chthoniobacteraceae | g__Candidatus_Udaeobacter |  |
| MJ034-1-barcode26-umi19890bins-ubs-10 | Bacteria | p__Proteobacteria | c__Gammaproteobacteria |  |  |  |  |
| 4d0cde36ce35fec184fc8899da65dff7 | Bacteria | p__Proteobacteria | c__Alphaproteobacteria | o__Rhodobacterales | f__Rhodobacteraceae |  |  |
| 91aff1d53f9542a3824d9841cad67d1e | Bacteria | p__Proteobacteria | c__Alphaproteobacteria | o__Rhizobiales | f__Beijerinckiaceae | g__Methylobacterium-Methylorubrum |  |
| 14335568 | Bacteria | p__Acidobacteriota | c__Vicinamibacteria | o__Vicinamibacterales | f__uncultured | g__uncultured |  |
| 12498197 | Bacteria | p__Proteobacteria | c__Gammaproteobacteria | o__Xanthomonadales | f__Rhodanobacteraceae | g__Chujaibacter | s__uncultured_bacterium |
| 05030326 | Bacteria | p__Proteobacteria | c__Gammaproteobacteria | o__Burkholderiales | f__Comamonadaceae |  |  |
| MJ020-2-barcode70-umi5715bins-ubs-99 | Bacteria | p__Proteobacteria | c__Gammaproteobacteria | o__Burkholderiales | f__Oxalobacteraceae |  |  |
| fd49d88a49882acf6e56fa294e03e750 | Bacteria | p__Proteobacteria | c__Gammaproteobacteria | o__Pseudomonadales | f__Moraxellaceae | g__Acinetobacter |  |
| ae73f4a28274d8c8cbc5ce10c3601803 | Bacteria | p__Proteobacteria | c__Gammaproteobacteria | o__Xanthomonadales | f__Xanthomonadaceae | g__Vulcaniibacterium | s__uncultured_bacterium |
| GB-GCA-005502345.1-SCPA01000004.1 | Bacteria | p__Proteobacteria | c__Alphaproteobacteria | o__Rhizobiales | f__Rhizobiales_Incertae_Sedis | g__Nordella | s__Nordella_oligomobilis |
| G000297495_1 | Bacteria | p__Actinobacteriota | c__Actinobacteria | o__Micrococcales | f__Intrasporangiaceae |  |  |
| 93024ae0313231f0fa3b4d66cb814515 | Bacteria | p__Desulfobacterota | c__Desulfovibrionia | o__Desulfovibrionales | f__Desulfovibrionaceae | g__Desulfovibrio |  |
| d40b87480156ade0261a7462091f2ec8 | Bacteria | p__Actinobacteriota | c__Coriobacteriia | o__Coriobacteriales | f__Eggerthellaceae | g__Senegalimassilia |  |
| de492beb8b9ffcc83f8e991a4e5d8ad0 | Bacteria |  |  |  |  |  |  |
| 4cd687e1883a790fd786705612a7a64a | Bacteria | p__Proteobacteria | c__Gammaproteobacteria | o__Pseudomonadales | f__Moraxellaceae | g__Acinetobacter |  |
| RS-GCF-015739525.1-NZ-JADSTG010000034.1 | Bacteria | p__Proteobacteria | c__Gammaproteobacteria | o__Enterobacterales | f__Morganellaceae | g__Providencia |  |
| 6542652ea86ccfa63098832f1acf0a16 | Bacteria | p__Proteobacteria | c__Gammaproteobacteria | o__Enterobacterales | f__Enterobacteriaceae |  |  |
| 0857a0c25275d96c79c45557a87433f3 | Bacteria | p__Actinobacteriota | c__Coriobacteriia | o__Coriobacteriales | f__Coriobacteriaceae | g__Collinsella |  |
| ebf1a84f38b28d4e76450c3c36bf3d6d | Bacteria | p__Proteobacteria | c__Gammaproteobacteria | o__Burkholderiales | f__Burkholderiaceae | g__Ralstonia |  |
| 8db9667857b9e77d60fc226035a26471 | Bacteria | p__Actinobacteriota | c__Actinobacteria | o__Corynebacteriales | f__Corynebacteriaceae | g__Corynebacterium |  |
| 71e1893c3eada09f3d94858a48542434 | Bacteria | p__Proteobacteria | c__Gammaproteobacteria | o__Pseudomonadales | f__Pseudomonadaceae | g__Pseudomonas |  |
| c12140e6a0aeba8fe05befcadcbb7269 | Bacteria | p__RCP2-54 | c__RCP2-54 | o__RCP2-54 | f__RCP2-54 | g__RCP2-54 |  |
| f45886e80c34140819f271f16cee7d5d | Bacteria | p__Acidobacteriota | c__Acidobacteriae | o__Subgroup_2 | f__Subgroup_2 | g__Subgroup_2 |  |
| 01335c77dcfaa79dc679c5e18a0d4857 | Bacteria | p__Proteobacteria | c__Gammaproteobacteria | o__Burkholderiales | f__Nitrosomonadaceae | g__Ellin6067 |  |
| 04276474 | Bacteria | p__Actinobacteriota | c__Actinobacteria | o__Micromonosporales | f__Micromonosporaceae | g__Actinoplanes |  |
| 11fadc70f0eb9787432529e728986c5b | Bacteria | p__Proteobacteria | c__Gammaproteobacteria | o__Pseudomonadales | f__Moraxellaceae | g__Acinetobacter |  |
| d73b0857a6d9edc8140b2474e5aae49a | Bacteria | p__Proteobacteria | c__Gammaproteobacteria | o__Burkholderiales | f__Comamonadaceae |  |  |
| ba8ae7eadf8c633c6ece5b56d374948a | Bacteria | p__Proteobacteria | c__Gammaproteobacteria | o__Burkholderiales | f__Comamonadaceae | g__Ottowia | s__uncultured_bacterium |
| MJ020-1-barcode44-umi47663bins-ubs-10 | Bacteria | p__Firmicutes | c__Clostridia | o__Clostridiales | f__Clostridiaceae | g__Clostridium_sensu_stricto_10 |  |
| 14521997 | Bacteria | p__Gemmatimonadota | c__Gemmatimonadetes | o__Gemmatimonadales | f__Gemmatimonadaceae | g__uncultured |  |
| ca03cd46bc9a46351380de6a48062acd | Bacteria | p__Proteobacteria | c__Alphaproteobacteria | o__Sphingomonadales | f__Sphingomonadaceae |  |  |
| RS-GCF-018064205.1-NZ-CP072916.1--8 | Bacteria | p__Proteobacteria | c__Gammaproteobacteria | o__Burkholderiales | f__Comamonadaceae | g__Comamonas | s__Comamonas_aquatica |
| MJ007-2-barcode58-umi19224bins-ubs-20 | Bacteria | p__Proteobacteria | c__Gammaproteobacteria | o__Pseudomonadales | f__Moraxellaceae | g__Acinetobacter |  |
| G008764015_1 | Bacteria | p__Firmicutes | c__Bacilli | o__Staphylococcales | f__Staphylococcaceae | g__Staphylococcus |  |
| 72e39c2cee5bcf70627e3827db891e6e | Bacteria | p__Proteobacteria | c__Gammaproteobacteria | o__Pseudomonadales | f__Pseudomonadaceae | g__Pseudomonas |  |
| 30e75dc2376107898b94bd8188c5d1d4 | Bacteria | p__Desulfobacterota | c__uncultured | o__uncultured | f__uncultured | g__uncultured |  |
| RS-GCF-000760555.1-NZ-BBNK01000070.1 | Bacteria | p__Proteobacteria | c__Gammaproteobacteria | o__Pseudomonadales | f__Moraxellaceae | g__Acinetobacter | s__Acinetobacter_tandoii |
| ebf4b5f6925d80f5c3a4573ce0438098 | Bacteria | p__Actinobacteriota | c__Acidimicrobiia |  |  |  |  |
| c99e0aac5bb6ea57995b0d1a6bab5c9f | Bacteria | p__Gemmatimonadota | c__Gemmatimonadetes | o__Gemmatimonadales | f__Gemmatimonadaceae | g__uncultured | s__Acidobacteria_bacterium |
| G000344785_1 | Bacteria | p__Actinobacteriota | c__Actinobacteria | o__Corynebacteriales | f__Corynebacteriaceae | g__Corynebacterium | s__Corynebacterium_callunae |
| MJ006-1-barcode40-umi158125bins-ubs-3 | Bacteria | p__Firmicutes | c__Clostridia | o__Lachnospirales | f__Lachnospiraceae | g__Eisenbergiella |  |
| MJ006-2-barcode62-umi94507bins-ubs-4 | Bacteria | p__Bacteroidota | c__Bacteroidia | o__Bacteroidales | f__Bacteroidaceae | g__Bacteroides |  |
| 05017854 | Bacteria | p__Bacteroidota | c__Bacteroidia | o__Bacteroidales | f__Rikenellaceae | g__Rikenellaceae_RC9_gut_group |  |
| 02238842 | Bacteria | p__Chloroflexi | c__Anaerolineae | o__Anaerolineales | f__Anaerolineaceae | g__Anaerolinea |  |
| 59790bbbf335162f8faf44655d457f94 | Bacteria | p__Firmicutes | c__Clostridia | o__Oscillospirales | f__Oscillospiraceae |  |  |
| 7c2bb7d029c1f83a611a0391c2815d69 | Bacteria | p__Chloroflexi | c__Ktedonobacteria | o__Ktedonobacterales | f__Ktedonobacteraceae | g__HSB_OF53-F07 |  |
| FJ423764 | Bacteria | p__Actinobacteriota | c__Actinobacteria | o__Micrococcales | f__Microbacteriaceae |  |  |
| FN394513 | Bacteria | p__Firmicutes | c__Bacilli | o__Paenibacillales | f__Paenibacillaceae | g__Paenibacillus |  |
| RS-GCF-013111465.1-NZ-JAACMN010000029.1 | Bacteria | p__Proteobacteria | c__Gammaproteobacteria | o__Vibrionales | f__Vibrionaceae | g__Vibrio |  |
| 08183595 | Bacteria | p__Proteobacteria | c__Alphaproteobacteria | o__Rhizobiales | f__Xanthobacteraceae |  |  |
| 05002713 | Bacteria | p__Bacteroidota | c__Bacteroidia | o__Sphingobacteriales | f__Sphingobacteriaceae | g__Parapedobacter |  |
| 3a3d644a0832703675655d90a1a93d3c | Bacteria | p__Proteobacteria | c__Gammaproteobacteria | o__Burkholderiales | f__Nitrosomonadaceae | g__MND1 |  |
| MJ034-1-barcode41-umi17564bins-ubs-14 | Bacteria | p__Acidobacteriota | c__Blastocatellia | o__Blastocatellales | f__Blastocatellaceae |  |  |
| f894864ad86ac4b60a93067f60a151f3 | Bacteria | p__Proteobacteria | c__Alphaproteobacteria | o__Defluviicoccales | f__Defluviicoccaceae | g__Defluviicoccus |  |
| 415aeceed1055b79b6d3f817e294ba18 | Bacteria | p__Firmicutes | c__Bacilli | o__Erysipelotrichales | f__Erysipelatoclostridiaceae | g__Catenibacterium |  |
| 08336671 | Bacteria | p__Proteobacteria | c__Alphaproteobacteria | o__Caulobacterales | f__Caulobacteraceae | g__uncultured |  |
| 8f713357bba9982651180b6107c9cb65 | Bacteria | p__Actinobacteriota | c__Acidimicrobiia | o__Microtrichales | f__Ilumatobacteraceae | g__CL500-29_marine_group |  |
| 247b2c0086fabb24691c4eb970c70b38 | Bacteria | p__Proteobacteria | c__Gammaproteobacteria | o__Burkholderiales | f__Comamonadaceae | g__Comamonas |  |
| c11e413424a03f981038d891b110a01e | Bacteria | p__Dependentiae | c__Babeliae | o__Babeliales |  |  |  |
| 712e73fa32e7b5281fc8c4db98f686f4 | Bacteria | p__Proteobacteria | c__Gammaproteobacteria | o__Burkholderiales | f__Comamonadaceae | g__Limnohabitans |  |
| MJ034-2-barcode65-umi4359bins-ubs-67 | Bacteria | p__Acidobacteriota | c__Subgroup_11 | o__Subgroup_11 | f__Subgroup_11 | g__Subgroup_11 | s__uncultured_bacterium |
| KM407667 | Bacteria | p__Proteobacteria | c__Alphaproteobacteria | o__Rhodobacterales | f__Rhodobacteraceae | g__Gemmobacter | s__Gemmobacter_intermedius |
| b06731e904e2d03845a71597cb013046 | Bacteria | p__Actinobacteriota | c__Actinobacteria | o__FFCH16263 | f__FFCH16263 | g__FFCH16263 | s__uncultured_bacterium |
| 8b98f4d7796578708cda599e4fb5b22f | Bacteria | p__Actinobacteriota | c__Thermoleophilia | o__Gaiellales |  |  |  |
| MJ030-1-barcode42-umi9309bins-ubs-44 | Bacteria | p__Proteobacteria | c__Gammaproteobacteria | o__Burkholderiales | f__Comamonadaceae | g__Rhodoferax |  |
| 661c564e3820d9f7bb87894f99c089c3 | Bacteria | p__Actinobacteriota | c__Actinobacteria | o__Bifidobacteriales | f__Bifidobacteriaceae | g__Bifidobacterium |  |
| 9e8b3d4bcab81c104b314d6a0b83d2c4 | Bacteria | p__Proteobacteria | c__Gammaproteobacteria | o__Pseudomonadales | f__Moraxellaceae | g__Acinetobacter |  |
| 661a978aec8ceb5a5bd8549b2c4c373f | Bacteria | p__Proteobacteria | c__Gammaproteobacteria | o__Enterobacterales | f__Enterobacteriaceae |  |  |
| 7e6689c8da1dee171cdff8388856d580 | Bacteria | p__Actinobacteriota | c__Actinobacteria | o__Micrococcales | f__Micrococcaceae |  |  |
| 04957300 | Bacteria | p__Proteobacteria | c__Gammaproteobacteria | o__Burkholderiales | f__Comamonadaceae |  |  |
| MJ031-1-barcode30-umi22215bins-ubs-16 | Bacteria | p__Bacteroidota | c__Bacteroidia | o__Chitinophagales | f__Chitinophagaceae |  |  |
| 827232587c31bfffea40437de1a045dd | Bacteria | p__Proteobacteria | c__Alphaproteobacteria | o__Rhizobiales | f__Rhizobiaceae | g__Allorhizobium-Neorhizobium-Pararhizobium-Rhizobium |  |
| 3fb91eb31cd0f28517d0262c71667487 | Bacteria | p__Planctomycetota | c__Planctomycetes | o__Gemmatales | f__Gemmataceae | g__uncultured |  |
| 2e3890ae5fe52104f354581e47ef532c | Bacteria | p__Acidobacteriota | c__Vicinamibacteria | o__Vicinamibacterales | f__uncultured | g__uncultured |  |
| 5be5e8478eb6856233ad905fa7515ef5 | Bacteria | p__Gemmatimonadota | c__Gemmatimonadetes | o__Gemmatimonadales | f__Gemmatimonadaceae |  |  |
| 04941944 | Bacteria | p__Firmicutes | c__Clostridia | o__Peptostreptococcales-Tissierellales | f__Peptostreptococcales-Tissierellales | g__Anaerococcus | s__Anaerococcus_octavius |
| MJ007-1-barcode36-umi32022bins-ubs-5 | Bacteria | p__Proteobacteria | c__Gammaproteobacteria | o__Pseudomonadales | f__Moraxellaceae | g__Acinetobacter |  |
| 02244073 | Bacteria | p__Actinobacteriota | c__Actinobacteria |  |  |  |  |
| 70b3e41cf3ac8969a4e7bd993891e72b | Bacteria | p__Proteobacteria | c__Alphaproteobacteria | o__Rhizobiales | f__Hyphomicrobiaceae | g__Pedomicrobium |  |
| RS-GCF-001580865.1-NZ-LHZO01000030.1 | Bacteria | p__Proteobacteria | c__Alphaproteobacteria | o__Acetobacterales | f__Acetobacteraceae | g__Gluconobacter |  |
| MJ006-2-barcode74-umi245472bins-ubs-3 | Bacteria | p__Firmicutes | c__Bacilli | o__Lactobacillales | f__Streptococcaceae | g__Streptococcus |  |
| f39fb71a8294b7638901cbb55f9b49d5 | Bacteria | p__Proteobacteria | c__Alphaproteobacteria | o__Rhodobacterales | f__Rhodobacteraceae | g__Defluviimonas |  |
| G900538815_1 | Bacteria | p__Firmicutes | c__Clostridia | o__Oscillospirales | f__Oscillospiraceae | g__UCG-005 |  |
| 5d6e158b6f3edf75f298d25ef73f717e | Bacteria | p__Proteobacteria | c__Alphaproteobacteria | o__Rhizobiales |  |  |  |
| 13902997 | Bacteria | p__Methylomirabilota | c__Methylomirabilia | o__Rokubacteriales | f__WX65 | g__WX65 |  |
| 02399670 | Bacteria | p__Chloroflexi | c__KD4-96 | o__KD4-96 | f__KD4-96 | g__KD4-96 |  |
| b027a2f884a8fe37d4e96e5401f46ba3 | Bacteria | p__Planctomycetota | c__Planctomycetes | o__uncultured | f__uncultured | g__uncultured | s__uncultured_bacterium |
| 40f541a40ded294b6d312826aa0e338e | Bacteria | p__Chloroflexi | c__Anaerolineae | o__1-20 | f__1-20 | g__1-20 | s__metagenome |
| MJ030-2-barcode65-umi79255bins-ubs-6 | Bacteria | p__Acidobacteriota | c__Acidobacteriae | o__Acidobacteriales | f__Koribacteraceae | g__Candidatus_Koribacter |  |
| JN695619 | Bacteria | p__Proteobacteria | c__Alphaproteobacteria | o__Sphingomonadales | f__Sphingomonadaceae | g__Novosphingobium |  |
| 8d75b20cc53d2954c0d998695b790989 | Bacteria | p__Firmicutes | c__Clostridia | o__Lachnospirales | f__Lachnospiraceae | g__CHKCI001 | s__uncultured_bacterium |
| 160392a2d81ec8a75ba23ece5b4247c9 | Bacteria | p__Actinobacteriota | c__Thermoleophilia | o__Gaiellales |  |  |  |
| dd801ef611d67505b4cbac784e2d6d10 | Bacteria | p__Acidobacteriota | c__Acidobacteriae | o__Solibacterales | f__Solibacteraceae | g__Candidatus_Solibacter |  |
| 90a13858ffe9e6129b17fe53f592b195 | Bacteria | p__Proteobacteria | c__Gammaproteobacteria | o__Xanthomonadales | f__Rhodanobacteraceae | g__Dyella |  |
| 1410bdec0e50d181d55da244d06479b7 | Bacteria | p__Actinobacteriota | c__Actinobacteria | o__Pseudonocardiales | f__Pseudonocardiaceae | g__Saccharopolyspora |  |
| fa25250e8de51ba5f665069812062085 | Bacteria | p__Proteobacteria | c__Alphaproteobacteria | o__Micropepsales | f__Micropepsaceae | g__uncultured |  |
| 09c9a212071d064ca957b35f9c744060 | Bacteria | p__Proteobacteria | c__Gammaproteobacteria | o__Enterobacterales | f__Enterobacteriaceae | g__Escherichia-Shigella |  |
| 210d01ad68142ce730c71783b26d6a38 | Bacteria | p__Firmicutes | c__Clostridia | o__Lachnospirales | f__Lachnospiraceae |  |  |
| G000164635_1 | Bacteria | p__Proteobacteria | c__Alphaproteobacteria | o__Acetobacterales | f__Acetobacteraceae | g__Roseomonas | s__Roseomonas_cervicalis |
| bbd91ef7d026eb632baf85d40f548fc0 | Bacteria | p__Firmicutes | c__Bacilli | o__Lactobacillales | f__Lactobacillaceae | g__Lactobacillus |  |
| 02360519 | Bacteria | p__Proteobacteria | c__Alphaproteobacteria | o__Rhizobiales | f__Xanthobacteraceae | g__uncultured |  |
| 12904624 | Bacteria | p__Bacteroidota | c__Bacteroidia | o__Chitinophagales | f__Chitinophagaceae | g__Aurantisolimonas |  |
| a0f8538b856d871185d8e0dad2d85aab | Bacteria | p__Bacteroidota | c__Bacteroidia | o__Chitinophagales | f__Saprospiraceae | g__uncultured |  |
| MJ020-1-barcode41-umi13292bins-ubs-27 | Bacteria | p__Bacteroidota | c__Bacteroidia | o__Chitinophagales | f__Chitinophagaceae |  |  |
| RS-GCF-018919545.1-NZ-JAHLRC010000262.1 | Bacteria | p__Proteobacteria | c__Gammaproteobacteria | o__Enterobacterales | f__Enterobacteriaceae |  |  |
| 8a9678a5e1ddb93e34494a4735db9ae8 | Bacteria | p__Gemmatimonadota | c__Gemmatimonadetes | o__Gemmatimonadales | f__Gemmatimonadaceae | g__uncultured | s__uncultured_actinobacterium |
| 0b28ac8ccb9d577c4d1ef503158c6174 | Bacteria | p__Verrucomicrobiota | c__Verrucomicrobiae | o__Chthoniobacterales | f__Chthoniobacteraceae | g__Candidatus_Udaeobacter | s__Spartobacteria_bacterium |
| 05032942 | Bacteria | p__Actinobacteriota | c__Actinobacteria | o__Corynebacteriales | f__Corynebacteriaceae | g__Corynebacterium |  |
| RS-GCF-017583145.1-NZ-JAGEVQ010000023.1 | Bacteria | p__Firmicutes | c__Bacilli | o__Staphylococcales | f__Staphylococcaceae | g__Staphylococcus |  |
| f2b6faf91195ba5d82f6bb8861148549 | Bacteria | p__Acidobacteriota | c__Acidobacteriae | o__Solibacterales | f__Solibacteraceae | g__Candidatus_Solibacter | s__uncultured_eubacterium |
| 84edeffbc6d22f716cbb885a033b240c | Bacteria | p__Firmicutes | c__Bacilli | o__Bacillales | f__Bacillaceae | g__Bacillus |  |
| f3329e58a1826b25b3c74b5dad512521 | Bacteria | p__Proteobacteria | c__Gammaproteobacteria | o__Steroidobacterales | f__Steroidobacteraceae | g__uncultured |  |
| 7c0f3f7ed84d5c7f06449d54d2191fd6 | Bacteria | p__Proteobacteria | c__Alphaproteobacteria | o__Rhodobacterales | f__Rhodobacteraceae | g__Amaricoccus |  |
| f8b82bad9b927bc844b2ebd6fb72cbdf | Bacteria | p__Actinobacteriota | c__Actinobacteria | o__Micrococcales | f__Microbacteriaceae | g__Microbacterium |  |
| RS-GCF-900457115.1-NZ-UGYX01000002.1--6 | Bacteria | p__Bacteroidota | c__Bacteroidia | o__Sphingobacteriales | f__Sphingobacteriaceae | g__Sphingobacterium |  |
| RS-GCF-004001085.1-NZ-BIMM01000176.1 | Bacteria | p__Firmicutes | c__Bacilli | o__Paenibacillales | f__Paenibacillaceae | g__Paenibacillus |  |
| 639736b4d14f55cc76ff9589a38b41db | Bacteria | p__Bacteroidota | c__Bacteroidia | o__Chitinophagales | f__Chitinophagaceae | g__Ferruginibacter |  |
| d5d76ee215b5afdd90065d2f0db64b1e | Bacteria | p__Firmicutes | c__Bacilli | o__Alicyclobacillales | f__Alicyclobacillaceae | g__Alicyclobacillus |  |
| ba15f86ec1f929a158efa78243e27c7b | Bacteria | p__Firmicutes | c__Incertae_Sedis | o__DTU014 | f__DTU014 | g__DTU014 | s__metagenome |
| 4184bfccc9639e49f771bb40e161d7fb | Bacteria | p__Actinobacteriota | c__Actinobacteria | o__Kineosporiales | f__Kineosporiaceae |  |  |
| 6767ef5c5ae05aa4060fc45c7d6041c7 | Bacteria | p__Proteobacteria | c__Gammaproteobacteria | o__Enterobacterales |  |  |  |
| 07202113 | Bacteria | p__Acidobacteriota | c__Vicinamibacteria | o__Vicinamibacterales | f__Vicinamibacteraceae | g__Vicinamibacteraceae |  |
| 02411956 | Bacteria | p__Proteobacteria | c__Alphaproteobacteria | o__Rhizobiales | f__Rhizobiaceae |  |  |
| 09091738 | Bacteria | p__Nitrospirota | c__Nitrospiria | o__Nitrospirales | f__Nitrospiraceae | g__Nitrospira |  |
| G003752175_1 | Bacteria | p__Proteobacteria | c__Gammaproteobacteria | o__Burkholderiales | f__Comamonadaceae | g__Comamonas | s__Comamonadaceae_bacterium |
| d6710c8108a587acbcb3dee955788610 | Bacteria | p__Proteobacteria | c__Alphaproteobacteria | o__Rhizobiales | f__Xanthobacteraceae | g__Bradyrhizobium |  |
| 05100448 | Bacteria | p__Proteobacteria | c__Gammaproteobacteria | o__Enterobacterales |  |  |  |
| RS-GCF-014653095.1-NZ-BNAC01000011.1 | Bacteria | p__Actinobacteriota | c__Actinobacteria | o__Frankiales | f__Geodermatophilaceae | g__Klenkia |  |
| c866d2a8571fa73e8c8715bbe17ac915 | Bacteria | p__Firmicutes | c__Bacilli | o__Bacillales | f__Planococcaceae | g__Lysinibacillus |  |
| 04878368 | Bacteria | p__Actinobacteriota | c__Actinobacteria | o__Pseudonocardiales | f__Pseudonocardiaceae | g__Actinomycetospora |  |
| MJ010-2-barcode64-umi147618bins-ubs-3 | Bacteria | p__Bacteroidota | c__Bacteroidia | o__Flavobacteriales | f__Flavobacteriaceae | g__Flavobacterium | s__Flavobacterium_ummariense |
| MJ006-2-barcode58-umi170693bins-ubs-3 | Bacteria | p__Bacteroidota | c__Bacteroidia | o__Bacteroidales | f__Bacteroidaceae | g__Bacteroides | s__bacterium_NLAE-zl-H496 |
| c777abd6028a8c9217b710809038369b | Bacteria | p__Bacteroidota | c__Bacteroidia | o__Flavobacteriales | f__Flavobacteriaceae | g__Flavobacterium |  |
| 50d46b3ed5fd3a591b4f84e544dba9c4 | Bacteria | p__Actinobacteriota | c__Rubrobacteria | o__Rubrobacterales | f__Rubrobacteriaceae | g__Rubrobacter | s__Rubrobacter_bracarensis |
| GU721026 | Bacteria | p__Proteobacteria | c__Gammaproteobacteria | o__Burkholderiales | f__Comamonadaceae | g__Pseudorhodoferax |  |
| 81c9eb85f9320f19949d2b231734126c | Bacteria | p__Firmicutes | c__Bacilli | o__Lactobacillales | f__Streptococcaceae | g__Streptococcus |  |
| MJ030-1-barcode36-umi108505bins-ubs-3 | Bacteria | p__Acidobacteriota | c__Acidobacteriae | o__Acidobacteriales | f__uncultured | g__uncultured |  |
| 919617913c6e1f899d19b855a1c72230 | Bacteria | p__Proteobacteria | c__Alphaproteobacteria | o__Rhodobacterales | f__Rhodobacteraceae | g__Amaricoccus |  |
| 252c982f1bbc6c815f7ade9039eabd1c | Bacteria | p__Acidobacteriota | c__Vicinamibacteria | o__Vicinamibacterales | f__Vicinamibacteraceae | g__Vicinamibacteraceae |  |
| 04933293 | Bacteria | p__Bacteroidota | c__Bacteroidia | o__Chitinophagales | f__Chitinophagaceae | g__Flavisolibacter | s__Flavisolibacter_tropicus |
| d3745fbe3fbf2ab11421e5a7bd608963 | Bacteria | p__Bacteroidota | c__Bacteroidia | o__Cytophagales | f__Microscillaceae | g__uncultured |  |
| 665bb99471039b4ed40e775d9074a610 | Bacteria | p__Myxococcota | c__Polyangia | o__Haliangiales | f__Haliangiaceae | g__Haliangium |  |
| 589f29f41e922d3d68de91de756ad419 | Bacteria | p__Actinobacteriota | c__Actinobacteria | o__Corynebacteriales | f__Corynebacteriaceae | g__Corynebacterium |  |
| MJ034-1-barcode25-umi29487bins-ubs-3 | Bacteria | p__Proteobacteria | c__Alphaproteobacteria | o__Micropepsales | f__Micropepsaceae | g__uncultured |  |
| eb011e0cc58eb14f205747b8d3784def | Bacteria | p__Actinobacteriota | c__Actinobacteria | o__Corynebacteriales | f__Nocardiaceae | g__Rhodococcus |  |
| 05030291 | Bacteria | p__Proteobacteria | c__Gammaproteobacteria | o__Burkholderiales | f__Burkholderiaceae | g__Burkholderia-Caballeronia-Paraburkholderia |  |
| MJ030-2-barcode58-umi51314bins-ubs-13 | Bacteria | p__Bacteroidota | c__Bacteroidia | o__Flavobacteriales | f__Weeksellaceae | g__Chryseobacterium |  |
| 425fac7c4c1aea8f9d3ab3e253889b2c | Bacteria | p__Proteobacteria | c__Gammaproteobacteria | o__Enterobacterales | f__Enterobacteriaceae |  |  |
| a5e272cc390531346bcef1823c1c366d | Bacteria | p__Bacteroidota | c__Bacteroidia | o__Bacteroidales | f__Bacteroidetes_vadinHA17 | g__Bacteroidetes_vadinHA17 | s__uncultured_bacterium |
| 0ca6fb03789fbb2d327ce509557d8b9b | Bacteria | p__Proteobacteria | c__Gammaproteobacteria | o__Pseudomonadales | f__Moraxellaceae | g__Acinetobacter |  |
| 04950107 | Bacteria | p__Firmicutes | c__Clostridia | o__Lachnospirales | f__Lachnospiraceae |  |  |
| EU216737 | Bacteria | p__Proteobacteria | c__Gammaproteobacteria | o__Enterobacterales |  |  |  |
| 04881419 | Bacteria | p__Actinobacteriota | c__Actinobacteria | o__Corynebacteriales | f__Corynebacteriaceae | g__Corynebacterium |  |
| b3eb5eb5132c3ba431c3e6b82f03cdb0 | Bacteria | p__Planctomycetota | c__Planctomycetes | o__Pirellulales | f__Pirellulaceae | g__Pirellula |  |
| cb003b56b085c69b75a8b4bb1c639cda | Bacteria | p__Proteobacteria | c__Gammaproteobacteria | o__Burkholderiales | f__Burkholderiaceae | g__Burkholderia-Caballeronia-Paraburkholderia | s__Paraburkholderia_caledonica |
| 847f0b696940ac6606fe75bf644f9363 | Bacteria | p__Bacteroidota | c__Bacteroidia | o__Bacteroidales | f__Muribaculaceae | g__Muribaculaceae | s__uncultured_bacterium |
| 67337cea8e2fcae08f0a27b340a2c7fa | Bacteria | p__Acidobacteriota | c__Acidobacteriae | o__Solibacterales | f__Solibacteraceae | g__Candidatus_Solibacter |  |
| 08345679 | Bacteria | p__Proteobacteria | c__Alphaproteobacteria | o__Caulobacterales | f__Caulobacteraceae | g__Brevundimonas |  |
| 8aa22ebb80ccbb3e3bdbb3cfb231de67 | Bacteria | p__Verrucomicrobiota | c__Verrucomicrobiae | o__Pedosphaerales | f__Pedosphaeraceae | g__Pedosphaeraceae |  |
| RS-GCF-019193065.1-NZ-JAHTKT010000007.1 | Bacteria | p__Firmicutes | c__Bacilli | o__Staphylococcales | f__Staphylococcaceae | g__Staphylococcus |  |
| f5728a38270317ddd7f0c66c9e8256a9 | Bacteria | p__Bacteroidota | c__Bacteroidia | o__Flavobacteriales | f__Weeksellaceae | g__Chryseobacterium |  |
| 57780fc6350cc70b460a93e282d7e688 | Bacteria | p__Chloroflexi | c__Anaerolineae |  |  |  |  |
| 82b6ac586f9323c7319e71a883ea40ef | Bacteria | p__Cyanobacteria | c__Cyanobacteriia |  |  |  |  |
| 08180412 | Bacteria | p__Proteobacteria | c__Alphaproteobacteria | o__Rhizobiales | f__Xanthobacteraceae |  |  |
| c29ebf4c0e9b4e3973b23d6042d26540 | Bacteria | p__Proteobacteria | c__Alphaproteobacteria |  |  |  |  |
| MJ030-1-barcode25-umi14141bins-ubs-31 | Bacteria | p__Firmicutes | c__Clostridia | o__Clostridiales | f__Clostridiaceae | g__Clostridium_sensu_stricto_8 | s__uncultured_bacterium |
| 951c23dea4b33df712c058a6e948dee6 | Bacteria | p__Acidobacteriota | c__Holophagae | o__Subgroup_7 | f__Subgroup_7 | g__Subgroup_7 |  |
| 699c03f2f77a817800d072ec9e008d1c | Bacteria | p__Proteobacteria | c__Gammaproteobacteria | o__Burkholderiales | f__Comamonadaceae | g__Curvibacter |  |
| 04994216 | Bacteria | p__Proteobacteria | c__Alphaproteobacteria | o__Caulobacterales | f__Caulobacteraceae | g__Brevundimonas | s__Brevundimonas_olei |
| 02248005 | Bacteria | p__Proteobacteria | c__Alphaproteobacteria |  |  |  |  |
| 04960562 | Bacteria | p__Chloroflexi | c__TK10 | o__TK10 | f__TK10 | g__TK10 |  |
| 5fab0723b868311e83399fbb383cf968 | Bacteria | p__Actinobacteriota | c__Actinobacteria | o__Frankiales | f__Frankiaceae | g__Jatrophihabitans |  |
| 8c40f16c95c0fdcf34fb9355b6ade400 | Bacteria | p__Patescibacteria | c__Saccharimonadia | o__Saccharimonadales | f__Saccharimonadales | g__Saccharimonadales |  |
| f5a49b28765c1980b673a5af893b4bd1 | Bacteria | p__Proteobacteria | c__Gammaproteobacteria | o__Pasteurellales | f__Pasteurellaceae | g__Haemophilus |  |
| MJ033-1-barcode40-umi13707bins-ubs-22 | Bacteria | p__Bacteroidota | c__Bacteroidia | o__Chitinophagales | f__Chitinophagaceae | g__Flavisolibacter | s__uncultured_Flavobacterium |
| 04947252 | Bacteria | p__Actinobacteriota | c__Actinobacteria | o__Actinomycetales | f__Actinomycetaceae | g__Mobiluncus |  |
| 02412114 | Bacteria | p__Proteobacteria | c__Alphaproteobacteria | o__Rhizobiales | f__Methyloligellaceae | g__uncultured | s__metagenome |
| 16ca0ef1898c7c98eb4ab9f26032faf6 | Bacteria | p__Proteobacteria | c__Gammaproteobacteria | o__Burkholderiales | f__Comamonadaceae | g__Aquabacterium |  |
| fad46ad02a9206a18a3fad52d4fdcff0 | Bacteria | p__Acidobacteriota | c__Vicinamibacteria | o__Vicinamibacterales | f__Vicinamibacteraceae | g__Vicinamibacteraceae |  |
| 4c66f66af42026ade7742043f62c2c47 | Bacteria | p__Firmicutes | c__Bacilli | o__Lactobacillales | f__Enterococcaceae | g__Enterococcus |  |
| MJ030-1-barcode25-umi17759bins-ubs-26 | Bacteria | p__Bacteroidota | c__Bacteroidia | o__Chitinophagales | f__Chitinophagaceae | g__Flavisolibacter |  |
| 842d188ff09f679ffd63e6e908c90d47 | Bacteria | p__Proteobacteria | c__Alphaproteobacteria | o__Rhizobiales | f__Beijerinckiaceae |  |  |
| 17847f6c29cea808b1d2b0724cc93259 | Bacteria | p__Proteobacteria | c__Alphaproteobacteria | o__Sphingomonadales | f__Sphingomonadaceae |  |  |
| 1c033420953f5da435538748b79f3f45 | Bacteria | p__Proteobacteria | c__Gammaproteobacteria | o__Cellvibrionales | f__Spongiibacteraceae | g__BD1-7_clade | s__metagenome |
| AJ237756 | Bacteria | p__Firmicutes | c__Clostridia | o__Clostridiales | f__Clostridiaceae | g__Clostridium_sensu_stricto_12 | s__Clostridium_acidisoli |
| cc02e7d17f0843f848a6196094f035d6 | Bacteria | p__Firmicutes | c__Negativicutes | o__Veillonellales-Selenomonadales | f__Veillonellaceae | g__Veillonella |  |
| 1556d23a52ea6f4c6748bd21c1a40d29 | Bacteria | p__Actinobacteriota | c__Acidimicrobiia | o__Microtrichales |  |  |  |
| 82f56c58aaa9a1e4e199434770ca1772 | Bacteria | p__Actinobacteriota | c__Actinobacteria | o__Bifidobacteriales | f__Bifidobacteriaceae | g__Gardnerella | s__Gardnerella_vaginalis |
| 01696526 | Bacteria | p__Proteobacteria | c__Alphaproteobacteria | o__Rhodobacterales | f__Rhodobacteraceae | g__Paracoccus |  |
| 66311bad1ca6436593f1270ab430dd18 | Bacteria | p__Proteobacteria | c__Alphaproteobacteria | o__Paracaedibacterales | f__Paracaedibacteraceae | g__Candidatus_Paracaedibacter |  |
| aead2a4088a06cf61da21b475934acde | Bacteria | p__Verrucomicrobiota | c__Chlamydiae | o__Chlamydiales | f__Simkaniaceae | g__uncultured |  |
| 11162c713b1bd27a14bbf03ffcf43317 | Bacteria | p__Myxococcota | c__Myxococcia | o__Myxococcales | f__Anaeromyxobacteraceae | g__Anaeromyxobacter |  |
| 99954de86968e294ba475165ef375713 | Bacteria | p__Firmicutes | c__Clostridia | o__Clostridiales | f__Clostridiaceae | g__Clostridium_sensu_stricto_1 |  |
| 04967512 | Bacteria | p__Firmicutes | c__Clostridia | o__Oscillospirales | f__Oscillospiraceae | g__uncultured |  |
| 06892301 | Bacteria | p__Verrucomicrobiota | c__Verrucomicrobiae | o__Pedosphaerales | f__Pedosphaeraceae | g__ADurb.Bin063-1 |  |
| 817011923e41972b53faa5b5d06dba83 | Bacteria | p__Actinobacteriota | c__Actinobacteria |  |  |  |  |
| 52fa33abd25c5296086f1936d4cc3847 | Bacteria | p__Actinobacteriota | c__Acidimicrobiia | o__Microtrichales |  |  |  |
| G010592515_1 | Bacteria | p__Proteobacteria | c__Gammaproteobacteria | o__Enterobacterales | f__Enterobacteriaceae |  |  |
| df407f688c2f3e46a3d9b16524e4bdee | Bacteria | p__Proteobacteria | c__Gammaproteobacteria |  |  |  |  |
| 96707d9165379835f52441cb54ce960a | Bacteria | p__Actinobacteriota | c__Actinobacteria | o__Actinomycetales | f__Actinomycetaceae | g__Actinomyces | s__Schaalia_odontolytica |
| 05007908 | Bacteria | p__Proteobacteria | c__Alphaproteobacteria | o__Sphingomonadales | f__Sphingomonadaceae | g__Sphingomonas |  |
| 223cad749a26c6820bebd2d1ee1df85e | Bacteria | p__Actinobacteriota | c__Thermoleophilia | o__Gaiellales | f__uncultured | g__uncultured |  |
| c4404789355461d0067869077b5c6aab | Bacteria | p__Bacteroidota | c__Bacteroidia | o__Chitinophagales | f__Chitinophagaceae | g__Flavisolibacter |  |
| GB-GCA-900756345.1-CAAEJT010000082.1 | Bacteria | p__Firmicutes | c__Clostridia | o__Oscillospirales | f__Oscillospiraceae | g__Flavonifractor |  |
| d7f3da886b945381a8592911f1efe1e6 | Bacteria | p__Actinobacteriota | c__MB-A2-108 | o__MB-A2-108 | f__MB-A2-108 | g__MB-A2-108 |  |
| e4be4d8cb484abed9bcaae01bb122f85 | Bacteria | p__Actinobacteriota | c__Thermoleophilia | o__Solirubrobacterales | f__Solirubrobacteraceae | g__Conexibacter |  |
| 04900675 | Bacteria | p__Proteobacteria | c__Gammaproteobacteria | o__Pseudomonadales | f__Moraxellaceae | g__Acinetobacter |  |
| AF290916 | Bacteria | p__Firmicutes | c__Bacilli | o__Paenibacillales | f__Paenibacillaceae | g__Paenibacillus | s__Paenibacillus_daejeonensis |
| 04957276 | Bacteria | p__Proteobacteria | c__Gammaproteobacteria | o__Burkholderiales | f__Comamonadaceae |  |  |
| b328a66e9777899bf1596c8bd74bed54 | Bacteria | p__Actinobacteriota | c__Actinobacteria | o__Corynebacteriales | f__Mycobacteriaceae | g__Mycobacterium |  |
| G900539105_1 | Bacteria | p__Firmicutes | c__Clostridia | o__Oscillospirales | f__[Eubacterium]_coprostanoligenes_group | g__[Eubacterium]_coprostanoligenes_group | s__gut_metagenome |
| 45d60c7b6a576816c58ecba44d6a4419 | Bacteria | p__Proteobacteria | c__Gammaproteobacteria | o__Legionellales | f__Legionellaceae | g__Legionella |  |
| bfbb49885420245488198d948951513d | Bacteria | p__Bacteroidota | c__Bacteroidia | o__Flavobacteriales | f__NS9_marine_group | g__NS9_marine_group |  |
| c4709f50ee64daee7ce83f442d579f4a | Bacteria | p__Actinobacteriota | c__Acidimicrobiia | o__Microtrichales | f__Iamiaceae | g__Iamia |  |
| 8c9e2fa78a91f632a651170594e8e533 | Bacteria | p__Proteobacteria | c__Gammaproteobacteria | o__Burkholderiales | f__SC-I-84 | g__SC-I-84 |  |
| 05006973 | Bacteria | p__Bacteroidota | c__Bacteroidia | o__Bacteroidales | f__Muribaculaceae | g__Muribaculaceae | s__uncultured_bacterium |
| 2a14fefb7de773cca8e53ee686a9daf5 | Bacteria | p__Actinobacteriota | c__Actinobacteria | o__Corynebacteriales | f__Mycobacteriaceae | g__Mycobacterium |  |
| 11875446 | Bacteria | p__Proteobacteria | c__Alphaproteobacteria | o__Sphingomonadales | f__Sphingomonadaceae | g__Sphingomonas |  |
| 17969627 | Bacteria | p__Chloroflexi | c__Ktedonobacteria | o__Ktedonobacterales | f__Ktedonobacteraceae | g__HSB_OF53-F07 | s__uncultured_bacterium |
| MJ030-1-barcode25-umi18986bins-ubs-27 | Bacteria | p__Firmicutes | c__Bacilli | o__Bacillales | f__Bacillaceae | g__Bacillus |  |
| f2aba33b8cb67fdb8dd6beaebc2a00bc | Bacteria | p__Patescibacteria | c__Saccharimonadia | o__Saccharimonadales | f__Saccharimonadales | g__Saccharimonadales | s__metagenome |
| a0ebc8e36d8fd7baf8be60aa6eeaf14c | Bacteria | p__Firmicutes | c__Bacilli | o__Paenibacillales | f__Paenibacillaceae | g__Paenibacillus |  |
| 13543704 | Bacteria | p__Desulfobacterota | c__Desulfovibrionia | o__Desulfovibrionales | f__Desulfovibrionaceae | g__uncultured | s__uncultured_bacterium |
| 04963750 | Bacteria | p__Firmicutes | c__Bacilli | o__Erysipelotrichales | f__Erysipelotrichaceae | g__Turicibacter | s__uncultured_bacterium |
| 9b955ccb80e05bad6966f4157a0cfa05 | Bacteria | p__Firmicutes | c__Clostridia | o__Clostridia_UCG-014 | f__Clostridia_UCG-014 | g__Clostridia_UCG-014 |  |
| MJ010-1-barcode47-umi132914bins-ubs-4 | Bacteria | p__Proteobacteria | c__Gammaproteobacteria | o__Pseudomonadales | f__Moraxellaceae | g__Acinetobacter |  |
| RS-GCF-001575215.1-NZ-LSRI01000014.1 | Bacteria | p__Actinobacteriota | c__Actinobacteria | o__Corynebacteriales | f__Tsukamurellaceae | g__Tsukamurella |  |
| 05021754 | Bacteria | p__Actinobacteriota | c__Actinobacteria | o__Micrococcales | f__Micrococcaceae | g__Kocuria |  |
| c6634a7191135e05a87b0c6d3dfd4aff | Bacteria | p__Bacteroidota | c__Bacteroidia | o__Sphingobacteriales | f__Sphingobacteriaceae | g__Sphingobacterium |  |
| 5b9c7bc03b4cfc8db889a3b6e0f28aa7 | Bacteria | p__Proteobacteria | c__Gammaproteobacteria | o__Burkholderiales | f__Oxalobacteraceae |  |  |
| 18959115 | Bacteria | p__Proteobacteria | c__Gammaproteobacteria | o__Burkholderiales | f__Nitrosomonadaceae | g__DSSD61 | s__uncultured_bacterium |
| a69e42a60e4f35f3428ceadc8115dc18 | Bacteria | p__Bacteroidota | c__Bacteroidia | o__Flavobacteriales | f__Flavobacteriaceae | g__Flavobacterium |  |
| 04913449 | Bacteria | p__Entotheonellaeota | c__Entotheonellia | o__Entotheonellales | f__Entotheonellaceae | g__Entotheonellaceae | s__uncultured_microorganism |
| MJ030-1-barcode36-umi5807bins-ubs-57 | Bacteria | p__Bacteroidota | c__Bacteroidia | o__Cytophagales | f__Microscillaceae | g__Chryseolinea |  |
| e226bee1472da53db824e39d312ecb47 | Bacteria | p__Actinobacteriota | c__Thermoleophilia | o__Gaiellales | f__uncultured | g__uncultured | s__uncultured_prokaryote |
| c3d5065cfe070963b58fc384c986c7ea | Bacteria | p__Actinobacteriota | c__Actinobacteria | o__Micromonosporales | f__Micromonosporaceae |  |  |
| bc4daff634819c5dffa34df351a89162 | Bacteria | p__Firmicutes | c__Bacilli | o__Staphylococcales | f__Staphylococcaceae | g__Jeotgalicoccus |  |
| 5754591117434d040140af56f011669a | Bacteria | p__Proteobacteria | c__Alphaproteobacteria | o__Rhizobiales |  |  |  |
| RS-GCF-003057675.2-NZ-QBJB02000030.1 | Bacteria | p__Proteobacteria | c__Gammaproteobacteria | o__Enterobacterales | f__Erwiniaceae |  |  |
| c8fdd20cdae8dd2ed4627d6f6a34ac02 | Bacteria | p__Chloroflexi | c__Chloroflexia | o__Thermomicrobiales | f__JG30-KF-CM45 | g__JG30-KF-CM45 | s__uncultured_Chloroflexi |
| f5930efe9014e1f854a89260d1c8bee4 | Bacteria | p__Proteobacteria | c__Gammaproteobacteria | o__Xanthomonadales | f__Xanthomonadaceae |  |  |
| MJ007-2-barcode58-umi3421bins-ubs-128 | Bacteria | p__Proteobacteria | c__Gammaproteobacteria | o__Pseudomonadales | f__Moraxellaceae | g__Acinetobacter |  |
| 08796338 | Bacteria | p__Proteobacteria | c__Gammaproteobacteria | o__Xanthomonadales | f__Xanthomonadaceae | g__Stenotrophomonas |  |
| 19266809 | Bacteria | p__Actinobacteriota | c__Actinobacteria | o__Propionibacteriales | f__Nocardioidaceae | g__Marmoricola |  |
| 1148cbab8157879a7aa02cb4b0c99ec3 | Bacteria | p__Acidobacteriota | c__Acidobacteriae | o__Solibacterales | f__Solibacteraceae | g__Candidatus_Solibacter |  |
| 91bdfb32f9b223eec9c1909fd39f8a3e | Bacteria | p__Acidobacteriota | c__Acidobacteriae | o__Acidobacteriales | f__Acidobacteriaceae_(Subgroup_1) | g__Edaphobacter |  |
| 04963431 | Bacteria | p__Chloroflexi | c__KD4-96 | o__KD4-96 | f__KD4-96 | g__KD4-96 |  |
| 02247660 | Bacteria | p__Proteobacteria | c__Alphaproteobacteria | o__Sphingomonadales | f__Sphingomonadaceae | g__Sphingomonas |  |
| G000372965_1 | Bacteria | p__Proteobacteria | c__Alphaproteobacteria | o__Rhizobiales | f__Rhizobiaceae |  |  |
| Y10999 | Bacteria | p__Actinobacteriota | c__Actinobacteria | o__Corynebacteriales | f__Corynebacteriaceae | g__Corynebacterium |  |
| 4fc76e4f1178b07b922ce9f6be3b97aa | Bacteria | p__Methylomirabilota | c__Methylomirabilia | o__Rokubacteriales | f__WX65 | g__WX65 | s__metagenome |
| 9e28f2fe33f98d0e70345ee5121f9ccf | Bacteria | p__Myxococcota | c__Polyangia | o__Polyangiales | f__Polyangiaceae |  |  |
| fe366c300e64be227d3393a17bc94414 | Bacteria | p__Proteobacteria | c__Alphaproteobacteria | o__Sphingomonadales | f__Sphingomonadaceae | g__Sphingomonas |  |
| bd3732b26815105514f672c7a1aebcf3 | Bacteria | p__Proteobacteria | c__Gammaproteobacteria | o__Burkholderiales | f__Burkholderiaceae | g__Burkholderia-Caballeronia-Paraburkholderia | s__Paraburkholderia_caledonica |
| 11098f3d308d340895b14b037feb7d03 | Bacteria | p__Proteobacteria | c__Gammaproteobacteria | o__Burkholderiales | f__Sutterellaceae | g__AAP99 | s__uncultured_bacterium |
| a1ac8cba610554eb1f3b438800888672 | Bacteria | p__Proteobacteria | c__Gammaproteobacteria | o__Burkholderiales | f__Comamonadaceae | g__Comamonas |  |
| DQ512860 | Bacteria | p__Actinobacteriota | c__Actinobacteria | o__Micrococcales | f__Intrasporangiaceae | g__Ornithinimicrobium |  |
| 8d7cf3cdef913017d25eda562289a1cf | Bacteria | p__Proteobacteria | c__Gammaproteobacteria | o__Burkholderiales | f__Burkholderiaceae | g__Burkholderia-Caballeronia-Paraburkholderia |  |
| KP993300 | Bacteria | p__Proteobacteria | c__Alphaproteobacteria | o__Rhizobiales | f__Pleomorphomonadaceae | g__Chthonobacter |  |
| G001896575_4 | Bacteria | p__Thermotogota | c__Thermotogae | o__Kosmotogales | f__Kosmotogaceae | g__Mesotoga | s__uncultured_bacterium |
| 04988902 | Bacteria | p__Proteobacteria | c__Gammaproteobacteria | o__Pseudomonadales | f__Pseudomonadaceae | g__Pseudomonas |  |
| G010020825_1 | Bacteria | p__Proteobacteria | c__Gammaproteobacteria | o__Burkholderiales | f__Comamonadaceae |  |  |
| 2d3ec7cdff0b20578850e1b6fcf4312b | Bacteria | p__Bacteroidota | c__Bacteroidia | o__Flavobacteriales | f__Weeksellaceae | g__Chryseobacterium |  |
| G002705755_1 | Bacteria | p__Actinobacteriota | c__Actinobacteria | o__Micrococcales | f__Microbacteriaceae | g__Microbacterium |  |
| 60a1d0ea0fcc7582290eaf2d7e73d5cf | Bacteria | p__Actinobacteriota | c__MB-A2-108 | o__MB-A2-108 | f__MB-A2-108 | g__MB-A2-108 | s__uncultured_actinobacterium |
| 17942836 | Bacteria | p__Actinobacteriota | c__Acidimicrobiia | o__uncultured | f__uncultured | g__uncultured |  |
| b8513f28482a3e14494783234fc23d80 | Bacteria | p__Chloroflexi | c__AD3 | o__AD3 | f__AD3 | g__AD3 | s__uncultured_bacterium |
| MJ010-2-barcode64-umi70547bins-ubs-7 | Bacteria | p__Proteobacteria | c__Gammaproteobacteria | o__Pseudomonadales | f__Moraxellaceae | g__Acinetobacter |  |
| MJ010-1-barcode26-umi116423bins-ubs-5 | Bacteria | p__Proteobacteria | c__Gammaproteobacteria | o__Steroidobacterales | f__Steroidobacteraceae | g__Steroidobacter |  |
| FJ210816 | Bacteria | p__Proteobacteria | c__Gammaproteobacteria | o__Burkholderiales | f__Burkholderiaceae | g__Burkholderia-Caballeronia-Paraburkholderia |  |
| RS-GCF-001157605.1-NZ-CKQU01000080.1 | Bacteria | p__Firmicutes | c__Bacilli | o__Bacillales | f__Bacillaceae | g__Bacillus |  |
| 5d3fc456b3f784c4a216534df0f71ee4 | Bacteria | p__Acidobacteriota | c__Blastocatellia | o__11-24 | f__11-24 | g__11-24 | s__uncultured_Acidobacteria |
| 04901265 | Bacteria | p__Proteobacteria | c__Gammaproteobacteria | o__Gammaproteobacteria_Incertae_Sedis | f__Unknown_Family | g__Acidibacter |  |
| 8a2202aec44f7cb13c7c30b47a27e197 | Bacteria | p__Actinobacteriota | c__Actinobacteria | o__Micrococcales | f__Micrococcaceae |  |  |
| 03663820 | Bacteria | p__Proteobacteria | c__Alphaproteobacteria |  |  |  |  |
| 1cdbe45945bf58023417a215d8b0c0b2 | Bacteria | p__Bacteroidota | c__Bacteroidia | o__Chitinophagales | f__Chitinophagaceae |  |  |
| RS-GCF-002915475.1-NZ-PQGK01000017.1 | Bacteria | p__Proteobacteria | c__Gammaproteobacteria | o__Enterobacterales | f__Yersiniaceae | g__Serratia |  |
| RS-GCF-900101575.1-NZ-FMUZ01000094.1 | Bacteria | p__Proteobacteria | c__Gammaproteobacteria | o__Pseudomonadales | f__Pseudomonadaceae | g__Pseudomonas |  |
| MJ031-1-barcode30-umi33310bins-ubs-6 | Bacteria | p__Proteobacteria | c__Gammaproteobacteria | o__Gammaproteobacteria_Incertae_Sedis | f__Unknown_Family | g__Acidibacter |  |
| ea7c21b441652cacf378afca68514309 | Bacteria | p__Firmicutes | c__Clostridia | o__Lachnospirales | f__Lachnospiraceae |  |  |
| 1f33016a69f47e174d831d9456d34f78 | Bacteria | p__Proteobacteria | c__Gammaproteobacteria | o__Pseudomonadales | f__Moraxellaceae | g__Acinetobacter |  |
| f4a6684888f542351e0deb669706f159 | Bacteria | p__Chloroflexi | c__Anaerolineae | o__1-20 | f__1-20 | g__1-20 |  |
| ab15bd9e46897f85a738f8646706071b | Bacteria | p__MBNT15 | c__MBNT15 | o__MBNT15 | f__MBNT15 | g__MBNT15 |  |
| 9f1ed0f8a205e265ce9f13e3062592a8 | Bacteria | p__Proteobacteria | c__Gammaproteobacteria | o__Cellvibrionales | f__Cellvibrionaceae | g__Cellvibrio | s__uncultured_compost |
| 46cb19f6c70c5ce656c163be6f32f4a4 | Bacteria | p__Actinobacteriota | c__Actinobacteria | o__Corynebacteriales | f__Corynebacteriaceae | g__Corynebacterium |  |
| 7c85fd8ccf95d1afbfd3c70ab0235aaf | Bacteria | p__Proteobacteria | c__Alphaproteobacteria | o__Tistrellales | f__Geminicoccaceae | g__Candidatus_Alysiosphaera |  |
| 915dd1926e42260cf0d5032e0895c66d | Bacteria | p__Bacteroidota | c__Bacteroidia | o__Bacteroidales | f__Muribaculaceae | g__Muribaculaceae |  |
| 7d9f5001d4164a260ae5e6da4ce06961 | Bacteria | p__Actinobacteriota | c__Thermoleophilia | o__Gaiellales | f__uncultured | g__uncultured |  |
| 04933327 | Bacteria | p__Bacteroidota | c__Bacteroidia | o__Chitinophagales | f__Chitinophagaceae |  |  |
| 9b689b7284f2ff0ed0147991f7d337ab | Bacteria | p__Firmicutes | c__Bacilli | o__Alicyclobacillales | f__Alicyclobacillaceae | g__Tumebacillus |  |
| MJ020-1-barcode31-umi105044bins-ubs-3 | Bacteria | p__Acidobacteriota | c__Blastocatellia | o__Pyrinomonadales | f__Pyrinomonadaceae | g__RB41 |  |
| 1c487e75218d25bc728a13e3b2f7bdb3 | Bacteria | p__Firmicutes | c__Bacilli | o__Bacillales | f__Bacillaceae | g__Geobacillus |  |
| 14337506 | Bacteria | p__Acidobacteriota | c__Vicinamibacteria | o__Vicinamibacterales | f__uncultured | g__uncultured |  |
| G001690535_4 | Bacteria | p__Proteobacteria | c__Gammaproteobacteria | o__Orbales | f__Orbaceae | g__Gilliamella | s__uncultured_gamma |
| 2523c01c2dc31e69a7f868f46e6fdd94 | Bacteria | p__Bacteroidota | c__Bacteroidia | o__Flavobacteriales | f__Weeksellaceae | g__Cloacibacterium |  |
| 3bbbbc143db7515ced5f38ef898148f2 | Bacteria | p__Actinobacteriota | c__Actinobacteria | o__Streptomycetales | f__Streptomycetaceae | g__Streptomyces |  |
| 04993577 | Bacteria | p__Proteobacteria | c__Alphaproteobacteria | o__Rhizobiales | f__Xanthobacteraceae | g__Bradyrhizobium |  |
| MJ034-2-barcode60-umi3529bins-ubs-102 | Bacteria | p__Bacteroidota | c__Bacteroidia | o__Sphingobacteriales | f__AKYH767 | g__AKYH767 |  |
| d85b266dbc115925f63d46b513e61987 | Bacteria | p__Firmicutes | c__Clostridia | o__Oscillospirales | f__Ruminococcaceae | g__Ruminococcus | s__Ruminococcus_callidus |
| 04941297 | Bacteria | p__Acidobacteriota | c__Vicinamibacteria | o__Vicinamibacterales | f__uncultured | g__uncultured |  |
| 02244827 | Bacteria | p__Actinobacteriota | c__Actinobacteria | o__Corynebacteriales | f__Corynebacteriaceae | g__Corynebacterium |  |
| 401975bf3f1790f7a0e047b5babdd430 | Bacteria | p__Bacteroidota | c__Bacteroidia | o__Bacteroidales | f__Marinifilaceae | g__Odoribacter | s__uncultured_bacterium |
| MJ033-1-barcode36-umi41636bins-ubs-3 | Bacteria | p__Proteobacteria | c__Alphaproteobacteria | o__Rhizobiales | f__Rhizobiales_Incertae_Sedis | g__Nordella |  |
| 02858227 | Bacteria | p__Chloroflexi | c__Chloroflexia | o__Chloroflexales | f__Roseiflexaceae | g__uncultured | s__uncultured_bacterium |
| 12cddc4b4bbe9911c29cfcff5d4db10d | Bacteria | p__Bacteroidota | c__Bacteroidia | o__Chitinophagales | f__Chitinophagaceae |  |  |
| 04908756 | Bacteria | p__Proteobacteria | c__Alphaproteobacteria | o__Rhizobiales | f__Rhizobiaceae | g__Aureimonas |  |
| 4d8861346bb81c52cbbdf1222032cc2d | Bacteria | p__Firmicutes | c__Bacilli | o__Lactobacillales | f__Enterococcaceae | g__Enterococcus |  |
| 05038049 | Bacteria | p__Firmicutes | c__Clostridia | o__Oscillospirales | f__Oscillospiraceae |  |  |
| AB681166 | Bacteria | p__Firmicutes | c__Bacilli | o__Entomoplasmatales | f__Spiroplasmataceae | g__Spiroplasma |  |
| 19707248 | Bacteria | p__Firmicutes | c__Bacilli | o__Erysipelotrichales | f__Erysipelotrichaceae | g__Ileibacterium | s__Ileibacterium_valens |
| 94f6c7c603c14665fca0452d6242319a | Bacteria | p__Firmicutes | c__Clostridia | o__Lachnospirales | f__Lachnospiraceae | g__Roseburia |  |
| 115752ebc656bc1be93bf6790c8f37da | Bacteria | p__Chloroflexi | c__JG30-KF-CM66 | o__JG30-KF-CM66 | f__JG30-KF-CM66 | g__JG30-KF-CM66 | s__uncultured_Armatimonadetes |
| c5249c5b6d12c973f51fc7210b5ae07b | Bacteria | p__Campilobacterota | c__Campylobacteria | o__Campylobacterales | f__Helicobacteraceae | g__Helicobacter |  |
| e3283a91dff0ebdb6024806ced6a41d2 | Bacteria | p__Proteobacteria | c__Alphaproteobacteria | o__Rhizobiales | f__Rhizobiaceae |  |  |
| 1b090723d28cf0d593174191a64d8a88 | Bacteria | p__Proteobacteria | c__Gammaproteobacteria | o__Burkholderiales | f__Comamonadaceae | g__Aquabacterium |  |
| GB-GCA-014647715.1-BMPS01000099.1 | Bacteria | p__Proteobacteria | c__Gammaproteobacteria | o__Pseudomonadales | f__Moraxellaceae | g__Enhydrobacter |  |
| 029c1565deaaf71f9aef78bb82960d30 | Bacteria | p__Actinobacteriota | c__Actinobacteria | o__Micrococcales | f__Intrasporangiaceae |  |  |
| 02411445 | Bacteria | p__Proteobacteria | c__Alphaproteobacteria | o__Sphingomonadales | f__Sphingomonadaceae |  |  |
| cb5f6a9bc28db37de1121cc01a315aa0 | Bacteria | p__Chloroflexi | c__KD4-96 | o__KD4-96 | f__KD4-96 | g__KD4-96 |  |
| 5f92356040d2979ee7849e29fcf49ff4 | Bacteria | p__Chloroflexi | c__Chloroflexia | o__Thermomicrobiales | f__AKYG1722 | g__AKYG1722 | s__uncultured_soil |
| 04968099 | Bacteria | p__Firmicutes | c__Bacilli | o__Lactobacillales | f__Enterococcaceae | g__Enterococcus |  |
| MJ034-1-barcode46-umi17880bins-ubs-13 | Bacteria | p__Bacteroidota | c__Bacteroidia | o__Chitinophagales | f__Chitinophagaceae |  |  |
| f3c49404be00e0ca1807f58e9d8e0db2 | Bacteria | p__Proteobacteria | c__Gammaproteobacteria | o__Enterobacterales | f__Enterobacteriaceae |  |  |
| X76740 | Bacteria | p__Firmicutes | c__Clostridia | o__Clostridiales | f__Clostridiaceae | g__Clostridium_sensu_stricto_3 |  |
| RS-GCF-004402745.1-NZ-SOFX01000009.1 | Bacteria | p__Actinobacteriota | c__Actinobacteria | o__Micrococcales | f__Microbacteriaceae |  |  |
| 08618317 | Bacteria | p__Proteobacteria | c__Alphaproteobacteria | o__Rhizobiales | f__Rhizobiaceae | g__Falsochrobactrum |  |
| 02863038 | Bacteria | p__Chloroflexi | c__Chloroflexia | o__Chloroflexales | f__Roseiflexaceae | g__uncultured | s__uncultured_Chloroflexi |
| 3a30dc958f6fb35bbf5f6f8884fa091d | Bacteria | p__Actinobacteriota | c__Actinobacteria | o__Kineosporiales | f__Kineosporiaceae |  |  |
| 8e95f8b81a5689fce9e4cdf311359e4e | Bacteria | p__Acidobacteriota | c__Vicinamibacteria | o__Subgroup_17 | f__Subgroup_17 | g__Subgroup_17 |  |
| 04522228 | Bacteria | p__Firmicutes | c__Bacilli | o__Staphylococcales | f__Staphylococcaceae | g__Staphylococcus |  |
| 61782aeb26e69cae35ad32d680065901 | Bacteria | p__Acidobacteriota | c__Acidobacteriae | o__Acidobacteriales | f__Koribacteraceae | g__Candidatus_Koribacter |  |
| RS-GCF-000584015.1-NZ-JFFH02000116.1 | Bacteria | p__Proteobacteria | c__Gammaproteobacteria | o__Pseudomonadales | f__Moraxellaceae | g__Acinetobacter |  |
| f3cc85c5ec8a5147989e131002d3405e | Bacteria | p__Chloroflexi | c__Anaerolineae | o__SBR1031 | f__A4b | g__A4b |  |
| 05024613 | Bacteria | p__Firmicutes | c__Clostridia | o__Lachnospirales | f__Lachnospiraceae |  |  |
| 05021386 | Bacteria | p__Actinobacteriota | c__Actinobacteria | o__Micrococcales | f__Micrococcaceae | g__Paenarthrobacter |  |
| d4cb2856bb5aa0a0b37bf100a5646bd6 | Bacteria | p__Deinococcota | c__Deinococci | o__Thermales | f__Thermaceae | g__Thermus |  |
| 18896753 | Bacteria | p__Proteobacteria | c__Gammaproteobacteria | o__Burkholderiales | f__Sutterellaceae | g__uncultured | s__beta_proteobacterium |
| 9abef35bda36f39e6a405a1dee304f37 | Bacteria | p__Gemmatimonadota | c__Gemmatimonadetes | o__Gemmatimonadales | f__Gemmatimonadaceae |  |  |
| b62131d44bbfd284e476550a15559554 | Bacteria | p__Proteobacteria | c__Gammaproteobacteria | o__Xanthomonadales | f__Xanthomonadaceae |  |  |
| 14585624 | Bacteria | p__Firmicutes | c__Clostridia | o__Peptostreptococcales-Tissierellales | f__Peptostreptococcales-Tissierellales | g__Anaerococcus |  |
| 664b30b08a1f3836380baae0117ac950 | Bacteria | p__Proteobacteria | c__Gammaproteobacteria | o__Burkholderiales | f__Nitrosomonadaceae | g__Ellin6067 |  |
| 6eded10887e2a267051aa39517a89ba7 | Bacteria | p__Chloroflexi | c__Chloroflexia | o__Thermomicrobiales | f__JG30-KF-CM45 | g__JG30-KF-CM45 |  |
| 0ad0de079ab45a5f0d8ec6c08bdefaac | Bacteria | p__Actinobacteriota | c__Actinobacteria | o__Frankiales | f__Nakamurellaceae | g__Nakamurella |  |
| 05035259 | Bacteria | p__Firmicutes | c__Bacilli | o__Bacillales | f__Bacillaceae | g__Bacillus |  |
| 751cc91d2d1881382a836f27a8ae8e8f | Bacteria | p__Firmicutes | c__Bacilli | o__Paenibacillales | f__Paenibacillaceae | g__Saccharibacillus |  |
| 08441389 | Bacteria | p__Proteobacteria | c__Alphaproteobacteria | o__Rhizobiales | f__Beijerinckiaceae | g__Microvirga |  |
| 3475287ebd453ecdd0e102d5440e47de | Bacteria | p__Actinobacteriota | c__Thermoleophilia | o__Solirubrobacterales | f__67-14 | g__67-14 |  |
| 18768541 | Bacteria | p__Bacteroidota | c__Bacteroidia | o__Cytophagales | f__Cytophagaceae | g__Siphonobacter | s__Siphonobacter_sp. |
| 03178354 | Bacteria | p__Planctomycetota | c__Phycisphaerae | o__Tepidisphaerales | f__WD2101_soil_group | g__WD2101_soil_group | s__uncultured_bacterium |
| 04946296 | Bacteria | p__Actinobacteriota | c__Actinobacteria | o__Micrococcales | f__Sanguibacteraceae | g__Sanguibacter |  |
| 04950539 | Bacteria | p__Firmicutes | c__Clostridia | o__Lachnospirales | f__Lachnospiraceae |  |  |
| MF405104 | Bacteria | p__Proteobacteria | c__Alphaproteobacteria | o__Sphingomonadales | f__Sphingomonadaceae | g__Sphingopyxis |  |
| 86fd7791bc95b9b50f3e365ab3c4a541 | Bacteria | p__Proteobacteria | c__Alphaproteobacteria | o__Acetobacterales | f__Acetobacteraceae |  |  |
| 7be244db0895a1c4d2ee97e3096fffa2 | Bacteria | p__Actinobacteriota | c__Actinobacteria | o__Micrococcales | f__Micrococcaceae |  |  |
| 04950642 | Bacteria | p__Firmicutes | c__Clostridia | o__Lachnospirales | f__Lachnospiraceae | g__Acetatifactor |  |
| 8318090db8c2347f08278ef19ffa3a61 | Bacteria | p__Actinobacteriota | c__Acidimicrobiia | o__Microtrichales | f__Microtrichaceae | g__uncultured |  |
| RS-GCF-002198715.1-NZ-MWLK01000036.1 | Bacteria | p__Actinobacteriota | c__Actinobacteria | o__Micrococcales | f__Intrasporangiaceae |  |  |
| 9461bdcbf37d6bf798c847a5baf7de17 | Bacteria | p__Actinobacteriota | c__Acidimicrobiia | o__Microtrichales |  |  |  |
| 59d5dc723507031b2a7811e15d8a3cf9 | Bacteria | p__Acidobacteriota | c__Blastocatellia | o__Pyrinomonadales | f__Pyrinomonadaceae | g__RB41 |  |
| 02360501 | Bacteria | p__Proteobacteria | c__Alphaproteobacteria | o__Rhizobiales | f__Xanthobacteraceae | g__uncultured |  |
| d72ea9b3a890a07feb6ade044908319c | Bacteria | p__Proteobacteria | c__Gammaproteobacteria | o__Burkholderiales | f__Comamonadaceae |  |  |
| 18813780 | Bacteria | p__Proteobacteria | c__Gammaproteobacteria | o__Burkholderiales | f__TRA3-20 | g__TRA3-20 |  |
| db6606629269c22ce8abcc6c68699744 | Bacteria | p__Proteobacteria | c__Gammaproteobacteria | o__Burkholderiales | f__SC-I-84 | g__SC-I-84 |  |
| 02247939 | Bacteria | p__Actinobacteriota | c__Actinobacteria | o__Propionibacteriales | f__Nocardioidaceae | g__Nocardioides |  |
| MJ010-1-barcode26-umi62720bins-ubs-12 | Bacteria | p__Firmicutes | c__Clostridia | o__Clostridia | f__Hungateiclostridiaceae | g__Ruminiclostridium |  |
| a538cf23fdd63ec381f6509a15442e91 | Bacteria | p__Proteobacteria | c__Alphaproteobacteria | o__Rhizobiales | f__A0839 | g__A0839 |  |
| MJ034-1-barcode40-umi27418bins-ubs-4 | Bacteria | p__Acidobacteriota | c__Acidobacteriae |  |  |  |  |
| KU573975 | Bacteria | p__Firmicutes | c__Bacilli | o__Paenibacillales | f__Paenibacillaceae | g__Paenibacillus |  |
| 391609cbad7c6a6235213e9a3ccfb803 | Bacteria | p__Proteobacteria | c__Gammaproteobacteria | o__Pseudomonadales | f__Moraxellaceae | g__Acinetobacter |  |
| 10577717 | Bacteria | p__Bacteroidota | c__Bacteroidia | o__Sphingobacteriales | f__Sphingobacteriaceae | g__Sphingobacterium |  |
| 2f955017503cba2d2b2f4148c420ff33 | Bacteria | p__Proteobacteria | c__Gammaproteobacteria |  |  |  |  |
| c88b39006d8960d6459089a779468846 | Bacteria | p__Proteobacteria | c__Gammaproteobacteria | o__Burkholderiales | f__Comamonadaceae |  |  |
| MJ005-barcode41-umi54088bins-ubs-6 | Bacteria | p__Firmicutes | c__Clostridia | o__Oscillospirales | f__Ruminococcaceae | g__Incertae_Sedis | s__uncultured_organism |
| 9a5c71e072292b3a1a215d01c1c11c90 | Bacteria | p__Proteobacteria | c__Gammaproteobacteria | o__Burkholderiales | f__Comamonadaceae | g__Pelomonas |  |
| 124441f4ca5f5d7523a6f103b877c17c | Bacteria | p__Firmicutes | c__Clostridia | o__Christensenellales | f__Christensenellaceae | g__Christensenellaceae_R-7_group |  |
| 4488fdd54191c2c83ded3049f2c16dd3 | Bacteria | p__Firmicutes | c__Bacilli | o__Erysipelotrichales | f__Erysipelotrichaceae | g__Turicibacter |  |
| 04321221 | Bacteria | p__Acidobacteriota | c__Acidobacteriae | o__Subgroup_2 | f__Subgroup_2 | g__Subgroup_2 | s__uncultured_forest |
| MJ030-2-barcode60-umi16330bins-ubs-35 | Bacteria | p__Firmicutes | c__Clostridia | o__Clostridiales | f__Clostridiaceae | g__Clostridium_sensu_stricto_5 | s__uncultured_bacterium |
| 394ef0ada3b295ef43c4515d73e3bfa3 | Bacteria | p__Proteobacteria | c__Alphaproteobacteria | o__Caulobacterales | f__Hyphomonadaceae | g__SWB02 | s__uncultured_bacterium |
| 9eafb32029eccb81f691437a58b3b402 | Bacteria | p__Actinobacteriota | c__Thermoleophilia | o__Gaiellales | f__uncultured | g__uncultured | s__uncultured_microorganism |
| 17973404 | Bacteria | p__Chloroflexi | c__Ktedonobacteria | o__Ktedonobacterales | f__Ktedonobacteraceae | g__1921-2 | s__uncultured_bacterium |
| 04875862 | Bacteria | p__Proteobacteria | c__Gammaproteobacteria | o__Pseudomonadales | f__Pseudomonadaceae | g__Pseudomonas |  |
| 8ef1889faf686e8f8bec88fb50c47a0d | Bacteria | p__Proteobacteria | c__Alphaproteobacteria | o__Sphingomonadales | f__Sphingomonadaceae |  |  |
| c9c9c5b3d63304477b4aaf4bc6fb64d3 | Bacteria | p__Firmicutes | c__Bacilli | o__Erysipelotrichales | f__Erysipelatoclostridiaceae | g__Erysipelotrichaceae_UCG-003 |  |
| RS-GCF-013180515.1-NZ-PYWU01000001.1--4 | Bacteria | p__Proteobacteria | c__Gammaproteobacteria | o__Enterobacterales | f__Enterobacteriaceae |  |  |
| 19706794 | Bacteria | p__Firmicutes | c__Bacilli | o__Erysipelotrichales | f__Erysipelotrichaceae | g__Ileibacterium | s__Ileibacterium_valens |
| 11179138 | Bacteria | p__Bacteroidota | c__Bacteroidia | o__Bacteroidales | f__Marinifilaceae | g__Odoribacter | s__uncultured_bacterium |
| 8ec1d65685f97333a7694208daf103bb | Bacteria | p__Firmicutes | c__Bacilli | o__Erysipelotrichales | f__Erysipelotrichaceae | g__Ileibacterium | s__Ileibacterium_valens |
| 3425acadb59192d7b1f19b7e7df6482c | Bacteria | p__Proteobacteria | c__Alphaproteobacteria | o__Elsterales | f__uncultured | g__uncultured |  |
| MJ020-1-barcode45-umi90725bins-ubs-4 | Bacteria | p__Bacteroidota | c__Bacteroidia | o__Chitinophagales | f__Chitinophagaceae | g__Terrimonas |  |
| 17246167 | Bacteria | p__Firmicutes | c__Clostridia | o__Clostridia_vadinBB60_group | f__Clostridia_vadinBB60_group | g__Clostridia_vadinBB60_group |  |
| 6330a23b1900a2bbb08c6272d77260aa | Bacteria | p__Acidobacteriota | c__Acidobacteriae | o__Solibacterales | f__Solibacteraceae | g__Candidatus_Solibacter |  |
| 457abc9b19bbad18f7387c6513ed7b45 | Bacteria | p__Proteobacteria | c__Gammaproteobacteria | o__Burkholderiales |  |  |  |
| MJ031-1-barcode27-umi37887bins-ubs-4 | Bacteria | p__Proteobacteria | c__Alphaproteobacteria | o__Rhodobacterales | f__Rhodobacteraceae |  |  |
| 19753855 | Bacteria | p__Firmicutes | c__Clostridia | o__Oscillospirales | f__UCG-010 | g__UCG-010 | s__uncultured_organism |
| 04956049 | Bacteria | p__Actinobacteriota | c__Thermoleophilia | o__Gaiellales | f__uncultured | g__uncultured |  |
| GB-GCA-017307195.1-JAFKHL010000041.1 | Bacteria | p__Proteobacteria | c__Gammaproteobacteria | o__Burkholderiales | f__Oxalobacteraceae | g__Herbaspirillum |  |
| 0033103e626b303bb89427c825acb229 | Bacteria | p__Proteobacteria | c__Gammaproteobacteria | o__Burkholderiales | f__Nitrosomonadaceae | g__Ellin6067 |  |
| d6b14b3bed2785e8079dd7968e74b606 | Bacteria | p__Actinobacteriota | c__Coriobacteriia | o__Coriobacteriales | f__Coriobacteriaceae | g__Collinsella |  |
| ef6b8ddcccdd7b28a01d0c6eae298d03 | Bacteria | p__Myxococcota | c__bacteriap25 | o__bacteriap25 | f__bacteriap25 | g__bacteriap25 | s__uncultured_Desulfovibrionales |
| MJ006-1-barcode29-umi157218bins-ubs-4 | Bacteria | p__Bacteroidota | c__Bacteroidia | o__Bacteroidales | f__Bacteroidaceae | g__Bacteroides |  |
| de0c25f96da3386d1141e6a7fee89c53 | Bacteria | p__Actinobacteriota | c__Actinobacteria | o__Propionibacteriales | f__Nocardioidaceae | g__Aeromicrobium |  |
| b6525f75a32cf55b5ee8c36b8650e6d3 | Bacteria | p__Acidobacteriota | c__Blastocatellia | o__Pyrinomonadales | f__Pyrinomonadaceae | g__RB41 | s__uncultured_Acidobacteriaceae |
| aae5c421c03a57de825ed4793b8145ef | Bacteria | p__Proteobacteria | c__Alphaproteobacteria | o__Rhizobiales | f__Xanthobacteraceae | g__uncultured |  |
| KM073966 | Bacteria | p__Actinobacteriota | c__Actinobacteria | o__Micrococcales | f__Dermabacteraceae | g__Brachybacterium |  |
| MJ030-2-barcode60-umi2334bins-ubs-95 | Bacteria | p__Firmicutes | c__Clostridia | o__Peptostreptococcales-Tissierellales | f__Peptostreptococcaceae |  |  |
| 04878365 | Bacteria | p__Actinobacteriota | c__Actinobacteria | o__Corynebacteriales | f__Corynebacteriaceae | g__Corynebacterium |  |
| c143332cf64928c9a5dbe7449200ed38 | Bacteria | p__Firmicutes | c__Clostridia | o__Lachnospirales | f__Lachnospiraceae | g__Tyzzerella | s__uncultured_Clostridium |
| 93f28580630d64748e2f776d59d060a1 | Bacteria | p__Actinobacteriota | c__Actinobacteria | o__Streptomycetales | f__Streptomycetaceae |  |  |
| 77406dcd4de974c6a4dab70efa6da054 | Bacteria | p__Actinobacteriota | c__Actinobacteria | o__Corynebacteriales | f__Corynebacteriaceae | g__Corynebacterium |  |
| 1904e3806b203279515d96680a32ef4e | Bacteria | p__Cyanobacteria | c__Cyanobacteriia | o__Oxyphotobacteria_Incertae_Sedis | f__Unknown_Family | g__uncultured |  |
| aa99baa979b419f70a00667d3d1e0c95 | Bacteria | p__Proteobacteria | c__Gammaproteobacteria |  |  |  |  |
| adfd77852dd2211415a0243ea8b747d7 | Bacteria | p__Bacteroidota | c__Bacteroidia | o__Bacteroidales | f__Marinifilaceae | g__Odoribacter | s__unidentified |
| MJ032-2-barcode73-umi37217bins-ubs-15 | Bacteria | p__Proteobacteria | c__Gammaproteobacteria | o__Xanthomonadales | f__Xanthomonadaceae | g__Stenotrophomonas |  |
| db374f2b15c547173da79eed47a72a4d | Bacteria | p__Acidobacteriota | c__Vicinamibacteria | o__Vicinamibacterales | f__uncultured | g__uncultured |  |
| 36eb466a1fe71525de356088ac947753 | Bacteria | p__Bacteroidota | c__Bacteroidia | o__Flavobacteriales | f__Weeksellaceae | g__Weeksella | s__uncultured_Flavobacterium |
| c19c249c9c2f734de78d2e2c7d8b3287 | Bacteria | p__Actinobacteriota | c__Acidimicrobiia | o__uncultured | f__uncultured | g__uncultured | s__uncultured_Acidimicrobiia |
| 993b6ea70f812de969adcf2992805ea5 | Bacteria | p__Proteobacteria | c__Alphaproteobacteria | o__Rhizobiales | f__Hyphomicrobiaceae |  |  |
| MJ032-2-barcode60-umi86526bins-ubs-4 | Bacteria | p__Proteobacteria | c__Gammaproteobacteria | o__Xanthomonadales | f__Xanthomonadaceae | g__Stenotrophomonas |  |
| 538474c3ee89db1bf7ddea7a2a6980bf | Bacteria | p__Bacteroidota | c__Bacteroidia | o__Bacteroidales | f__Rikenellaceae | g__Alistipes |  |
| 25f7d862a222c68b4d13ff92af0f1940 | Bacteria | p__Proteobacteria | c__Gammaproteobacteria | o__Enterobacterales |  |  |  |
| 37a4c091300a494a24f8b1277d3c1d23 | Bacteria | p__Proteobacteria | c__Gammaproteobacteria | o__Burkholderiales | f__Comamonadaceae |  |  |
| 17ae0246a83e56f44415bc3537ad5472 | Bacteria | p__Proteobacteria | c__Alphaproteobacteria | o__Sphingomonadales | f__Sphingomonadaceae |  |  |
| 4da321d099013a46b86efad32b887282 | Bacteria | p__Bacteroidota | c__Bacteroidia | o__Sphingobacteriales | f__Sphingobacteriaceae | g__Sphingobacterium |  |
| 2d4bd0fe388826d08e6fa0fc15bc5dab | Bacteria | p__Proteobacteria | c__Alphaproteobacteria | o__Azospirillales | f__Azospirillaceae | g__Skermanella |  |
| RS-GCF-009013695.1-NZ-BKXR01000133.1 | Bacteria | p__Proteobacteria | c__Gammaproteobacteria | o__Pseudomonadales | f__Moraxellaceae | g__Acinetobacter |  |
| DQ342322 | Bacteria | p__Proteobacteria | c__Alphaproteobacteria | o__Rhodobacterales | f__Rhodobacteraceae | g__Rhodobacter |  |
| MJ010-2-barcode64-umi119672bins-ubs-4 | Bacteria | p__Proteobacteria | c__Gammaproteobacteria | o__Burkholderiales | f__Comamonadaceae | g__Pseudorhodoferax |  |
| e8d85bf1119f3a2b2bf01b2a9f19074a | Bacteria | p__Chloroflexi | c__Ktedonobacteria | o__Ktedonobacterales | f__JG30-KF-AS9 | g__JG30-KF-AS9 | s__uncultured_bacterium |
| MJ032-2-barcode74-umi45491bins-ubs-11 | Bacteria | p__Proteobacteria | c__Gammaproteobacteria | o__Pseudomonadales | f__Moraxellaceae | g__Acinetobacter |  |
| 0469e63b871657ff35060df39b1bc2f4 | Bacteria | p__Proteobacteria | c__Gammaproteobacteria | o__Pseudomonadales | f__Moraxellaceae | g__Acinetobacter |  |
| 04960731 | Bacteria | p__Actinobacteriota | c__Actinobacteria | o__Corynebacteriales | f__Nocardiaceae | g__Gordonia |  |
| 1d71b68166e45dd709057033c67c4077 | Bacteria | p__Bacteroidota | c__Bacteroidia | o__Flavobacteriales | f__Flavobacteriaceae | g__Flavobacterium |  |
| 04963863 | Bacteria | p__Firmicutes | c__Bacilli | o__Bacillales | f__Bacillaceae | g__Bacillus |  |
| G900538515_1 | Bacteria | p__Actinobacteriota | c__Coriobacteriia | o__Coriobacteriales | f__Coriobacteriales_Incertae_Sedis | g__uncultured | s__gut_metagenome |
| 10554137 | Bacteria | p__Bacteroidota | c__Bacteroidia | o__Sphingobacteriales | f__Sphingobacteriaceae | g__Sphingobacterium |  |
| 02247880 | Bacteria | p__Proteobacteria | c__Alphaproteobacteria | o__Rhizobiales | f__Beijerinckiaceae | g__Bosea |  |
| db280e4dcbead17525115ac125c1b462 | Bacteria | p__Bacteroidota | c__Bacteroidia | o__Sphingobacteriales | f__Sphingobacteriaceae |  |  |
| AB512285 | Bacteria | p__Actinobacteriota | c__Actinobacteria | o__Kineosporiales | f__Kineosporiaceae |  |  |
| 18312889 | Bacteria | p__Actinobacteriota | c__Actinobacteria | o__Propionibacteriales | f__Nocardioidaceae | g__Kribbella |  |
| 02416642 | Bacteria | p__Proteobacteria | c__Alphaproteobacteria | o__Caulobacterales | f__Caulobacteraceae | g__uncultured |  |
| 02408791 | Bacteria | p__Firmicutes | c__Clostridia | o__Lachnospirales | f__Lachnospiraceae |  |  |
| G004793665_1 | Bacteria | p__Actinobacteriota | c__Coriobacteriia | o__Coriobacteriales | f__Atopobiaceae |  |  |
| f3e287448b4b69cfc5a807801ff0e923 | Bacteria | p__Chloroflexi | c__Anaerolineae | o__SBR1031 | f__A4b | g__A4b |  |
| 9dc779987c6b065c2b5e4d6ee2b59a45 | Bacteria | p__Verrucomicrobiota | c__Verrucomicrobiae | o__Chthoniobacterales | f__Chthoniobacteraceae | g__Chthoniobacter | s__uncultured_bacterium |
| RS-GCF-003936705.1-NZ-RHRH01000047.1 | Bacteria | p__Proteobacteria | c__Gammaproteobacteria | o__Pseudomonadales | f__Pseudomonadaceae | g__Pseudomonas |  |
| GB-GCA-905212915.1-CAJMGJ010000030.1 | Bacteria | p__Firmicutes | c__Clostridia | o__Oscillospirales | f__Oscillospiraceae | g__UCG-002 |  |
| ab68fa4ae7496661f823dacdb91185fd | Bacteria | p__Chloroflexi | c__Ktedonobacteria | o__Ktedonobacterales | f__Ktedonobacteraceae | g__1921-2 | s__uncultured_Ktedobacteria |
| MJ031-2-barcode69-umi24140bins-ubs-17 | Bacteria | p__Proteobacteria | c__Gammaproteobacteria | o__Xanthomonadales | f__Xanthomonadaceae | g__Stenotrophomonas |  |
| 9d23a347c2cde03c863e11f533bca5fd | Bacteria | p__Proteobacteria | c__Alphaproteobacteria | o__Rhodobacterales | f__Rhodobacteraceae |  |  |
| e264539779234afea18e1d381895cbe7 | Bacteria | p__Proteobacteria | c__Gammaproteobacteria | o__Enterobacterales | f__Enterobacteriaceae |  |  |
| 8bd72164e61ef816f50efffff9244d89 | Bacteria | p__MBNT15 | c__MBNT15 | o__MBNT15 | f__MBNT15 | g__MBNT15 |  |
| 139176fcc8a17d683fcd1c3e3f0ba066 | Bacteria | p__Proteobacteria | c__Alphaproteobacteria | o__Rhizobiales | f__Rhizobiaceae | g__Ochrobactrum |  |
| KT884900 | Bacteria | p__Proteobacteria | c__Alphaproteobacteria | o__Sphingomonadales | f__Sphingomonadaceae |  |  |
| 1aa01edd945a5ba55884740cc280c6d4 | Bacteria | p__Chloroflexi | c__Anaerolineae | o__SBR1031 | f__A4b | g__A4b |  |
| AY039817 | Bacteria | p__Proteobacteria | c__Alphaproteobacteria | o__Rhizobiales | f__Kaistiaceae | g__Kaistia |  |
| MJ006-1-barcode49-umi104059bins-ubs-5 | Bacteria | p__Firmicutes | c__Negativicutes | o__Veillonellales-Selenomonadales | f__Veillonellaceae | g__Dialister | s__Dialister_invisus |
| AF025364 | Bacteria | p__Proteobacteria | c__Gammaproteobacteria | o__Enterobacterales | f__Enterobacteriaceae |  |  |
| 84f5f3e63b1b129a421b586893b4c62c | Bacteria | p__Bacteroidota | c__Bacteroidia | o__Bacteroidales | f__Prevotellaceae | g__Prevotella |  |
| 7e7dc6411949888230c37656ceb7cfb7 | Bacteria | p__Proteobacteria | c__Gammaproteobacteria | o__Pseudomonadales | f__Moraxellaceae | g__Perlucidibaca | s__uncultured_bacterium |
| 226386f33c292f504a86b152db5f1ff8 | Bacteria | p__Actinobacteriota | c__Actinobacteria | o__Frankiales | f__Acidothermaceae | g__Acidothermus |  |
| 154249a79587f3c1608c3bcf1cae2579 | Bacteria | p__Firmicutes | c__Bacilli | o__Erysipelotrichales | f__Erysipelotrichaceae | g__Faecalibaculum | s__uncultured_bacterium |
| 05170684 | Bacteria |  |  |  |  |  |  |
| AB377289 | Bacteria | p__Actinobacteriota | c__Actinobacteria | o__Corynebacteriales | f__Dietziaceae | g__Dietzia | s__Dietzia_timorensis |
| d84e4fd83200e33f6e1930ad27ab4db1 | Bacteria | p__Proteobacteria | c__Gammaproteobacteria |  |  |  |  |
| 2c97cdf4bc98ace7e332db2cc4e402b7 | Bacteria | p__Actinobacteriota | c__Actinobacteria | o__PeM15 | f__PeM15 | g__PeM15 |  |
| MJ030-2-barcode65-umi87066bins-ubs-5 | Bacteria | p__Acidobacteriota | c__Acidobacteriae | o__Bryobacterales | f__Bryobacteraceae | g__Bryobacter |  |
| 305bf80fc9b13310ce4acb33b9eda215 | Bacteria | p__Proteobacteria | c__Gammaproteobacteria | o__Burkholderiales | f__Nitrosomonadaceae | g__mle1-7 |  |
| 02354370 | Bacteria | p__Firmicutes | c__Clostridia | o__Peptostreptococcales-Tissierellales | f__Peptostreptococcales-Tissierellales | g__Peptoniphilus |  |
| bee5ba28df0ef393b30201a1d6bfac29 | Bacteria | p__Proteobacteria | c__Gammaproteobacteria | o__Burkholderiales | f__A21b | g__A21b |  |
| 08348162 | Bacteria | p__Proteobacteria | c__Alphaproteobacteria | o__Caulobacterales | f__Caulobacteraceae |  |  |
| MJ006-1-barcode37-umi157705bins-ubs-3 | Bacteria | p__Firmicutes | c__Clostridia | o__Lachnospirales | f__Lachnospiraceae |  |  |
| AB681399 | Bacteria | p__Proteobacteria | c__Gammaproteobacteria | o__Xanthomonadales | f__Xanthomonadaceae | g__Thermomonas |  |
| 05666866 | Bacteria | p__Verrucomicrobiota | c__Verrucomicrobiae | o__Opitutales | f__Opitutaceae |  |  |
| abbf5c3176e543ee52e96f5a2cfe2476 | Bacteria | p__Proteobacteria | c__Gammaproteobacteria | o__Enterobacterales |  |  |  |
| 04986529 | Bacteria | p__RCP2-54 | c__RCP2-54 | o__RCP2-54 | f__RCP2-54 | g__RCP2-54 |  |
| 751efb03ce0f26a3fb572b657cfbbe98 | Bacteria | p__Bacteroidota | c__Bacteroidia | o__Bacteroidales | f__Prevotellaceae | g__Prevotella |  |
| e7a941b69f25109fadc39bff9a5f1eff | Bacteria | p__Bacteroidota | c__Bacteroidia | o__Chitinophagales | f__Chitinophagaceae |  |  |
| a928d2c82dfca8bbab23562d551e88d7 | Bacteria | p__Proteobacteria | c__Gammaproteobacteria | o__Pseudomonadales | f__Moraxellaceae | g__Perlucidibaca | s__uncultured_bacterium |
| G003223455_1 | Bacteria | p__Gemmatimonadota | c__Gemmatimonadetes | o__Gemmatimonadales | f__Gemmatimonadaceae | g__Gemmatimonas |  |
| ef3e5e7f9230f9240ae0e432ae6e5161 | Bacteria | p__Proteobacteria | c__Gammaproteobacteria | o__Diplorickettsiales | f__Diplorickettsiaceae | g__Rickettsiella |  |
| 40b57ae054ab860442dd256a2ca0c076 | Bacteria | p__Proteobacteria | c__Gammaproteobacteria | o__Xanthomonadales | f__Rhodanobacteraceae | g__Dyella |  |
| d18167ec207d8c08f602e97dacf4c8ee | Bacteria | p__Actinobacteriota | c__Actinobacteria | o__Bifidobacteriales | f__Bifidobacteriaceae | g__Bifidobacterium |  |
| 625a97fcd4fdfd4880f15861481794e9 | Bacteria | p__Bacteroidota | c__Bacteroidia | o__Sphingobacteriales | f__Sphingobacteriaceae | g__Sphingobacterium | s__Sphingobacterium_nematocida |
| G000516555_1 | Bacteria | p__Proteobacteria | c__Alphaproteobacteria | o__Rhizobiales | f__Xanthobacteraceae | g__uncultured |  |
| 77ac8bc3d31c1e89cf7efdf5400c771b | Bacteria | p__Bacteroidota | c__Bacteroidia | o__Bacteroidales | f__Muribaculaceae | g__Muribaculaceae |  |
| 583e80aa86c9228a16f856b70f403235 | Bacteria | p__Actinobacteriota | c__Actinobacteria | o__Corynebacteriales | f__Corynebacteriaceae | g__Corynebacterium |  |
| G001602455_1 | Bacteria | p__Proteobacteria | c__Gammaproteobacteria | o__Burkholderiales | f__Nitrosomonadaceae | g__Ellin6067 |  |
| 73cc27f3c059132d1c12b3226f302eca | Bacteria | p__Proteobacteria | c__Gammaproteobacteria |  |  |  |  |
| MJ033-1-barcode39-umi8909bins-ubs-37 | Bacteria | p__Verrucomicrobiota | c__Verrucomicrobiae | o__Pedosphaerales | f__Pedosphaeraceae | g__uncultured |  |
| 04959976 | Bacteria | p__Proteobacteria | c__Gammaproteobacteria | o__Burkholderiales | f__Comamonadaceae | g__Schlegelella |  |
| 886618cfdef9dfc73111e73053be44d4 | Bacteria | p__Methylomirabilota | c__Methylomirabilia | o__Rokubacteriales | f__Rokubacteriales | g__Rokubacteriales | s__uncultured_bacterium |
| ec6bf443e034846141b306908b25c067 | Bacteria | p__Firmicutes | c__Clostridia | o__Oscillospirales | f__Ruminococcaceae | g__Ruminococcus | s__Ruminococcus_callidus |
| 8e3d17990a512f0f4801007442f0d0b6 | Bacteria | p__Acidobacteriota | c__Acidobacteriae | o__Acidobacteriales | f__Koribacteraceae | g__Candidatus_Koribacter | s__uncultured_bacterium |
| 02408299 | Bacteria | p__Firmicutes | c__Clostridia | o__Lachnospirales | f__Lachnospiraceae | g__Blautia |  |
| 5b3cdfd36db92a3314b90b360506854c | Bacteria | p__Proteobacteria | c__Alphaproteobacteria | o__Rhizobiales | f__Beijerinckiaceae | g__Qingshengfania | s__Pseudochelatococcus_lubricantis |
| c8bde976b7bc1535249e57708da6798d | Bacteria | p__Patescibacteria | c__Parcubacteria | o__Candidatus_Moranbacteria | f__Candidatus_Moranbacteria | g__Candidatus_Moranbacteria |  |
| 516a39ceb00d682b90477aac331fc73a | Bacteria | p__Proteobacteria | c__Gammaproteobacteria | o__Burkholderiales | f__SC-I-84 | g__SC-I-84 |  |
| GB-GCA-005502595.1-SCOF01000066.1 | Bacteria | p__Proteobacteria | c__Alphaproteobacteria | o__Sphingomonadales | f__Sphingomonadaceae |  |  |
| af72414cf27cb886607c336a957d1599 | Bacteria | p__Proteobacteria | c__Alphaproteobacteria | o__Rhizobiales | f__Rhizobiaceae |  |  |
| d656e0962a495f31c767477c83a69924 | Bacteria | p__Actinobacteriota | c__Actinobacteria | o__Corynebacteriales | f__Mycobacteriaceae | g__Mycobacterium |  |
| 56fc51913946dbdb73e76e2359d9db3a | Bacteria | p__Firmicutes | c__Bacilli | o__Lactobacillales | f__Carnobacteriaceae | g__Marinilactibacillus | s__uncultured_bacterium |
| fd39f1748204e731fa440954e0ff0937 | Bacteria | p__Actinobacteriota | c__Acidimicrobiia | o__Microtrichales | f__uncultured | g__uncultured | s__wastewater_metagenome |
| AY170847 | Bacteria | p__Proteobacteria | c__Gammaproteobacteria | o__Burkholderiales | f__Alcaligenaceae | g__Achromobacter |  |
| 27486d9155f971cd00a21366db76c281 | Bacteria | p__Proteobacteria | c__Gammaproteobacteria | o__Burkholderiales | f__Sutterellaceae | g__Parasutterella | s__uncultured_bacterium |
| aec83f1c7befb70ea8b5d4038ece0f9d | Bacteria | p__Acidobacteriota | c__Vicinamibacteria | o__Subgroup_17 | f__Subgroup_17 | g__Subgroup_17 | s__metagenome |
| 04925939 | Bacteria | p__Proteobacteria | c__Alphaproteobacteria | o__Sphingomonadales | f__Sphingomonadaceae | g__Altererythrobacter |  |
| 04907623 | Bacteria | p__Proteobacteria | c__Alphaproteobacteria | o__Caulobacterales | f__Caulobacteraceae |  |  |
| 3e622e2279e35c8ac834f29313ac5d0c | Bacteria | p__Firmicutes | c__Bacilli | o__Lactobacillales | f__Lactobacillaceae | g__Lactobacillus |  |
| 15391209 | Bacteria | p__Chloroflexi | c__Ktedonobacteria | o__Ktedonobacterales | f__Ktedonobacteraceae | g__uncultured | s__uncultured_Thermosporothrix |
| MK224650 | Bacteria | p__Bacteroidota | c__Bacteroidia | o__Flavobacteriales | f__Weeksellaceae | g__Empedobacter | s__Empedobacter_sp. |
| 239a53bb4438a755703553f250006923 | Bacteria | p__Proteobacteria | c__Alphaproteobacteria | o__Rhodospirillales | f__uncultured | g__uncultured | s__Azospirillum_sp. |
| 04878334 | Bacteria | p__Actinobacteriota | c__Actinobacteria | o__Corynebacteriales | f__Corynebacteriaceae | g__Corynebacterium |  |
| RS-GCF-004002395.1-NZ-RPJY01000114.1 | Bacteria | p__Proteobacteria | c__Alphaproteobacteria | o__Rhizobiales | f__Rhizobiaceae |  |  |
| 3352024a1f76a1dddae16b6f1da085ad | Bacteria | p__Actinobacteriota | c__Thermoleophilia | o__Solirubrobacterales | f__67-14 | g__67-14 |  |
| RS-GCF-009765215.1-NZ-VLNV01000003.1 | Bacteria | p__Proteobacteria | c__Gammaproteobacteria | o__Xanthomonadales | f__Xanthomonadaceae | g__Lysobacter |  |
| JN620361 | Bacteria | p__Proteobacteria | c__Alphaproteobacteria | o__Rhodobacterales | f__Rhodobacteraceae | g__Falsirhodobacter |  |
| 17ca8ed8101d6274936c811196889a67 | Bacteria | p__Methylomirabilota | c__Methylomirabilia | o__Rokubacteriales | f__WX65 | g__WX65 | s__uncultured_bacterium |
| 65784b4fc80fe30a908bafe57ed0295d | Bacteria | p__Firmicutes | c__Bacilli | o__Erysipelotrichales | f__Erysipelotrichaceae | g__Turicibacter |  |
| 27ee1bb4e401b5a064e98b853c6297a1 | Bacteria | p__Proteobacteria | c__Alphaproteobacteria | o__Sphingomonadales | f__Sphingomonadaceae | g__Sphingomonas |  |
| a22770288de93140bb8e8b73cffd482b | Bacteria | p__Proteobacteria | c__Alphaproteobacteria | o__Sphingomonadales | f__Sphingomonadaceae | g__Sphingomonas |  |
| 8797d2e91ab44f2729dc51f034223a86 | Bacteria | p__Proteobacteria | c__Gammaproteobacteria | o__Enterobacterales | f__Enterobacteriaceae |  |  |
| 7f06620d73f425722bc96e5b3e9404aa | Bacteria | p__Proteobacteria | c__Gammaproteobacteria | o__Burkholderiales | f__SC-I-84 | g__SC-I-84 | s__uncultured_Nitrosomonadaceae |
| 87bc0949e4ac6e72ee2376ab3f97ad32 | Bacteria | p__Firmicutes | c__Incertae_Sedis | o__DTU014 | f__DTU014 | g__DTU014 |  |
| 47c2b539d6d6b3fcbb93d53d109cc0f7 | Bacteria | p__Patescibacteria | c__Saccharimonadia | o__Saccharimonadales | f__Saccharimonadales | g__Saccharimonadales | s__metagenome |
| RS-GCF-018612915.1-NZ-JAGGRL010000015.1 | Bacteria | p__Proteobacteria | c__Gammaproteobacteria | o__Xanthomonadales | f__Xanthomonadaceae | g__Stenotrophomonas |  |
| ad2b87ca85b8a4b07e2410b10bf75cbb | Bacteria | p__Actinobacteriota | c__Acidimicrobiia | o__Microtrichales | f__Ilumatobacteraceae | g__Ilumatobacter |  |
| 4f3bf1df6e74e7c7bd52775c11d5fc6d | Bacteria | p__Firmicutes | c__Clostridia | o__Clostridia | f__Hungateiclostridiaceae | g__HN-HF0106 | s__uncultured_prokaryote |
| 5b7ef07540333415d36deef47733b06e | Bacteria | p__Firmicutes | c__Clostridia | o__Lachnospirales | f__Lachnospiraceae | g__Tuzzerella |  |
| G011106855_4 | Bacteria | p__Proteobacteria | c__Gammaproteobacteria | o__Pseudomonadales | f__Pseudomonadaceae | g__Pseudomonas |  |
| 26cdf5cc651b20806ab5da8970c21849 | Bacteria | p__Firmicutes | c__Bacilli | o__Erysipelotrichales | f__Erysipelotrichaceae | g__Turicibacter | s__uncultured_bacterium |
| GB-GCA-000373445.1-KB891467.1 | Bacteria | p__Actinobacteriota | c__Actinobacteria | o__Streptomycetales | f__Streptomycetaceae | g__Streptomyces |  |
| MJ030-1-barcode25-umi22757bins-ubs-22 | Bacteria | p__Firmicutes | c__Bacilli | o__Erysipelotrichales | f__Erysipelotrichaceae | g__Turicibacter | s__uncultured_bacterium |
| MJ007-1-barcode25-umi2674bins-ubs-91 | Bacteria | p__Proteobacteria | c__Gammaproteobacteria | o__Enterobacterales |  |  |  |
| e7f1842193e0c8af329a60a906692e7c | Bacteria | p__Chloroflexi | c__Anaerolineae | o__Caldilineales | f__Caldilineaceae | g__uncultured |  |
| 02237917 | Bacteria | p__Chloroflexi | c__Chloroflexia | o__Thermomicrobiales | f__JG30-KF-CM45 | g__JG30-KF-CM45 | s__uncultured_bacterium |
| a5d8fc51184d2aa6156c3a89b5090a7a | Bacteria | p__Proteobacteria | c__Gammaproteobacteria | o__Enterobacterales | f__Enterobacteriaceae |  |  |
| MJ020-1-barcode47-umi66bins-ubs-1712 | Bacteria | p__Proteobacteria | c__Gammaproteobacteria | o__Pseudomonadales | f__Moraxellaceae | g__Acinetobacter |  |
| MJ010-2-barcode58-umi105011bins-ubs-5 | Bacteria | p__Firmicutes | c__Bacilli | o__Lactobacillales | f__Enterococcaceae | g__Enterococcus |  |
| AB109889 | Bacteria | p__Proteobacteria | c__Gammaproteobacteria | o__Burkholderiales | f__Comamonadaceae | g__Curvibacter | s__Curvibacter_gracilis |
| c7edbc4ecfa7b91f37e3311e70cff7fc | Bacteria |  |  |  |  |  |  |
| 02e9d6a14d0ae570f97b94b5f09cbec6 | Bacteria | p__Chloroflexi | c__Chloroflexia | o__Thermomicrobiales | f__JG30-KF-CM45 | g__JG30-KF-CM45 |  |
| 72bc064cfb372edfbc848bc17d270fcd | Bacteria | p__Actinobacteriota | c__Coriobacteriia | o__Coriobacteriales | f__Coriobacteriales_Incertae_Sedis | g__Raoultibacter |  |
| RS-GCF-000621265.1-NZ-JHYU01000054.1 | Bacteria | p__Proteobacteria | c__Gammaproteobacteria | o__Pseudomonadales | f__Pseudomonadaceae | g__Pseudomonas |  |
| KU569905 | Bacteria | p__Proteobacteria | c__Alphaproteobacteria | o__Elsterales | f__Elsteraceae | g__Elstera |  |
| 04959823 | Bacteria | p__Proteobacteria | c__Gammaproteobacteria | o__Burkholderiales | f__Comamonadaceae |  |  |
| 05021811 | Bacteria | p__Actinobacteriota | c__Actinobacteria | o__Micrococcales | f__Sanguibacteraceae | g__Sanguibacter |  |
| 8954477901e389494cff56078c8fb3ec | Bacteria | p__Proteobacteria | c__Gammaproteobacteria | o__Burkholderiales | f__Comamonadaceae |  |  |
| MJ006-1-barcode26-umi150343bins-ubs-3 | Bacteria | p__Firmicutes | c__Clostridia | o__Lachnospirales | f__Lachnospiraceae |  |  |
| 510cceaa799b380b96f1abd388ae3f17 | Bacteria | p__Actinobacteriota | c__Thermoleophilia | o__Gaiellales | f__uncultured | g__uncultured | s__uncultured_microorganism |
| 04958264 | Bacteria | p__Actinobacteriota | c__Actinobacteria | o__Corynebacteriales | f__Nocardiaceae | g__Rhodococcus |  |
| 7612af7a6a13f6f311dce2101c772859 | Bacteria | p__Proteobacteria | c__Alphaproteobacteria | o__Rhodobacterales | f__Rhodobacteraceae | g__Paracoccus |  |
| 14d303a78a96bf9774e6355592393a6a | Bacteria | p__Actinobacteriota | c__Coriobacteriia | o__Coriobacteriales | f__Eggerthellaceae | g__Enterorhabdus | s__uncultured_bacterium |
| 48b239256ec30e47bc4f797dee6b0d5c | Bacteria | p__Cyanobacteria | c__Cyanobacteriia | o__Cyanobacteriales | f__Chroococcidiopsaceae | g__uncultured | s__Pseudocapsa_sp. |
| a2297563717d436e2dcd00c0ff10b6b3 | Bacteria | p__Bacteroidota | c__Bacteroidia | o__Flavobacteriales | f__Flavobacteriaceae | g__Flavobacterium |  |
| 5ff37ddb7ec292d9f841cbeb07d2530a | Bacteria | p__Bacteroidota | c__Bacteroidia | o__Sphingobacteriales | f__Sphingobacteriaceae | g__Mucilaginibacter |  |
| MJ020-1-barcode31-umi45504bins-ubs-10 | Bacteria | p__Verrucomicrobiota | c__Verrucomicrobiae | o__Chthoniobacterales | f__Chthoniobacteraceae | g__Chthoniobacter | s__uncultured_bacterium |
| d1013466217eece24835572b401cd58d | Bacteria | p__Firmicutes | c__Bacilli | o__Staphylococcales | f__Staphylococcaceae | g__Staphylococcus |  |
| d61b4bd3022b0151af7acca20489c730 | Bacteria | p__Proteobacteria | c__Alphaproteobacteria | o__Rhizobiales | f__Beijerinckiaceae | g__Microvirga |  |
| RS-GCF-001150945.1-NZ-CLAX01000006.1 | Bacteria | p__Firmicutes | c__Bacilli | o__Lactobacillales | f__Streptococcaceae | g__Streptococcus |  |
| MJ006-1-barcode27-umi114501bins-ubs-4 | Bacteria | p__Firmicutes | c__Clostridia | o__Oscillospirales | f__Ruminococcaceae | g__Negativibacillus | s__uncultured_bacterium |
| 185c065f0874969cc6897fe76d1164ec | Bacteria | p__Bacteroidota | c__Bacteroidia | o__Bacteroidales | f__Tannerellaceae | g__Parabacteroides |  |
| dd3cca0870e4c7114252aa7ae807c60f | Bacteria | p__Actinobacteriota | c__Actinobacteria | o__Micrococcales | f__Microbacteriaceae | g__Microbacterium |  |
| ade281775746a413c36e2062e783c972 | Bacteria | p__Proteobacteria | c__Gammaproteobacteria | o__Burkholderiales | f__Comamonadaceae |  |  |
| 0ac2ec8dadaf97f1a4ce4bfee7279351 | Bacteria | p__Acidobacteriota | c__Vicinamibacteria | o__Vicinamibacterales | f__Vicinamibacteraceae | g__Vicinamibacteraceae |  |
| 8afffe8f524fae12eb1a63e63ff20efd | Bacteria | p__Verrucomicrobiota | c__Verrucomicrobiae | o__Pedosphaerales | f__Pedosphaeraceae | g__Pedosphaeraceae |  |
| 02859846 | Bacteria | p__Chloroflexi | c__Chloroflexia | o__Chloroflexales | f__Roseiflexaceae | g__uncultured | s__uncultured_bacterium |
| LC468799 | Bacteria | p__Bacteroidota | c__Bacteroidia | o__Bacteroidales | f__Rikenellaceae | g__Alistipes | s__Alistipes_obesi |
| JN600332 | Bacteria | p__Proteobacteria | c__Gammaproteobacteria | o__Enterobacterales | f__Pectobacteriaceae | g__Pectobacterium |  |
| GB-GCA-016713725.1-JADJOO010000001.1 | Bacteria | p__Proteobacteria | c__Gammaproteobacteria | o__Steroidobacterales | f__Steroidobacteraceae | g__uncultured | s__uncultured_prokaryote |
| AJ438177 | Bacteria | p__Bacteroidota | c__Bacteroidia | o__Sphingobacteriales | f__Sphingobacteriaceae | g__Sphingobacterium | s__Sphingobacterium_thalpophilum |
| b57058eb818df8b5aacfa5494ca1be42 | Bacteria | p__Bacteroidota | c__Bacteroidia | o__Bacteroidales | f__Bacteroidaceae | g__Bacteroides |  |
| 1fccc1fa3553b241a9ddac7eac07a30b | Bacteria | p__Proteobacteria | c__Gammaproteobacteria | o__Pseudomonadales | f__Moraxellaceae | g__Acinetobacter |  |
| AJ854057 | Bacteria | p__Actinobacteriota | c__Actinobacteria | o__Corynebacteriales | f__Nocardiaceae | g__Nocardia |  |
| 36b8d10e040fc3848f7744a82b29864d | Bacteria | p__Chloroflexi | c__KD4-96 | o__KD4-96 | f__KD4-96 | g__KD4-96 | s__uncultured_bacterium |
| 02290359 | Bacteria | p__Proteobacteria | c__Alphaproteobacteria | o__Rhodobacterales | f__Rhodobacteraceae |  |  |
| MJ030-2-barcode60-umi88722bins-ubs-5 | Bacteria | p__Acidobacteriota | c__Acidobacteriae | o__Acidobacteriales | f__Acidobacteriaceae_(Subgroup_1) | g__Occallatibacter |  |
| 834ae0ce5ab7778a92bd8d3e2b39a970 | Bacteria | p__Actinobacteriota | c__Acidimicrobiia | o__Microtrichales | f__Microtrichaceae | g__IMCC26207 |  |
| GB-GCA-902364865.1-CABJFE010000006.1 | Bacteria | p__Actinobacteriota | c__Coriobacteriia | o__Coriobacteriales | f__Coriobacteriaceae | g__Collinsella |  |
| RS-GCF-007641235.1-NZ-CP038157.1 | Bacteria | p__Actinobacteriota | c__Actinobacteria | o__Corynebacteriales | f__Corynebacteriaceae | g__Corynebacterium |  |
| 8004f476987a1e17444182dc4271bc6d | Bacteria | p__Proteobacteria | c__Gammaproteobacteria | o__Xanthomonadales | f__Xanthomonadaceae | g__Stenotrophomonas |  |
| 10568189 | Bacteria | p__Bacteroidota | c__Bacteroidia | o__Sphingobacteriales | f__Sphingobacteriaceae | g__Sphingobacterium |  |
| a4b2a138f36a65b422dbb154f185f547 | Bacteria | p__Bacteroidota | c__Bacteroidia | o__Chitinophagales | f__Chitinophagaceae | g__Terrimonas | s__Terrimonas_rubra |
| 66572773e8ce8157ac9fad48f49d0898 | Bacteria | p__Chloroflexi | c__Anaerolineae | o__Caldilineales | f__Caldilineaceae | g__uncultured | s__uncultured_bacterium |
| 05007824 | Bacteria | p__Proteobacteria | c__Alphaproteobacteria | o__Sphingomonadales | f__Sphingomonadaceae | g__Sphingobium |  |
| 04933291 | Bacteria | p__Bacteroidota | c__Bacteroidia | o__Chitinophagales | f__Chitinophagaceae | g__Chitinophagaceae | s__Flavisolibacter_sp. |
| 24d09940bd6b811f14d6f8e40026b737 | Bacteria | p__Proteobacteria | c__Alphaproteobacteria | o__Rhodobacterales | f__Rhodobacteraceae | g__Gemmobacter |  |
| 38b8d8c3c74c03f4e05e7b0c3fd7818a | Bacteria | p__Proteobacteria | c__Gammaproteobacteria | o__Burkholderiales | f__Nitrosomonadaceae | g__MND1 |  |
| 018362984639e09f628e6603a6c86e50 | Bacteria | p__Bacteroidota | c__Bacteroidia | o__Bacteroidales | f__Bacteroidaceae | g__Bacteroides | s__Bacteroides_plebeius |
| b91b366de1c5257ddf2c4064f6078341 | Bacteria | p__Proteobacteria | c__Alphaproteobacteria | o__Rhizobiales | f__Rhizobiales_Incertae_Sedis | g__uncultured |  |
| fcc6345506d7c6e87f505951be5035fd | Bacteria | p__Bacteroidota | c__Bacteroidia | o__Flavobacteriales | f__Weeksellaceae | g__Empedobacter |  |
| AM905849 | Bacteria | p__Proteobacteria | c__Alphaproteobacteria | o__Acetobacterales | f__Acetobacteraceae | g__Acetobacter |  |
| 7df46337546883dac57a9b576d77742c | Bacteria | p__Firmicutes | c__Bacilli | o__Erysipelotrichales | f__Erysipelotrichaceae | g__Turicibacter |  |
| bbc83fc5fb0c67114d25fe6b763fa93c | Bacteria | p__Proteobacteria | c__Gammaproteobacteria | o__Burkholderiales | f__Comamonadaceae | g__Variovorax |  |
| 920629ac382687d1c13ac9b979a4919d | Bacteria | p__Proteobacteria | c__Gammaproteobacteria | o__Pseudomonadales | f__Pseudomonadaceae | g__Pseudomonas |  |
| 380f1e27cf7f3189cba18e450bbc3803 | Bacteria | p__Actinobacteriota | c__Acidimicrobiia | o__Microtrichales | f__Iamiaceae | g__Iamia |  |
| 04961227 | Bacteria | p__Actinobacteriota | c__Actinobacteria | o__Corynebacteriales | f__Corynebacteriaceae | g__Corynebacterium |  |
| a07c5df68439a499c23bf7cd3a5ced18 | Bacteria | p__Acidobacteriota | c__Vicinamibacteria | o__Vicinamibacterales | f__uncultured | g__uncultured |  |
| c6f3d19203ead4f3784f078587f2ab7c | Bacteria | p__Myxococcota | c__Polyangia | o__Polyangiales | f__Sandaracinaceae | g__uncultured |  |
| MJ006-2-barcode68-umi149393bins-ubs-3 | Bacteria | p__Firmicutes | c__Clostridia | o__Lachnospirales | f__Lachnospiraceae | g__GCA-900066575 |  |
| a153dc8f666dea2178580e36ee7bed78 | Bacteria | p__Proteobacteria | c__Gammaproteobacteria | o__Enterobacterales | f__Morganellaceae | g__endosymbionts |  |
| MJ010-2-barcode64-umi87061bins-ubs-6 | Bacteria | p__Proteobacteria | c__Gammaproteobacteria | o__Enterobacterales | f__Morganellaceae | g__Providencia |  |
| MG970331 | Bacteria | p__Bacteroidota | c__Bacteroidia | o__Bacteroidales | f__Muribaculaceae | g__Muribaculaceae |  |
| 28a5015caae8a305273c26790a5ed341 | Bacteria | p__Bacteroidota | c__Bacteroidia | o__Chitinophagales | f__Chitinophagaceae | g__Taibaiella |  |
| MJ030-2-barcode65-umi112205bins-ubs-3 | Bacteria | p__Acidobacteriota | c__Acidobacteriae | o__Bryobacterales | f__Bryobacteraceae | g__Bryobacter |  |
| 99363b9105194945fdc25382afb9ff12 | Bacteria | p__Myxococcota | c__Polyangia | o__Haliangiales | f__Haliangiaceae | g__Haliangium |  |
| c2f028e4602f07655582b847b406d14d | Bacteria | p__Actinobacteriota | c__Acidimicrobiia | o__Microtrichales |  |  |  |
| G002117145_1 | Bacteria | p__Proteobacteria | c__Alphaproteobacteria | o__Caedibacterales | f__Caedibacteraceae | g__Candidatus_Nucleicultrix | s__Candidatus_Nucleicultrix |
| 856e4489f208c83679ea9275795c41dc | Bacteria | p__Proteobacteria | c__Gammaproteobacteria | o__Pseudomonadales | f__Moraxellaceae | g__Acinetobacter |  |
| 381d60611fcfdf4e10b5010c4f8d83dc | Bacteria | p__Bdellovibrionota | c__Bdellovibrionia | o__Bdellovibrionales | f__Bdellovibrionaceae | g__Bdellovibrio | s__Bdellovibrio_bacteriovorus |
| 06814804 | Bacteria | p__Planctomycetota | c__Phycisphaerae | o__Tepidisphaerales | f__CPla-3_termite_group | g__CPla-3_termite_group | s__metagenome |
| c3e68fd55b74f92e3cd9c60fe4b5e195 | Bacteria | p__Patescibacteria | c__Saccharimonadia | o__Saccharimonadales | f__Saccharimonadales | g__Saccharimonadales |  |
| 8199d91d3bc1689bcf0c01709f6f8b51 | Bacteria | p__Actinobacteriota | c__MB-A2-108 | o__MB-A2-108 | f__MB-A2-108 | g__MB-A2-108 |  |
| MJ032-1-barcode47-umi36819bins-ubs-9 | Bacteria | p__Firmicutes | c__Clostridia | o__Clostridiales | f__Clostridiaceae | g__Clostridium_sensu_stricto_1 |  |
| ddec2b964cd77de28f6c2f1eb575735f | Bacteria | p__Desulfobacterota | c__Desulfuromonadia | o__Geobacterales | f__Geobacteraceae | g__uncultured | s__uncultured_delta |
| bc4c84b3f52d774b5fa4370bf8a8c38f | Bacteria | p__Proteobacteria | c__Gammaproteobacteria | o__Enterobacterales | f__Enterobacteriaceae |  |  |
| 04875882 | Bacteria | p__Proteobacteria | c__Gammaproteobacteria | o__Pseudomonadales | f__Moraxellaceae | g__Acinetobacter |  |
| 1e6f32d6af00492e332b1854bc68b330 | Bacteria | p__Acidobacteriota | c__Vicinamibacteria | o__Vicinamibacterales | f__uncultured | g__uncultured | s__uncultured_Acidobacteriales |
| e5c26807840c0cf5e6ed637f3c65665e | Bacteria | p__Bacteroidota | c__Bacteroidia | o__Bacteroidales | f__Muribaculaceae | g__Muribaculaceae | s__uncultured_bacterium |
| 27b0df25f10bd2a08fbb8cc713431324 | Bacteria | p__Proteobacteria | c__Gammaproteobacteria |  |  |  |  |
| G000154525_5 | Bacteria | p__Bacteroidota | c__Bacteroidia | o__Bacteroidales | f__Bacteroidaceae | g__Bacteroides | s__Bacteroides_stercoris |
| cd3251023d3a51543c328cf2b4efad60 | Bacteria | p__Proteobacteria | c__Gammaproteobacteria | o__Burkholderiales | f__Rhodocyclaceae |  |  |
| 02387629 | Bacteria | p__Proteobacteria | c__Alphaproteobacteria | o__Rhizobiales | f__Rhizobiaceae | g__Allorhizobium-Neorhizobium-Pararhizobium-Rhizobium |  |
| KC967412 | Bacteria | p__Firmicutes | c__Clostridia | o__Clostridiales | f__Clostridiaceae | g__Clostridium_sensu_stricto_13 |  |
| RS-GCF-014306135.1-NZ-JACRWD010000026.1 | Bacteria | p__Firmicutes | c__Clostridia | o__Peptostreptococcales-Tissierellales | f__Peptostreptococcaceae |  |  |
| cd52b314ac5e6b7b871e2057d2ebd9e5 | Bacteria | p__Firmicutes | c__Bacilli | o__Lactobacillales | f__Enterococcaceae | g__Enterococcus |  |
| 023fad7cac54f07220d85a5126c5c5c9 | Bacteria | p__Actinobacteriota | c__Thermoleophilia | o__Solirubrobacterales | f__Solirubrobacteraceae | g__Parviterribacter | s__uncultured_bacterium |
| 04954928 | Bacteria | p__Firmicutes | c__Bacilli | o__Bacillales | f__Bacillaceae | g__Geobacillus |  |
| EU912483 | Bacteria | p__Actinobacteriota | c__Actinobacteria | o__Micrococcales | f__Microbacteriaceae | g__Leifsonia |  |
| RS-GCF-013317925.1-NZ-JAALZB010000002.1 | Bacteria | p__Proteobacteria | c__Alphaproteobacteria | o__Rhizobiales | f__Rhizobiaceae | g__Allorhizobium-Neorhizobium-Pararhizobium-Rhizobium |  |
| e04792f5e0ad8d08021e23c08bfd3566 | Bacteria | p__Actinobacteriota | c__Actinobacteria | o__Micromonosporales | f__Micromonosporaceae | g__Planosporangium |  |
| MJ010-2-barcode64-umi146610bins-ubs-3 | Bacteria | p__Proteobacteria | c__Gammaproteobacteria | o__Enterobacterales | f__Erwiniaceae |  |  |
| 04908753 | Bacteria | p__Proteobacteria | c__Alphaproteobacteria | o__Rhizobiales | f__Rhizobiaceae |  |  |
| 209382d317ed43ea3fb5c80971606683 | Bacteria | p__Proteobacteria | c__Alphaproteobacteria | o__Sphingomonadales | f__Sphingomonadaceae | g__Sphingobium |  |
| G000338055_1 | Bacteria | p__Actinobacteriota | c__Actinobacteria | o__Micrococcales | f__Dermabacteraceae | g__Brachybacterium |  |
| b7d064b916d8b3148efd8b91e4ded9a2 | Bacteria | p__Firmicutes | c__Bacilli | o__Erysipelotrichales | f__Erysipelotrichaceae | g__Erysipelothrix |  |
| a6cd937f6d7970734f4886f3d329eccb | Bacteria | p__Firmicutes | c__Clostridia | o__Lachnospirales | f__Lachnospiraceae |  |  |
| 2c7019e11f812764e62f52941a7a60bb | Bacteria | p__Chloroflexi | c__Anaerolineae | o__SBR1031 | f__SBR1031 | g__SBR1031 | s__wastewater_metagenome |
| G001436895_1 | Bacteria | p__Firmicutes | c__Bacilli | o__Lactobacillales | f__Leuconostocaceae | g__Weissella |  |
| RS-GCF-000364185.1-NZ-KB822413.1 | Bacteria | p__Firmicutes | c__Bacilli | o__Lactobacillales | f__Lactobacillaceae | g__Lactobacillus | s__Lactobacillus_intestinalis |
| 8463fe9c6976889838b9db6dfabd0277 | Bacteria | p__Firmicutes | c__Bacilli | o__Lactobacillales | f__Streptococcaceae | g__Streptococcus |  |
| f9dd086d17f2ea064bf482ddfc283d9c | Bacteria | p__Proteobacteria | c__Gammaproteobacteria | o__Burkholderiales | f__Comamonadaceae |  |  |
| GB-GCA-002730895.1-PCBC01000015.1 | Bacteria | p__Proteobacteria | c__Gammaproteobacteria | o__Burkholderiales | f__Burkholderiaceae | g__Cupriavidus |  |
| 04898516 | Bacteria | p__Acidobacteriota | c__Acidobacteriae | o__Subgroup_2 | f__Subgroup_2 | g__Subgroup_2 |  |
| 99da280444ee5e7e1b57f45cba73d870 | Bacteria | p__Proteobacteria | c__Gammaproteobacteria | o__Burkholderiales | f__Comamonadaceae |  |  |
| FR733885 | Bacteria | p__Proteobacteria | c__Alphaproteobacteria | o__Rhizobiales | f__Beijerinckiaceae | g__Methylobacterium-Methylorubrum |  |
| 16612836 | Bacteria | p__Firmicutes | c__Clostridia | o__Lachnospirales | f__Lachnospiraceae |  |  |
| 8b3486725db73c4fa0333b2db1b09a06 | Bacteria | p__Proteobacteria | c__Gammaproteobacteria | o__Burkholderiales | f__Comamonadaceae |  |  |
| 1b382539fb0cda8b96998a7323de4884 | Bacteria | p__Bacteroidota | c__Bacteroidia | o__Bacteroidales | f__Bacteroidaceae | g__Bacteroides | s__Bacteroides_massiliensis |
| 350d5353b5a48fe85a7b9835a000af06 | Bacteria |  |  |  |  |  |  |
| 08800923 | Bacteria | p__Proteobacteria | c__Gammaproteobacteria | o__Xanthomonadales | f__Xanthomonadaceae | g__Lysobacter |  |
| f767dcaebb571f2c24fa72a1518a66ac | Bacteria | p__Chloroflexi | c__Anaerolineae | o__Anaerolineales | f__Anaerolineaceae | g__Longilinea |  |
| 1cc57777f82b2af295f974fa40ddc4f6 | Bacteria | p__Firmicutes | c__Bacilli | o__Bacillales | f__Bacillaceae | g__Bacillus |  |
| c53ee6b68be6a2f794ab6d9c8f737ad8 | Bacteria |  |  |  |  |  |  |
| 5b2e02922afbe64fb1591a03e2ad9187 | Bacteria | p__Firmicutes | c__Bacilli | o__Lactobacillales | f__Streptococcaceae | g__Streptococcus |  |
| ff03382a3f48ee6c9eb7c57481ce40c2 | Bacteria | p__Actinobacteriota | c__Actinobacteria | o__Micrococcales | f__Micrococcaceae |  |  |
| 05027224 | Bacteria | p__Firmicutes | c__Clostridia | o__Lachnospirales | f__Lachnospiraceae | g__Blautia | s__unidentified |
| 02395654 | Bacteria | p__Proteobacteria | c__Gammaproteobacteria | o__Aeromonadales | f__Succinivibrionaceae | g__Succinivibrio | s__uncultured_bacterium |
| 90bbdf11565ff909f40d215bdf3a2e2b | Bacteria | p__Actinobacteriota | c__Acidimicrobiia | o__Microtrichales | f__Ilumatobacteraceae | g__CL500-29_marine_group |  |
| a0caee949e17ef56e41a2857d070d4ef | Bacteria | p__Firmicutes | c__Clostridia | o__Lachnospirales | f__Lachnospiraceae |  |  |
| a04e2a4abece1561deb11d762b246285 | Bacteria | p__Firmicutes | c__Bacilli | o__Erysipelotrichales | f__Erysipelotrichaceae | g__ZOR0006 |  |
| 06184887 | Bacteria | p__Proteobacteria | c__Gammaproteobacteria | o__Beggiatoales | f__Beggiatoaceae | g__uncultured | s__uncultured_gamma |
| a9c1b76b53501a273c81a4616652f251 | Bacteria | p__Actinobacteriota | c__MB-A2-108 | o__MB-A2-108 | f__MB-A2-108 | g__MB-A2-108 | s__uncultured_Methylocystaceae |
| def93cc62968fb382172185c45c920b9 | Bacteria | p__Bacteroidota | c__Bacteroidia | o__Flavobacteriales | f__Weeksellaceae | g__Chryseobacterium |  |
| aa3f5be2fe194365364ac23be51297d6 | Bacteria | p__Verrucomicrobiota | c__Verrucomicrobiae | o__Chthoniobacterales | f__Xiphinematobacteraceae | g__Candidatus_Xiphinematobacter |  |
| 17965726 | Bacteria | p__Chloroflexi | c__Ktedonobacteria | o__Ktedonobacterales | f__Ktedonobacteraceae | g__BacC-u-018 | s__uncultured_bacterium |
| 6f078ec1bada61223c7cc427a72d0e66 | Bacteria | p__Proteobacteria | c__Alphaproteobacteria | o__Rhodobacterales | f__Rhodobacteraceae | g__Rhodobacter |  |
| 01873433 | Bacteria | p__Actinobacteriota | c__Coriobacteriia | o__Coriobacteriales | f__Eggerthellaceae |  |  |
| 760a439f5dfb60160644df5e3ac2b2c0 | Bacteria | p__Methylomirabilota | c__Methylomirabilia | o__Rokubacteriales | f__Rokubacteriales | g__Rokubacteriales | s__metagenome |
| 1c9c1bf950aef3dcd5141b2554b9f535 | Bacteria | p__Actinobacteriota | c__Thermoleophilia | o__Gaiellales | f__Gaiellaceae | g__Gaiella |  |
| MJ020-2-barcode70-umi12071bins-ubs-43 | Bacteria | p__Firmicutes | c__Clostridia | o__Peptostreptococcales-Tissierellales | f__Peptostreptococcaceae |  |  |
| 96a05bfb42b7906f32efbbfcd7171da4 | Bacteria | p__Proteobacteria | c__Gammaproteobacteria | o__Enterobacterales | f__Enterobacteriaceae |  |  |
| G001952815_1 | Bacteria | p__Bacteroidota | c__Bacteroidia | o__Sphingobacteriales | f__Sphingobacteriaceae | g__Sphingobacterium |  |
| 06543276 | Bacteria | p__Proteobacteria | c__Gammaproteobacteria | o__Gammaproteobacteria_Incertae_Sedis | f__Unknown_Family | g__Acidibacter |  |
| G000153905_5 | Bacteria | p__Firmicutes | c__Clostridia | o__Lachnospirales | f__Lachnospiraceae | g__Blautia |  |
| G001758345_1 | Bacteria | p__Proteobacteria | c__Gammaproteobacteria | o__Pseudomonadales | f__Moraxellaceae | g__Acinetobacter |  |
| 69d070a555976e540cca864519e2ea56 | Bacteria | p__Proteobacteria | c__Alphaproteobacteria | o__Rhizobiales | f__Hyphomicrobiaceae | g__Pedomicrobium |  |
| 5e6656b672e9a2fc772e61ca667c2ad6 | Bacteria | p__Proteobacteria | c__Alphaproteobacteria | o__Azospirillales | f__Azospirillales_Incertae_Sedis | g__Stella |  |
| MJ030-1-barcode25-umi95028bins-ubs-4 | Bacteria | p__Nitrospirota | c__Nitrospiria | o__Nitrospirales | f__Nitrospiraceae | g__Nitrospira |  |
| 16329513 | Bacteria | p__Firmicutes | c__Clostridia | o__Peptostreptococcales-Tissierellales | f__Anaerovoracaceae | g__[Eubacterium]_brachy_group | s__uncultured_bacterium |
| 05011338 | Bacteria | p__Proteobacteria | c__Gammaproteobacteria | o__Enterobacterales | f__Enterobacteriaceae |  |  |
| 09e9ec0fcdfc24e638fe6ce04862599a | Bacteria | p__Firmicutes | c__Bacilli | o__Lactobacillales | f__Streptococcaceae | g__Lactococcus |  |
| d9379cb38c0841dcf2dd19f30fabc992 | Bacteria | p__Zixibacteria | c__Zixibacteria | o__Zixibacteria | f__Zixibacteria | g__Zixibacteria | s__bacterium_enrichment |
| 04968789 | Bacteria | p__Firmicutes | c__Bacilli | o__Erysipelotrichales | f__Erysipelotrichaceae | g__Faecalibaculum | s__uncultured_bacterium |
| d0183b4836518dfb088e7977b15c0401 | Bacteria | p__Proteobacteria | c__Alphaproteobacteria | o__Rhizobiales | f__Beijerinckiaceae | g__Microvirga |  |
| b704f2a3778238f5c56ad96f7d4b901b | Bacteria | p__Chloroflexi | c__KD4-96 | o__KD4-96 | f__KD4-96 | g__KD4-96 | s__metagenome |
| MF033384 | Bacteria | p__Bacteroidota | c__Bacteroidia | o__Chitinophagales | f__Chitinophagaceae | g__uncultured | s__Panacibacter_sp. |
| bfb8fd28c737cbcd916f245c4b50c369 | Bacteria | p__Firmicutes | c__Clostridia | o__Lachnospirales | f__Lachnospiraceae |  |  |
| 05038880 | Bacteria | p__Bacteroidota | c__Bacteroidia | o__Cytophagales | f__Microscillaceae | g__uncultured | s__uncultured_soil |
| 7a647c63f181be5a3d3bd63dedb8844a | Bacteria | p__Proteobacteria | c__Gammaproteobacteria | o__Oceanospirillales | f__Hahellaceae | g__Hahella | s__uncultured_bacterium |
| ea3ea67755aa77519263771932547d19 | Bacteria | p__Proteobacteria | c__Alphaproteobacteria | o__Caulobacterales | f__Caulobacteraceae |  |  |
| f15a07e1427d242f309202174b4eb0bd | Bacteria | p__Proteobacteria | c__Gammaproteobacteria | o__Burkholderiales | f__Burkholderiaceae | g__Burkholderia-Caballeronia-Paraburkholderia |  |
| 04946343 | Bacteria | p__Actinobacteriota | c__Actinobacteria | o__Micrococcales | f__Dermabacteraceae | g__Brachybacterium |  |
| d291cc8dd7ea9a6366e30b0648e54d87 | Bacteria | p__Firmicutes | c__Bacilli | o__Lactobacillales |  |  |  |
| 00ba17bd6466659ac3a2fa99918ee94e | Bacteria | p__Proteobacteria | c__Alphaproteobacteria | o__Rhodobacterales | f__Rhodobacteraceae | g__Rhodobacter |  |
| f05ce6ab480dc0d00044ca78546effaa | Bacteria | p__Proteobacteria | c__Alphaproteobacteria | o__Caulobacterales | f__Hyphomonadaceae | g__SWB02 | s__uncultured_bacterium |
| d8effa5880171c10735e675ae11a3755 | Bacteria | p__Proteobacteria | c__Gammaproteobacteria | o__Burkholderiales | f__Burkholderiaceae | g__Cupriavidus |  |
| 6cb982603b577195042db06e2430e3f7 | Bacteria | p__Gemmatimonadota | c__Gemmatimonadetes | o__Gemmatimonadales | f__Gemmatimonadaceae | g__uncultured |  |
| 380a91a32134babde19dc532d0346598 | Bacteria | p__Planctomycetota | c__Phycisphaerae | o__Tepidisphaerales | f__WD2101_soil_group | g__WD2101_soil_group |  |
| 624a9fd269c7b107ae7d4e20bb4ab3b7 | Bacteria | p__Firmicutes | c__Clostridia | o__Lachnospirales | f__Lachnospiraceae |  |  |
| bbfa7a6266779b8b3e50298e6eb3fa26 | Bacteria | p__Proteobacteria | c__Gammaproteobacteria | o__Burkholderiales | f__Comamonadaceae |  |  |
| bf845404b002c61dca7e2cdf00a5a62c | Bacteria | p__Actinobacteriota | c__Thermoleophilia | o__Solirubrobacterales | f__67-14 | g__67-14 | s__metagenome |
| GU295962 | Bacteria | p__Bacteroidota | c__Bacteroidia | o__Chitinophagales | f__Chitinophagaceae | g__Flavitalea |  |
| G000285595_1 | Bacteria | p__Firmicutes | c__Bacilli | o__Bacillales | f__Planococcaceae | g__Kurthia |  |
| 7348940b78eee267a33e9df321e8bf4b | Bacteria | p__Proteobacteria | c__Gammaproteobacteria | o__Enterobacterales |  |  |  |
| aaca16964712d8a0ca57ef6cf7eec2e2 | Bacteria | p__Actinobacteriota | c__Acidimicrobiia | o__Microtrichales | f__Microtrichaceae | g__IMCC26207 |  |
| RS-GCF-003353055.1-NZ-QKWJ01000246.1 | Bacteria | p__Proteobacteria | c__Gammaproteobacteria | o__Burkholderiales | f__Burkholderiaceae | g__Cupriavidus |  |
| RS-GCF-002141085.1-NZ-NGLY01000006.1 | Bacteria | p__Firmicutes | c__Bacilli | o__Lactobacillales | f__Enterococcaceae | g__Enterococcus |  |
| e8772b0fe07315207321614ef34dde50 | Bacteria | p__Acidobacteriota | c__Vicinamibacteria | o__Vicinamibacterales | f__uncultured | g__uncultured |  |
| 4deac8b1c1bb4a3cd029c721fccd8c3a | Bacteria | p__Proteobacteria | c__Gammaproteobacteria | o__Xanthomonadales | f__Xanthomonadaceae | g__Stenotrophomonas |  |
| 7399eef1992a6258a356ca7589664518 | Bacteria | p__Actinobacteriota | c__Actinobacteria | o__Micrococcales | f__Intrasporangiaceae |  |  |
| 2a328a10dfac900064b35393c7c01304 | Bacteria | p__Firmicutes | c__Clostridia | o__Oscillospirales | f__Oscillospiraceae | g__uncultured |  |
| 02923458 | Bacteria | p__Planctomycetota | c__Phycisphaerae | o__Tepidisphaerales | f__WD2101_soil_group | g__WD2101_soil_group |  |
| 02405075 | Bacteria | p__Firmicutes | c__Clostridia | o__Lachnospirales | f__Lachnospiraceae | g__Agathobacter |  |
| c6d7e8fd125ca89bc57f9287ad2ee084 | Bacteria | p__Proteobacteria | c__Gammaproteobacteria |  |  |  |  |
| 04907700 | Bacteria | p__Proteobacteria | c__Alphaproteobacteria | o__Caulobacterales | f__Caulobacteraceae |  |  |
| ebb809e1cc3b1ef9aa3911b58ed5660f | Bacteria | p__WPS-2 | c__WPS-2 | o__WPS-2 | f__WPS-2 | g__WPS-2 | s__uncultured_bacterium |
| 3dafa1620907c9a00dc00d6d659878eb | Bacteria | p__Proteobacteria | c__Gammaproteobacteria | o__Pseudomonadales | f__Pseudomonadaceae | g__Pseudomonas |  |
| G000010525_1 | Bacteria | p__Proteobacteria | c__Alphaproteobacteria | o__Rhizobiales | f__Xanthobacteraceae | g__Azorhizobium |  |
| RS-GCF-002803325.1-NZ-MKZM01000016.1 | Bacteria | p__Firmicutes | c__Bacilli | o__Paenibacillales | f__Paenibacillaceae | g__Paenibacillus | s__Paenibacillus_lautus |
| 3aa603ce5d08f2bd7f717feb7c5b943f | Bacteria | p__Firmicutes | c__Bacilli | o__Lactobacillales | f__Enterococcaceae | g__Enterococcus |  |
| 12560f05dd7fa50d2aa67a34d891f626 | Bacteria | p__Proteobacteria | c__Alphaproteobacteria | o__Reyranellales | f__Reyranellaceae | g__Reyranella |  |
| 2cee950f165f4af1769ce3cefce8fd7c | Bacteria | p__Firmicutes | c__Bacilli | o__Erysipelotrichales | f__Erysipelotrichaceae | g__Turicibacter |  |
| 04910974 | Bacteria | p__Proteobacteria | c__Gammaproteobacteria | o__Xanthomonadales | f__Xanthomonadaceae | g__Stenotrophomonas |  |
| ad4c9aef301fc8c3f48d03cd12fe57a9 | Bacteria | p__Proteobacteria | c__Gammaproteobacteria | o__Xanthomonadales | f__Xanthomonadaceae |  |  |
| 655f4e39fc8ea3f99c83119b6b6d09a5 | Bacteria | p__Proteobacteria | c__Alphaproteobacteria | o__Rhizobiales | f__Rhizobiaceae | g__Allorhizobium-Neorhizobium-Pararhizobium-Rhizobium |  |
| RS-GCF-900111905.1-NZ-FOKM01000037.1 | Bacteria | p__Proteobacteria | c__Alphaproteobacteria | o__Rhizobiales | f__Rhizobiaceae | g__Allorhizobium-Neorhizobium-Pararhizobium-Rhizobium |  |
| c2bb465af84bc1df0a0f81a7dc1ebe23 | Bacteria | p__Proteobacteria | c__Alphaproteobacteria | o__Rhizobiales | f__Rhizobiaceae |  |  |
| 4bb340ca55eca6c397e61eba6a2f906d | Bacteria | p__Proteobacteria | c__Alphaproteobacteria | o__Rhizobiales | f__Beijerinckiaceae |  |  |
| b593a49d1df4c4365876f653479458c3 | Bacteria | p__Proteobacteria | c__Alphaproteobacteria | o__Rhizobiales |  |  |  |
| MJ031-1-barcode38-umi19310bins-ubs-20 | Bacteria | p__Firmicutes | c__Bacilli | o__RF39 | f__RF39 | g__RF39 |  |
| 870c26a0dc5ac288c649aeb19f0df4ab | Bacteria | p__Firmicutes | c__Bacilli | o__Erysipelotrichales | f__Erysipelatoclostridiaceae | g__Erysipelatoclostridium | s__uncultured_Clostridium |
| e7374b289af3782e2e5e3ff2cd209b70 | Bacteria | p__Proteobacteria | c__Gammaproteobacteria | o__Pseudomonadales | f__Moraxellaceae | g__Acinetobacter |  |
| 9f6e1990db2df20c01cbf1289e13e0fd | Bacteria | p__Acidobacteriota | c__Vicinamibacteria | o__Vicinamibacterales | f__uncultured | g__uncultured |  |
| KC424519 | Bacteria | p__Proteobacteria | c__Gammaproteobacteria | o__Burkholderiales | f__Comamonadaceae |  |  |
| 0a909a36c5f3097da5126be430378413 | Bacteria | p__Acidobacteriota | c__Aminicenantia | o__Aminicenantales | f__Aminicenantales | g__Aminicenantales | s__uncultured_soil |
| G004803915_4 | Bacteria | p__Bacteroidota | c__Bacteroidia | o__Bacteroidales | f__Muribaculaceae | g__Muribaculaceae | s__uncultured_Bacteroidales |
| MJ010-1-barcode47-umi105838bins-ubs-6 | Bacteria | p__Bacteroidota | c__Bacteroidia | o__Sphingobacteriales | f__Sphingobacteriaceae | g__Sphingobacterium |  |
| MJ006-2-barcode53-umi127626bins-ubs-5 | Bacteria | p__Firmicutes | c__Clostridia | o__Lachnospirales | f__Lachnospiraceae | g__Lachnospiraceae_UCG-003 | s__uncultured_bacterium |
| G000725485_1 | Bacteria | p__Actinobacteriota | c__Actinobacteria | o__Streptosporangiales | f__Streptosporangiaceae | g__Nonomuraea |  |
| aa323df6b73a44a203013434bf9a1a24 | Bacteria | p__Spirochaetota | c__Leptospirae | o__Leptospirales | f__Leptospiraceae | g__Turneriella | s__metagenome |
| 22906df6c71732f2f1bff9e8e937514e | Bacteria | p__Actinobacteriota | c__Thermoleophilia | o__Solirubrobacterales | f__67-14 | g__67-14 |  |
| 4a079a1f5e3e3e60a6607ddac93306e8 | Bacteria | p__Proteobacteria | c__Gammaproteobacteria | o__Burkholderiales | f__Comamonadaceae | g__Pelomonas |  |
| 61ac4869f45d0012f5bede59cae39205 | Bacteria | p__Proteobacteria | c__Alphaproteobacteria | o__Rhizobiales | f__Xanthobacteraceae | g__Rhodoplanes |  |
| 02247771 | Bacteria | p__Proteobacteria | c__Alphaproteobacteria | o__Azospirillales | f__Azospirillaceae | g__Skermanella |  |
| 5ba54484ecfc4d7a6be8ce2569e1bd18 | Bacteria | p__Actinobacteriota | c__Thermoleophilia | o__Solirubrobacterales | f__67-14 | g__67-14 |  |
| 199d83ec0edaee1ec6f24eab073b4430 | Bacteria | p__Firmicutes | c__Bacilli | o__Bacillales | f__Marinococcaceae | g__Alteribacillus |  |
| c7912c2604276d15a7b5382db3b5771c | Bacteria | p__Chloroflexi | c__Chloroflexia | o__Chloroflexales | f__Roseiflexaceae | g__uncultured |  |
| 4371754cd454b6cae9d5caca47585f18 | Bacteria | p__Actinobacteriota | c__Actinobacteria | o__Corynebacteriales | f__Mycobacteriaceae | g__Mycobacterium |  |
| 5e17b3333e26bc0ed14137760ce1b92f | Bacteria | p__Actinobacteriota | c__Actinobacteria | o__Propionibacteriales | f__Nocardioidaceae | g__Aeromicrobium |  |
| abebaa50549b45d622414668458c8068 | Bacteria | p__Actinobacteriota | c__Actinobacteria | o__Frankiales | f__Nakamurellaceae | g__Nakamurella | s__Sanguibacter_sp. |
| 307617fbf5c0255259926e3d913ae0ee | Bacteria | p__Bacteroidota | c__Bacteroidia | o__Flavobacteriales | f__Flavobacteriaceae | g__Myroides |  |
| 051a7253597aefd33ea32e752b548c2e | Bacteria | p__Bacteroidota | c__Bacteroidia | o__Flavobacteriales | f__Weeksellaceae | g__Chryseobacterium | s__Chryseobacterium_taeanense |
| 59ba189ac4d5b537e496b5dabf92b170 | Bacteria | p__Actinobacteriota | c__Acidimicrobiia | o__IMCC26256 | f__IMCC26256 | g__IMCC26256 | s__uncultured_Ferrimicrobium |
| f44ebfe4a3ae8ced8410a170e38dbc05 | Bacteria | p__Proteobacteria | c__Gammaproteobacteria | o__Burkholderiales | f__Comamonadaceae | g__Curvibacter |  |
| 1033b0dae1f3ff14d4487c0164a1c0b4 | Bacteria | p__Acidobacteriota | c__Acidobacteriae | o__Solibacterales | f__Solibacteraceae | g__Candidatus_Solibacter |  |
| 04966142 | Bacteria | p__Firmicutes | c__Bacilli | o__Bacillales | f__Bacillaceae | g__Bacillus |  |
| 02267006 | Bacteria | p__Proteobacteria | c__Alphaproteobacteria | o__Rhizobiales | f__Rhizobiaceae | g__Allorhizobium-Neorhizobium-Pararhizobium-Rhizobium |  |
| 11153261 | Bacteria | p__Bacteroidota | c__Bacteroidia | o__Bacteroidales | f__Muribaculaceae | g__Muribaculaceae |  |
| 834c03e8a6bc27fc3cd25a2172e75299 | Bacteria | p__Acidobacteriota | c__Blastocatellia | o__Pyrinomonadales | f__Pyrinomonadaceae | g__RB41 |  |
| 5a2e28361502ee99016ec1cea6ff9476 | Bacteria | p__Actinobacteriota | c__Actinobacteria | o__Bifidobacteriales | f__Bifidobacteriaceae | g__Gardnerella |  |
| edef67784fd0cca89f095c9d634f320d | Bacteria | p__Proteobacteria | c__Gammaproteobacteria | o__Pasteurellales | f__Pasteurellaceae |  |  |
| db5fe493964c69ffc84f16a96f83c304 | Bacteria | p__Firmicutes | c__Clostridia | o__Lachnospirales | f__Lachnospiraceae |  |  |
| dffe341cff4dbd627ef0a40956c30552 | Bacteria | p__Bacteroidota | c__Bacteroidia | o__Bacteroidales | f__Rikenellaceae | g__DMER64 |  |
| RS-GCF-014193785.1-NZ-BLZI01000049.1 | Bacteria | p__Proteobacteria | c__Alphaproteobacteria | o__Rhizobiales | f__Rhizobiaceae |  |  |
| GB-GCA-910577715.1-CAJTOQ010000285.1 | Bacteria | p__Bacteroidota | c__Bacteroidia | o__Bacteroidales | f__Bacteroidaceae | g__Bacteroides |  |
| 04878173 | Bacteria | p__Proteobacteria | c__Gammaproteobacteria | o__Burkholderiales | f__Burkholderiaceae | g__Lautropia |  |
| 8667ab2452f6f713cd809ece847957fd | Bacteria | p__Actinobacteriota | c__Actinobacteria | o__Frankiales | f__Sporichthyaceae | g__Longivirga |  |
| 05019631 | Bacteria | p__Acidobacteriota | c__Acidobacteriae | o__Bryobacterales | f__Bryobacteraceae | g__Bryobacter |  |
| FM882231 | Bacteria | p__Actinobacteriota | c__Actinobacteria | o__Micromonosporales | f__Micromonosporaceae | g__Dactylosporangium |  |
| 04983357 | Bacteria | p__Actinobacteriota | c__Actinobacteria | o__Frankiales | f__Acidothermaceae | g__Acidothermus |  |
| 19396671350a44bbc57aa0babcd795ad | Bacteria | p__Entotheonellaeota | c__Entotheonellia | o__Entotheonellales | f__Entotheonellaceae | g__Entotheonellaceae |  |
| e46017b7bdccda42eeb8197eaeaca680 | Bacteria | p__Proteobacteria | c__Alphaproteobacteria | o__Caulobacterales | f__Caulobacteraceae |  |  |
| 5e990a51cbeae62b4c660db8b133f26a | Bacteria |  |  |  |  |  |  |
| KX009027 | Bacteria | p__Actinobacteriota | c__Actinobacteria | o__Propionibacteriales | f__Propionibacteriaceae | g__Propioniciclava | s__Propioniciclava_sinopodophylli |
| 9e772577febbecc44730e1ab94924164 | Bacteria | p__Proteobacteria | c__Gammaproteobacteria | o__Burkholderiales | f__Methylophilaceae | g__Methylobacillus |  |
| 2b146071d425d2a3481111c9d32439ec | Bacteria | p__Firmicutes | c__Clostridia | o__Lachnospirales | f__Lachnospiraceae | g__Agathobacter |  |
| 11c273bc15fd508960fdbc63a879251f | Bacteria | p__Actinobacteriota | c__Actinobacteria | o__Micrococcales |  |  |  |
| MJ007-1-barcode49-umi43073bins-ubs-3 | Bacteria | p__Bacteroidota | c__Bacteroidia | o__Bacteroidales | f__Marinilabiliaceae | g__Ruminofilibacter |  |
| b2c785120fc7f3a37c53517059b84c75 | Bacteria | p__Proteobacteria |  |  |  |  |  |
| a7fd13a99814bc1cdc6a3f43320538e7 | Bacteria | p__Firmicutes | c__Bacilli | o__Staphylococcales | f__Staphylococcaceae | g__Corticicoccus | s__Corticicoccus_populi |
| 0fa85d0d14b78ad5161534876512e1cf | Bacteria | p__Proteobacteria | c__Gammaproteobacteria | o__Burkholderiales | f__Rhodocyclaceae | g__Thauera |  |
| 7c7721a99d764a14c388c448c45a48c7 | Bacteria | p__Bacteroidota | c__Bacteroidia | o__Bacteroidales | f__Bacteroidetes_vadinHA17 | g__Bacteroidetes_vadinHA17 | s__uncultured_bacterium |
| b54ff79e0c3709872672c395d331c0b7 | Bacteria | p__Acidobacteriota | c__Blastocatellia | o__DS-100 | f__DS-100 | g__DS-100 | s__uncultured_bacterium |
| 59f513a92df0bf3879becbca395dd7f9 | Bacteria | p__Actinobacteriota | c__Coriobacteriia | o__Coriobacteriales | f__Coriobacteriaceae | g__Collinsella |  |
| a5079b21c99f588899eca286fb68f096 | Bacteria | p__Firmicutes | c__Bacilli | o__Lactobacillales | f__Lactobacillaceae | g__Lactobacillus |  |
| 2e0fdf72be779ab79179e7091cc9a3a3 | Bacteria | p__Actinobacteriota | c__Thermoleophilia | o__Gaiellales | f__uncultured | g__uncultured |  |
| e1ddf2a1e03243ee78ec92f7eec6e39f | Bacteria | p__Proteobacteria | c__Gammaproteobacteria | o__Burkholderiales | f__TRA3-20 | g__TRA3-20 |  |
| 08176610 | Bacteria | p__Proteobacteria | c__Alphaproteobacteria | o__Rhizobiales | f__Beijerinckiaceae | g__alphaI_cluster |  |
| MJ006-2-barcode69-umi136916bins-ubs-4 | Bacteria | p__Firmicutes | c__Clostridia | o__Oscillospirales | f__Ruminococcaceae | g__Faecalibacterium |  |
| MJ034-1-barcode26-umi26181bins-ubs-5 | Bacteria | p__Acidobacteriota | c__Blastocatellia | o__Pyrinomonadales | f__Pyrinomonadaceae | g__RB41 |  |
| 12aed87f19099c1df955e83a19b410c6 | Bacteria | p__Patescibacteria | c__Saccharimonadia | o__Saccharimonadales | f__Saccharimonadales | g__Saccharimonadales |  |
| RS-GCF-003477445.1-NZ-QUDH01000034.1 | Bacteria | p__Firmicutes | c__Clostridia | o__Lachnospirales | f__Lachnospiraceae |  |  |
| b6b5bf71319c8e955ec37494dff979ff | Bacteria | p__Proteobacteria | c__Alphaproteobacteria | o__Rhodobacterales | f__Rhodobacteraceae |  |  |
| 14871243 | Bacteria | p__Chloroflexi | c__Ktedonobacteria | o__Ktedonobacterales | f__JG30-KF-AS9 | g__JG30-KF-AS9 | s__uncultured_bacterium |
| 5e3d23af3953bfa872bb01f71631ea6d | Bacteria | p__Actinobacteriota | c__MB-A2-108 | o__MB-A2-108 | f__MB-A2-108 | g__MB-A2-108 | s__uncultured_Firmicutes |
| 55ff98187e9ace1d0959f14f91c6cdb4 | Bacteria | p__Actinobacteriota | c__Acidimicrobiia | o__Microtrichales | f__Ilumatobacteraceae |  |  |
| MJ007-2-barcode74-umi14600bins-ubs-23 | Bacteria | p__Proteobacteria | c__Alphaproteobacteria | o__Micropepsales | f__Micropepsaceae | g__uncultured |  |
| RS-GCF-008764045.1-NZ-VTQK01000028.1 | Bacteria | p__Firmicutes | c__Bacilli | o__Staphylococcales | f__Staphylococcaceae | g__Staphylococcus |  |
| 04876765 | Bacteria | p__Proteobacteria | c__Alphaproteobacteria | o__Sphingomonadales | f__Sphingomonadaceae | g__Sphingomonas |  |
| 05035665 | Bacteria | p__Firmicutes | c__Clostridia | o__Oscillospirales | f__Oscillospiraceae | g__Colidextribacter |  |
| KT301938 | Bacteria | p__Actinobacteriota | c__Actinobacteria | o__Corynebacteriales | f__Nocardiaceae | g__Rhodococcus |  |
| 67f7be0e6dcaeefa5ff335ad6971599b | Bacteria | p__Actinobacteriota | c__Thermoleophilia | o__Gaiellales | f__uncultured | g__uncultured | s__uncultured_Alphaproteobacteria |
| MJ034-1-barcode31-umi27691bins-ubs-3 | Bacteria | p__Acidobacteriota | c__Acidobacteriae | o__Solibacterales | f__Solibacteraceae | g__Candidatus_Solibacter |  |
| 59bece86452d976c2853b1d943afb92c | Bacteria | p__Actinobacteriota | c__Thermoleophilia | o__Gaiellales | f__uncultured | g__uncultured |  |
| MJ006-1-barcode43-umi162497bins-ubs-3 | Bacteria | p__Firmicutes | c__Clostridia | o__Lachnospirales | f__Lachnospiraceae |  |  |
| bb7ffc35ca66199dd45ee66b029d8be5 | Bacteria | p__Firmicutes | c__Clostridia | o__Lachnospirales | f__Lachnospiraceae |  |  |
| 11844412 | Bacteria | p__Proteobacteria | c__Alphaproteobacteria | o__Sphingomonadales | f__Sphingomonadaceae | g__Sphingomonas |  |
| 14422577 | Bacteria | p__Acidobacteriota | c__Vicinamibacteria | o__Vicinamibacterales | f__uncultured | g__uncultured |  |
| 04930760 | Bacteria | p__Proteobacteria | c__Gammaproteobacteria | o__Enterobacterales | f__Enterobacteriaceae |  |  |
| 04946695 | Bacteria | p__Actinobacteriota | c__Actinobacteria | o__Actinomycetales | f__Actinomycetaceae | g__Actinomyces |  |
| 79d981e73c0ad93f6839c3ca33118167 | Bacteria | p__Chloroflexi | c__Anaerolineae | o__1-20 | f__1-20 | g__1-20 | s__uncultured_Chloroflexia |
| 02266060 | Bacteria | p__Actinobacteriota | c__Actinobacteria | o__Frankiales | f__Frankiales | g__Frankiales | s__uncultured_bacterium |
| 595bb989fdbaf1d0ff065a6262988794 | Bacteria | p__Proteobacteria | c__Gammaproteobacteria | o__Enterobacterales | f__Enterobacteriaceae |  |  |
| 1dcef2e1f59016a1a10176117329baf4 | Bacteria | p__Proteobacteria | c__Alphaproteobacteria | o__Dongiales | f__Dongiaceae | g__Dongia |  |
| d61e96f96bc290648701af91de7ca639 | Bacteria | p__Proteobacteria | c__Alphaproteobacteria | o__Sphingomonadales | f__Sphingomonadaceae | g__Sphingomonas |  |
| 3d4380c8229afcc971e9ac00793c7581 | Bacteria | p__Firmicutes | c__Bacilli | o__Erysipelotrichales | f__Erysipelotrichaceae | g__Faecalibaculum | s__uncultured_bacterium |
| 1cd0c07426fe635c9f34e3ff8cb42e87 | Bacteria | p__Proteobacteria | c__Alphaproteobacteria | o__Rhizobiales | f__Rhizobiales_Incertae_Sedis |  |  |
| 6aa3033de63e4e7a0361075ddf18e8ef | Bacteria | p__Bacteroidota | c__Bacteroidia | o__Chitinophagales | f__Chitinophagaceae |  |  |
| fc6d3486a67ead3f7eb8600c3fe05e4f | Bacteria | p__Bdellovibrionota | c__Bdellovibrionia | o__Bacteriovoracales | f__Bacteriovoracaceae | g__Peredibacter | s__metagenome |
| 8861ea93e4ae7670fedfc1a38a2ae028 | Bacteria | p__Actinobacteriota | c__Actinobacteria | o__Pseudonocardiales | f__Pseudonocardiaceae | g__Pseudonocardiaceae | s__Bounagaea_algeriensis |
| 19976106 | Bacteria | p__Firmicutes | c__Clostridia | o__Oscillospirales | f__Oscillospiraceae | g__Intestinimonas |  |
| 9b8be6d9b13093f6b7296549bcf5d611 | Bacteria | p__Firmicutes | c__Bacilli | o__Lactobacillales | f__Enterococcaceae | g__Enterococcus |  |
| MJ020-2-barcode70-umi55535bins-ubs-8 | Bacteria | p__Bacteroidota | c__Bacteroidia | o__Sphingobacteriales | f__Sphingobacteriaceae | g__Sphingobacterium | s__Sphingobacterium_nematocida |
| 6fbf67319d2f612500f084eb4198fb96 | Bacteria | p__Proteobacteria | c__Alphaproteobacteria | o__Rhizobiales | f__Xanthobacteraceae | g__uncultured |  |
| AM040493 | Bacteria | p__Actinobacteriota | c__Actinobacteria | o__Micrococcales | f__Microbacteriaceae |  |  |
| 04933459 | Bacteria | p__Bacteroidota | c__Bacteroidia | o__Chitinophagales | f__Chitinophagaceae | g__Sediminibacterium |  |
| da262c1521f6f5b949604810b6605be8 | Bacteria | p__Proteobacteria | c__Alphaproteobacteria | o__Sphingomonadales | f__Sphingomonadaceae |  |  |
| RS-GCF-012956805.1-NZ-JABBZM010000054.1 | Bacteria | p__Proteobacteria | c__Gammaproteobacteria | o__Burkholderiales | f__Burkholderiaceae | g__Ralstonia |  |
| MJ010-1-barcode26-umi117398bins-ubs-5 | Bacteria | p__Proteobacteria | c__Alphaproteobacteria | o__Rhizobiales | f__Devosiaceae | g__Devosia |  |
| FJ169465 | Bacteria | p__Firmicutes | c__Bacilli | o__Bacillales | f__Planococcaceae | g__Lysinibacillus |  |
| 2ecee532840b911881d2027f492b9147 | Bacteria | p__Proteobacteria | c__Alphaproteobacteria | o__Rhizobiales | f__Xanthobacteraceae | g__Pseudolabrys |  |
| 04912646 | Bacteria | p__Acidobacteriota | c__Acidobacteriae | o__Acidobacteriales | f__Acidobacteriaceae_(Subgroup_1) | g__Granulicella |  |
| 08095268 | Bacteria | p__Proteobacteria | c__Alphaproteobacteria | o__Dongiales | f__Dongiaceae | g__Dongia |  |
| deb0379bb9696049dd999a211c9b3570 | Bacteria | p__Proteobacteria | c__Alphaproteobacteria | o__Rhizobiales | f__Amb-16S-1323 | g__Amb-16S-1323 | s__uncultured_proteobacterium |
| f341a0d1dc3dd48f1b21e85e00542805 | Bacteria | p__Patescibacteria | c__Saccharimonadia | o__Saccharimonadales | f__Saccharimonadales | g__Saccharimonadales |  |
| AB245358 | Bacteria | p__Proteobacteria | c__Gammaproteobacteria | o__Burkholderiales | f__Comamonadaceae | g__Variovorax |  |
| e16dc70201fae9be973bd59797dda5e1 | Bacteria | p__Actinobacteriota | c__Actinobacteria | o__Micrococcales | f__Micrococcaceae |  |  |
| AB214328 | Bacteria | p__Bacteroidota | c__Bacteroidia | o__Bacteroidales | f__Bacteroidaceae | g__Bacteroides |  |
| 761e24c9414d53dd1c96909f195ac93c | Bacteria | p__Chloroflexi | c__Anaerolineae | o__Anaerolineales | f__Anaerolineaceae | g__uncultured |  |
| G000374625_1 | Bacteria | p__Proteobacteria | c__Gammaproteobacteria | o__Burkholderiales | f__Comamonadaceae |  |  |
| 04994720 | Bacteria | p__Proteobacteria | c__Alphaproteobacteria | o__Rhizobiales | f__Beijerinckiaceae | g__Microvirga |  |
| 19706754 | Bacteria | p__Firmicutes | c__Bacilli | o__Erysipelotrichales | f__Erysipelotrichaceae | g__Ileibacterium | s__Ileibacterium_valens |
| 0c5dcbad84c3340e2683e0317994a0ca | Bacteria | p__Chloroflexi | c__Anaerolineae | o__Anaerolineales | f__Anaerolineaceae | g__uncultured |  |
| bb8d18eb30fc4083d9214544befb9ddc | Bacteria | p__Proteobacteria | c__Gammaproteobacteria | o__Burkholderiales | f__Comamonadaceae | g__Diaphorobacter | s__uncultured_compost |
| 322f34e6a679c16c12f92e75e650aa01 | Bacteria | p__Proteobacteria | c__Alphaproteobacteria | o__Rhizobiales | f__Beijerinckiaceae | g__Methylobacterium-Methylorubrum |  |
| 783b63033fdf5bb614c3c15a75cbadbb | Bacteria | p__Proteobacteria | c__Gammaproteobacteria | o__Xanthomonadales | f__Rhodanobacteraceae |  |  |
| MJ034-2-barcode72-umi1284bins-ubs-318 | Bacteria | p__Bacteroidota | c__Bacteroidia | o__Chitinophagales | f__Chitinophagaceae | g__Segetibacter |  |
| KR611794 | Bacteria | p__Proteobacteria | c__Gammaproteobacteria | o__Pseudomonadales | f__Moraxellaceae | g__Acinetobacter |  |
| 375db154838ceb9b1d5091f33dca7388 | Bacteria | p__Proteobacteria | c__Gammaproteobacteria | o__Burkholderiales | f__Rhodocyclaceae |  |  |
| 0d828082831d9689b23fb0da4e64c075 | Bacteria | p__Desulfobacterota | c__uncultured | o__uncultured | f__uncultured | g__uncultured |  |
| 17968781 | Bacteria | p__Chloroflexi | c__Ktedonobacteria | o__Ktedonobacterales | f__Ktedonobacteraceae | g__HSB_OF53-F07 | s__uncultured_bacterium |
| 02f84dd4dad7a5079e5ba73897a3dc13 | Bacteria |  |  |  |  |  |  |
| 9a15691ee6237e8e0314d3baab735b47 | Bacteria | p__Verrucomicrobiota | c__Verrucomicrobiae | o__Chthoniobacterales | f__Chthoniobacteraceae | g__Candidatus_Udaeobacter |  |
| 637a7ee3fa51a7a76fb910edde7f07ea | Bacteria | p__Bacteroidota | c__Bacteroidia | o__Flavobacteriales | f__Flavobacteriaceae | g__Myroides |  |
| RS-GCF-013179615.1-NZ-JABMCO010000328.1 | Bacteria | p__Actinobacteriota | c__Actinobacteria | o__Micrococcales | f__Micrococcaceae | g__Glutamicibacter |  |
| 37a9e67db3dcfb3e09a3cc7b49ba2aac | Bacteria | p__Actinobacteriota | c__Acidimicrobiia | o__IMCC26256 | f__IMCC26256 | g__IMCC26256 |  |
| ead439db399a276fefbd9aa43bd0857f | Bacteria | p__Actinobacteriota | c__Coriobacteriia | o__Coriobacteriales | f__Eggerthellaceae |  |  |
| 5045ddb0e60154e9f14a36070bf8a1ae | Bacteria | p__Actinobacteriota | c__Thermoleophilia | o__Solirubrobacterales | f__Solirubrobacteraceae | g__Conexibacter | s__uncultured_bacterium |
| 858f1f0ed102463c87cb030d730414b9 | Bacteria | p__Acidobacteriota | c__Acidobacteriae |  |  |  |  |
| cbc57ffa969c8ee0591dbae68905571b | Bacteria | p__Verrucomicrobiota | c__Verrucomicrobiae | o__Pedosphaerales | f__Pedosphaeraceae | g__Pedosphaeraceae |  |
| 08627674 | Bacteria | p__Proteobacteria | c__Alphaproteobacteria | o__Rhizobiales | f__Rhizobiaceae | g__Paenochrobactrum |  |
| MJ006-1-barcode39-umi242745bins-ubs-3 | Bacteria | p__Firmicutes | c__Clostridia | o__Oscillospirales | f__Ruminococcaceae | g__CAG-352 | s__uncultured_bacterium |
| beb21060619c0ae4879749bdc54fdeb8 | Bacteria | p__NB1-j | c__NB1-j | o__NB1-j | f__NB1-j | g__NB1-j | s__uncultured_soil |
| 04893559 | Bacteria | p__Myxococcota | c__Polyangia | o__Polyangiales | f__Polyangiaceae | g__uncultured | s__uncultured_delta |
| b9d33a0598b0b7e6f66effede189e077 | Bacteria | p__Proteobacteria | c__Alphaproteobacteria | o__Caulobacterales | f__Caulobacteraceae | g__Phenylobacterium |  |
| ff2b951ab85f414a72bbd18a082678b8 | Bacteria | p__Actinobacteriota | c__Actinobacteria | o__Micrococcales | f__Microbacteriaceae | g__Leucobacter | s__Leucobacter_holotrichiae |
| 04912566 | Bacteria | p__Acidobacteriota | c__Acidobacteriae | o__Acidobacteriales | f__Acidobacteriaceae_(Subgroup_1) | g__Edaphobacter |  |
| 1aa2222f64a9ffb3653603ee6588ac47 | Bacteria | p__Myxococcota | c__Polyangia | o__Haliangiales | f__Haliangiaceae | g__Haliangium |  |
| MJ034-1-barcode40-umi28905bins-ubs-3 | Bacteria | p__Myxococcota | c__bacteriap25 | o__bacteriap25 | f__bacteriap25 | g__bacteriap25 | s__uncultured_soil |
| G009774865_1 | Bacteria | p__Bacteroidota | c__Bacteroidia | o__Bacteroidales | f__Rikenellaceae | g__Alistipes | s__uncultured_bacterium |
| 04928039 | Bacteria | p__Proteobacteria | c__Alphaproteobacteria | o__Sphingomonadales | f__Sphingomonadaceae | g__Sphingomonas |  |
| 13720086 | Bacteria | p__Gemmatimonadota | c__Gemmatimonadetes | o__Gemmatimonadales | f__Gemmatimonadaceae | g__Gemmatimonas |  |
| b04814a77b2afbed90a8a6e9ad39ab8c | Bacteria | p__Proteobacteria | c__Gammaproteobacteria | o__Burkholderiales | f__Comamonadaceae |  |  |
| 6423b8547bfa4d57b4cae4f55702ee7e | Bacteria | p__Chloroflexi | c__OLB14 | o__OLB14 | f__OLB14 | g__OLB14 | s__uncultured_bacterium |
| 3c0b3b5d6d5ae355c0e0aafd015e6e27 | Bacteria | p__Chloroflexi |  |  |  |  |  |
| b03cbb60333a095004c08f3024ccfeb0 | Bacteria | p__Chloroflexi | c__Ktedonobacteria | o__Ktedonobacterales | f__Ktedonobacteraceae | g__uncultured | s__uncultured_Thermosporothrix |
| 04927896 | Bacteria | p__Proteobacteria | c__Alphaproteobacteria | o__Sphingomonadales | f__Sphingomonadaceae | g__Sphingomonas |  |
| 9393ea9f44a945f9b3c5baa14c8f2318 | Bacteria | p__Proteobacteria | c__Gammaproteobacteria | o__Burkholderiales | f__Oxalobacteraceae | g__Oxalicibacterium | s__Oxalicibacterium_faecigallinarum |
| 0f8cf8e1e04106e662930e42bac44f29 | Bacteria | p__Actinobacteriota | c__Acidimicrobiia | o__IMCC26256 | f__IMCC26256 | g__IMCC26256 |  |
| 04881143 | Bacteria | p__Firmicutes | c__Clostridia | o__Lachnospirales | f__Lachnospiraceae | g__Blautia | s__Lachnospiraceae_bacterium |
| 88fb1a770599c8dd441a24c5ec825433 | Bacteria | p__Acidobacteriota | c__Vicinamibacteria | o__Vicinamibacterales | f__uncultured | g__uncultured |  |
| 07423498 | Bacteria | p__Firmicutes | c__Clostridia | o__Clostridia_UCG-014 | f__Clostridia_UCG-014 | g__Clostridia_UCG-014 |  |
| AB245367 | Bacteria | p__Proteobacteria | c__Gammaproteobacteria | o__Xanthomonadales | f__Rhodanobacteraceae | g__Dyella |  |
| 19706746 | Bacteria | p__Firmicutes | c__Bacilli | o__Erysipelotrichales | f__Erysipelotrichaceae | g__Ileibacterium | s__Ileibacterium_valens |
| 05038609 | Bacteria | p__Firmicutes | c__Bacilli | o__Lactobacillales | f__Lactobacillaceae | g__Lactobacillus |  |
| b44e34b106e5902a801a5d68a2c2bf3d | Bacteria | p__Proteobacteria | c__Gammaproteobacteria | o__Steroidobacterales | f__Steroidobacteraceae | g__Steroidobacter |  |
| d616a1db32fa2a66746df9675bc223cf | Bacteria | p__Chloroflexi | c__KD4-96 | o__KD4-96 | f__KD4-96 | g__KD4-96 |  |
| fd238c28f9f868e126cd25098ca1b3cf | Bacteria | p__Firmicutes | c__Bacilli | o__Lactobacillales | f__Enterococcaceae | g__Enterococcus |  |
| 11843384 | Bacteria | p__Proteobacteria | c__Alphaproteobacteria | o__Sphingomonadales | f__Sphingomonadaceae | g__Sphingomonas |  |
| b9ad1b606c21eafa7be4366ae40df9d5 | Bacteria | p__Gemmatimonadota | c__Gemmatimonadetes | o__Gemmatimonadales | f__Gemmatimonadaceae |  |  |
| c4cd122b7b97c7322cf28660245f4b12 | Bacteria | p__Firmicutes | c__Bacilli | o__Lactobacillales | f__Enterococcaceae | g__Enterococcus |  |
| 0765f82b0f96cecb0017d0ee2a42b64d | Bacteria | p__Actinobacteriota | c__Thermoleophilia | o__Gaiellales |  |  |  |
| 7e53b041ee5bc778be9915558caed737 | Bacteria | p__Proteobacteria | c__Gammaproteobacteria | o__Methylococcales | f__Methylococcaceae |  |  |
| 93f1cb6faf0f1d07188e89cd441ccdb4 | Bacteria | p__Proteobacteria | c__Gammaproteobacteria | o__Burkholderiales | f__Nitrosomonadaceae | g__MND1 |  |
| f782501a88418d6bd021990c0fd5ec8e | Bacteria | p__Bacteroidota | c__Bacteroidia | o__Bacteroidales | f__Bacteroidaceae | g__Bacteroides |  |
| c19c436499f30a5a4f5da687f38bd335 | Bacteria | p__Proteobacteria | c__Gammaproteobacteria | o__Enterobacterales |  |  |  |
| LT545981 | Bacteria | p__Proteobacteria | c__Alphaproteobacteria | o__Rhizobiales | f__Rhizobiaceae |  |  |
| RS-GCF-001485005.1-NZ-BCMA01000016.1 | Bacteria | p__Proteobacteria | c__Gammaproteobacteria | o__Pseudomonadales | f__Moraxellaceae | g__Acinetobacter |  |
| GB-GCA-016794805.1-JAEUJI010000092.1 | Bacteria | p__Gemmatimonadota | c__Gemmatimonadetes | o__Gemmatimonadales | f__Gemmatimonadaceae | g__uncultured |  |
| 422e04428d1a43defd184caad9915b0d | Bacteria | p__Bacteroidota | c__Ignavibacteria | o__Ignavibacteriales | f__PHOS-HE36 | g__PHOS-HE36 |  |
| 02d0eb76fac31f112b2da1983f18ee16 | Bacteria | p__Bacteroidota | c__Bacteroidia | o__Flavobacteriales | f__Flavobacteriaceae |  |  |
| 20c4952434d4cc770efe6492a88d294c | Bacteria | p__Proteobacteria | c__Gammaproteobacteria | o__Gammaproteobacteria_Incertae_Sedis | f__Unknown_Family | g__Acidibacter |  |
| 05007870 | Bacteria | p__Proteobacteria | c__Alphaproteobacteria | o__Sphingomonadales | f__Sphingomonadaceae | g__Sphingomonas |  |
| 17031761 | Bacteria | p__Firmicutes | c__Clostridia | o__Lachnospirales | f__Lachnospiraceae | g__[Eubacterium]_xylanophilum_group | s__uncultured_bacterium |
| 5d9bbe807888bcf79f66bfaa9bdeae16 | Bacteria | p__Acidobacteriota | c__Acidobacteriae | o__Acidobacteriales |  |  |  |
| MJ030-2-barcode60-umi63906bins-ubs-9 | Bacteria | p__Bdellovibrionota | c__Oligoflexia | o__0319-6G20 | f__0319-6G20 | g__0319-6G20 | s__uncultured_Roseobacter |
| 5ebb200ff8141a0d5edda78f65be1586 | Bacteria | p__Proteobacteria | c__Gammaproteobacteria | o__Burkholderiales | f__Comamonadaceae | g__Aquabacterium |  |
| 54f3a8890cb4d43635eddd50800b0215 | Bacteria | p__Chloroflexi | c__Anaerolineae | o__SBR1031 | f__SBR1031 | g__SBR1031 | s__metagenome |
| RS-GCF-001510975.1-NZ-CZPS01000041.1 | Bacteria | p__Firmicutes | c__Bacilli | o__Lactobacillales | f__Lactobacillaceae | g__Lactobacillus |  |
| 95d9a0ee639829215f4afa6a199afccf | Bacteria | p__Myxococcota | c__Polyangia | o__Polyangiales | f__Polyangiaceae | g__Pajaroellobacter |  |
| 55ee7803665e50ce3fe02a85361c0bf3 | Bacteria | p__Firmicutes | c__Bacilli | o__Bacillales | f__Bacillaceae | g__Bacillus |  |
| a1963aa95e6f0634e63f5a4a1439f241 | Bacteria | p__Actinobacteriota | c__Actinobacteria | o__Frankiales | f__Frankiaceae | g__Jatrophihabitans |  |
| 376c5e685664d9467a3850ad1a5d89ec | Bacteria | p__Firmicutes | c__Clostridia | o__Clostridiales | f__Clostridiaceae |  |  |
| AY444822 | Bacteria | p__Proteobacteria | c__Gammaproteobacteria | o__Pseudomonadales | f__Moraxellaceae | g__Psychrobacter |  |
| ff60eac2ecf61560acb451adaba827c2 | Bacteria | p__Actinobacteriota | c__Actinobacteria | o__Micrococcales | f__Microbacteriaceae |  |  |
| 3747bf8ea0257cbfb1303f424dac807d | Bacteria | p__Proteobacteria | c__Gammaproteobacteria | o__Enterobacterales |  |  |  |
| 99794ee375d0feb0f552c33b00b1478a | Bacteria | p__Actinobacteriota | c__Thermoleophilia | o__Solirubrobacterales | f__67-14 | g__67-14 | s__Solirubrobacterales_bacterium |
| 7cbb6b6255d20b329f2a6ccecc789fde | Bacteria | p__Desulfobacterota | c__Desulfovibrionia | o__Desulfovibrionales | f__Desulfovibrionaceae | g__Desulfovibrio | s__uncultured_bacterium |
| e08d1c63d3f807f284efd59c213f343a | Bacteria | p__Chloroflexi | c__Ktedonobacteria | o__Ktedonobacterales | f__JG30-KF-AS9 | g__JG30-KF-AS9 |  |
| 4721dd4f6a30d4f08d20dc3df98b43a0 | Bacteria | p__Myxococcota | c__Myxococcia | o__Myxococcales | f__Myxococcaceae |  |  |
| 81ec242c338240acad98114128faf3be | Bacteria | p__Proteobacteria | c__Alphaproteobacteria | o__Caulobacterales | f__Caulobacteraceae | g__Brevundimonas |  |
| MJ030-2-barcode59-umi30199bins-ubs-25 | Bacteria | p__Bacteroidota | c__Bacteroidia | o__Chitinophagales | f__Chitinophagaceae | g__Lacibacter | s__uncultured_Chitinophagaceae |
| 1db1b58c2469783a8725359509c29b9e | Bacteria | p__Actinobacteriota | c__Thermoleophilia | o__Gaiellales | f__uncultured | g__uncultured |  |
| RS-GCF-002023065.1-NZ-MAIC01000006.1 | Bacteria | p__Bacteroidota | c__Bacteroidia | o__Flavobacteriales | f__Weeksellaceae | g__Elizabethkingia |  |
| 6e5acc774f5bc8aafe5d7bd0e5b32a63 | Bacteria | p__Verrucomicrobiota | c__Verrucomicrobiae | o__Verrucomicrobiales | f__Verrucomicrobiaceae | g__Verrucomicrobium |  |
| 04309643 | Bacteria | p__WPS-2 | c__WPS-2 | o__WPS-2 | f__WPS-2 | g__WPS-2 | s__uncultured_bacterium |
| 128f04070cda0ca65b50ae728e60fac3 | Bacteria | p__Proteobacteria | c__Gammaproteobacteria | o__Burkholderiales | f__Nitrosomonadaceae | g__Ellin6067 |  |
| 09088389 | Bacteria | p__Nitrospirota | c__Nitrospiria | o__Nitrospirales | f__Nitrospiraceae | g__Nitrospira |  |
| 3b6ad2218df7c68b4fc7bbd1f6e95370 | Bacteria | p__Proteobacteria | c__Alphaproteobacteria | o__Acetobacterales | f__Acetobacteraceae |  |  |
| 04878346 | Bacteria | p__Actinobacteriota | c__Actinobacteria | o__Corynebacteriales | f__Corynebacteriaceae | g__Corynebacterium |  |
| 89149f954d5a0b04a125643dc355cde2 | Bacteria | p__Firmicutes | c__Clostridia | o__Clostridiales | f__Clostridiaceae | g__Clostridium_sensu_stricto_1 |  |
| cc3101668bf11a040780cd3dd678951a | Bacteria | p__Actinobacteriota | c__Actinobacteria | o__Bifidobacteriales | f__Bifidobacteriaceae | g__Bifidobacterium |  |
| 8bab3cac4b0ccef4d3ca2605e703b5a9 | Bacteria | p__Firmicutes | c__Bacilli | o__Exiguobacterales | f__Exiguobacteraceae | g__Exiguobacterium |  |
| dedfc49167c185bffa2306690bf4488f | Bacteria | p__Actinobacteriota | c__Actinobacteria |  |  |  |  |
| 9488c8a8c0c99d31488a3e9faf57f7ee | Bacteria | p__Actinobacteriota | c__Actinobacteria | o__Propionibacteriales | f__Nocardioidaceae | g__Nocardioides |  |
| 20949332 | Bacteria | p__Firmicutes | c__Bacilli | o__Erysipelotrichales | f__Erysipelotrichaceae | g__Faecalibaculum | s__uncultured_bacterium |
| 85afae2b9f10e3fc30ad7ab0a7e8b500 | Bacteria | p__Actinobacteriota | c__Actinobacteria | o__Propionibacteriales | f__Nocardioidaceae | g__Nocardioides |  |
| 9e92ced127d235c09f0573e9caf1ecd5 | Bacteria | p__Actinobacteriota | c__Rubrobacteria | o__Rubrobacterales | f__Rubrobacteriaceae | g__Rubrobacter | s__Rubrobacter_bracarensis |
| 04878323 | Bacteria | p__Actinobacteriota | c__Actinobacteria | o__Micrococcales | f__Micrococcaceae | g__Micrococcus |  |
| G009911385_3 | Bacteria | p__Firmicutes | c__Bacilli | o__Lactobacillales | f__Streptococcaceae | g__Lactococcus | s__Lactococcus_garvieae |
| d2eb02210f56bbfb4efbe161e411f803 | Bacteria | p__Actinobacteriota | c__Actinobacteria | o__Micrococcales | f__Microbacteriaceae | g__Microbacterium |  |
| MN453488 | Bacteria | p__Proteobacteria | c__Gammaproteobacteria | o__Burkholderiales | f__Burkholderiaceae | g__Cupriavidus |  |
| 41e8187f10a435f07bbbecf1c60a70d8 | Bacteria | p__Firmicutes | c__Bacilli | o__Bacillales | f__Bacillaceae | g__Virgibacillus |  |
| 0adec41c67963240b5a205add71a2213 | Bacteria | p__Actinobacteriota | c__Actinobacteria | o__Micrococcales | f__Microbacteriaceae |  |  |
| GQ246672 | Bacteria | p__Actinobacteriota | c__Actinobacteria | o__Micrococcales | f__Microbacteriaceae | g__Leucobacter |  |
| 43ffff9c503757071d0cea25d6998e07 | Bacteria | p__Bacteroidota | c__Bacteroidia | o__Bacteroidales | f__Muribaculaceae | g__Muribaculaceae |  |
| 02360419 | Bacteria | p__Proteobacteria | c__Alphaproteobacteria | o__Rhizobiales | f__Rhizobiaceae | g__Allorhizobium-Neorhizobium-Pararhizobium-Rhizobium | s__[Pseudomonas]_geniculata |
| 4b4afa6faca1043b5bdcf0f75ad387db | Bacteria | p__Proteobacteria | c__Alphaproteobacteria | o__Rhizobiales | f__Rhizobiales_Incertae_Sedis | g__Bauldia |  |
| 05021596 | Bacteria | p__Actinobacteriota | c__Actinobacteria | o__Streptomycetales | f__Streptomycetaceae | g__Streptomyces |  |
| HM036655 | Bacteria | p__Actinobacteriota | c__Actinobacteria | o__Micrococcales | f__Microbacteriaceae | g__Agromyces |  |
| 235efc4e7c7c97c706f0283c86fcd6be | Bacteria | p__Proteobacteria | c__Gammaproteobacteria | o__Burkholderiales | f__Nitrosomonadaceae | g__Nitrosospira |  |
| c4d02f876ac8671c0fea08323026abbe | Bacteria | p__Proteobacteria | c__Gammaproteobacteria | o__PLTA13 | f__PLTA13 | g__PLTA13 | s__uncultured_bacterium |
| 065618d6e86c50a955c2b88602184960 | Bacteria | p__Firmicutes | c__Clostridia | o__Oscillospirales | f__[Eubacterium]_coprostanoligenes_group | g__[Eubacterium]_coprostanoligenes_group |  |
| RS-GCF-001320345.1-NZ-BCBD01000323.1 | Bacteria | p__Proteobacteria | c__Gammaproteobacteria | o__Pseudomonadales | f__Pseudomonadaceae | g__Pseudomonas |  |
| AJ305238 | Bacteria | p__Firmicutes | c__Clostridia | o__Oscillospirales | f__Ruminococcaceae | g__Incertae_Sedis | s__[Clostridium]_leptum |
| ea76911d2e8834909af177fc17541886 | Bacteria | p__Bacteroidota | c__Bacteroidia | o__Sphingobacteriales | f__Sphingobacteriaceae |  |  |
| 3572f102feb5e4e0f5693020f3513fd9 | Bacteria | p__Acidobacteriota | c__Acidobacteriae | o__Acidobacteriales | f__uncultured | g__uncultured |  |
| c4e741fb85f357cec0e9361cccc17b63 | Bacteria | p__Proteobacteria | c__Alphaproteobacteria | o__Sphingomonadales | f__Sphingomonadaceae | g__Sphingobium |  |
| 920b4df01348c0a960bda1a73b7092ef | Bacteria | p__Proteobacteria | c__Gammaproteobacteria | o__Pseudomonadales | f__Pseudomonadaceae | g__Pseudomonas |  |
| JN657222 | Bacteria | p__Firmicutes | c__Clostridia | o__Peptostreptococcales-Tissierellales | f__Peptostreptococcales-Tissierellales | g__Peptoniphilus |  |
| 05031333 | Bacteria | p__Actinobacteriota | c__Actinobacteria | o__Frankiales | f__Geodermatophilaceae | g__Klenkia |  |
| 2261b4f3ce0852edea46ee23f1a1190a | Bacteria | p__Proteobacteria | c__Gammaproteobacteria | o__Pseudomonadales | f__Moraxellaceae | g__Acinetobacter |  |
| b91dc714cda7c475f89feeafeacc06dd | Bacteria | p__Bacteroidota | c__Bacteroidia | o__Bacteroidales | f__Bacteroidaceae | g__Bacteroides |  |
| MJ006-2-barcode59-umi115715bins-ubs-4 | Bacteria | p__Proteobacteria | c__Gammaproteobacteria | o__Pseudomonadales | f__Pseudomonadaceae | g__Pseudomonas |  |
| 9f88619de27e875e1e02c103fb02ea87 | Bacteria | p__Proteobacteria | c__Alphaproteobacteria | o__Rhizobiales | f__Beijerinckiaceae | g__FukuN57 | s__uncultured_bacterium |
| MJ031-1-barcode35-umi10623bins-ubs-46 | Bacteria | p__Bacteroidota | c__Bacteroidia | o__Sphingobacteriales | f__Sphingobacteriaceae | g__Sphingobacterium |  |
| 17709328 | Bacteria | p__Chloroflexi | c__Ktedonobacteria | o__Ktedonobacterales | f__Ktedonobacteraceae |  |  |
| 4f346cf56fce2d01f737677e1911bac7 | Bacteria | p__Firmicutes | c__Bacilli | o__Alicyclobacillales | f__Alicyclobacillaceae | g__Tumebacillus | s__uncultured_Tumebacillus |
| 11762957 | Bacteria | p__Armatimonadota | c__Chthonomonadetes | o__Chthonomonadales | f__Chthonomonadaceae | g__Chthonomonas | s__uncultured_Armatimonadetes |
| 04906420 | Bacteria | p__Proteobacteria | c__Alphaproteobacteria | o__Rhizobiales | f__Beijerinckiaceae | g__Methylobacterium-Methylorubrum |  |
| GB-GCA-000292775.1-ALXA01000190.1 | Bacteria | p__Proteobacteria | c__Gammaproteobacteria | o__Pseudomonadales | f__Pseudomonadaceae | g__Pseudomonas |  |
| 1dd3d202b64f67ad6919e23bc843db59 | Bacteria | p__Proteobacteria | c__Alphaproteobacteria | o__Caulobacterales | f__Hyphomonadaceae | g__Hirschia |  |
| 2379aa1f908c7c8c8e681c3d01526e73 | Bacteria | p__Proteobacteria | c__Gammaproteobacteria | o__Burkholderiales | f__Comamonadaceae |  |  |
| 122ae1af6928d156b634f4ce833e5410 | Bacteria | p__Proteobacteria | c__Alphaproteobacteria | o__Elsterales |  |  |  |
| 0940ac215ac4cfa06a03e20c22071fd6 | Bacteria | p__Chloroflexi | c__KD4-96 | o__KD4-96 | f__KD4-96 | g__KD4-96 | s__metagenome |
| e684c3eac4c791b66b8e6639ff2f3bf0 | Bacteria | p__Actinobacteriota | c__Acidimicrobiia | o__Microtrichales | f__uncultured | g__uncultured |  |
| 5f72acaddcd49584f244bc3fb98f440a | Bacteria | p__Acidobacteriota | c__Acidobacteriae | o__Bryobacterales | f__Bryobacteraceae | g__Bryobacter |  |
| 5688e30c895cff6a6fffe2406ab197d0 | Bacteria | p__Firmicutes | c__Bacilli | o__Erysipelotrichales | f__Erysipelotrichaceae | g__ZOR0006 | s__uncultured_organism |
| MJ007-1-barcode36-umi16133bins-ubs-15 | Bacteria | p__Proteobacteria | c__Gammaproteobacteria | o__Pseudomonadales | f__Moraxellaceae | g__Acinetobacter | s__Acinetobacter_gerneri |
| 19248290 | Bacteria | p__Actinobacteriota | c__Actinobacteria | o__Micrococcales | f__Intrasporangiaceae | g__Terrabacter |  |
| RS-GCF-000632205.1-NZ-JFYW01000015.1 | Bacteria | p__Firmicutes | c__Bacilli | o__Exiguobacterales | f__Exiguobacteraceae | g__Exiguobacterium |  |
| 4635cd3d8e70824093052406cd24ae97 | Bacteria | p__Actinobacteriota | c__Thermoleophilia | o__Gaiellales | f__uncultured | g__uncultured | s__Gaiella_sp. |
| 51415028160489265edaa402bf73165e | Bacteria | p__Gemmatimonadota | c__Gemmatimonadetes | o__Gemmatimonadales | f__Gemmatimonadaceae | g__Roseisolibacter | s__uncultured_bacterium |
| b954975813627029aa776f9873bb35e1 | Bacteria | p__Proteobacteria | c__Alphaproteobacteria | o__Rhizobiales | f__Xanthobacteraceae | g__Pseudolabrys |  |
| 4aa67a5c5671eac61f68687bf6c700e7 | Bacteria | p__Proteobacteria | c__Gammaproteobacteria | o__Burkholderiales | f__Comamonadaceae |  |  |
| 09999f57dd622ebe1d689338781decad | Bacteria | p__Actinobacteriota | c__Actinobacteria | o__Micrococcales | f__Microbacteriaceae |  |  |
| 060efbce54acc0e4588edd8b6fcb94a0 | Bacteria | p__Proteobacteria | c__Gammaproteobacteria | o__Burkholderiales |  |  |  |
| f250614b2d5d089bfa87117d957861a7 | Bacteria | p__Proteobacteria | c__Alphaproteobacteria | o__Rhizobiales | f__Xanthobacteraceae | g__uncultured |  |
| f200e60852a45129e5bc4f9fec352897 | Bacteria | p__Actinobacteriota | c__Thermoleophilia | o__Solirubrobacterales | f__67-14 | g__67-14 |  |
| KU355878 | Bacteria | p__Proteobacteria | c__Gammaproteobacteria | o__Burkholderiales | f__Comamonadaceae |  |  |
| G005063315_1 | Bacteria | p__Proteobacteria | c__Alphaproteobacteria | o__Rhizobiales | f__Rhizobiaceae | g__Mesorhizobium |  |
| KX250270 | Bacteria | p__Actinobacteriota | c__Actinobacteria | o__Micrococcales | f__Microbacteriaceae |  |  |
| e830d7820a16d49a791f48e7157c82e3 | Bacteria | p__Proteobacteria | c__Alphaproteobacteria | o__Rhizobiales | f__Rhizobiaceae |  |  |
| abf9dde8502eec9c4fd2bf9d956e8654 | Bacteria | p__Myxococcota | c__bacteriap25 | o__bacteriap25 | f__bacteriap25 | g__bacteriap25 |  |
| 88572e4af4e81599029affdac82a9d97 | Bacteria | p__Firmicutes | c__Clostridia | o__Clostridiales | f__Clostridiaceae |  |  |
| RS-GCF-009935865.1-NZ-QWEL01000022.1 | Bacteria | p__Bacteroidota | c__Bacteroidia | o__Bacteroidales | f__Marinifilaceae | g__Odoribacter | s__uncultured_bacterium |
| LC042213 | Bacteria | p__Actinobacteriota | c__Actinobacteria | o__Micrococcales |  |  |  |
| 4f8aaefcda0622a61f5c776081940d51 | Bacteria | p__Actinobacteriota | c__Thermoleophilia | o__Solirubrobacterales | f__Solirubrobacteraceae | g__uncultured |  |
| 04957439 | Bacteria | p__Proteobacteria | c__Gammaproteobacteria | o__Burkholderiales | f__Alcaligenaceae | g__Achromobacter |  |
| c86d8d37d1d5c5c77195ec08638381eb | Bacteria | p__Proteobacteria | c__Gammaproteobacteria | o__Enterobacterales |  |  |  |
| 0bfa5cb85ea4662b2687ee463dcadd9b | Bacteria | p__Proteobacteria | c__Alphaproteobacteria | o__Rhodobacterales | f__Rhodobacteraceae |  |  |
| 8b2032f04745dec11d62906c857a642b | Bacteria | p__Firmicutes | c__Clostridia | o__Lachnospirales | f__Lachnospiraceae | g__Lachnospiraceae_NK4A136_group |  |
| 8ea2f35cbd901ecb1e3af5ad744f827e | Bacteria | p__Actinobacteriota | c__MB-A2-108 | o__MB-A2-108 | f__MB-A2-108 | g__MB-A2-108 |  |
| G000368585_6 | Bacteria | p__Proteobacteria | c__Gammaproteobacteria | o__Pseudomonadales | f__Moraxellaceae | g__Acinetobacter |  |
| MJ006-2-barcode61-umi157398bins-ubs-3 | Bacteria | p__Firmicutes | c__Clostridia | o__Lachnospirales | f__Lachnospiraceae |  |  |
| 29218abf75b1a888e40cc22aabe9cd30 | Bacteria |  |  |  |  |  |  |
| 0cd9d80f20e3acb91362ef7bfe8d501c | Bacteria | p__Bacteroidota | c__Bacteroidia | o__Bacteroidales | f__Bacteroidetes_vadinHA17 | g__Bacteroidetes_vadinHA17 |  |
| 67ed90419b269f7f6b423b79851c0fd2 | Bacteria | p__Bacteroidota | c__Bacteroidia | o__Bacteroidales | f__Marinifilaceae | g__Odoribacter | s__Odoribacter_splanchnicus |
| 10823123 | Bacteria | p__Firmicutes | c__Bacilli | o__Erysipelotrichales | f__Erysipelotrichaceae | g__Turicibacter | s__uncultured_bacterium |
| 4bbc6cf52d0f4892eed1d86ff374524d | Bacteria | p__Nitrospirota | c__Nitrospiria | o__Nitrospirales | f__Nitrospiraceae | g__Nitrospira |  |
| 0528c1f6073e6776fa61d950d4819535 | Bacteria | p__Actinobacteriota | c__Actinobacteria | o__Corynebacteriales | f__Corynebacteriaceae | g__Corynebacterium |  |
| 32cd1ef952f39683643f63164732dd43 | Bacteria | p__Firmicutes | c__Clostridia | o__Lachnospirales | f__Lachnospiraceae |  |  |
| 2b84757a714c6b9bd0017002d6fee24b | Bacteria | p__Actinobacteriota | c__Thermoleophilia | o__Gaiellales | f__Gaiellaceae | g__Gaiella |  |
| 02336161 | Bacteria | p__Actinobacteriota | c__Actinobacteria | o__Propionibacteriales | f__Propionibacteriaceae | g__Cutibacterium |  |
| 91f38e7f55103750f62ecf7a280896db | Bacteria | p__Acidobacteriota | c__Acidobacteriae | o__Acidobacteriales | f__Acidobacteriaceae_(Subgroup_1) | g__Acidipila |  |
| 6c90fa641ae14781d85d577a666c31a0 | Bacteria | p__Deinococcota | c__Deinococci | o__Deinococcales | f__Deinococcaceae | g__Deinococcus | s__bacterium_1227R |
| 3a5f653f9a734bc4cf91195fc7b2a1ee | Bacteria | p__Actinobacteriota | c__Actinobacteria | o__Micromonosporales | f__Micromonosporaceae | g__Micromonospora |  |
| 213c145ccf3c2480856f256d464408cc | Bacteria |  |  |  |  |  |  |
| 53a755ba22e8ef575f7f56e9d8279464 | Bacteria | p__Chloroflexi | c__Anaerolineae | o__Caldilineales | f__Caldilineaceae | g__uncultured |  |
| 652055d9f5a8eb1add25d0531471a027 | Bacteria | p__Proteobacteria | c__Gammaproteobacteria | o__Xanthomonadales | f__Rhodanobacteraceae | g__Dokdonella |  |
| a96f72e4ca8e8763cf044eafeb0556f1 | Bacteria | p__Chloroflexi | c__JG30-KF-CM66 | o__JG30-KF-CM66 | f__JG30-KF-CM66 | g__JG30-KF-CM66 |  |
| 4c6fff99a56cce72359c0b9bd724e24c | Bacteria | p__RCP2-54 | c__RCP2-54 | o__RCP2-54 | f__RCP2-54 | g__RCP2-54 |  |
| JN615417 | Bacteria | p__Actinobacteriota | c__Actinobacteria | o__Corynebacteriales | f__Nocardiaceae | g__Gordonia |  |
| 74dd47df23332b63865d0e260c548d9a | Bacteria | p__Bacteroidota | c__Bacteroidia | o__Flavobacteriales | f__Crocinitomicaceae | g__Fluviicola |  |
| RS-GCF-007681945.1-NZ-VELY01000030.1 | Bacteria | p__Actinobacteriota | c__Actinobacteria | o__Micrococcales | f__Brevibacteriaceae | g__Brevibacterium |  |
| f63b8152a7af70e531b757683b4d6bf5 | Bacteria | p__Bacteroidota | c__Bacteroidia | o__Sphingobacteriales | f__Sphingobacteriaceae | g__Pedobacter |  |
| f3b532ec83b5f1c0f5aabac85c0dcc1d | Bacteria | p__Proteobacteria | c__Gammaproteobacteria | o__Enterobacterales | f__Enterobacteriaceae | g__Escherichia-Shigella |  |
| 02324958 | Bacteria | p__Actinobacteriota | c__Actinobacteria | o__Propionibacteriales | f__Nocardioidaceae | g__Marmoricola |  |
| e673679fe90e96be47064864e1df8d57 | Bacteria | p__Firmicutes | c__Bacilli | o__Lactobacillales | f__Streptococcaceae | g__Streptococcus | s__Streptococcus_danieliae |
| ba732bda1b7461d346a36bf5fd51f524 | Bacteria | p__Firmicutes | c__Bacilli | o__Lactobacillales | f__Lactobacillaceae | g__Lactobacillus |  |
| JF763851 | Bacteria | p__Proteobacteria | c__Gammaproteobacteria | o__Burkholderiales | f__Burkholderiaceae | g__Burkholderia-Caballeronia-Paraburkholderia |  |
| 2316c1cdc0909577844deae3035fdc75 | Bacteria | p__Bacteroidota | c__Bacteroidia | o__Chitinophagales | f__Chitinophagaceae | g__Flavisolibacter | s__uncultured_Flavobacterium |
| 60466f4d0d71f4a43d7b38c88868f07f | Bacteria | p__Actinobacteriota | c__Actinobacteria | o__Micrococcales | f__Microbacteriaceae | g__Agromyces |  |
| MJ030-2-barcode59-umi106137bins-ubs-3 | Bacteria | p__Proteobacteria | c__Alphaproteobacteria | o__Rhodobacterales | f__Rhodobacteraceae | g__Rhodobacter |  |
| 04918221 | Bacteria | p__Bacteroidota | c__Bacteroidia | o__Sphingobacteriales | f__Sphingobacteriaceae | g__Pedobacter |  |
| 5a433d502e574cab10af10d4a98d4565 | Bacteria | p__Acidobacteriota | c__Vicinamibacteria | o__Subgroup_17 | f__Subgroup_17 | g__Subgroup_17 |  |
| MJ034-2-barcode50-umi9668bins-ubs-19 | Bacteria | p__Verrucomicrobiota | c__Verrucomicrobiae | o__Pedosphaerales | f__Pedosphaeraceae | g__Pedosphaeraceae |  |
| X77446 | Bacteria | p__Actinobacteriota | c__Actinobacteria | o__Micrococcales | f__Microbacteriaceae | g__Microbacterium |  |
| 02266695 | Bacteria | p__Proteobacteria | c__Alphaproteobacteria | o__Sphingomonadales | f__Sphingomonadaceae | g__Sphingomonas |  |
| d58a06d13e7a30aa8e25c2dad904df15 | Bacteria | p__Firmicutes | c__Bacilli | o__Lactobacillales | f__Enterococcaceae | g__Enterococcus |  |
| f56f4bdb0c80657bf1c43f86d7d85a85 | Bacteria | p__Firmicutes | c__Bacilli | o__Erysipelotrichales | f__Erysipelotrichaceae | g__ZOR0006 | s__uncultured_organism |
| 16723565 | Bacteria | p__Firmicutes | c__Clostridia | o__Lachnospirales | f__Lachnospiraceae | g__Lachnoclostridium |  |
| 99cc1272989b09ec7f437d4df915f803 | Bacteria | p__Proteobacteria | c__Alphaproteobacteria | o__Rhizobiales | f__Rhizobiales_Incertae_Sedis | g__Nordella |  |
| 0c620b141ece52676bdfd77c732c2621 | Bacteria | p__Actinobacteriota | c__Acidimicrobiia | o__Microtrichales | f__Ilumatobacteraceae | g__CL500-29_marine_group |  |
| f7e5ace711fd25483ab1fc47738beab8 | Bacteria | p__Proteobacteria | c__Gammaproteobacteria | o__Enterobacterales | f__Enterobacteriaceae |  |  |
| 71581c39e30e46fdb1f8ac9e22738f63 | Bacteria | p__Bacteroidota | c__Bacteroidia | o__Flavobacteriales | f__Flavobacteriaceae | g__Flavobacterium |  |
| MJ006-1-barcode28-umi164319bins-ubs-3 | Bacteria | p__Bacteroidota | c__Bacteroidia | o__Bacteroidales | f__Bacteroidaceae | g__Bacteroides |  |
| ce9af5824a69c4fd2c7951fc4c95c045 | Bacteria | p__Proteobacteria | c__Alphaproteobacteria | o__Rhizobiales |  |  |  |
| b1955066aea5fa850c02cf32995b8eed | Bacteria | p__Actinobacteriota | c__Actinobacteria | o__Corynebacteriales | f__Mycobacteriaceae | g__Mycobacterium |  |
| 1acfa1bc8c3e47bfd1291bc2fa5d0a5a | Bacteria | p__Myxococcota | c__Polyangia | o__Nannocystales | f__Nannocystaceae | g__Nannocystis |  |
| ee01cf62e4d9ead439b89f6e369a23da | Bacteria | p__Acidobacteriota | c__Acidobacteriae | o__Acidobacteriales | f__Acidobacteriaceae_(Subgroup_1) | g__Granulicella |  |
| aca510e84f5f94f4bbd3b0562f5ce329 | Bacteria | p__Bacteroidota | c__Bacteroidia | o__Cytophagales | f__Cytophagaceae | g__Cytophaga | s__Cytophaga_hutchinsonii |
| MJ020-1-barcode29-umi49361bins-ubs-10 | Bacteria | p__Bacteroidota | c__Bacteroidia | o__Flavobacteriales | f__Flavobacteriaceae | g__Flavobacterium | s__Flavobacterium_ummariense |
| ad31c255f4caf481ffbff24307b60f9e | Bacteria | p__Proteobacteria | c__Gammaproteobacteria | o__Enterobacterales | f__Enterobacteriaceae |  |  |
| RS-GCF-001743465.1-NZ-MIZY01000070.1 | Bacteria | p__Proteobacteria | c__Gammaproteobacteria | o__Enterobacterales |  |  |  |
| 0639f17dea4ab0c74b62c47f4f2c4386 | Bacteria | p__Proteobacteria | c__Alphaproteobacteria | o__Rhizobiales | f__Beijerinckiaceae | g__Methylobacterium-Methylorubrum |  |
| b435722913ef280c5475f6b308a63ae4 | Bacteria | p__Bacteroidota | c__Bacteroidia | o__Flavobacteriales | f__Flavobacteriaceae | g__Flavobacterium |  |
| 1516917d38f424fb20e858fc11a2a89e | Bacteria | p__Proteobacteria | c__Gammaproteobacteria | o__Cellvibrionales | f__Spongiibacteraceae | g__BD1-7_clade |  |
| RS-GCF-000719665.1-NZ-JOHJ01000076.1 | Bacteria | p__Actinobacteriota | c__Actinobacteria | o__Streptomycetales | f__Streptomycetaceae | g__Streptomyces |  |
| 62d3af4820d29ffca5ef2f792e9cbc0b | Bacteria | p__Actinobacteriota | c__Acidimicrobiia | o__Microtrichales | f__Iamiaceae | g__Iamia |  |
| 3326500089ac63f3769fcb7ca7f9fa52 | Bacteria | p__Chloroflexi | c__KD4-96 | o__KD4-96 | f__KD4-96 | g__KD4-96 | s__uncultured_bacterium |
| 11048188 | Bacteria | p__Bacteroidota | c__Bacteroidia | o__Bacteroidales | f__Muribaculaceae | g__Muribaculaceae |  |
| 2d6b42fe0beb8bfbb7cb6e498c44e402 | Bacteria | p__Proteobacteria | c__Alphaproteobacteria | o__Rhizobiales | f__Rhizobiales_Incertae_Sedis | g__Phreatobacter |  |
| e6d174696bc6f4f1a51c4a22f89b4886 | Bacteria | p__Proteobacteria | c__Gammaproteobacteria | o__Burkholderiales | f__Comamonadaceae |  |  |
| 00199051 | Bacteria | p__Chloroflexi | c__Anaerolineae | o__SBR1031 | f__A4b | g__A4b | s__uncultured_Caldilineales |
| X97884 | Bacteria | p__Actinobacteriota | c__Actinobacteria | o__Streptosporangiales | f__Nocardiopsaceae | g__Nocardiopsis |  |
| 05023113 | Bacteria | p__Firmicutes | c__Clostridia | o__Lachnospirales | f__Lachnospiraceae | g__Tuzzerella |  |
| G000223155_1 | Bacteria | p__Firmicutes | c__Bacilli | o__Bacillales | f__Bacillaceae | g__Bacillus | s__Bacillus_coagulans |
| RS-GCF-900102995.1-NZ-FNKT01000009.1 | Bacteria | p__Proteobacteria | c__Alphaproteobacteria | o__Caulobacterales | f__Caulobacteraceae | g__Brevundimonas |  |
| 1b2444708861f2b5d402148f980aac48 | Bacteria | p__Bacteroidota | c__Bacteroidia | o__Bacteroidales | f__Muribaculaceae | g__Muribaculaceae | s__uncultured_bacterium |
| 85b3e7c76ab24b766331ba9e534237a8 | Bacteria | p__Acidobacteriota | c__Holophagae | o__Subgroup_7 | f__Subgroup_7 | g__Subgroup_7 |  |
| d914ed4560048ca4d887ab3691d73ae7 | Bacteria | p__Proteobacteria | c__Gammaproteobacteria | o__Burkholderiales | f__Comamonadaceae |  |  |
| 05131733 | Bacteria | p__Proteobacteria | c__Gammaproteobacteria | o__Burkholderiales | f__Alcaligenaceae | g__Parapusillimonas |  |
| bf8affacb07c895b1fc2b577ecbe47b8 | Bacteria | p__Proteobacteria | c__Gammaproteobacteria | o__Cellvibrionales | f__Cellvibrionaceae | g__Cellvibrio |  |
| GB-GCA-905205225.1-CAJLCQ010000084.1 | Bacteria | p__Firmicutes | c__Clostridia | o__Oscillospirales | f__Oscillospiraceae | g__NK4A214_group |  |
| 626766352feacf65cf584e35d2801174 | Bacteria | p__Proteobacteria | c__Gammaproteobacteria | o__Burkholderiales | f__SC-I-84 | g__SC-I-84 |  |
| ea53d23ee1ba1f962fb6b8a4219472fb | Bacteria | p__Proteobacteria | c__Gammaproteobacteria | o__Burkholderiales |  |  |  |
| 08186174 | Bacteria | p__Proteobacteria | c__Alphaproteobacteria | o__Rhizobiales | f__Xanthobacteraceae | g__Rhodoplanes |  |
| MJ006-2-barcode63-umi184111bins-ubs-3 | Bacteria | p__Desulfobacterota | c__Desulfovibrionia | o__Desulfovibrionales | f__Desulfovibrionaceae | g__Bilophila |  |
| e6c61e6b98d20c6f774f1728a9db8683 | Bacteria | p__Actinobacteriota | c__Actinobacteria | o__Pseudonocardiales | f__Pseudonocardiaceae |  |  |
| G002070655_1 | Bacteria | p__Bacteroidota | c__Bacteroidia | o__Bacteroidales | f__Prolixibacteraceae | g__uncultured |  |
| MJ034-1-barcode40-umi26494bins-ubs-4 | Bacteria | p__Acidobacteriota | c__Acidobacteriae | o__Bryobacterales | f__Bryobacteraceae | g__Bryobacter |  |
| 03178730 | Bacteria | p__Planctomycetota | c__Phycisphaerae | o__Tepidisphaerales | f__WD2101_soil_group | g__WD2101_soil_group |  |
| a623704a970344e65e53d5fa9f1bece3 | Bacteria | p__Proteobacteria | c__Alphaproteobacteria | o__Rhizobiales | f__Beijerinckiaceae |  |  |
| MJ006-1-barcode39-umi147065bins-ubs-3 | Bacteria | p__Firmicutes | c__Clostridia | o__Lachnospirales | f__Lachnospiraceae |  |  |
| 97cbe1328ac8c142b2ea1e00644fd6cd | Bacteria | p__Proteobacteria | c__Gammaproteobacteria | o__Pseudomonadales | f__Moraxellaceae | g__Acinetobacter |  |
| 1afa2b9bf236f592388ef4d07d097f76 | Bacteria | p__Proteobacteria | c__Alphaproteobacteria | o__Rhodobacterales | f__Rhodobacteraceae | g__Amaricoccus |  |
| 10702656 | Bacteria | p__Bacteroidota | c__Bacteroidia | o__Flavobacteriales | f__Flavobacteriaceae | g__uncultured | s__gut_metagenome |
| 12549c0b17b4bf5d81ae134f09e30950 | Bacteria | p__Actinobacteriota | c__Actinobacteria | o__Propionibacteriales | f__Propionibacteriaceae | g__Brooklawnia |  |
| DQ022958 | Bacteria | p__Proteobacteria | c__Alphaproteobacteria | o__Azospirillales | f__Azospirillaceae | g__Azospirillum |  |
| RS-GCF-002334765.1-NZ-BEGN01000009.1 | Bacteria | p__Proteobacteria | c__Gammaproteobacteria | o__Enterobacterales |  |  |  |
| d7ebcc60266147c8fc3502ac9fa25677 | Bacteria | p__Bacteroidota | c__Bacteroidia | o__Sphingobacteriales | f__Sphingobacteriaceae | g__Sphingobacterium |  |
| ef62f040524e77f45c54f1626ce801cf | Bacteria | p__Chloroflexi | c__Anaerolineae | o__1-20 | f__1-20 | g__1-20 | s__metagenome |
| 02358627 | Bacteria | p__Firmicutes | c__Clostridia | o__Oscillospirales | f__Ruminococcaceae | g__Faecalibacterium |  |
| 04926785 | Bacteria | p__Deferribacterota | c__Deferribacteres | o__Deferribacterales | f__Deferribacteraceae | g__Mucispirillum | s__Mucispirillum_schaedleri |
| fa5aef33f5286509159a651a44f19c40 | Bacteria | p__Firmicutes | c__Bacilli | o__Paenibacillales | f__Paenibacillaceae | g__Paenibacillus |  |
| 8d407e8a737db859224ed5cf8db12e19 | Bacteria | p__Proteobacteria | c__Gammaproteobacteria | o__Pseudomonadales | f__Moraxellaceae | g__Acinetobacter |  |
| MJ034-1-barcode34-umi21262bins-ubs-9 | Bacteria | p__Acidobacteriota | c__Blastocatellia | o__Pyrinomonadales | f__Pyrinomonadaceae | g__RB41 |  |
| 04994564 | Bacteria | p__Proteobacteria | c__Alphaproteobacteria | o__Rhizobiales | f__Beijerinckiaceae |  |  |
| GB-GCA-016191205.1-JACPPB010000018.1 | Bacteria | p__Proteobacteria | c__Gammaproteobacteria |  |  |  |  |
| 5ff8cde105b49924346ea95975ff48f4 | Bacteria | p__Proteobacteria | c__Alphaproteobacteria | o__Elsterales | f__uncultured | g__uncultured |  |
| 44d514c7015ef3badbe6918184fa63f1 | Bacteria | p__Bacteroidota | c__Bacteroidia | o__Sphingobacteriales | f__Sphingobacteriaceae | g__Sphingobacterium |  |
| ba4dfeb223ad1d2772de25a6df5dfe97 | Bacteria | p__Actinobacteriota | c__Actinobacteria | o__Streptomycetales | f__Streptomycetaceae | g__Kitasatospora |  |
| 757f39762797538f21e557d4b07d9e9f | Bacteria | p__Chloroflexi | c__Dehalococcoidia | o__S085 | f__S085 | g__S085 |  |
| fc3ebceb99a2a98a83aef46c5aab7f27 | Bacteria | p__Proteobacteria | c__Alphaproteobacteria | o__Sphingomonadales | f__Sphingomonadaceae |  |  |
| 82626528482d4c8011c9d338f2daccbc | Bacteria | p__Actinobacteriota | c__Acidimicrobiia | o__Microtrichales | f__Microtrichaceae | g__IMCC26207 |  |
| 18686319 | Bacteria | p__Actinobacteriota | c__Actinobacteria | o__Frankiales | f__Sporichthyaceae | g__uncultured |  |
| 09f514ace11f3189ebd194b0cd7e51c7 | Bacteria | p__Proteobacteria | c__Gammaproteobacteria | o__Pseudomonadales | f__Moraxellaceae | g__Acinetobacter |  |
| 04995420 | Bacteria | p__Proteobacteria | c__Alphaproteobacteria | o__Rhizobiales | f__Rhizobiaceae | g__Allorhizobium-Neorhizobium-Pararhizobium-Rhizobium |  |
| 08579902 | Bacteria | p__Proteobacteria | c__Alphaproteobacteria | o__uncultured | f__uncultured | g__uncultured | s__uncultured_Acetobacteraceae |
| G900110015_1 | Bacteria | p__Firmicutes | c__Bacilli | o__Bacillales | f__Bacillaceae | g__Terribacillus |  |
| 9c29fd7828d25d08ab3f7db18e082e09 | Bacteria | p__Firmicutes | c__Bacilli | o__Erysipelotrichales | f__Erysipelatoclostridiaceae | g__Erysipelatoclostridium | s__uncultured_Clostridium |
| 8d33882a9c3fdc1a3f4ff25fc9f5b2e8 | Bacteria | p__Firmicutes | c__Clostridia | o__Oscillospirales | f__Ruminococcaceae | g__Subdoligranulum |  |
| 02307264 | Bacteria | p__Actinobacteriota | c__Acidimicrobiia | o__Microtrichales | f__Ilumatobacteraceae |  |  |
| d5811365aae262cbca764db7d8a9c90b | Bacteria | p__Proteobacteria | c__Alphaproteobacteria | o__uncultured | f__uncultured | g__uncultured |  |
| RS-GCF-001297705.1-NZ-LIRF01000002.1 | Bacteria | p__Bacteroidota | c__Bacteroidia | o__Flavobacteriales | f__Weeksellaceae | g__Chryseobacterium |  |
| a7a797bb15ef28bad4453f4b9444db00 | Bacteria | p__Proteobacteria | c__Alphaproteobacteria | o__Rhizobiales | f__Rhizobiaceae |  |  |
| 13686536 | Bacteria | p__Gemmatimonadota | c__Gemmatimonadetes | o__Gemmatimonadales | f__Gemmatimonadaceae |  |  |
| 299f394d45b9ebe86a26c63d5aa4231c | Bacteria | p__Proteobacteria | c__Alphaproteobacteria | o__Rhizobiales | f__Xanthobacteraceae | g__uncultured |  |
| 04952109 | Bacteria | p__Firmicutes | c__Bacilli | o__Paenibacillales | f__Paenibacillaceae | g__Paenibacillus |  |
| FN870023 | Bacteria | p__Actinobacteriota | c__Actinobacteria | o__Micrococcales | f__Microbacteriaceae | g__Microbacterium |  |
| 33a792d1f6b2f1f0849ee203f8a02714 | Bacteria | p__Proteobacteria | c__Alphaproteobacteria | o__Rhizobiales | f__Hyphomicrobiaceae | g__Pedomicrobium |  |
| 826f98216690e95709d416a24762155c | Bacteria | p__Proteobacteria | c__Alphaproteobacteria | o__Rhizobiales | f__Beijerinckiaceae | g__Methylobacterium-Methylorubrum |  |
| 17730223 | Bacteria | p__Firmicutes | c__Bacilli | o__RF39 | f__RF39 | g__RF39 |  |
| 54063b377f1713763df0baafaa9a047e | Bacteria | p__Chloroflexi | c__Ktedonobacteria | o__Ktedonobacterales | f__JG30-KF-AS9 | g__JG30-KF-AS9 | s__uncultured_bacterium |
| 14833928 | Bacteria | p__Chloroflexi | c__Ktedonobacteria | o__C0119 | f__C0119 | g__C0119 | s__uncultured_bacterium |
| eb21a780aefaafe999daf2f1969df522 | Bacteria | p__Bdellovibrionota | c__Bdellovibrionia | o__Bacteriovoracales | f__Bacteriovoracaceae | g__Peredibacter |  |
| CP002857 | Bacteria | p__Actinobacteriota | c__Actinobacteria | o__Corynebacteriales | f__Corynebacteriaceae | g__Corynebacterium |  |
| 85f738b7b3a78b1a7ea60cf942c0f40b | Bacteria | p__Actinobacteriota | c__Thermoleophilia | o__Gaiellales |  |  |  |
| 04962066 | Bacteria | p__Actinobacteriota | c__Actinobacteria | o__Propionibacteriales | f__Nocardioidaceae | g__Nocardioides |  |
| 04907648 | Bacteria | p__Proteobacteria | c__Alphaproteobacteria | o__Caulobacterales | f__Caulobacteraceae | g__Brevundimonas |  |
| 13682962 | Bacteria | p__Gemmatimonadota | c__Gemmatimonadetes | o__Gemmatimonadales | f__Gemmatimonadaceae | g__uncultured | s__uncultured_proteobacterium |
| eed397836318114bc33e4cac04b62161 | Bacteria | p__Firmicutes | c__Bacilli | o__Lactobacillales | f__Lactobacillaceae | g__Lactobacillus |  |
| G001477055_1 | Bacteria | p__Actinobacteriota | c__Actinobacteria | o__Micrococcales | f__Microbacteriaceae |  |  |
| ecb6bf908904ef55309643e74799b45f | Bacteria | p__Chloroflexi | c__KD4-96 | o__KD4-96 | f__KD4-96 | g__KD4-96 |  |
| AB075017 | Bacteria | p__Proteobacteria | c__Gammaproteobacteria | o__Burkholderiales | f__Comamonadaceae | g__Delftia |  |
| 93cefe0fcb84e2cfd6b9ef81d3f5fbfe | Bacteria | p__Proteobacteria | c__Gammaproteobacteria | o__Steroidobacterales | f__Steroidobacteraceae | g__Steroidobacter |  |
| 04933503 | Bacteria | p__Bacteroidota | c__Bacteroidia | o__Chitinophagales | f__Chitinophagaceae | g__uncultured |  |
| e169914929d7b0202090c5e740c90186 | Bacteria | p__Firmicutes | c__Bacilli | o__Lactobacillales | f__Enterococcaceae | g__Enterococcus |  |
| 04941289 | Bacteria | p__Acidobacteriota | c__Vicinamibacteria | o__Vicinamibacterales | f__uncultured | g__uncultured |  |
| GB-GCA-900113425.1-FOZF01000008.1 | Bacteria | p__Proteobacteria | c__Gammaproteobacteria | o__Xanthomonadales | f__Xanthomonadaceae | g__Stenotrophomonas |  |
| 8a97ac84cbc40ee92c0ea247bddc4e2f | Bacteria | p__Proteobacteria | c__Gammaproteobacteria | o__Pseudomonadales | f__Moraxellaceae | g__Acinetobacter |  |
| 04989985 | Bacteria | p__Gemmatimonadota | c__Gemmatimonadetes | o__Gemmatimonadales | f__Gemmatimonadaceae | g__uncultured | s__Acidobacteria_bacterium |
| bedf09005948cb06e758b68a59d131e1 | Bacteria | p__Bacteroidota | c__Bacteroidia | o__Cytophagales | f__Hymenobacteraceae | g__Hymenobacter | s__Siccationidurans_occulans |
| 551d33f277f34f351e17b9323b7f4bbe | Bacteria | p__Acidobacteriota | c__Thermoanaerobaculia | o__Thermoanaerobaculales | f__Thermoanaerobaculaceae | g__Subgroup_10 | s__metagenome |
| ab60a9b5208726157350e8ae4375459d | Bacteria | p__Bacteroidota | c__Bacteroidia | o__Chitinophagales | f__Chitinophagaceae |  |  |
| 05001115 | Bacteria | p__Cyanobacteria | c__Cyanobacteriia | o__Cyanobacteriales | f__Coleofasciculaceae | g__Microcoleus_PCC-7113 | s__Microcoleus_sp. |
| 6fb632f72848052b763ba16f9d451632 | Bacteria | p__Firmicutes | c__Syntrophomonadia | o__Syntrophomonadales | f__Syntrophomonadaceae | g__Pelospora | s__uncultured_bacterium |
| 02411515 | Bacteria | p__Proteobacteria | c__Alphaproteobacteria | o__Rhizobiales | f__Methyloligellaceae |  |  |
| dfac5213649a771eb11905bfe7b9037c | Bacteria | p__Proteobacteria | c__Alphaproteobacteria | o__Rhizobiales | f__Rhizobiales_Incertae_Sedis | g__Bauldia |  |
| f4ea53fa59cfd918c7803815ab18ef6f | Bacteria | p__Acidobacteriota | c__Acidobacteriae | o__Subgroup_2 | f__Subgroup_2 | g__Subgroup_2 |  |
| 3f2f5508b70f1d0e30787949d898ad93 | Bacteria | p__Actinobacteriota | c__Actinobacteria | o__Micrococcales | f__Microbacteriaceae |  |  |
| 8a11bfa33f62476073a14eab3769704b | Bacteria | p__Firmicutes | c__Bacilli | o__Bacillales | f__Planococcaceae | g__Lysinibacillus |  |
| AY633969 | Bacteria | p__Actinobacteriota | c__Actinobacteria | o__Propionibacteriales | f__Nocardioidaceae | g__Nocardioides |  |
| 2a56fdf28c54eec5fff38ae63a293280 | Bacteria | p__Proteobacteria | c__Gammaproteobacteria | o__Pseudomonadales | f__Pseudomonadaceae | g__Pseudomonas |  |
| bc0a34e2f59a415b757ed2144193e7c7 | Bacteria | p__Actinobacteriota | c__Actinobacteria | o__Corynebacteriales | f__Corynebacteriaceae | g__Corynebacterium |  |
| 1c867c0d257c9177e16f96cf75755ca3 | Bacteria | p__Acidobacteriota | c__Vicinamibacteria | o__Vicinamibacterales | f__Vicinamibacteraceae | g__Vicinamibacteraceae |  |
| RS-GCF-016467395.1-NZ-JAELWA010000007.1 | Bacteria | p__Proteobacteria | c__Alphaproteobacteria | o__Reyranellales | f__Reyranellaceae | g__Reyranella |  |
| 6042e9a90bfaf68a4dc003c4ae531317 | Bacteria | p__Actinobacteriota | c__Actinobacteria | o__Micrococcales | f__Microbacteriaceae |  |  |
